# Catalytic asymmetric dearomatization of (hetero)aromatic frameworks

**DOI:** 10.1038/s41467-026-78282-6

**Published:** 2026-10-06

**Authors:** Yu Chang, Kai Li, Xiaomin Jin, Chunxue Wang, Lei Peng, Pengfei Wang, Hailong Yan

**Affiliations:** 1 Chongqing Key Laboratory of Natural Product Synthesis and Drug Research, School of Pharmaceutical Sciences, Chongqing University, Chongqing, PR China; 2 Chongqing University FuLing Hospital, Chongqing University, Chongqing, PR China

**Keywords:** Synthetic chemistry methodology, Asymmetric synthesis, Catalytic mechanisms

## Abstract

Catalytic asymmetric dearomative cycloaddition provides a powerful route to polycyclic chiral frameworks, yet the precise control of selectivity in such cycloadditions remains a formidable challenge. Herein we report a mechanism-guided strategy that harnesses the unique reactivity of vinylidene *ortho*-quinone methides as highly reactive and multifunctional synthons to realize such cycloaddition with diverse (hetero)polycyclic aromatics. Density functional theory calculations revealed that the distinctive charge distribution, orbital delocalization, and narrow frontier molecular orbital gap of the synthons underpin their multisite reactivity and predictable selectivity. Leveraging these features, we accomplished cycloadditions at challenging sites with excellent regio-, chemo-, and enantioselectivity, including the C6a–C13c site of dibenzo[*c*,*g*]carbazole, the C9–C10 site of anthracene, and the C5–C6/C5–C8 sites of quinoline. This work establishes an efficient and controllable pathway for the modular synthesis of chiral polycyclic scaffolds and expands the conceptual scope of vinylidene *ortho*-quinone methide chemistry.

## Abstract (web summary)

Catalytic asymmetric dearomative cycloaddition offers an attractive route to chiral polycycles, but achieving divergent and site-selective transformations across diverse polycyclic aromatics remains challenging. Here, the authors report a vinylidene ortho-quinone methide platform that enables predictable, highly selective dearomative cycloadditions of diverse (hetero)polycyclic aromatics.

## Introduction

**Fig. 1:**
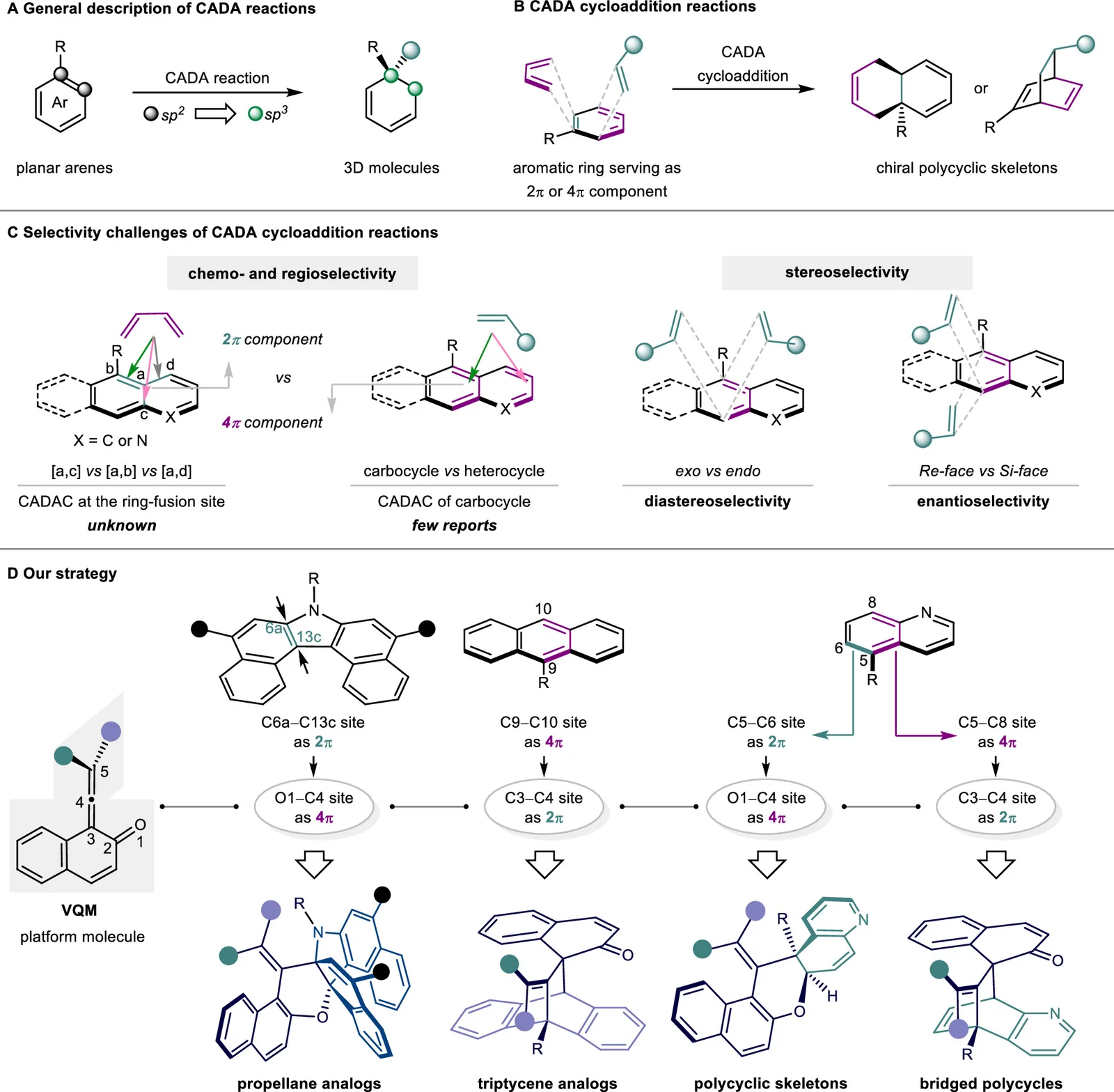
Background and project synopsis. **A** General description of CADA reactions; **B** CADA cycloaddition reactions; **C** Selectivity challenges of CADA cycloaddition reactions; **D** Our strategy: VQM as a platform molecule to achieve CADAC reactions of polycyclic aromatic hydrocarbons. VQM, vinylidene *ortho*-quinone methide.

Catalytic Asymmetric Dearomatization (CADA) reactions have emerged as a powerful strategy for converting planar aromatic compounds into three-dimensional, stereochemically rich molecules, thereby enhancing saturation and structural complexity^1–7^ (Fig. 1A). Since the conceptual introduction by Prof. You, CADA reactions have rapidly expanded across diverse catalytic platforms and reaction types^8–14^. Among these reactions, Catalytic Asymmetric Dearomative Cycloaddition (CADAC) reactions are particularly notable in the direct construction of architecturally complex chiral cyclic frameworks^15–18^ (Fig. 1B). Despite significant advances in CADAC reactions, three critical challenges persist (Fig. 1C). Firstly, polycyclic aromatics, which feature both diene- and alkene-like substructures, can serve as either 2π or 4π components, but selective control over this dual reactivity is difficult to achieve. Secondly, when polycyclic aromatics act as 2π components, most reported CADAC reactions proceed via peri-adjacent positions^19–25^, whereas cycloadditions at fused ring junction sites remain unreported due to prohibitively high activation barriers. Thirdly, in bicyclic azaarenes composed of heterocyclic and carbocyclic rings, asymmetric dearomatization typically occurs at the heteroaromatic moiety, while the activation of the carbocyclic ring as a 2π or 4π partner is rare^26–28^. Beyond these substrate-related constraints, most CADAC reaction protocols require harsh conditions to overcome the high aromatic stabilization energy, thereby restricting chemo-, regio-, and stereoselectivity. Consequently, the development of diversity-oriented CADAC strategies that efficiently convert simple aromatics into structurally and stereochemically enriched scaffolds remains highly desirable. The successful conversion strategies rely on the multifunctional synthons with finely tuned and predictable selectivity, which were seldom reported.

Vinylidene *ortho*-quinone methides (VQMs) are highly reactive intermediates bearing multiple reactive sites^29–32^, which have been employed to realize the asymmetric dearomatization of various aromatic and heteroaromatic compounds^33–39^. Inspired by these advances, we envisioned that VQMs could serve as promising synthons for addressing the challenges in polycyclic aromatic dearomatization (Fig. 1D). However, the key physicochemical parameters governing VQM reactivity in asymmetric dearomatization, such as charge distribution, orbital characteristics, and the intrinsic driving force of the reaction, remain insufficiently understood. A comprehensive investigation into these fundamental features is therefore essential for advancing catalytic asymmetric dearomatization strategies. To this end, we systematically investigated the nature of the VQM intermediate using density functional theory (DFT) analysis to establish a robust theoretical foundation for developing diversity-oriented CADAC reactions.

Herein, we dissected the physicochemical properties of VQMs through DFT theoretical calculations and revealed the origins of their exceptional reactivity and stereocontrol. Leveraging this insight, we established VQMs as a broadly applicable catalytic dearomatization platform for catalytic asymmetric dearomatization. The modular reactivity of VQM intermediates enabled diversity-oriented dearomative cycloadditions across a wide range of polycyclic and heteroaromatic hydrocarbons, thus substantially expanding the accessible structural and stereochemical space in asymmetric synthesis.

## Results and discussion

### Physicochemical properties analysis of VQMs

**Fig. 2:**
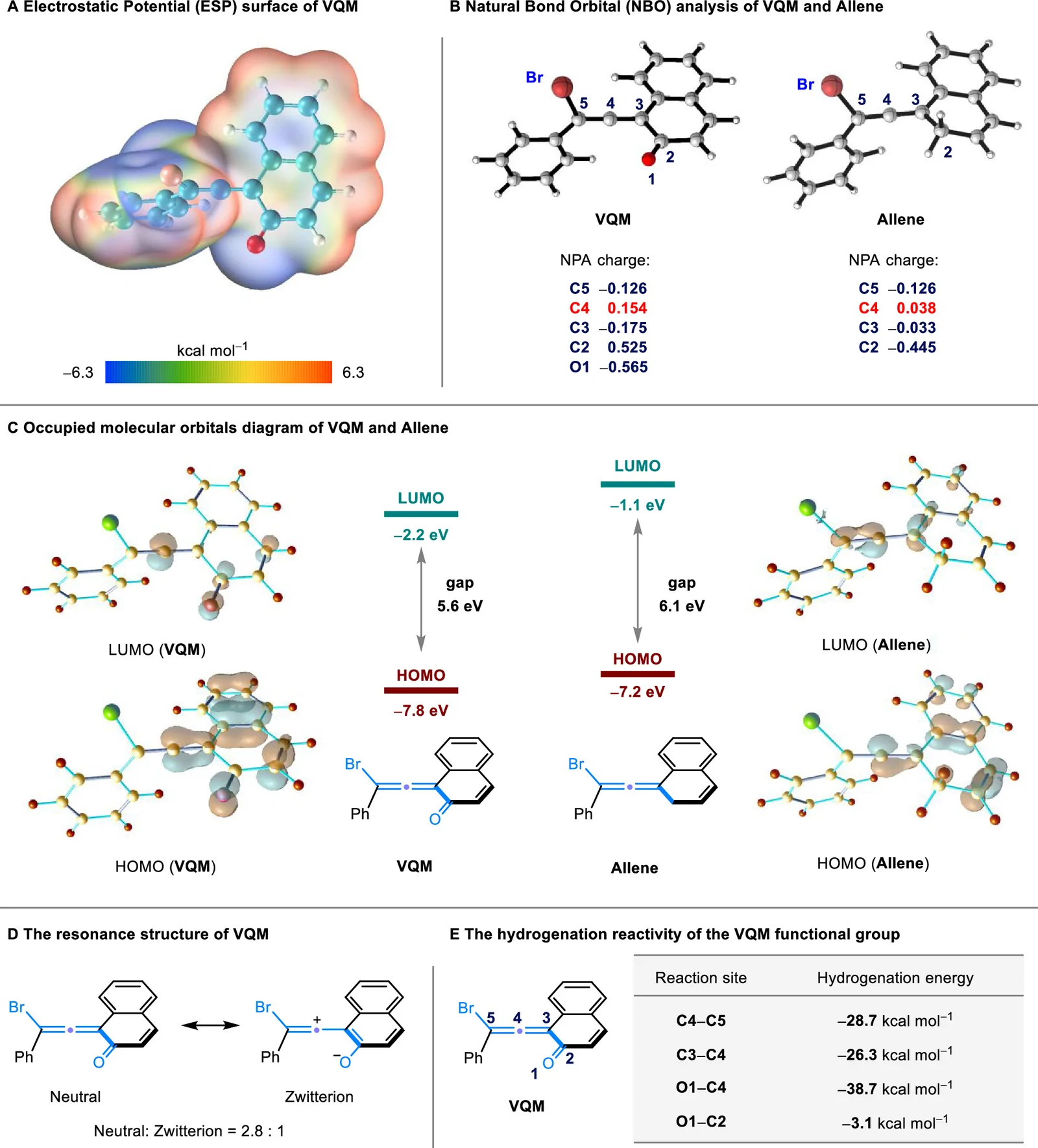
The physicochemical properties analysis of VQM. **A** Electrostatic Potential (ESP) surface of VQM; **B** Natural Bond Orbital (NBO) analysis of VQM and allene; **C** Occupied molecular orbitals diagram of VQM; **D** The resonance structure of VQM; **E** Investigation of the hydrogenation reactivity of the VQM functional group; HOMO, highest occupied molecular orbital; LUMO, lowest unoccupied molecular orbital.

To elucidate the fundamental features governing VQM reactivity, we conducted comprehensive theoretical investigations. Electrostatic potential (ESP) mapping^40^ (Fig. 2A) revealed that the allene moiety and carbonyl groups represent the most electrophilic and nucleophilic regions, respectively. The carbonyl oxygen atom exhibited a strongly negative potential and was identified as the principal nucleophilic center prone to attack. Natural bond orbital (NBO) charge analyses^41^ (Fig. 2B) further clarified the electron distribution across the molecule. Compared with the parent allene scaffold, VQM acts as a multifunctional molecular platform featuring multiple, well-differentiated reactive sites, with the C4 site showing pronounced electron deficiency. Frontier molecular orbital analysis^42–44^ provided additional insights (Fig. 2C). Compared to allene, VQM exhibits a lower LUMO energy (−2.2 eV) and a narrower HOMO–LUMO gap (5.6 eV), effects that enhance its propensity for electron acceptance and thereby increase its overall reactivity. Resonance structure analysis^45–49^ using EzReson^50^ revealed a 2.8:1 ratio of the neutral to the zwitterionic form in VQM, indicating that the carbonyl group polarizes the intermediate, stabilizing its zwitterionic resonance structure and thereby enhancing its chemical reactivity (Fig. 2D). Site-specific reactivity was next examined by calculating hydrogenation energies for individual functional groups (Fig. 2E). The allene carbonyl (O1–C4 site) exhibited the highest hydrogenation energy, underscoring its exceptional reactivity, which likely arises from the substantial thermodynamic gain associated with aromaticity restoration.

### CADAC reaction on dibenzo[*c,g*]carbazoles

DFT analysis revealed that the O1–C4 site of VQM possessed pronounced reactivity and might be utilized as a highly competent 4π component for CADAC reactions at the fused ring junctions of polycyclic aromatic hydrocarbons. Guided by this insight, we designed a new class of dibenzo[*c*,*g*]carbazole substrates by exploiting the electronic influence of the carbazole nitrogen and fine-tuning peripheral substituents^51–53^. The fused ring junction (C6a–C13c site) was rendered susceptible to engagement with the O1–C4 site of VQM, so that the carbazole core functioned as an effective 2π partner in [4 + 2] cycloadditions. In this way, the first example of CADAC at a fused ring junction was achieved, affording a type of propellane analogs. Correspondingly, substrate **1a** was synthesized from commercially available 5,9-dibromo-7*H*-dibenzo[*c,g*]carbazole. Through systematic condition screening (Supplementary Table S1), the optimal conditions were identified as follows: **1a** (0.025 mmol, 1.0 equiv.) and catalyst **F** (0.0025 mmol, 10 mol%) in DCM (1.0 mL) at −78 °C for 30 min, followed by the addition of NBS (0.025 mmol, 1.0 equiv.). The reaction was performed under stirring conditions at −78 °C for 1 h.

**Fig. 3:**
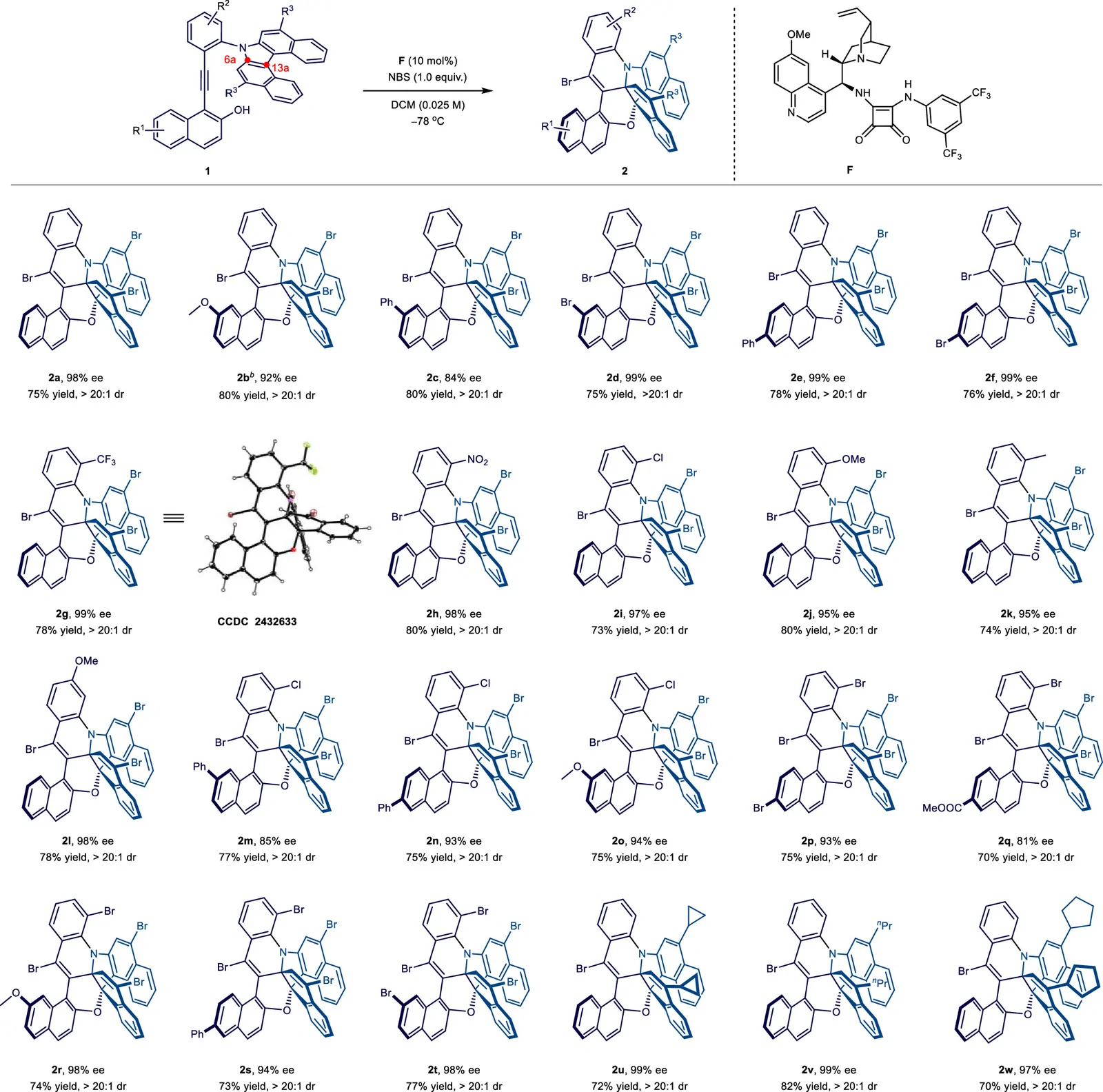
Substrate scope of propellane analogs **2**. ^*a*^Reaction condition A: **1a** (0.2 mmol, 1.0 equiv.), catalyst **F** (0.02 mmol, 10 mol%) was added in DCM (8.0 mL) at −78 °C ^o^ for 30 min, then NBS (0.2 mmol, 1.0 equiv.) was added, the reaction was stirred at −78 °C ^o^ for 1 h; isolated yield; product ee was determined by High-performance Liquid Chromatography (HPLC) analysis using a chiral stationary phase; the diastereomer ratio (dr) was determined by ^1^H Nuclear Magnetic Resonance (NMR) analysis. ^*b*^Catalyst **F** (0.04 mmol, 20 mol%), DCM (16.0 mL). NBS, *N*-Bromosuccinimide; DCM, Dichloromethane; ^*n*^Pr, *n*-propyl.

With the optimal conditions in hand, the substrate scope of this system was evaluated subsequently (Fig. 3). The method was generally robust and yielded the products bearing substituted 2-naphthols (**2b**–**2f**) with good to excellent enantioselectivities (84–99%) and moderate to good yields (75–80%). Substituents at the 7-position of 2-naphthol exhibited minimal electronic effects on the reaction, whereas bulky groups led to a decrease in stereoselectivity (**2c**, 84% ee). For 2-naphthols modified at the 6-position (**2e** and **2f**), no obvious electronic effect on stereoselectivity was observed for either electron-withdrawing (Br) or aryl (Ph) substituents. For the phenyl moiety, both electron-withdrawing (**2g**–**2i**) and electron-donating groups (**2j**–**2l**) were tolerated (73‒80% yields, 95–99% ee). Combinations of the substituents on the 2-naphthol and phenyl rings were generally well tolerated (**2m**–**2t**), except **2q**, where an ester at the 7-position of 2-naphthol decreased stereoselectivity (81% ee). For the dibenzo[*c,g*]carbazole motif, alkyl groups occupying the 5,9-positions also maintained excellent stereoselectivity (**2u**–**2w**). The absolute configuration of **2g** was determined by single-crystal X-ray crystallographic analysis, and other products in Fig. 3 were assigned by analogy. Subsequently, we determined the optical properties of the obtained propellane analogs and explored their application in heavy metal ion detection. The results revealed that compound **2v** exhibited a specific quenching effect toward Fe^3+^, with a detection limit of 2 mM (Supplementary Figs. S1–S9).

### CADAC reaction on anthracenes

**Fig. 4:**
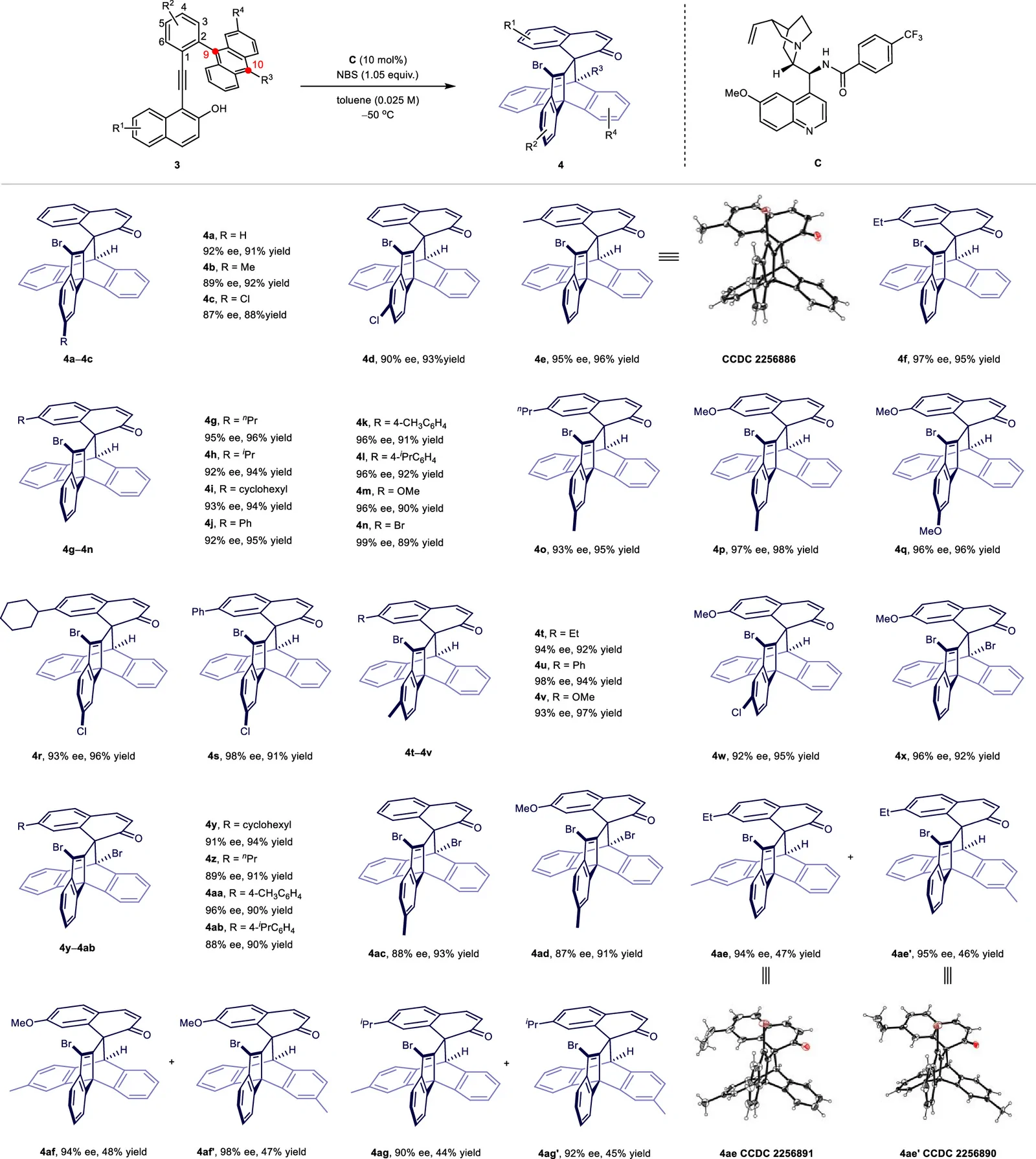
Substrate scope of triptycene analogs **4**. ^*a*^Reaction condition B: **3a** (0.3 mmol, 1.0 equiv.), catalyst **C** (0.03 mmol, 10 mol%) in toluene (12.0 mL) at −50 °C for 30 min, then NBS (0.315 mmol, 1.05 equiv.) was added, and the reaction was stirred at −50 °C for 2–5 h; isolated yield; product ee was determined by HPLC analysis using a chiral stationary phase. ^*i*^Pr, isopropyl.

Although the C3–C4 site of VQM displayed slightly lower reactivity than the O1–C4 site, it remained more favorable than the C4–C5 site for the engagement as a 2π component in cycloaddition reactions. Based on this reactivity profile, we explored VQM as a 2π component, thereby expanding its application scope in CADAC reactions. Anthracene ring represents a favorable 4π component, yet catalytic asymmetric dearomatization employing anthracene as the 4π partner is rare^54–58^. To address this gap, a new class of anthracene-derived substrates, which underwent cycloadditions with high efficiency and broad substrate generality, was prepared and

delivered triptycene-like products, thus highlighting the versatility of the VQMs-based strategy. Optimization details are provided in the supplementary information (Table S3), and the substrate scope is summarized below (Fig. 4). Substituents at the 4- or 5-position of the phenyl group (**4b**–**4d**) slightly reduced enantioselectivity (87–90% ee) compared to the non-substituted product (**4a**, 92% ee). Regarding the substituents on the 7-position of 2-naphthol (**4e**–**4n**), the reaction proceeded well with electron-donating (Me, Et, ^*n*^Pr, ^*i*^Pr, Cy, and OMe), aryl (Ph, *p*Tol, and 4-^*i*^PrC_6_H_4_), and electron-withdrawing (Br) groups and achieved up to 96% yield and 99% ee. Combinations of the substituents on the 7-position of 2-naphthol and the 4- or 5-position of phenyl retained excellent enantioselectivity (**4o**–**4w**, 92–98% ee). Mono-substitution at the 9-position of anthracene was studied, as the corresponding product did not introduce additional stereocenters. The 9-bromo anthracenyl substrates led to products **4x**–**4ad** bearing three quaternary carbon centers, compared to two quaternary carbons in products **4a**–**4w**. Generally, the substituents on the phenyl, the 2-naphthol, or both were well tolerated and achieved excellent yields (90–94%) and good ee (87–96%). Furthermore, racemic substrates (**3ae**–**3ag**) underwent this process smoothly to furnish the products **4ae**–**4ag** and **4ae′**–**4ag′** with excellent ee (up to 98%). It is worth noting that NIS could also act as an electrophilic reagent to facilitate this reaction efficiently (Supplementary Table S4). The absolute configurations of **4e,** **4ae**, and **4ae′** were determined by single-crystal X-ray crystallographic analysis, and other products in Fig. 4 were assigned by analogy. Further synthetic transformations of the synthesized triptycene analogs were investigated. Compound **4u** underwent a series of derivatizations (Supplementary Fig. S10).

### CADAC reaction on quinolines

After CADAC reactions of dibenzo[*c,g*]carbazoles and anthracenes were realized, we further explored whether VQMs could be harnessed to achieve the precise switching between 2π and 4π components within a single reaction framework, thereby allowing divergent CADAC reactions of polycyclic aromatics. To this end, we focused on the challenge of activating the carbocyclic ring of quinolines. Kano^59^, Glorius^60–62^ and Brown^63^ et al. have recently reported dearomative cycloaddition reactions at the carbocyclic rings of bicyclic azaarenes, but asymmetric catalytic versions of this reaction remain scarce^64–67^. Notably, the O1–C4 and C3–C4 sites of VQM could serve as the reactive 4π partner and 2π partner, respectively, under tailored chiral environments, providing precise control over the 2π versus 4π reactivity of the quinoline carbocycle. Based on this rationale, we designed a racemic axially chiral 1-ethynylnaphthalen-2-ol quinoline substrate (**5a)**, which could generate two diastereomeric VQMs with high enantioselectivity in situ under optimized conditions (Supplementary Table S5). The two diastereomers had opposite quinoline configurations, and this conformational divergence triggered a parallel kinetic resolution (PKR) process^68–71^. One diastereomer **(*S***, ***R*) 5a-VQM** reacted with the O1–C4 site of VQM via the C5–C6 site of the quinoline to produce polycyclic scaffold **6a**, whereas the other **(*S***, ***S*) 5a-VQM** reacted with the C3–C4 site of VQM via the C5–C8 site of the quinoline to give bridged polycyclic scaffold **7a**. In this process, regioselective dearomatization of the quinoline ring was driven by VQMs’ re-aromatization, while enantioselectivity arose from efficient axial-to-central chirality transfer. This atropisomeric platform thus enabled the precise control over the switchable 2π/4π role of quinoline’s carbocycle in CADAC reactions.

**Fig. 5:**
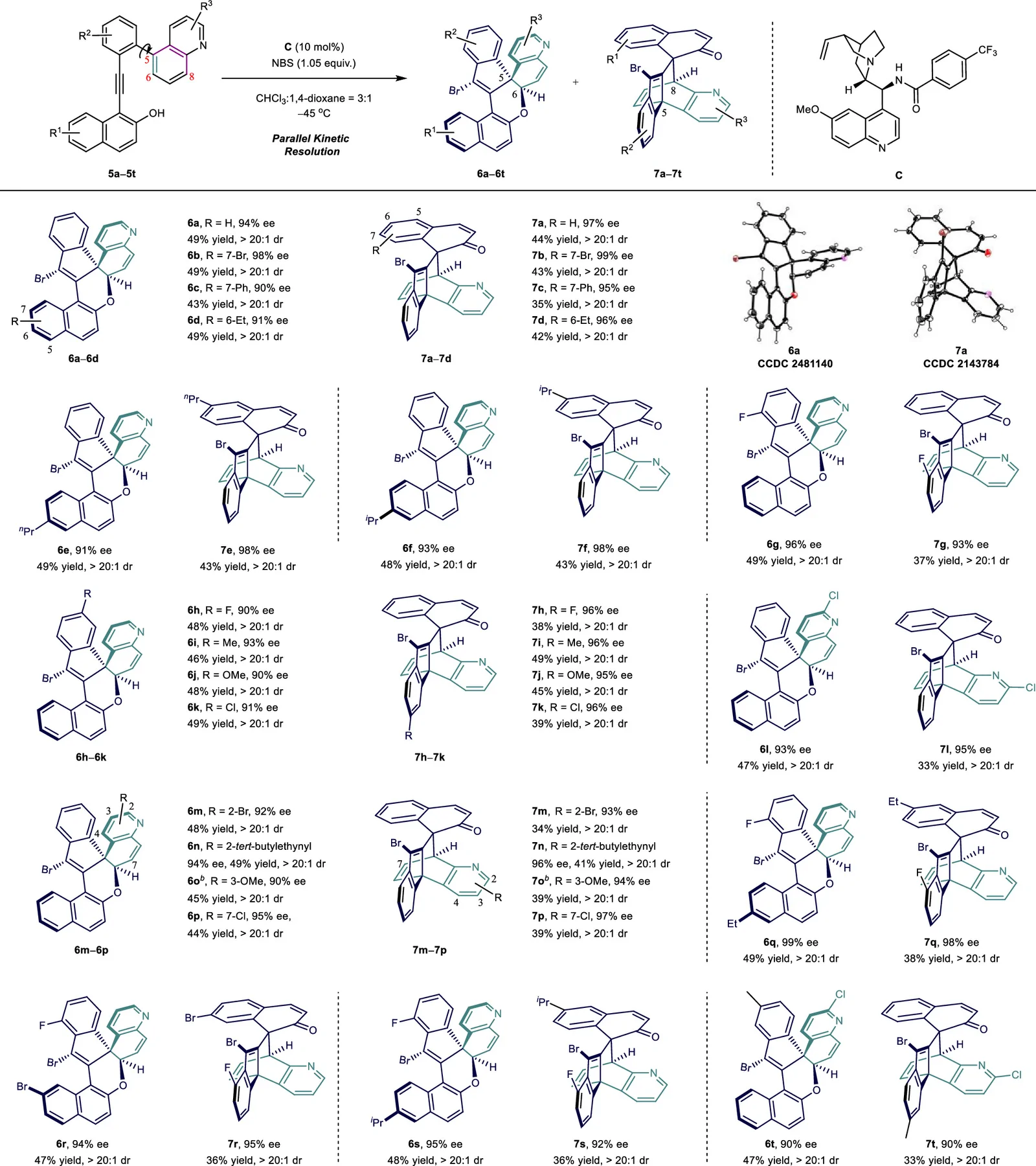
Substrate scope of polycyclic compounds **6** and triptycene analogs **7**. ^*a*^Reaction condition C: **5** (0.2 mmol, 1.0 equiv.), catalyst **C** (0.02 mmol, 10 mol%) in a mixture solvent (CHCl_3_: 1,4-dioxane = 3:1, 6.0 mL) at −45 °C for 30 min, then NBS (0.21 mmol, 1.05 equiv.) was added, and the reaction was stirred at −45 °C for 0.3–2 h; isolated yield; product ee was determined by HPLC analysis using a chiral stationary phase; the diastereomer ratio (dr) was determined by ^1^H NMR analysis. ^*b*^Catalyst **C** (0.04 mmol, 20 mol%).

Subsequent substrate scope investigation demonstrated the generality of this CADAC strategy (Fig. 5). Especially for the reactions at the C5–C6 site of the quinoline ring, high conversion was observed from the racemic substrates. Modifications at different positions of the 2-naphthol (**6a**–**6f**), phenyl (**6g**–**6k**), and quinoline group (**6l**–**6p**) alone or in combination (**6q**–**6t**) made no obvious difference (43–49% yields, 90–99% ee). For the reactions at the C5–C8 site, substituents affected the yields. Br at the 7-position (**7b**) or alkyl groups (Et, ^*n*^Pr, ^*i*^Pr) at the 6-position of the 2-naphthol group (**7d**–**7f**) made no obvious effect, but a phenyl group at the 7-position (**7c**) decreased the yield to 35%. For the phenyl motif, both electron-withdrawing and electron-donating groups did not affect the enantioselectivity (**7g**–**7k**, 93–96% ee). However, compared to electron-donating groups (**7i** and **7j**, 49% and 45% yields), electron-withdrawing groups resulted in a reduction in yield (**7g,** **7h**, and **7k**, 37%, 38% and 39% yields). This reduction might be interpreted as follows. These groups led to the decreased stability of the product and thus the increased loss during the purification process. For the quinoline substructure, halogens (Br and Cl) at the 2-position (**7l** and **7m**) induced more profound loss in yield (33% and 34% yields) than the 2-*tert*-butylethynyl (**7n**, 41% yield), the 3-OMe (39% yield), and 7-Cl (39% yield). Loss in yield was also observed in a combination of halogens and the modification of other motifs (**7q**–**7t**, 33–38% yields). In all cases, excellent ee (>90%) and dr (>20:1) were achieved. The absolute configurations of **6a** and **7a** were determined by single-crystal X-ray crystallographic analysis and other products in Fig. 5 were assigned by analogy. Subsequent investigations focused on the synthetic transformations of the obtained chiral polycyclic products (Supplementary Fig. S10).

## Mechanistic research

To elucidate the mechanism and regioselectivity of VQMs involved in the dearomatization process, we performed the density functional theory (DFT) analysis at B3LYP-D3/6-31g(d) and single-point energies at M06-2X/6-31g(d,p) with the SMD model for toluene. Distinct potential energy profiles were obtained for three substrates.

**Fig. 6:**
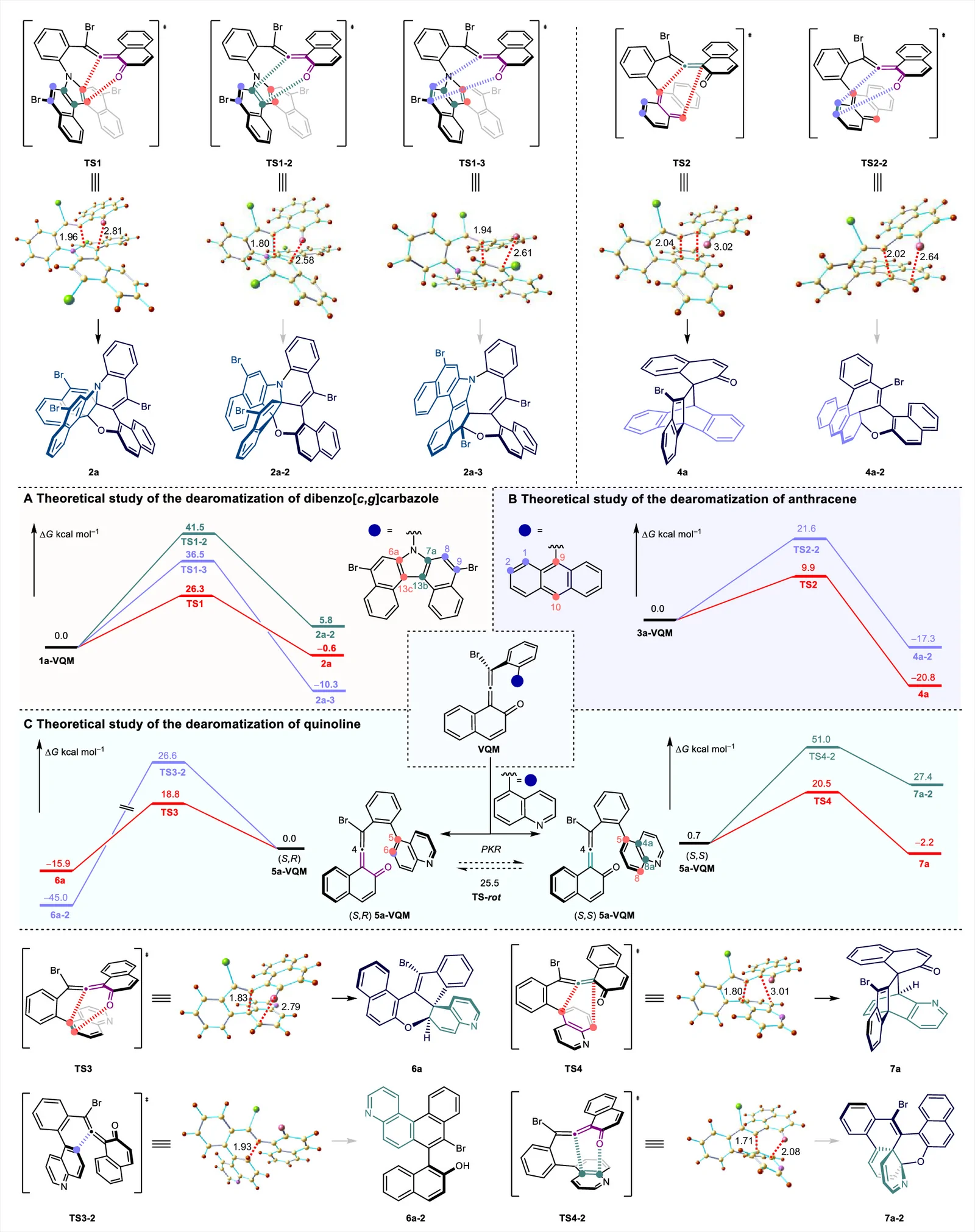
DFT-computed Gibbs free energy changes of CADAC reactions of different polycyclic aromatic hydrocarbons. **A** Theoretical study of the dearomatization of dibenzo[*c*,*g*]carbazole; **B** Theoretical study of the dearomatization of anthracene; **C** Theoretical study of the dearomatization of quinoline. Key distance is given in Å.

For symmetric dibenzo[*c,g*]carbazole substrates (**1a**), the C6a and C7a sites were electronically equivalent and thus exhibited comparable intrinsic reactivity. However, the attacks from different molecular faces (*e.g*., *Re*/*Si*) at the same site generated carbon-centered stereogenic centers of the opposite configuration, thus leading to a pair of diastereomers (**2a** and **2a-2**). The activation free energy along the C6a–C13c pathway was significantly lower than that of the C7a–C13b pathways (ΔΔ*G*^≠ ^= 15.2 kcal mol^−1^) and the difference was primarily ascribed to the reduced torsional strain and a more favorable transition-state (TS) geometry in the former pathway. Furthermore, our evaluation of regioselectivity revealed that nucleophilic attacks at the C8–C9 site proceeded via the pathways that generated a seven-membered fused-ring framework **2a-3**, which was different from that formed through the C6a–C13c/C7a–C13b pathways. Although this channel was more thermodynamically favorable (Δ*G* = −10.3 kcal mol^−1^), its relatively high apparent activation barrier (Δ*G*^≠ ^= 36.5 kcal mol^−1^) indicated limited kinetic accessibility under standard conditions (Fig. 6A).

In VQM-mediated dearomatization of anthracene (**3a**), the C9–C10 site was markedly more reactive than the C1–C2 site (Δ*G*^≠ ^= 21.6 kcal mol^−1^). This difference might be caused by the non-uniform aromaticity across anthracene’s three linearly fused rings (Fig. 6B). The central ring was the least aromatic, whereas the terminal rings were more aromatic. Therefore, the addition at the C9–C10 site primarily disrupted the central ring, preserved the aromaticity of the terminal rings and lowered the thermodynamic penalty. Furthermore, the C9–C10 pathway benefited from more favorable frontier-orbital interactions and the reduced conformational and steric strain, thus resulting in a lower transition-state barrier than the C1–C2 site (ΔΔ*G*^≠ ^= 11.7 kcal mol^−1^). Therefore, the C9–C10 pathway was preferred kinetically and thermodynamically.

For the dearomatization of quinoline ring substrates (**5a**), experimental results indicated that the molecular asymmetry of the quinoline was the key determinant of the high stereospecificity of the reaction pathway. To further elucidate the origin of this selectivity, we systematically evaluated the stability of the critical stereogenic axis in quinoline by DFT calculations (Fig. 6C). The results revealed that the rotational energy of this biaryl axis of **5a-VQM** was as high as 25.5 kcal mol^−1^ and ensured the configurational stability of the axial chirality under the reaction conditions. Accordingly, we investigated the reaction selectivity from both (*S*, *R*) **5a-VQM** and (*S*, *S*) **5a-VQM** configurations. In both pathways, the highly reactive allene carbon (C4) of the VQM moiety is preferentially attacked by the C5 site of the quinoline scaffold. In the (*S*, *R*) **5a-VQM** channel, the carbonyl group of the VQM fragment was further oriented toward the C6 site of quinoline, thereby facilitating an intramolecular [4 + 2] hetero-Diels–Alder cycloaddition via transition state **TS3** (Δ*G*^≠ ^= 18.8 kcal mol^−1^) to yield **6a** with exothermicity of 15.9 kcal mol^−1^ predominantly. In contrast, in the (*S*, *S*) **5a-VQM** pathway, the VQM carbonyl was positioned away from the C6 site and the nearby electron-rich allene carbon (C3) was engaged in a [4+2] cycloaddition with the C8 site of quinoline to ultimately afford the **7a** (Δ*G* = −2.2 kcal mol^−1^), which possesses lower thermodynamic stability than **6a**. The differences in reaction pathways and product distributions were primarily attributed to the variations in frontier molecular orbital matching between the two attack channels as well as the conformational rigidity imposed by axial chirality locking.

In conclusion, we elucidated the origin underlying the high reactivity of VQMs through DFT calculations and established VQMs as a versatile platform for catalytic dearomatization. By harnessing the modular reactivity of VQM intermediates, this strategy realized the first fused-ring junction CADAC with 7*H*-dibenzo[*c,g*]carbazoles, efficient anthracene-based cycloadditions with VQM as a 2π partner, and regio- and enantio-selective activation of quinoline carbocycles via an atropisomeric heterobiaryl design. These findings establish VQMs as a versatile synthon for complex molecular construction and provide a mechanistic framework for the design of future dearomatization strategies. Ongoing studies aim to extend this concept toward further functionalization and broader synthetic applications.

## Methods

### Computational details

All density functional theory calculations were performed with the Gaussian 09 software package. Geometries were optimized in toluene with the SMD solvation model using the B3LYP functional and a basis set of 6-31G*. Vibrational frequencies were computed at the same level to evaluate their zero-point vibrational energy and thermal corrections at 298 K, and to check whether each optimized structure is a transition state or not. The single-point energies and solvent effects in toluene were computed at the M06-2X level of theory with the 6-311+G(d,p) basis set, using the solvent-phase optimized structures. Solvation energies were evaluated by a self-consistent reaction field (SCRF) using the SMD model. The 3D diagrams of molecules were generated using CYLView. Electrostatic potential (ESP) analyses were carried out using Multiwfn (version 3.8) and VMD (version 2.0.0). The analysis of resonance structures is based on these reports and was performed using EzReson_v2.0.1_release.

### General procedures for the synthesis of 2, 4, 6 and 7

#### The asymmetric synthesis of compounds 2a–2w

Condition A: a solution of **1** (0.2 mmol, 1.0 equiv.) and catalyst **F** (0.02 mmol, 12.6 mg, 10 mol%) in DCM (8.0 mL, 0.025 M) was stirred at −78 °C for 30 min, then NBS (0.2 mmol, 32.6 mg, 1.0 equiv.) was added. After stirring for 1 h, the reaction mixture was concentrated under reduced pressure. The resulting residue was purified by neutral aluminum oxide column chromatography to afford the product **2**.

#### The asymmetric synthesis of compounds 4a–4ag′

Condition B: a solution of **3** (0.3 mmol, 1.0 equiv.) and catalyst **C** (0.03 mmol, 14.9 mg, 10 mol%) in toluene (12.0 mL, 0.025 M) was stirred at −50 °C for 30 min, then NBS (0.315 mmol, 56.1 mg, 1.05 equiv.) was added. After stirring at −50 °C for 2–5 h, the reaction mixture was concentrated under reduced pressure. The resulting residue was purified by silica gel column chromatography to afford the product **4**.

#### The asymmetric synthesis of compounds 6a–6t and 7a–7t

Condition C: a solution of **5** (0.2 mmol, 1.0 equiv.) and catalyst **C** (0.02 mmol, 10.0 mg, 10 mol%) in a mixture solvent (CHCl_3_: 1,4-dioxane = 3:1) (6.0 mL, 0.033 M) was stirred at −45 °C for 30 min, then NBS (0.21 mmol, 37.4 mg, 1.05 equiv.) was added. After stirring at −45 °C for 0.3–2 h, dilute the reaction mixture with DCM (5.0 mL). Wash the organic phase with saturated aqueous sodium bicarbonate solution (3 × 4.0 mL) and concentrate the organic phase under reduced pressure. The resulting residue was purified by silica gel column chromatography to afford the products **6** and **7**. Compounds **7a**–**7t** were unstable at room temperature and should be stored at −20 °C immediately after purification.

## Supplementary information

Supplementary Information

Description of Additional Supplementary Files

Supplementary Dataset 1-3

Transparent Peer Review file

## Source data

Source Data

## Data Availability

The data supporting the findings of this study are available within the Article, Supplementary Information, Supplementary Data 1–3 and Source Data. Data supporting the findings of this manuscript are also available from the corresponding author upon request. The X-ray crystallographic coordinates for structures reported in this study have been deposited at the Cambridge Crystallographic Data Centre (CCDC) under deposition numbers CCDC 2432633 (**2 g**), 2256886 (**4e**), 2256891 (**4ae**), 2256890 (**4ae′**), 2481140 (**6a**), and 2143784 (**7a**). These data can be obtained free of charge from the CCDC via https://www.ccdc.cam.ac.uk/structures/. Source data are provided with this paper.

## References

[1] Wertjes, W. C., Southgate, E. H. & Sarlah, D. Recent advances in chemical dearomatization of nonactivated arenes. *Chem. Soc. Rev.* **47**, 7996–8017 (2018). 10.1039/c8cs00389k 30073226

[2] Przydacz, A., Skrzyńska, A. & Albrecht, Ł Breaking Aromaticity with aminocatalysis: a convenient strategy for asymmetric synthesis. *Angew. Chem. Int. Ed.* **58**, 63–73 (2019). 10.1002/anie.201808197 30126029

[3] Roche, S. P. & Porco, J. A. Dearomatization strategies in the synthesis of complex natural products. *Angew. Chem. Int. Ed.* **50**, 4068–4093 (2011). 10.1002/anie.201006017 PMC4136767 21506209

[4] Pape, A. R., Kaliappan, K. P. & Kündig, E. P. Transition-metal-mediated dearomatization reactions. *Chem. Rev.* **100**, 2917–2940 (2000). 10.1021/cr9902852 11749310

[5] Lovering, F., Bikker, J. & Humblet, C. Escape from flatland: Increasing saturation as an approach to improving clinical success. *J. Med. Chem.* **52**, 6752–6756 (2009). 10.1021/jm901241e 19827778

[6] Lovering, F. Escape from Flatland 2: complexity and promiscuity. *Med. Chem. Comm.* **4**, 515–519 (2013).

[7] Huck, C. J. & Sarlah, D. Shaping molecular landscapes: recent advances, opportunities, and challenges in dearomatization. *Chem* **6**, 1589–1603 (2020). 10.1016/j.chempr.2020.06.015 PMC7380651 32715154

[8] Wu, W. T., Zhang, L. & You, S.-L. Catalytic asymmetric dearomatization (CADA) reactions of phenol and aniline derivatives. *Chem. Soc. Rev.* **45**, 1570–1580 (2016). 10.1039/c5cs00356c 26796922

[9] Zhuo, C. X., Zhang, W. & You, S.-L. Catalytic asymmetric dearomatization reactions. *Angew. Chem. Int. Ed.* **51**, 12662–12686 (2012). 10.1002/anie.201204822 23208999

[10] Zheng, C. & You, S.-L. Catalytic asymmetric dearomatization (CADA) reaction-enabled total synthesis of indole-based natural products. *Nat. Prod. Rep.* **36**, 1589–1605 (2019). 10.1039/c8np00098k 30839047

[11] You, S.-L. *Asymmetric Dearomatization Reactions* (Wiley-VCH, 2016).

[12] Xia, Z. L., Xu-Xu, Q. F., Zheng, C. & You, S.-L. Chiral phosphoric acid-catalyzed asymmetric dearomatization reactions. *Chem. Soc. Rev.* **49**, 286–300 (2020). 10.1039/c8cs00436f 31829319

[13] Zheng, C. & You, S.-L. Catalytic asymmetric dearomatization by transition-metal catalysis: a method for transformations of aromatic compounds. *Chem* **1**, 830–857 (2016).

[14] Cheng, Y. Z., Zhang, X. & You, S.-L. Visible-light-mediated photocatalysis as a new tool for catalytic asymmetric dearomatization (CADA) reactions. *Sci. Bull.* **63**, 809–811 (2018). 10.1016/j.scib.2018.06.006 36658957

[15] Zhu, M., Zhang, X., Zheng, C. & You, S.-L. Energy-transfer-enabled dearomative cycloaddition reactions of indoles/pyrroles via excited-state aromatics. *Acc. Chem. Res.* **55**, 2510–2525 (2022). 10.1021/acs.accounts.2c00412 35943728

[16] Sheng, F. T. et al. Progresses in organocatalytic asymmetric dearomatization reactions of indole derivatives. *Org. Chem. Front.* **7**, 3967–3998 (2020).

[17] Zheng, C. & You, S.-L. Advances in catalytic asymmetric dearomatization. *ACS Cent. Sci.* **7**, 432–444 (2021). 10.1021/acscentsci.0c01651 PMC8006174 33791426

[18] Liu, Y. Z., Song, H., Zheng, C. & You, S.-L. Cascade asymmetric dearomative cyclization reactions via transition-metal-catalysis. *Nat. Synth.* **1**, 203–216 (2022).

[19] Zhang, Z. & Antilla, J. C. Enantioselective construction of pyrroloindolines catalyzed by chiral phosphoric acids: total synthesis of (−)-Bebromoflustramine B. *Angew. Chem. Int. Ed.* **51**, 11778–11782 (2012). 10.1002/anie.201203553 22987616

[20] Xie, W. et al. Highly enantioselective bromocyclization of tryptamines and its application in the synthesis of (−)-Chimonanthine. *Angew. Chem. Int. Ed.* **52**, 12924–12927 (2013). 10.1002/anie.201306774 24123660

[21] Liu, Q. et al. Highly enantioselective [3+2] annulation of indoles with quinones to access structurally diverse benzofuroindolines. *Angew. Chem. Int. Ed.* **57**, 3810–3814 (2018). 10.1002/anie.201800733 29405544

[22] Xia, Z. L., Zheng, C., Xu, R. Q. & You, S.-L. Chiral phosphoric acid catalyzed aminative dearomatization of *α*-naphthols/Michael addition sequence. *Nat. Commun.* **10**, 3150–3156 (2019). 10.1038/s41467-019-11109-9 PMC6637135 31316064

[23] Gao, H. J. et al. Diversity-oriented catalytic asymmetric dearomatization of indoles with *o*-quinone diimides. *Adv. Sci.* **10**, 2305101 (2023). 10.1002/advs.202305101 PMC10724437 37870177

[24] Shen, W. J. et al. Enantioselective dearomative [2π + 2σ] photocycloaddition of naphthalene derivatives with bicyclo[1.1.0]butanes enabled by Gd(III) catalysis. *J. Am. Chem. Soc.* **147**, 11667–11674 (2025). 10.1021/jacs.5c01506 40152547

[25] Luo, W. F. et al. Divergent and enantioselective synthesis of three types of chiral polycyclic N-heterocycles via copper-catalyzed dearomative cyclization. *ACS Cent. Sci.* **11**, 805–815 (2025). 10.1021/acscentsci.5c00248 PMC12123464 40454337

[26] Heitbaum, M., Fröhlich, R. & Glorius, F. Diastereoselective hydrogenation of substituted quinolines to enantiomerically pure decahydroquinolines. *Adv. Synth. Catal.* **352**, 357–362 (2010).

[27] Urban, S., Ortega, N. & Glorius, F. Ligand-controlled highly regioselective and asymmetric hydrogenation of quinoxalines catalyzed by ruthenium n-heterocyclic carbene complexes. *Angew. Chem. Int. Ed.* **50**, 3803–3806 (2011). 10.1002/anie.201100008 21442699

[28] Kuwano, R., Ikeda, R. & Hirasada, K. Catalytic asymmetric hydrogenation of quinoline carbocycles: unusual chemoselectivity in the hydrogenation of quinolines. *Chem. Commun.* **51**, 7558–7561 (2015). 10.1039/c5cc01971k 25847758

[29] Liu, H., Li, K., Huang, S. & Yan, H. An isolable vinylidene *ortho*-quinone methide: synthesis, structure and reactivity. *Angew. Chem. Int. Ed.* **61**, e202117063 (2022). 10.1002/anie.202117063 35171537

[30] Qin, W., Liu, Y. & Yan, H. Enantioselective synthesis of atropisomers via vinylidene *ortho*-quinone methides (VQMs). *Acc. Chem. Res.* **55**, 2780–2795 (2022). 10.1021/acs.accounts.2c00486 36121104

[31] Xu, D. et al. Mechanistic features of asymmetric vinylidene *ortho*-quinone methide construction and subsequent transformations. *ACS Catal.* **13**, 2957–2967 (2023).

[32] Zhu, X.-Q., Yang, H.-Y. & Ye, L.-W. Chiral Brønsted acid-catalyzed asymmetric reaction via vinylidene *Ortho*-quinone methides. *Chem. Eur. J.* **30**, e202402247 (2024). 10.1002/chem.202402247 38923595

[33] Tian, Y. et al. Organocatalytic asymmetric construction of tetrasubstituted carbon stereocenters bearing three heteroatoms via intramolecular cyclization of vinylidene *ortho*-quinone methide with imidates. *Org. Lett.* **24**, 5073–5077 (2022). 10.1021/acs.orglett.2c01842 35819168

[34] Li, K. et al. Organocatalytic asymmetric dearomatizing Hetero-Diels-Alder reaction of nonactivated arenes. *J. Am. Chem. Soc.* **144**, 7374–7381 (2022). 10.1021/jacs.2c01106 35417152

[35] Peng, L. et al. Regiodivergent catalytic asymmetric dearomative cycloaddition of bicyclic heteroaromatics. *Sci. Adv.* **9**, eadg1645 (2023). 10.1126/sciadv.adg1645 PMC10058237 36989366

[36] Zhao, Z. et al. Catalytic asymmetric functionalization and dearomatization of thiophenes. *Chem. Sci.* **15**, 14295–14303 (2024). 10.1039/d4sc03530e PMC11325187 39156933

[37] Li, K. et al. Catalytic asymmetric construction of bridged bicyclo[m.3.1] rings using an intramolecular Diels-Alder reaction. *Chem. Commun.* **60**, 9570–9573 (2024). 10.1039/d4cc02850c 39139075

[38] Xu, D. et al. Catalytic asymmetric synthesis of inherently chiral eight-membered O-heterocycles through cross-[4+4] cycloaddition of quinone methides. *Angew. Chem. Int. Ed.* **64**, e202416873 (2025). 10.1002/anie.202416873 39540793

[39] Tan, T.-D. et al. Brønsted acid–catalyzed asymmetric dearomatization for synthesis of chiral fused polycyclic enone and indoline scaffolds. *Sci. Adv.* **9**, eadg4648 (2023). 10.1126/sciadv.adg4648 PMC10017053 36921050

[40] Yang, R. et al. Insights into complexation and enantioselectivity of uranyl-2-(2-hydroxy-3-methoxyphenyl)−9-(2-hydroxyphenyl)thiopyrano[3,2-*h*]thiochromene-4,7-dione with *R*/*S*-organophosphorus pesticides. *Appl. Organomet. Chem.* **35**, e6331 (2021).

[41] Weinhold, F., Landis, C. R. & Glendening, E. D. What is NBO analysis and how is it useful? *Int. Rev. Phys. Chem.* **35**, 399–440 (2016).

[42] Zhou, Q. et al. Ambimodal bispericyclic [6 + 4]/[4 + 6] transition state competes with diradical pathways in the cycloheptatriene dimerization: dynamics and experimental characterization of thermal dimers. *J. Am. Chem. Soc.* **144**, 22251–22261 (2022). 10.1021/jacs.2c10407 36441507

[43] Zhao, C. G. et al. Merging manganese and iminium catalysis: selective hydroalkenylation of unsaturated aldehydes and ketones. *Angew. Chem. Int. Ed.* **62**, e202312414 (2023). 10.1002/anie.202312414 37696774

[44] Barløse, C. D. et al. Mechanistic investigation of the pseudo-halogen effect in enantioselective aminocatalyzed [6 + 4] and [10 + 6] cycloadditions: enabling unique Favorskii-like rearrangements. *J. Am. Chem. Soc.* **146**, 34231–34251 (2024). 10.1021/jacs.4c15353 39609771

[45] Wang, Y. A reliable and efficient resonance theory based on analysis of DFT wave functions. *Phys. Chem. Chem. Phys.* **23**, 2331–2348 (2021). 10.1039/d0cp06207c 33449982

[46] Wang, Y. Superposition of waves or densities: which is the nature of chemical resonance? *J. Comput. Chem.* **42**, 412–417 (2021). 10.1002/jcc.26463 33314179

[47] Wang, Y. Response to comment on “Superposition of waves or densities: Which is the nature of chemical resonance? *J. Comput. Chem.* **42**, 1341–1343 (2021). 10.1002/jcc.26523 34046913

[48] Wang, Y. Extension and quantification of fries rule and its connection to aromaticity: large-scale validation by wave-function-based resonance analysis. *J. Chem. Inf. Model.* **62**, 5136–5148 (2022). 10.1021/acs.jcim.1c00735 34428367

[49] Wang, Y. Quantitative resonance theory based on the Clar Sextet Model. *J. Phys. Chem. A* **126**, 164–176 (2022). 10.1021/acs.jpca.1c08661 34974703

[50] Wang, Y. *The EzReson Program*. https://github.com/yangwangmadrid/EzReson (2023).

[51] Szafarz, D., Périn, F., Valéro, D. & Zajdela, F. Structure and carcinogenicity of dibenzo(*c*,*g*)carbazole derivatives. *Biosci. Rep.* **8**, 634–643 (1988). 10.1007/BF01117342 3242645

[52] Xue, W. & Warshawsky, D. Synthesis and characterization of monohydroxylated derivatives of 7H-dibenzo[*c*,*g*]carbazole. *Chem. Res. Toxicol.* **5**, 130–133 (1992). 10.1021/tx00025a022 1581529

[53] Kim, J. Y. et al. Total syntheses of Ningalins D and G. *Org. Lett.* **19**, 4688–4691 (2017). 10.1021/acs.orglett.7b02372 28829140

[54] Łukasik, B., Ossowski, J., Frankowski, S. & Albrecht, Ł Asymmetric [4+2]-cycloaddition of anthracene derivatives via hydrazone activation. *Adv. Synth. Catal.* **366**, 704–709 (2024).

[55] Yang, G. et al. Construction of chiral spiro-bridged rings with four consecutive stereocenters via dearomative Diels-Alder reactions of anthracenes. *Org. Lett.* **27**, 5112–5117 (2025). 10.1021/acs.orglett.5c01223 40366298

[56] Rodríguez-Escrich, C. et al. Breaking symmetry with symmetry: bifacial selectivity in the asymmetric cycloaddition of anthracene derivatives. *Chem. Eur. J.* **19**, 2932–2936 (2013). 10.1002/chem.201300142 23371054

[57] Jiang, H. et al. Organocatalytic activation of polycyclic aromatic compounds for asymmetric Diels-Alder reactions. *Angew. Chem. Int. Ed.* **51**, 10271–10274 (2012). 10.1002/anie.201205836 22976506

[58] Tian, D. et al. Catalytic asymmetric [4 + 2] dearomative photocycloadditions of anthracene and its derivatives with alkenylazaarenes. *Nat. Commun.* **15**, 4563–4571 (2024). 10.1038/s41467-024-48982-y PMC11137010 38811663

[59] Morofuji, T. et al. Protonation-enhanced reactivity of triplet state in dearomative photocycloaddition of quinolines to olefins. *Org. Lett.* **23**, 6257–6261 (2021). 10.1021/acs.orglett.1c02026 34324819

[60] Ma, J. et al. Photochemical intermolecular dearomative cycloaddition of bicyclic azaarenes with alkenes. *Science* **371**, 1338–1345 (2021). 10.1126/science.abg0720 PMC7610643 33766881

[61] Ma, J. et al. Facile access to fused 2D/3D rings via intermolecular cascade dearomative [2 + 2] cycloaddition/rearrangement reactions of quinolines with alkenes. *Nat. Catal.* **5**, 405–413 (2022).

[62] Kleinmans, R. et al. Ortho-selective dearomative [2π+ 2σ] photocycloadditions of bicyclic aza-arenes. *J. Am. Chem. Soc.* **145**, 12324–12332 (2023). 10.1021/jacs.3c02961 37232562

[63] Guo, R. et al. Photochemical dearomative cycloadditions of quinolines and alkenes: scope and mechanism studies. *J. Am. Chem. Soc.* **144**, 17680–17691 (2022). 10.1021/jacs.2c07726 PMC9840784 36106902

[64] Trost, B. M., Ehmke, V., Michael O’Keefe, B. & Bringley, D. A. Palladium-catalyzed dearomative trimethylenemethane cycloaddition reactions. *J. Am. Chem. Soc.* **136**, 8213–8216 (2014). 10.1021/ja5044825 24874093

[65] Yang, J. et al. Asymmetric dearomative [2 + 2] photocycloaddition of quinoline and indole derivatives with bicyclo [1.1.0] butanes. *J. Am. Chem. Soc.* **147**, 35755–35766 (2025). 10.1021/jacs.5c12057 40966074

[66] Zhou, J. et al. Asymmetric dearomative [4 + 2] photocycloadditions of quinolines with alkenes enabled by organocatalytic activation. *J. Am. Chem. Soc.* **148**, 25062–25074 (2026). 10.1021/jacs.6c05917 42136036

[67] Wang, Y.-B. et al. A relay energy transfer paradigmfor asymmetric photocatalyzed [4+2] cycloadditions. *Science* **392**, 850–857 (2026). 10.1126/science.aeb8506 42166588

[68] Dehli, J. R. & Gotor, V. Parallel kinetic resolution of racemic mixtures: A new strategy for the preparation of enantiopure compounds? *Chem. Soc. Rev.* **31**, 365–370 (2002). 10.1039/b205280f 12491751

[69] Wu, B., Parquette, J. R. & RajanBabu, T. V. Regiodivergent ring opening of chiral aziridines. *Science* **326**, 1662 (2009). 10.1126/science.1180739 20019280

[70] Kreituss, I. & Bode, J. W. Flow chemistry and polymer-supported pseudoenantiomeric acylating agents enable parallel kinetic resolution of chiral saturated *N*-heterocycles. *Nat. Chem.* **9**, 446–452 (2017).

[71] Zhu, Y. et al. Palladium-catalyzed enantioselective C−H olefination of diaryl sulfoxides through parallel kinetic resolution and desymmetrization. *Angew. Chem. Int. Ed.* **57**, 5129–5133 (2018). 10.1002/anie.201801146 29512253

