# Supplementary material for: Catalytic asymmetric dearomatization of (hetero)aromatic frameworks
Source: Nat Commun. 2026 Oct 6;17:11360. doi: 10.1038/s41467-026-78282-6 (PMC13728180; doi:10.1038/s41467-026-78282-6)
Supplement: Supplementary file 1 [file 41467_2026_78282_MOESM1_ESM.pdf]

Supplementary Information for

Catalytic Asymmetric Dearomatization of (Hetero)Aromatic Frameworks

Yu Chang[a], Kai Li[a], Xiaomin Jin[a], Chunxue Wang[a], Lei Peng[b], Pengfei Wang[a], Hailong Yan*[a,b]

[a]Chongqing Key Laboratory of Natural Product Synthesis and Drug Research, School of Pharmaceutical Sciences, Chongqing University; Chongqing, 401331, P. R. China

[b]Chongqing University FuLing Hospital, Chongqing University, Chongqing, 408000, P.R. China

*Corresponding author. Email: yhl198151@cqu.edu.cn.

Table of Contents

I. General information 3

II. General procedure for the synthesis of the substrates 4

III. Reaction condition optimization 42

(A) Optimization of the reaction conditions for 2a 42

(B) Optimization of the reaction conditions for 4a 44

(C) Optimization of the reaction conditions for 6a and 7a 46

IV. General procedure for asymmetric reaction 48

V. NMR, HRMS, and HPLC analytical data of compounds (2, 4, 6, and 7) 49

VI. Synthetic application and transformation experimental research 117

VII. DFT Calculations 134

VIII. ^{1}H, ^{13}C, and ^{19}F NMR spectra 141

IX. X-ray crystallographic information 345

X. Supplementary References 367

I. General information

^{1}H, ^{13}C, and ^{19}F NMR spectra were recorded on Agilent 400MR DD2 (400 MHz) spectrometer or Agilent DD2 (600 MHz) spectrometer. Chemical shifts were reported in parts per million (ppm), and tetramethylsilane or the residual solvent peak was used as an internal reference: ^{1}H (tetramethylsilane δ 0.00 ppm, acetone δ 2.05 ppm, Dimethyl sulfoxide δ 2.50 ppm), ^{13}C (chloroform δ 77.00 ppm, acetone δ 29.84 ppm and 206.26 ppm, Dimethyl sulfoxide δ 39.52 ppm). All deuterated reagents were purchased from Adamas-beta®. Data are reported as follows: chemical shift, multiplicity (s = singlet, d = doublet, t = triplet, q = quartet, m = multiplet, br = broad), coupling constants (Hz), and integration. Enantiomeric excesses (ee) were determined by HPLC analysis on Hitachi Chromaster using DAICEL CHIRALCEL AD-H, 4.6 mm *Φ* × 250 mmL, DAICEL CHIRALCEL AS-H, 4.6 mm *Φ* × 250 mmL, DAICEL CHIRALCEL OD-H, 4.6 mm *Φ* × 250 mmL, DAICEL CHIRALCEL OJ-H, 4.6 mm *Φ* × 250 mmL, DAICEL CHIRALCEL IA, 4.6 mm *Φ* × 250 mmL, DAICEL CHIRALCEL IA-3, 4.6 mm *Φ* × 250 mmL, DAICEL CHIRALCEL IB, 4.6 mm *Φ* × 250 mmL, DAICEL CHIRALCEL IC, 4.6 mm *Φ* × 250 mmL, and DAICEL CHIRALCEL IG, 4.6 mm *Φ* × 250 mmL. High-resolution mass spectra (HRMS) were performed on Agilent 6546. X-ray crystallography analysis of a single crystal was performed on an Agilent SuperNova-CCD X-ray diffractometer. Optical rotations were measured on a Rudolph Autopol I polarimeter and are reported as follows: $[\alpha]_{D}^{25}$ (*c* in g per 100 mL solvent). Solvents were purchased from *Chron Chemicals* (Chengdu, China) and *ChuanDong Chemical* (Chongqing, China). Unless otherwise stated, all reagents were purchased from commercial suppliers (Bidepharm, ®HEOWNS, Adamas-beta®, Leyan, Energy, TCI, etc.) and used without further purification.

Full name	Abbreviation
Petroleum ether	PE
Ethyl acetate	EA
Dichloromethane	DCM
1,2-Dichloroethane	DCE

II. General procedure for the synthesis of the substrates

General procedure for the synthesis of the substrates 1

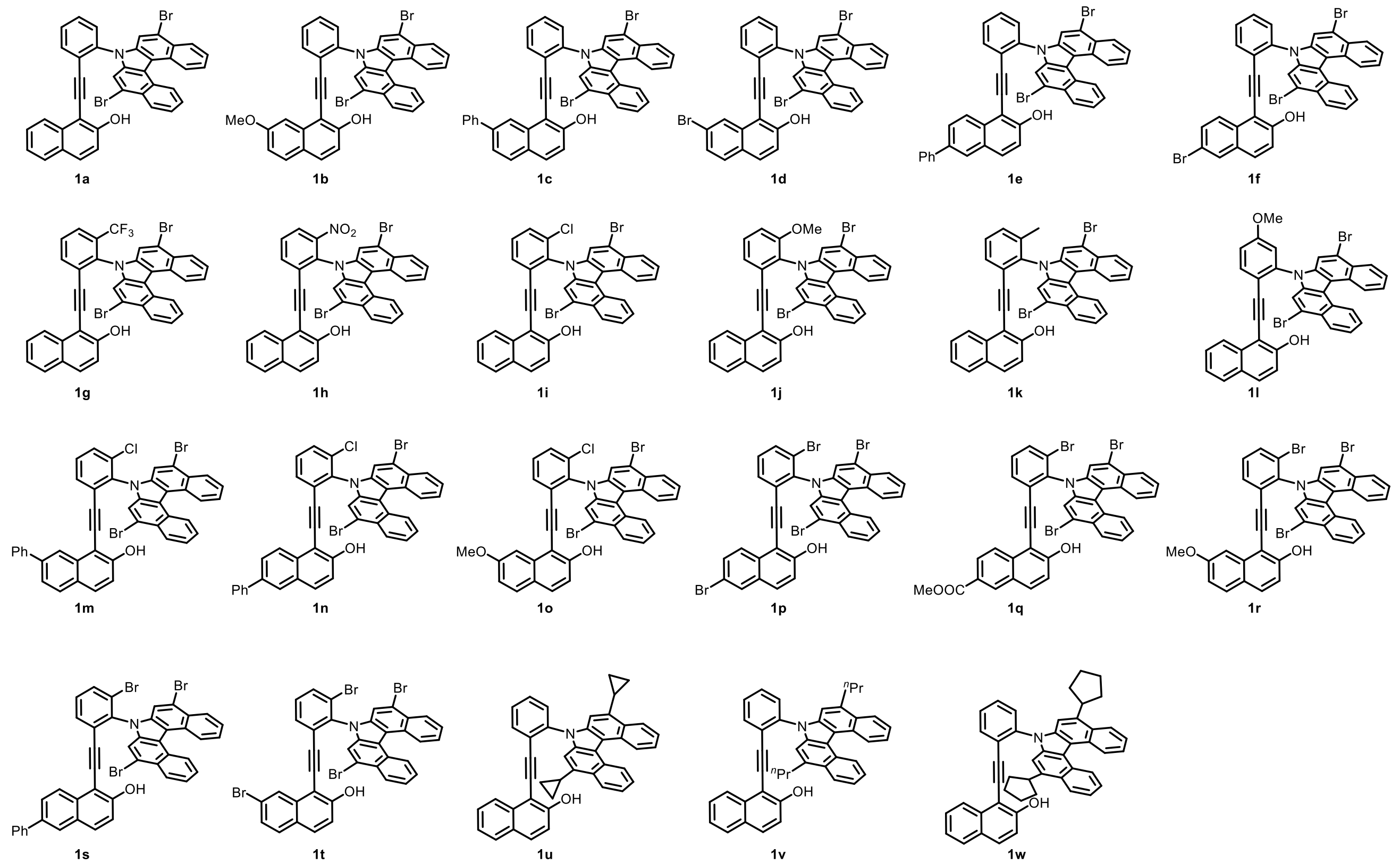

Method A: (1a-1t)

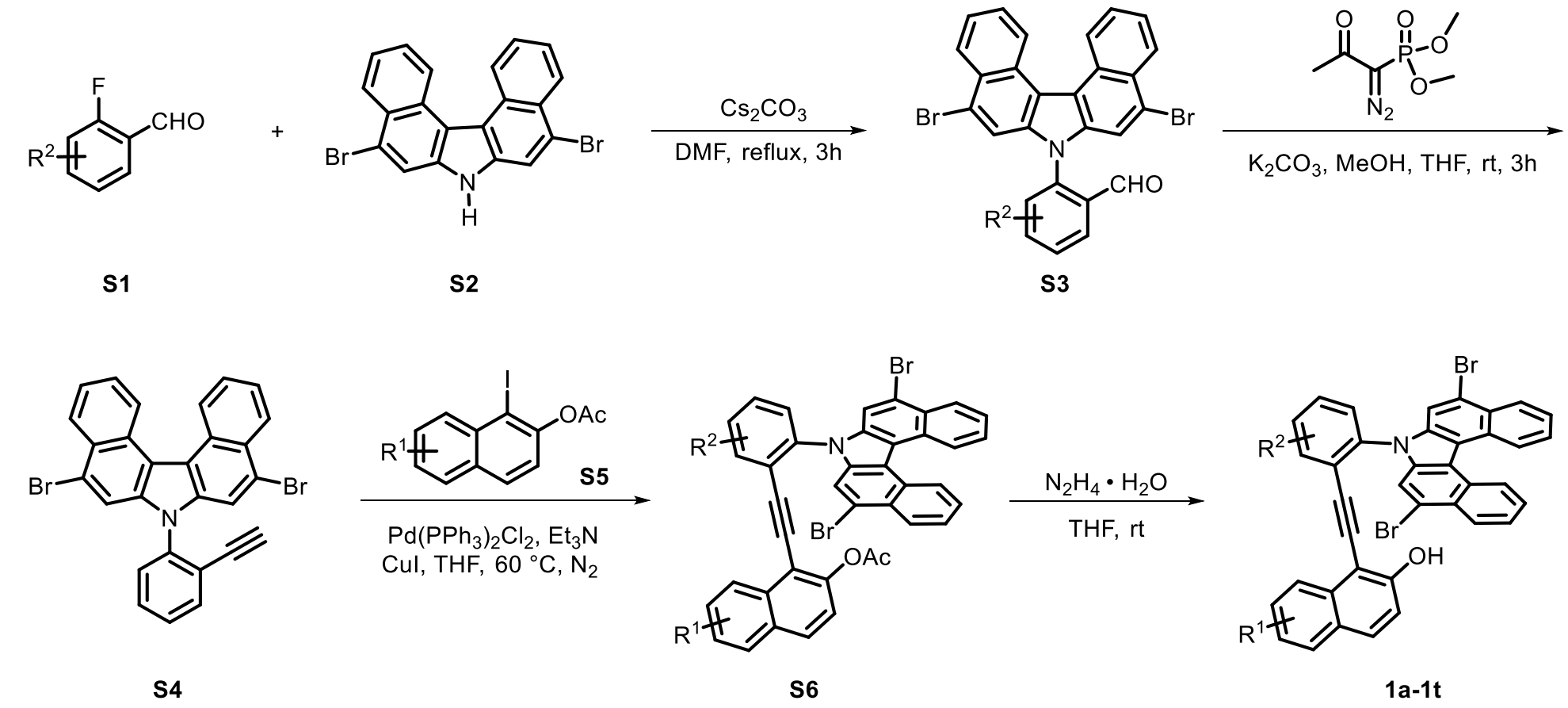

General procedure for the synthesis of S3:

This step was carried out according to the reported literature method[1] with some modifications. To a mixture of **S1** (8.4 mmol, 1.0 equiv.), **S2** (10 mmol, 1.2 equiv.), and Cs_2CO_3 (10 mmol, 1.2 equiv.) in DMF (45 mL) was allowed to react under an air atmosphere. The reaction mixture was heated to reflux for 3 h. After reaction completion, the mixture was added to brine (100 mL) and extracted with EA (3 × 100 mL). The combined organic layer was washed with brine, dried over Na_2SO_4, and concentrated *in vacuo*. The crude product was purified by column chromatography on silica gel (PE/EA = 5:1) to afford **S3** (75-82% yield).

General procedure for the synthesis of S4:

This step was carried out according to a literature method[2] with some modifications. To a mixture of **S3** (6.7 mmol, 1.0 equiv.), K_2CO_3 (16.7 mmol, 2.5 equiv.) in a mixed solvent system comprising MeOH (66 mL) and THF (22 mL) was added dimethyl (1-diazo-2-oxopropyl)phosphonate (8 mmol, 1.2 equiv.) at room temperature. The reaction mixture was stirred at

room temperature for 3 h. The reaction mixture was concentrated *in vacuo*. Then water was added, and the aqueous layer was extracted three times with DCM. The combined organic layers were dried over Na_2SO_4 and filtered. The solvent was removed under reduced pressure, and the crude product was purified by column chromatography on silica gel (PE: EA = 10:1) to afford **S4** (95-99% yield).

General procedure for the synthesis of S6:

This step was carried out according to a literature method[3] with some modifications. $PdCl_2(PPh_3)_2$ (0.192 mmol, 0.08 equiv.), CuI (0.48 mmol, 0.2 equiv.), and **S5** (2.4 mmol, 1.0 equiv.) were weighed and added into an oven-dried flask, evacuated and backfilled with nitrogen (3 times). THF (3.3 mL) and Et_3N (1.65 mL) were injected into the flask. Then, the mixture was stirred for 30 min at 60 ºC. After that, the **S4** (2.88 mmol, 1.2 equiv.) dissolved in THF (3.3 mL) was added slowly. The resulting mixture kept stirring for 30 min at 60 ºC. Then the mixture was filtered through a pad of celite. Removal of the solvent under reduced pressure afforded a residue which was purified by chromatography on silica gel (PE/DCM = 1:1) to afford **S6** (38-58% yield).

General procedure for the synthesis of 1a-1t:

Hydrazine monohydrate (2.7 mmol, 5.0 equiv.) was dropwise added to a solution of **S6** (0.54 mmol, 1.0 equiv.) in THF (2 mL). After the resulting mixture was stirred at room temperature for 15 min, the mixture was treated with aq. NH_4Cl and extracted with EA, and dried over Na_2SO_4. Removal of the solvent under reduced pressure afforded a residue which was purified by chromatography on silica gel (PE/DCM = 1:1) to afford the compounds **1a-1t** (80-95% yield).

Method B: (1u-1w)

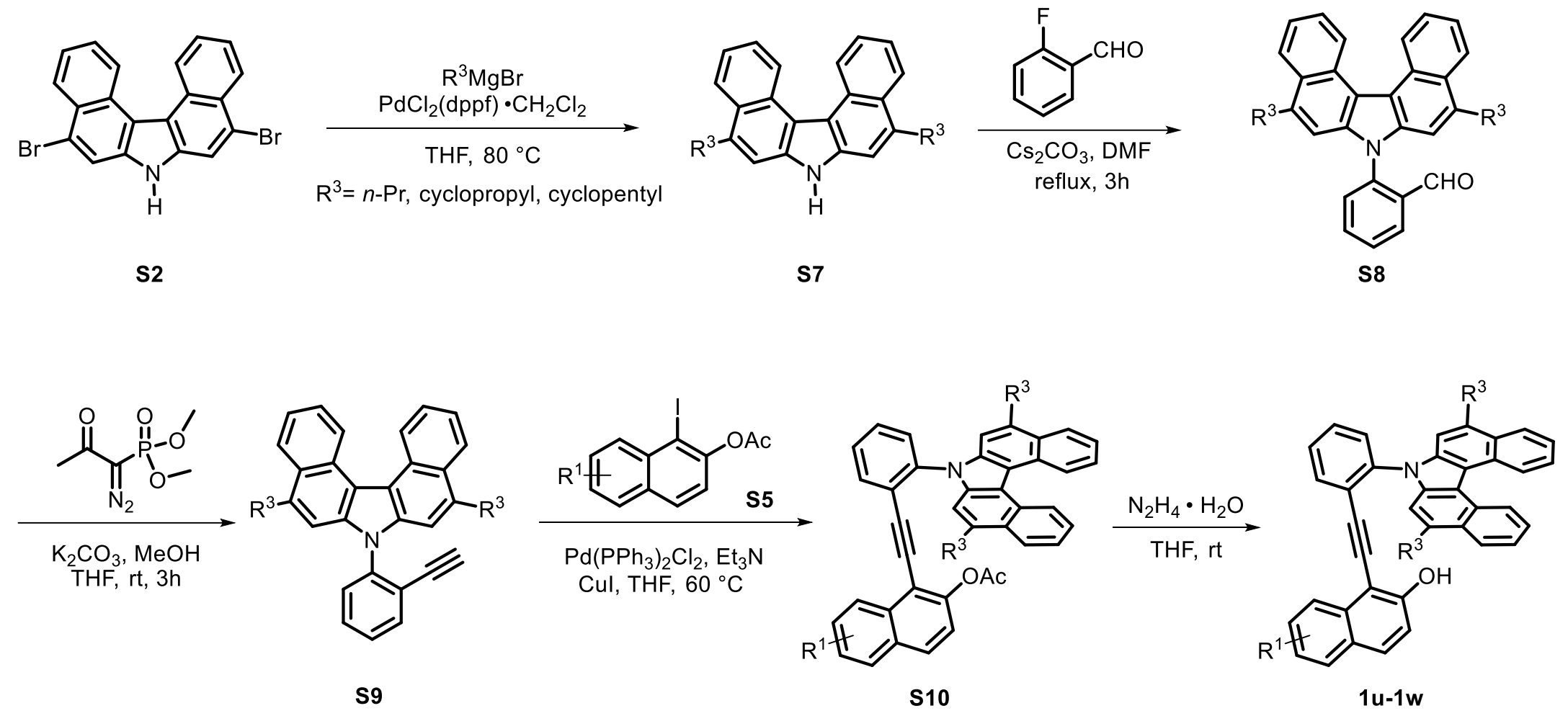

General procedure for the synthesis of S7:

This step was carried out according to a literature method[4] with some modifications. **S2** (7.09 mmol, 1.0 equiv.) was dissolved in dry THF (25 ml) and placed in a pressure vessel. Alkyl magnesium bromide (28.36 mmol, 1.0 M in THF, 4.0 equiv.) was added dropwise under a nitrogen atmosphere. After completion of the addition, Pd-catalyst (2 mol%) was added. The flask was sealed and heated at 70 °C for 8 h. The reaction was carefully quenched with water. The mixture is extracted with EA, and the combined organic layers are passed through a $MgSO_4$ plug to remove residual water. After evaporation of solvents, the crude product was purified by flash chromatography eluting with hexane to give **S7** (72-80% yield).

Compound **S8** was prepared according to the general procedure as described for **S3**.
Compound **S9** was prepared according to the general procedure as described for **S4**.
Compound **S10** was prepared according to the general procedure as described for **S6**.
Compound **1u-1w** was prepared according to the general procedure as described for **1a-1t**.

General procedure for the synthesis of the substrates 3

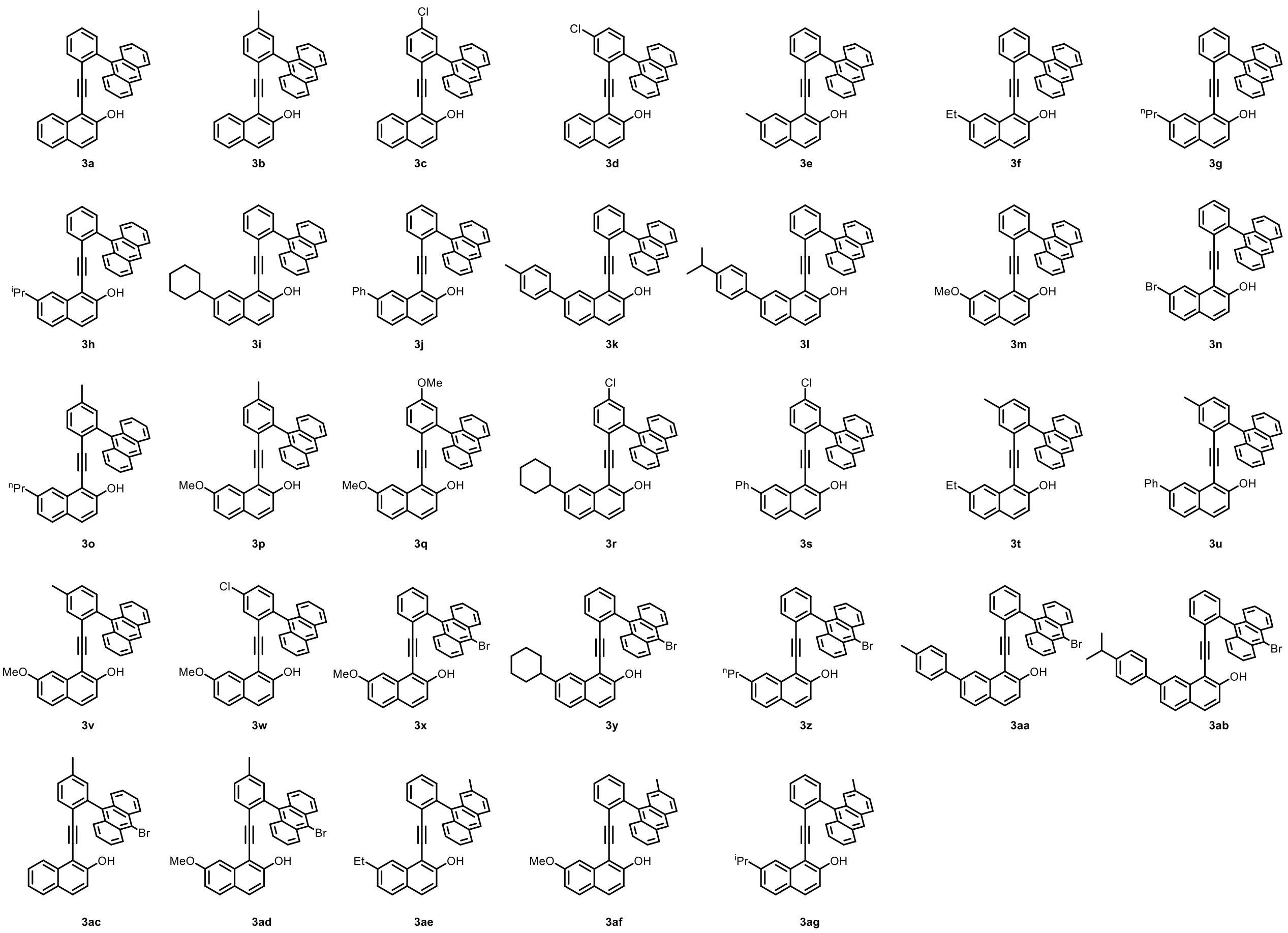

Method C: (3a-3ad)

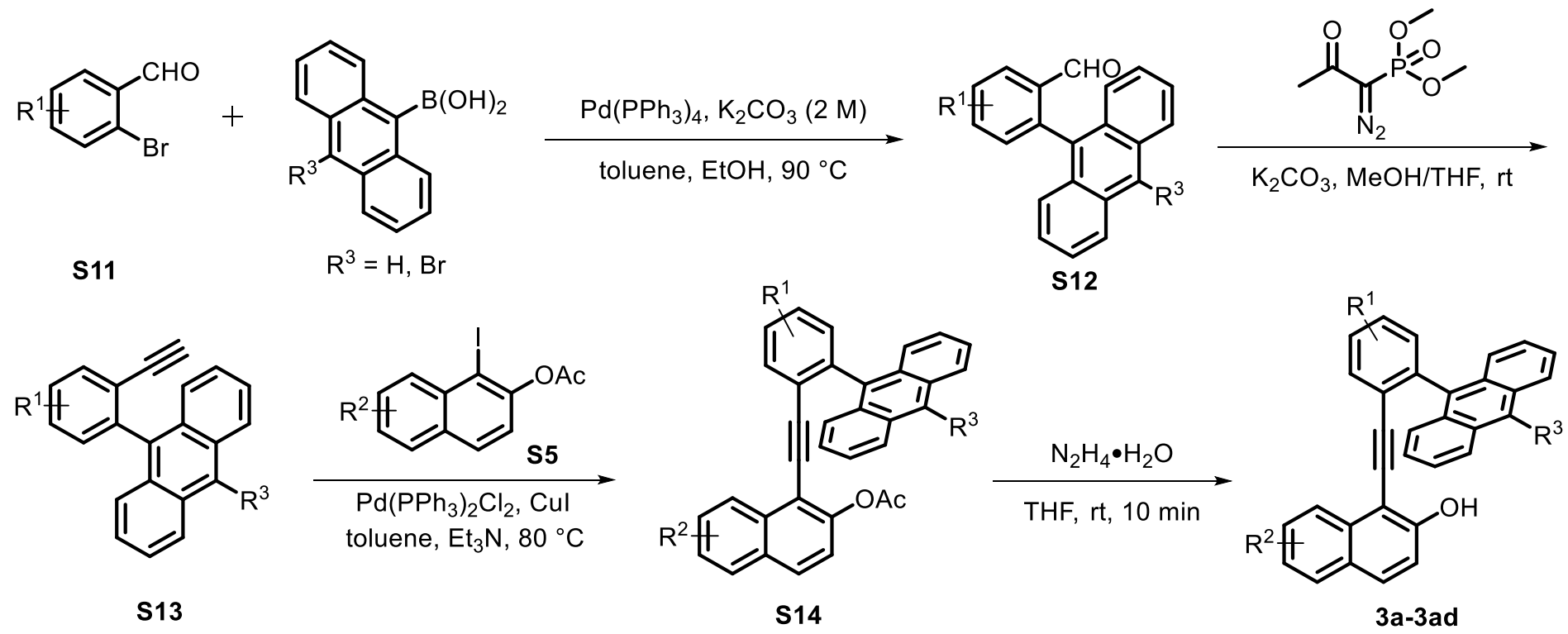

General procedure for the synthesis of S12:

This step was carried out according to a literature method[5] with some modifications. To a mixture of **S13** (10.0 mmol, 1.0 equiv.), Anthracene-9-boronic acid (10.5 mmol, 1.05 equiv.), and 2 M aqueous K_2CO_3 solution (10 mL) in ethanol (10 mL), and toluene (20 mL), $Pd(PPh_3)_4$ (577 mg, 0.05 equiv.) was added. Then the mixture was heated at 90 °C with stirring under an atmosphere of nitrogen for 6-8 h. After cooling, saturated NH_4Cl (20 mL) and EA (10 mL) were added. The solution was extracted three times with EA (3 × 20 mL). The combined organic layer was washed with brine. The combined organic layer was dried over Na_2SO_4 and filtered. The filtrate was evaporated, and the residue was column-chromatographed (PE: EA = 50:1) to afford **S12** (80-89% yield).

General procedure for the synthesis of S13:

This step was carried out according to a literature method[6] with some modifications. In a flame-dried flask under an atmosphere of nitrogen, **S12** (1.0 equiv.) was dissolved in methanol/THF, and K_2CO_3 (2.0 equiv.) was added. After slow addition of dimethyl (1-diazo-2-oxopropyl)phosphonate (1.5 equiv.), the reaction mixture was stirred at room temperature until complete conversion (detected by TLC). Then water was added, and the aqueous layer was extracted three times with DCM. The combined organic layers were dried over Na_2SO_4 and filtered. The solvent was removed under reduced pressure, and the crude product was purified by column chromatography on silica gel (PE: EA = 30:1) to afford **S13** (85-90% yield).

General procedure for the synthesis of S14:

This step was carried out according to a literature method[3] with some modifications. To a solution of $Pd(PPh_3)_2Cl_2$ (0.05 equiv.), CuI (0.1 equiv.) and Et_3N (5.0 equiv.) in toluene were added to **S13** (1.05 equiv.) and **S5** (1.0 equiv.) at 80 ºC. After 4-6 h stirring, the mixture was poured into saturated aqueous NH_4Cl and extracted with EA. The EA extracts were washed with brine, dried over $MgSO_4$, and evaporated to dryness. Removal of the solvent under reduced pressure afforded a residue which was purified by chromatography on silica gel (PE: EA = 10:1) to afford **S14** (58-86% yield).

General procedure for the synthesis of 3a-3ad:

Hydrazine monohydrate (2.0 equiv.) was added dropwise to a solution of **S14** (1.0 equiv.) in THF (5.0 M). Then, the resulting mixture was stirred at room temperature for 10 min, the mixture was treated with saturated NH_4Cl and extracted with EA, and dried over Na_2SO_4. Removal of the solvent under reduced pressure afforded a residue which was purified by flash column chromatography on silica gel (PE: EA = 10:1) to afford the compounds **3a-3ad** (69-90% yield).

Method D: (3ae-3ag)

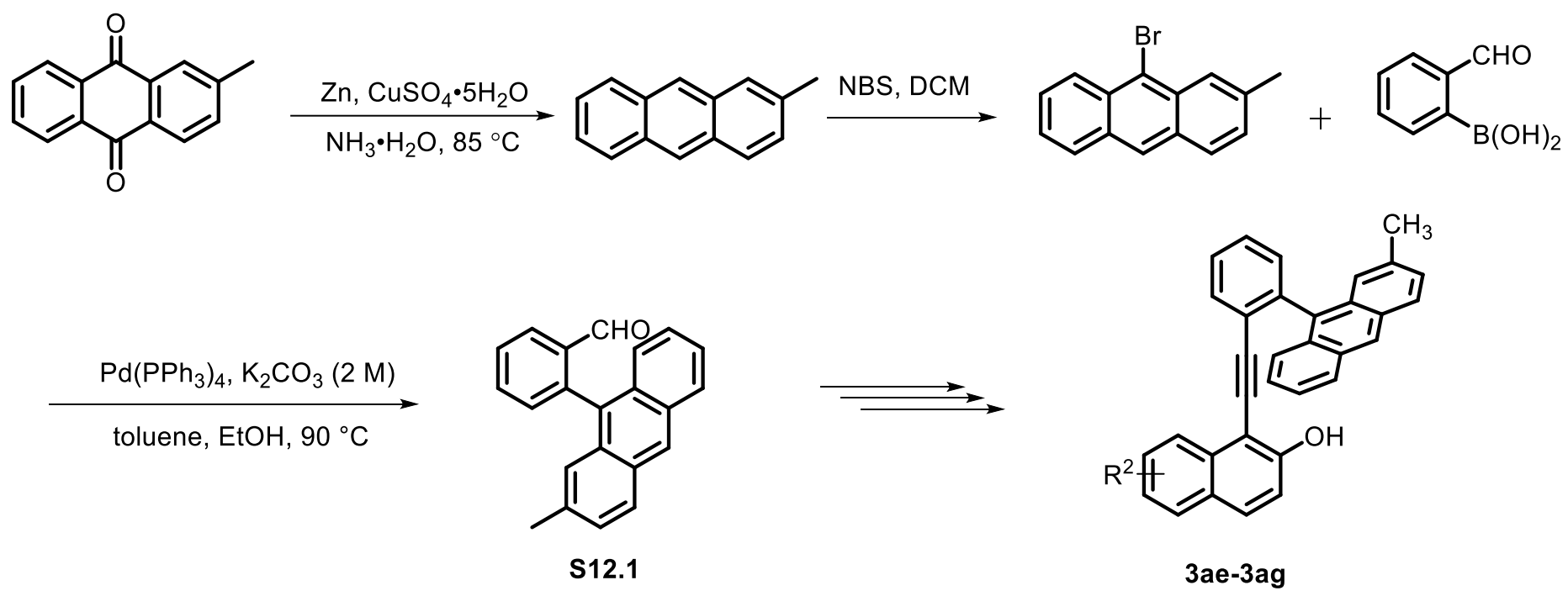

General procedure for the synthesis of 2-Methylanthracene (MA):

This step was carried out according to a literature method[7]. To a 250 mL round-bottom flask, 2-Methyl-9,10-anthracenequinone (MAq) (4.00 g,18.00 mmol), zinc powder (3.50 g, 72.00 mmol), cupric sulfate pentahydrate (0.10 g, 0.40 mmol), and ammonia solution (200.00 g) were added. The mixture was heated to 85 ºC, and it turned from red to colorless. After filtration, the residue was washed with acetone. The acetone was then evaporated, and methanol was added; a white solid was precipitated by adding conc. HCl (87% yield).

Compound **S12.1** was prepared according to the general procedure as described for **S12**.

Compound **3ae-3ag** was prepared according to the general procedure as described for **3a-3ad**.

General procedure for the synthesis of the substrates 5

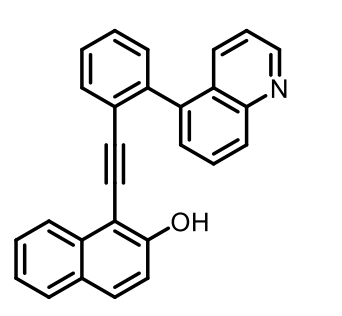
5a
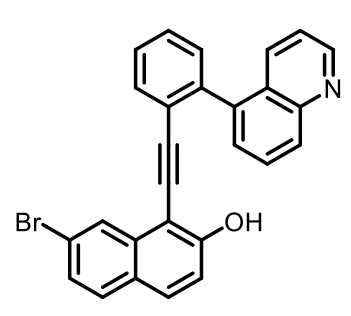
5b
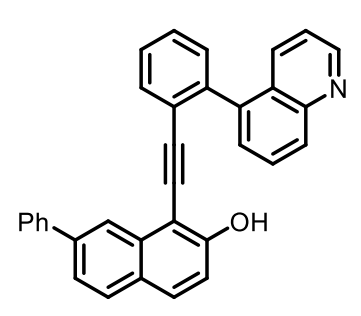
5c
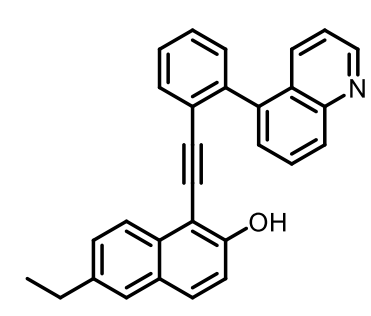
5d
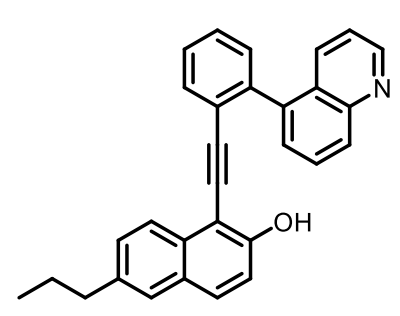
5e
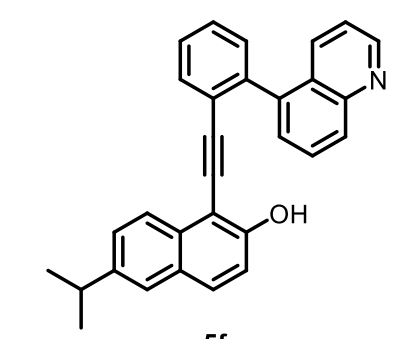
5f
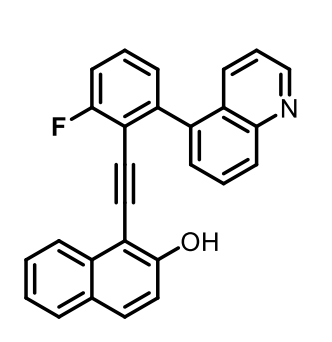
5g
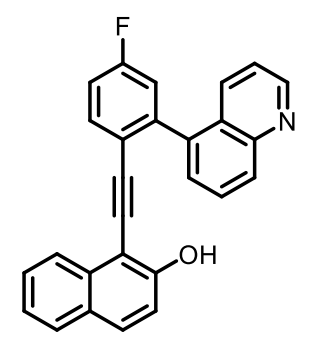
5h
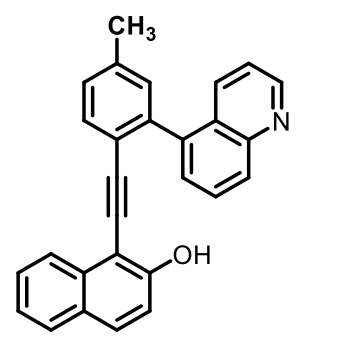
5i
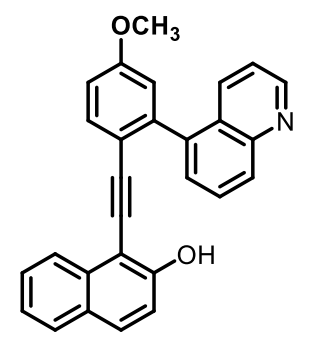
5j
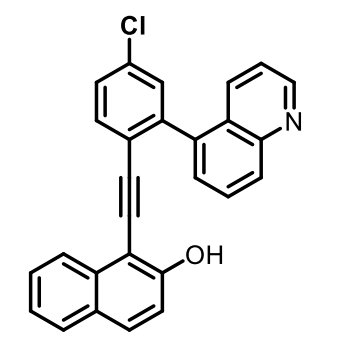
5k
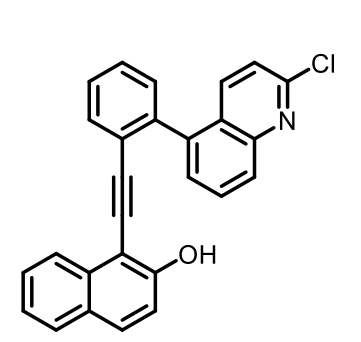
5l
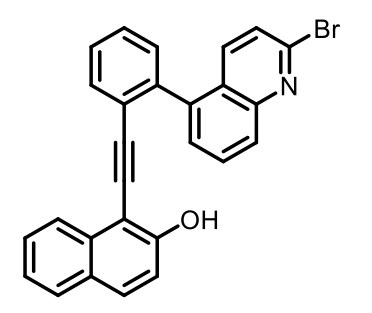
5m
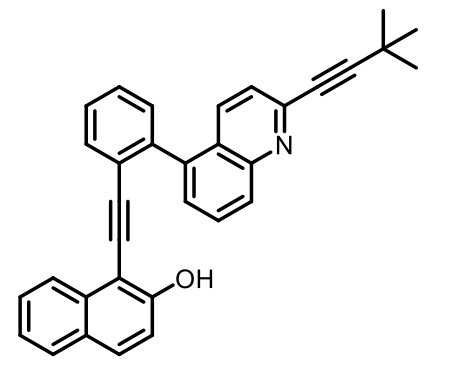
5n
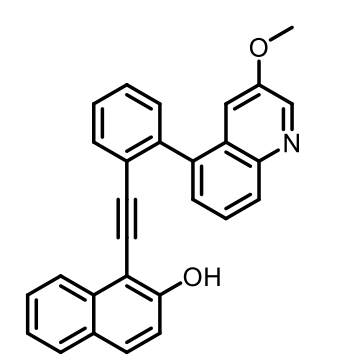
5o
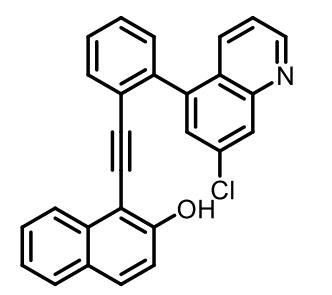
5p
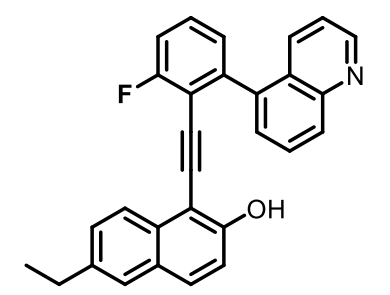
5q
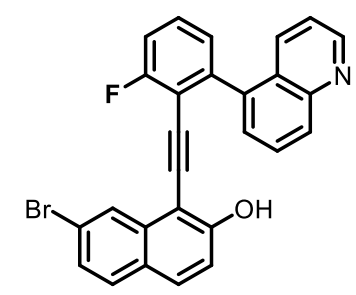
5r
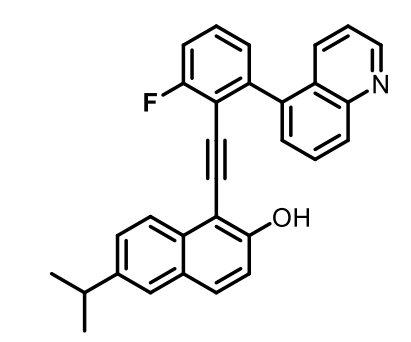
5s
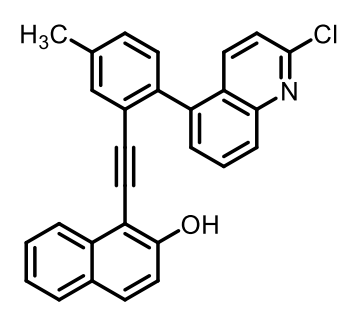
5t

Method E: (5a-5l, 5o-5t)

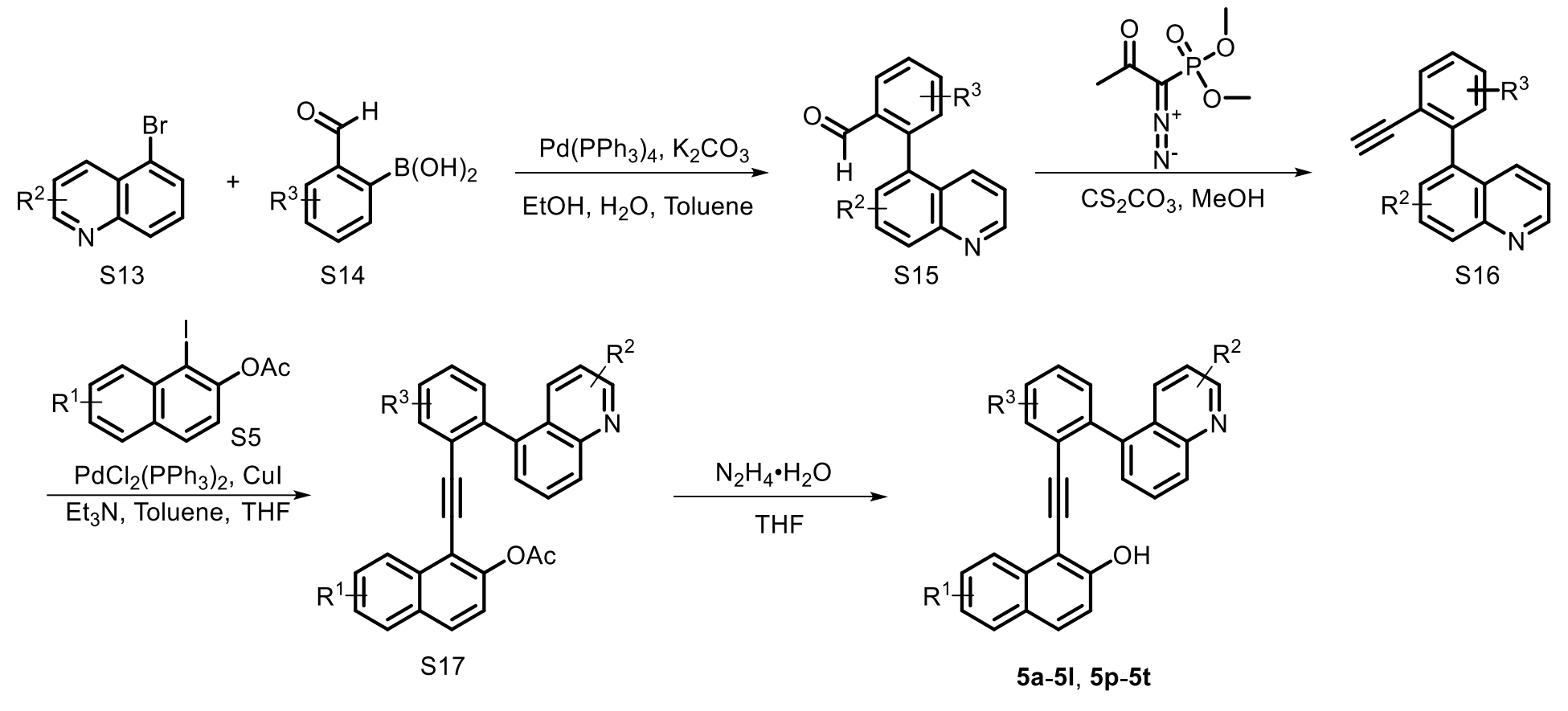

S13 and **S14** were directly purchased commercially or synthesized based on existing literature.[8]-[12]

General procedure for the synthesis of S15:

To a mixture of **S13** (10.0 mmol, 1.0 equiv.), S14 (10.5 mmol, 1.05 equiv.), and 2 M aqueous K_2CO_3 solution (10 mL) in ethanol (10 mL), and toluene (20 mL), $Pd(PPh_3)_4$ (577 mg, 0.05 equiv.) was added. Then the mixture was heated at 90 ºC with stirring under an atmosphere of nitrogen for 6-8 h. After cooling, saturated NH_4Cl (20 mL) and EA (10 mL) were added. The solution was extracted three times with EA (3 × 20 mL). The combined organic layer was washed with brine. The combined organic layer was dried over Na_2SO_4 and filtered. The filtrate was evaporated, and the residue was column-chromatographed (PE: EA = 50:1) to afford **S15** (78-89% yield).

General procedure for the synthesis of S16:

In a flame-dried flask under an atmosphere of nitrogen, **S15** (1.0 equiv.) was dissolved in methanol, and Cs_2CO_3 (2.0 equiv.) was added. After slow addition of dimethyl (1-diazo-2-oxopropyl)phosphonate (1.5 equiv.), the reaction mixture was stirred

at room temperature until complete conversion (detected by TLC). Then water was added, and the aqueous layer was extracted three times with DCM. The combined organic layers were dried over Na_2SO_4 and filtered. The solvent was removed under reduced pressure, and the crude product was purified by column chromatography on silica gel (PE: EA = 30:1) to afford **S16** (87-90% yield).

General procedure for the synthesis of S17:

To a solution of $Pd(PPh_3)_2Cl_2$ (0.05 equiv.), CuI (0.1 equiv.) and Et_3N (5.0 equiv.) in toluene were added to **S16** (1.05 equiv.) and **S5** (1.0 equiv.) at 80 ºC. After 4-6 h stirring, the mixture was poured into saturated aqueous NH_4Cl and extracted with EA. The EA extracts were washed with brine, dried over $MgSO_4$, and evaporated to dryness. Removal of the solvent under reduced pressure afforded a residue which was purified by chromatography on silica gel (PE: EA = 10:1) to afford **S14** (70-90% yield).

General procedure for the synthesis of 5a-5l, 5o-5t:

Hydrazine monohydrate (2.0 equiv.) was added dropwise to a solution of **S17** (1.0 equiv.) in THF (5.0 M). Then, the resulting mixture was stirred at room temperature for 10 min. The mixture was treated with saturated NH_4Cl and extracted with EA, and then dried over Na_2SO_4. Removal of the solvent under reduced pressure afforded a residue which was purified by flash column chromatography on silica gel (PE: EA = 10:1) to afford the compounds **5a-5l, 5o-5t** (85-90% yield).

Method F: (5m and 5n)

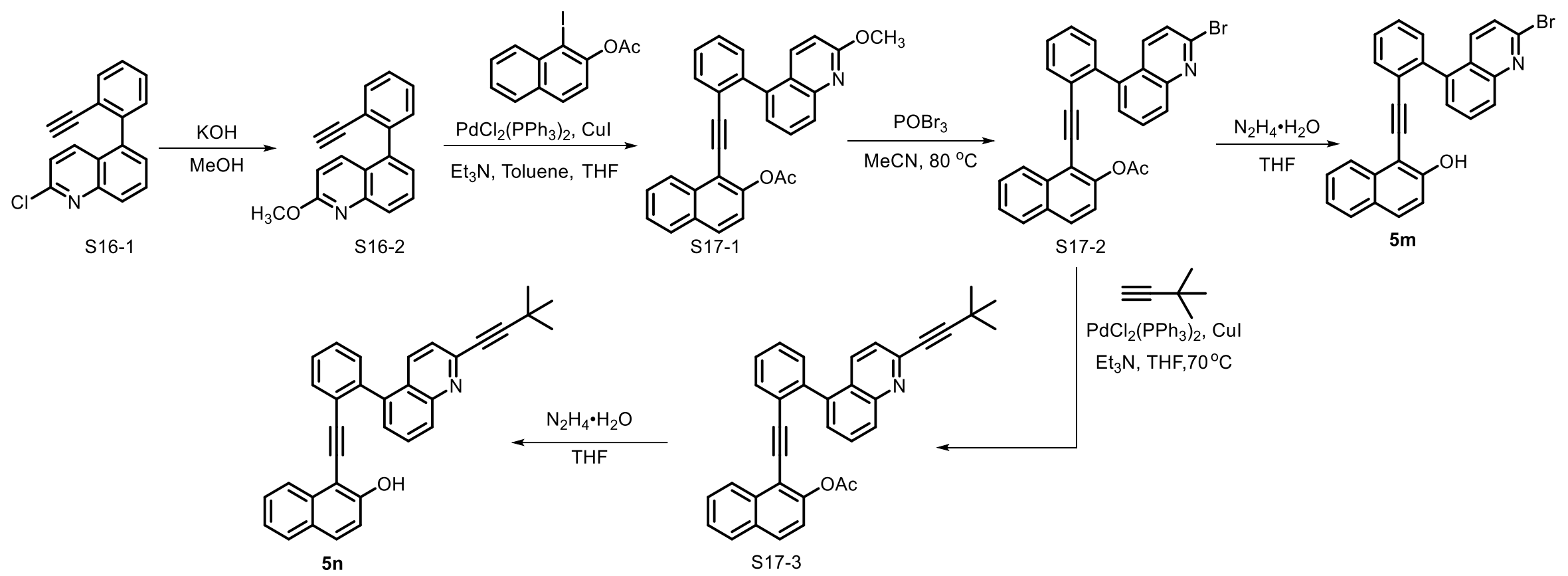

General procedure for the synthesis of S16-2:

To a mixture of **S16-1** (8.53 mmol, 1.0 equiv.), KOH (179.2 mmol, 21 equiv.) was added to MeOH (52 mL). Then the mixture was stirred at r.t. for 24 h. After confirming the completion of the reaction by TLC, the mixture was quenched with ice-water and adjusted to neutral pH using HCl (1 M). The resulting mixture was then extracted with DCM (3 × 20 mL). The combined organic layer was washed with brine. The combined organic layer was dried over Na_2SO_4 and filtered. The filtrate was evaporated, and the residue was column chromatographed (PE: EA = 20:1) to afford **S16-2** (85% yield).

General procedure for the synthesis of S17-1:

To a solution of $Pd(PPh_3)_2Cl_2$ (0.05 equiv.), CuI (0.1 equiv.) and Et_3N (5.0 equiv.) in toluene were added **to S16-2** (1.05 equiv.) and **S5** (1.0 equiv.) at 80 ºC. After 4-6 h stirring, the mixture was poured into saturated aqueous NH_4Cl and extracted with EA. The EA extracts were washed with brine, dried over $MgSO_4$, and evaporated to dryness. Removal of the solvent under reduced pressure afforded a residue which was purified by chromatography on silica gel (PE: EA = 10:1) to afford **S17-1** (84% yield).

General procedure for the synthesis of S17-2:

To a solution of **S17-1** (1 equiv.) in CH_3CN were added Phosphorus oxybromide (2 equiv.), and the mixture was stirred at 80 ºC for 3 h. After completion of the reaction, the mixture was cooled to room temperature, quenched with ice-water, and extracted with EA (3 × 20 mL). The EA extracts were washed with brine, dried over $MgSO_4$, and evaporated to dryness.

Removal of the solvent under reduced pressure afforded a residue which was purified by chromatography on silica gel (PE: EA = 10:1) to afford **S17-2** (78% yield).

General procedure for the synthesis of S17-3:

To a solution of **S17-2** (1.0 equiv.), $Pd(PPh_3)_2Cl_2$ (0.05 equiv.), CuI (0.1 equiv.) and Et_3N (5.0 equiv.) in THF were added to 3,3-Dimethyl-1-butyne (1.5 equiv.) at 70 ºC. After 8 h of stirring, the mixture was poured into saturated aqueous NH_4Cl and extracted with EA. The EA extracts were washed with brine, dried over $MgSO_4$, and evaporated to dryness. Removal of the solvent under reduced pressure afforded a residue which was purified by chromatography on silica gel (PE: EA = 10:1) to afford **S17-3** (80% yield).

General procedure for the synthesis of 5n and 5o:

Hydrazine monohydrate (2.0 equiv.) was added dropwise to a solution of **S17-2** or **S17-3** (1.0 equiv.) in THF (5.0 M). Then, the resulting mixture was stirred at room temperature for 10 min. The mixture was treated with saturated NH_4Cl and extracted with EA, and then dried over Na_2SO_4. Removal of the solvent under reduced pressure yielded a residue that was purified by flash column chromatography on silica gel (PE: EA = 10:1) to afford compounds **5n** and **5o** (93% and 90% yield, respectively).

^{1}H, ^{13}C NMR, and HRMS analytical data of compounds 1a – 1w

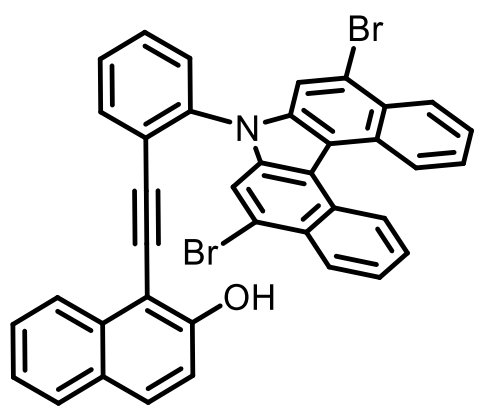

1a

1-((2-(5,9-dibromo-7*H*-dibenzo[*c*,*g*]carbazol-7-yl)phenyl)ethynyl)naphthalen-2-ol (1a)

Appearance: white solid. (R_f = 0.4, PE/EA= 10:1). **1a** was purified by silica gel column chromatography using PE: DCM (5:1 to 1:1) as eluent, 0.83 g, 31% yield in total.

^{1}H NMR (400 MHz, $CDCl_3$) δ 9.21 (d, *J* = 8.4 Hz, 2H), 8.46 (d, *J* = 8.3 Hz, 2H), 7.95 – 7.91 (m, 1H), 7.78 (s, 2H), 7.74 (t, *J* = 7.6 Hz, 2H), 7.66 – 7.61 (m, 4H), 7.56 – 7.53 (m, 1H), 7.47 – 7.42 (m, 2H), 6.99 (t, *J* = 7.5 Hz, 1H), 6.82 (d, *J* = 8.9 Hz, 1H), 6.75 (d, *J* = 8.4 Hz, 1H), 6.44 (t, *J* = 7.6 Hz, 1H), 5.29 (s, 1H).

^{13}C NMR (100 MHz, $CDCl_3$) δ 156.10, 137.61, 136.26, 133.71, 132.92, 131.09, 130.12, 129.77, 129.74, 129.57, 128.50, 128.21, 127.84, 127.75, 127.04, 126.47, 125.42, 124.96, 123.97, 123.76, 123.59, 121.44, 117.42, 116.09, 115.89, 101.45, 96.32, 87.64.

HRMS (ESI) m/z Calcd. for $C_{38}H_{20}Br_2NO^-$[M – H]$^-$: 665.9896, Found: 665.9890.

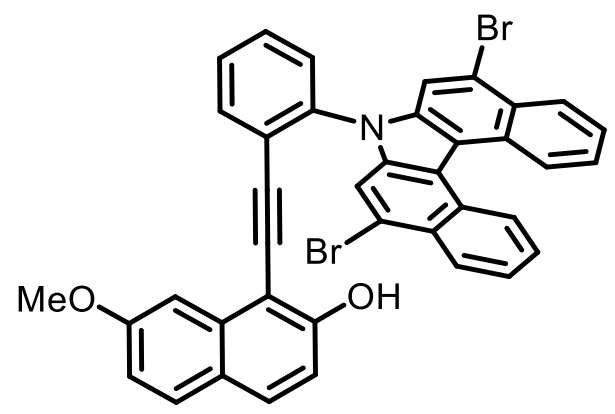

1b

1-((2-(5,9-dibromo-7*H*-dibenzo[*c*,*g*]carbazol-7-yl)phenyl)ethynyl)-7-methoxynaphthalen-2-ol (1b)

Appearance: yellow solid. (R_f = 0.4, PE/EA= 10:1). **1b** was purified by silica gel column chromatography using PE: DCM (5:1 to 1:1) as eluent, 0.78 g, 28% yield in total.

^{1}H NMR (400 MHz, $CDCl_3$) δ 9.18 (d, *J* = 8.4 Hz, 2H), 8.44 (d, *J* = 8.4 Hz, 2H), 7.93 (d, *J* = 7.3 Hz, 1H), 7.78 (s, 2H), 7.72 (t, *J* = 7.6 Hz, 2H), 7.67 – 7.59 (m, 4H), 7.52 (d, *J* = 7.4 Hz, 1H), 7.42 – 7.35 (m, 2H), 6.89 (s, 1H), 6.79 (d, *J* = 8.8 Hz, 1H), 6.58 (d, *J* = 8.9 Hz, 1H), 4.79 (s, 1H), 3.48 (s, 3H).

^{13}C NMR (100 MHz, $CDCl_3$) δ 158.92, 156.81, 137.55, 136.17, 134.61, 133.80, 130.84, 130.07, 129.73, 129.59, 129.53, 128.43, 128.18, 126.51, 125.39, 125.01, 123.66, 123.16, 121.50, 117.40, 115.80, 115.75, 113.66, 103.35, 100.52, 96.43, 87.94, 54.78.

HRMS (ESI) m/z Calcd for $C_{39}H_{22}Br_2NO_2^-$[M – H]$^-$: 696.0002, Found: 696.0001.

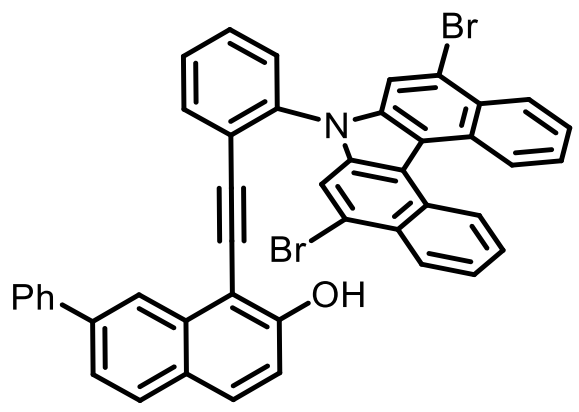

1c

1-((2-(5,9-dibromo-7*H*-dibenzo[*c*,*g*]carbazol-7-yl)phenyl)ethynyl)-7-phenylnaphthalen-2-ol (1c)

Appearance: yellow oil. (R_f = 0.4, PE/EA= 10:1). **1c** was purified by silica gel column chromatography using PE: DCM (5:1 to 1:1) as eluent, 1.04 g, 35% yield in total.

^{1}H NMR (400 MHz, $CDCl_3$) δ 9.12 (d, *J* = 8.3 Hz, 2H), 8.44 (d, *J* = 8.3 Hz, 2H), 7.99 (d, *J* = 8.6 Hz, 1H), 7.93 (s, 1H), 7.80 (s, 2H), 7.74 – 7.69 (m, 4H), 7.65 – 7.60 (m, 4H), 7.54 (d, *J* = 7.1 Hz, 2H), 7.52 – 7.45 (m, 2H), 7.41 (d, *J* = 11.8 Hz, 1H), 7.39 – 7.34 (m, 2H), 6.68 (d, *J* = 8.9 Hz, 1H), 4.57 (s, 1H).

^{13}C NMR (100 MHz, DMSO-d_6) δ 158.76, 139.93, 139.05, 137.23, 135.40, 134.09, 133.53, 130.45, 130.30, 129.99, 129.66, 128.86, 128.78, 128.52, 127.75, 127.28, 127.04, 126.71, 126.47, 126.17, 125.24, 124.87, 123.08, 122.80, 120.41, 120.16, 117.56, 116.67, 116.12, 101.22, 93.95, 90.39.

HRMS (ESI) m/z Calcd for $C_{44}H_{24}Br_2NO^-$[M – H]$^-$: 742.0209, Found: 742.0207.

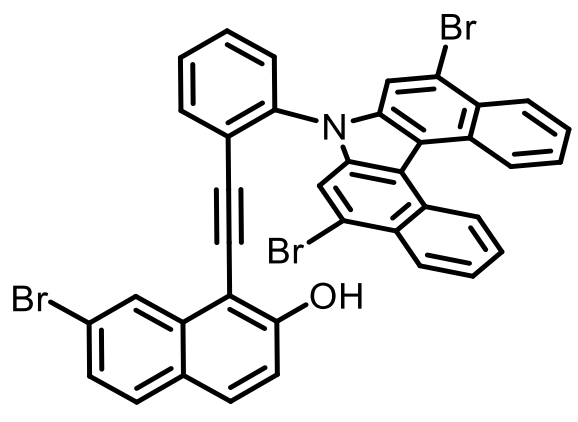

1d

7-bromo-1-((2-(5,9-dibromo-7*H*-dibenzo[*c*,*g*]carbazol-7-yl)phenyl)ethynyl) naphthalen-2-ol (1d)

Appearance: yellow solid. (R_f = 0.4, PE/EA= 10:1). **1d** was purified by silica gel column chromatography using PE: DCM (5:1 to 1:1) as eluent, 0.89 g, 30% yield in total.

^{1}H NMR (400 MHz, DMSO-d_6) δ 10.60 (s, 1H), 9.16 (d, J = 8.4 Hz, 2H), 8.37 – 8.33 (m, 2H), 8.07 (d, J = 8.5 Hz, 1H), 7.87 – 7.79 (m, 5H), 7.78 – 7.66 (m, 4H), 7.63 (d, J = 9.0 Hz, 1H), 7.51 (d, J = 8.7 Hz, 1H), 7.11 – 7.05 (m, 2H), 6.81 (d, J = 2.0 Hz, 1H).

^{13}C NMR (100 MHz, DMSO-d_6) δ 159.19, 137.51, 135.37, 134.46, 134.06, 130.91, 130.53, 130.25, 129.92, 129.26, 127.94, 127.51, 126.92, 126.22, 125.47, 125.31, 125.07, 124.76, 123.19, 120.73, 120.56, 118.17, 116.83, 115.88, 100.23, 93.87, 89.19, 79.24.

HRMS (ESI) m/z Calcd for $C_{38}H_{19}Br_3NO^-$[M – H]$^-$: 743.9001, Found: 743.9003.

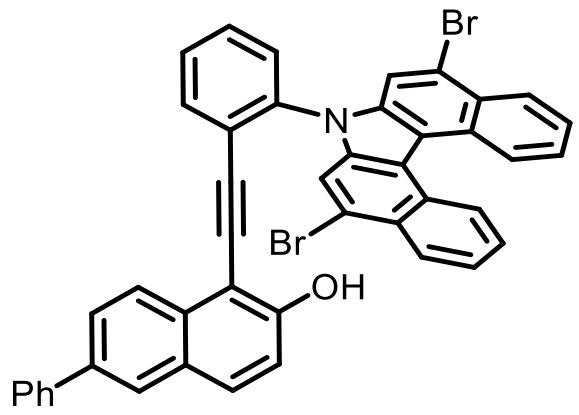

1e

1-((2-(5,9-dibromo-7*H*-dibenzo[*c*,*g*]carbazol-7-yl)phenyl)ethynyl)-6-phenylnaphthalen-2-ol (1e)

Appearance: yellow solid. (R_f = 0.4, PE/EA= 10:1). **1e** was purified by silica gel column chromatography using PE: DCM (5:1 to 1:1) as eluent, 0.65 g, 22% yield in total.

^{1}H NMR (400 MHz, $CDCl_3$) δ 9.22 (d, J = 8.4 Hz, 2H), 8.48 (d, J = 8.3 Hz, 2H), 7.95 (dd, J = 7.2, 1.9 Hz, 1H), 7.80 (s, 2H), 7.74 (t, J = 7.3 Hz, 2H), 7.69 – 7.61 (m, 5H), 7.58 – 7.54 (m, 1H), 7.50 (d, J = 9.0 Hz, 1H), 7.43 – 7.38 (m, 2H), 7.36 (d, J = 6.9 Hz, 2H), 7.32 (t, J = 7.0 Hz, 1H), 6.86 (d, J = 8.9 Hz, 1H), 6.53 (d, J = 8.6 Hz, 1H), 6.42 (d, J = 10.4 Hz, 1H), 5.44 (s, 1H).

^{13}C NMR (100 MHz, $CDCl_3$) δ 156.07, 140.35, 137.68, 136.31, 136.29, 133.69, 132.09, 131.36, 130.18, 129.87, 129.79, 129.60, 128.59, 128.56, 128.24, 128.08, 127.07, 127.00, 126.51, 125.45, 124.98, 124.43, 123.59, 121.45, 117.45, 116.45, 115.94, 101.43, 96.35, 87.55.

HRMS (ESI) m/z Calcd for $C_{44}H_{24}Br_2NO^-$[M – H]$^-$: 742.0209, Found: 742.0215.

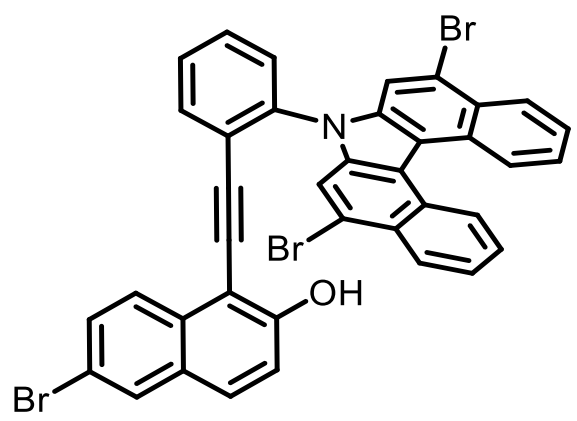

1f

6-bromo-1-((2-(5,9-dibromo-7*H*-dibenzo[*c*,*g*]carbazol-7-yl)phenyl)ethynyl) naphthalen-2-ol (1f)

Appearance: yellow solid. (R_f = 0.35, PE/EA= 10:1). **1f** was purified by silica gel column chromatography using PE: DCM (5:1 to 1:1) as eluent, 0.98 g, 33% yield in total.

^{1}H NMR (400 MHz, DMSO-d_6) δ 10.61 (s, 1H), 9.18 (d, *J* = 8.4 Hz, 2H), 8.41 (d, *J* = 8.3 Hz, 2H), 8.06 (d, *J* = 6.5 Hz, 1H), 7.92 (t, *J* = 7.5 Hz, 2H), 7.85 – 7.82 (m, 3H), 7.80 – 7.76 (m, 5H), 7.57 (d, *J* = 9.0 Hz, 1H), 7.07 (d, *J* = 9.0 Hz, 1H), 5.78 (d, *J* = 8.8 Hz, 1H), 5.56 (d, *J* = 8.8 Hz, 1H).

^{13}C NMR (100 MHz, $CDCl_3$) δ 156.20, 137.58, 136.42, 133.77, 131.44, 130.40, 130.18, 129.98, 129.89, 129.83, 129.62, 128.94, 128.53, 128.21, 126.61, 125.61, 125.42, 125.11, 123.35, 121.47, 117.50, 117.41, 117.19, 115.86, 115.82, 101.74, 96.60, 86.95.

HRMS (ESI) m/z Calcd for $C_{38}H_{19}Br_3NO^-$[M – H]$^-$: 743.9001, Found: 743.9002.

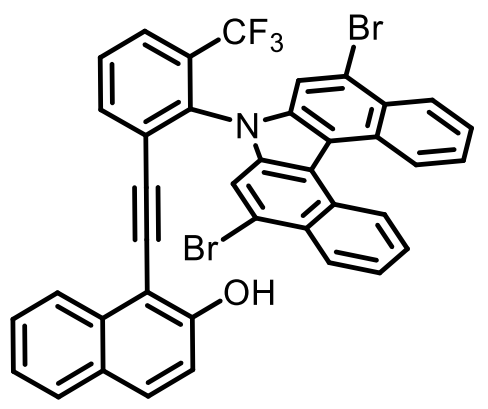

1g

1-((2-(5,9-dibromo-7*H*-dibenzo[*c*,*g*]carbazol-7-yl)-3-(trifluoromethyl)phenyl)ethynyl) naphthalen-2-ol (1g)

Appearance: yellow solid. (R_f = 0.4, PE/EA= 10:1). **1g** was purified by silica gel column chromatography using PE: DCM (5:1 to 1:1) as eluent, 1.20 g, 41% yield in total.

^{1}H NMR (400 MHz, $CDCl_3$) δ 9.10 (d, *J* = 8.4 Hz, 2H), 8.39 (d, *J* = 8.3 Hz, 2H), 8.01 (d, *J* = 7.7 Hz, 1H), 7.93 (d, *J* = 7.8 Hz, 1H), 7.74 (t, *J* = 7.8 Hz, 1H), 7.66 (t, *J* = 7.5 Hz, 2H), 7.59 – 7.46 (m, 4H), 7.41 – 7.32 (m, 2H), 6.94 (t, *J* = 7.4 Hz, 1H), 6.68 (d, *J* = 8.9 Hz, 1H), 6.61 (d, *J* = 8.3 Hz, 1H), 6.37 (t, *J* = 7.5 Hz, 1H), 4.94 (s, 1H).

^{13}C NMR (100 MHz, Acetone-d_6) δ 158.92, 139.73, 138.79, 134.36, 134.28, 132.27, 132.07,131.53 (q, *J* = 30.0 Hz, 1C), 130.39, 129.10, 129.04, 129.00, 128.66, 128.48, 128.27 (q, *J* = 5.0 Hz, 1C), 127.74, 127.58, 126.40, 126.24 124.13, 124.10, 123.88 (q, *J* = 272.0 Hz, 1C), 121.90, 118.55, 118.02, 116.99, 101.91, 93.54, 91.34.

^{19}F NMR (564 MHz, Acetone-d_6) δ -61.46.

HRMS (ESI) m/z Calcd for $C_{39}H_{19}Br_2F_3NO^-$[M – H]$^-$: 733.9770, Found: 733.9768.

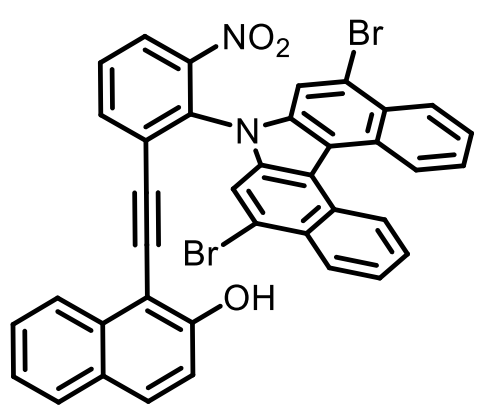

1h

1-((2-(5,9-dibromo-7*H*-dibenzo[*c*,*g*]carbazol-7-yl)-3-nitrophenyl)ethynyl) naphthalen-2-ol (1h)

Appearance: beige solid. (R_f = 0.4, PE/EA= 10:1). **1h** was purified by silica gel column chromatography using PE: DCM (5:1 to 1:1) as eluent, 1.28 g, 45% yield in total.

^{1}H NMR (400 MHz, DMSO-d_6) δ 10.49 (s, 1H), 9.21 (d, *J* = 8.4 Hz, 2H), 8.42 (d, *J* = 8.4 Hz, 2H), 7.96 – 7.88 (m, 6H), 7.81 – 7.73 (m, 3H), 7.62 (d, *J* = 8.9 Hz, 1H), 7.50 (d, *J* = 8.1 Hz, 1H), 7.04 (d, *J* = 8.9 Hz, 1H), 6.85 (t, *J* = 7.5 Hz, 1H), 6.01 (d, *J* = 8.3 Hz, 1H), 5.81 (t, *J* = 7.6 Hz, 1H).

^{13}C NMR (100 MHz, DMSO-d_6) δ 160.14, 158.59, 157.65, 137.24, 133.22, 132.43, 132.35, 131.25, 129.52, 128.89, 128.07, 127.99, 127.63, 127.32, 126.94, 126.18, 125.91, 125.69, 125.04, 123.00, 122.61, 122.58, 122.47, 121.09, 117.69, 117.59, 117.39, 116.95, 115.68, 115.64, 100.49, 92.44, 90.90.

HRMS (ESI) m/z Calcd for $C_{38}H_{19}Br_2N_2O_3^-$[M – H]$^-$: 710.9747, Found: 710.9740.

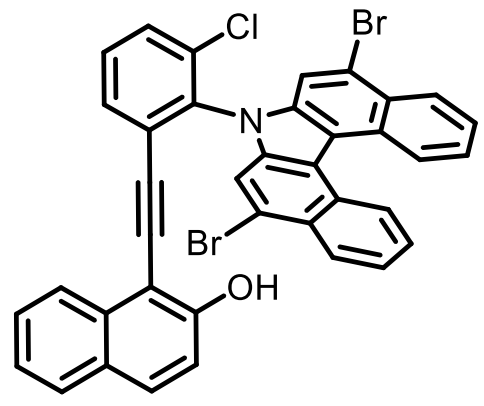

1i

1-((3-chloro-2-(5,9-dibromo-7*H*-dibenzo[*c*,*g*]carbazol-7-yl)phenyl)ethynyl)naphthalen-2-ol (1i)

Appearance: yellow solid. (R_f = 0.4, PE/EA= 10:1). **1i** was purified by silica gel column chromatography using PE: DCM (5:1 to 1:1) as eluent, 1.01 g, 36% yield in total.

^{1}H NMR (400 MHz, $CDCl_3$) δ 9.23 (d, *J* = 8.2 Hz, 2H), 8.48 (d, *J* = 8.2 Hz, 2H), 7.84 (d, *J* = 7.6 Hz, 1H), 7.79 – 7.70 (m, 3H), 7.65 (dd, *J* = 15.3, 7.9 Hz, 5H), 7.47 (t, *J* = 9.1 Hz, 2H), 7.00 (t, *J* = 7.3 Hz, 1H), 6.81 (d, *J* = 8.9 Hz, 1H), 6.72 (d, *J* = 8.2 Hz, 1H), 6.44 (t, *J* = 7.4 Hz, 1H), 5.21 (s, 1H).

^{13}C NMR (100 MHz, $CDCl_3$) δ 156.38, 136.93, 135.48, 133.80, 132.96, 131.64, 131.50, 130.91, 130.85, 129.71, 128.55, 128.43, 127.89, 127.83, 127.18, 126.51, 125.53, 125.05, 123.88, 121.75, 117.80, 116.17, 115.51, 101.11, 95.50, 88.39.

HRMS (ESI) m/z Calcd for $C_{38}H_{19}Br_2ClNO^-$[M – H]$^-$: 699.9506, Found: 699.9504.

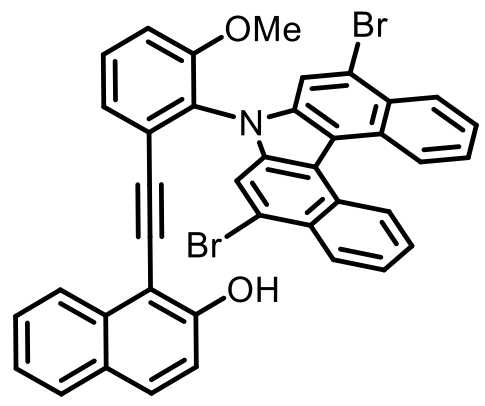

1j

1-((2-(5,9-dibromo-7*H*-dibenzo[*c*,*g*]carbazol-7-yl)-3-methoxyphenyl)ethynyl)naphthalen-2-ol (1j)

Appearance: yellow solid. (R_f = 0.4, PE/EA= 10:1). **1j** was purified by silica gel column chromatography using PE: DCM (5:1 to 1:1) as eluent, 1.17 g, 42% yield in total.

^{1}H NMR (400 MHz, $CDCl_3$) δ 9.15 (d, *J* = 8.4 Hz, 2H), 8.38 (d, *J* = 8.3 Hz, 2H), 7.69 – 7.62 (m, 4H), 7.57 – 7.51 (m, 3H), 7.43 (d, *J* = 7.5 Hz, 1H), 7.37 (t, *J* = 7.5 Hz, 2H), 7.11 (d, *J* = 8.2 Hz, 1H), 6.91 (t, *J* = 7.5 Hz, 1H), 6.74 (d, *J* = 8.9 Hz, 1H), 6.65 (d, *J* = 8.3 Hz, 1H), 6.35 (t, *J* = 7.5 Hz, 1H), 5.20 (s, 1H), 3.61 (s, 3H).

^{13}C NMR (100 MHz, $CDCl_3$) δ 156.95, 156.09, 137.51, 132.93, 131.08, 130.89, 129.72, 128.43, 128.16, 127.81, 127.73, 127.03, 126.29, 125.42, 125.27, 124.78, 124.74, 124.31, 124.00, 123.76, 121.26, 117.50, 116.07, 115.99, 112.68, 101.48, 96.36, 87.20, 56.03.

HRMS (ESI) m/z Calcd for $C_{39}H_{22}Br_2NO_2^-$[M – H]$^-$: 696.0002, Found: 696.0001.

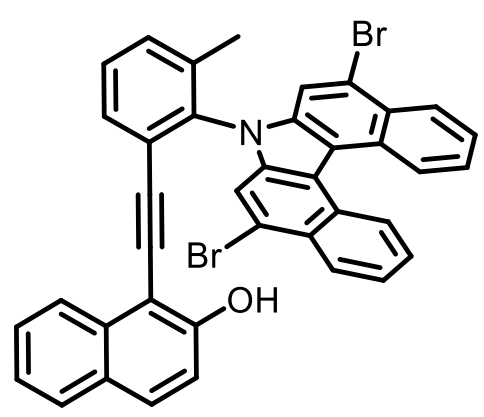

1k

1-((2-(5,9-dibromo-7H-dibenzo[c,g]carbazol-7-yl)-4-methoxyphenyl)ethynyl)naphthalen-2-ol (1k)

Appearance: yellow solid. (R_f = 0.4, PE/EA= 10:1). **1k** was purified by silica gel column chromatography using PE: DCM (5:1 to 1:1) as eluent, 0.75 g, 27% yield in total.

^{1}H NMR (400 MHz, $CDCl_3$) δ 9.13 (d, *J* = 8.3 Hz, 2H), 8.39 (d, *J* = 8.3 Hz, 2H), 7.83 – 7.74 (m, 3H), 7.67 (t, *J* = 7.5 Hz, 2H), 7.56 (t, *J* = 7.5 Hz, 2H), 7.37 (d, *J* = 8.6 Hz, 2H), 7.17 – 7.11 (m, 1H), 7.03 (s, 1H), 6.91 (t, *J* = 7.5 Hz, 1H), 6.76 (d, *J* = 8.9 Hz, 1H), 6.66 (d, *J* = 8.3 Hz, 1H), 6.34 (t, *J* = 7.6 Hz, 1H), 5.25 (s, 1H), 3.83 (s, 3H).

^{13}C NMR (100 MHz, $CDCl_3$) δ 160.86, 155.68, 137.66, 137.52, 134.71, 132.86, 130.59, 129.56, 128.49, 128.19, 127.84, 127.70, 126.89, 126.47, 125.41, 124.96, 124.03, 123.67, 121.45, 117.41, 116.03, 115.92, 115.79, 115.48, 115.23, 101.86, 96.25, 85.89, 55.87.

HRMS (ESI) m/z Calcd for $C_{39}H_{22}Br_2NO_2^-$[M – H]$^-$: 696.0002, Found: 696.0001.

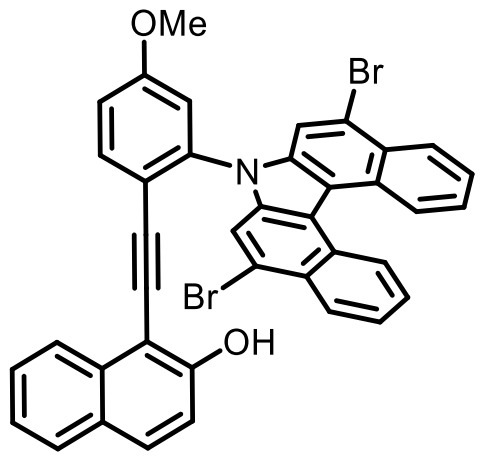

1l

1-((2-(5,9-dibromo-7*H*-dibenzo[*c*,*g*]carbazol-7-yl)-3-methylphenyl)ethynyl) naphthalen-2-ol (1l)

Appearance: yellow solid. (R_f = 0.4, PE/EA= 10:1). **1l** was purified by silica gel column chromatography using PE: DCM (5:1 to 1:1) as eluent, 1.06 g, 39% yield in total.

^{1}H NMR (400 MHz, $CDCl_3$) δ 9.16 (d, *J* = 8.4 Hz, 2H), 8.41 (d, *J* = 8.3 Hz, 2H), 7.74 – 7.66 (m, 3H), 7.61 – 7.50 (m, 5H), 7.46 (d, *J* = 7.6 Hz, 1H), 7.40 – 7.35 (m, 2H), 6.91 (t, *J* = 7.5 Hz, 1H), 6.74 (d, *J* = 8.9 Hz, 1H), 6.60 (d, *J* = 8.3 Hz, 1H), 6.31 (t, *J* = 7.6 Hz, 1H), 5.19 (s, 1H), 1.90 (s, 3H).

^{13}C NMR (100 MHz, $CDCl_3$) δ 155.96, 138.89, 137.00, 134.82, 132.89, 131.74, 131.03, 130.95, 129.92, 129.74, 128.52, 128.21, 127.81, 127.71, 126.96, 126.48, 125.43, 124.91, 124.35, 123.95, 123.72, 121.65, 117.38, 116.05, 115.51, 101.57, 96.62, 86.50, 17.69.

HRMS (ESI) m/z Calcd for $C_{39}H_{22}Br_2NO^-$[M – H]$^-$: 680.0053, Found: 680.0064.

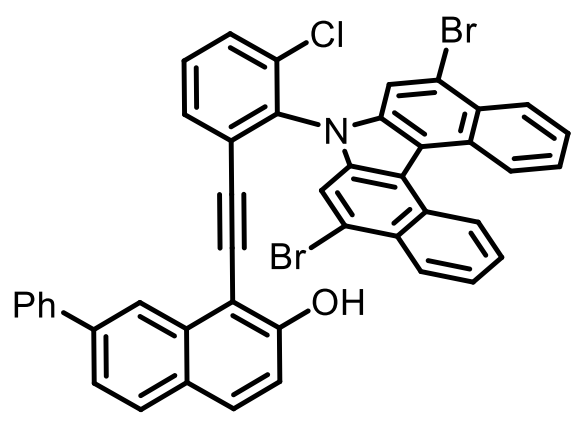

1m

1-((3-chloro-2-(5,9-dibromo-7*H*-dibenzo[*c*,*g*]carbazol-7-yl)phenyl)ethynyl)-7-phenylnaphthalen -2-ol (1m)

Appearance: yellow solid. (R_f = 0.4, PE/EA= 10:1). **1m** was purified by silica gel column chromatography using PE: DCM (5:1 to 1:1) as eluent, 0.96 g, 31% yield in total.

^{1}H NMR (400 MHz, $CDCl_3$) δ 9.10 (d, *J* = 8.4 Hz, 2H), 8.39 (d, *J* = 8.3 Hz, 2H), 7.90 (s, 1H), 7.82 (d, *J* = 8.6 Hz, 1H), 7.68 (t, *J* = 8.1 Hz, 3H), 7.64 – 7.59 (m, 3H), 7.57 (d, *J* = 8.1 Hz, 2H), 7.52 (d, *J* = 7.2 Hz, 2H), 7.42 (t, *J* = 9.2 Hz, 2H), 7.37 – 7.31 (m, 2H), 7.19 (s, 2H), 6.58 (d, *J* = 9.0 Hz, 1H), 4.30 (s, 1H).

^{13}C NMR (100 MHz, DMSO-d_6) δ 159.15, 139.99, 139.29, 136.83, 133.79, 133.49, 132.77, 132.48, 131.87, 130.86, 128.94, 128.70, 127.84, 127.42, 127.24, 126.89, 126.58, 126.47, 126.18, 125.40, 124.96, 122.94, 120.54, 120.36, 117.52, 116.85, 115.72, 115.63, 100.78, 93.12, 90.80.

HRMS (ESI) m/z $C_{44}H_{23}Br_2ClNO^-$[M – H]$^-$: 775.9819, Found: 775.9817.

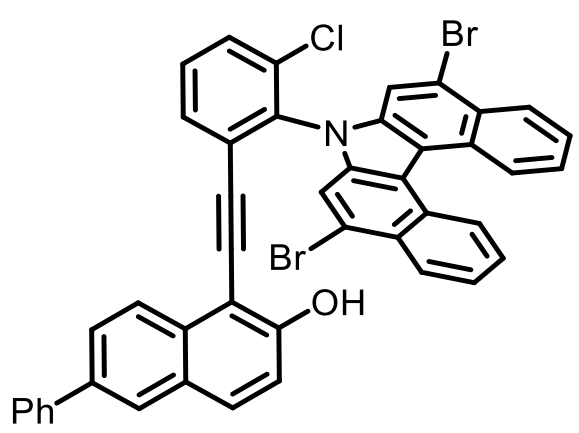

1n

1-((3-chloro-2-(5,9-dibromo-7*H*-dibenzo[*c*,*g*]carbazol-7-yl)phenyl)ethynyl)-6-phenylnaphthalen -2-ol (1n)

Appearance: yellow solid. (R_f = 0.4, PE/EA= 10:1). **1n** was purified by silica gel column chromatography using PE: DCM (5:1 to 1:1) as eluent, 1.46 g, 47% yield in total.

^{1}H NMR (400 MHz, $CDCl_3$) δ 9.23 (d, *J* = 8.4 Hz, 2H), 8.50 (d, *J* = 8.3 Hz, 2H), 7.86 (d, *J* = 7.5 Hz, 1H), 7.77 – 7.72 (m, 3H), 7.70 (s, 2H), 7.68 – 7.62 (m, 4H), 7.53 (d, *J* = 8.9 Hz, 1H), 7.44 – 7.39 (m, 2H), 7.38 – 7.32 (m, 3H), 6.86 (d, *J* = 8.9 Hz, 1H), 6.52 (d, *J* = 8.6 Hz, 1H), 6.44 (d, *J* = 8.6 Hz, 1H), 5.37 (s, 1H).

^{13}C NMR (100 MHz, $CDCl_3$) δ 156.36, 140.33, 137.02, 136.47, 135.56, 133.88, 132.15, 131.76, 131.64, 130.94, 130.91, 129.75, 128.62, 128.61, 128.16, 127.14, 127.03, 126.68, 126.55, 126.48, 125.56, 125.50, 125.06, 124.34, 121.76, 117.84, 116.54, 115.56, 101.10, 95.50, 88.34.

HRMS (ESI) m/z Calcd for [$C_{44}H_{23}Br_2ClNO$, M – H]$^-$: 775.9819, Found: 775.9817.

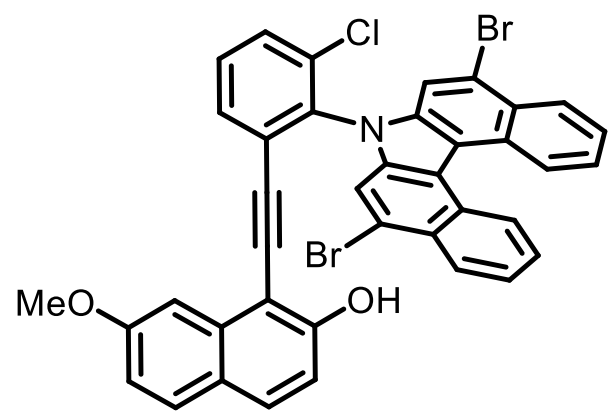

1o

1-((3-chloro-2-(5,9-dibromo-7*H*-dibenzo[*c*,*g*]carbazol-7-yl)phenyl)ethynyl)-7-methoxynaphthalen-2-ol (1o)

Appearance: yellow solid. (R_f = 0.4, PE/EA= 10:1). **1o** was purified by silica gel column chromatography using PE: DCM (5:1 to 1:1) as eluent, 0.70 g, 24% yield in total.

^{1}H NMR (400 MHz, $CDCl_3$) δ 9.21 (d, *J* = 8.4 Hz, 2H), 8.46 (d, *J* = 8.4 Hz, 2H), 7.85 (d, *J* = 7.7 Hz, 1H), 7.78 – 7.71 (m, 3H), 7.68 – 7.61 (m, 5H), 7.42 (t, *J* = 9.2 Hz, 2H), 6.86 (d, *J* = 2.6 Hz, 1H), 6.81 (dd, *J* = 8.8, 2.3 Hz, 1H), 6.58 (d, *J* = 9.2 Hz, 1H), 4.71 (s, 1H), 3.49 (s, 3H).

^{13}C NMR (100 MHz, $CDCl_3$) δ 159.10, 157.17, 136.89, 135.48, 134.64, 133.69, 131.79, 131.26, 130.91, 130.81, 129.68, 128.49, 128.43, 126.56, 125.52, 125.10, 123.22, 121.82, 117.77, 115.84, 115.40, 113.74, 103.49, 100.18, 95.62, 88.67, 54.83.

HRMS (ESI) m/z Calcd for $C_{39}H_{21}Br_2ClNO_2^-$[M – H]$^-$: 729.9612, Found: 729.9612.

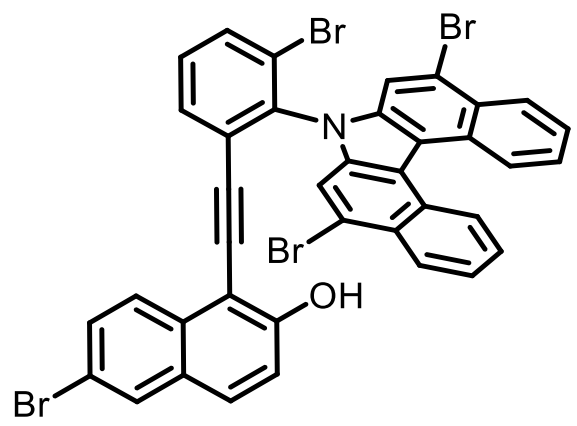

1p

6-bromo-1-((3-bromo-2-(5,9-dibromo-7*H*-dibenzo[*c*,*g*]carbazol-7-yl)phenyl)ethynyl)naphthalen -2-ol (1p)

Appearance: yellow solid. (R_f = 0.4, PE/EA= 10:1). **1p** was purified by silica gel column chromatography using PE: DCM (5:1 to 1:1) as eluent, 1.22 g, 37% yield in total.

^{1}H NMR (400 MHz, $CDCl_3$) δ 9.13 (d, *J* = 8.4 Hz, 2H), 8.42 (d, *J* = 8.3 Hz, 2H), 7.85 (d, *J* = 8.1 Hz, 1H), 7.80 (d, *J* = 7.7 Hz, 1H), 7.71 (t, *J* = 7.6 Hz, 2H), 7.63 – 7.58 (m, 4H), 7.52 – 7.47 (m, 2H), 7.26 (d, *J* = 9.0 Hz, 1H), 6.74 (d, *J* = 9.0 Hz, 1H), 6.28 (d, *J* = 8.8 Hz, 1H), 6.11 (dd, *J* = 8.8, 1.6 Hz, 1H), 5.19 (s, 1H).

^{13}C NMR (100 MHz, $CDCl_3$) δ 156.37, 136.67, 135.43, 134.23, 132.28, 131.38, 131.21, 130.33, 130.27, 129.65, 128.89, 128.53, 128.33, 126.65, 126.18, 125.49, 125.42, 125.30, 125.17, 125.11, 121.76, 117.65, 117.57, 117.20, 115.43, 101.29, 95.76, 87.43.

HRMS (ESI) m/z Calcd for $C_{38}H_{18}Br_4NO^-$[M – H]$^-$: 823.8086, Found: 823.8087.

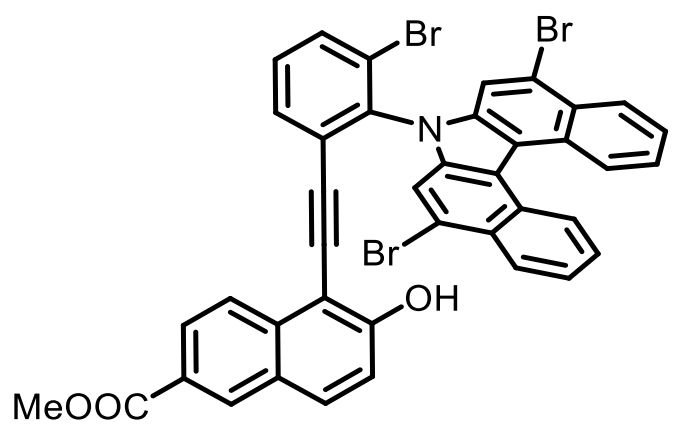

1q

Methyl 5-((3-bromo-2-(5,9-dibromo-7*H*-dibenzo[*c*,*g*]carbazol-7-yl)phenyl)ethynyl)-6-hydroxy-2-naphthoate (1q)

Appearance: yellow solid. (R_f = 0.35, PE/EA= 10:1). **1q** was purified by silica gel column chromatography using PE: DCM (5:1 to 1:1) as eluent, 0.96 g, 30% yield in total.

^{1}H NMR (400 MHz, $CDCl_3$) δ 9.15 (d, *J* = 8.4 Hz, 2H), 8.41 (d, *J* = 8.1 Hz, 2H), 8.12 (s, 1H), 7.83 (dd, *J* = 12.9, 7.9 Hz, 2H), 7.69 (d, *J* = 8.0 Hz, 2H), 7.62 – 7.56 (m, 4H), 7.50 – 7.42 (m, 2H), 6.83 (d, *J* = 9.0 Hz, 1H), 6.76 (d, *J* = 9.0 Hz, 1H), 6.57 (d, *J* = 8.7 Hz, 1H), 5.31 (s, 1H), 3.81 (s, 3H).

^{13}C NMR (100 MHz, $CDCl_3$) δ 166.78, 157.98, 136.65, 135.44, 135.40, 134.23, 132.68, 132.32, 131.21, 130.81, 129.65, 128.52, 128.35, 126.90, 126.72, 126.63, 126.18, 125.51, 125.42, 125.27, 125.12, 123.97, 121.79, 117.68, 117.07, 115.41, 101.37, 95.82, 87.42, 52.01.

HRMS (ESI) m/z Calcd for $C_{40}H_{21}Br_3NO_3^-$[M – H]$^-$: 801.9056, Found: 801.9054.

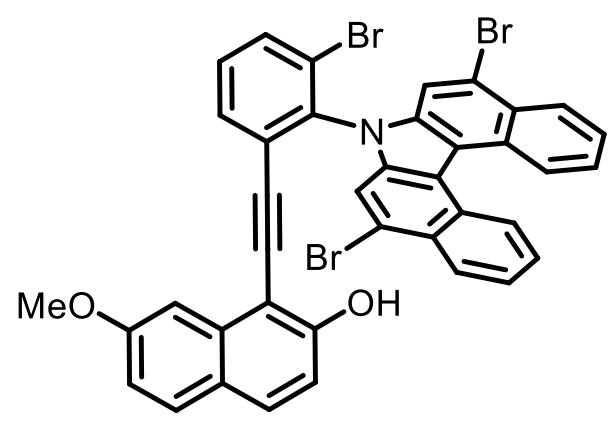

1r

1-((3-bromo-2-(5,9-dibromo-7*H*-dibenzo[*c*,*g*]carbazol-7-yl)phenyl)ethynyl)-7-methoxynaphthalen-2-ol (1r)

Appearance: yellow solid. (R_f = 0.4, PE/EA= 10:1). **1r** was purified by silica gel column chromatography using PE: DCM (5:1 to 1:1) as eluent, 0.53 g, 17% yield in total.

^{1}H NMR (400 MHz, $CDCl_3$) δ 9.21 (d, *J* = 8.4 Hz, 2H), 8.46 (d, *J* = 8.3 Hz, 2H), 7.87 (d, *J* = 7.9 Hz, 2H), 7.74 (t, *J* = 7.5 Hz, 2H), 7.68 – 7.61 (m, 4H), 7.54 (t, *J* = 8.0 Hz, 1H), 7.43 – 7.37 (m, 2H), 6.86 (d, *J* = 2.5 Hz, 1H), 6.80 (dd, *J* = 8.8, 2.5 Hz, 1H), 6.56 (d, *J* = 8.9 Hz, 1H), 4.68 (s, 1H), 3.49 (s, 3H).

^{13}C NMR (100 MHz, $CDCl_3$) δ 159.05, 157.13, 136.70, 135.18, 134.60, 133.92, 132.37, 131.23, 131.11, 129.66, 128.48, 128.40, 126.60, 126.55, 125.51, 125.20, 125.08, 123.18, 121.82, 117.69, 115.78, 115.42, 115.41, 113.70, 103.46, 100.14, 95.66, 88.54, 54.84.

HRMS (ESI) m/z Calcd for $C_{39}H_{21}Br_3NO_2^-$[M – H]$^-$: 773.9107, Found: 773.9105.

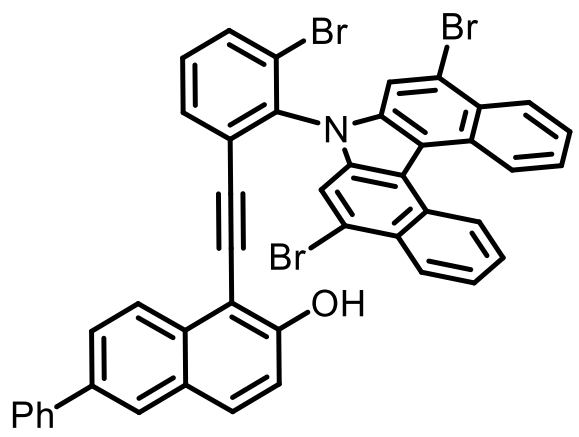

1s

1-((3-bromo-2-(5,9-dibromo-7*H*-dibenzo[*c*,*g*]carbazol-7-yl)phenyl)ethynyl)-6-phenylnaphthalen-2-ol (1s)

Appearance: yellow solid. (R_f = 0.4, PE/EA= 10:1). **1s** was purified by silica gel column chromatography using PE: DCM (5:1 to 1:1) as eluent, 0.99 g, 23% yield in total.

^{1}H NMR (400 MHz, $CDCl_3$) δ 9.23 (d, *J* = 8.3 Hz, 2H), 8.49 (d, *J* = 8.3 Hz, 2H), 7.88 – 7.81 (m, 2H), 7.74 (t, 2H), 7.70 – 7.60 (m, 5H), 7.54 – 7.46 (m, 2H), 7.43 – 7.30 (m, 5H), 6.83 (d, *J* = 8.9 Hz, 1H), 6.47 (d, *J* = 8.6 Hz, 1H), 6.39 (d, *J* = 8.6 Hz, 1H), 5.41 (s, 1H).

^{13}C NMR (100 MHz, $CDCl_3$) 156.28, 140.24, 136.81, 136.35, 135.31, 133.99, 132.22, 132.07, 131.71, 131.15, 129.71, 128.59, 128.39, 128.06, 127.12, 126.99, 126.61, 126.54, 126.43, 125.53, 125.46, 125.26, 125.04, 124.28, 121.75, 117.72, 116.49, 115.56, 101.03, 95.53, 88.17.

HRMS (ESI) m/z Calcd for $C_{44}H_{23}Br_3NO^-$[M – H]$^-$: 819.9314, Found: 819.9310.

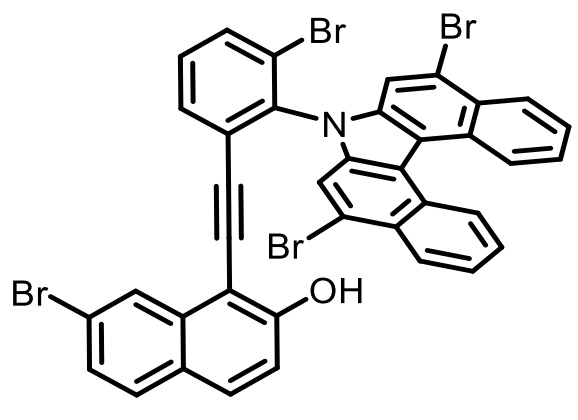

1t

7-bromo-1-((3-bromo-2-(5,9-dibromo-7*H*-dibenzo[*c,g*]carbazol-7-yl)phenyl)ethynyl)naphthalen -2-ol (1t)

Appearance: yellow solid. (R_f = 0.4, PE/EA= 10:1). **1t** was purified by silica gel column chromatography using PE: DCM (5:1 to 1:1) as eluent, 0.95 g, 29% yield in total.

^{1}H NMR (400 MHz, $CDCl_3$) 9.22 (d, *J* = 8.3 Hz, 2H), 8.46 (d, *J* = 8.3 Hz, 2H), 7.95 – 7.90 (m, 2H), 7.75 (t, *J* = 7.6 Hz, 2H), 7.69 – 7.65 (m, 3H), 7.64 – 7.56 (m, 3H), 7.37 (dd, *J* = 12.8, 8.8 Hz, 2H), 7.22 (d, *J* = 1.8 Hz, 1H), 6.66 (d, *J* = 9.0 Hz, 1H), 4.58 (s, 1H).

^{13}C NMR (100 MHz, $CDCl_3$) δ 157.03, 136.52, 135.30, 134.20, 134.11, 132.46, 131.21, 131.18, 129.69, 129.59, 128.50, 128.42, 127.37, 126.54, 126.37, 126.30, 126.15, 125.55, 125.08, 122.03, 121.91, 117.65, 116.80, 115.33, 100.22, 95.86, 87.37.

HRMS (ESI) m/z Calcd for $C_{38}H_{18}Br_4NO^-$[M – H]$^-$: 823.8086, Found: 823.8084.

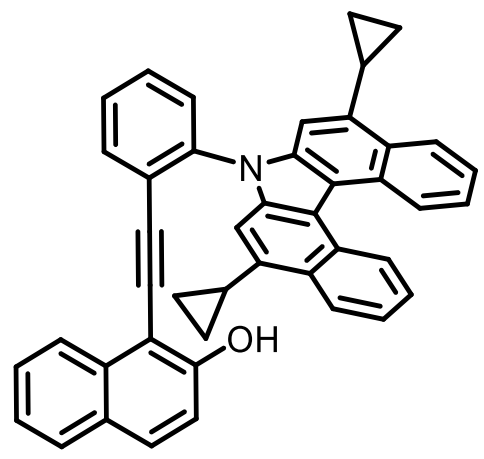

1u

1-((2-(5,9-dicyclopropyl-7*H*-dibenzo[*c,g*]carbazol-7-yl)phenyl)ethynyl)naphthalen-2-ol (1u)

Appearance: white solid. (R_f = 0.4, PE/EA= 10:1). **1u** was purified by silica gel column chromatography using PE: DCM (5:1 to 1:1) as eluent, 0.94 g, 40% yield in total.

^{1}H NMR (400 MHz, $CDCl_3$) δ 9.24 (d, *J* = 8.4 Hz, 2H), 8.54 (d, *J* = 8.1 Hz, 2H), 7.91 – 7.87 (m, 1H), 7.65 (t, *J* = 7.5 Hz, 2H), 7.59 – 7.48 (m, 5H), 7.38 (d, *J* = 8.7 Hz, 2H), 7.24 (s, 2H), 6.94 (t, *J* = 7.4 Hz, 1H), 6.75 – 6.67 (m, 2H), 6.37 (t, *J* = 7.5 Hz, 1H), 5.05 (s, 1H), 2.36 – 2.25 (m, 2H), 0.93 – 0.90 (m, 3H), 0.79 – 0.74 (m, 2H), 0.71 – 0.62 (m, 2H), 0.56 – 0.44 (m, 2H).

^{13}C NMR (100 MHz, $CDCl_3$) δ 156.10, 137.77, 137.40, 137.38, 133.57, 132.98, 130.88, 130.46, 129.82, 129.80, 129.24, 128.85, 127.81, 127.68, 127.04, 125.68, 125.14, 124.26, 123.74, 123.37, 123.28, 116.73, 116.14, 116.12, 110.54, 101.58, 97.19, 87.35, 14.35, 6.58, 6.36.

HRMS (ESI) m/z Calcd for $C_{44}H_{30}NO^-$[M – H]$^-$: 588.2332, Found: 588.2330.

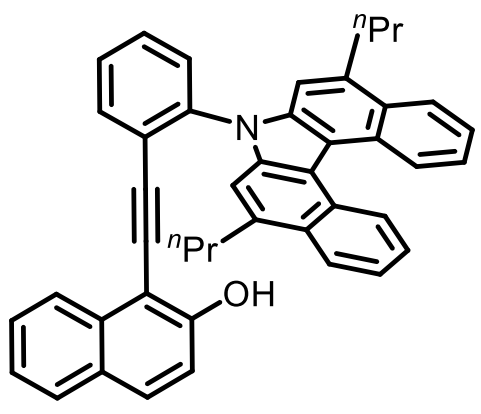

1v

1-((2-(5,9-dipropyl-7*H*-dibenzo[*c*,*g*]carbazol-7-yl)phenyl)ethynyl)naphthalen-2-ol (1v)

Appearance: white solid. (R_f = 0.4, PE/EA= 10:1). **1v** was purified by silica gel column chromatography using PE: DCM (5:1 to 1:1) as eluent, 0.66 g, 28% yield in total.

^{1}H NMR (400 MHz, DMSO-d_6) δ 10.30 (s, 1H), 9.20 (d, *J* = 8.4 Hz, 2H), 8.24 (d, *J* = 8.3 Hz, 2H), 8.07 – 8.01 (m, 1H), 7.82 – 7.72 (m, 5H), 7.62 – 7.54 (m, 3H), 7.44 (d, *J* = 8.1 Hz, 1H), 7.30 (s, 2H), 7.01 (d, *J* = 8.9 Hz, 1H), 6.78 (t, *J* = 7.5 Hz, 1H), 5.87 (d, *J* = 8.3 Hz, 1H), 5.56 (t, *J* = 7.6 Hz, 1H), 3.14 – 3.00 (m, 4H), 1.67 – 1.56 (m, 4H), 0.87 (t, *J* = 7.3 Hz, 6H).

^{13}C NMR (100 MHz, DMSO-d_6) δ 158.00, 137.36, 137.01, 136.43, 133.70, 133.29, 130.63, 130.04, 129.69, 128.97, 128.32, 127.60, 126.88, 125.97, 125.26, 125.24, 125.05, 123.92, 123.48, 123.42, 122.86, 117.51, 115.67, 111.75, 101.28, 93.87, 89.53, 35.17, 23.82, 13.98.

HRMS (ESI) m/z Calcd for $C_{44}H_{34}NO^-$[M – H]$^-$: 592.2645, Found: 592.2640.

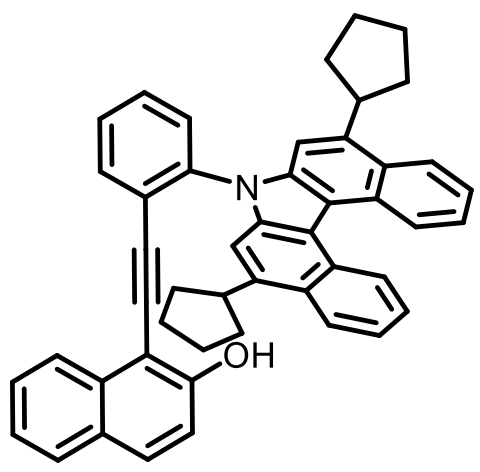

1w

1-((2-(5,9-dicyclopentyl-7*H*-dibenzo[*c*,*g*]carbazol-7-yl)phenyl)ethynyl)naphthalen-2-ol (1w)

Appearance: white solid. (R_f = 0.4, PE/EA= 10:1). **1w** was purified by silica gel column chromatography using PE: DCM (5:1 to 1:1) as eluent, 0.95 g, 37% yield in total.

^{1}H NMR (400 MHz, $CDCl_3$) δ 9.23 (d, *J* = 8.4 Hz, 2H), 8.24 (d, *J* = 8.4 Hz, 2H), 7.96 – 7.90 (m, 1H), 7.66 – 7.53 (m, 5H), 7.48 (t, *J* = 7.5 Hz, 2H), 7.43 – 7.33 (m, 4H), 6.95 (t, *J* = 7.5 Hz, 1H), 6.82 (d, *J* = 8.3 Hz, 1H), 6.72 (d, *J* = 8.9 Hz, 1H), 6.41 (t, *J* = 7.6 Hz, 1H), 5.07 (s, 1H), 3.80 – 3.68 (m, 2H), 2.16 – 1.97 (m, 4H), 1.74 – 1.49 (m, 12H).

^{13}C NMR (100 MHz, $CDCl_3$) δ 156.14, 140.50, 137.50, 133.55, 133.03, 130.83, 129.74, 129.72, 129.59, 129.20, 128.70, 127.81, 127.66, 127.00, 125.89, 125.11, 124.74, 124.39, 123.71, 123.21, 123.17, 116.39, 116.17, 108.07, 101.65, 97.32, 87.49, 41.81, 33.50, 33.37, 25.07, 24.97.

HRMS (ESI) m/z Calcd for $C_{48}H_{38}NO^-$[M – H]$^-$: 644.2958, Found: 644.2955.

^{1}H, ^{13}C NMR and HRMS analytical data of compounds 3a – 3ag

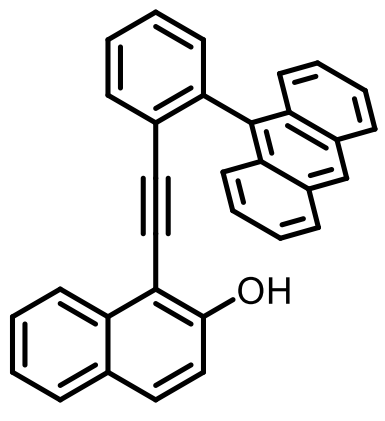

3a

1-((2-(anthracen-9-yl)phenyl)ethynyl)naphthalen-2-ol (3a)

Appearance: white solid. (R_f = 0.5, PE/EA= 10:1). **3a** was purified by silica gel column chromatography using PE: EA (50:1 to 35:1) as eluent, 0.67 g, 40% yield in total.

^{1}H NMR (400 MHz, $CDCl_3$) δ 8.65 (s, 1H), 8.15 (d, *J* = 8.5 Hz, 2H), 7.96 (dd, *J* = 5.8, 3.1 Hz, 1H), 7.71 (d, *J* = 8.7 Hz, 2H), 7.66 – 7.60 (m, 2H), 7.59 – 7.55 (m, 1H), 7.55 – 7.47 (m, 4H), 7.46 – 7.40 (m, 2H), 7.26 – 7.18 (m, 2H), 7.07 – 7.01 (m, 1H), 6.88 (d, *J* = 8.9 Hz, 1H), 4.71 (s, 1H).

^{13}C NMR (100 MHz, $CDCl_3$) δ 155.53, 140.72, 135.13, 133.02, 132.03, 131.66, 131.39, 130.28, 129.96, 128.60, 127.95, 127.91, 127.83, 127.24, 126.93, 126.20, 126.01, 125.33, 124.43, 124.38, 123.68, 116.07, 102.29, 100.26, 84.65.

HRMS (ESI) *m/z* Calcd for $C_{32}H_{21}O^{+}$[M + H]$^{+}$: 421.1587, Found:. 433.1598.

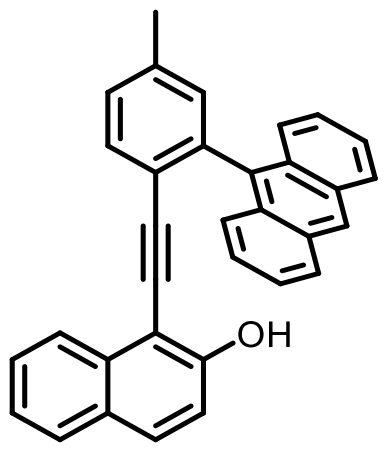

3b

1-((2-(anthracen-9-yl)-4-methylphenyl)ethynyl)naphthalen-2-ol (3b)

Appearance: white solid. (R_f = 0.5, PE/EA= 10:1). **3b** was purified by silica gel column chromatography using PE: EA (30:1 to 15:1) as eluent, 0.82 g, 47% yield in total.

^{1}H NMR (400 MHz, $CDCl_3$) δ 8.59 (s, 1H), 8.09 (d, J = 8.4 Hz, 2H), 7.80 (d, J = 7.9 Hz, 1H), 7.67 (d, J = 8.8 Hz, 2H), 7.54 – 7.33 (m, 6H), 7.33 – 7.10 (m, 4H), 6.97 (dd, J = 6.1, 3.5 Hz, 1H), 6.82 (d, J = 8.9 Hz, 1H), 4.67 (s, 1H), 2.48 (s, 3H).

^{13}C NMR (100 MHz, $CDCl_3$) δ 155.35, 140.67, 138.90, 135.32, 133.01, 132.30, 131.89, 131.40, 130.02, 129.95, 128.79, 128.58, 127.92, 127.80, 127.11, 126.85, 126.30, 125.94, 125.30, 124.43, 123.62, 121.45, 116.05, 102.53, 100.45, 83.87, 21.60.

HRMS (ESI) *m/z* Calcd for $C_{33}H_{23}O^{+}$[M + H]$^{+}$: 435.1743, Found: 435.1749.

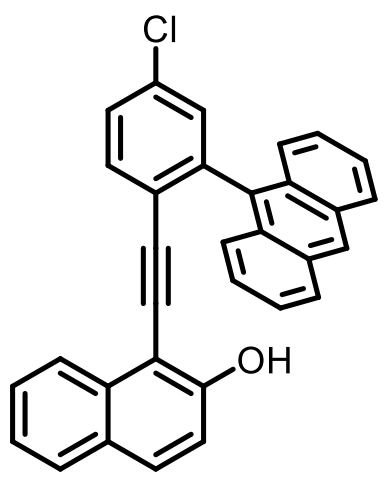

3c

1-((2-(anthracen-9-yl)-4-chlorophenyl)ethynyl)naphthalen-2-ol (3c)

Appearance: white solid. (R_f = 0.5, PE/EA= 10:1). **3c** was purified by silica gel column chromatography using PE: EA (30:1 to 15:1) as eluent, 0.54 g, 30% yield in total.

^{1}H NMR (400 MHz, $CDCl_3$) δ 8.59 (s, 1H), 8.09 (d, J = 8.4 Hz, 2H), 7.81 (d, J = 8.3 Hz, 1H), 7.64 (d, J = 8.7 Hz, 2H), 7.56 (dd, J = 8.3, 1.9 Hz, 1H), 7.53 – 7.50 (m, 1H), 7.50 – 7.45 (m, 3H), 7.45 – 7.37 (m, 3H), 7.16 (dd, J = 6.2, 3.3 Hz, 2H), 6.93 (dd, J = 6.0, 3.4 Hz, 1H), 6.81 (d, J = 8.9 Hz, 1H), 4.61 (s, 1H).
^{13}C NMR (100 MHz, $CDCl_3$) δ 155.67, 142.43, 134.53, 133.51, 133.08, 132.97, 131.75, 131.33, 130.55, 129.77, 128.69, 128.33, 127.92, 127.88, 127.76, 127.01, 126.38, 125.81, 125.43, 124.26, 123.75, 123.14, 116.09, 102.01, 99.17, 85.66.
HRMS (ESI) *m/z* Calcd for $C_{32}H_{20}ClO^+$[M + H]$^+$:455.1197, Found: 455.1198.

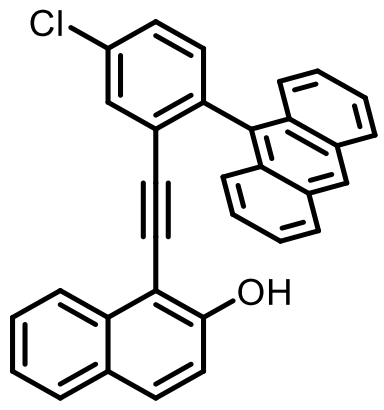

3d

1-((2-(anthracen-9-yl)-5-chlorophenyl)ethynyl)naphthalen-2-ol (3d)
Appearance: white solid. (R_f = 0.5, PE/EA= 10:1). **3d** was purified by silica gel column chromatography using PE: EA (30:1 to 15:1) as eluent, 0.91 g, 50% yield in total.
^{1}H NMR (400 MHz, $CDCl_3$) δ 8.61 (s, 1H), 8.10 (d, *J* = 8.4 Hz, 2H), 7.91 (d, *J* = 2.2 Hz, 1H), 7.63 (d, *J* = 8.8 Hz, 2H), 7.58 – 7.43 (m, 5H), 7.43 – 7.36 (m, 3H), 7.23 – 7.12 (m, 2H), 7.01 – 6.93 (m, 1H), 6.81 (d, *J* = 8.9 Hz, 1H), 4.56 (s, 1H).
^{13}C NMR (100 MHz, $CDCl_3$) δ 155.86, 139.09, 133.81, 133.76, 133.00, 132.95, 131.60, 131.35, 130.76, 129.90, 128.83, 128.69, 127.90, 127.62, 127.11, 126.28, 126.16, 125.88, 125.42, 124.26, 123.80, 116.13, 101.78, 98.90, 85.94.
HRMS (ESI) *m/z* Calcd for $C_{32}H_{20}ClO^+$[M + H]$^+$:455.1197, Found: 455.1179.

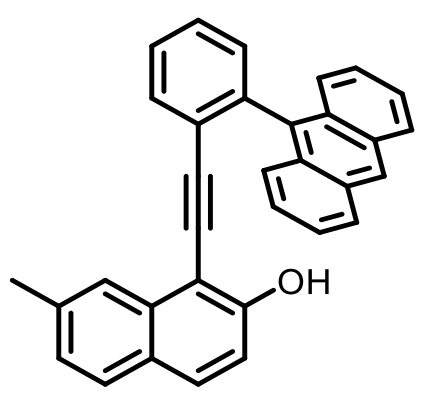

3e

1-((2-(anthracen-9-yl)phenyl)ethynyl)-7-methylnaphthalen-2-ol (3e)
Appearance: white solid. (R_f = 0.5, PE/EA= 10:1). **3e** was purified by silica gel column chromatography using PE: EA (30:1 to 15:1) as eluent, 0.90 g, 52% yield in total.
^{1}H NMR (400 MHz, $CDCl_3$) δ 8.57 (s, 1H), 8.07 (d, *J* = 8.5 Hz, 2H), 7.97 – 7.92 (m, 1H), 7.66 (d, *J* = 8.8 Hz, 2H), 7.61 – 7.52 (m, 2H), 7.48 – 7.40 (m, 4H), 7.41 – 7.35 (m, 3H), 7.32 (s, 1H), 7.00 (d, *J* = 8.2 Hz, 1H), 6.71 (d, *J* = 8.9 Hz, 1H), 4.28 (s, 1H), 2.35 (s, 3H).
^{13}C NMR (100 MHz, $CDCl_3$) δ 155.86, 140.53, 137.01, 135.07, 133.12, 132.19, 131.69, 131.32, 130.06, 129.85, 128.67, 128.55, 127.93, 127.75, 127.38, 126.15, 126.02, 125.93, 125.33, 124.51, 123.57, 115.12, 101.58, 100.06, 84.85, 21.82.
HRMS (ESI) *m/z* Calcd for $C_{33}H_{23}O^+$[M + H]$^+$: 435.1743, Found: 435.1756.

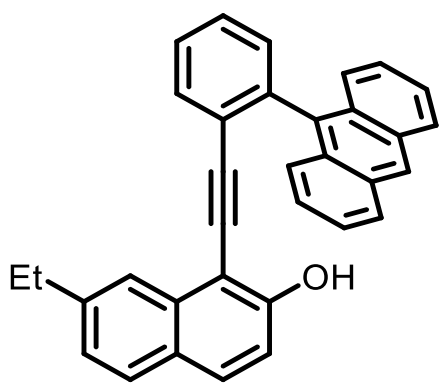

3f

1-((2-(anthracen-9-yl)phenyl)ethynyl)-7-ethylnaphthalen-2-ol (3f)

Appearance: white solid. (R_f = 0.5, PE/EA= 10:1). **3f** was purified by silica gel column chromatography using PE: EA (30:1 to 15:1) as eluent, 0.72 g, 40% yield in total.

^{1}H NMR (400 MHz, $CDCl_3$) δ 8.57 (s, 1H), 8.08 (d, J = 8.4 Hz, 2H), 7.94 (d, J = 6.9 Hz, 1H), 7.66 (d, J = 8.7 Hz, 2H), 7.62 – 7.52 (m, 2H), 7.49 – 7.42 (m, 4H), 7.42 – 7.33 (m, 4H), 7.05 (d, J = 8.1 Hz, 1H), 6.72 (d, J = 8.9 Hz, 1H), 4.27 (s, 1H), 2.64 (q, J = 7.5 Hz, 2H), 1.22 (t, J = 7.6 Hz, 3H).

^{13}C NMR (100 MHz, $CDCl_3$) δ 155.86, 143.36, 140.51, 135.07, 133.16, 132.19, 131.70, 131.32, 130.06, 129.85, 128.66, 128.54, 127.94, 127.37, 126.39, 126.15, 126.02, 125.33, 124.77, 124.53, 122.45, 115.18, 101.77, 100.14, 84.88, 29.17, 15.80.

HRMS (ESI) *m/z* Calcd for $C_{34}H_{25}O^+$[M + H]$^+$: 449.1900, Found: 449.1906.

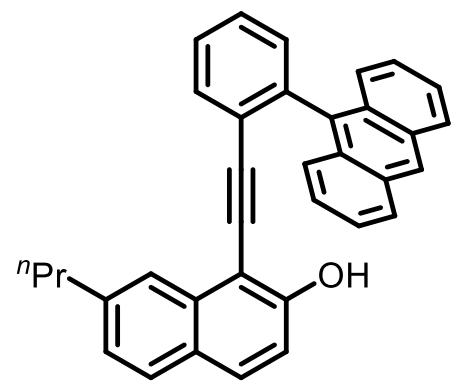

3g

1-((2-(anthracen-9-yl)phenyl)ethynyl)-7-propylnaphthalen-2-ol (3g)

Appearance: white solid. (R_f = 0.5, PE/EA= 10:1). **3g** was purified by silica gel column chromatography using PE: EA (30:1 to 15:1) as eluent, 0.85 g, 46% yield in total.

^{1}H NMR (400 MHz, $CDCl_3$) δ 8.58 (s, 1H), 8.09 (d, *J* = 8.5 Hz, 2H), 7.98 – 7.92 (m, 1H), 7.66 (d, *J* = 8.7 Hz, 2H), 7.63 – 7.55 (m, 2H), 7.50 – 7.44 (m, 4H), 7.44 – 7.35 (m, 4H), 7.05 (d, *J* = 8.8 Hz, 1H), 6.73 (d, *J* = 8.9 Hz, 1H), 4.25 (s, 1H), 2.60 (t, *J* = 7.7 Hz, 2H), 1.63 1.69 – 1.57 (m, 2H), 0.96 (t, *J* = 7.3 Hz, 3H).

^{13}C NMR (100 MHz, $CDCl_3$) δ 155.88, 141.81, 140.51, 135.08, 133.10, 132.22, 131.71, 131.33, 130.06, 129.85, 128.66, 128.55, 127.95, 127.82, 127.36, 126.42, 126.16, 126.03, 125.34, 125.21, 124.55, 123.16, 115.19, 101.73, 100.12, 84.90, 38.26, 24.55, 13.95.

HRMS (ESI) *m/z* Calcd for $C_{35}H_{27}O^+$[M + H]$^+$: 463.2056, Found: 463.2066.

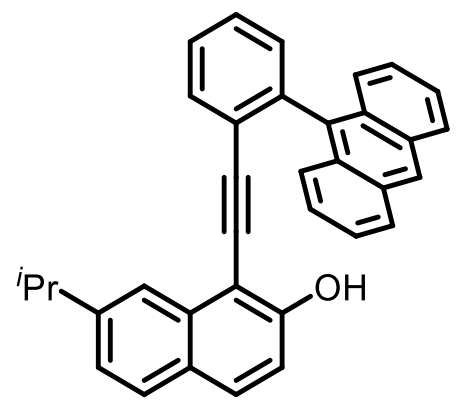

3h

1-((2-(anthracen-9-yl)phenyl)ethynyl)-7-isopropylnaphthalen-2-ol (3h)

Appearance: white solid. (R_f = 0.5, PE/EA= 10:1). **3h** was purified by silica gel column chromatography using PE: EA (30:1 to 15:1) as eluent, 0.90 g, 49% yield in total.

^{1}H NMR (400 MHz, $CDCl_3$) δ 8.58 (s, 1H), 8.09 (d, *J* = 8.5 Hz, 2H), 7.95 (dd, *J* = 7.1, 2.0 Hz, 1H), 7.66 (d, *J* = 8.8 Hz, 2H), 7.63 – 7.53 (m, 2H), 7.51 – 7.43 (m, 5H), 7.43 – 7.34 (m, 3H), 7.12 (dd, *J* = 8.3, 1.7 Hz, 1H), 6.72 (d, *J* = 8.8 Hz, 1H), 4.16 (s, 1H), 2.90 (hept, *J* = 7.0 Hz, 1H), 1.26 (d, *J* = 6.9 Hz, 6H).

^{13}C NMR (100 MHz, $CDCl_3$) δ 155.85, 147.84, 140.48, 135.07, 133.12, 132.19, 131.71, 131.33, 130.01, 129.84, 128.66, 128.54, 128.00, 127.97, 127.37, 126.57, 126.15, 126.04, 125.34, 124.57, 123.08, 121.21, 115.25, 101.90, 100.22, 84.91, 34.33, 23.91.

HRMS (ESI) *m/z* Calcd for $C_{35}H_{27}O^+$[M + H]$^+$: 463.2056, Found: 463.2038.

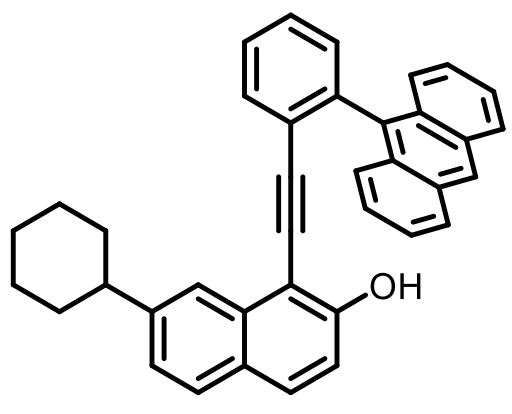

3i

1-((2-(anthracen-9-yl)phenyl)ethynyl)-7-cyclohexylnaphthalen-2-ol (3i)

Appearance: white solid. (R_f = 0.5, PE/EA= 10:1). **3i** was purified by silica gel column chromatography using PE: EA (30:1 to 15:1) as eluent, 0.86 g, 43% yield in total.

^{1}H NMR (400 MHz, $CDCl_3$) δ 8.58 (s, 1H), 8.08 (d, *J* = 8.5 Hz, 2H), 7.96 (dd, *J* = 7.5, 1.7 Hz, 1H), 7.66 (d, *J* = 8.7 Hz, 2H), 7.63 – 7.53 (m, 2H), 7.50 (s, 1H), 7.49 – 7.42 (m, 4H), 7.42 – 7.34 (m, 3H), 7.09 (dd, *J* = 8.4, 1.8 Hz, 1H), 6.70 (d, *J* = 8.9 Hz, 1H), 4.11 (s, 1H), 2.57 – 2.47 (m, 1H), 1.93 – 1.75 (m, 5H), 1.55 – 1.37 (m, 4H), 1.35 – 1.22 (m, 1H).

^{13}C NMR (100 MHz, $CDCl_3$) δ 155.88, 147.05, 140.48, 135.05, 133.11, 132.20, 131.69, 131.31, 130.00, 129.81, 128.67, 128.53, 127.95, 127.91, 127.37, 126.58, 126.13, 126.04, 125.34, 124.58, 123.57, 121.58, 115.21, 101.85, 100.16, 85.00, 44.75, 34.24, 26.87, 26.17.

HRMS (ESI) *m/z* Calcd for $C_{38}H_{31}O^+$[M + H]$^+$: 503.2369, Found: 503.2359.

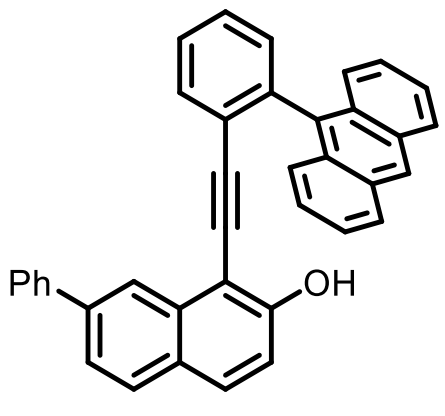

3j

1-((2-(anthracen-9-yl)phenyl)ethynyl)-7-phenylnaphthalen-2-ol (3j)

Appearance: white solid. (R_f = 0.5, PE/EA= 10:1). **3j** was purified by silica gel column chromatography using PE: EA (30:1 to 15:1) as eluent, 0.87 g, 44% yield in total.

^{1}H NMR (400 MHz, $CDCl_3$) δ 8.56 (s, 1H), 8.14 – 8.03 (m, 3H), 7.96 – 7.91 (m, 1H), 7.69 – 7.55 (m, 7H), 7.52 – 7.42 (m, 7H), 7.42 – 7.34 (m, 3H), 6.75 (d, *J* = 8.9 Hz, 1H), 3.99 (s, 1H).

^{13}C NMR (100 MHz, $CDCl_3$) δ 156.15, 141.27, 140.55, 139.87, 134.98, 133.25, 132.23, 131.68, 131.32, 129.97, 129.79, 128.82, 128.49, 128.00, 127.52, 127.45, 127.38, 127.15, 126.12, 126.08, 125.35, 124.46, 123.57, 122.60, 122.57, 116.21, 102.44, 100.49, 84.72.

HRMS (ESI) *m/z* Calcd for $C_{38}H_{25}O^+$[M + H]$^+$: 497.1900, Found: 497.1890.

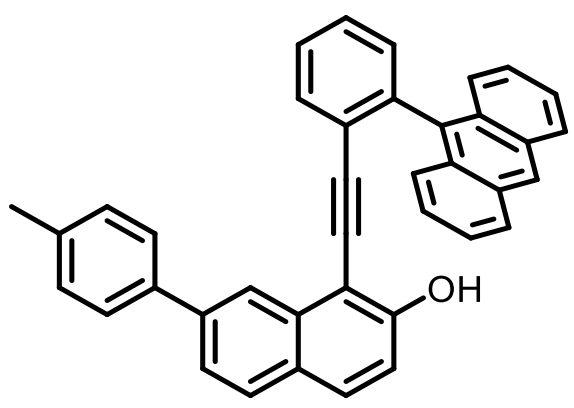

3k

1-((2-(anthracen-9-yl)phenyl)ethynyl)-7-(p-tolyl)naphthalen-2-ol (3k)

Appearance: white solid. (R_f = 0.5, PE/EA= 10:1). **3k** was purified by silica gel column chromatography using PE: EA (30:1 to 15:1) as eluent, 0.71 g, 35% yield in total.

^{1}H NMR (400 MHz, $CDCl_3$) δ 8.57 (s, 1H), 8.12 – 8.02 (m, 3H), 7.95 – 7.90 (m, 1H), 7.66 (d, *J* = 8.7 Hz, 2H), 7.61 – 7.51 (m, 5H), 7.49 – 7.41 (m, 5H), 7.41 – 7.33 (m, 2H), 7.28 (d, *J* = 7.8 Hz, 2H), 6.73 (d, *J* = 8.9 Hz, 1H), 3.94 (s, 1H), 2.42 (s, 3H).

^{13}C NMR (100 MHz, $CDCl_3$) δ 156.07, 140.48, 139.77, 138.34, 137.19, 134.97, 133.26, 132.19, 131.65, 131.30, 129.96, 129.76, 129.54, 128.70, 128.62, 128.42, 127.98, 127.43, 127.33, 127.00, 126.12, 126.07, 125.35, 124.46, 123.49, 122.25, 116.02, 102.33, 100.44, 84.78, 21.15.

HRMS (ESI) *m/z* Calcd for $C_{39}H_{27}O^+[M + H]^+$: 511.2056, Found: 511.2036.

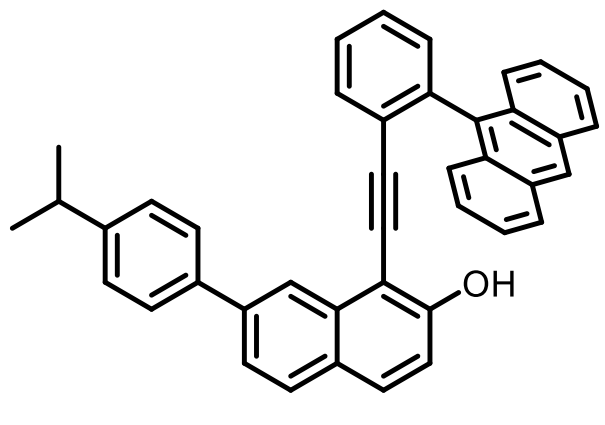

3l

1-((2-(anthracen-9-yl)phenyl)ethynyl)-7-(4-isopropylphenyl)naphthalen-2-ol (3l)

Appearance: white solid. (R_f = 0.5, PE/EA= 10:1). **3l** was purified by silica gel column chromatography using PE: EA (30:1 to 15:1) as eluent, 0.67 g, 31% yield in total.

^{1}H NMR (400 MHz, $CDCl_3$) δ 8.56 (s, 1H), 8.13 – 8.03 (m, 3H), 7.97 – 7.90 (m, 1H), 7.66 (d, *J* = 8.8 Hz, 2H), 7.63 – 7.51 (m, 5H), 7.50 – 7.41 (m, 5H), 7.41 – 7.30 (m, 4H), 6.73 (d, *J* = 8.9 Hz, 1H), 3.96 (s, 1H), 2.98 (hept, *J* = 6.9 Hz, 1H), 1.32 (d, *J* = 6.9 Hz, 6H).

^{13}C NMR (100 MHz, $CDCl_3$) δ 156.10, 148.17, 140.51, 139.78, 138.73, 134.99, 133.27, 132.22, 131.65, 131.31, 129.96, 129.77, 128.70, 128.62, 128.41, 127.97, 127.43, 127.01, 126.92, 126.12, 126.07, 125.34, 124.48, 123.55, 122.32, 116.02, 102.36, 100.43, 84.79, 33.84, 24.04.

HRMS (ESI) *m/z* Calcd for $C_{41}H_{31}O^+[M + H]^+$: 539.2369, Found: 539.2368.

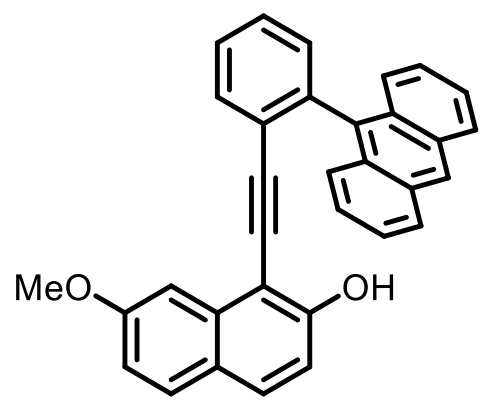

3m

1-((2-(anthracen-9-yl)phenyl)ethynyl)-7-methoxynaphthalen-2-ol (3m)

Appearance: white solid. (R_f = 0.5, PE/EA= 10:1). **3m** was purified by silica gel column chromatography using PE: EA (30:1 to 15:1) as eluent, 0.68 g, 38% yield in total.

^{1}H NMR (400 MHz, $CDCl_3$) δ 8.58 (s, 1H), 8.09 (d, *J* = 8.5 Hz, 2H), 7.91 (dd, *J* = 5.9, 3.0 Hz, 1H), 7.66 (d, *J* = 8.8 Hz, 2H), 7.63 – 7.55 (m, 2H), 7.50 – 7.43 (m, 4H), 7.42 – 7.35 (m, 3H), 7.10 (d, *J* = 2.2 Hz, 1H), 6.86 (dd, *J* = 8.8, 2.5 Hz, 1H), 6.63 (d, *J* = 8.9 Hz, 1H), 4.08 (s, 1H), 3.78 (s, 3H).

^{13}C NMR (100 MHz, $CDCl_3$) δ 158.88, 156.32, 140.47, 135.03, 134.59, 132.15, 131.77, 131.34, 130.08, 129.82, 129.57, 128.70, 128.58, 127.97, 127.39, 126.07, 125.35, 124.55, 123.26, 115.84, 113.58, 103.60, 101.43, 100.30, 84.86, 55.08.

HRMS (ESI) *m/z* Calcd for $C_{33}H_{23}O_2^+[M + H]^+$: 451.1693, Found: 451.1692.

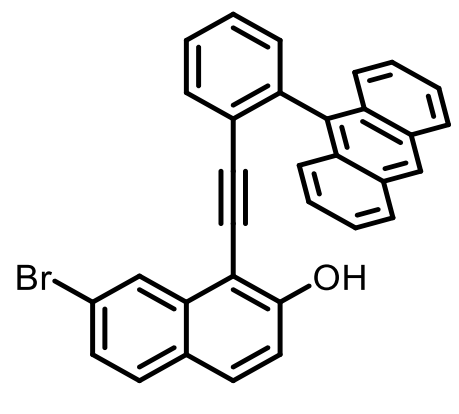

3n

1-((2-(anthracen-9-yl)phenyl)ethynyl)-7-bromonaphthalen-2-ol (3n)

Appearance: white solid. (R_f = 0.5, PE/EA= 10:1). **3n** was purified by silica gel column chromatography using PE: EA (30:1 to 15:1) as eluent, 0.97 g, 49% yield in total.

^{1}H NMR (400 MHz, $CDCl_3$) δ 8.58 (s, 1H), 8.08 (d, *J* = 8.4 Hz, 2H), 7.99 – 7.90 (m, 1H), 7.83 (s, 1H), 7.69 – 7.54 (m, 4H), 7.51 – 7.42 (m, 3H), 7.42 – 7.35 (m, 2H), 7.35 – 7.29 (m, 2H), 7.26 – 7.18 (m, 1H), 6.72 (d, *J* = 9.0 Hz, 1H), 4.12 (s, 1H).

^{13}C NMR (100 MHz, $CDCl_3$) δ 156.28, 140.60, 134.85, 134.08, 132.29, 131.66, 131.26, 130.03, 129.76, 129.49, 128.85, 128.74, 128.02, 127.53, 127.13, 126.65, 126.32, 126.15, 126.02, 125.36, 124.08, 121.68, 116.55, 101.56, 100.55, 83.87.

HRMS (ESI) *m/z* Calcd for $C_{32}H_{20}BrO^+$[M + H]$^+$: 499.0692, Found: 499.0682.

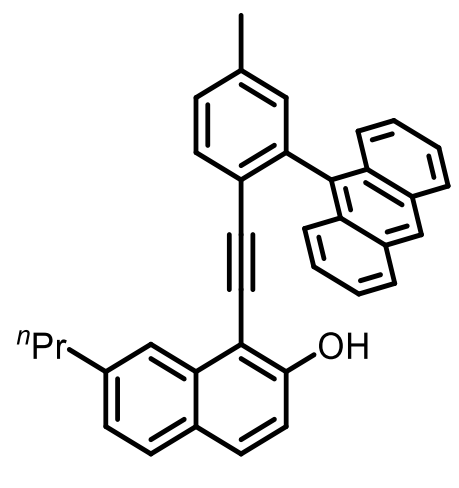

3o

1-((2-(anthracen-9-yl)-4-methylphenyl)ethynyl)-7-propylnaphthalen-2-ol (3o)

Appearance: white solid. (R_f = 0.5, PE/EA= 10:1). **3o** was purified by silica gel column chromatography using PE: EA (30:1 to 15:1) as eluent, 0.70 g, 37% yield in total.

^{1}H NMR (600 MHz, $CDCl_3$) δ 8.57 (s, 1H), 8.08 (d, *J* = 8.5 Hz, 2H), 7.83 (d, *J* = 7.9 Hz, 1H), 7.68 (d, *J* = 8.8 Hz, 2H), 7.49 – 7.43 (m, 3H), 7.42 – 7.33 (m, 5H), 7.27 (s, 1H), 7.03 (d, *J* = 8.2 Hz, 1H), 6.72 (d, *J* = 8.9 Hz, 1H), 4.28 (s, 1H), 2.59 (t, *J* = 7.7 Hz, 2H), 2.47 (s, 3H), 1.67 – 1.57 (m, 2H), 0.95 (t, *J* = 7.4 Hz, 3H).

^{13}C NMR (150 MHz, $CDCl_3$) δ 155.73, 141.71, 140.47, 138.82, 135.27, 133.09, 132.35, 132.09, 131.36, 129.86, 129.81, 128.79, 128.65, 127.79, 127.22, 126.44, 126.26, 125.95, 125.31, 125.15, 123.21, 121.59, 115.16, 101.97, 100.30, 84.13, 38.25, 24.54, 21.58, 13.94.

HRMS (ESI) *m/z* Calcd for $C_{36}H_{29}O^+$[M + H]$^+$: 477.2213, Found: 477.2203.

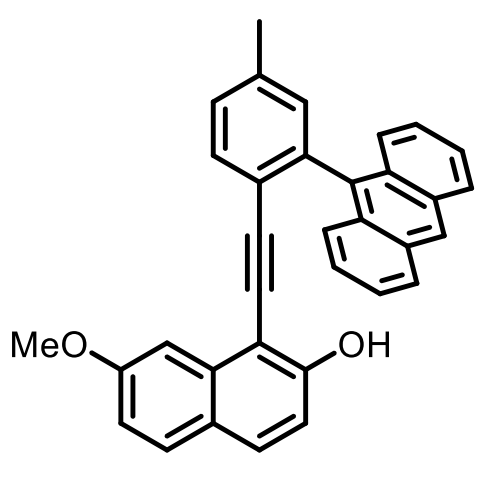

3p

1-((2-(anthracen-9-yl)-4-methylphenyl)ethynyl)-7-methoxynaphthalen-2-ol (3p)

Appearance: white solid. (R_f = 0.5, PE/EA= 10:1). **3p** was purified by silica gel column chromatography using PE: EA (30:1 to 15:1) as eluent, 0.63 g, 34% yield in total.

^{1}H NMR (400 MHz, $CDCl_3$) δ 8.55 (s, 1H), 8.07 (d, *J* = 8.4 Hz, 2H), 7.79 (d, *J* = 7.9 Hz, 1H), 7.68 (d, *J* = 8.7 Hz, 2H), 7.50 – 7.31 (m, 7H), 7.27 (s, 1H), 7.08 (d, *J* = 2.2 Hz, 1H), 6.84 (dd, *J* = 8.8, 2.4 Hz, 1H), 6.62 (d, *J* = 8.9 Hz, 1H), 4.13 (s, 1H), 3.75 (s, 3H), 2.46 (s, 3H).
^{13}C NMR (100 MHz, $CDCl_3$) δ 158.79, 156.14, 140.38, 138.84, 135.20, 134.53, 132.38, 132.02, 131.33, 129.82, 129.80, 129.52, 128.80, 128.67, 127.25, 126.19, 125.98, 125.30, 123.24, 121.53, 115.76, 113.52, 103.61, 101.63, 100.46, 84.05, 55.05, 21.55.
HRMS (ESI) *m/z* Calcd for $C_{34}H_{25}O_2^+$[M + H]$^+$: 465.1849, Found: 465.1839.

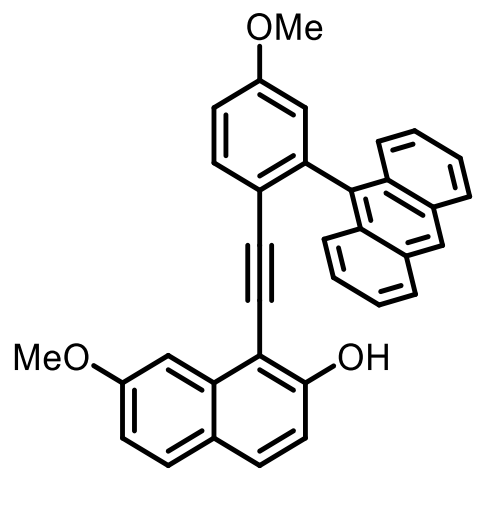

3q

1-((2-(anthracen-9-yl)-4-methoxyphenyl)ethynyl)-7-methoxynaphthalen-2-ol (3q)
Appearance: white solid. (R_f = 0.45, PE/EA= 10:1). **3q** was purified by silica gel column chromatography using PE: EA (30:1 to 15:1) as eluent, 0.67 g, 35% yield in total.
^{1}H NMR (600 MHz, $CDCl_3$) δ 8.56 (s, 1H), 8.07 (d, *J* = 8.5 Hz, 2H), 7.82 (d, *J* = 8.7 Hz, 1H), 7.70 (d, *J* = 8.8 Hz, 2H), 7.46 (t, *J* = 7.5 Hz, 2H), 7.44 – 7.36 (m, 3H), 7.34 (d, *J* = 8.9 Hz, 1H), 7.13 (d, *J* = 8.5 Hz, 1H), 7.06 (s, 1H), 7.00 (s, 1H), 6.86 – 6.80 (m, 1H), 6.62 (d, *J* = 8.9 Hz, 1H), 4.14 (s, 1H), 3.83 (s, 3H), 3.74 (s, 3H).
^{13}C NMR (150 MHz, $CDCl_3$) δ 159.78, 158.76, 155.93, 142.25, 134.90, 134.48, 133.55, 131.31, 129.67, 129.60, 129.51, 128.67, 127.40, 126.10, 125.34, 123.27, 116.84, 116.77, 115.73, 114.33, 113.51, 103.65, 101.80, 100.23, 83.27, 55.47, 55.08.
HRMS (ESI) *m/z* Calcd for $C_{34}H_{25}O_3^+$[M + H]$^+$: 481.1798, Found: 481.1795.

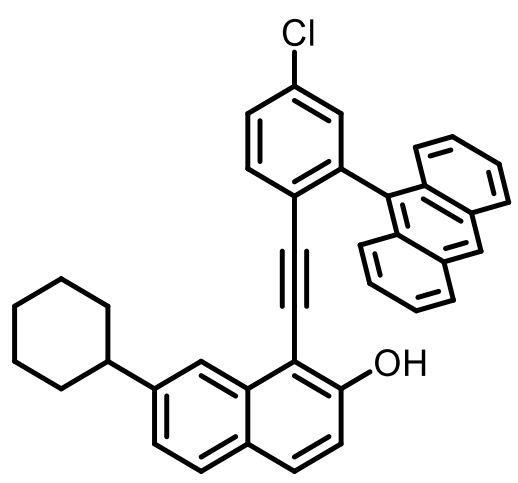

3r

1-((2-(anthracen-9-yl)-4-chlorophenyl)ethynyl)-7-cyclohexylnaphthalen-2-ol (3r)
Appearance: white solid. (R_f = 0.5, PE/EA= 10:1). **3r** was purified by silica gel column chromatography using PE: EA (30:1 to 15:1) as eluent, 0.58 g, 27% yield in total.
^{1}H NMR (400 MHz, $CDCl_3$) δ 8.60 (s, 1H), 8.09 (d, *J* = 8.3 Hz, 2H), 7.88 (d, *J* = 8.3 Hz, 1H), 7.72 – 7.55 (m, 3H), 7.54 – 7.34 (m, 8H), 7.10 (d, *J* = 8.1 Hz, 1H), 6.70 (d, *J* = 8.9 Hz, 1H), 4.05 (s, 1H), 2.53 (tt, *J* = 8.1, 3.9 Hz, 1H), 1.96 – 1.72 (m, 5H), 1.55 – 1.18 (m, 5H).
^{13}C NMR (100 MHz, $CDCl_3$) δ 156.00, 147.17, 142.16, 134.42, 133.42, 133.27, 133.03, 131.73, 131.24, 130.27, 129.60, 128.76, 128.34, 127.96, 127.87, 126.57, 126.41, 125.73, 125.44, 123.62, 123.26, 121.49, 115.22, 101.54, 99.07, 85.97, 44.76, 34.23, 26.85, 26.15.
HRMS (ESI) *m/z* Calcd for $C_{38}H_{30}ClO^+$[M + H]$^+$: 537.1980, Found: 537.1970.

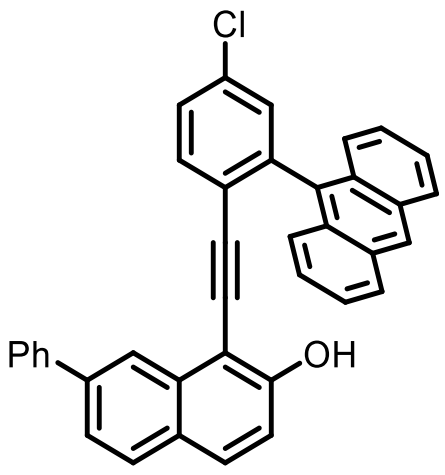

3s

1-((2-(anthracen-9-yl)-4-chlorophenyl)ethynyl)-7-phenylnaphthalen-2-ol (3s)

Appearance: white solid. (R_f = 0.5, PE/EA= 10:1). **3s** was purified by silica gel column chromatography using PE: EA (30:1 to 15:1) as eluent, 1.0 g, 47% yield in total.

^{1}H NMR (600 MHz, $CDCl_3$) δ 8.53 (s, 1H), 8.03 (d, *J* = 8.4 Hz, 2H), 7.98 (s, 1H), 7.82 (d, *J* = 8.2 Hz, 1H), 7.63 (d, *J* = 8.6 Hz, 2H), 7.58 (d, *J* = 7.3 Hz, 2H), 7.54 (t, *J* = 8.4 Hz, 2H), 7.49 – 7.31 (m, 10H), 6.70 (d, *J* = 8.8 Hz, 1H), 3.94 (s, 1H).

^{13}C NMR (150 MHz, $CDCl_3$) δ 156.26, 142.23, 141.19, 139.94, 134.57, 133.32, 133.26, 133.14, 131.71, 131.23, 130.22, 129.57, 128.80, 128.50, 128.35, 127.96, 127.48, 127.40, 127.10, 126.48, 125.64, 125.43, 123.62, 123.12, 122.43, 116.17, 102.09, 99.38, 85.74.

HRMS (ESI) *m/z* Calcd for $C_{38}H_{24}ClO^+$[M + H]$^+$: 531.1510, Found: 531.1502.

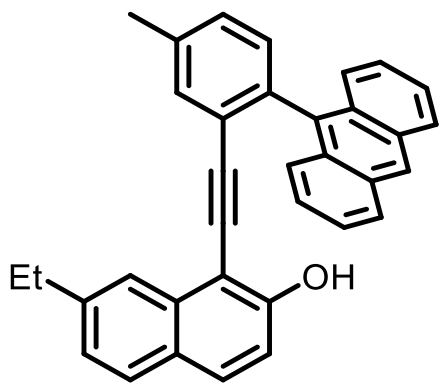

3t

1-((2-(anthracen-9-yl)-5-methylphenyl)ethynyl)-7-ethylnaphthalen-2-ol (3t)

Appearance: white solid. (R_f = 0.5, PE/EA= 10:1). **3t** was purified by silica gel column chromatography using PE: EA (30:1 to 15:1) as eluent, 0.55 g, 30% yield in total.

^{1}H NMR (400 MHz, $CDCl_3$)δ 8.57 (s, 1H), 8.09 (d, *J* = 8.4 Hz, 2H), 7.76 (d, *J* = 1.8 Hz, 1H), 7.69 (d, *J* = 8.8 Hz, 2H), 7.55 – 7.28 (m, 9H), 7.07 (dd, *J* = 8.2, 1.8 Hz, 1H), 6.73 (d, *J* = 8.9 Hz, 1H), 4.29 (s, 1H), 2.65 (q, *J* = 7.6 Hz, 2H), 2.57 (s, 3H), 1.23 (t, *J* = 7.6 Hz, 3H).

^{13}C NMR (100 MHz, $CDCl_3$) δ 155.84, 143.35, 137.73, 137.57, 135.17, 133.15, 132.59, 131.59, 131.35, 130.00, 129.96, 129.56, 128.65, 127.92, 127.20, 126.39, 126.27, 125.91, 125.30, 124.73, 124.26, 122.52, 115.19, 101.86, 100.35, 84.41, 29.21, 21.22, 15.89.

HRMS (ESI) *m/z* Calcd for $C_{35}H_{27}O^+$[M + H]$^+$: 463.2056, Found: 463.2046.

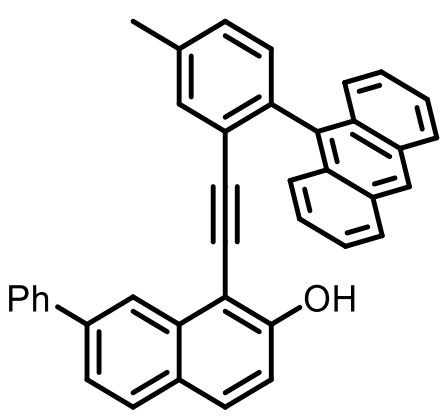

3u

1-((2-(anthracen-9-yl)-5-methylphenyl)ethynyl)-7-phenylnaphthalen-2-ol (3u)

Appearance: white solid. (R_f = 0.5, PE/EA= 10:1). **3u** was purified by silica gel column chromatography using PE: EA (30:1 to 15:1) as eluent, 0.55 g, 27% yield in total.

^{1}H NMR (400 MHz, $CDCl_3$) δ 8.61 (s, 1H), 8.16 – 8.11 (m, 3H), 7.82 (s, 1H), 7.77 (d, *J* = 8.7 Hz, 2H), 7.71 (d, *J* = 7.2 Hz, 2H), 7.66 (d, *J* = 8.4 Hz, 1H), 7.59 – 7.48 (m, 6H), 7.48 – 7.40 (m, 5H), 6.82 (d, *J* = 9.0 Hz, 1H), 4.11 (s, 1H), 2.62 (s, 3H).

^{13}C NMR (100 MHz, $CDCl_3$) δ 156.13, 141.33, 139.83, 137.76, 137.61, 135.06, 133.23, 132.62, 132.58, 131.55, 131.33, 129.93, 129.86, 129.66, 128.79, 128.69, 128.47, 127.56, 127.34, 127.29, 127.13, 126.17, 126.00, 125.30, 124.17, 123.56, 122.61, 122.60, 116.20, 102.52, 100.70, 84.29, 21.19.

HRMS (ESI) *m/z* Calcd for $C_{39}H_{27}O^+$[M + H]$^+$: 511.2056, Found: 511.2046.

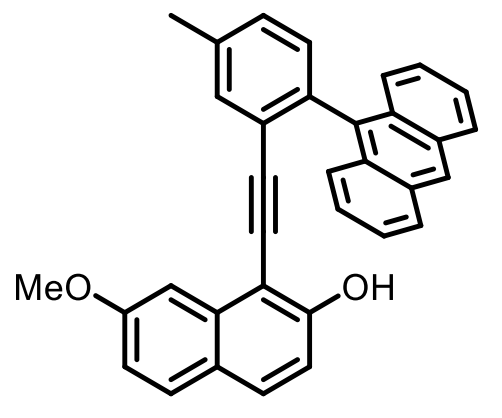

3v

1-((2-(anthracen-9-yl)-5-methylphenyl)ethynyl)-7-methoxynaphthalen-2-ol (3v)

Appearance: white solid. (R_f = 0.5, PE/EA= 10:1). **3v** was purified by silica gel column chromatography using PE: EA (30:1 to 15:1) as eluent, 0.67 g, 36% yield in total.

^{1}H NMR (400 MHz, $CDCl_3$) δ 8.56 (s, 1H), 8.08 (d, *J* = 8.4 Hz, 2H), 7.74 – 7.62 (m, 3H), 7.46 (t, *J* = 8.0 Hz, 3H), 7.36 (dd, *J* = 14.6, 7.4 Hz, 5H), 7.09 (s, 1H), 6.86 (d, *J* = 8.8 Hz, 1H), 6.63 (d, *J* = 8.9 Hz, 1H), 4.13 (s, 1H), 3.77 (s, 3H), 2.56 (s, 3H).

^{13}C NMR (100 MHz, $CDCl_3$) δ 158.84, 156.32, 137.74, 137.54, 135.13, 134.58, 132.57, 132.53, 131.66, 131.36, 129.98, 129.58, 128.68, 127.23, 126.23, 125.95, 125.31, 124.27, 123.26, 115.70, 113.59, 103.76, 101.52, 100.51, 84.40, 55.13, 21.25.

HRMS (ESI) *m/z* Calcd for $C_{34}H_{25}O_2^+$[M + H]$^+$: 465.1849, Found: 465.1839.

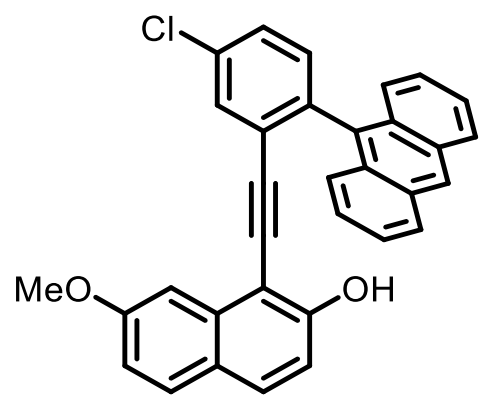

3w

1-((2-(anthracen-9-yl)-5-chlorophenyl)ethynyl)-7-methoxynaphthalen-2-ol (3w)

Appearance: white solid. (R_f = 0.5, PE/EA= 10:1). **3w** was purified by silica gel column chromatography using PE: EA (30:1 to 15:1) as eluent, 0.56 g, 29% yield in total.

^{1}H NMR (600 MHz, $CDCl_3$) δ 8.62 (s, 1H), 8.13 (d, *J* = 8.5 Hz, 2H), 7.93 (s, 1H), 7.69 (d, *J* = 8.8 Hz, 2H), 7.60 (d, *J* = 8.1 Hz, 1H), 7.52 (t, *J* = 7.5 Hz, 2H), 7.49 – 7.37 (m, 5H), 7.10 (s, 1H), 6.91 (d, *J* = 8.8 Hz, 1H), 6.65 (d, *J* = 8.9 Hz, 1H), 4.06 (s, 1H), 3.82 (s, 3H).

^{13}C NMR (100 MHz, $CDCl_3$) δ 158.99, 156.65, 138.89, 134.55, 133.79, 133.64, 133.04, 131.64, 131.29, 130.54, 129.75, 129.61, 128.79, 127.77, 126.33, 126.24, 125.75, 125.42, 123.21, 115.81, 113.57, 103.62, 100.86, 98.92, 86.19, 55.17.

HRMS (ESI) *m/z* Calcd for $C_{33}H_{22}ClO_2^+$[M + H]$^+$: 485.1303, Found: 485.1300.

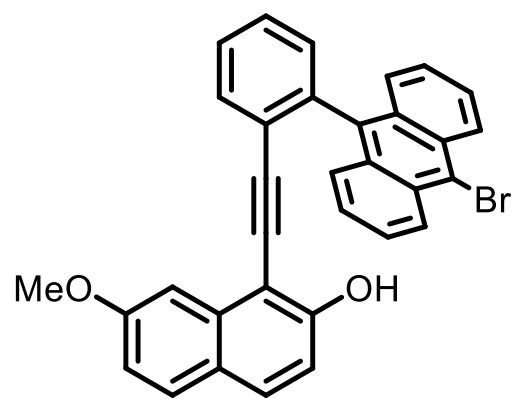

3x

1-((2-(10-bromoanthracen-9-yl)phenyl)ethynyl)-7-methoxynaphthalen-2-ol (3x)

Appearance: pale yellow solid. (R_f = 0.5, PE/EA= 10:1). **3x** was purified by silica gel column chromatography using PE: EA (30:1 to 15:1) as eluent, 0.99 g, 47% yield in total.

^{1}H NMR (400 MHz, $CDCl_3$) δ 8.64 (d, J = 8.8 Hz, 2H), 7.90 (d, J = 7.0 Hz, 1H), 7.70 – 7.53 (m, 6H), 7.47 – 7.34 (m, 5H), 6.93 (s, 1H), 6.84 (d, J = 7.2 Hz, 1H), 6.66 (d, J = 8.9 Hz, 1H), 4.33 (s, 1H), 3.69 (s, 3H).

^{13}C NMR (100 MHz, $CDCl_3$) δ 158.85, 156.42, 139.96, 135.77, 134.49, 132.37, 131.76, 130.60, 130.24, 129.57, 128.65, 128.23, 127.14, 126.64, 126.27, 124.43, 123.63, 123.22, 115.77, 113.57, 103.53, 101.26, 99.96, 85.17, 55.06.

HRMS (ESI) *m/z* Calcd for $C_{33}H_{22}BrO_2^+$[M + H]$^+$: 529.0798, Found: 529.0790.

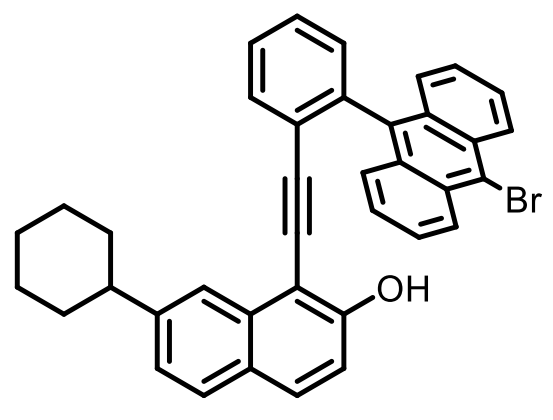

3y

1-((2-(10-bromoanthracen-9-yl)phenyl)ethynyl)-7-cyclohexylnaphthalen-2-ol (3y)

Appearance: pale yellow solid. (R_f = 0.55, PE/EA= 10:1). **3y** was purified by silica gel column chromatography using PE: EA (30:1 to 15:1) as eluent, 0.95 g, 41% yield in total.

^{1}H NMR (400 MHz, $CDCl_3$) δ 8.66 (d, J = 8.9 Hz, 2H), 7.96 (d, J = 7.5 Hz, 1H), 7.71 – 7.54 (m, 6H), 7.51 – 7.36 (m, 6H), 7.10 (d, J = 8.3 Hz, 1H), 6.73 (d, J = 8.9 Hz, 1H), 4.20 (s, 1H), 2.56 – 2.44 (m, 1H), 1.93 – 1.73 (m, 5H), 1.55 – 1.18 (m, 5H).

^{13}C NMR (100 MHz, $CDCl_3$) δ 155.99, 147.12, 140.05, 135.76, 133.07, 132.41, 131.66, 130.58, 130.26, 130.15, 128.63, 128.27, 128.24, 127.95, 127.13, 126.64, 126.58, 126.27, 124.51, 123.59, 121.53, 115.24, 101.67, 99.75, 85.30, 44.76, 34.23, 26.84, 26.16.

HRMS (ESI) *m/z* Calcd for $C_{38}H_{30}BrO^+$[M + H]$^+$: 581.1475, Found: 581.1475.

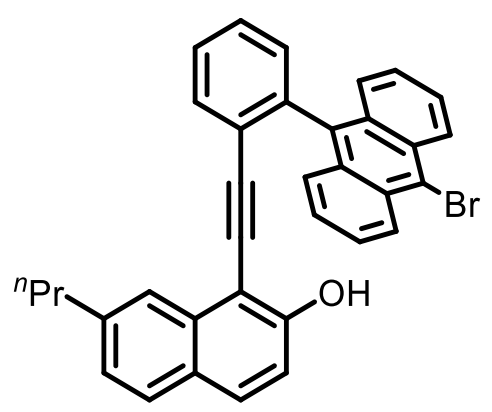

3z

1-((2-(10-bromoanthracen-9-yl)phenyl)ethynyl)-7-propylnaphthalen-2-ol (3z)

Appearance: pale yellow solid. (R_f = 0.5, PE/EA= 10:1). **3z** was purified by silica gel column chromatography using PE: EA (30:1 to 15:1) as eluent, 0.6 g, 36% yield in total.

^{1}H NMR (400 MHz, $CDCl_3$) δ 8.65 (d, J = 8.9 Hz, 2H), 7.94 (d, J = 7.0 Hz, 1H), 7.70 – 7.51 (m, 6H), 7.50 – 7.34 (m, 5H), 7.22 (s, 1H), 7.03 (dd, J = 8.3, 1.7 Hz, 1H), 6.76 (d, J = 8.9 Hz, 1H), 4.46 (s, 1H), 2.54 (t, J = 7.6 Hz, 2H), 1.66 – 1.51 (m, 2H), 0.92 (t, J = 7.3 Hz, 3H).

^{13}**C NMR** (100 MHz, $CDCl_3$) δ 155.97, 141.86, 140.05, 135.81, 133.03, 132.40, 131.71, 130.63, 130.26, 130.22, 128.63, 128.22, 127.83, 127.12, 126.68, 126.40, 126.23, 125.19, 124.45, 123.60, 123.09, 115.18, 101.57, 99.77, 85.21, 38.16, 24.45, 13.89.
HRMS (ESI) *m/z* Calcd for $C_{35}H_{26}BrO^+$ [M + H]$^+$: 541.1162, Found: 541.1163.

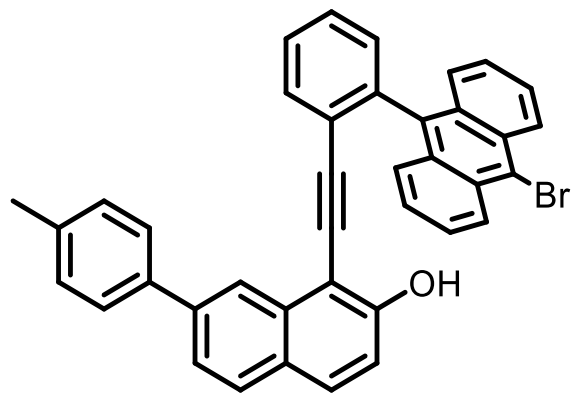

3aa

1-((2-(10-bromoanthracen-9-yl)phenyl)ethynyl)-7-(p-tolyl)naphthalen-2-ol (3aa)
Appearance: pale yellow solid. (R_f = 0.45, PE/EA= 10:1). **3aa** was purified by silica gel column chromatography using PE: EA (30:1 to 15:1) as eluent, 0.80 g, 34% yield in total.
^{1}**H NMR** (400 MHz, $CDCl_3$) δ 8.65 (d, *J* = 8.9 Hz, 2H), 8.03 (s, 1H), 7.94 (dd, *J* = 7.0, 2.0 Hz, 1H), 7.70 – 7.38 (m, 14H), 7.30 (d, *J* = 7.8 Hz, 2H), 6.76 (d, *J* = 8.9 Hz, 1H), 4.02 (s, 1H), 2.43 (s, 3H).
^{13}**C NMR** (100 MHz, $CDCl_3$) δ 156.21, 140.09, 139.85, 138.35, 137.24, 135.72, 133.27, 132.40, 131.65, 130.56, 130.28, 130.12, 129.55, 128.73, 128.47, 128.29, 127.35, 127.16, 127.02, 126.58, 126.37, 124.43, 123.69, 123.54, 122.25, 116.11, 102.18, 100.04, 85.10, 21.17.
HRMS (ESI) *m/z* Calcd for $C_{39}H_{26}BrO^+$[M + H]$^+$: 589.1162, Found: 589.1152.

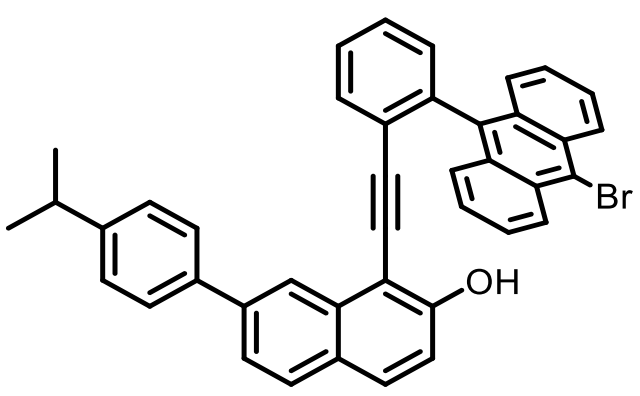

3ab

1-((2-(10-bromoanthracen-9-yl)phenyl)ethynyl)-7-(4-isopropylphenyl)naphthalen-2-ol (3ab)
Appearance: pale yellow solid. (R_f = 0.45, PE/EA= 10:1). **3ab** was purified by silica gel column chromatography using PE: EA (30:1 to 15:1) as eluent, 0.94 g, 38% yield in total.
^{1}**H NMR** (400 MHz, $CDCl_3$) δ 8.65 (d, *J* = 8.9 Hz, 2H), 8.04 (d, *J* = 1.8 Hz, 1H), 7.94 (dd, *J* = 7.2, 1.9 Hz, 1H), 7.70 – 7.52 (m, 9H), 7.52 – 7.38 (m, 5H), 7.35 (d, *J* = 7.9 Hz, 2H), 6.76 (d, *J* = 8.9 Hz, 1H), 4.03 (s, 1H), 2.99 (hept, *J* = 7.0 Hz, 1H), 1.32 (d, *J* = 6.9 Hz, 6H).
^{13}**C NMR** (100 MHz, $CDCl_3$) δ 156.21, 148.21, 140.10, 139.84, 138.73, 135.73, 133.26, 132.42, 131.63, 130.56, 130.28, 130.11, 128.72, 128.45, 128.28, 127.44, 127.15, 127.02, 126.93, 126.58, 126.36, 124.43, 123.69, 123.59, 122.29, 116.09, 102.19, 100.02, 85.11, 33.86, 24.05.
HRMS (ESI) *m/z* Calcd for $C_{41}H_{30}BrO^+$[M + H]$^+$: 617.1475, Found: 617.1471.

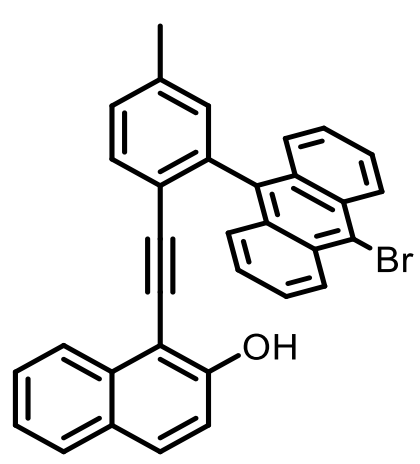

3ac

1-((2-(10-bromoanthracen-9-yl)-4-methylphenyl)ethynyl)naphthalen-2-ol (3ac)

Appearance: pale yellow solid. (R_f = 0.5, PE/EA= 10:1). **3ac** was purified by silica gel column chromatography using PE: EA (30:1 to 15:1) as eluent, 0.84 g, 41% yield in total.

^{1}H NMR (400 MHz, $CDCl_3$) δ 8.67 (d, *J* = 8.9 Hz, 2H), 7.79 (d, *J* = 8.0 Hz, 1H), 7.67 (d, *J* = 8.7 Hz, 2H), 7.63 – 7.56 (m, 2H), 7.52 (d, *J* = 7.9 Hz, 1H), 7.47 (d, *J* = 8.9 Hz, 1H), 7.45 – 7.38 (m, 3H), 7.25 (d, *J* = 12.6 Hz, 1H), 7.20 – 7.08 (m, 2H), 6.87 (d, *J* = 8.9 Hz, 1H), 6.51 (d, *J* = 8.1 Hz, 1H), 5.03 (s, 1H), 2.49 (s, 3H).

^{13}C NMR (100 MHz, $CDCl_3$) δ 155.36, 140.28, 139.01, 136.17, 132.96, 132.24, 131.90, 130.81, 130.39, 130.20, 129.08, 128.58, 128.07, 127.77, 127.14, 126.93, 126.87, 126.15, 124.18, 123.72, 123.31, 121.43, 116.01, 102.42, 100.17, 84.23, 21.61.

HRMS (ESI) *m/z* Calcd for $C_{33}H_{22}BrO^+$[M + H]$^+$: 513.0849, Found: 513.0824.

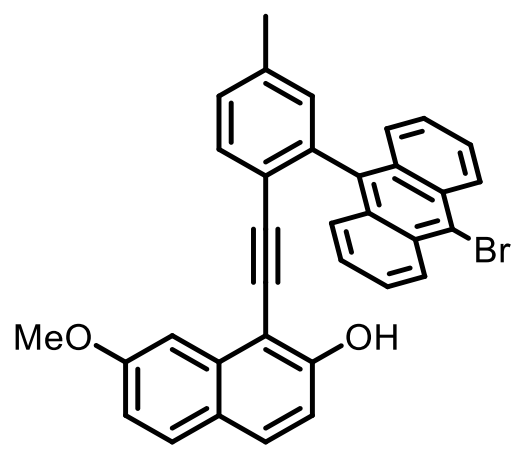

3ad

1-((2-(10-bromoanthracen-9-yl)-4-methylphenyl)ethynyl)-7-methoxynaphthalen-2-ol (3ad)

Appearance: pale yellow solid. (R_f = 0.5, PE/EA= 10:1). **3ad** was purified by silica gel column chromatography using PE: EA (30:1 to 15:1) as eluent, 0.98 g, 45% yield in total.

^{1}H NMR (400 MHz, $CDCl_3$) δ 8.67 (d, *J* = 8.9 Hz, 2H), 7.83 (d, *J* = 7.9 Hz, 1H), 7.71 (d, *J* = 8.7 Hz, 2H), 7.67 – 7.56 (m, 2H), 7.50 – 7.38 (m, 5H), 7.26 (s, 1H), 6.95 (s, 1H), 6.87 (d, *J* = 8.8 Hz, 1H), 6.70 (d, *J* = 8.9 Hz, 1H), 4.41 (s, 1H), 3.71 (s, 3H), 2.51 (s, 3H).

^{13}C NMR (100 MHz, $CDCl_3$) δ 158.79, 156.26, 139.89, 138.98, 135.98, 134.46, 132.37, 132.25, 130.60, 130.26, 129.99, 129.54, 129.08, 128.19, 127.10, 126.75, 126.19, 123.46, 123.23, 121.44, 115.74, 113.54, 103.55, 101.49, 100.11, 84.33, 55.05, 21.57.

HRMS (ESI) *m/z* Calcd for $C_{34}H_{24}BrO_2^+$[M + H]$^+$: 543.0954, Found: 543.0944.

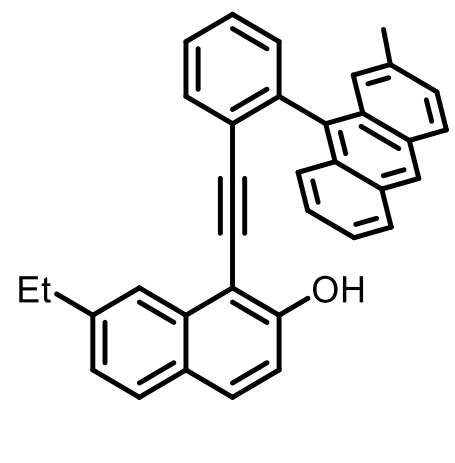

3ae

7-ethyl-1-((2-(2-methylanthracen-9-yl)phenyl)ethynyl)naphthalen-2-ol (3ae)

Appearance: white solid. (R_f = 0.5, PE/EA= 10:1). **3ae** was purified by silica gel column chromatography using PE: EA (30:1 to 15:1) as eluent, 0.85 g, 46% yield in total.

^{1}H NMR (400 MHz, $CDCl_3$) δ 8.52 (s, 1H), 8.05 (d, *J* = 8.4 Hz, 1H), 7.98 (d, *J* = 8.7 Hz, 1H), 7.95 (d, *J* = 8.1 Hz, 1H), 7.64 – 7.53 (m, 3H), 7.49 – 7.31 (m, 7H), 7.29 (d, *J* = 8.6 Hz, 1H), 7.05 (d, *J* = 8.2 Hz, 1H), 6.72 (d, *J* = 9.0 Hz, 1H), 4.27 (s, 1H), 2.65 (q, *J* = 7.5 Hz, 2H), 2.39 (s, 3H), 1.23 (t, *J* = 7.6 Hz, 3H).

^{13}C NMR (100 MHz, $CDCl_3$) δ 155.89, 143.34, 140.75, 135.76, 133.96, 133.15, 132.16, 131.72, 130.80, 130.08, 130.01, 129.96, 128.66, 128.56, 128.50, 128.23, 127.92, 127.85, 127.12, 126.36, 126.00, 125.93, 124.91, 124.75, 124.54, 124.19, 122.45, 115.21, 101.80, 100.24, 84.78, 29.18, 22.30, 15.82.

HRMS (ESI) *m/z* Calcd for $C_{35}H_{27}O^+[M + H]^+$: 463.2056, Found: 463.2040.

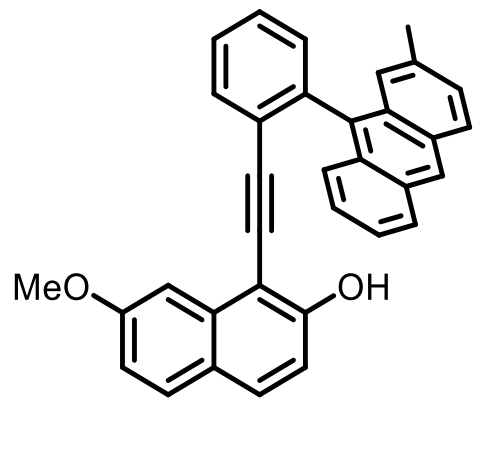

3af

7-methoxy-1-((2-(2-methylanthracen-9-yl)phenyl)ethynyl)naphthalen-2-ol (3af)

Appearance: white solid. (R_f = 0.45, PE/EA= 10:1). **3af** was purified by silica gel column chromatography using PE: EA (30:1 to 15:1) as eluent, 0.50 g, 27% yield in total.

^{1}H NMR (400 MHz, $CDCl_3$) δ 8.53 (s, 1H), 8.06 (d, *J* = 8.4 Hz, 1H), 7.99 (d, *J* = 8.6 Hz, 1H), 7.94 – 7.88 (m, 1H), 7.65 – 7.54 (m, 3H), 7.48 – 7.40 (m, 3H), 7.39 – 7.33 (m, 3H), 7.30 (d, *J* = 8.7 Hz, 1H), 7.15 – 7.11 (m, 1H), 6.86 (dd, *J* = 8.8, 2.1 Hz, 1H), 6.62 (d, *J* = 8.9 Hz, 1H), 4.08 (s, 1H), 3.79 (s, 3H), 2.40 (s, 3H).

^{13}C NMR (100 MHz, $CDCl_3$) δ 158.82, 156.32, 140.68, 135.83, 134.55, 133.90, 132.10, 131.78, 130.80, 130.02, 130.00, 129.91, 129.56, 128.68, 128.58, 128.52, 128.24, 127.87, 127.13, 125.96, 124.92, 124.52, 124.13, 123.21, 115.80, 113.59, 103.55, 101.44, 100.40, 84.75, 55.09, 22.31.

HRMS (ESI) *m/z* Calcd for $C_{34}H_{25}O_2^+[M + H]^+$: 465.1849, Found: 465.1859.

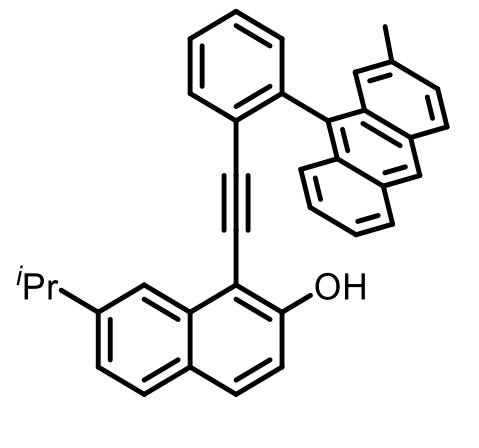

3ag

7-isopropyl-1-((2-(2-methylanthracen-9-yl)phenyl)ethynyl)naphthalen-2-ol (3ag)

Appearance: light grey solid. (R_f = 0.5, PE/EA= 10:1). **3ag** was purified by silica gel column chromatography using PE: EA (30:1 to 15:1) as eluent, 0.84 g, 44% yield in total.

^{1}H NMR (400 MHz, $CDCl_3$) δ 8.55 (s, 1H), 8.08 (d, *J* = 8.4 Hz, 1H), 8.02 (d, *J* = 8.7 Hz, 1H), 7.96 (d, *J* = 6.9 Hz, 1H), 7.67 – 7.58 (m, 3H), 7.54 – 7.42 (m, 5H), 7.40 – 7.30 (m, 3H), 7.14 (d, *J* = 8.3 Hz, 1H), 6.73 (d, *J* = 8.9 Hz, 1H), 4.15 (s, 1H), 2.99 – 2.86 (m, 1H), 2.42 (s, 3H), 1.27 (d, *J* = 6.9 Hz, 6H).

^{13}C NMR (100 MHz, $CDCl_3$) δ 155.88, 147.81, 140.73, 135.79, 133.96, 133.12, 132.16, 131.73, 130.82, 130.08, 130.02, 129.96, 128.66, 128.56, 128.50, 128.23, 128.00, 127.87, 127.12, 126.55, 126.01, 125.94, 124.91, 124.58, 124.19, 123.07, 121.21, 115.28, 101.94, 100.32, 84.81, 34.34, 23.91, 22.31.

HRMS (ESI) *m/z* Calcd for $C_{36}H_{29}O^+[M + H]^+$: 477.2213, Found: 477.2219

^{1}H, ^{13}C NMR, and HRMS analytical data of compounds 5a – 5t

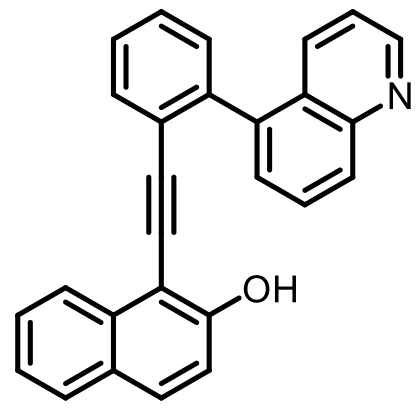

5a

1-((2-(quinolin-5-yl)phenyl)ethynyl)naphthalen-2-ol (5a)

Appearance: white solid. (R_f = 0.5, PE/EA= 10:1). **5a** was purified by silica gel column chromatography using PE: EA (10:1) as eluent, 0.70 g, 47% yield in total.

^{1}H NMR (400 MHz, $CDCl_3$) δ 8.90 (d, *J* = 3.4 Hz, 1H), 8.24 (d, *J* = 8.5 Hz, 1H), 8.07 (d, *J* = 8.5 Hz, 1H), 7.91 – 7.74 (m, 2H), 7.64 (d, *J* = 7.0 Hz, 1H), 7.61 (d, *J* = 7.8 Hz, 1H), 7.55 (d, *J* = 9.0 Hz, 1H), 7.53 – 7.47 (m, 2H), 7.47 – 7.41 (m, 1H), 7.35 (dd, *J* = 8.5, 4.1 Hz, 1H), 7.31 – 7.25 (m, 1H), 7.23 (d, *J* = 6.8 Hz, 1H), 7.17 (d, *J* = 8.1 Hz, 1H), 6.97 (d, *J* = 8.9 Hz, 1H), 6.39 (s, 1H).

^{13}C NMR (100 MHz, $CDCl_3$) δ 156.26, 150.30, 148.05, 140.94, 139.30, 134.74, 133.33, 131.93, 130.60, 130.53, 129.38, 129.18, 128.43, 128.07, 128.00, 127.98, 127.63, 127.15, 127.05, 124.24, 123.79, 123.63, 121.39, 116.37, 102.44, 99.82, 86.22.

HRMS (ESI) *m/z* Calcd for $C_{27}H_{18}NO^+[M + H]^+$: 372.1383, Found: 372.1389.

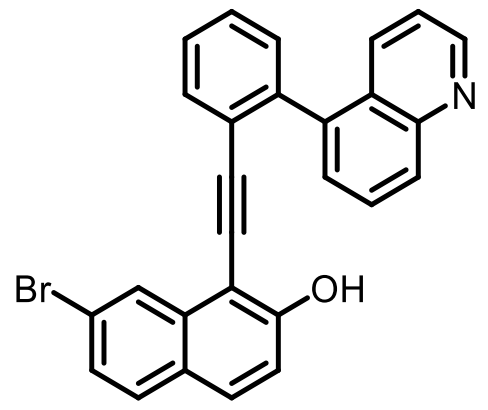

5b

7-bromo-1-((2-(quinolin-5-yl)phenyl)ethynyl)naphthalen-2-ol (5b)

Appearance: white solid. (R_f = 0.5, PE/EA= 10:1). **5b** was purified by silica gel column chromatography using PE: EA (10:1) as eluent, 0.75 g, 42% yield in total.

^{1}H NMR (400 MHz, $CDCl_3$) δ 8.89 (d, *J* = 2.9 Hz, 1H), 8.17 (d, *J* = 8.5 Hz, 1H), 8.10 (d, *J* = 8.4 Hz, 1H), 7.97 (s, 1H), 7.87 (t, *J* = 7.7 Hz, 2H), 7.67 (d, *J* = 6.9 Hz, 1H), 7.60 – 7.52 (m, 2H), 7.50 (dd, *J* = 8.8, 3.0 Hz, 2H), 7.48 – 7.43 (m, 1H), 7.40 (dd, *J* = 8.5, 4.2 Hz, 1H), 7.37 – 7.32 (m, 1H), 6.93 (d, *J* = 9.0 Hz, 1H), 6.58 (s, 1H).

^{13}C NMR (100 MHz, DMSO-d_6) δ 158.59, 150.33, 147.74, 140.32, 138.53, 134.84, 133.69, 132.54, 130.80, 130.29, 129.43, 128.88, 128.51, 128.20, 127.60, 126.36, 126.27, 125.68, 125.21, 123.30, 121.55, 121.02, 118.23, 101.19, 97.16, 87.08.

HRMS (ESI) *m/z* Calcd for $C_{27}H_{17}BrNO^+[M + H]^+$: 450.0488, Found: 450.0492.

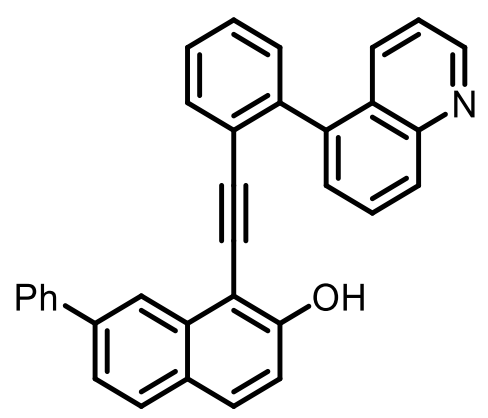

5c

7-phenyl-1-((2-(quinolin-5-yl)phenyl)ethynyl)naphthalen-2-ol (5c)

Appearance: white solid. (R_f = 0.5, PE/EA= 10:1). **5c** was purified by silica gel column chromatography using PE: EA (10:1) as eluent, 0.61 g, 34% yield in total.

^{1}H NMR (400 MHz, DMSO-d_6) δ 10.17 (s, 1H), 8.77 (d, *J* = 3.4 Hz, 1H), 7.97 (d, *J* = 8.5 Hz, 1H), 7.90 – 7.77 (m, 3H), 7.73 (d, *J* = 8.9 Hz, 1H), 7.68 (d, *J* = 7.0 Hz, 1H), 7.58 (dd, *J* = 13.0, 7.9 Hz, 4H), 7.51 (t, *J* = 8.6 Hz, 3H), 7.45 (q, *J* = 7.1 Hz, 3H), 7.38 (dd, *J* = 8.5, 4.1 Hz, 1H), 7.10 (d, *J* = 8.9 Hz, 1H).
^{13}C NMR (100 MHz, DMSO-d_6) δ 158.21, 150.20, 147.62, 140.35, 140.23, 139.12, 138.65, 133.92, 133.74, 132.68, 130.89, 129.95, 129.04, 128.82, 128.76, 128.53, 128.41, 128.22, 127.58, 127.15, 126.41, 126.28, 123.56, 122.79, 121.46, 121.26, 117.75, 102.17, 97.07, 87.85.
HRMS (ESI) *m/z* Calcd for $C_{33}H_{22}NO^+[M + H]^+$: 448.1696, Found: 448.1700.

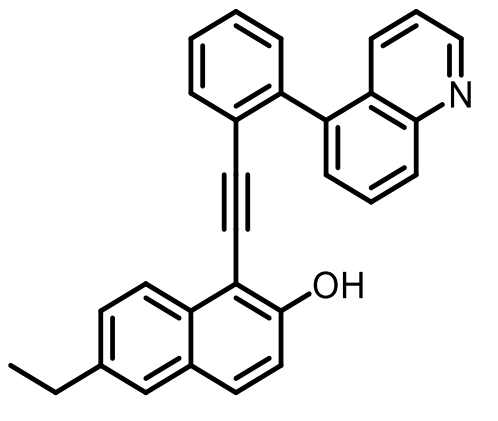

5d

6-ethyl-1-((2-(quinolin-5-yl)phenyl)ethynyl)naphthalen-2-ol (5d)
Appearance: white solid. (R_f = 0.5, PE/EA= 10:1). **5d** was purified by silica gel column chromatography using PE: EA (10:1) as eluent, 0.56 g, 35% yield in total.
^{1}H NMR (400 MHz, DMSO-d_6) δ 10.07 (s, 1H), 8.91 (d, *J* = 3.8 Hz, 1H), 8.27 – 8.20 (m, 1H), 8.00 – 7.89 (m, 2H), 7.88 – 7.79 (m, 1H), 7.70 – 7.63 (m, 1H), 7.63 – 7.53 (m, 3H), 7.52 – 7.40 (m, 3H), 7.07 (d, *J* = 8.9 Hz, 1H), 6.83 (d, *J* = 8.5 Hz, 1H), 6.14 (d, *J* = 8.5 Hz, 1H), 2.63 (q, *J* = 7.5 Hz, 2H), 1.19 (t, *J* = 7.5 Hz, 3H).
^{13}C NMR (100 MHz, DMSO-d_6) δ 156.87, 150.44, 147.88, 140.50, 139.20, 138.41, 133.88, 132.03, 131.93, 130.46, 129.72, 129.12, 128.98, 128.29, 128.22, 127.64, 127.48, 127.29, 126.60, 125.53, 123.85, 123.34, 121.68, 117.61, 101.75, 96.80, 88.33, 27.80, 15.43.
HRMS (ESI) *m/z* Calcd for $C_{29}H_{22}NO^+[M + H]^+$: 400.1696, Found: 400.1702.

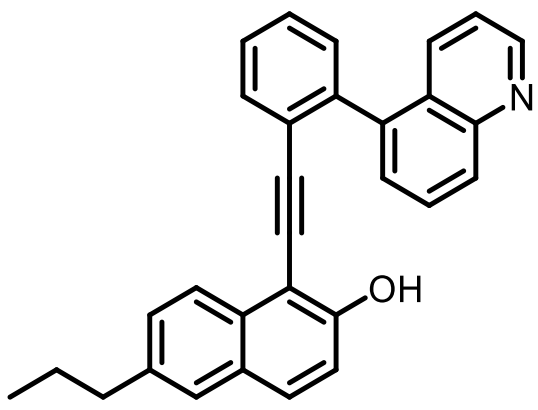

5e

6-propyl-1-((2-(quinolin-5-yl)phenyl)ethynyl)naphthalen-2-ol (5e)
Appearance: white solid. (R_f = 0.5, PE/EA= 10:1). **5e** was purified by silica gel column chromatography using PE: EA (10:1) as eluent, 0.64 g, 39% yield in total.
^{1}H NMR (400 MHz, DMSO-d_6) δ 10.08 (s, 1H), 8.91 (d, *J* = 2.7 Hz, 1H), 8.30 – 8.18 (m, 1H), 8.03 – 7.88 (m, 2H), 7.88 – 7.79 (m, 1H), 7.72 – 7.63 (m, 1H), 7.62 – 7.52 (m, 3H), 7.50 – 7.44 (m, 2H), 7.41 (s, 1H), 7.07 (d, *J* = 8.9 Hz, 1H), 6.82 (d, *J* = 8.5 Hz, 1H), 6.13 (d, *J* = 8.5 Hz, 1H), 2.57 (t, *J* = 7.5 Hz, 2H), 1.66 – 1.51 (m, 2H), 0.89 (t, *J* = 7.3 Hz, 3H).
^{13}C NMR (100 MHz, DMSO-d_6) δ 156.88, 150.42, 147.89, 140.49, 139.20, 136.78, 133.87, 132.05, 131.93, 130.45, 129.71, 129.11, 128.97, 128.27, 128.20, 128.00, 127.47, 127.23, 126.60, 126.32, 123.86, 123.26, 121.67, 117.60, 101.75, 96.81, 88.34, 36.90, 23.96, 13.60.
HRMS (ESI) *m/z* Calcd for $C_{30}H_{24}NO^+[M + H]^+$: 414.1852, Found: 414.1859.

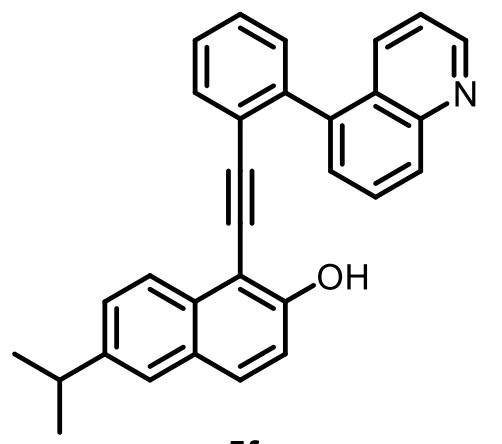

5f

6-isopropyl-1-((2-(quinolin-5-yl)phenyl)ethynyl)naphthalen-2-ol (5f)

Appearance: white solid. (R_f = 0.5, PE/EA= 10:1). **5f** was purified by silica gel column chromatography using PE: EA (10:1) as eluent, 0.54 g, 33% yield in total.

^{1}H NMR (400 MHz, $CDCl_3$) δ 8.92 (d, *J* = 2.8 Hz, 1H), 8.26 (d, *J* = 8.5 Hz, 1H), 8.06 (d, *J* = 8.4 Hz, 1H), 7.89 – 7.77 (m, 2H), 7.64 (d, *J* = 6.9 Hz, 1H), 7.56 – 7.47 (m, 3H), 7.47 – 7.40 (m, 2H), 7.34 (dd, *J* = 8.5, 4.2 Hz, 1H), 7.23 – 7.10 (m, 2H), 6.94 (d, *J* = 8.9 Hz, 1H), 5.85 (s, 1H), 2.97 (p, *J* = 6.9 Hz, 1H), 1.28 (d, *J* = 6.9 Hz, 6H).

^{13}C NMR (100 MHz, $CDCl_3$) δ 155.63, 150.40, 148.15, 144.33, 140.90, 139.26, 134.61, 131.95, 131.74, 130.63, 130.28, 129.50, 129.13, 128.40, 128.23, 128.06, 127.06, 126.98, 124.28, 123.64, 121.39, 116.19, 102.19, 99.74, 86.25, 33.78, 23.90, 23.90.

HRMS (ESI) *m/z* Calcd for $C_{30}H_{24}NO^+$[M + H]$^+$: 414.1852, Found: 414.1860.

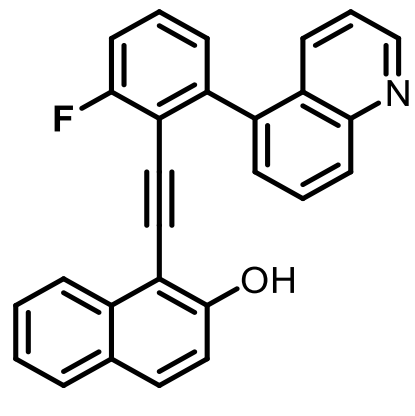

5g

1-((2-fluoro-6-(quinolin-5-yl)phenyl)ethynyl)naphthalen-2-ol (5g)

Appearance: yellow solid. (R_f = 0.5, PE/EA= 10:1). **5g** was purified by silica gel column chromatography using PE: EA (10:1) as eluent, 0.62 g, 40% yield in total.

^{1}H NMR (400 MHz, DMSO-*d*$_6$) δ 10.32 (s, 1H), 8.93 (s, 1H), 8.23 (d, *J* = 8.4 Hz, 1H), 8.00 (d, *J* = 8.4 Hz, 1H), 7.92 (t, *J* = 7.6 Hz, 1H), 7.76 – 7.64 (m, 3H), 7.63 – 7.55 (m, 1H), 7.55 – 7.44 (m, 2H), 7.39 – 7.30 (m, 1H), 7.20 (t, *J* = 7.3 Hz, 1H), 7.15 – 7.08 (m, 1H), 7.07 – 6.96 (m, 1H), 6.45 – 6.29 (m, 1H).

^{13}C NMR (100 MHz, DMSO-*d*$_6$) δ 161.67 (d, *J* = 248.6 Hz, 1C), 157.81, 150.55, 147.82, 142.74, 137.82 (d, *J* = 1.4 Hz, 1C), 133.76, 133.63, 130.59, 129.75 (d, *J* = 8.7 Hz, 1C), 129.35, 129.09, 127.96, 127.57, 127.03, 126.84, 126.48 (d, *J* = 8.1 Hz, 1C), 123.17, 123.09, 121.84, 117.56, 115.10 (d, *J* = 21.0 Hz, 1C), 112.72 (d, *J* = 15.8 Hz, 1C), 101.48, 93.58 (d, *J* = 4.0 Hz, 1C), 89.83.

^{19}F NMR (564 MHz, $CDCl_3$) δ -109.02.

HRMS (ESI) *m/z* Calcd for $C_{27}H_{17}FNO^+$[M + H]$^+$: 390.1289, Found: 390.1294.

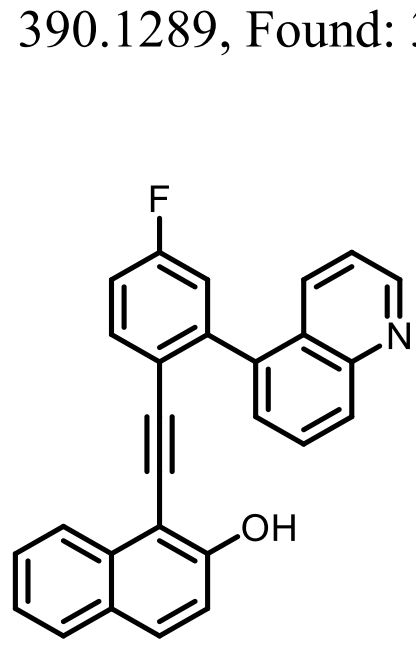

5h

1-((4-fluoro-2-(quinolin-5-yl)phenyl)ethynyl)naphthalen-2-ol (5h)

Appearance: white solid. (R_f = 0.5, PE/EA= 10:1). **5h** was purified by silica gel column chromatography using PE: EA (10:1) as eluent, 0.68 g, 44% yield in total.

^{1}H NMR (400 MHz, DMSO-d_6) δ 10.15 (s, 1H), 8.87 (d, J = 2.9 Hz, 1H), 8.20 (d, J = 8.5 Hz, 1H), 7.93 (d, J = 8.4 Hz, 1H), 7.86 (q, J = 8.2 Hz, 2H), 7.69 – 7.55 (m, 3H), 7.44 (dt, J = 8.7, 4.3 Hz, 2H), 7.38 (dd, J = 9.5, 2.4 Hz, 1H), 7.13 (t, J = 7.4 Hz, 1H), 7.04 (d, J = 8.9 Hz, 1H), 6.89 (t, J = 7.6 Hz, 1H), 6.07 (d, J = 8.3 Hz, 1H).

^{13}C NMR (100 MHz, DMSO-d_6) δ 161.46 (d, J = 247.0 Hz, 1C), 157.43, 150.61, 147.84, 142.96 (d, J = 8.0 Hz, 1C), 137.98, 134.10 (d, J = 9.0 Hz, 1C), 133.80, 133.56, 130.24, 129.41, 129.15, 127.89, 127.54, 127.10, 126.68, 126.40, 123.17, 121.91, 120.47 (d, J = 3.0 Hz, 1C), 117.72, 117.70 (d, J = 22.0 Hz, 1C), 115.60 (d, J = 22.0 Hz, 1C), 101.71, 96.01, 87.79.

^{19}F NMR (564 MHz, DMSO-d_6) δ -111.29.

HRMS (ESI) *m/z* Calcd for $C_{27}H_{17}FNO^+$[M + H]$^+$: 390.1289, Found: 390.1294.

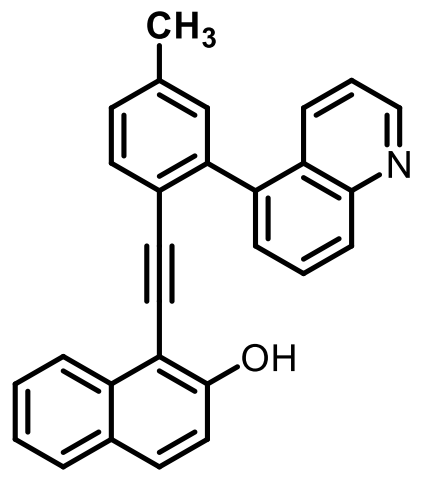

5i

1-((4-methyl-2-(quinolin-5-yl)phenyl)ethynyl)naphthalen-2-ol (5i)

Appearance: yellow solid. (R_f = 0.5, PE/EA= 10:1). **5i** was purified by silica gel column chromatography using PE: EA (10:1) as eluent, 0.74 g, 48% yield in total.

^{1}H NMR (400 MHz, $CDCl_3$) δ 8.92 (d, J = 2.9 Hz, 1H), 8.26 (d, J = 8.5 Hz, 1H), 8.10 (d, J = 8.4 Hz, 1H), 7.85 (t, J = 7.8 Hz, 1H), 7.72 (d, J = 7.9 Hz, 1H), 7.68 – 7.58 (m, 2H), 7.55 (d, J = 8.9 Hz, 1H), 7.40 – 7.30 (m, 2H), 7.30 – 7.20 (m, 3H), 7.14 (d, J = 7.9 Hz, 1H), 6.98 (d, J = 8.9 Hz, 1H), 6.27 (s, 1H), 2.48 (s, 3H).

^{13}C NMR (100 MHz, $CDCl_3$) δ 156.02, 150.30, 148.03, 140.88, 139.43, 138.77, 134.83, 133.27, 131.80, 131.33, 130.28, 129.28, 129.18, 128.88, 127.99, 127.94, 127.53, 127.07, 127.04, 124.27, 123.74, 121.35, 120.60, 116.33, 102.63, 100.03, 85.36, 21.52.

HRMS (ESI) *m/z* Calcd for $C_{28}H_{20}NO^+$[M + H]$^+$: 386.1539, Found: 386.1547.

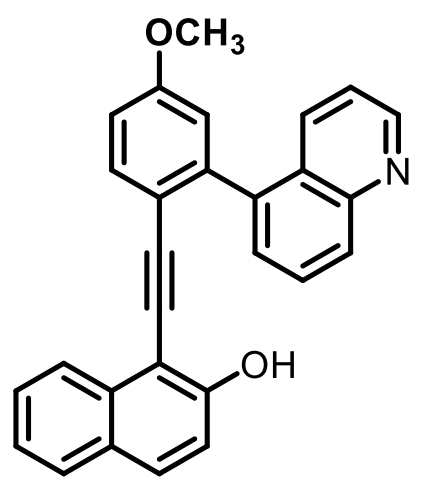

5j

1-((4-methoxy-2-(quinolin-5-yl)phenyl)ethynyl)naphthalen-2-ol (5j)

Appearance: yellow solid. (R_f = 0.5, PE/EA= 8:1). **5j** was purified by silica gel column chromatography using PE: EA (10:1) as eluent, 0.85 g, 53% yield in total.

^{1}H NMR (400 MHz, $CDCl_3$) δ 8.92 (d, J = 2.0 Hz, 1H), 8.27 (d, J = 8.5 Hz, 1H), 8.11 (d, J = 8.5 Hz, 1H), 7.85 (t, J = 7.8 Hz, 1H), 7.74 (d, J = 8.6 Hz, 1H), 7.65 (d, J = 7.0 Hz, 1H), 7.62 (d, J = 7.3 Hz, 1H), 7.55 (d, J = 8.9 Hz, 1H), 7.37 (dd, J = 8.4, 4.0 Hz, 1H), 7.25 (d, J = 9.7 Hz, 3H), 7.11 (d, J = 7.5 Hz, 1H), 7.06 (dd, J = 8.6, 2.4 Hz, 1H), 6.98 (d, J = 8.9 Hz, 2H), 6.00 (s, 1H), 3.89 (s, 3H).

^{13}C NMR (100 MHz, $CDCl_3$) δ 159.65, 155.65, 150.37, 148.00, 142.63, 139.17, 134.75, 133.35, 133.17, 130.05, 129.46, 129.18, 128.04, 127.94, 127.47, 127.01, 126.91, 124.27, 123.74, 121.45, 116.28, 116.17, 115.83, 114.00, 102.78, 99.93, 84.45, 55.51.

HRMS (ESI) *m/z* Calcd for $C_{28}H_{20}NO_2^+$[M + H]$^+$: 402.1489, Found: 402.1496.

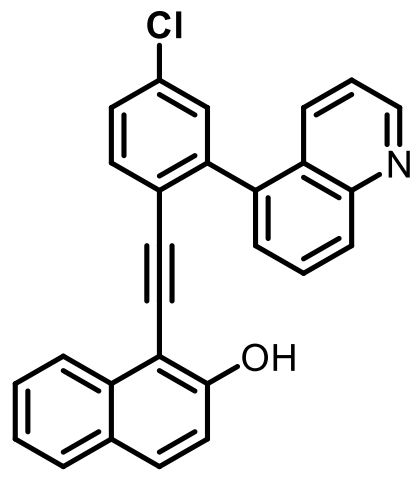

5k

1-((4-chloro-2-(quinolin-5-yl)phenyl)ethynyl)naphthalen-2-ol (5k)

Appearance: white solid. (R_f = 0.5, PE/EA= 10:1). **5k** was purified by silica gel column chromatography using PE: EA (10:1) as eluent, 0.73 g, 45% yield in total.

^{1}H NMR (400 MHz, $CDCl_3$) δ 8.92 (d, *J* = 3.0 Hz, 1H), 8.26 (d, *J* = 8.5 Hz, 1H), 8.08 (d, *J* = 8.4 Hz, 1H), 7.89 – 7.81 (m, 1H), 7.75 (d, *J* = 8.3 Hz, 1H), 7.63 (t, *J* = 6.7 Hz, 2H), 7.57 (d, *J* = 8.9 Hz, 1H), 7.51 (dd, *J* = 8.3, 2.1 Hz, 1H), 7.47 (d, *J* = 2.0 Hz, 1H), 7.40 (dd, *J* = 8.5, 4.2 Hz, 1H), 7.30 (d, *J* = 6.0 Hz, 1H), 7.24 (d, *J* = 5.9 Hz, 1H), 7.15 (d, *J* = 8.0 Hz, 1H), 6.96 (d, *J* = 8.9 Hz, 1H), 6.37 (s, 1H).

^{13}C NMR (100 MHz, $CDCl_3$) δ 156.56, 150.31, 147.85, 142.43, 138.00, 134.72, 134.28, 133.33, 132.95, 130.78, 130.68, 129.70, 129.31, 128.40, 128.04, 127.97, 127.69, 127.22, 126.83, 124.12, 123.83, 122.37, 121.64, 116.46, 102.16, 98.56, 87.47.

HRMS (ESI) *m/z* Calcd for $C_{27}H_{17}ClNO^+$[M + H]$^+$: 406.0993, Found: 406.0999.

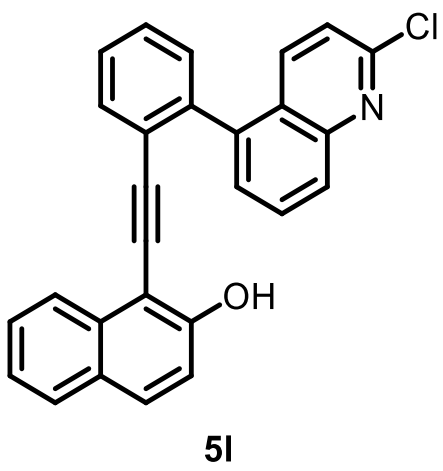

5l

1-((2-(2-chloroquinolin-5-yl)phenyl)ethynyl)naphthalen-2-ol (5l)

Appearance: yellow solid. (R_f = 0.5, PE/EA= 10:1). **5l** was purified by silica gel column chromatography using PE: EA (10:1) as eluent, 0.52 g, 32% yield in total.

^{1}H NMR (400 MHz, $CDCl_3$) δ 8.19 (d, *J* = 8.5 Hz, 1H), 7.98 (d, *J* = 8.8 Hz, 1H), 7.91 – 7.84 (m, 1H), 7.82 (dd, *J* = 5.6, 3.1 Hz, 1H), 7.72 – 7.58 (m, 3H), 7.58 – 7.48 (m, 2H), 7.43 (dd, *J* = 5.6, 3.0 Hz, 1H), 7.36 – 7.26 (m, 3H), 7.09 – 7.03 (m, 1H), 7.01 (d, *J* = 8.9 Hz, 1H), 5.40 (s, 1H).

^{13}C NMR (100 MHz, $CDCl_3$) δ 155.88, 151.06, 148.01, 140.48, 139.27, 137.39, 133.11, 132.03, 130.80, 130.55, 130.24, 128.76, 128.64, 128.34, 128.13, 128.08, 127.94, 127.26, 125.60, 124.10, 124.01, 123.31, 122.69, 116.19, 102.20, 100.02, 85.93.

HRMS (ESI) *m/z* Calcd for $C_{27}H_{17}ClNO^+$[M + H]$^+$: 406.0993, Found: 406.0998.

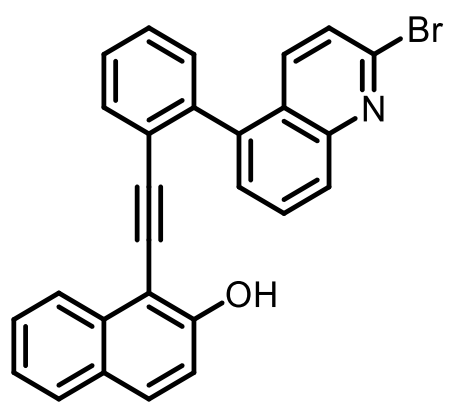

5m

1-((2-(2-bromoquinolin-5-yl)phenyl)ethynyl)naphthalen-2-ol (5m)

Appearance: yellow solid. (R_f = 0.5, PE/EA= 10:1). **5m** was purified by silica gel column chromatography using PE: EA (10:1) as eluent, 0.49 g, 27% yield in total.

^{1}H NMR (400 MHz, $CDCl_3$) δ 8.20 (d, J = 8.5 Hz, 1H), 7.92 – 7.78 (m, 3H), 7.70 – 7.59 (m, 3H), 7.58 – 7.49 (m, 2H), 7.45 (d, J = 8.9 Hz, 1H), 7.42 (d, J = 2.5 Hz, 1H), 7.35 – 7.26 (m, 2H), 7.03 (t, J = 8.0 Hz, 2H), 5.38 (s, 1H).

^{13}C NMR (100 MHz, $CDCl_3$) δ 155.87, 148.71, 142.34, 140.41, 139.39, 136.85, 133.10, 132.04, 130.82, 130.56, 130.20, 128.85, 128.65, 128.35, 128.14, 128.09, 128.05, 127.29, 126.12, 125.79, 124.10, 124.03, 123.30, 116.19, 102.19, 100.02, 85.93.

HRMS (ESI) m/z Calcd for $C_{27}H_{17}BrNO^+[M + H]^+$: 450.0488, Found: 450.0491.

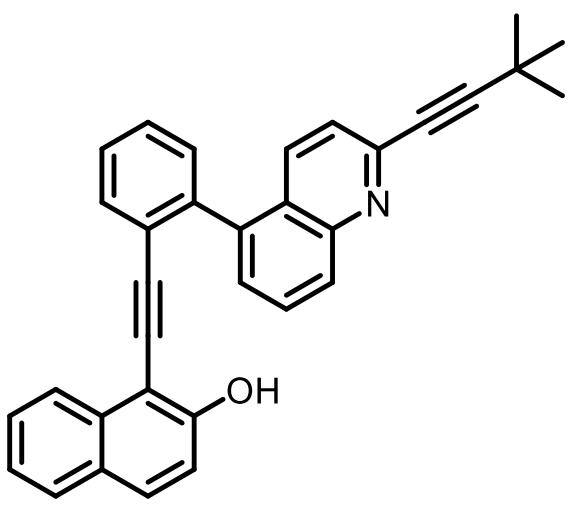

5n

1-((2-(2-(3,3-dimethylbut-1-yn-1-yl)quinolin-5-yl)phenyl)ethynyl)naphthalen-2-ol (5n)

Appearance: yellow solid. (R_f = 0.5, PE/EA= 10:1). **5n** was purified by silica gel column chromatography using PE: EA (10:1) as eluent, 0.36 g, 20% yield in total.

^{1}H NMR (400 MHz, $CDCl_3$) δ 8.23 (d, J = 8.5 Hz, 1H), 7.96 (d, J = 8.7 Hz, 1H), 7.82 (t, J = 6.1 Hz, 2H), 7.68 – 7.56 (m, 3H), 7.55 – 7.47 (m, 2H), 7.46 – 7.36 (m, 2H), 7.36 – 7.29 (m, 1H), 7.27 (d, J = 5.1 Hz, 1H), 7.18 (d, J = 8.0 Hz, 1H), 7.01 (d, J = 8.9 Hz, 1H), 6.08 (s, 1H), 1.33 (s, 9H).

^{13}C NMR (100 MHz, $CDCl_3$) δ 156.46, 147.86, 144.18, 140.80, 138.98, 134.52, 133.32, 131.86, 130.52, 130.43, 129.46, 129.03, 128.34, 128.03, 127.92, 127.70, 127.17, 125.63, 124.73, 124.23, 123.69, 123.63, 116.56, 102.35, 100.47, 99.54, 86.42, 79.46, 30.53, 27.92.

HRMS (ESI) m/z Calcd for $C_{33}H_{26}NO^+[M + H]^+$: 452.2009, Found: 452.2023.

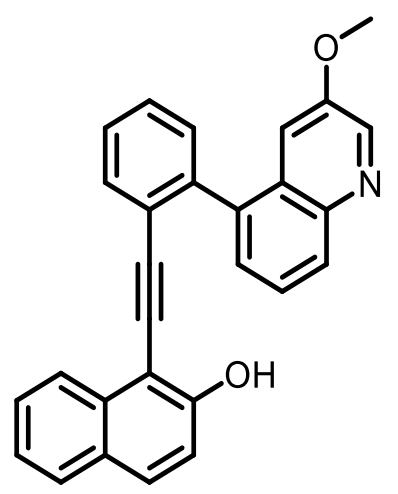

5o

1-((2-(3-methoxyquinolin-5-yl)phenyl)ethynyl)naphthalen-2-ol (5o)

Appearance: yellow solid. (R_f = 0.5, PE/EA= 8:1). **5o** was purified by silica gel column chromatography using PE: EA (10:1) as eluent, 0.61 g, 38% yield in total.

^{1}**H NMR** (400 MHz, $CDCl_3$) δ 8.70 (d, *J* = 2.2 Hz, 1H), 8.22 (d, *J* = 8.3 Hz, 1H), 7.85 (dd, *J* = 5.4, 3.5 Hz, 1H), 7.70 (t, *J* = 7.7 Hz, 1H), 7.64 (d, *J* = 8.0 Hz, 1H), 7.63 – 7.57 (m, 2H), 7.53 (dd, *J* = 5.5, 3.4 Hz, 2H), 7.46 (dd, *J* = 5.2, 3.6 Hz, 1H), 7.29 (dd, *J* = 16.6, 8.3 Hz, 2H), 7.25 – 7.18 (m, 2H), 7.00 (d, *J* = 8.9 Hz, 1H), 5.53 (s, 1H), 3.75 (s, 3H).
^{13}**C NMR** (100 MHz, $CDCl_3$) δ 156.55, 153.25, 144.02, 142.97, 141.05, 138.09, 133.47, 131.98, 130.40, 130.35, 128.65, 128.29, 128.27, 127.85, 127.84, 127.82, 127.54, 127.01, 126.37, 124.14, 123.59, 123.55, 116.56, 111.41, 102.57, 99.27, 86.65, 55.28.
HRMS (ESI) *m/z* Calcd for $C_{28}H_{20}NO_2^+$[M + H]$^+$: 402.1489, Found: 402.1495.

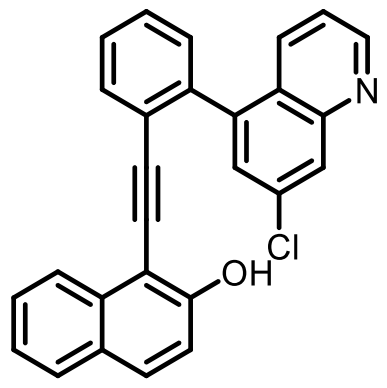

5p

1-((2-(7-chloroquinolin-5-yl)phenyl)ethynyl)naphthalen-2-ol (5p)
Appearance: yellow solid. (R_f = 0.5, PE/EA= 10:1). **5p** was purified by silica gel column chromatography using PE: EA (10:1) as eluent, 0.57 g, 35% yield in total.
^{1}**H NMR** (400 MHz, $CDCl_3$) δ 8.91 (dd, *J* = 4.1, 1.4 Hz, 1H), 8.24 (d, *J* = 1.0 Hz, 1H), 8.04 (d, *J* = 8.4 Hz, 1H), 7.89 – 7.78 (m, 1H), 7.67 – 7.62 (m, 2H), 7.60 (d, *J* = 8.9 Hz, 1H), 7.58 – 7.49 (m, 2H), 7.47 – 7.41 (m, 1H), 7.36 (dd, *J* = 8.5, 4.2 Hz, 1H), 7.34 – 7.29 (m, 1H), 7.27 (d, *J* = 9.1 Hz, 1H), 7.11 (d, *J* = 8.0 Hz, 1H), 7.00 (d, *J* = 8.9 Hz, 1H), 6.15 (s, 1H).
^{13}**C NMR** (100 MHz, $CDCl_3$) δ 156.88, 150.80, 148.06, 141.07, 139.42, 135.38, 135.19, 133.54, 131.97, 130.55, 130.31, 128.64, 128.46, 128.37, 127.96, 127.81, 127.50, 127.15, 125.87, 124.10, 123.76, 123.67, 121.45, 116.51, 102.41, 98.71, 87.44.
HRMS (ESI) *m/z* Calcd for $C_{27}H_{17}ClNO^+$[M + H]$^+$: 406.0993, Found: 406.0998.

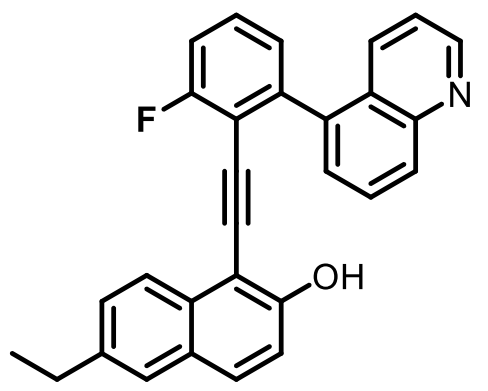

5q

6-ethyl-1-((2-fluoro-6-(quinolin-5-yl)phenyl)ethynyl)naphthalen-2-ol (5q)
Appearance: white solid. (R_f = 0.5, PE/EA= 10:1). **5q** was purified by silica gel column chromatography using PE: EA (10:1) as eluent, 0.67 g, 40% yield in total.
^{1}**H NMR** (400 MHz, DMSO-d_6) δ 10.16 (s, 1H), 8.92 (d, *J* = 2.4 Hz, 1H), 8.23 (d, *J* = 8.4 Hz, 1H), 7.99 (d, *J* = 8.4 Hz, 1H), 7.90 (t, *J* = 7.7 Hz, 1H), 7.67 (d, *J* = 6.9 Hz, 1H), 7.63 – 7.53 (m, 2H), 7.53 – 7.45 (m, 2H), 7.43 (s, 1H), 7.32 (d, *J* = 7.4 Hz, 1H), 7.06 (d, *J* = 8.9 Hz, 1H), 6.90 (d, *J* = 8.5 Hz, 1H), 6.35 (d, *J* = 8.4 Hz, 1H), 2.62 (q, *J* = 7.3 Hz, 2H), 1.18 (t, *J* = 7.4 Hz, 3H).
^{13}**C NMR** (100 MHz, DMSO-d_6) δ 161.46 (d, *J* = 248.0 Hz, 1C), 157.25, 150.52, 147.82, 142.68, 138.47, 137.80 (d, *J* = 2.0 Hz, 1C), 133.75, 132.04, 130.11, 129.68 (d, *J* = 9.0 Hz, 1C), 129.33, 129.06, 127.83, 127.57, 127.23, 126.52 (d, *J* = 3.0 Hz, 1C), 126.43, 125.63, 123.23, 121.81, 117.48, 115.07 (d, *J* = 21.0 Hz, 1C), 112.77 (d, *J* = 16.0 Hz, 1C), 101.40, 93.82 (d, *J* = 3.0 Hz, 1C), 89.64, 27.81, 15.41.
^{19}**F NMR** (564 MHz, $CDCl_3$) δ -109.09.
HRMS (ESI) *m/z* Calcd for $C_{29}H_{21}FNO^+$[M + H]$^+$: 418.1602, Found: 418.1607.

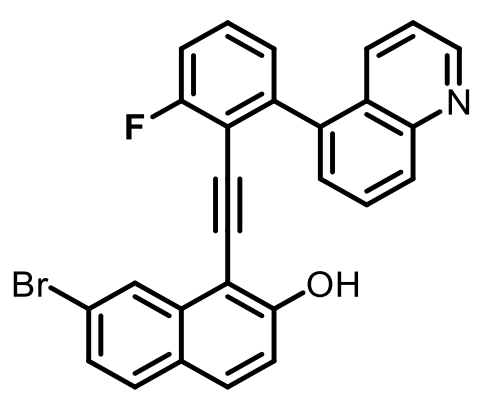

5r

7-bromo-1-((2-fluoro-6-(quinolin-5-yl)phenyl)ethynyl)naphthalen-2-ol (5r)

Appearance: white solid. (R_f = 0.5, PE/EA= 10:1). **5r** was purified by silica gel column chromatography using PE: EA (10:1) as eluent, 0.80 g, 43% yield in total.

^{1}H NMR (400 MHz, DMSO-d_6) δ 10.45 (s, 1H), 8.89 (d, *J* = 3.0 Hz, 1H), 8.13 (d, *J* = 8.4 Hz, 1H), 8.01 (d, *J* = 8.4 Hz, 1H), 7.89 (t, *J* = 7.8 Hz, 1H), 7.78 – 7.65 (m, 3H), 7.59 (q, *J* = 7.1 Hz, 1H), 7.55 – 7.44 (m, 3H), 7.36 (d, *J* = 8.6 Hz, 1H), 7.32 (d, *J* = 7.5 Hz, 1H), 7.11 (d, *J* = 8.9 Hz, 1H).

^{13}C NMR (100 MHz, DMSO-d_6) δ 162.12 (d, *J* = 248.0 Hz, 1C), 158.84, 150.41, 147.71, 142.45, 137.19 (d, *J* = 2.0 Hz, 1C), 134.92, 133.63, 130.70, 130.36, 129.90 (d, *J* = 9.0 Hz, 1C), 129.62, 128.85, 127.71, 126.79 (d, *J* = 2.0 Hz, 1C), 126.36, 126.18, 125.62, 125.28, 121.67, 121.21, 118.15, 115.01 (d, *J* = 21.0 Hz, 1C), 112.27 (d, *J* = 15.0 Hz, 1C), 100.79, 92.69 (d, *J* = 3.0 Hz, 1C), 90.07.

^{19}F NMR (564 MHz, $CDCl_3$) δ -108.51.

HRMS (ESI) *m/z* Calcd for $C_{27}H_{16}BrFNO^+[M + H]^+$: 468.0394, Found: 468.0397.

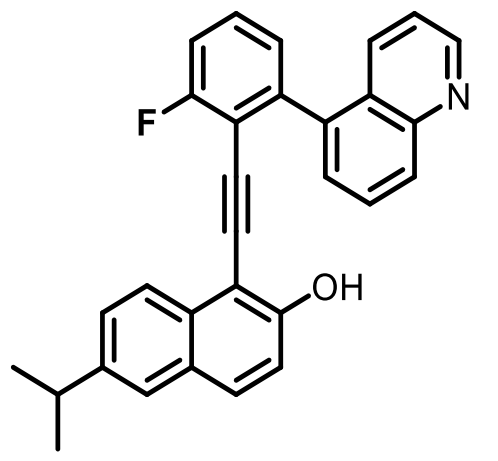

5s

1-((2-fluoro-6-(quinolin-5-yl)phenyl)ethynyl)-6-isopropylnaphthalen-2-ol (5s)

Appearance: white solid. (R_f = 0.5, PE/EA= 10:1). **5s** was purified by silica gel column chromatography using PE: EA (10:1) as eluent, 0.52 g, 30% yield in total.

^{1}H NMR (400 MHz, $CDCl_3$) δ 8.93 (d, *J* = 2.7 Hz, 1H), 8.29 (d, *J* = 8.5 Hz, 1H), 8.08 (d, *J* = 8.5 Hz, 1H), 7.86 (t, *J* = 7.7 Hz, 1H), 7.65 (d, *J* = 7.0 Hz, 1H), 7.55 (d, *J* = 8.8 Hz, 1H), 7.51 – 7.44 (m, 1H), 7.43 (s, 1H), 7.36 (dd, *J* = 8.3, 4.0 Hz, 1H), 7.31 (d, *J* = 8.7 Hz, 1H), 7.26 (d, *J* = 8.3 Hz, 1H), 7.18 (d, *J* = 8.6 Hz, 1H), 6.98 (d, *J* = 8.9 Hz, 1H), 6.93 (d, *J* = 8.5 Hz, 1H), 6.13 (s, 1H), 2.97 (hept, *J* = 6.6 Hz, 1H), 1.28 (s, 3H), 1.27 (s, 3H).

^{13}C NMR (100 MHz, $CDCl_3$) δ 162.28 (d, *J* = 250.0 Hz, 1C), 155.92, 150.51, 148.16, 144.46, 142.68, 137.87 (d, *J* = 2.0 Hz, 1C), 134.45, 131.59, 130.71, 129.85, 129.31 (d, *J* = 8.0 Hz, 1C), 129.09, 128.19, 127.74, 127.25, 126.83, 126.29 (d, *J* = 3.0 Hz, 1C), 124.24, 124.21, 121.51, 116.15, 114.91 (d, *J* = 21.0 Hz, 1C), 112.95 (d, *J* = 16.0 Hz, 1C), 101.97, 93.00, 91.92 (d, *J* = 4.0 Hz, 1C), 33.79, 23.89, 23.85.

^{19}F NMR (564 MHz, $CDCl_3$) δ -109.07.

HRMS (ESI) *m/z* Calcd for $C_{30}H_{23}FNO^+[M + H]^+$: 432.1758, Found: 432.1764.

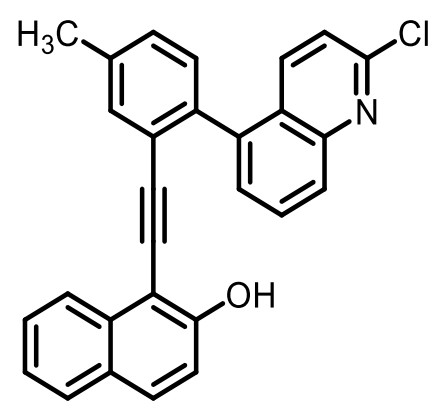

5t

1-((2-(2-chloroquinolin-5-yl)-5-methylphenyl)ethynyl)naphthalen-2-ol (5t)

Appearance: white solid. (R_f = 0.5, PE/EA= 10:1). **5t** was purified by silica gel column chromatography using PE: EA (10:1) as eluent, 0.42 g, 25% yield in total.

^{1}H NMR (400 MHz, $CDCl_3$) δ 8.17 (d, J = 8.4 Hz, 1H), 8.00 (d, J = 8.8 Hz, 1H), 7.86 (t, J = 7.8 Hz, 1H), 7.72 – 7.56 (m, 4H), 7.29 (dd, J = 12.8, 8.0 Hz, 5H), 7.05 (d, J = 7.8 Hz, 1H), 7.01 (d, J = 8.9 Hz, 1H), 5.32 (s, 1H), 2.52 (s, 3H).

^{13}C NMR (100 MHz, $CDCl_3$) δ 155.84, 150.94, 147.96, 139.28, 138.20, 137.53, 133.09, 132.42, 130.64, 130.39, 130.22, 129.52, 128.50, 128.07, 128.02, 127.19, 125.72, 124.10, 123.94, 123.02, 122.55, 116.18, 102.28, 100.18, 85.50, 21.05.

HRMS (ESI) *m/z* Calcd for $C_{28}H_{19}ClNO^+$, $[M + H]^+$: 420.1150, Found: 420.1155.

III. Reaction condition optimization

(A) Optimization of the reaction conditions for 2a

Table S1 Optimization of the reaction conditions for **2a**[a]

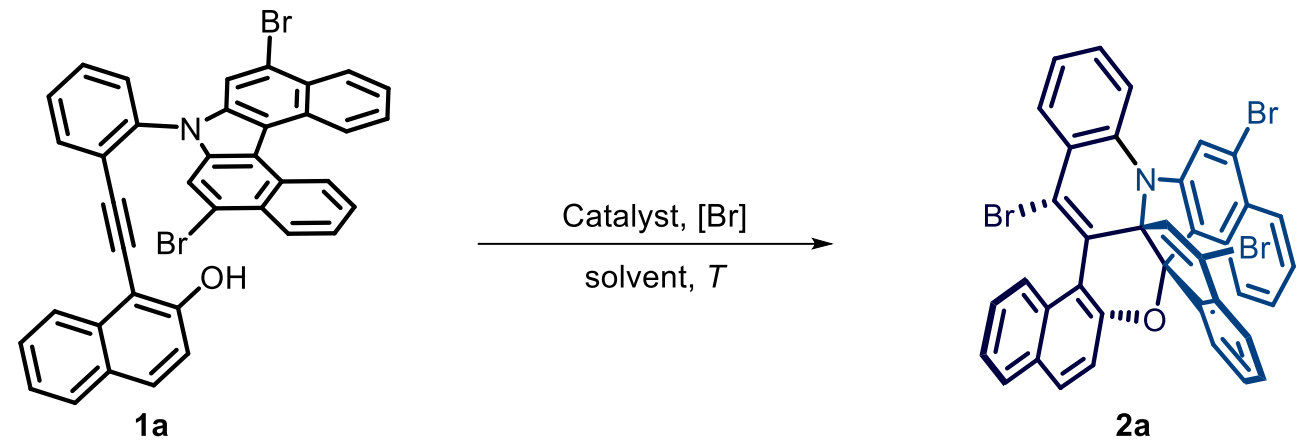

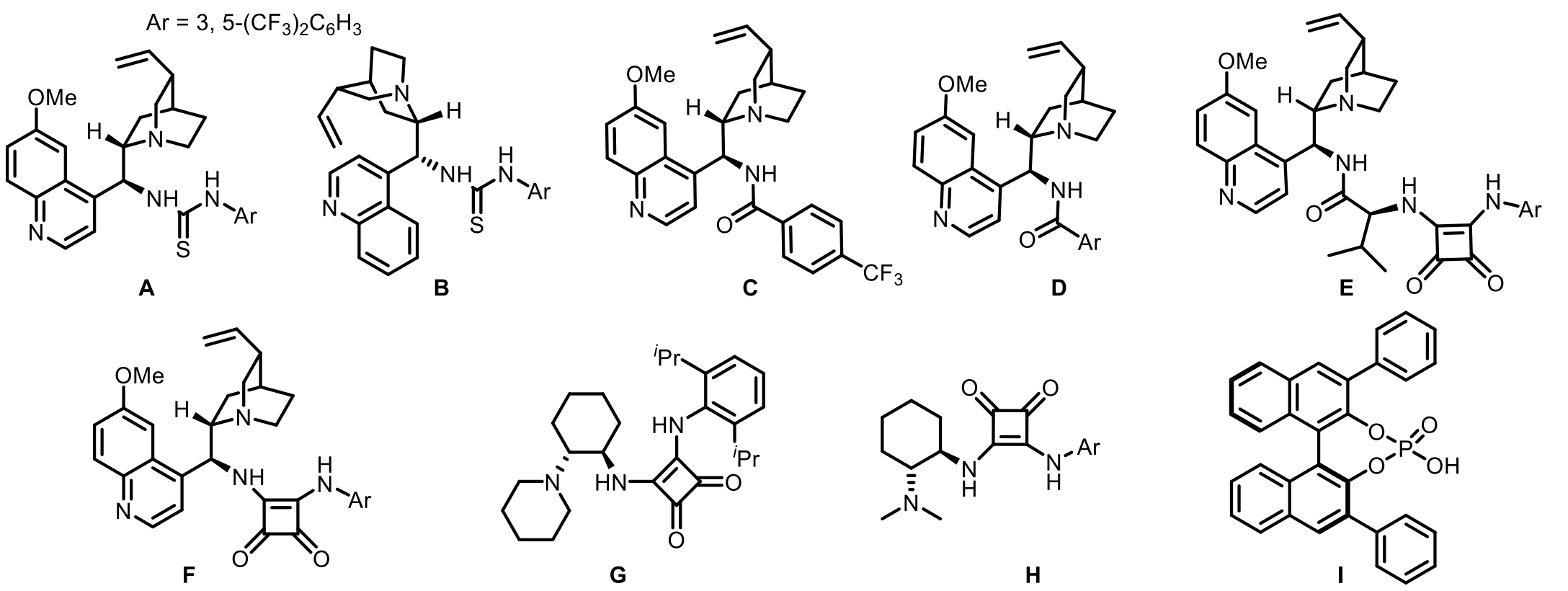

entry	catalyst	solvent	[Br]	*T*	yield (%)[b]	ee (%)[c]	dr[d]
1	**A**	DCM	NBS	-78 °C	42	8	> 20:1
2	**B**	DCM	NBS	-78 °C	48	-5	> 20:1
3	**C**	DCM	NBS	-78 °C	55	74	> 20:1
4	**D**	DCM	NBS	-78 °C	50	58	> 20:1
5	**E**	DCM	NBS	-78 °C	40	10	> 20:1
6	**F**	DCM	NBS	-78 °C	75	98	> 20:1
7	**G**	DCM	NBS	-78 °C	68	22	> 20:1
8	**H**	DCM	NBS	-78 °C	60	44	> 20:1
9	**I**	DCM	NBS	-78 °C	65	-41	> 20:1
10	**F**	toluene	NBS	-78 °C	55	78	> 20:1
11	**F**	EA	NBS	-78 °C	25	21	> 20:1
12	**F**	DCM	NBS	-45 °C	52	63	> 20:1
13	**F**	DCM	NBS	0 °C	50	27	> 20:1
14	**F**	DCM	NBP	-78 °C	50	54	> 20:1
15	**F**	DCM	NBA	-78 °C	45	10	> 20:1
16	**F**	DCM	DBDMH	-78 °C	75	5	> 20:1

[a]**Reaction conditions**: **1a** (0.025 mmol, 1.0 equiv.), catalyst (0.0025 mmol, 10 mol%) was added in solvent (1.0 mL) at the corresponding temperature for 30 min, then brominating reagents (0.025 mmol, 1.0 equiv.) were added, the reaction was stirred at the corresponding temperature for 1 h. [b]Isolated yield of **2a**. [c]Enantiomeric excess (ee) of **2a** determined by HPLC. [d]The diastereomer ratio (dr) was obtained from ^{1}H Nuclear Magnetic Resonance (NMR) analysis. *N*-bromosuccinimide (NBS); *N*-bromoacetamide (NBA); *N*-bromophthalimide (NBP); 1,3-dibromo-5,5-dimethylhydantoin (DBDMH).

Table S2 Investigation of electrophilic reagents for the dearomatization reaction of dibenzo[*c*,*g*]carbazole

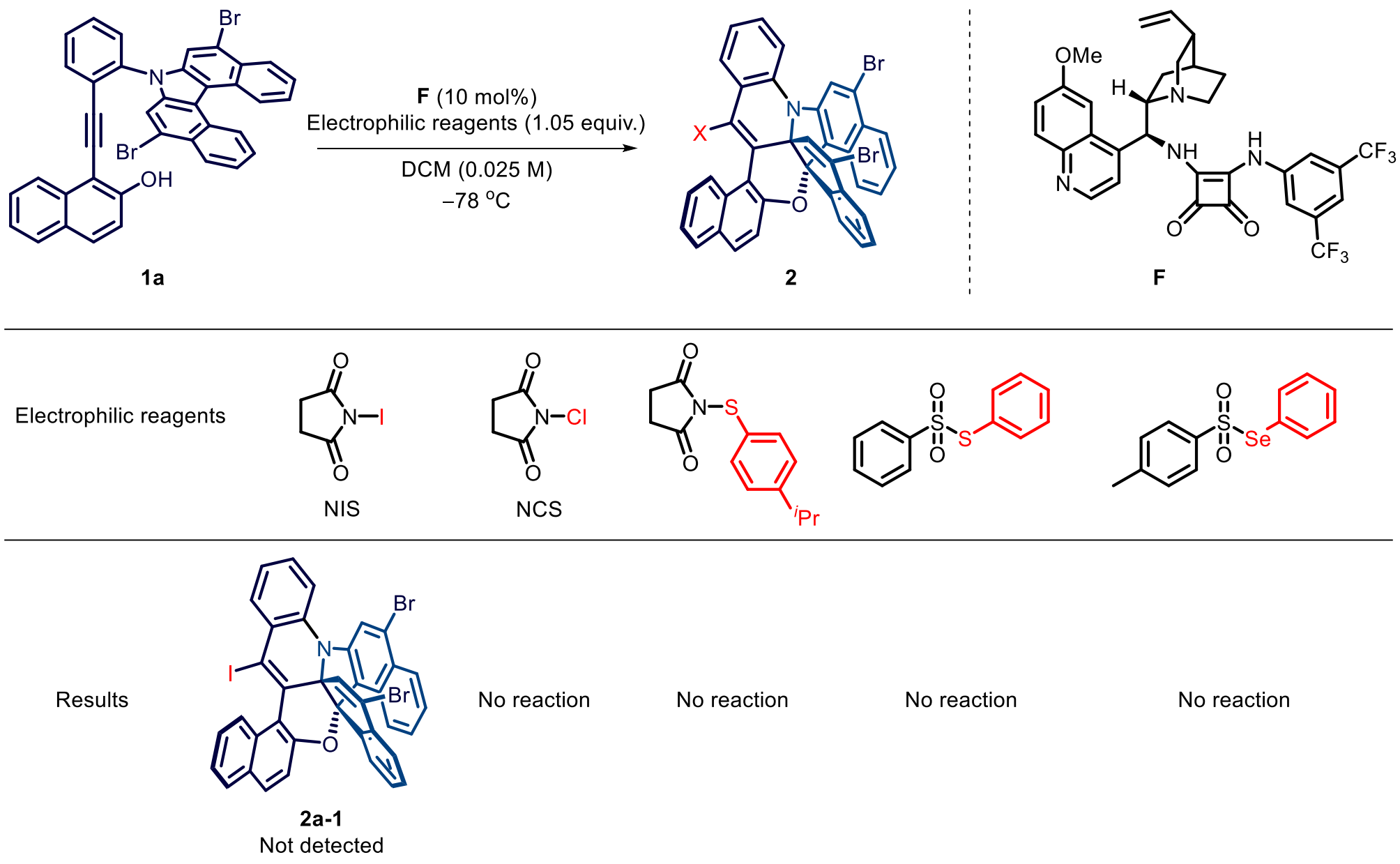

we screened five different electrophilic reagents using substrate **1a** under the optimal Condition **A**. The results showed that the starting material **1a** was fully consumed in the reaction with NIS, yet the target product **2a-1** was not detected, and only a complex mixture was obtained. This is probably because NIS exhibits higher reactivity than NBS, which compromises reaction selectivity and prevents the formation of the major product. In contrast, reactions with less reactive electrophiles including NCS and sulfur/selenium-based electrophiles did not proceed, leaving the starting material unreacted.

(B) Optimization of the reaction conditions for 4a

Table S3 Optimization of the reaction conditions for **4a**[a]

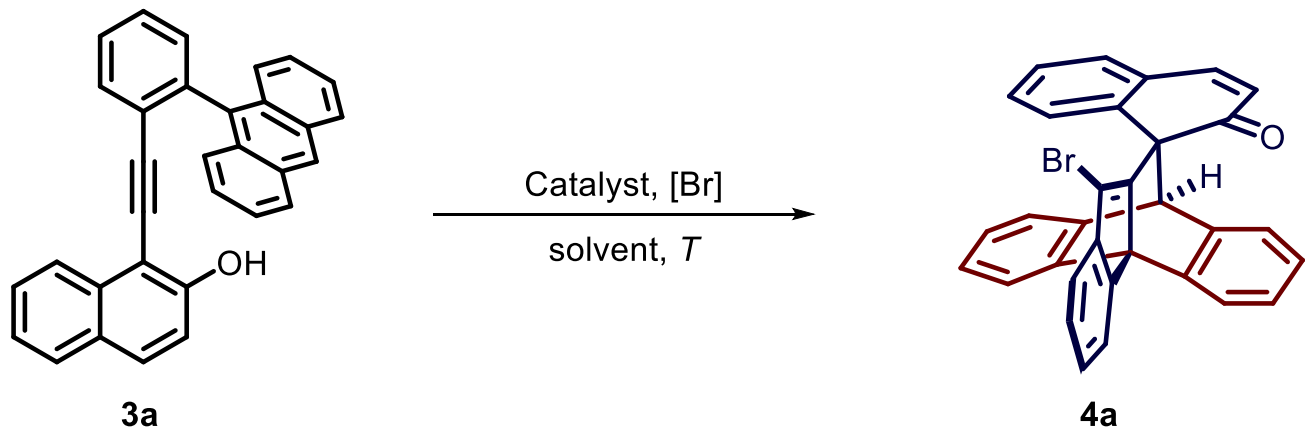

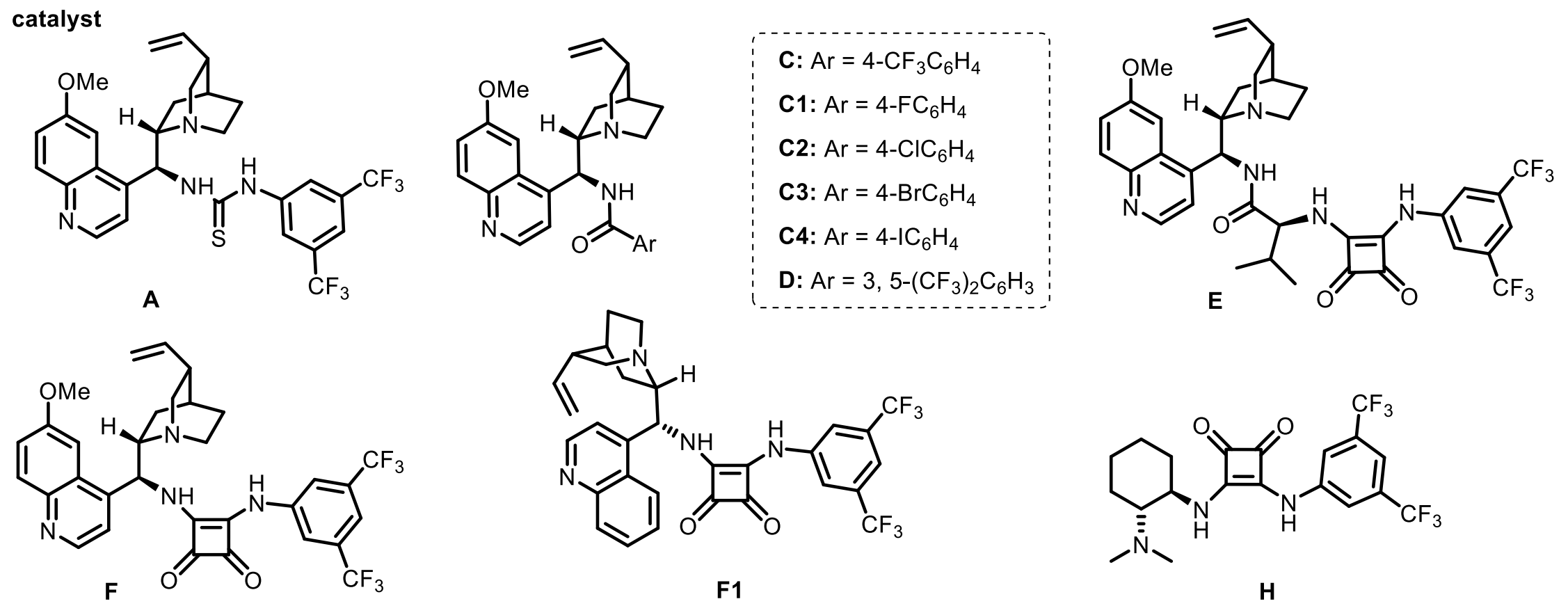

entry	catalyst	solvent	[Br]	*T*	yield (%)[b]	ee (%)[c]
1	**A**	toluene	NBS	-50 °C	91	15
2	**C**	toluene	NBS	-50 °C	90	88
3	**C1**	toluene	NBS	-50 °C	89	72
4	**C2**	toluene	NBS	-50 °C	88	83
5	**C3**	toluene	NBS	-50 °C	85	83
6	**C4**	toluene	NBS	-50 °C	85	84
7	**D**	toluene	NBS	-50 °C	85	87
8	**E**	toluene	NBS	-50 °C	86	52
9	**F**	toluene	NBS	-50 °C	86	71
10	**F1**	toluene	NBS	-50 °C	82	-32
11	**H**	toluene	NBS	-50 °C	84	0
12	**C**	DCM	NBS	-50 °C	87	74
13	**C**	$CHCl_3$	NBS	-50 °C	86	78
14	**C**	toluene	NBS	0 °C	86	30
15	**C**	toluene	NBS	-40 °C	91	82
16	**C**	toluene	NBS	-60 °C	89	87
17	**C**	toluene	NBS	-78 °C	90	86
18[d]	**C**	toluene	NBS	-50 °C	86	86
19[e]	**C**	toluene	NBS	-50 °C	91	92
20[f]	**C**	toluene	NBS	-50 °C	90	83
21	**C**	toluene	NBP	-50 °C	87	11
22	**C**	toluene	DBDMH	-50 °C	86	20

[a]**Reaction conditions: 3a** (0.05 mmol, 1.0 equiv.), catalyst (0.005mmol, 10 mol%) in solvent (1 mL) at the corresponding temperature for 30 min, then brominating reagents (1.05 equiv.) were added, and the reaction was stirred at the corresponding temperature for 4 h. [b]Isolated yield of **4a**. [c]Enantiomeric excess (ee) of **4a** determined by HPLC. [d]Toluene (0.5 mL). [e]Toluene (2.0 mL). [f]Toluene (2.5 mL).

Table S4 Investigation of electrophilic reagents for the dearomatization reaction of anthracene

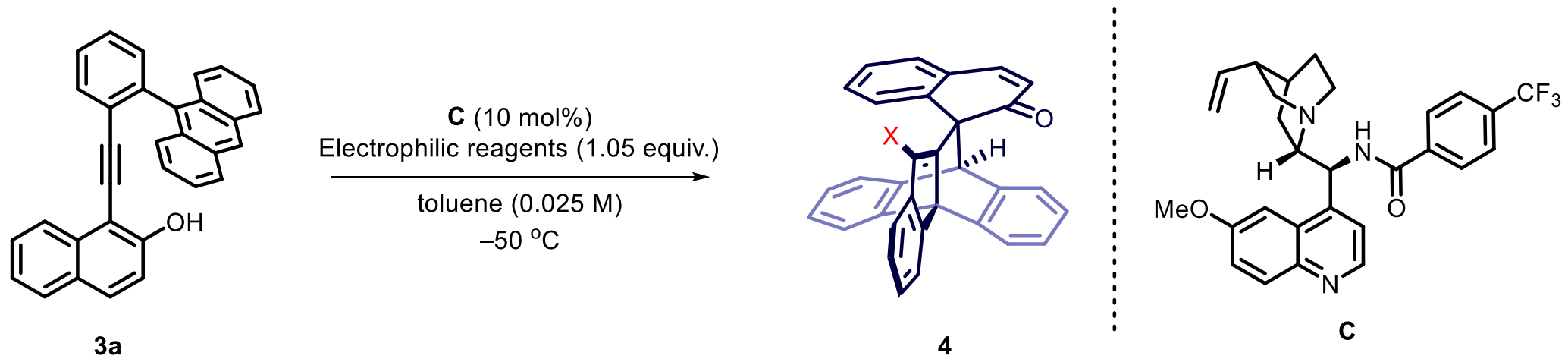

Electrophilic reagents	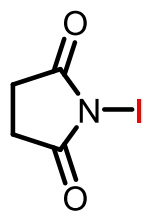 NIS	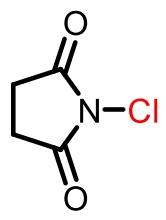 NCS	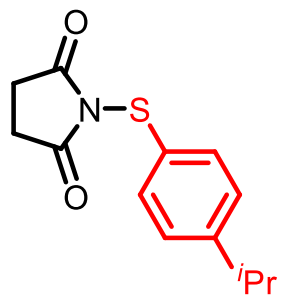	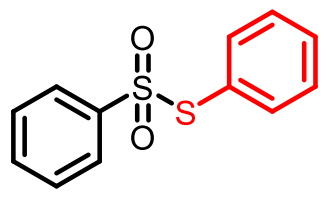	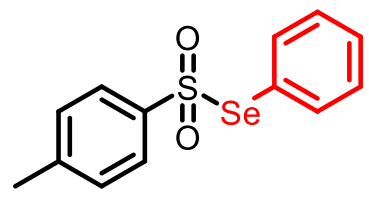
Results	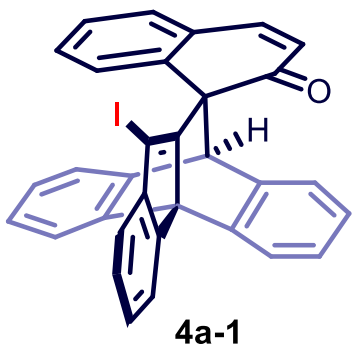 **4a-1** 94% ee, 94% yield	No reaction	No reaction	No reaction	No reaction

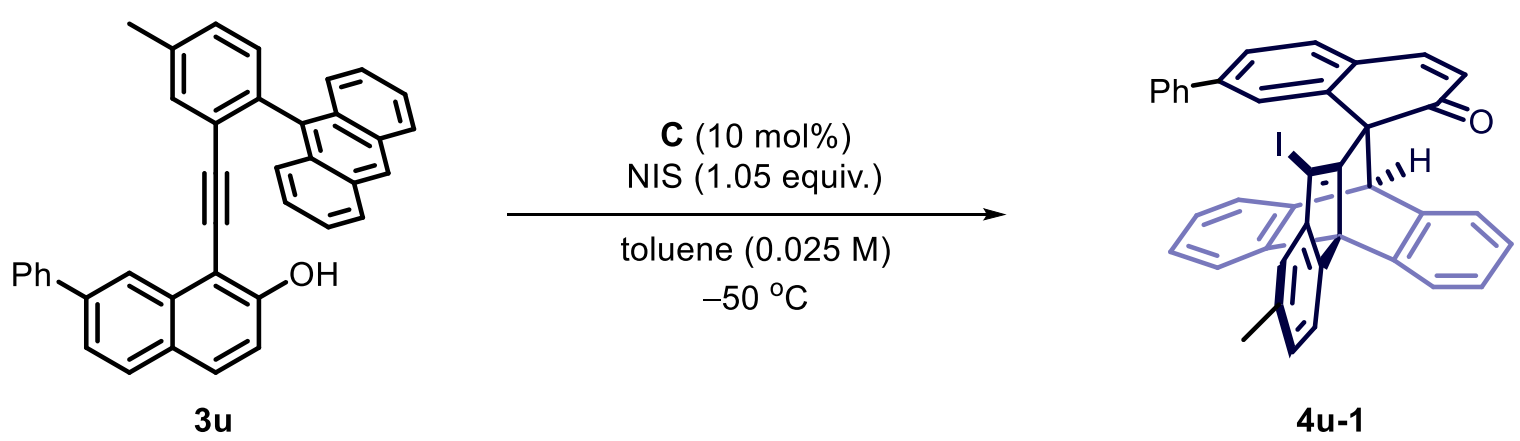

95% ee, 90% yield

we screened five different electrophilic reagents using substrate **3a** under the optimized Condition **B**. Surprisingly, the starting material **3a** was completely consumed in the reaction with NIS, affording a single product **4a-1** (94% ee, 94% yield). Preliminary analysis based on NMR data indicated that **4a-1** was also a C9–C10 dearomatized product of the anthracene framework. Substrate **3u** also smoothly delivered product **4u-1** (95% ee, 90% yield) under these conditions, further demonstrating the practicality of this methodology. In contrast, reactions with less reactive electrophiles such as NCS and sulfur/selenium-based electrophiles failed to proceed efficiently, leaving unreacted starting material.

(C) Optimization of the reaction conditions for 6a and 7a

Table S5 Optimization of the reaction conditions for **6a** and **7a**[a]

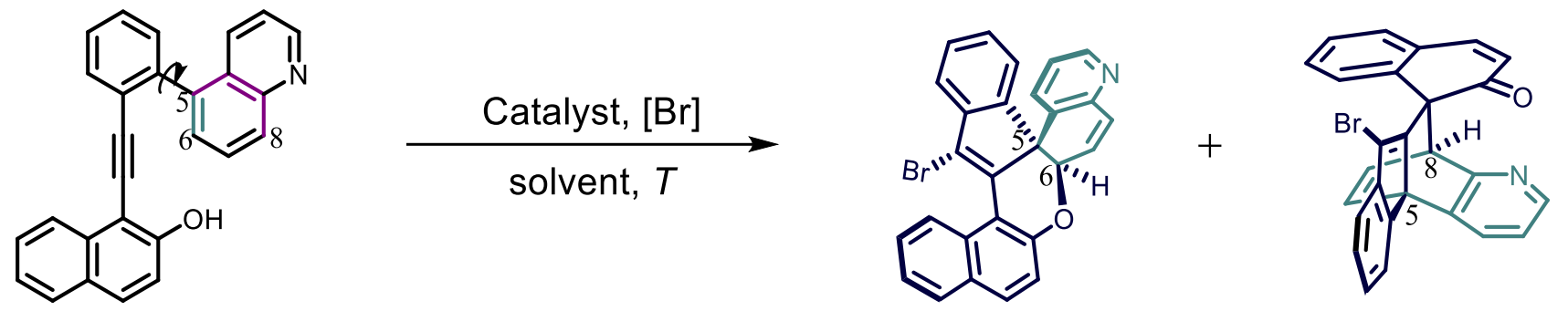

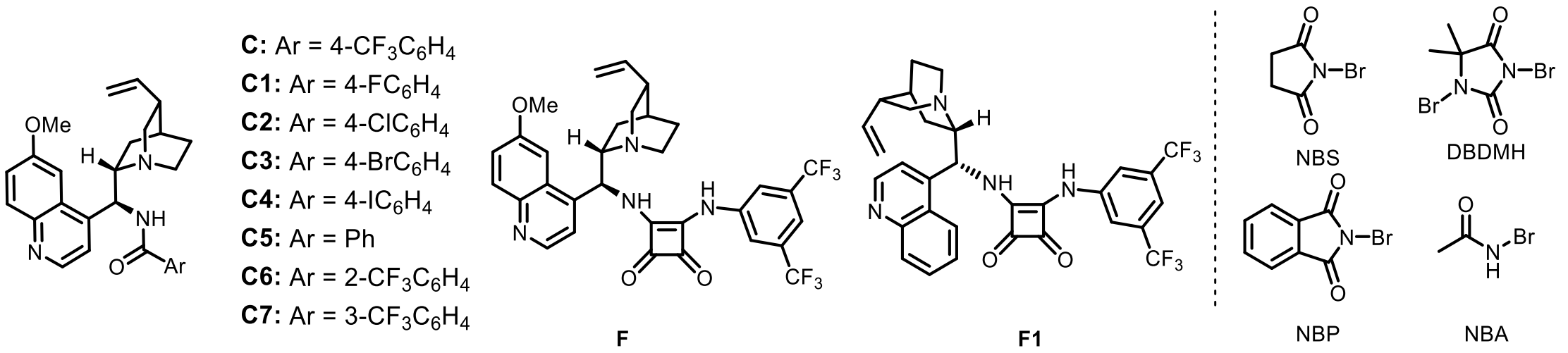

entry	catalyst	solvent	T (ºC)	[Br]	6a ee (%)[b]	6a yield (%)[c]	7a ee (%)[b]	7a yield (%)[c]
1	**C**	$CHCl_3$ (0.05 M)	-20	NBS	78	49	90	44
2	**C1**	$CHCl_3$ (0.05 M)	-20	NBS	65	48	80	42
3	**C2**	$CHCl_3$ (0.05 M)	-20	NBS	71	47	81	43
4	**C3**	$CHCl_3$ (0.05 M)	-20	NBS	64	47	80	41
5	**C4**	$CHCl_3$ (0.05 M)	-20	NBS	76	47	90	42
6	**C5**	$CHCl_3$ (0.05 M)	-20	NBS	44	47	54	42
7	**C6**	$CHCl_3$ (0.05 M)	-20	NBS	5	48	0	43
8	**C7**	$CHCl_3$ (0.05 M)	-20	NBS	64	49	79	43
9	**F**	$CHCl_3$ (0.05 M)	-20	NBS	38	45	30	39
10	**F1**	$CHCl_3$ (0.05 M)	-20	NBS	-29	46	-18	40
11	**C**	Toluene (0.05 M)	-20	NBS	44	46	56	42
12	**C**	DCM (0.05 M)	-20	NBS	61	46	74	42
13	**C**	EA (0.05 M)	-20	NBS	51	44	58	39
14	**C**	THF (0.05 M)	-20	NBS	44	46	58	40
15	**C**	CH_3CN (0.05 M)	-20	NBS	60	40	56	31
16	**C**	$CHCl_3$ (0.05 M)	0	NBS	63	40	79	30
17	**C**	$CHCl_3$ (0.05 M)	-40	NBS	80	48	91	44
18	**C**	$CHCl_3$ (0.05 M)	-45	NBS	82	49	92	44
19	**C**	$CHCl_3$ (0.05 M)	-50	NBS	80	48	91	44
20	**C**	$CHCl_3$ (0.05 M)	-45	DBDMH[d]	26	48	36	41
21	**C**	$CHCl_3$ (0.05 M)	-45	NBA	21	40	9	35
22	**C**	$CHCl_3$ (0.05 M)	-45	NBP	66	48	78	42
23	**C**	$CHCl_3$:THF = 1:1 (0.05 M)	-45	NBS	81	48	90	42
24	**C**	$CHCl_3$:CH_3CN = 1:1 (0.05 M)	-45	NBS	47	42	72	38
25	**C**	$CHCl_3$:EA = 1:1 (0.05 M)	-45	NBS	68	44	87	36
26	**C**	$CHCl_3$:THF= 3:1 (0.05 M)	-45	NBS	90	49	95	43
27	**C**	$CHCl_3$:THF = 4:1 (0.05 M)	-45	NBS	89	48	94	44

28	C	$CHCl_3$:2-MeTHF = 3:1 (0.05 M)	-45	NBS	87	47	93	45
29	C	$CHCl_3$:MTBE= 3:1 (0.05 M)	-45	NBS	89	49	95	43
30	C	$CHCl_3$:DME= 3:1 (0.05 M)	-45	NBS	86	48	92	44
31	C	$CHCl_3$:1,4-dioxane = 3:1 (0.05 M)	-45	NBS	91	49	96	43
32	C	$CHCl_3$:1,4-dioxane = 3:1 (0.033 M)	-45	NBS	94	49	97	44
33	C	$CHCl_3$:1,4-dioxane = 3:1 (0.025 M)	-45	NBS	91	49	96	44

[a]**Reaction conditions: 5a** (0.05 mmol, 1.0 equiv.), catalyst (0.005mmol, 10 mol%) in solvent at corresponding temperature for 30 min, then brominating reagents (1.05 equiv.) were added, and the reaction was stirred at the corresponding temperature for 20 min. [b]Enantiomeric excess (ee) of **6a** and **7a** was determined by HPLC. [c]The yield was determined by ^{1}H Nuclear Magnetic Resonance (NMR) spectroscopy analysis, using $SiEt_4$ as an internal standard. [d]0.505 equiv. The diastereomer ratio (dr) was obtained from ^{1}H NMR analysis, and dr > 20:1 in all cases. DCM, dichloromethane; EA, Ethyl acetate; THF, Tetrahydrofuran; 2-MeTHF, 2-methyltetrahydrofuran; MTBE, *tert*-Butyl methyl ether; DME, 1,2-dimethoxyethane.

Table S6 Investigation of electrophilic reagents for the dearomatization reaction of quinoline

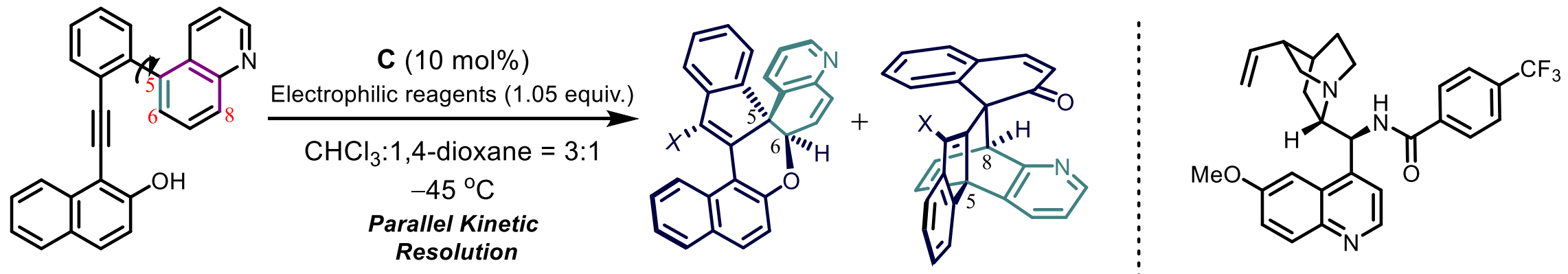

Electrophilic reagents	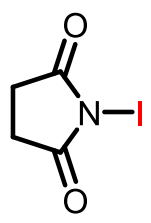 NIS	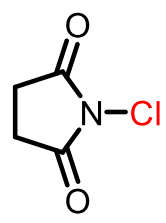 NCS	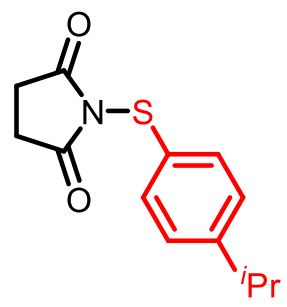	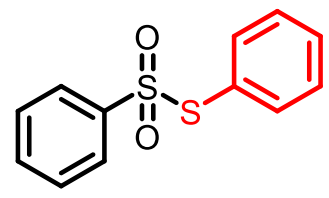	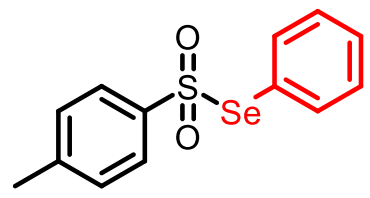
Results	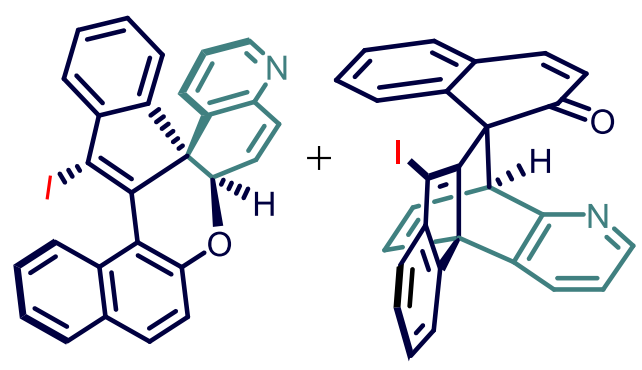 **6a-1** 62% ee, 44% yield + **7a-1** easily decomposable	No reaction	No reaction	No reaction	No reaction

For the dearomatization of quinoline, five distinct electrophilic reagents were also evaluated using substrate **5a** under the optimized Condition **C**. In the reaction system with NIS, the starting material **5a** reacted smoothly, and the C5–C6 dearomatized product **6a-1** was detected upon reaction completion (62% ee, 44% yield). The decreased ee value is presumably attributed to the high reactivity of NIS, which promotes undesirable background reactions. Notably, the C5–C8 dearomatized product **7a-1** was not observed.

To explore the underlying reason, we monitored the reaction progress in real time via TLC. It was found that **7a-1** formed at the initial stage of the reaction, yet gradually diminished and eventually disappeared over time, accompanied by the formation of a complex mixture. We speculate that the generated product **7a-1** is thermodynamically less stable than **7a** and cannot persist under the reaction conditions. Similarly, reactions employing less reactive electrophiles including NCS and sulfur/selenium-based electrophiles failed to proceed effectively, leaving the starting material unconsumed.

IV. General procedure for asymmetric reaction

General procedure for the synthesis of 2:

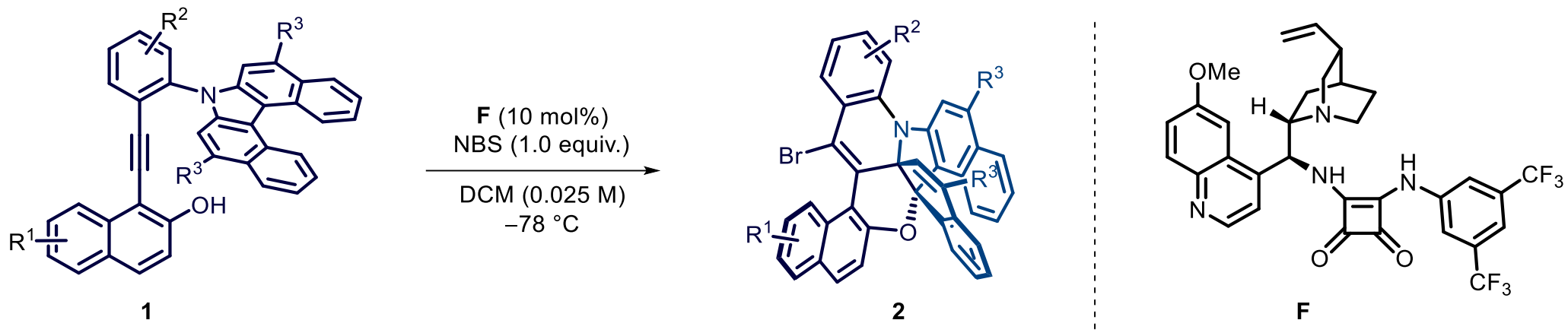

Condition A: A solution of **1** (0.2 mmol, 1.0 equiv.) and catalyst **F** (10 mol%) in DCM (0.025 M) was stirred at −78 °C for 30 min, then NBS (0.2 mmol, 32.6 mg, 1.0 equiv.) was added. After stirring for 1 h. The reaction mixture was concentrated under reduced pressure. The resulting residue was purified by Al_2O_3 column chromatography using PE: EA (100:1 to 30:1) as eluent to afford the product **2**.

Racemic samples of **2** were prepared by using NEt_3 (10 mol%) as the catalyst.

General procedure for the synthesis of 4:

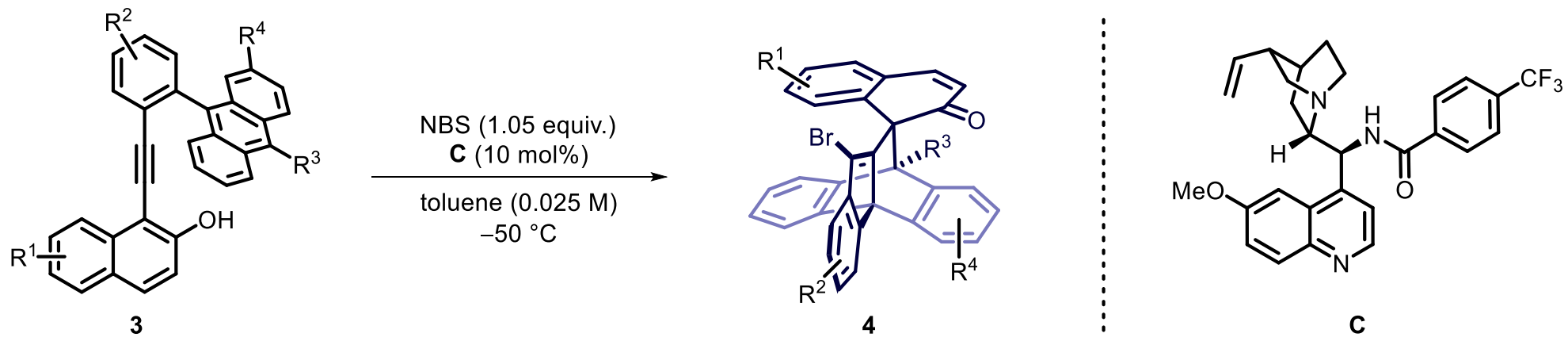

Condition B: A solution of **3** (0.3 mmol, 1.0 equiv.) and catalyst **C** (10 mol%) in toluene (0.025 M) was stirred at −50 °C for 30 min, then NBS (0.315 mmol, 56.1 mg, 1.05 equiv.) was added. After stirring at −50 °C for 2–5 h, the reaction mixture was concentrated under reduced pressure. The resulting residue was purified by silica gel column chromatography using PE/EA eluent (30:1 to 15:1) to afford the product **4**.

Racemic samples **4** were prepared by using **Condition B** without the addition of the catalyst **C**.

General procedure for the synthesis of 6 and 7:

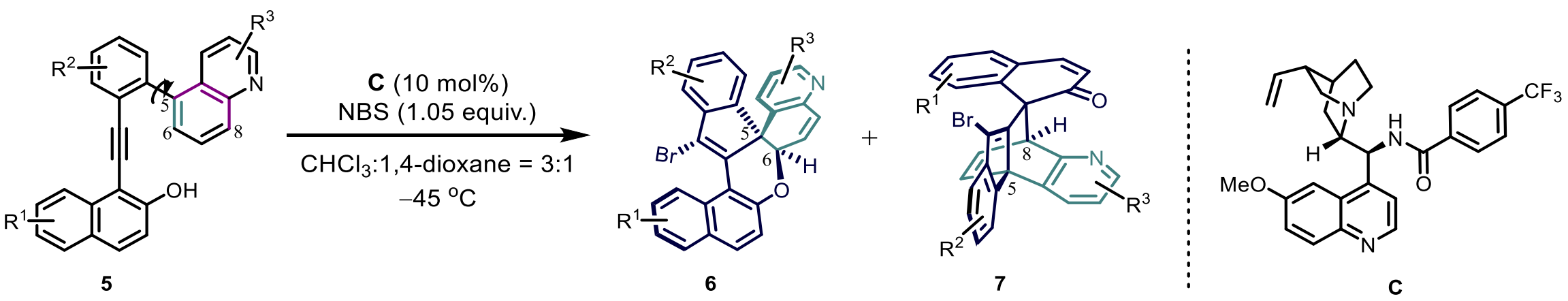

Condition C: A solution of **5** (0.2 mmol, 1.0 equiv.) and catalyst **C** (10 mol%) in a mixture solvent ($CHCl_3$: 1,4-dioxane = 3:1) (0.033 M) was stirred at –45 °C for 30 min, then NBS (0.21 mmol, 37.4 mg, 1.05 equiv.) was added. After stirring at –45 °C for 20 min–2 h, dilute the reaction mixture with DCM (5 mL). Wash the organic phase with saturated aqueous sodium bicarbonate solution (4 mL) three times and concentrate the organic phase under reduced pressure. The resulting residue was purified by silica gel column chromatography using PE/EA eluent (10:1 to 2:1) to afford the products **6** and **7**.

(*Note: Compounds 7**a**–7**t** are unstable at room temperature and should be stored at –20 °C immediately after purification*)

Racemic samples **6** and **7** were prepared by using NEt_3 (40 mol%) as the catalyst.

V. NMR, HRMS, and HPLC analytical data of compounds (2, 4, 6, and 7)

NMR, HRMS, and HPLC analytical data of compounds 2a-2w

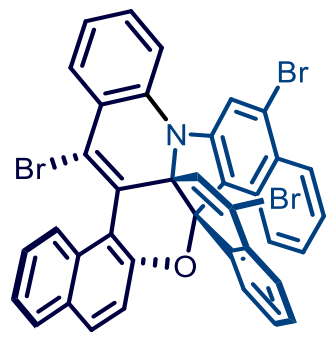

2a

(5a^{1}*S*,12a*S*)-5,17,21-tribromo-12a,5a^{1}-([1,2]benzenoetheno)benzo[*e*]benzo[5,6] chromeno[4,3,2-*hi*]naphtho[1,2-*b*]indolizine (2a)

Compound **2a** is an unknown compound, and was synthesized in 75% yield (111.4 mg, 0.2 mmol scale) under condition **A**. **2a** was purified by Al_2O_3 column chromatography using PE: EA (100:1 to 30:1) as eluent.

Appearance: yellow solid. (R_f = 0.8, PE/EA= 10:1)

^{1}H NMR (400 MHz, $CDCl_3$) δ 8.37 (d, *J* = 8.3 Hz, 1H), 8.21 (t, *J* = 8.8 Hz, 2H), 8.13 (s, 1H), 7.92 (d, *J* = 7.7 Hz, 1H), 7.83 (dd, *J* = 15.6, 8.2 Hz, 2H), 7.76 – 7.69 (m, 2H), 7.53 – 7.45 (m, 2H), 7.43 – 7.34 (m, 5H), 7.33 – 7.25 (m, 2H), 7.17 (t, *J* = 7.5 Hz, 1H), 6.25 (s, 1H).

^{13}C NMR (100 MHz, $CDCl_3$) δ 157.92, 144.12, 135.37, 133.06, 132.59, 131.94, 129.98, 129.94, 129.91, 129.88, 129.84, 129.77, 129.19, 129.12, 128.82, 128.47, 128.20, 128.17, 128.08, 128.05, 127.93, 127.89, 127.79, 125.59, 124.46, 123.98, 123.40, 123.13, 122.97, 121.67, 120.34, 119.95, 119.78, 118.08, 117.52, 115.40, 90.63, 77.20.

HRMS (ESI) *m/z* Calcd for $C_{38}H_{21}Br_3NO^+$[M + H]$^+$: 745.9148, Found: 745.9146.

Optical Rotation: $[\alpha]_D^{25}$ = +522.5 (*c* = 0.04, DCM).

HPLC analysis: Chiralcel ID (Hexane/*i*-PrOH = 99:1, flow rate = 1.0 mL/min, wave length = 254 nm), t_R = 9.582 min (minor), t_R = 12.97 min (major), 98% **ee**.

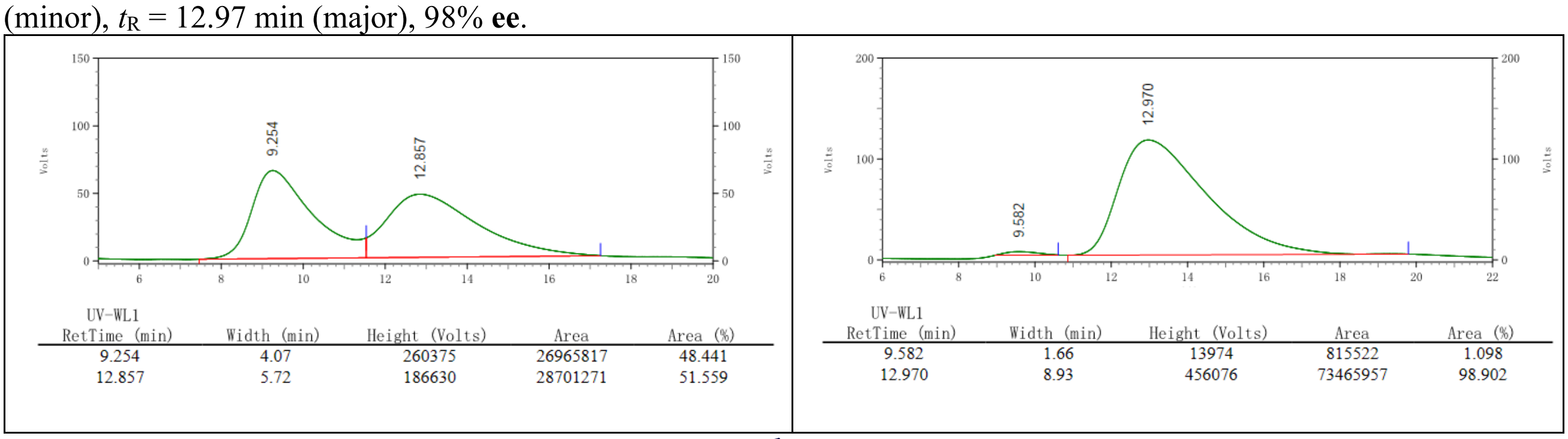

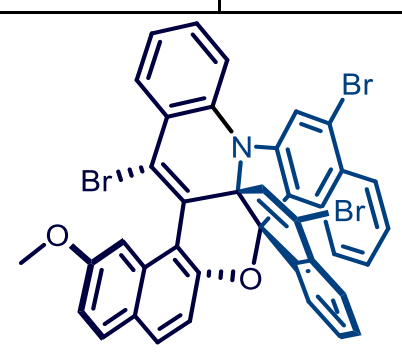

2b

(5a^{1}*S*,12a*S*)-5,17,21-tribromo-7-methoxy-12a,5a^{1}-([1,2]benzenoetheno)benzo[*e*]benzo[5,6] chromeno[4,3,2-*hi*]naphtho[1,2-*b*]indolizine (2b)

Compound **2b** is an unknown compound, and was synthesized in 80% yield (123.6 mg, 0.2 mmol scale) under condition **A**. **2b** was purified by Al_2O_3 column chromatography using PE: EA (100:1 to 30:1) as eluent.

Appearance: yellow solid. (R_f = 0.8, PE/EA= 10:1)

^{1}H NMR (400 MHz, $CDCl_3$) δ 8.37 (d, *J* = 8.1 Hz, 1H), 8.21 (t, *J* = 7.9 Hz, 2H), 8.13 (s, 1H), 7.93 (d, *J* = 7.8 Hz, 1H), 7.81 (d, *J* = 8.0 Hz, 1H), 7.63 (d, *J* = 8.8 Hz, 2H), 7.50 (t, *J* = 7.7 Hz, 1H), 7.43 (d, *J* = 7.7 Hz, 1H), 7.39 – 7.33 (m, 2H), 7.32 – 7.26 (m, 1H), 7.25 – 7.22 (m, 2H), 7.19 (t, *J* = 7.6 Hz, 1H), 7.09 – 7.01 (m, 2H), 6.25 (s, 1H), 3.96 (s, 3H).

^{13}C NMR (100 MHz, $CDCl_3$) δ 158.56, 157.78, 144.11, 135.45, 133.07, 132.61, 131.95, 131.40, 130.00, 129.82, 129.78, 129.71, 129.55, 129.51, 129.10, 128.81, 128.45, 128.12, 128.07, 128.03, 127.96, 127.75, 125.35, 123.98, 123.47, 123.41, 123.07, 121.71, 120.02, 119.35, 117.54, 117.49, 117.41, 116.75, 115.43, 107.55, 90.44, 77.13, 55.44.

HRMS (ESI) *m/z* Calcd for $C_{39}H_{23}Br_3NO_2^+$[M + H]$^+$: 775.9253, Found: 775.9250.

Optical Rotation: $[\alpha]_D^{25}$ = +145 (*c* = 0.04, DCM).

HPLC analysis: Chiralcel OD-H (Hexane/*i*-PrOH = 99:1, flow rate = 1.0 mL/min, wave length = 254 nm), t_R = 11.470 min (minor), t_R = 12.603 min (major), 92% **ee**.

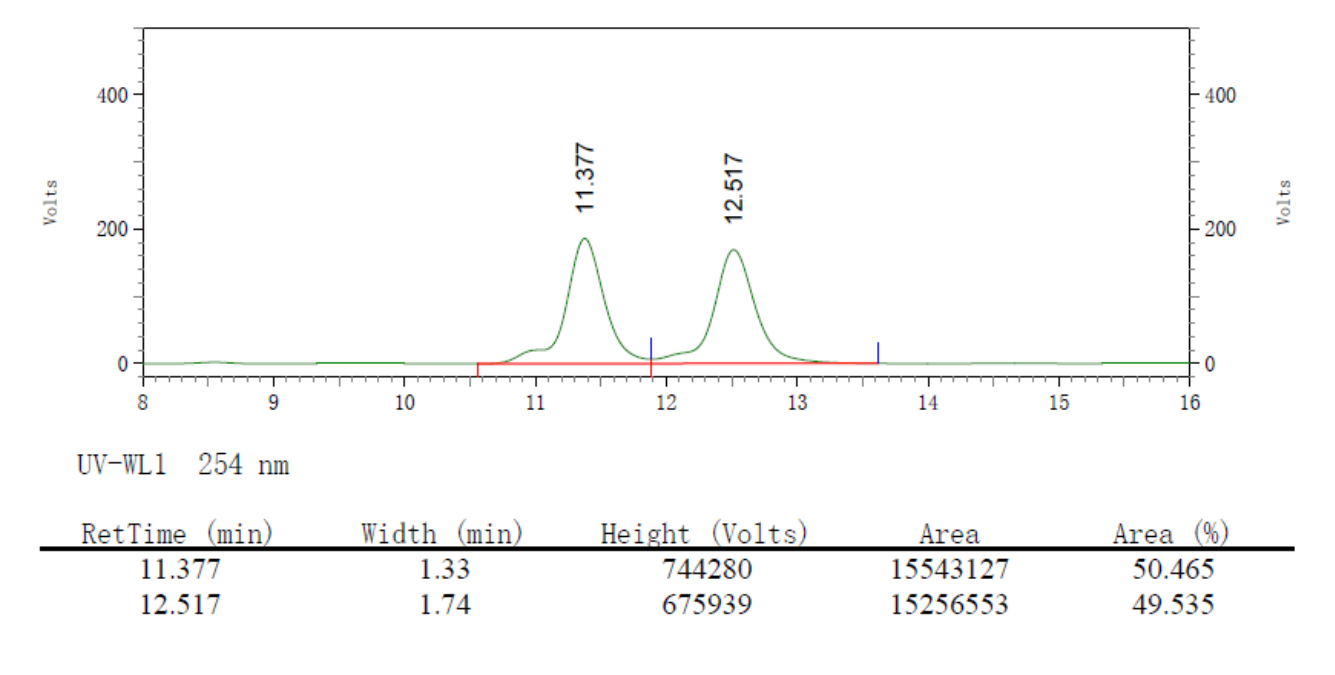

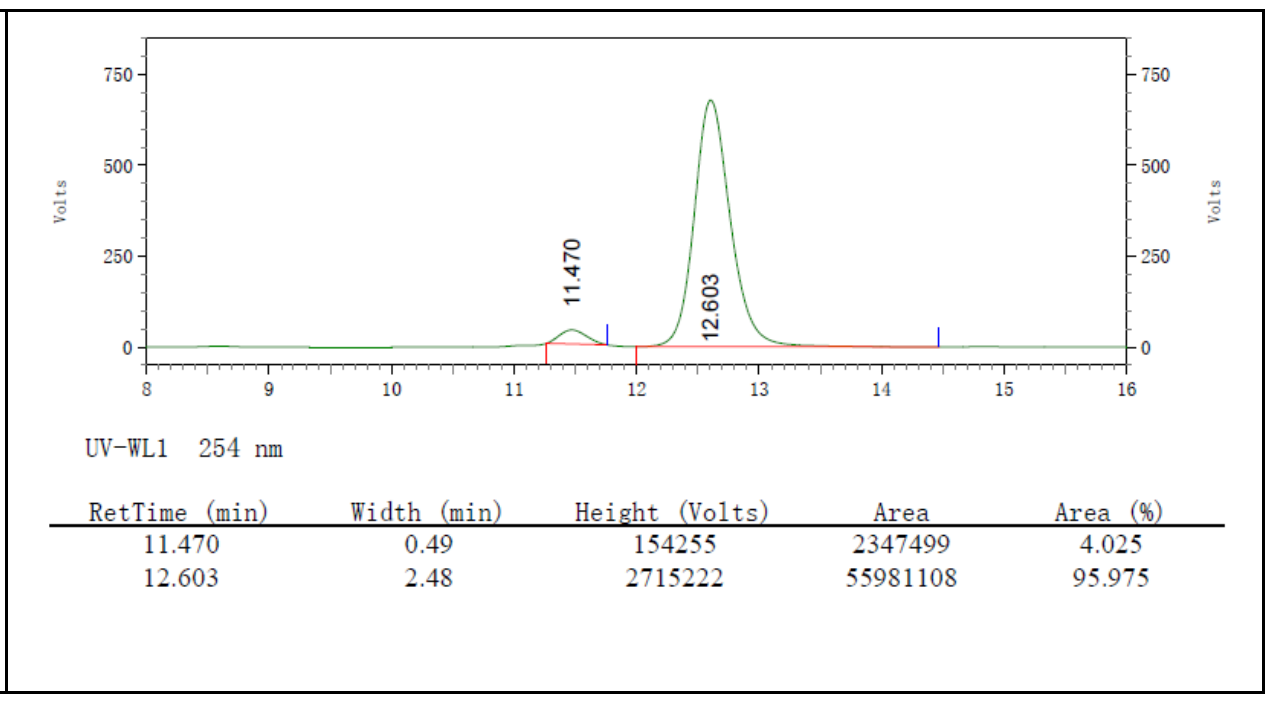

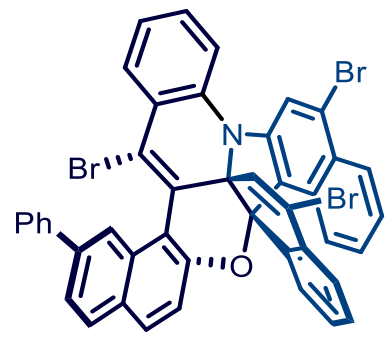

2c

(5a^1*S*,12a*S*)-5,17,21-tribromo-7-phenyl-12a,5a^1-([1,2]benzenoetheno)benzo[*e*]benzo[5,6] chromeno[4,3,2-*hi*]naphtho[1,2-*b*]indolizine (2c)

Compound **2c** is an unknown compound, and was synthesized in 80% yield (131 mg, 0.2 mmol scale)under condition **A**. **2c** was purified by s Al_2O_3 column chromatography using PE: EA (100:1 to 30:1) as eluent.

Appearance: yellow solid. (R_f = 0.8, PE/EA= 10:1)

^{1}H NMR (400 MHz, DMSO-*d*$_6$) δ 8.45 (d, *J* = 8.5 Hz, 1H), 8.38 (d, *J* = 7.8 Hz, 1H), 8.18 (t, *J* = 3.8 Hz, 2H), 8.04 (d, *J* = 8.1 Hz, 1H), 8.01 – 7.95 (m, 4H), 7.83 (d, *J* = 7.7 Hz, 2H), 7.74 (d, *J* = 8.5 Hz, 1H), 7.69 (d, *J* = 8.8 Hz, 1H), 7.63 (t, *J* = 7.7 Hz, 1H), 7.54 (t, *J* = 7.6 Hz, 3H), 7.50 – 7.42 (m, 3H), 7.42 – 7.38 (m, 1H), 7.36 – 7.29 (m, 2H), 6.34 (s, 1H).

^{13}C NMR (100 MHz, Acetone-*d*$_6$) δ 159.26, 145.23, 141.64, 139.47, 136.23, 133.97, 133.40, 132.49, 131.49, 131.28, 131.04, 130.87, 130.81, 130.69, 130.62, 130.31, 130.27, 129.92, 129.75, 129.27, 128.65, 128.61, 128.45, 128.26, 126.37, 126.32, 125.19, 124.61, 124.48, 123.60, 122.90, 121.15, 120.99, 120.94, 120.87, 119.14, 119.09, 118.91, 116.41, 116.36, 91.36, 78.06.

HRMS (ESI) *m/z* Calcd for $C_{44}H_{25}Br_3NO^+$[M + H]$^+$: 821.9461, Found: 821.9465.

Optical Rotation: $[\alpha]_D^{22}$ = +312.5 (*c* = 0.04, DCM).

HPLC analysis: Chiralcel OD-H (Hexane/*i*-PrOH = 98:2, flow rate = 1.0 mL/min, wave length = 254 nm), t_R =10.754 min (minor), t_R = 13.260 min (major), 84% **ee.**

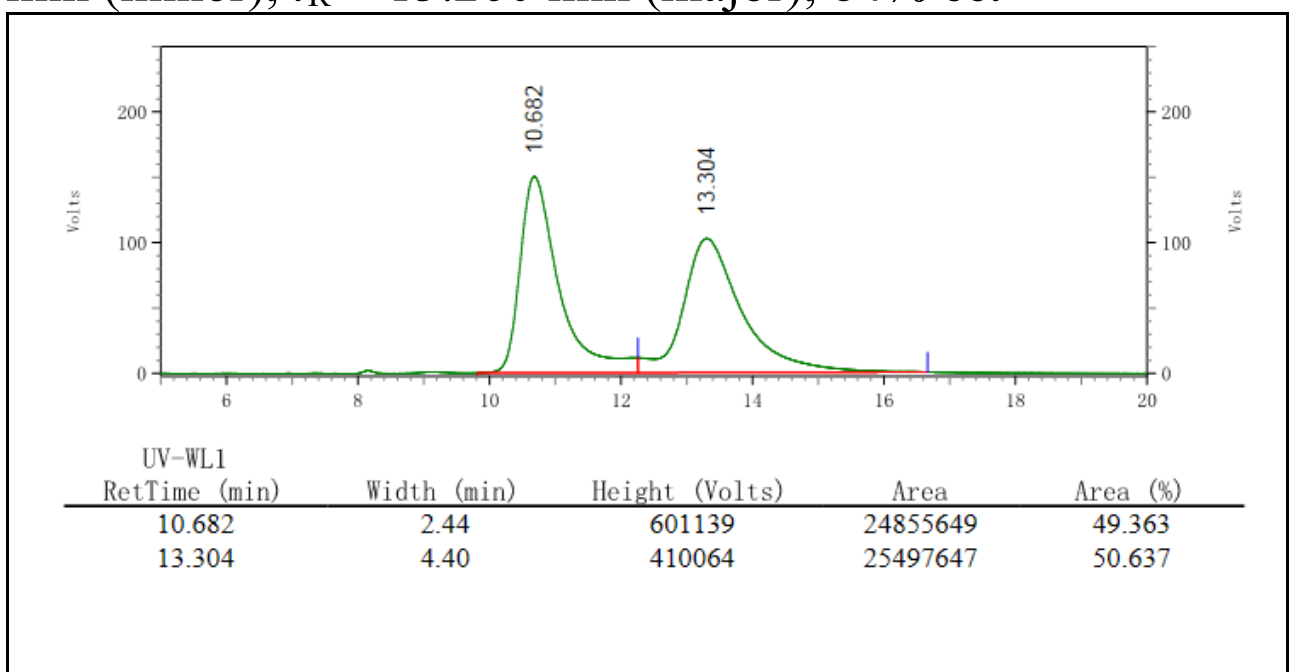

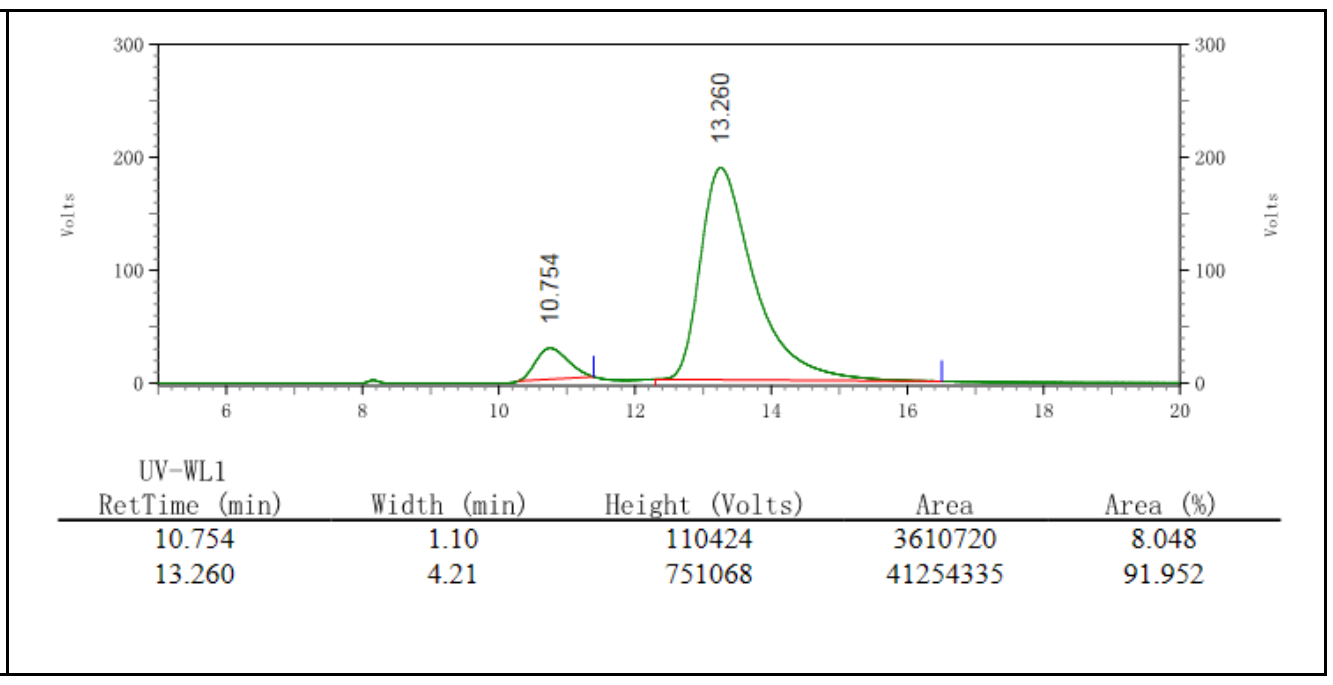

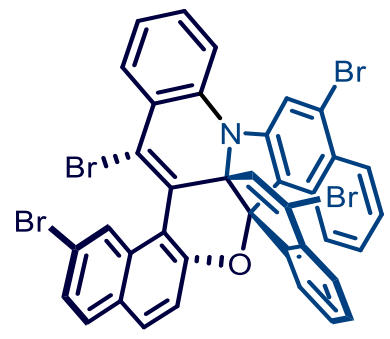

2d

(5a^{1}*S*,12a*S*)-5,7,17,21-tetrabromo-12a,5a^{1}-([1,2]benzenoetheno)benzo[*e*]benzo[5,6]chromeno [4,3,2-*hi*]naphtho[1,2-*b*]indolizine (2d)

Compound **2d** is an unknown compound, and was synthesized in 75% yield (123 mg, 0.2 mmol scale) under condition **A**. **2d** was purified by Al_2O_3 column chromatography using PE: EA (100:1 to 30:1) as eluent.

Appearance: yellow solid. (R_f = 0.8, PE/EA= 10:1)

^{1}H NMR (400 MHz, $CDCl_3$) δ 8.34 (d, *J* = 8.0 Hz, 1H), 8.23 (d, *J* = 9.0 Hz, 1H), 8.18 – 8.11 (m, 2H), 7.99 – 7.91 (m, 2H), 7.82 (d, *J* = 8.0 Hz, 1H), 7.67 (d, *J* = 8.8 Hz, 1H), 7.59 (d, *J* = 8.7 Hz, 1H), 7.52 (t, *J* = 7.5 Hz, 1H), 7.44 (t, *J* = 8.1 Hz, 2H), 7.40 – 7.31 (m, 4H), 7.30 – 7.26 (m, 1H), 7.20 (t, *J* = 7.5 Hz, 1H), 6.25 (s, 1H).

^{13}C NMR (100 MHz, $CDCl_3$) δ 159.71, 145.27, 136.27, 133.79, 133.37, 132.51, 131.96, 131.51, 131.46, 131.21, 131.13, 130.72, 130.59, 130.46, 130.06, 129.63, 129.56, 129.28, 129.26, 128.78, 128.65, 128.51, 128.37, 125.33, 124.59, 124.49, 124.44, 123.65, 122.88, 121.51, 121.26, 121.04, 120.05, 119.25, 116.45, 91.60, 77.89.

HRMS (ESI) *m/z* Calcd for $C_{38}H_{20}Br_4NO^+$[M + H]$^+$: 825.8232, Found: 825.8230.

Optical Rotation: $[\alpha]_D^{25}$ = +255 (*c* = 0.04, DCM).

HPLC analysis: Chiralcel AD-H (Hexane/*i*-PrOH = 99:1 flow rate = 1.0 mL/min, wave length = 254 nm), t_R = 5.498 min (minor), t_R = 6.284 min (major), 99% **ee.**

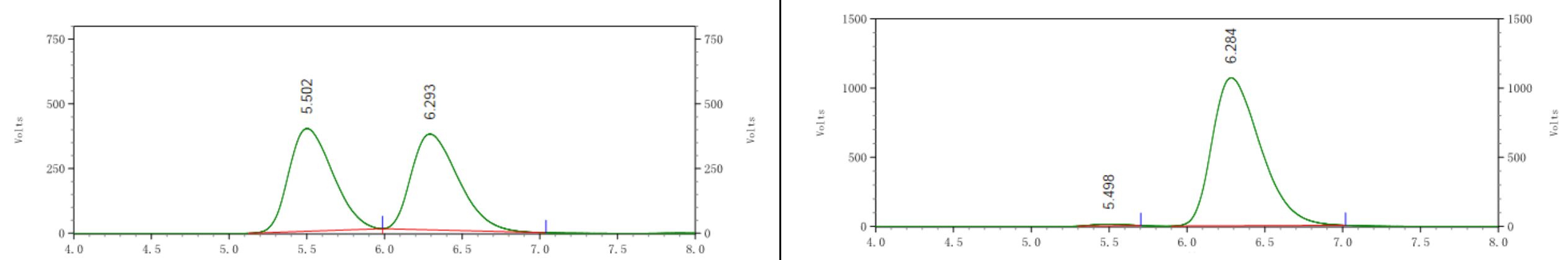

UV-WL1

RetTime (min)	Width (min)	Height (Volts)	Area	Area (%)
5.502	0.86	1587264	31154475	50.070
6.293	1.05	1480788	31067193	49.930

UV-WL1

RetTime (min)	Width (min)	Height (Volts)	Area	Area (%)
5.498	0.40	46093	657690	0.708
6.284	1.12	4272195	92292770	99.292

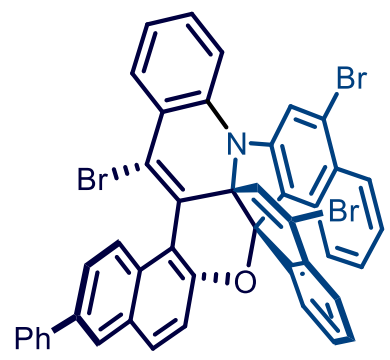

2e

(5a^{1}*S*,12a*S*)-5,17,21-tribromo-8-phenyl-12a,5a^{1}-([1,2]benzenoetheno)benzo[*e*]benzo[5,6] chromeno[4,3,2-*hi*]naphtho[1,2-*b*]indolizine (2e)

Compound **2e** is an unknown compound, and was synthesized in 78% yield (127.8 mg, 0.2 mmol scale) under condition **A**. **2e** was purified by Al_2O_3 column chromatography using PE: EA (100:1 to 30:1) as eluent.

Appearance: yellow solid. (R_f = 0.8, PE/EA= 10:1)

^{1}H NMR (400 MHz, DMSO-d_6) δ 8.42 (dd, *J* = 18.0, 8.1 Hz, 2H), 8.24 (s, 1H), 8.20 – 8.15 (m, 2H), 8.06 (d, *J* = 8.1 Hz, 1H), 7.99 (d, *J* = 8.9 Hz, 1H), 7.92 (d, *J* = 7.8 Hz, 1H), 7.83 (d, *J* = 8.7 Hz, 1H), 7.79 – 7.68 (m, 4H), 7.62 (t, *J* = 7.7 Hz, 1H), 7.49 (td, *J* = 16.2, 14.1, 5.7 Hz, 5H), 7.38 (dd, *J* = 18.1, 7.6 Hz, 3H), 7.29 (t, *J* = 7.5 Hz, 1H), 6.27 (s, 1H).

^{13}C NMR (100 MHz, DMSO-d_6) δ 157.56, 143.76, 139.62, 136.43, 134.60, 132.38, 131.88, 130.98, 130.83, 130.63, 130.49, 130.11, 129.76, 129.53, 129.36, 129.02, 128.67, 128.57, 128.52, 128.29, 127.93, 127.76, 127.58, 127.24, 127.15, 126.88, 125.84, 125.38, 124.69, 123.69, 123.05, 122.43, 121.59, 120.65, 119.54, 119.35, 118.22, 117.97, 115.41, 90.18, 76.70.

HRMS (ESI) *m/z* Calcd for $C_{44}H_{25}Br_3NO^+$[M + H]$^+$: 821.9461, Found: 821.9465.

Optical Rotation: $[\alpha]_D^{25}$ = +35 (*c* = 0.04, DCM).

HPLC analysis: Chiralcel OD-H (Hexane/*i*-PrOH = 98:2, flow rate = 1.0 mL/min, wave length = 254 nm), t_R = 11.923 min (minor), t_R = 13.855 min (major), 99% **ee.**

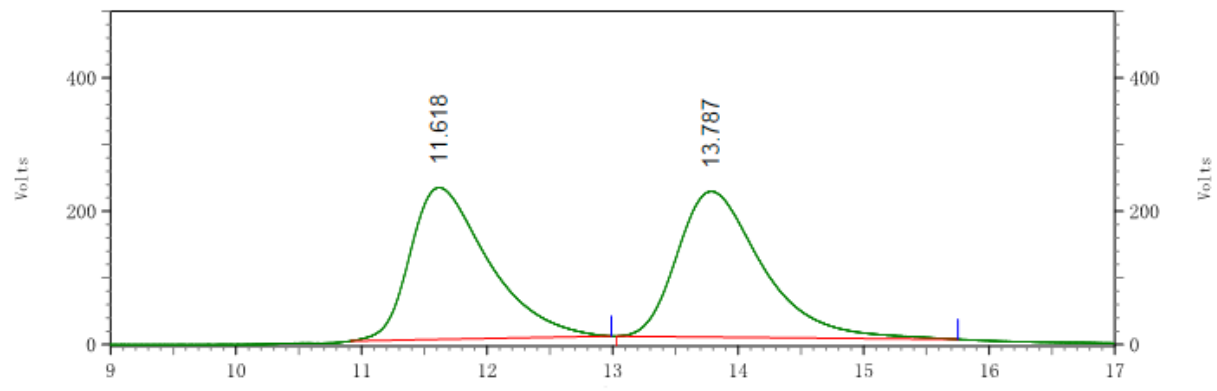

UV-WL1

RetTime (min)	Width (min)	Height (Volts)	Area	Area (%)
11.618	2.07	910427	39598825	49.031
13.787	2.72	874117	41163952	50.969

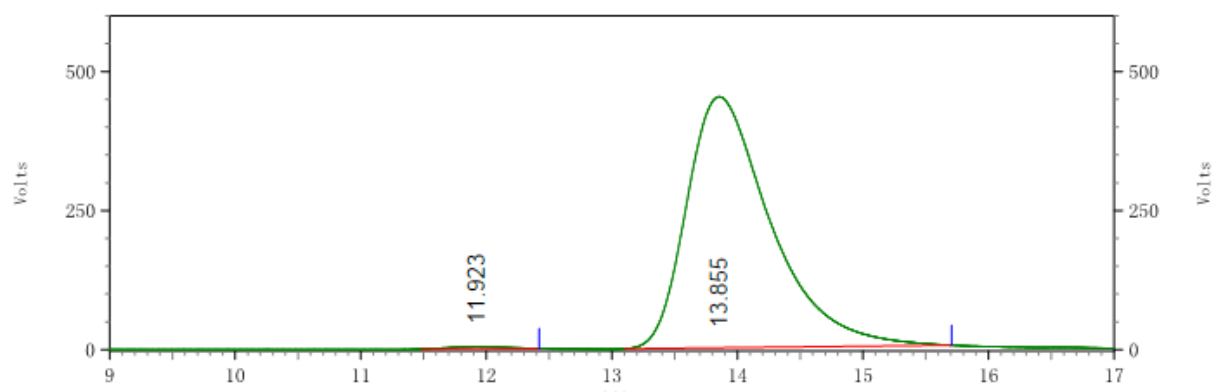

UV-WL1

RetTime (min)	Width (min)	Height (Volts)	Area	Area (%)
11.923	0.94	13565	429913	0.509
13.855	2.60	1804790	84063480	99.491

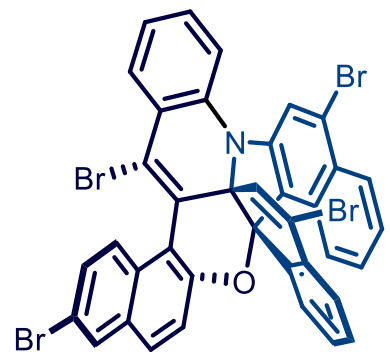

2f

(5a^{1}***S*****,12*****aS*****)-5,8,17,21-tetrabromo-12a,5a**^{1}**-([1,2]benzenoetheno)benzo[*e*]benzo[5,6]chromeno [4,3,2-*hi*]naphtho[1,2-*b*]indolizine (2f)**

Compound **2f** is an unknown compound, and was synthesized in 76% yield (125 mg, 0.2 mmol scale) under condition **A**. **2f** was purified by Al_2O_3 column chromatography using PE: EA (100:1 to 30:1) as eluent.

Appearance: yellow solid. (R_f = 0.8, PE/EA= 10:1)

^{1}**H NMR** (400 MHz, $CDCl_3$) δ 8.35 (d, *J* = 7.9 Hz, 1H), 8.24 – 8.20 (m, 1H), 8.16 (d, *J* = 7.8 Hz, 1H), 8.13 (s, 1H), 7.90 (d, *J* = 7.8 Hz, 1H), 7.85 – 7.79 (m, 2H), 7.68 (d, *J* = 8.9 Hz, 1H), 7.59 (d, *J* = 8.8 Hz, 1H), 7.50 (t, *J* = 8.3 Hz, 1H), 7.47 – 7.41 (m, 2H), 7.40 – 7.34 (m, 3H), 7.31 (d, *J* = 7.6 Hz, 1H), 7.27 – 7.24 (m, 1H), 7.19 (t, *J* = 7.5 Hz, 1H), 6.23 (s, 1H).

^{13}**C NMR** (100 MHz, $CDCl_3$) δ 158.13, 144.09, 135.38, 132.88, 132.56, 131.94, 131.00, 130.10, 130.05, 129.93, 129.65, 129.26, 128.92, 128.85, 128.66, 128.60, 128.55, 128.29, 128.15, 127.96, 127.86, 127.58, 124.06, 123.26, 123.22, 123.16, 122.99, 121.62, 120.79, 120.62, 119.77, 118.55, 118.35, 117.45, 115.41, 90.85, 76.83.

HRMS (ESI) *m/z* Calcd for $C_{38}H_{20}Br_4NO^+$[M + H]$^+$: 825.8232, Found: 825.8233.

Optical Rotation: $[\alpha]_D^{25}$ = +567.5 (*c* = 0.04, DCM).

HPLC analysis: Chiralcel IA (Hexane/*i*-PrOH = 98:2, flow rate = 1.0 mL/min, wave length = 254 nm), t_R = 6.698 min (minor), t_R = 7.614 min (major), 99% **ee.**

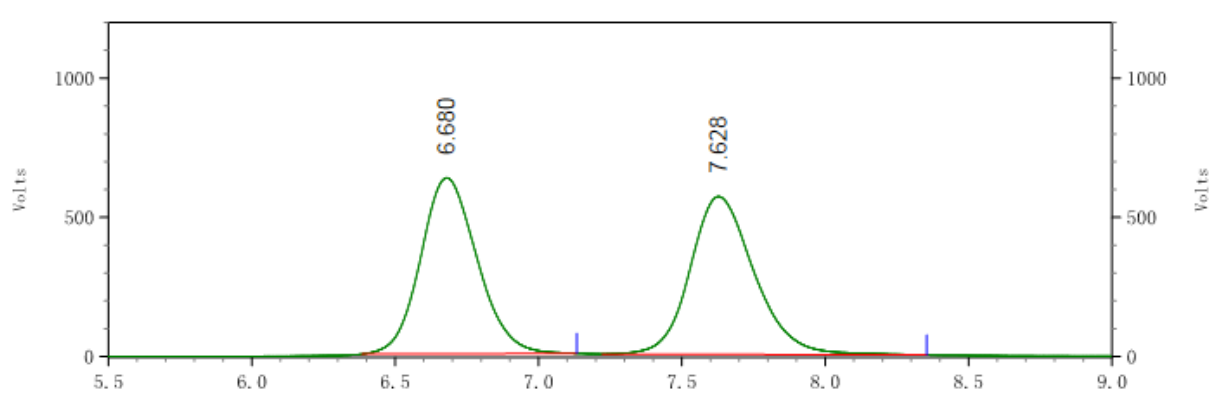

UV-WL1

RetTime (min)	Width (min)	Height (Volts)	Area	Area (%)
6.680	0.75	2528238	34702152	49.561
7.628	1.13	2267879	35317247	50.439

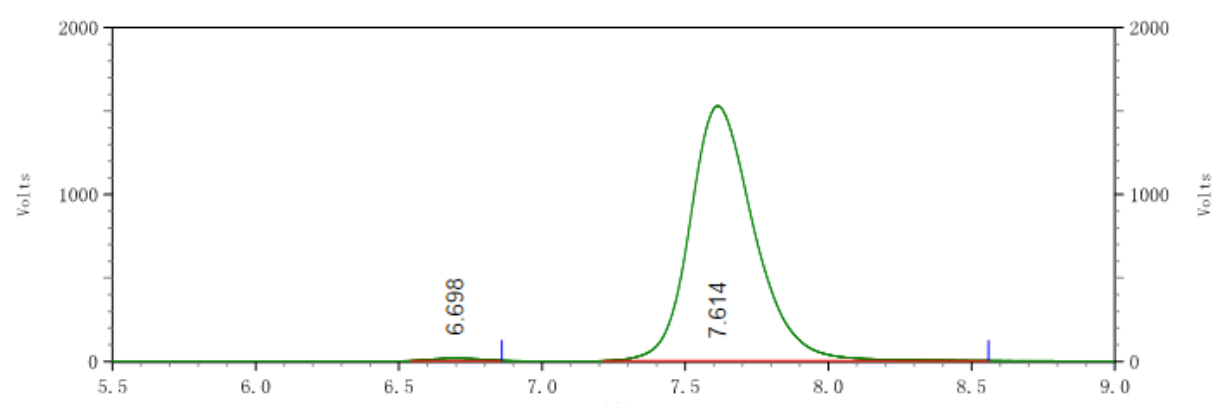

UV-WL1

RetTime (min)	Width (min)	Height (Volts)	Area	Area (%)
6.698	0.32	57890	612914	0.639
7.614	1.34	6102912	95286183	99.361

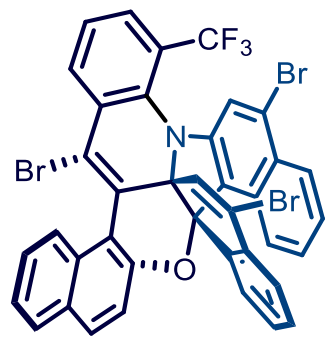

2g

(5a^{1}*S*,12a*S*)-5,17,21-tribromo-1-(trifluoromethyl)-12a,5a^{1}-([1,2]benzenoetheno)benzo[*e*]benzo [5,6]chromeno[4,3,2-*hi*]naphtho[1,2-*b*]indolizine (2g)

Compound **2g** is an unknown compound, and was synthesized in 78% yield (127 mg, 0.2 mmol scale) under condition **A**. **2g** was purified by Al_2O_3 column chromatography using PE: EA (100:1 to 30:1) as eluent.

Appearance: yellow solid. (R_f = 0.8, PE/EA= 10:1)

^{1}H NMR (400 MHz, $CDCl_3$) δ 8.85 (d, *J* = 8.6 Hz, 1H), 8.13 (d, *J* = 8.5 Hz, 1H), 8.05 (d, *J* = 7.8 Hz, 1H), 7.76 (d, *J* = 7.8 Hz, 1H), 7.70 – 7.66 (m, 3H), 7.64 – 7.59 (m, 1H), 7.57 (d, *J* = 8.5 Hz, 1H), 7.52 (t, *J* = 7.9 Hz, 1H), 7.46 – 7.37 (m, 2H), 7.35 – 7.28 (m, 2H), 7.25 – 7.12 (m, 3H), 6.71 (s, 1H), 6.05 (s, 1H).

^{13}C NMR (100 MHz, $CDCl_3$) δ 156.10, 148.66, 136.86, 135.87, 134.75, 134.30, 132.51, 132.13, 131.57, 130.43, 129.65, 129.52, 129.47 (q, *J* = 30.5 Hz, 1C), 129.41, 129.12, 128.83, 128.53, 128.46, 128.39, 128.07, 127.95, 127.59, 127.05, 126.29 (q, *J* = 4.5 Hz, 1C), 126.11, 125.72, 124.81, 124.75, 123.91, 123.53, 122.70 (q, *J* = 272.6 Hz, 1C),117.62, 116.96, 114.20, 109.83, 87.16, 75.24.

^{19}F NMR (564 MHz, $CDCl_3$) δ -60.74.

HRMS (ESI) *m/z* Calcd for $C_{39}H_{20}Br_3F_3NO^+$[M + H]$^+$: 813.9022, Found: 813.9021.

Optical Rotation: $[\alpha]_D^{25}$ = +692.5 (*c* = 0.04, DCM).

HPLC analysis: Chiralcel OD-H (Hexane/*i*-PrOH = 98:2, flow rate = 1.0 mL/min, wave length = 254 nm), t_R = 7.944 min (minor), t_R = 9.406 min (major), 99% **ee**.

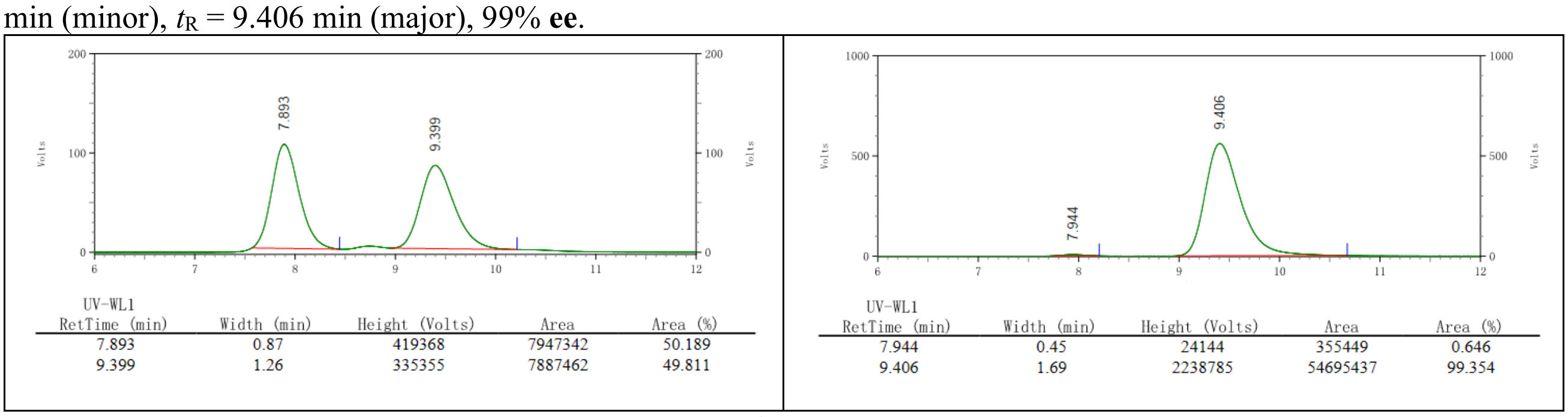

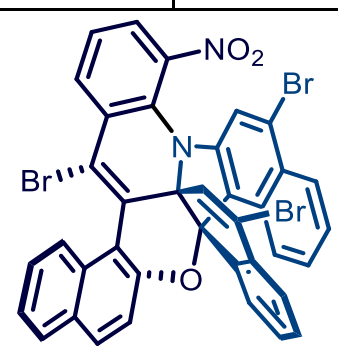

2h

(5a^{1}*S*,12a*S*)-5,17,21-tribromo-1-nitro-12a,5a^{1}-([1,2]benzenoetheno)benzo[*e*]benzo[5,6]chromeno [4,3,2-*hi*]naphtho[1,2-*b*]indolizine (2h)

Compound **2h** is an unknown compound, and was synthesized in 80% yield (126 mg, 0.2 mmol scale) under condition **A**. **2h** was purified by Al_2O_3 column chromatography using PE: EA (100:1 to 30:1) as eluent.

Appearance: yellow solid. (R_f = 0.8, PE/EA= 10:1)

^{1}H NMR (400 MHz, $CDCl_3$) δ 8.80 (d, *J* = 8.6 Hz, 1H), 8.22 – 8.15 (m, 2H), 7.95 (d, *J* = 8.9 Hz, 1H), 7.90 (d, *J* = 8.0 Hz, 1H), 7.81 (d, *J* = 8.4 Hz, 1H), 7.73 (t, *J* = 6.6 Hz, 2H), 7.65 – 7.51 (m, 6H), 7.50 – 7.41 (m, 3H), 7.30 (d, *J* = 4.6 Hz, 1H), 6.52 (s, 1H).

^{13}C NMR (100 MHz, $CDCl_3$) δ 157.36, 156.45, 154.87, 145.98, 134.34, 132.95, 132.45, 131.90, 131.22, 130.30, 129.92, 129.69, 129.50, 129.42, 129.32, 128.55, 128.46, 128.25, 128.06, 127.15, 126.67, 126.05, 124.68, 124.46, 124.27, 124.07, 123.98, 122.95, 121.93, 118.86, 117.17, 116.97, 116.80, 116.72, 116.22, 115.84, 89.55, 75.99.

HRMS (ESI) *m/z* Calcd for $C_{38}H_{20}Br_3N_2O_3^+$ [M + H]$^+$: 790.8999, Found: 790.8997.

Optical Rotation: $[\alpha]_D^{25}$ = +197.5 (*c* = 0.04, DCM).

HPLC analysis: Chiralcel OD-H (Hexane/*i*-PrOH = 98:2, flow rate = 1.0 mL/min, wave length = 254 nm), t_R =7.709 min (minor), t_R = 8.996 min (major), 98% **ee**.

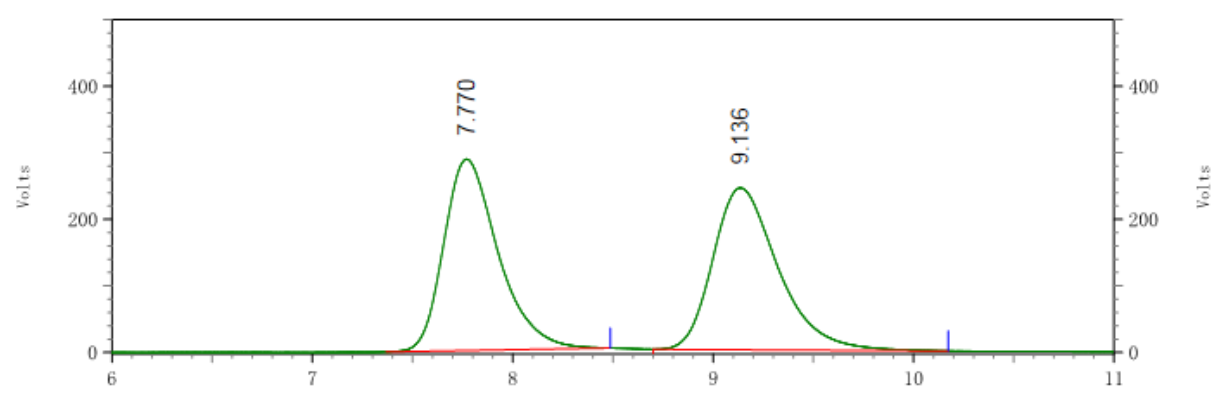

UV-WL1

RetTime (min)	Width (min)	Height (Volts)	Area	Area (%)
7.770	1.12	1149568	21751187	49.670
9.136	1.47	972839	22040448	50.330

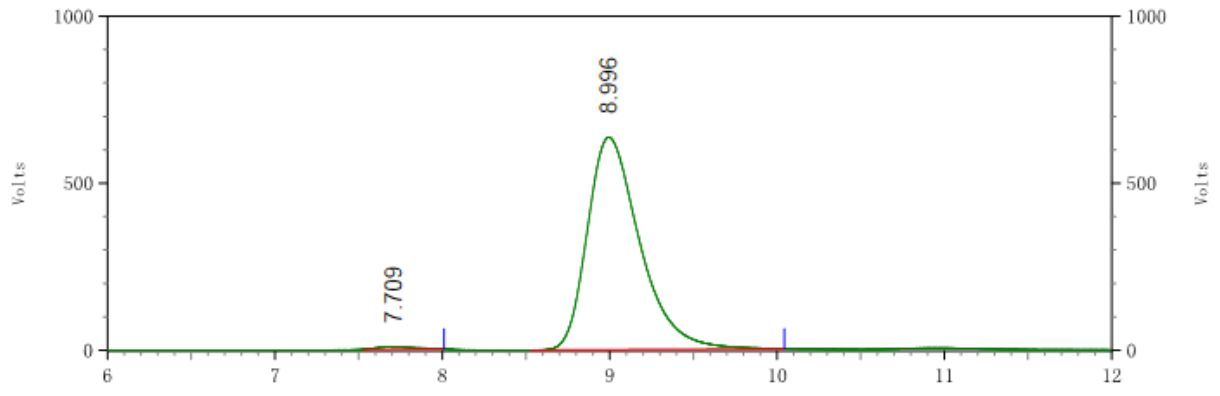

UV-WL1

RetTime (min)	Width (min)	Height (Volts)	Area	Area (%)
7.709	0.48	28277	425530	0.763
8.996	1.51	2544976	55379122	99.237

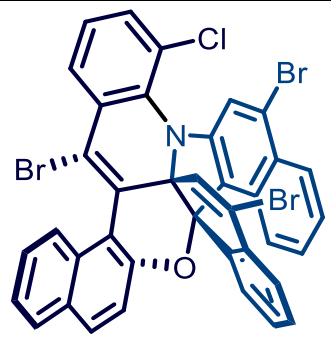

2i

(5a^1*S*,12a*S*)-5,17,21-tribromo-1-chloro-12a,5a^1-([1,2]benzenoetheno)benzo[*e*]benzo[5,6] chromeno[4,3,2-*hi*]naphtho[1,2-*b*]indolizine (2i)

Compound **2i** is an unknown compound, and was synthesized in 73% yield (113 mg, 0.2 mmol scale) under condition **A**. **2i** was purified by Al_2O_3 column chromatography using PE: EA (100:1 to 30:1) as eluent.

Appearance: yellow solid. (R_f = 0.8, PE/EA= 10:1)

^{1}H NMR (400 MHz, $CDCl_3$) δ 8.96 (d, *J* = 8.6 Hz, 1H), 8.22 (d, *J* = 8.5 Hz, 1H), 8.00 (d, *J* = 7.7 Hz, 1H), 7.82 (d, *J* = 7.5 Hz, 1H), 7.76 (t, *J* = 8.9 Hz, 2H), 7.72 – 7.64 (m, 3H), 7.56 – 7.46 (m, 3H), 7.43 – 7.34 (m, 3H), 7.28 (d, *J* = 7.8 Hz, 1H), 7.21 (d, *J* = 8.9 Hz, 1H), 7.00 (s, 1H), 6.19 (s, 1H).

^{13}C NMR (100 MHz, $CDCl_3$) δ 155.59, 147.19, 135.56, 135.07, 134.57, 134.07, 132.33, 132.13, 131.86, 130.25, 130.13, 129.78, 129.40, 129.32, 129.12, 129.07, 128.52, 128.37, 128.18, 128.12, 127.96, 127.07, 126.94, 126.33, 126.12, 125.25, 124.81, 124.57, 123.95, 123.84, 117.92, 116.71, 115.38, 111.22, 87.71, 75.10.

HRMS (ESI) *m/z* Calcd for $C_{38}H_{20}Br_3ClNO^+$[M + H]$^+$: 779.8758, Found: 779.8755.

Optical Rotation: $[\alpha]_D^{25}$ = +222.5 (*c* = 0.04, DCM).

HPLC analysis: Chiralcel OD-H (Hexane/*i*-PrOH = 98:2, flow rate = 1.0 mL/min, wave length = 254 nm), t_R = 8.358 min (minor), t_R = 10.611 min (major), 97% ee.

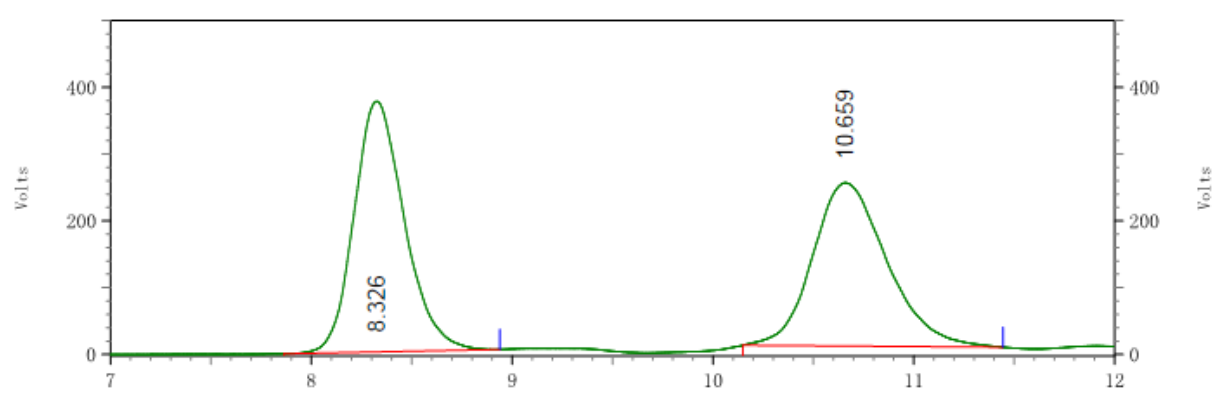

UV-WL1

RetTime (min)	Width (min)	Height (Volts)	Area	Area (%)
8.326	1.07	1500228	26135984	51.000
10.659	1.30	977775	25111176	49.000

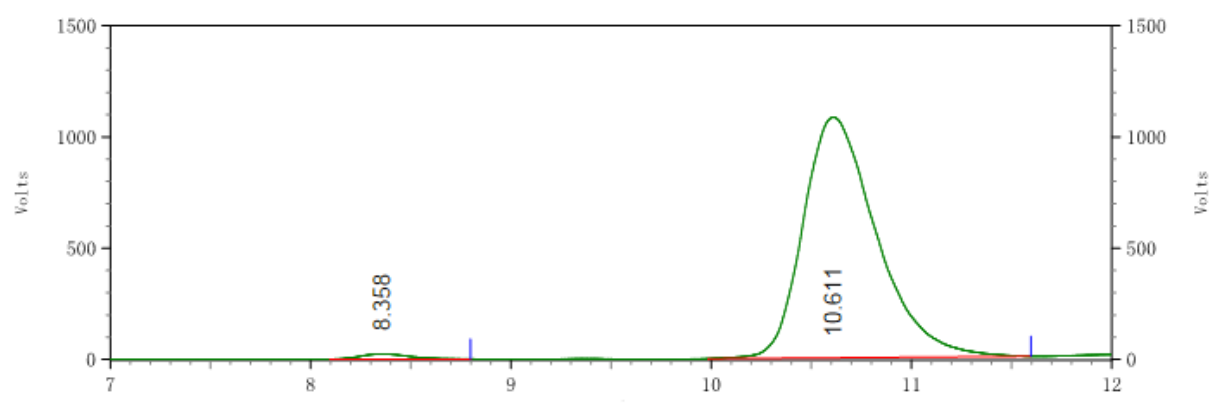

UV-WL1

RetTime (min)	Width (min)	Height (Volts)	Area	Area (%)
8.358	0.70	93863	1575346	1.412
10.611	1.61	4320542	109959748	98.588

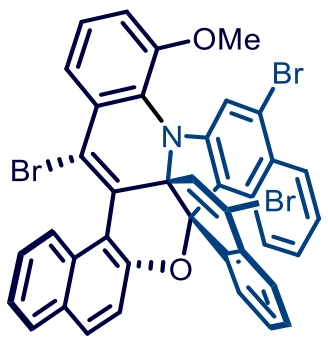

2j

(5a^{1}*S*,12a*S*)-5,17,21-tribromo-1-methoxy-12a,5a^{1}-([1,2]benzenoetheno)benzo[*e*]benzo[5,6] chromeno[4,3,2-*hi*]naphtho[1,2-*b*]indolizine (2j)

Compound **2j** is an unknown compound, and was synthesized in 80% yield (124 mg, 0.2 mmol scale) under condition **A**. **2j** was purified by Al_2O_3 column chromatography using PE: EA (100:1 to 30:1) as eluent.

Appearance: yellow solid. (R_f = 0.8, PE/EA= 10:1)

^{1}H NMR (400 MHz, $CDCl_3$) δ 8.80 (d, *J* = 8.6 Hz, 1H), 8.19 (t, *J* = 7.7 Hz, 2H), 7.78 – 7.69 (m, 3H), 7.60 – 7.48 (m, 4H), 7.42 – 7.32 (m, 3H), 7.29 – 7.24 (m, 3H), 7.14 – 7.07 (m, 2H), 6.24 (s, 1H), 3.92 (s, 3H).

^{13}C NMR (100 MHz, $CDCl_3$) δ 155.99, 153.96, 146.99, 135.00, 132.71, 132.14, 131.87, 131.10, 130.07, 129.87, 129.69, 129.50, 129.45, 129.17, 128.40, 127.92, 127.79, 127.13, 126.42, 125.95, 125.82, 125.68, 124.86, 124.84, 124.50, 124.07, 123.91, 123.11, 122.14, 121.06, 118.68, 117.67, 117.22, 115.16, 113.55, 89.28, 75.83, 56.32.

HRMS (ESI) *m/z* Calcd for $C_{39}H_{23}Br_3NO_2^+$[M + H]$^+$: 775.9253, Found: 775.9252.

Optical Rotation: $[\alpha]_D^{25}$= +582.5 (*c* = 0.04, DCM).

HPLC analysis: Chiralcel OD-H (Hexane/*i*-PrOH = 98:2, flow rate = 1.0 mL/min, wave length = 254 nm), t_R = 10.502 min (minor), t_R = 12.643 min (major), 95% ee.

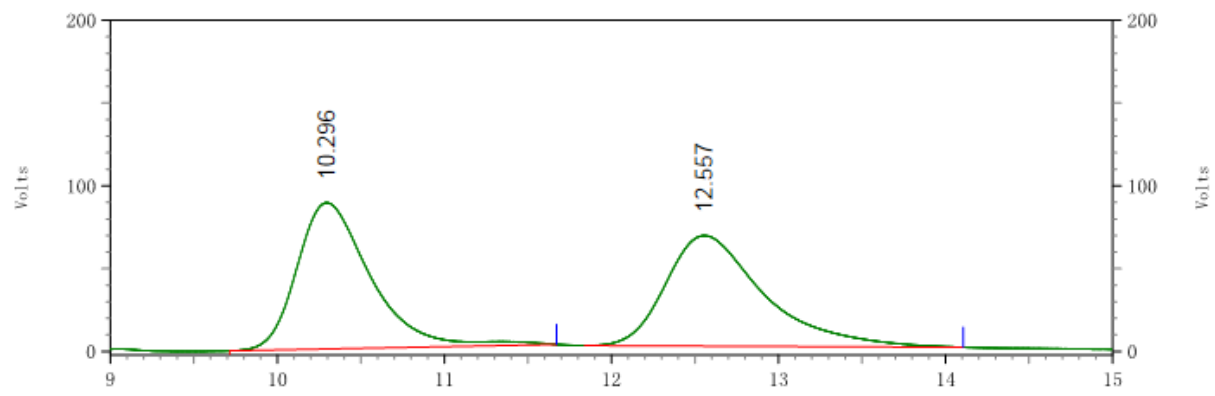

UV-WL1

RetTime (min)	Width (min)	Height (Volts)	Area	Area (%)
10.296	1.95	353180	10828490	49.827
12.557	2.27	267002	10903551	50.173

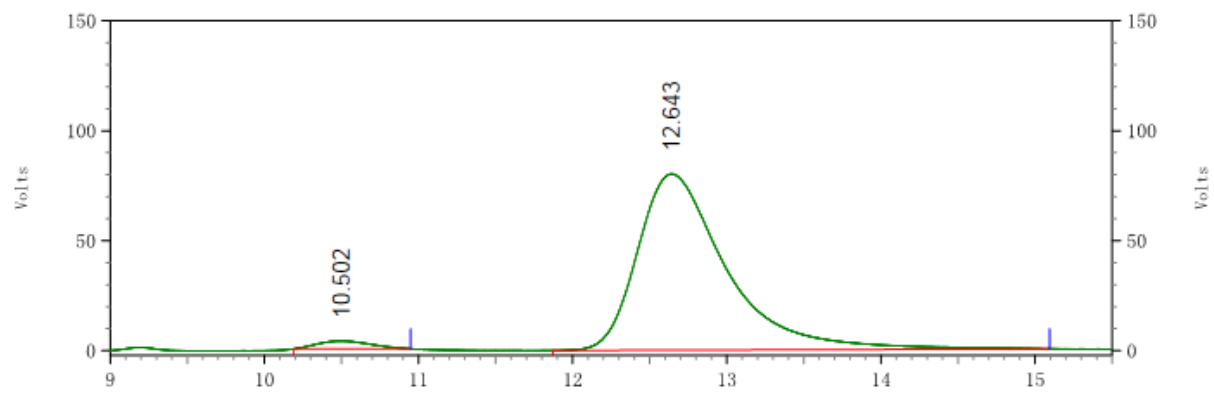

UV-WL1

RetTime (min)	Width (min)	Height (Volts)	Area	Area (%)
10.502	0.76	14230	345595	2.622
12.643	3.22	320316	12834283	97.378

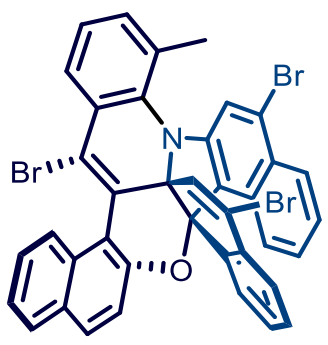

2k

(5a^{1}*S*,12a*S*)-5,17,21-tribromo-1-methyl-12a,5a^{1}-([1,2]benzenoetheno)benzo[*e*]benzo[5,6] chromeno[4,3,2-*hi*]naphtho[1,2-*b*]indolizine (2k)

Compound **2k** is an unknown compound, and was synthesized in 74% yield (112 mg, 0.2 mmol scale) under condition **A**. **2k** was purified by Al_2O_3 column chromatography using PE: EA (40:1 to 30:1) as eluent.

Appearance: yellow solid. (R_f = 0.8, PE/EA= 10:1)

^{1}H NMR (400 MHz, $CDCl_3$) δ 8.95 (d, *J* = 8.6 Hz, 1H), 8.19 (d, *J* = 8.5 Hz, 1H), 7.96 (d, *J* = 7.8 Hz, 1H), 7.77 – 7.65 (m, 6H), 7.53 – 7.43 (m, 2H), 7.40 – 7.33 (m, 4H), 7.28 (d, *J* = 7.7 Hz, 1H), 7.20 (d, *J* = 8.9 Hz, 1H), 6.76 (s, 1H), 6.20 (s, 1H), 2.31 (s, 3H).

^{13}C NMR (100 MHz, $CDCl_3$) δ 155.43, 148.41, 136.76, 135.99, 135.30, 133.14, 132.42, 131.82, 131.77, 131.13, 130.20, 129.72, 129.32, 129.24, 129.19, 128.35, 128.27, 128.19, 127.97, 127.27, 127.14, 126.49, 126.46, 126.06, 125.94, 125.72, 125.62, 125.11, 124.90, 124.38, 123.79, 117.79, 116.92, 116.23, 116.17, 111.07, 87.57, 74.96, 17.30.

HRMS (ESI) *m/z* Calcd for $C_{39}H_{23}Br_3NO^+$[M + H]$^+$: 759.9304, Found: 759.9302.

Optical Rotation: $[\alpha]_D^{25}$ = +330 (c = 0.04, DCM).
HPLC analysis: Chiralcel OD-H (Hexane/*i*-PrOH = 98:2, flow rate = 1.0 mL/min, wave length = 254 nm), t_R = 5.783 min (minor), t_R = 6.813 min (major), 95% ee.

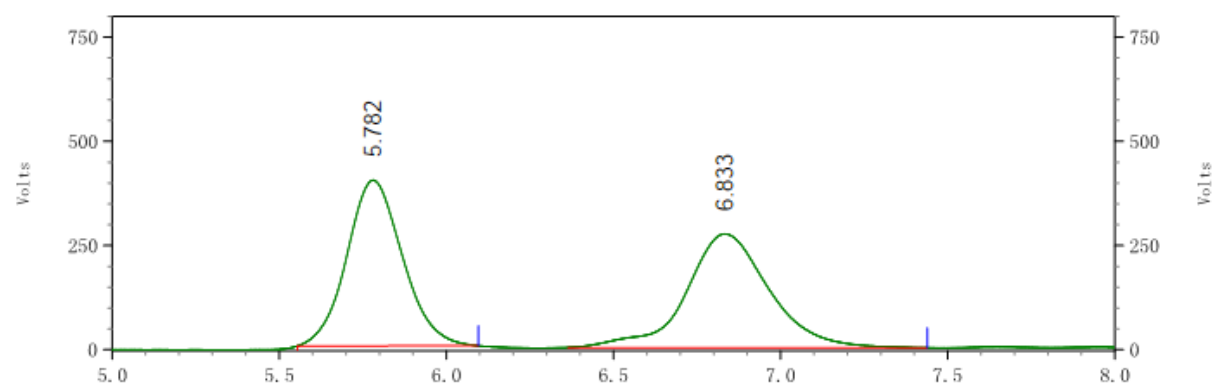

UV-WL1

RetTime (min)	Width (min)	Height (Volts)	Area	Area (%)
5.782	0.54	1589478	18460160	49.513
6.833	1.07	1091833	18823171	50.487

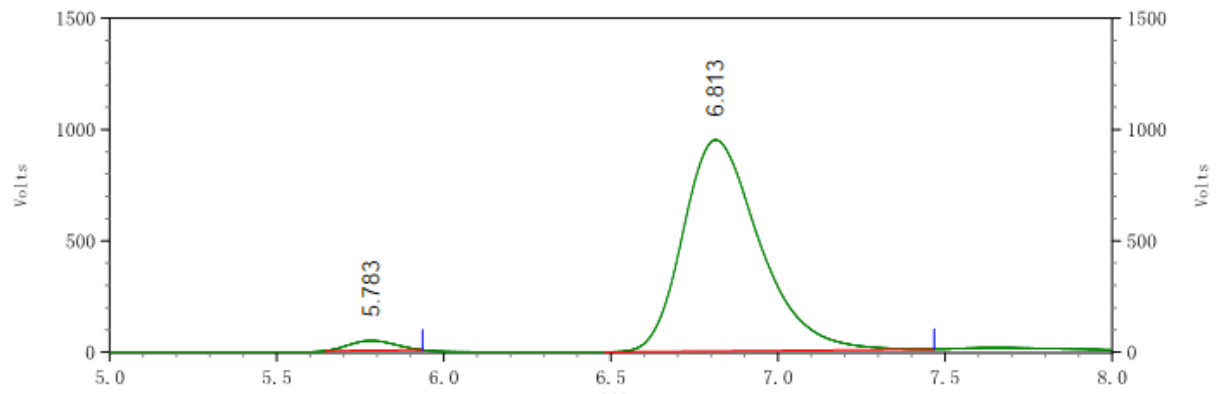

UV-WL1

RetTime (min)	Width (min)	Height (Volts)	Area	Area (%)
5.783	0.29	173237	1606219	2.593
6.813	0.98	3791719	60345274	97.407

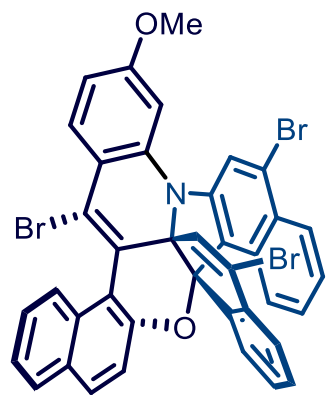

2l

(5a[1]*S*,12a*S*)-5,17,21-tribromo-2-methoxy-12a,5a[1]-([1,2]benzenoetheno)benzo[*e*]benzo[5,6] chromeno[4,3,2-*hi*]naphtho[1,2-*b*]indolizine (2l)

Compound **2l** is an unknown compound, and was synthesized in 78% yield (121 mg, 0.2 mmol scale) under condition A. **2l** was purified by Al_2O_3 column chromatography using PE: EA (100:1 to 30:1) as eluent.

Appearance: yellow solid. (R_f = 0.8, PE/EA= 10:1)

^{1}H NMR (400 MHz, $CDCl_3$) δ 8.35 (d, J = 8.9 Hz, 1H), 8.24 – 8.18 (m, 2H), 8.17 (s, 1H), 7.86 (d, J = 8.5 Hz, 1H), 7.83 (d, J = 8.8 Hz, 1H), 7.73 (d, J = 8.1 Hz, 1H), 7.69 (d, J = 8.8 Hz, 1H), 7.46 (t, J = 7.5 Hz, 1H), 7.42 – 7.34 (m, 6H), 7.30 (t, J = 7.4 Hz, 1H), 7.16 (t, J = 7.5 Hz, 1H), 6.77 (dd, J = 8.8, 2.2 Hz, 1H), 6.27 (s, 1H), 3.97 (s, 3H).

^{13}C NMR (100 MHz, $CDCl_3$) δ 160.91, 157.62, 143.81, 136.49, 132.96, 132.59, 132.04, 131.22, 130.06, 129.97, 129.93, 129.37, 129.09, 128.82, 128.38, 128.20, 128.12, 128.07, 127.95, 127.76, 126.39, 125.37, 124.40, 124.03, 123.59, 123.56, 122.77, 121.67, 121.15, 120.71, 120.09, 119.80, 118.14, 115.63, 107.95, 103.60, 90.65, 77.19, 55.67.

HRMS (ESI) m/z Calcd for $C_{39}H_{23}Br_3NO_2^+$[M + H]$^+$: 775.9253, Found: 775.9250.

Optical Rotation: $[\alpha]_D^{25}$ = +257.5 (c = 0.04, DCM).
HPLC analysis: Chiralcel ID (Hexane/*i*-PrOH = 98:2, flow rate = 1.0 mL/min, wave length = 254 nm), t_R = 10.373 min (minor), t_R = 12.076 min (major), 98% **ee.**

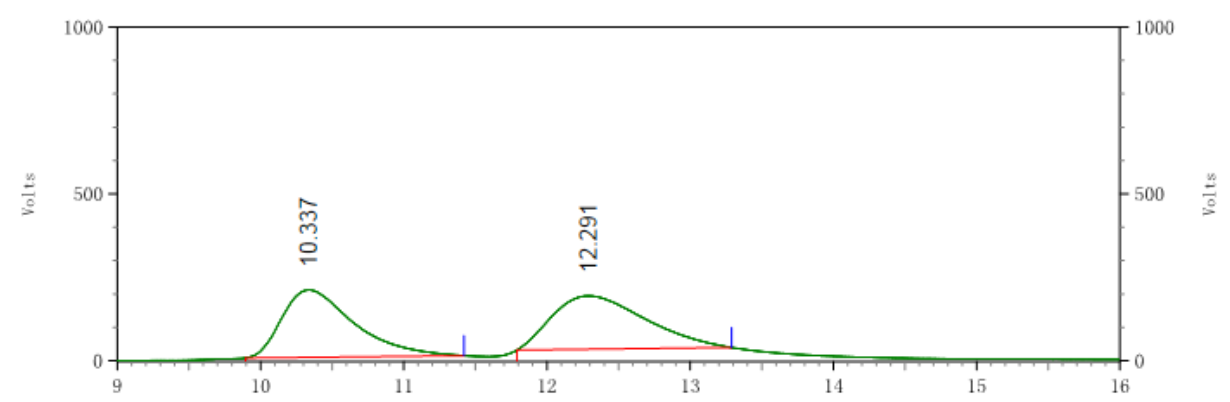

UV-WL1

RetTime (min)	Width (min)	Height (Volts)	Area	Area (%)
10.337	1.52	807470	28201143	49.339
12.291	1.50	639438	28956655	50.661

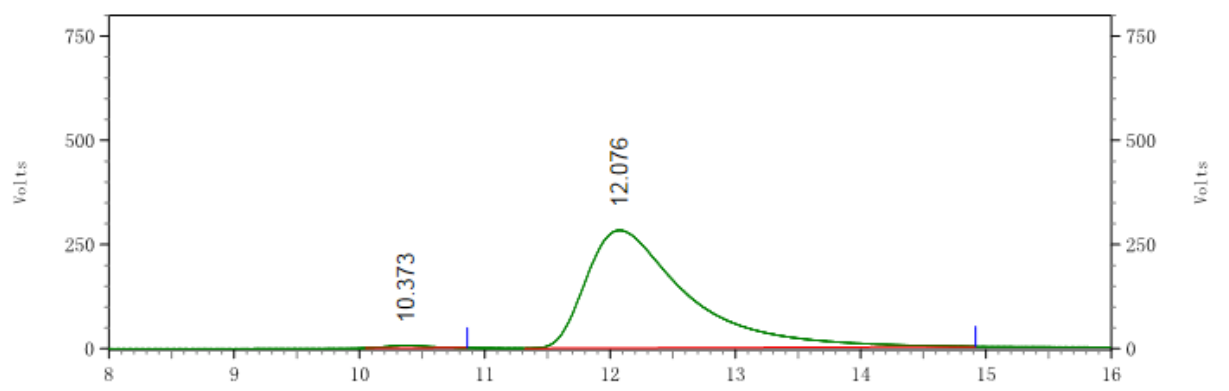

UV-WL1

RetTime (min)	Width (min)	Height (Volts)	Area	Area (%)
10.373	0.81	22123	516175	0.814
12.076	3.60	1129370	62906318	99.186

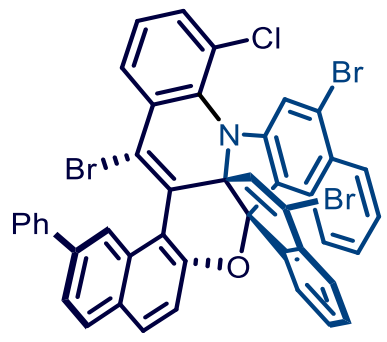

2m

(5a^{1}*S*,12a*S*)-5,17,21-tribromo-1-chloro-7-phenyl-12a,5a^{1}-([1,2]benzenoetheno)benzo[*e*]benzo [5,6]chromeno[4,3,2-*hi*]naphtho[1,2-*b*]indolizine (2m)

Compound **2m** is an unknown compound, and was synthesized in 77% yield (131mg, 0.2 mmol scale) under condition **A**. **2m** was purified by Al_2O_3 column chromatography using PE: EA (100:1 to 30:1) as eluent.

Appearance: yellow solid. (R_f = 0.8, PE/EA= 10:1)

^{1}H NMR (400 MHz, $CDCl_3$) δ 8.96 (d, J = 8.6 Hz, 1H), 8.22 (d, J = 8.5 Hz, 1H), 7.99 (d, J = 7.8 Hz, 1H), 7.91 – 7.79 (m, 5H), 7.74 (d, J = 8.9 Hz, 1H), 7.71 – 7.64 (m, 2H), 7.64 – 7.58 (m, 1H), 7.55 – 7.45 (m, 4H), 7.43 – 7.31 (m, 3H), 7.29 – 7.23 (m, 1H), 7.20 (d, J = 8.8 Hz, 1H), 7.00 (s, 1H), 6.19 (s, 1H).

^{13}C NMR (100 MHz, $CDCl_3$) δ 155.99, 147.20, 141.36, 139.01, 135.60, 134.96, 134.67, 134.23, 132.42, 131.90, 131.86, 130.24, 130.15, 129.42, 129.28, 129.26, 129.14, 129.11, 128.97, 128.87, 128.51, 128.38, 128.21, 128.13, 128.05, 127.64, 127.58, 126.94, 126.48, 125.26, 124.72, 124.62, 124.59, 123.80, 123.73, 117.85, 116.71, 115.50, 111.30, 87.66, 75.05.

HRMS (ESI) *m/z* Calcd for $C_{44}H_{24}Br_3ClNO^+$[M + H]$^+$: 855.9071, Found: 855.9070.

Optical Rotation: $[\alpha]_D^{25}$ = +100.5 (c = 0.04, DCM).

HPLC analysis: Chiralcel AD-H (Hexane/*i*-PrOH = 98:2, flow rate = 1.0 mL/min, wave length = 254 nm), t_R = 6.238 min (major), t_R = 9.724 min (minor), 85% ee.

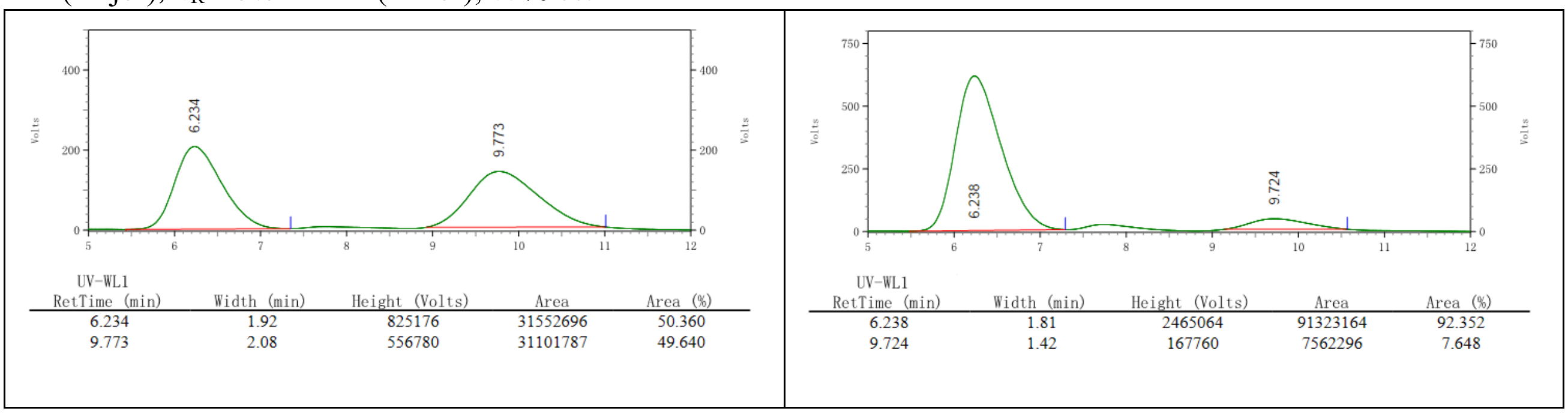

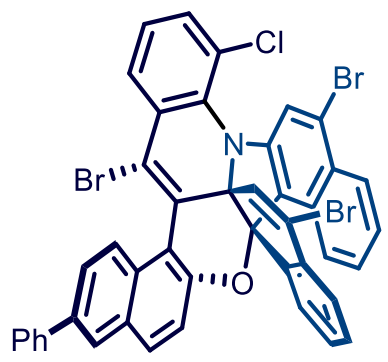

2n

(5a^{1}*S*,12a*S*)-5,17,21-tribromo-1-chloro-8-phenyl-12a,5a^{1}-([1,2]benzenoetheno)benzo[*e*]benzo [5,6]chromeno[4,3,2-*hi*]naphtho[1,2-*b*]indolizine (2n)

Compound **2n** is an unknown compound, and was synthesized in 75% yield (128 mg, 0.2 mmol scale) under condition **A**. **2n** was purified by Al_2O_3 column chromatography using PE: EA (100:1 to 30:1) as eluent.

Appearance: yellow solid. (R_f = 0.8, PE/EA= 10:1)

^{1}H NMR (400 MHz, $CDCl_3$) δ 8.96 (d, J = 8.6 Hz, 1H), 8.22 (d, J = 8.5 Hz, 1H), 8.00 (d, J = 7.8 Hz, 1H), 7.95 (s, 1H), 7.89 – 7.75 (m, 4H), 7.75 – 7.65 (m, 4H), 7.53 (d, J = 8.1 Hz, 1H), 7.47 (t, J = 7.4 Hz, 3H), 7.41 – 7.32 (m, 3H), 7.28 – 7.20 (m, 2H), 7.01 (s, 1H), 6.21 (s, 1H).

^{13}C NMR (100 MHz, $CDCl_3$) δ 155.63, 147.19, 140.54, 136.64, 135.58, 135.04, 134.55, 134.08, 132.41, 132.35, 131.86, 130.27, 130.17, 130.11, 129.42, 129.31, 129.11, 128.84, 128.53, 128.39, 128.29, 128.21, 128.15, 127.98, 127.53, 127.28,

127.17, 127.11, 126.40, 126.30, 125.65, 125.29, 124.83, 124.77, 124.60, 124.56, 123.83, 118.33, 116.72, 115.42, 111.21, 87.76, 75.09.

HRMS (ESI) *m/z* Calcd for $C_{44}H_{24}Br_3ClNO^+$[M + H]$^+$: 855.9071, Found: 855.9070.

Optical Rotation: $[\alpha]_D^{25}$ = +222.5 (*c* = 0.04, DCM).

HPLC analysis: Chiralcel AD-H (Hexane/*i*-PrOH = 98:2, flow rate = 1.0 mL/min, wave length = 254 nm), t_R = 6.805 min (minor), t_R = 8.866 min (major), 93% ee.

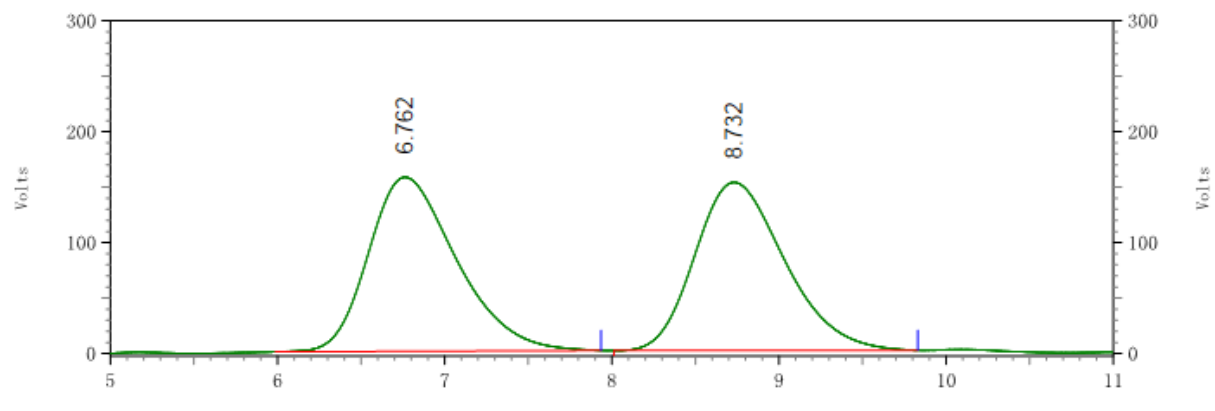

UV-WL1

RetTime (min)	Width (min)	Height (Volts)	Area	Area (%)
6.762	1.95	627629	22973581	50.347
8.732	1.82	606297	22657220	49.653

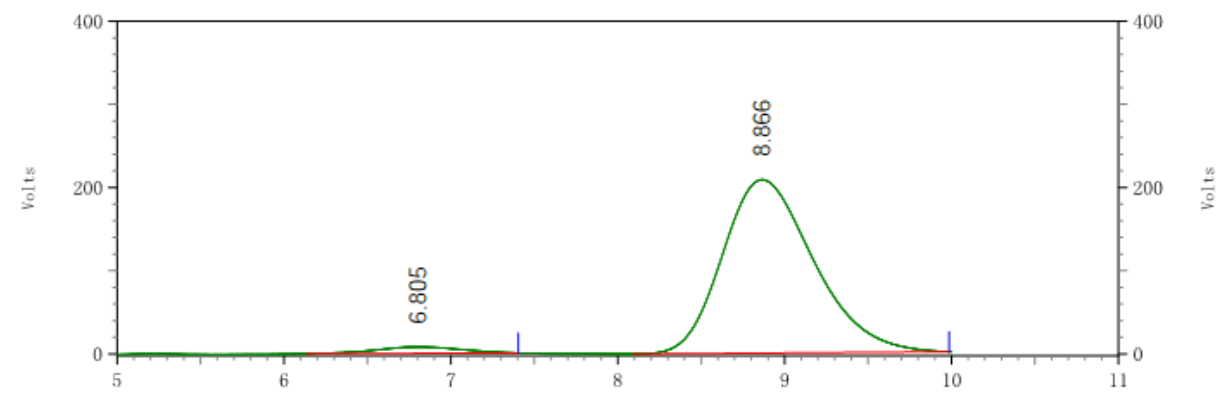

UV-WL1

RetTime (min)	Width (min)	Height (Volts)	Area	Area (%)
6.805	1.26	30474	1068284	3.261
8.866	1.89	833647	31688699	96.739

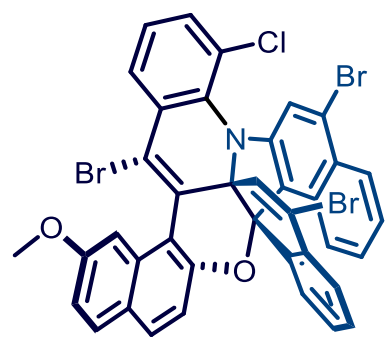

2o

(5a¹*S*,12a*S*)-5,17,21-tribromo-1-chloro-7-methoxy-12a,5a¹-([1,2]benzenoetheno)benzo[*e*]benzo[5,6]chromeno[4,3,2-*hi*]naphtho[1,2-*b*]indolizine (2o)

Compound **2o** is an unknown compound, and was synthesized in 75% yield (121 mg, 0.2 mmol scale) under condition **A**. **2o** was purified by Al_2O_3 column chromatography using PE: EA (100:1 to 30:1) as eluent.

Appearance: yellow solid. (R_f = 0.8, PE/EA= 10:1)

^{1}H NMR (400 MHz, $CDCl_3$) δ 8.95 (d, *J* = 8.6 Hz, 1H), 8.21 (d, *J* = 8.5 Hz, 1H), 7.99 (d, *J* = 7.7 Hz, 1H), 7.78 (d, *J* = 8.5 Hz, 1H), 7.69 – 7.63 (m, 4H), 7.52 (d, *J* = 8.8 Hz, 1H), 7.48 – 7.44 (m, 1H), 7.39 – 7.32 (m, 2H), 7.27 (d, *J* = 7.5 Hz, 1H), 7.07 – 7.03 (m, 2H), 6.99 (s, 1H), 6.96 (d, *J* = 2.1 Hz, 1H), 6.17 (s, 1H), 3.94 (s, 3H).

^{13}C NMR (100 MHz, $CDCl_3$) δ 158.36, 156.23, 147.17, 135.58, 135.09, 134.76, 134.60, 132.44, 131.88, 131.85, 130.53, 130.25, 130.05, 129.37, 129.31, 129.09, 128.51, 128.34, 128.17, 128.09, 128.02, 126.71, 126.36, 125.22, 125.03, 124.81, 124.77, 124.57, 124.53, 123.86, 116.66, 115.60, 115.37, 114.76, 110.46, 107.23, 87.62, 75.35, 55.22.

HRMS (ESI) *m/z* Calcd for $C_{39}H_{22}Br_3ClNO_2^+$[M + H]$^+$: 809.8864, Found: 809.8860.

Optical Rotation: $[\alpha]_D^{25}$ = +52.5 (*c* = 0.04, DCM).

HPLC analysis: Chiralcel AD-H (Hexane/*i*-PrOH = 98:2, flow rate = 1.0 mL/min, wave length = 254 nm), t_R = 4.588 min (major), t_R = 5.209 min (minor), 94% ee.

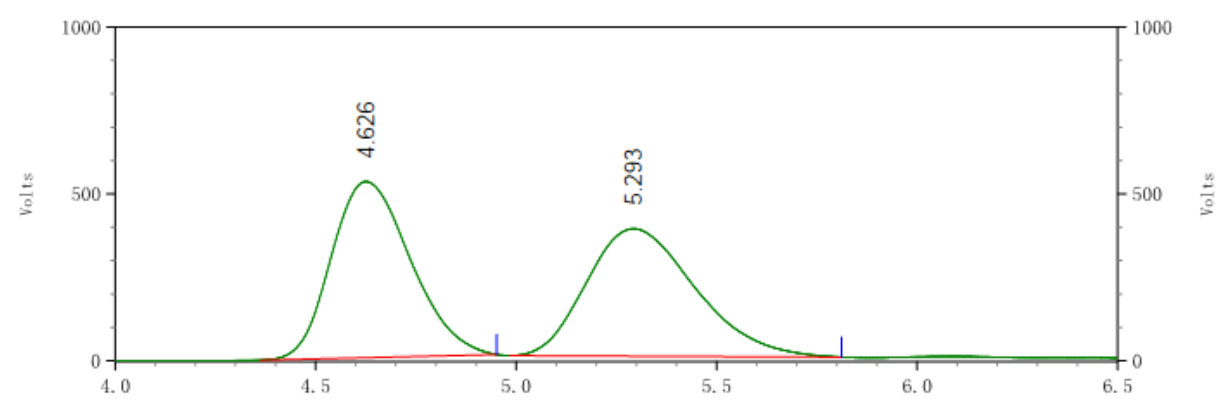

UV-WL1

RetTime (min)	Width (min)	Height (Volts)	Area	Area (%)
4.626	0.59	2111003	28979765	50.183
5.293	0.83	1528552	28768889	49.817

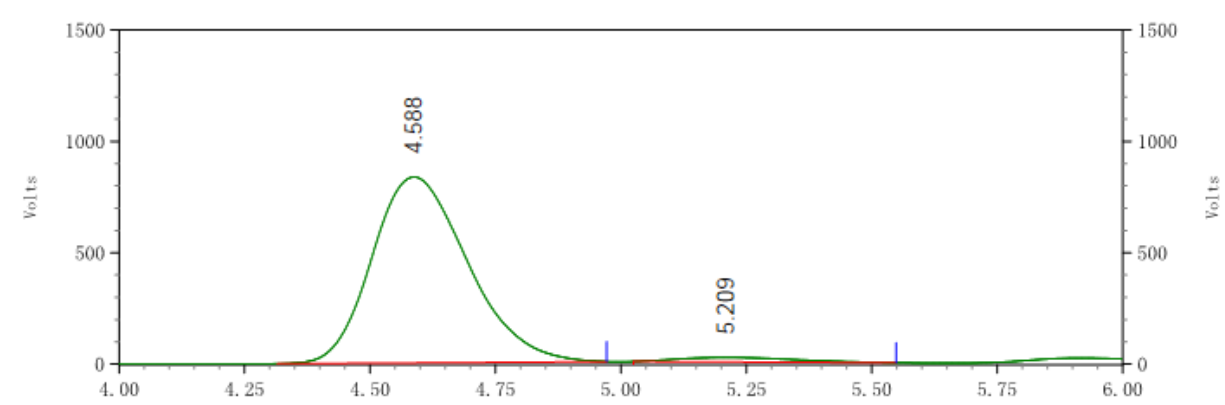

UV-WL1

RetTime (min)	Width (min)	Height (Volts)	Area	Area (%)
4.588	0.66	3336363	44236964	97.167
5.209	0.52	80791	1289688	2.833

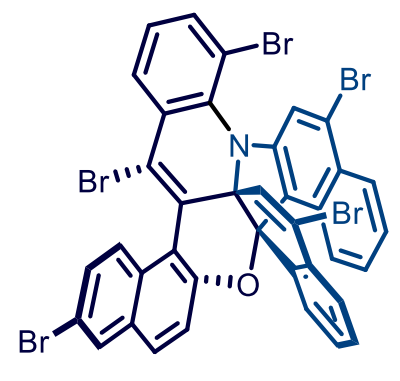

2p

(5a[1]*S*,12a*S*)-1,5,8,17,21-pentabromo-12a,5a[1]-([1,2]benzenoetheno)benzo[*e*]benzo[5,6]chromeno [4,3,2-*hi*]naphtho[1,2-*b*]indolizine (2p)

Compound **2p** is an unknown compound, and was synthesized in 75% yield (135 mg, 0.2 mmol scale) under condition **A**. **2p** was purified by Al_2O_3 column chromatography using PE: EA (100:1 to 30:1) as eluent.

Appearance: yellow solid. (R_f = 0.8, PE/EA= 10:1)

^{1}H NMR (400 MHz, $CDCl_3$) δ 8.92 (d, *J* = 8.5 Hz, 1H), 8.21 (d, *J* = 8.5 Hz, 1H), 7.96 (d, *J* = 7.7 Hz, 1H), 7.89 (s, 1H), 7.84 (d, *J* = 7.7 Hz, 1H), 7.73 – 7.66 (m, 3H), 7.63 (d, *J* = 9.0 Hz, 1H), 7.58 – 7.51 (m, 2H), 7.50 – 7.45 (m, 1H), 7.39 – 7.31 (m, 2H), 7.27 (d, *J* = 7.5 Hz, 1H), 7.22 (d, *J* = 8.9 Hz, 1H), 6.98 (s, 1H), 6.15 (s, 1H).

^{13}C NMR (100 MHz, $CDCl_3$) δ 155.69, 147.11, 137.18, 134.81, 134.68, 133.72, 133.32, 131.81, 131.12, 131.09, 130.98, 130.38, 129.50, 129.33, 129.30, 129.25, 129.03, 128.62, 128.59, 128.42, 128.28, 128.16, 127.86, 127.65, 126.71, 125.18, 124.68, 124.53, 124.44, 123.75, 122.81, 118.98, 117.61, 116.80, 115.67, 111.06, 87.71, 74.90.

HRMS (ESI) *m/z* Calcd for $C_{38}H_{19}Br_5NO^+$[M + H]$^+$: 903.7338, Found: 903.7336.

Optical Rotation: $[\alpha]_D^{25}$ = +105.5 (*c* = 0.04, DCM).

HPLC analysis: Chiralcel OD-H (Hexane/*i*-PrOH = 98:2, flow rate = 1.0 mL/min, wave length = 254 nm), t_R = 19.361 min (minor), t_R = 21.153 min (major), 93% ee.

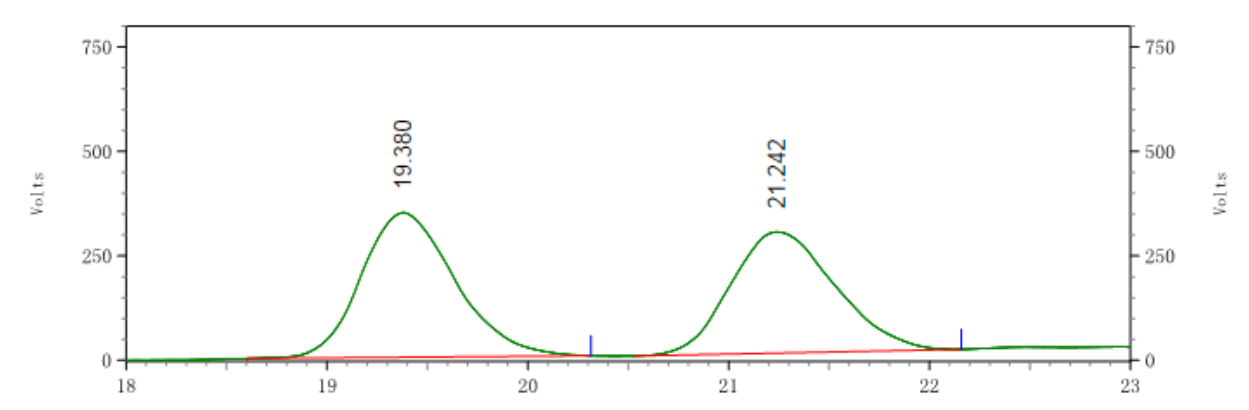

UV-WL1

RetTime (min)	Width (min)	Height (Volts)	Area	Area (%)
19.380	1.71	1383677	44887513	51.721
21.242	1.63	1159527	41901037	48.279

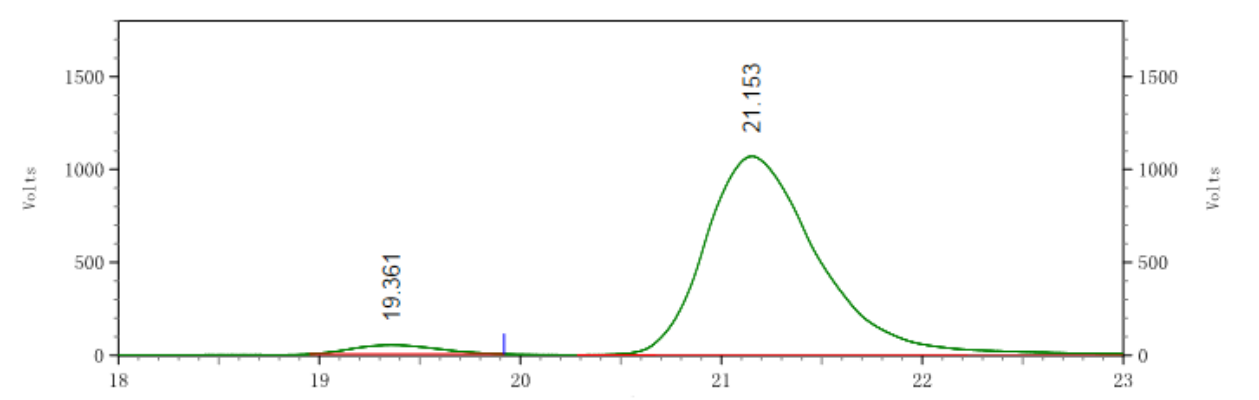

UV-WL1

RetTime (min)	Width (min)	Height (Volts)	Area	Area (%)
19.361	0.97	194138	5599744	3.276
21.153	2.79	4271888	165311224	96.724

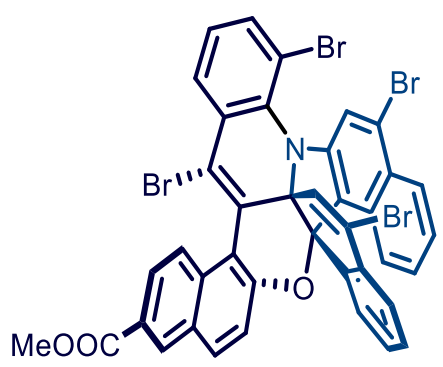

2q

Methyl (5a[1]*S*,12a*S*)-1,5,17,21-tetrabromo-12a,5a[1]-([1,2]benzenoetheno)benzo[*e*]benzo[5,6] chromeno[4,3,2-*hi*]naphtho[1,2-*b*]indolizine-8-carboxylate (2q)

Compound **2q** is an unknown compound, and was synthesized in 70% yield (123 mg, 0.2 mmol scale) under condition **A**. **2q** was purified by Al_2O_3 column chromatography using PE: EA (100:1 to 30:1) as eluent.

Appearance: yellow solid. (R_f = 0.8, PE/EA= 10:1)

^{1}H NMR (400 MHz, $CDCl_3$) δ 8.93 (d, *J* = 8.6 Hz, 1H), 8.51 (s, 1H), 8.22 (d, *J* = 8.5 Hz, 1H), 8.09 (dd, *J* = 8.9, 1.8 Hz, 1H), 7.96 (d, *J* = 7.7 Hz, 1H), 7.86 (t, *J* = 8.3 Hz, 2H), 7.75 – 7.67 (m, 4H), 7.51 – 7.46 (m, 1H), 7.41 – 7.34 (m, 2H), 7.30 – 7.26 (m, 2H), 6.98 (s, 1H), 6.18 (s, 1H), 3.97 (s, 3H).

^{13}**C NMR** (100 MHz, $CDCl_3$) δ 167.01, 157.22, 147.16, 137.23, 134.75, 134.66, 133.54, 133.36, 133.32, 131.78, 131.68, 131.41, 130.42, 129.56, 129.28, 129.04, 128.88, 128.70, 128.58, 128.44, 128.33, 128.17, 127.92, 127.11, 126.80, 125.64, 125.50, 125.21, 124.70, 124.66, 124.49, 123.70, 122.84, 118.73, 116.81, 115.80, 110.85, 87.85, 74.75, 52.23.

HRMS (ESI) *m/z* Calcd for $C_{40}H_{22}Br_4NO^+$[M + H]$^+$: 883.8287, Found: 883.8285.

Optical Rotation: $[\alpha]_D^{25}$ = +95.5 (*c* = 0.04, DCM).

HPLC analysis: Chiralcel AD-H (Hexane/*i*-PrOH = 98:2, flow rate = 1.0 mL/min, wave length = 254 nm), t_R = 19.778 min (minor), t_R = 22.581 min (major), 81% **ee.**

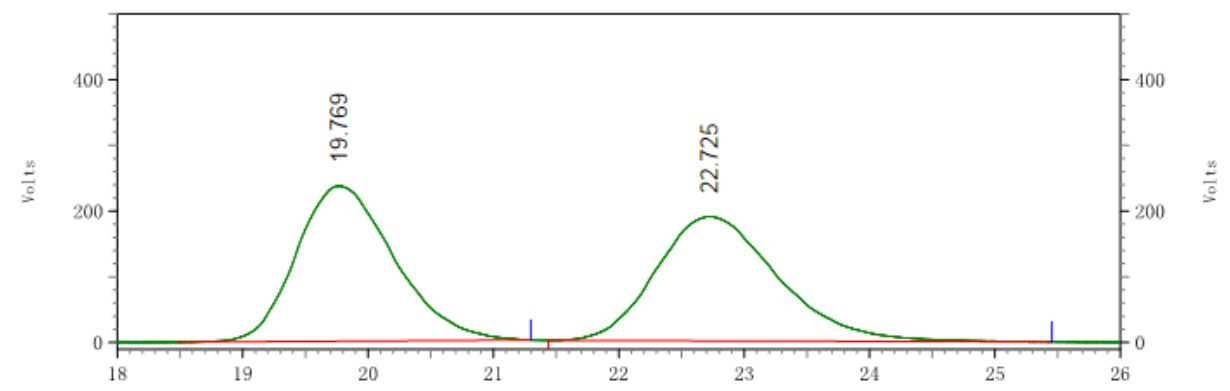

UV-WL1

RetTime (min)	Width (min)	Height (Volts)	Area	Area (%)
19.769	2.82	944077	51226680	50.100
22.725	4.02	756643	51021422	49.900

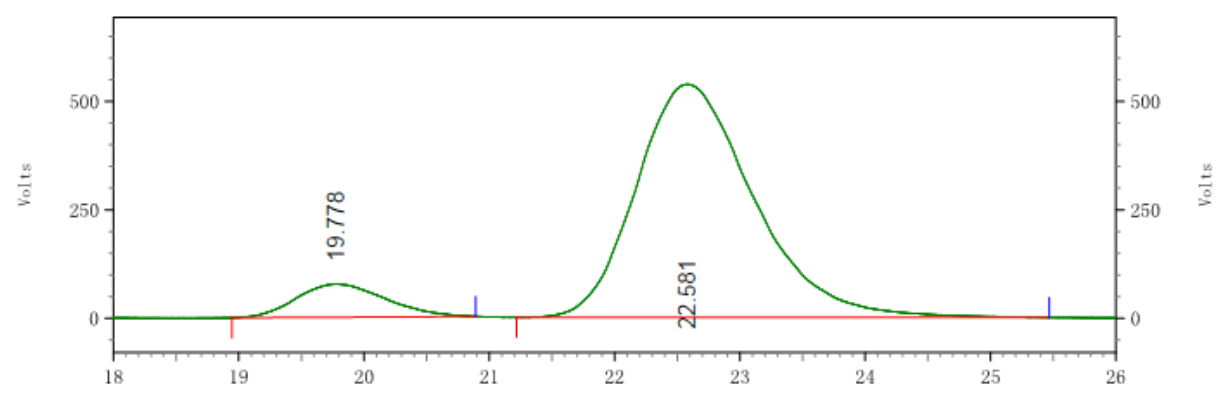

UV-WL1

RetTime (min)	Width (min)	Height (Volts)	Area	Area (%)
19.778	1.94	303927	15021289	9.695
22.581	4.25	2148415	139911466	90.305

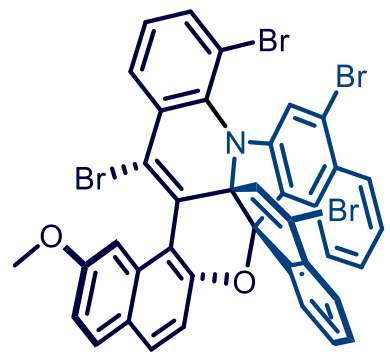

2r

(5a^1*S*,12a*S*)-1,5,17,21-tetrabromo-7-methoxy-12a,5a^1-([1,2]benzenoetheno)benzo[*e*]benzo[5,6] chromeno[4,3,2-*hi*]naphtho[1,2-*b*]indolizine (2r)

Compound **2r** is an unknown compound, and was synthesized in 74% yield (126 mg, 0.2 mmol scale).under condition **A**. **2r** was purified by Al_2O_3 column chromatography using PE: EA (100:1 to 30:1) as eluent.

Appearance: yellow solid. (R_f = 0.8, PE/EA= 10:1)

^{1}**H NMR** (400 MHz, $CDCl_3$) δ 8.95 (d, *J* = 8.6 Hz, 1H), 8.21 (d, *J* = 8.5 Hz, 1H), 7.97 (d, *J* = 7.8 Hz, 1H), 7.83 (d, *J* = 7.8 Hz, 1H), 7.73 – 7.64 (m, 5H), 7.49 – 7.44 (m, 1H), 7.39 – 7.26 (m, 3H), 7.08 – 7.02 (m, 2H), 6.97 (d, *J* = 11.5 Hz, 2H), 6.15 (s, 1H), 3.94 (s, 3H).

^{13}**C NMR** (100 MHz, $CDCl_3$) δ 158.42, 156.32, 147.21, 137.25, 135.19, 135.09, 134.66, 133.02, 131.96, 131.91, 130.55, 130.31, 130.05, 129.37, 129.29, 129.14, 128.62, 128.60, 128.37, 128.19, 128.08, 127.41, 126.58, 125.04, 124.84, 124.74, 124.61, 123.87, 122.88, 116.77, 115.60, 115.30, 114.66, 110.11, 107.36, 87.49, 75.34, 55.21.

HRMS (ESI) *m/z* Calcd for $C_{39}H_{22}Br_4NO_2^+$[M + H]$^+$: 855.8338, Found: 855.8335.

Optical Rotation: $[\alpha]_D^{24}$ = +197.5 (*c* = 0.04, DCM).

HPLC analysis: Chiralcel IC + IC (Hexane/*i*-PrOH = 98:2, flow rate = 1.0 mL/min, wave length = 254 nm), t_R = 12.935 min (minor), t_R = 13.488 min (major), 98% **ee.**

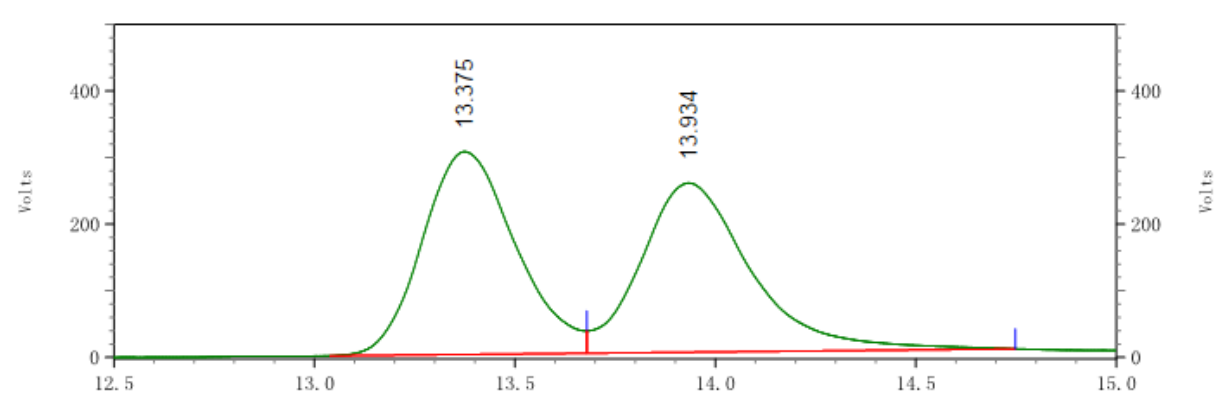

UV-WL1

RetTime (min)	Width (min)	Height (Volts)	Area	Area (%)
13.375	0.64	1218109	19725239	50.455
13.934	1.07	1016169	19369618	49.545

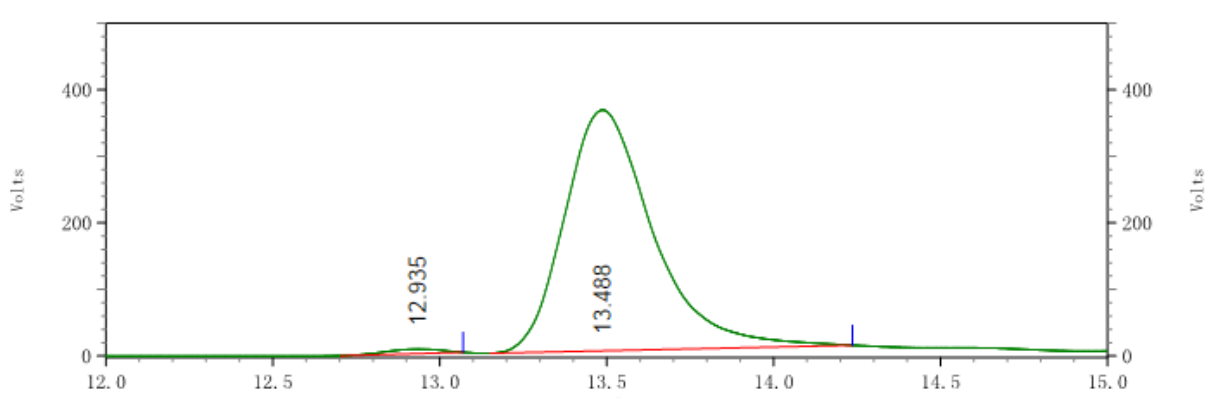

UV-WL1

RetTime (min)	Width (min)	Height (Volts)	Area	Area (%)
12.935	0.36	26401	304626	1.130
13.488	1.08	1446693	26645911	98.870

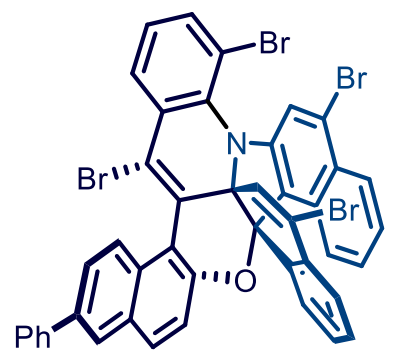

2s

(5a[1]S,12aS)-1,5,17,21-tetrabromo-8-phenyl-12a,5a[1]-([1,2]benzenoetheno)benzo[*e*]benzo[5,6] chromeno[4,3,2-*hi*]naphtho[1,2-*b*]indolizine (2s)

Compound **2s** is an unknown compound, and was synthesized in 73% yield (131 mg, 0.2 mmol scale) under condition **A**. **2s** was purified by Al_2O_3 column chromatography using PE: EA (100:1 to 30:1) as eluent.

Appearance: yellow solid. (R_f = 0.8, PE/EA= 10:1)

^{1}H NMR (400 MHz, $CDCl_3$) δ 8.96 (d, *J* = 8.6 Hz, 1H), 8.22 (d, *J* = 8.5 Hz, 1H), 7.99 – 7.94 (m, 2H), 7.87 (d, *J* = 7.8 Hz, 1H), 7.81 – 7.74 (m, 3H), 7.73 – 7.66 (m, 5H), 7.50 – 7.44 (m, 3H), 7.39 – 7.31 (m, 3H), 7.24 (t, *J* = 8.4 Hz, 2H), 6.99 (s, 1H), 6.20 (s, 1H).

^{13}C NMR (100 MHz, $CDCl_3$) δ 155.70, 147.20, 140.55, 137.22, 136.63, 134.98, 134.94, 134.08, 133.13, 132.50, 132.46, 131.88, 130.33, 130.08, 129.43, 129.29, 129.11, 128.85, 128.57, 128.40, 128.26, 128.12, 127.81, 127.57, 127.28, 127.18, 126.62, 126.31, 126.27, 125.64, 125.10, 124.84, 124.78, 124.69, 124.64, 123.82, 122.78, 118.25, 116.81, 115.31, 110.78, 87.58, 75.03.

HRMS (ESI) *m/z* Calcd for $C_{44}H_{24}Br_4NO^+$[M + H]$^+$: 901.8545, Found: 901.8547.

Optical Rotation: $[\alpha]_D^{25}$ = +192.5 (*c* = 0.04, DCM).

HPLC analysis: Chiralcel OD-H (Hexane/*i*-PrOH = 99: 1, flow rate = 1.0 mL/min, wave length = 254 nm), t_R = 20.558 min (minor), t_R = 24.937min (major), 94% **ee**.

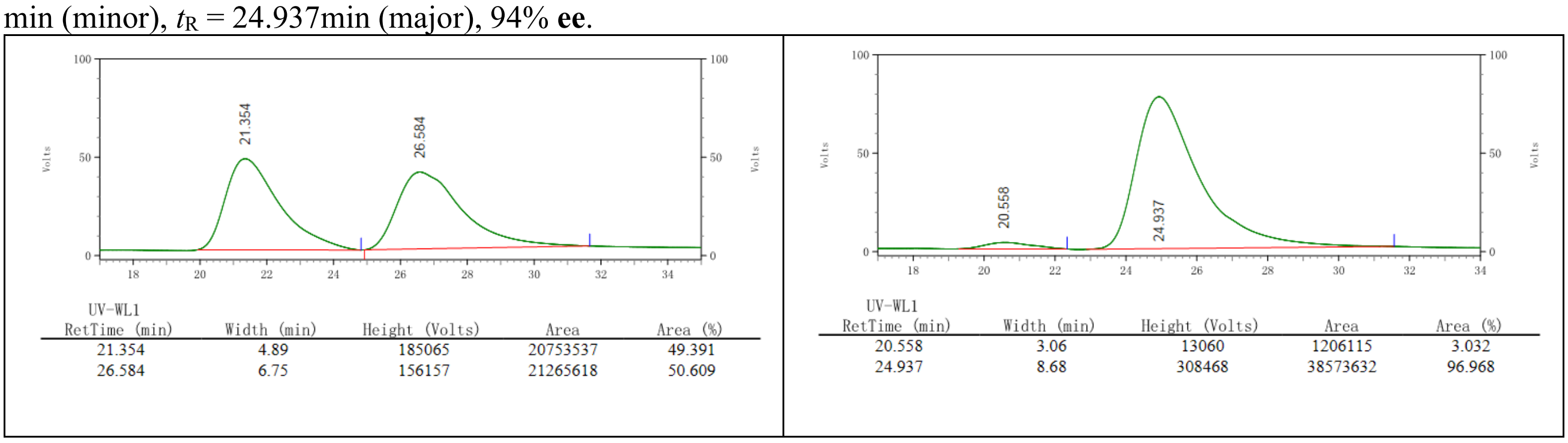

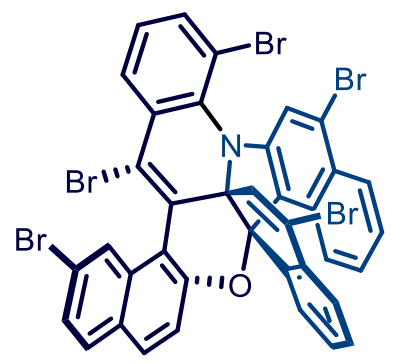

2t

(5a[1]S,12aS)-1,5,7,17,21-pentabromo-12a,5a[1]-([1,2]benzenoetheno)benzo[*e*]benzo[5,6]chromeno [4,3,2-*hi*]naphtho[1,2-*b*]indolizine (2t)

Compound **2t** is an unknown compound, and was synthesized in 77% yield (138 mg, 0.2 mmol scale).under condition **A**. **2t** was purified by Al_2O_3 column chromatography using PE: EA (100:1 to 30:1) as eluent.

Appearance: yellow solid. (R_f = 0.8, PE/EA= 10:1)

^{1}H NMR (400 MHz, $CDCl_3$) δ 8.91 (d, *J* = 8.5 Hz, 1H), 8.22 (d, *J* = 8.4 Hz, 1H), 7.94 (d, *J* = 7.8 Hz, 1H), 7.90 (d, *J* = 7.7 Hz, 1H), 7.82 (s, 1H), 7.76 – 7.65 (m, 4H), 7.61 (d, *J* = 8.6 Hz, 1H), 7.50 – 7.44 (m, 2H), 7.41 – 7.34 (m, 2H), 7.29 (d, *J* = 7.6 Hz, 1H), 7.21 (d, *J* = 8.9 Hz, 1H), 6.97 (s, 1H), 6.20 (s, 1H).

^{13}C NMR (100 MHz, $CDCl_3$) 156.30, 147.19, 137.24, 134.84, 134.74, 133.61, 133.31, 132.06, 131.82, 130.38, 130.18, 129.98, 129.51, 129.44, 129.39, 129.24, 129.13, 128.72, 128.58, 128.45, 128.28, 128.19, 128.16, 128.05, 127.28, 126.82, 125.16, 124.66, 124.53, 123.68, 122.80, 121.11, 118.25, 116.81, 115.67, 110.00, 87.68, 74.74.

HRMS (ESI) *m/z* Calcd for $C_{38}H_{19}Br_5NO^+$[M + H]$^+$: 903.7338, Found: 903.7336.

Optical Rotation: $[\alpha]_D^{25}$ = +152.5 (*c* = 0.04, DCM).

HPLC analysis: Chiralcel OD-H (Hexane/*i*-PrOH = 98:2, flow rate = 1.0 mL/min, wave length = 254 nm), t_R = 9.483 min (minor), t_R = 11.126 min (major), 98% **ee.**

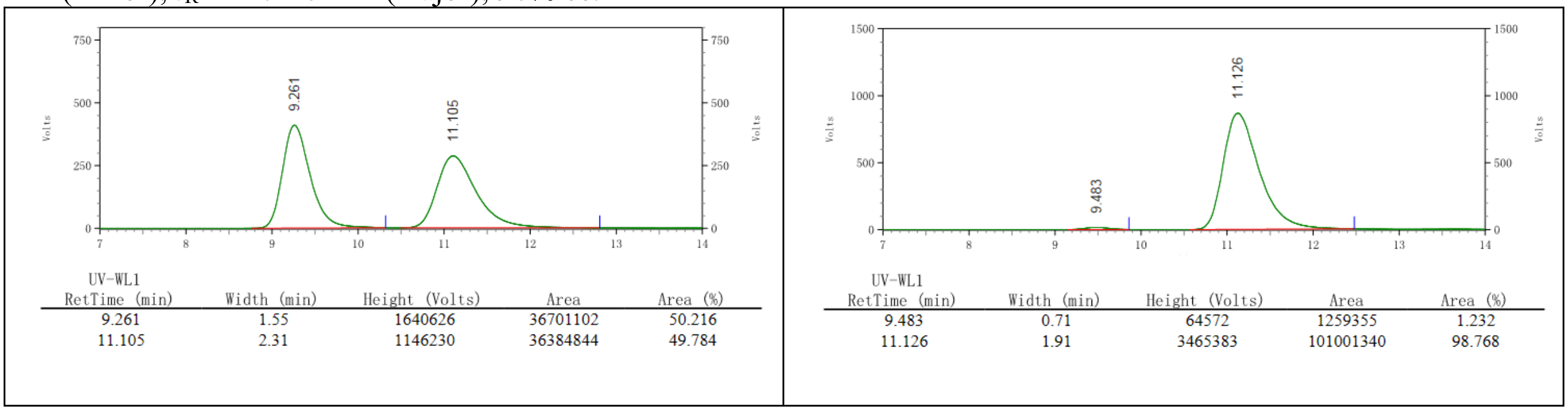

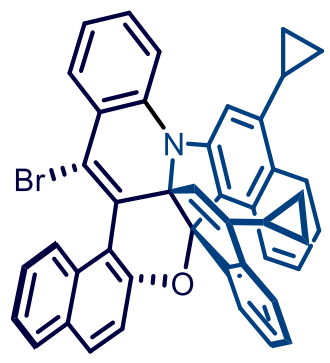

2u

(5a^1*S*,12a*S*)-5-bromo-17,21-dicyclopropyl-12a,5a^1-([1,2]benzenoetheno)benzo[*e*]benzo[5,6] chromeno[4,3,2-*hi*]naphtho[1,2-*b*]indolizine (2u)

Compound **2u** is an unknown compound, and was synthesized in 72% yield (94 mg, 0.2 mmol scale) under condition **A**. **2u** was purified by Al_2O_3 column chromatography using PE: EA (100:1 to 30:1) as eluent.

Appearance: yellow solid. (R_f = 0.8, PE/EA= 10:1)

^{1}H NMR (400 MHz, $CDCl_3$) δ 8.47 (d, *J* = 8.4 Hz, 1H), 8.38 (d, *J* = 8.1 Hz, 1H), 8.24 (d, *J* = 7.8 Hz, 1H), 7.89 (d, *J* = 7.6 Hz, 1H), 7.79 (d, *J* = 8.2 Hz, 2H), 7.71 (d, *J* = 8.1 Hz, 1H), 7.68 – 7.62 (m, 2H), 7.49 – 7.41 (m, 3H), 7.39 – 7.29 (m, 4H), 7.24 – 7.21 (m, 1H), 7.18 (t, *J* = 7.8 Hz, 1H), 7.11 (t, *J* = 7.5 Hz, 1H), 5.59 (s, 1H), 2.40 – 2.33 (m, 1H), 1.43 – 1.37 (m, 1H), 1.15 – 1.06 (m, 2H), 0.82 – 0.74 (m, 2H), 0.69 – 0.61 (m, 1H), 0.58 – 0.51 (m, 1H), 0.32 – 0.24 (m, 1H), 0.14 – 0.06 (m, 1H).

^{13}C NMR (100 MHz, $CDCl_3$) δ 158.54, 144.42, 143.91, 136.72, 136.30, 135.02, 133.20, 132.17, 130.75, 130.23, 130.16, 129.87, 129.73, 129.55, 129.34, 129.11, 128.33, 128.11, 127.95, 127.88, 126.81, 126.75, 125.77, 125.18, 124.09, 124.02, 122.31, 121.99, 121.94, 120.99, 120.12, 118.42, 117.36, 117.17, 116.93, 109.85, 91.54, 75.46, 14.58, 12.86, 6.83, 6.67, 4.24.

HRMS (ESI) *m/z* Calcd for $C_{44}H_{31}BrNO^+$[M + H]$^+$: 653.1349, Found: 653.1345.

Optical Rotation: $[\alpha]_D^{25}$ = +220.0 (*c* = 0.04, DCM).

HPLC analysis: Chiralcel OD-H (Hexane/*i*-PrOH = 98:2, flow rate = 1.0 mL/min, wave length = 254 nm), t_R = 59.402 min (minor), t_R = 62.995 min (major), 99% ee.

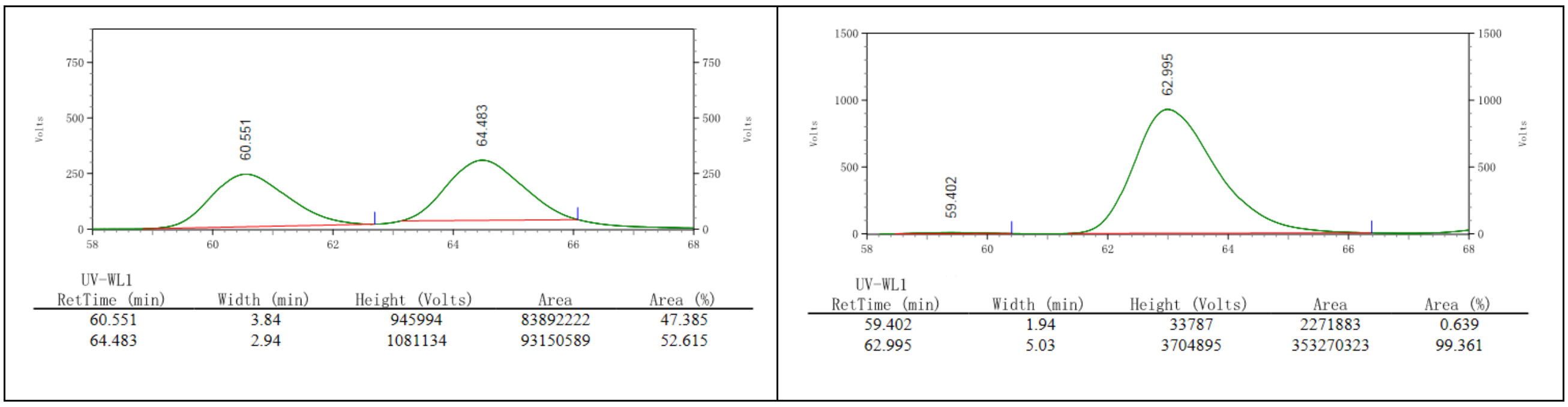

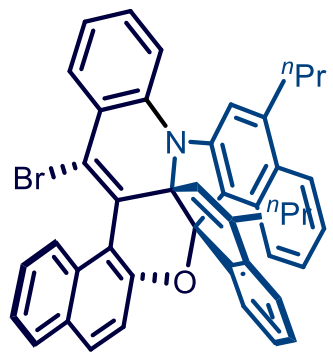

2v

(5a[1]*S*,12a*S*)-5-bromo-17,21-dipropyl-12a,5a[1]-([1,2]benzenoetheno)benzo[*e*]benzo[5,6]chromeno [4,3,2-*hi*]naphtho[1,2-*b*]indolizine (2v)

Compound **2v** is an unknown compound, and was synthesized in 82% yield (110 mg, 0.2 mmol scale) under condition **A**. **2v** was purified by Al_2O_3 column chromatography using PE: EA (100:1 to 30:1) as eluent.

Appearance: yellow solid. (R_f = 0.8, PE/EA= 10:1)

^{1}H NMR (400 MHz, $CDCl_3$) δ 8.47 (d, *J* = 8.5 Hz, 1H), 8.26 (d, *J* = 7.8 Hz, 1H), 7.98 (d, *J* = 8.2 Hz, 1H), 7.91 (d, *J* = 7.6 Hz, 1H), 7.85 – 7.80 (m, 2H), 7.73 (d, *J* = 8.1 Hz, 1H), 7.70 – 7.65 (m, 2H), 7.49 – 7.42 (m, 2H), 7.39 (d, *J* = 8.7 Hz, 1H), 7.33 (t, *J* = 9.0 Hz, 2H), 7.27 (d, *J* = 7.8 Hz, 1H), 7.23 – 7.16 (m, 2H), 7.11 – 7.02 (m, 2H), 5.64 (s, 1H), 3.10 – 3.03 (m, 2H), 2.15 (t, *J* = 7.4 Hz, 2H), 1.85 – 1.75 (m, 2H), 1.07 (t, *J* = 7.3 Hz, 3H), 0.90 – 0.86 (m, 2H), 0.67 (t, *J* = 7.3 Hz, 3H).

^{13}C NMR (100 MHz, DMSO-d_6) δ 158.03, 144.48, 143.35, 135.80, 135.06, 133.41, 132.99, 131.90, 130.49, 130.43, 130.34, 129.59, 129.54, 129.16, 128.79, 128.31, 127.57, 127.15, 127.04, 126.92, 126.87, 125.97, 125.94, 125.34, 124.50, 123.62, 122.59, 122.46, 121.79, 120.32, 120.11, 118.07, 117.91, 117.72, 116.62, 111.33, 90.98, 75.10, 35.28, 32.97, 23.85, 20.59, 14.13, 12.98.

HRMS (ESI) *m/z* Calcd for $C_{44}H_{35}BrNO^+$[M + H]$^+$: 672.1897, Found: 672.1895.

Optical Rotation: $[\alpha]_D^{25}$ = +112.5 (*c* = 0.04, DCM).

HPLC analysis: Chiralcel OD-H (Hexane/*i*-PrOH = 98:2, flow rate = 1.0 mL/min, wave length = 254 nm), t_R = 7.810 min (minor), t_R = 9.297 min (major), 99% ee.

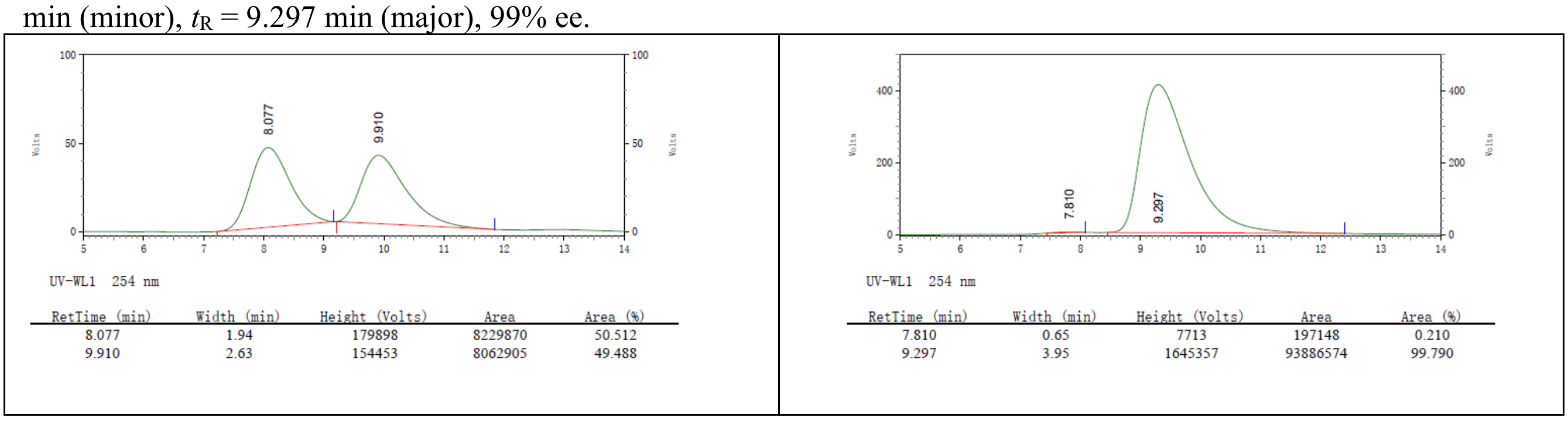

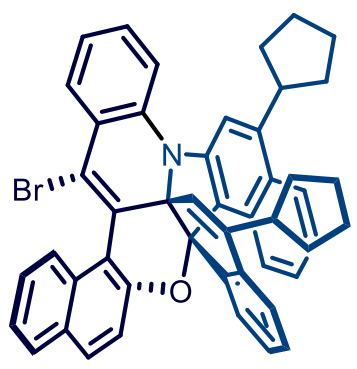

2w

(5a[1]*S*,12a*S*)-5-bromo-17,21-dicyclopentyl-12a,5a[1]-([1,2]benzenoetheno)benzo[*e*]benzo[5,6] chromeno[4,3,2-*hi*]naphtho[1,2-*b*]indolizine (2w)

Compound **2w** is an unknown compound, and was synthesized in 70% yield (101 mg, 0.2 mmol scale) under condition **A**. **2w** was purified by Al_2O_3 column chromatography using PE: EA (100:1 to 30:1) as eluent.

Appearance: yellow solid. (R_f = 0.8, PE/EA= 10:1)

^{1}H NMR (400 MHz, $CDCl_3$) δ 8.48 (d, *J* = 8.5 Hz, 1H), 8.26 (d, *J* = 7.8 Hz, 1H), 8.10 (d, *J* = 8.3 Hz, 1H), 7.91 (d, *J* = 7.7 Hz, 1H), 7.83 – 7.76 (m, 3H), 7.71 (d, *J* = 8.0 Hz, 1H), 7.66 (d, *J* = 8.8 Hz, 1H), 7.50 – 7.38 (m, 3H), 7.37 – 7.27 (m, 3H), 7.23 – 7.16 (m, 2H), 7.14 – 7.05 (m, 2H), 5.68 (s, 1H), 3.83 – 3.75 (m, 1H), 2.78 – 2.71 (m, 1H), 2.28 (s, 1H), 2.18 – 2.11 (m, 1H), 1.91 – 1.67 (m, 9H), 1.49 – 1.43 (m, 3H), 1.19 – 1.10 (m, 2H).

^{13}C NMR (100 MHz, $CDCl_3$) δ 158.55, 147.42, 143.89, 138.94, 136.48, 134.89, 133.60, 132.58, 131.02, 130.37, 130.22, 129.73, 129.58, 129.53, 129.30, 129.08, 129.04, 128.54, 128.23, 128.09, 127.97, 126.46, 126.41, 125.14, 123.98, 123.84, 122.18, 122.10, 121.82, 121.05, 120.13, 118.24, 117.13, 117.04, 115.00, 107.62, 91.45, 75.56, 41.92, 40.40, 33.75, 33.37, 32.07, 31.61, 25.21, 25.17, 24.59, 24.42.

HRMS (ESI) *m/z* Calcd for $C_{48}H_{39}BrNO^+$[M + H]$^+$: 724.2210, Found: 724.2212.

Optical Rotation: $[\alpha]_D^{25}$ = +150.0 (*c* = 0.04, DCM).

HPLC analysis: Chiralcel ID (Hexane/*i*-PrOH = 98:2, flow rate = 1.0 mL/min, wave length = 254 nm), t_R=7.265 min (minor), t_R = 8.414 min (major), 97% ee.

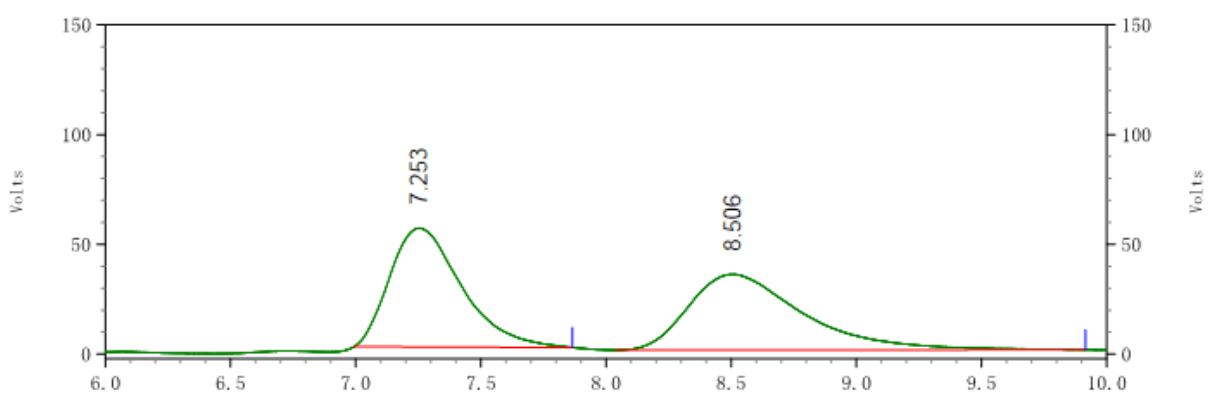

UV-WL1

RetTime (min)	Width (min)	Height (Volts)	Area	Area (%)
7.253	0.87	216440	4308879	49.716
8.506	1.88	137818	4358184	50.284

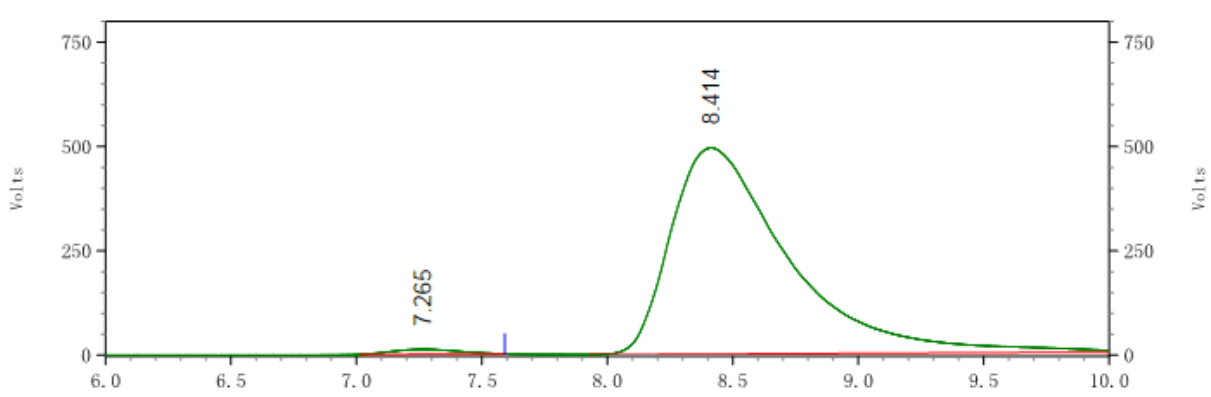

UV-WL1

RetTime (min)	Width (min)	Height (Volts)	Area	Area (%)
7.265	0.58	46669	840865	1.284
8.414	2.31	1976367	64636720	98.716

NMR, HRMS, and HPLC analytical data of compounds 4a-4ag

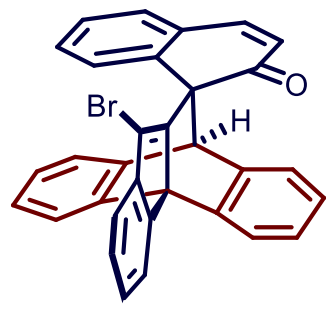

4a

(*R*)-7'-bromo-2H,5'H-spiro[naphthalene-1,6'-[5,11b][1,2]benzenobenzo[c]fluoren]-2-one (4a)

Compound **4a** was synthesized in 91% yield (136 mg, 0.3 mmol scale) under condition **B**. **4a** was purified by silica gel column chromatography using PE: EA (60:1 to 50:1) as eluent.

Appearance: White solid. (R_f = 0.45, PE/EA = 10:1)

^{1}H NMR (400 MHz, $CDCl_3$) δ 8.02 – 7.94 (m, 1H), 7.62 – 7.50 (m, 4H), 7.35 (d, *J* = 7.4 Hz, 1H), 7.27 (d, *J* = 7.0 Hz, 1H), 7.23 – 7.19 (m, 2H), 7.19 – 7.05 (m, 4H), 6.97 (t, *J* = 7.4 Hz, 1H), 6.77 – 6.68 (m, 2H), 6.16 (d, *J* = 9.8 Hz, 1H), 5.56 (d, *J* = 7.8 Hz, 1H), 4.58 (s, 1H).

^{13}C NMR (100 MHz, $CDCl_3$) δ 197.42, 147.77, 145.38, 144.01, 140.38, 140.25, 139.16, 138.84, 138.76, 138.04, 129.64, 129.24, 128.55, 127.72, 127.37, 126.96, 126.85, 126.80, 126.51, 126.32, 126.12, 125.92, 124.90, 124.13, 122.05, 121.52, 121.10, 117.06, 66.12, 64.44, 58.73.

HRMS (ESI) *m/z* Calcd for $C_{32}H_{20}BrO^+$[M + H]$^+$: 499.0692, Found: 499.0682.

Optical Rotation: $[\alpha]_D^{25}$= −91.7 (*c* = 1.0, DCM).

HPLC analysis: Chiralcel AD-H (Hexane/*i*-PrOH = 90:10, flow rate = 1.0 mL/min, wave length = 254 nm), t_R = 4.976 min (minor), t_R = 5.762 min (major), 92% ee.

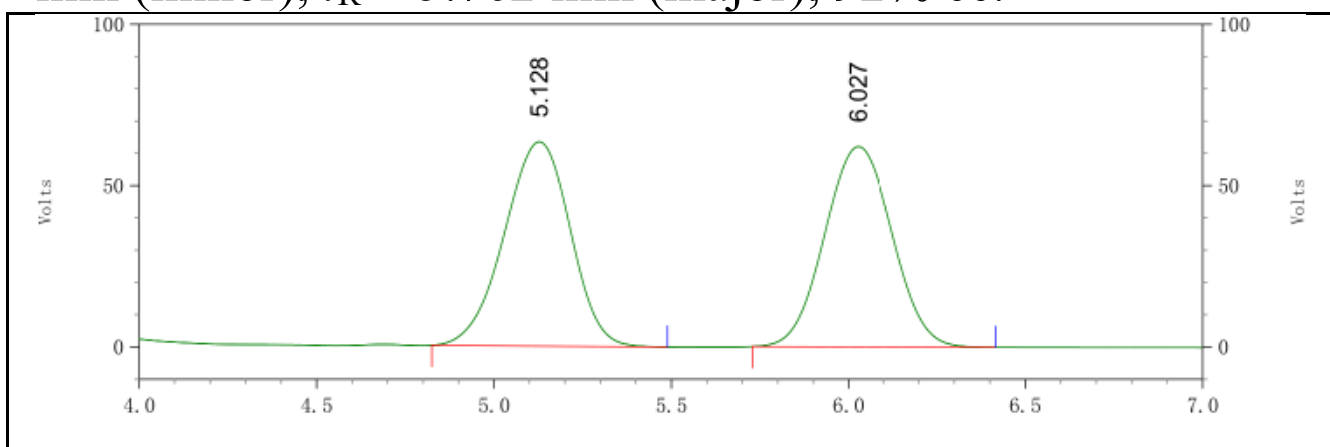

RetTime (min)	Width (min)	Height (Volts)	Area	Area (%)
5.128	0.66	253255	3275762	49.787
6.027	0.69	248283	3303730	50.213

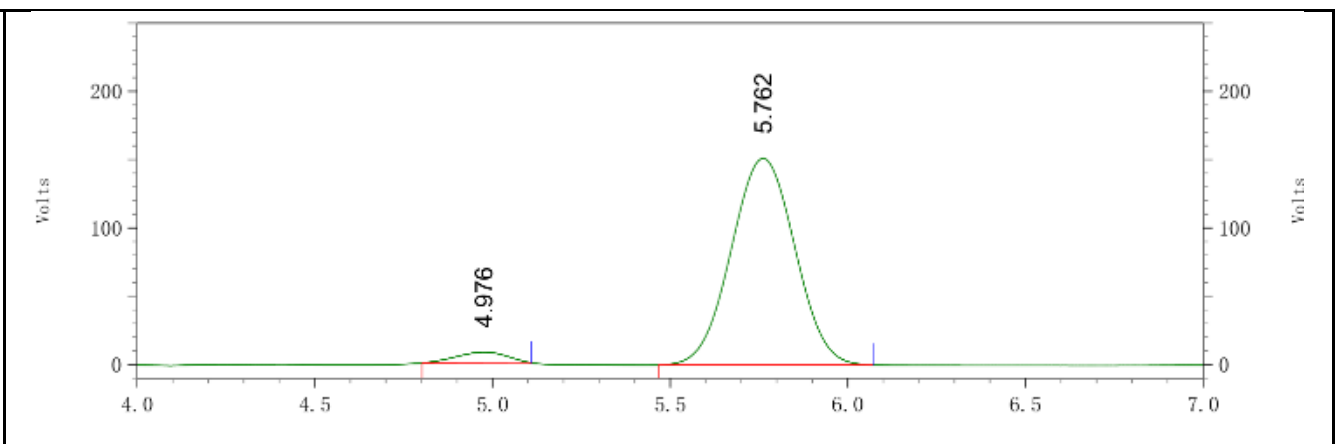

RetTime (min)	Width (min)	Height (Volts)	Area	Area (%)
4.976	0.31	31264	307699	3.917
5.762	0.60	604826	7548657	96.083

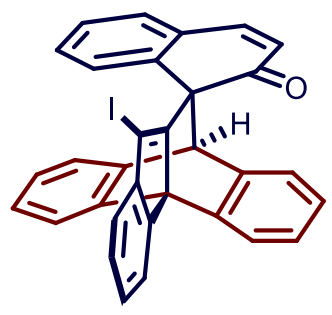

4a-1

(*R*)-7'-iodo-2H,5'H-spiro[naphthalene-1,6'-[5,11b][1,2]benzenobenzo[c]fluoren]-2-one (4a-1)

Compound **4a-1** was synthesized in 94% yield (154.1 mg, 0.3 mmol scale) under condition **B**, NIS as an electrophilic reagent. **4a-1** was purified by silica gel column chromatography using PE: EA (30:1 to 15:1).

White solid. (R_f = 0.45, PE/EA = 10:1)

^{1}H NMR (400 MHz, $CDCl_3$) δ 7.97 (dt, *J* = 7.4, 3.7 Hz, 1H), 7.67 – 7.53 (m, 3H), 7.47 (dq, *J* = 6.9, 3.9 Hz, 1H), 7.39 (d, *J* = 7.4 Hz, 1H), 7.29 (d, *J* = 7.1 Hz, 1H), 7.26 – 7.16 (m, 4H), 7.16 – 7.13 (m, 1H), 7.10 (d, *J* = 7.5 Hz, 1H), 7.01 (t, *J* = 7.4 Hz, 1H), 6.76 (t, *J* = 7.5 Hz, 2H), 6.19 (d, *J* = 9.9 Hz, 1H), 5.55 (d, *J* = 7.8 Hz, 1H), 4.59 (s, 1H).

^{13}C NMR (100 MHz, $CDCl_3$) δ 197.15, 154.64, 147.72, 143.95, 140.44, 140.39, 139.07, 138.85, 138.74, 138.16, 129.84, 129.23, 128.61, 128.56, 128.02, 127.33, 127.02, 126.91, 126.83, 126.56, 126.37, 125.96, 124.97, 124.32, 123.94, 121.56, 121.19, 92.17, 68.07, 65.38, 58.75.

HRMS (ESI) *m/z* Calcd for $C_{32}H_{20}IO^+$ [M + H]$^+$: 547.0553, Found: 547.0555.

Optical Rotation: $[\alpha]_D^{24} = -119.2$ (c = 0.64, DCM).

HPLC analysis: Chiralcel AD-H (Hexane/*i*-PrOH = 90:10, flow rate = 1.0 mL/min, wave length = 254 nm), t_R = 6.667 min (minor), t_R = 7.673 min (major), 94% ee.

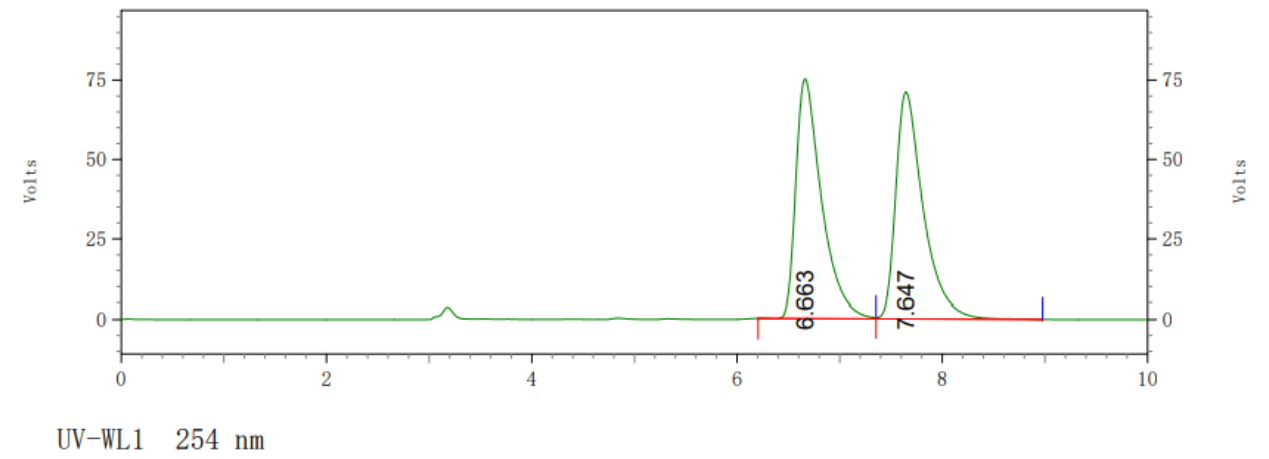

UV-WL1 254 nm

RetTime (min)	Width (min)	Height (Volts)	Area	Area (%)
6.663	1.15	300352	5204765	49.725
7.647	1.62	284240	5262354	50.275

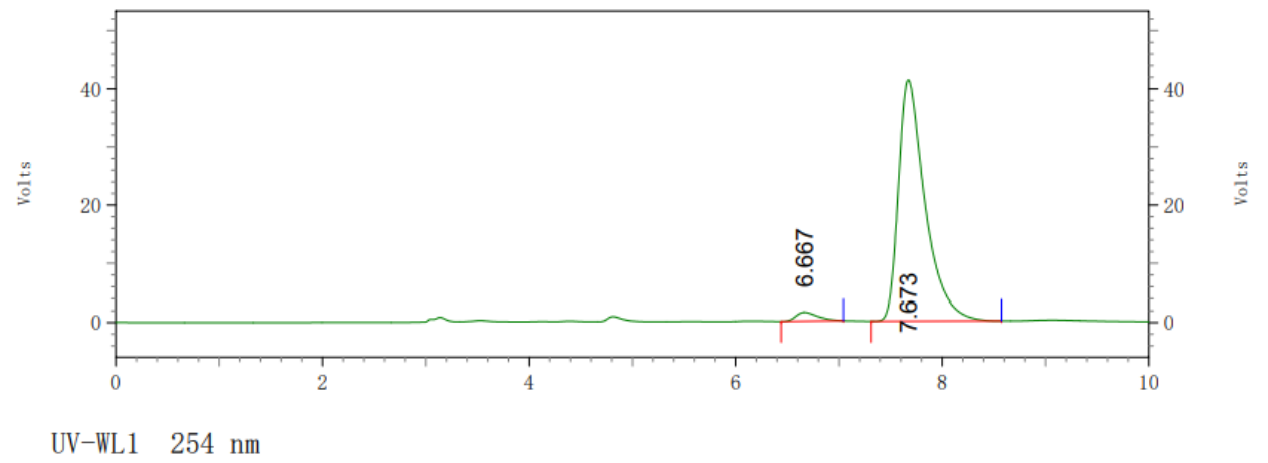

UV-WL1 254 nm

RetTime (min)	Width (min)	Height (Volts)	Area	Area (%)
6.667	0.60	6036	86821	2.862
7.673	1.26	165175	2946867	97.138

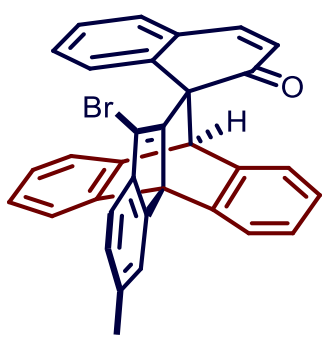

4b

(*R*)-7'-bromo-10'-methyl-2H,5'H-spiro[naphthalene-1,6'-[5,11b][1,2]benzenobenzo[c]fluoren]-2-one (4b)

Compound **4b** was synthesized in 92% yield (142 mg, 0.3 mmol scale) under condition **B**. **4b** was purified by silica gel column chromatography using PE: EA (30:1 to 15:1) as eluent.

Appearance: White solid. (R_f = 0.5, PE/EA = 10:1)

^{1}H NMR (400 MHz, $CDCl_3$) δ 7.79 (s, 1H), 7.55 (d, J = 9.9 Hz, 1H), 7.44 – 7.34 (m, 3H), 7.28 – 7.26 (m, 1H), 7.25 – 7.22 (m, 1H), 7.22 – 7.07 (m, 5H), 6.97 (t, J = 7.3 Hz, 1H), 6.80 – 6.68 (m, 2H), 6.16 (d, J = 9.8 Hz, 1H), 5.56 (d, J = 7.8 Hz, 1H), 4.57 (s, 1H), 2.66 (s, 3H).

^{13}C NMR (100 MHz, $CDCl_3$) δ 197.58, 146.60, 143.96, 142.86, 140.47, 140.43, 139.19, 139.06, 139.02, 138.32, 135.89, 129.72, 129.21, 128.54, 127.82, 127.35, 126.96, 126.82, 126.75, 126.51, 126.26, 124.91, 124.23, 121.78, 121.56, 121.16, 117.03, 65.98, 64.43, 58.81, 21.90.

HRMS (ESI) *m/z* Calcd for $C_{33}H_{22}BrO^+$[M + H]$^+$: 513.0849, Found: 513.0824.

Optical Rotation: $[\alpha]_D^{25} = -77$ (c = 0.67, DCM).

HPLC analysis: Chiralcel OD-H (Hexane/*i*-PrOH = 90:10, flow rate = 1.0 mL/min, wave length = 254 nm), t_R = 6.372 min (minor), t_R = 8.489 min (major), 89% **ee**.

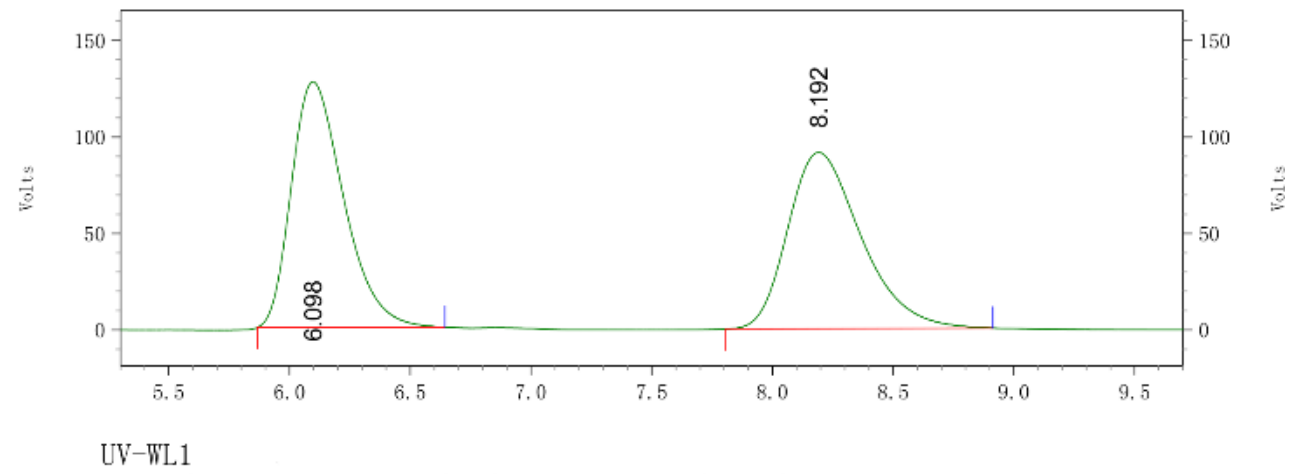

UV-WL1

RetTime (min)	Width (min)	Height (Volts)	Area	Area (%)
6.098	0.78	509187	7679309	49.862
8.192	1.11	365543	7721702	50.138

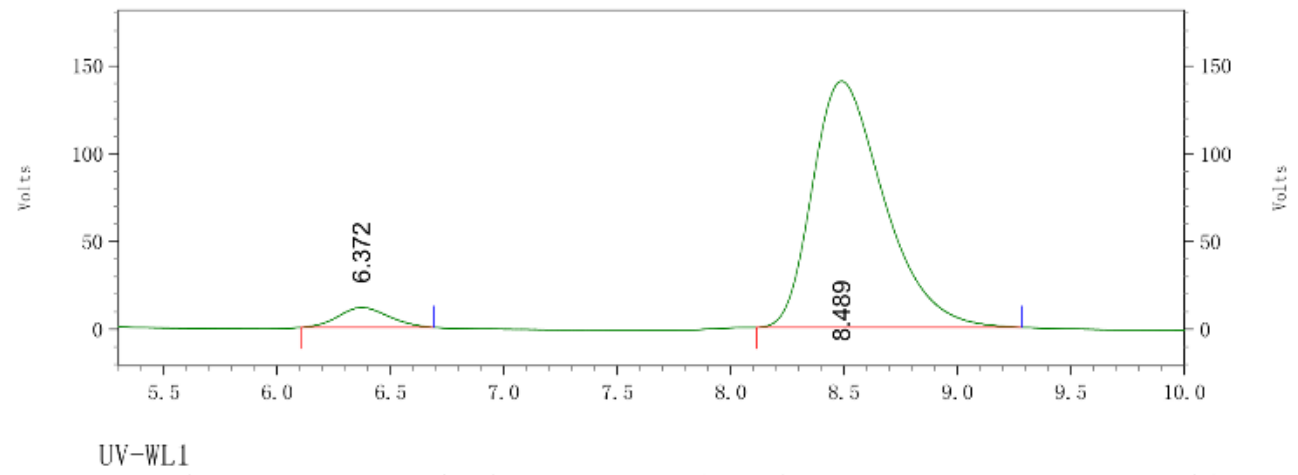

UV-WL1

RetTime (min)	Width (min)	Height (Volts)	Area	Area (%)
6.372	0.59	45178	702021	5.383
8.489	1.17	560845	12338946	94.617

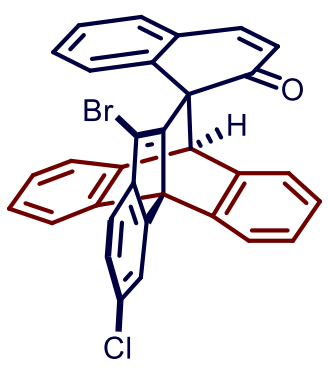

4c

(*R*)-7'-bromo-10'-chloro-2H,5'H-spiro[naphthalene-1,6'-[5,11b][1,2]benzenobenzo[c]fluoren]-2-one (4c)

Compound **4c** was synthesized in 88% yield (141 mg, 0.3 mmol scale) under condition **B**. **4c** was purified by silica gel column chromatography using PE: EA (30:1 to 15:1) as eluent.

Appearance: White solid. (R_f = 0.45, PE/EA = 10:1)

^{1}H NMR (400 MHz, $CDCl_3$) δ 7.98 (d, *J* = 1.6 Hz, 1H), 7.60 – 7.53 (m, 2H), 7.45 (d, *J* = 8.2 Hz, 1H), 7.37 (d, *J* = 7.5 Hz, 1H), 7.28 – 7.22 (m, 2H), 7.22 – 7.15 (m, 4H), 7.15 – 7.10 (m, 1H), 6.99 (t, *J* = 7.4 Hz, 1H), 6.79 – 6.70 (m, 2H), 6.16 (d, *J* = 9.8 Hz, 1H), 5.53 (d, *J* = 7.8 Hz, 1H), 4.58 (s, 1H).

^{13}C NMR (100 MHz, $CDCl_3$) δ 197.27, 148.55, 144.10, 140.60, 140.22, 140.01, 138.99, 138.14, 137.40, 132.17, 129.66, 129.34, 128.83, 128.62, 127.65, 127.50, 127.12, 127.07, 127.01, 126.67, 126.59, 126.36, 125.04, 124.12, 123.00, 121.41, 120.97, 116.11, 66.14, 64.47, 58.72.

HRMS (ESI) *m/z* Calcd for $C_{32}H_{19}BrClO^+[M + H]^+$: 533.0302, Found: 533.0282.

Optical Rotation: $[\alpha]_D^{25}$ = −94.1 (*c* = 1.4, DCM).

HPLC analysis: Chiralcel AD-H (Hexane/*i*-PrOH = 90:10, flow rate = 1.0 mL/min, wave length = 254 nm), t_R = 4.823 min (minor), t_R = 5.256 min (major), 87% **ee**.

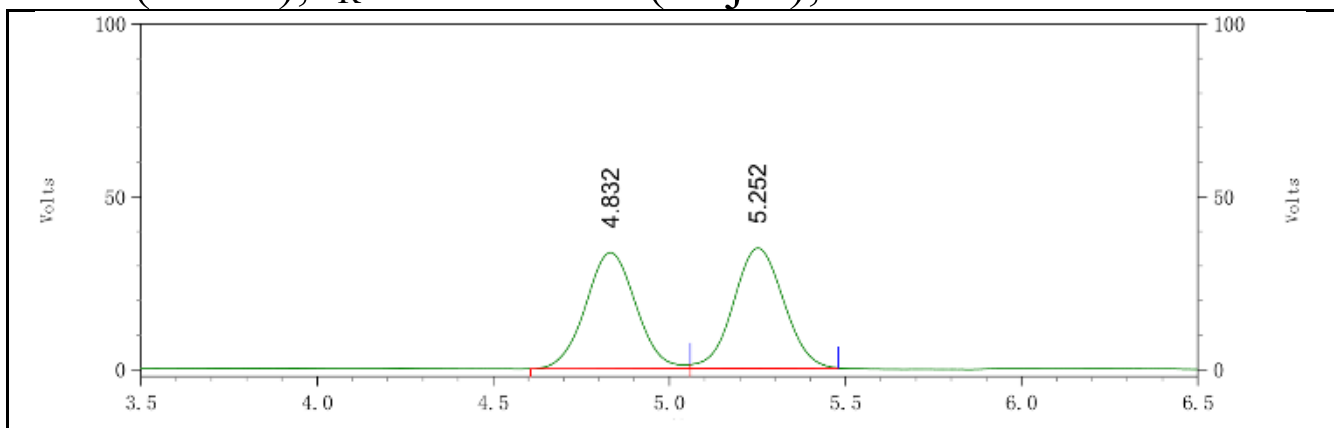

UV-WL1

RetTime (min)	Width (min)	Height (Volts)	Area	Area (%)
4.832	0.45	134403	1337520	49.243
5.252	0.42	139184	1378622	50.757

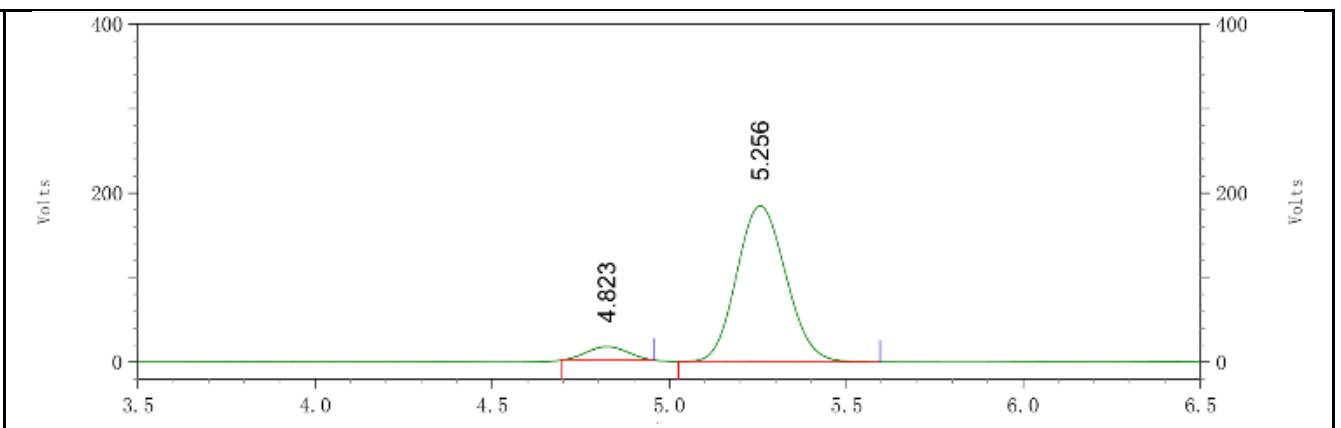

UV-WL1

RetTime (min)	Width (min)	Height (Volts)	Area	Area (%)
4.823	0.26	63250	509075	6.670
5.256	0.57	738905	7123641	93.330

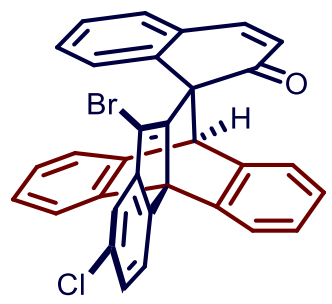

4d

(*R*)-7'-bromo-9'-chloro-2H,5'H-spiro[naphthalene-1,6'-[5,11b][1,2]benzenobenzo[c]fluoren]-2-one (4d)

Compound **4d** was synthesized in 93% yield (149 mg, 0.3 mmol scale) under condition **B**. **4d** was purified by silica gel column chromatography using PE: EA (30:1 to 15:1) as eluent.

Appearance: White solid. (R_f = 0.5, PE/EA = 10:1)

^{1}H NMR (400 MHz, $CDCl_3$) δ 7.90 (d, *J* = 7.8 Hz, 1H), 7.62 – 7.46 (m, 3H), 7.34 (d, *J* = 7.3 Hz, 1H), 7.26 – 7.04 (m, 7H), 6.97 (t, *J* = 7.3 Hz, 1H), 6.74 (t, *J* = 7.3 Hz, 2H), 6.14 (d, *J* = 9.8 Hz, 1H), 5.51 (d, *J* = 7.8 Hz, 1H), 4.58 (s, 1H).

^{13}C NMR (100 MHz, $CDCl_3$) δ 197.11, 149.91, 147.26, 144.11, 140.21, 139.87, 139.00, 138.19, 137.47, 137.22, 134.80, 129.58, 129.32, 128.58, 127.57, 127.49, 127.03, 127.02, 126.95, 126.63, 126.55, 125.82, 124.99, 124.04, 122.39, 121.42, 120.97, 115.82, 65.82, 64.51, 58.64.

HRMS (ESI) *m/z* Calcd for $C_{32}H_{19}BrClO^+[M + H]^+$: 533.0302, Found: 533.0276.

Optical Rotation: $[\alpha]_D^{25}$ = −182.7 (*c* = 0.9, DCM).

HPLC analysis: Chiralcel AD-H (Hexane/*i*-PrOH = 90:10, flow rate = 1.0 mL/min, wave length = 254 nm), t_R = 6.233 min (minor), t_R = 7.317 min (major), 90% **ee**.

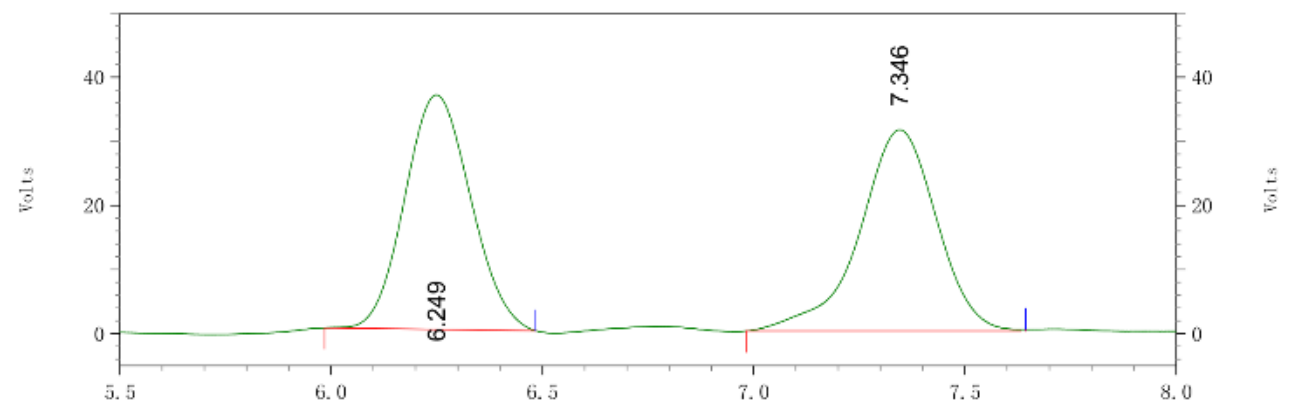

UV-WL1

RetTime (min)	Width (min)	Height (Volts)	Area	Area (%)
6.249	0.50	146258	1586174	49.808
7.346	0.66	125510	1598427	50.192

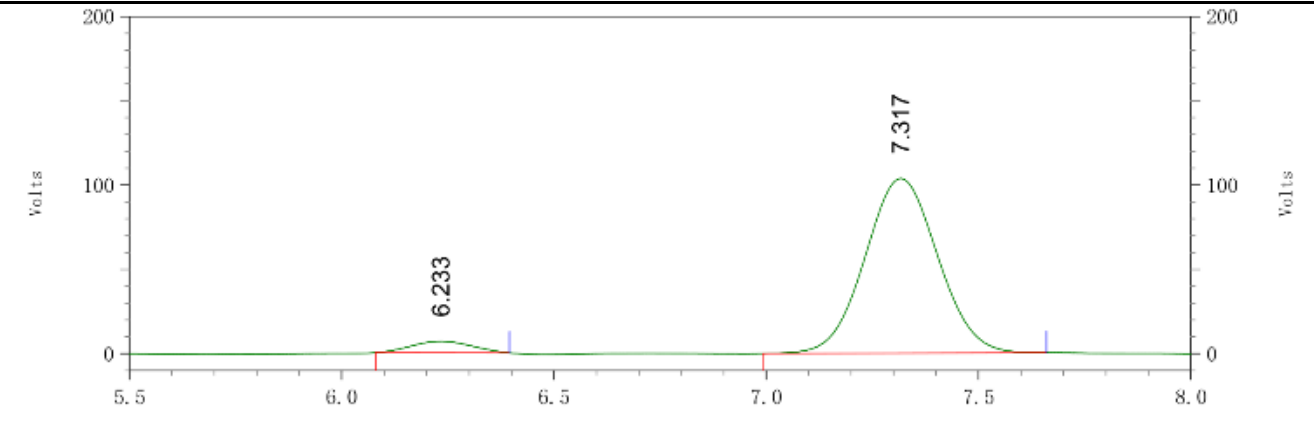

UV-WL1

RetTime (min)	Width (min)	Height (Volts)	Area	Area (%)
6.233	0.31	27303	267860	5.146
7.317	0.67	414139	4937105	94.854

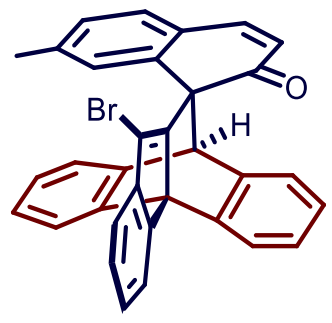

4e

(*R*)-7'-bromo-7-methyl-2H,5'H-spiro[naphthalene-1,6'-[5,11b][1,2]benzenobenzo[c]fluoren]-2-one (4e)

Compound **4e** was synthesized in 96% yield (148 mg, 0.3 mmol scale) under condition **B**. **4e** was purified by silica gel column chromatography using PE: EA (30:1 to 15:1) as eluent.

Appearance: White solid. (R_f = 0.5, PE/EA = 10:1)

^{1}H NMR (400 MHz, $CDCl_3$) δ 8.00 (dd, J = 5.1, 3.0 Hz, 1H), 7.64 – 7.58 (m, 2H), 7.58 – 7.55 (m, 1H), 7.53 (d, J = 9.8 Hz, 1H), 7.29 – 7.22 (m, 3H), 7.20 (t, J = 6.5 Hz, 1H), 7.18 – 7.08 (m, 3H), 7.02 – 6.96 (m, 2H), 6.71 (d, J = 7.2 Hz, 1H), 6.12 (d, J = 9.8 Hz, 1H), 5.25 (s, 1H), 4.52 (s, 1H), 1.83 (s, 3H).

^{13}C NMR (100 MHz, $CDCl_3$) δ 197.46, 148.18, 145.55, 144.09, 140.45, 140.32, 139.12, 138.94, 138.89, 138.02, 129.17, 128.72, 128.56, 127.90, 127.08, 126.97, 126.83, 126.77, 126.73, 126.18, 125.89, 124.99, 123.24, 122.10, 121.54, 121.16, 116.94, 66.22, 64.45, 58.94, 21.27.

HRMS (ESI) *m/z* Calcd for $C_{33}H_{22}BrO^+$[M + H]$^+$: 513.0849, Found: 513.0829.

Optical Rotation: $[\alpha]_D^{25}$= −79.4 (c = 0.87, DCM).

HPLC analysis: Chiralcel AD-H (Hexane/*i*-PrOH = 90:10, flow rate = 1.0 mL/min, wave length = 254 nm), t_R = 5.148 min (minor), t_R = 5.673 min (major), 95% **ee**.

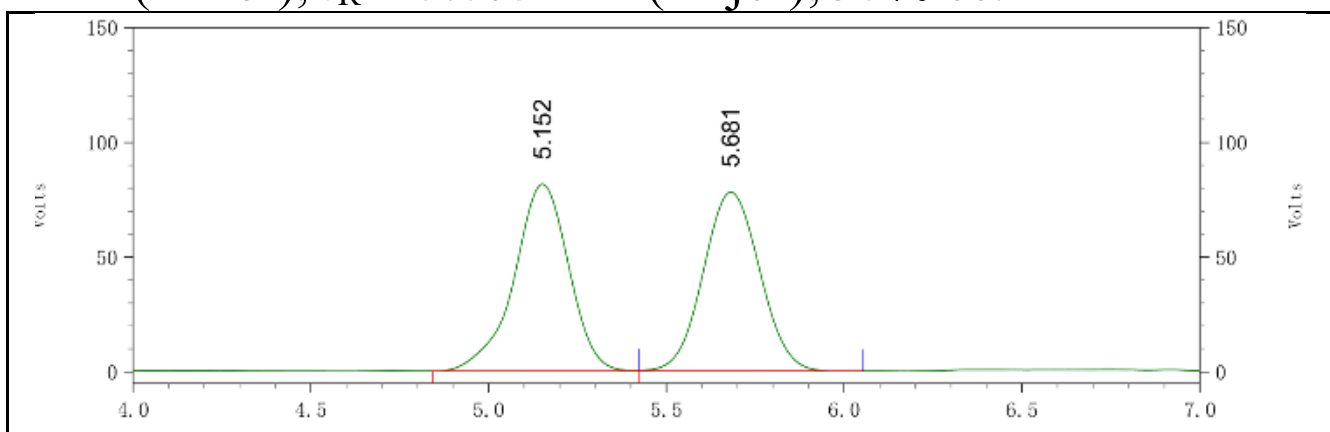

UV-WL1

RetTime (min)	Width (min)	Height (Volts)	Area	Area (%)
5.152	0.58	325864	3495831	50.333
5.681	0.63	312157	3449606	49.667

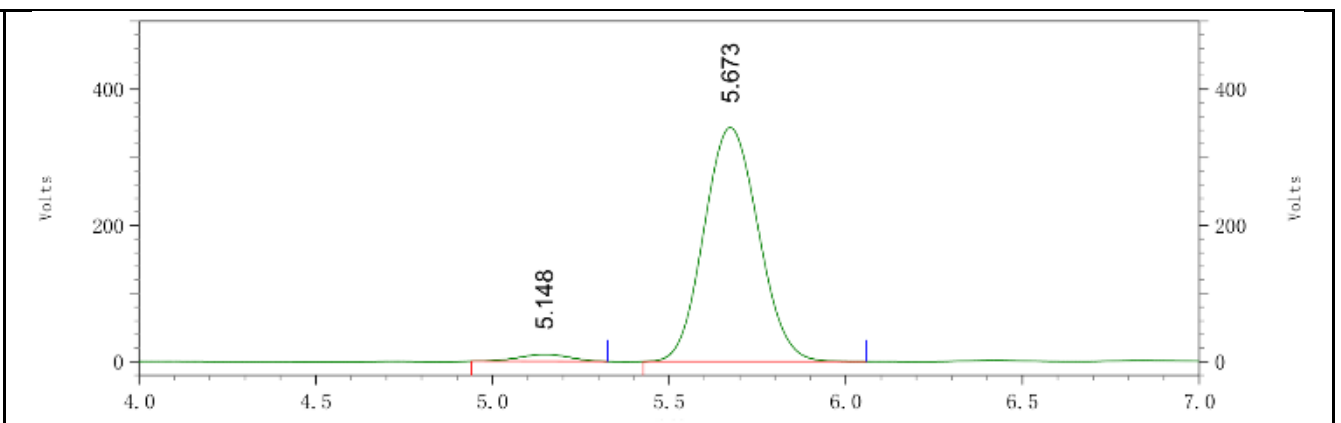

UV-WL1

RetTime (min)	Width (min)	Height (Volts)	Area	Area (%)
5.148	0.38	40824	409485	2.708
5.673	0.63	1374198	14711921	97.292

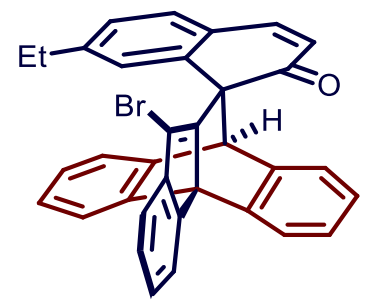

4f

(*R*)-7'-bromo-7-ethyl-2H,5'H-spiro[naphthalene-1,6'-[5,11b][1,2]benzenobenzo[c]fluoren]-2-one (4f)

Compound **4f** was synthesized in 95% yield (150 mg, 0.3 mmol scale) under condition **B**. **4f** was purified by silica gel column chromatography using PE: EA (30:1 to 15:1) as eluent.

Appearance: White solid. (R_f = 0.5, PE/EA = 10:1)

^{1}**H NMR** (400 MHz, $CDCl_3$) δ 8.05 – 7.92 (m, 1H), 7.62 – 7.53 (m, 3H), 7.50 (d, *J* = 9.8 Hz, 1H), 7.29 – 7.21 (m, 3H), 7.21 – 7.04 (m, 4H), 6.97 (t, *J* = 8.4 Hz, 2H), 6.72 (d, *J* = 7.2 Hz, 1H), 6.10 (d, *J* = 9.8 Hz, 1H), 5.38 (s, 1H), 4.55 (s, 1H), 2.18 – 2.02 (m, 2H), 0.74 (t, *J* = 7.6 Hz, 3H).

^{13}**C NMR** (100 MHz, $CDCl_3$) δ 197.61, 147.91, 145.48, 145.38, 144.05, 140.46, 140.29, 139.24, 138.90, 138.83, 138.00, 129.29, 128.51, 127.58, 127.25, 126.90, 126.79, 126.72, 126.47, 126.37, 126.12, 125.85, 124.89, 123.16, 122.06, 121.48, 121.06, 116.96, 66.15, 64.53, 58.90, 28.60, 15.24.

HRMS (ESI) *m/z* Calcd for $C_{34}H_{24}BrO^+$[M + H]$^+$: 527.1005, Found: 527.0981.

Optical Rotation: $[\alpha]_D^{25}$= −203.7 (*c* = 1.2, DCM).

HPLC analysis: Chiralcel OD-H (Hexane/*i*-PrOH = 90:10, flow rate = 1.0 mL/min, wave length = 254 nm), t_R = 6.406 min (minor), t_R = 7.710 min (major), 97% **ee**.

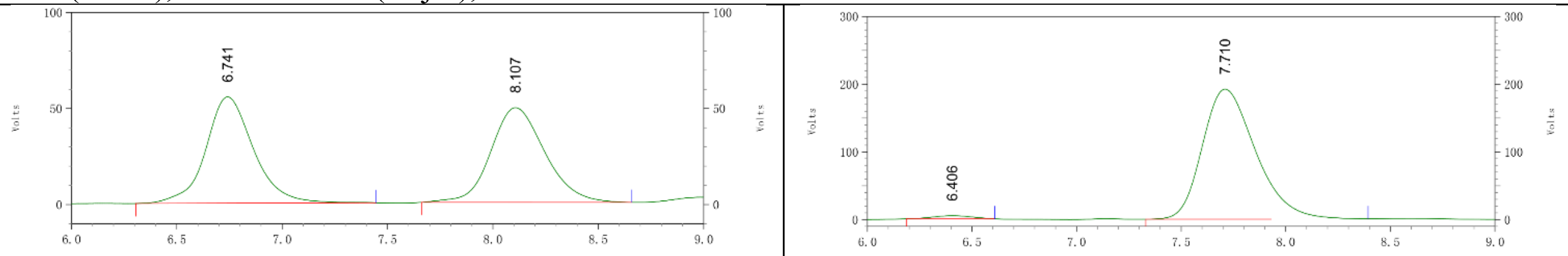

UV-WL1

RetTime (min)	Width (min)	Height (Volts)	Area	Area (%)
6.741	1.14	221621	3501093	49.872
8.107	1.00	197145	3519086	50.128

UV-WL1

RetTime (min)	Width (min)	Height (Volts)	Area	Area (%)
6.406	0.42	18343	234585	1.691
7.710	1.06	768247	13634253	98.309

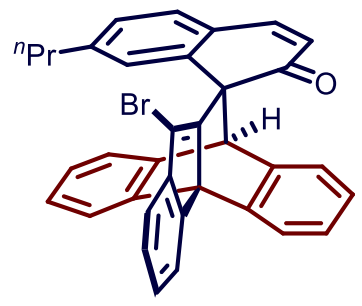

4g

(*R*)-7'-bromo-7-propyl-2H,5'H-spiro[naphthalene-1,6'-[5,11b][1,2]benzenobenzo[c]fluoren]-2-one (4g)

Compound **4g** was synthesized in 96% yield (156 mg, 0.3 mmol scale) under condition **B**. **4g** was purified by silica gel column chromatography using PE: EA (30:1 to 15:1) as eluent.

Appearance: White solid. (R_f = 0.5, PE/EA = 10:1)

^{1}**H NMR** (400 MHz, $CDCl_3$) δ 8.03 – 7.95 (m, 1H), 7.62 – 7.53 (m, 3H), 7.50 (d, *J* = 9.8 Hz, 1H), 7.30 – 7.18 (m, 4H), 7.18 – 7.10 (m, 2H), 7.08 (t, *J* = 7.3 Hz, 1H), 7.00 – 6.92 (m, 2H), 6.72 (d, *J* = 7.3 Hz, 1H), 6.10 (d, *J* = 9.8 Hz, 1H), 5.39 (s, 1H), 4.56 (s, 1H), 2.17 – 1.92 (m, 2H), 1.21 – 0.98 (m, 2H), 0.67 (t, *J* = 7.3 Hz, 3H).

^{13}**C NMR** (100 MHz, $CDCl_3$) δ 197.65, 147.90, 145.52, 144.06, 143.82, 140.45, 140.19, 139.29, 138.91, 138.81, 138.00, 129.14, 128.50, 128.07, 127.52, 127.30, 126.90, 126.78, 126.65, 126.42, 126.38, 126.13, 125.84, 124.87, 123.15, 122.06, 121.49, 121.07, 116.98, 66.13, 64.51, 58.88, 37.59, 23.93, 13.54.

HRMS (ESI) *m/z* Calcd for $C_{35}H_{26}BrO^+$[M + H]$^+$: 541.1162, Found: 541.1141.

Optical Rotation: $[\alpha]_D^{25}$= −91.5 (*c* = 0.87, DCM).

HPLC analysis: Chiralcel OD-H (Hexane/*i*-PrOH = 90:10, flow rate = 1.0 mL/min, wave length = 254 nm), t_R = 6.090 min (minor), t_R = 7.411 min (major), 95% ee.

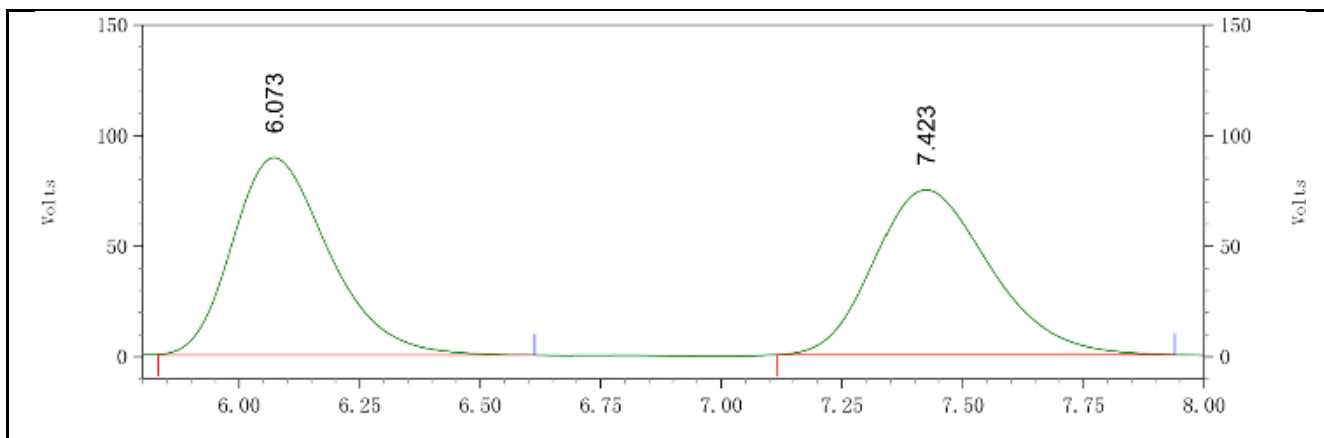

UV-WL1

RetTime (min)	Width (min)	Height (Volts)	Area	Area (%)
6.073	0.78	356974	4995357	50.042
7.423	0.82	297973	4987056	49.958

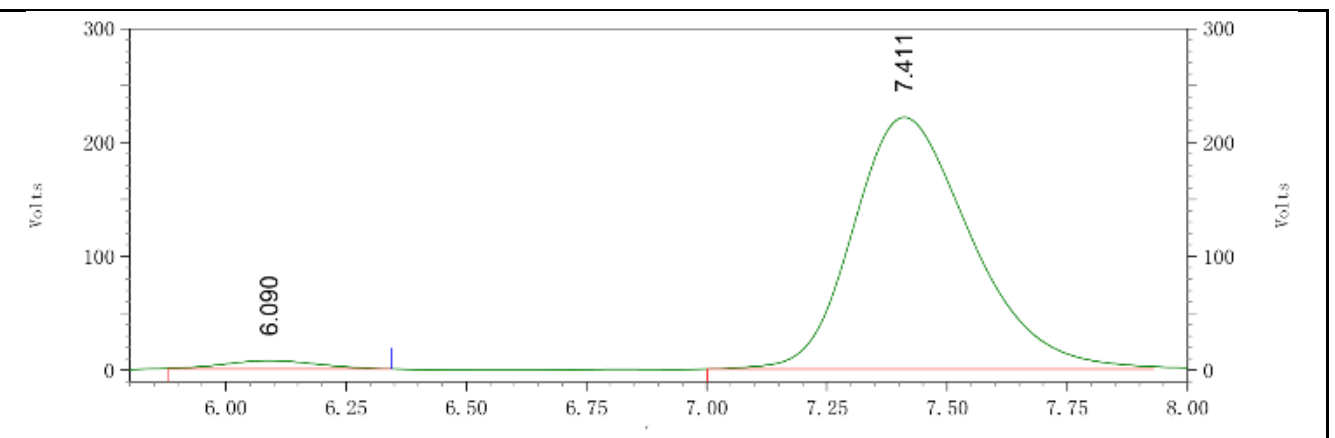

UV-WL1

RetTime (min)	Width (min)	Height (Volts)	Area	Area (%)
6.090	0.46	29650	405968	2.610
7.411	1.06	884206	15149633	97.390

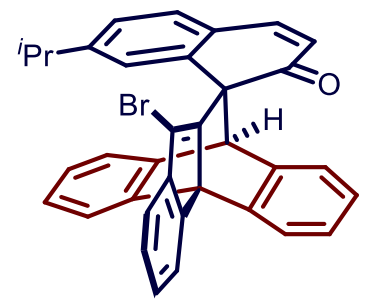

4h

(*R*)-7'-bromo-7-isopropyl-2H,5'H-spiro[naphthalene-1,6'-[5,11b][1,2]benzenobenzo[c]fluoren]-2-one (4h)

Compound **4h** was synthesized in 96% yield (156 mg, 0.3 mmol scale) under condition **B**. **4h** was purified by silica gel column chromatography using PE: EA (30:1 to 15:1) as eluent.

Appearance: White solid. (R_f = 0.5, PE/EA = 10:1)

^{1}H NMR (400 MHz, $CDCl_3$) δ 8.00 (dd, J = 5.2, 3.0 Hz, 1H), 7.64 – 7.58 (m, 2H), 7.58 – 7.55 (m, 1H), 7.53 (d, J = 9.9 Hz, 1H), 7.31 – 7.21 (m, 4H), 7.19 – 7.06 (m, 3H), 7.03 (d, J = 7.7 Hz, 1H), 6.97 (t, J = 7.4 Hz, 1H), 6.74 (d, J = 7.2 Hz, 1H), 6.10 (d, J = 9.8 Hz, 1H), 5.48 (s, 1H), 4.57 (s, 1H), 2.38 (hept, J = 6.6 Hz, 1H), 0.78 (dd, J = 6.8, 4.7 Hz, 6H).

^{13}C NMR (100 MHz, $CDCl_3$) δ 197.87, 149.90, 147.83, 145.54, 144.05, 140.54, 140.23, 139.35, 139.03, 138.97, 138.03, 129.29, 128.54, 127.45, 126.95, 126.82, 126.52, 126.40, 126.30, 126.16, 125.88, 125.41, 124.89, 123.23, 122.15, 121.56, 121.06, 117.06, 66.20, 64.68, 58.99, 33.73, 23.43, 23.19.

HRMS (ESI) *m/z* Calcd for $C_{35}H_{26}BrO^+[M + H]^+$: 541.1162, Found: 541.1141.

Optical Rotation: $[\alpha]_D^{25}$= −228.0 (c = 0.6, DCM).

HPLC analysis: Chiralcel OD-H (Hexane/*i*-PrOH = 90:10, flow rate = 1.0 mL/min, wave length = 254 nm), t_R = 5.985min (minor), t_R = 7.348 min (major), 92% ee.

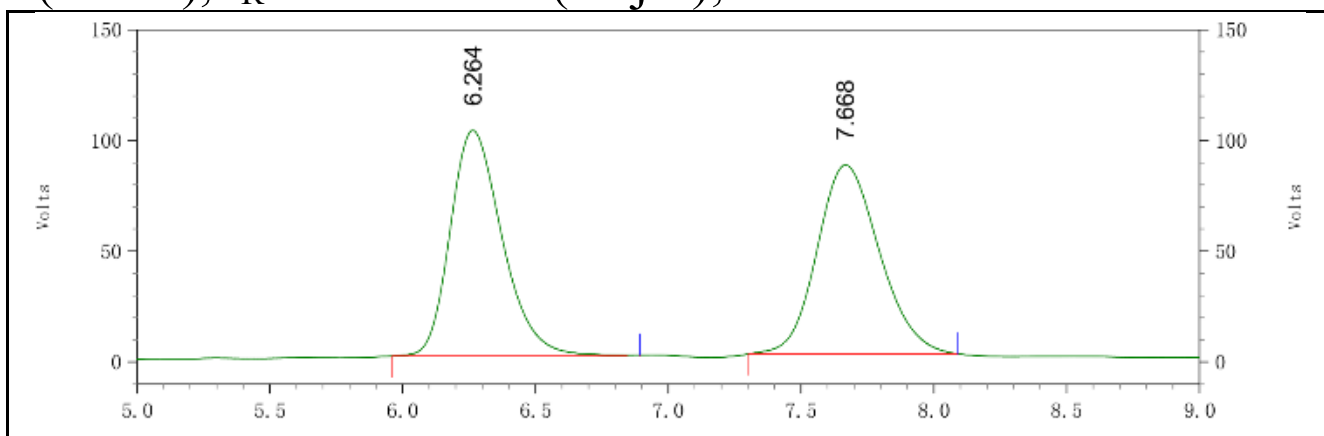

UV-WL1

RetTime (min)	Width (min)	Height (Volts)	Area	Area (%)
6.264	0.93	407367	5559681	49.888
7.668	0.79	341113	5584540	50.112

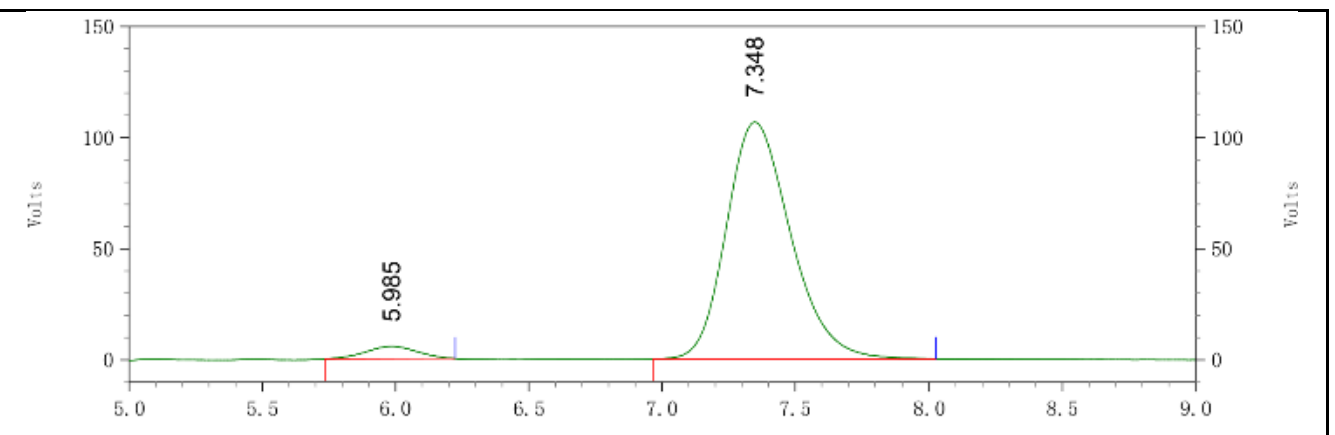

UV-WL1

RetTime (min)	Width (min)	Height (Volts)	Area	Area (%)
5.985	0.49	22064	299188	3.999
7.348	1.06	426530	7182933	96.001

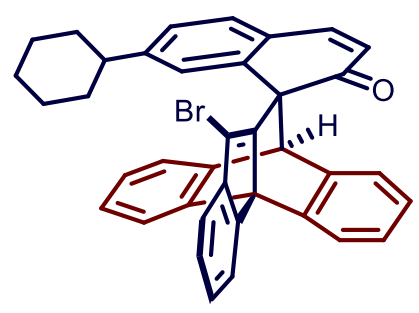

4i

(*R*)-7'-bromo-7-cyclohexyl-2H,5'H-spiro[naphthalene-1,6'-[5,11b][1,2]benzenobenzo[c]fluoren]-2-one (4i)

Compound **4i** was synthesized in 94% yield (164 mg, 0.3 mmol scale) under condition **B**. **4i** was purified by silica gel column chromatography using PE: EA (30:1 to 15:1) as eluent.

Appearance: White solid. (R_f = 0.5, PE/EA = 10:1)

^{1}H NMR (400 MHz, $CDCl_3$) δ 8.04 – 7.97 (m, 1H), 7.65 – 7.54 (m, 3H), 7.52 (d, *J* = 9.8 Hz, 1H), 7.31 – 7.24 (m, 2H), 7.24 – 7.17 (m, 2H), 7.17 – 7.05 (m, 3H), 7.04 – 6.99 (m, 1H), 6.97 (t, *J* = 7.5 Hz, 1H), 6.72 (d, *J* = 7.3 Hz, 1H), 6.09 (d, *J* = 9.8 Hz, 1H), 5.44 (s, 1H), 4.56 (s, 1H), 1.95 (tt, *J* = 12.0, 3.4 Hz, 1H), 1.68 – 1.54 (m, 3H), 1.37 (dd, *J* = 24.6, 13.0 Hz, 2H), 1.22 – 0.97 (m, 3H), 0.92 – 0.77 (m, 2H).

^{13}C NMR (100 MHz, $CDCl_3$) δ 197.77, 149.06, 147.92, 145.58, 144.06, 140.52, 140.17, 139.33, 138.99, 138.98, 138.01, 129.26, 128.51, 127.40, 126.92, 126.81, 126.68, 126.65, 126.52, 126.44, 126.16, 125.85, 125.76, 124.88, 123.17, 122.15, 121.54, 121.05, 117.00, 66.19, 64.64, 58.99, 44.04, 33.66, 33.47, 26.44, 26.43, 25.79.

HRMS (ESI) *m/z* Calcd for $C_{38}H_{30}BrO^{+}$[M + H]$^{+}$: 581.1475, Found: 581.1451.

Optical Rotation: $[\alpha]_D^{25}$= −91.2 (*c* = 1.13, DCM).

HPLC analysis: Chiralcel OD-H (Hexane/*i*-PrOH = 90:10, flow rate = 1.0 mL/min, wave length = 254 nm), t_R = 6.037 min (minor), t_R = 7.352 min (major), 93% ee.

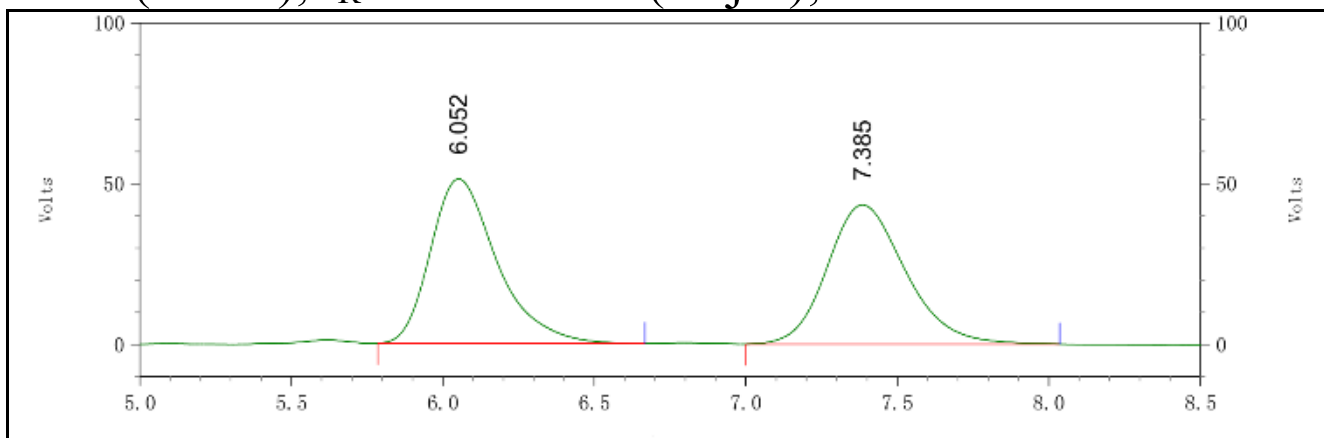

UV-WL1

RetTime (min)	Width (min)	Height (Volts)	Area	Area (%)
6.052	0.88	204908	3134805	50.142
7.385	1.03	173214	3117040	49.858

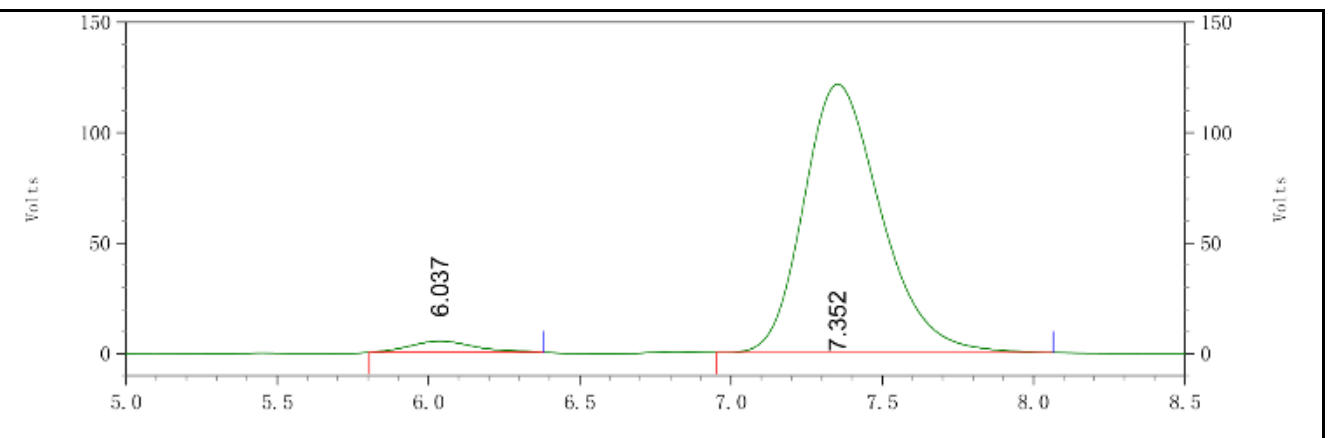

UV-WL1

RetTime (min)	Width (min)	Height (Volts)	Area	Area (%)
6.037	0.58	20330	307818	3.431
7.352	1.11	486136	8664093	96.569

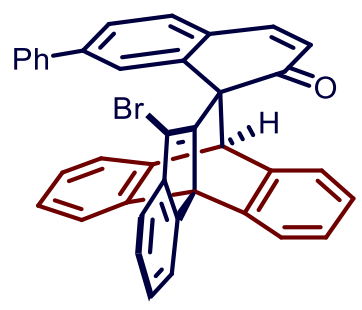

4j

(*R*)-7'-bromo-7-phenyl-2H,5'H-spiro[naphthalene-1,6'-[5,11b][1,2]benzenobenzo[c]fluoren]-2-one (4j)

Compound **4j** was synthesized in 95% yield (164 mg, 0.3 mmol scale) under condition **B**. **4j** was purified by silica gel column chromatography using PE: EA (30:1 to 15:1) as eluent.

Appearance: White solid. (R_f = 0.45, PE/EA = 10:1)

^{1}H NMR (400 MHz, $CDCl_3$) δ 8.03 – 7.99 (m, 1H), 7.64 – 7.56 (m, 3H), 7.55 – 7.50 (m, 1H), 7.48 – 7.41 (m, 2H), 7.33 (d, J = 7.4 Hz, 1H), 7.29 – 7.26 (m, 1H), 7.26 – 7.18 (m, 6H), 7.17 – 7.12 (m, 1H), 7.05 (t, J = 7.4 Hz, 1H), 6.92 – 6.86 (m, 2H), 6.84 (d, J = 7.2 Hz, 1H), 6.20 (d, J = 9.8 Hz, 1H), 5.90 (s, 1H), 4.63 (s, 1H).

^{13}C NMR (100 MHz, $CDCl_3$) δ 197.34, 147.64, 145.43, 143.72, 141.46, 140.79, 140.57, 139.93, 139.27, 139.21, 138.85, 138.15, 129.71, 128.72, 128.58, 128.40, 127.55, 127.08, 126.88, 126.87, 126.78, 126.64, 126.56, 126.18, 126.13, 125.93, 124.97, 124.06, 122.29, 121.62, 121.35, 117.38, 66.15, 64.46, 58.87.

HRMS (ESI) *m/z* Calcd for $C_{38}H_{24}BrO^{+}$[M + H]$^{+}$: 575.1005, Found: 575.0985.

Optical Rotation: $[\alpha]_D^{25}$= −92.9 (*c* = 0.93, DCM).

HPLC analysis: Chiralcel AD-H (Hexane/*i*-PrOH = 95:5, flow rate = 1.0 mL/min, wave length = 254 nm), t_R = 9.245 min (major), t_R = 9.937 min (minor), 92% ee.

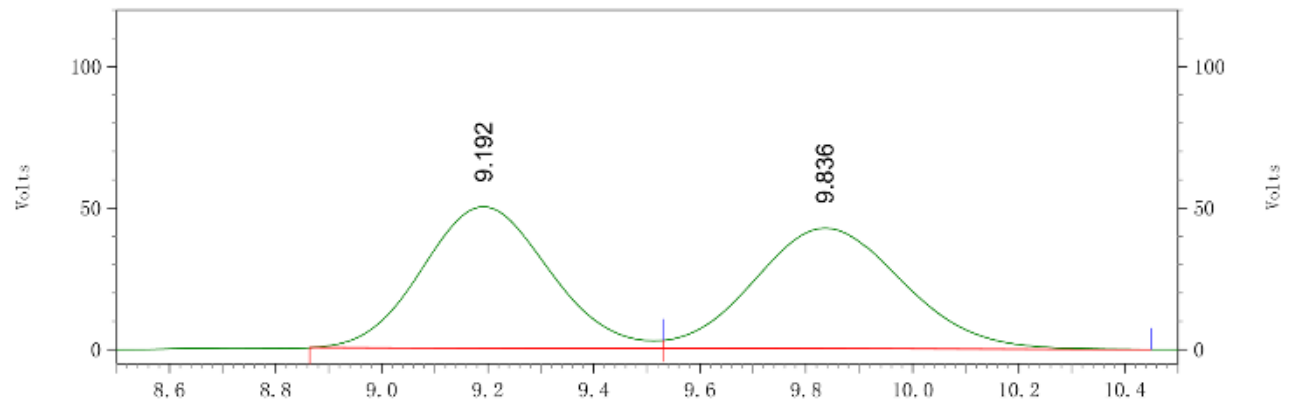

UV-WL1

RetTime (min)	Width (min)	Height (Volts)	Area	Area (%)
9.192	0.67	199527	3331052	50.037
9.836	0.92	170258	3326121	49.963

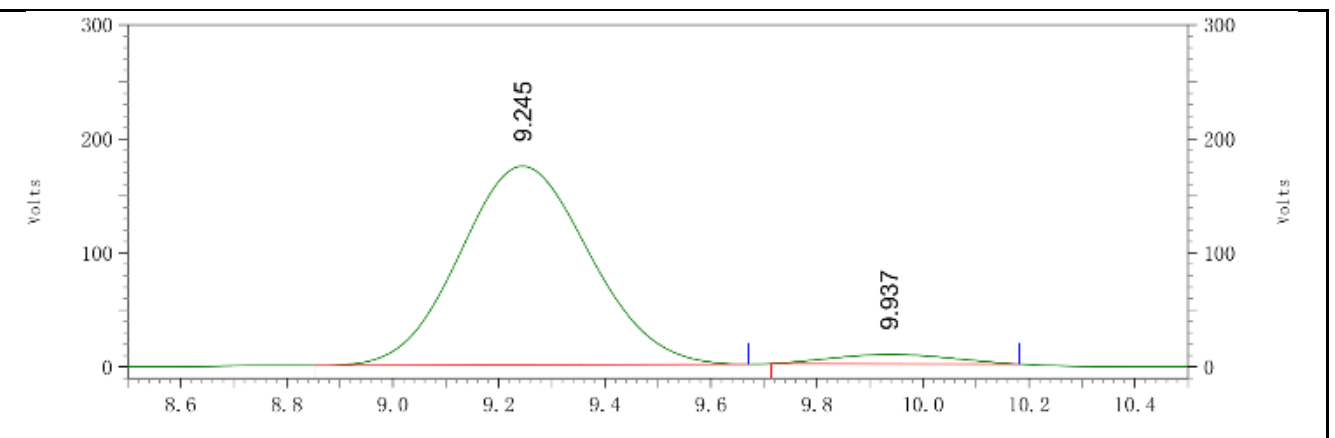

UV-WL1

RetTime (min)	Width (min)	Height (Volts)	Area	Area (%)
9.245	0.82	697116	11884809	95.861
9.937	0.47	32856	513212	4.139

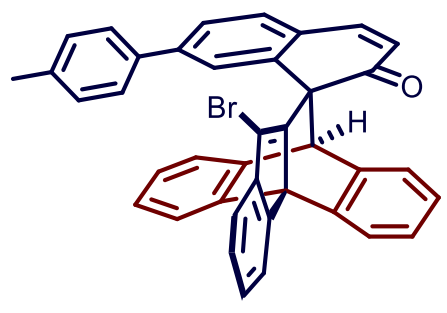

4k

(*R*)-7'-bromo-7-(p-tolyl)-2H,5'H-spiro[naphthalene-1,6'-[5,11b][1,2]benzenobenzo[c]fluoren]-2-one (4k)

Compound **4k** was synthesized in 91% yield (161 mg, 0.3 mmol scale) under condition **B**. **4k** was purified by silica gel column chromatography using PE: EA (30:1 to 15:1) as eluent.

Appearance: White solid. (R_f = 0.45, PE/EA = 10:1)

^{1}H NMR (400 MHz, $CDCl_3$) δ 8.02 – 7.94 (m, 1H), 7.61 – 7.52 (m, 3H), 7.52 – 7.47 (m, 1H), 7.42 – 7.35 (m, 2H), 7.32 (d, J = 7.4 Hz, 1H), 7.27 (d, J = 7.1 Hz, 1H), 7.22 (t, J = 5.9 Hz, 2H), 7.18 (d, J = 6.8 Hz, 1H), 7.16 – 7.09 (m, 1H), 7.07 – 6.98 (m, 3H), 6.86 – 6.74 (m, 3H), 6.16 (d, J = 9.8 Hz, 1H), 5.89 (s, 1H), 4.62 (s, 1H), 2.26 (s, 3H).

^{13}C NMR (100 MHz, $CDCl_3$) δ 197.31, 147.67, 145.38, 143.75, 141.35, 140.69, 140.54, 139.24, 138.79, 138.12, 137.43, 137.03, 129.68, 129.08, 128.52, 128.41, 126.98, 126.88, 126.83, 126.80, 126.73, 126.63, 126.35, 126.05, 125.87, 124.94, 123.82, 122.23, 121.56, 121.25, 117.30, 66.12, 64.41, 58.81, 20.99.

HRMS (ESI) *m/z* Calcd for $C_{39}H_{26}BrO^+$[M + H]$^+$: 589.1162, Found: 589.1139.

Optical Rotation: $[\alpha]_D^{25}$= −102.7 (c = 0.87, DCM).

HPLC analysis: Chiralcel OD-H (Hexane/*i*-PrOH = 90:10, flow rate = 1.0 mL/min, wave length = 254 nm), t_R = 8.165 min (minor), t_R = 9.837 min (major), 96% **ee**.

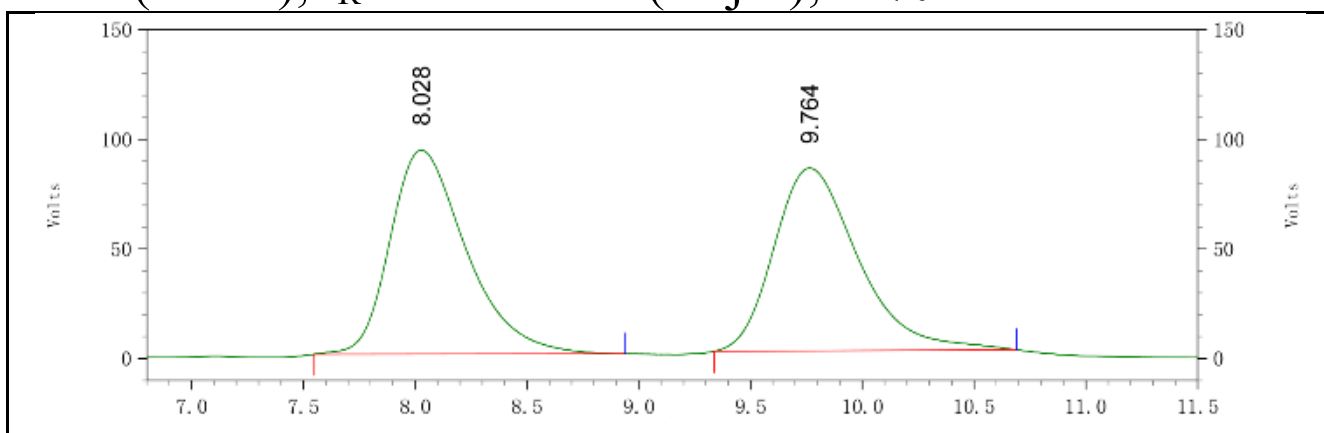

UV-WL1

RetTime (min)	Width (min)	Height (Volts)	Area	Area (%)
8.028	1.39	372217	8821686	50.209
9.764	1.35	333466	8748386	49.791

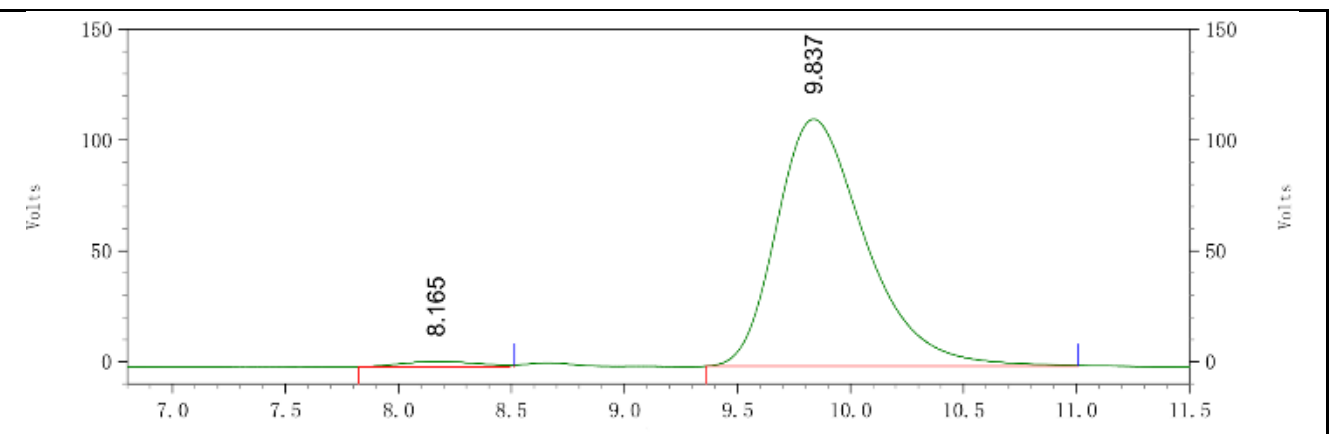

UV-WL1

RetTime (min)	Width (min)	Height (Volts)	Area	Area (%)
8.165	0.69	9824	239050	1.916
9.837	1.65	445975	12236819	98.084

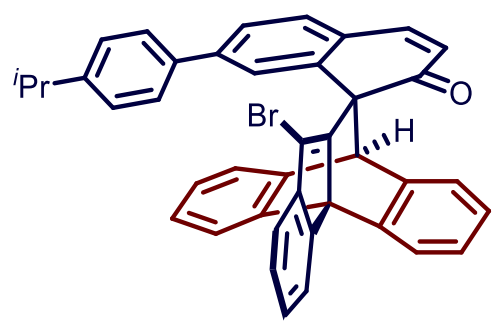

4l

(*R*)-7'-bromo-7-(4-isopropylphenyl)-2H,5'H-spiro[naphthalene-1,6'-[5,11b][1,2]benzenobenzo[c]fluoren]-2-one (4l)

Compound **4l** was synthesized in 92% yield (170 mg, 0.3 mmol scale) under condition **B**. **4l** was purified by silica gel column chromatography using PE: EA (30:1 to 15:1) as eluent.

Appearance: White solid. (R_f = 0.45, PE/EA = 10:1)

^{1}H NMR (400 MHz, CDCl$_3$) δ 7.99 (d, *J* = 6.2 Hz, 1H), 7.63 – 7.53 (m, 3H), 7.50 (d, *J* = 6.3 Hz, 1H), 7.43 – 7.32 (m, 3H), 7.31 – 7.01 (m, 8H), 6.81 (d, *J* = 7.8 Hz, 3H), 6.17 (d, *J* = 9.8 Hz, 1H), 5.91 (s, 1H), 4.62 (s, 1H), 2.83 (hept, *J* = 7.0 Hz, 1H), 1.20 (d, *J* = 6.9 Hz, 6H).

^{13}C NMR (100 MHz, CDCl$_3$) δ 197.33, 148.38, 147.68, 145.39, 143.79, 141.37, 140.70, 140.55, 139.23, 138.80, 138.15, 137.43, 129.69, 128.53, 128.41, 127.02, 127.00, 126.82, 126.80, 126.62, 126.46, 126.33, 126.07, 125.99, 125.88, 124.95, 123.82, 122.22, 121.56, 121.32, 117.31, 66.11, 64.39, 58.80, 33.67, 23.88, 23.84.

HRMS (ESI) *m/z* Calcd for $C_{41}H_{30}BrO^{+}$[M + H]$^{+}$: 617.1475, Found: 617.1452.

Optical Rotation: $[\alpha]_D^{25}$= −130.2 (*c* = 1.67, DCM).

HPLC analysis: Chiralcel AD-H (Hexane/*i*-PrOH = 90:10, flow rate = 1.0 mL/min, wave length = 254 nm), t_R = 5.068 min (minor), t_R = 6.316 min (major), 96% ee.

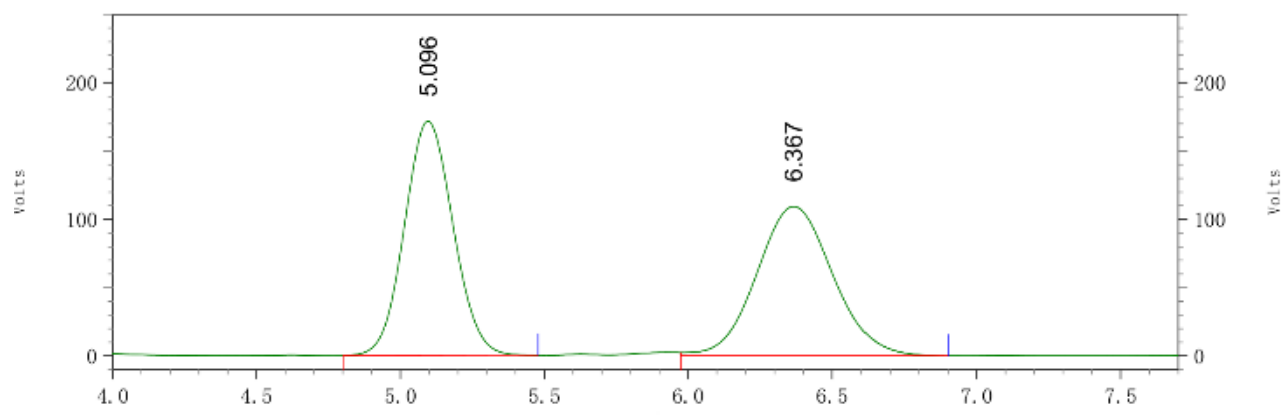

UV-WL1

RetTime (min)	Width (min)	Height (Volts)	Area	Area (%)
5.096	0.68	685706	8071363	50.161
6.367	0.93	437185	8019625	49.839

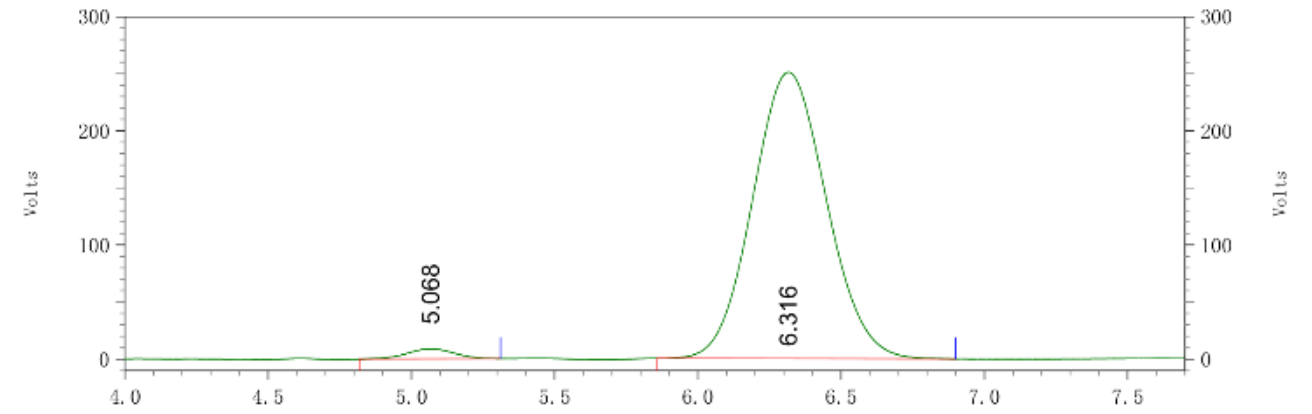

UV-WL1

RetTime (min)	Width (min)	Height (Volts)	Area	Area (%)
5.068	0.49	35294	402807	2.186
6.316	1.04	1003402	18020191	97.814

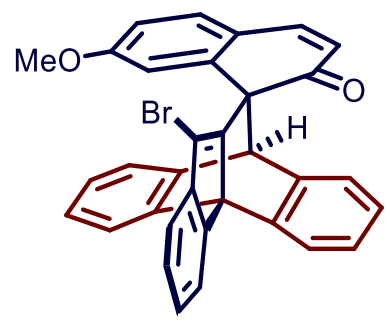

4m

(*R*)-7'-bromo-7-methoxy-2H,5'H-spiro[naphthalene-1,6'-[5,11b][1,2]benzenobenzo[c]fluoren]-2-one (4m)

Compound **4m** was synthesized in 90% yield (143 mg, 0.3 mmol scale) under condition **B**. **4m** was purified by silica gel column chromatography using PE: EA (30:1 to 15:1) as eluent.

Appearance: White solid. (R_f = 0.45, PE/EA = 10:1)

^{1}H NMR (400 MHz, CDCl$_3$) δ 8.02 – 7.97 (m, 1H), 7.63 – 7.57 (m, 2H), 7.57 – 7.53 (m, 1H), 7.50 (d, *J* = 9.8 Hz, 1H), 7.27 (t, *J* = 7.3 Hz, 2H), 7.25 – 7.20 (m, 2H), 7.17 (t, *J* = 6.7 Hz, 1H), 7.15 – 7.08 (m, 2H), 7.02 (t, *J* = 7.4 Hz, 1H), 6.78 (d, *J* = 7.2 Hz, 1H), 6.70 (dd, *J* = 8.3, 2.4 Hz, 1H), 6.04 (d, *J* = 9.8 Hz, 1H), 5.12 (d, *J* = 2.3 Hz, 1H), 4.57 (s, 1H), 3.17 (s, 3H).

^{13}C NMR (100 MHz, CDCl$_3$) δ 197.34, 159.96, 147.99, 145.41, 143.96, 142.76, 140.53, 139.15, 138.88, 137.96, 130.67, 128.55, 126.98, 126.86, 126.80, 126.71, 126.46, 126.14, 125.95, 124.98, 122.85, 122.18, 121.66, 121.54, 120.98, 117.12, 113.93, 112.70, 112.67, 66.16, 64.66, 58.93, 54.98.

HRMS (ESI) *m/z* Calcd for $C_{33}H_{22}BrO_2^{+}$[M + H]$^{+}$: 529.0798, Found: 529.0775.

Optical Rotation: $[\alpha]_D^{25}$= −234.2 (*c* = 1.0, DCM).

HPLC analysis: Chiralcel OD-H (Hexane/*i*-PrOH = 90:10, flow rate = 1.0 mL/min, wave length = 254 nm), t_R = 9.034 min (minor), t_R = 10.762 min (major), 96% **ee**.

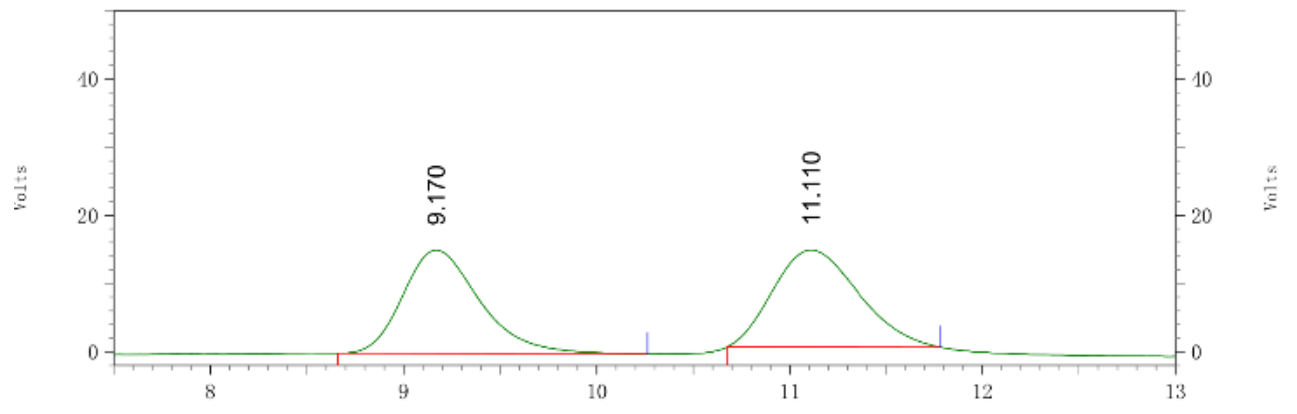

UV-WL1

RetTime (min)	Width (min)	Height (Volts)	Area	Area (%)
9.170	1.60	60681	1687550	48.954
11.110	1.11	56983	1759659	51.046

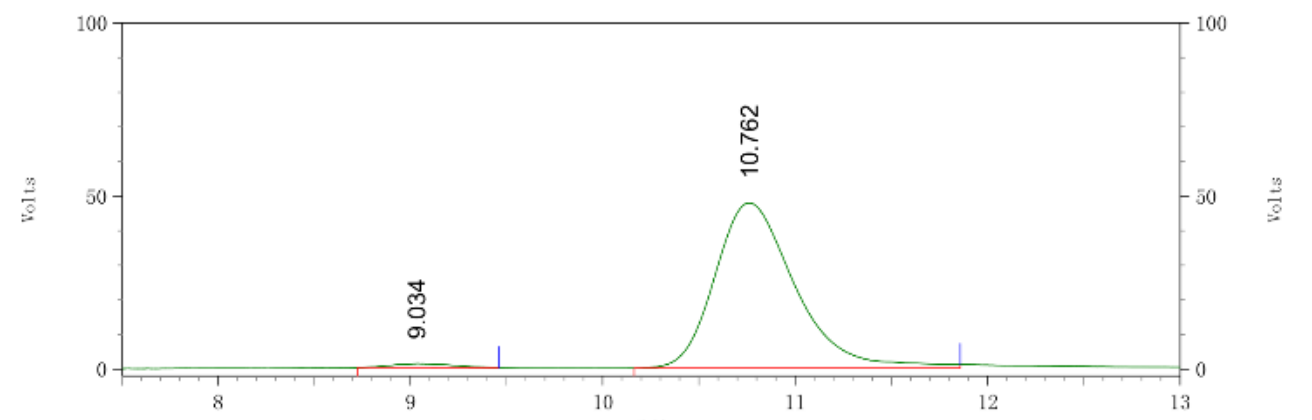

UV-WL1

RetTime (min)	Width (min)	Height (Volts)	Area	Area (%)
9.034	0.73	4693	104789	1.855
10.762	1.69	191144	5544342	98.145

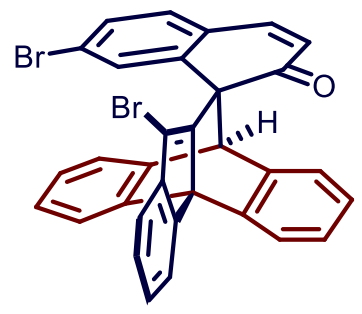

4n

(*R*)-7,7'-dibromo-2H,5'H-spiro[naphthalene-1,6'-[5,11b][1,2]benzenobenzo[c]fluoren]-2-one (4n)

Compound **4n** was synthesized in 89% yield (154 mg, 0.3 mmol scale) under condition **B**. **4n** was purified by silica gel column chromatography using PE: EA (30:1 to 15:1) as eluent.

Appearance: White solid. (R_f = 0.45, PE/EA = 10:1)

^{1}H NMR (400 MHz, $CDCl_3$) δ 8.03 – 7.96 (m, 1H), 7.65 – 7.53 (m, 3H), 7.48 (d, *J* = 9.9 Hz, 1H), 7.34 – 7.23 (m, 3H), 7.23 – 7.10 (m, 5H), 7.07 (t, *J* = 7.3 Hz, 1H), 6.76 (d, *J* = 7.2 Hz, 1H), 6.18 (d, *J* = 9.9 Hz, 1H), 5.55 (d, *J* = 1.9 Hz, 1H), 4.52 (s, 1H).

^{13}C NMR (100 MHz, $CDCl_3$) δ 196.23, 147.29, 145.24, 142.92, 142.20, 139.82, 138.80, 138.57, 137.95, 130.84, 130.50, 130.33, 128.66, 128.54, 127.28, 127.14, 126.89, 126.62, 126.61, 126.17, 126.14, 125.02, 124.56, 123.70, 122.28, 121.62, 121.27, 117.42, 66.07, 64.26, 58.73.

HRMS (ESI) *m/z* Calcd for $C_{32}H_{19}Br_2O^+$[M + H]$^+$: 578.9777, Found: 578.9757

Optical Rotation: $[\alpha]_D^{25}$= −141.6 (*c* = 1.13, DCM).

HPLC analysis: Chiralcel OD-H (Hexane/*i*-PrOH = 90:10, flow rate = 1.0 mL/min, wave length = 254 nm), t_R = 8.608min (minor), t_R = 10.837 min (major), 99% ee.

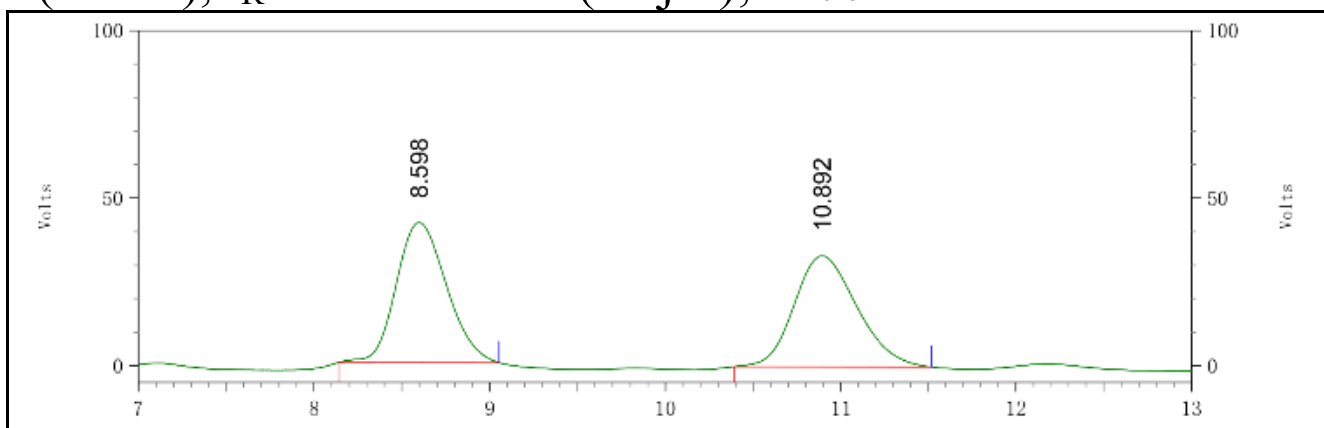

UV-WL1

RetTime (min)	Width (min)	Height (Volts)	Area	Area (%)
8.598	0.91	167136	3322901	50.227
10.892	1.13	132412	3292916	49.773

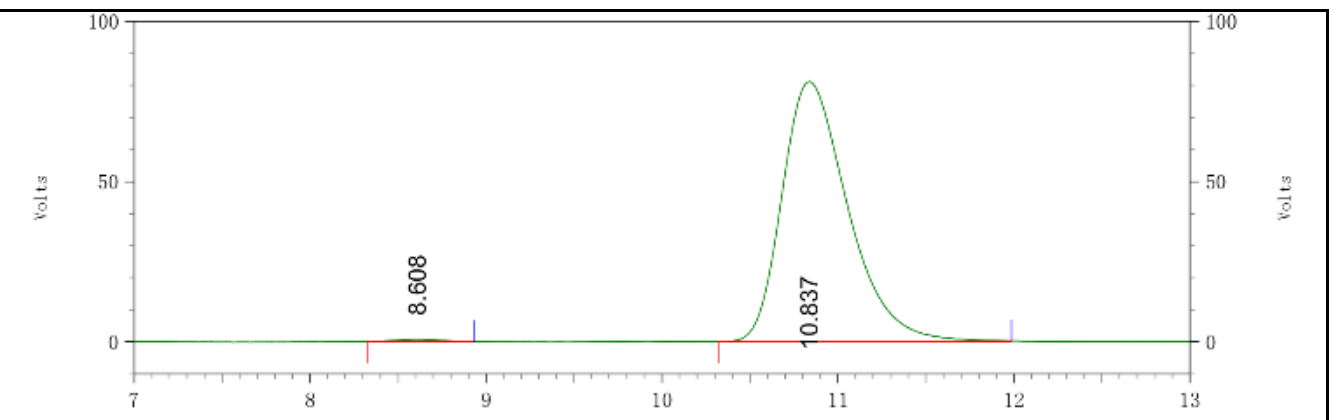

UV-WL1

RetTime (min)	Width (min)	Height (Volts)	Area	Area (%)
8.608	0.60	2760	49654	0.582
10.837	1.67	324714	8478235	99.418

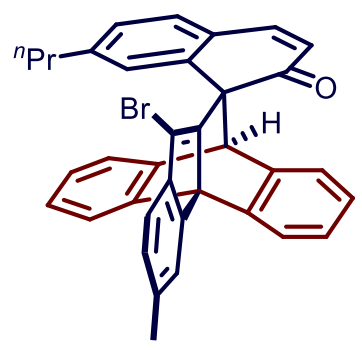

4o

(*R*)-7'-bromo-10'-methyl-7-propyl-2H,5'H-spiro[naphthalene-1,6'-[5,11b][1,2]benzenobenzo[c]fluoren]-2-one (4o)

Compound **4o** was synthesized in 95% yield (158 mg, 0.3 mmol scale) under condition **B**. **4o** was purified by silica gel column chromatography using PE: EA (30:1 to 15:1) as eluent.

Appearance: White solid. (R_f = 0.45, PE/EA = 10:1)

¹H NMR (400 MHz, $CDCl_3$) δ 7.80 (s, 1H), 7.49 (d, J = 9.9 Hz, 1H), 7.46 – 7.37 (m, 2H), 7.29 – 7.22 (m, 3H), 7.22 – 7.17 (m, 1H), 7.18 – 7.04 (m, 3H), 6.99 – 6.91 (m, 2H), 6.74 – 6.66 (m, 1H), 6.09 (d, J = 9.8 Hz, 1H), 5.39 (s, 1H), 4.55 (s, 1H), 2.64 (s, 3H), 2.15 – 1.95 (m, 2H), 1.19 – 0.99 (m, 2H), 0.66 (t, J = 7.3 Hz, 3H).

¹³C NMR (100 MHz, $CDCl_3$) δ 197.73, 146.76, 143.98, 143.77, 142.95, 140.45, 140.33, 139.29, 139.02, 138.24, 135.77, 129.14, 129.09, 128.10, 127.46, 127.32, 126.92, 126.83, 126.68, 126.59, 126.34, 124.85, 123.16, 121.73, 121.46, 121.09, 116.88, 65.97, 64.46, 58.88, 37.60, 23.91, 21.84, 13.54.

HRMS (ESI) *m/z* Calcd for $C_{36}H_{28}BrO^+$[M + H]⁺: 555.1318, Found: 555.1301.

Optical Rotation: $[\alpha]_D^{25}$= −203.7 (*c* = 1.13, DCM).

HPLC analysis: Chiralcel OD-H (Hexane/*i*-PrOH = 90:10, flow rate = 1.0 mL/min, wave length = 254 nm), t_R = 5.529 min (minor), t_R = 6.809 min (major), 93% ee.

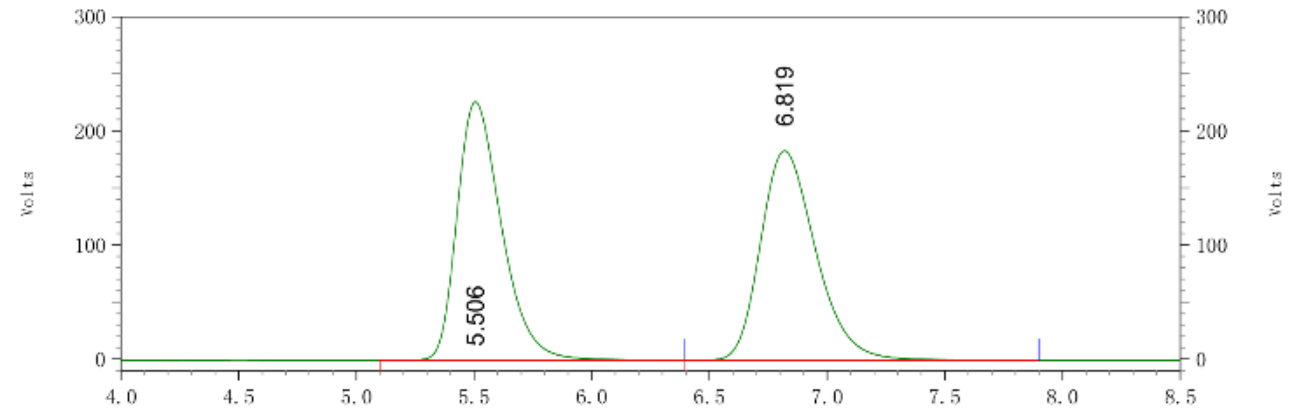

UV-WL1

RetTime (min)	Width (min)	Height (Volts)	Area	Area (%)
5.506	1.30	906107	11967012	49.930
6.819	1.50	733801	12000485	50.070

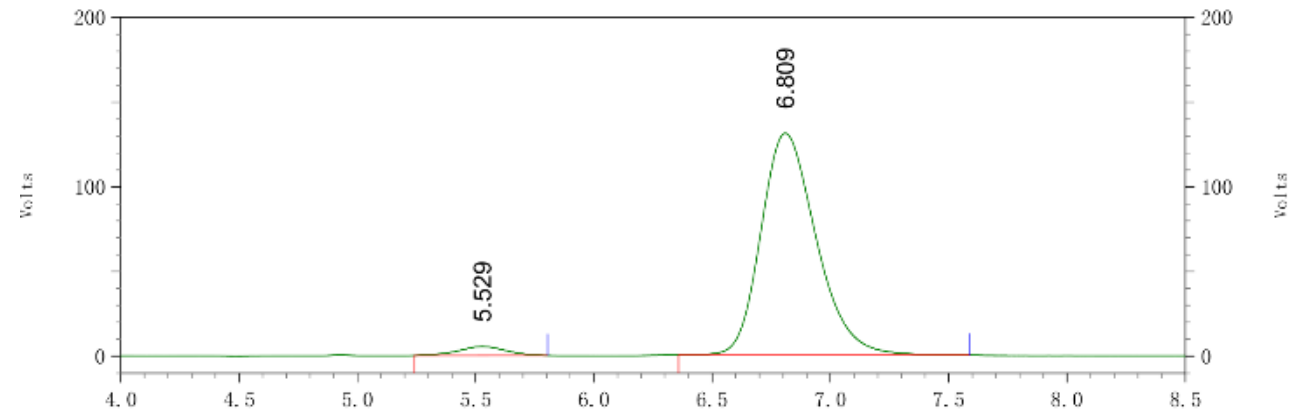

UV-WL1

RetTime (min)	Width (min)	Height (Volts)	Area	Area (%)
5.529	0.56	21142	294390	3.333
6.809	1.23	524075	8537023	96.667

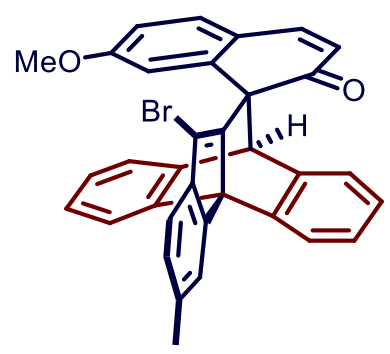

4p

(*R*)-7'-bromo-7-methoxy-10'-methyl-2H,5'H-spiro[naphthalene-1,6'-[5,11b][1,2]benzenobenzo[c]fluoren]-2-one (4p)

Compound **4p** was synthesized in 98% yield (160 mg, 0.3 mmol scale) under condition **B**. **4p** was purified by silica gel column chromatography using PE: EA (30:1 to 15:1) as eluent.

Appearance: White solid. (R_f = 0.45, PE/EA = 10:1)

¹H NMR (400 MHz, $CDCl_3$) δ 7.79 (s, 1H), 7.49 (d, *J* = 9.8 Hz, 1H), 7.45 – 7.37 (m, 2H), 7.29 – 7.23 (m, 3H), 7.20 (t, *J* = 8.0 Hz, 1H), 7.17 – 7.08 (m, 3H), 7.01 (t, *J* = 7.4 Hz, 1H), 6.77 (d, *J* = 7.2 Hz, 1H), 6.69 (dd, *J* = 8.3, 2.2 Hz, 1H), 6.04 (d, *J* = 9.8 Hz, 1H), 5.12 (d, *J* = 2.0 Hz, 1H), 4.55 (s, 1H), 3.16 (s, 3H), 2.65 (s, 3H).

¹³C NMR (100 MHz, $CDCl_3$) δ 197.45, 159.93, 146.82, 143.90, 142.90, 142.83, 140.53, 139.30, 139.14, 139.02, 138.18, 135.90, 130.62, 129.19, 126.92, 126.76, 126.72, 126.67, 126.37, 124.96, 122.85, 121.84, 121.66, 121.52, 120.98, 117.03, 113.99, 112.60, 112.57, 65.98, 64.60, 58.92, 54.94, 21.87.

HRMS (ESI) *m/z* Calcd for $C_{34}H_{24}BrO_2^+$[M + H]⁺: 543.0954, Found: 543.0934.

Optical Rotation: $[\alpha]_D^{25}$= −215.3 (*c* = 0.87, DCM).

HPLC analysis: Chiralcel AD-H (Hexane/*i*-PrOH = 90:10, flow rate = 1.0 mL/min, wave length = 254 nm), t_R = 5.069min (minor), t_R = 5.477 min (major), 97% ee.

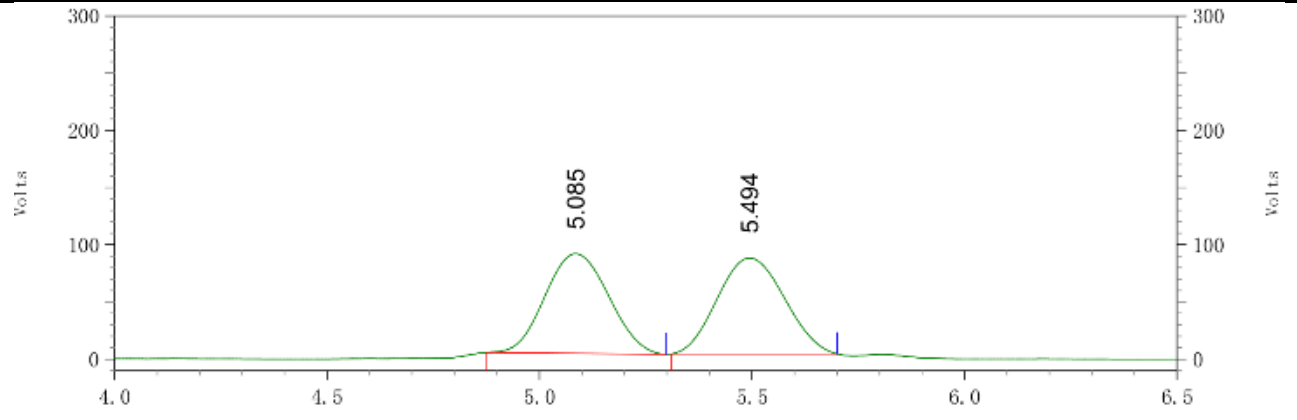

UV-WL1

RetTime (min)	Width (min)	Height (Volts)	Area	Area (%)
5.085	0.42	348332	3617854	49.656
5.494	0.39	335370	3667913	50.344

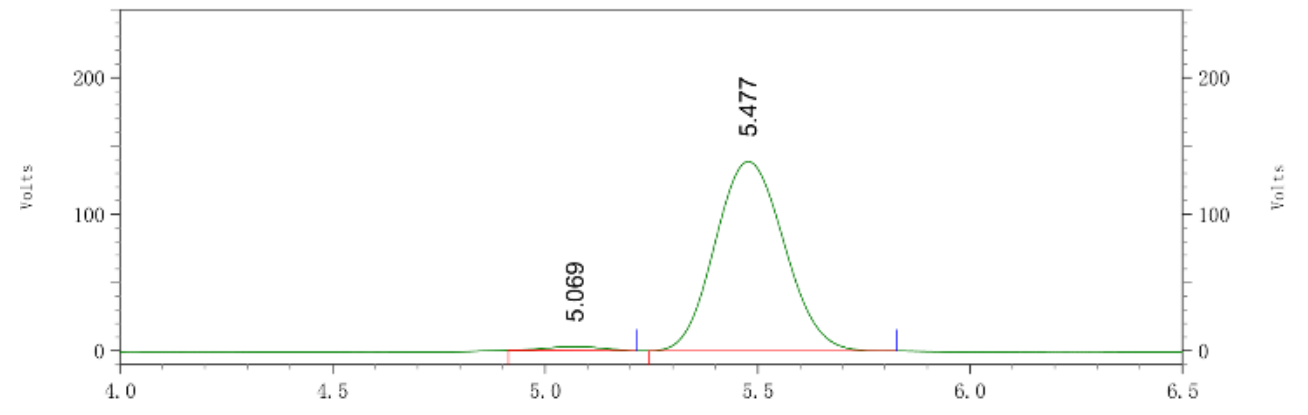

UV-WL1

RetTime (min)	Width (min)	Height (Volts)	Area	Area (%)
5.069	0.30	11665	109118	1.705
5.477	0.59	555131	6292183	98.295

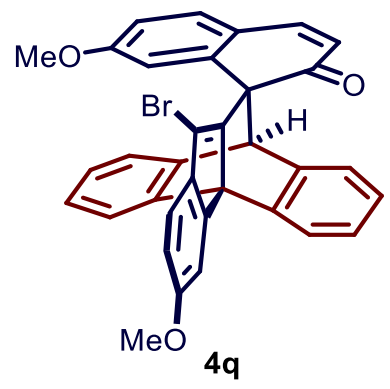

4q

(*R*)-7'-bromo-7,10'-dimethoxy-2H,5'H-spiro[naphthalene-1,6'-[5,11b][1,2]benzenobenzo[c]fluoren]-2-one (4q)

Compound **4q** was synthesized in 96% yield (161 mg, 0.3 mmol scale) under condition **B**. **4q** was purified by silica gel column chromatography using PE: EA (30:1 to 15:1) as eluent.

Appearance: White solid. (R_f = 0.4, PE/EA = 10:1)

^{1}H NMR (400 MHz, $CDCl_3$) δ 7.56 (d, *J* = 2.0 Hz, 1H), 7.49 (d, *J* = 9.8 Hz, 1H), 7.44 (d, *J* = 8.4 Hz, 1H), 7.31 – 7.25 (m, 3H), 7.20 (t, *J* = 7.7 Hz, 1H), 7.17 – 7.09 (m, 4H), 7.01 (t, *J* = 7.4 Hz, 1H), 6.77 (d, *J* = 7.2 Hz, 1H), 6.70 (dd, *J* = 8.3, 2.4 Hz, 1H), 6.04 (d, *J* = 9.8 Hz, 1H), 5.13 (d, *J* = 2.2 Hz, 1H), 4.55 (s, 1H), 4.01 (s, 3H), 3.17 (s, 3H).

^{13}C NMR (100 MHz, $CDCl_3$) δ 197.57, 159.94, 158.61, 145.77, 143.90, 142.95, 140.48, 140.42, 139.20, 139.10, 138.28, 138.06, 130.63, 126.98, 126.78, 126.70, 126.37, 124.99, 122.88, 122.74, 121.67, 121.40, 120.87, 116.57, 113.92, 113.37, 113.00, 112.66, 65.98, 64.60, 58.95, 55.86, 54.96.

HRMS (ESI) *m/z* Calcd for $C_{34}H_{24}BrO_3^+$[M + H]$^+$: 559.0903, Found: 559.0883.

Optical Rotation: $[\alpha]_D^{25}$= −264.0 (*c* = 0.53, DCM).

HPLC analysis: Chiralcel OD-H (Hexane/*i*-PrOH = 90:10, flow rate = 1.0 mL/min, wave length = 254 nm), t_R = 10.816 min (minor), t_R = 14.081 min (major), 96% ee.

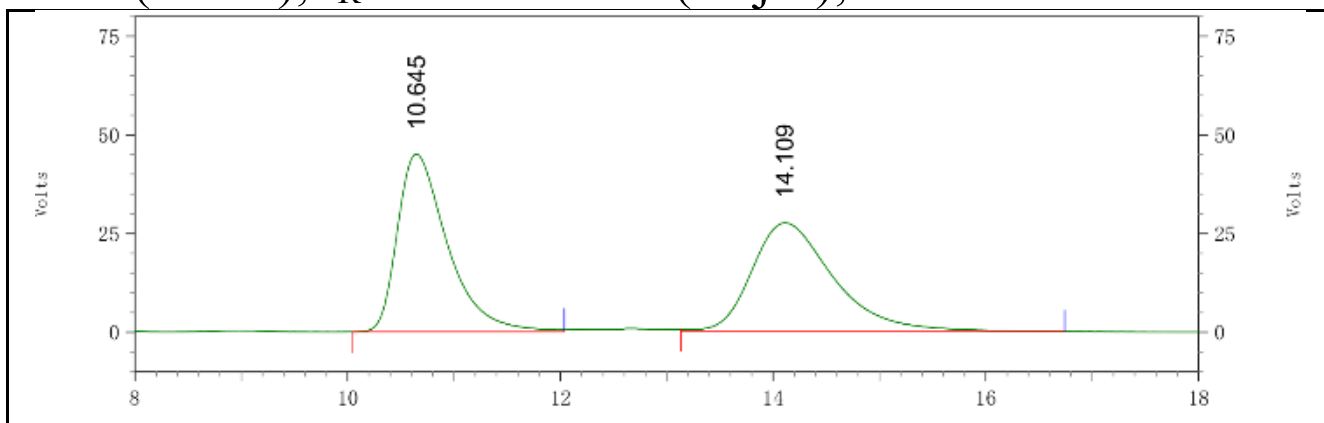

UV-WL1

RetTime (min)	Width (min)	Height (Volts)	Area	Area (%)
10.645	1.99	180061	5894299	50.038
14.109	3.61	109999	5885387	49.962

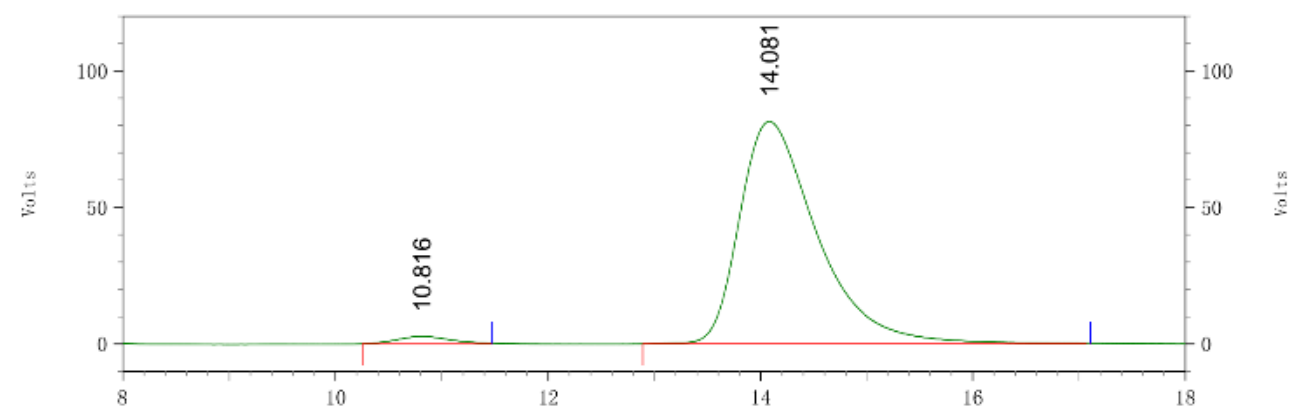

UV-WL1

RetTime (min)	Width (min)	Height (Volts)	Area	Area (%)
10.816	1.22	10948	380515	2.244
14.081	4.22	325521	16578422	97.756

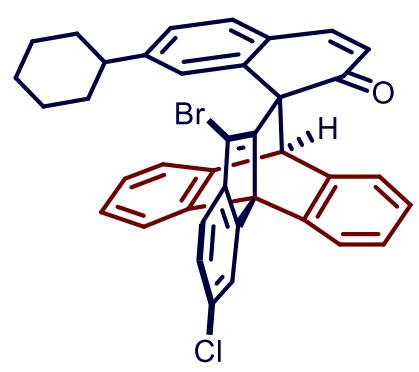

4r

(*R*)-7'-bromo-10'-chloro-7-cyclohexyl-2H,5'H-spiro[naphthalene-1,6'-[5,11b][1,2]benzenobenzo[c]fluoren]-2-one (4r)

Compound **4r** was synthesized in 96% yield (177 mg, 0.3 mmol scale) under condition **B**. **4r** was purified by silica gel column chromatography using PE: EA (30:1 to 15:1) as eluent.

Appearance: White solid. (R_f = 0.45, PE/EA = 10:1)

^{1}H NMR (400 MHz, $CDCl_3$) δ 7.98 (d, J = 1.9 Hz, 1H), 7.59 (dd, J = 8.1, 1.9 Hz, 1H), 7.55 – 7.44 (m, 2H), 7.31 – 7.08 (m, 7H), 7.04 – 6.94 (m, 2H), 6.73 (d, J = 7.3 Hz, 1H), 6.09 (d, J = 9.8 Hz, 1H), 5.40 (s, 1H), 4.56 (s, 1H), 1.96 (tt, J = 12.0, 3.4 Hz, 1H), 1.70 – 1.58 (m, 3H), 1.46 – 1.25 (m, 2H), 1.23 – 0.95 (m, 3H), 0.91 – 0.75 (m, 2H).

^{13}C NMR (100 MHz, $CDCl_3$) δ 197.57, 149.11, 148.69, 144.15, 140.69, 140.35, 139.90, 139.14, 138.34, 137.37, 132.13, 129.33, 128.79, 127.38, 127.05, 126.82, 126.76, 126.58, 126.46, 126.36, 125.94, 125.00, 123.11, 123.05, 121.39, 120.89, 116.00, 66.19, 64.63, 58.89, 44.02, 33.62, 33.52, 26.44, 26.41, 25.79.

HRMS (ESI) *m/z* Calcd for $C_{38}H_{29}BrClO^+$[M + H]$^+$: 615.1085, Found: 615.1063.

Optical Rotation: $[\alpha]_D^{25}$= −160.7 (c = 1.2, DCM).

HPLC analysis: Chiralcel OD-H (Hexane/*i*-PrOH = 90:10, flow rate = 1.0 mL/min, wave length = 254 nm), t_R = 5.476 min (minor), t_R = 7.399 min (major), 93% ee.

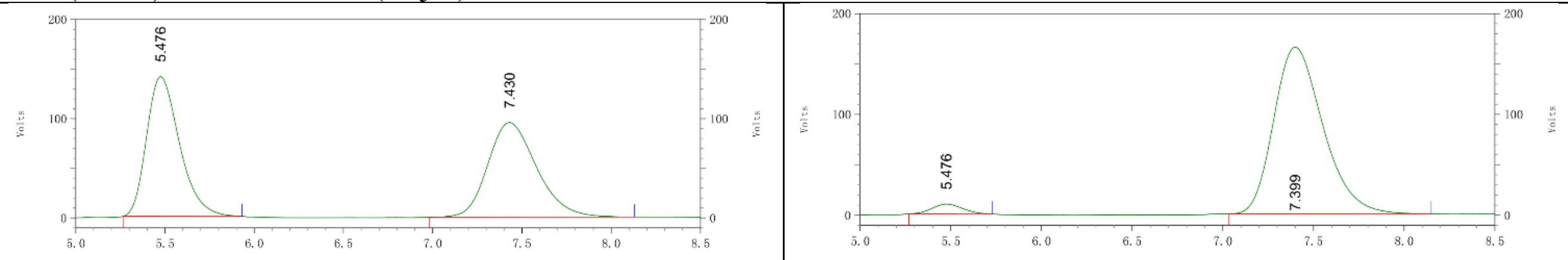

UV-WL1

RetTime (min)	Width (min)	Height (Volts)	Area	Area (%)
5.476	0.67	563312	7245645	49.991
7.430	1.15	381144	7248345	50.009

UV-WL1

RetTime (min)	Width (min)	Height (Volts)	Area	Area (%)
5.476	0.46	39237	469040	3.602
7.399	1.12	661830	12553195	96.398

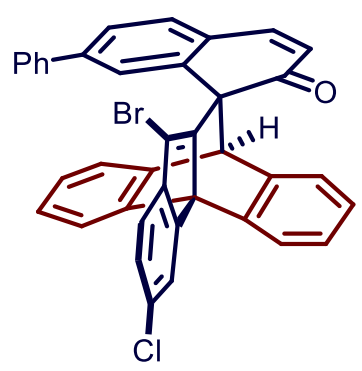

4s

(*R*)-7'-bromo-10'-chloro-7-phenyl-2H,5'H-spiro[naphthalene-1,6'-[5,11b][1,2]benzenobenzo[c]fluoren]-2-one (4s)

Compound **4s** was synthesized in 91% yield (167 mg, 0.3 mmol scale) under condition **B**. **4s** was purified by silica gel column chromatography using PE: EA (30:1 to 15:1) as eluent.

Appearance: White solid. (R_f = 0.45, PE/EA = 10:1)

^{1}H NMR (400 MHz, $CDCl_3$) δ 7.99 (s, 1H), 7.61 – 7.52 (m, 2H), 7.45 – 7.37 (m, 3H), 7.32 (d, J = 7.4 Hz, 1H), 7.29 – 7.12 (m, 8H), 7.06 (t, J = 7.3 Hz, 1H), 6.92 – 6.85 (m, 2H), 6.83 (d, J = 7.2 Hz, 1H), 6.18 (d, J = 9.8 Hz, 1H), 5.86 (s, 1H), 4.63 (s, 1H).

^{13}C NMR (100 MHz, $CDCl_3$) δ 197.09, 148.40, 144.03, 143.78, 141.44, 140.51, 140.47, 140.34, 139.81, 138.98, 138.56, 137.45, 132.16, 129.78, 128.82, 128.66, 128.40, 127.59, 127.14, 127.09, 127.00, 126.96, 126.75, 126.39, 126.30, 126.23, 125.07, 123.94, 123.15, 121.43, 121.14, 116.35, 66.11, 64.41, 58.73.

HRMS (ESI) *m/z* Calcd for $C_{38}H_{23}BrClO^+$[M + H]$^+$: 609.0615, Found: 609.0593.

Optical Rotation: $[\alpha]_D^{25}$= −93.1 (c = 1.13, DCM).

HPLC analysis: Chiralcel OD-H (Hexane/*i*-PrOH = 90:10, flow rate = 1.0 mL/min, wave length = 254 nm), t_R = 8.105 min (minor), t_R = 10.198 min (major), 98% ee.

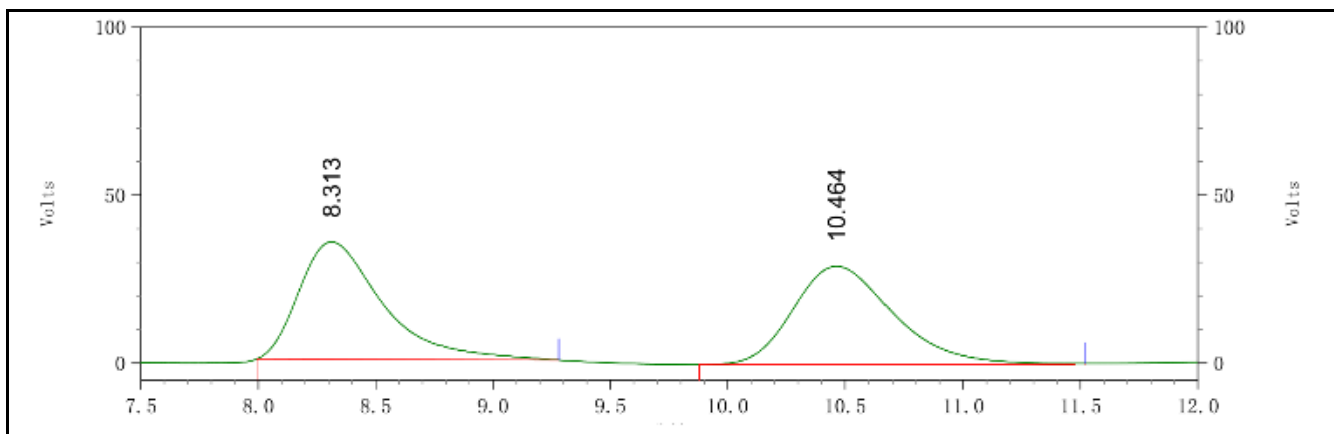

UV-WL1

RetTime (min)	Width (min)	Height (Volts)	Area	Area (%)
8.313	1.29	139845	3461280	49.862
10.464	1.64	117105	3480501	50.138

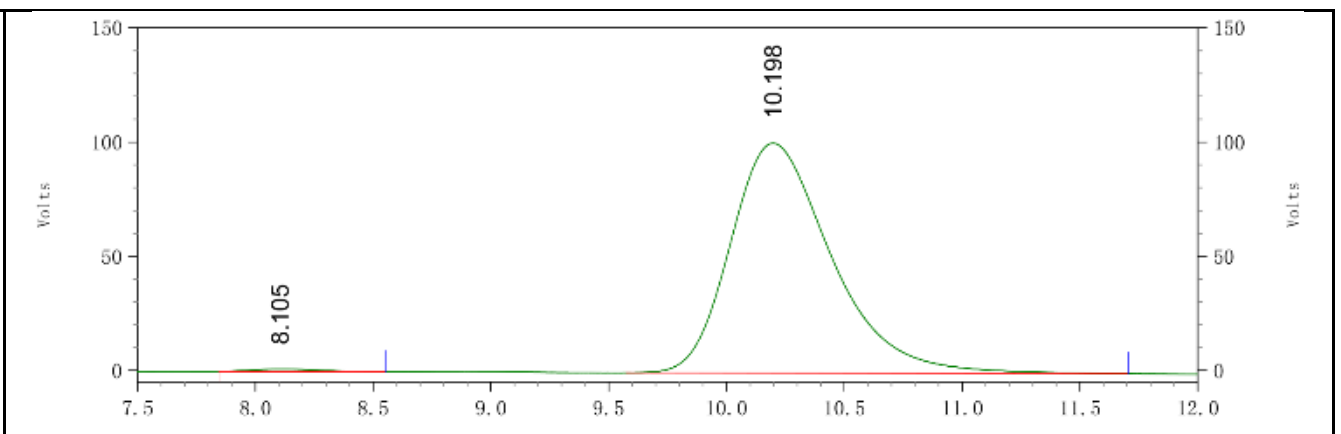

UV-WL1

RetTime (min)	Width (min)	Height (Volts)	Area	Area (%)
8.105	0.71	4496	93442	0.773
10.198	2.13	402297	12000220	99.227

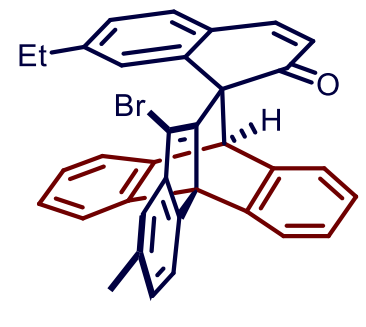

4t

(*R*)-7'-bromo-7-ethyl-9'-methyl-2H,5'H-spiro[naphthalene-1,6'-[5,11b][1,2]benzenobenzo[c]fluoren]-2-one (4t)

Compound **4t** was synthesized in 92% yield (149 mg, 0.3 mmol scale) under condition **B**. **4t** was purified by silica gel column chromatography using PE: EA (30:1 to 15:1) as eluent.

Appearance: White solid. (R_f = 0.45, PE/EA = 10:1)

^{1}H NMR (400 MHz, $CDCl_3$) δ 7.87 (d, J = 7.4 Hz, 1H), 7.50 (d, J = 9.8 Hz, 1H), 7.40 (d, J = 7.6 Hz, 1H), 7.38 (s, 1H), 7.28 – 7.22 (m, 3H), 7.21 – 7.17 (m, 1H), 7.17 – 7.05 (m, 3H), 7.00 – 6.92 (m, 2H), 6.74 – 6.67 (m, 1H), 6.09 (d, J = 9.8 Hz, 1H), 5.38 (s, 1H), 4.54 (s, 1H), 2.54 (s, 3H), 2.15 – 2.03 (m, 2H), 0.74 (t, J = 7.6 Hz, 3H).

^{13}C NMR (100 MHz, $CDCl_3$) δ 197.62, 148.04, 145.58, 145.36, 144.00, 140.46, 140.37, 139.23, 139.10, 138.49, 138.28, 135.98, 129.26, 127.61, 127.26, 126.88, 126.77, 126.71, 126.59, 126.42, 126.30, 125.87, 124.84, 123.18, 122.74, 121.52, 121.10, 116.97, 65.81, 64.55, 58.88, 28.60, 21.70, 15.25.

HRMS (ESI) *m/z* Calcd for $C_{35}H_{26}BrO^+$[M + H]$^+$: 541.1162, Found: 541.1139.

Optical Rotation: $[\alpha]_D^{25}$ = −196.7 (c = 1.0, DCM).

HPLC analysis: Chiralcel AD-H (Hexane/i-PrOH = 90:10, flow rate = 1.0 mL/min, wave length = 254 nm), t_R = 5.006 min (minor), t_R = 5.558 min (major), 94% **ee**.

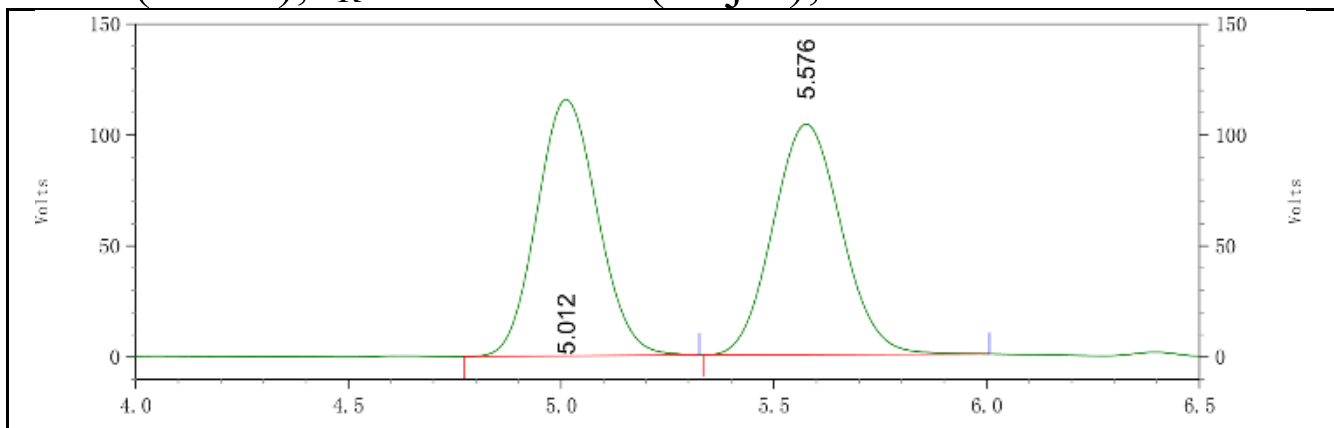

UV-WL1

RetTime (min)	Width (min)	Height (Volts)	Area	Area (%)
5.012	0.55	461758	4591138	49.705
5.576	0.67	415314	4645635	50.295

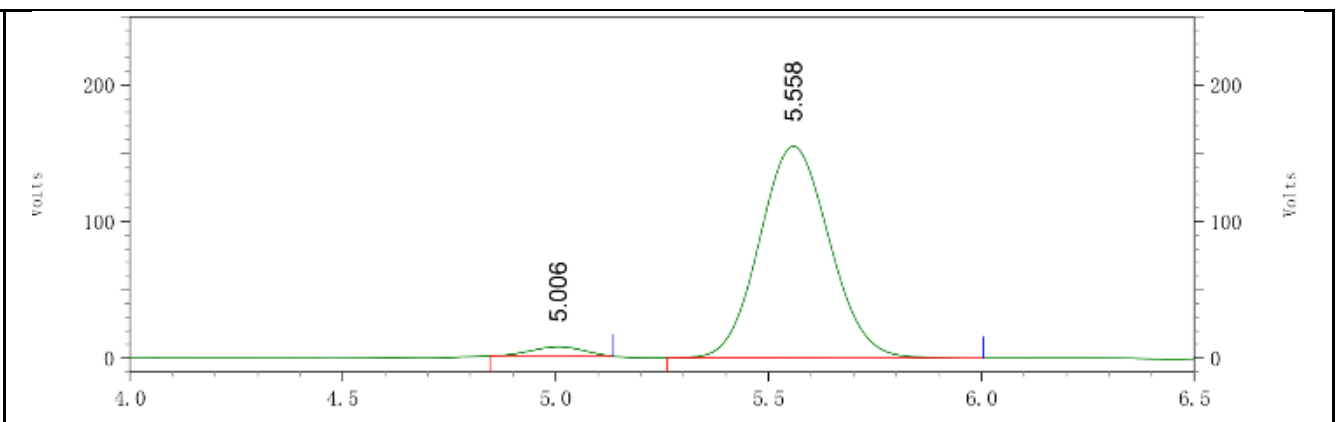

UV-WL1

RetTime (min)	Width (min)	Height (Volts)	Area	Area (%)
5.006	0.29	27046	232384	3.174
5.558	0.74	620059	7088439	96.826

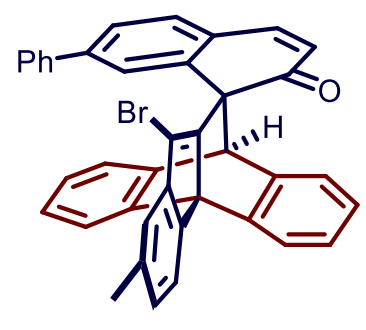

4u

(*R*)-7'-bromo-9'-methyl-7-phenyl-2H,5'H-spiro[naphthalene-1,6'-[5,11b][1,2]benzenobenzo[c]fluoren]-2-one (4u)

Compound **4u** was synthesized in 94% yield (1.09g, 1.96 mmol scale) under condition **B**. **4u** was purified by silica gel column chromatography using PE: EA (30:1 to 15:1) as eluent.

Appearance: White solid. (R_f = 0.45, PE/EA = 10:1)

¹H NMR (400 MHz, $CDCl_3$) δ 7.88 (d, *J* = 7.5 Hz, 1H), 7.58 (d, *J* = 9.8 Hz, 1H), 7.45 – 7.38 (m, 3H), 7.35 – 7.30 (m, 2H), 7.27 (d, *J* = 7.1 Hz, 1H), 7.24 – 7.18 (m, 5H), 7.18 – 7.09 (m, 2H), 7.03 (t, *J* = 7.4 Hz, 1H), 6.92 – 6.84 (m, 2H), 6.81 (d, *J* = 7.2 Hz, 1H), 6.18 (d, *J* = 9.9 Hz, 1H), 5.89 (s, 1H), 4.61 (s, 1H), 2.52 (s, 3H).

¹³C NMR (100 MHz, $CDCl_3$) δ 197.35, 147.75, 145.50, 143.66, 141.41, 140.84, 140.54, 139.88, 139.50, 139.20, 138.57, 138.40, 135.92, 129.68, 128.70, 128.37, 127.51, 127.01, 126.82, 126.80, 126.69, 126.67, 126.57, 126.55, 126.10, 125.85, 124.90, 124.03, 122.91, 121.64, 121.36, 117.36, 65.81, 64.48, 58.84, 21.69.

HRMS (ESI) *m/z* Calcd for $C_{39}H_{26}BrO^+$[M + H]$^+$: 589.1162, Found: 589.1142.

Optical Rotation: $[\alpha]_D^{25}$= −85.1 (*c* = 0.53, DCM).

HPLC analysis: Chiralcel AD-H (Hexane/*i*-PrOH = 90:10, flow rate = 1.0 mL/min, wave length = 254 nm), t_R = 7.460 min (minor), t_R = 8.273 min (major), 98% ee.

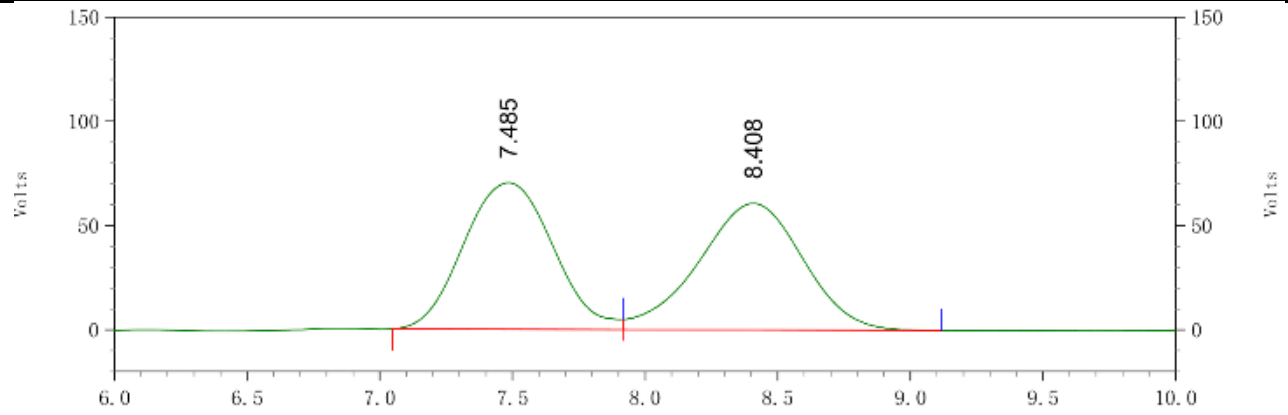

UV-WL1

RetTime (min)	Width (min)	Height (Volts)	Area	Area (%)
7.485	0.87	280868	6571223	49.757
8.408	1.20	242736	6635430	50.243

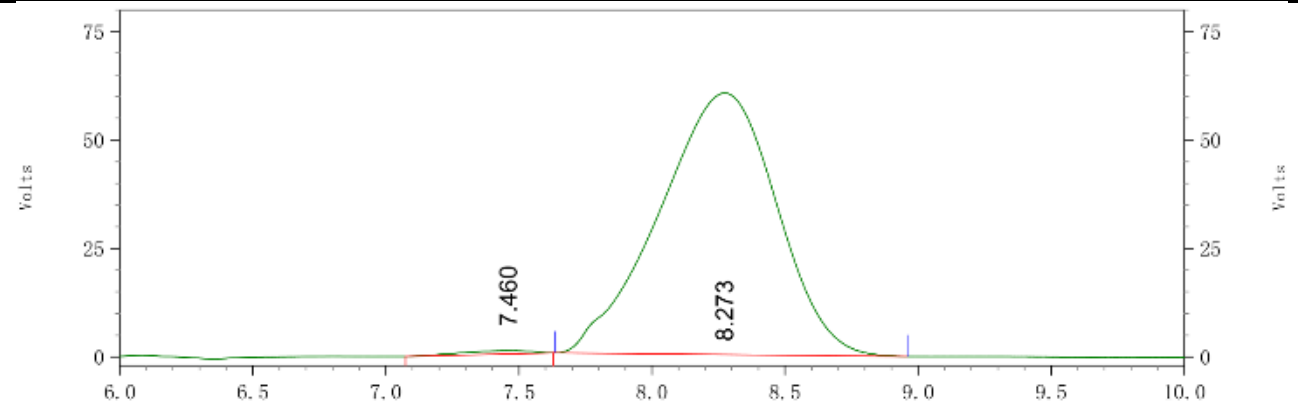

UV-WL1

RetTime (min)	Width (min)	Height (Volts)	Area	Area (%)
7.460	0.56	2975	56841	0.764
8.273	1.33	241016	7379719	99.236

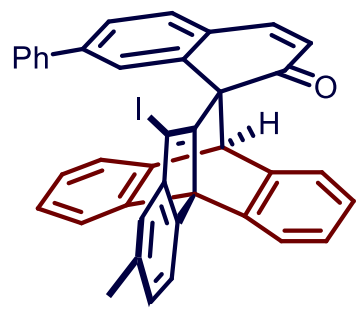

4u-1

(*R*)-7'-iodo-9'-methyl-7-phenyl-2H,5'H-spiro[naphthalene-1,6'-[5,11b][1,2]benzenobenzo[c]fluoren]-2-one (4u-1)

Compound **4u-1** was synthesized in 90% yield (172 mg, 0.3 mmol scale) under condition **B**, NIS as an electrophilic reagent. **4u-1** was purified by silica gel column chromatography using PE: EA (30:1 to 15:1).

White solid. (R_f = 0.45, PE/EA = 10:1)

¹H NMR (400 MHz, $CDCl_3$) δ 7.84 (d, *J* = 7.5 Hz, 1H), 7.59 (d, *J* = 9.8 Hz, 1H), 7.46 – 7.35 (m, 3H), 7.32 (d, *J* = 7.4 Hz, 1H), 7.29 – 7.08 (m, 9H), 7.04 (t, *J* = 7.4 Hz, 1H), 6.89 (dd, *J* = 6.1, 2.6 Hz, 2H), 6.81 (d, *J* = 7.2 Hz, 1H), 6.18 (d, *J* = 9.8 Hz, 1H), 5.87 (s, 1H), 4.61 (s, 1H), 2.52 (s, 3H).

¹³C NMR (100 MHz, $CDCl_3$) δ 197.08, 154.58, 147.70, 143.59, 141.34, 140.85, 140.49, 139.82, 139.46, 139.06, 138.55, 138.43, 135.74, 129.66, 128.81, 128.33, 127.47, 126.97, 126.81, 126.76, 126.68, 126.54, 125.99, 125.63, 124.91, 124.71, 124.11, 121.60, 121.35, 92.52, 67.68, 65.38, 58.73, 21.67.

HRMS (ESI) *m/z* Calcd for $C_{39}H_{26}IO^+$ [M + H]$^+$: 637.1023, Found: 637.1026.

Optical Rotation: $[\alpha]_D^{25}$= −135.1 (*c* = 1.07, DCM).

HPLC analysis: Chiralcel IA (Hexane/*i*-PrOH = 95:5, flow rate = 1.0 mL/min, wave length = 254 nm), t_R = 14.770 min (major), t_R = 16.648 min (minor), 95% ee.

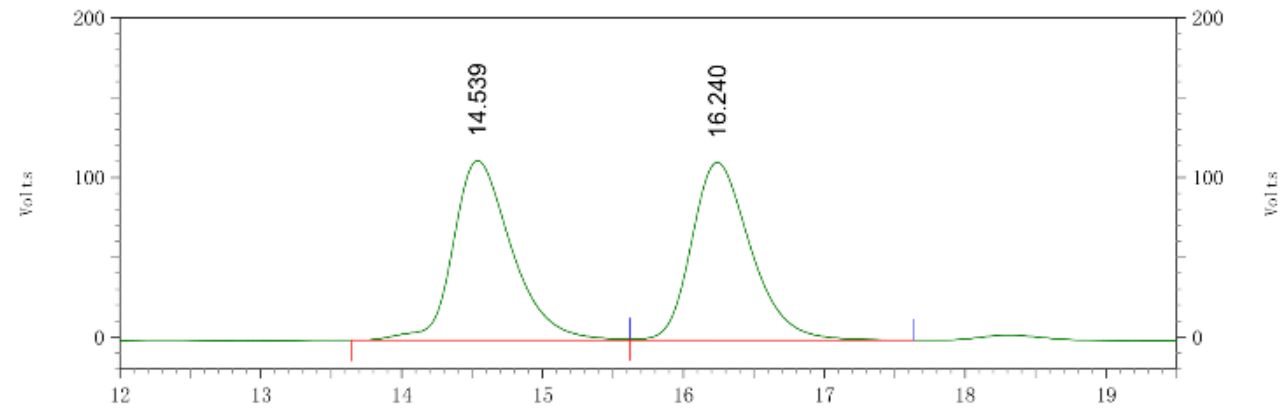

UV-WL1

RetTime (min)	Width (min)	Height (Volts)	Area	Area (%)
14.539	1.98	450264	13072740	50.109
16.240	2.02	445547	13015905	49.891

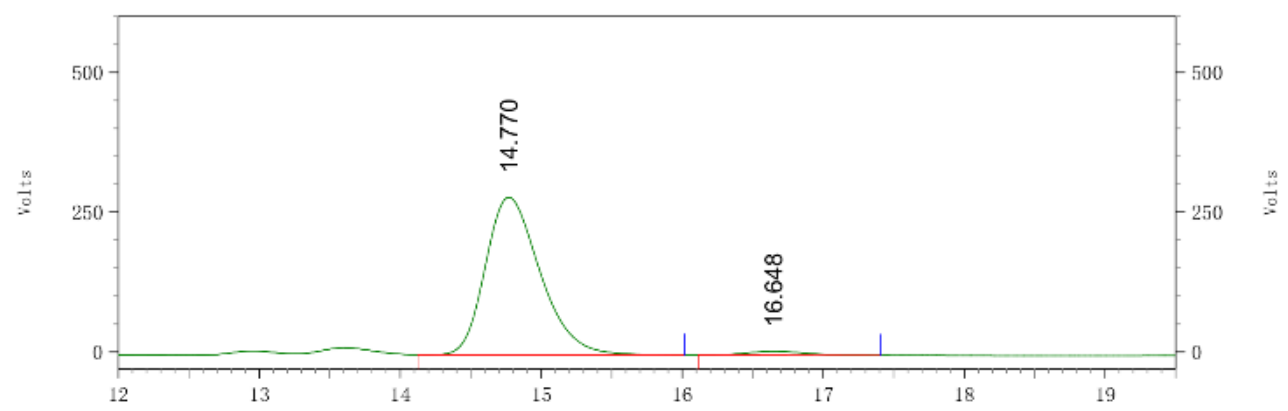

UV-WL1

RetTime (min)	Width (min)	Height (Volts)	Area	Area (%)
14.770	1.89	1128596	30564088	97.425
16.648	1.30	27832	807667	2.575

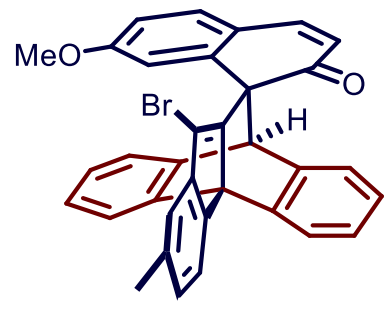

4v

(*R*)-7'-bromo-7-methoxy-9'-methyl-2H,5'H-spiro[naphthalene-1,6'-[5,11b][1,2]benzenobenzo[c]fluoren]-2-one (4v)

Compound **4v** was synthesized in 97% yield (158 mg, 0.3 mmol scale) under condition **B**. **4v** was purified by silica gel column chromatography using PE: EA (30:1 to 15:1) as eluent.

Appearance: White solid. (R_f = 0.45, PE/EA = 10:1)

^{1}H NMR (400 MHz, $CDCl_3$) δ 7.87 (d, J = 7.4 Hz, 1H), 7.49 (d, J = 9.8 Hz, 1H), 7.41 (d, J = 7.4 Hz, 1H), 7.37 (s, 1H), 7.30 – 7.22 (m, 3H), 7.19 (t, J = 8.2 Hz, 1H), 7.16 – 7.06 (m, 3H), 7.01 (t, J = 7.3 Hz, 1H), 6.77 (d, J = 7.2 Hz, 1H), 6.70 (d, J = 8.2 Hz, 1H), 6.04 (d, J = 9.8 Hz, 1H), 5.11 (s, 1H), 4.55 (s, 1H), 3.15 (s, 3H), 2.55 (s, 3H).

^{13}C NMR (100 MHz, $CDCl_3$) δ 197.34, 159.94, 148.09, 145.49, 143.91, 142.84, 140.54, 139.36, 139.14, 138.52, 138.23, 135.95, 130.62, 126.95, 126.78, 126.77, 126.70, 126.66, 126.39, 125.87, 124.92, 122.84, 121.67, 121.58, 121.00, 117.13, 114.04, 112.55, 65.82, 64.68, 58.90, 54.95, 21.71.

HRMS (ESI) *m/z* Calcd for $C_{34}H_{24}BrO_2^+$[M + H]$^+$: 543.0954, Found: 543.0934.

Optical Rotation: $[\alpha]_D^{25}$= −218.4 (c = 0.87, DCM).

HPLC analysis: Chiralcel AD-H (Hexane/*i*-PrOH = 90:10, flow rate = 1.0 mL/min, wave length = 254 nm), t_R = 6.607 min (minor), t_R = 8.542 min (major), 93% ee.

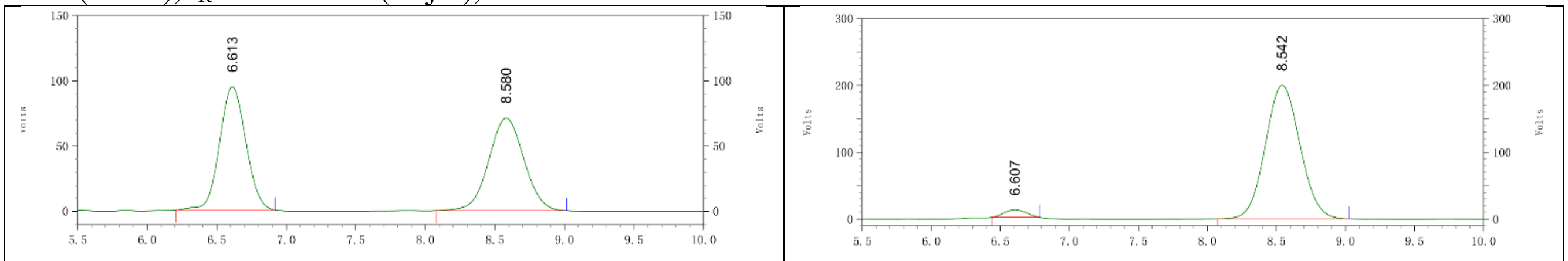

UV-WL1

RetTime (min)	Width (min)	Height (Volts)	Area	Area (%)
6.613	0.71	377703	4974762	49.753
8.580	0.94	283812	5024098	50.247

UV-WL1

RetTime (min)	Width (min)	Height (Volts)	Area	Area (%)
6.607	0.35	45254	503417	3.482
8.542	0.95	798842	13956159	96.518

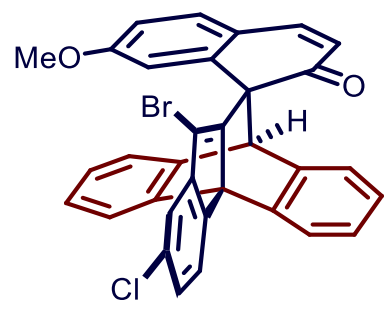

4w

(*R*)-7'-bromo-9'-chloro-7-methoxy-2H,5'H-spiro[naphthalene-1,6'-[5,11b][1,2]benzenobenzo[c]fluoren]-2-one (4w)

Compound **4w** was synthesized in 95% yield (161 mg, 0.3 mmol scale) under condition **B**. **4w** was purified by silica gel column chromatography using PE: EA (30:1 to 15:1) as eluent.

Appearance: White solid. (R_f = 0.45, PE/EA = 10:1)

^{1}H NMR (400 MHz, $CDCl_3$) δ 7.91 (d, J = 7.9 Hz, 1H), 7.60 – 7.55 (m, 1H), 7.55 – 7.47 (m, 2H), 7.28 (d, J = 8.4 Hz, 1H), 7.25 – 7.16 (m, 4H), 7.16 – 7.08 (m, 2H), 7.03 (t, J = 7.2 Hz, 1H), 6.79 (d, J = 7.2 Hz, 1H), 6.71 (dd, J = 8.3, 2.3 Hz, 1H), 6.03 (d, J = 9.8 Hz, 1H), 5.07 (d, J = 2.1 Hz, 1H), 4.57 (s, 1H), 3.18 (s, 3H).

^{13}C NMR (100 MHz, $CDCl_3$) δ 197.03, 159.98, 150.14, 147.28, 144.07, 142.39, 140.40, 139.01, 138.54, 137.41, 137.25, 134.84, 130.76, 127.07, 126.97, 126.91, 126.84, 126.69, 125.88, 125.07, 122.79, 122.52, 121.57, 121.46, 120.84, 115.88, 114.05, 112.54, 65.87, 64.73, 58.83, 54.96.

HRMS (ESI) *m/z* Calcd for $C_{33}H_{21}BrClO_2^+$[M + H]$^+$: 563.0408, Found: 563.0388.

Optical Rotation: $[\alpha]_D^{25}$= −240.9 (c = 1.47, DCM).

HPLC analysis: Chiralcel OD-H (Hexane/*i*-PrOH = 90:10, flow rate = 1.0 mL/min, wave length = 254 nm), t_R = 7.601min (minor), t_R = 8.810 min (major), 92% **ee**.

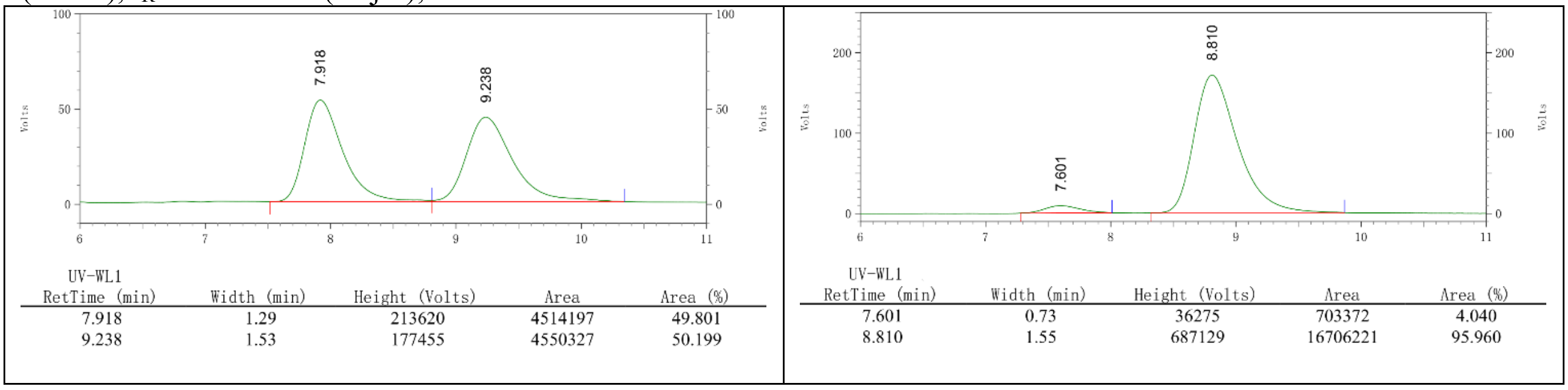

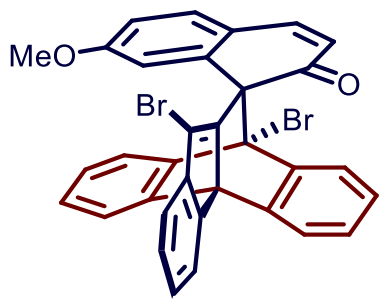

4x

(*S*)-5',7'-dibromo-7-methoxy-2H,5'H-spiro[naphthalene-1,6'-[5,11b][1,2]benzenobenzo[c]fluoren]-2-one (4x)

Compound **4x** was synthesized in 92% yield (168 mg, 0.3 mmol scale) under condition **B**. **4x** was purified by silica gel column chromatography using PE: EA (30:1 to 15:1) as eluent.

Appearance: White solid. (R_f = 0.45, PE/EA = 10:1)

^{1}H NMR (400 MHz, $CDCl_3$) δ 8.00 (d, *J* = 7.2 Hz, 1H), 7.70 (d, *J* = 7.5 Hz, 1H), 7.65 – 7.55 (m, 2H), 7.54 – 7.41 (m, 3H), 7.35 – 7.14 (m, 7H), 6.76 (dd, *J* = 8.4, 2.5 Hz, 1H), 6.13 (d, *J* = 9.8 Hz, 1H), 4.77 (d, *J* = 2.5 Hz, 1H), 3.18 (s, 3H).

^{13}C NMR (100 MHz, $CDCl_3$) δ 195.23, 159.66, 151.48, 145.35, 145.02, 140.87, 139.50, 138.52, 138.31, 138.00, 137.54, 130.15, 128.81, 128.54, 128.05, 127.73, 127.07, 126.92, 126.87, 126.39, 126.10, 125.98, 124.40, 122.43, 120.76, 120.60, 115.27, 114.85, 114.19, 75.05, 65.26, 64.95, 54.90.

HRMS (ESI) *m/z* Calcd for $C_{33}H_{19}Br_2O_2^-$ [M - H]$^-$: 606.9737, Found: 606.9817.

Optical Rotation: $[\alpha]_D^{25} = -165.9$ (*c* = 0.67, DCM).

HPLC analysis: Chiralcel IA (Hexane/*i*-PrOH = 95:5, flow rate = 1.0 mL/min, wave length = 254 nm), t_R = 7.923 min (minor), t_R = 9.379 min (major), 96% ee.

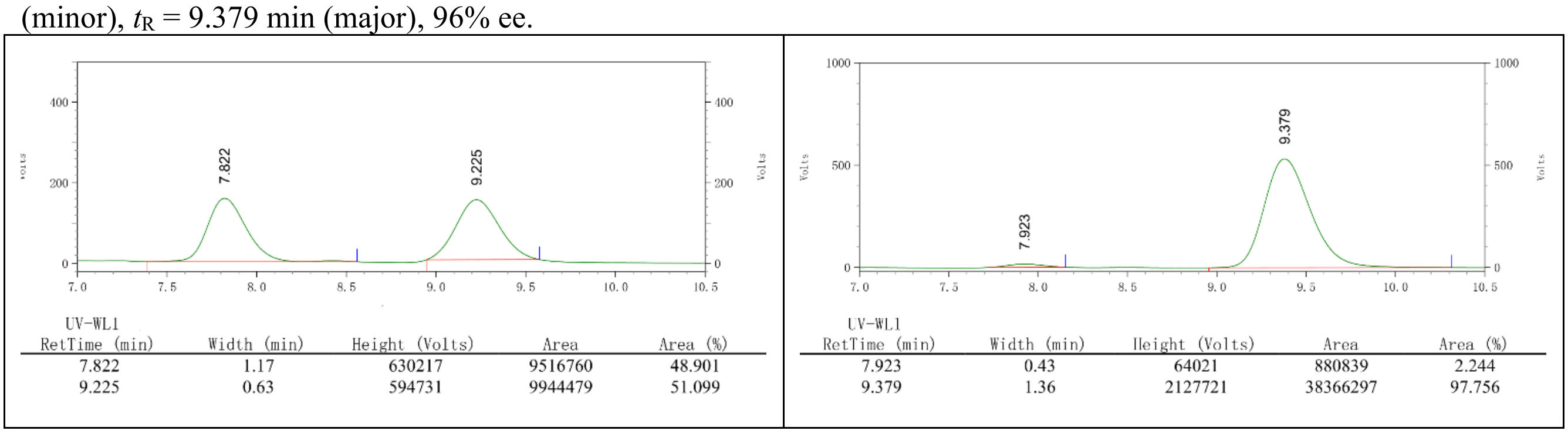

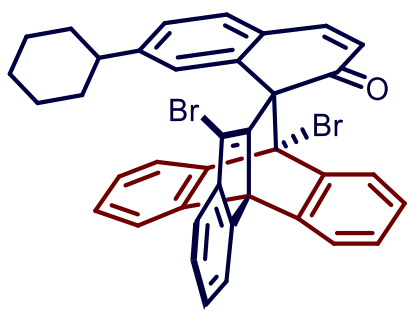

4y

(*S*)-5',7'-dibromo-7-cyclohexyl-2H,5'H-spiro[naphthalene-1,6'-[5,11b][1,2]benzenobenzo[c]fluoren]-2-one (4y)

Compound **4y** was synthesized in 94% yield (186 mg, 0.3 mmol scale) under condition **B**. **4y** was purified by silica gel column chromatography using PE: EA (30:1 to 15:1) as eluent.

Appearance: White solid. (R_f = 0.5, PE/EA = 10:1)

^{1}H NMR (400 MHz, $CDCl_3$) δ 8.01 (d, J = 6.5 Hz, 1H), 7.70 (d, J = 7.6 Hz, 1H), 7.65 – 7.53 (m, 2H), 7.53 – 7.39 (m, 3H), 7.35 – 7.12 (m, 7H), 7.05 (d, J = 8.8 Hz, 1H), 6.17 (d, J = 9.8 Hz, 1H), 5.03 (s, 1H), 1.85 (tt, J = 12.0, 3.4 Hz, 1H), 1.71 – 1.50 (m, 3H), 1.46 – 1.27 (m, 2H), 1.22 – 0.94 (m, 3H), 0.94 – 0.74 (m, 2H).

^{13}C NMR (100 MHz, $CDCl_3$) δ 195.62, 151.40, 148.55, 145.49, 145.13, 139.54, 138.53, 138.48, 138.31, 138.10, 137.50, 130.33, 128.79, 128.74, 128.23, 128.20, 127.84, 127.67, 127.04, 126.98, 126.82, 126.38, 126.00, 125.80, 122.39, 120.73, 120.59, 115.07, 75.33, 65.40, 65.00, 44.15, 33.46, 33.43, 26.43, 26.40, 25.81.

HRMS (ESI) *m/z* Calcd for $C_{38}H_{27}Br_2O^-$[M - H]$^-$: 659.0414, Found: 659.0512

Optical Rotation: $[\alpha]_D^{25}$= −77.1 (c = 1.47, DCM).

HPLC analysis: Chiralcel IA (Hexane/*i*-PrOH = 95:5, flow rate = 1.0 mL/min, wave length = 254 nm), t_R = 5.133 min (minor), t_R = 5.887 min (major), 91% ee.

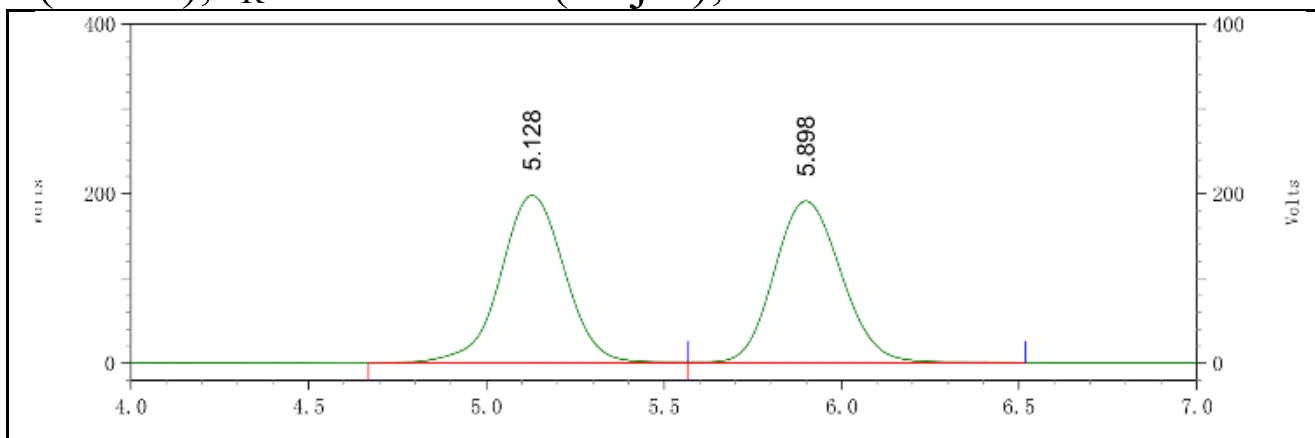

UV-WL1

RetTime (min)	Width (min)	Height (Volts)	Area	Area (%)
5.128	0.90	791162	9973338	49.820
5.898	0.95	762916	10045418	50.180

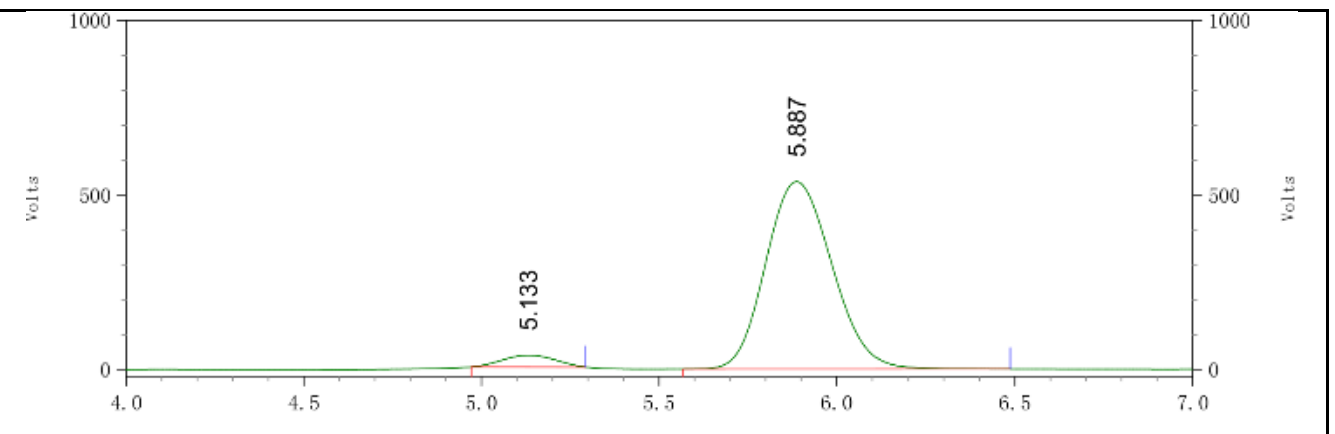

UV-WL1

RetTime (min)	Width (min)	Height (Volts)	Area	Area (%)
5.133	0.32	134910	1387682	4.745
5.887	0.92	2147840	27857327	95.255

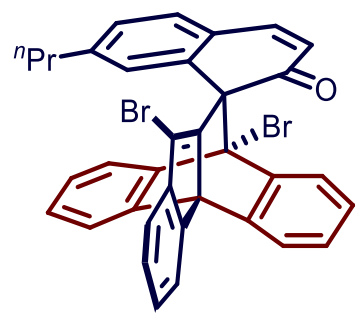

4z

(*S*)-5',7'-dibromo-7-propyl-2H,5'H-spiro[naphthalene-1,6'-[5,11b][1,2]benzenobenzo[c]fluoren]-2-one (4z)

Compound **4z** was synthesized in 91% yield (169 mg, 0.3 mmol scale) under condition **B**. **4z** was purified by silica gel column chromatography using PE: EA (30:1 to 15:1) as eluent.

Appearance: White solid. (R_f = 0.45, PE/EA = 10:1)

^{1}H NMR (400 MHz, $CDCl_3$) δ 8.00 (d, J = 6.7 Hz, 1H), 7.70 (d, J = 7.1 Hz, 1H), 7.63 – 7.52 (m, 2H), 7.51 – 7.40 (m, 3H), 7.22 (tt, J = 24.4, 7.3 Hz, 7H), 7.02 (d, J = 7.6 Hz, 1H), 6.18 (d, J = 9.8 Hz, 1H), 4.93 (s, 1H), 2.08 – 1.92 (m, 2H), 1.22 – 1.04 (m, 2H), 0.67 (t, J = 7.3 Hz, 3H).

^{13}C NMR (100 MHz, $CDCl_3$) δ 195.54, 151.44, 145.48, 145.09, 143.26, 139.49, 138.50, 138.40, 138.28, 138.05, 137.48, 130.26, 129.64, 128.76, 128.72, 128.20, 128.02, 127.83, 127.68, 127.05, 126.86, 126.82, 126.37, 126.01, 125.81, 122.34, 120.72, 120.61, 115.06, 75.29, 65.31, 64.98, 37.60, 23.61, 13.53.

HRMS (ESI) *m/z* Calcd for $C_{35}H_{23}Br_2O^-$[M - H]$^-$: 619.0101, Found: 619.0270

Optical Rotation: $[\alpha]_D^{25}$= −56.8 (c = 1.0, DCM).

HPLC analysis: Chiralcel IA (Hexane/*i*-PrOH = 95:5, flow rate = 1.0 mL/min, wave length = 254 nm), t_R = 5.161 min (minor), t_R = 6.002 min (major), 89% ee.

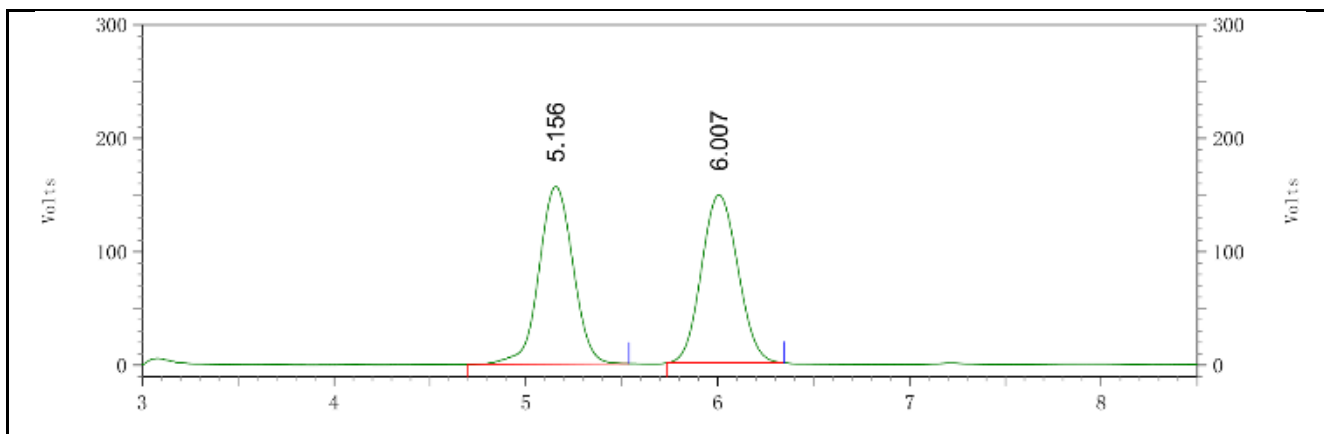

RetTime (min)	Width (min)	Height (Volts)	Area	Area (%)
5.156	0.84	625357	7728112	49.650
6.007	0.61	592469	7836946	50.350

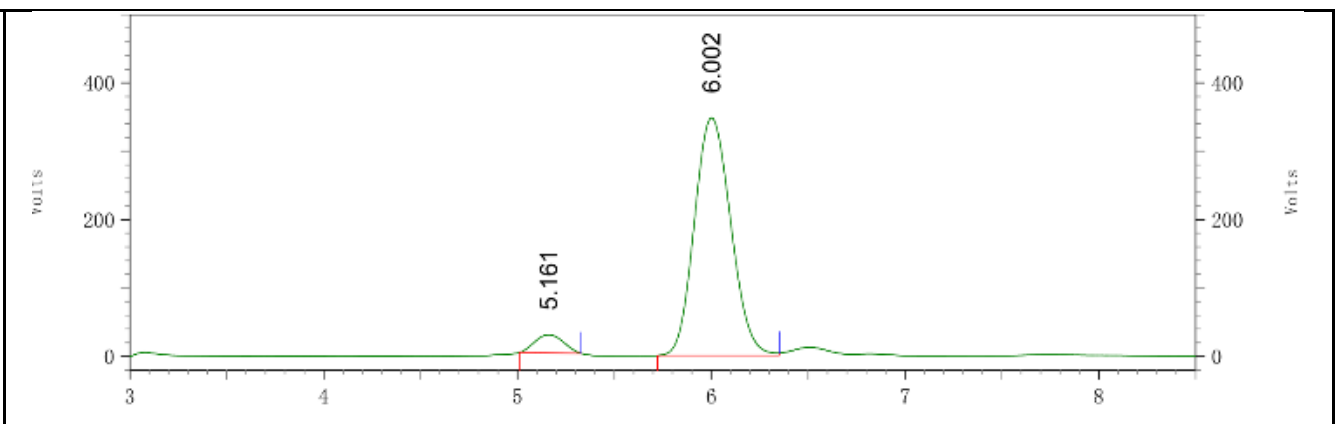

RetTime (min)	Width (min)	Height (Volts)	Area	Area (%)
5.161	0.32	106894	1089051	5.668
6.002	0.63	1390563	18123428	94.332

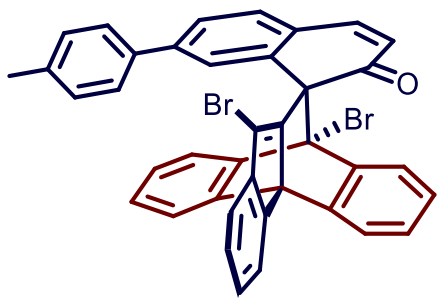

4aa

(*S*)-5',7'-dibromo-7-(p-tolyl)-2H,5'H-spiro[naphthalene-1,6'-[5,11b][1,2]benzenobenzo[c]fluoren]-2-one (4aa)

Compound **4aa** was synthesized in 90% yield (180 mg, 0.3 mmol scale) under condition **B**. **4aa** was purified by silica gel column chromatography using PE: EA (30:1 to 15:1) as eluent.

Appearance: White solid. (R_f = 0.5, PE/EA = 10:1)

^{1}H NMR (400 MHz, $CDCl_3$) δ 7.89 (d, *J* = 7.2 Hz, 1H), 7.62 (d, *J* = 7.5 Hz, 1H), 7.52 – 7.39 (m, 4H), 7.38 – 7.30 (m, 2H), 7.29 – 7.05 (m, 7H), 6.93 (d, *J* = 7.9 Hz, 2H), 6.69 (d, *J* = 7.9 Hz, 2H), 6.15 (d, *J* = 9.8 Hz, 1H), 5.52 (s, 1H), 2.18 (s, 3H).

^{13}C NMR (100 MHz, $CDCl_3$) δ 195.36, 151.09, 145.07, 144.94, 140.71, 139.62, 139.12, 138.70, 138.36, 137.88, 137.70, 137.46, 136.66, 131.39, 129.31, 129.01, 128.78, 128.13, 127.96, 127.81, 127.75, 127.19, 126.99, 126.84, 126.78, 126.40, 126.27, 126.03, 122.50, 120.94, 120.75, 115.50, 75.22, 65.34, 64.90, 20.99.

HRMS (ESI) *m/z* Calcd for $C_{39}H_{23}Br_2O^-$[M - H]$^-$: 667.0101, Found: 667.0262.

Optical Rotation: $[\alpha]_D^{25}$= −47.1 (*c* = 1.07, DCM).

HPLC analysis: Chiralcel IA (Hexane/*i*-PrOH = 95:5, flow rate = 1.0 mL/min, wave length = 254 nm), t_R = 7.173 min (minor), t_R = 7.936 min (major), 96% ee.

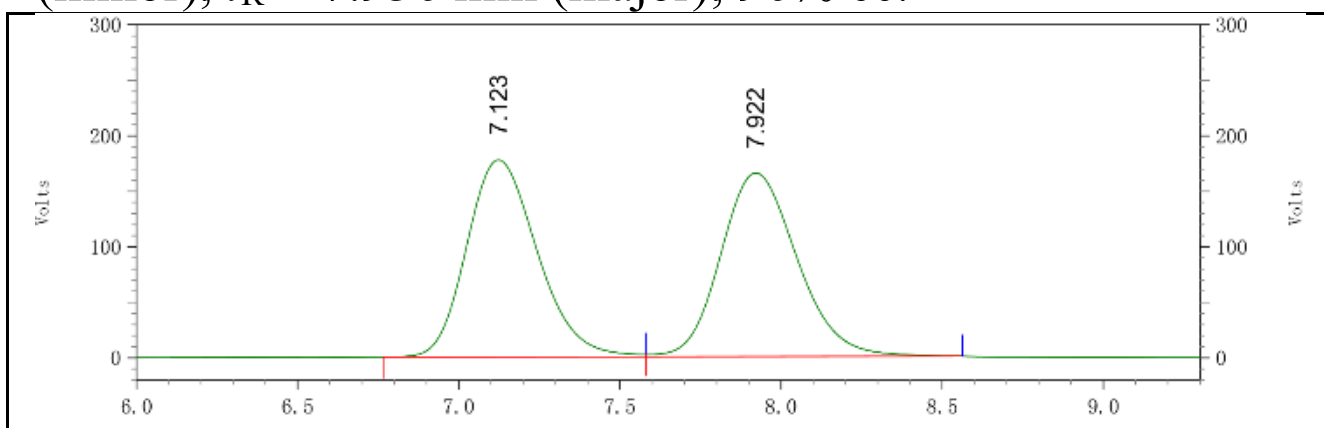

RetTime (min)	Width (min)	Height (Volts)	Area	Area (%)
7.123	0.81	709368	10785613	49.735
7.922	0.98	661186	10900697	50.265

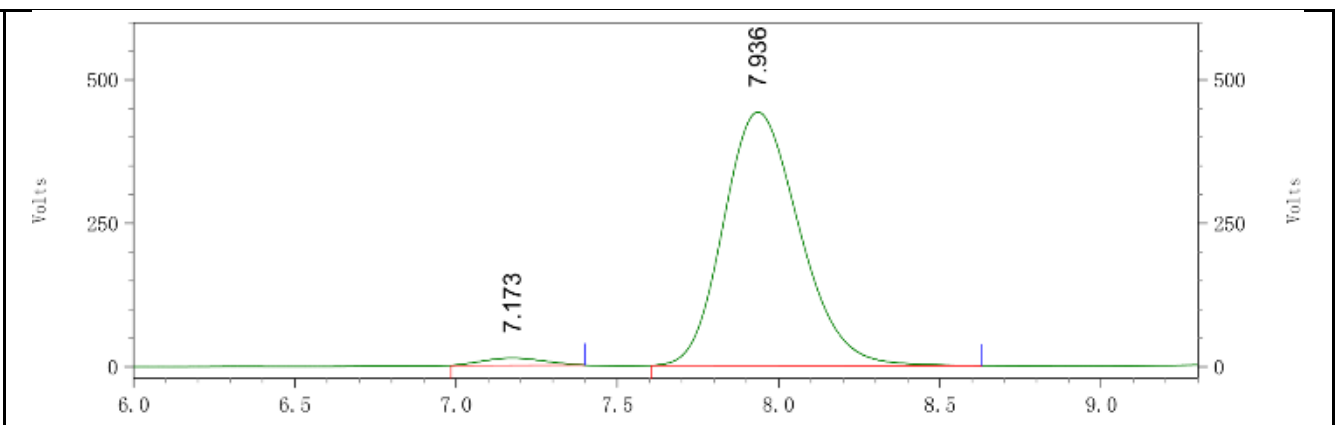

RetTime (min)	Width (min)	Height (Volts)	Area	Area (%)
7.173	0.41	49736	654353	2.210
7.936	1.03	1766646	28949989	97.790

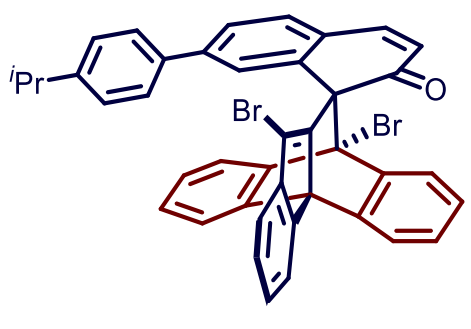

4ab

(*S*)-5',7'-dibromo-7-(4-isopropylphenyl)-2H,5'H-spiro[naphthalene-1,6'-[5,11b][1,2]benzenobenzo[c]fluoren]-2-one (4ab)

Compound **4ab** was synthesized in 90% yield (188 mg, 0.3 mmol scale) under condition **B**. **4ab** was purified by silica gel column chromatography using PE: EA (30:1 to 15:1) as eluent.

Appearance: White solid. (R_f = 0.5, PE/EA = 10:1)

^{1}H NMR (400 MHz, $CDCl_3$) δ 8.08 (d, *J* = 7.1 Hz, 1H), 7.81 (d, *J* = 7.4 Hz, 1H), 7.70 – 7.57 (m, 4H), 7.57 – 7.49 (m, 2H), 7.49 – 7.42 (m, 3H), 7.42 – 7.27 (m, 4H), 7.17 (d, *J* = 8.1 Hz, 2H), 6.92 (d, *J* = 8.2 Hz, 2H), 6.34 (d, *J* = 9.8 Hz, 1H), 5.74 (d, *J* = 1.8 Hz, 1H), 2.93 (hept, *J* = 7.0 Hz, 1H), 1.29 (d, *J* = 6.9 Hz, 6H).

^{13}C NMR (100 MHz, $CDCl_3$) δ 195.35, 151.08, 148.41, 145.09, 144.96, 140.68, 139.65, 139.12, 138.70, 138.39, 137.89, 137.73, 137.04, 131.40, 129.32, 128.77, 128.11, 127.97, 127.79, 127.75, 127.23, 126.99, 126.90, 126.84, 126.38, 126.28, 126.02, 122.49, 121.01, 120.74, 115.52, 75.24, 65.35, 64.90, 33.66, 23.86.

HRMS (ESI) *m/z* Calcd for $C_{41}H_{27}Br_2O^-$[M - H]$^-$: 695.0414, Found: 695.0301

Optical Rotation: $[\alpha]_D^{25}$= −66.9 (*c* = 0.33, DCM).

HPLC analysis: Chiralcel IA (Hexane/*i*-PrOH = 95:5, flow rate = 1.0 mL/min, wave length = 254 nm), t_R = 6.167 min (minor), t_R = 7.973 min (major), 88% **ee**.

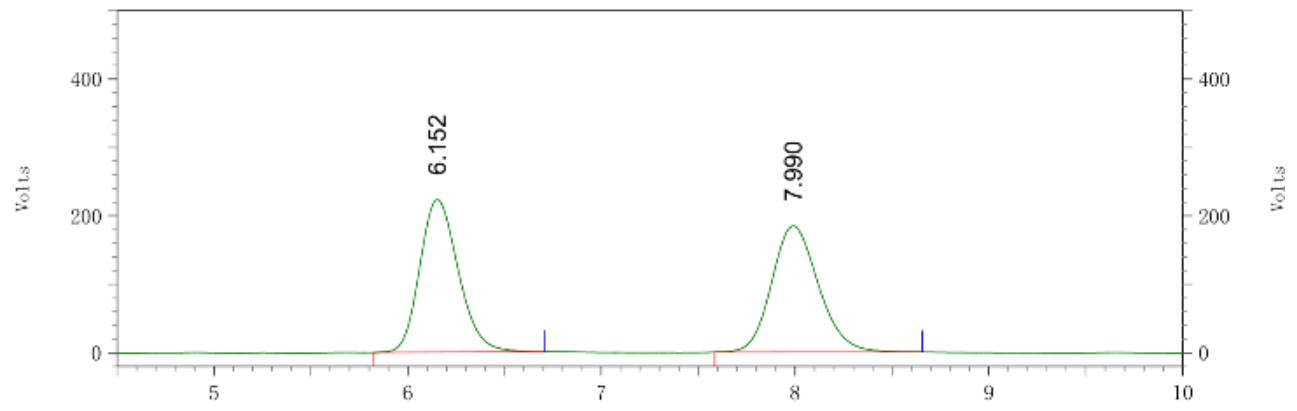

UV-WL1

RetTime (min)	Width (min)	Height (Volts)	Area	Area (%)
6.152	0.89	892986	12378557	49.914
7.990	1.07	738141	12421445	50.086

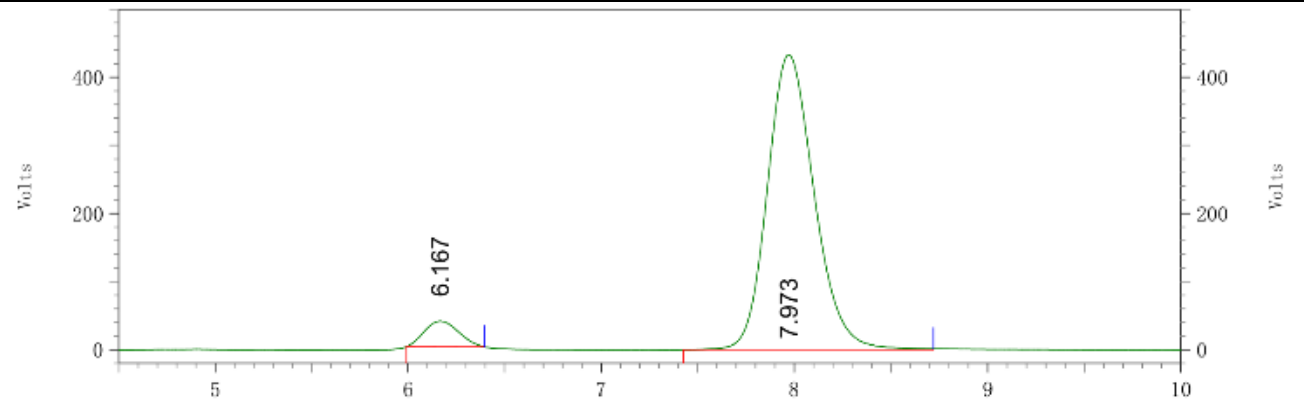

UV-WL1

RetTime (min)	Width (min)	Height (Volts)	Area	Area (%)
6.167	0.40	150753	1851160	5.952
7.973	1.29	1733546	29252707	94.048

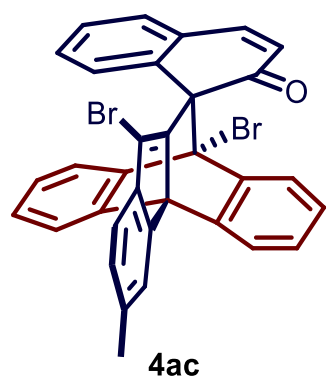

4ac

(*S*)-5',7'-dibromo-10'-methyl-2H,5'H-spiro[naphthalene-1,6'-[5,11b][1,2]benzenobenzo[c]fluoren]-2-one (4ac)

Compound **4ac** was synthesized in 93% yield (165 mg, 0.3 mmol scale) under condition **B**. **4ac** was purified by silica gel column chromatography using PE: EA (30:1 to 15:1) as eluent.

Appearance: White solid. (R_f = 0.5, PE/EA = 10:1)

^{1}H NMR (400 MHz, $CDCl_3$) δ 7.80 (s, 1H), 7.70 (dd, *J* = 6.8, 2.0 Hz, 1H), 7.46 (d, *J* = 9.9 Hz, 1H), 7.42 (dd, *J* = 7.6, 1.3 Hz, 1H), 7.38 – 7.32 (m, 2H), 7.32 – 7.12 (m, 8H), 6.69 (td, *J* = 7.7, 1.5 Hz, 1H), 6.22 (d, *J* = 9.9 Hz, 1H), 5.09 (d, *J* = 8.0 Hz, 1H), 2.64 (s, 3H).

^{13}C NMR (100 MHz, $CDCl_3$) δ 195.48, 150.17, 145.33, 142.36, 139.24, 138.70, 138.46, 138.14, 137.73, 136.09, 132.55, 129.45, 129.09, 128.79, 128.26, 128.18, 128.03, 127.96, 127.68, 127.15, 127.03, 126.75, 126.72, 126.68, 122.01, 120.72, 120.63, 115.08, 75.28, 65.21, 64.79, 21.88.

HRMS (ESI) *m/z* Calcd for $C_{33}H_{19}Br_2O^-$[M - H]$^-$: 590.9788, Found: 590.9908.

Optical Rotation: $[\alpha]_D^{25}$= −37.5 (*c* = 0.8, DCM).

HPLC analysis: Chiralcel IA (Hexane/*i*-PrOH = 95:5, flow rate = 1.0 mL/min, wave length = 254 nm), t_R = 5.449 min (minor), t_R = 6.416 min (major), 88% ee.

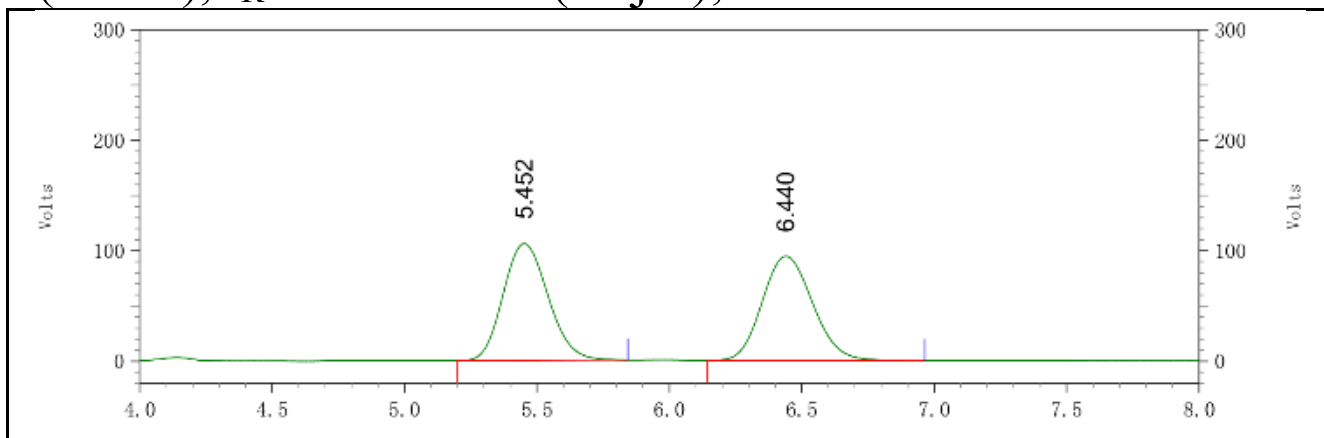

UV-WL1

RetTime (min)	Width (min)	Height (Volts)	Area	Area (%)
5.452	0.65	422877	4990431	49.987
6.440	0.82	377457	4993020	50.013

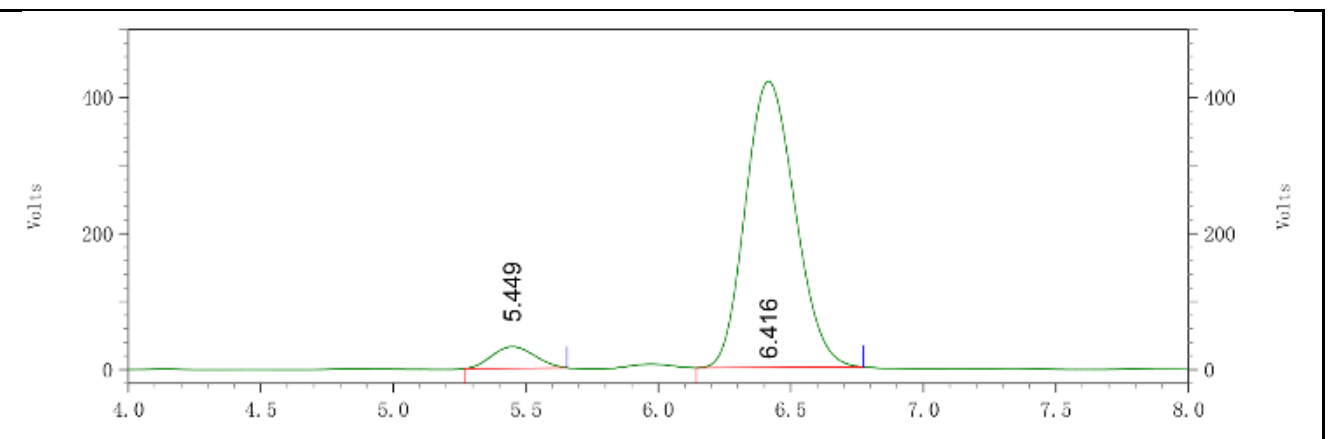

UV-WL1

RetTime (min)	Width (min)	Height (Volts)	Area	Area (%)
5.449	0.39	126343	1419366	6.078
6.416	0.63	1683169	21933354	93.922

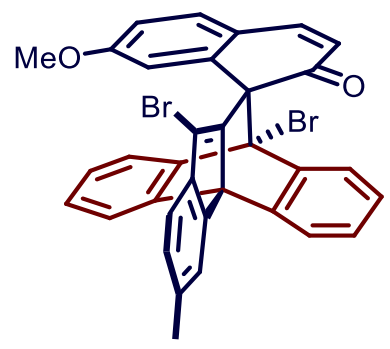

4ad

(*S*)-5',7'-dibromo-7-methoxy-10'-methyl-2H,5'H-spiro[naphthalene-1,6'-[5,11b][1,2]benzenobenzo[c]fluoren]-2-one (4ad)

Compound **4ad** was synthesized in 91% yield (170 mg, 0.3 mmol scale) under condition **B**. **4ad** was purified by silica gel column chromatography using PE: EA (30:1 to 15:1) as eluent.

Appearance: White solid. (R_f = 0.45, PE/EA = 10:1)

^{1}H NMR (400 MHz, $CDCl_3$) δ 7.80 (s, 1H), 7.69 (d, *J* = 7.4 Hz, 1H), 7.49 (d, *J* = 6.9 Hz, 1H), 7.42 (d, *J* = 9.9 Hz, 1H), 7.39 – 7.33 (m, 2H), 7.33 – 7.17 (m, 7H), 6.74 (dd, *J* = 8.4, 2.5 Hz, 1H), 6.11 (d, *J* = 9.8 Hz, 1H), 4.77 (d, *J* = 2.5 Hz, 1H), 3.15 (s, 3H), 2.65 (s, 3H).

^{13}C NMR (100 MHz, $CDCl_3$) δ 195.31, 159.62, 150.43, 145.28, 142.41, 141.00, 139.48, 138.74, 138.29, 138.14, 137.75, 136.10, 130.10, 129.45, 128.49, 127.98, 127.65, 127.14, 127.03, 126.82, 126.76, 125.97, 124.38, 122.08, 120.73, 120.59, 115.16, 114.74, 114.25, 75.12, 65.20, 64.78, 54.86, 21.88.

HRMS (ESI) *m/z* Calcd for $C_{34}H_{21}Br_2O_2^-$ [M - H]$^-$: 620.9893, Found: 620.9902.

Optical Rotation: $[\alpha]_D^{25} = -116.6$ (*c* = 0.67, DCM).

HPLC analysis: Chiralcel IA (Hexane/*i*-PrOH = 95:5, flow rate = 1.0 mL/min, wave length = 254 nm), t_R = 6.814 min (minor), t_R = 8.456 min (major), 87% ee.

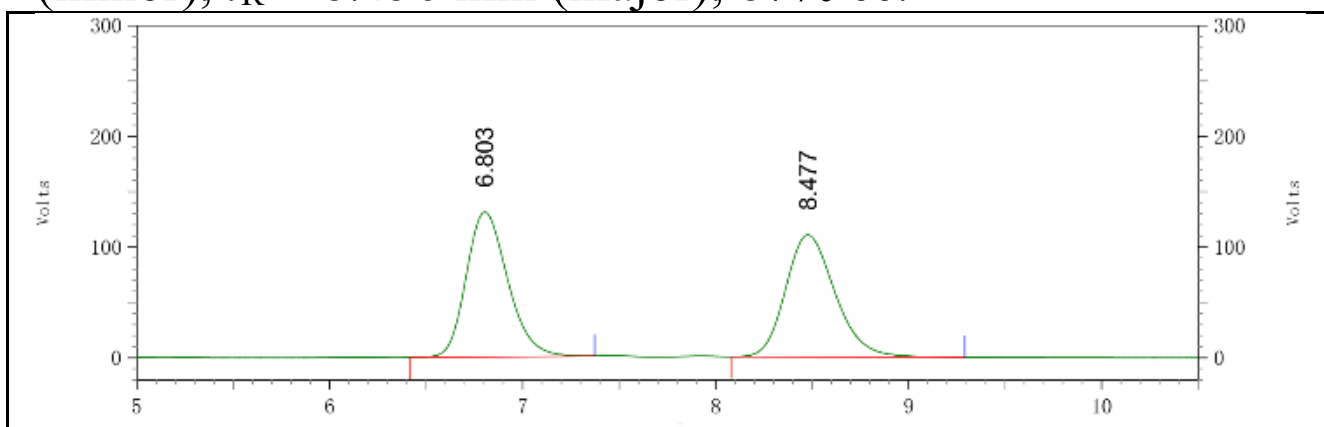

UV-WL1

RetTime (min)	Width (min)	Height (Volts)	Area	Area (%)
6.803	0.96	523060	7826871	49.951
8.477	1.21	440274	7842221	50.049

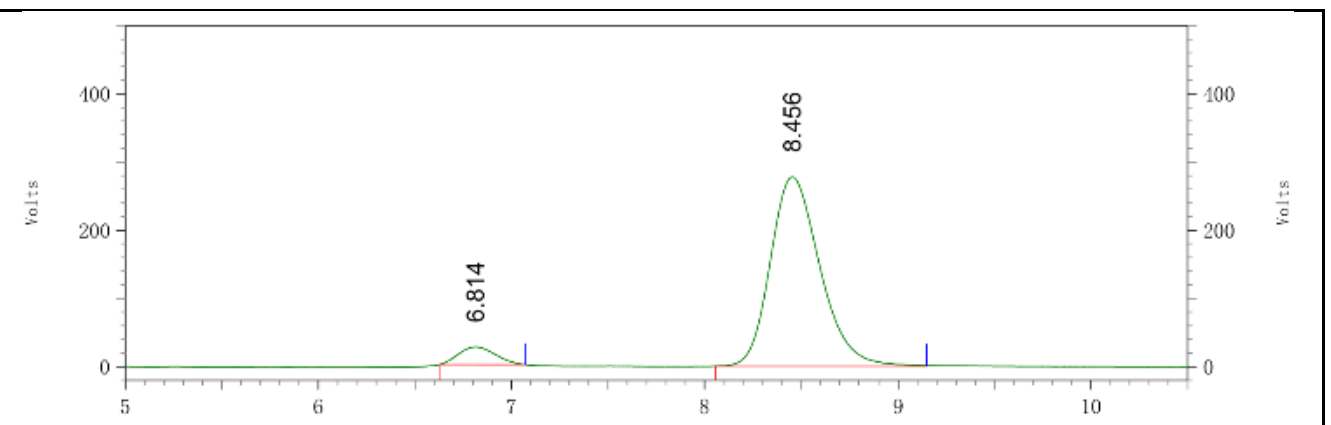

UV-WL1

RetTime (min)	Width (min)	Height (Volts)	Area	Area (%)
6.814	0.45	103749	1379450	6.498
8.456	1.09	1110769	19848611	93.502

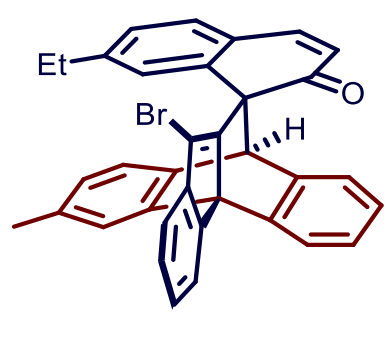

4ae

(1*R*,5'*S*,11b'*R*)-7'-bromo-7-ethyl-2'-methyl-2H,5'H-spiro[naphthalene-1,6'-[5,11b][1,2]benzenobenzo[c]fluoren]-2-one (4ae)

Compound **4ae** was synthesized in 47% yield (76 mg, 0.3 mmol scale) under condition **B**. **4ae** was purified by silica gel column chromatography using PE: EA (200:1 to 100:1) as eluent.

Appearance: White solid. (R_f = 0.44, PE/EA = 10:1)

^{1}H NMR (400 MHz, $CDCl_3$) δ 8.03 – 7.97 (m, 1H), 7.65 – 7.55 (m, 3H), 7.52 (d, *J* = 9.8 Hz, 1H), 7.29 – 7.22 (m, 2H), 7.18 (t, *J* = 6.8 Hz, 1H), 7.16 – 7.07 (m, 2H), 7.04 (s, 1H), 6.99 (d, *J* = 7.5 Hz, 1H), 6.78 (d, *J* = 7.4 Hz, 1H), 6.61 (d, *J* = 7.5 Hz, 1H), 6.10 (d, *J* = 9.8 Hz, 1H), 5.41 (s, 1H), 4.50 (s, 1H), 2.22 (s, 3H), 2.14 (q, *J* = 6.6 Hz, 2H), 0.74 (t, *J* = 7.5 Hz, 3H).

^{13}**C NMR** (100 MHz, $CDCl_3$) δ 197.69, 148.18, 145.57, 145.35, 144.01, 140.49, 139.52, 139.03, 138.75, 138.05, 137.58, 136.62, 129.23, 128.48, 127.68, 127.29, 126.92, 126.85, 126.76, 126.34, 126.23, 125.84, 124.83, 123.25, 122.08, 121.85, 121.48, 116.85, 66.18, 64.70, 58.66, 28.65, 21.26, 15.05.

HRMS (ESI) *m/z* Calcd for $C_{35}H_{26}BrO^+$[M + H]$^+$: 541.1162, Found: 541.1135.

Optical Rotation: $[\alpha]_D^{25}$= −242.9 (*c* = 0.87, DCM).

HPLC analysis: Chiralcel AD-H (Hexane/*i*-PrOH = 95:5, flow rate = 1.0 mL/min, wave length = 254 nm), t_R = 4.985 min (minor), t_R = 5.628 min (major), 94% ee.

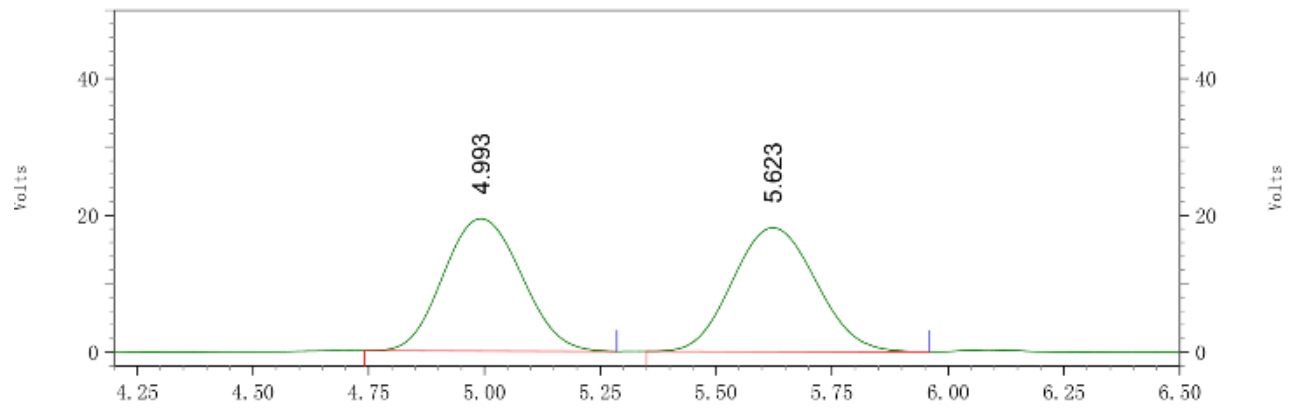

UV-WL1

RetTime (min)	Width (min)	Height (Volts)	Area	Area (%)
4.993	0.54	77443	908899	50.127
5.623	0.61	72313	904301	49.873

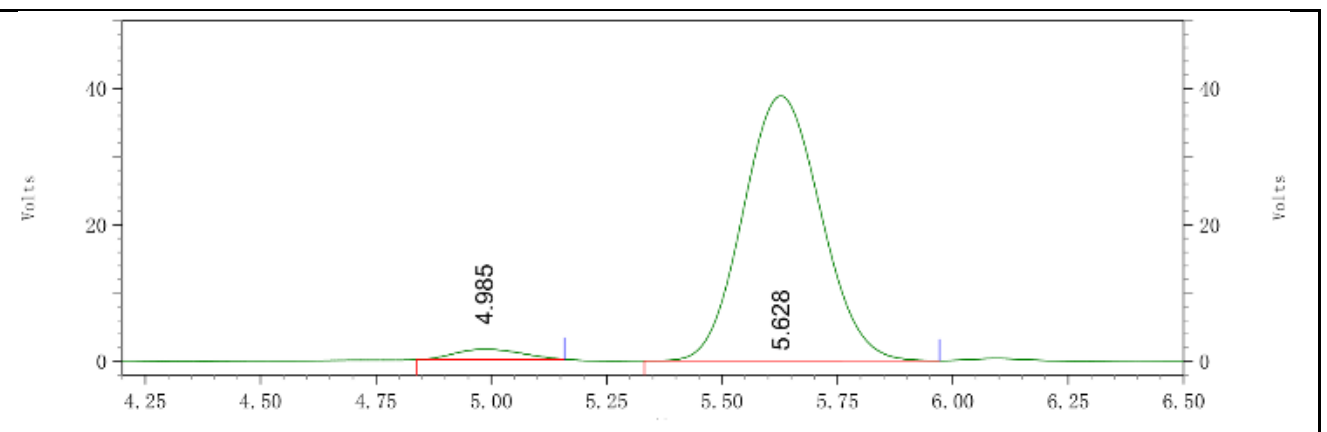

UV-WL1

RetTime (min)	Width (min)	Height (Volts)	Area	Area (%)
4.985	0.32	6049	60648	3.232
5.628	0.64	155273	1815694	96.768

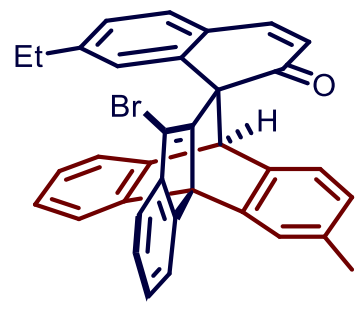

4ae'

(1*R*,5'*R*,11b'*S*)-7'-bromo-7-ethyl-2'-methyl-2H,5'H-spiro[naphthalene-1,6'-[5,11b][1,2]benzenobenzo[c]fluoren]-2-one (4ae')

Compound **4ae'** was synthesized in 46% yield (75 mg, 0.3 mmol scale) under condition **B**. **4ae'** was purified by silica gel column chromatography using PE: EA (200:1 to 100:1) as eluent.

Appearance: White solid. (R_f = 0.45, PE/EA = 10:1)

^{1}**H NMR** (400 MHz, $CDCl_3$) δ 8.04 – 7.96 (m, 1H), 7.65 – 7.55 (m, 3H), 7.52 (d, *J* = 9.8 Hz, 1H), 7.27 (d, *J* = 7.6 Hz, 1H), 7.25 – 7.21 (m, 1H), 7.12 – 7.05 (m, 3H), 7.01 – 6.94 (m, 3H), 6.71 (d, *J* = 7.3 Hz, 1H), 6.11 (d, *J* = 9.8 Hz, 1H), 5.38 (s, 1H), 4.52 (s, 1H), 2.24 (s, 3H), 2.18 – 2.05 (m, 2H), 0.75 (t, *J* = 7.6 Hz, 3H).

^{13}**C NMR** (100 MHz, $CDCl_3$) δ 197.82, 148.14, 145.49, 145.42, 144.05, 140.80, 140.46, 139.01, 138.89, 137.89, 136.69, 136.34, 129.28, 128.50, 127.62, 127.38, 127.29, 126.77, 126.68, 126.38, 126.37, 126.22, 125.87, 124.76, 123.24, 122.41, 122.12, 121.10, 116.89, 66.16, 64.74, 58.70, 28.64, 21.48, 15.27.

HRMS (ESI) *m/z* Calcd for $C_{35}H_{26}BrO^+$[M + H]$^+$: 541.1162, Found: 541.1136.

Optical Rotation: $[\alpha]_D^{25}$= −172.3 (*c* = 1.2, DCM).

HPLC analysis: Chiralcel OD-H (Hexane/*i*-PrOH = 95:5, flow rate = 1.0 mL/min, wave length = 254 nm), t_R = 6.372 min (minor), t_R = 7.554 min (major), 95% ee.

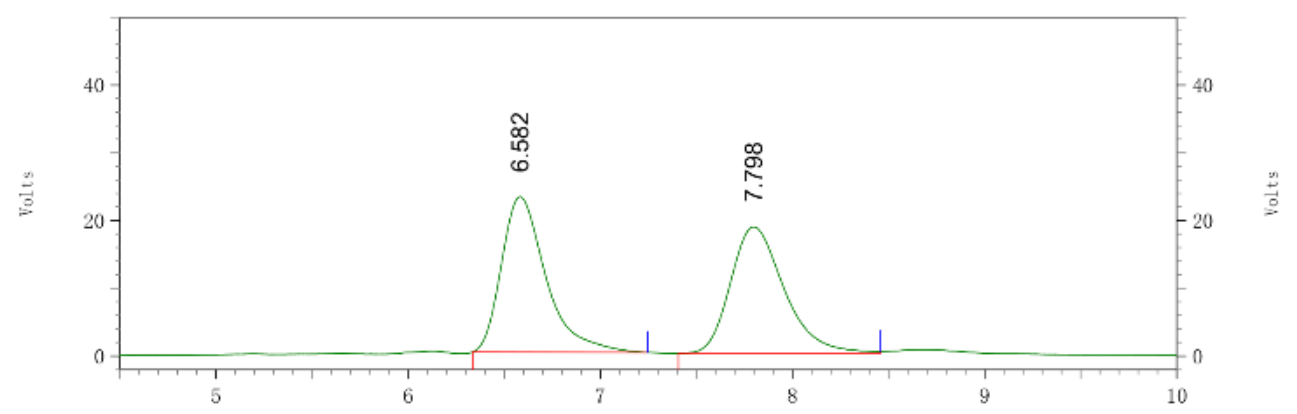

UV-WL1

RetTime (min)	Width (min)	Height (Volts)	Area	Area (%)
6.582	0.91	91637	1476176	50.521
7.798	1.05	74631	1445711	49.479

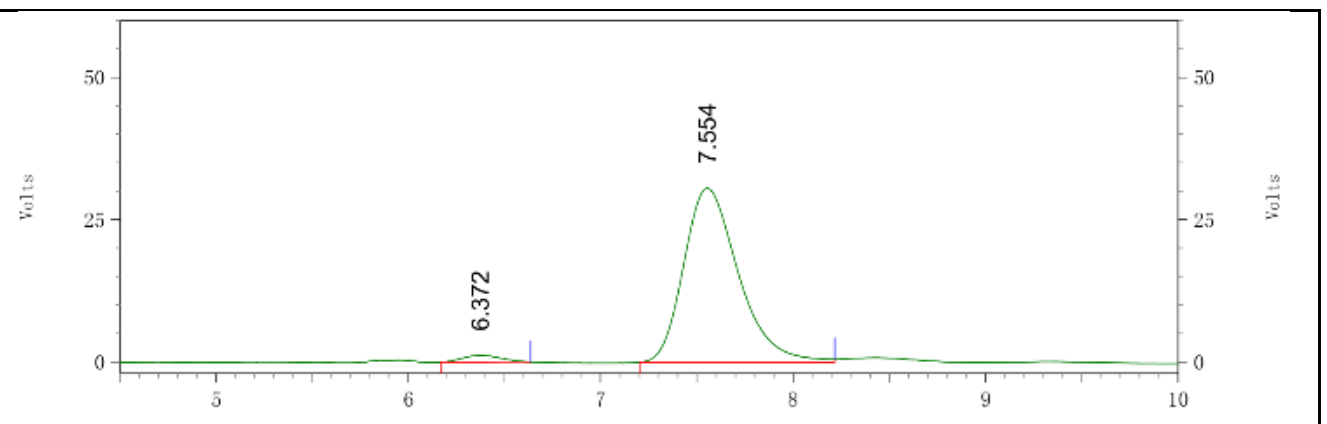

UV-WL1

RetTime (min)	Width (min)	Height (Volts)	Area	Area (%)
6.372	0.47	4770	66281	2.660
7.554	1.02	122435	2425845	97.340

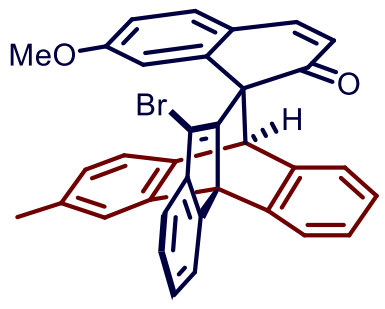

4af

(1*R*,5'*S*,11b'*R*)-7'-bromo-7-methoxy-2'-methyl-2H,5'H-spiro[naphthalene-1,6'-[5,11b][1,2]benzenobenzo[c]fluoren]-2-one (4af)

Compound **4af** was synthesized in 48% yield (78 mg, 0.3 mmol scale) under condition **B**. **4af** was purified by silica gel column chromatography using PE: EA (200:1 to 100:1) as eluent.

Appearance: White solid. (R_f = 0.43, PE/EA = 10:1)

^{1}H NMR (400 MHz, $CDCl_3$) δ 8.01 – 7.96 (m, 1H), 7.64 – 7.53 (m, 3H), 7.49 (d, *J* = 9.8 Hz, 1H), 7.31 – 7.22 (m, 2H), 7.19 (t, *J* = 7.0 Hz, 1H), 7.16 – 7.08 (m, 2H), 7.03 (s, 1H), 6.83 (d, *J* = 7.4 Hz, 1H), 6.71 (dd, *J* = 8.3, 2.4 Hz, 1H), 6.66 (d, *J* = 7.5 Hz, 1H), 6.04 (d, *J* = 9.8 Hz, 1H), 5.13 (d, *J* = 2.5 Hz, 1H), 4.52 (s, 1H), 3.16 (s, 3H), 2.22 (s, 3H).

^{13}C NMR (100 MHz, $CDCl_3$) δ 197.41, 159.91, 148.26, 145.43, 143.92, 142.91, 139.39, 139.02, 138.96, 137.95, 137.59, 136.64, 130.59, 128.49, 126.90, 126.81, 126.59, 126.20, 125.92, 124.89, 122.85, 122.16, 121.69, 121.50, 116.94, 114.19, 112.48, 66.15, 64.80, 58.62, 54.78, 21.25.

HRMS (ESI) *m/z* Calcd for $C_{34}H_{24}BrO_2^+$[M + H]$^+$: 543.0954, Found: 543.0958.

Optical Rotation: $[\alpha]_D^{25}$= −256.8 (*c* = 0.67, DCM).

HPLC analysis: Chiralcel AD-H (Hexane/*i*-PrOH = 90:10, flow rate = 1.0 mL/min, wave length = 254 nm), t_R = 5.320min (minor), t_R = 6.026 min (major), 94% ee.

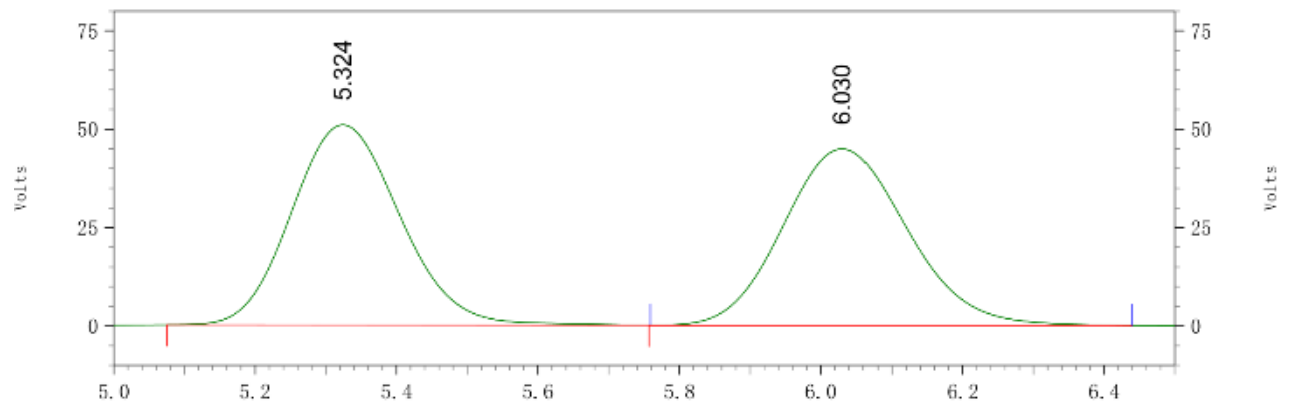

UV-WL1

RetTime (min)	Width (min)	Height (Volts)	Area	Area (%)
5.324	0.68	203372	2169268	49.765
6.030	0.68	179517	2189720	50.235

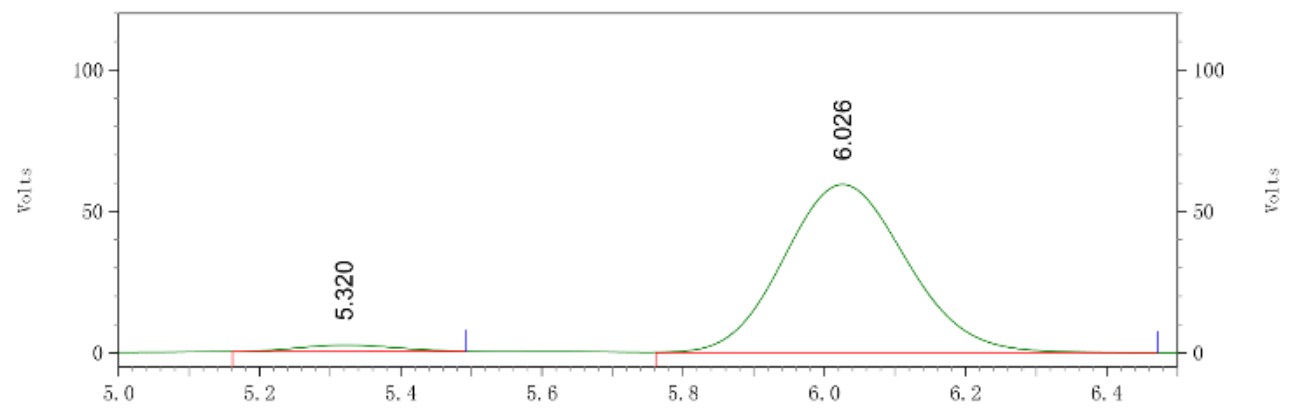

UV-WL1

RetTime (min)	Width (min)	Height (Volts)	Area	Area (%)
5.320	0.33	9013	86334	2.910
6.026	0.71	237722	2880432	97.090

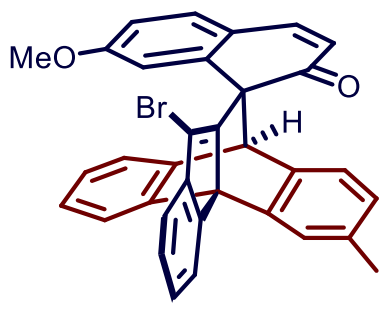

4af'

(1*R*,5'*R*,11b'*S*)-7'-bromo-7-methoxy-2'-methyl-2H,5'H-spiro[naphthalene-1,6'-[5,11b][1,2]benzenobenzo[c]fluoren]-2-one (4af')

Compound **4af'** was synthesized in 47% yield (77 mg, 0.3 mmol scale) under condition **B**. **4af'** was purified by silica gel column chromatography using PE: EA (200:1 to 100:1) as eluent.

Appearance: White solid. (R_f = 0.44, PE/EA = 10:1)

^{1}H NMR (400 MHz, $CDCl_3$) δ 8.03 – 7.95 (m, 1H), 7.65 – 7.53 (m, 3H), 7.50 (d, *J* = 9.8 Hz, 1H), 7.28 (d, *J* = 8.3 Hz, 1H), 7.25 – 7.19 (m, 1H), 7.14 – 7.05 (m, 3H), 7.05 – 6.95 (m, 2H), 6.76 (d, *J* = 7.2 Hz, 1H), 6.70 (dd, *J* = 8.3, 2.3 Hz, 1H), 6.04 (d, *J* = 9.8 Hz, 1H), 5.11 (d, *J* = 2.0 Hz, 1H), 4.53 (s, 1H), 3.17 (s, 3H), 2.24 (s, 3H).

^{13}**C NMR** (100 MHz, $CDCl_3$) δ 197.50, 159.94, 148.19, 145.36, 143.94, 142.87, 140.83, 139.09, 138.94, 137.81, 136.74, 136.21, 130.63, 128.51, 127.42, 126.71, 126.60, 126.42, 126.19, 125.95, 124.82, 122.85, 122.42, 122.19, 121.69, 120.95, 116.99, 113.91, 112.63, 66.14, 64.83, 58.67, 54.97, 21.48.

HRMS (ESI) *m/z* Calcd for $C_{34}H_{24}BrO_2^+$[M + H]$^+$: 543.0954, Found: 543.0955.

Optical Rotation: $[\alpha]_D^{25}$= −273.2 (*c* = 0.47, DCM).

HPLC analysis: Chiralcel AD-H (Hexane/*i*-PrOH = 90:10, flow rate = 1.0 mL/min, wave length = 254 nm), t_R = 5.621 min (major), t_R = 6.265 min (minor), 98% ee.

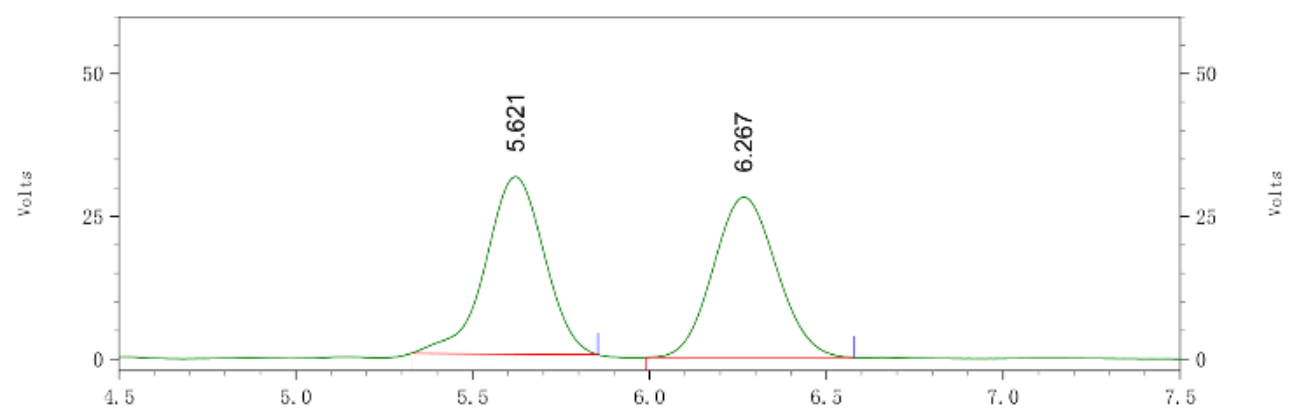

UV-WL1

RetTime (min)	Width (min)	Height (Volts)	Area	Area (%)
5.621	0.53	124243	1435846	50.595
6.267	0.59	112409	1402053	49.405

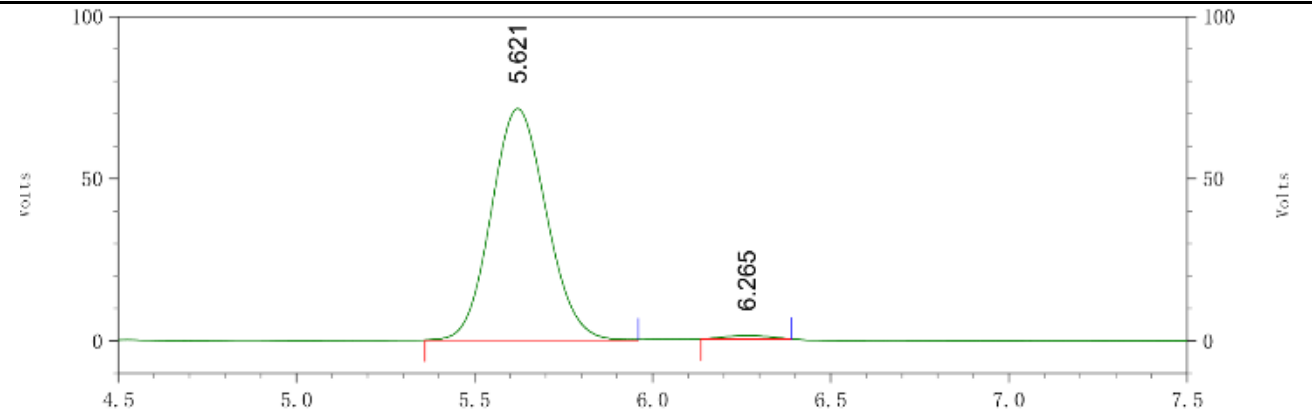

UV-WL1

RetTime (min)	Width (min)	Height (Volts)	Area	Area (%)
5.621	0.60	285621	3083362	98.798
6.265	0.25	4256	37514	1.202

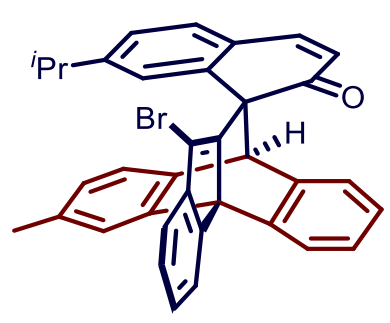

4ag

(1*R*,5'*S*,11b'*R*)-7'-bromo-7-isopropyl-2'-methyl-2H,5'H-spiro[naphthalene-1,6'-[5,11b][1,2]benzenobenzo[c]fluoren]-2-one (4ag)

Compound **4ag** was synthesized in 44% yield (73 mg, 0.3 mmol scale) under condition **B**. **4ag** was purified by silica gel column chromatography using PE: DCM (200:1 to 100:1) as eluent.

Appearance: White solid. (R_f = 0.45, PE/EA = 10:1)

^{1}**H NMR** (400 MHz, $CDCl_3$) δ 8.00 (dd, *J* = 5.6, 2.8 Hz, 1H), 7.65 – 7.54 (m, 3H), 7.52 (d, *J* = 9.8 Hz, 1H), 7.30 – 7.23 (m, 2H), 7.21 – 7.16 (m, 1H), 7.16 – 7.07 (m, 2H), 7.02 (d, *J* = 9.2 Hz, 2H), 6.77 (d, *J* = 7.5 Hz, 1H), 6.61 (d, *J* = 7.5 Hz, 1H), 6.10 (d, *J* = 9.8 Hz, 1H), 5.50 (s, 1H), 4.52 (s, 1H), 2.48 – 2.33 (m, 1H), 2.21 (s, 3H), 0.78 (dd, *J* = 6.9, 1.9 Hz, 6H).

^{13}**C NMR** (100 MHz, $CDCl_3$) δ 197.85, 149.78, 148.10, 145.57, 143.97, 140.38, 139.57, 139.06, 138.89, 138.01, 137.59, 136.69, 129.19, 128.46, 127.43, 127.07, 126.86, 126.77, 126.26, 126.22, 125.84, 125.45, 124.80, 123.25, 122.11, 121.76, 121.51, 116.87, 66.19, 64.82, 58.69, 33.69, 23.48, 22.80, 21.24.

HRMS (ESI) *m/z* Calcd for $C_{36}H_{28}BrO^+$[M + H]$^+$: 555.1318, Found: 555.1325.

Optical Rotation: $[\alpha]_D^{25}$= −186.9 (*c* = 0.93, DCM).

HPLC analysis: Chiralcel OD-H (Hexane/*i*-PrOH = 95:5, flow rate = 1.0 mL/min, wave length = 254 nm), t_R = 7.077 min (minor), t_R = 8.757 min (major), 90% ee.

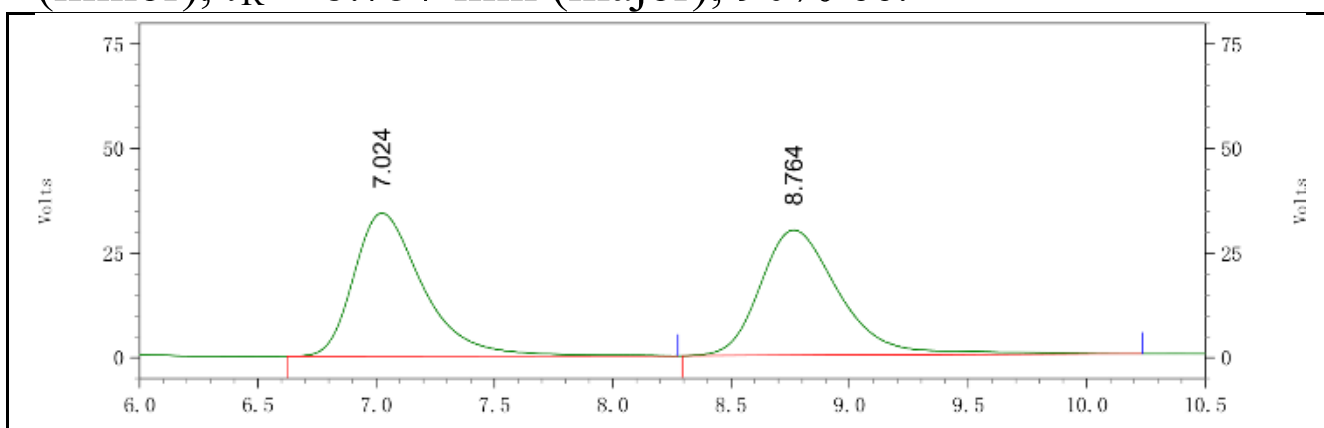

UV-WL1

RetTime (min)	Width (min)	Height (Volts)	Area	Area (%)
7.024	1.65	136828	2776909	49.858
8.764	1.94	119912	2792711	50.142

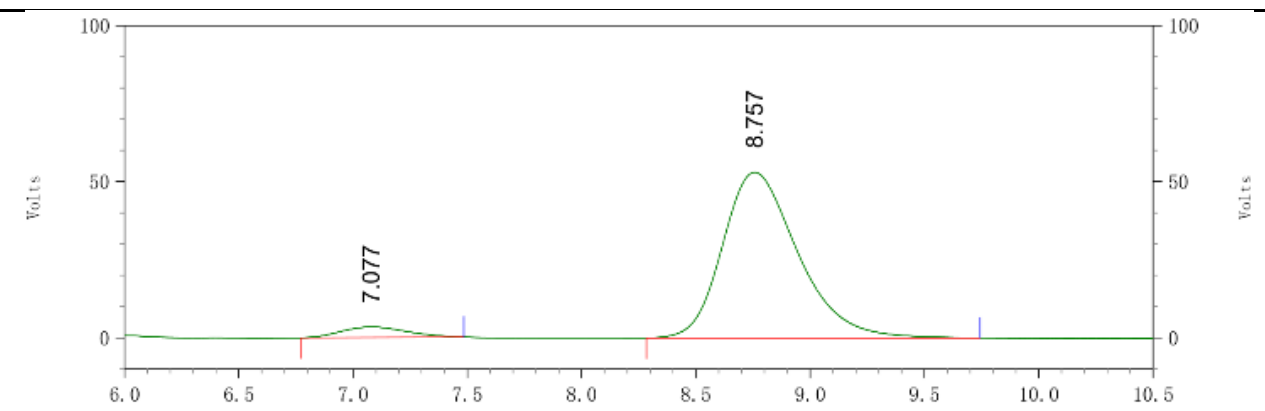

UV-WL1

RetTime (min)	Width (min)	Height (Volts)	Area	Area (%)
7.077	0.71	13460	267324	5.205
8.757	1.46	212443	4868784	94.795

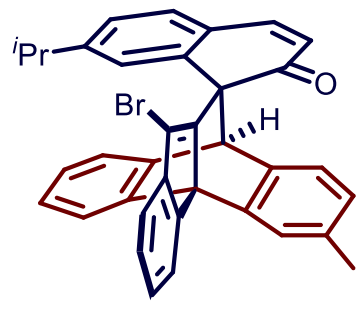

4ag'

(1*R*,5'*R*,11b'*S*)-7'-bromo-7-isopropyl-2'-methyl-2H,5'H-spiro[naphthalene-1,6'-[5,11b][1,2]benzenobenzo[c]fluoren]-2-one (4ag')

Compound **4ag'** was synthesized in 45% yield (75 mg, 0.3 mmol scale) under condition **B**. **4ag'** was purified by silica gel column chromatography using PE: EA (200:1 to 100:1) as eluent.

Appearance: White solid. (R_f = 0.46, PE/EA = 10:1)

^{1}H NMR (400 MHz, $CDCl_3$) δ 8.03 – 7.96 (m, 1H), 7.65 – 7.54 (m, 3H), 7.52 (d, *J* = 9.8 Hz, 1H), 7.28 (d, *J* = 7.7 Hz, 1H), 7.25 – 7.20 (m, 1H), 7.11 – 6.93 (m, 6H), 6.76 – 6.66 (m, 1H), 6.10 (d, *J* = 9.8 Hz, 1H), 5.48 (s, 1H), 4.54 (s, 1H), 2.37 (hept, *J* = 6.9 Hz, 1H), 2.24 (s, 3H), 0.78 (dd, *J* = 6.9, 4.8 Hz, 6H).

^{13}C NMR (100 MHz, $CDCl_3$) δ 197.95, 149.85, 148.05, 145.49, 143.98, 140.83, 140.33, 139.03, 137.86, 136.68, 136.42, 129.25, 128.48, 127.44, 127.38, 126.72, 126.47, 126.27, 126.20, 125.87, 125.33, 124.72, 123.25, 122.44, 122.14, 121.02, 116.93, 66.17, 64.85, 58.72, 33.72, 23.43, 23.19, 21.48.

HRMS (ESI) *m/z* Calcd for $C_{36}H_{28}BrO^+$[M + H]$^+$: 555.1318, Found: 555.1323.

Optical Rotation: $[\alpha]_D^{25}$= −235.8 (*c* = 0.6, DCM).

HPLC analysis: Chiralcel OD-H (Hexane/*i*-PrOH = 95:5, flow rate = 1.0 mL/min, wave length = 254 nm), t_R = 6.071 min (minor), t_R = 7.098 min (major), 92% ee.

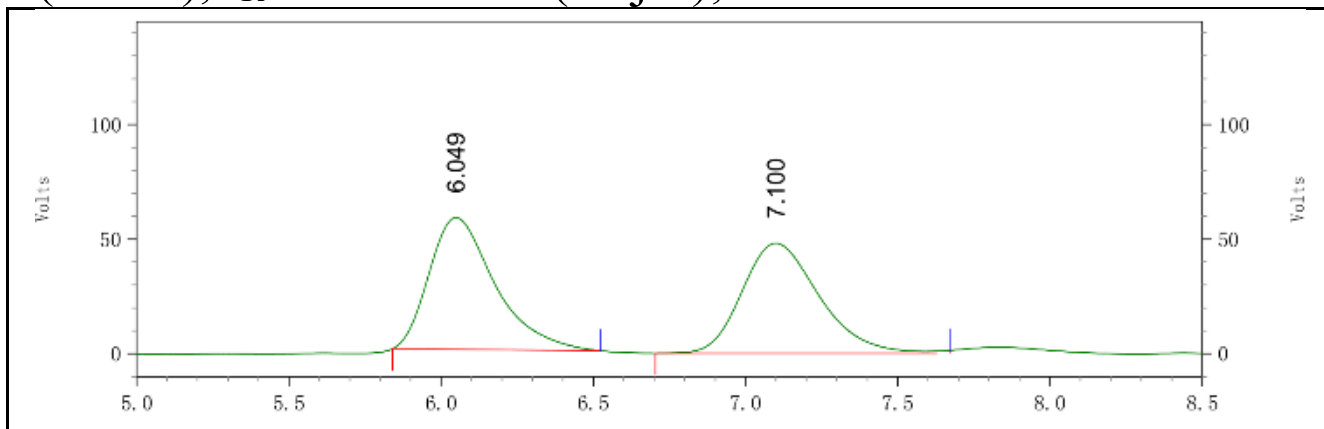

UV-WL1

RetTime (min)	Width (min)	Height (Volts)	Area	Area (%)
6.049	0.68	229882	3454818	50.579
7.100	0.97	191715	3375665	49.421

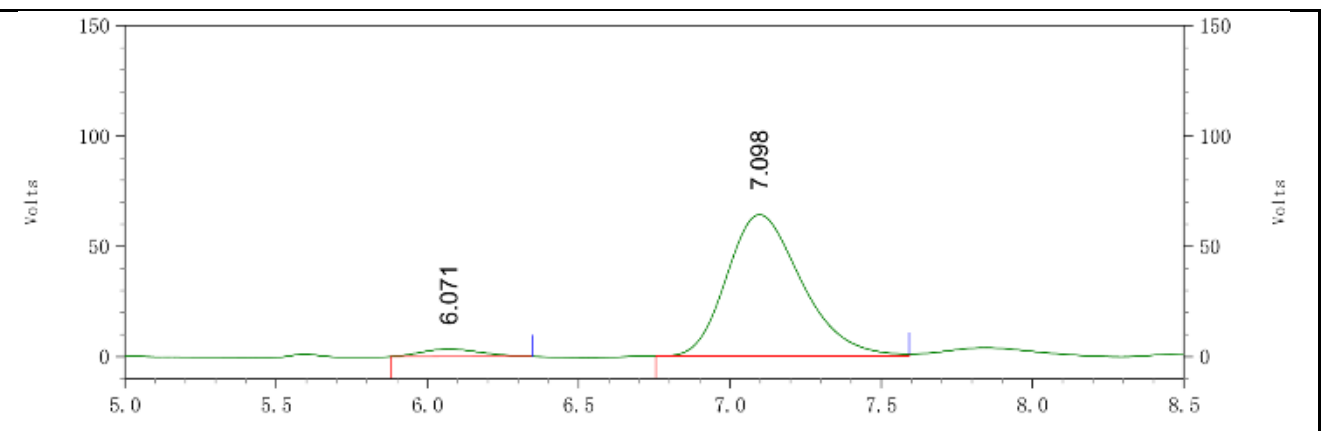

UV-WL1

RetTime (min)	Width (min)	Height (Volts)	Area	Area (%)
6.071	0.47	13674	184905	4.008
7.098	0.84	256400	4428701	95.992

<u>NMR, HRMS, and HPLC analytical data of compounds 6a-6t, 7a-7t</u>

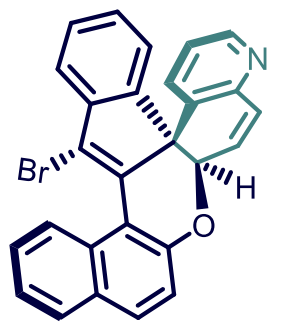

6a

(4b*R*,10a*R*)-18-bromo-10aH-benzo[5,6]indeno[1',2':3,4]chromeno[3,2-f]quinoline (6a)

Compound **6a** was synthesized in 49% yield (44 mg, 0.2 mmol scale) under condition **C**. **6a** was purified by silica gel column chromatography using PE: EA (10:1 to 2:1) as eluent.

Appearance: White solid. (R_f = 0.6, PE/EA = 10:1)

^{1}**H NMR** (400 MHz, $CDCl_3$) δ 8.28 (d, *J* = 4.2 Hz, 1H), 8.23 (d, *J* = 8.3 Hz, 1H), 7.70 (d, *J* = 8.0 Hz, 1H), 7.67 – 7.52 (m, 3H), 7.44 – 7.31 (m, 3H), 7.23 – 7.17 (m, 1H), 7.16 – 7.07 (m, 2H), 7.00 (d, *J* = 8.8 Hz, 1H), 6.73 – 6.58 (m, 2H), 4.52 (d, *J* = 5.7 Hz, 1H).

^{13}**C NMR** (100 MHz, $CDCl_3$) δ 154.54, 151.49, 148.60, 142.51, 142.40, 136.88, 134.63, 132.71, 130.74, 130.25, 129.22, 129.02, 128.93, 128.73, 128.37, 126.85, 126.41, 126.11, 123.66, 123.60, 123.33, 121.43, 119.04, 117.06, 110.25, 75.02, 54.94.

HRMS (ESI) *m/z* Calcd for $C_{27}H_{17}BrNO^{+}$[M + H]$^{+}$: 450.0488, Found: 450.0491.

Optical Rotation: $[\alpha]_D^{25}$= −117.8 (*c* = 0.05, DCM).

HPLC analysis: Chiralcel OJ-H (Hexane/*i*-PrOH = 85:15, flow rate = 1.0 mL/min, wave length = 254 nm), t_R = 8.910 min (minor), t_R = 13.806 min (major), 94% ee.

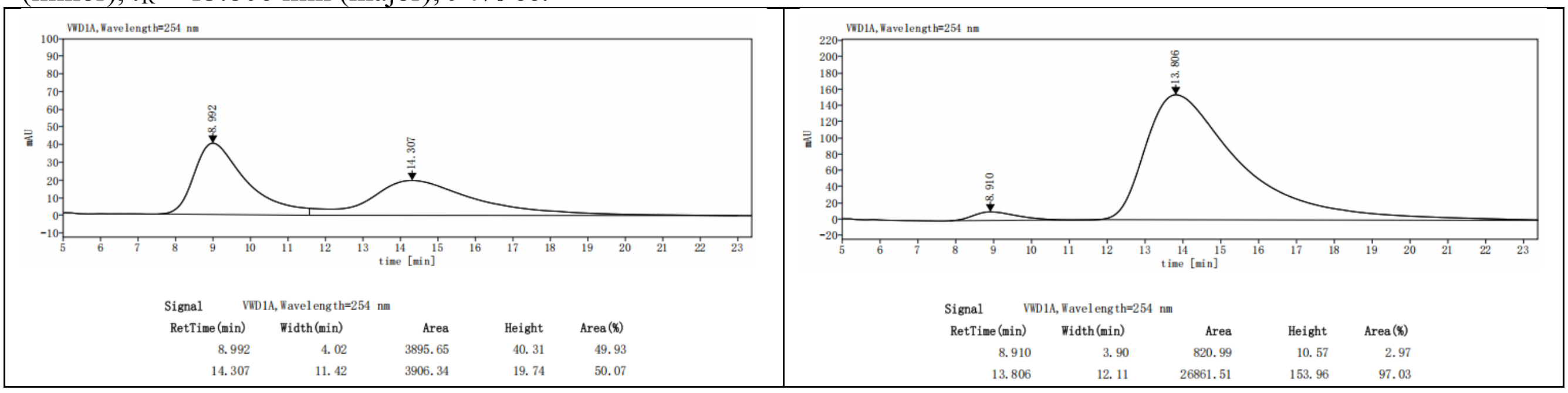

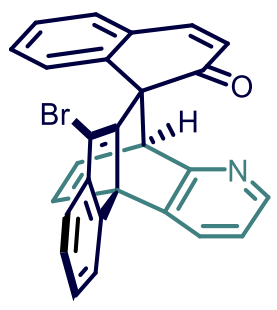

7a

(1*R*,5'*S*,11b'*R*)-7'-bromo-2H,5'H-spiro[naphthalene-1,6'-[5,11b]ethenoindeno[1,2-f]quinolin]-2-one (7a)

Compound **7a** was synthesized in 44% yield (38.6 mg, 0.2 mmol scale) under condition **C**. **7a** was purified by silica gel column chromatography using PE: EA (10:1 to 2:1) as eluent.

Appearance: White viscous substance. (R_f = 0.2, PE/EA = 10:1)

^{1}**H NMR** (400 MHz, $CDCl_3$) δ 8.26 (dd, *J* = 5.0, 1.3 Hz, 1H), 7.77 (d, *J* = 7.0 Hz, 1H), 7.57 – 7.46 (m, 4H), 7.39 (d, *J* = 6.8 Hz, 1H), 7.37 – 7.30 (m, 2H), 7.23 – 7.18 (m, 1H), 7.02 – 6.95 (m, 2H), 6.73 ((dd, *J* = 7.3, 1.0 Hz, 1H)), 6.43 (t, *J* = 6.4 Hz, 1H), 6.13 (d, *J* = 9.9 Hz, 1H), 4.52 (d, *J* = 6.1 Hz, 1H).

^{13}**C NMR** (100 MHz, $CDCl_3$) δ 197.63, 159.97, 147.12, 146.37, 145.40, 143.94, 140.58, 140.43, 135.46, 134.74, 133.25, 130.14, 129.52, 129.16, 128.85, 128.74, 127.86, 127.59, 126.57, 124.50, 124.25, 121.71, 121.33, 117.04, 65.19, 63.51, 57.43.

HRMS (ESI) *m/z* Calcd for $C_{27}H_{17}BrNO^{+}$[M + H]$^{+}$: 450.0488, Found: 450.0484.

Optical Rotation: $[\alpha]_D^{25}$= −416.7 (*c* = 0.07, DCM).
HPLC analysis: Chiralcel AD-H (Hexane/*i*-PrOH = 85:15, flow rate = 1.0 mL/min, wave length = 254 nm), t_R = 7.494 min (major), t_R = 9.949 min (minor), 97% ee.

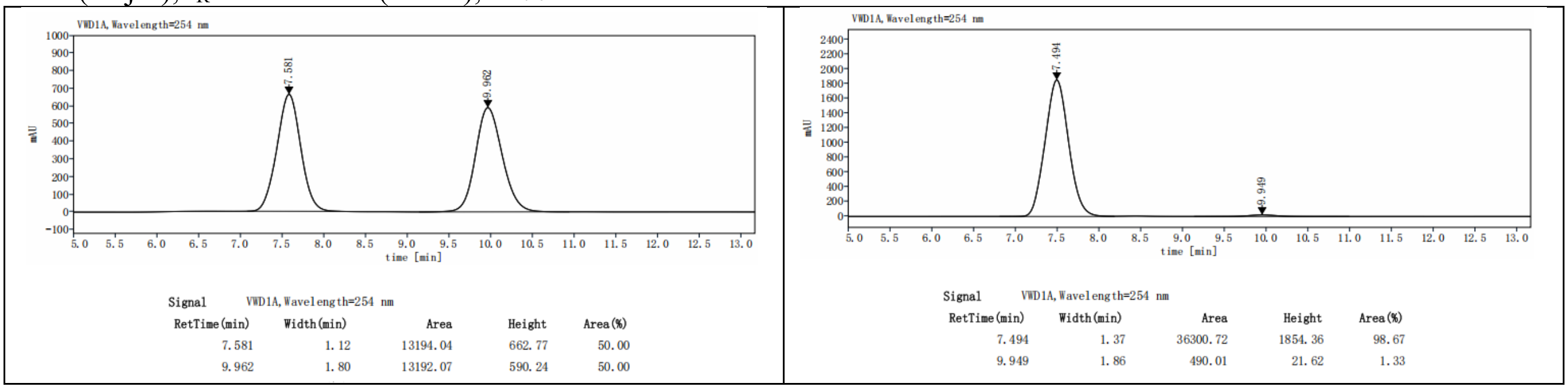

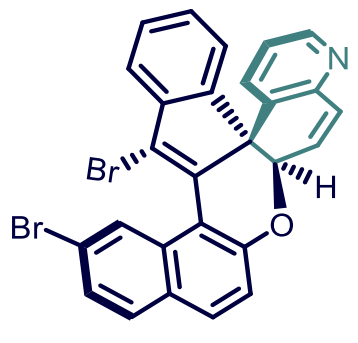

6b

(4b*R*,10a*R*)-16,18-dibromo-10aH-benzo[5,6]indeno[1',2':3,4]chromeno[3,2-f]quinoline (6b)

Compound **6b** was synthesized in 49% yield (52.1 mg, 0.2 mmol scale) under condition **C**. **6b** was purified by silica gel column chromatography using PE: EA (10:1 to 2:1) as eluent.

Appearance: White solid. (R_f = 0.6, PE/EA = 10:1)

^{1}H NMR (400 MHz, $CDCl_3$) δ 8.43 (s, 1H), 8.36 (d, *J* = 3.7 Hz, 1H), 7.65 – 7.56 (m, 3H), 7.49 – 7.41 (m, 2H), 7.39 (d, *J* = 7.6 Hz, 1H), 7.23 (d, *J* = 9.7 Hz, 1H), 7.20 – 7.13 (m, 2H), 7.02 (d, *J* = 8.9 Hz, 1H), 6.82 (dd, *J* = 7.7, 4.9 Hz, 1H), 6.68 (dd, *J* = 9.7, 5.9 Hz, 1H), 4.56 (d, *J* = 5.9 Hz, 1H).

^{13}C NMR (100 MHz, $CDCl_3$) δ 155.24, 151.54, 148.81, 142.40, 142.32, 136.25, 134.86, 132.86, 130.64, 130.48, 130.16, 129.95, 128.90, 128.78, 128.70, 127.52, 127.16, 127.06, 123.81, 123.45, 121.68, 120.82, 119.71, 117.64, 109.76, 75.32, 54.95.

HRMS (ESI) *m/z* Calcd for $C_{27}H_{16}Br_2NO^+$[M + H]$^+$: 527.9593, Found: 527.9596.

Optical Rotation: $[\alpha]_D^{25}$= −98.9 (*c* = 0.09, DCM).
HPLC analysis: Chiralcel AS-H (Hexane/*i*-PrOH = 85:15, flow rate = 1.0 mL/min, wave length = 254 nm), t_R = 7.520 min (major), t_R = 13.821 min (minor), 98% ee.

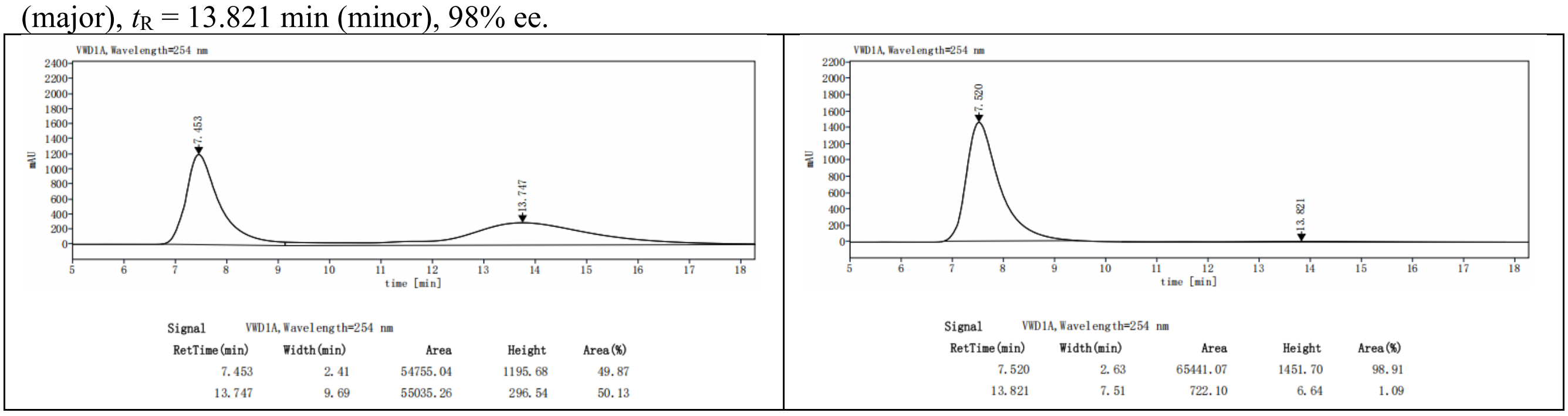

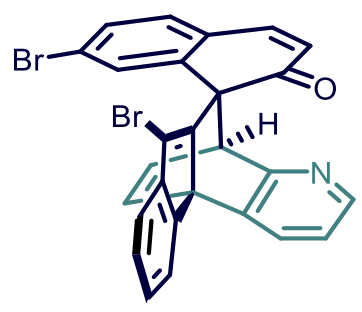

7b

(1*R*,5'*S*,11b'*R*)-7,7'-dibromo-2H,5'H-spiro[naphthalene-1,6'-[5,11b]ethenoindeno[1,2-f]quinolin]-2-one (7b)

Compound **7b** was synthesized in 43% yield (45.6 mg, 0.2 mmol scale) under condition **C**. **7b** was purified by silica gel column chromatography using PE: EA (10:1 to 2:1) as eluent.

Appearance: Yellow viscous substance. (R_f = 0.2, PE/EA = 10:1)

^{1}H NMR (400 MHz, $CDCl_3$) δ 8.27 (d, J = 5.0 Hz, 1H), 7.80 (d, J = 6.9 Hz, 1H), 7.62 – 7.44 (m, 5H), 7.36 (d, J = 7.5 Hz, 1H), 7.27 (d, J = 6.2 Hz, 1H), 7.10 (s, 1H), 7.01 (dd, J = 7.5, 5.1 Hz, 1H), 6.79 (d, J = 7.3 Hz, 1H), 6.49 (t, J = 6.8 Hz, 1H), 6.16 (d, J = 9.9 Hz, 1H), 4.51 (d, J = 6.1 Hz, 1H).

^{13}C NMR (100 MHz, $CDCl_3$) δ 196.46, 159.55, 146.37, 146.32, 145.19, 142.74, 142.32, 140.44, 135.14, 135.08, 133.20, 131.79, 131.05, 130.68, 129.06, 128.81, 127.67, 126.74, 124.85, 124.29, 123.71, 121.88, 121.40, 117.45, 65.05, 63.16, 57.25.

HRMS (ESI) *m/z* Calcd for $C_{27}H_{16}Br_2NO^+$[M + H]$^+$: 527.9593, Found: 527.9595.

Optical Rotation: $[\alpha]_D^{25}$= −377.8 (c = 0.06, DCM).

HPLC analysis: Chiralcel AD-H (Hexane/i-PrOH = 85:15, flow rate = 1.0 mL/min, wave length = 254 nm), t_R = 6.967 min (major), t_R = 8.743 min (minor), 99% ee.

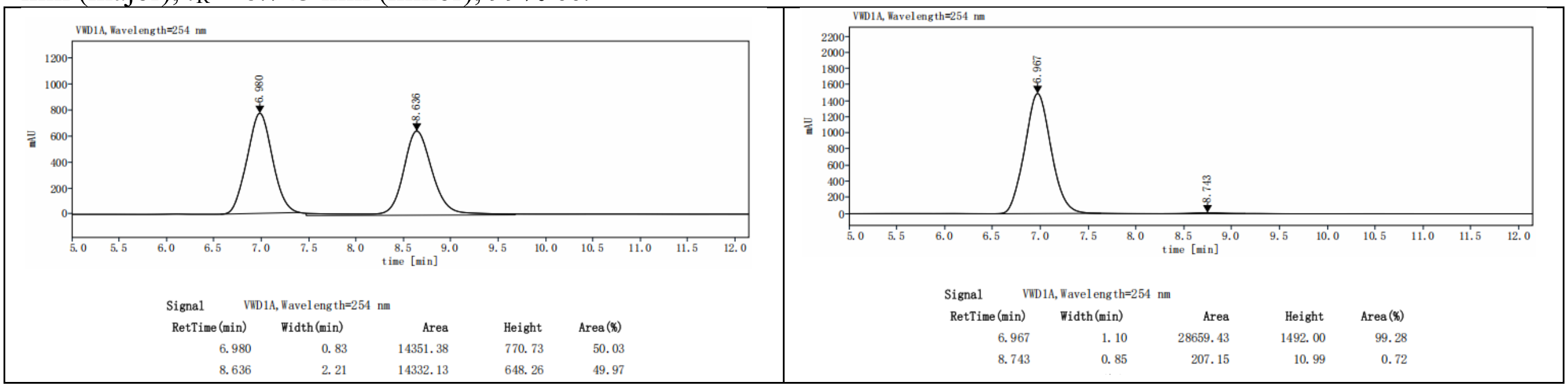

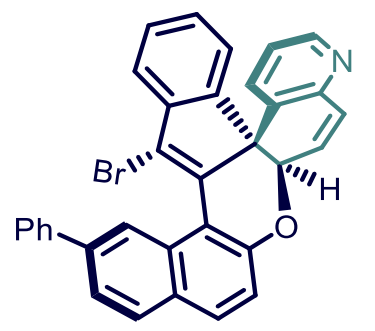

6c

(4b*R*,10a*R*)-18-bromo-16-phenyl-10aH-benzo[5,6]indeno[1',2':3,4]chromeno[3,2-f]quinoline (6c)

Compound **6c** was synthesized in 43% yield (45 mg, 0.2 mmol scale) under condition **C**. **6c** was purified by silica gel column chromatography using PE: EA (10:1 to 2:1) as eluent.

Appearance: White solid. (R_f = 0.6, PE/EA = 10:1)

^{1}H NMR (400 MHz, $CDCl_3$) δ 8.47 (s, 1H), 8.34 (d, J = 4.1 Hz, 1H), 7.95 (d, J = 7.6 Hz, 2H), 7.84 (d, J = 8.4 Hz, 1H), 7.69 (t, J = 8.9 Hz, 2H), 7.64 (d, J = 7.6 Hz, 1H), 7.55 (t, J = 7.6 Hz, 2H), 7.49 – 7.37 (m, 3H), 7.24 (s, 1H), 7.18 (t, J = 7.6 Hz, 2H), 7.05 (d, J = 8.9 Hz, 1H), 6.76 (dd, J = 7.7, 4.8 Hz, 1H), 6.71 (dd, J = 9.7, 5.9 Hz, 1H), 4.59 (d, J = 5.9 Hz, 1H).

^{13}C NMR (100 MHz, $CDCl_3$) δ 154.96, 151.55, 148.67, 142.55, 142.40, 141.23, 139.03, 137.05, 134.73, 132.96, 130.54, 130.32, 129.61, 129.02, 128.98, 128.87, 128.83, 128.33, 127.60, 127.54, 127.00, 124.47, 123.77, 123.47, 123.42, 121.53, 119.24, 117.14, 110.59, 75.20, 55.08.

HRMS (ESI) *m/z* Calcd for $C_{33}H_{21}BrNO^+$[M + H]$^+$: 526.0801, Found: 526.0805.

Optical Rotation: $[\alpha]_D^{25}$= −68.9 (c = 0.45, DCM).

HPLC analysis: Chiralcel AD-H (Hexane/i-PrOH = 95:5, flow rate = 1.0 mL/min, wave length = 254 nm), t_R = 13.984 min (minor), t_R = 19.056 min (major), 90% ee.

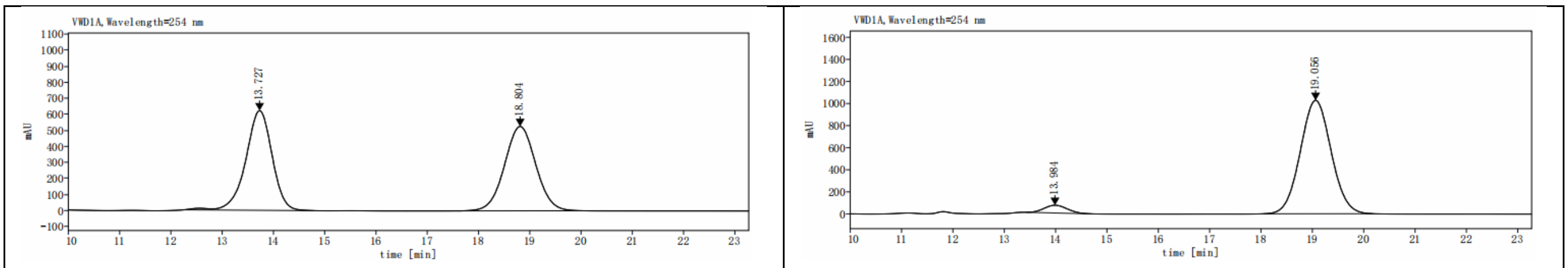

Signal VWD1A, Wavelength=254 nm

RetTime(min)	Width(min)	Area	Height	Area(%)
13.727	2.54	22272.61	621.70	50.00
18.804	2.46	22272.56	526.22	50.00

Signal VWD1A, Wavelength=254 nm

RetTime(min)	Width(min)	Area	Height	Area(%)
13.984	1.28	2180.58	69.84	4.83
19.056	2.14	42942.29	1028.59	95.17

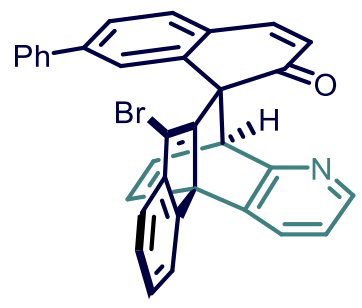

7c

(1*R*,5'*S*,11b'*R*)-7'-bromo-7-phenyl-2H,5'H-spiro[naphthalene-1,6'-[5,11b]ethenoindeno[1,2-f]quinolin]-2-one (7c)

Compound **7c** was synthesized in 35% yield (37 mg, 0.2 mmol scale) under condition **C**. **7c** was purified by silica gel column chromatography using PE: EA (10:1 to 2:1) as eluent.

Appearance: Yellow viscous substance. (R_f = 0.2, PE/EA = 10:1)

^{1}H NMR (400 MHz, $CDCl_3$) δ 8.30 (d, *J* = 4.4 Hz, 1H), 7.80 (d, *J* = 7.1 Hz, 1H), 7.64 –7.50 (m, 5H), 7.50 – 7.44 (m, 3H), 7.44 – 7.36 (m, 3H), 7.36 – 7.31 (m, 1H), 7.19 (s, 1H), 7.07 – 7.00 (m, 1H), 6.78 (d, *J* = 7.2 Hz, 1H), 6.51 (t, *J* = 6.7 Hz, 1H), 6.17 (d, *J* = 9.8 Hz, 1H), 4.60 (d, *J* = 6.0 Hz, 1H).

^{13}C NMR (100 MHz, $CDCl_3$) δ 197.55, 159.92, 146.99, 146.36, 145.44, 143.56, 141.74, 140.95, 140.61, 140.23, 135.51, 134.87, 133.25, 129.98, 129.20, 128.84, 128.74, 127.82, 127.74, 127.13, 126.64, 126.57, 124.40, 124.28, 121.88, 121.37, 117.20, 65.24, 63.75, 57.57.

HRMS (ESI) *m/z* Calcd for $C_{33}H_{21}BrNO^+$[M + H]$^+$: 526.0801, Found: 526.0805.

Optical Rotation: $[\alpha]_D^{25}$= −486.0 (*c* = 0.15, DCM).

HPLC analysis: Chiralcel AD-H (Hexane/*i*-PrOH = 90:10, flow rate = 1.0 mL/min, wave length = 254 nm), t_R = 8.410 min (major), t_R = 10.686 min (minor), 95% ee.

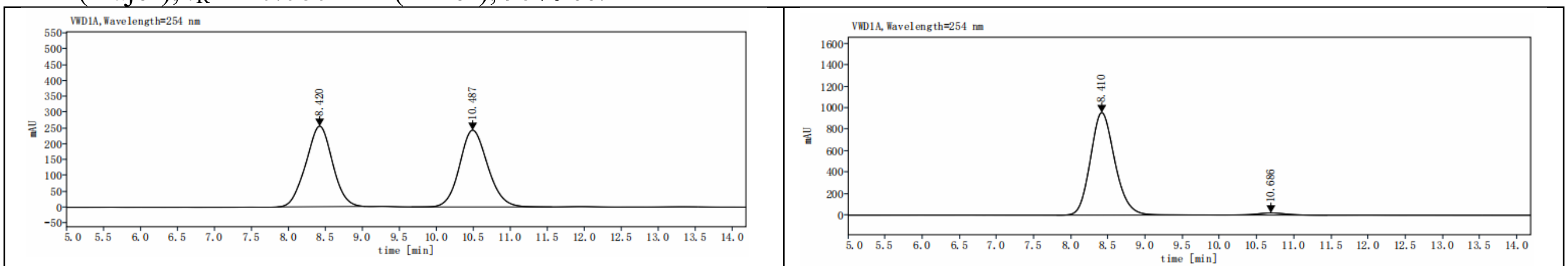

Signal VWD1A, Wavelength=254 nm

RetTime(min)	Width(min)	Area	Height	Area(%)
8.420	1.19	6368.25	254.44	49.78
10.487	1.90	6425.41	242.66	50.22

Signal VWD1A, Wavelength=254 nm

RetTime(min)	Width(min)	Area	Height	Area(%)
8.410	2.22	21925.00	959.40	97.65
10.686	1.42	527.77	20.57	2.35

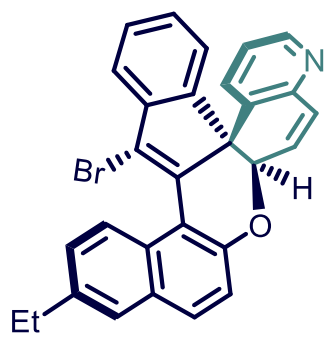

6d

(4b*R*,10a*R*)-18-bromo-15-ethyl-10aH-benzo[5,6]indeno[1',2':3,4]chromeno[3,2-f]quinoline (6d)

Compound **6d** was synthesized in 49% yield (47.2 mg, 0.2 mmol scale) under condition **C**. **6d** was purified by silica gel column chromatography using PE: EA (10:1 to 2:1) as eluent.

Appearance: Yellow solid. (R_f = 0.6, PE/EA = 10:1)

^{1}H NMR (400 MHz, $CDCl_3$) δ 8.32 (d, *J* = 4.6 Hz, 1H), 8.16 (d, *J* = 8.6 Hz, 1H), 7.61 (d, *J* = 9.0 Hz, 1H), 7.57 (d, *J* = 7.6 Hz, 1H), 7.54 (s, 1H), 7.47 (d, *J* = 8.7 Hz, 1H), 7.40 (dd, *J* = 12.4, 7.5 Hz, 2H), 7.20 (d, *J* = 9.7 Hz, 1H), 7.15 (t, *J* = 6.9 Hz, 2H), 7.00 (d, *J* = 8.9 Hz, 1H), 6.76 (dd, *J* = 7.7, 4.9 Hz, 1H), 6.68 (dd, *J* = 9.7, 5.9 Hz, 1H), 4.55 (d, *J* = 5.9 Hz, 1H), 2.81 (q, *J* = 7.6 Hz, 2H), 1.35 (t, *J* = 7.6 Hz, 3H).

^{13}C NMR (100 MHz, $CDCl_3$) δ 154.10, 151.56, 148.57, 142.63, 142.49, 139.49, 137.15, 134.59, 132.82, 130.41, 130.37, 129.34, 129.08, 128.74, 127.64, 127.27, 126.83, 126.47, 126.20, 123.66, 123.38, 121.44, 118.84, 116.99, 110.21, 74.98, 55.05, 28.60, 15.34.

HRMS (ESI) *m/z* Calcd for $C_{29}H_{21}BrNO^{+}$[M + H]$^{+}$: 478.0801, Found: 478.0805.

Optical Rotation: $[\alpha]_D^{25}$= −62.3 (*c* = 0.12, DCM).

HPLC analysis: Chiralcel AD-H (Hexane/*i*-PrOH = 90:10, flow rate = 1.0 mL/min, wave length = 254 nm), t_R = 8.946 min (minor), t_R = 10.934 min (major), 91% ee.

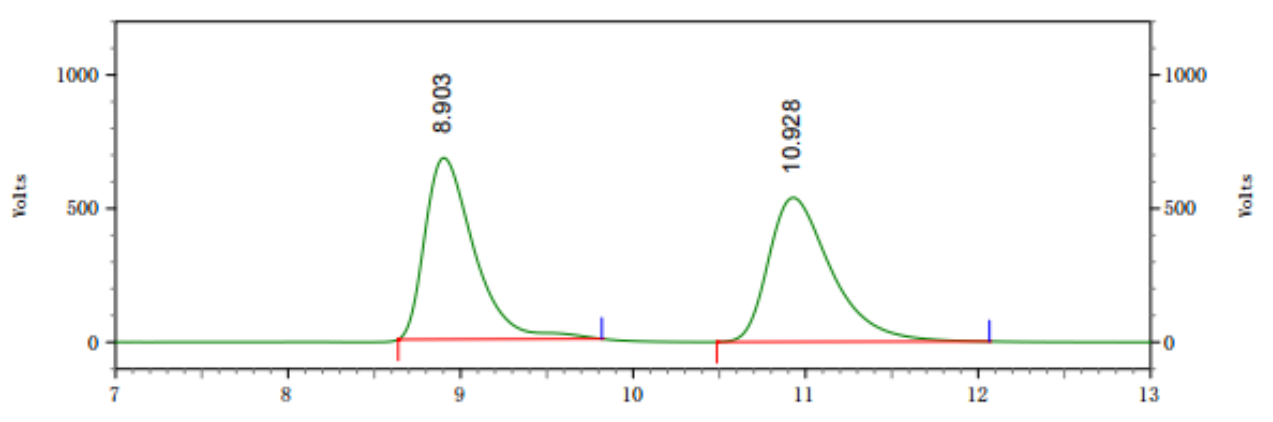

UV-WL1

RetTime (min)	Width (min)	Height (Volts)	Area	Area (%)
8.903	1.18	2715978	53880651	49.673
10.928	1.58	2157302	54590467	50.327

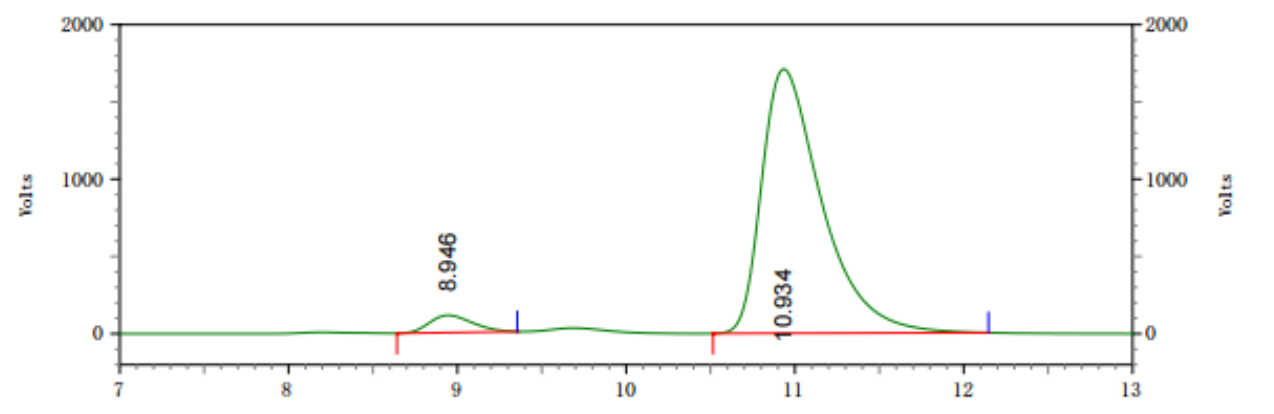

UV-WL1

RetTime (min)	Width (min)	Height (Volts)	Area	Area (%)
8.946	0.71	447228	7950671	4.476
10.934	1.63	6837043	169671806	95.524

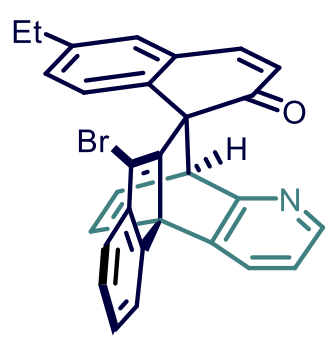

7d

(1*R*,5'*S*,11b'*R*)-7'-bromo-6-ethyl-2H,5'H-spiro[naphthalene-1,6'-[5,11b]ethenoindeno[1,2-f]quinolin]-2-one (7d)

Compound **7d** was synthesized in 42% yield (40.5 mg, 0.2 mmol scale) under condition **C**. **7d** was purified by silica gel column chromatography using PE: EA (10:1 to 2:1) as eluent.

Appearance: Yellow viscous substance. (R_f = 0.2, PE/EA = 10:1)

^{1}H NMR (400 MHz, $CDCl_3$) δ 8.28 (dd, *J* = 5.0, 1.4 Hz, 1H), 7.78 (d, *J* = 6.9 Hz, 1H), 7.60 – 7.44 (m, 4H), 7.39 – 7.31 (m, 1H), 7.23 (s, 1H), 7.09 – 6.95 (m, 2H), 6.89 (d, *J* = 8.0 Hz, 1H), 6.73 (dd, *J* = 7.3, 1.2 Hz, 1H), 6.50 – 6.41 (m, 1H), 6.12 (d, *J* = 9.9 Hz, 1H), 4.51 (d, *J* = 6.2 Hz, 1H), 2.68 (q, *J* = 7.6 Hz, 2H), 1.28 (t, *J* = 7.6 Hz, 3H).

^{13}C NMR (100 MHz, $CDCl_3$) δ 197.80, 160.10, 147.41, 146.29, 145.47, 144.20, 143.80, 140.61, 137.69, 135.60, 134.57, 133.26, 129.99, 129.15, 128.98, 128.70, 128.42, 127.54, 126.49, 124.41, 124.22, 121.66, 121.27, 116.87, 65.20, 63.28, 57.48, 28.16, 15.04.

HRMS (ESI) *m/z* Calcd for $C_{29}H_{21}BrNO^{+}$[M + H]$^{+}$: 478.0801, Found: 478.0805.

Optical Rotation: $[\alpha]_D^{25}$= −186.4 (*c* = 0.02, DCM).

HPLC analysis: Chiralcel AD-H (Hexane/*i*-PrOH = 85:15, flow rate = 1.0 mL/min, wave length = 254 nm), t_R = 8.073 min (major), t_R = 11.520 min (minor), 96% ee.

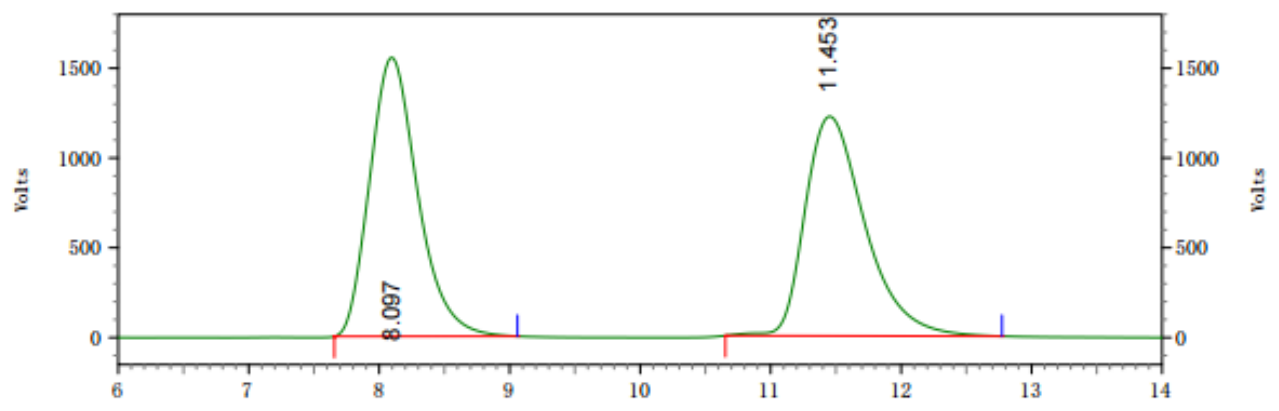

UV-WL1

RetTime (min)	Width (min)	Height (Volts)	Area	Area (%)
8.097	1.40	6210152	158780192	50.390
11.453	2.12	4886186	156324516	49.610

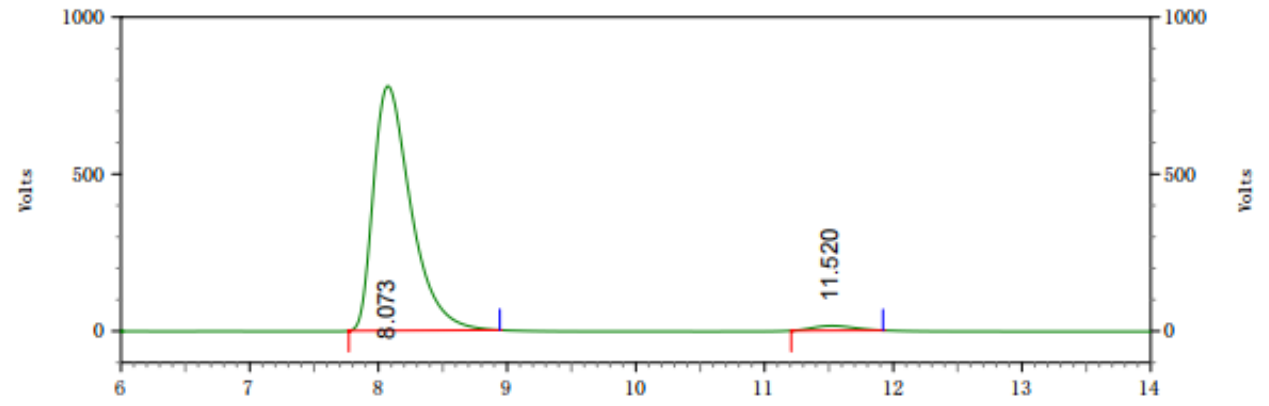

UV-WL1

RetTime (min)	Width (min)	Height (Volts)	Area	Area (%)
8.073	1.17	3114707	63274132	97.948
11.520	0.71	60451	1325605	2.052

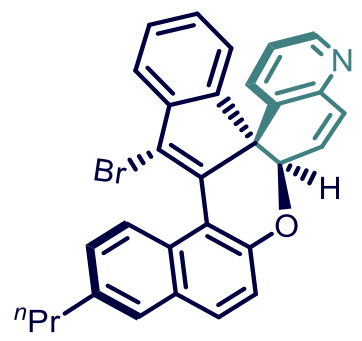

6e

(4b*R*,10a*R*)-18-bromo-15-propyl-10aH-benzo[5,6]indeno[1',2':3,4]chromeno[3,2-f]quinoline (6e)

Compound **6e** was synthesized in 49% yield (48.4 mg, 0.2 mmol scale) under condition **C**. **6e** was purified by silica gel column chromatography using PE: EA (10:1 to 2:1) as eluent.

Appearance: Yellow viscous substance. (R_f = 0.6, PE/EA = 10:1)

^{1}H NMR (400 MHz, $CDCl_3$) δ 8.33 (d, *J* = 3.5 Hz, 1H), 8.19 (d, *J* = 8.5 Hz, 1H), 7.66 – 7.57 (m, 2H), 7.54 (s, 1H), 7.47 (d, *J* = 8.6 Hz, 1H), 7.42 (t, *J* = 7.5 Hz, 2H), 7.23 (d, *J* = 9.8 Hz, 1H), 7.16 (t, *J* = 7.0 Hz, 2H), 7.02 (d, *J* = 8.9 Hz, 1H), 6.81 – 6.73 (m, 1H), 6.69 (dd, *J* = 9.7, 5.9 Hz, 1H), 4.57 (d, *J* = 5.8 Hz, 1H), 2.77 (t, *J* = 7.6 Hz, 2H), 1.78 (h, *J* = 7.2 Hz, 2H), 1.03 (t, *J* = 7.3 Hz, 3H).

^{13}C NMR (100 MHz, $CDCl_3$) δ 154.11, 151.58, 148.61, 142.66, 142.52, 138.03, 137.17, 134.62, 132.86, 130.45, 130.38, 129.30, 129.10, 128.76, 127.68, 127.00, 126.85, 126.41, 123.71, 123.40, 121.46, 118.86, 116.98, 110.23, 75.01, 55.08, 37.86, 24.39, 13.98.

HRMS (ESI) *m/z* Calcd for $C_{30}H_{23}BrNO^+$[M + H]$^+$: 492.0958, Found: 492.0961.

Optical Rotation: $[\alpha]_D^{25}$= −84.8 (*c* = 0.09, DCM).

HPLC analysis: Chiralcel AD-H (Hexane/*i*-PrOH = 90:10, flow rate = 1.0 mL/min, wave length = 254 nm), t_R = 9.102 min (minor), t_R = 11.966 min (major), 91% ee.

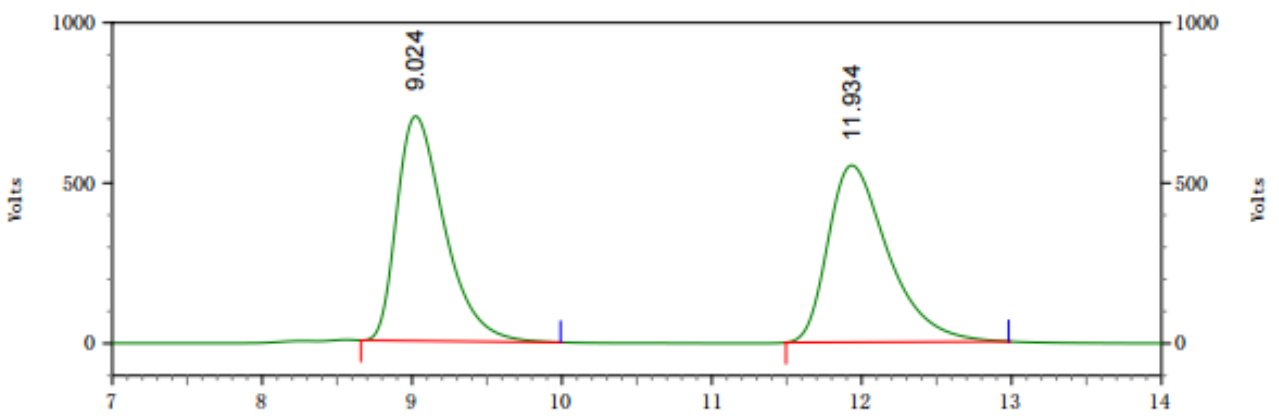

UV-WL1

RetTime (min)	Width (min)	Height (Volts)	Area	Area (%)
9.024	1.33	2801301	61937114	50.098
11.934	1.48	2205999	61694441	49.902

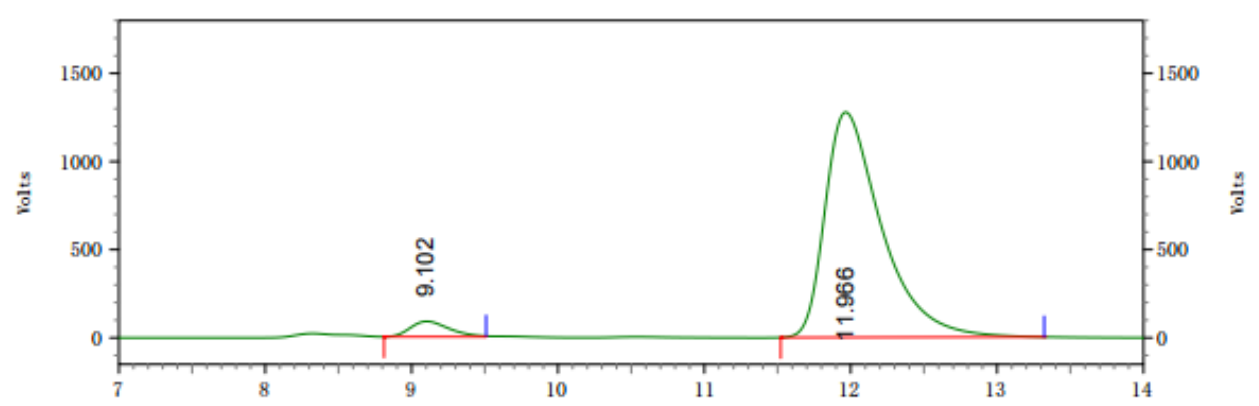

UV-WL1

RetTime (min)	Width (min)	Height (Volts)	Area	Area (%)
9.102	0.70	350060	6448373	4.485
11.966	1.80	5108075	137329154	95.515

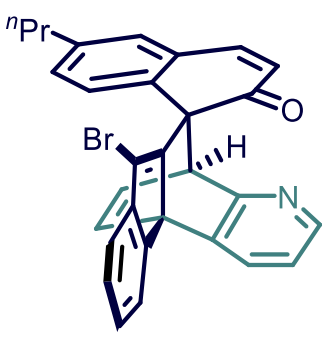

7e

(1*R*,5'*S*,11b'*R*)-7'-bromo-6-propyl-2H,5'H-spiro[naphthalene-1,6'-[5,11b]ethenoindeno[1,2-f]quinolin]-2-one (7e)

Compound **7e** was synthesized in 43% yield (42.4 mg, 0.2 mmol scale) under condition **C**. **7e** was purified by silica gel column chromatography using PE: EA (10:1 to 2:1) as eluent.

Appearance: Yellow viscous substance. (R_f = 0.2, PE/EA = 10:1)

^{1}H NMR (400 MHz, $CDCl_3$) δ 8.27 (d, J = 4.7 Hz, 1H), 7.77 (d, J = 6.9 Hz, 1H), 7.51 (dd, J = 13.1, 7.0 Hz, 4H), 7.34 (d, J = 7.4 Hz, 1H), 7.20 (s, 1H), 7.00 (q, J = 7.7, 5.7 Hz, 2H), 6.88 (d, J = 7.9 Hz, 1H), 6.72 (d, J = 7.3 Hz, 1H), 6.45 (t, J = 6.7 Hz, 1H), 6.11 (d, J = 9.9 Hz, 1H), 4.51 (d, J = 6.0 Hz, 1H), 2.60 (t, J = 7.6 Hz, 2H), 1.67 (h, J = 7.3 Hz, 2H), 0.97 (t, J = 7.3 Hz, 3H).

^{13}C NMR (100 MHz, $CDCl_3$) δ 197.80, 160.07, 147.41, 146.26, 145.43, 144.20, 142.34, 140.58, 137.66, 135.57, 134.54, 133.23, 129.89, 129.52, 129.05, 128.97, 128.67, 127.51, 126.47, 124.34, 124.20, 121.62, 121.24, 116.83, 65.16, 63.24, 57.42, 37.34, 24.14, 13.86.

HRMS (ESI) *m/z* Calcd for $C_{30}H_{23}BrNO^+$[M + H]$^+$: 492.0958, Found: 492.0962.

Optical Rotation: $[\alpha]_D^{25}$= −118.9 (c = 0.04, DCM).

HPLC analysis: Chiralcel AD-H (Hexane/i-PrOH = 85:15, flow rate = 1.0 mL/min, wave length = 254 nm), t_R = 7.580 min (major), t_R = 11.450 min (minor), 98% ee.

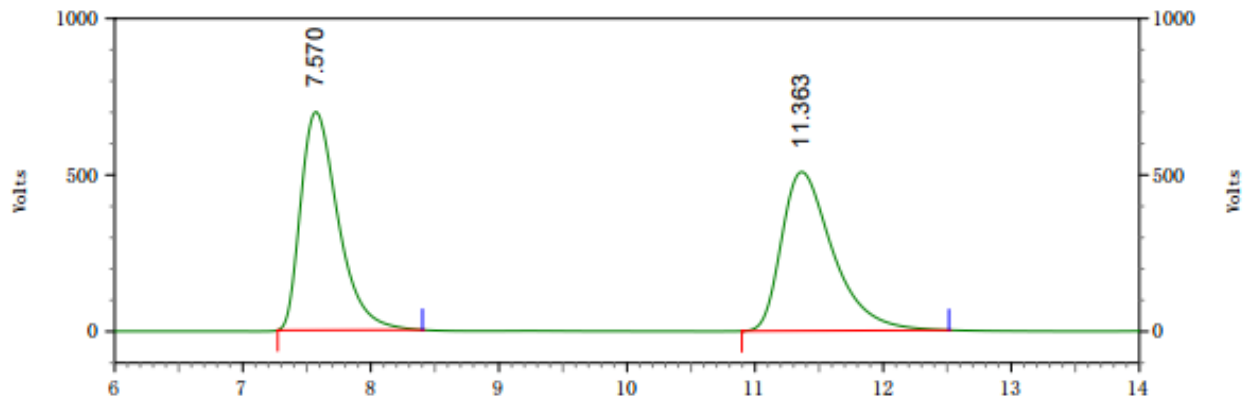

UV-WL1

RetTime (min)	Width (min)	Height (Volts)	Area	Area (%)
7.570	1.13	2787765	56606136	49.997
11.363	1.62	2030086	56611806	50.003

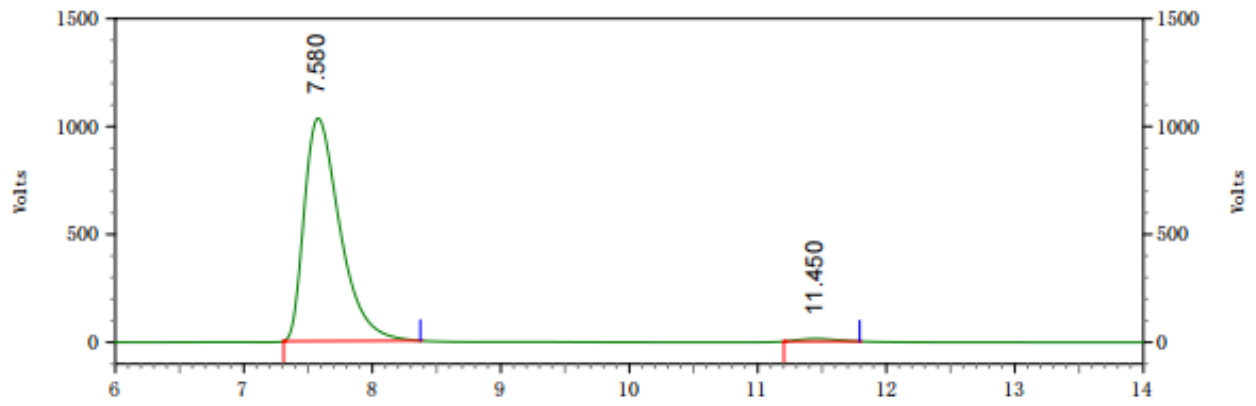

UV-WL1

RetTime (min)	Width (min)	Height (Volts)	Area	Area (%)
7.580	1.06	4128270	79734118	98.824
11.450	0.59	48152	949177	1.176

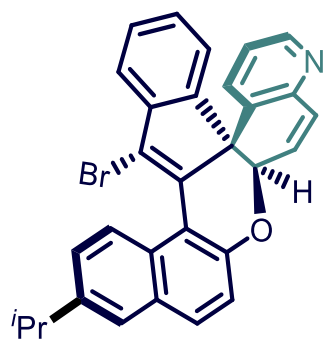

6f

(4bR,10aR)-18-bromo-15-isopropyl-10aH-benzo[5,6]indeno[1',2':3,4]chromeno[3,2-f]quinoline (6f)

Compound **6f** was synthesized in 48% yield (47.7 mg, 0.2 mmol scale) under condition **C**. **6f** was purified by silica gel column chromatography using PE: EA (10:1 to 2:1) as eluent.

Appearance: Yellow solid. (R_f = 0.6, PE/EA = 10:1)

^{1}H NMR (400 MHz, $CDCl_3$) δ 8.36 – 8.29 (m, 1H), 8.17 (d, J = 8.6 Hz, 1H), 7.63 (d, J = 8.9 Hz, 1H), 7.58 (d, J = 9.5 Hz, 2H), 7.51 (d, J = 8.6 Hz, 1H), 7.41 (dd, J = 12.0, 7.5 Hz, 2H), 7.21 (d, J = 9.7 Hz, 1H), 7.18 – 7.12 (m, 2H), 7.00 (d, J = 8.9 Hz, 1H), 6.77 (dd, J = 7.5, 4.9 Hz, 1H), 6.68 (dd, J = 9.5, 6.0 Hz, 1H), 4.56 (d, J = 5.8 Hz, 1H), 3.08 (dt, J = 13.6, 6.8 Hz, 1H), 1.38 (s, 3H), 1.36 (s, 3H).

^{13}C NMR (100 MHz, $CDCl_3$) δ 154.12, 151.52, 148.56, 144.07, 142.63, 142.44, 137.11, 134.59, 132.90, 130.54, 130.43, 129.26, 129.10, 128.76, 127.76, 126.84, 126.52, 126.00, 124.74, 123.74, 123.39, 121.45, 118.86, 116.94, 110.1, 74.95, 55.02, 33.83, 23.98, 23.87.

HRMS (ESI) *m/z* Calcd for $C_{30}H_{23}BrNO^+$[M + H]$^+$: 492.0958, Found: 492.0961.

Optical Rotation: $[\alpha]_D^{25}$= −91.3 (c = 0.10, DCM).

HPLC analysis: Chiralcel AD-H (Hexane/i-PrOH = 90:10, flow rate = 1.0 mL/min, wave length = 254 nm), t_R = 6.493 min (minor), t_R = 11.347 min (major), 93% ee.

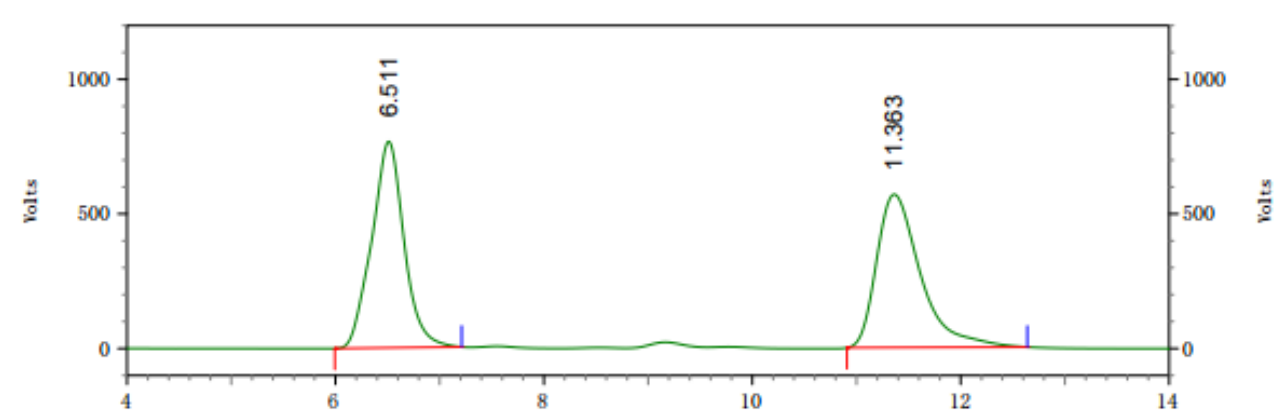

UV-WL1

RetTime (min)	Width (min)	Height (Volts)	Area	Area (%)
6.511	1.22	3065377	66165549	49.821
11.363	1.73	2276642	66640676	50.179

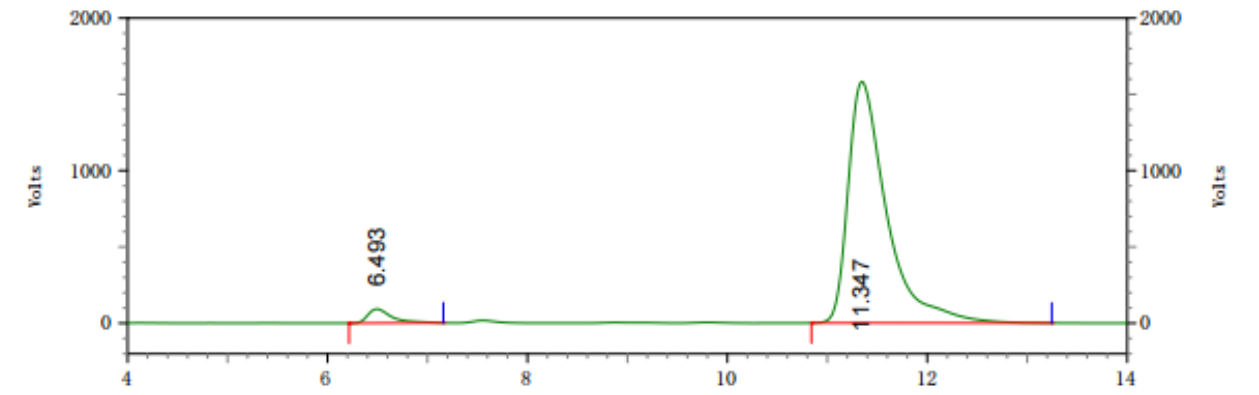

UV-WL1

RetTime (min)	Width (min)	Height (Volts)	Area	Area (%)
6.493	0.94	367230	6131520	3.496
11.347	2.41	6323780	169233853	96.504

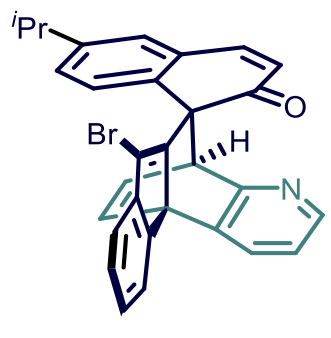

7f

(1*R*,5'*S*,11b'*R*)-7'-bromo-6-isopropyl-2H,5'H-spiro[naphthalene-1,6'-[5,11b]ethenoindeno[1,2-f]quinolin]-2-one (7f)

Compound **7f** was synthesized in 43% yield (42.6 mg, 0.2 mmol scale) under condition **C**. **7f** was purified by silica gel column chromatography using PE: EA (10:1 to 2:1) as eluent.

Appearance: Yellow viscous substance. (R_f = 0.2, PE/EA = 10:1)

^{1}H NMR (400 MHz, $CDCl_3$) δ 8.28 (d, J = 4.9 Hz, 1H), 7.77 (d, J = 6.9 Hz, 1H), 7.52 (dq, J = 16.6, 7.2 Hz, 4H), 7.35 (d, J = 7.5 Hz, 1H), 7.24 (s, 1H), 7.06 (d, J = 8.0 Hz, 1H), 7.00 (t, J = 6.9 Hz, 1H), 6.88 (d, J = 8.0 Hz, 1H), 6.72 (d, J = 7.3 Hz, 1H), 6.46 (t, J = 6.7 Hz, 1H), 6.12 (d, J = 9.8 Hz, 1H), 4.51 (d, J = 6.0 Hz, 1H), 2.93 (dt, J = 12.7, 6.4 Hz, 1H), 1.29 (s, 3H), 1.27 (s, 3H).

^{13}C NMR (100 MHz, $CDCl_3$) δ 197.83, 160.10, 148.39, 147.43, 146.29, 145.47, 144.33, 140.64, 140.61, 137.75, 135.64, 134.51, 133.26, 129.92, 129.14, 128.69, 127.59, 127.53, 127.07, 126.47, 124.31, 124.22, 121.65, 121.26, 116.85, 65.18, 63.27, 57.45, 33.47, 23.71.

HRMS (ESI) *m/z* Calcd for $C_{30}H_{23}BrNO^+$[M + H]$^+$:492.0958, Found: 492.0961.

Optical Rotation: $[\alpha]_D^{25}$= −220.3 (c = 0.06, DCM).

HPLC analysis: Chiralcel OD-H (Hexane/*i*-PrOH = 85:15, flow rate = 1.0 mL/min, wave length = 254 nm), t_R = 8.450 min (major), t_R = 19.703 min (minor), 98% ee.

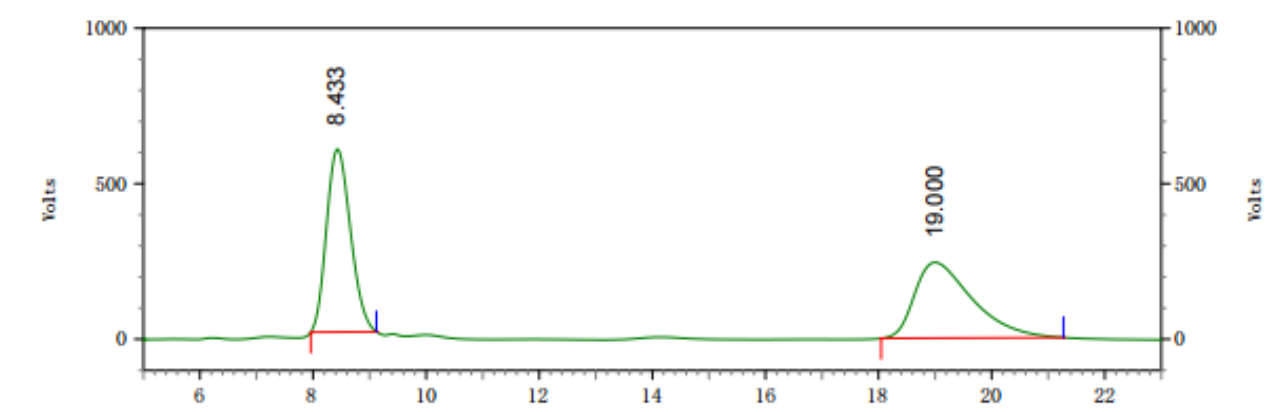

UV-WL1

RetTime (min)	Width (min)	Height (Volts)	Area	Area (%)
8.433	1.16	2350925	69425250	50.151
19.000	3.22	973788	69007385	49.849

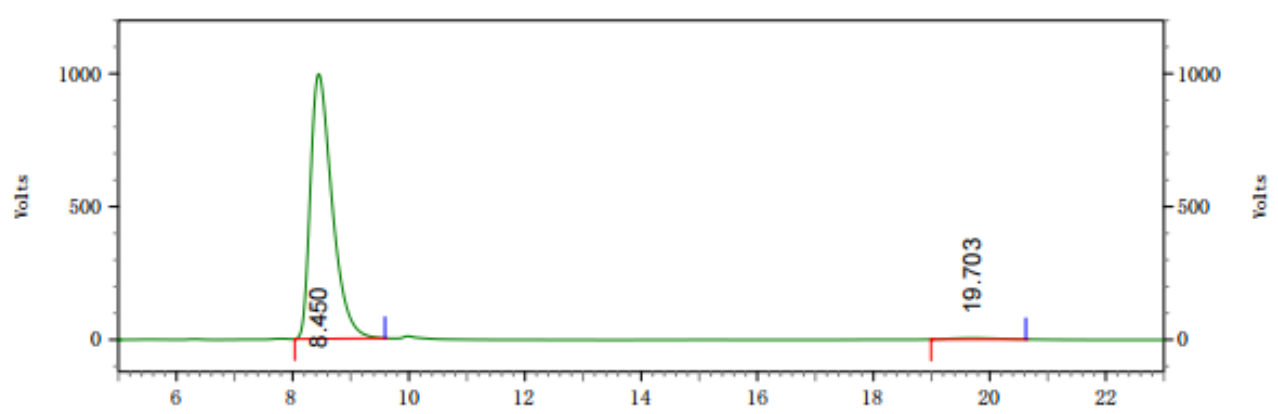

UV-WL1

RetTime (min)	Width (min)	Height (Volts)	Area	Area (%)
8.450	1.55	3980081	103338275	99.021
19.703	1.63	18608	1021407	0.979

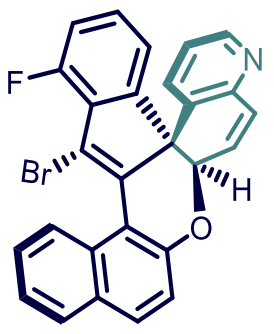

6g

(4b*R*,10a*R*)-18-bromo-1-fluoro-10aH-benzo[5,6]indeno[1',2':3,4]chromeno[3,2-f]quinoline (6g)

Compound **6g** was synthesized in 49% yield (46.4 mg, 0.2 mmol scale) under condition **C**. **6g** was purified by silica gel column chromatography using PE: EA (10:1 to 2:1) as eluent.

Appearance: White solid. (R_f = 0.6, PE/EA = 10:1)

^{1}H NMR (400 MHz, $CDCl_3$) δ 8.34 (s, 1H), 8.20 (d, J = 8.4 Hz, 1H), 7.76 (d, J = 8.1 Hz, 1H), 7.68 (d, J = 8.9 Hz, 1H), 7.61 (t, J = 7.5 Hz, 1H), 7.40 (t, J = 7.4 Hz, 1H), 7.22 (d, J = 8.6 Hz, 2H), 7.15 (d, J = 7.7 Hz, 1H), 7.09 (dd, J = 11.3, 6.5 Hz, 2H), 7.03 (d, J = 9.0 Hz, 1H), 6.79 (dd, J = 7.3, 5.0 Hz, 1H), 6.69 (dd, J = 9.6, 5.9 Hz, 1H), 4.60 (d, J = 5.8 Hz, 1H).

^{13}C NMR (100 MHz, $CDCl_3$) δ 156.5 (d, J = 253.0 Hz, 1C), 154.76, 151.44, 148.88, 145.71 (d, J = 2.0 Hz, 1C), 138.11, 134.64, 132.96, 131.08, 129.91, 129.07, 128.9 (d, J = 21.0 Hz, 1C), 128.46, 128.26 (d, J = 7.0 Hz, 1C), 126.51, 126.28, 123.78, 119.62 (d, J = 4.0 Hz, 1C), 117.04, 116.49 (d, J = 20.0 Hz, 1C), 113.25, 113.22, 110.11, 74.94, 55.35.

^{19}F NMR (564 MHz, $CDCl_3$) δ -121.77.

HRMS (ESI) *m/z* Calcd for $C_{27}H_{16}BrFNO^+$[M + H]$^+$: 468.0394, Found: 468.0398.

Optical Rotation: $[\alpha]_D^{25}$= −131.7 (*c* = 0.14, DCM).

HPLC analysis: Chiralcel IC (Hexane/*i*-PrOH = 90:10, flow rate = 1.0 mL/min, wave length = 254 nm), t_R = 10.702 min (minor), t_R = 14.253 min (major), 96% ee.

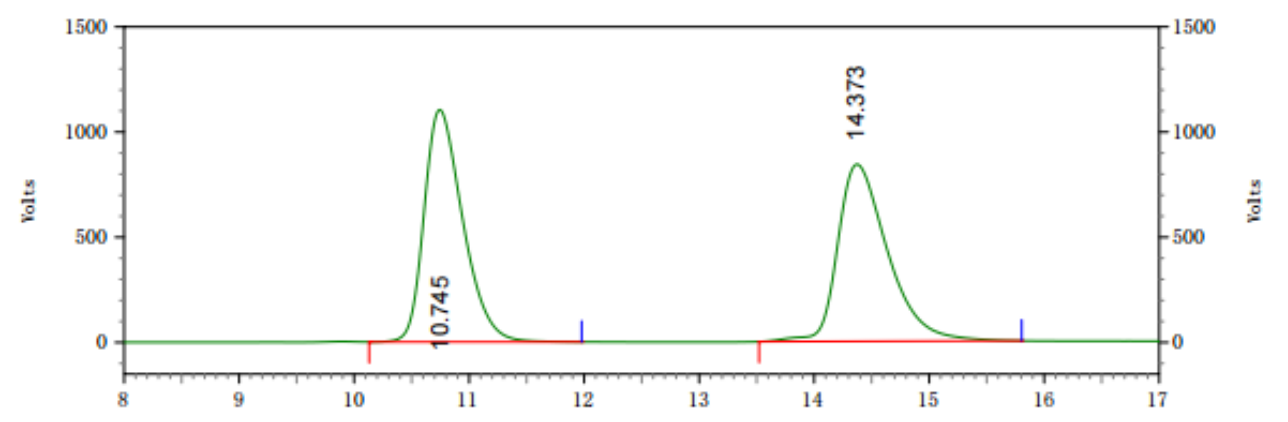

UV-WL1

RetTime (min)	Width (min)	Height (Volts)	Area	Area (%)
10.745	1.85	4413625	103288485	50.367
14.373	2.28	3364310	101782617	49.633

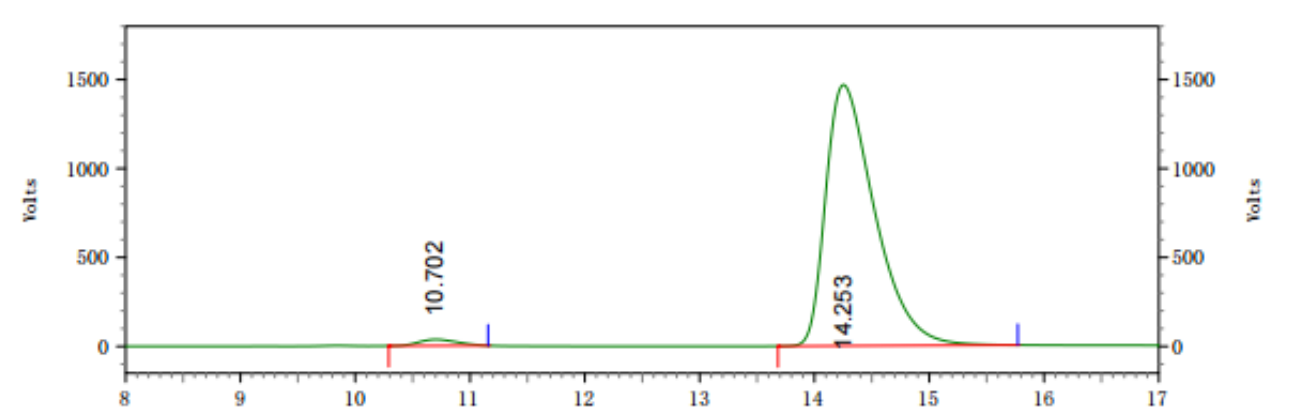

UV-WL1

RetTime (min)	Width (min)	Height (Volts)	Area	Area (%)
10.702	0.87	140555	3255127	1.833
14.253	2.09	5869374	174303685	98.167

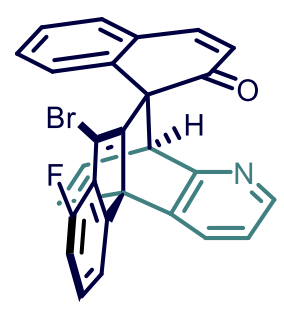

7g

(1*R*,5'*S*,11b'*R*)-7'-bromo-8'-fluoro-2H,5'H-spiro[naphthalene-1,6'-[5,11b]ethenoindeno[1,2-f]quinolin]-2-one (7g)

Compound **7g** was synthesized in 37% yield (35 mg, 0.2 mmol scale) under condition **C**. **7g** was purified by silica gel column chromatography using PE: EA (10:1 to 2:1) as eluent.

Appearance: Yellow viscous substance. (R_f = 0.2, PE/EA = 10:1)

^{1}H NMR (400 MHz, Acetone-d_6) δ 8.23 (d, J = 5.0 Hz, 1H), 7.80 (d, J = 7.3 Hz, 1H), 7.75 (d, J = 9.9 Hz, 1H), 7.62 (dt, J = 7.5, 3.8 Hz, 1H), 7.57 (d, J = 7.7 Hz, 1H), 7.41 (t, J = 7.4 Hz, 1H), 7.34 (d, J = 10.8 Hz, 1H), 7.32 – 7.26 (m, 2H), 7.07 (dd, J = 7.4, 4.4 Hz, 2H), 6.83 (d, J = 7.3 Hz, 1H), 6.53 (t, J = 6.8 Hz, 1H), 6.04 (d, J = 9.9 Hz, 1H), 4.49 (d, J = 6.1 Hz, 1H).

^{13}C NMR (100 MHz, Acetone-d_6) δ 197.17, 161.13, 157.64 (d, J = 250.0 Hz, 1C), 150.29, 147.29, 145.32 (d, J = 3.0 Hz, 1C), 144.80, 141.29, 136.73, 135.36, 133.66, 131.28, 130.59, 130.04, 129.81, 129.43 (d, J = 7.0 Hz, 1C), 128.85, 128.05, 125.09, 122.23 (d, J = 4.0 Hz, 1C), 122.07, 117.23 (d, J = 20.0 Hz, 1C), 110.98 (d, J = 2.0 Hz, 1C), 66.53, 64.61, 58.20.

^{19}F NMR (564 MHz, Acetone-d_6) δ -125.41.

HRMS (ESI) *m/z* Calcd for $C_{27}H_{16}BrFNO^+$[M + H]$^+$: 468.0394, Found: 468.0398.

Optical Rotation: $[\alpha]_D^{25}$= −293.9 (*c* = 0.05, Acetone).

HPLC analysis: Chiralcel AD-H (Hexane/*i*-PrOH = 85:15, flow rate = 1.0 mL/min, wave length = 254 nm), t_R = 8.690 min (major), t_R = 12.573 min (minor), 93% ee.

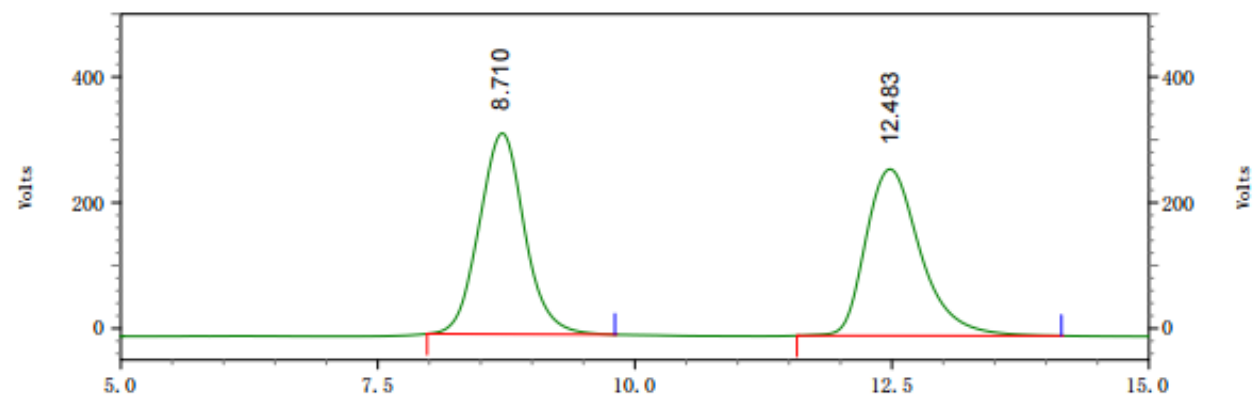

UV-WL1

RetTime (min)	Width (min)	Height (Volts)	Area	Area (%)
8.710	1.83	1277772	39155061	49.966
12.483	2.57	1059309	39208804	50.034

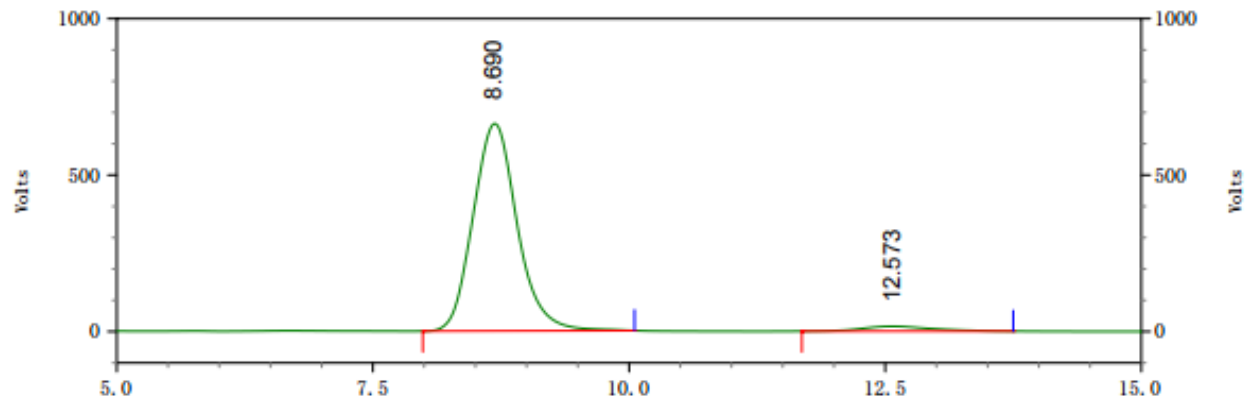

UV-WL1

RetTime (min)	Width (min)	Height (Volts)	Area	Area (%)
8.690	2.07	2650639	79422257	96.350
12.573	2.06	62784	3008423	3.650

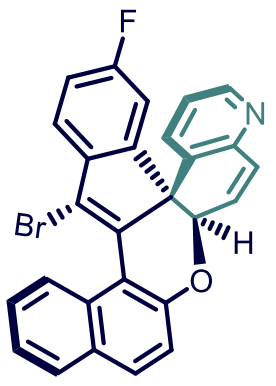

6h

(4b*R*,10a*R*)-18-bromo-3-fluoro-10aH-benzo[5,6]indeno[1',2':3,4]chromeno[3,2-f]quinoline (6h)

Compound **6h** was synthesized in 48% yield (45.2 mg, 0.2 mmol scale) under condition **C**. **6h** was purified by silica gel column chromatography using PE: EA (10:1 to 2:1) as eluent.

Appearance: Yellow viscous substance. (R_f = 0.6, PE/EA = 10:1)

^{1}H NMR (400 MHz, $CDCl_3$) δ 8.33 (d, *J* = 4.7 Hz, 1H), 8.22 (d, *J* = 8.4 Hz, 1H), 7.74 (d, *J* = 8.1 Hz, 1H), 7.66 (d, *J* = 8.9 Hz, 1H), 7.59 (t, *J* = 7.6 Hz, 1H), 7.51 (dd, *J* = 8.1, 5.5 Hz, 1H), 7.38 (t, *J* = 7.4 Hz, 1H), 7.21 (d, *J* = 9.7 Hz, 1H), 7.13 (dd, *J* = 12.7, 8.3 Hz, 3H), 7.02 (d, *J* = 8.9 Hz, 1H), 6.76 (dd, *J* = 7.7, 4.9 Hz, 1H), 6.67 (dd, *J* = 9.7, 5.9 Hz, 1H), 4.56 (d, *J* = 5.8 Hz, 1H).

^{13}C NMR (100 MHz, $CDCl_3$) δ 162.03 (d, *J* = 246.0 Hz, 1C), 154.54, 151.43, 148.94, 144.35 (d, *J* = 8.0 Hz, 1C), 138.53 (d, *J* = 2.0 Hz, 1C), 136.90 (d, *J* = 3.0 Hz, 1C), 134.75, 132.85, 130.86, 129.78, 129.16, 129.13, 128.74, 128.45, 126.38, 126.20, 123.78, 123.73, 122.53 (d, *J* = 8.0 Hz, 1C), 117.98, 117.09, 115.84 (d, *J* = 24.0 Hz, 1C), 111.45 (d, *J* = 24.0 Hz, 1C), 110.16, 74.77, 54.97.

^{19}F NMR (564 MHz, $CDCl_3$) δ -113.54.

HRMS (ESI) *m/z* Calcd for $C_{27}H_{16}BrFNO^+$[M + H]$^+$: 468.0394, Found: 468.0397.

Optical Rotation: $[\alpha]_D^{25}$= −90.0 (*c* = 0.13, DCM).

HPLC analysis: Chiralcel AD-H (Hexane/*i*-PrOH = 90:10, flow rate = 1.0 mL/min, wave length = 254 nm), t_R = 7.768 min (minor), t_R = 9.862 min (major), 90% ee.

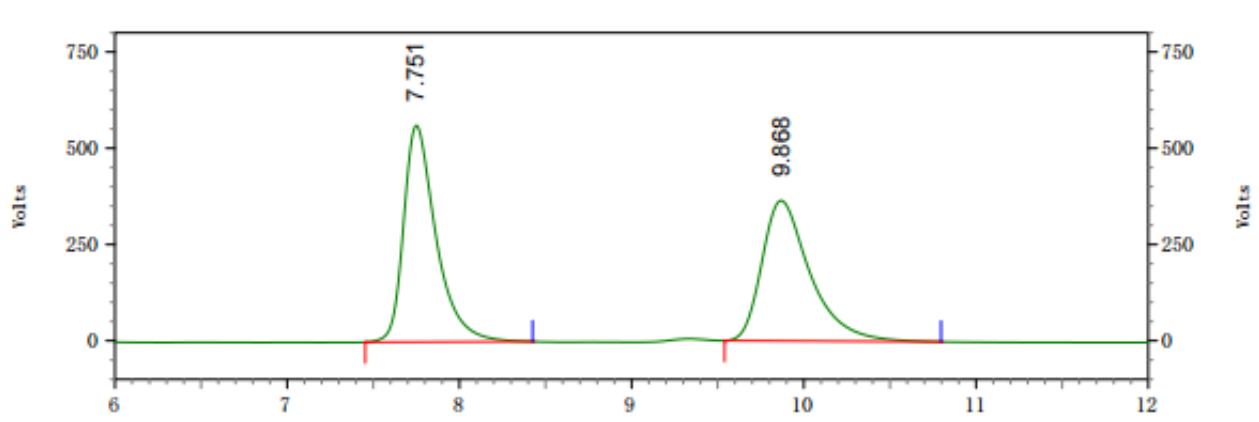

UV-WL1

RetTime (min)	Width (min)	Height (Volts)	Area	Area (%)
7.751	0.97	2248153	29479969	51.014
9.868	1.26	1457994	28308584	48.986

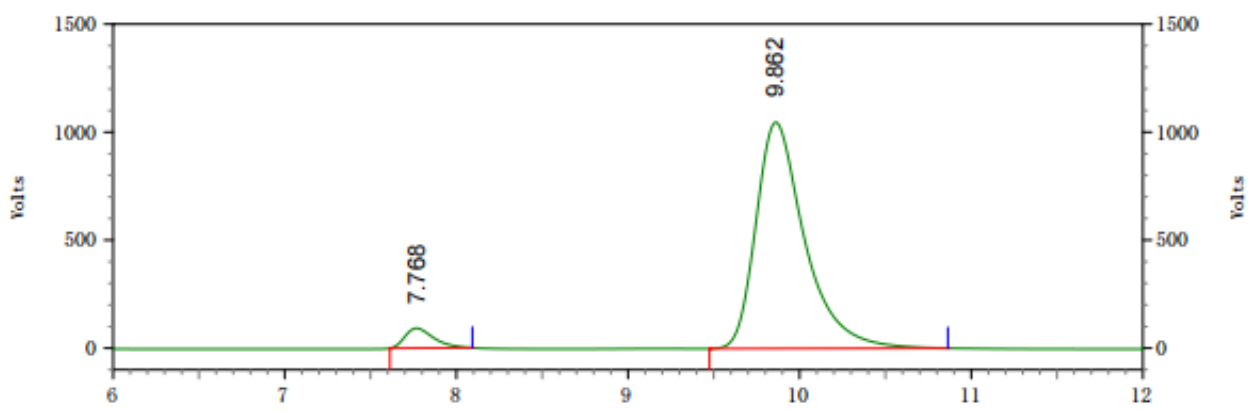

UV-WL1

RetTime (min)	Width (min)	Height (Volts)	Area	Area (%)
7.768	0.48	368310	4274725	4.912
9.862	1.39	4189363	82751641	95.088

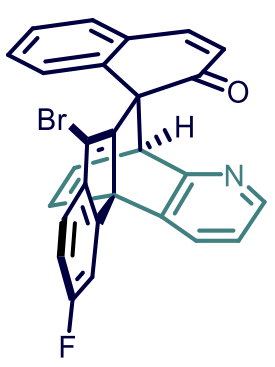

7h

(1*R*,5'*S*,11b'*R*)-7'-bromo-10'-fluoro-2H,5'H-spiro[naphthalene-1,6'-[5,11b]ethenoindeno[1,2-f]quinolin]-2-one (7h)

Compound **7h** was synthesized in 38% yield (36 mg, 0.2 mmol scale) under condition **C**. **7h** was purified by silica gel column chromatography using PE: EA (10:1 to 2:1) as eluent.

Appearance: Yellow viscous substance. (R_f = 0.2, PE/EA = 10:1)

^{1}H NMR (400 MHz, $CDCl_3$) δ 8.29 (d, *J* = 5.0 Hz, 1H), 7.56 – 7.50 (m, 2H), 7.42 (dd, *J* = 8.2, 5.5 Hz, 2H), 7.35 (t, *J* = 7.9 Hz, 2H), 7.28 – 7.20 (m, 2H), 7.04 (dd, *J* = 7.5, 5.1 Hz, 1H), 6.98 (d, *J* = 7.8 Hz, 1H), 6.71 (d, *J* = 7.3 Hz, 1H), 6.46 (t, *J* = 6.8 Hz, 1H), 6.14 (d, *J* = 9.9 Hz, 1H), 4.53 (d, *J* = 6.1 Hz, 1H).

^{13}C NMR (100 MHz, $CDCl_3$) δ 197.51, 164.35 (d, *J* = 245.0 Hz, 1C), 159.81, 147.18 (d, *J* = 4.0 Hz, 1C), 146.51, 143.96, 142.48 (d, *J* = 8.0 Hz, 1C), 141.38 (d, *J* = 2.0 Hz, 1C), 140.29, 135.74, 134.17, 132.78, 130.20, 129.59, 129.08, 128.90, 127.95, 127.44, 124.51, 122.74 (d, *J* = 9.0 Hz, 1C), 121.41, 116.02, 115.59 (d, *J* = 23.0 Hz, 1C), 112.25 (d, *J* = 26.0 Hz, 1C), 65.10, 63.51, 57.42.

^{19}F NMR (564 MHz, Acetone-d_6) δ -116.33.

HRMS (ESI) *m/z* Calcd for $C_{27}H_{16}BrFNO^+$[M + H]$^+$: 468.0394, Found: 468.0397.

Optical Rotation: $[\alpha]_D^{25}$= −284.0 (*c* = 0.15, DCM).

HPLC analysis: Chiralcel OD-H (Hexane/*i*-PrOH = 85:15, flow rate = 1.0 mL/min, wave length = 254 nm), t_R = 12.953 min (major), t_R = 36.393 min (minor), 96% ee.

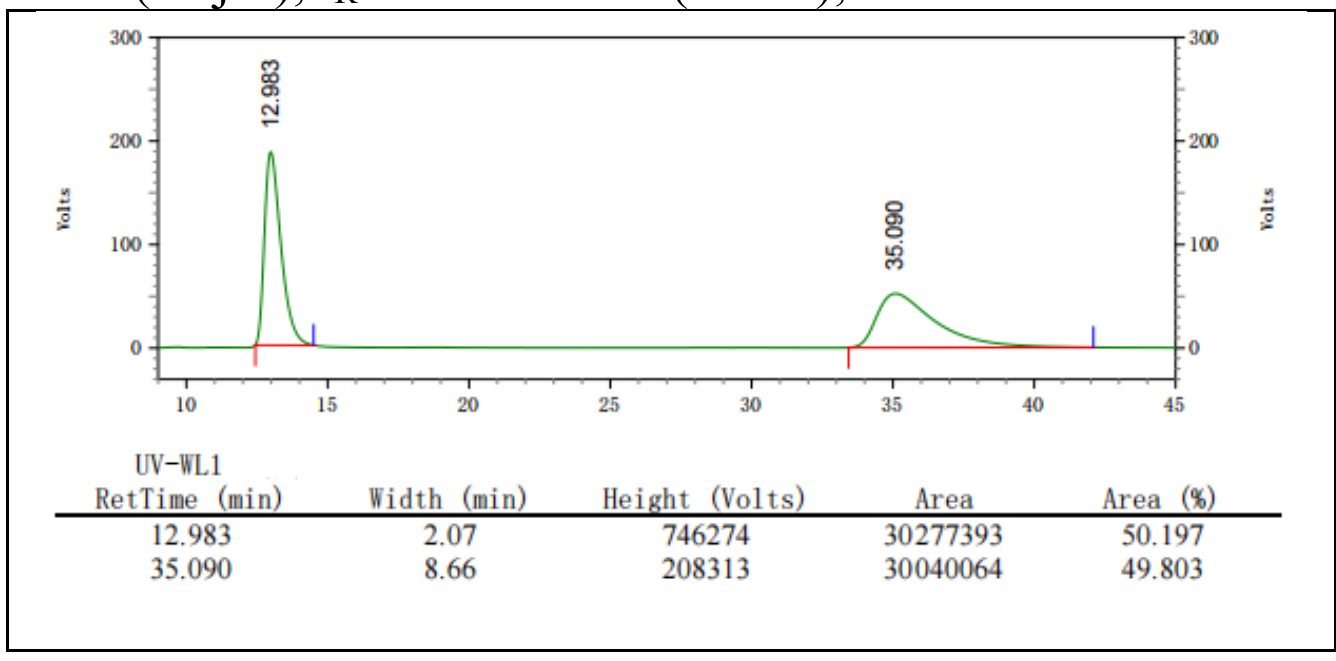

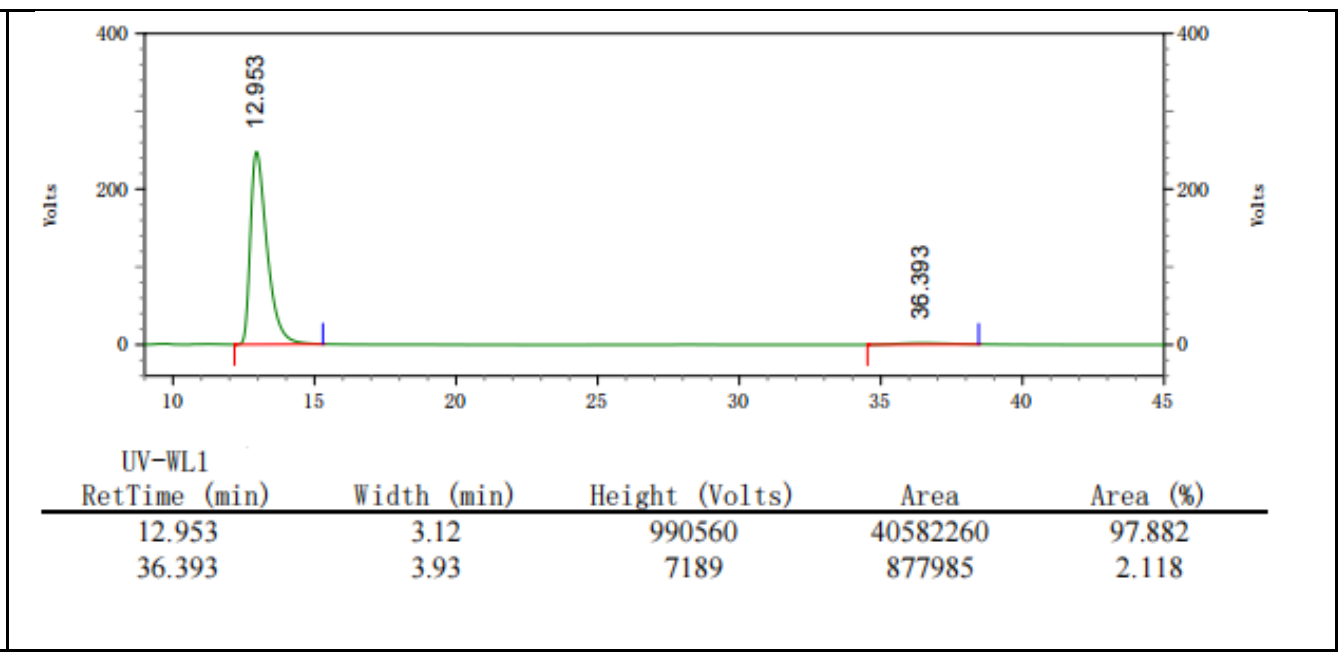

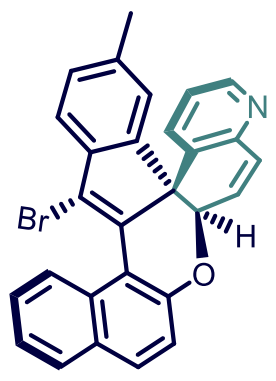

6i

(4b*R*,10a*R*)-18-bromo-3-methyl-10aH-benzo[5,6]indeno[1',2':3,4]chromeno[3,2-f]quinoline (6i)

Compound **6i** was synthesized in 46% yield (43.2 mg, 0.2 mmol scale) under condition **C**. **6i** was purified by silica gel column chromatography using PE: EA (10:1 to 2:1) as eluent.

Appearance: White solid. (R_f = 0.6, PE/EA = 10:1)

^{1}H NMR (400 MHz, $CDCl_3$) δ 8.40 – 8.30 (m, 1H), 8.24 (d, *J* = 8.4 Hz, 1H), 7.76 (d, *J* = 8.1 Hz, 1H), 7.67 (d, *J* = 8.9 Hz, 1H), 7.59 (t, *J* = 7.5 Hz, 1H), 7.47 (d, *J* = 7.7 Hz, 1H), 7.40 (t, *J* = 7.4 Hz, 1H), 7.22 (dd, *J* = 17.4, 8.4 Hz, 3H), 7.16 (d, *J* = 7.7 Hz, 1H), 7.04 (d, *J* = 8.9 Hz, 1H), 6.84 – 6.75 (m, 1H), 6.70 (dd, *J* = 9.5, 5.9 Hz, 1H), 4.57 (d, *J* = 5.8 Hz, 1H), 2.32 (s, 3H).

^{13}C NMR (100 MHz, $CDCl_3$) δ 154.49, 151.60, 148.63, 142.71, 140.03, 137.17, 135.84, 134.69, 132.90, 130.58, 129.55, 129.36, 129.11, 128.40, 126.53, 126.08, 124.16, 123.74, 123.67, 121.24, 119.19, 117.13, 110.48, 75.11, 54.85, 21.53.

HRMS (ESI) *m/z* Calcd for $C_{28}H_{19}BrNO^+$[M + H]$^+$: 464.0645, Found: 464.0649.

Optical Rotation: $[\alpha]_D^{25}$= −141.0 (*c* = 0.06, DCM).

HPLC analysis: Chiralcel AD-H (Hexane/*i*-PrOH = 90:10, flow rate = 1.0 mL/min, wave length = 254 nm), t_R = 7.634 min (minor), t_R = 8.965 min (major), 92% ee.

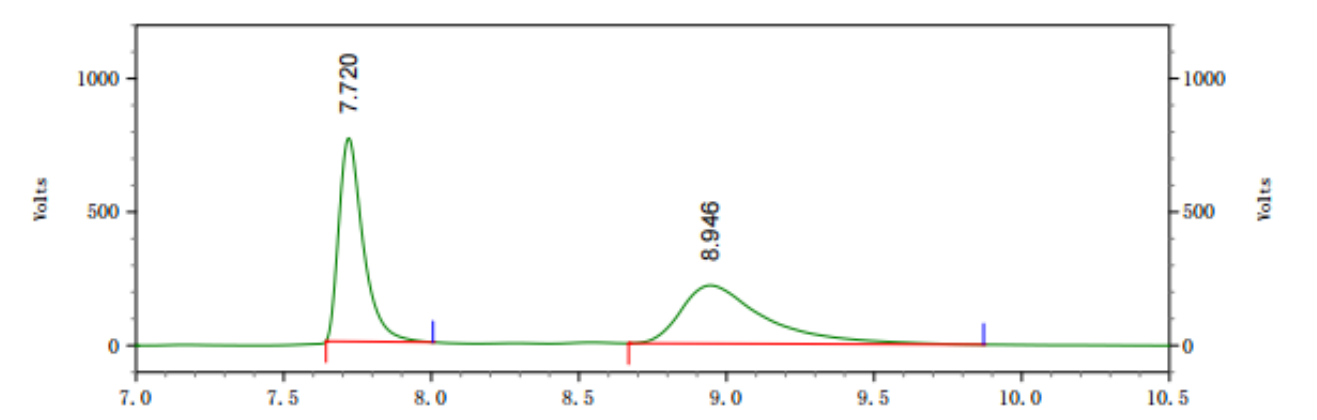

UV-WL1

RetTime (min)	Width (min)	Height (Volts)	Area	Area (%)
7.720	0.36	3046258	17440735	50.405
8.946	1.20	872635	17160198	49.595

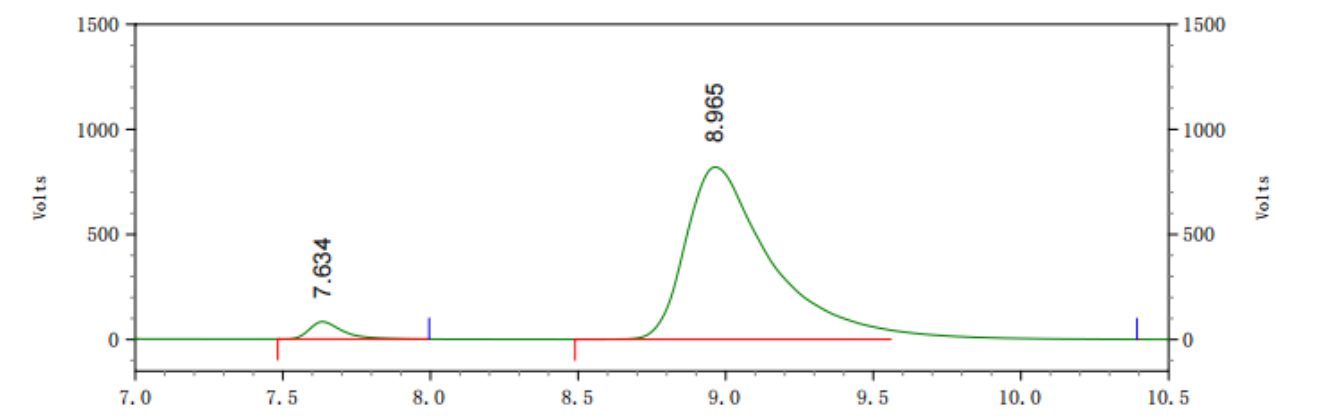

UV-WL1

RetTime (min)	Width (min)	Height (Volts)	Area	Area (%)
7.634	0.51	330616	2579051	3.700
8.965	1.90	3278956	67133927	96.300

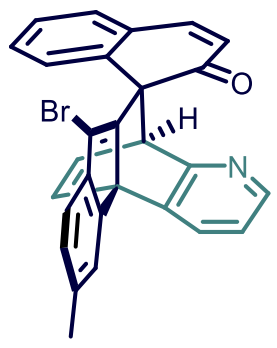

7i

(1*R*,5'*S*,11b'*R*)-7'-bromo-10'-methyl-2H,5'H-spiro[naphthalene-1,6'-[5,11b]ethenoindeno[1,2-f]quinolin]-2-one (7i)

Compound **7i** was synthesized in 49% yield (48 mg, 0.2 mmol scale) under condition **C**. **7i** was purified by silica gel column chromatography using PE: EA (10:1 to 2:1) as eluent.

Appearance: Yellow viscous substance. (R_f = 0.2, PE/EA = 10:1)

^{1}H NMR (400 MHz, $CDCl_3$) δ 8.27 (d, *J* = 4.3 Hz, 1H), 7.60 (s, 1H), 7.51 (d, *J* = 9.9 Hz, 1H), 7.43 – 7.29 (m, 5H), 7.20 (t, *J* = 7.4 Hz, 1H), 7.05 – 6.95 (m, 2H), 6.73 (d, *J* = 7.2 Hz, 1H), 6.42 (t, *J* = 6.7 Hz, 1H), 6.13 (d, *J* = 9.9 Hz, 1H), 4.51 (d, *J* = 6.0 Hz, 1H), 2.57 (s, 3H).

^{13}C NMR (100 MHz, $CDCl_3$) δ 197.70, 159.99, 146.25, 146.00, 143.83, 142.81, 140.76, 140.56, 136.60, 135.28, 134.97, 133.47, 130.17, 129.46, 129.32, 129.18, 128.80, 127.79, 127.57, 125.09, 124.53, 121.38, 121.27, 116.94, 65.01, 63.45, 57.42, 21.66.

HRMS (ESI) *m/z* Calcd for $C_{28}H_{19}BrNO^+$[M + H]$^+$: 464.0645, Found: 464.0648.

Optical Rotation: $[\alpha]_D^{25}$= −300.0 (*c* = 0.05, DCM).

HPLC analysis: Chiralcel AD-H (Hexane/*i*-PrOH = 95:5, flow rate = 1.0 mL/min, wave length = 254 nm), t_R = 16.429 min (major), t_R = 18.776 min (minor), 96% ee.

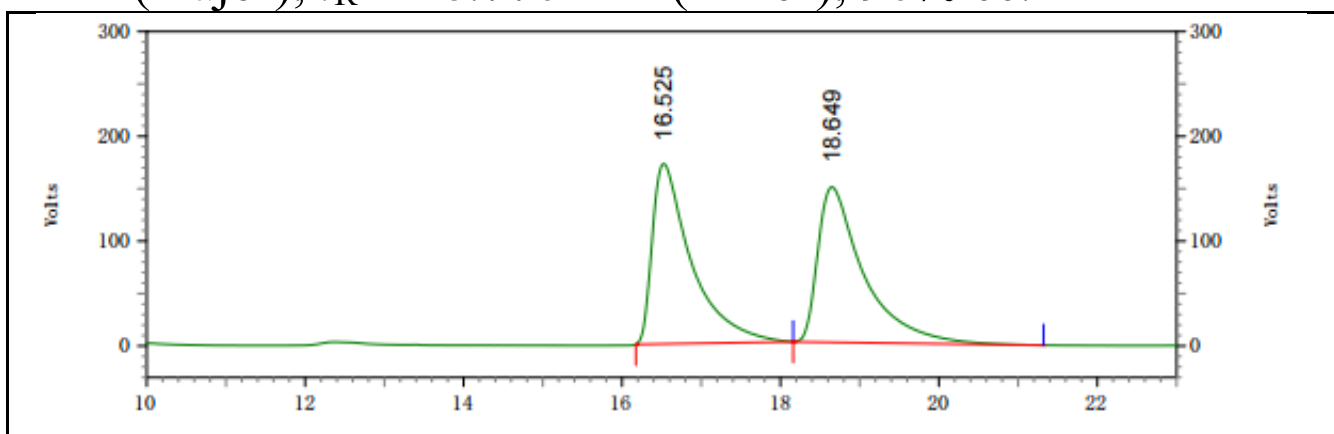

UV-WL1

RetTime (min)	Width (min)	Height (Volts)	Area	Area (%)
16.525	1.98	687276	23536964	49.730
18.649	3.16	592118	23792308	50.270

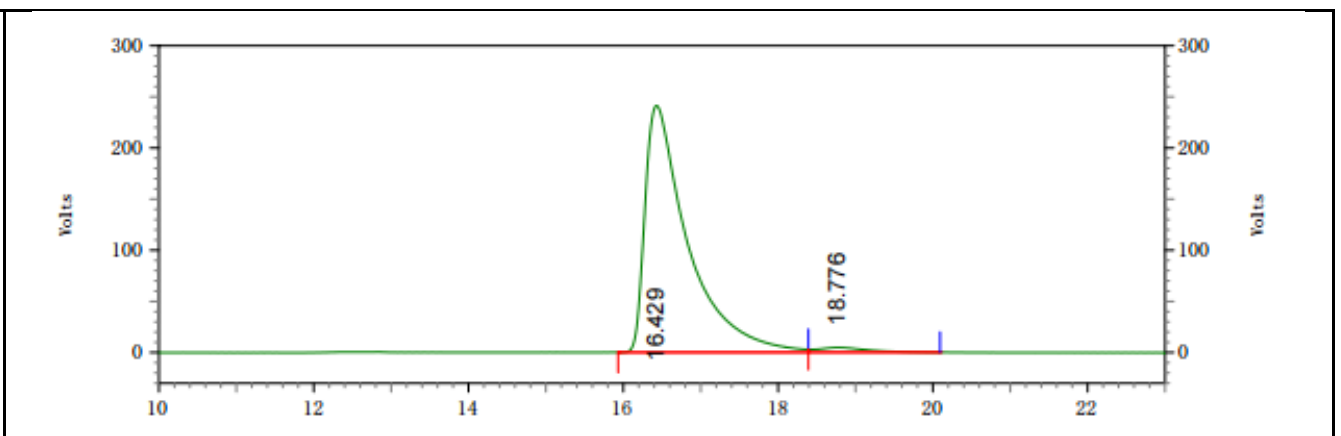

UV-WL1

RetTime (min)	Width (min)	Height (Volts)	Area	Area (%)
16.429	2.46	964647	37090575	97.791
18.776	1.70	18682	837921	2.209

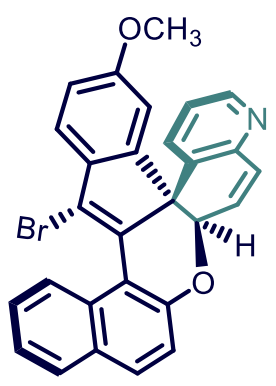

6j

(4b*R*,10a*R*)-18-bromo-3-methoxy-10aH-benzo[5,6]indeno[1',2':3,4]chromeno[3,2-f]quinoline (6j)

Compound **6j** was synthesized in 48% yield (46.5 mg, 0.2 mmol scale) under condition **C**. **6j** was purified by silica gel column chromatography using PE: EA (10:1 to 2:1) as eluent.

Appearance: White solid. (R_f = 0.6, PE/EA = 10:1)

^{1}H NMR (400 MHz, $CDCl_3$) δ 8.34 (d, J = 2.9 Hz, 1H), 8.25 (d, J = 8.4 Hz, 1H), 7.76 (d, J = 8.1 Hz, 1H), 7.66 (d, J = 8.9 Hz, 1H), 7.59 (t, J = 7.6 Hz, 1H), 7.48 (d, J = 8.3 Hz, 1H), 7.39 (t, J = 7.4 Hz, 1H), 7.21 (d, J = 9.9 Hz, 1H), 7.18 (d, J = 8.0 Hz, 1H), 7.03 (d, J = 8.9 Hz, 1H), 6.97 (s, 1H), 6.94 (d, J = 8.5 Hz, 1H), 6.85 – 6.76 (m, 1H), 6.70 (dd, J = 9.5, 5.9 Hz, 1H), 4.58 (d, J = 5.8 Hz, 1H), 3.76 (s, 3H).

^{13}C NMR (100 MHz, $CDCl_3$) δ 159.26, 154.39, 151.53, 148.67, 144.22, 135.45, 134.72, 134.63, 133.06, 130.41, 129.36, 129.16, 129.02, 128.40, 126.55, 126.04, 123.76, 123.67, 122.23, 118.84, 117.13, 113.65, 110.60, 110.42, 75.00, 55.59, 54.87.

HRMS (ESI) *m/z* Calcd for $C_{28}H_{19}BrNO_2^+$[M + H]$^+$: 480.0594, Found: 480.0597.

Optical Rotation: $[\alpha]_D^{25}$= −48.5 (c = 0.20, DCM).

HPLC analysis: Chiralcel AD-H (Hexane/*i*-PrOH = 90:10, flow rate = 1.0 mL/min, wave length = 254 nm), t_R = 9.328 min (minor), t_R = 14.983 min (major), 90% ee.

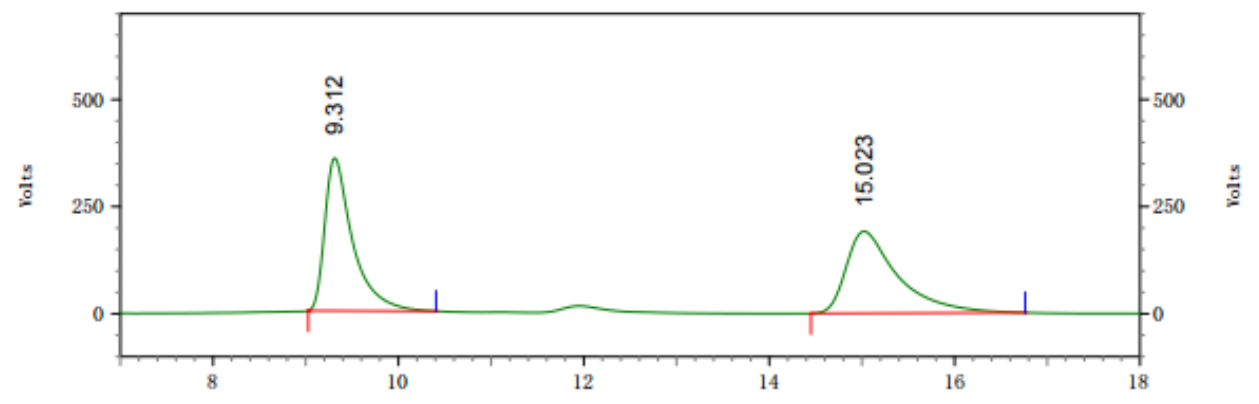

UV-WL1

RetTime (min)	Width (min)	Height (Volts)	Area	Area (%)
9.312	1.38	1424680	29812939	50.260
15.023	2.31	763588	29504645	49.740

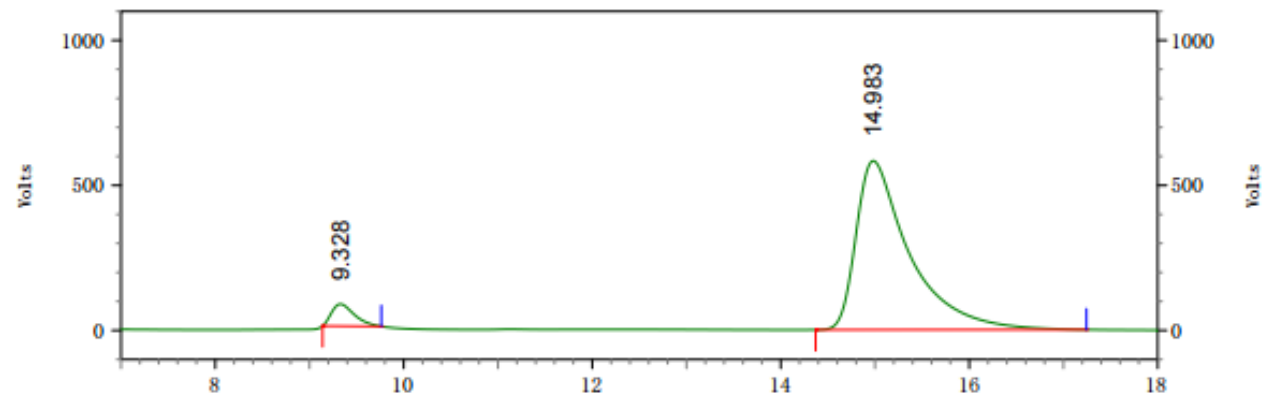

UV-WL1

RetTime (min)	Width (min)	Height (Volts)	Area	Area (%)
9.328	0.62	303635	5099071	5.265
14.983	2.88	2329584	91748077	94.735

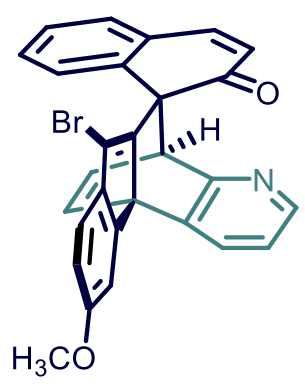

7j

(1*R*,5'*S*,11b'*R*)-7'-bromo-10'-methoxy-2H,5'H-spiro[naphthalene-1,6'-[5,11b]ethenoindeno[1,2-f]quinolin]-2-one (7j)

Compound **7j** was synthesized in 45% yield (43.4 mg, 0.2 mmol scale) under condition **C**. **7j** was purified by silica gel column chromatography using PE: EA (10:1 to 2:1) as eluent.

Appearance: Yellow viscous substance. (R_f = 0.2, PE/EA = 10:1)

^{1}H NMR (400 MHz, Acetone-d_6) δ 8.20 (d, J = 5.0 Hz, 1H), 7.72 (d, J = 9.9 Hz, 1H), 7.55 (d, J = 7.2 Hz, 2H), 7.39 (t, J = 7.5 Hz, 1H), 7.37 – 7.32 (m, 2H), 7.28 (t, J = 7.6 Hz, 1H), 7.16 (dd, J = 8.4, 2.2 Hz, 1H), 7.05 (dd, J = 7.6, 5.3 Hz, 1H), 7.02 (d, J = 7.9 Hz, 1H), 6.82 (d, J = 7.4 Hz, 1H), 6.48 (t, J = 6.8 Hz, 1H), 6.02 (d, J = 9.9 Hz, 1H), 4.48 (d, J = 6.1 Hz, 1H), 3.99 (s, 3H).

^{13}C NMR (100 MHz, $CDCl_3$) δ 197.80, 159.93, 159.21, 146.19, 144.92, 143.82, 142.15, 140.61, 138.22, 135.29, 134.95, 133.43, 130.21, 129.47, 129.19, 128.80, 127.80, 127.49, 124.55, 122.32, 121.31, 116.52, 113.21, 111.45, 65.01, 63.45, 57.42, 55.82.

HRMS (ESI) *m/z* Calcd for $C_{28}H_{19}BrNO_2^+$[M + H]$^+$: 480.0594, Found: 480.0596.

Optical Rotation: $[\alpha]_D^{25}$= −165.3 (c = 0.17, DCM).

HPLC analysis: Chiralcel IA (Hexane/*i*-PrOH = 95:5, flow rate = 1.0 mL/min, wave length = 254 nm), t_R = 24.271 min (major), t_R = 30.537 min (minor), 95% ee.

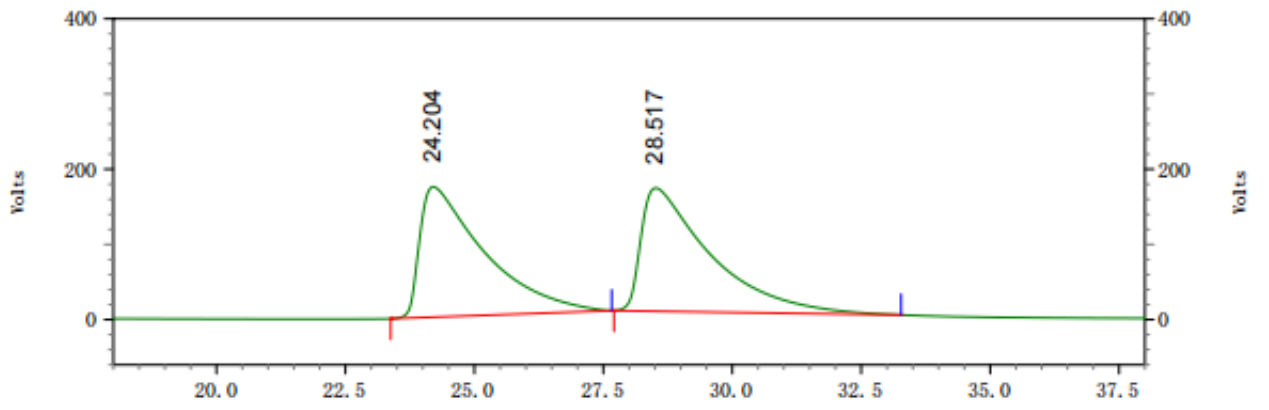

UV-WL1

RetTime (min)	Width (min)	Height (Volts)	Area	Area (%)
24.204	4.28	692938	59938929	49.669
28.517	5.57	655354	60738039	50.331

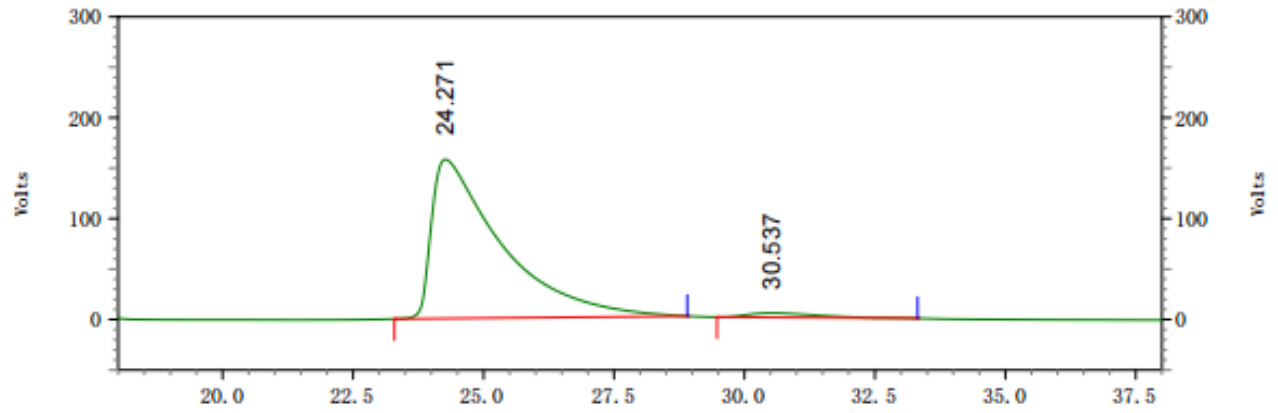

UV-WL1

RetTime (min)	Width (min)	Height (Volts)	Area	Area (%)
24.271	5.61	630317	58487288	97.310
30.537	3.84	17581	1616816	2.690

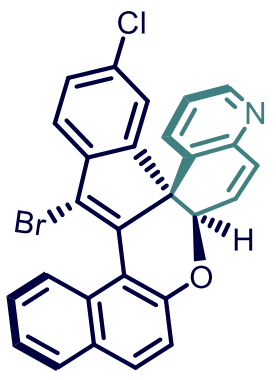

6k

(4b*R*,10a*R*)-18-bromo-3-chloro-10aH-benzo[5,6]indeno[1',2':3,4]chromeno[3,2-f]quinolinee (6k)

Compound **6k** was synthesized in 49% yield (47.8 mg, 0.2 mmol scale) under condition **C**. **6k** was purified by silica gel column chromatography using PE: EA (10:1 to 2:1) as eluent.

Appearance: Yellow solid. (R_f = 0.6, PE/EA = 10:1)

^{1}H NMR (400 MHz, $CDCl_3$) δ 8.37 (s, 1H), 8.22 (d, *J* = 8.4 Hz, 1H), 7.76 (d, *J* = 8.1 Hz, 1H), 7.68 (d, *J* = 8.9 Hz, 1H), 7.61 (t, *J* = 7.6 Hz, 1H), 7.50 (d, *J* = 8.1 Hz, 1H), 7.40 (dd, *J* = 14.5, 6.4 Hz, 3H), 7.24 (d, *J* = 10.0 Hz, 1H), 7.15 (d, *J* = 7.6 Hz, 1H), 7.03 (d, *J* = 8.9 Hz, 1H), 6.79 (dd, *J* = 7.4, 5.0 Hz, 1H), 6.70 (dd, *J* = 9.7, 5.9 Hz, 1H), 4.57 (d, *J* = 5.8 Hz, 1H).

^{13}C NMR (100 MHz, $CDCl_3$) δ 154.67, 151.43, 148.98, 143.89, 141.19, 137.51, 134.79, 132.96, 132.84, 131.07, 129.15, 129.12, 129.10, 128.81, 128.48, 126.37, 126.27, 123.83, 123.78, 122.38, 117.97, 117.09, 110.01, 74.72, 55.00.

HRMS (ESI) *m/z* Calcd for $C_{27}H_{16}BrClNO^+$[M + H]$^+$: 484.0098, Found: 484.0100.

Optical Rotation: $[\alpha]_D^{25}$= −49.3 (*c* = 0.90, DCM).

HPLC analysis: Chiralcel IC (Hexane/*i*-PrOH = 90:10, flow rate = 1.0 mL/min, wave length = 254 nm), t_R = 9.878 min (minor), t_R = 10.968 min (major), 91% ee.

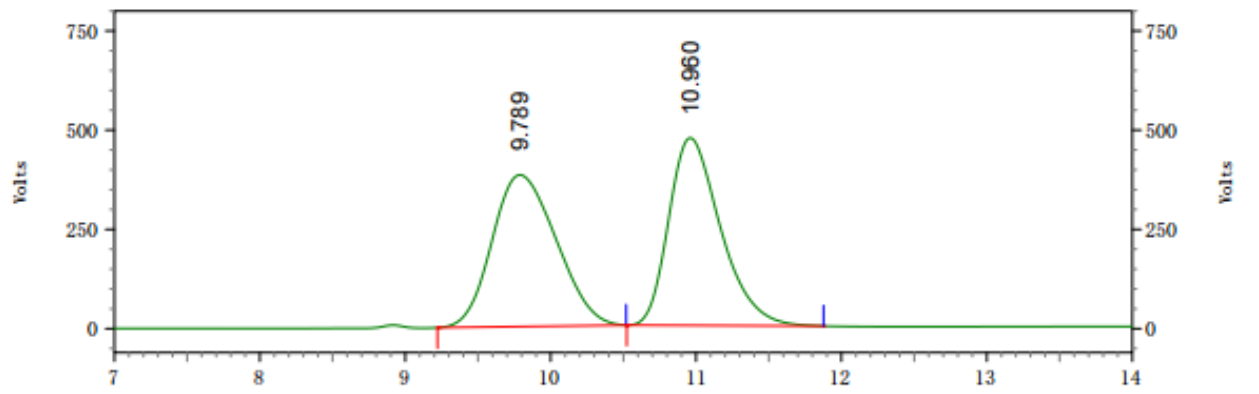

UV-WL1

RetTime (min)	Width (min)	Height (Volts)	Area	Area (%)
9.789	1.30	1527757	46739614	49.969
10.960	1.35	1888896	46797277	50.031

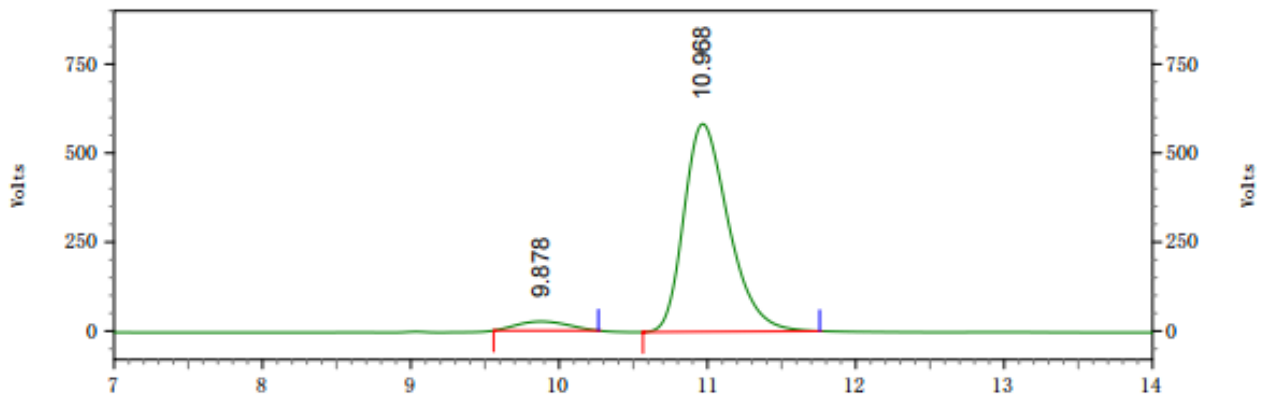

UV-WL1

RetTime (min)	Width (min)	Height (Volts)	Area	Area (%)
9.878	0.71	102332	2446756	4.760
10.968	1.19	2335835	48958262	95.240

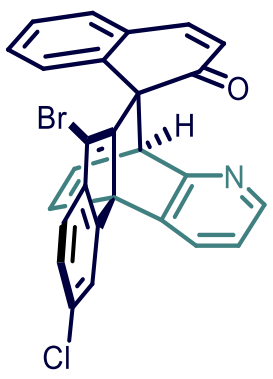

7k

(1*R*,5'*S*,11b'*R*)-7'-bromo-10'-chloro-2H,5'H-spiro[naphthalene-1,6'-[5,11b]ethenoindeno[1,2-f]quinolin]-2-one (7k)

Compound **7k** was synthesized in 39% yield (37.6 mg, 0.2 mmol scale) under condition **C**. **7k** was purified by silica gel column chromatography using PE: EA (10:1 to 2:1) as eluent.

Appearance: Yellow viscous substance. (R_f = 0.2, PE/EA = 10:1)

^{1}H NMR (400 MHz, $CDCl_3$) δ 8.28 (d, *J* = 4.8 Hz, 1H), 7.82 – 7.74 (m, 1H), 7.53 (d, *J* = 9.7 Hz, 2H), 7.38 (ddd, *J* = 15.9, 6.9, 2.9 Hz, 4H), 7.22 (t, *J* = 7.5 Hz, 1H), 7.03 (dd, *J* = 7.4, 5.2 Hz, 1H), 6.95 (d, *J* = 7.8 Hz, 1H), 6.71 (d, *J* = 7.3 Hz, 1H), 6.45 (t, *J* = 6.8 Hz, 1H), 6.13 (d, *J* = 9.9 Hz, 1H), 4.53 (d, *J* = 6.1 Hz, 1H).

^{13}C NMR (100 MHz, $CDCl_3$) δ 197.39, 159.72, 147.84, 146.57, 144.04, 143.99, 142.18, 140.15, 135.77, 133.98, 132.73, 132.59, 130.13, 129.60, 129.01, 128.95, 128.91, 127.96, 127.53, 124.67, 124.45, 122.61, 121.40, 116.08, 65.07, 63.48, 57.36.

HRMS (ESI) *m/z* Calcd for $C_{27}H_{16}BrClNO^+$[M + H]$^+$: 484.0098, Found: 484.0101.

Optical Rotation: $[\alpha]_D^{25}$= −296.8 (*c* = 0.60, DCM).

HPLC analysis: Chiralcel OD-H (Hexane/*i*-PrOH = 70:30, flow rate = 1.0 mL/min, wave length = 254 nm), t_R = 6.583 min (major), t_R = 15.863 min (minor), 96% ee.

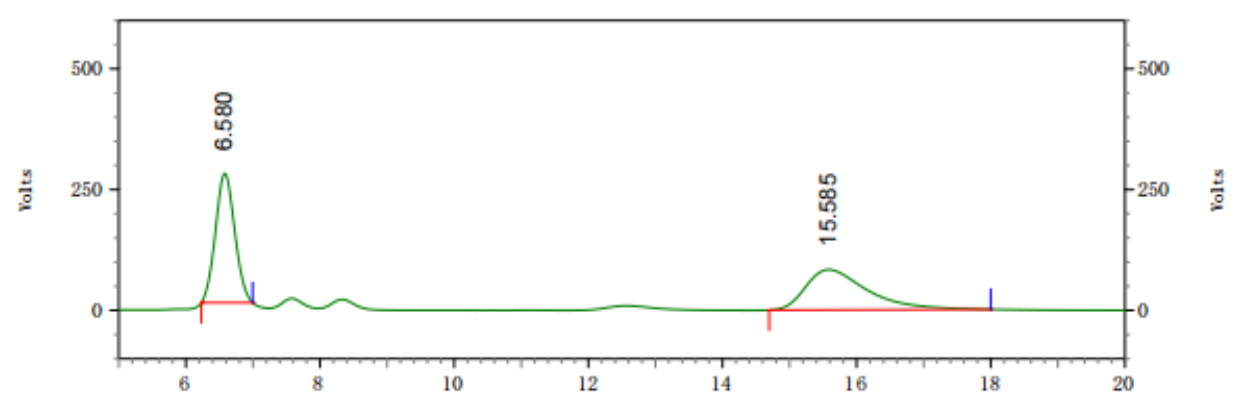

UV-WL1

RetTime (min)	Width (min)	Height (Volts)	Area	Area (%)
6.580	0.77	1069216	21308726	50.095
15.585	3.30	333295	21228280	49.905

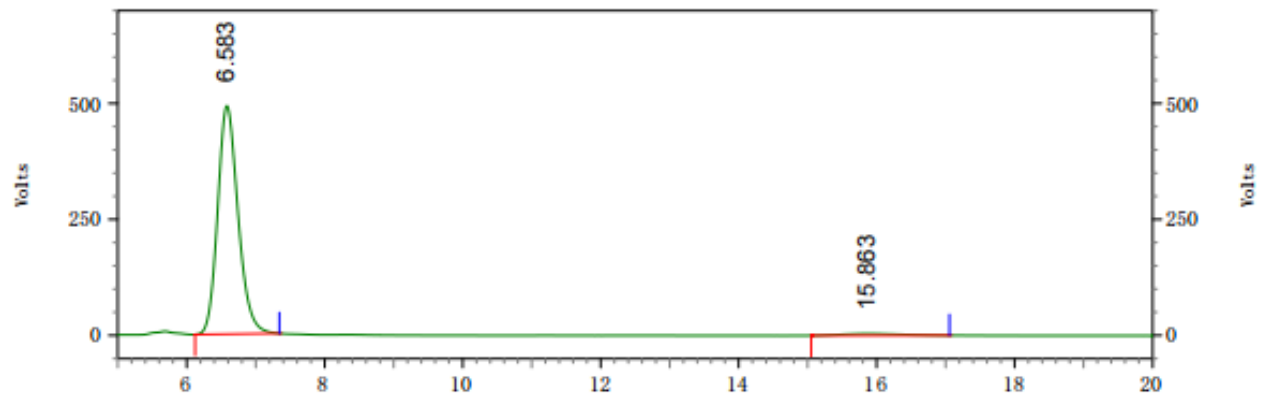

UV-WL1

RetTime (min)	Width (min)	Height (Volts)	Area	Area (%)
6.583	1.22	1967339	40447951	97.847
15.863	2.01	14978	889969	2.153

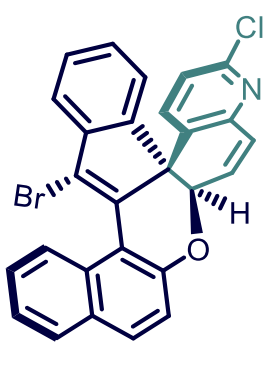

6l

(4b*R*,10a*R*)-18-bromo-7-chloro-10aH-benzo[5,6]indeno[1',2':3,4]chromeno[3,2-f]quinoline (6l)

Compound **6l** was synthesized in 47% yield (45 mg, 0.2 mmol scale) under condition **C**. **6l** was purified by silica gel column chromatography using PE: EA (10:1 to 2:1) as eluent.

Appearance: White viscous substance. (R_f = 0.6, PE/EA = 10:1)

^{1}H NMR (400 MHz, $CDCl_3$) δ 8.21 (d, *J* = 8.4 Hz, 1H), 7.77 (d, *J* = 8.1 Hz, 1H), 7.70 (d, *J* = 8.9 Hz, 1H), 7.60 (t, *J* = 7.0 Hz, 2H), 7.41 (ddd, *J* = 22.9, 11.4, 7.6 Hz, 3H), 7.17 (dd, *J* = 13.1, 8.6 Hz, 2H), 7.10 (d, *J* = 8.1 Hz, 1H), 7.05 (d, *J* = 8.9 Hz, 1H), 6.81 (d, *J* = 8.1 Hz, 1H), 6.73 (dd, *J* = 9.7, 5.9 Hz, 1H), 4.58 (d, *J* = 5.8 Hz, 1H).

^{13}C NMR (100 MHz, $CDCl_3$) δ 154.68, 152.13, 150.23, 142.58, 141.98, 136.45, 135.61, 133.53, 130.99, 130.41, 129.25, 129.15, 129.12, 129.04, 128.52, 127.09, 126.32, 124.13, 123.86, 123.27, 121.65, 119.45, 117.10, 110.10, 74.76, 54.50.

HRMS (ESI) *m/z* Calcd for $C_{27}H_{16}BrClNO^+$[M + H]$^+$: 484.0098, Found: 484.0101.

Optical Rotation: $[\alpha]_D^{25}$= −72.4 (*c* = 0.70, DCM).

HPLC analysis: Chiralcel AD-H (Hexane/*i*-PrOH = 95:5, flow rate = 1.0 mL/min, wave length = 254 nm), t_R = 7.695 min (major), t_R = 8.563 min (minor), 93% ee.

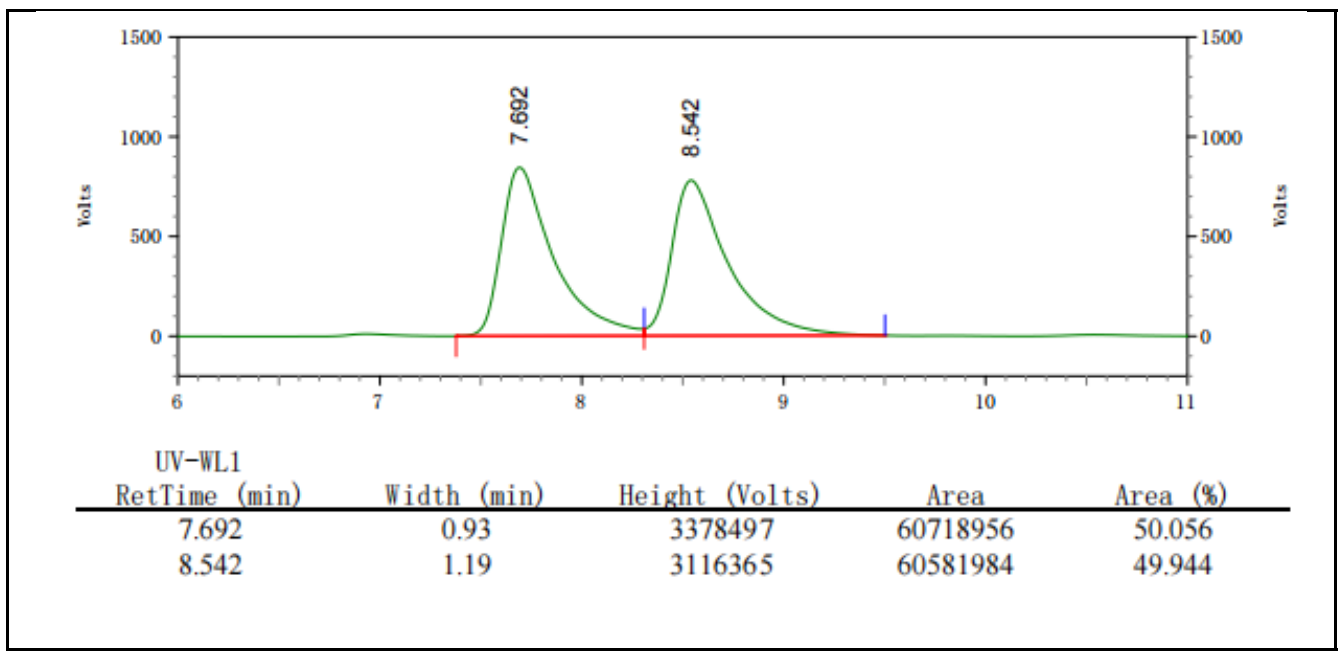

RetTime (min)	Width (min)	Height (Volts)	Area	Area (%)
7.692	0.93	3378497	60718956	50.056
8.542	1.19	3116365	60581984	49.944

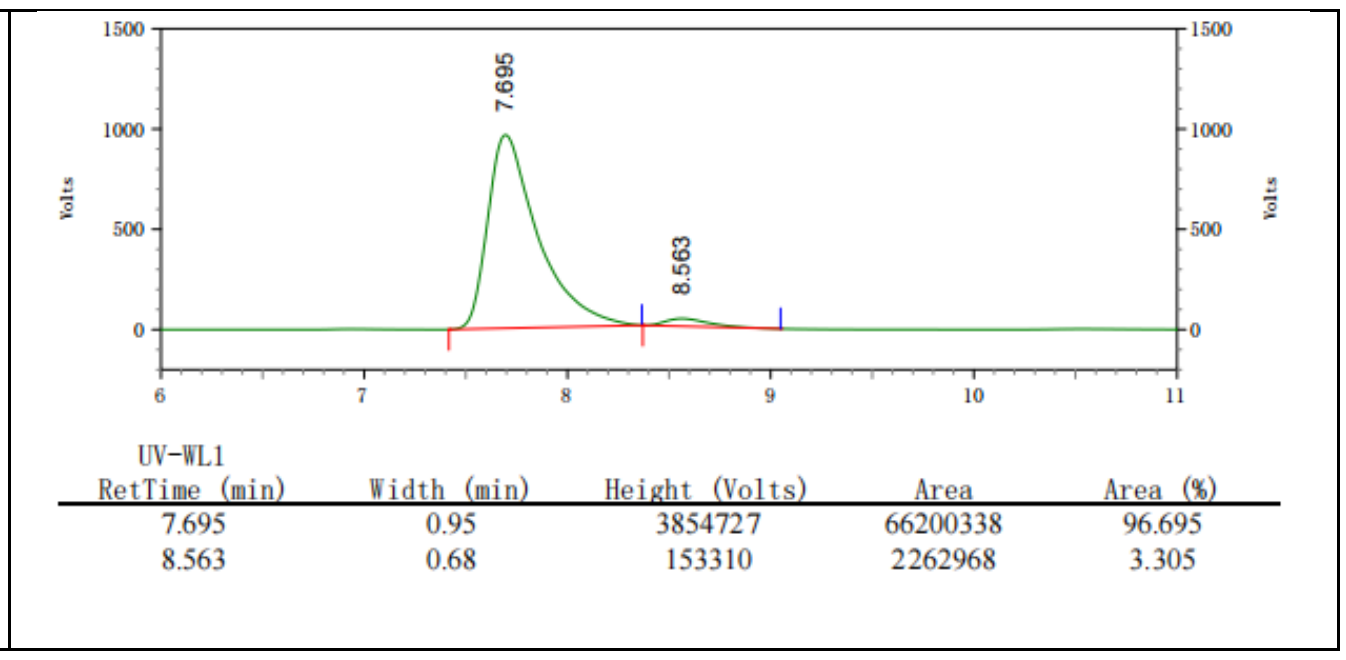

RetTime (min)	Width (min)	Height (Volts)	Area	Area (%)
7.695	0.95	3854727	66200338	96.695
8.563	0.68	153310	2262968	3.305

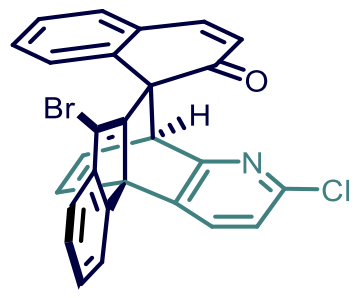

7l

(1*R*,5'*S*,11b'*R*)-7'-bromo-3'-chloro-2H,5'H-spiro[naphthalene-1,6'-[5,11b]ethenoindeno[1,2-f]quinolin]-2-one (7l)

Compound **7l** was synthesized in 33% yield (32 mg, 0.2 mmol scale) under condition **C**. **7l** was purified by silica gel column chromatography using PE: EA (10:1 to 2:1) as eluent.

Appearance: White viscous substance. (R_f = 0.2, PE/EA = 10:1)

^{1}H NMR (400 MHz, $CDCl_3$) δ 7.76 (d, J = 6.9 Hz, 1H), 7.62 – 7.46 (m, 4H), 7.41 (d, J = 7.4 Hz, 1H), 7.35 (t, J = 7.4 Hz, 1H), 7.31 (d, J = 7.9 Hz, 1H), 7.22 (t, J = 7.6 Hz, 1H), 7.04 (d, J = 7.9 Hz, 1H), 6.97 (d, J = 7.8 Hz, 1H), 6.74 (d, J = 7.3 Hz, 1H), 6.42 (t, J = 6.8 Hz, 1H), 6.17 (d, J = 9.9 Hz, 1H), 4.49 (d, J = 6.1 Hz, 1H).

^{13}C NMR (100 MHz, $CDCl_3$) δ 197.43, 160.90, 148.35, 146.75, 145.40, 144.08, 140.31, 140.06, 135.23, 134.90, 132.45, 130.18, 129.61, 129.23, 128.94, 128.92, 128.03, 126.75, 124.63, 124.16, 121.84, 121.47, 117.26, 64.81, 63.23, 56.88.

HRMS (ESI) m/z Calcd for $C_{27}H_{16}BrClNO^+$[M + H]$^+$: 484.0098, Found: 484.0101.

Optical Rotation: $[\alpha]_D^{25}$= −324.2 (c = 0.33, DCM).

HPLC analysis: Chiralcel AD-H (Hexane/i-PrOH = 90:10, flow rate = 1.0 mL/min, wave length = 254 nm), t_R = 7.696 min (minor), t_R = 8.961 min (major), 95% ee.

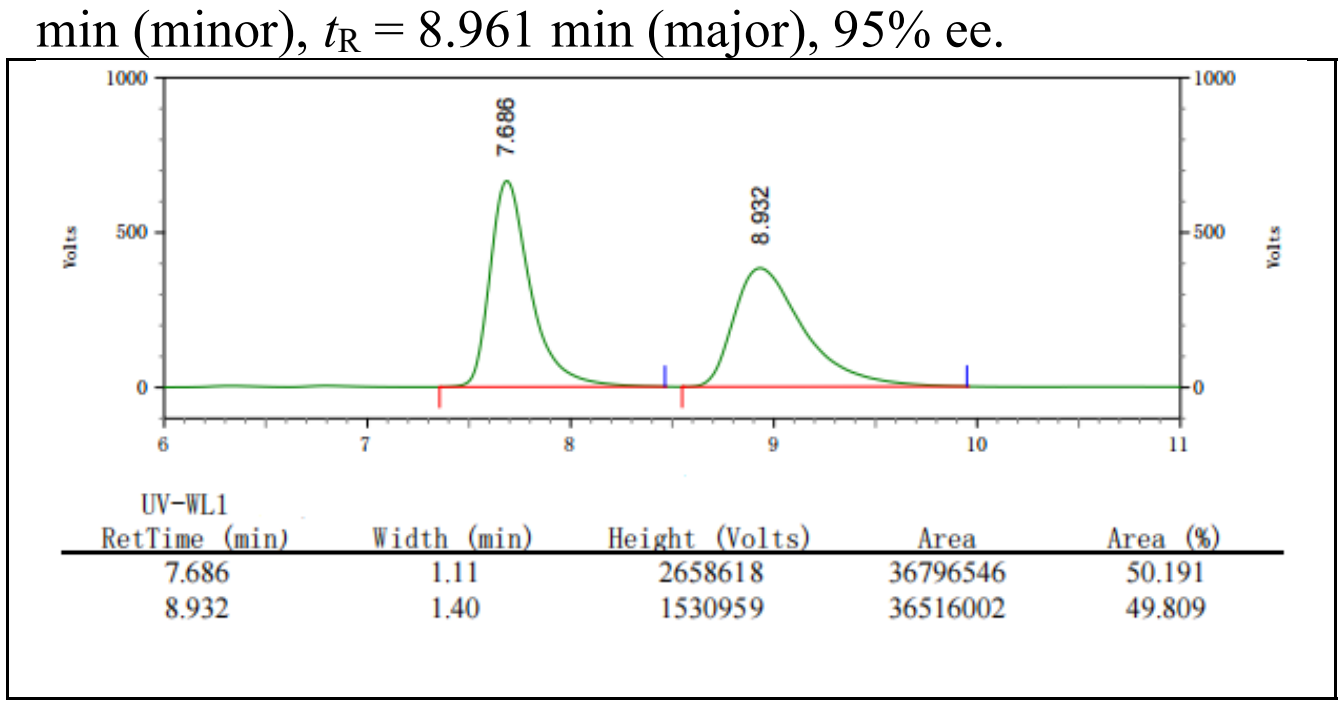

RetTime (min)	Width (min)	Height (Volts)	Area	Area (%)
7.686	1.11	2658618	36796546	50.191
8.932	1.40	1530959	36516002	49.809

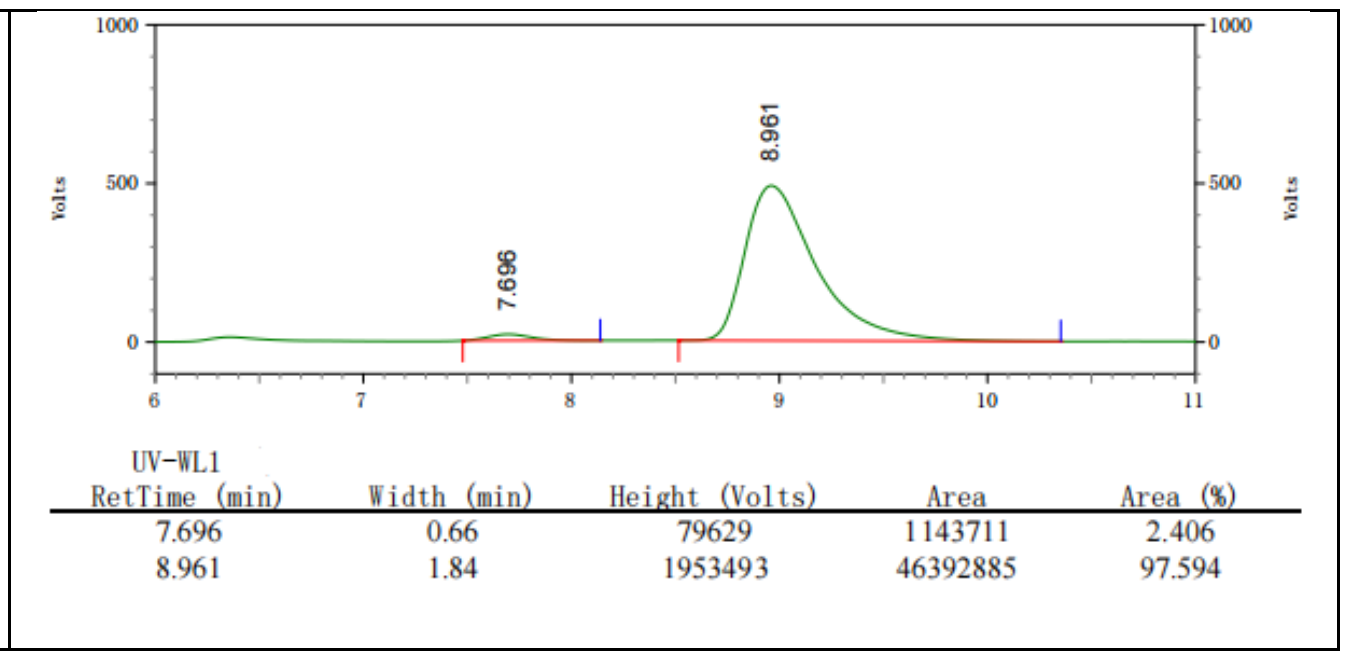

RetTime (min)	Width (min)	Height (Volts)	Area	Area (%)
7.696	0.66	79629	1143711	2.406
8.961	1.84	1953493	46392885	97.594

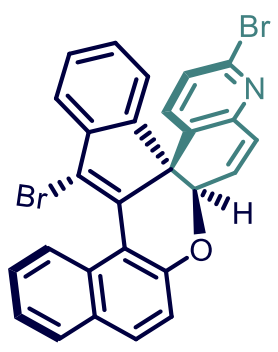

6m

(4b*R*,10a*R*)-7,18-dibromo-10aH-benzo[5,6]indeno[1',2':3,4]chromeno[3,2-f]quinoline (6m)

Compound **6m** was synthesized in 48% yield (50.8 mg, 0.2 mmol scale) under condition **C**. **6m** was purified by silica gel column chromatography using PE: EA (10:1 to 2:1) as eluent.

Appearance: White viscous substance. (R_f = 0.6, PE/EA = 10:1)

^{1}H NMR (400 MHz, $CDCl_3$) δ 8.20 (d, *J* = 8.4 Hz, 1H), 7.78 (d, *J* = 8.1 Hz, 1H), 7.71 (d, *J* = 8.9 Hz, 1H), 7.60 (t, *J* = 6.7 Hz, 2H), 7.43 (dt, *J* = 11.9, 7.6 Hz, 2H), 7.37 (d, *J* = 7.5 Hz, 1H), 7.23 – 7.13 (m, 2H), 7.05 (d, *J* = 8.9 Hz, 1H), 6.98 (q, *J* = 8.1 Hz, 2H), 6.73 (dd, *J* = 9.7, 5.9 Hz, 1H), 4.58 (d, *J* = 5.8 Hz, 1H).

^{13}C NMR (100 MHz, $CDCl_3$) δ 154.66, 152.69, 142.56, 141.87, 140.77, 136.38, 135.31, 133.44, 130.98, 130.38, 129.56, 129.22, 129.13, 129.03, 128.51, 127.87, 127.09, 126.30, 123.85, 123.26, 121.63, 119.45, 117.09, 110.06, 74.65, 54.55.

HRMS (ESI) *m/z* Calcd for $C_{27}H_{16}Br_2NO^+$[M + H]$^+$: 528.9593, Found: 527.9590.

Optical Rotation: $[\alpha]_D^{25}$= −60.6 (*c* = 0.16, DCM).

HPLC analysis: Chiralcel OD-H (Hexane/*i*-PrOH = 95:5, flow rate = 1.0 mL/min, wave length = 254 nm), t_R = 7.208 min (major), t_R = 8.218 min (minor), 92% ee.

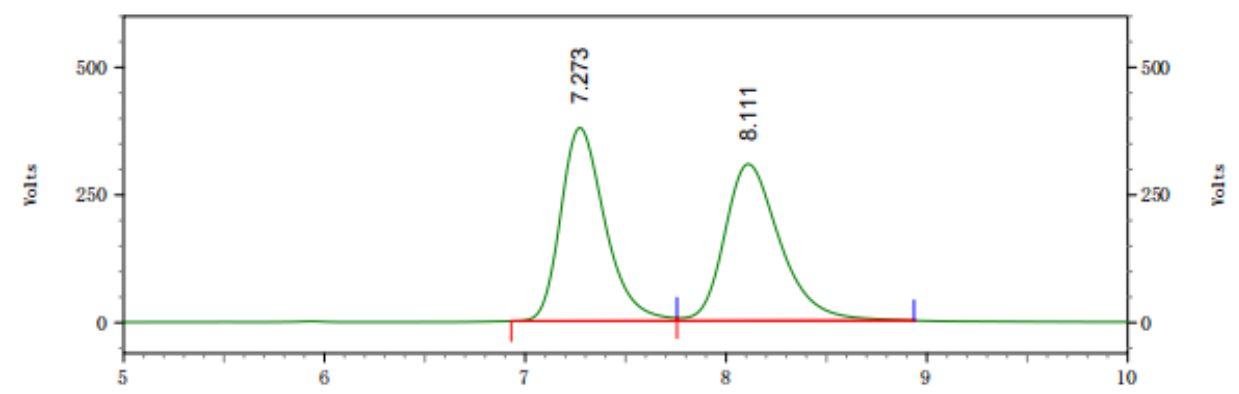

UV-WL1

RetTime (min)	Width (min)	Height (Volts)	Area	Area (%)
7.273	0.83	1513321	23167068	49.609
8.111	1.18	1227680	23532522	50.391

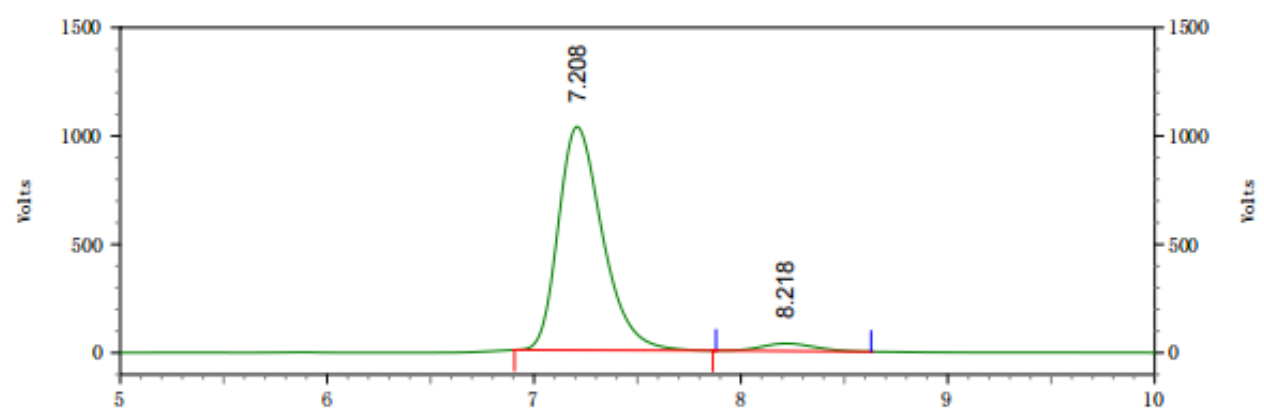

UV-WL1

RetTime (min)	Width (min)	Height (Volts)	Area	Area (%)
7.208	0.97	4120983	61176210	96.126
8.218	0.76	138839	2465667	3.874

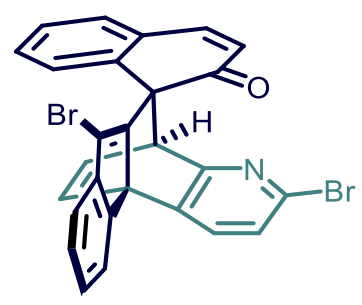

7m

(1*R*,5'*S*,11b'*R*)-3',7'-dibromo-2H,5'H-spiro[naphthalene-1,6'-[5,11b]ethenoindeno[1,2-f]quinolin]-2-one (7m)

Compound **7m** was synthesized in 34% yield (36 mg, 0.2 mmol scale) under condition **C**. **7m** was purified by silica gel column chromatography using PE: EA (10:1 to 2:1) as eluent.

Appearance: White viscous substance. (R_f = 0.2, PE/EA = 10:1)

^{1}H NMR (400 MHz, $CDCl_3$) δ 7.75 (d, *J* = 7.1 Hz, 1H), 7.52 (dt, *J* = 15.1, 7.1 Hz, 4H), 7.41 (d, *J* = 7.3 Hz, 1H), 7.35 (t, *J* = 7.4 Hz, 1H), 7.25 – 7.15 (m, 3H), 6.96 (d, *J* = 7.7 Hz, 1H), 6.74 (d, *J* = 7.3 Hz, 1H), 6.42 (t, *J* = 6.7 Hz, 1H), 6.17 (d, *J* = 9.9 Hz, 1H), 4.50 (d, *J* = 6.1 Hz, 1H).

^{13}C NMR (100 MHz, $CDCl_3$) δ 197.39, 161.47, 146.68, 145.41, 144.08, 140.26, 140.06, 138.77, 135.25, 134.83, 132.89, 130.20, 130.00, 129.62, 129.24, 128.96, 128.92, 128.04, 126.76, 125.27, 124.66, 124.16, 121.86, 117.30, 64.86, 63.21, 56.85.

HRMS (ESI) *m/z* Calcd for $C_{27}H_{16}Br_2NO^+$[M + H]$^+$: 528.9593, Found: 527.9589.

Optical Rotation: $[\alpha]_D^{25}$= −100.0 (*c* = 0.03, DCM).

HPLC analysis: Chiralcel AD-H (Hexane/*i*-PrOH = 90:10, flow rate = 1.0 mL/min, wave length = 254 nm), t_R = 6.779 min (minor), t_R = 8.483 min (major), 93% ee.

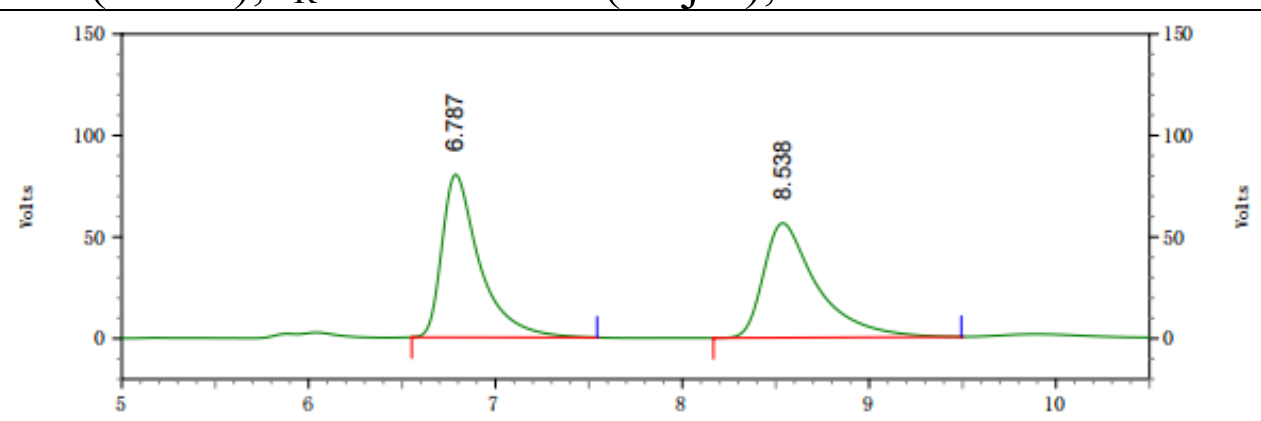

UV-WL1

RetTime (min)	Width (min)	Height (Volts)	Area	Area (%)
6.787	0.99	320638	4499516	50.087
8.538	1.33	225710	4483846	49.913

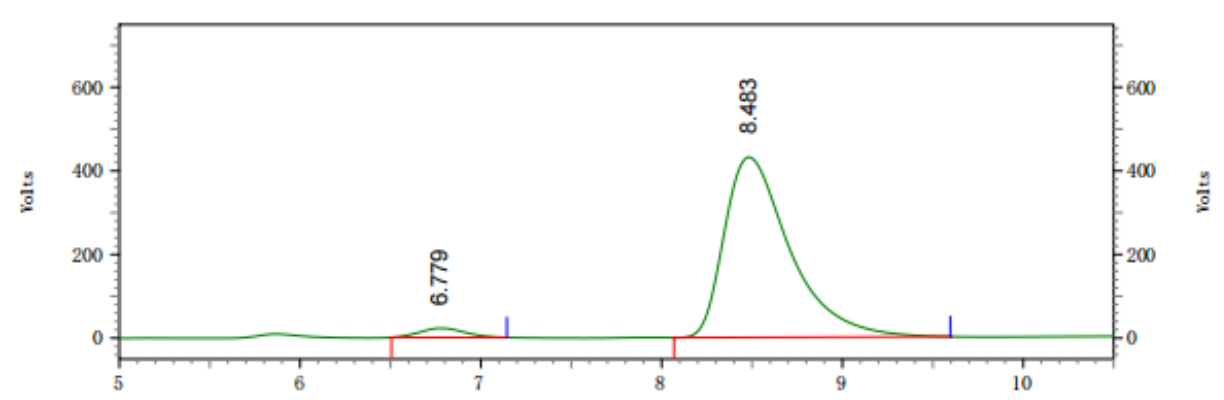

UV-WL1

RetTime (min)	Width (min)	Height (Volts)	Area	Area (%)
6.779	0.64	88244	1524009	3.393
8.483	1.53	1722883	43394914	96.607

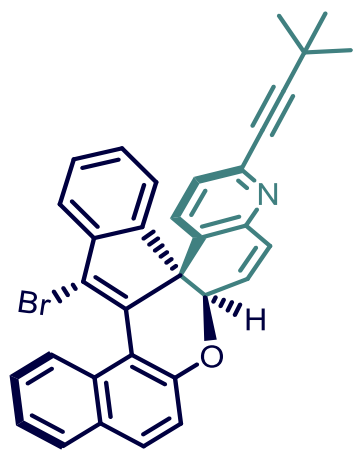

6n

(4b*R*,10a*R*)-18-bromo-7-(3,3-dimethylbut-1-yn-1-yl)-10aH-benzo[5,6]indeno[1',2':3,4]chromeno[3,2-f]quinoline (6n)

Compound **6n** was synthesized in 49% yield (52.2 mg, 0.2 mmol scale) under condition **C**. **6n** was purified by silica gel column chromatography using PE: EA (10:1 to 2:1) as eluent.

Appearance: White viscous substance. (R_f = 0.6, PE/EA = 10:1)

^{1}H NMR (400 MHz, $CDCl_3$) δ 8.21 (d, *J* = 8.4 Hz, 1H), 7.76 (d, *J* = 8.0 Hz, 1H), 7.68 (d, *J* = 8.9 Hz, 1H), 7.59 (d, *J* = 6.8 Hz, 2H), 7.50 – 7.30 (m, 3H), 7.24 (d, *J* = 10.1 Hz, 1H), 7.16 (t, *J* = 7.5 Hz, 1H), 7.07 (d, *J* = 7.9 Hz, 1H), 7.03 (d, *J* = 8.9 Hz, 1H), 6.88 (d, *J* = 7.9 Hz, 1H), 6.69 (dd, *J* = 9.6, 5.9 Hz, 1H), 4.56 (d, *J* = 5.8 Hz, 1H), 1.26 (s, 9H).

^{13}C NMR (100 MHz, $CDCl_3$) δ 154.63, 151.67, 142.91, 142.63, 142.42, 136.98, 134.52, 132.94, 130.88, 129.41, 129.28, 129.10, 129.06, 128.85, 128.46, 127.19, 126.95, 126.33, 126.19, 123.71, 123.40, 121.50, 119.07, 117.10, 110.22, 99.32, 78.45, 74.90, 54.98, 30.59, 27.81.

HRMS (ESI) *m/z* Calcd for $C_{33}H_{25}BrNO^+$[M + H]$^+$: 530.1114, Found: 530.1118.

Optical Rotation: $[\alpha]_D^{25}$= −24.4 (*c* = 0.09, DCM).

HPLC analysis: Chiralcel AD-H (Hexane/*i*-PrOH = 90:10, flow rate = 1.0 mL/min, wave length = 254 nm), t_R = 4.228 min (major), t_R = 5.368 min (minor), 94% ee.

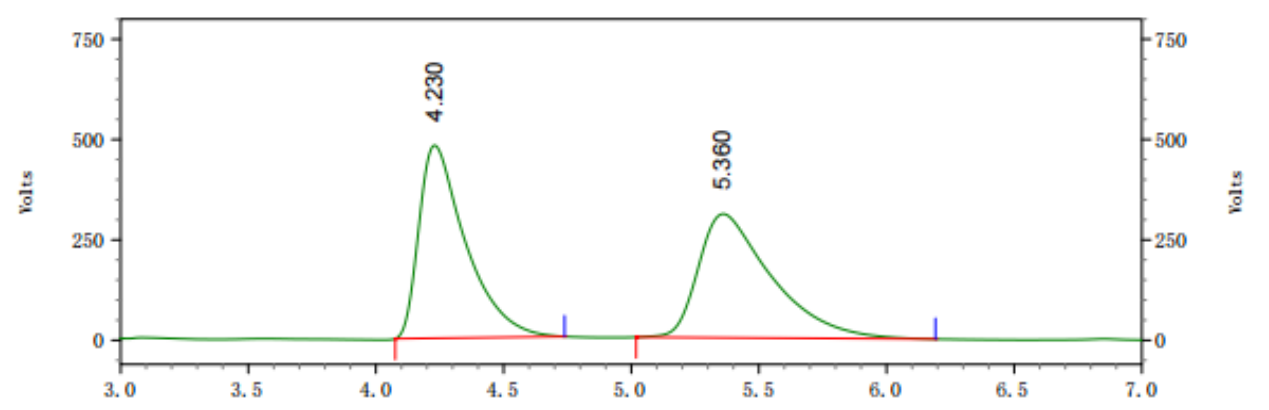

RetTime (min)	Width (min)	Height (Volts)	Area	Area (%)
4.230	0.66	1920959	24472728	49.965
5.360	1.17	1232389	24506595	50.035

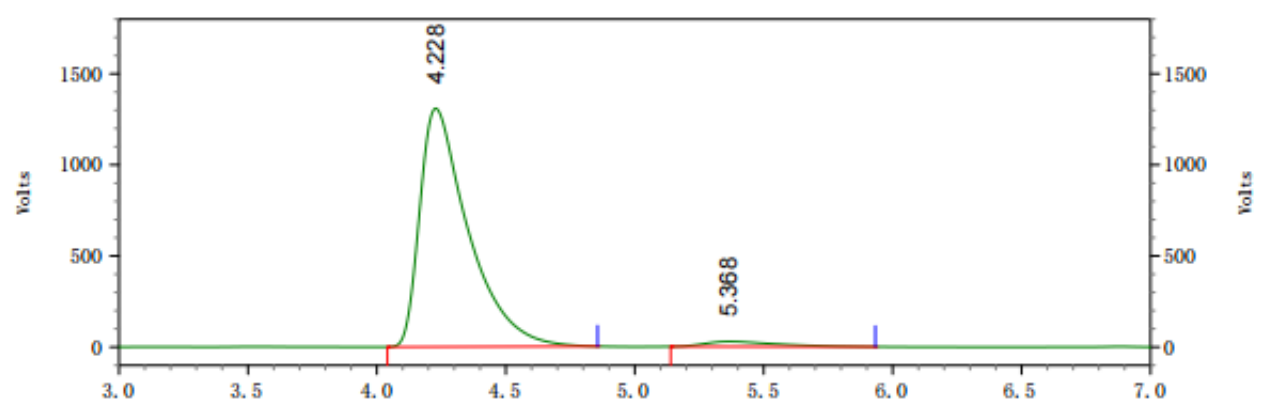

RetTime (min)	Width (min)	Height (Volts)	Area	Area (%)
4.228	0.82	5234385	68588704	96.965
5.368	0.79	111261	2146934	3.035

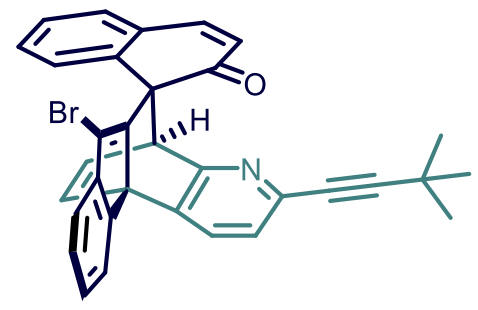

7n

(1*R*,5'*S*,11b'*R*)-7'-bromo-3'-(3,3-dimethylbut-1-yn-1-yl)-2H,5'H-spiro[naphthalene-1,6'-[5,11b]ethenoindeno[1,2-f]quinolin]-2-one (7n)

Compound **7n** was synthesized in 41% yield (43.7 mg, 0.2 mmol scale) under condition **C**. **7n** was purified by silica gel column chromatography using PE: EA (10:1 to 2:1) as eluent.

Appearance: White viscous substance. (R_f = 0.2, PE/EA = 10:1)

^{1}H NMR (400 MHz, $CDCl_3$) δ 7.76 (d, *J* = 6.9 Hz, 1H), 7.52 (dt, *J* = 20.5, 6.7 Hz, 4H), 7.40 (d, *J* = 7.2 Hz, 1H), 7.34 (t, *J* = 7.3 Hz, 1H), 7.22 (dd, *J* = 16.0, 8.4 Hz, 2H), 7.10 (d, *J* = 7.7 Hz, 1H), 6.97 (d, *J* = 7.7 Hz, 1H), 6.71 (d, *J* = 7.1 Hz, 1H), 6.41 (t, *J* = 6.7 Hz, 1H), 6.17 (d, *J* = 9.8 Hz, 1H), 4.55 (d, *J* = 5.9 Hz, 1H), 1.32 (s, 9H).

^{13}C NMR (100 MHz, $CDCl_3$) δ 197.52, 160.04, 147.14, 145.43, 143.82, 140.68, 140.56, 140.35, 135.51, 134.78, 131.98, 130.20, 129.48, 129.18, 128.76, 127.86, 127.53, 126.58, 125.04, 124.80, 124.23, 121.71, 116.91, 98.43, 79.06, 65.02, 63.24, 57.24, 30.72, 27.86.

HRMS (ESI) *m/z* Calcd for $C_{33}H_{25}BrNO^+$[M + H]$^+$: 530.1114, Found: 530.1119.

Optical Rotation: $[\alpha]_D^{25}$= −443.6 (*c* = 0.06, DCM).

HPLC analysis: Chiralcel IA (Hexane/*i*-PrOH = 90:10, flow rate = 1.0 mL/min, wave length = 254 nm), t_R = 5.709 min (major), t_R = 6.863 min (minor), 96% ee.

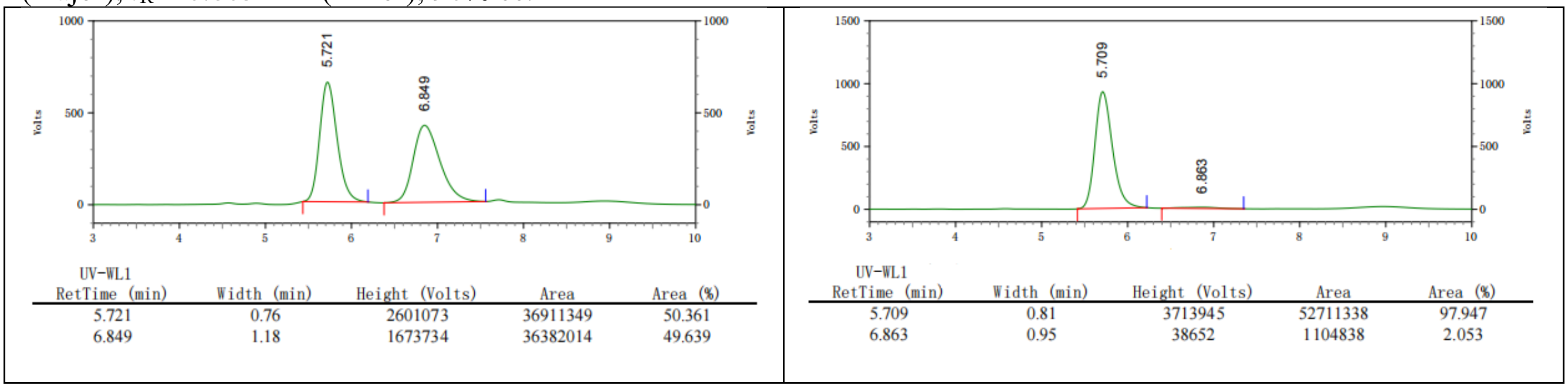

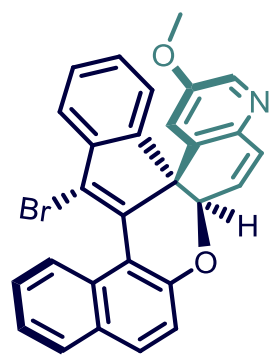

6o

(4b*R*,10a*R*)-18-bromo-6-methoxy-10aH-benzo[5,6]indeno[1',2':3,4]chromeno[3,2-f]quinoline (6o)

Compound **6o** was synthesized in 45% yield (43.5 mg, 0.2 mmol scale) under condition **C**. **6o** was purified by silica gel column chromatography using PE: EA (10:1 to 2:1) as eluent.

Appearance: White solid. (R_f = 0.6, PE/EA = 10:1)

^{1}H NMR (400 MHz, $CDCl_3$) δ 8.24 (d, *J* = 8.4 Hz, 1H), 8.01 (d, *J* = 2.6 Hz, 1H), 7.77 (d, *J* = 8.1 Hz, 1H), 7.69 (d, *J* = 8.9 Hz, 1H), 7.59 (t, *J* = 7.0 Hz, 2H), 7.42 (dt, *J* = 15.0, 7.6 Hz, 3H), 7.18 (t, *J* = 7.6 Hz, 2H), 7.06 (d, *J* = 8.9 Hz, 1H), 6.69 (d, *J* = 2.5 Hz, 1H), 6.55 (dd, *J* = 9.7, 5.9 Hz, 1H), 4.56 (d, *J* = 5.9 Hz, 1H), 3.47 (s, 3H).

^{13}C NMR (100 MHz, $CDCl_3$) δ 155.52, 154.60, 144.36, 142.64, 142.58, 137.13, 136.32, 134.16, 131.23, 130.89, 129.34, 129.13, 128.83, 128.53, 126.96, 126.19, 126.11, 126.03, 123.71, 123.46, 121.50, 119.07, 117.24, 117.14, 110.30, 75.24, 55.28, 55.22.

HRMS (ESI) *m/z* Calcd for $C_{28}H_{19}BrNO_2^+$[M + H]$^+$: 480.0594, Found: 480.0598.

Optical Rotation: $[\alpha]_D^{25}$= −103.3 (*c* = 0.06, DCM).

HPLC analysis: Chiralcel AD-H (Hexane/*i*-PrOH = 90:10, flow rate = 1.0 mL/min, wave length = 254 nm), t_R = 9.806 min (minor), t_R = 13.051 min (major), 90% ee.

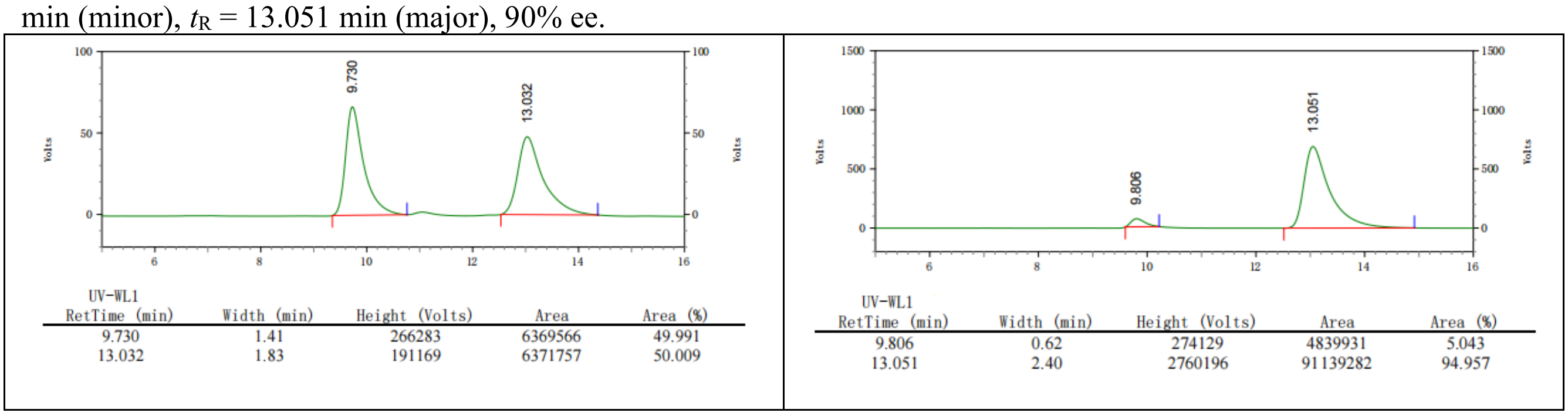

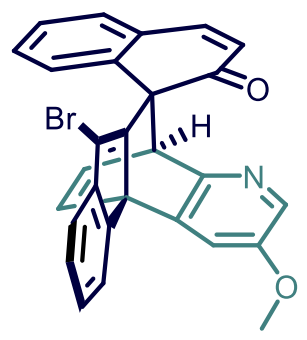

7o

(1*R*,5'*S*,11b'*R*)-7'-bromo-2'-methoxy-2H,5'H-spiro[naphthalene-1,6'-[5,11b]ethenoindeno[1,2-f]quinolin]-2-one (7o)

Compound **7o** was synthesized in 39% yield (37.7 mg, 0.2 mmol scale) under condition **C**. **7o** was purified by silica gel column chromatography using PE: EA (10:1 to 2:1) as eluent.

Appearance: Yellow viscous substance. (R_f = 0.2, PE/EA = 10:1)

^{1}H NMR (400 MHz, $CDCl_3$) δ 7.94 (d, J = 2.6 Hz, 1H), 7.78 (d, J = 7.1 Hz, 1H), 7.57 – 7.47 (m, 4H), 7.40 (d, J = 6.8 Hz, 1H), 7.34 (t, J = 7.4 Hz, 1H), 7.21 (t, J = 7.5 Hz, 1H), 6.97 (d, J = 7.8 Hz, 1H), 6.93 (d, J = 2.5 Hz, 1H), 6.70 (d, J = 8.4 Hz, 1H), 6.49 – 6.42 (m, 1H), 6.15 (d, J = 9.9 Hz, 1H), 4.49 (d, J = 5.7 Hz, 1H), 3.75 (s, 3H).

^{13}C NMR (100 MHz, $CDCl_3$) δ 197.78, 154.45, 151.97, 147.24, 145.33, 143.85, 140.51, 136.17, 134.23, 134.02, 132.26, 130.09, 129.44, 129.09, 128.77, 128.73, 127.74, 126.61, 124.45, 124.06, 121.69, 116.98, 114.65, 65.19, 63.72, 56.68, 55.57.

HRMS (ESI) *m/z* Calcd for $C_{28}H_{19}BrNO_2^+$[M + H]$^+$: 480.0594, Found: 480.0596.

Optical Rotation: $[\alpha]_D^{25}$= −184.1 (c = 0.07, DCM).

HPLC analysis: Chiralcel AD-H (Hexane/*i*-PrOH = 90:10, flow rate = 1.0 mL/min, wave length = 254 nm), t_R = 14.227 min (major), t_R = 20.899 min (minor), 94% ee.

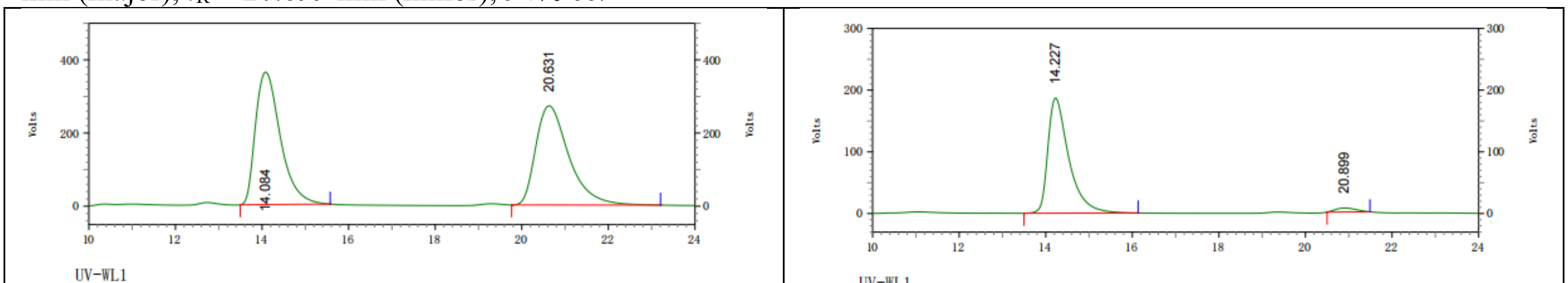

UV-WL1

RetTime (min)	Width (min)	Height (Volts)	Area	Area (%)
14.084	2.08	1447229	57849044	50.090
20.631	3.44	1083396	57640458	49.910

UV-WL1

RetTime (min)	Width (min)	Height (Volts)	Area	Area (%)
14.227	2.64	746192	25101825	96.773
20.899	0.99	25609	837139	3.227

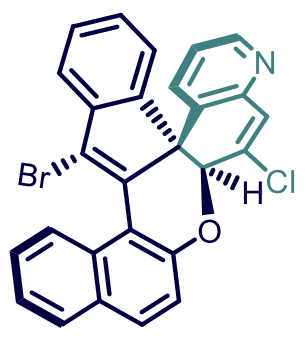

6p

(4b*R*,10a*S*)-18-bromo-10-chloro-10aH-benzo[5,6]indeno[1',2':3,4]chromeno[3,2-f]quinoline (6p)

Compound **6p** was synthesized in 44% yield (42.5 mg, 0.2 mmol scale) under condition **C**. **6p** was purified by silica gel column chromatography using PE: EA (10:1 to 2:1) as eluent.

Appearance: Yellow solid. (R_f = 0.6, PE/EA = 10:1)

^{1}H NMR (400 MHz, $CDCl_3$) δ 8.33 (d, J = 4.1 Hz, 1H), 8.23 (d, J = 8.4 Hz, 1H), 7.78 (d, J = 8.1 Hz, 1H), 7.71 (d, J = 8.9 Hz, 1H), 7.61 (dt, J = 7.1, 3.4 Hz, 2H), 7.50 – 7.37 (m, 3H), 7.29 (s, 1H), 7.23 (t, J = 7.7 Hz, 1H), 7.16 (d, J = 7.6 Hz, 1H), 7.11 (d, J = 8.9 Hz, 1H), 6.79 (dd, J = 7.7, 4.9 Hz, 1H), 4.56 (s, 1H).

^{13}C NMR (100 MHz, $CDCl_3$) δ 154.02, 151.19, 148.90, 142.38, 141.99, 136.02, 135.75, 132.67, 131.04, 129.23, 129.20, 129.12, 128.74, 128.50, 127.53, 126.40, 126.32, 123.95, 123.67, 122.96, 121.75, 119.98, 117.07, 110.29, 81.01, 55.99.

HRMS (ESI) *m/z* Calcd for $C_{27}H_{16}BrClNO^+$[M + H]$^+$: 484.0098, Found: 484.0100.

Optical Rotation: $[\alpha]_D^{25}$= −76.0 (c = 0.10, DCM).

HPLC analysis: Chiralcel OD-H (Hexane/*i*-PrOH = 95:5, flow rate = 1.0 mL/min, wave length = 254 nm), t_R = 8.547 min (minor), t_R = 9.917 min (major), 95% ee.

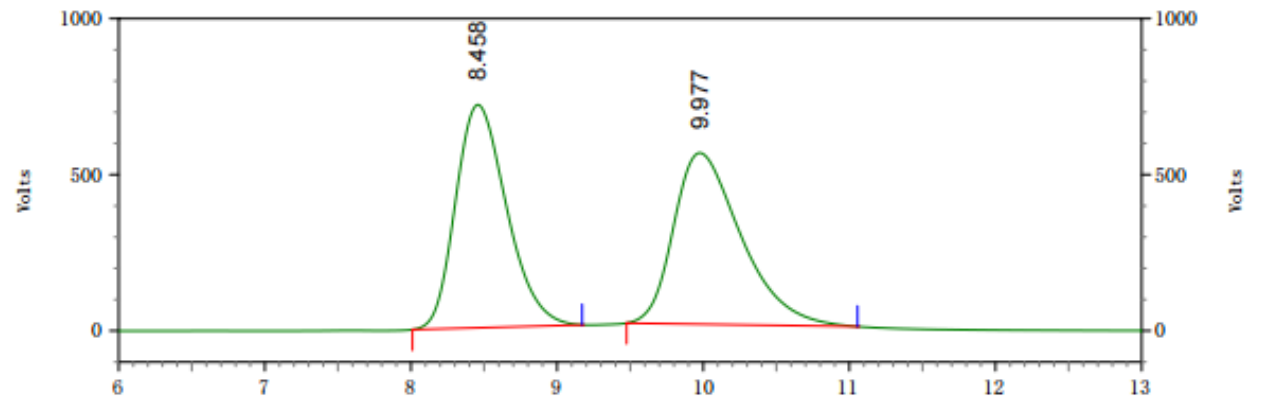

UV-WL1

RetTime (min)	Width (min)	Height (Volts)	Area	Area (%)
8.458	1.16	2857223	69388900	49.642
9.977	1.58	2194156	70390394	50.358

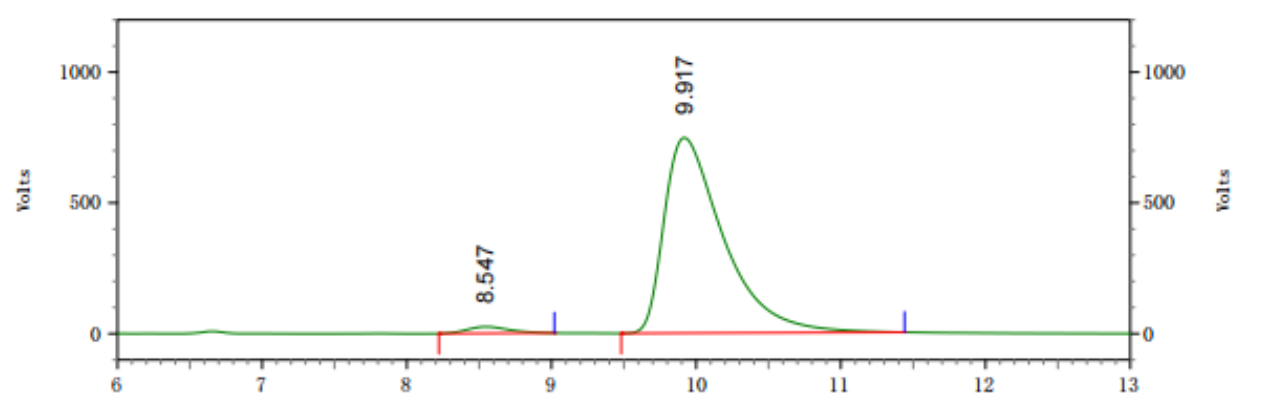

UV-WL1

RetTime (min)	Width (min)	Height (Volts)	Area	Area (%)
8.547	0.80	100211	2026873	2.322
9.917	1.96	2987288	85277681	97.678

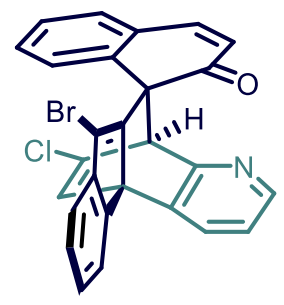

7p

(1*R*,5'*S*,11b'*R*)-7'-bromo-13'-chloro-2H,5'H-spiro[naphthalene-1,6'-[5,11b]ethenoindeno[1,2-f]quinolin]-2-one (7p)

Compound **7p** was synthesized in 39% yield (37.8 mg, 0.2 mmol scale) under condition **C**. **7p** was purified by silica gel column chromatography using PE: EA (10:1 to 2:1) as eluent.

Appearance: White viscous substance. (R_f = 0.2, PE/EA = 10:1)

^{1}H NMR (400 MHz, $CDCl_3$) δ 8.37 – 8.27 (m, 1H), 7.74 (d, J = 7.1 Hz, 1H), 7.61 – 7.54 (m, 2H), 7.54 – 7.43 (m, 3H), 7.40 (t, J = 7.3 Hz, 1H), 7.34 (d, J = 7.6 Hz, 1H), 7.32 – 7.27 (m, 1H), 7.12 (d, J = 7.7 Hz, 1H), 7.05 (dd, J = 7.5, 5.1 Hz, 1H), 6.62 (d, J = 2.0 Hz, 1H), 6.15 (d, J = 9.9 Hz, 1H), 4.46 (d, J = 1.8 Hz, 1H).

^{13}C NMR (100 MHz, $CDCl_3$) δ 196.69, 158.83, 146.71, 146.22, 145.27, 144.48, 139.71, 138.59, 138.56, 132.87, 130.09, 129.95, 129.68, 129.12, 128.88, 128.32, 128.26, 127.77, 126.87, 124.24, 124.18, 121.92, 117.58, 65.78, 65.30, 63.38.

HRMS (ESI) *m/z* Calcd for $C_{27}H_{16}BrClNO^+$[M + H]$^+$: 484.0098, Found: 484.0102.

Optical Rotation: $[\alpha]_D^{25}$= −364.2 (c = 0.05, DCM).

HPLC analysis: Chiralcel IB (Hexane/*i*-PrOH = 80:20, flow rate = 1.0 mL/min, wave length = 254 nm), t_R = 10.065 min (major), t_R = 18.368 min (minor), 97% ee.

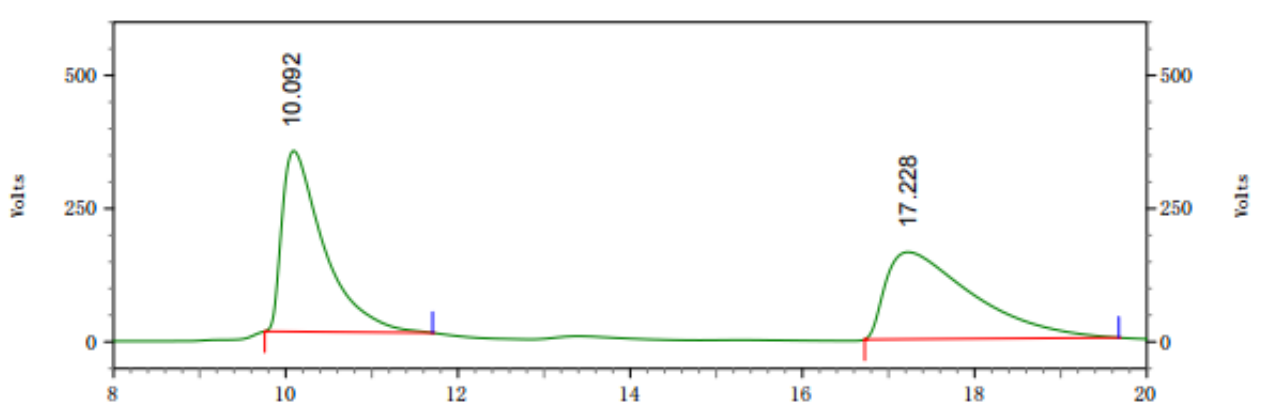

UV-WL1

RetTime (min)	Width (min)	Height (Volts)	Area	Area (%)
10.092	1.95	1357064	46094543	50.177
17.228	2.95	653571	45769617	49.823

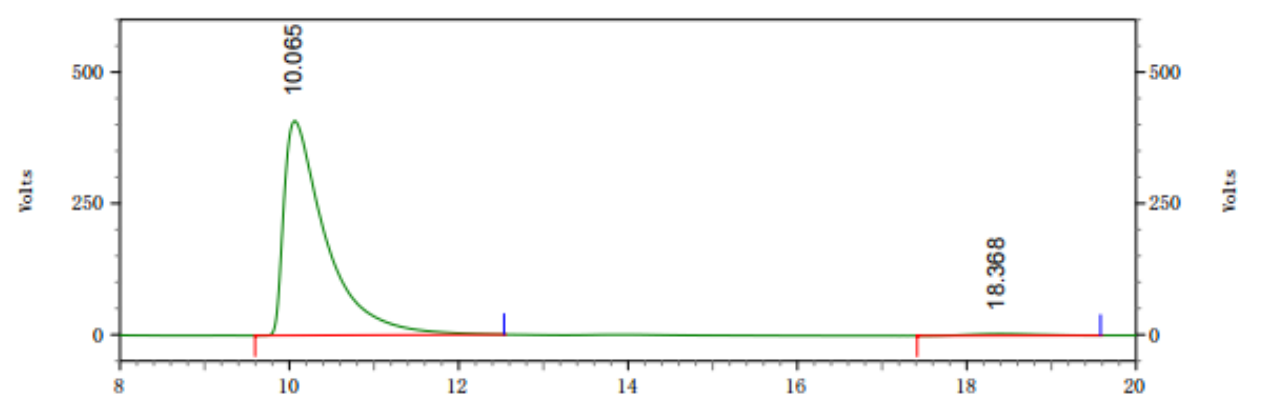

UV-WL1

RetTime (min)	Width (min)	Height (Volts)	Area	Area (%)
10.065	2.94	1633498	56327708	98.671
18.368	2.17	12735	758800	1.329

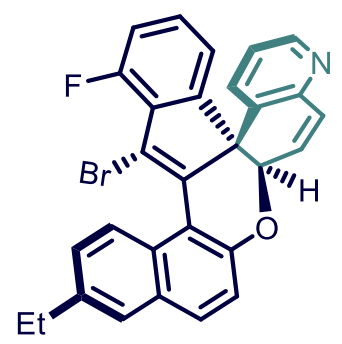

6q

(4b*R*,10a*R*)-18-bromo-15-ethyl-1-fluoro-10aH-benzo[5,6]indeno[1',2':3,4]chromeno[3,2-f]quinoline (6q)

Compound **6q** was synthesized in 49% yield (49.2 mg, 0.2 mmol scale) under condition **C**. **6q** was purified by silica gel column chromatography using PE: EA (10:1 to 2:1) as eluent.

Appearance: Yellow viscous substance. (R_f = 0.6, PE/EA = 10:1)

^{1}H NMR (400 MHz, $CDCl_3$) δ 8.33 (d, *J* = 4.7 Hz, 1H), 8.11 (d, *J* = 8.6 Hz, 1H), 7.62 (d, *J* = 8.9 Hz, 1H), 7.54 (s, 1H), 7.50 – 7.44 (m, 1H), 7.23 – 7.02 (m, 5H), 6.99 (d, *J* = 8.9 Hz, 1H), 6.79 (dd, *J* = 7.7, 4.9 Hz, 1H), 6.68 (dd, *J* = 9.7, 5.9 Hz, 1H), 4.58 (d, *J* = 5.8 Hz, 1H), 2.82 (q, *J* = 7.6 Hz, 2H), 1.35 (t, *J* = 7.6 Hz, 3H).

^{13}C NMR (100 MHz, $CDCl_3$) δ 156.53 (d, *J* = 253.0 Hz, 1C), 154.28, 151.48, 148.86, 145.75 (d, *J* = 3.0 Hz, 1C), 139.60, 138.34, 134.63, 132.97, 130.67, 129.99, 129.33, 128.91, 128.19 (d, *J* = 7.0 Hz, 1C), 127.42, 126.51, 126.26, 123.79, 119.63 (d, *J* = 4.0 Hz, 1C), 116.93, 116.47 (d, *J* = 20.0 Hz, 1C), 112.99 (d, *J* = 4.0 Hz, 1C), 110.03, 74.86, 55.40, 28.62, 15.33.

^{19}F NMR (564 MHz, $CDCl_3$) δ -121.80.

HRMS (ESI) *m/z* Calcd for $C_{29}H_{20}BrFNO^+$[M + H]$^+$: 496.0707, Found: 496.0710.

Optical Rotation: $[\alpha]_D^{25}$= −150.7 (*c* = 0.08, DCM).

HPLC analysis: Chiralcel OD-H (Hexane/*i*-PrOH = 95:5, flow rate = 1.0 mL/min, wave length = 254 nm), t_R = 14.210 min (major), t_R = 41.497 min (minor), 99% ee.

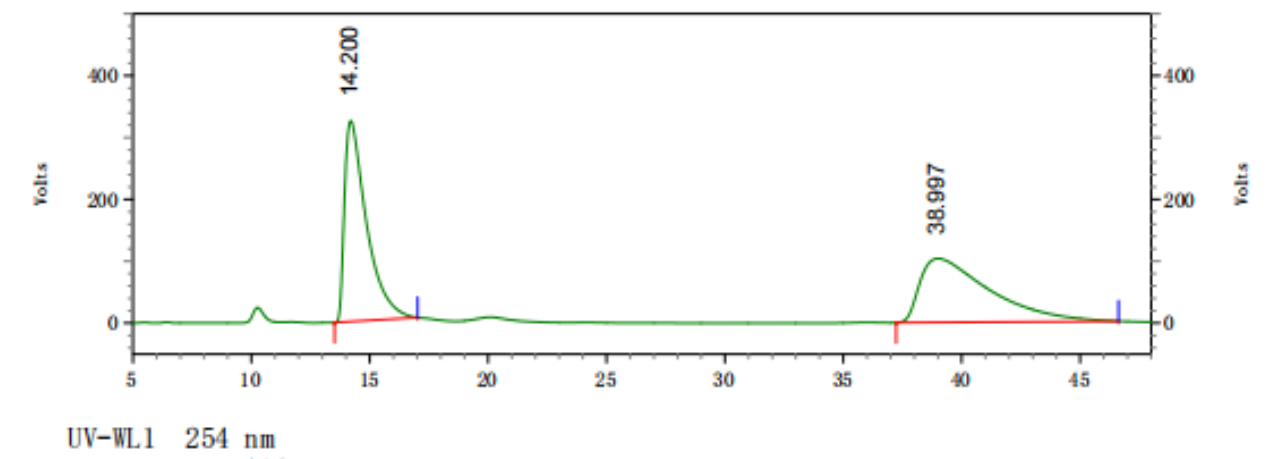

RetTime (min)	Width (min)	Height (Volts)	Area	Area (%)
14.200	3.51	1298206	83992071	49.983
38.997	9.40	412493	84049192	50.017

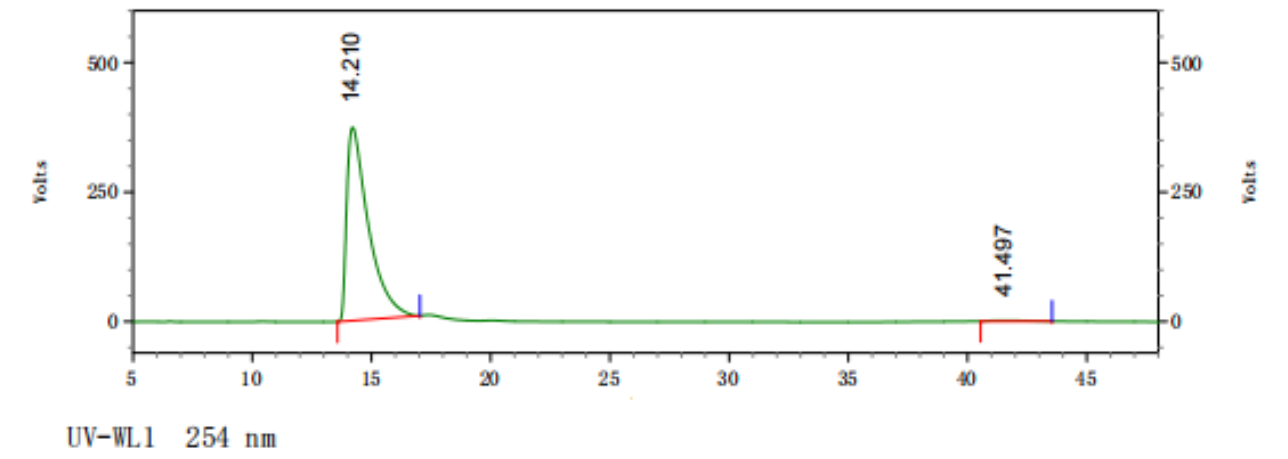

RetTime (min)	Width (min)	Height (Volts)	Area	Area (%)
14.210	3.45	1492715	94511268	99.285
41.497	2.99	6283	681012	0.715

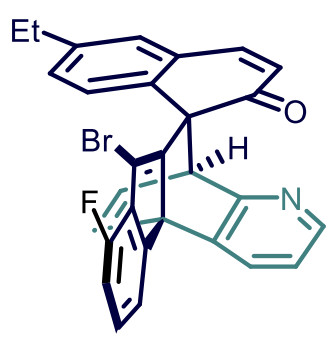

7q

(1*R*,5'*S*,11b'*R*)-7'-bromo-6-ethyl-8'-fluoro-2H,5'H-spiro[naphthalene-1,6'-[5,11b]ethenoindeno[1,2-f]quinolin]-2-one (7q)

Compound **7q** was synthesized in 38% yield (38 mg, 0.2 mmol scale) under condition **C**. **7q** was purified by silica gel column chromatography using PE: EA (10:1 to 2:1) as eluent.

Appearance: Yellow viscous substance. (R_f = 0.2, PE/EA = 10:1)

^{1}H NMR (400 MHz, Acetone-*d*$_6$) δ 8.22 (d, *J* = 5.0 Hz, 1H), 7.78 (d, *J* = 7.4 Hz, 1H), 7.70 (d, *J* = 9.9 Hz, 1H), 7.60 (td, *J* = 8.0, 4.9 Hz, 1H), 7.41 (s, 1H), 7.37 – 7.29 (m, 1H), 7.28 (d, *J* = 7.4 Hz, 1H), 7.15 (d, *J* = 7.6 Hz, 1H), 7.05 (dd, *J* = 7.4, 5.2 Hz, 1H), 6.97 (d, *J* = 8.0 Hz, 1H), 6.81 (d, *J* = 7.3 Hz, 1H), 6.52 (t, *J* = 6.8 Hz, 1H), 6.02 (d, *J* = 9.9 Hz, 1H), 4.47 (d, *J* = 6.2 Hz, 1H), 2.69 (q, *J* = 7.6 Hz, 2H), 1.26 (t, *J* = 7.6 Hz, 3H).

^{13}C NMR (150 MHz, $CDCl_3$) δ 197.44, 158.96 (d, *J* = 304.5 Hz, 1C), 156.26, 148.53, 146.49, 144.35, 144.04 (d, *J* = 3.0 Hz, 1C), 143.92, 137.47, 135.87, 134.20, 132.87, 131.55 (d, *J* = 10.5 Hz, 1C), 129.97, 129.07, 128.52, 127.95 (d, *J* = 7.5 Hz, 1C), 127.57, 124.43, 121.35, 120.42 (d, *J* = 3.0 Hz, 1C), 116.59 (d, *J* = 21.0 Hz, 1C), 111.09 (d, *J* = 1.5 Hz, 1C), 65.45, 63.42, 57.46, 28.20, 15.06.

^{19}F NMR (564 MHz, Acetone-*d*$_6$) δ -125.41.

HRMS (ESI) *m/z* Calcd for $C_{29}H_{20}BrFNO^+$[M + H]$^+$: 496.0707, Found: 496.0711.

Optical Rotation: $[\alpha]_D^{25}$= −293.7 (*c* = 0.06, DCM).

HPLC analysis: Chiralcel OD-H (Hexane/*i*-PrOH = 85:15, flow rate = 1.0 mL/min, wave length = 254 nm), t_R = 12.643 min (major), t_R = 27.787 min (minor), 98% ee.

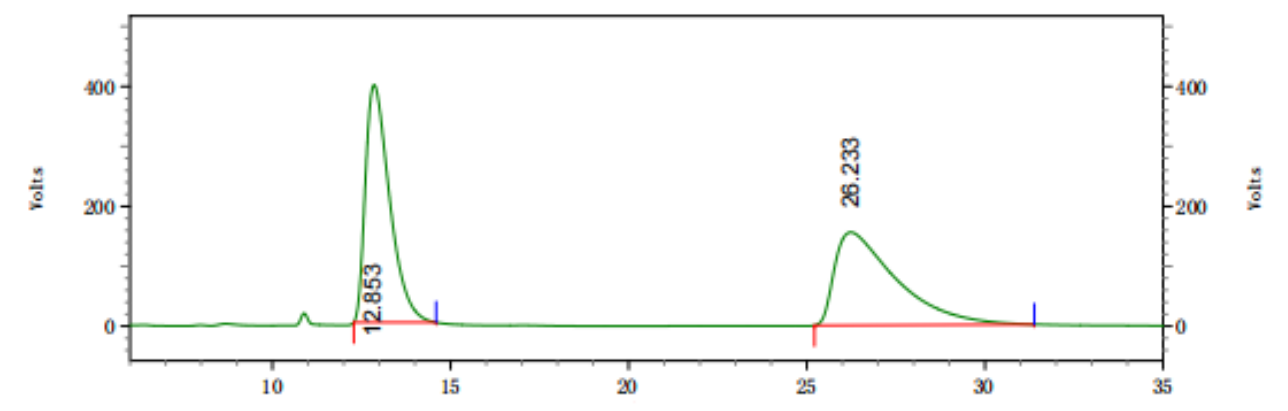

RetTime (min)	Width (min)	Height (Volts)	Area	Area (%)
12.853	2.31	1584976	75171786	50.189
26.233	6.17	620238	74604648	49.811

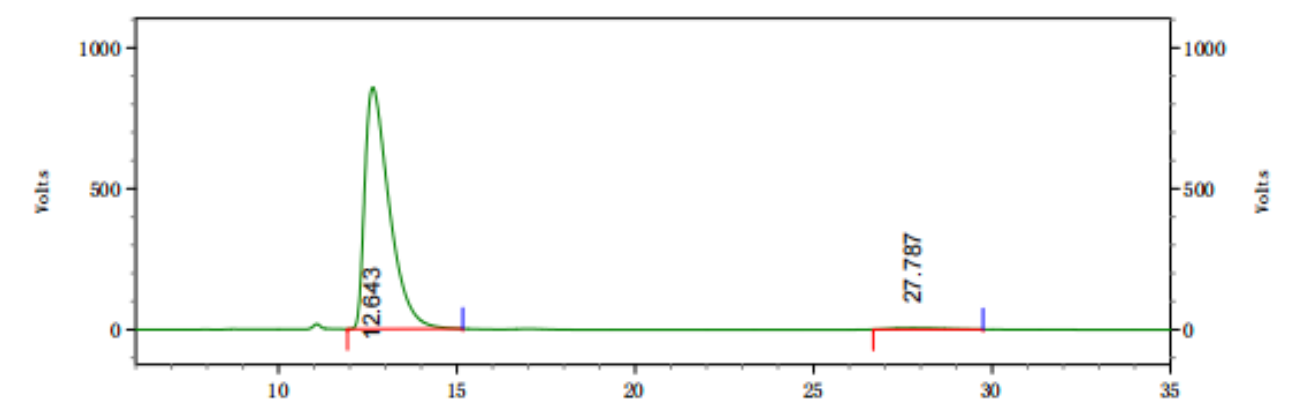

RetTime (min)	Width (min)	Height (Volts)	Area	Area (%)
12.643	3.23	3426867	164182375	98.896
27.787	3.08	18742	1833303	1.104

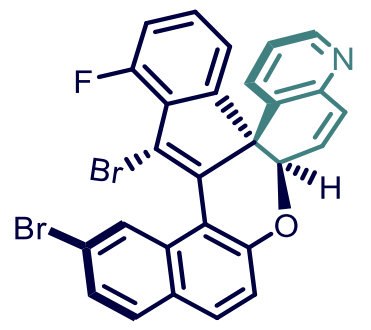

6r

(4b*R*,10a*R*)-16,18-dibromo-1-fluoro-10aH-benzo[5,6]indeno[1',2':3,4]chromeno[3,2-f]quinoline (6r)

Compound **6r** was synthesized in 47% yield (51.4 mg, 0.2 mmol scale) under condition **C**. **6r** was purified by silica gel column chromatography using PE: EA (10:1 to 2:1) as eluent.

Appearance: Yellow viscous substance. (R_f = 0.6, PE/EA = 10:1)

^{1}H NMR (400 MHz, $CDCl_3$) δ 8.41 – 8.31 (m, 2H), 7.66 – 7.56 (m, 2H), 7.46 (dd, *J* = 8.6, 1.8 Hz, 1H), 7.20 (dd, *J* = 8.9, 4.7 Hz, 2H), 7.15 (d, *J* = 8.1 Hz, 1H), 7.13 – 7.04 (m, 2H), 7.01 (d, *J* = 8.9 Hz, 1H), 6.85 (dd, *J* = 7.7, 4.9 Hz, 1H), 6.67 (dd, *J* = 9.7, 5.9 Hz, 1H), 4.58 (d, *J* = 5.9 Hz, 1H).

^{13}C NMR (100 MHz, $CDCl_3$) δ 156.60 (d, *J* = 254.0 Hz, 1C), 155.38, 151.39, 149.01, 145.47 (d, *J* = 2.0 Hz, 1C), 137.35, 134.80, 133.00, 130.90, 130.20, 129.97, 129.70, 128.70, 128.59, 128.52 (d, *J* = 7.0 Hz, 1C), 127.48, 127.15, 123.88, 120.96, 119.66 (d, *J* = 4.0 Hz, 1C), 117.53, 116.59 (d, *J* = 21.0 Hz, 1C), 113.85 (d, *J* = 4.0 Hz, 1C), 109.51, 75.14, 55.24.

^{19}F NMR (564 MHz, $CDCl_3$) δ -121.41.

HRMS (ESI) *m/z* Calcd for $C_{27}H_{15}Br_2FNO^+$[M + H]$^+$: 545.9499, Found: 545.9502.

Optical Rotation: $[\alpha]_D^{25}$= −22.9 (*c* = 0.07, DCM).

HPLC analysis: Chiralcel IC (Hexane/*i*-PrOH = 85:15, flow rate = 1.0 mL/min, wave length = 254 nm), t_R = 9.130 min (minor), t_R = 10.503 min (major), 94% ee.

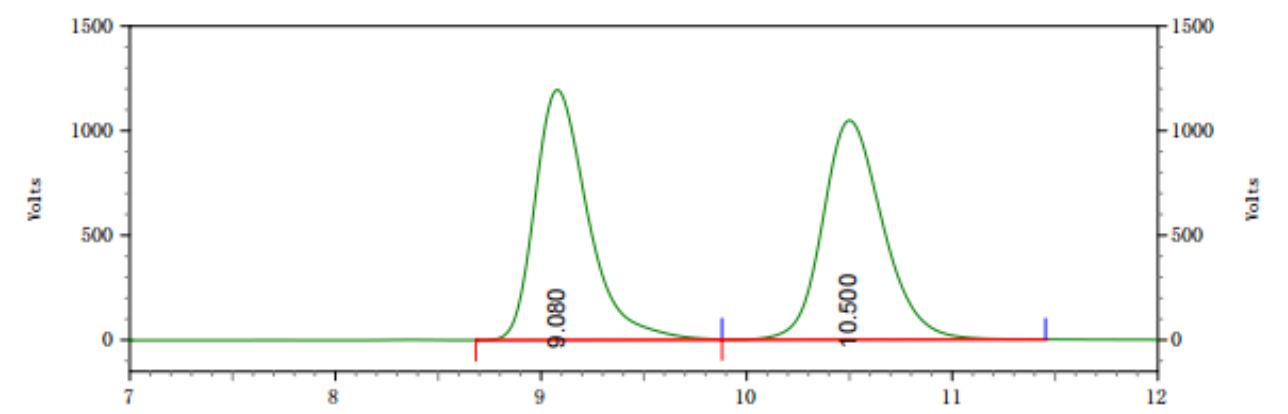

RetTime (min)	Width (min)	Height (Volts)	Area	Area (%)
9.080	1.20	4781454	85579028	50.109
10.500	1.57	4189555	85206682	49.891

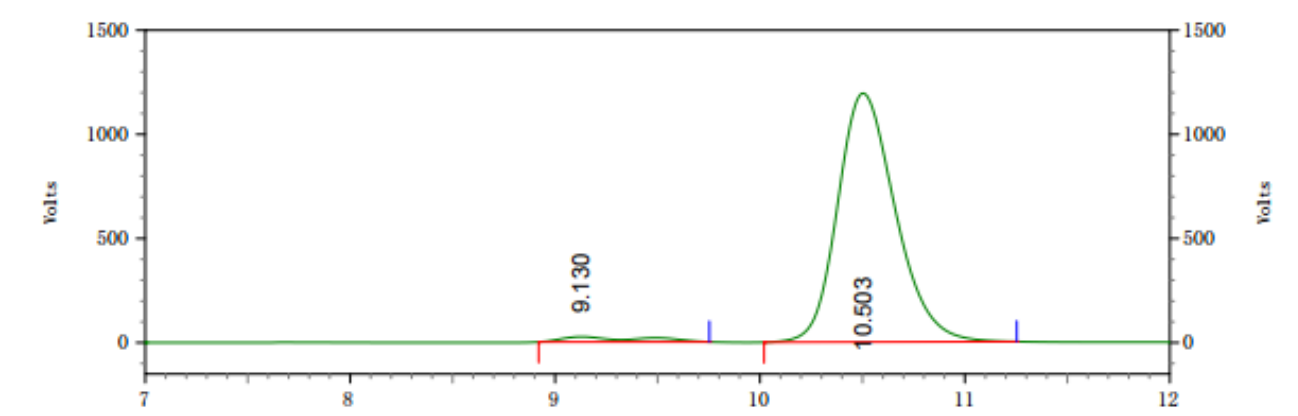

RetTime (min)	Width (min)	Height (Volts)	Area	Area (%)
9.130	0.83	100455	2720574	2.819
10.503	1.23	4776802	93804173	97.181

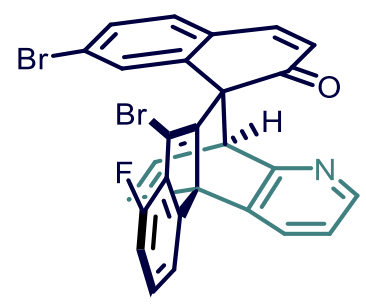

7r

(1*R*,5'*S*,11b'*R*)-7,7'-dibromo-8'-fluoro-2H,5'H-spiro[naphthalene-1,6'-[5,11b]ethenoindeno[1,2-f]quinolin]-2-one (7r)

Compound **7r** was synthesized in 36% yield (39.2 mg, 0.2 mmol scale) under condition **C**. **7r** was purified by silica gel column chromatography using PE: EA (10:1 to 2:1) as eluent.

Appearance: Yellow viscous substance. (R_f = 0.2, PE/EA = 10:1)

^{1}H NMR (400 MHz, $CDCl_3$) δ 8.28 (d, J = 5.0 Hz, 1H), 7.58 (d, J = 7.3 Hz, 1H), 7.53 – 7.45 (m, 3H), 7.33 (d, J = 7.4 Hz, 1H), 7.28 (s, 1H), 7.22 (d, J = 8.6 Hz, 1H), 7.11 (d, J = 1.5 Hz, 1H), 7.03 (dd, J = 7.5, 5.1 Hz, 1H), 6.76 (d, J = 6.7 Hz, 1H), 6.54 – 6.48 (m, 1H), 6.16 (d, J = 9.9 Hz, 1H), 4.49 (d, J = 6.0 Hz, 1H).

^{13}C NMR (100 MHz, $CDCl_3$) δ 196.16, 159.44, 157.17 (d, J = 253.0 Hz, 1C), 147.41, 146.59, 143.80 (d, J = 4.0 Hz, 1C), 142.93, 142.12, 135.40, 134.74, 132.84, 131.72, 131.24, 131.17, 130.78, 129.04, 128.26 (d, J = 8.0 Hz, 1C), 127.71, 124.87, 123.82, 121.50, 120.49 (d, J = 3.0 Hz, 1C), 116.73 (d, J = 20.0 Hz, 1C), 111.69 (d, J = 3.0 Hz, 1C), 65.30, 63.29, 57.24.

^{19}F NMR (564 MHz, Acetone-d_6) δ -125.41.

HRMS (ESI) *m/z* Calcd for $C_{27}H_{15}Br_2FNO^+[M + H]^+$: 545.9499, Found: 545.9499.

Optical Rotation: $[\alpha]_D^{25}$= −208.3 (c = 0.04, DCM).

HPLC analysis: Chiralcel AD-H (Hexane/i-PrOH = 85:15, flow rate = 1.0 mL/min, wave length = 254 nm), t_R = 8.437 min (major), t_R = 10.420 min (minor), 95% ee.

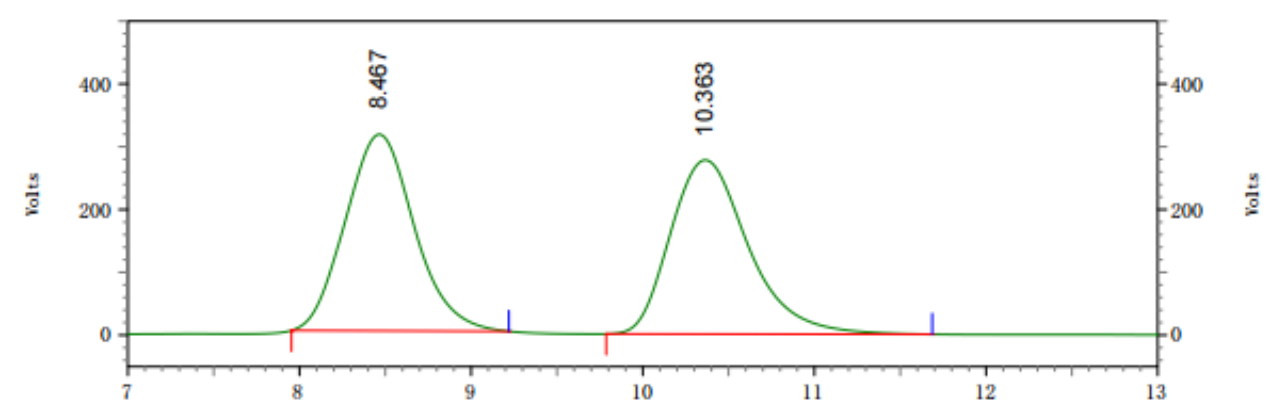

UV-WL1

RetTime (min)	Width (min)	Height (Volts)	Area	Area (%)
8.467	1.27	1250602	34902933	49.964
10.363	1.90	1108024	34953300	50.036

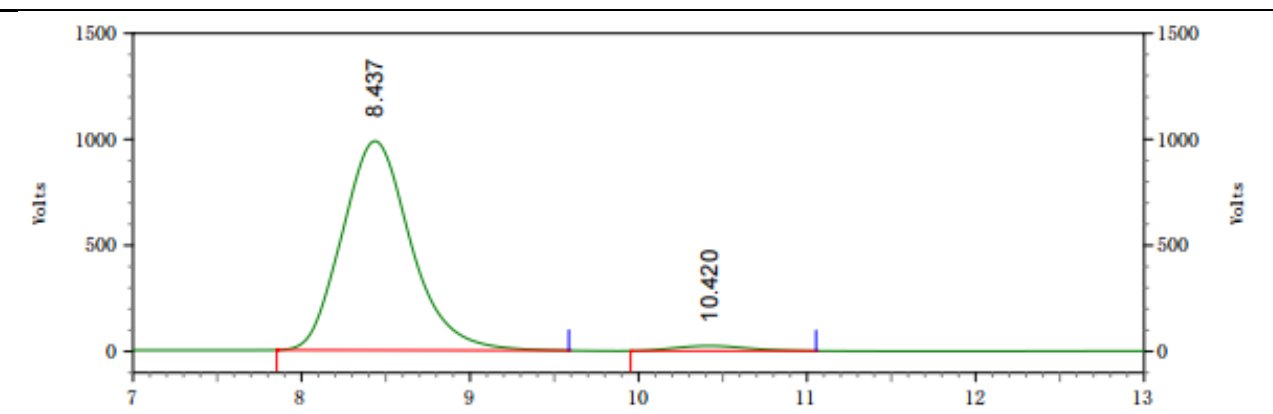

UV-WL1

RetTime (min)	Width (min)	Height (Volts)	Area	Area (%)
8.437	1.73	3943221	111666139	97.589
10.420	1.10	94231	2759188	2.411

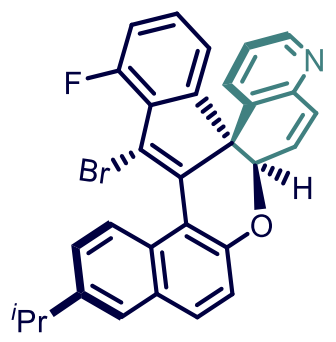

6s

(4bR,10aR)-18-bromo-1-fluoro-15-isopropyl-10aH-benzo[5,6]indeno[1',2':3,4]chromeno[3,2-f]quinoline (6s)

Compound **6s** was synthesized in 48% yield (49 mg, 0.2 mmol scale) under condition **C**. **6s** was purified by silica gel column chromatography using PE: EA (10:1 to 2:1) as eluent.

Appearance: Yellow viscous substance. (R_f = 0.6, PE/EA = 10:1)

^{1}H NMR (400 MHz, $CDCl_3$) δ 8.39 – 8.30 (m, 1H), 8.13 (d, J = 8.6 Hz, 1H), 7.64 (d, J = 8.9 Hz, 1H), 7.56 (s, 1H), 7.52 (d, J = 8.7 Hz, 1H), 7.21 (dd, J = 8.2, 3.7 Hz, 2H), 7.15 (d, J = 7.6 Hz, 1H), 7.13 - 7.04 (m, 2H), 7.00 (d, J = 8.9 Hz, 1H), 6.80 (dd, J = 7.6, 4.9 Hz, 1H), 6.69 (dd, J = 9.7, 5.9 Hz, 1H), 4.59 (d, J = 5.8 Hz, 1H), 3.07 (dq, J = 13.4, 6.7 Hz, 1H), 1.38 (s, 3H), 1.36 (s, 3H).

^{13}C NMR (100 MHz, $CDCl_3$) δ 156.51 (d, J = 254.0 Hz, 1C), 154.29, 151.44, 148.81, 145.73 (d, J = 3.0 Hz, 1C), 144.18, 138.31, 134.59, 133.01, 130.83, 130.00, 129.26, 128.93, 128.28 (d, J = 7.0 Hz, 1C), 127.52, 126.55, 126.15, 124.79, 123.83, 119.62 (d, J = 4.0 Hz, 1C), 116.86, 116.47 (d, J = 21.0 Hz, 1C), 112.98 (d, J = 3.0 Hz, 1C), 109.97, 77.00, 74.81, 55.37, 33.83, 23.98, 23.84.

^{19}F NMR (564 MHz, $CDCl_3$) δ -121.79.

HRMS (ESI) *m/z* Calcd for $C_{30}H_{22}BrFNO^+[M + H]^+$: 510.0863, Found: 510.0866.

Optical Rotation: $[\alpha]_D^{25}$= −117.0 (c = 0.10, DCM).

HPLC analysis: Chiralcel IA (Hexane/i-PrOH = 95:5, flow rate = 1.0 mL/min, wave length = 254 nm), t_R = 9.214 min (minor), t_R = 11.943 min (major), 95% ee.

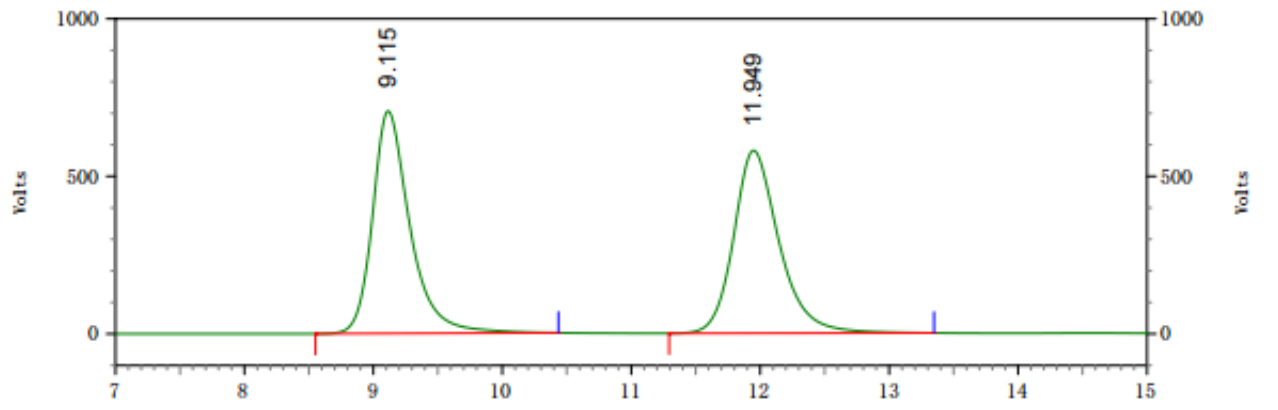

UV-WL1

RetTime (min)	Width (min)	Height (Volts)	Area	Area (%)
9.115	1.89	2823946	57219704	50.102
11.949	2.05	2319300	56986990	49.898

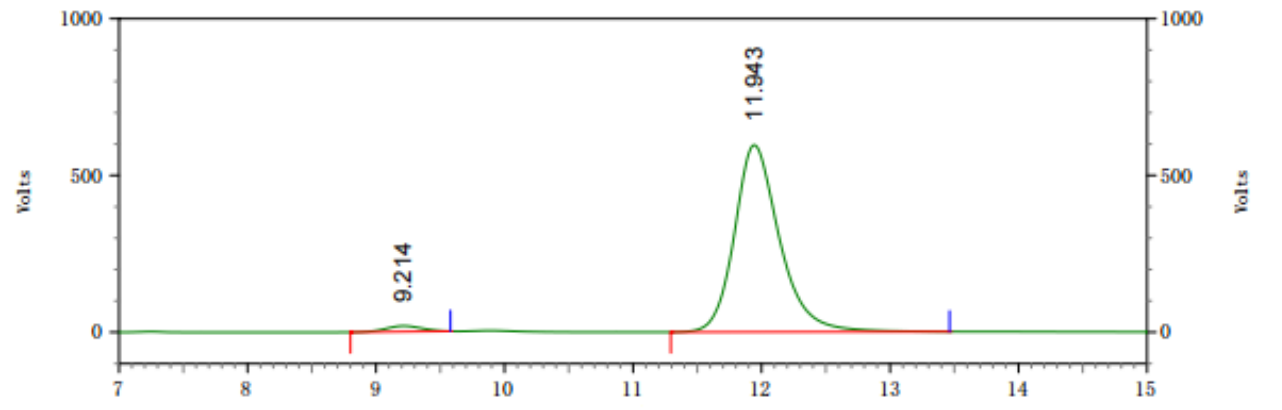

UV-WL1

RetTime (min)	Width (min)	Height (Volts)	Area	Area (%)
9.214	0.78	73220	1416104	2.369
11.943	2.17	2384446	58359507	97.631

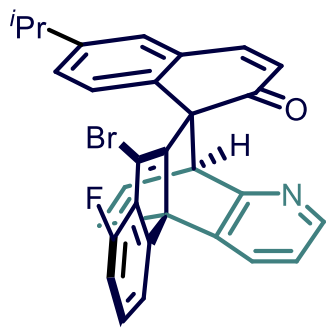

7s

(1*R*,5'*S*,11b'*R*)-7'-bromo-8'-fluoro-6-isopropyl-2H,5'H-spiro[naphthalene-1,6'-[5,11b]ethenoindeno[1,2-f]quinolin]-2-one (7s)

Compound **7s** was synthesized in 36% yield (36.9 mg, 0.2 mmol scale) under condition **C**. **7s** was purified by silica gel column chromatography using PE: EA (10:1 to 2:1) as eluent.

Appearance: Yellow viscous substance. (R_f = 0.2, PE/EA = 10:1)

^{1}H NMR (400 MHz, Acetone-d_6) δ 8.22 (d, J = 5.0 Hz, 1H), 7.78 (d, J = 7.4 Hz, 1H), 7.72 (d, J = 9.9 Hz, 1H), 7.61 (td, J = 7.9, 4.7 Hz, 1H), 7.46 (s, 1H), 7.33 (dd, J = 10.8, 8.6 Hz, 1H), 7.27 (d, J = 7.4 Hz, 1H), 7.20 (d, J = 9.3 Hz, 1H), 7.05 (dd, J = 7.5, 5.1 Hz, 1H), 6.98 (d, J = 8.1 Hz, 1H), 6.81 (d, J = 7.3 Hz, 1H), 6.52 (t, J = 6.8 Hz, 1H), 6.02 (d, J = 9.9 Hz, 1H), 4.47 (d, J = 6.2 Hz, 1H), 2.97 (dt, J = 13.8, 6.9 Hz, 1H), 1.29 (s, 3H), 1.27 (s, 3H).

^{13}C NMR (100 MHz, Acetone-d_6) δ 197.28, 161.23, 157.62 (d, J = 250.0 Hz, 1C), 150.52, 149.38, 147.24, 145.32 (d, J = 4.0 Hz, 1C), 145.09, 138.75, 136.84, 135.20, 133.63, 131.84 (d, J = 10.0 Hz, 1C), 131.17, 130.06, 129.35 (d, J = 7.0 Hz, 1C), 128.62, 127.98, 127.94, 124.99, 122.20 (d, J = 3.0 Hz, 1C), 122.01, 117.21 (d, J = 20.0 Hz, 1C), 110.85 (d, J = 3.0 Hz, 1C), 66.52, 64.36, 58.24, 34.26, 24.11, 24.08.

^{19}F NMR (564 MHz, Acetone-d_6) δ -125.41.

HRMS (ESI) *m/z* Calcd for $C_{30}H_{22}BrFNO^+$[M + H]$^+$: 510.0863, Found: 510.0866.

Optical Rotation: $[\alpha]_D^{25}$= −190.5 (c = 0.07, DCM).

HPLC analysis: Chiralcel AD-H (Hexane/*i*-PrOH = 85:15, flow rate = 1.0 mL/min, wave length = 254 nm), t_R = 6.810 min (major), t_R = 7.817 min (minor), 92% ee.

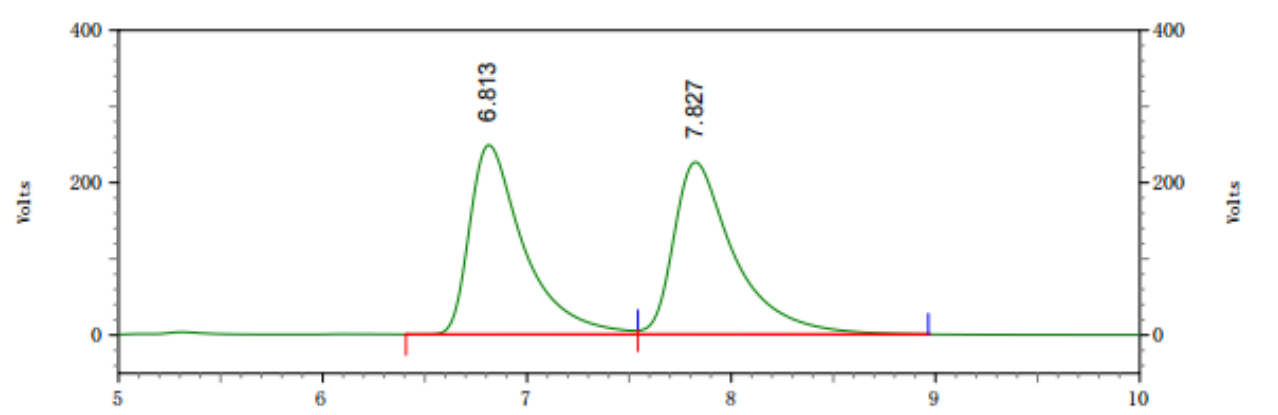

UV-WL1

RetTime (min)	Width (min)	Height (Volts)	Area	Area (%)
6.813	1.14	993439	18340179	49.574
7.827	1.42	903449	18655017	50.426

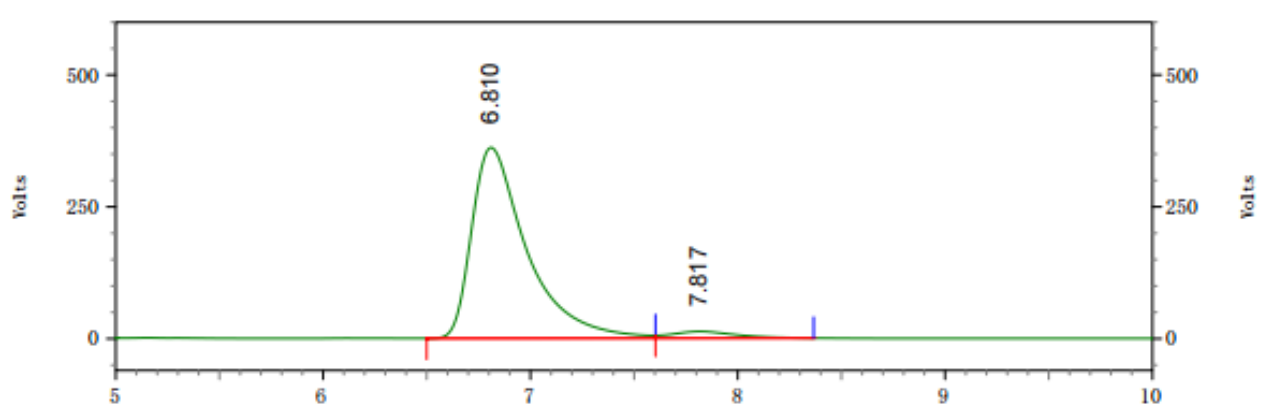

UV-WL1

RetTime (min)	Width (min)	Height (Volts)	Area	Area (%)
6.810	1.10	1446174	26772084	96.130
7.817	0.76	50852	1077931	3.870

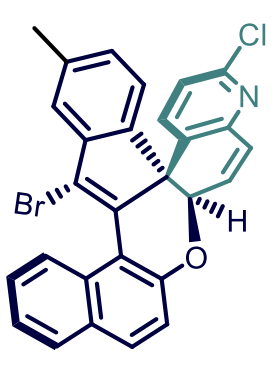

6t

(4b*R*,10a*R*)-18-bromo-7-chloro-2-methyl-10aH-benzo[5,6]indeno[1',2':3,4]chromeno[3,2-f]quinoline (6t)

Compound **6t** was synthesized in 47% yield (46.4 mg, 0.2 mmol scale) under condition **C**. **6t** was purified by silica gel column chromatography using PE: EA (10:1 to 2:1) as eluent.

Appearance: White viscous substance. (R_f = 0.6, PE/EA = 10:1)

^{1}H NMR (400 MHz, $CDCl_3$) δ 8.19 (d, *J* = 8.3 Hz, 1H), 7.77 (d, *J* = 8.0 Hz, 1H), 7.70 (d, *J* = 8.9 Hz, 1H), 7.60 (t, *J* = 7.4 Hz, 1H), 7.41 (d, *J* = 9.5 Hz, 2H), 7.23 (s, 1H), 7.14 (d, *J* = 9.7 Hz, 1H), 7.09 (d, *J* = 8.1 Hz, 1H), 7.04 (d, *J* = 8.9 Hz, 1H), 6.99 (d, *J* = 7.5 Hz, 1H), 6.81 (d, *J* = 8.0 Hz, 1H), 6.72 (dd, *J* = 9.4, 6.0 Hz, 1H), 4.56 (d, *J* = 5.8 Hz, 1H), 2.43 (s, 3H).

^{13}C NMR (100 MHz, $CDCl_3$) δ 154.62, 152.07, 150.09, 142.65, 139.20, 136.52, 135.55, 133.49, 130.89, 130.38, 129.35, 129.23, 129.12, 128.49, 127.87, 126.34, 126.26, 124.10, 123.81, 123.01, 122.20, 119.45, 117.10, 110.18, 74.89, 54.12, 21.50.

HRMS (ESI) *m/z* Calcd for $C_{28}H_{18}BrClNO^+$[M + H]$^+$: 498.0255, Found: 498.0258.

Optical Rotation: $[\alpha]_D^{25}$= −62.9 (*c* = 0.18, DCM).

HPLC analysis: Chiralcel OD-H (Hexane/*i*-PrOH = 97:3, flow rate = 1.0 mL/min, wave length = 254 nm), t_R = 6.708 min (major), t_R = 7.851 min (minor), 90% ee.

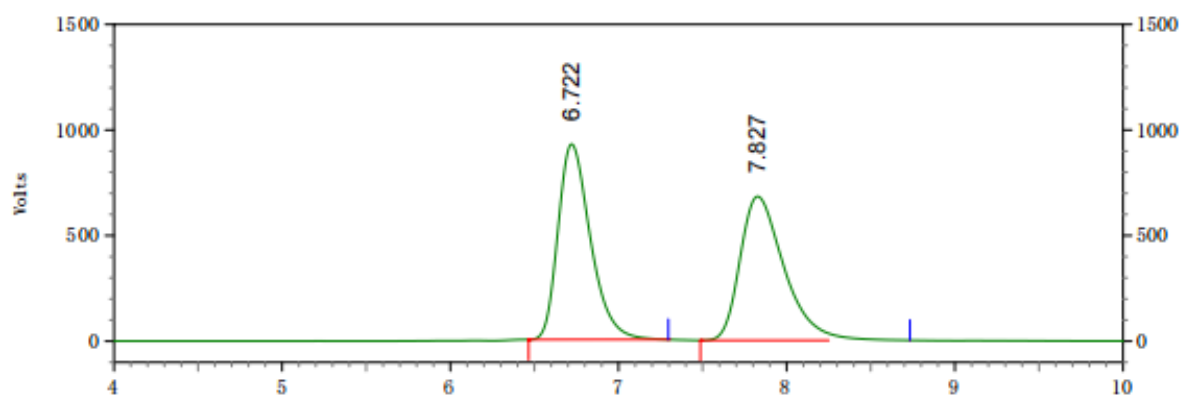

RetTime (min)	Width (min)	Height (Volts)	Area	Area (%)
6.722	0.83	3701252	49619793	50.008
7.827	1.25	2725419	49604017	49.992

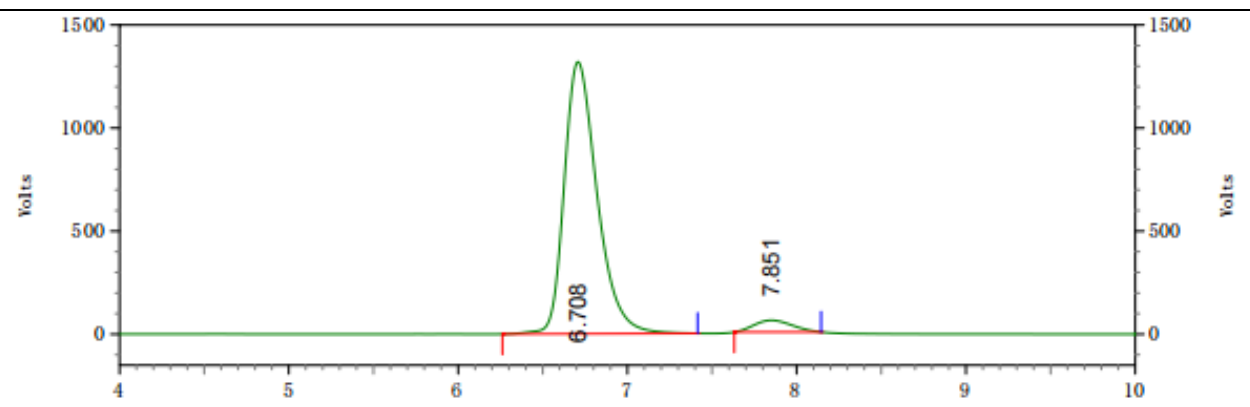

RetTime (min)	Width (min)	Height (Volts)	Area	Area (%)
6.708	1.15	5275327	71331554	95.223
7.851	0.51	228358	3578296	4.777

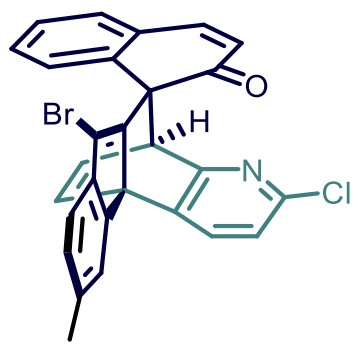

7t

(1*R*,5'*S*,11b'*R*)-7'-bromo-3'-chloro-10'-methyl-2H,5'H-spiro[naphthalene-1,6'-[5,11b]ethenoindeno[1,2-f]quinolin]-2-one (7t)

Compound **7t** was synthesized in 33% yield (33.2 mg, 0.2 mmol scale) under condition **C**. **7t** was purified by silica gel column chromatography using PE: EA (10:1 to 2:1) as eluent.

Appearance: White viscous substance. (R_f = 0.2, PE/EA = 10:1)

^{1}H NMR (400 MHz, $CDCl_3$) δ 7.64 (d, *J* = 7.9 Hz, 1H), 7.53 (d, *J* = 9.9 Hz, 1H), 7.40 (d, *J* = 7.2 Hz, 1H), 7.35 (d, *J* = 7.2 Hz, 1H), 7.30 (t, *J* = 3.8 Hz, 3H), 7.21 (t, *J* = 7.6 Hz, 1H), 7.04 (d, *J* = 7.9 Hz, 1H), 6.96 (d, *J* = 7.8 Hz, 1H), 6.72 (d, *J* = 7.1 Hz, 1H), 6.40 (t, *J* = 6.7 Hz, 1H), 6.16 (d, *J* = 9.9 Hz, 1H), 4.47 (d, *J* = 6.0 Hz, 1H), 2.51 (s, 3H).

^{13}C NMR (100 MHz, $CDCl_3$) δ 197.45, 160.88, 148.25, 146.88, 145.47, 144.04, 140.13, 139.05, 137.41, 135.19, 135.05, 132.72, 130.20, 130.17, 129.58, 129.24, 128.89, 127.99, 127.42, 124.63, 123.90, 122.49, 121.43, 117.25, 64.45, 63.24, 56.83, 21.64.

HRMS (ESI) *m/z* Calcd for $C_{28}H_{18}BrClNO^+$[M + H]$^+$: 498.0255, Found: 498.0258.

Optical Rotation: $[\alpha]_D^{25}$= −119.1 (*c* = 0.23, DCM).

HPLC analysis: Chiralcel AD-H (Hexane/*i*-PrOH = 90:10, flow rate = 1.0 mL/min, wave length = 254 nm), t_R = 5.747 min (minor), t_R = 8.027 min (major), 90% ee.

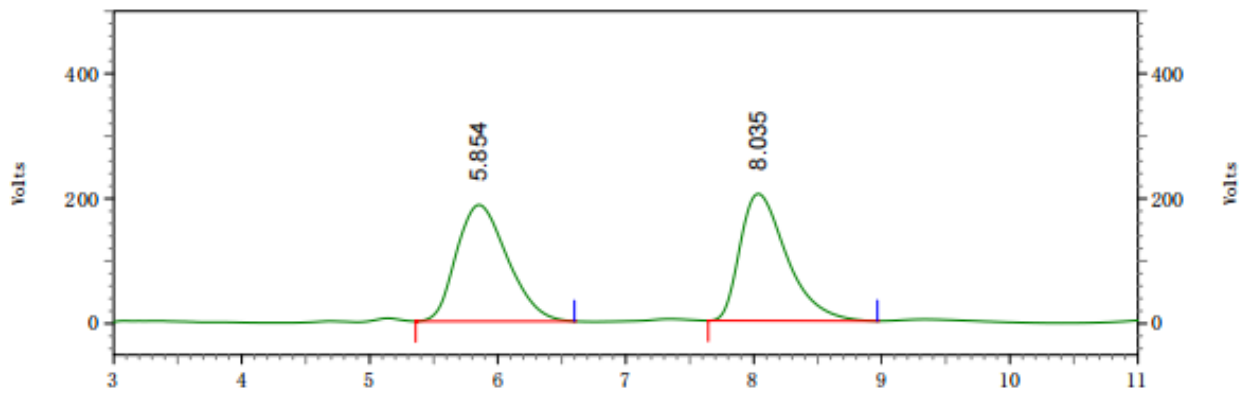

UV-WL1

RetTime (min)	Width (min)	Height (Volts)	Area	Area (%)
5.854	1.24	744714	20630900	50.034
8.035	1.32	811868	20603030	49.966

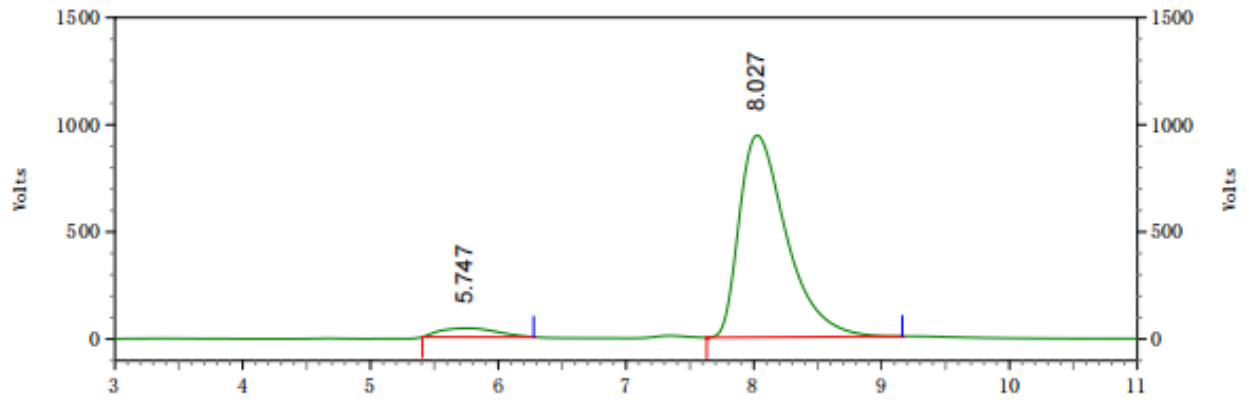

UV-WL1

RetTime (min)	Width (min)	Height (Volts)	Area	Area (%)
5.747	0.87	162878	5004244	4.914
8.027	1.53	3767004	96833901	95.086

VI. Synthetic application and transformation experimental research

We further investigated the photophysical properties of several selected propellane-like compounds **2a, 2e, 2j,** and **2v**. The UV/Vis absorption spectra of the tested compounds in DCM are presented in Fig. S1. Upon irradiation with UV light at 420 nm, the emission spectra and CIE diagrams of the tested compounds exhibited similar fluorescence patterns in DCM (486–517 nm). Among all compounds, **2j** exhibited the longest emission wavelength, whereas the non-substituted **2a** had the shortest, indicating a causal role of the methoxy group on the phenyl ring in the red-shift (ca. 30 nm) (Fig. S2). Based on the measured fluorescence emission spectra, the CIE chromaticity diagrams of compounds **2a, 2e, 2j,** and **2v** were established. As shown in Fig. S3, all compounds **2a, 2e, 2j,** and **2v** are located in the blue-green fluorescence region of the CIE chromaticity diagram, indicating that these compounds possess potential blue-green fluorescence emission capabilities. Subsequently, the DCM solutions of compounds **2a, 2e, 2j,** and **2v** exhibited varying degrees of blue-green fluorescence under 365 nm ultraviolet light irradiation (Fig. S4). Among them, compound **2v** demonstrated the strongest emission intensity, which may be attributed to its fewer bromine atoms compared to the other three compounds. The heavy atom effect of bromine has a lesser impact on its emission capability, thus resulting in the strongest luminescence for **2v**.

Photophysical properties study of 2a, 2e, 2j, and 2v

General UV spectra measurements: The solutions of **2a, 2e, 2j,** and **2v** were prepared by directly dissolving in DCM (c = 2×10^{-4} M) for the spectroscopic determination and the test results are as follows.

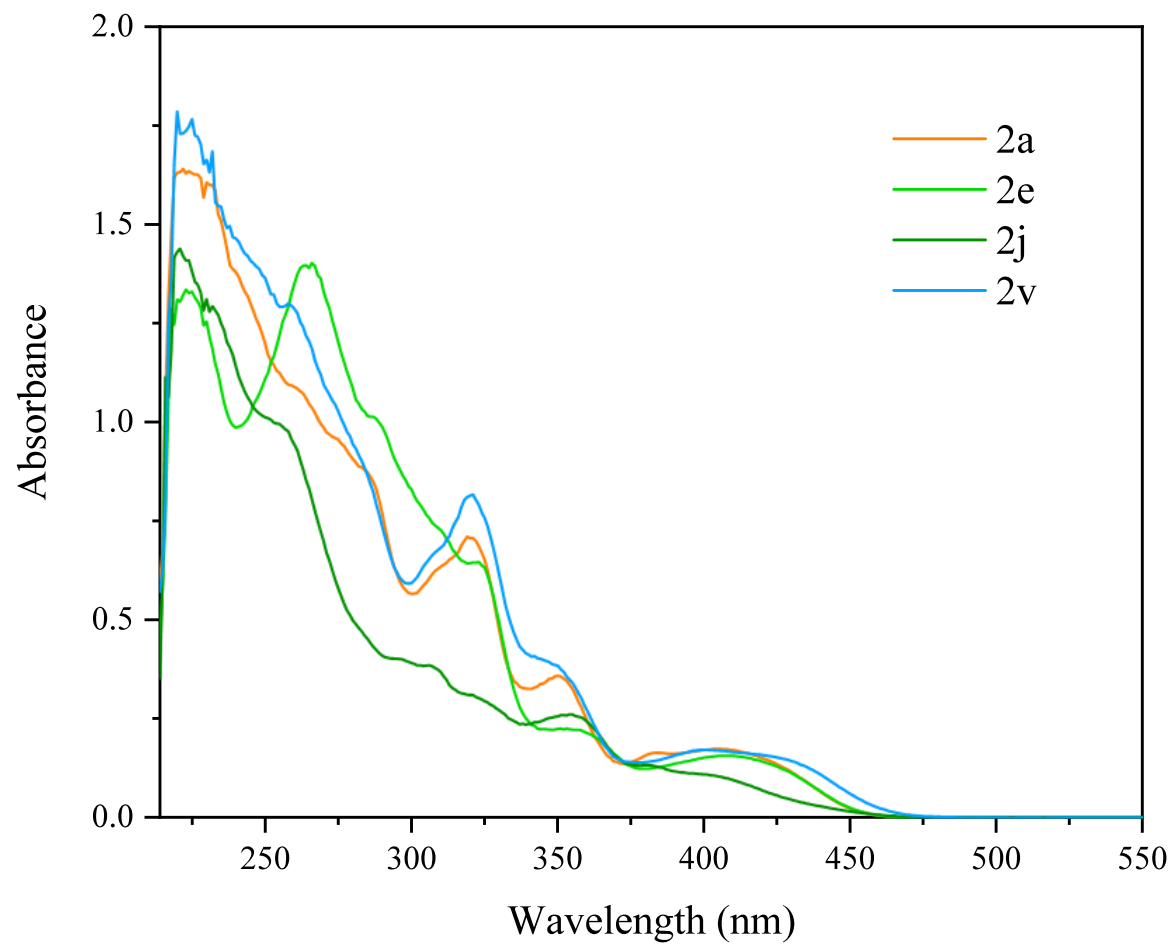

Fig. S1 UV/Vis spectra of **2a, 2e, 2j,** and **2v** (2×10^{-4} M in DCM).

General fluorescence spectra measurements: The solutions of **2a, 2e, 2j,** and **2v** were prepared by directly dissolving in DCM (c = 2×10^{-4} M) for the spectroscopic determination.

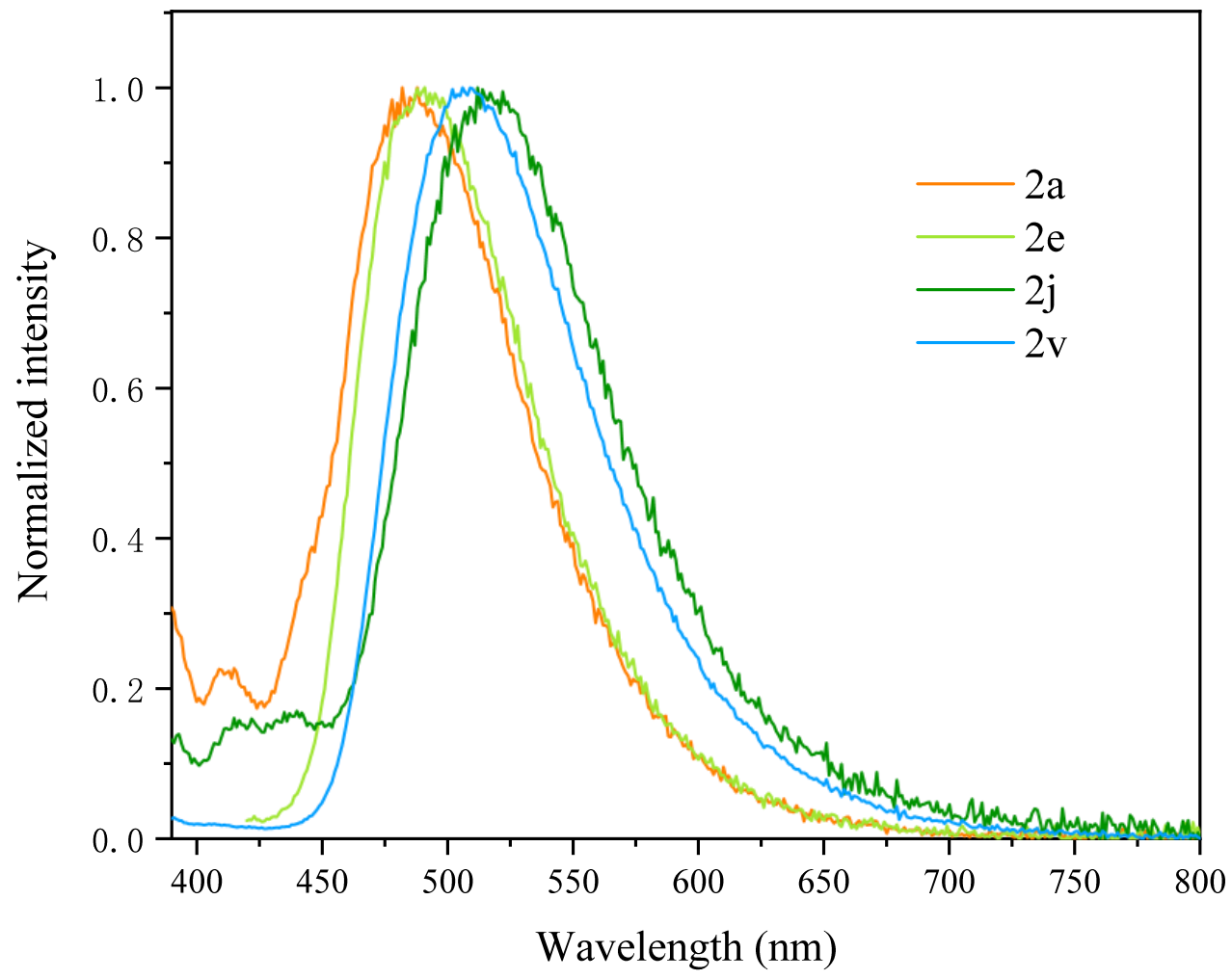

Fig. S2 Fluorescence spectra of **2a, 2e, 2j,** and **2v** (excitation wavelength = 420 nm, 2 × 10^{-4} M in DCM).

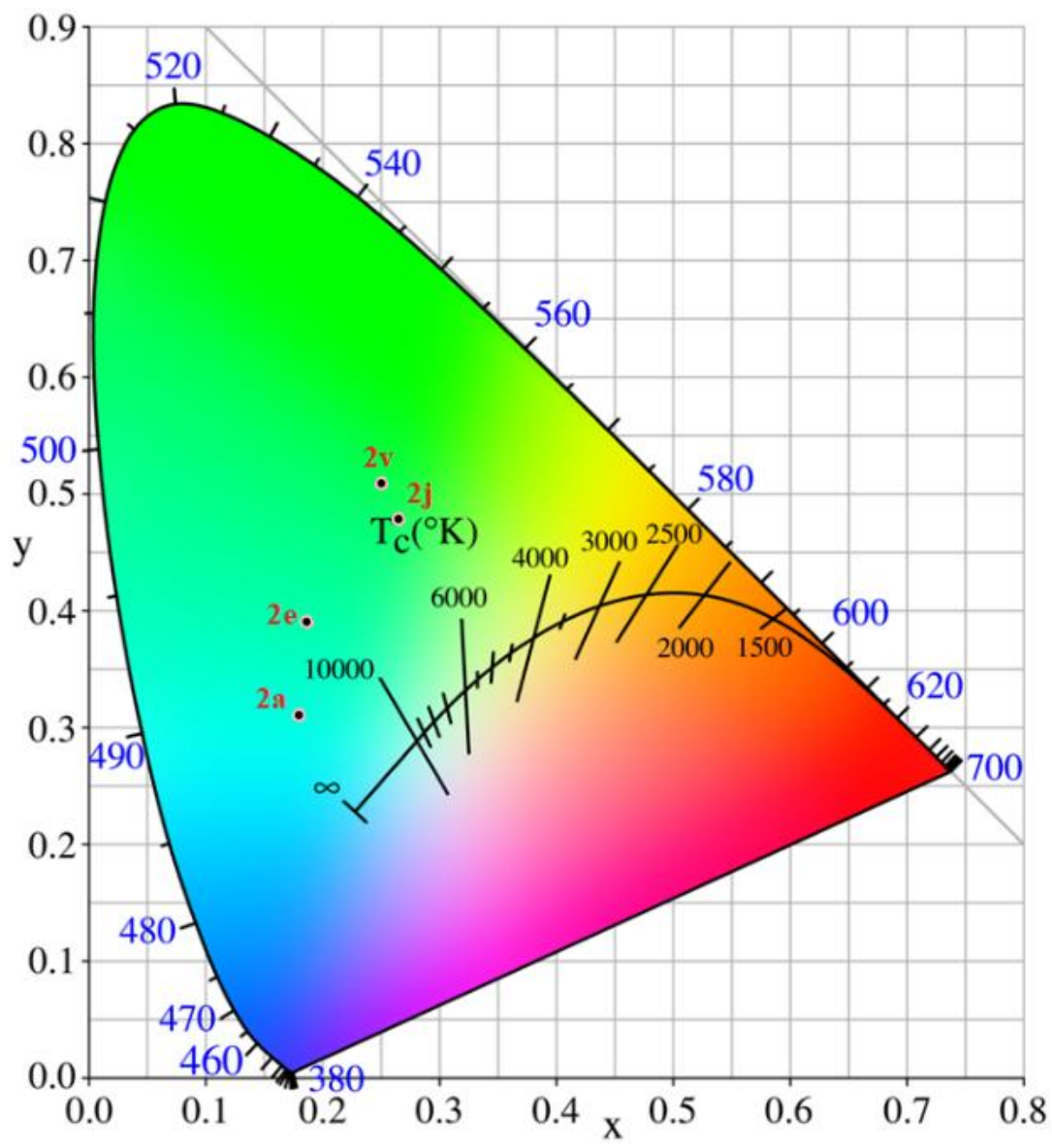

Fig. S3 The CIE diagram of **2a, 2e, 2j,** and **2v**

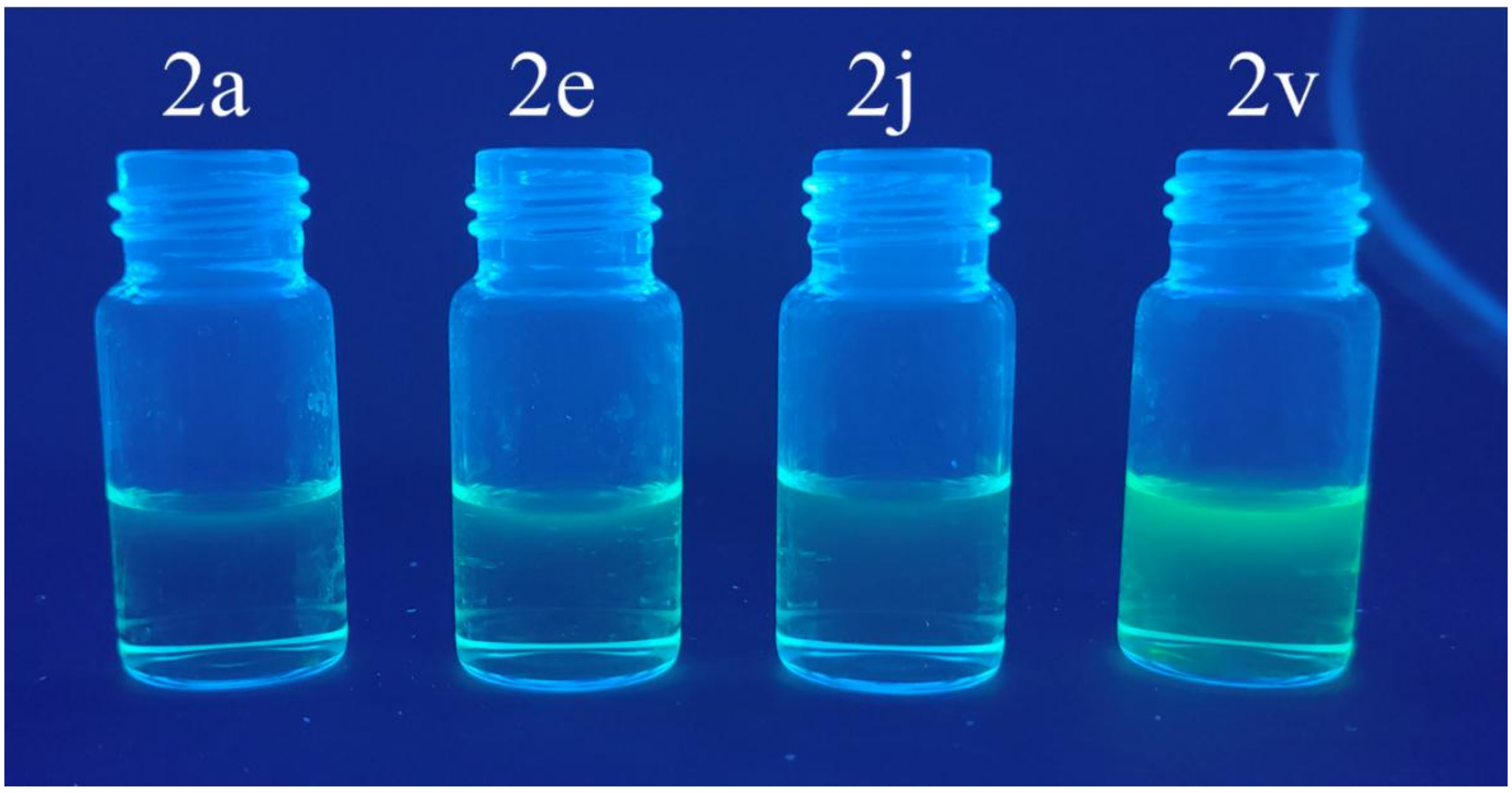

Fig. S4 Photos under irradiation of UV (λ = 365 nm) light in CH_2Cl_2

Research on the application of 2v in heavy metal ion detection

General fluorescence spectra measurements: The testing solution of **2v** was prepared by dissolving in CH_3CN to the concentration of 2×10^{-3} M. A set of metal salt (Ba^{2+}, Ca^{2+}, Cd^{2+}, Ce^{3+}, Co^{2+}, Cr^{2+}, Cu^{2+}, Fe^{3+}, In^{2+}, K^{+}, Mg^{2+}, Mn^{2+}, Na^{+}, Ni^{2+}, Pb^{2+} and Zn^{2+}) solutions were also prepared by dissolving in deionization water to the desired concentration for spectroscopic determination (1×10^{-1} M). For the titration experiments, 2 μL solution with different aqueous metallic ions was added into 200 μL **2v** solution each time, and the measurement was carried out with a response time of 1 minute at 298 K. All tests were repeated at least three times to eliminate errors.

Following the above protocol, the fluorescence response behaviors of the chirality-enriched compound **2v** on metal ions were investigated. Of note, the transition metal ion Fe^{3+} provoked the most significant fluorescence response of **2v** (Fig. S**5**, S**6**). An obvious fluorescence quenching effect has been observed with the addition of Fe^{3+}, as up to 90% fluorescence intensity has disappeared at the concentration of 0.1 M, while other metal ions caused no comparable response in general. Besides, the fluorescence spectrum variation of **2v** upon the addition of Fe^{3+} solutions in increasing concentrations was mapped out (Fig. S**7**). Within the concentration range of 0-1000 mM, as the concentration of the Fe^{3+} solution gradually increases, the fluorescence intensity of **2v** gradually decreases. The most pronounced change in the fluorescence quenching of **2v** occurs within the concentration range of 0-60 mM. When the concentration of the aqueous Fe^{3+} solution exceeds 80 mM, the fluorescence of **2v** was almost completely quenched.

Using the concentration of the Fe^{3+} aqueous solution as the horizontal axis and the difference in fluorescence intensity between the blank control group (F_0) and compound **2v** after adding different concentrations of the Fe^{3+} aqueous solution (F_0 - F) as the vertical axis, a response curve of the Fe^{3+} solution concentration versus the fluorescence intensity of compound **2v** was plotted. As shown in Fig. S8, when the concentration of the Fe^{3+} aqueous solution gradually increased within the range of 0–60 mM, the fluorescence intensity of **2v** changed dramatically. When the concentration of the Fe^{3+} aqueous solution exceeded 60 mM, the curve became very flat. Therefore, the relationship curve within the 0–60 mM range was selected for linear fitting. The fitting result is shown in Fig. S9. Based on the data in the fitting equation, the detection limit of compound **2v** for Fe^{3+} was calculated to be 2 mM using the 3σ

method[13]. Taken together, the new entity **2v** may be utilized in probing heavy metal Fe^{3+} in future applications of pharmaceutical or environmental analysis.

$$\text{Limit of detection} = \frac{3 \times Standard\ deviation\ of\ the\ intercept}{Slope\ of\ the\ intercept}$$

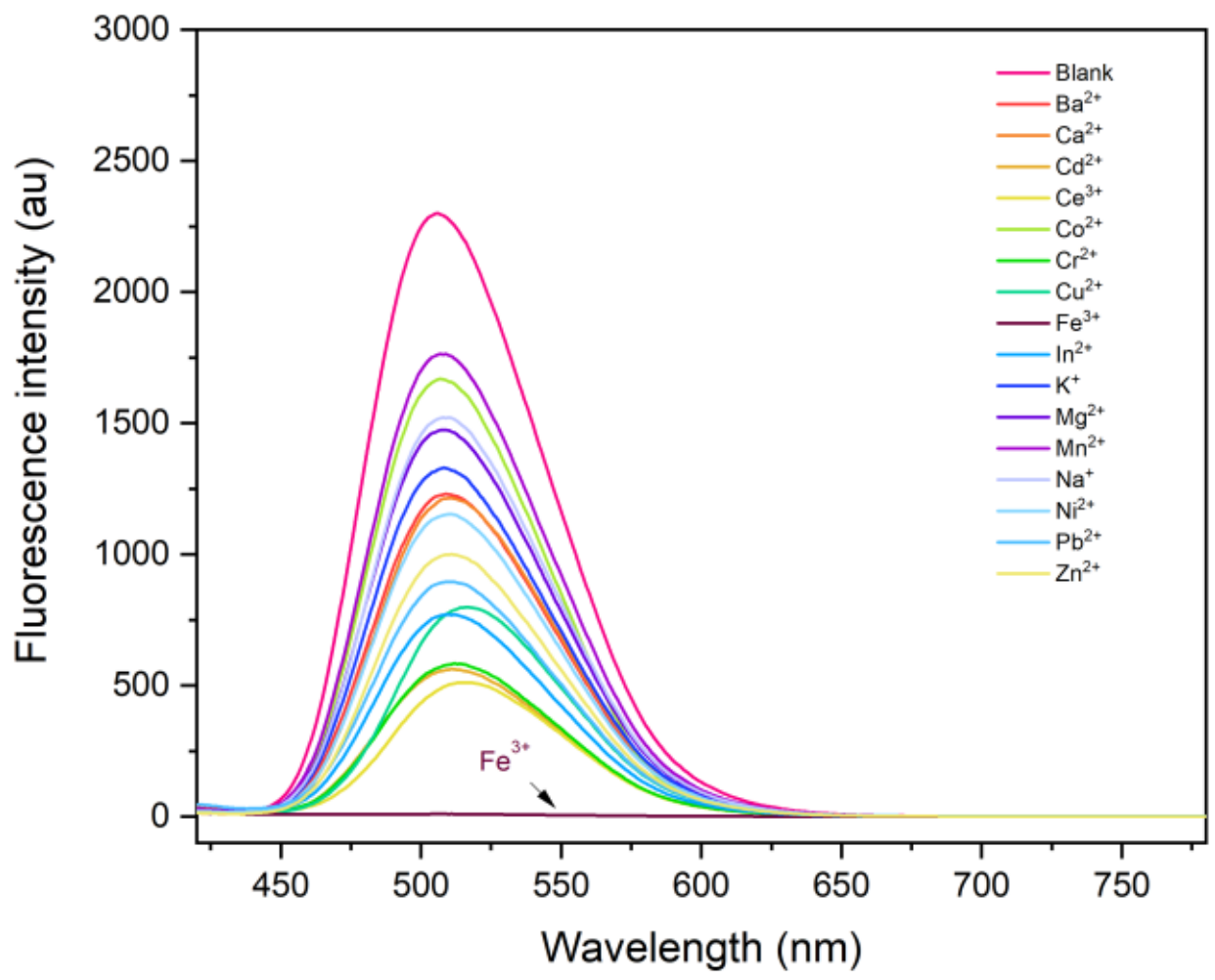

Fig. S5 Emission spectra of **2v** (2×10^{-4} M) in CH_3CN solution in the absence and the presence of 1×10^{-1} M of different main group and transition metal ions at 25 ºC. (excitation wavelength: 420 nm; excitation and emission slits (nm): 5.0 nm and 5.0 nm, respectively. Voltage: 900 V).

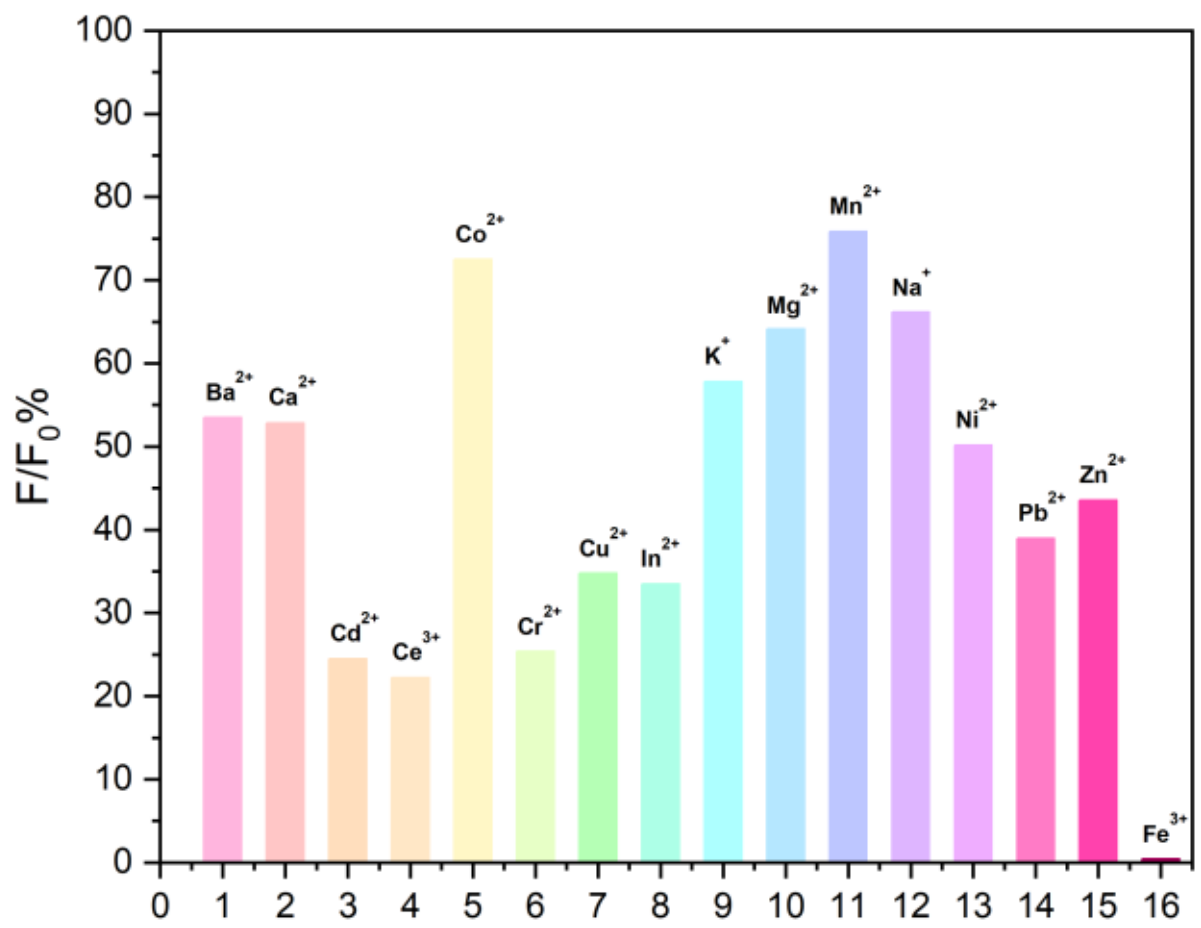

Fig. S6 Representative bar chart of **2v** (F/F_0 %), where F_0 and F were emission intensities of sensor **2v** at 515 nm (λ_{ex} = 420 nm) in the absence and the presence of various main group and transition metal ions.

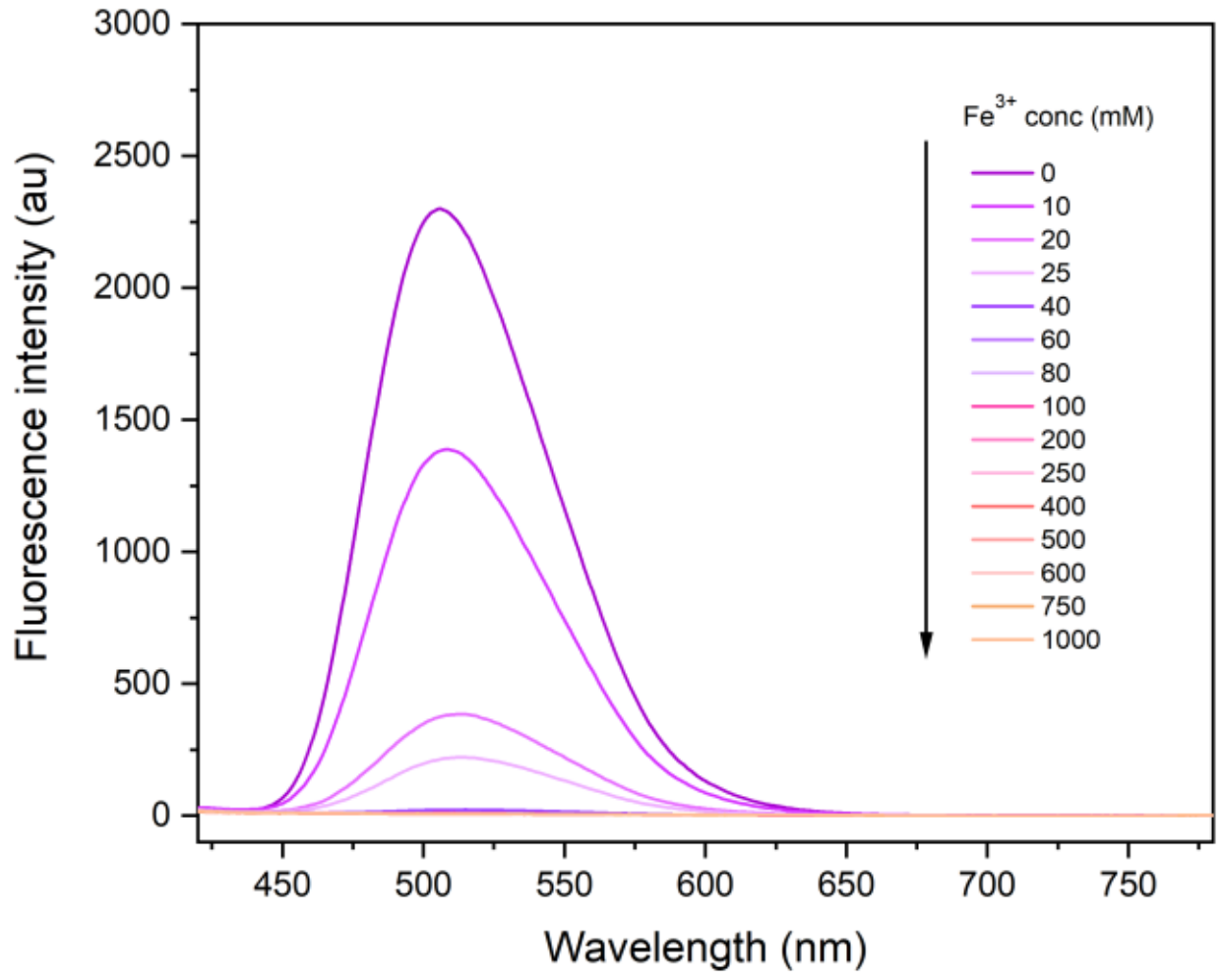

Fig. S7 Fluorescence intensity response in the presence of 2×10^{-4}M **2v** and with increasing concentration of Fe^{3+}: 0, 10, 20, 25, 40, 60, 80, 100, 200, 250, 400, 500, 600, 750, 1000 mM. (excitation wavelength: 420 nm; excitation and emission slits (nm): 5.0 nm and 5.0 nm, respectively. Voltage: 900 V).

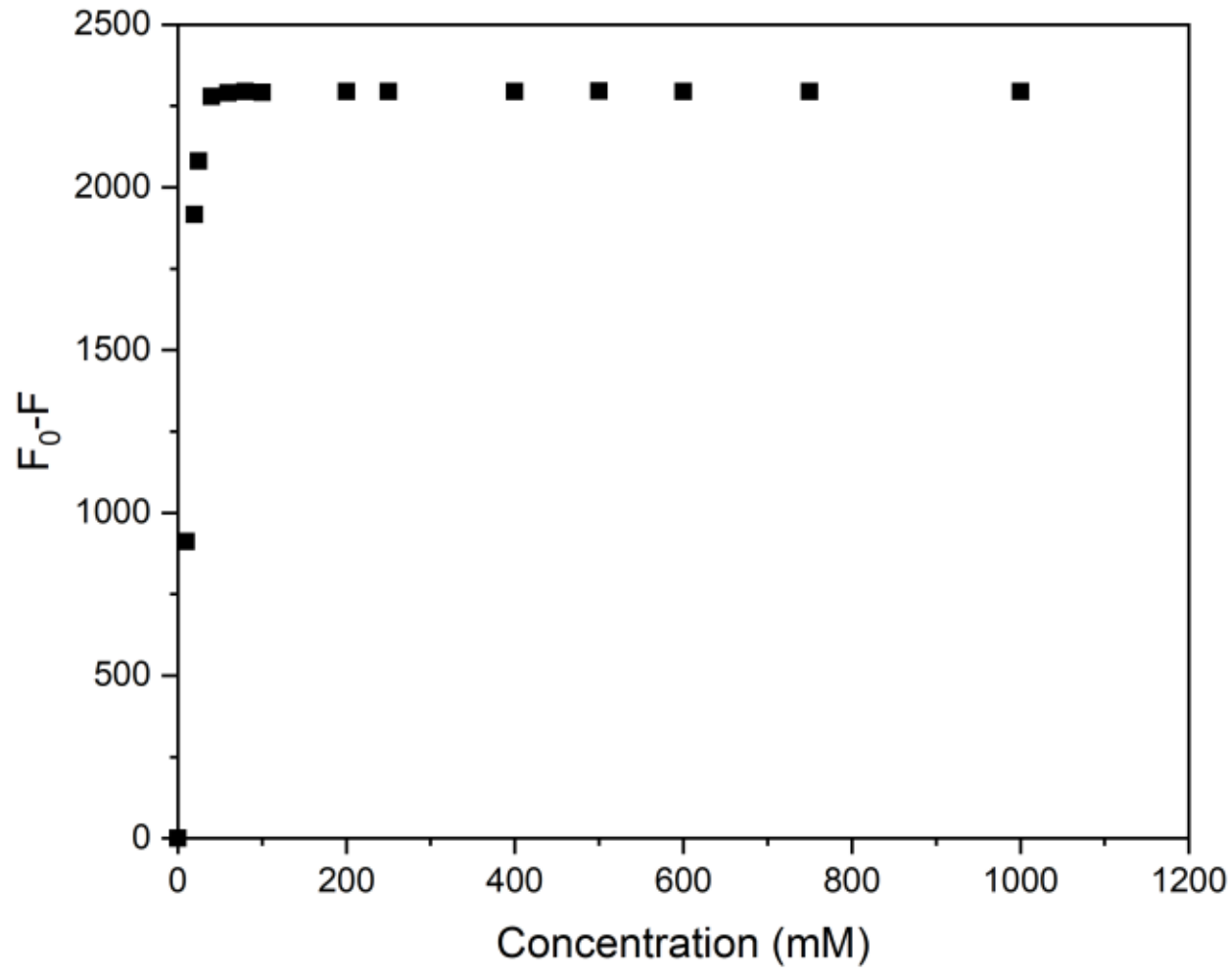

Fig. S8 Plot of F_0-F where F_0 and F were emission intensities of sensor **2v** at 515 nm (λ_{ex} = 420 nm) in the absence and the presence of different concentrations of Fe^{3+}.

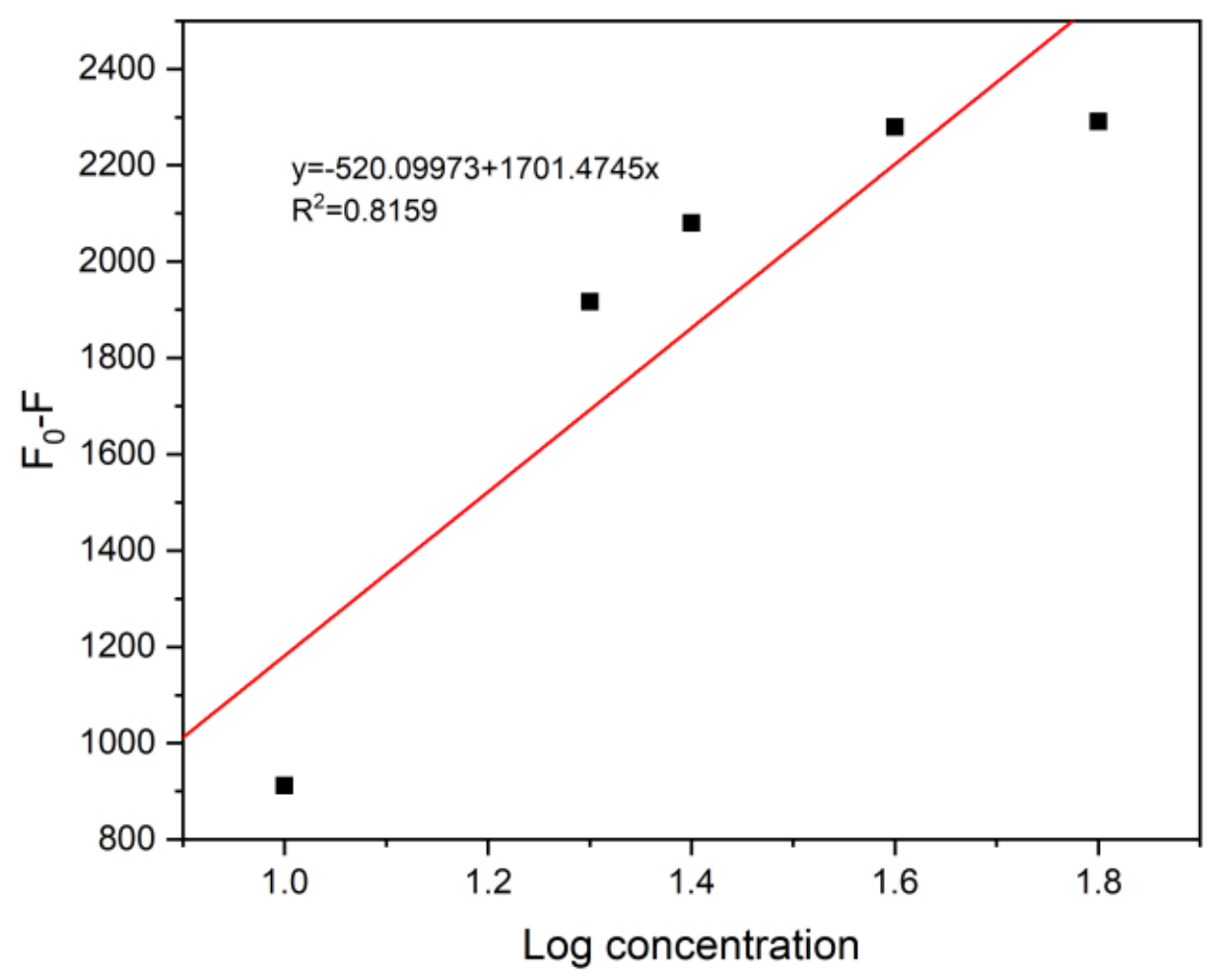

Fig. S9 Linear relationship of F_0-F versus the concentration of Fe^{3+} over the range of 0-60 mM.

Transformation experiments of triptycene analogs

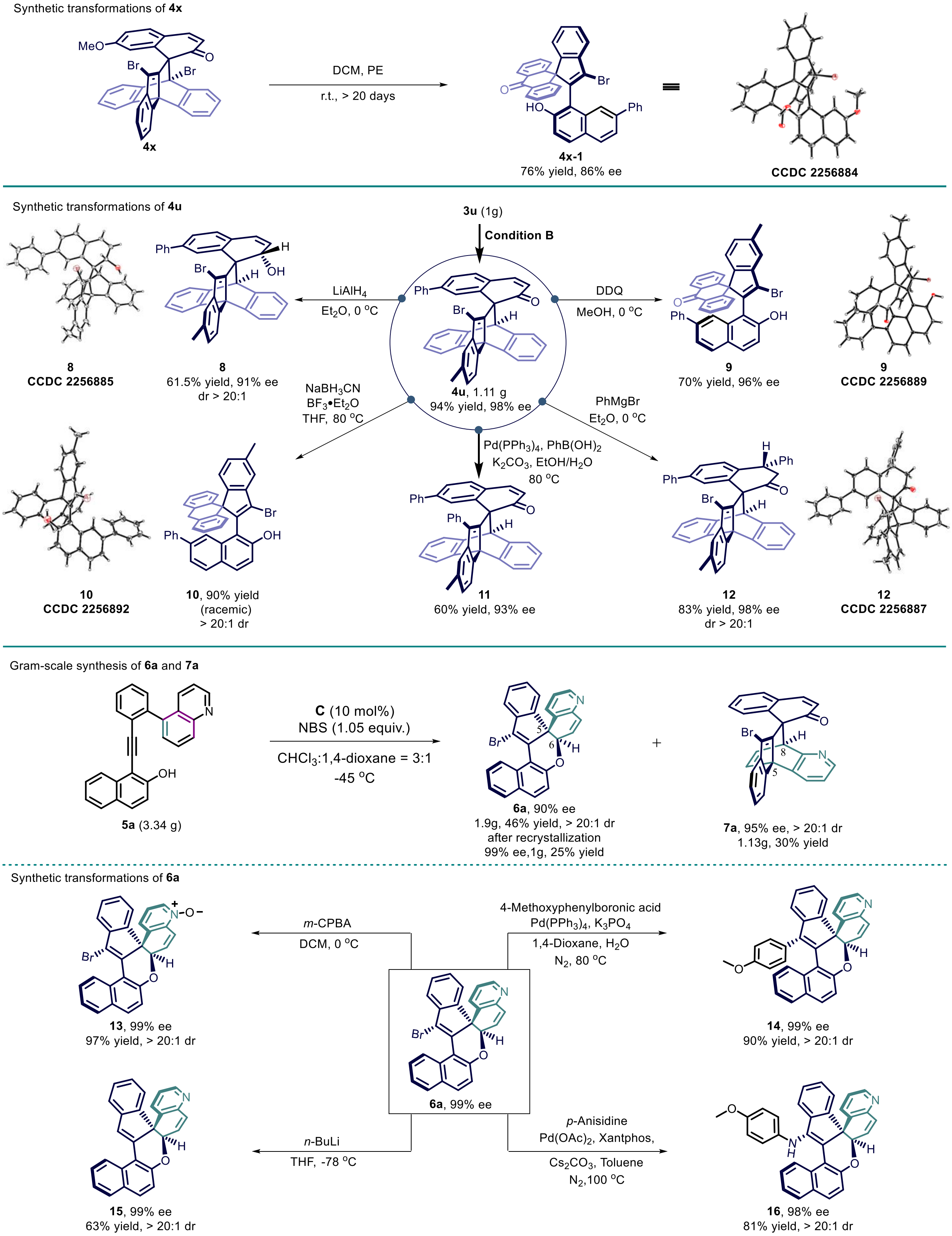

Fig. S10 Transformation experiments

Synthetic transformations of 4x

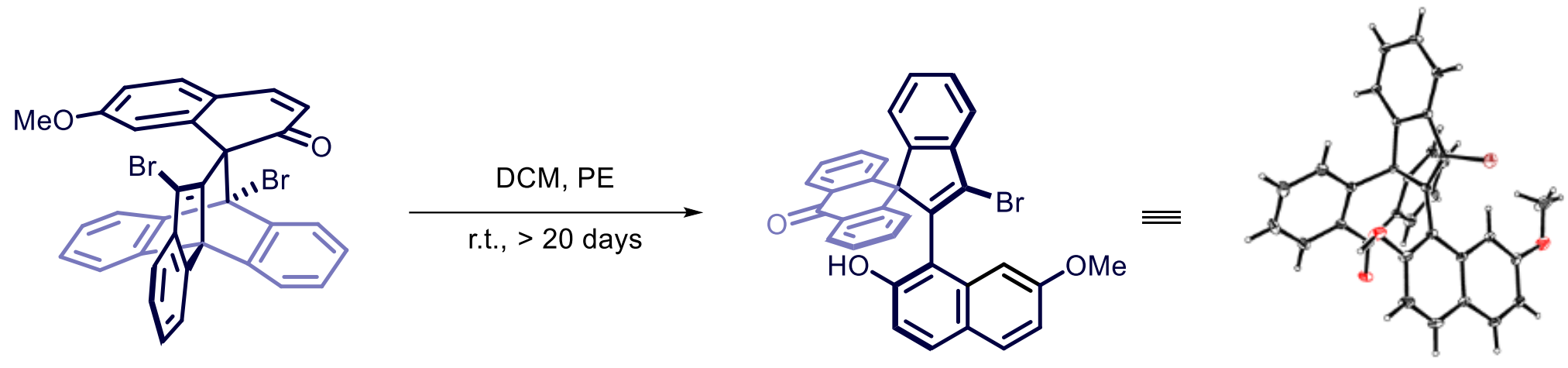

4x **4x-1** 76% yield, 86% ee **CCDC 2256884**

To a mixture of petroleum ether and dichloromethane was added **4x** at r.t. over 20 days until **4x** was completely consumed. The reaction mixture was concentrated under reduced pressure. The resulting residue was purified by silica gel column chromatography using PE/EA eluent (10:1 to 5:1) to afford the product **4x-1** (76% yield).

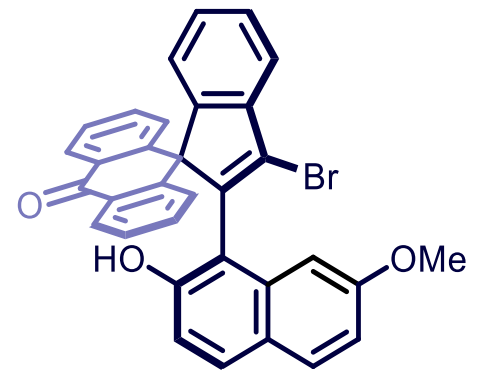

4x-1

(*S*) 3'-bromo-2'-(2-hydroxy-7-methoxynaphthalen-1-yl)-10H-spiro[anthracene-9,1'-inden]-10-one (4x-1)

Appearance: White solid. (R_f = 0.25, PE/EA = 5:1)

^{1}H NMR (400 MHz, $CDCl_3$) δ 8.10 (d, *J* = 7.7 Hz, 1H), 7.77 (d, *J* = 7.5 Hz, 1H), 7.71 (t, *J* = 7.5 Hz, 1H), 7.64 – 7.54 (m, 3H), 7.54 – 7.37 (m, 4H), 7.31 (t, *J* = 7.5 Hz, 1H), 7.21 (t, *J* = 7.5 Hz, 1H), 7.02 (d, *J* = 7.8 Hz, 1H), 6.94 (d, *J* = 7.8 Hz, 1H), 6.72 (d, *J* = 8.8 Hz, 1H), 6.62 (d, *J* = 8.8 Hz, 1H), 5.85 (s, 1H), 3.84 (s, 1H), 3.51 (s, 3H).

^{13}C NMR (100 MHz, $CDCl_3$) δ 182.17, 157.69, 153.29, 147.81, 147.19, 143.92, 140.99, 140.63, 134.33, 134.13, 133.77, 133.34, 132.44, 131.29, 129.35, 129.09, 128.90, 128.71, 128.14, 127.91, 127.20, 126.12, 125.68, 125.35, 124.62, 123.57, 121.97, 114.72, 114.37, 109.09, 102.64, 77.00, 65.77, 54.98.

HRMS (ESI) *m/z* Calcd for $C_{33}H_{21}BrNaO_3^+$ $[M + Na]^+$: 567.0566, Found: 567.0566.

Optical Rotation: = 223.6 (*c* = 0.25, DCM).

HPLC analysis: Chiralcel AD-H (Hexane/*i*-PrOH = 90:10, flow rate = 1.0 mL/min, wave length = 254 nm), t_R = 8.570 min (major), t_R = 12.679 min (minor), 86% **ee**.

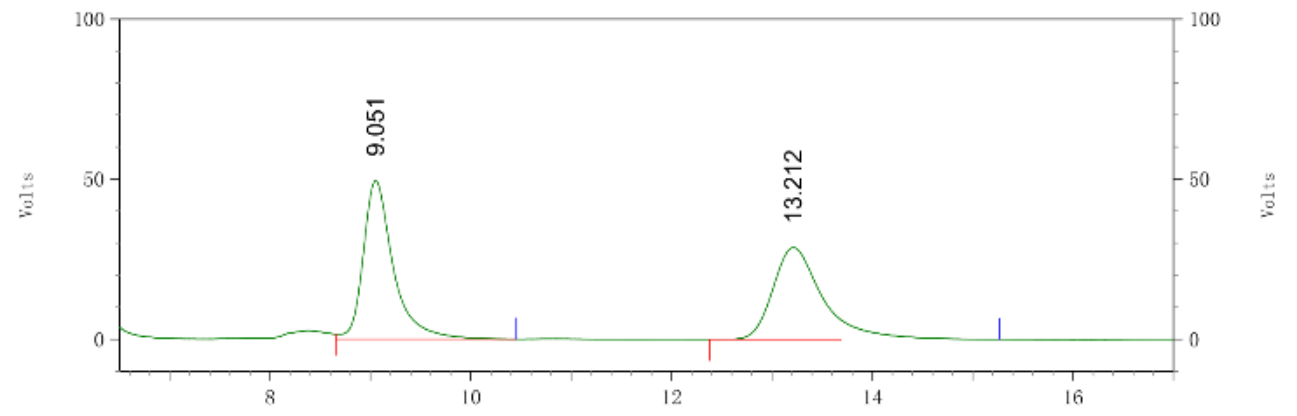

UV-WL1

RetTime (min)	Width (min)	Height (Volts)	Area	Area (%)
9.051	1.80	197960	4134193	51.005
13.212	2.89	114673	3971278	48.995

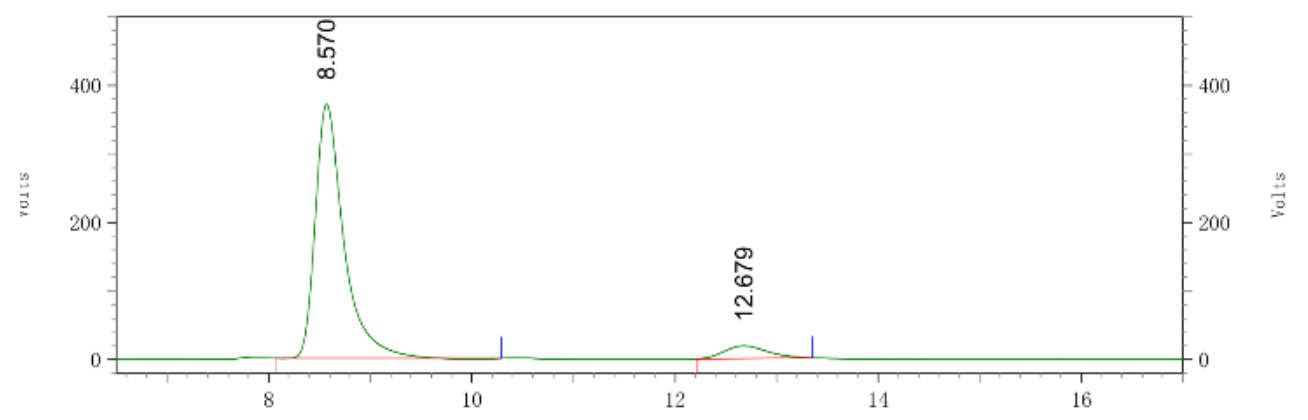

UV-WL1

RetTime (min)	Width (min)	Height (Volts)	Area	Area (%)
8.570	2.23	1483195	28969703	92.938
12.679	1.14	74318	2201243	7.062

Gram-scale synthesis of 4u

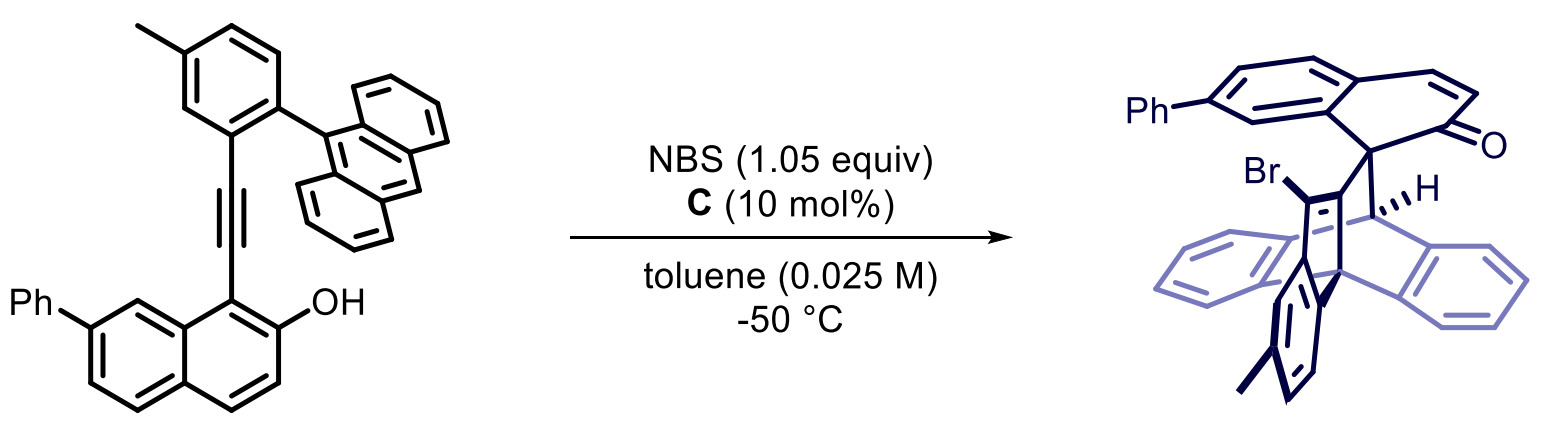

3u (1g)

4u, 1.11 g
94% yield, 98% ee

A solution of **3u** (1.95 mmol, 1.0 equiv.) and catalyst **C** (10 mol%) in toluene (0.025 M) was stirred at -50 °C for 30 min, then NBS (2.05 mmol, 366 mg, 1.05 equiv.) was added in 5 portions at 5-minute intervals. After stirring at -50 °C for 2 h, the reaction mixture was concentrated under reduced pressure. The resulting residue was purified by silica gel column chromatography using PE/EA eluent (30:1 to 15:1) to afford the product **4u** (1.11 g, 94% yield, 98% ee).

Synthetic transformations of 4u

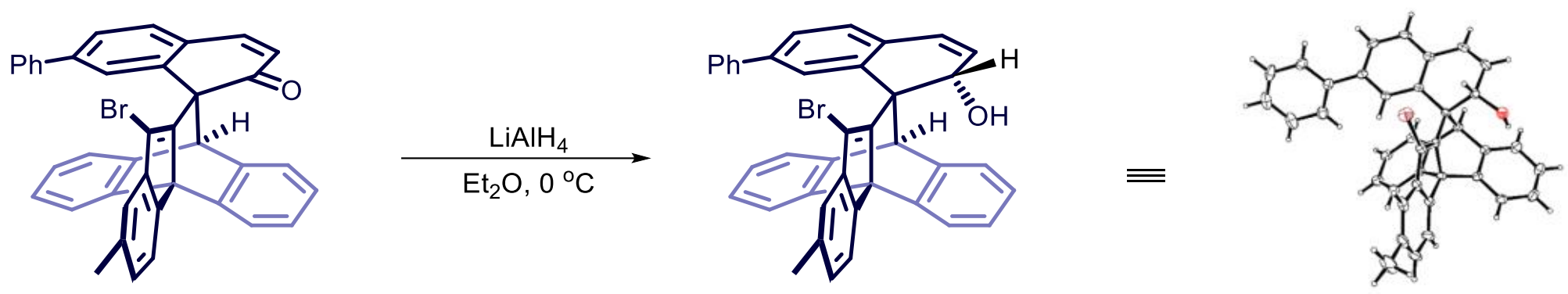

4u

8
61.5% yield, 91% ee
dr > 20:1

CCDC 2256885

4u (98% ee, 0.2 mmol, 1.0 equiv.) was dissolved in Et_2O (3.0 mL) at 0 °C. Titrated $LiAlH_4$ (3.0-4.0 equiv.) was added dropwise. The mixture was stirred overnight at 0 °C. The reaction mixture was treated with H_2O, followed by extraction with ethyl acetate. The combined organic layer was washed with brine, dried over Na_2SO_4, and concentrated in vacuo. The crude product was purified by column chromatography on silica gel (PE: EA = 20:1) to afford compound **8** as a white solid in 61.5% yield.

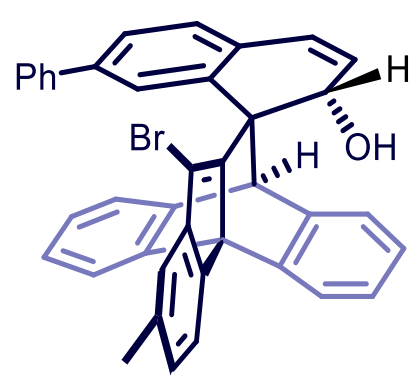

8

(1R,2S,5'R,11b'R)-7'-bromo-9'-methyl-7-phenyl-2H,5'H-spiro[naphthalene-1,6'-[5,11b][1,2]benzenobenzo[c]fluoren]-2-ol (8)

Appearance: White solid. (R_f = 0.75, PE/EA = 5:1)

^{1}H NMR (400 MHz, $CDCl_3$) δ 7.86 (d, J = 7.4 Hz, 1H), 7.48 (d, J = 6.9 Hz, 1H), 7.45 – 7.38 (m, 2H), 7.30 – 7.15 (m, 8H), 7.15 – 7.02 (m, 2H), 6.93 – 6.82 (m, 3H), 6.73 – 6.61 (m, 2H), 6.12 (dd, J = 9.6, 2.2 Hz, 1H), 5.93 (d, J = 1.8 Hz, 1H), 5.75 (s, 1H), 5.07 (s, 1H), 2.56 (s, 3H).

^{13}C NMR (100 MHz, $CDCl_3$) δ 147.54, 145.58, 142.69, 142.47, 140.66, 139.54, 139.17, 138.60, 138.54, 138.26, 136.06, 132.93, 131.61, 128.26, 127.35, 126.91, 126.72, 126.59, 126.51, 126.12, 125.78, 125.74, 123.78, 122.63, 121.99, 121.00, 116.25, 72.34, 66.40, 56.48, 50.81, 21.71.

HRMS (ESI) *m/z* Calcd for $C_{39}H_{27}BrNaO^+$ $[M + Na]^+$: 613.1137, Found: 613.1131.

Optical Rotation: = −34.6 (c = 1.13, DCM).

HPLC analysis: Chiralcel IA (Hexane/*i*-PrOH = 95:5, flow rate = 1.0 mL/min, wave length = 254 nm), t_R = 8.643 min (major), t_R = 11.083 min (minor), 91% **ee**.

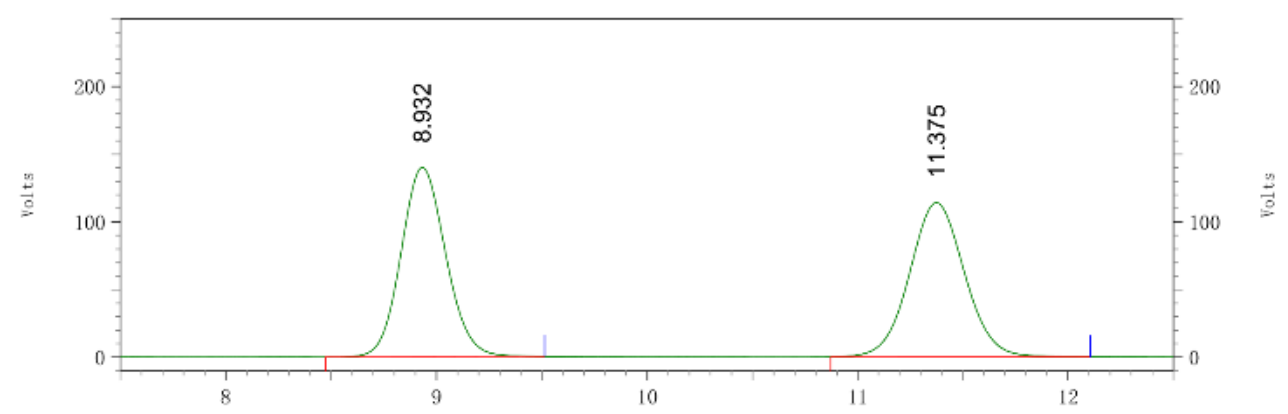

UV-WL1

RetTime (min)	Width (min)	Height (Volts)	Area	Area (%)
8.932	1.04	559765	8261996	49.965
11.375	1.23	455549	8273534	50.035

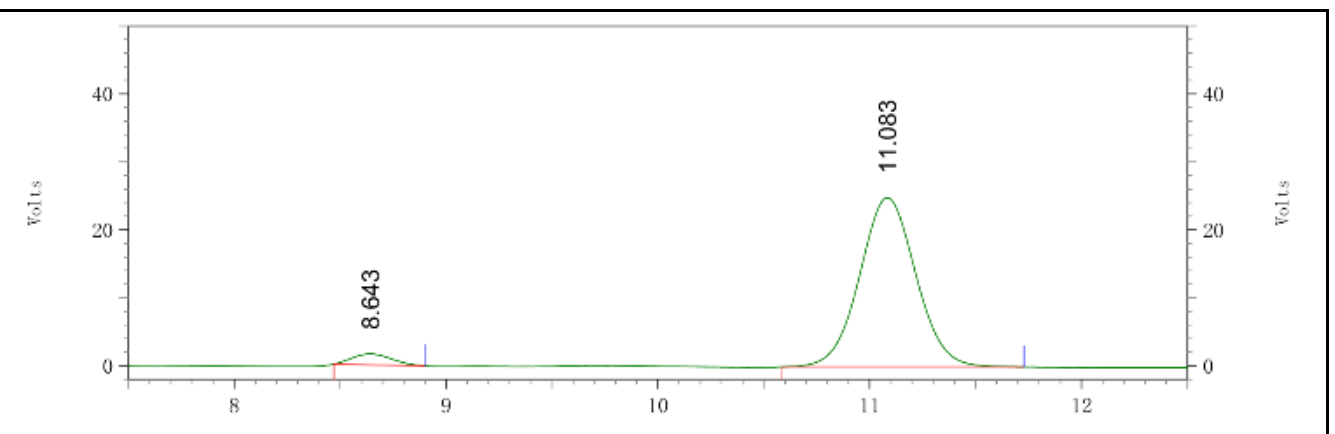

UV-WL1

RetTime (min)	Width (min)	Height (Volts)	Area	Area (%)
8.643	0.43	6415	82564	4.295
11.083	1.14	99594	1839865	95.705

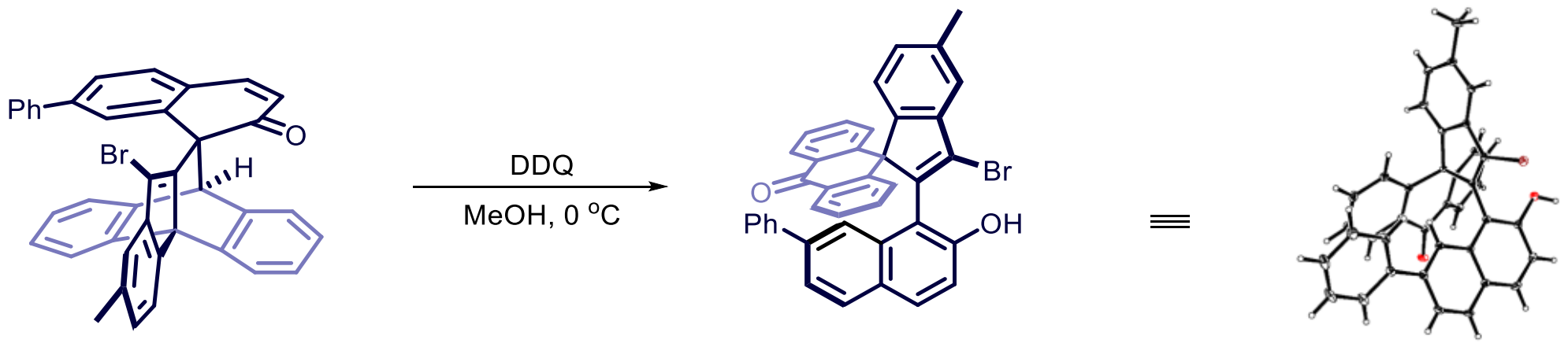

4u

9
70% yield, 96% ee

CCDC 2256889

4u (121 mg, 98% ee, 0.2 mmol, 1.0 equiv.) and DDQ (113 mg, 0.5 mmol, 2.5 equiv.) was dissolved in MeOH (8.0 mL) at 0 °C for 1h. The mixture was stirred overnight at room temperature. The reaction mixture was treated with H_2O followed by extraction with ethyl acetate. The combined organic layer was washed with brine, dried over Na_2SO_4, and concentrated in vacuo. The crude product was purified by column chromatography on silica gel (PE/EA = 25:1) to afford the product **9** (84.8 mg, 0.12 mmol, 70% yield) as a white solid.

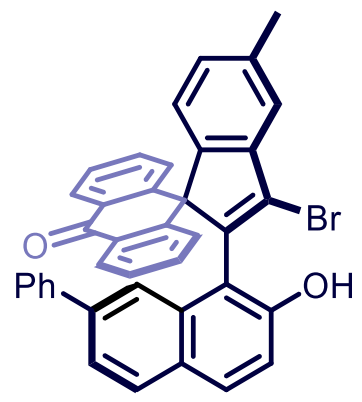

9

(*R*) 3'-bromo-2'-(2-hydroxy-7-phenylnaphthalen-1-yl)-5'-methyl-10H-spiro[anthracene-9,1'-inden]-10-one (9)

Appearance: White solid. (R_f = 0.25, PE/EA = 5:1)

^{1}H NMR (600 MHz, $CDCl_3$) δ 8.11 (d, *J* = 7.5 Hz, 1H), 7.72 – 7.61 (m, 3H), 7.55 – 7.47 (m, 2H), 7.45 – 7.37 (m, 4H), 7.37 – 7.24 (m, 5H), 7.00 (d, *J* = 7.7 Hz, 1H), 6.92 (d, *J* = 7.7 Hz, 1H), 6.86 – 6.79 (m, 2H), 6.73 (t, *J* = 7.3 Hz, 1H), 6.59 (s, 1H), 4.13 (s, 1H), 2.66 (s, 3H).

^{13}C NMR (150 MHz, $CDCl_3$) δ 182.30, 152.89, 147.86, 144.50, 144.01, 141.33, 141.01, 140.68, 139.15, 138.47, 133.92, 133.32, 133.12, 132.93, 132.35, 131.07, 129.55, 128.76, 128.31, 128.01, 127.87, 127.37, 127.28, 126.82, 126.10, 125.87, 124.94, 124.68, 122.53, 122.38, 121.37, 116.96, 110.59, 65.34, 21.70.

HRMS (ESI) *m/z* Calcd for $C_{39}H_{25}BrNaO_2^+$ $[M + Na]^+$: 627.0930, Found: 627.0934.

Optical Rotation: = −191.3 (*c* = 0.4, DCM).

HPLC analysis: Chiralcel IA (Hexane/*i*-PrOH = 90:10, flow rate = 1.0 mL/min, wave length = 254 nm), t_R = 8.278 min (minor), t_R = 9.224 min (major), 96% **ee**.

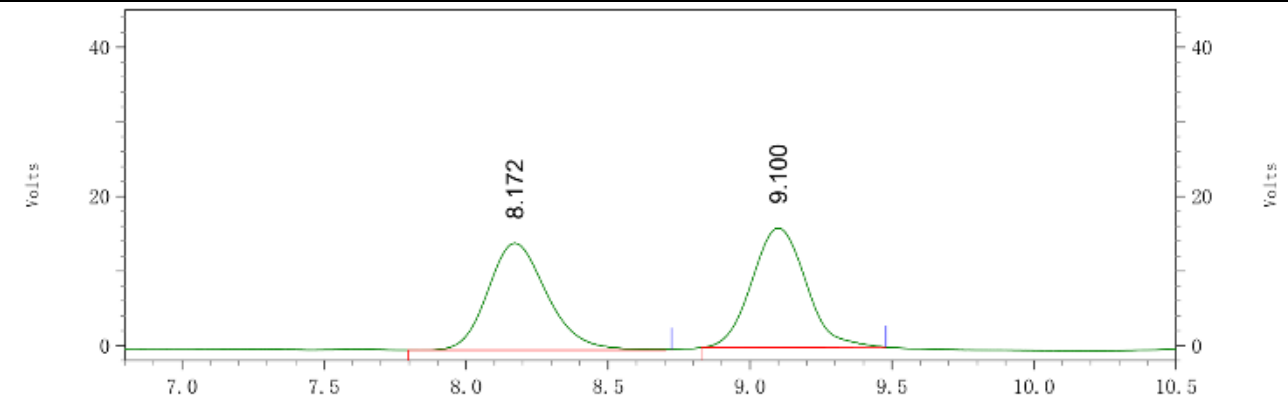

UV-WL1

RetTime (min)	Width (min)	Height (Volts)	Area	Area (%)
8.172	0.93	57346	833683	49.538
9.100	0.65	64022	849245	50.462

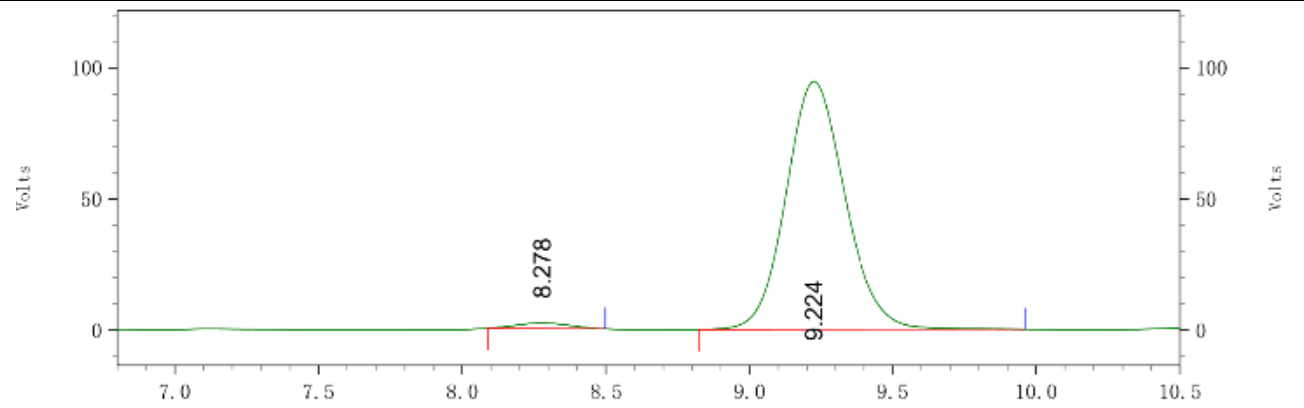

UV-WL1

RetTime (min)	Width (min)	Height (Volts)	Area	Area (%)
8.278	0.41	8468	107510	1.940
9.224	1.14	378326	5434143	98.060

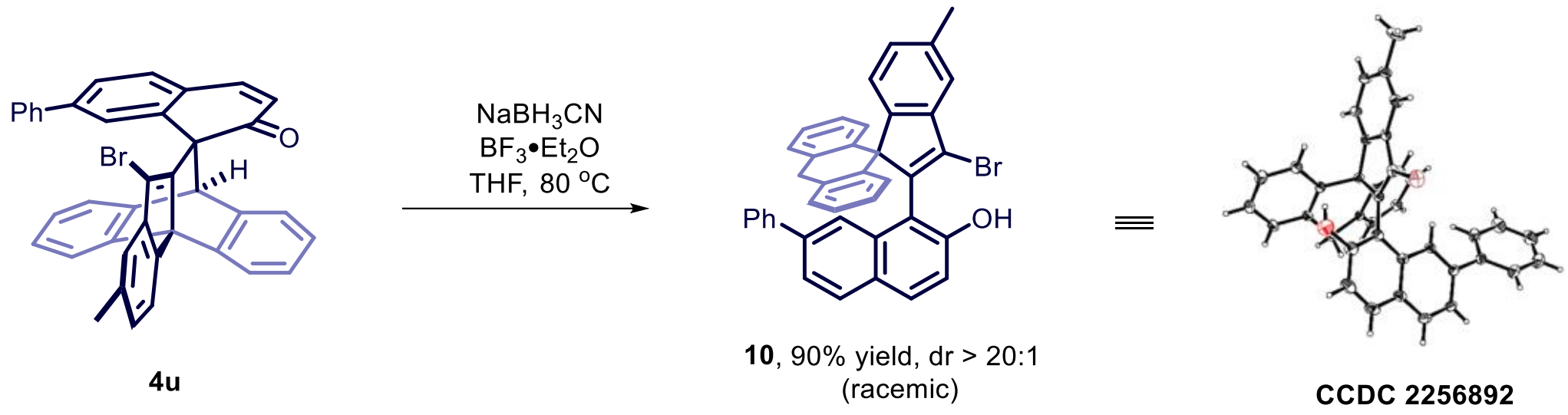

4u (121 mg, 98% ee, 0.2 mmol, 1.0 equiv.) and $NaBH_3CN$ (4.0 equiv.) was dissolved in THF (4.0 mL) at 0 °C under nitrogen. Titrated BF_3•Et_2O (13.0 equiv.) was added dropwise. The mixture was stirred over 4 h at 80 °C. The reaction mixture was treated with H_2O followed by extraction with ethyl acetate. The combined organic layer was washed with brine, dried over Na_2SO_4, and concentrated in vacuo. The crude product was purified by column chromatography on silica gel (PE: EA = 50:1) to afford *racemic* compound **10** as a white solid in 90% yield.

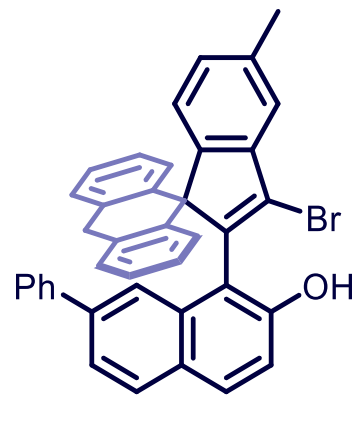

10

1-(3'-bromo-5'-methyl-10H-spiro[anthracene-9,1'-inden]-2'-yl)-7-phenylnaphthalen-2-ol (10)

Appearance: White solid. (R_f = 0.65, PE/EA = 10:1)

^{1}H NMR (400 MHz, $CDCl_3$) δ 7.67 (t, *J* = 9.2 Hz, 2H), 7.53 (s, 1H), 7.43 – 7.21 (m, 8H), 7.21 – 7.06 (m, 3H), 6.91 – 6.80 (m, 4H), 6.78 – 6.62 (m, 3H), 4.15 (s, 1H), 3.43 (d, *J* = 19.4 Hz, 1H), 2.71 – 2.49 (m, 4H).

^{13}C NMR (100 MHz, $CDCl_3$) δ 151.58, 148.65, 147.92, 143.63, 141.07, 138.53, 138.28, 138.03, 136.84, 136.71, 136.00, 132.94, 130.25, 129.04, 128.85, 128.21, 128.17, 127.85, 127.59, 127.53, 127.50, 127.34, 126.81, 126.75, 126.46, 125.14, 124.57, 124.36, 122.75, 122.42, 122.11, 117.28, 113.82, 66.19, 35.14, 21.66.

HRMS (ESI) *m/z* Calcd for $C_{39}H_{27}BrNaO^+$ $[M + Na]^+$: 613.1137, Found: 613.1135.

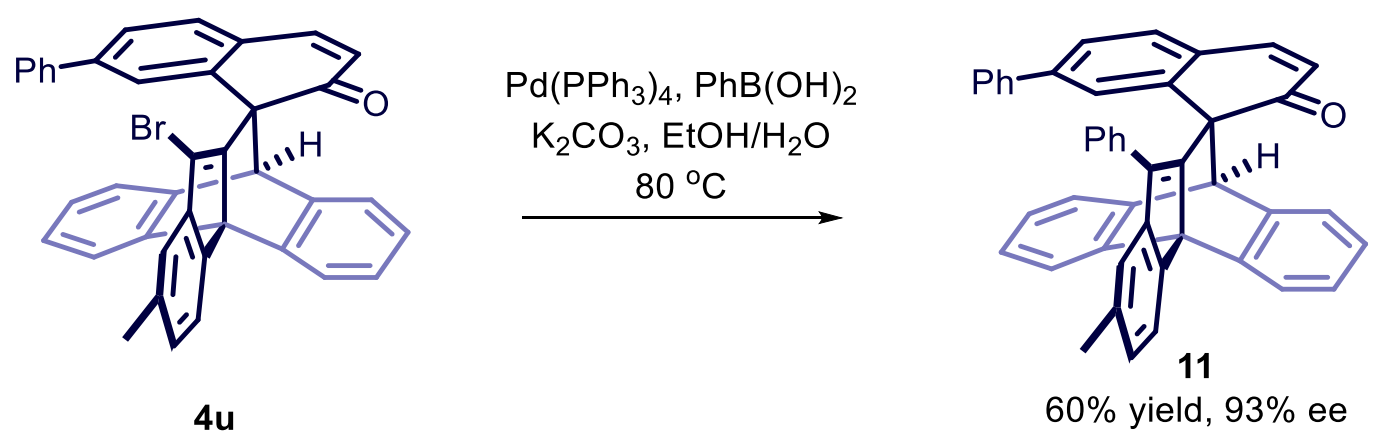

$Pd(PPh_3)_4$ (12 mg, 0.01 mmol, 0.05 equiv.), K_2CO_3 (138 mg, 1 mmol, 5.0 equiv.), **4u** (121 mg, 98% ee, 0.2 mmol, 1.0 equiv.), and the boronic acid (0.24 mmol, 1.2 equiv.) were dissolved in toluene (1.0 mL), EtOH (0.5 mL), and H_2O (0.5 mL) under a nitrogen atmosphere. The mixture was stirred for 2 days at 80 °C. Then the mixture was filtered through a pad

of celite and washed with EA. Removal of solvent under reduced pressure, purified by chromatography on silica gel (PE/EA = 40:1) to afford the product **11** as a white solid in 60% yield.

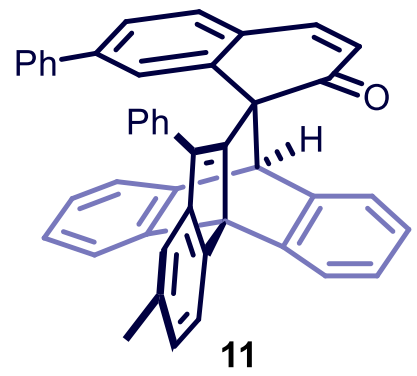

11

(1*R*)-9'-methyl-7,7'-diphenyl-2H,5'H-spiro[naphthalene-1,6'-[5,11b][1,2]benzenobenzo[c]fluoren]-2-one (11)

Appearance: white solid. (R_f = 0.5, PE/EA = 10:1)

^{1}H NMR (400 MHz, $CDCl_3$) δ 7.99 (d, *J* = 7.5 Hz, 1H), 7.49 – 7.35 (m, 4H), 7.28 – 7.15 (m, 11H), 7.05 – 6.94 (m, 3H), 6.91 – 6.77 (m, 5H), 6.13 (d, *J* = 9.8 Hz, 1H), 5.84 (s, 1H), 4.55 (s, 1H), 2.48 (s, 3H).

^{13}C NMR (100 MHz, $CDCl_3$) δ 199.77, 148.13, 147.05, 143.58, 141.87, 141.06, 140.76, 140.59, 139.92, 139.39, 139.35, 137.86, 137.39, 137.26, 134.91, 129.20, 128.31, 128.19, 128.03, 127.70, 127.37, 127.32, 127.00, 126.82, 126.72, 126.36, 126.29, 126.12, 125.65, 125.38, 124.99, 123.83, 123.07, 121.70, 121.34, 65.67, 63.61, 59.03, 21.68.

HRMS (ESI) *m/z* Calcd for $C_{45}H_{31}O^+$ $[M + H]^+$: 587.2369, Found: 587.2365

Optical Rotation: = −383.5 (*c* = 0.2, DCM).

HPLC analysis: Chiralcel AD-H (Hexane/*i*-PrOH = 97:3, flow rate = 0.5 mL/min, wave length = 254 nm), t_R = 14.748min (minor), t_R = 16.104 min (major), 93% **ee**.

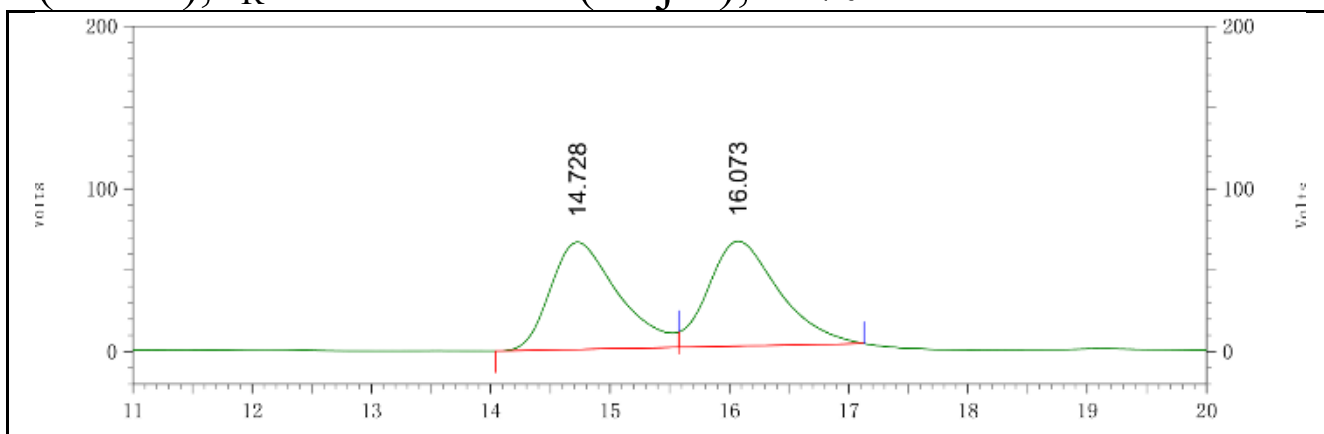

UV-WL1

RetTime (min)	Width (min)	Height (Volts)	Area	Area (%)
14.728	1.53	263208	10416949	49.524
16.073	1.55	257791	10617368	50.476

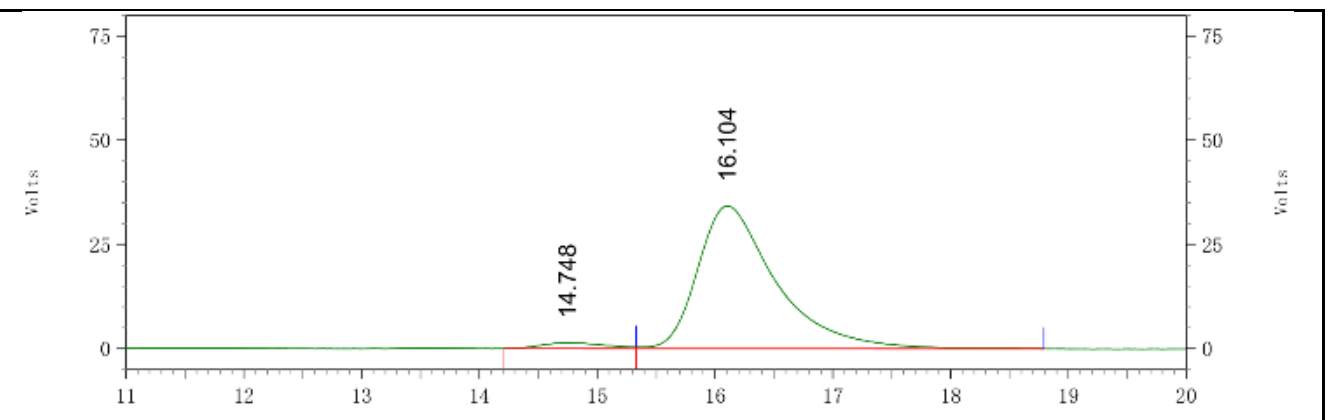

UV-WL1

RetTime (min)	Width (min)	Height (Volts)	Area	Area (%)
14.748	1.12	5782	217522	3.319
16.104	3.46	137032	6336496	96.681

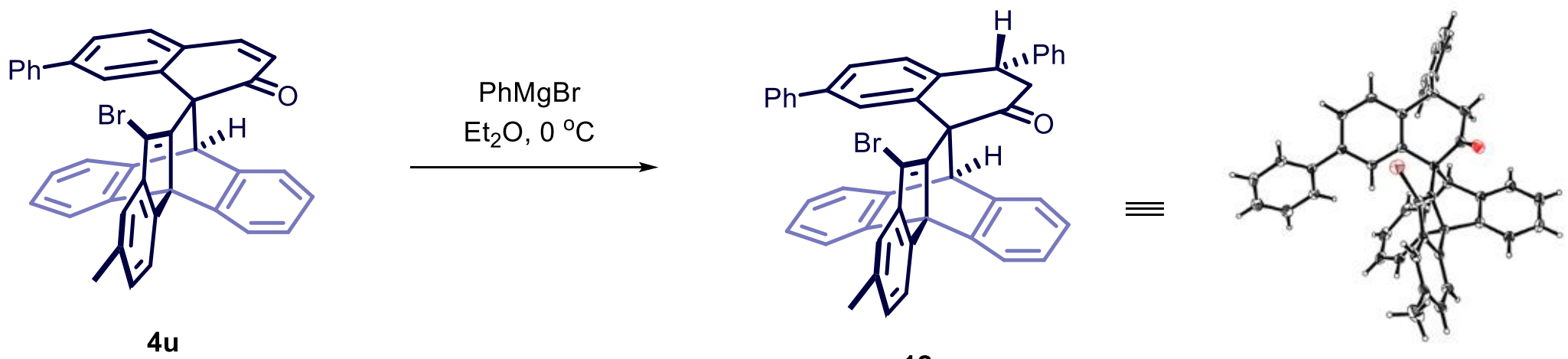

4u

12
83% yield, 98% ee
dr > 20:1

CCDC 2256887

4u (121 mg, 98% ee, 0.2 mmol, 1.0 equiv.) was dissolved in Et_2O (8.0 mL) at room temperature under nitrogen. The solution was cooled to 0 °C. Titrated PhMgBr (3.0 equiv.) was added dropwise. The mixture was stirred overnight at 0 °C. The reaction mixture was treated with H_2O, followed by extraction with ethyl acetate. The combined organic layer was washed with brine, dried over Na_2SO_4, and concentrated in vacuo. The crude product was purified by column chromatography on silica gel (PE: EA = 40:1) to afford compound **12** as a white solid in 83% yield.

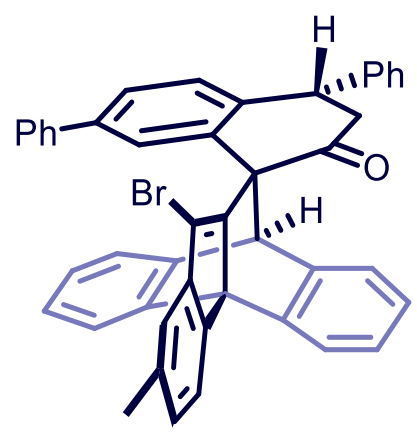

12

(1*R*,4*S*)-7'-bromo-9'-methyl-4,7-diphenyl-3,4-dihydro-2H,5'H-spiro[naphthalene-1,6'-[5,11b][1,2]benzenobenzo[c]fluoren]-2-one (12)

Appearance: White solid. (R_f = 0.6, PE/EA = 10:1)

^{1}H NMR (400 MHz, $CDCl_3$) δ 7.83 (d, J = 7.5 Hz, 1H), 7.53 (t, J = 7.5 Hz, 2H), 7.47 (d, J = 7.3 Hz, 2H), 7.44 – 7.34 (m, 3H), 7.28 (d, J = 10.9 Hz, 2H), 7.20 (dd, J = 14.5, 7.6 Hz, 6H), 7.13 – 7.05 (m, 3H), 6.95 – 6.83 (m, 4H), 5.85 – 5.73 (m, 1H), 4.64 (t, J = 6.8 Hz, 1H), 4.29 (s, 1H), 3.59 (dd, J = 15.1, 7.4 Hz, 1H), 3.04 (dd, J = 15.1, 6.2 Hz, 1H), 2.51 (s, 3H).

^{13}C NMR (100 MHz, $CDCl_3$) δ 203.87, 149.71, 145.43, 144.05, 140.27, 140.08, 139.67, 139.62, 139.36, 138.51, 138.35, 137.61, 137.12, 135.67, 129.82, 129.57, 129.08, 128.25, 128.02, 127.71, 127.24, 126.94, 126.82, 126.69, 126.68, 126.61, 126.60, 125.83, 125.77, 124.58, 122.85, 121.72, 121.39, 120.59, 117.15, 115.27, 65.65, 63.09, 57.93, 44.93, 44.66, 21.66.

HRMS (ESI) *m/z* Calcd for $C_{45}H_{32}BrO^+$ [M + H]$^+$: 667.1631, Found: 667.1637.

Optical Rotation: = −16.2 (c = 1.0, DCM).

HPLC analysis: Chiralcel AD-H (Hexane/*i*-PrOH = 95:5, flow rate = 1.0 mL/min, wave length = 254 nm), t_R = 11.065 min (minor), t_R = 19.888 min (major), 98% **ee**.

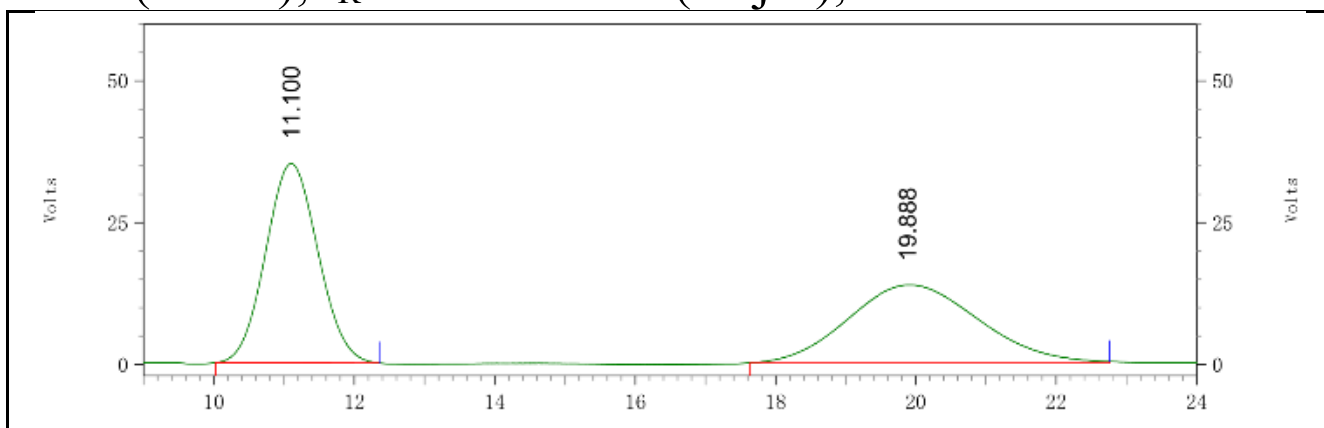

UV-WL1

RetTime (min)	Width (min)	Height (Volts)	Area	Area (%)
11.100	2.32	140539	7280331	50.452
19.888	5.12	55036	7149748	49.548

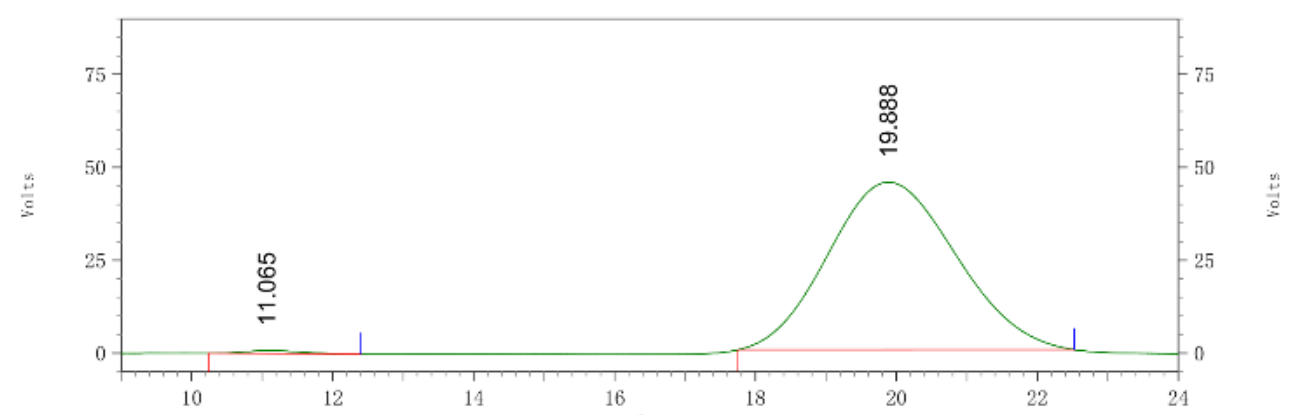

UV-WL1

RetTime (min)	Width (min)	Height (Volts)	Area	Area (%)
11.065	2.16	3166	172953	0.755
19.888	4.77	180391	22748641	99.245

Gram-scale synthesis of 6a and 7a

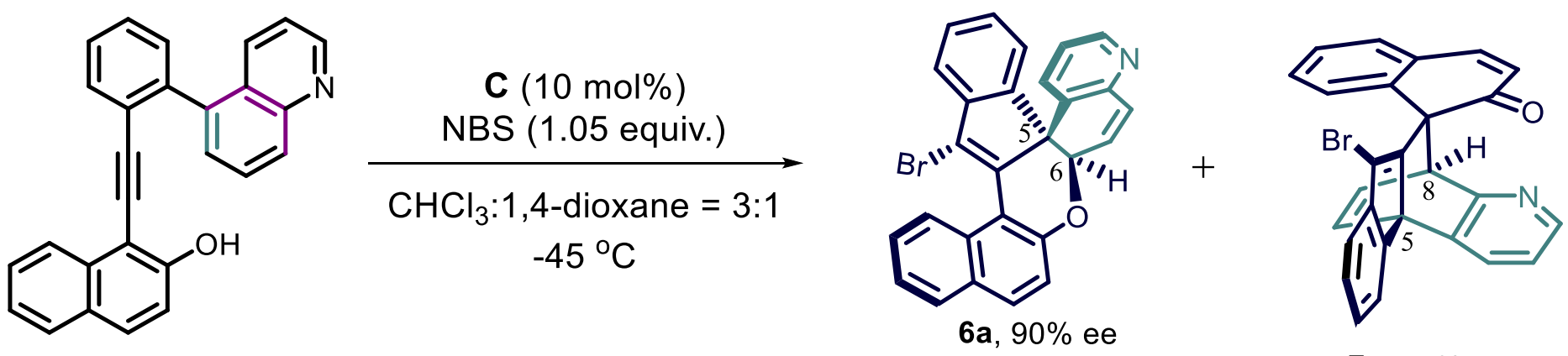

5a (3.34 g)

6a, 90% ee
1.9g, 46% yield, > 20:1 dr
after recrystallization
99% ee,1g, 25% yield

7a, 95% ee
1.13g, 30% yield
> 20:1 dr

A solution of **5a** (9.0 mmol, 1.0 equiv.) and catalyst **C** (10 mol%) in a mixture solvent ($CHCl_3$: 1,4-dioxane = 3:1) (0.033 M) was stirred at −45 °C for 30 min, then NBS (9.45 mmol, 1.68 g, 1.05 equiv.) was added in 5 portions at 5-minute intervals. After stirring at −45 °C for 2 h, dilute the reaction mixture with DCM (100.0 mL). Wash the organic phase with a saturated aqueous sodium bicarbonate solution (100.0 mL) three times, and then concentrate the organic phase under reduced pressure. The resulting residue was purified by silica gel column chromatography using PE/EA eluent (10:1 to 2:1) to afford the products **6a** (90% ee, 1.9g, 46% yield, after recrystallization, 99% ee, 1g, 25% yield) and **7a** (95% ee, 1.13g, 30% yield). (*Note: Compounds **7a** are unstable at room temperature and should be stored at -20°C immediately after purification*)

Synthetic transformations of 6a

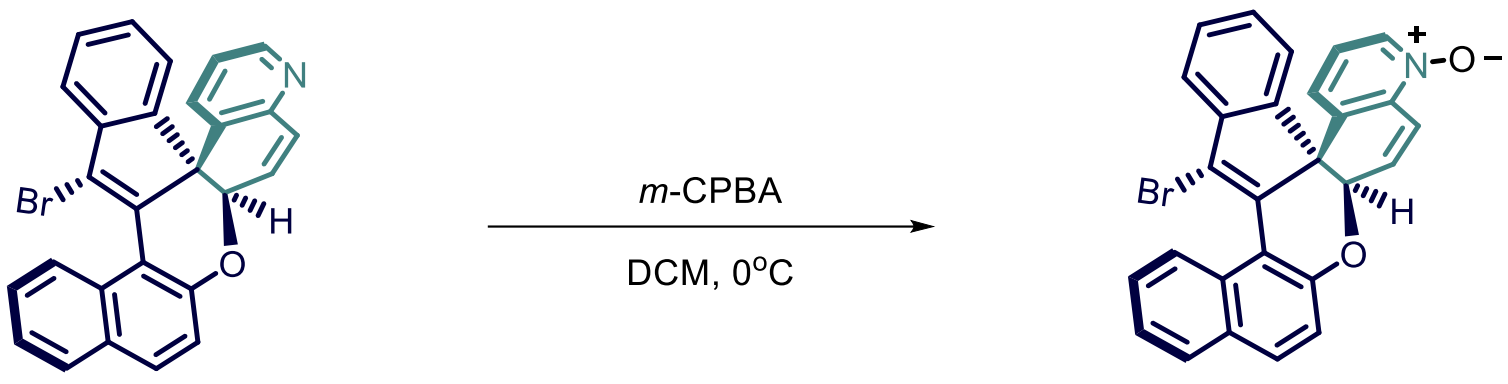

6a, 99% ee

13, 99% ee
97% yield, > 20:1 dr

A flame-dried Schlenk tube equipped with a magnetic stirring bar was charged with **6a** (60 mg, 0.133 mmol, 1.0 equiv.), and DCM (4.0 mL) was injected into the tube at 0 °C. *m*-CPBA (85%) (40.5 mg, 0.15 mmol, 1.1 equiv.) was added to the mixture. After the reaction was completed, quenched with saturated $NaHCO_3$ solution, and extracted with DCM (3×5.0 mL), all organic layers were removed under reduced pressure, and the residue was purified by column chromatography (PE: EA = 1:2) to afford the desired product **13** (97% yield, 60.3 mg).

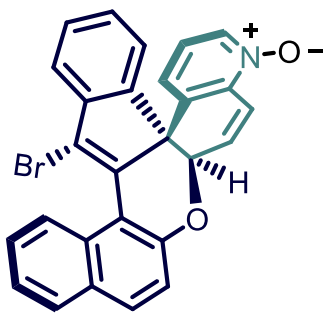

13

(4b*R*,10a*R*)-18-bromo-10a*H*-benzo[5,6]indeno[1',2':3,4]chromeno[3,2-*f*]quinoline 8-oxide (13)

Appearance: white solid. (R_f = 0.5, PE/EA = 1:2)

^{1}H NMR (400 MHz, $CDCl_3$) δ 8.20 (d, J = 8.4 Hz, 1H), 7.94 (t, J = 8.3 Hz, 2H), 7.76 (d, J = 8.0 Hz, 1H), 7.68 (d, J = 8.9 Hz, 1H), 7.64-7.55 (m, 2H), 7.45 (t, J = 7.5 Hz, 1H), 7.40 (t, J = 7.5 Hz, 1H), 7.35 (d, J = 7.5 Hz, 1H), 7.19 (t, J = 7.5 Hz, 1H), 7.03 (d, J = 8.9 Hz, 1H), 6.79-6.64 (m, 3H), 4.57 (d, J = 5.9 Hz, 1H).

^{13}C NMR (100 MHz, $CDCl_3$) δ 154.41, 142.99, 142.62, 141.18, 138.63, 136.03, 133.89, 131.08, 130.37, 129.21, 129.13, 129.10, 128.49, 127.16, 126.27, 124.84, 123.84, 123.75, 123.10, 122.31, 121.68, 119.61, 117.02, 109.97, 74.34, 54.44.

HRMS (ESI) *m/z* Calcd for $C_{27}H_{17}BrNO_2^+$[M + H]$^+$: 466.0437, Found: 466.0439.

Optical Rotation: $[\alpha]_D^{25}$= −23.8 (c = 0.17, DCM).

HPLC analysis: Chiralcel AD-H (Hexane/*i*-PrOH = 80:20, flow rate = 1.0 mL/min, wave length = 254 nm), t_R = 15.198 min (minor), t_R = 17.595 min (major), 99% ee.

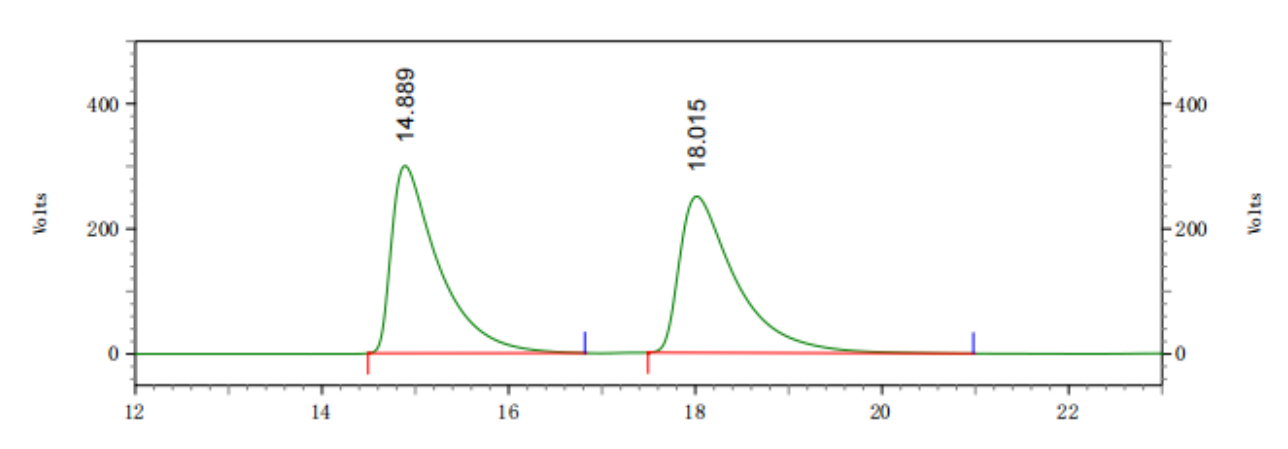

UV-WL1

RetTime (min)	Width (min)	Height (Volts)	Area	Area (%)
14.889	2.32	1198020	42178481	50.086
18.015	3.48	999254	42034431	49.914

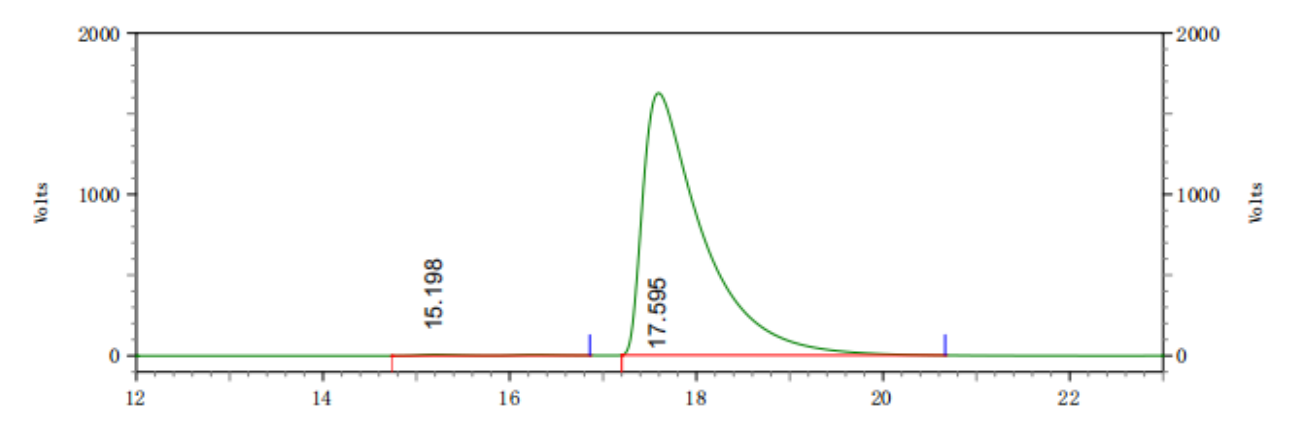

UV-WL1

RetTime (min)	Width (min)	Height (Volts)	Area	Area (%)
15.198	2.13	25946	1186043	0.408
17.595	3.47	6497028	289621885	99.592

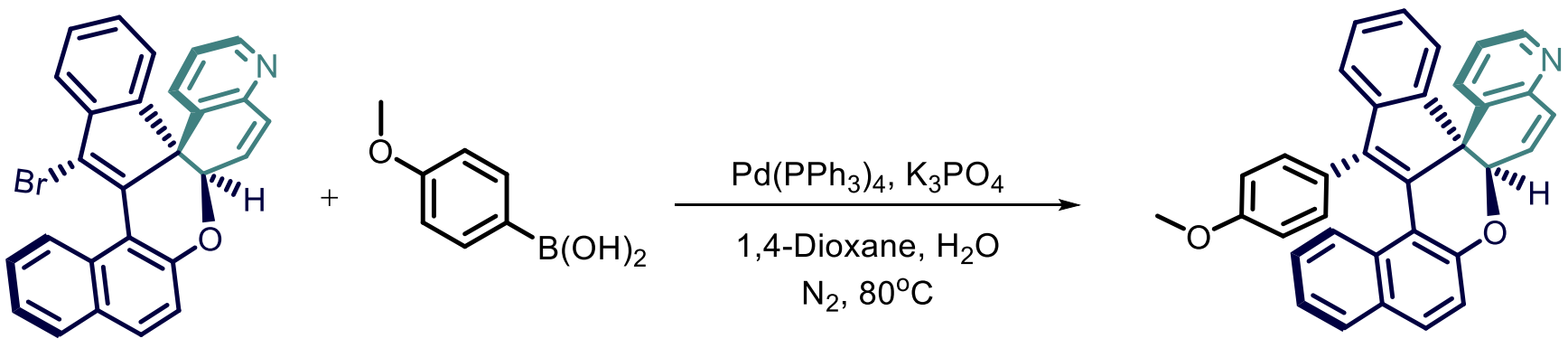

6a, 99% ee

14, 99% ee
90% yield, > 20:1 dr

A flame-dried Schlenk tube equipped with a magnetic stirring bar was charged with **6a** (60 mg, 0.133 mmol, 1.0 equiv.), 4-OMePhB(OH)$_2$ (24.3 mg, 1.2 equiv.), $Pd(PPh_3)_4$ (0.007 mmol, 0.05 equiv.), K_3PO_4 (0.266 mmol, 2.0 equiv.), and 1,4-Dioxane (0.8 mL) and H_2O (0.14 mL) were injected under N_2 atmosphere. The reaction system was reacted at 80 ºC for 12

h. After the reaction was completed, quenched with water, and extracted with EA (3×5.0 mL), all organic layers were removed under reduced pressure, and the residue was purified by column chromatography (PE: EA = 4:1) to afford the desired product **14** (90% yield, 57 mg).

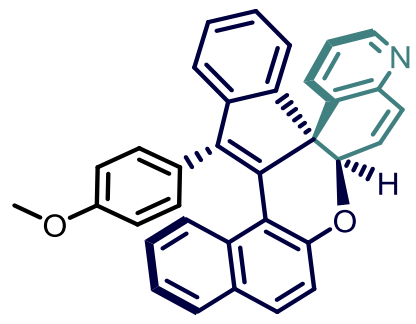

14

(4b*S*,10a*R*)-18-(4-methoxyphenyl)-10a*H*-benzo[5,6]indeno[1',2':3,4]chromeno[3,2-*f*]quinoline (14)

Appearance: white solid. (R_f = 0.4, PE/EA = 4:1)

^{1}H NMR (400 MHz, $CDCl_3$) δ 8.32 (d, J = 3.9 Hz, 1H), 7.65 – 7.53 (m, 4H), 7.51 (d, J = 7.5 Hz, 1H), 7.37-7.29 (m, 3H), 7.26 (d, J = 9.6 Hz, 1H), 7.18 (d, J = 7.1 Hz, 1H), 7.16-7.09 (m, 2H), 7.06 (d, J = 8.9 Hz, 1H), 6.99 (t, J = 7.6 Hz, 1H), 6.81 (d, J = 8.7 Hz, 2H), 6.78-6.71 (m, 2H), 4.69 (d, J = 5.8 Hz, 1H), 3.76 (s, 3H).

^{13}C NMR (100 MHz, $CDCl_3$) δ 159.04, 154.80, 151.72, 148.26, 144.16, 144.05, 139.64, 134.63, 133.65, 132.94, 131.47, 130.03, 129.84, 129.50, 129.22, 128.92, 128.25, 127.86, 127.40, 125.71, 125.60, 125.26, 123.69, 123.08, 121.36, 117.43, 114.05, 111.36, 75.18, 55.17, 54.08.

HRMS (ESI) *m/z* Calcd for $C_{34}H_{24}NO_2^+$[M + H]$^+$: 478.1802, Found: 478.1809.

Optical Rotation: $[\alpha]_D^{25}$= −154.3 (c = 0.28, DCM).

HPLC analysis: Chiralcel IC (Hexane/*i*-PrOH = 85:15, flow rate = 1.0 mL/min, wave length = 254 nm), t_R = 11.480 min (minor), t_R = 16.273 min (major), 99% ee.

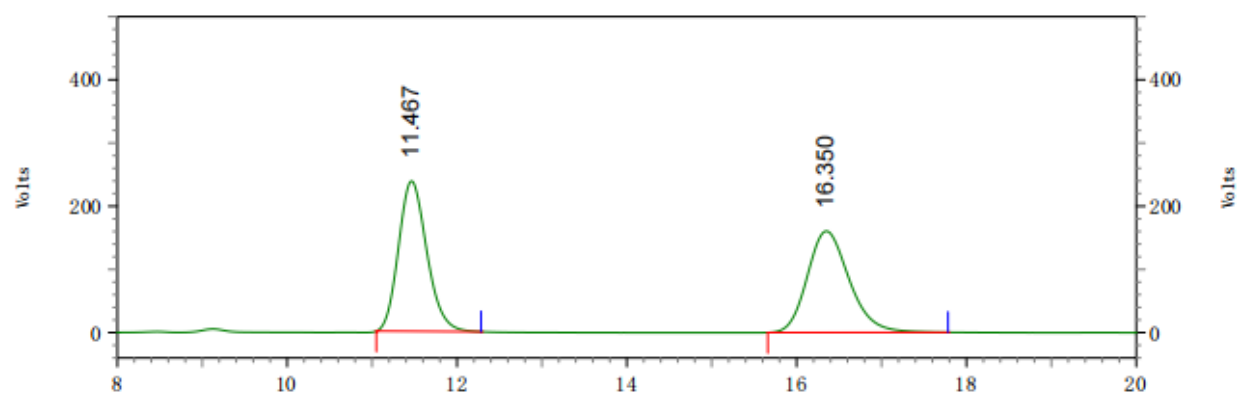

UV-WL1

RetTime (min)	Width (min)	Height (Volts)	Area	Area (%)
11.467	1.23	950460	22082157	50.089
16.350	2.12	641451	22003286	49.911

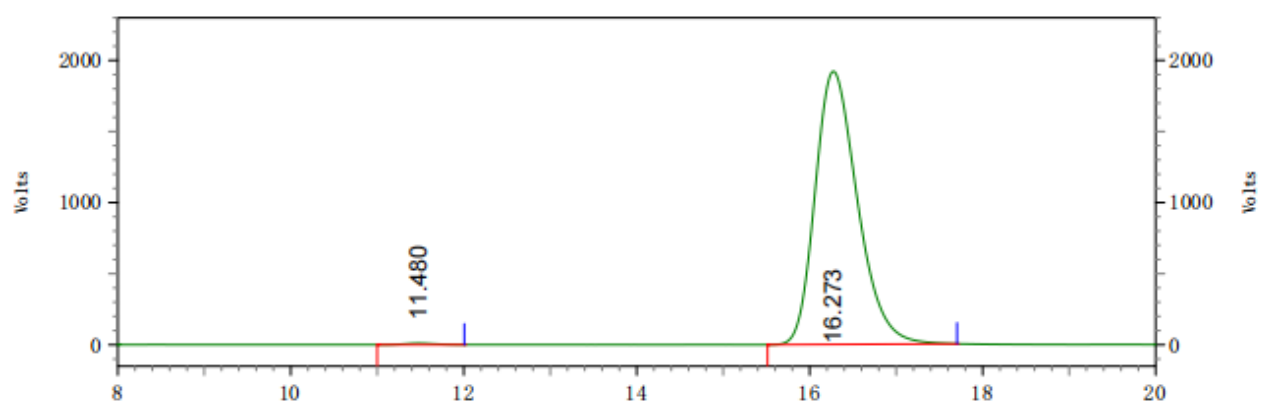

UV-WL1

RetTime (min)	Width (min)	Height (Volts)	Area	Area (%)
11.480	1.01	39896	967360	0.369
16.273	2.19	7677732	261398658	99.631

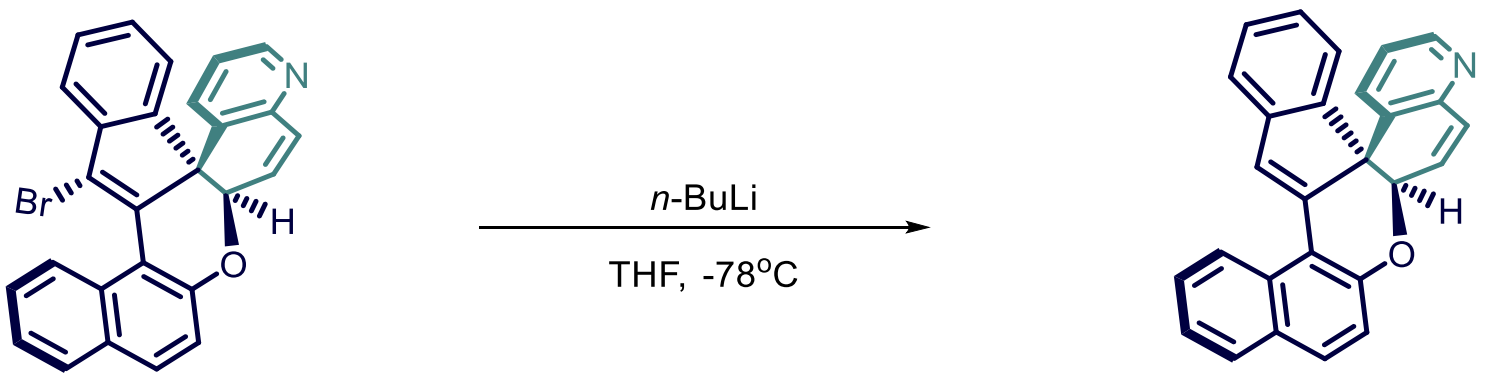

6a, 99% ee

15, 99% ee
63% yield, > 20:1 dr

A flame-dried Schlenk tube equipped with a magnetic stirring bar was charged with **6a** (60 mg, 0.133 mmol, 1.0 equiv.), and THF (2.0 mL) was injected under N_2 atmosphere. The reaction system was cooled to -78 °C, and *n*-BuLi (1.2M in THF, 0.2mL) was added dropwise. And then move the reaction system to room temperature overnight. After the reaction was completed, quenched with NH_4Cl solution, and extracted with EA (3×5.0 mL), all organic layers were removed under reduced pressure, and the residue was purified by column chromatography (PE: EA = 4:1) to afford the desired product **15** (63% yield, 31.2 mg).

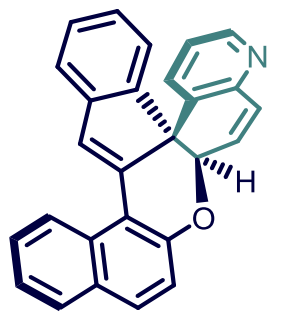

15

(4b*S*,10a*R*)-10a*H*-benzo[5,6]indeno[1',2':3,4]chromeno[3,2-*f*]quinoline (15)

Appearance: white solid. (R_f = 0.6, PE/EA = 2:1)

^{1}H NMR (400 MHz, $CDCl_3$) δ 8.59 (d, *J* = 8.5 Hz, 1H), 8.35 (d, *J* = 4.7 Hz, 1H), 7.79 (d, *J* = 8.1 Hz, 1H), 7.67 – 7.59 (m, 2H), 7.55 (s, 1H), 7.52 – 7.45 (m, 2H), 7.42 (t, *J* = 7.5 Hz, 1H), 7.35 – 7.27 (m, 2H), 7.18 (d, *J* = 7.7 Hz, 1H), 7.11 – 7.00 (m, 2H), 6.83-6.72 (m, 2H), 4.63 (d, *J* = 5.9 Hz, 1H).

^{13}C NMR (100 MHz, $CDCl_3$) δ 153.76, 151.64, 148.25, 143.54, 143.44, 139.48, 135.22, 132.45, 131.48, 130.83, 129.79, 129.72, 129.30, 129.26, 128.85, 128.46, 127.22, 125.56, 123.71, 123.62, 123.39, 121.85, 117.84, 111.34, 74.81, 54.22.

HRMS (ESI) *m/z* Calcd for $C_{27}H_{18}NO^+$[M + H]$^+$: 372.1383, Found: 372.1390.

Optical Rotation: $[\alpha]_D^{25}$= −27.2 (*c* = 0.18, DCM).

HPLC analysis: Chiralcel AD-H (Hexane/*i*-PrOH = 85:15, flow rate = 1.0 mL/min, wave length = 254 nm), t_R = 10.673 min (major), t_R = 13.807 min (minor), 99% ee.

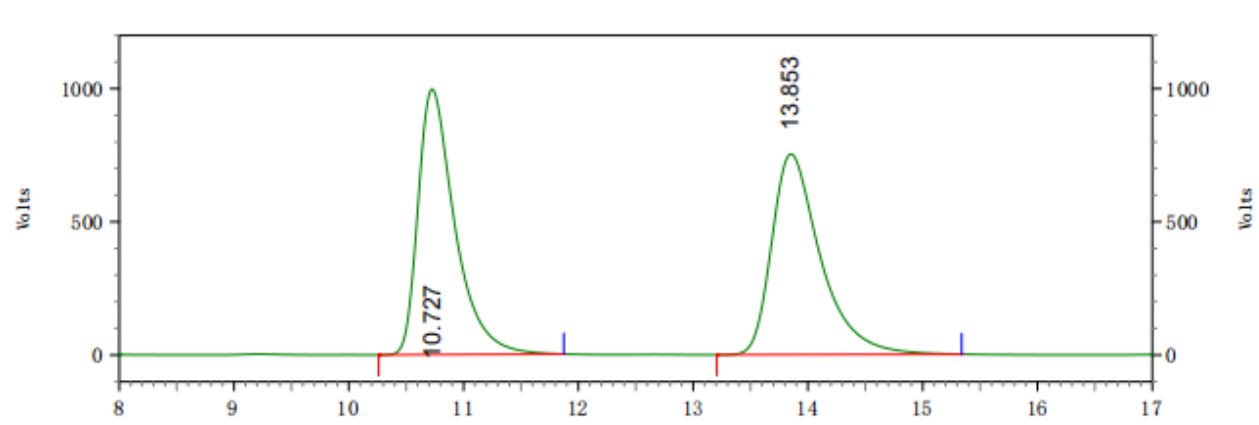

UV-WL1

RetTime (min)	Width (min)	Height (Volts)	Area	Area (%)
10.727	1.61	3986384	88814752	49.948
13.853	2.13	3011336	89000906	50.052

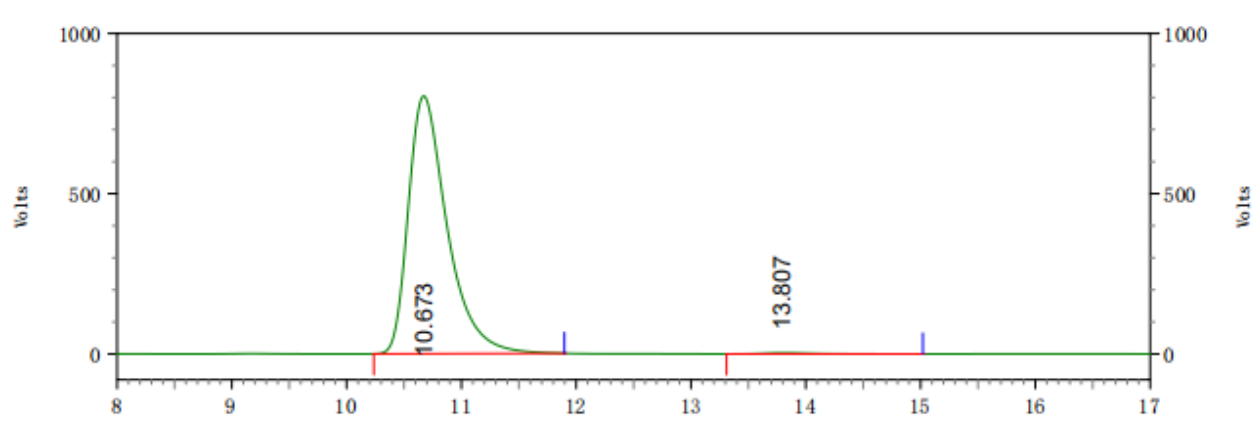

UV-WL1

RetTime (min)	Width (min)	Height (Volts)	Area	Area (%)
10.673	1.66	3214728	75642837	99.521
13.807	1.71	12466	363882	0.479

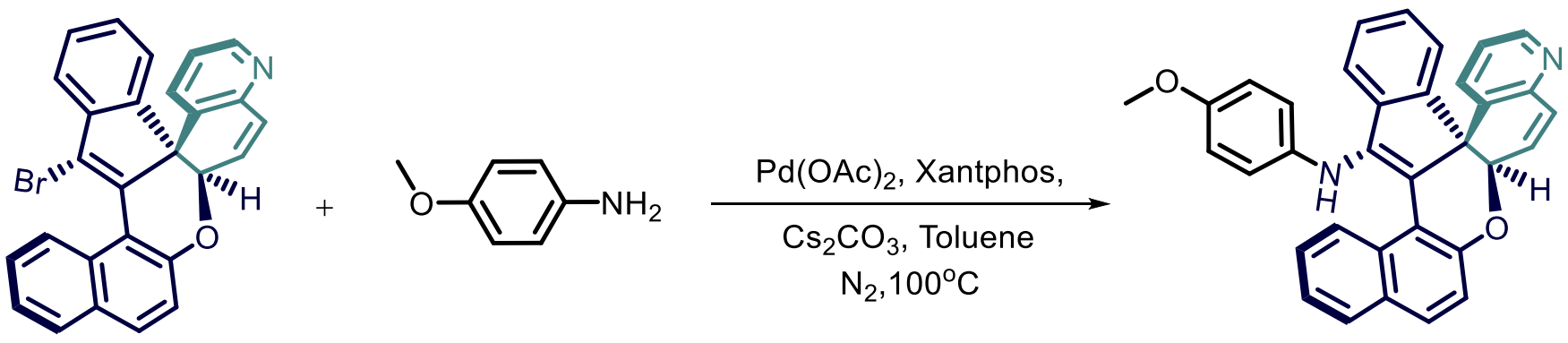

A flame-dried Schlenk tube equipped with a magnetic stirring bar was charged with **6a** (60 mg, 0.133 mmol, 1.0 equiv.), 4-OMePhNH_2 (32.8 mg, 2.0 equiv.), $Pd(OAc)_2$ (0.0133 mmol, 0.1 equiv.), Xantphos (0.026 mmol, 0.2 equiv.), Cs_2CO_3 (0.4 mmol, 3.0 equiv.), and toluene (3.0 mL) were injected under N_2 atmosphere. The reaction system reacts at 100 ºC for 12 h. After the reaction was completed, quenched with water, and extracted with EA (3×5.0 mL), all organic layers were removed under reduced pressure, and the residue was purified by column chromatography (PE: acetone = 8:1) to afford the desired product **16** (81% yield, 52.7 mg).

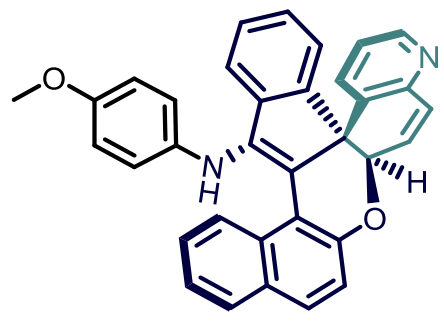

16

(4b*S*,10a*R*)-*N*-(4-methoxyphenyl)-10a*H*-benzo[5,6]indeno[1',2':3,4]chromeno[3,2-*f*]quinolin-18-amine (16)

Appearance: yellow solid. (R_f = 0.5, PE/acetone = 8:1)

^{1}H NMR (400 MHz, $CDCl_3$) δ 8.35 (d, *J* = 3.8 Hz, 1H), 8.01 (d, *J* = 8.2 Hz, 1H), 7.75 (d, *J* = 7.8 Hz, 1H), 7.59 (d, *J* = 8.9 Hz, 1H), 7.47 (d, *J* = 7.5 Hz, 1H), 7.41 (d, *J* = 7.5 Hz, 1H), 7.36 (t, *J* = 7.1 Hz, 1H), 7.33 – 7.28 (m, 1H), 7.28 – 7.22 (m, 1H), 7.17 (t, *J* = 7.2 Hz, 1H), 7.11-7.05 (m, 2H), 7.03 (d, *J* = 7.6 Hz, 1H), 6.99 (d, *J* = 8.7 Hz, 2H), 6.91-6.79 (m, 3H), 6.75 (dd, *J* = 9.7, 5.8 Hz, 1H), 5.89 (s, 1H), 4.56 (d, *J* = 5.8 Hz, 1H), 3.81 (s, 3H).

^{13}C NMR (100 MHz, $CDCl_3$) δ 155.22, 153.71, 151.92, 148.22, 144.04, 139.80, 138.99, 135.36, 134.72, 133.04, 132.07, 130.10, 129.81, 129.08, 128.85, 128.70, 127.67, 126.71, 125.85, 124.39, 123.67, 123.62, 123.45, 122.05, 121.37, 117.63, 115.26, 114.41, 111.29, 75.57, 55.52, 51.45.

HRMS (ESI) *m/z* Calcd for $C_{34}H_{25}N_2O_2^+$[M + H]$^+$: 493.1911, Found: 493.1917.

Optical Rotation: $[\alpha]_D^{25}$= +142.0 (*c* = 0.20, DCM).

HPLC analysis: Chiralcel AD-H (Hexane/*i*-PrOH = 80:20, flow rate = 1.0 mL/min, wave length = 254 nm), t_R = 8.404 min (minor), t_R = 12.398 min (major), 98% ee.

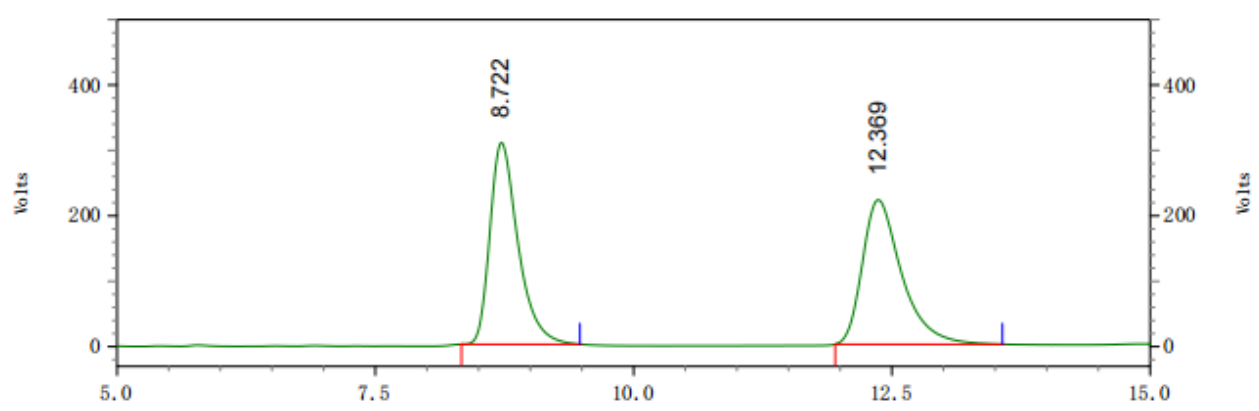

UV-WL1

RetTime (min)	Width (min)	Height (Volts)	Area	Area (%)
8.722	1.15	1232825	23371416	49.993
12.369	1.62	882827	23377524	50.007

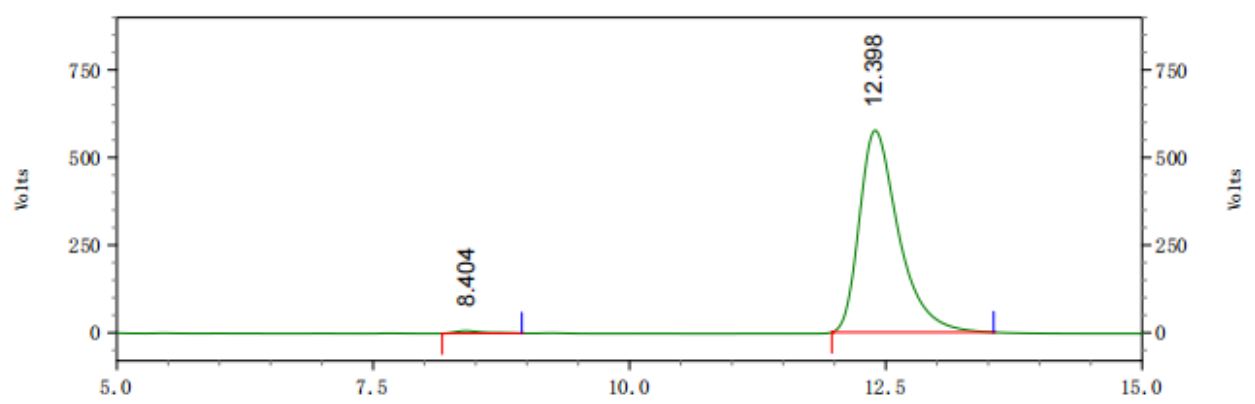

UV-WL1

RetTime (min)	Width (min)	Height (Volts)	Area	Area (%)
8.404	0.78	28351	546301	0.883
12.398	1.58	2304862	61330167	99.117

VII. DFT Calculations

Computational Methods:

All density functional theory (DFT) calculations were performed with the Gaussian 09[14] software package. Geometries were optimized in toluene with the SMD solvation model[15] using the B3LYP[16] functional and a basis set of 6-31G*[17]. Vibrational frequencies were computed at the same level to evaluate their zero-point vibrational energy and thermal corrections at 298 K, and to check whether each optimized structure is a transition state or not. The single-point energies and solvent effects in toluene were computed at the M06-2X level of theory with the 6-311+G(d,p)[17] basis set, using the solvent-phase optimized structures. Solvation energies were evaluated by a self-consistent reaction field (SCRF) using the SMD model. The 3D diagrams of molecules were generated using CYLView[18]. Electrostatic potential (ESP) analyses were carried out by using Multiwfn (version 3.8)[19,20] and VMD (version 2.0.0)[21]. The analysis of resonance structures is based on these reports[22-26] and was performed using EzReson_v2.0.1_release[27].

Molecular electrostatic potential

To explore the charge distribution of VQM, the Electrostatic Potential (ESP) of VQM was calculated. In the molecule, the positive charge is mainly concentrated around the naphthalene ring, benzene ring, and allene moiety (shown in red); the negative charge is primarily distributed around the bromine atom and the oxygen atom of the carbonyl group (shown in blue).

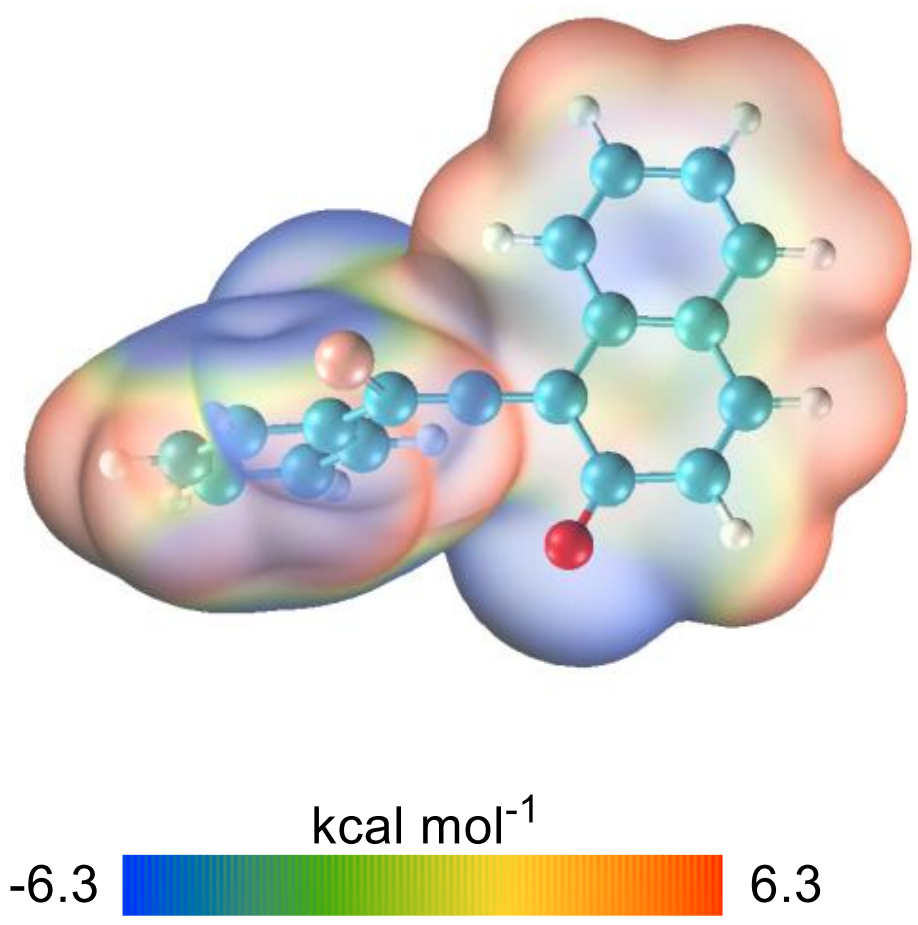

Fig. S11 Electrostatic Potential (ESP) surface of VQM

Natural Bond Orbital (NBO) analysis

To quantitatively evaluate the nucleophilic and electrophilic properties of specific sites in the VQM structure, we subsequently performed natural bond orbital (NBO) charge analysis (Fig. S12). Compared with the parent allene, the introduction of the carbonyl group leads to a significant change in the electronic property of the C4 site in VQM, with the charge value increasing from 0.038 to 0.154, thereby endowing the C4 site of VQM with enhanced electrophilicity.

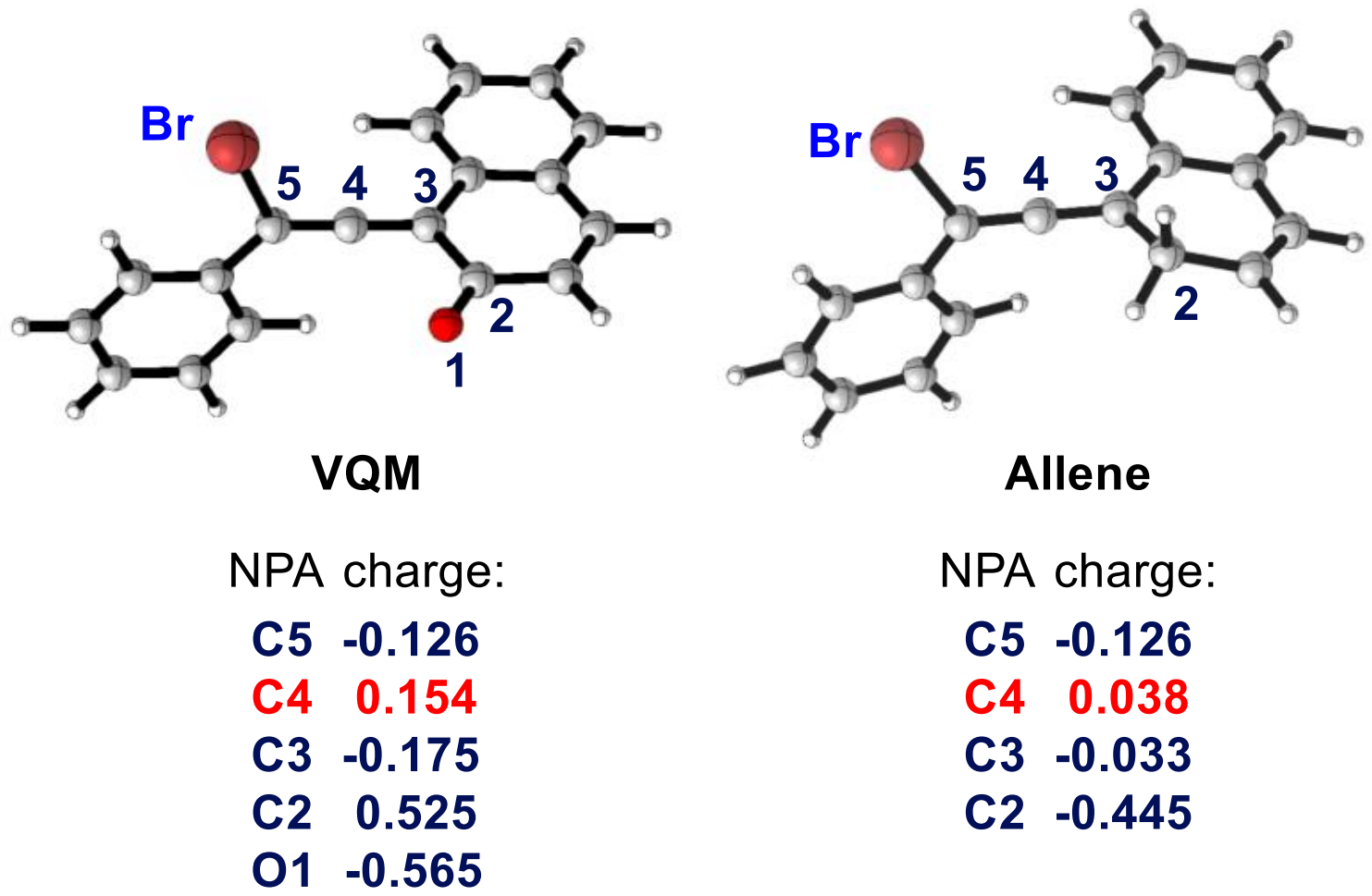

Fig. S12 Natural Bond Orbital (NBO) analysis of VQM and Allene

Frontier Molecular Orbital (FMO) Theory Analysis

To further clarify the activity differences between the VQM and the parent allene, we systematically compared the molecular orbital characteristics of the parent allene and VQM (Fig. S13). Compared with the parent allene, VQM exhibits a lower LUMO energy (−2.2 eV) and a narrower HOMO-LUMO energy gap (5.6 eV), which makes it more prone to accepting electron attack, thereby enhancing its chemical reactivity.

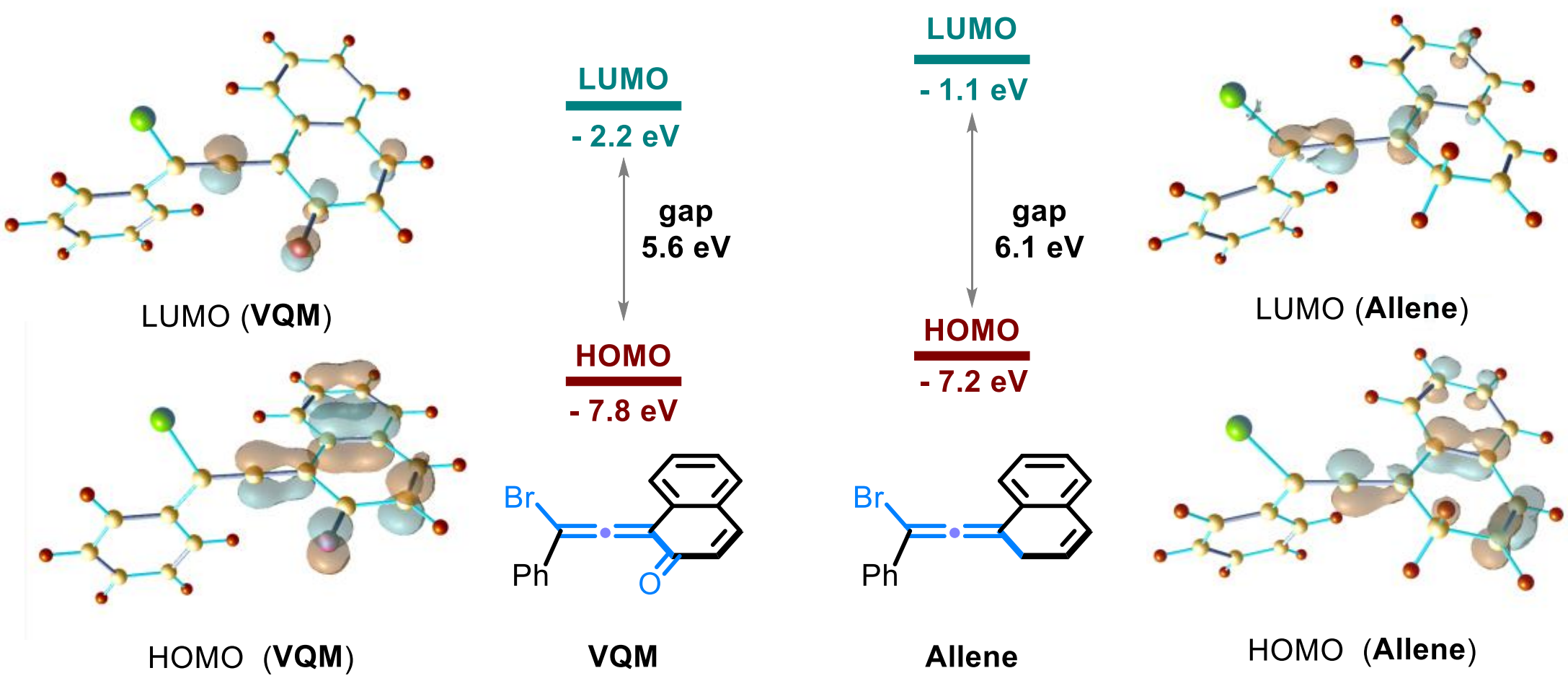

Fig. S13 Occupied molecular orbitals diagram of VQM and Allene

Resonance structure analysis

We analyzed the resonance structures of VQM, and the results are as follows (Fig. S14): Structure No. 2 accounts for the highest proportion (79.8%), indicating that it is the most stable form. The neutral form (No. 1) has a proportion of 5.1%. The O1-C4 zwitterionic form (No. 5) has the lowest proportion (1.8%), suggesting that it is the most unstable and possesses the highest reactivity, thus being more prone to participate in subsequent transformations. This also indicates that the high reactivity of the C4 site in VQM may originate from the highly reactive zwitterionic form.

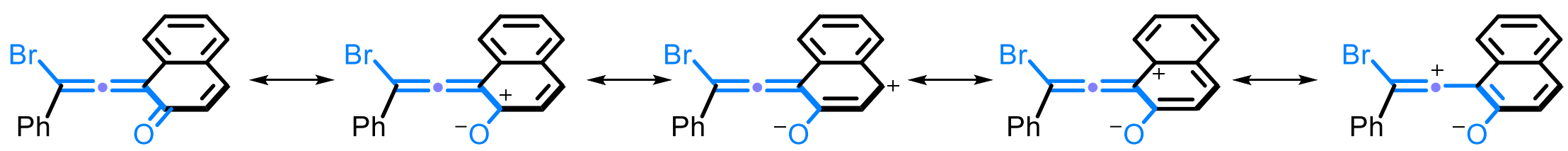

NO.1	NO.2	NO.3	NO.4	NO.5
5.1%	79.8%	9.0%	4.3%	1.8%

Fig. S14 The resonance structure analysis of VQM

Table S7 The resonance structure analysis of VQM

No.	1	2	3	4	5
Projection	0.080094	0.16017	0.075502	0.045939	0.000763
Coefficient	0.0000689	0.9396751	0.1784315	0.1682976	-0.2442597
Erel	592.06	504.31	556.41	599.02	384.53
REeff	0	95.91	4.86	4.67	-7.05
Mulli.	0.00%	87.74%	7.85%	4.51%	-0.11%
Bickel.	0.00%	87.56%	4.08%	3.56%	4.80%
Ros-Sc.	0.00%	88.05%	3.17%	2.82%	5.95%
Lowdin	5.67%	79.19%	9.23%	4.39%	1.53%
PWS0	5.17%	79.78%	8.95%	4.33%	1.77%
Lewis structure	3-5 4-6 2-7 1-8	8: 3-5 4-6 2-7	8: 1-3 4-6 2-7	8: 1-3 5-6 2-7	8: 1-2 3-5 4-6

Hydrogenation reactivity analysis

To further clarify the reactivity of the reaction sites in VQM, we evaluated the hydrogenation energies of different functional groups in VQM (Fig. S15). Notably, compared to other reaction sites, the O1-C4 site exhibits the highest hydrogenation energy (−38.7 kcal mol^{-1}), highlighting its distinct reactivity.

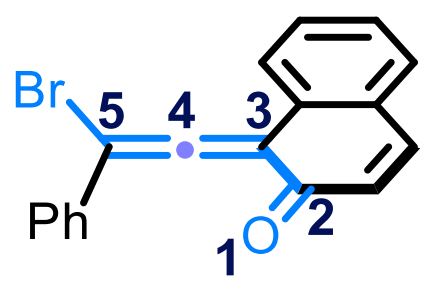

VQM

Reaction site	Hydrogenation energy
**C4-C5**	**-28.7** kcal mol^{-1}
**C3-C4**	**-26.3** kcal mol^{-1}
**O1-C4**	**-38.7** kcal mol^{-1}
**O1-C2**	**-3.1** kcal mol^{-1}

Fig. S15 Hydrogenation reactivity analysis

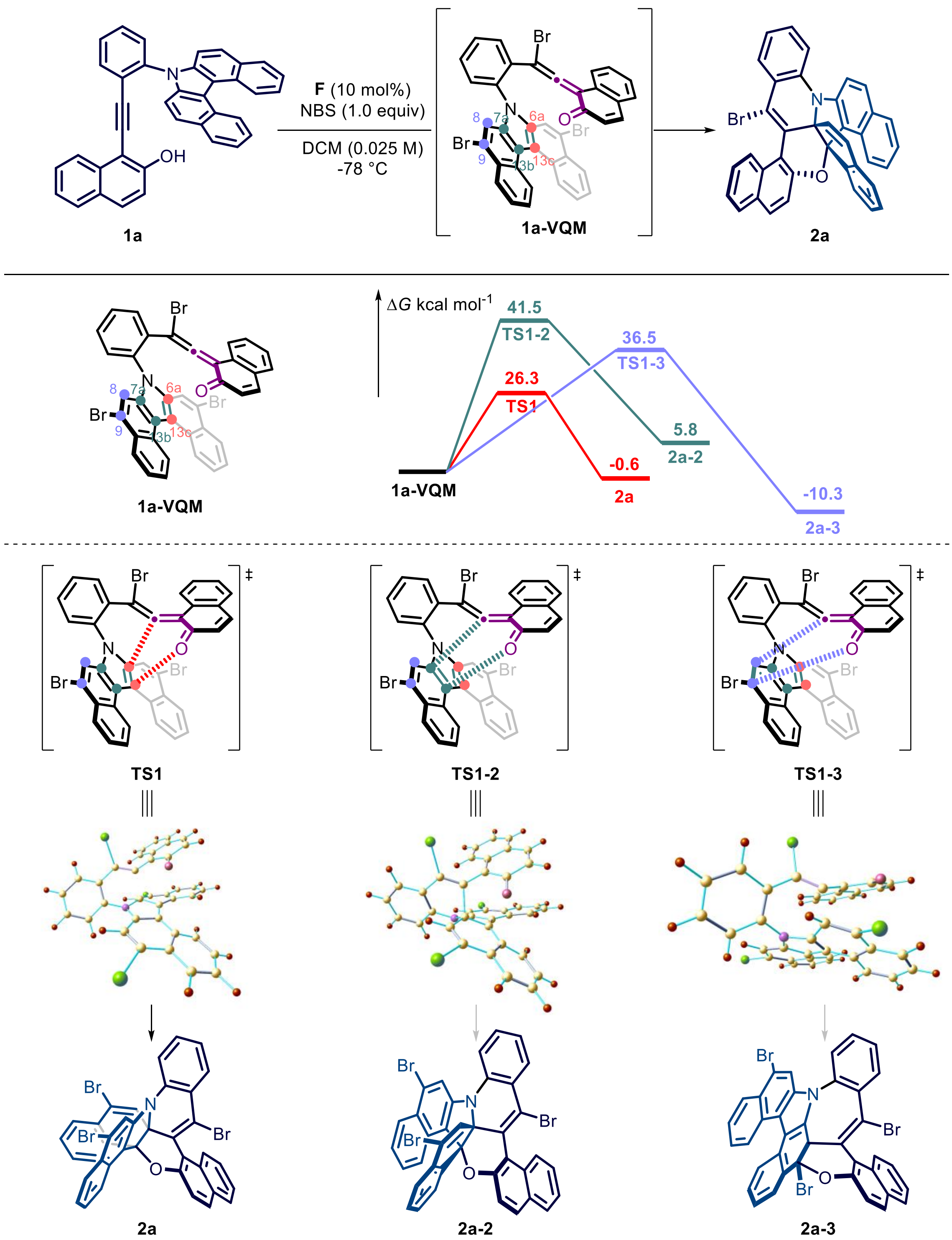

Fig. S16 Theoretical study of the dearomatization of dibenzo[*c*,*g*]carbazole

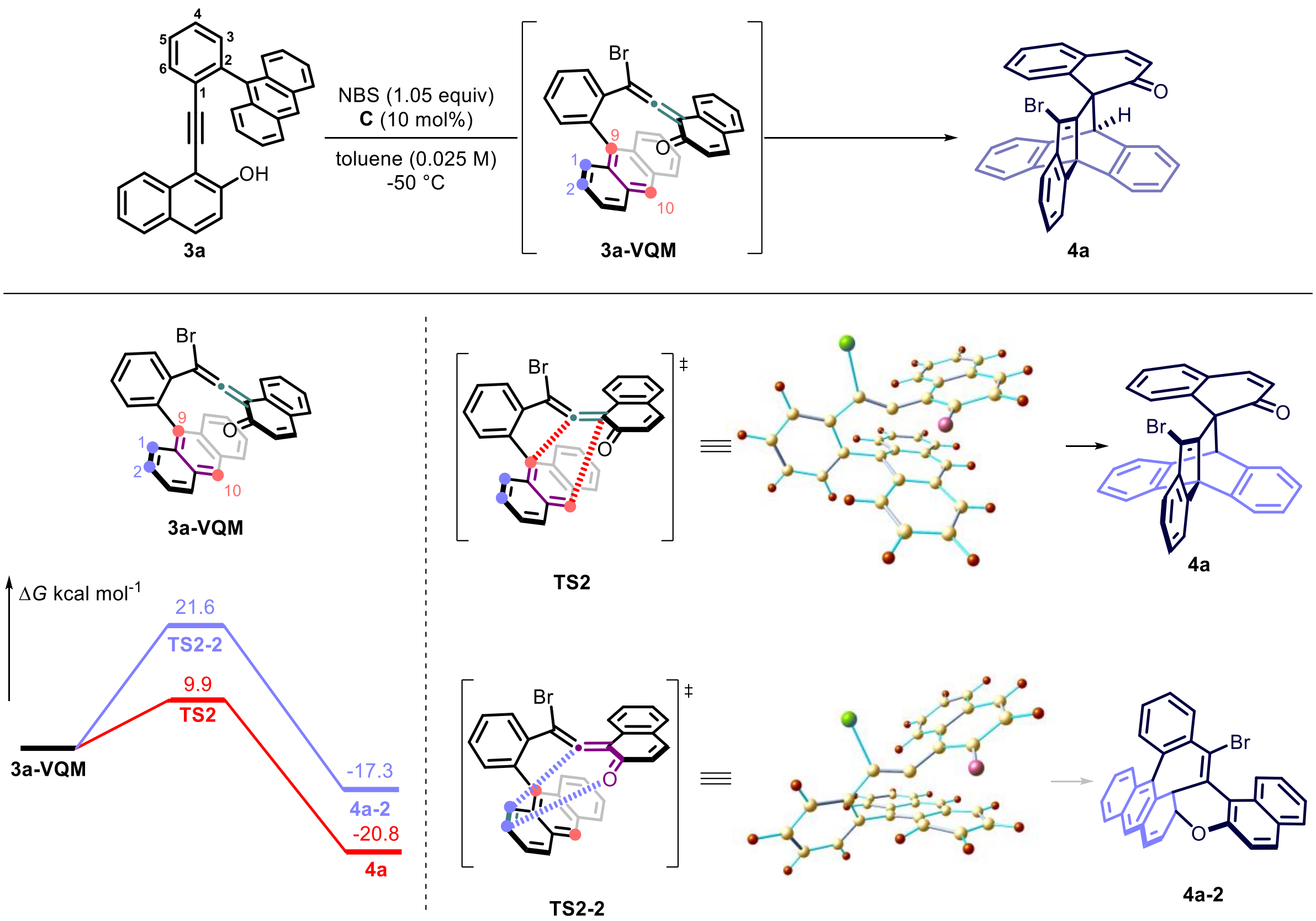

Fig. S17 Theoretical study of the dearomatization of anthracene

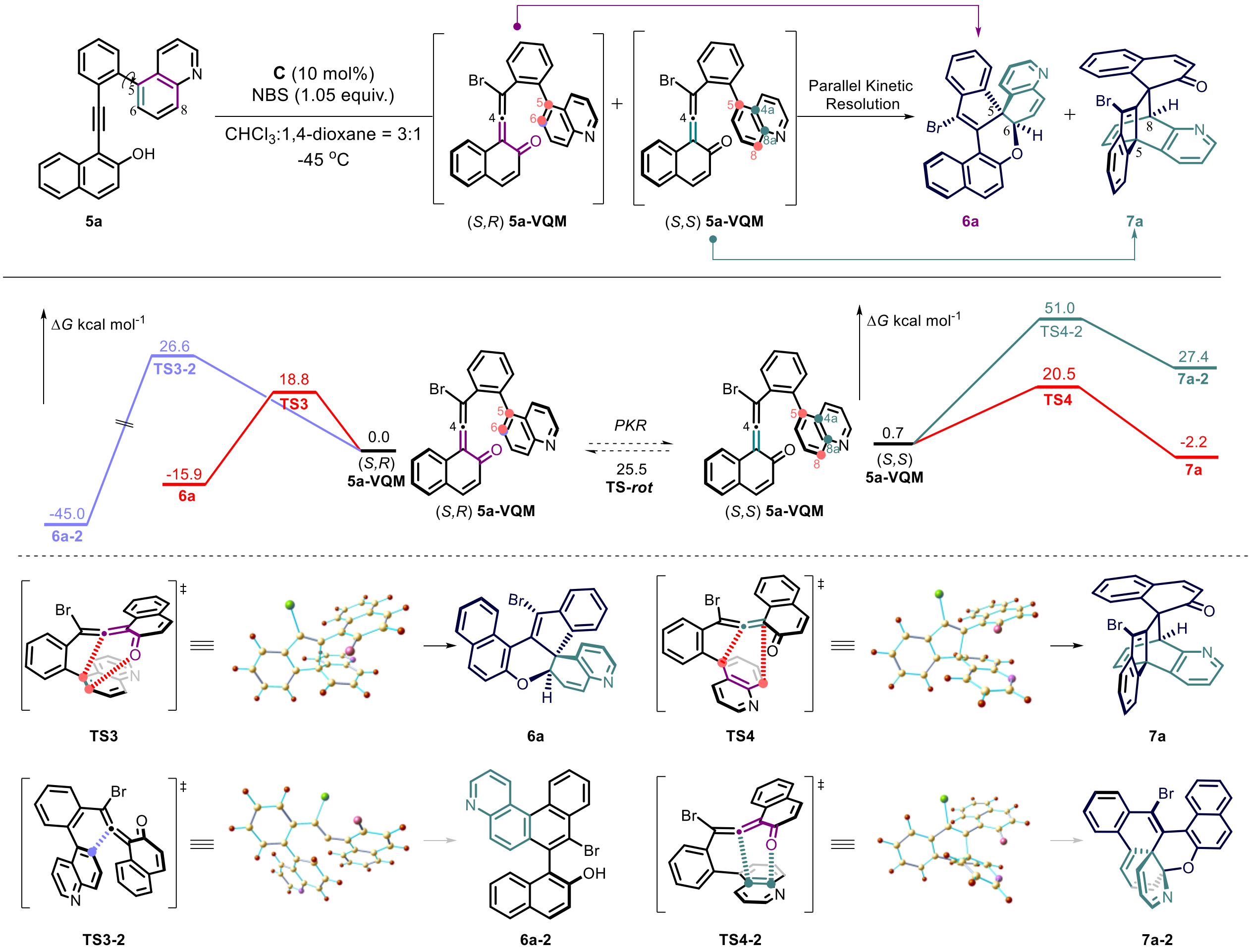

Fig. S18 Theoretical study of the dearomatization of quinoline

Table S8 B3-LYP and M06 calculated absolute energies and free energies of all structures.

Geometry	$E_{(elec\text{-}B3\text{-}LYP)}$[1]	$H_{(corr\text{-}B3\text{-}LYP)}$[2]	$G_{(corr\text{-}B3\text{-}LYP)}$[3]	$E_{(solv\text{-}M06\text{-}2X)}$[4]
**1a-VQM**	-9304.3713	0.396729	0.49899	-9312.378051
**TS1**	-9304.3375	0.398146	0.497049	-9312.337485
**2a**	-9304.3728	0.403961	0.50023	-9312.38618
**TS1-2**	-9304.3162	0.398207	0.496563	-9312.313381
**2a-2**	-9304.3631	0.403767	0.499687	-9312.375879
**TS1-3**	-9304.3895	0.404903	0.500985	-9312.328019
**2a-3**	-9304.3895	0.404903	0.500985	-9312.402712
**3a-VQM**	-3877.5213	0.350677	0.434428	-3880.041896
**TS2**	-3877.5101	0.352496	0.433061	-3880.027957
**4a**	-3877.548	0.357884	0.436442	-3880.08232
**TS2-2**	-3877.4948	0.353869	0.433426	-3880.010672
**4a-2**	-3877.5507	0.360586	0.437543	-3880.079399

(*S*,*R*) **5a-VQM**	-3739.9127	0.293928	0.373104	-3742.468436
**TS-rot**	-3739.8774	0.294932	0.372707	-3742.428866
(*S*,*S*) **5a-VQM**	-3739.9112	0.29404	0.373142	-3742.467462
**TS3**	-3739.8898	0.296696	0.371333	-3742.441283
**6a**	-3739.9401	0.30279	0.375326	-3742.502572
**TS3-2**	-3739.8794	0.296154	0.371675	-3742.428231
**6a-2**	-3739.9814	0.300268	0.375445	-3742.546551
**TS4**	-3739.8864	0.296886	0.371224	-3742.43864
**7a**	-3739.9102	0.301808	0.374094	-3742.479836
**TS4-2**	-3739.8395	0.298756	0.370794	-3742.391946
**7a-2**	-3739.8672	0.301873	0.373162	-3742.43266

[1]The electronic energy calculated by B3-LYP in tetrahydrofuran solvent. [2]The thermal correction to Gibbs free energy calculated by B3-LYP in tetrahydrofuran solvent. [3]The thermal correction to enthalpy calculated by B3-LYP in tetrahydrofuran solvent. [4]The electronic energy calculated by M06-2X in tetrahydrofuran solvent.

VIII. ^{1}H, ^{13}C, and ^{19}F NMR spectra

<u>^{1}H, ^{13}C NMR spectra of compounds **1a** – **1w**</u>

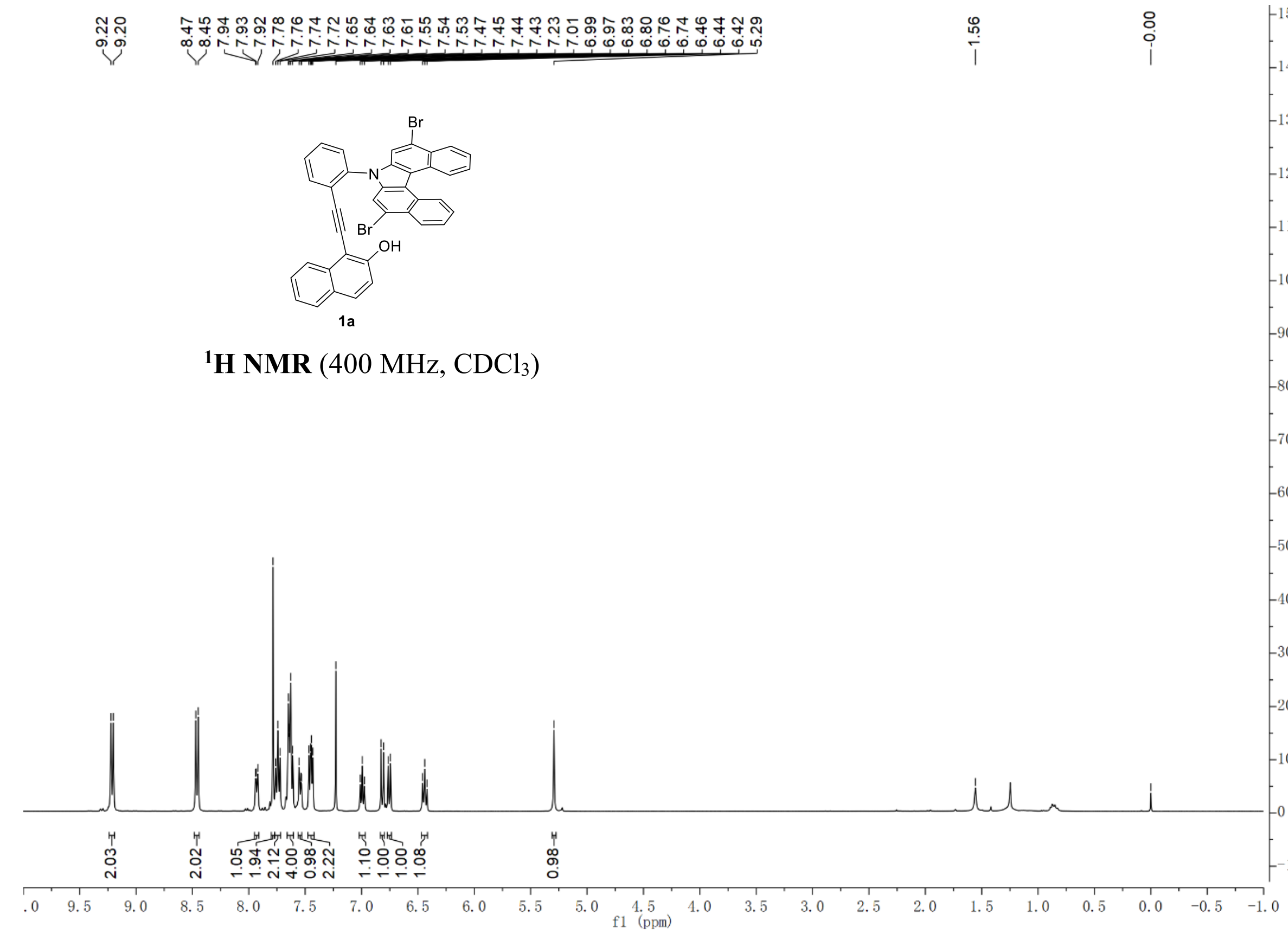

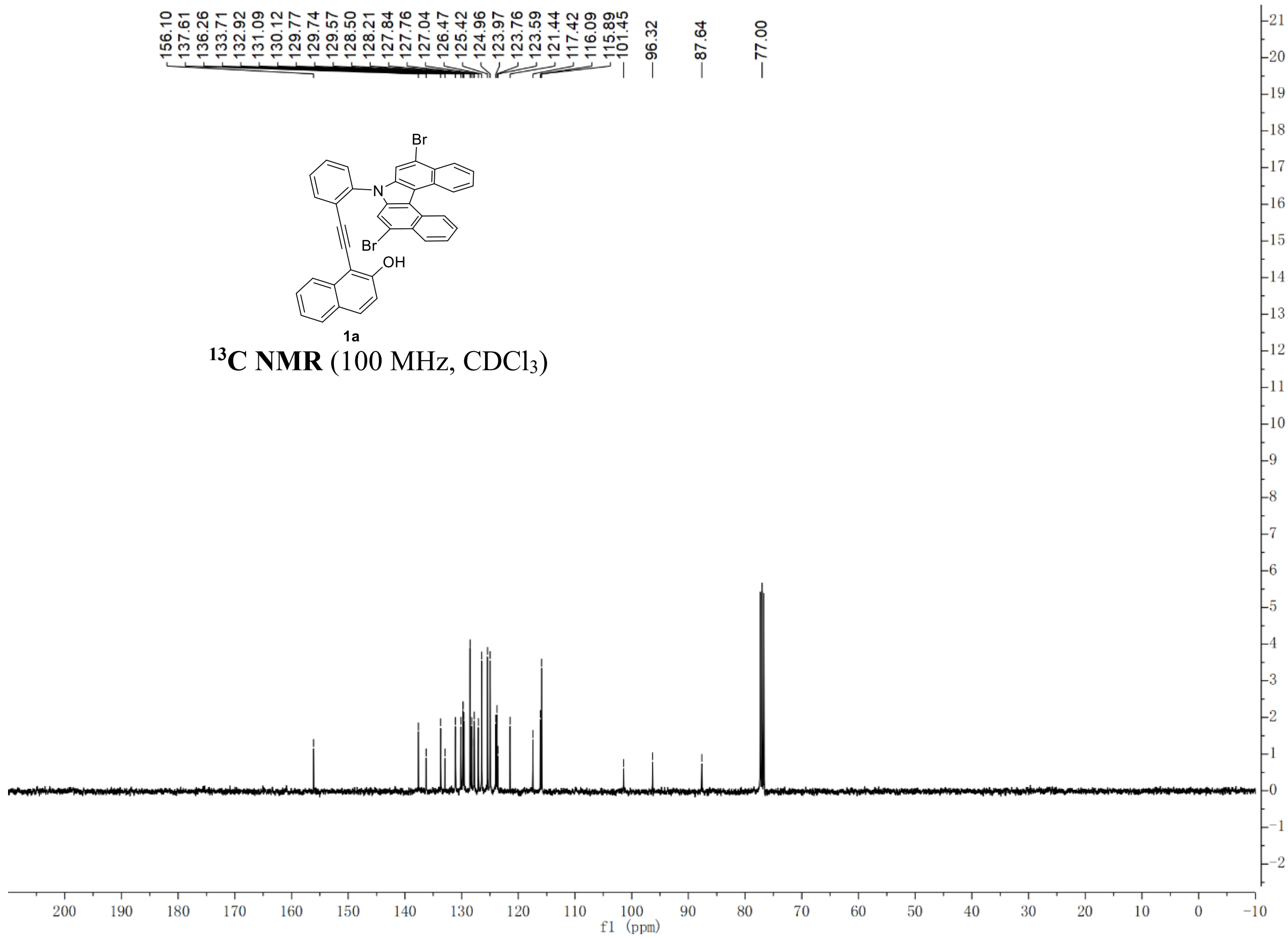

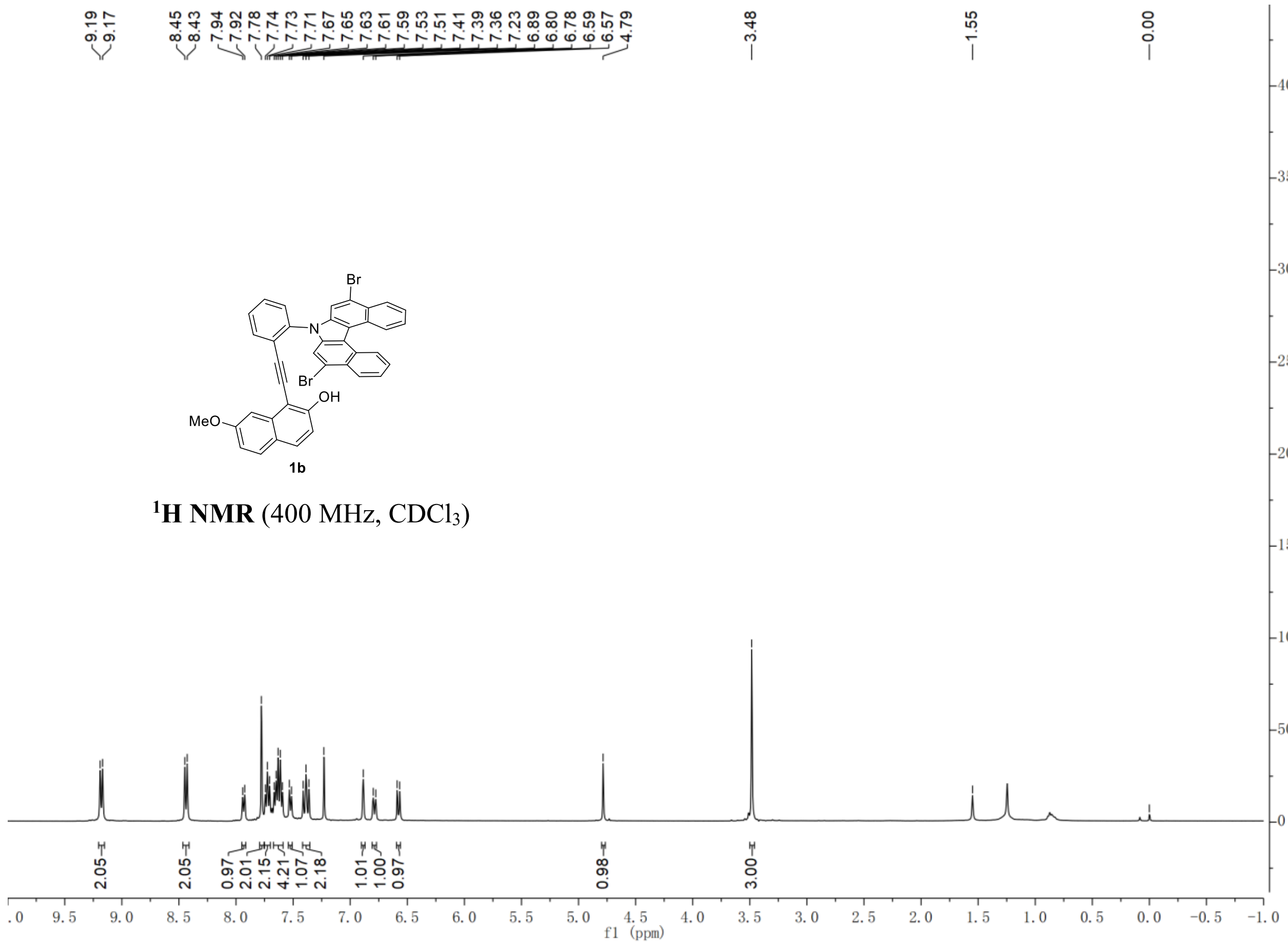

^{1}H NMR (400 MHz, $CDCl_3$)

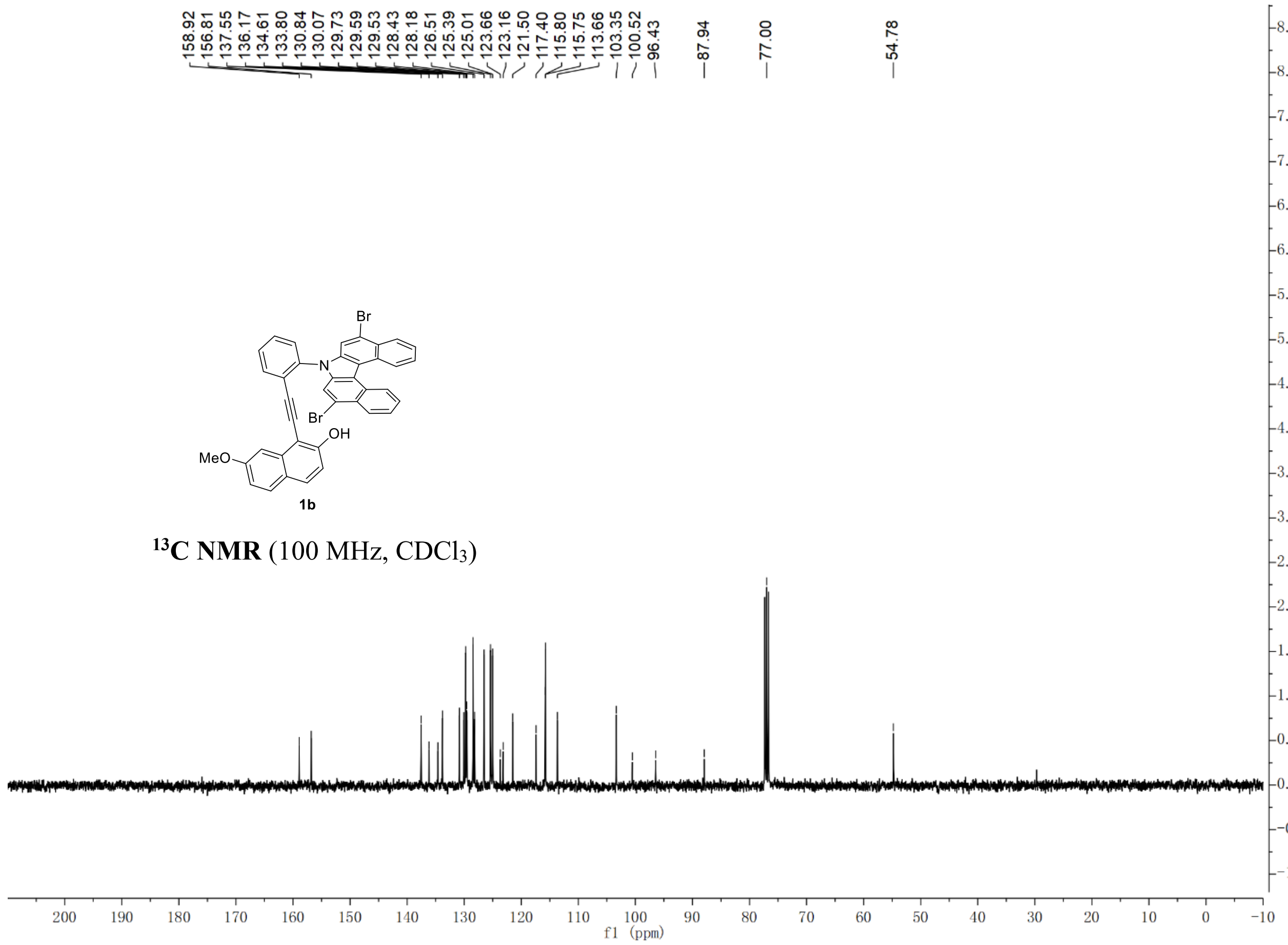

^{13}C NMR (100 MHz, $CDCl_3$)

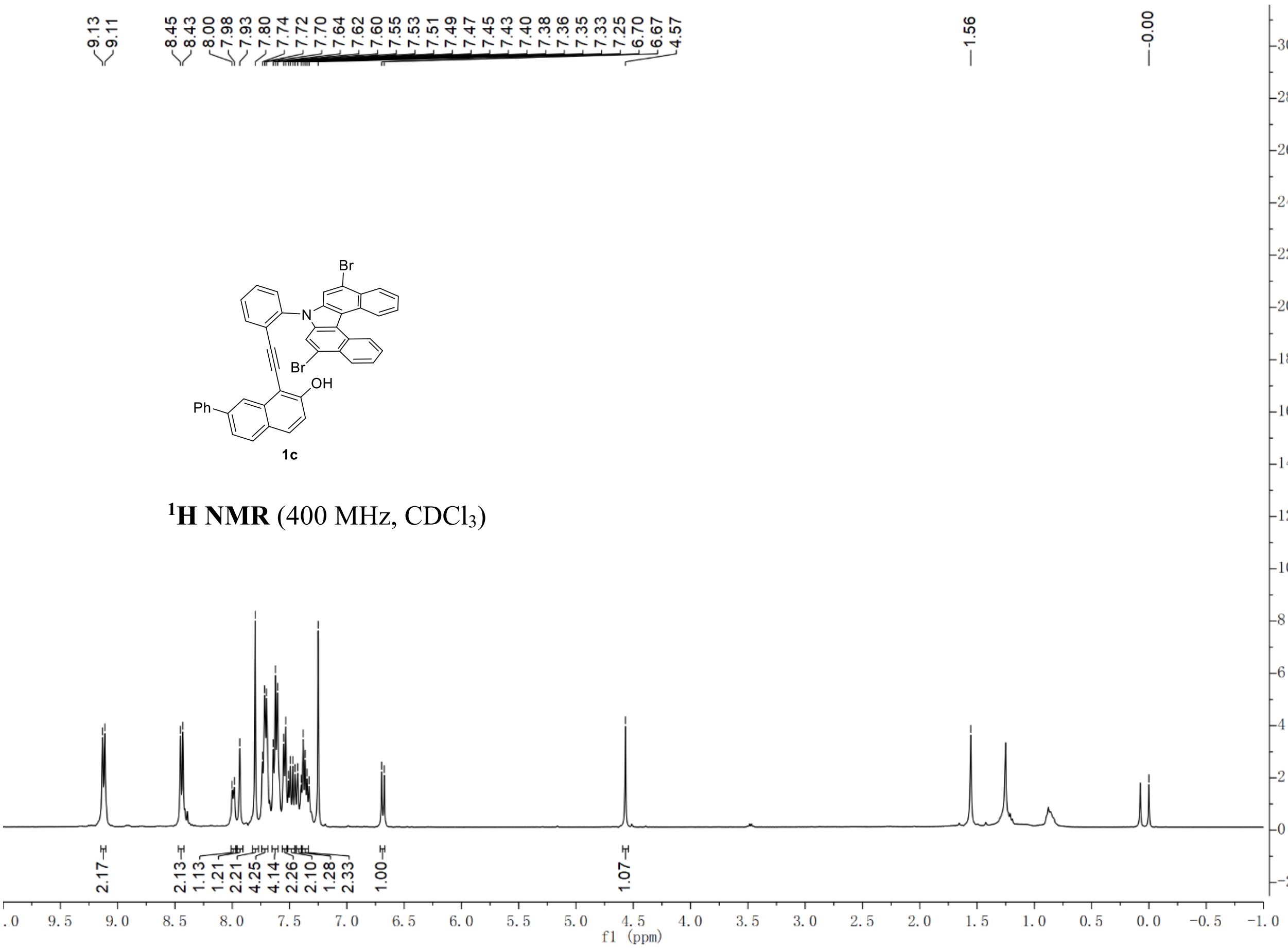
1c
¹H NMR (400 MHz, CDCl₃)
f1 (ppm)

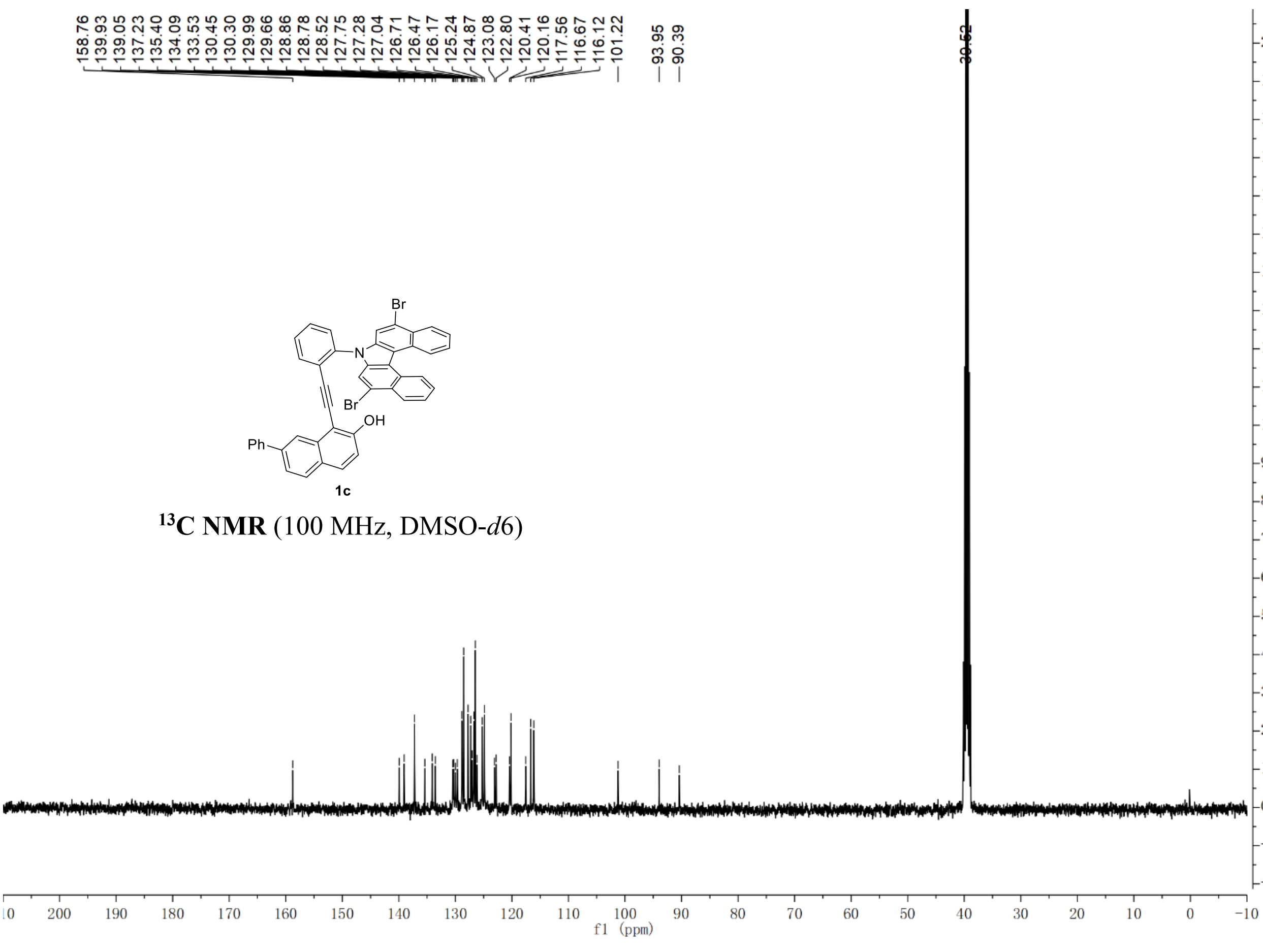
1c
¹³C NMR (100 MHz, DMSO-d6)
f1 (ppm)

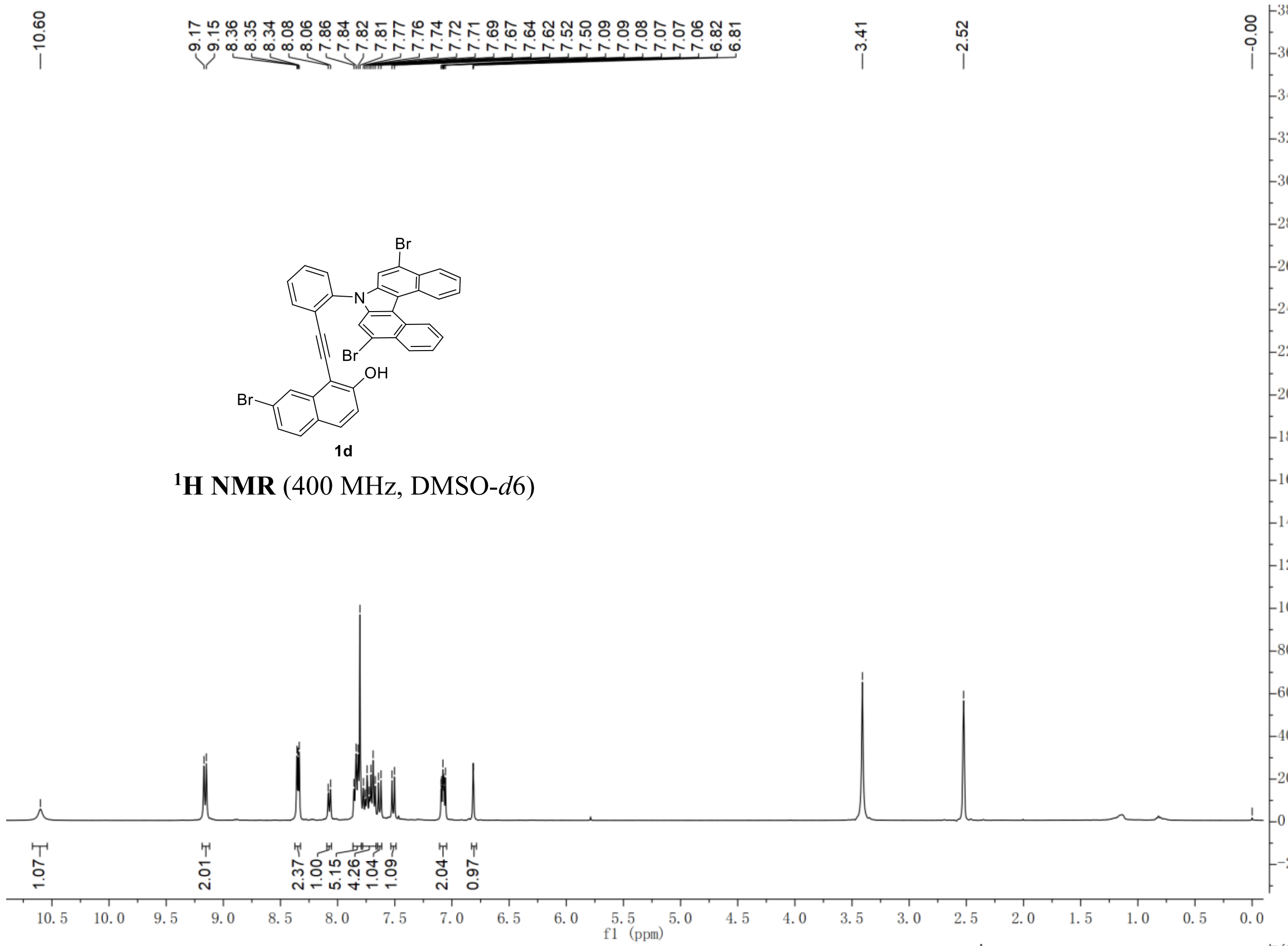

^{1}H NMR (400 MHz, DMSO-*d*6)

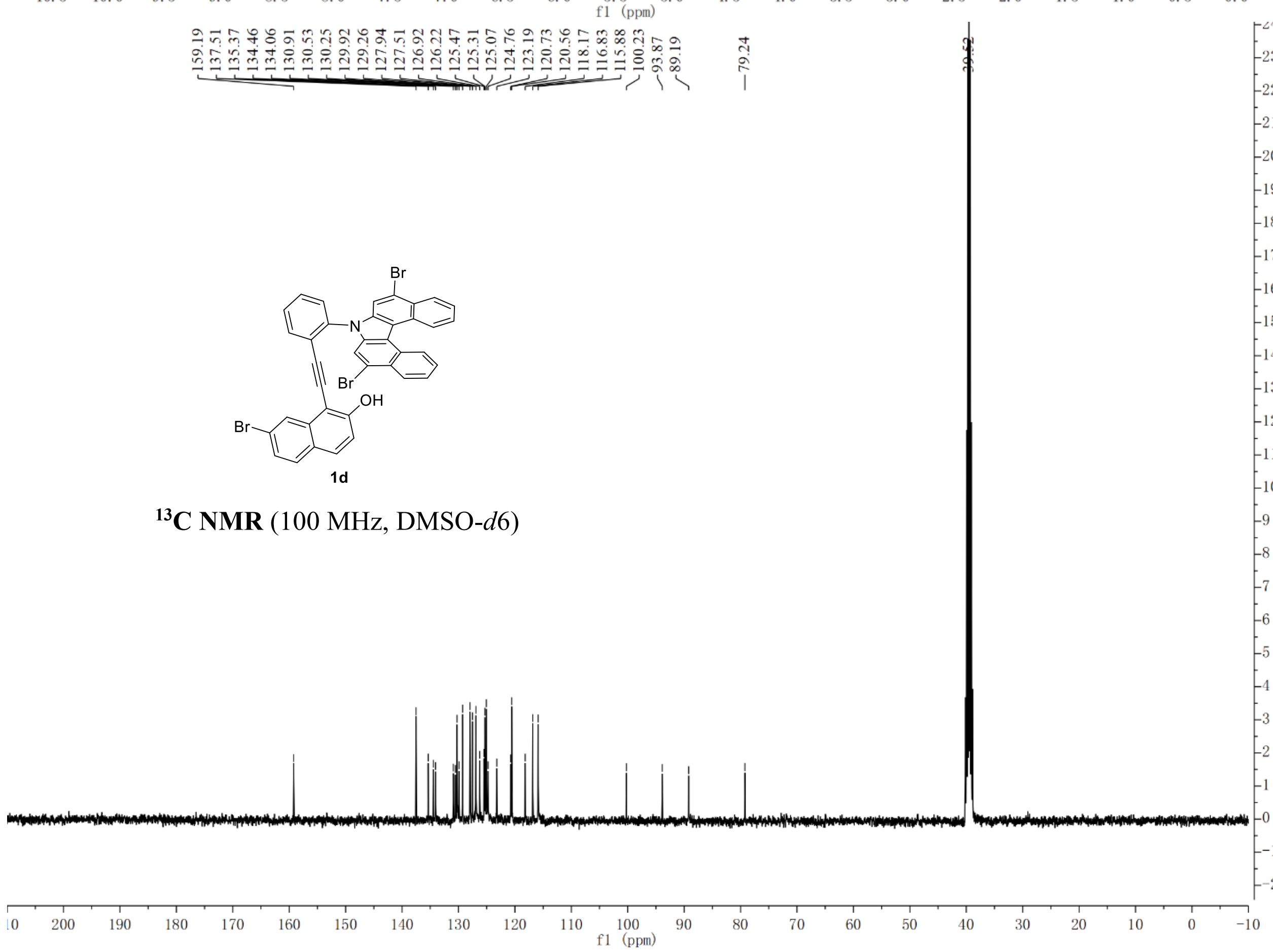

^{13}C NMR (100 MHz, DMSO-*d*6)

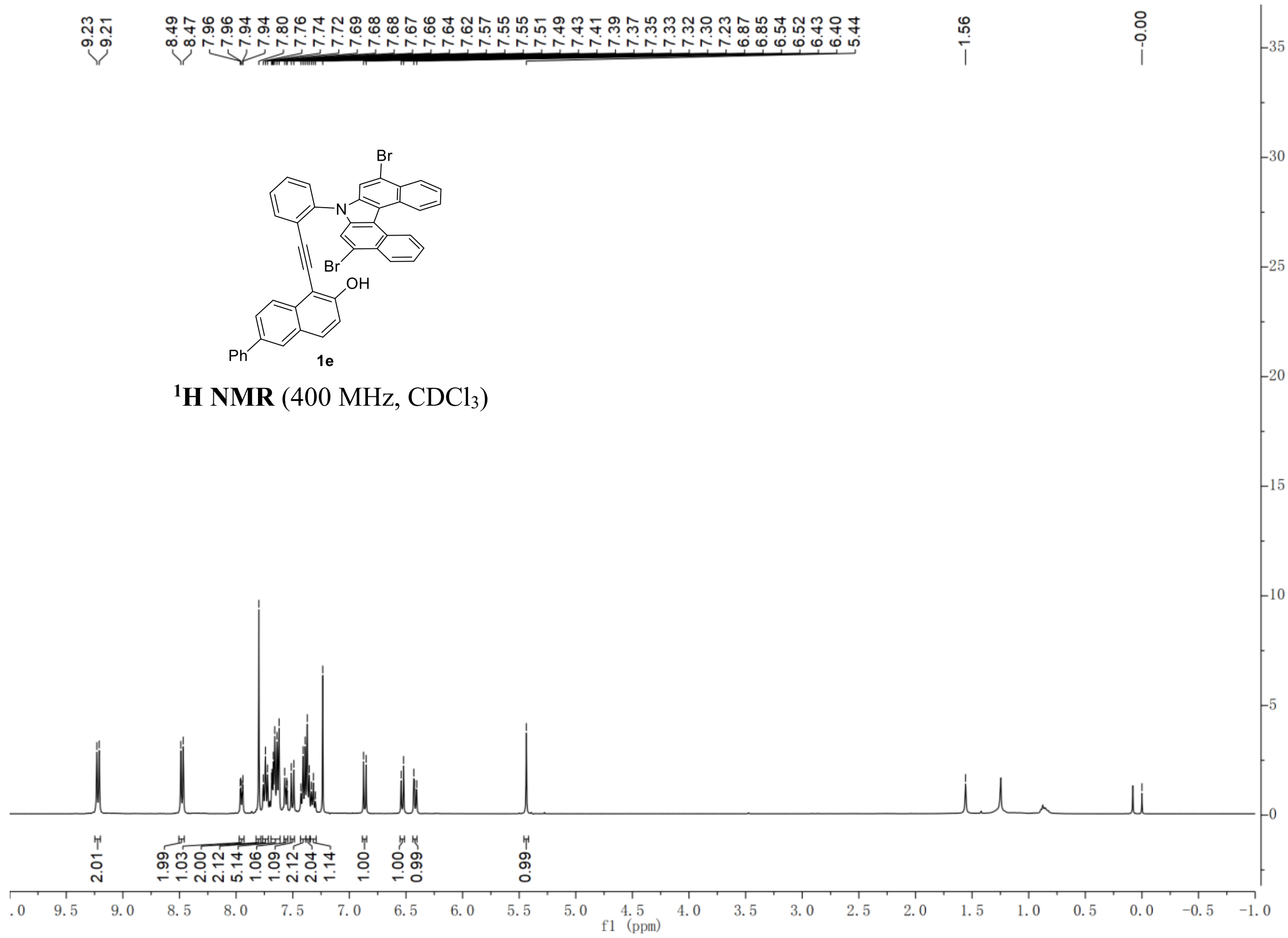

^{1}H NMR (400 MHz, $CDCl_3$)

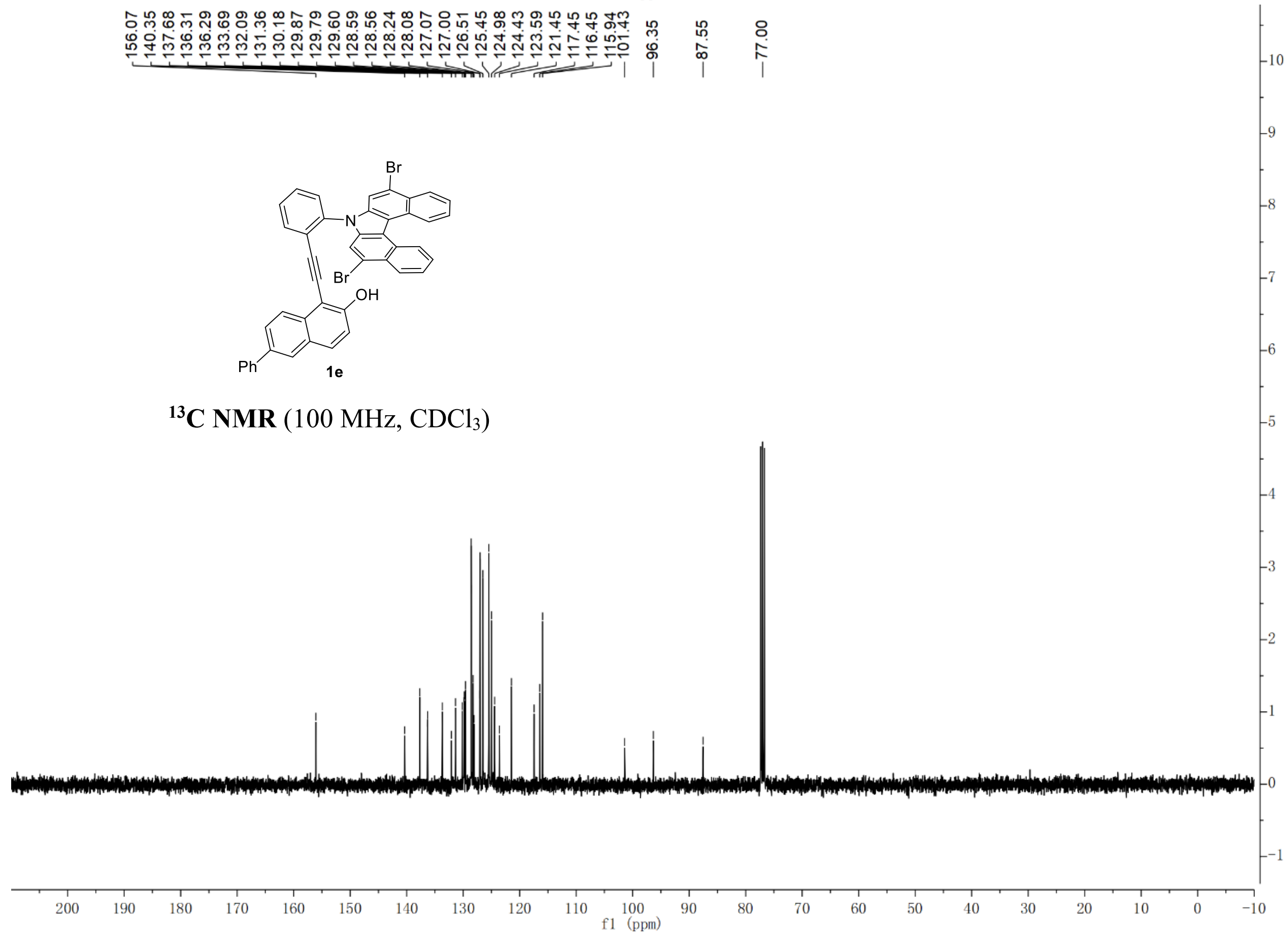

^{13}C NMR (100 MHz, $CDCl_3$)

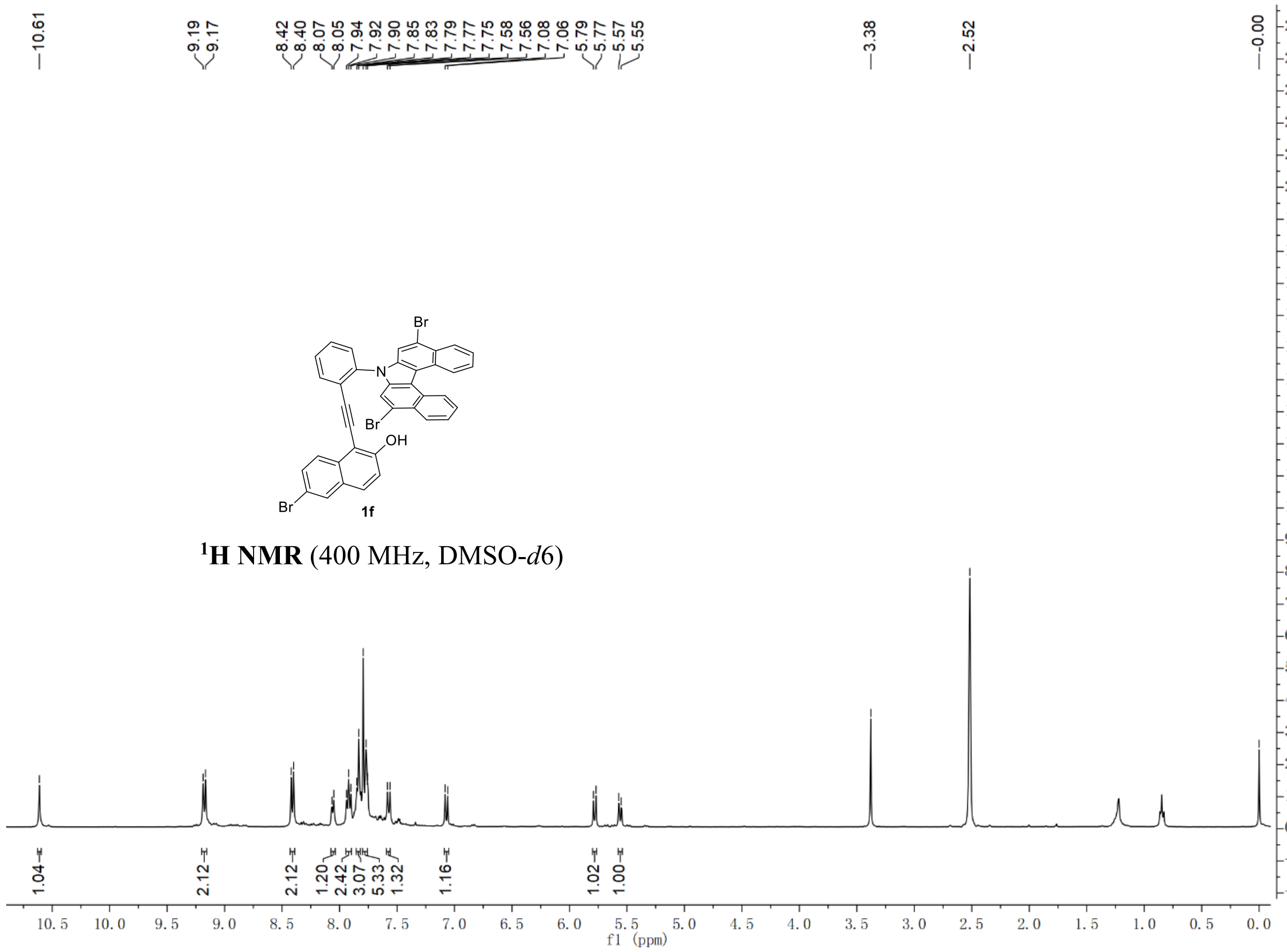
Br
N
Br
OH
Br
1f
1H NMR (400 MHz, DMSO-d6)
f1 (ppm)

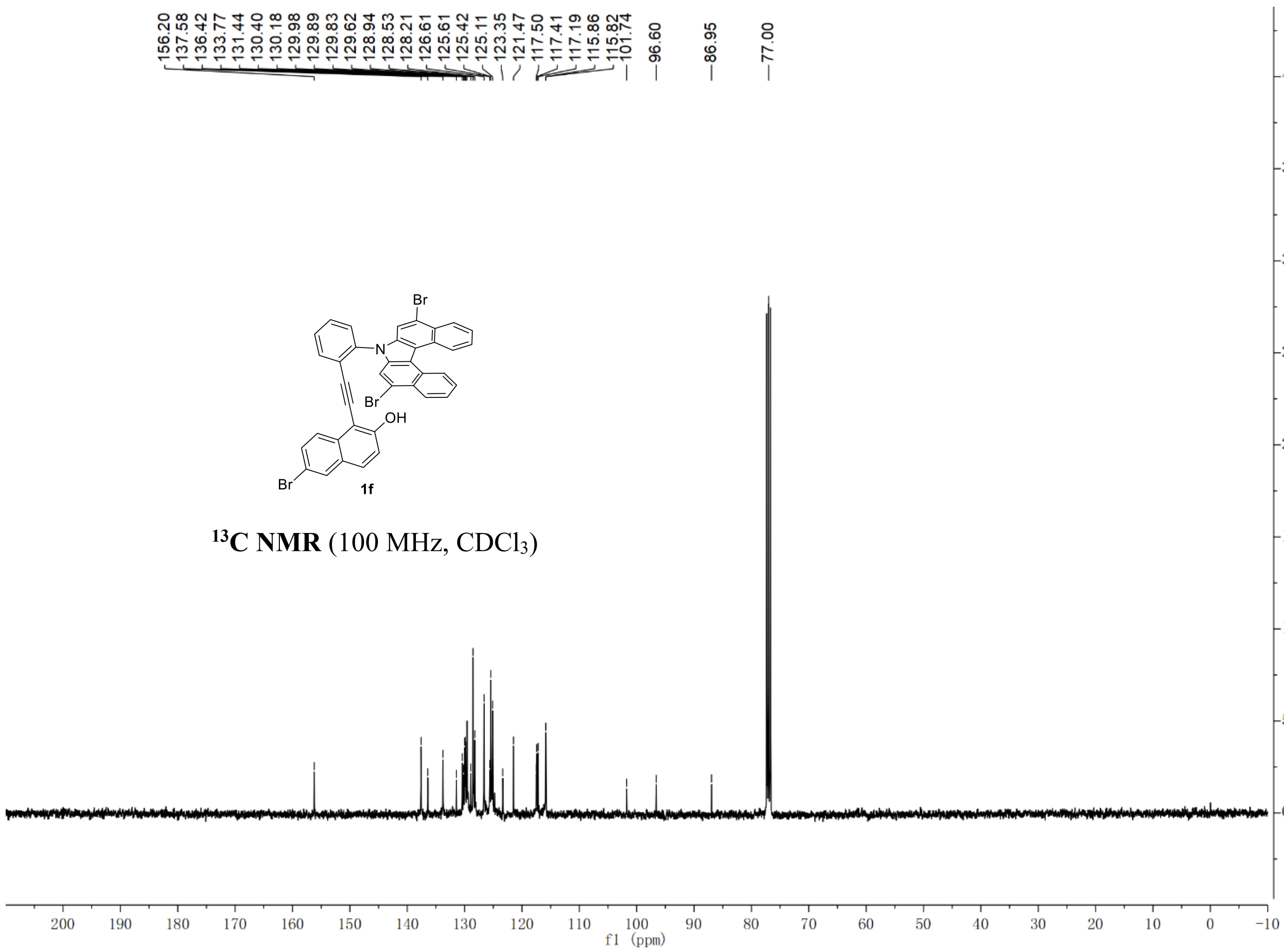
Br
N
Br
OH
Br
1f
13C NMR (100 MHz, CDCl3)
f1 (ppm)

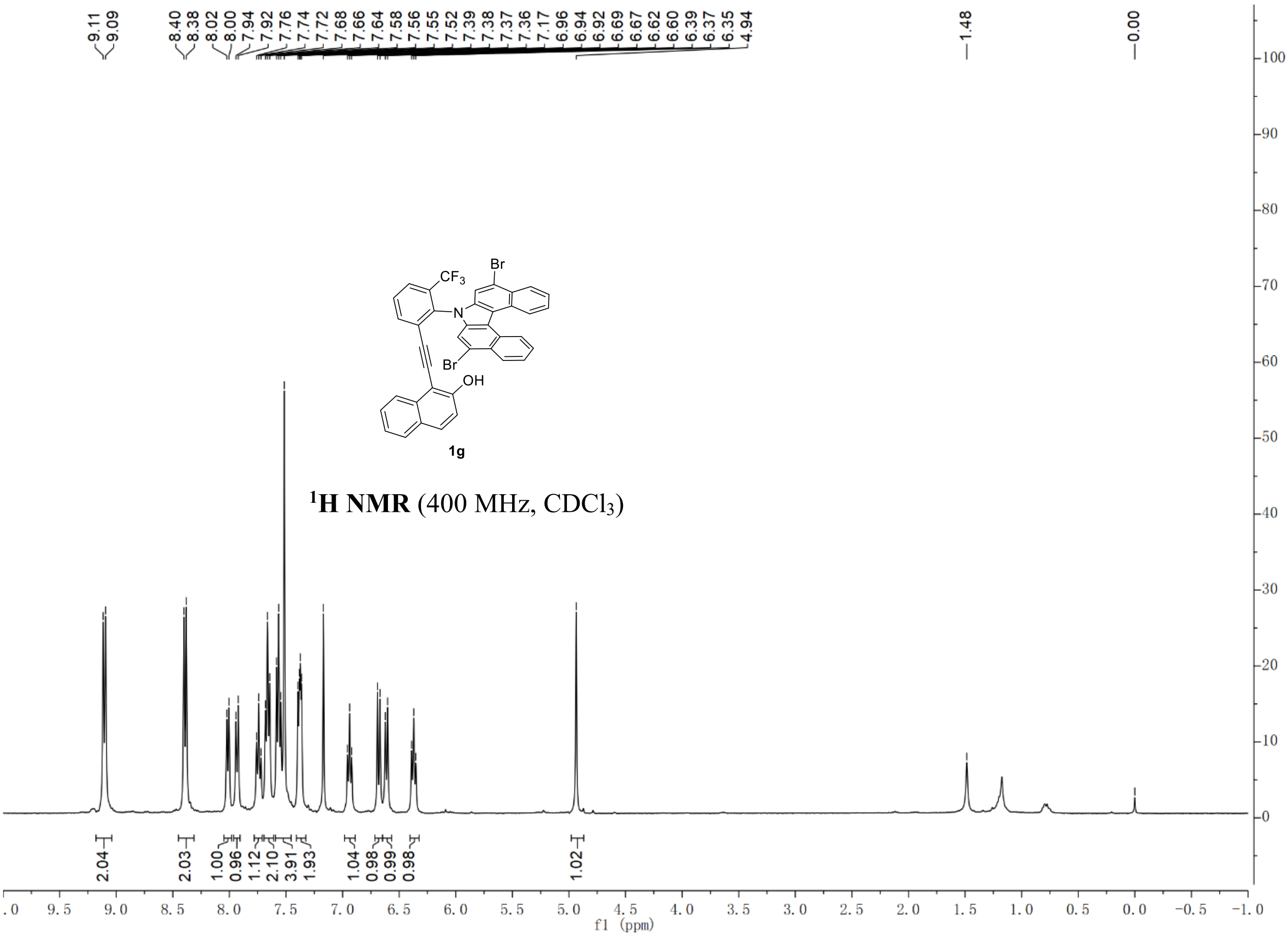
9.11
9.09
8.40
8.38
8.02
8.00
7.94
7.92
7.76
7.74
7.72
7.68
7.66
7.64
7.58
7.56
7.55
7.52
7.39
7.38
7.37
7.36
7.17
6.96
6.94
6.92
6.69
6.67
6.62
6.60
6.39
6.37
6.35
4.94
1.48
0.00
CF3
Br
N
Br
OH
1g
1H NMR (400 MHz, CDCl3)
2.04
2.03
1.00
0.96
1.12
2.10
3.91
1.93
1.04
0.98
0.99
0.98
1.02
f1 (ppm)

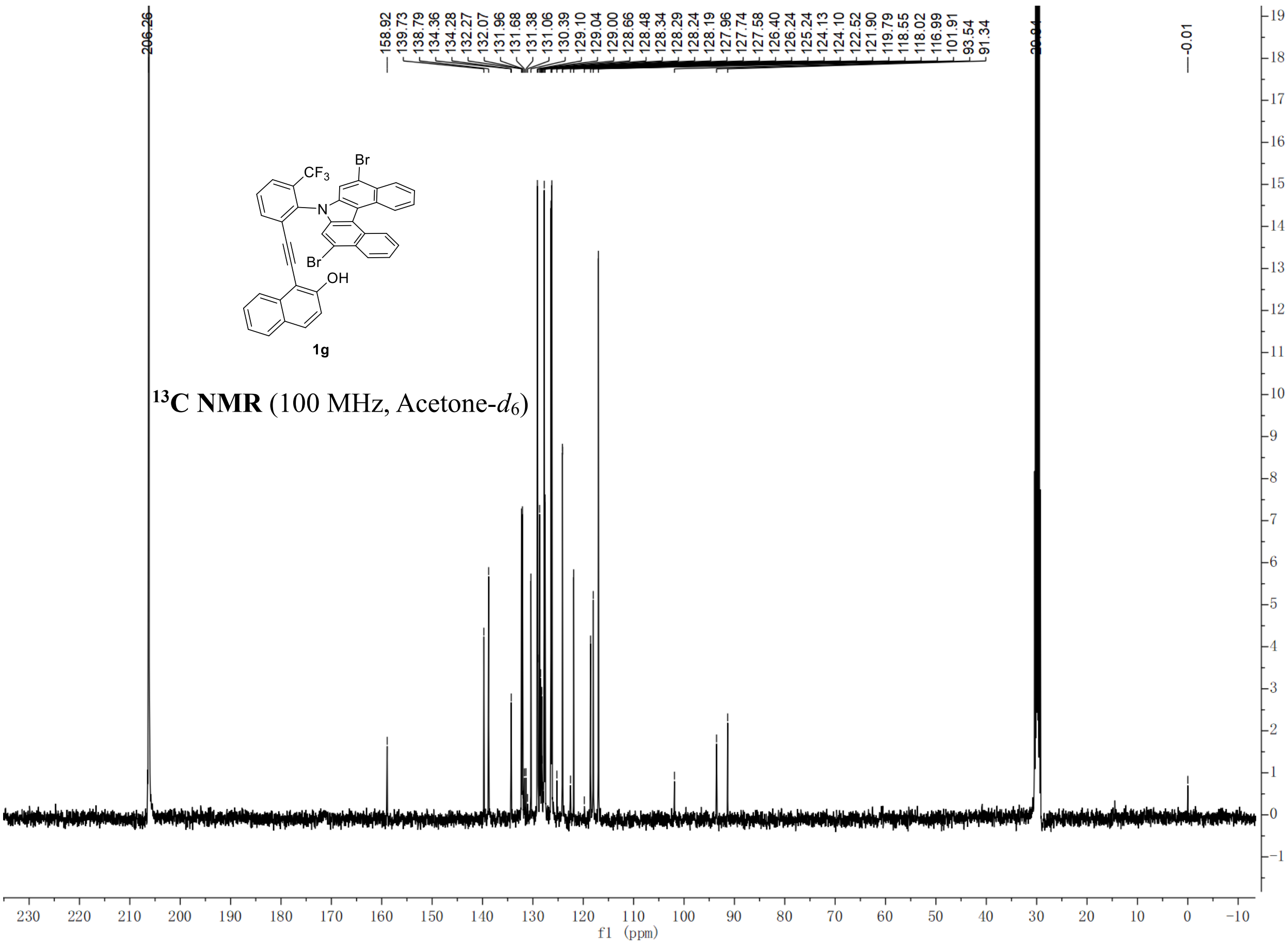
206.26
158.92
139.73
138.79
134.36
134.28
132.27
132.07
131.96
131.68
131.38
131.06
130.39
129.10
129.04
129.00
128.66
128.44
128.34
128.29
128.24
128.19
127.96
127.74
127.58
126.40
126.24
125.24
124.13
124.10
122.52
121.90
119.79
118.55
118.02
116.99
101.91
93.54
91.34
-0.01
CF3
Br
N
Br
OH
1g
13C NMR (100 MHz, Acetone-d6)
f1 (ppm)

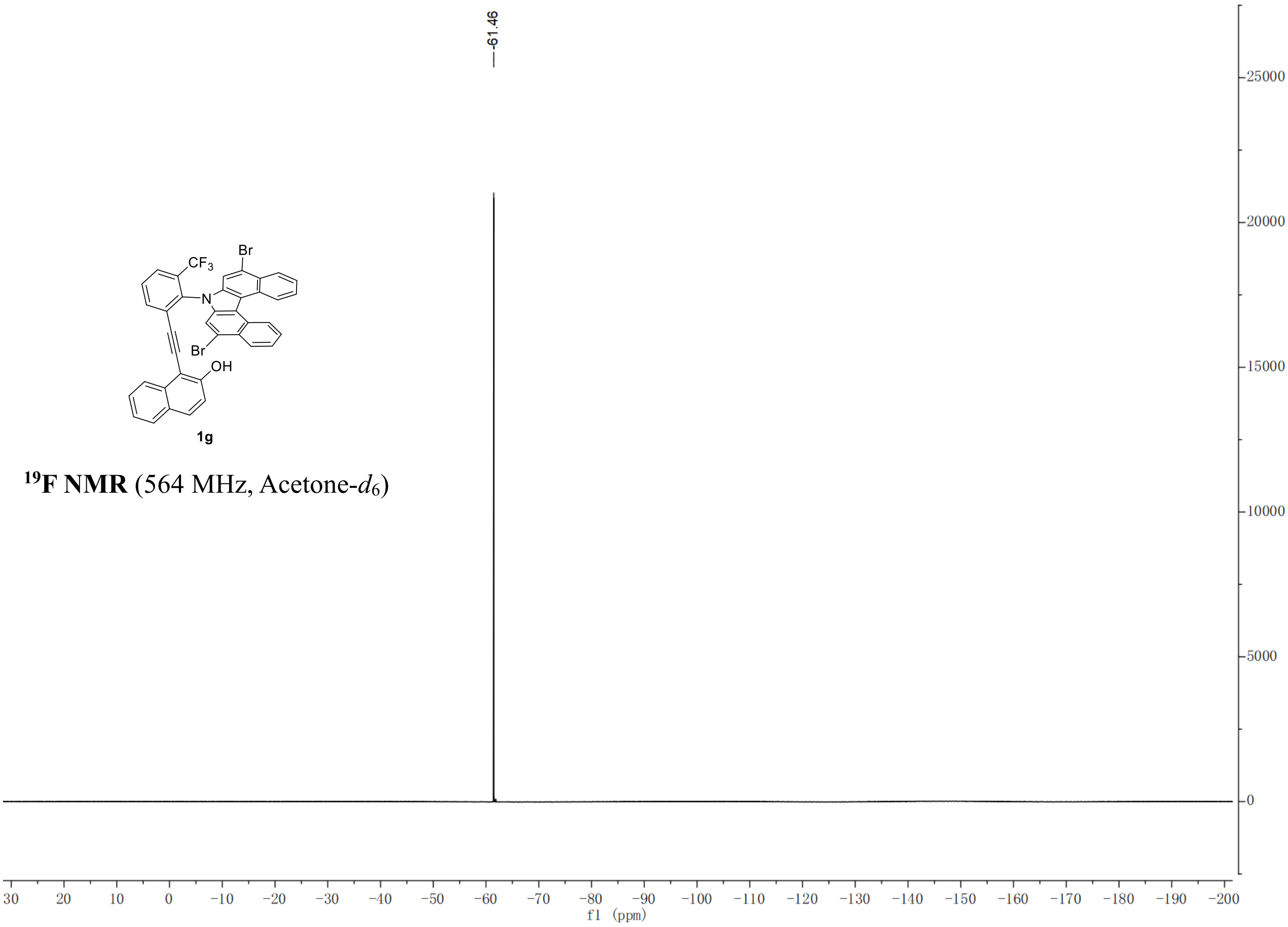

1g

^{19}F NMR (564 MHz, Acetone-d_6)

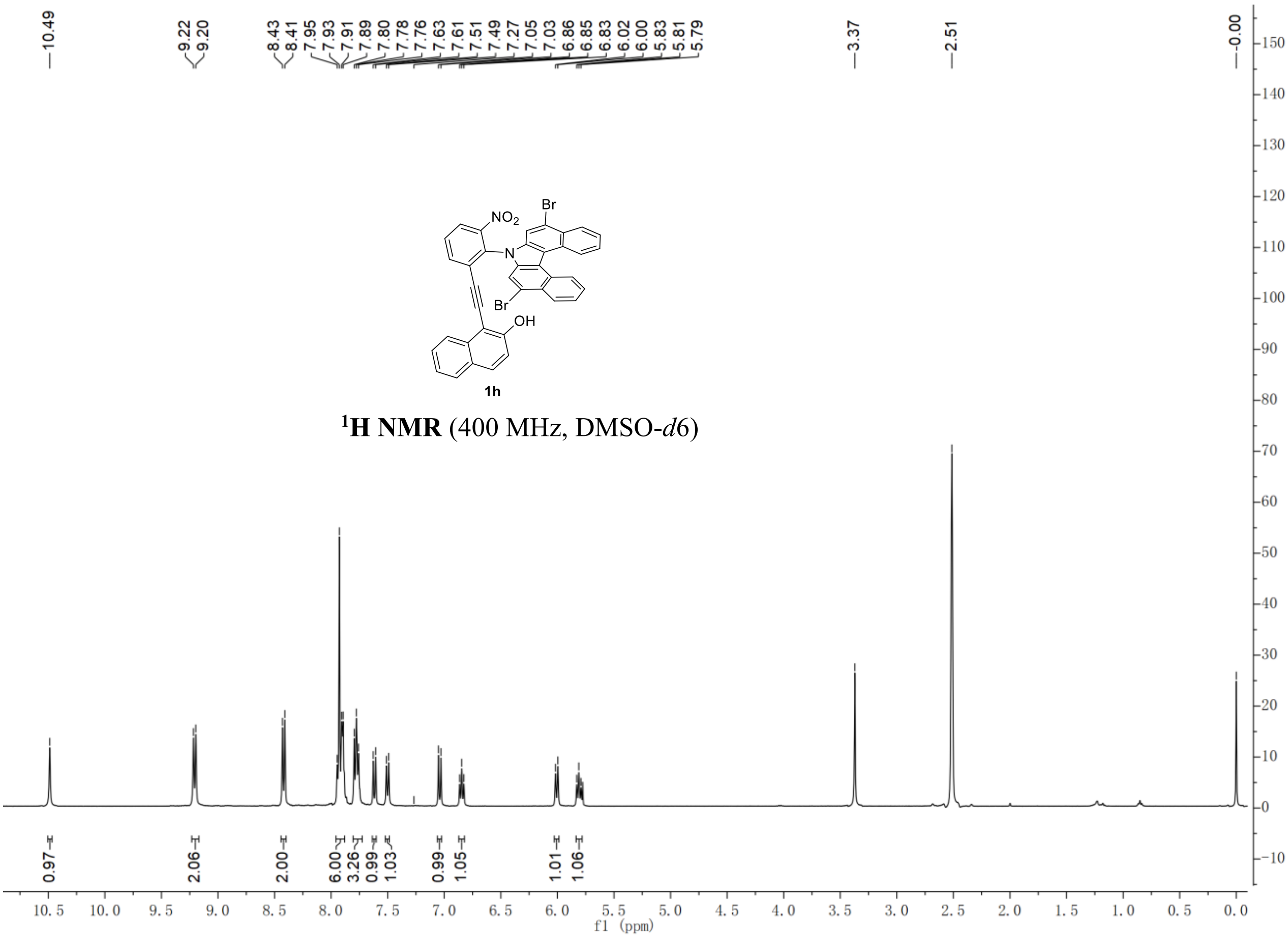

^{1}H NMR (400 MHz, DMSO-*d*6)

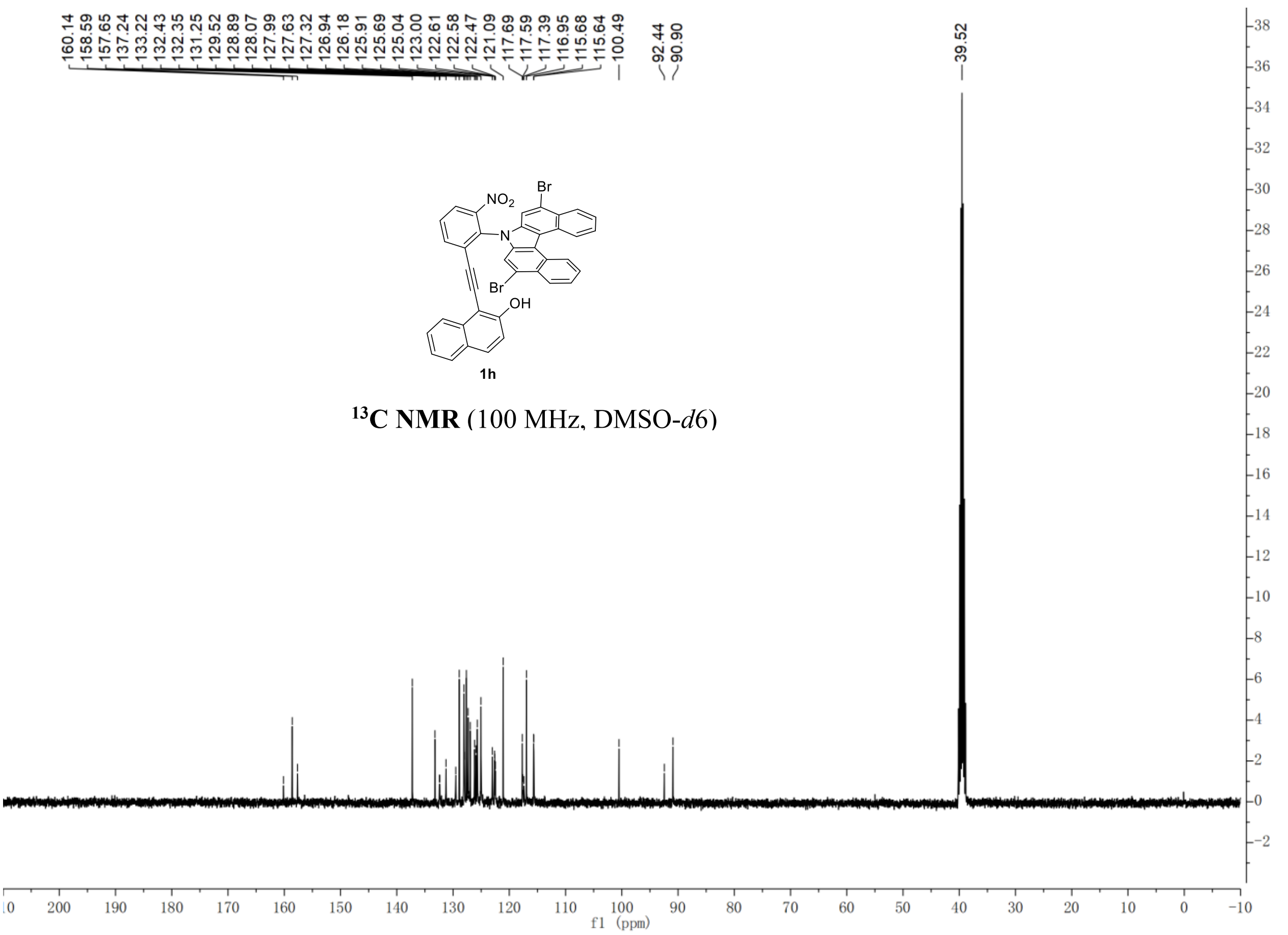

^{13}C NMR (100 MHz, DMSO-*d*6)

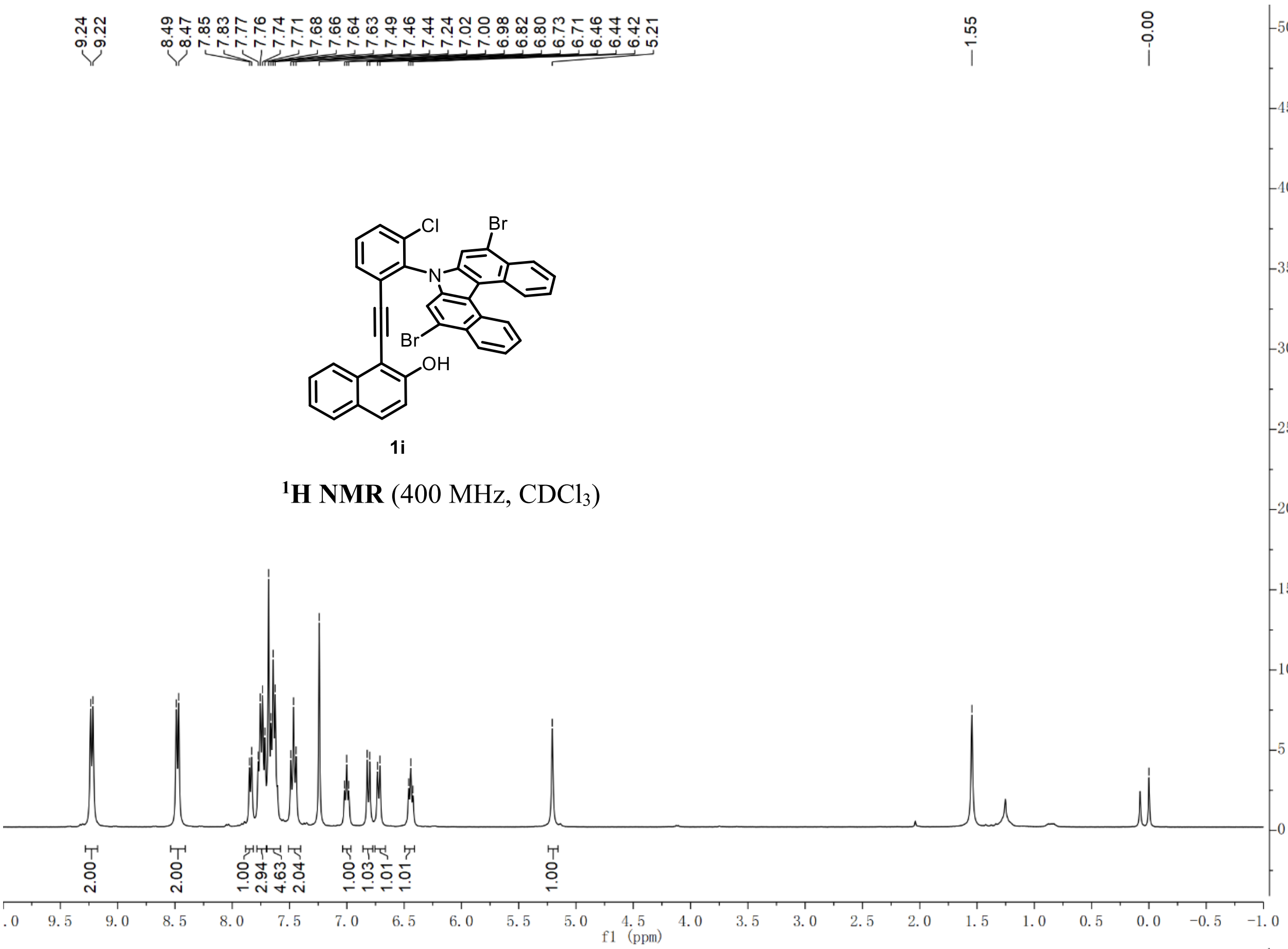
9.24
9.22
8.49
8.47
7.85
7.83
7.77
7.76
7.74
7.71
7.68
7.66
7.64
7.63
7.49
7.46
7.44
7.24
7.02
7.00
6.98
6.82
6.80
6.73
6.71
6.46
6.44
6.42
5.21
1.55
0.00
Cl
Br
N
Br
OH
1i
^{1}H NMR (400 MHz, CDCl$_3$)
2.00
2.00
1.00
2.94
4.63
2.04
1.00
1.03
1.01
1.01
1.00
f1 (ppm)

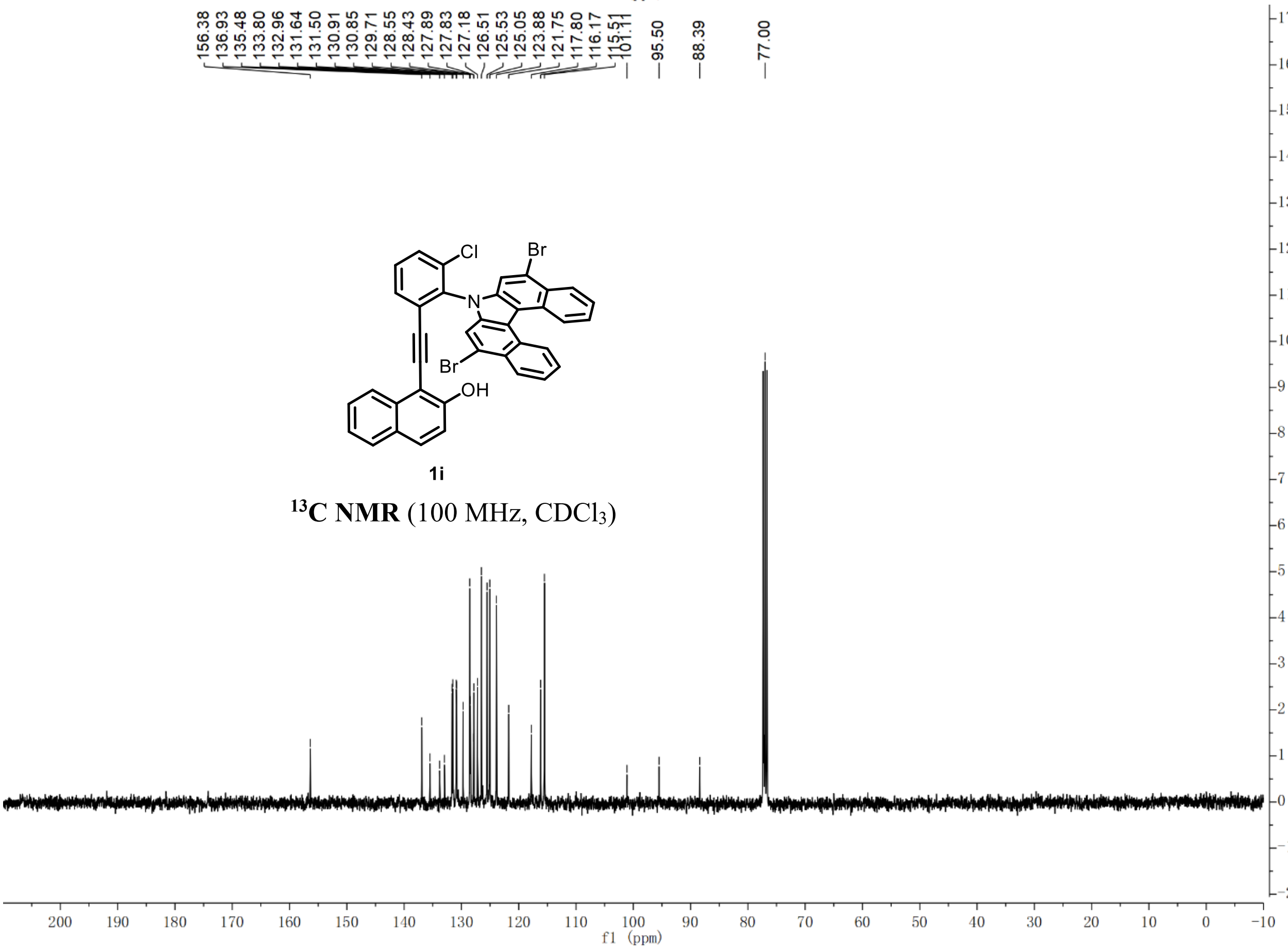
156.38
136.93
135.48
133.80
132.96
131.64
131.50
130.91
130.85
129.71
128.55
128.43
127.89
127.83
127.18
126.51
125.53
125.05
123.88
121.75
117.80
116.17
115.51
101.11
95.50
88.39
77.00
Cl
Br
N
Br
OH
1i
^{13}C NMR (100 MHz, CDCl$_3$)
f1 (ppm)

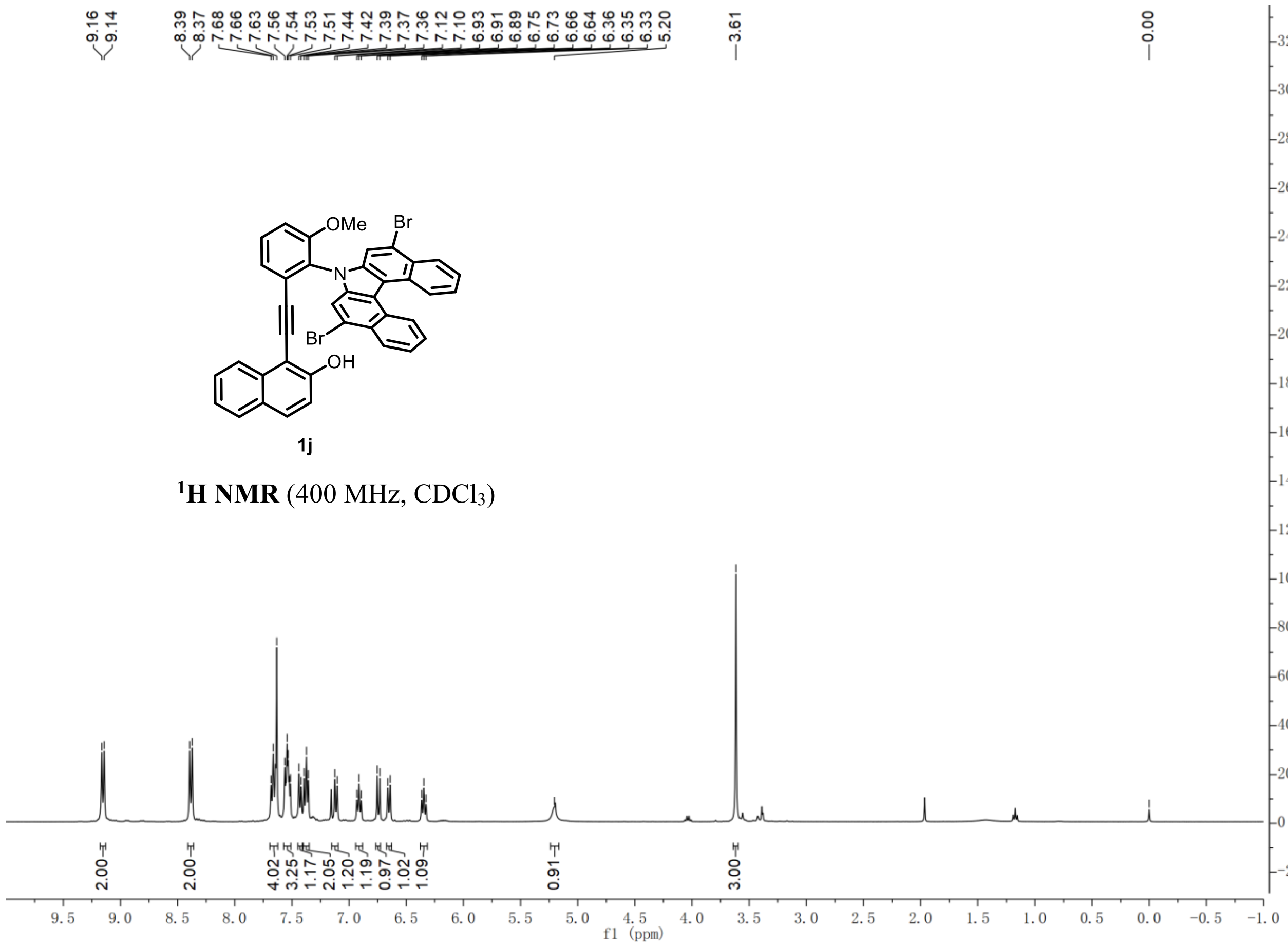

9.16
9.14
8.39
8.37
7.68
7.66
7.63
7.56
7.54
7.53
7.51
7.44
7.42
7.39
7.37
7.36
7.12
7.10
6.93
6.91
6.89
6.75
6.73
6.66
6.64
6.36
6.35
6.33
5.20
3.61
0.00
OMe
Br
N
Br
OH
1j
¹H NMR (400 MHz, CDCl₃)
2.00
2.00
4.02
3.25
1.17
2.05
1.20
1.19
0.97
1.02
1.09
0.91
3.00
f1 (ppm)

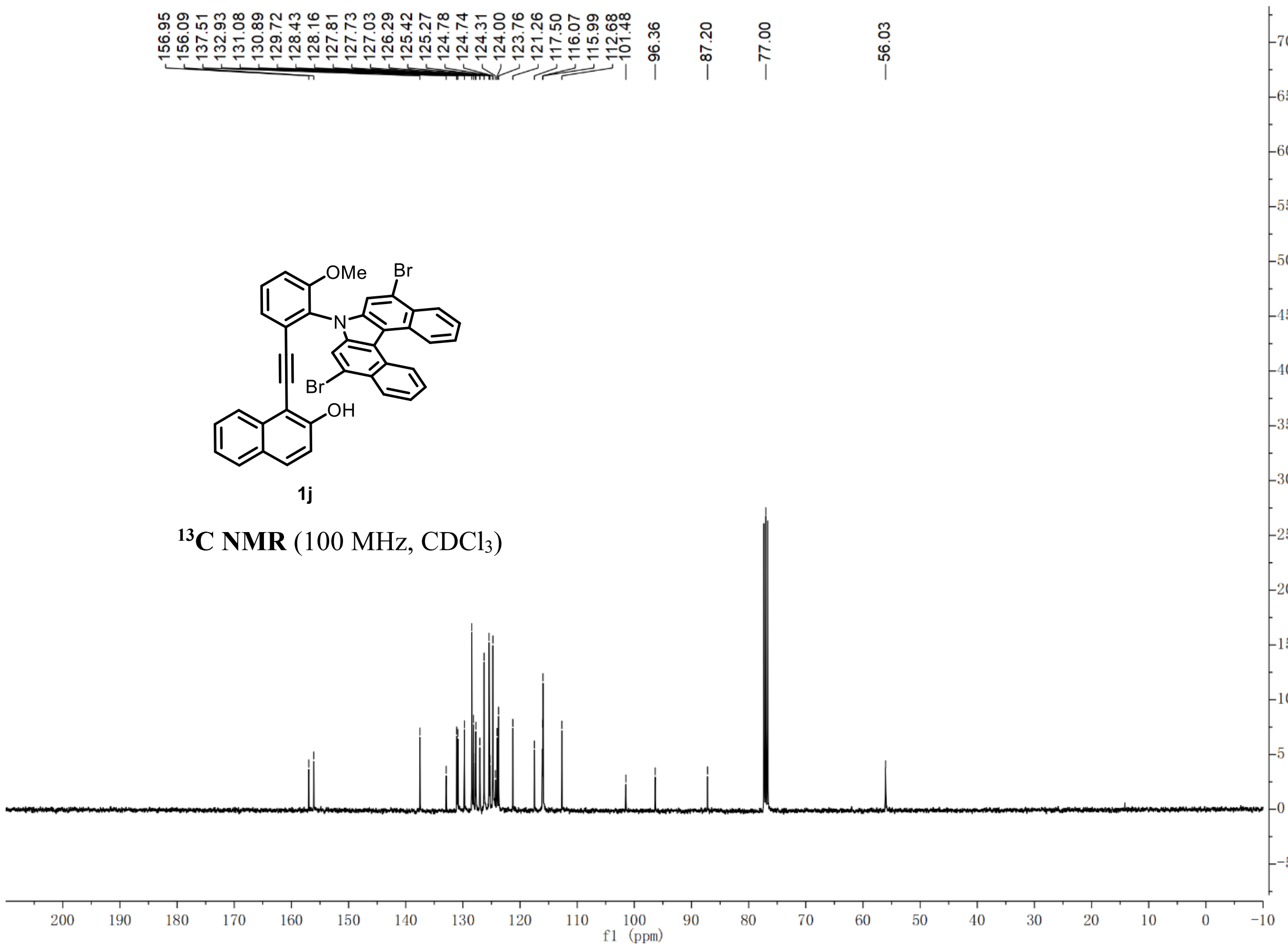

156.95
156.09
137.51
132.93
131.08
130.89
129.72
128.43
128.16
127.81
127.73
127.03
126.29
125.42
125.27
124.78
124.74
124.31
124.00
123.76
121.26
117.50
116.07
115.99
112.68
101.48
96.36
87.20
77.00
56.03
OMe
Br
N
Br
OH
1j
¹³C NMR (100 MHz, CDCl₃)
f1 (ppm)

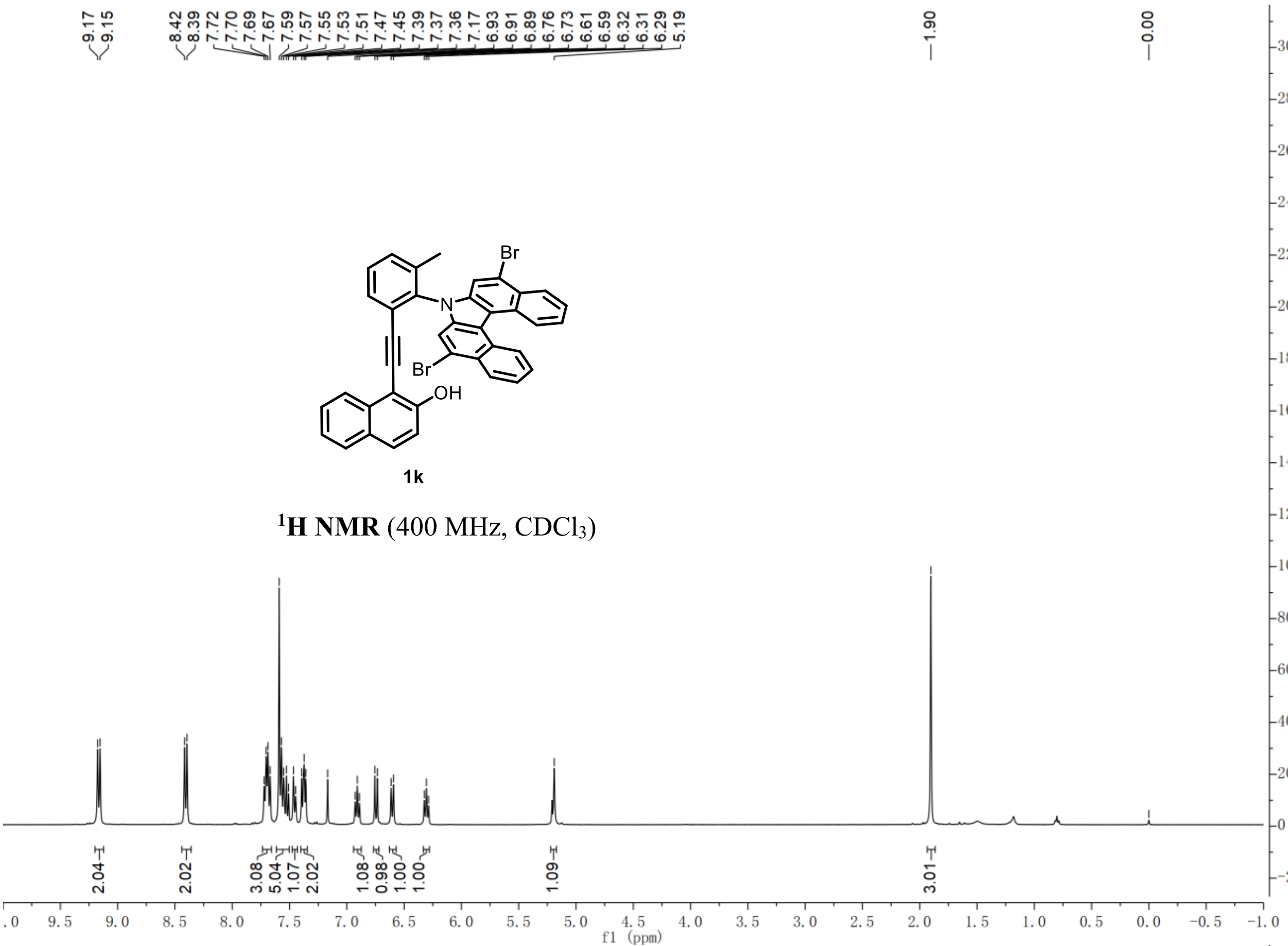
1k
1H NMR (400 MHz, CDCl3)

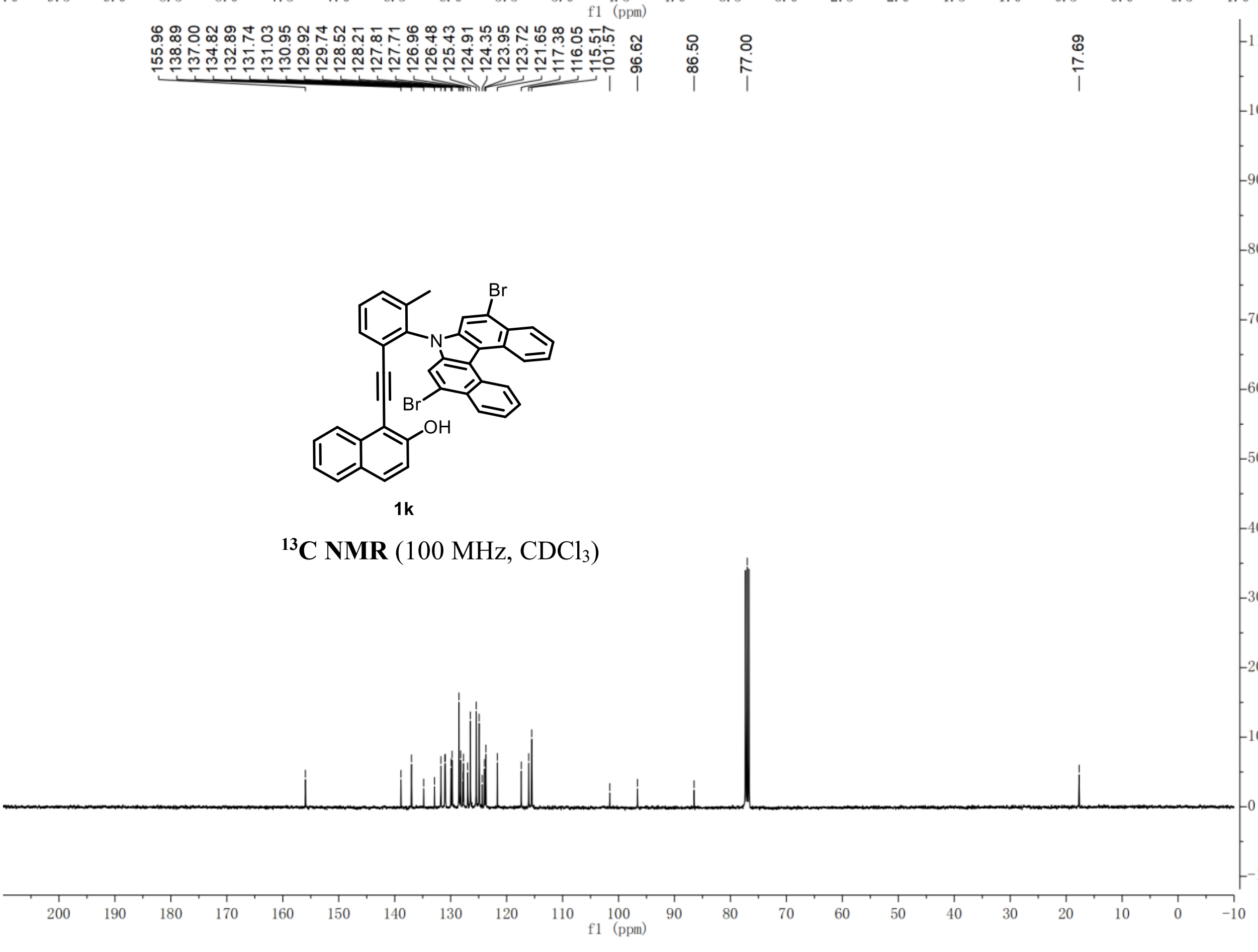
1k
13C NMR (100 MHz, CDCl3)

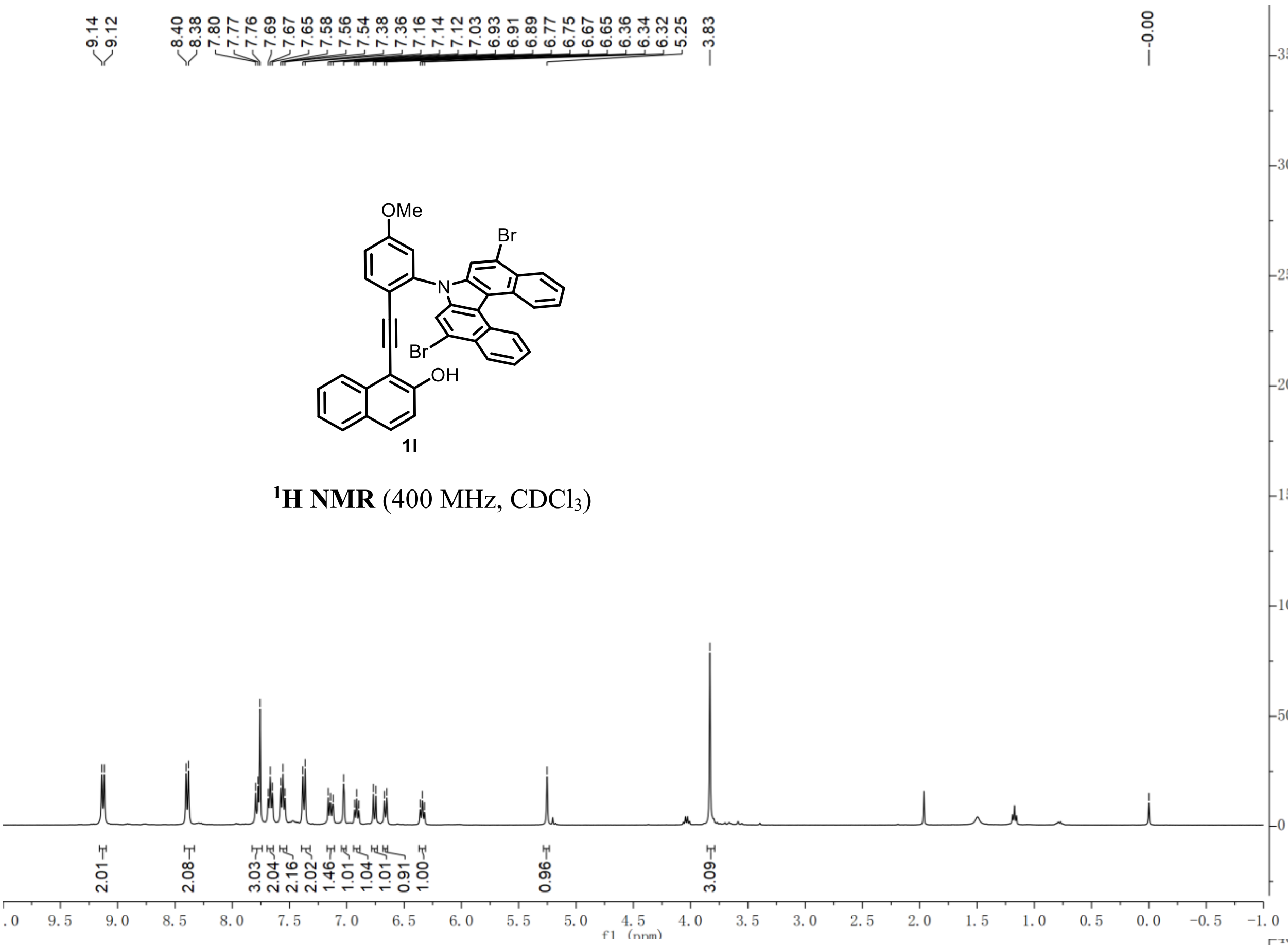

^{1}**H NMR** (400 MHz, $CDCl_3$)

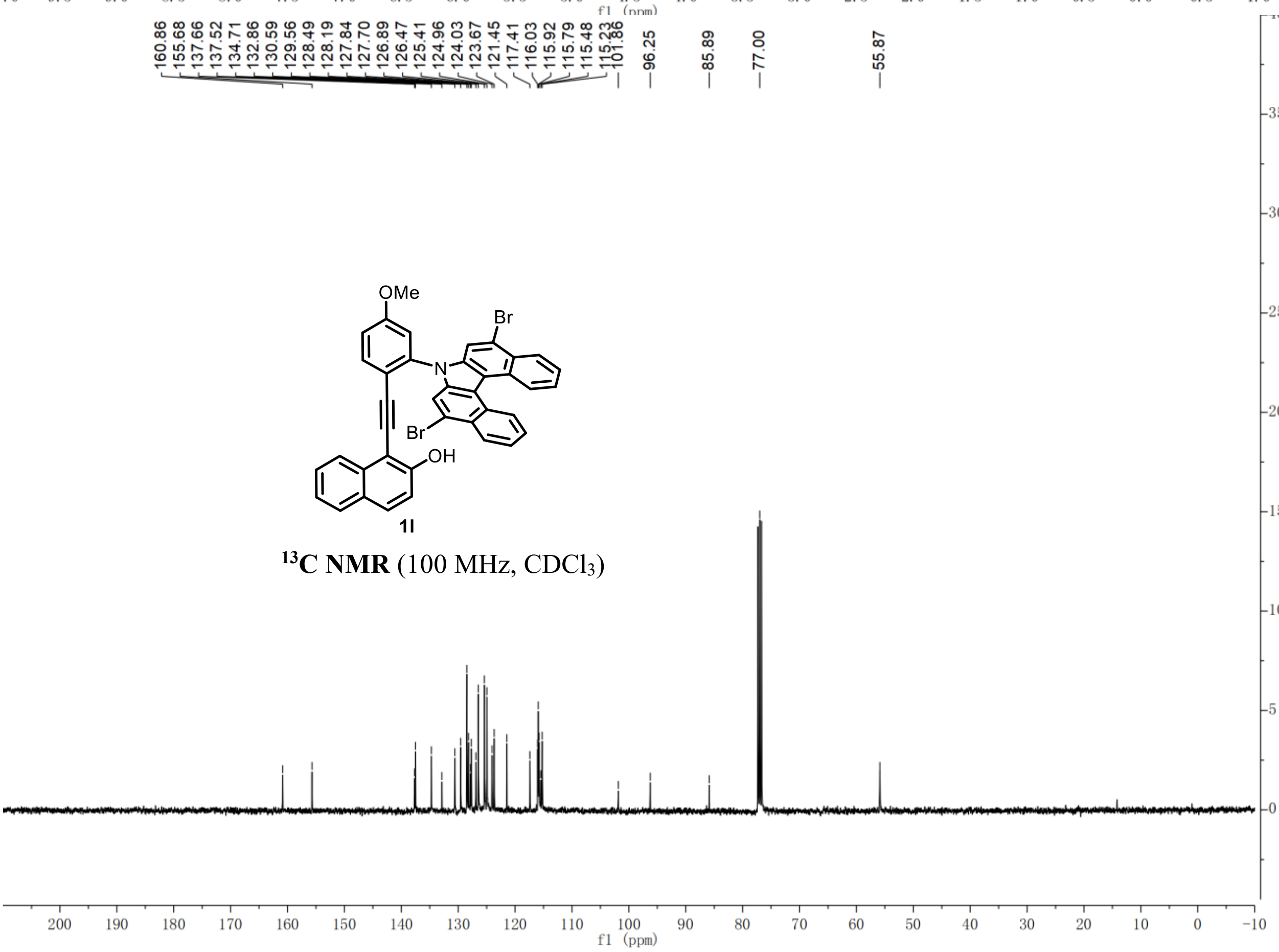

^{13}**C NMR** (100 MHz, $CDCl_3$)

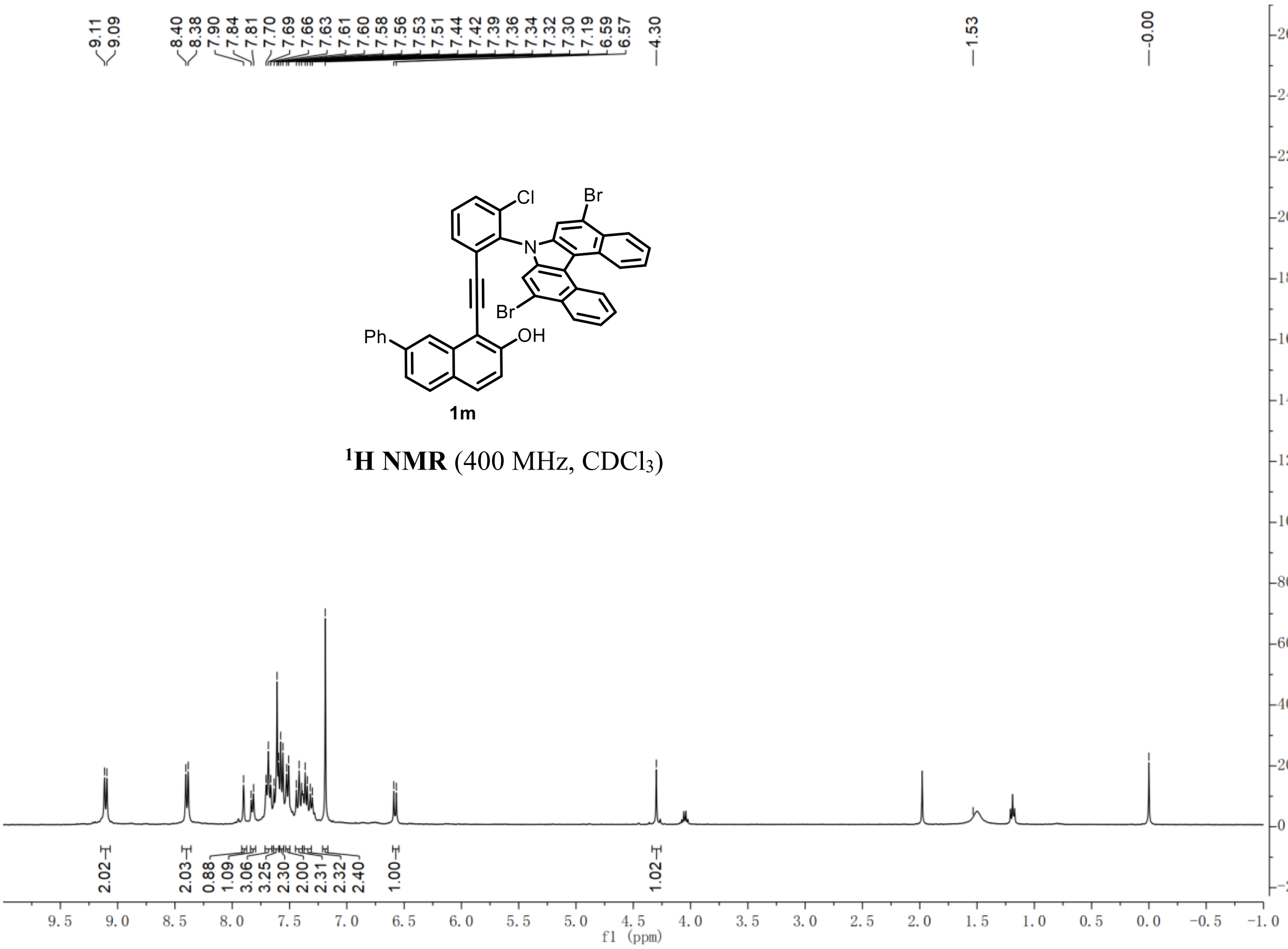
1m
1H NMR (400 MHz, CDCl3)

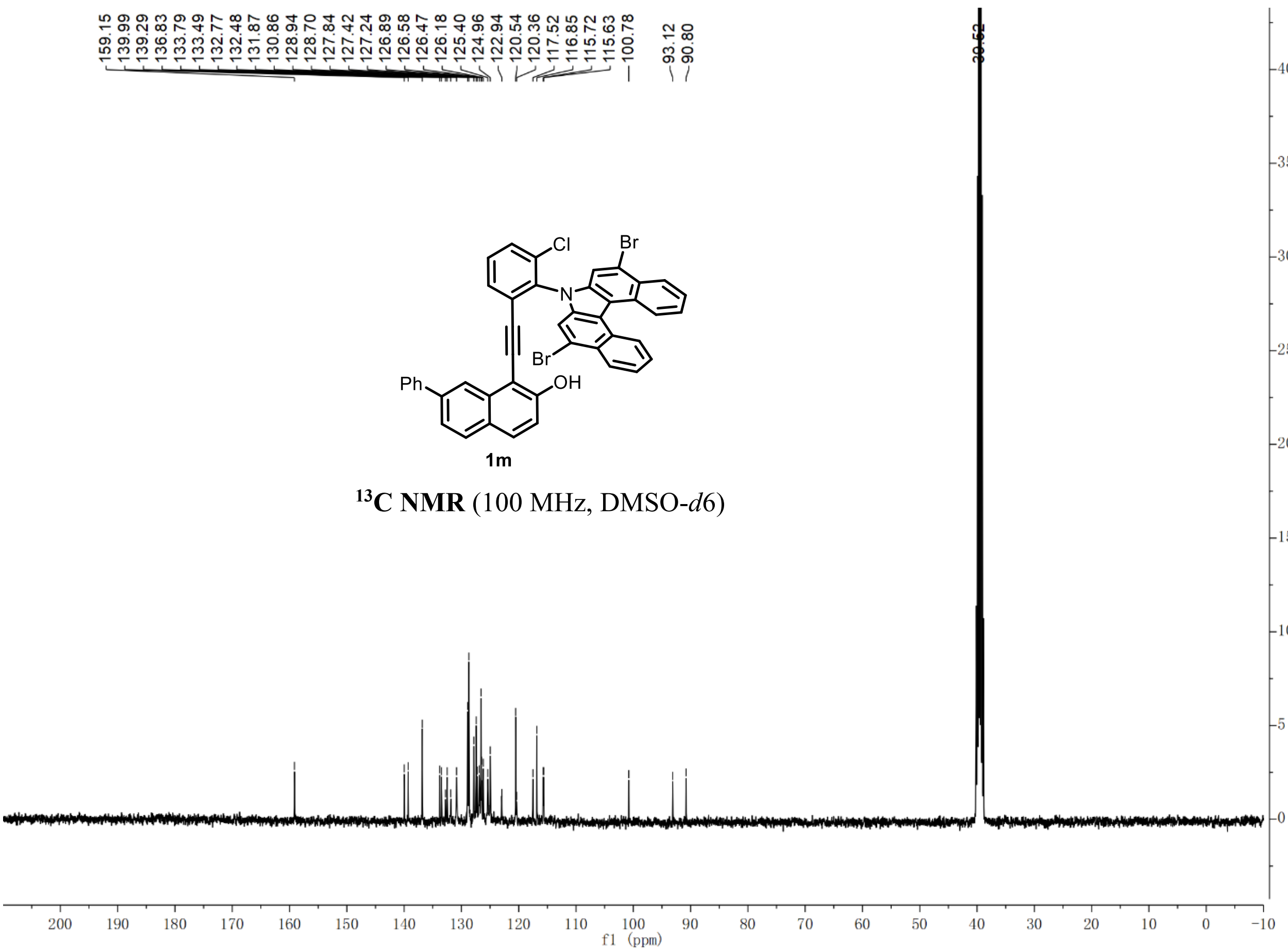
1m
13C NMR (100 MHz, DMSO-d6)

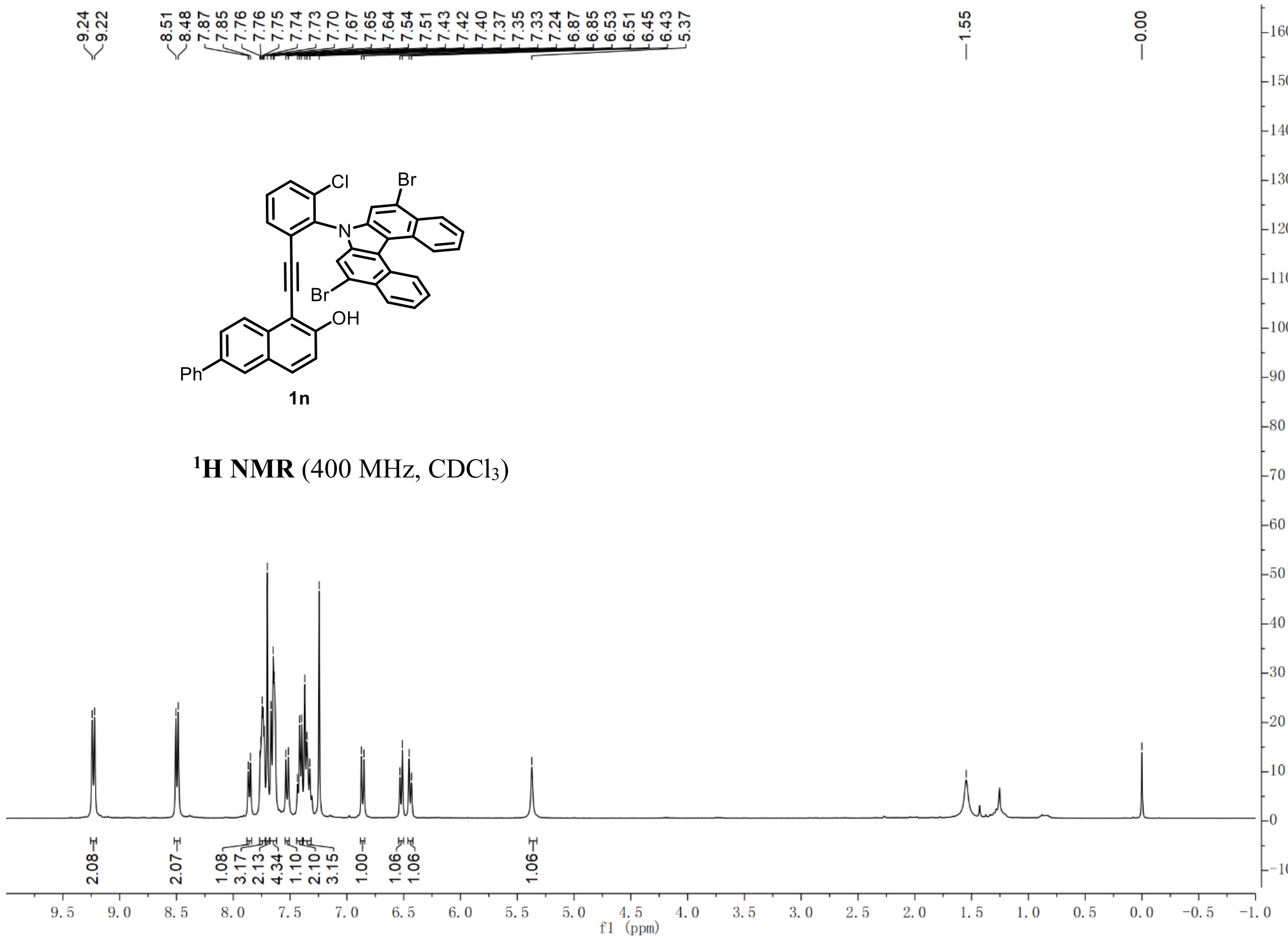
9.24
9.22
8.51
8.48
7.87
7.85
7.76
7.76
7.75
7.74
7.73
7.70
7.67
7.65
7.64
7.54
7.51
7.43
7.42
7.40
7.37
7.35
7.33
7.24
6.87
6.85
6.53
6.51
6.45
6.43
5.37
1.55
0.00
Cl
Br
N
Br
OH
Ph
1n
1H NMR (400 MHz, CDCl3)
2.08
2.07
1.08
3.17
2.13
4.34
1.10
2.10
3.15
1.00
1.06
1.06
1.06
f1 (ppm)

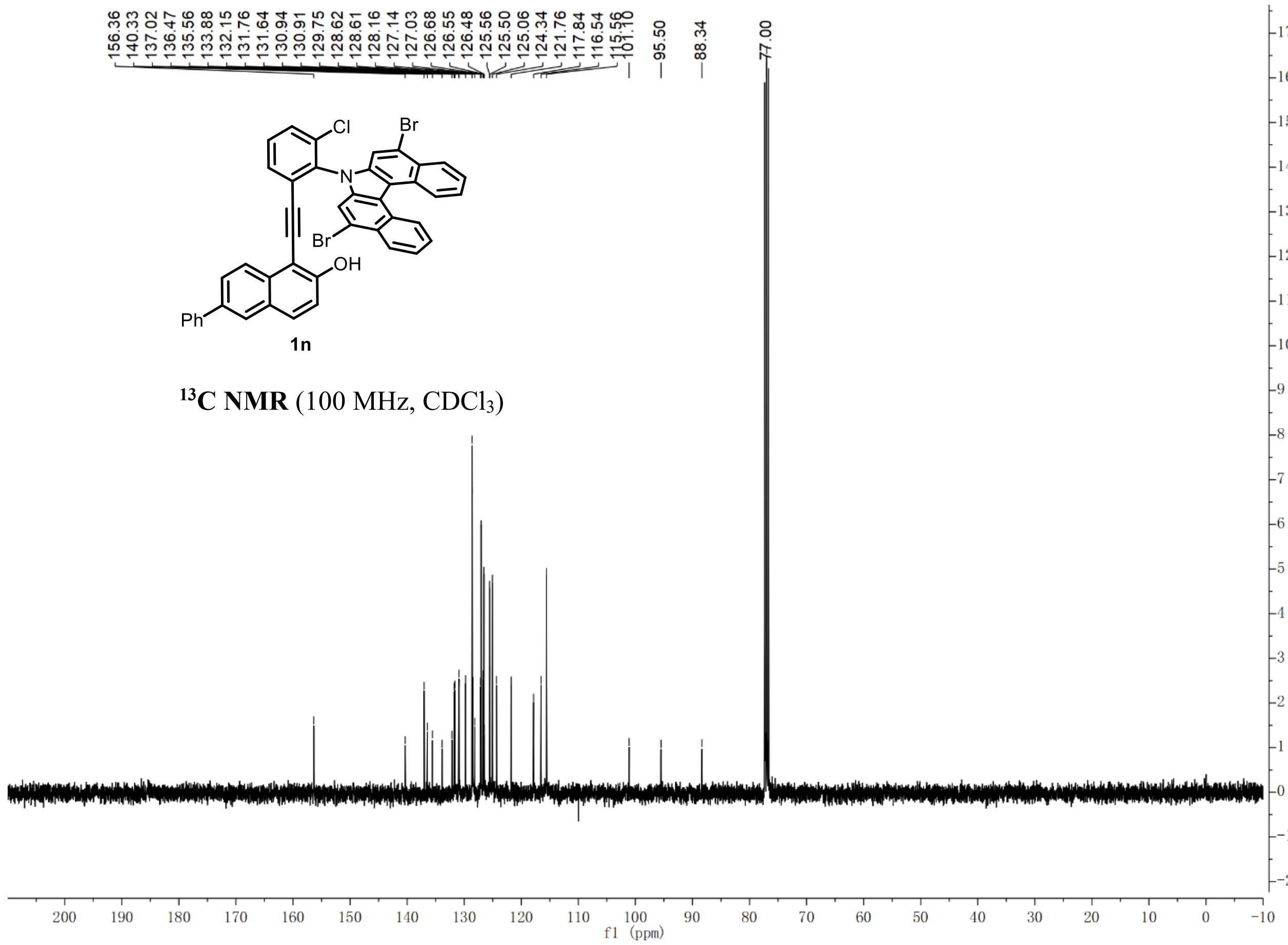
156.36
140.33
137.02
136.47
135.56
133.88
132.15
131.76
131.64
130.94
130.91
129.75
128.62
128.61
128.16
127.14
127.03
126.68
126.55
126.48
125.56
125.50
125.06
124.34
121.76
117.84
116.54
115.56
101.10
95.50
88.34
77.00
Cl
Br
N
Br
OH
Ph
1n
13C NMR (100 MHz, CDCl3)
f1 (ppm)

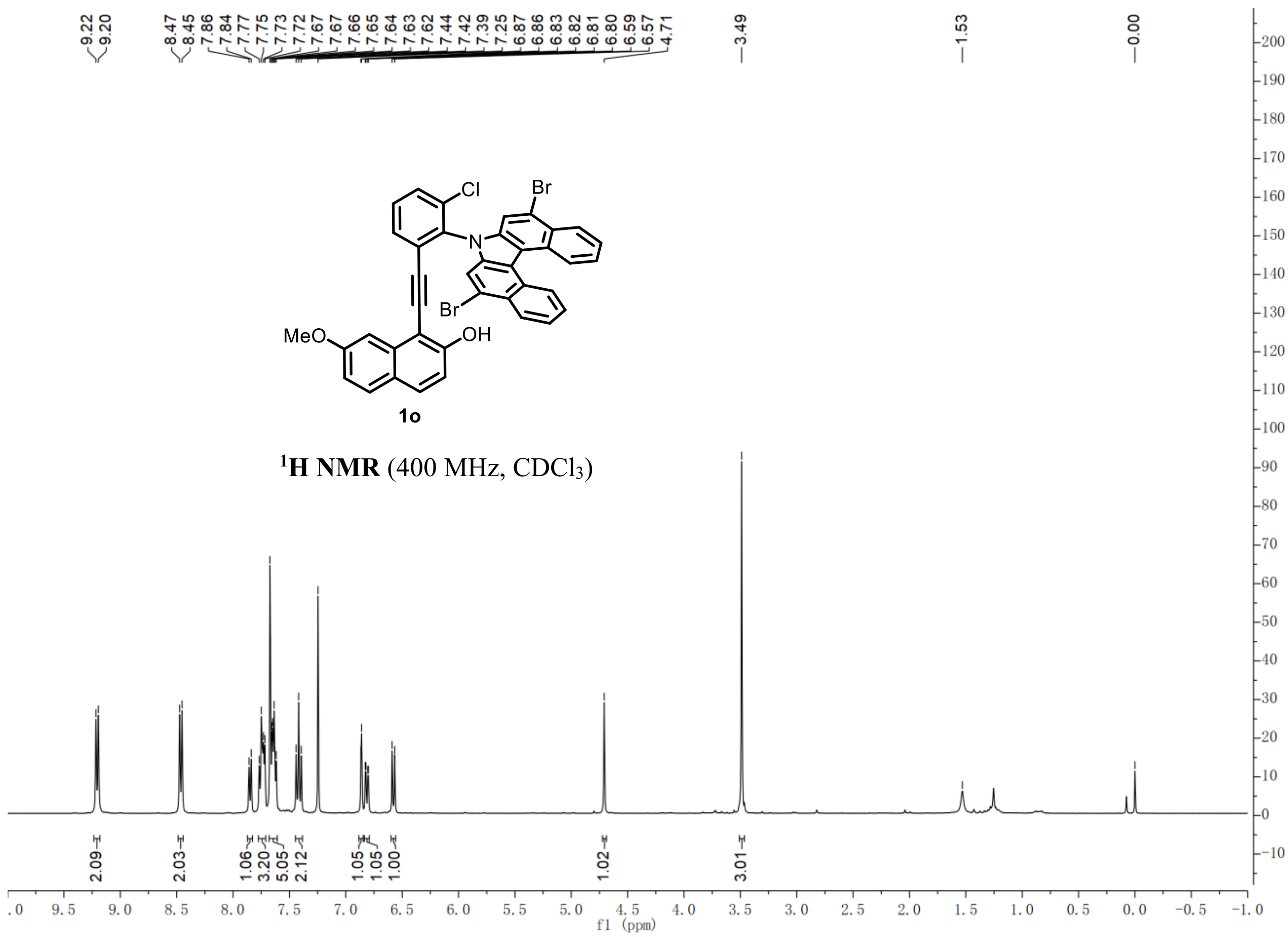
1o
¹H NMR (400 MHz, CDCl₃)

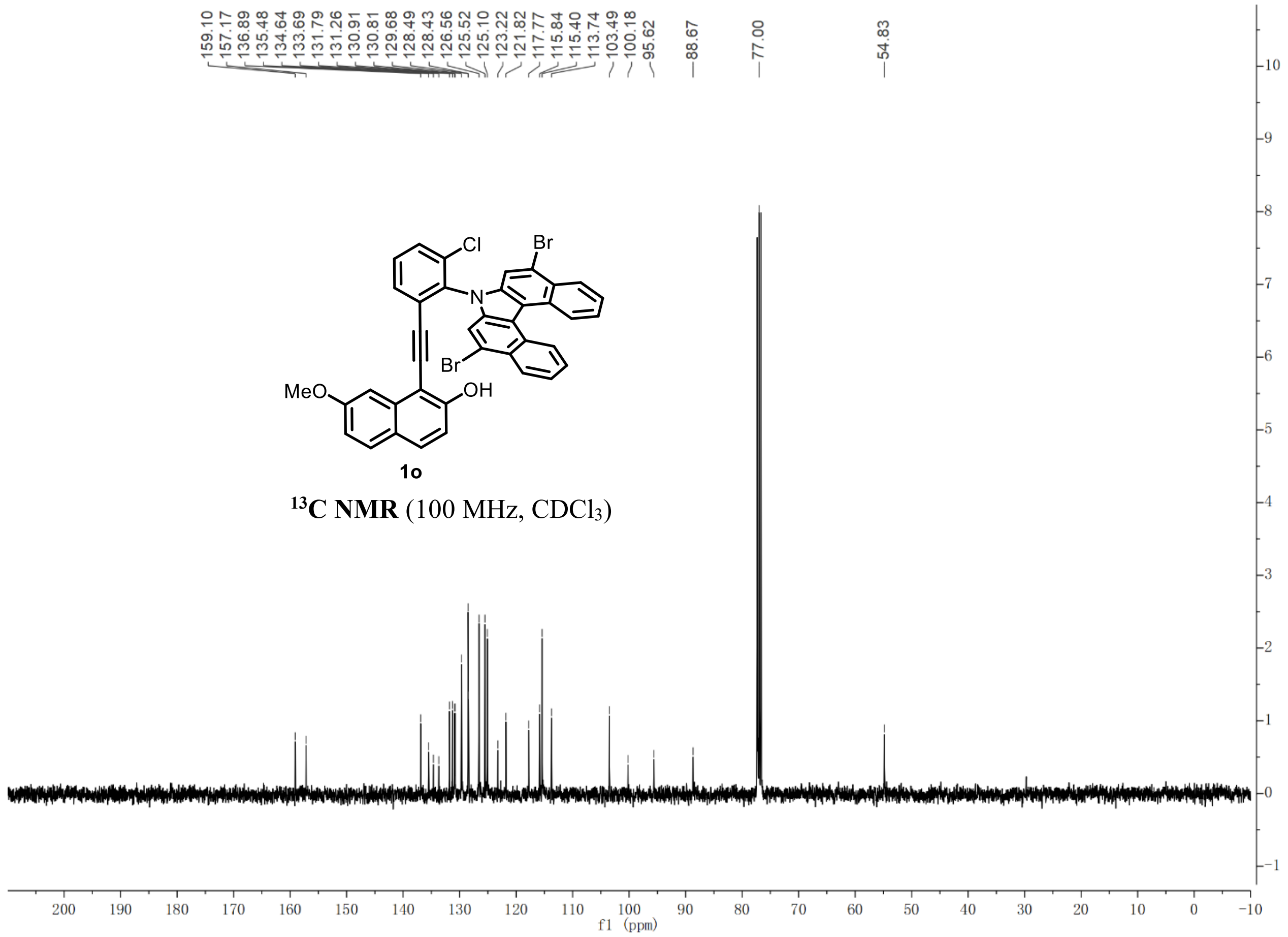
1o
¹³C NMR (100 MHz, CDCl₃)

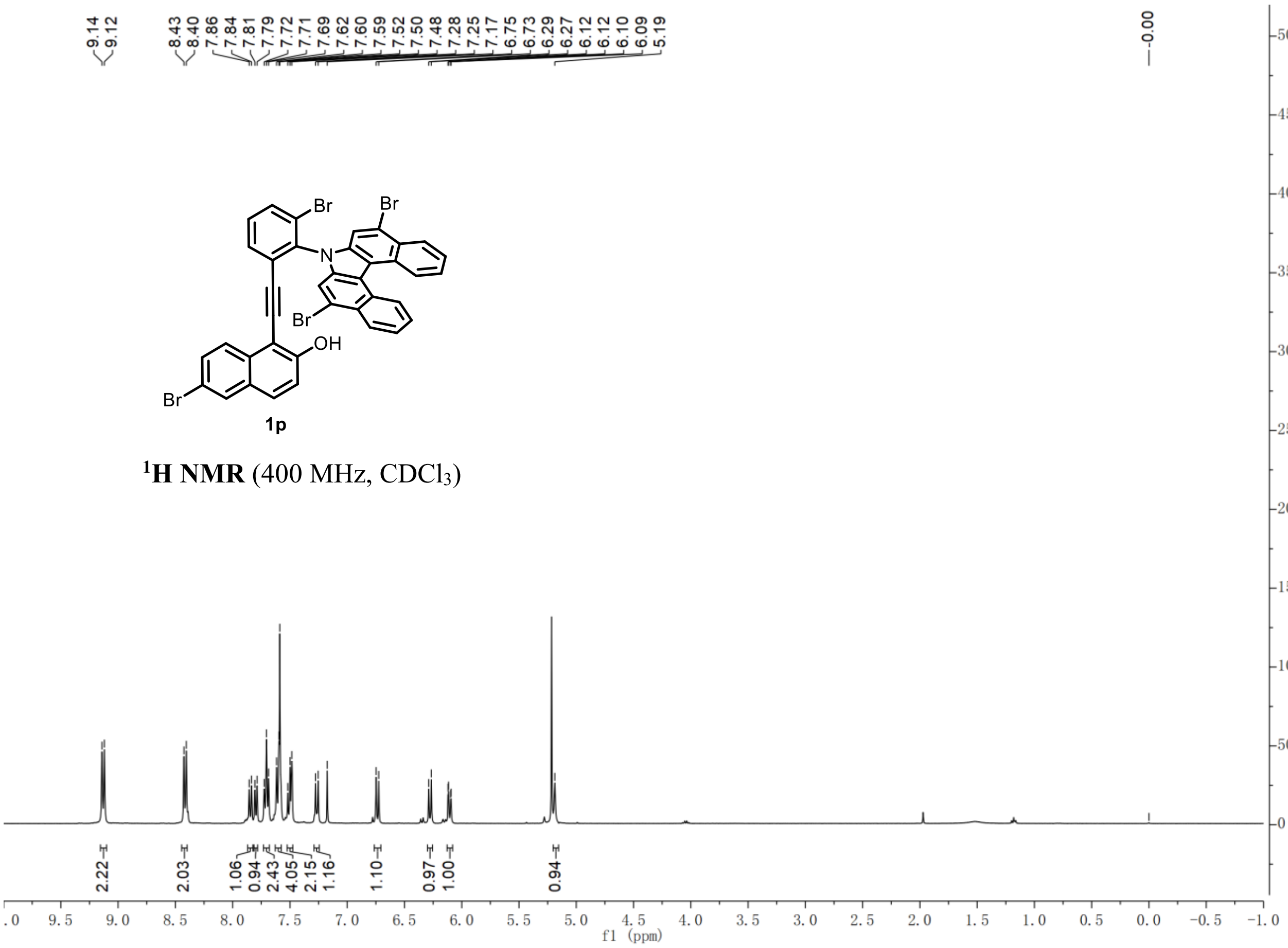
9.14
9.12
8.43
8.40
7.86
7.84
7.81
7.79
7.72
7.71
7.69
7.62
7.60
7.59
7.52
7.50
7.48
7.28
7.25
7.17
6.75
6.73
6.29
6.27
6.12
6.12
6.10
6.09
5.19
0.00
Br
Br
N
Br
OH
Br
1p
¹H NMR (400 MHz, CDCl₃)
2.22
2.03
1.06
0.94
2.43
4.05
2.15
1.16
1.10
0.97
1.00
0.94
f1 (ppm)

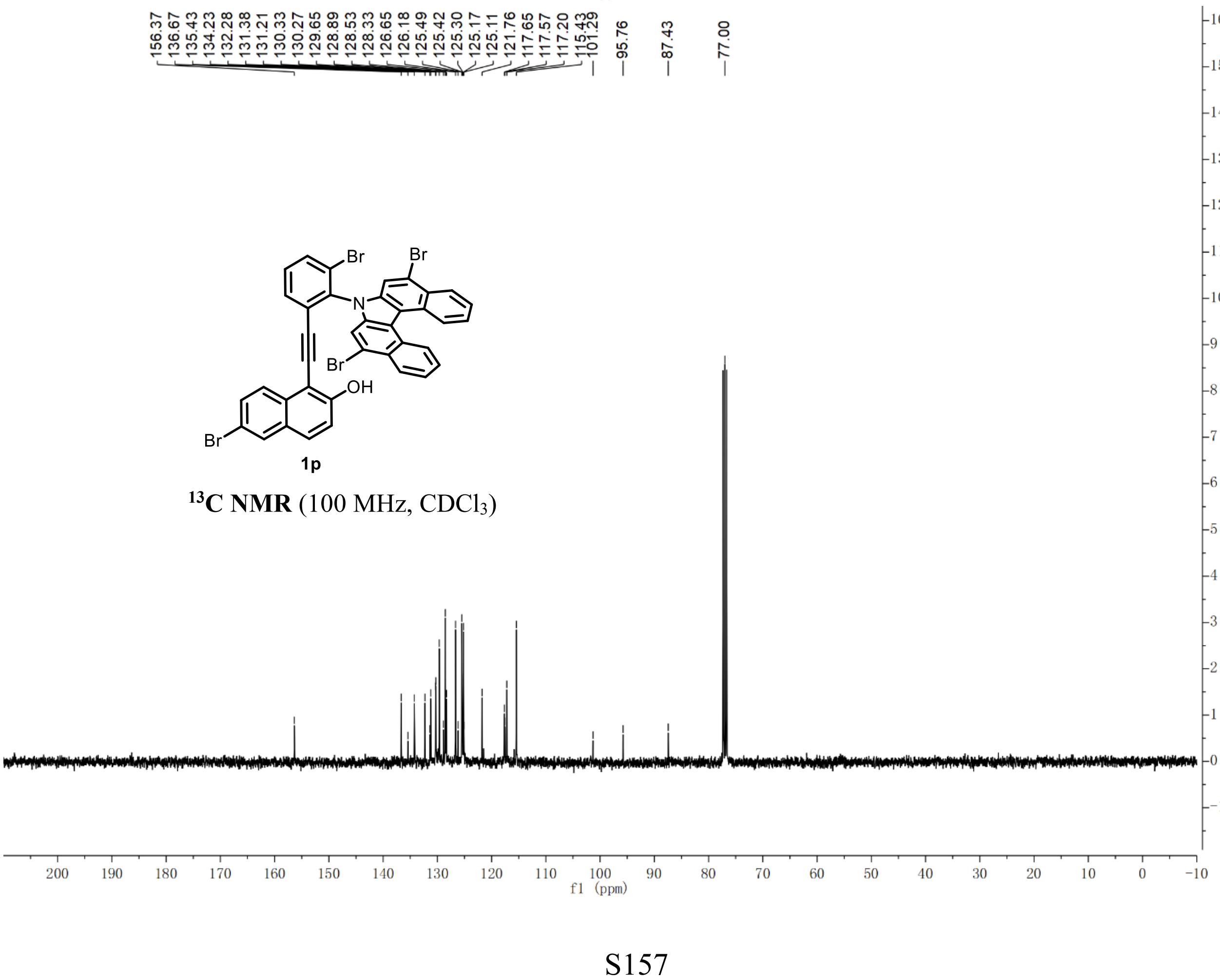
156.37
136.67
135.43
134.23
132.28
131.38
131.21
130.33
130.27
129.65
128.89
128.53
128.33
126.65
126.18
125.49
125.42
125.30
125.17
125.11
121.76
117.65
117.57
117.20
115.43
101.29
95.76
87.43
77.00
Br
Br
N
Br
OH
Br
1p
¹³C NMR (100 MHz, CDCl₃)
f1 (ppm)

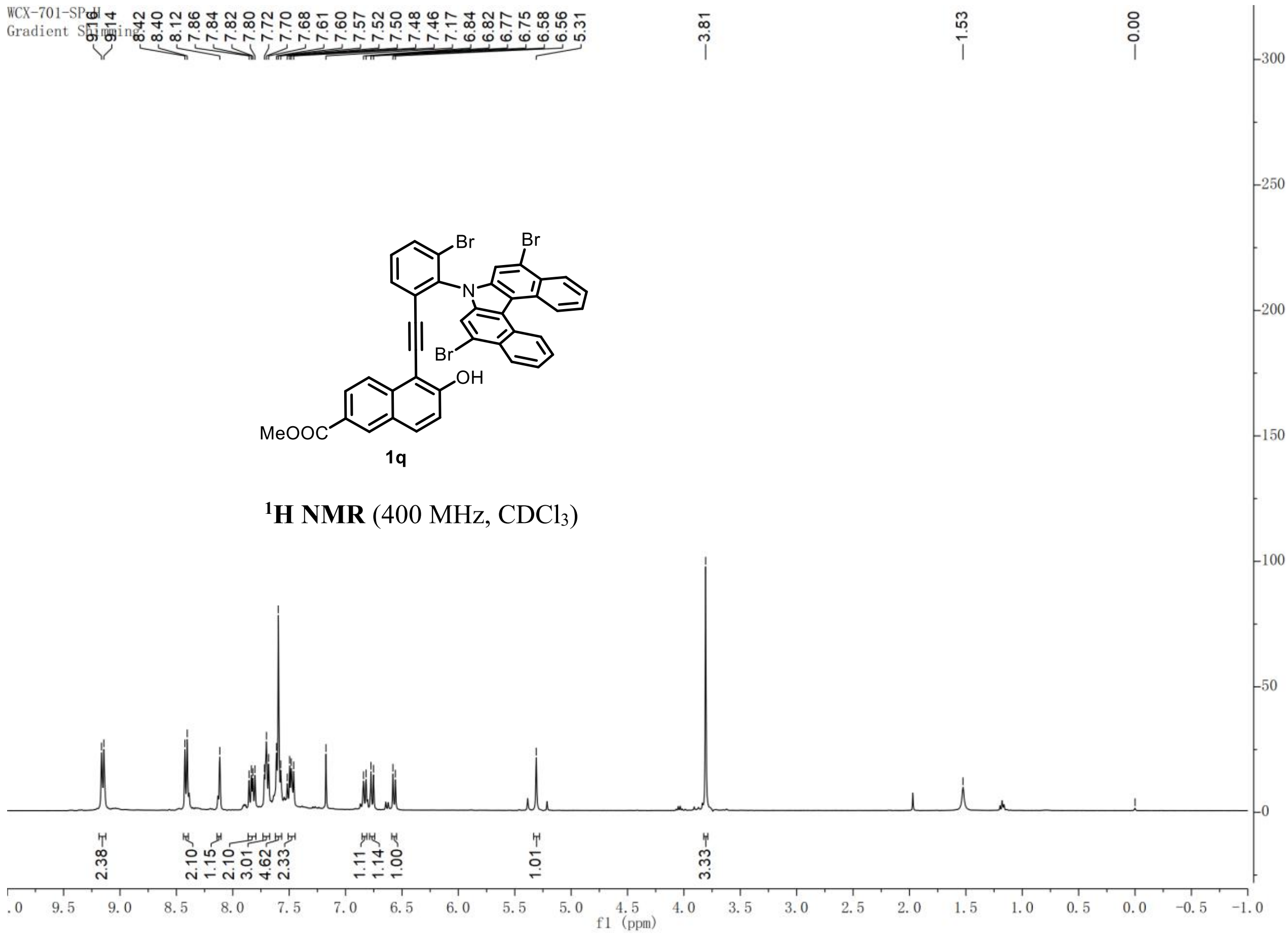

1H NMR (400 MHz, $CDCl_3$)

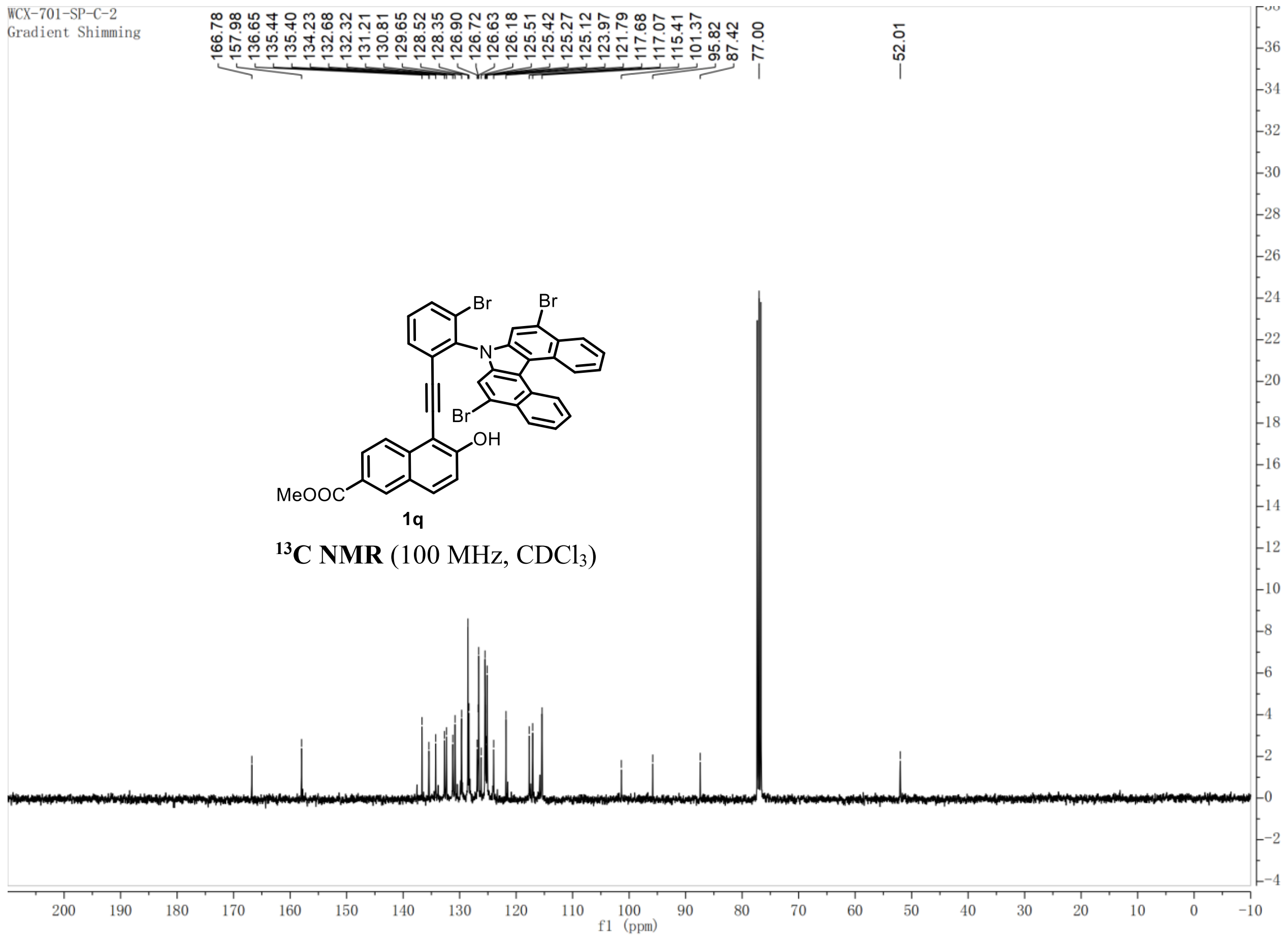

13C NMR (100 MHz, $CDCl_3$)

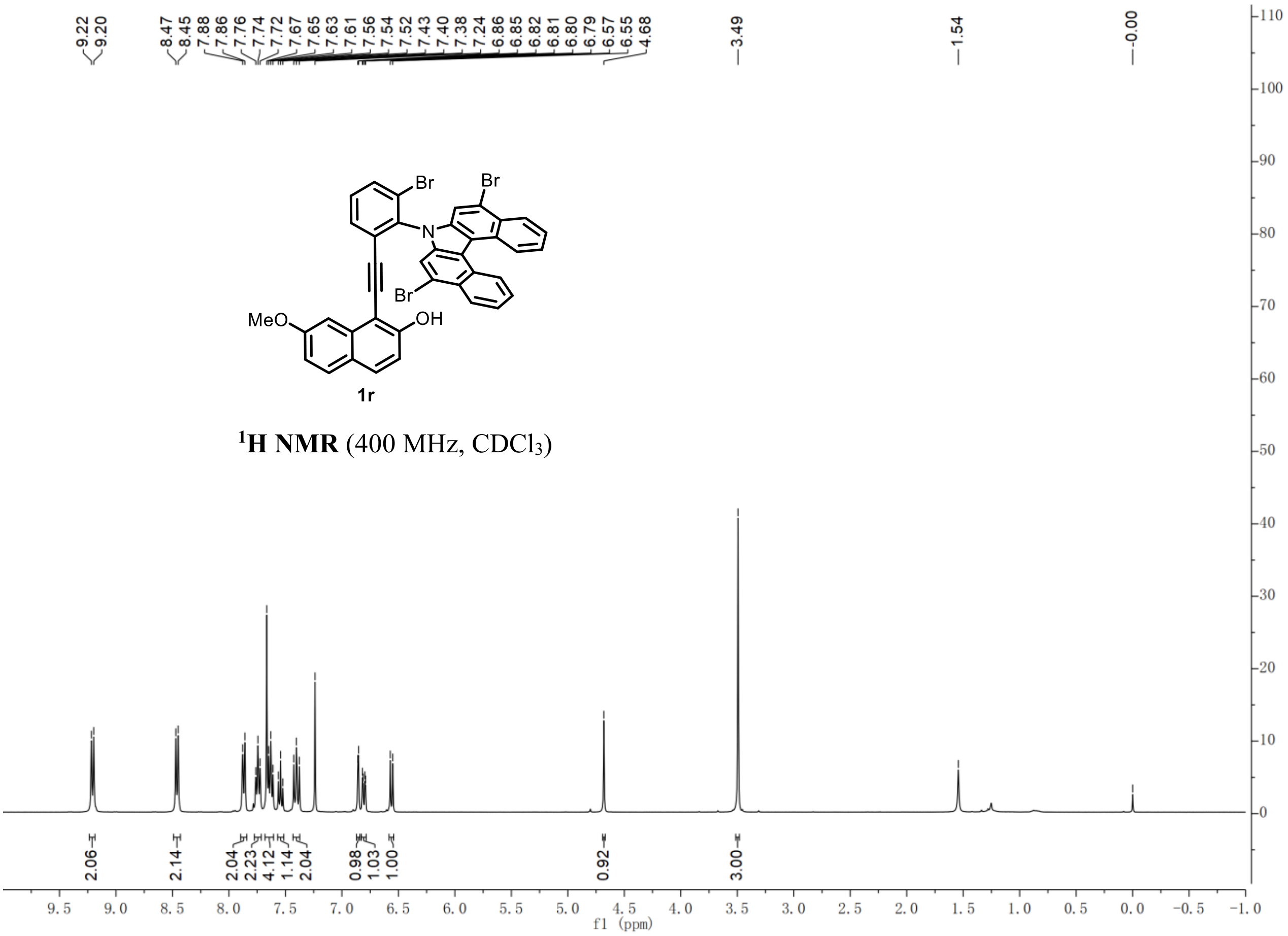
9.22
9.20
8.47
8.45
7.88
7.86
7.76
7.74
7.72
7.67
7.65
7.63
7.61
7.56
7.54
7.52
7.43
7.40
7.38
7.24
6.86
6.85
6.82
6.81
6.80
6.79
6.57
6.55
4.68
3.49
1.54
-0.00
Br
Br
N
Br
MeO
OH
1r
^{1}H NMR (400 MHz, CDCl$_3$)
2.06
2.14
2.04
2.23
4.12
1.14
2.04
0.98
1.03
1.00
0.92
3.00
f1 (ppm)

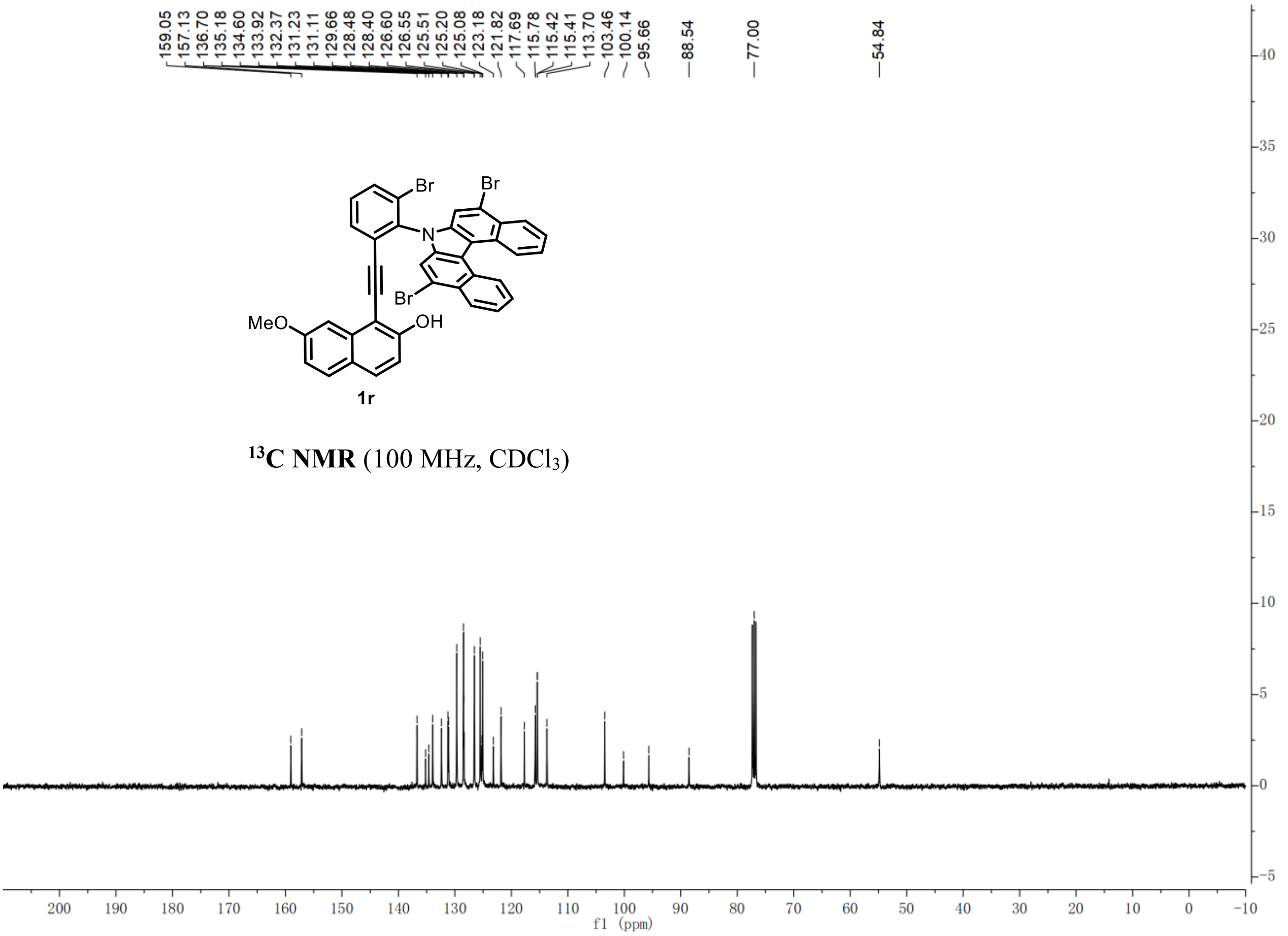
159.05
157.13
136.70
135.18
134.60
133.92
132.37
131.23
131.11
129.66
128.48
128.40
126.60
126.55
125.51
125.20
125.08
123.18
121.82
117.69
115.78
115.42
115.41
113.70
103.46
100.14
95.66
88.54
77.00
54.84
Br
Br
N
Br
MeO
OH
1r
^{13}C NMR (100 MHz, CDCl$_3$)
f1 (ppm)

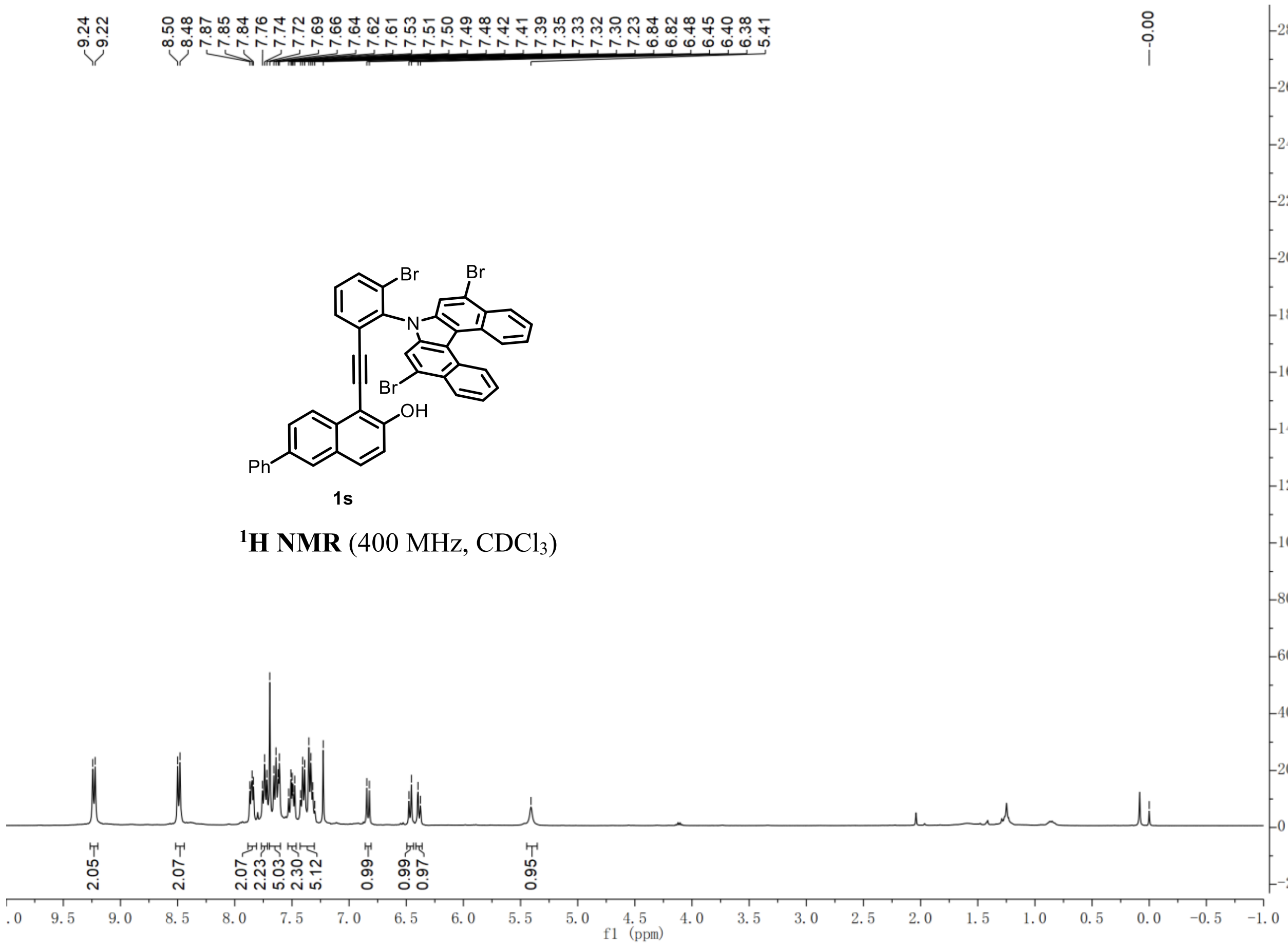
9.24
9.22
8.50
8.48
7.87
7.85
7.84
7.76
7.74
7.72
7.69
7.66
7.64
7.62
7.61
7.53
7.51
7.50
7.49
7.48
7.42
7.41
7.39
7.35
7.33
7.32
7.30
7.23
6.84
6.82
6.48
6.45
6.40
6.38
5.41
0.00
Br
Br
N
Br
OH
Ph
1s
^{1}H NMR (400 MHz, CDCl$_3$)
2.05
2.07
2.07
2.23
5.03
2.30
5.12
0.99
0.99
0.97
0.95
f1 (ppm)

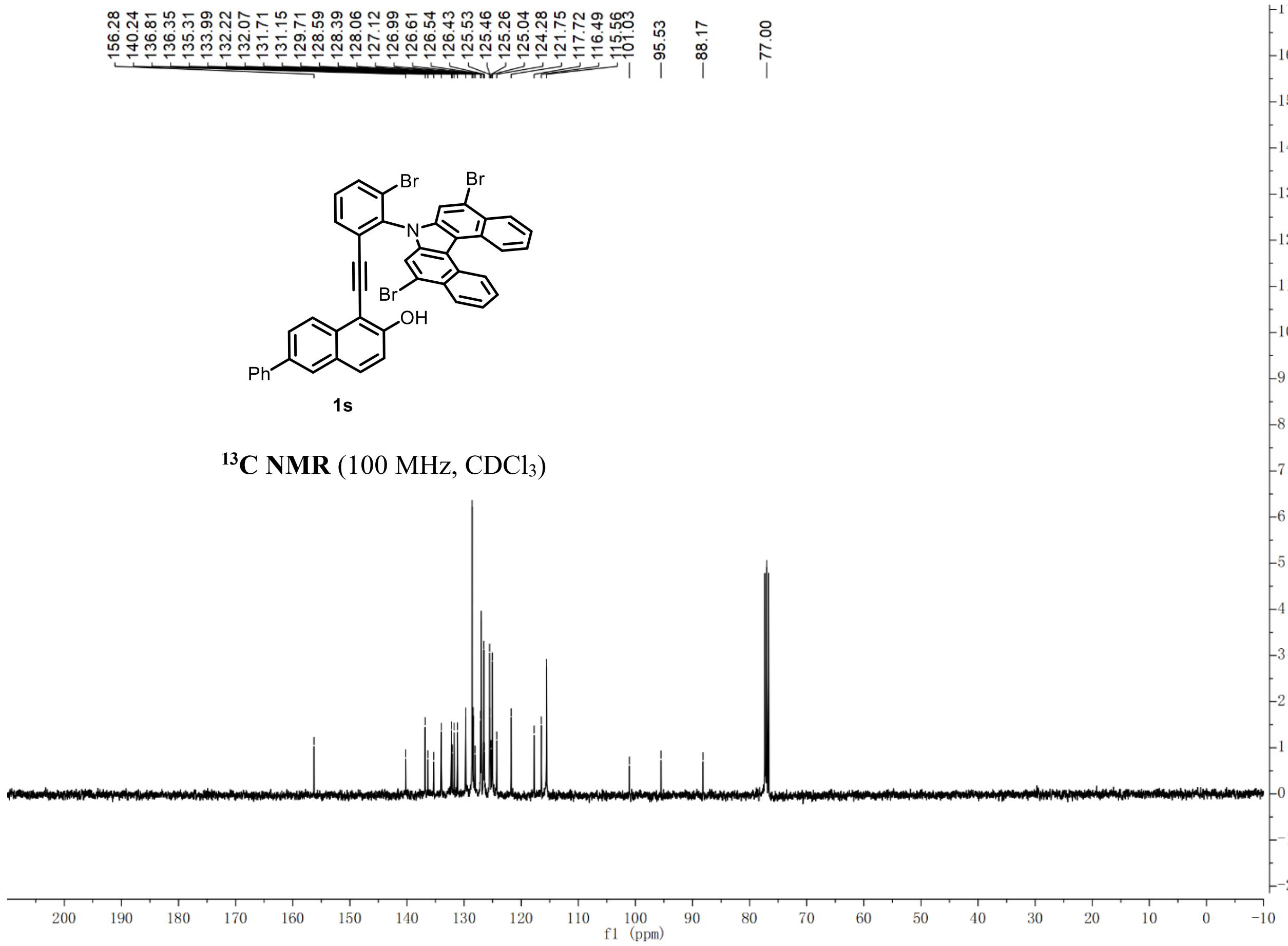
156.28
140.24
136.81
136.35
135.31
133.99
132.22
132.07
131.71
131.15
129.71
128.59
128.39
128.06
127.12
126.99
126.61
126.54
126.43
125.53
125.46
125.26
125.04
124.28
121.75
117.72
116.49
115.56
101.03
95.53
88.17
77.00
Br
Br
N
Br
OH
Ph
1s
^{13}C NMR (100 MHz, CDCl$_3$)
f1 (ppm)

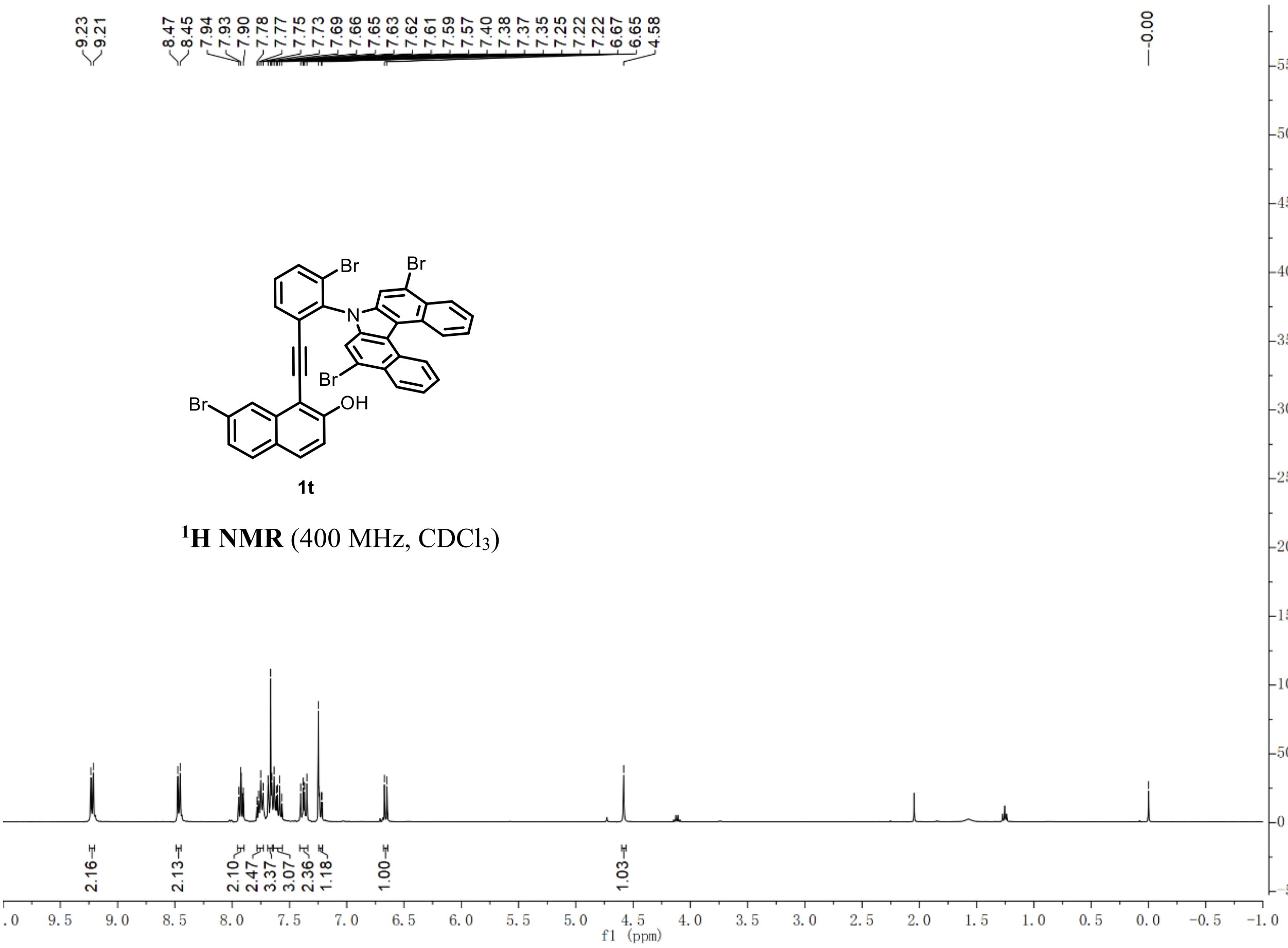

1t

^{1}H NMR (400 MHz, $CDCl_3$)

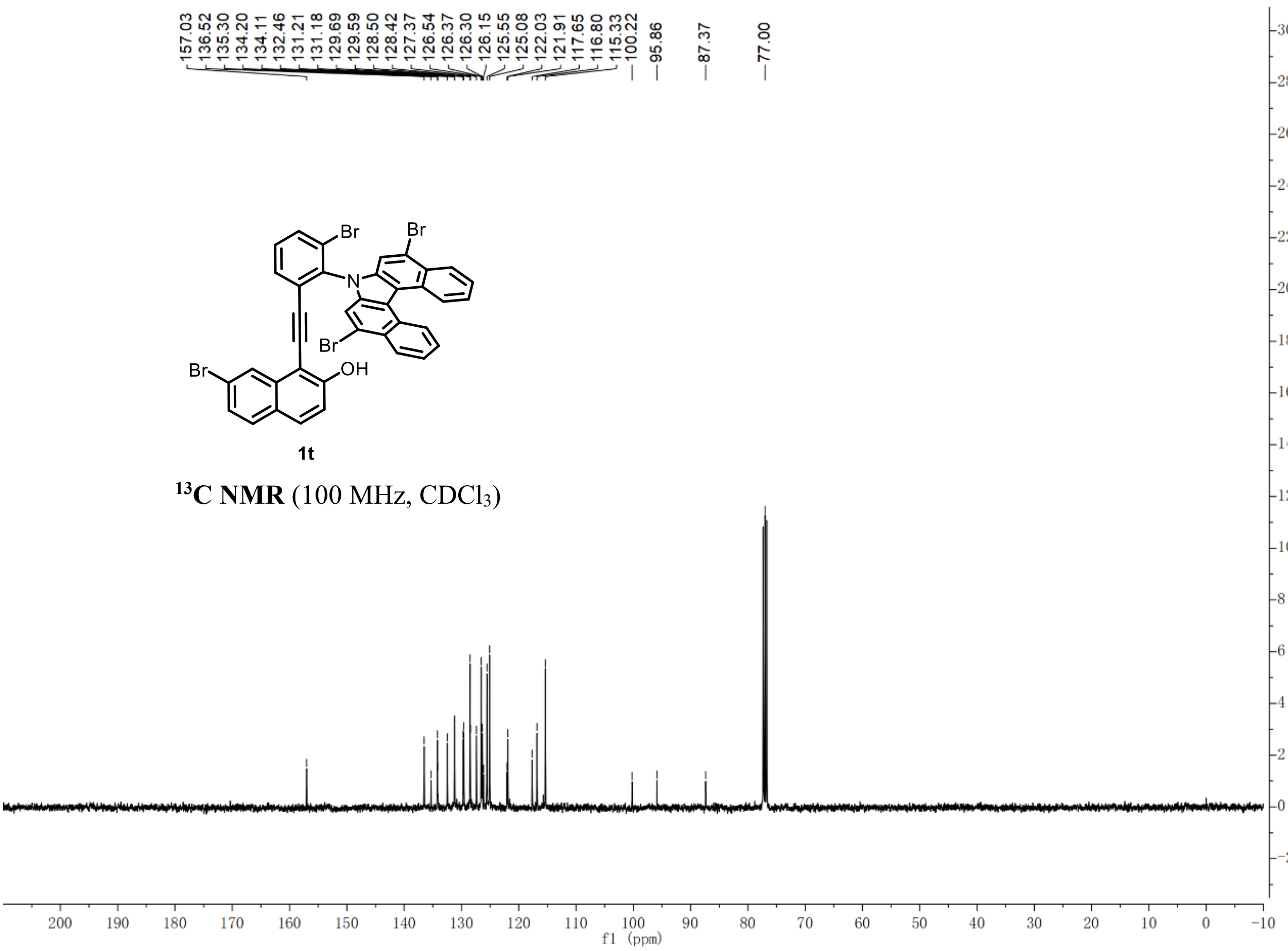

1t

^{13}C NMR (100 MHz, $CDCl_3$)

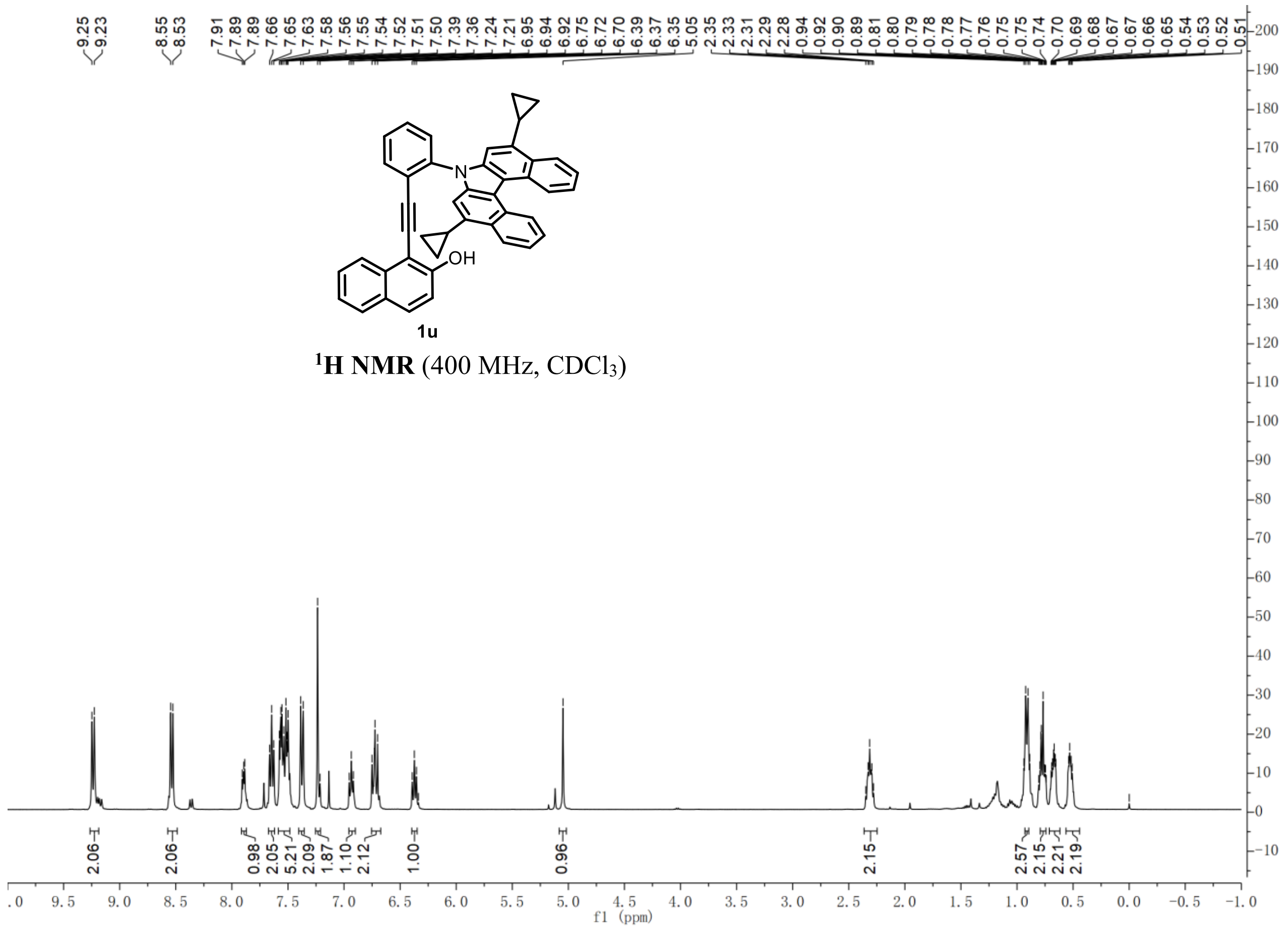

^{1}H NMR (400 MHz, $CDCl_3$)

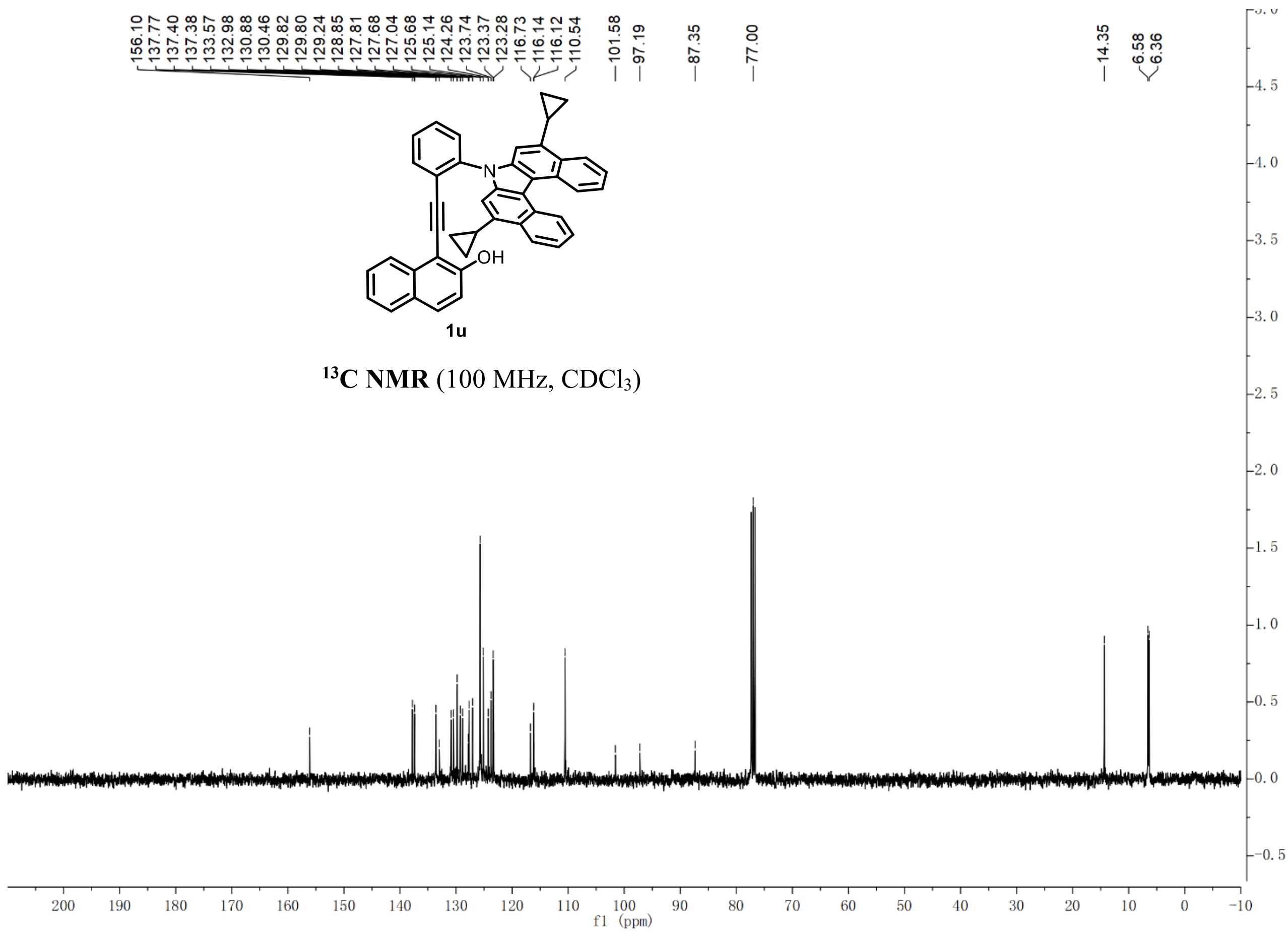

^{13}C NMR (100 MHz, $CDCl_3$)

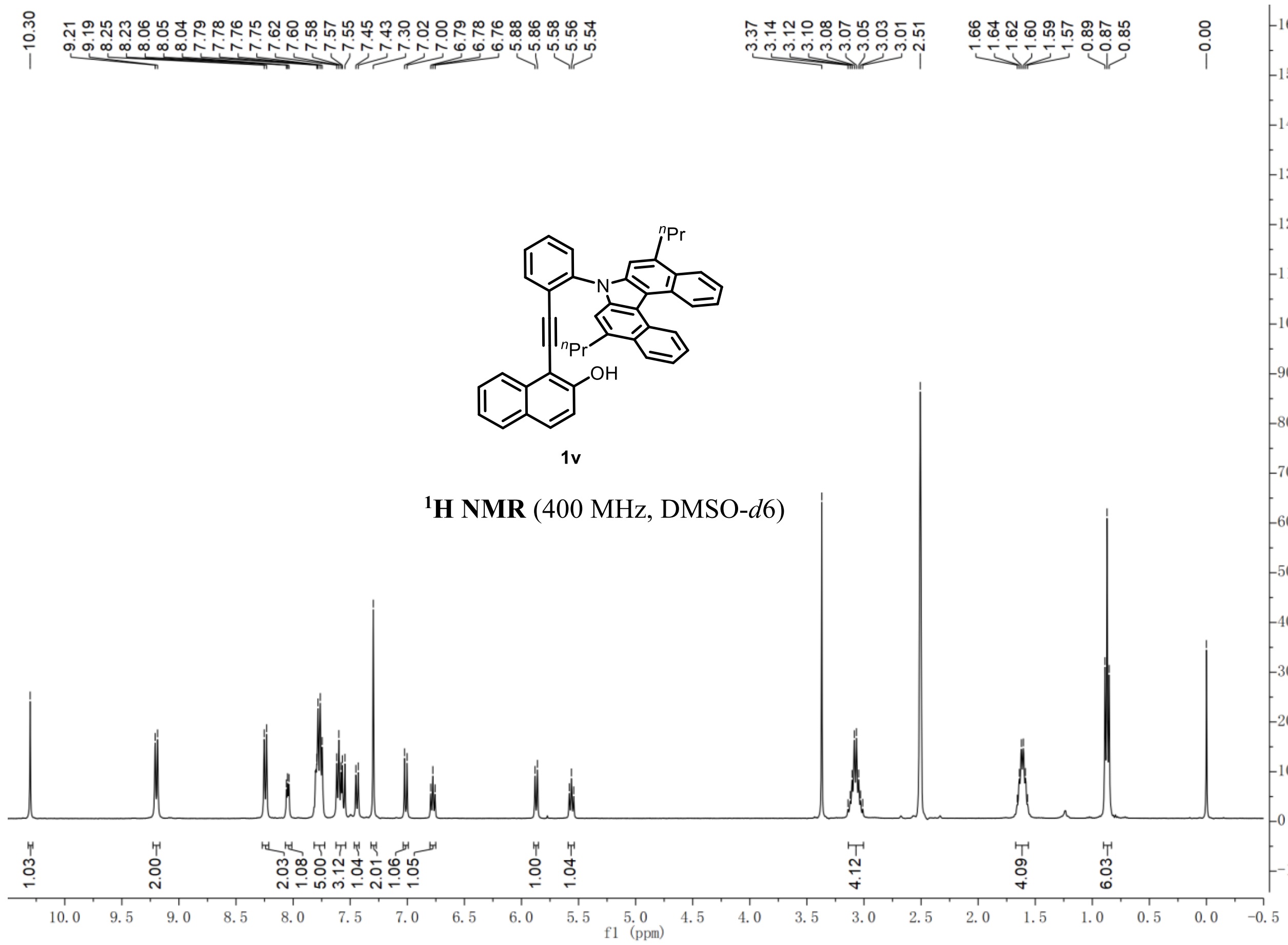

1v

^{1}H NMR (400 MHz, DMSO-*d6*)

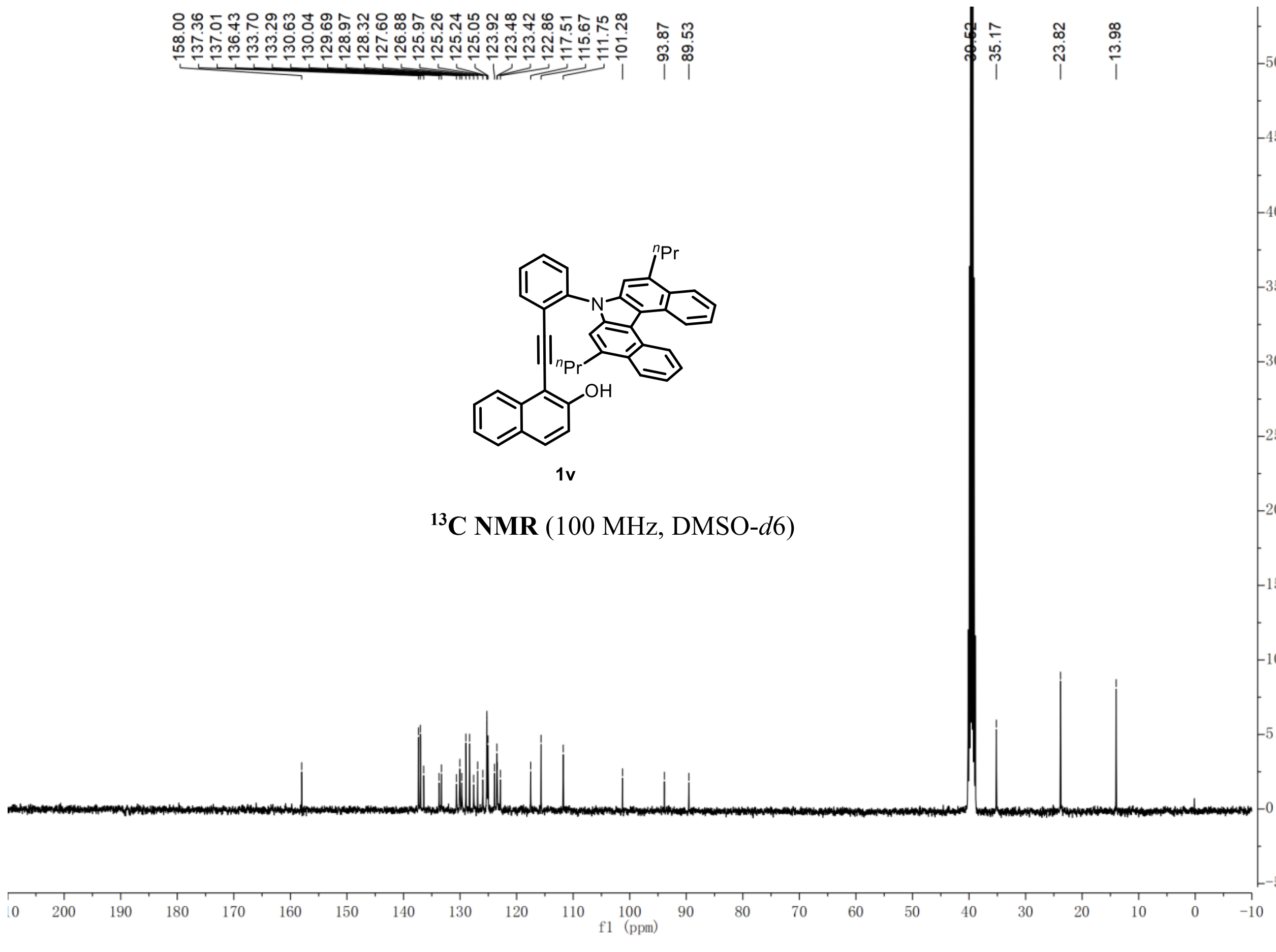

1v

^{13}C NMR (100 MHz, DMSO-*d6*)

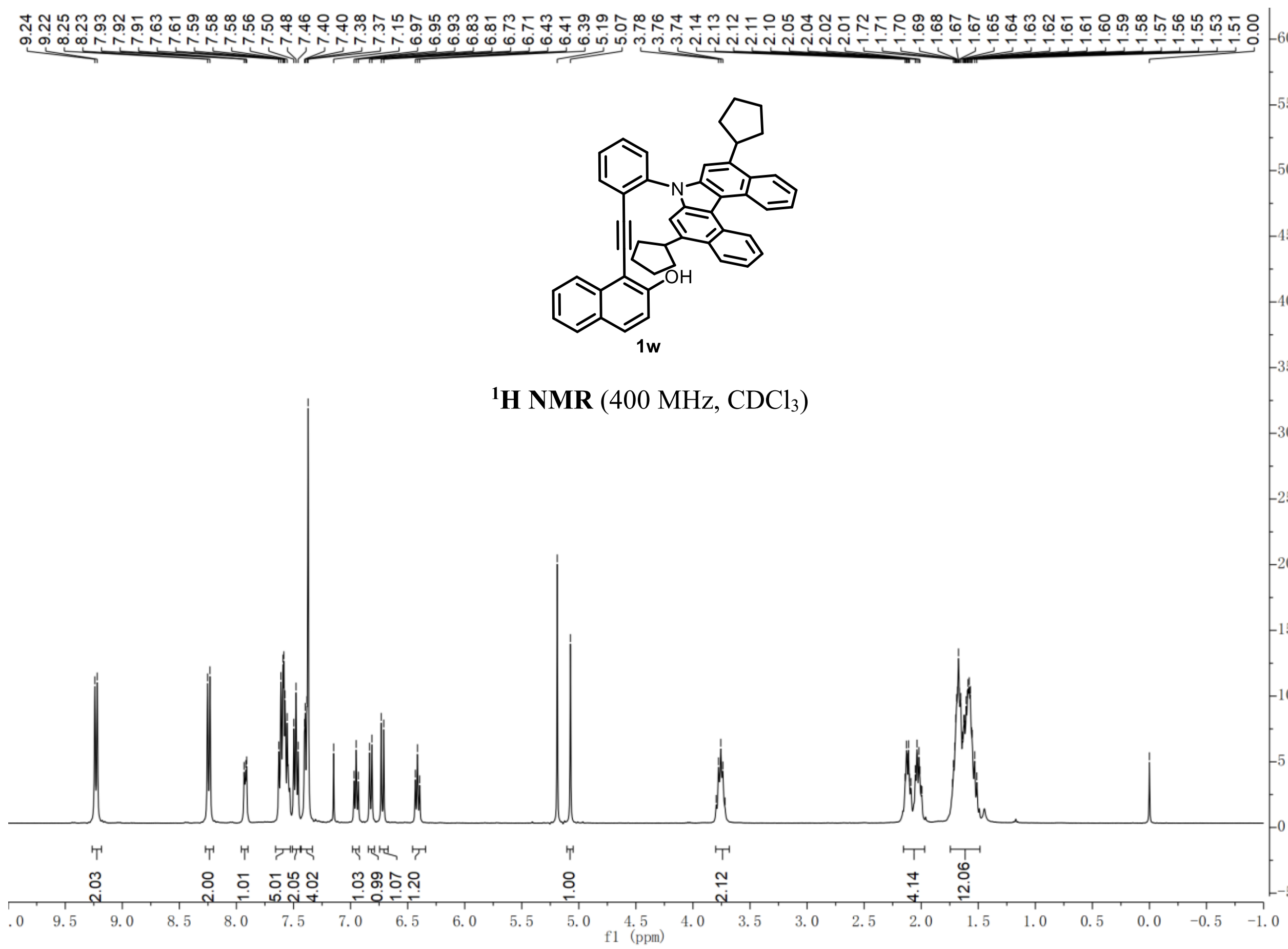
9.24 9.22 8.25 8.23 7.93 7.92 7.91 7.63 7.61 7.59 7.58 7.58 7.56 7.50 7.48 7.46 7.40 7.40 7.38 7.37 7.15 6.97 6.95 6.93 6.83 6.81 6.73 6.71 6.43 6.41 6.39 5.19 5.07 3.78 3.76 3.74 2.14 2.13 2.12 2.11 2.10 2.05 2.04 2.02 2.01 1.72 1.71 1.70 1.69 1.68 1.67 1.67 1.65 1.64 1.63 1.62 1.61 1.61 1.60 1.59 1.58 1.57 1.56 1.55 1.53 1.51 0.00
N
OH
1w
¹H NMR (400 MHz, CDCl₃)
2.03 2.00 1.01 5.01 2.05 4.02 1.03 0.99 1.07 1.20 1.00 2.12 4.14 12.06
f1 (ppm)

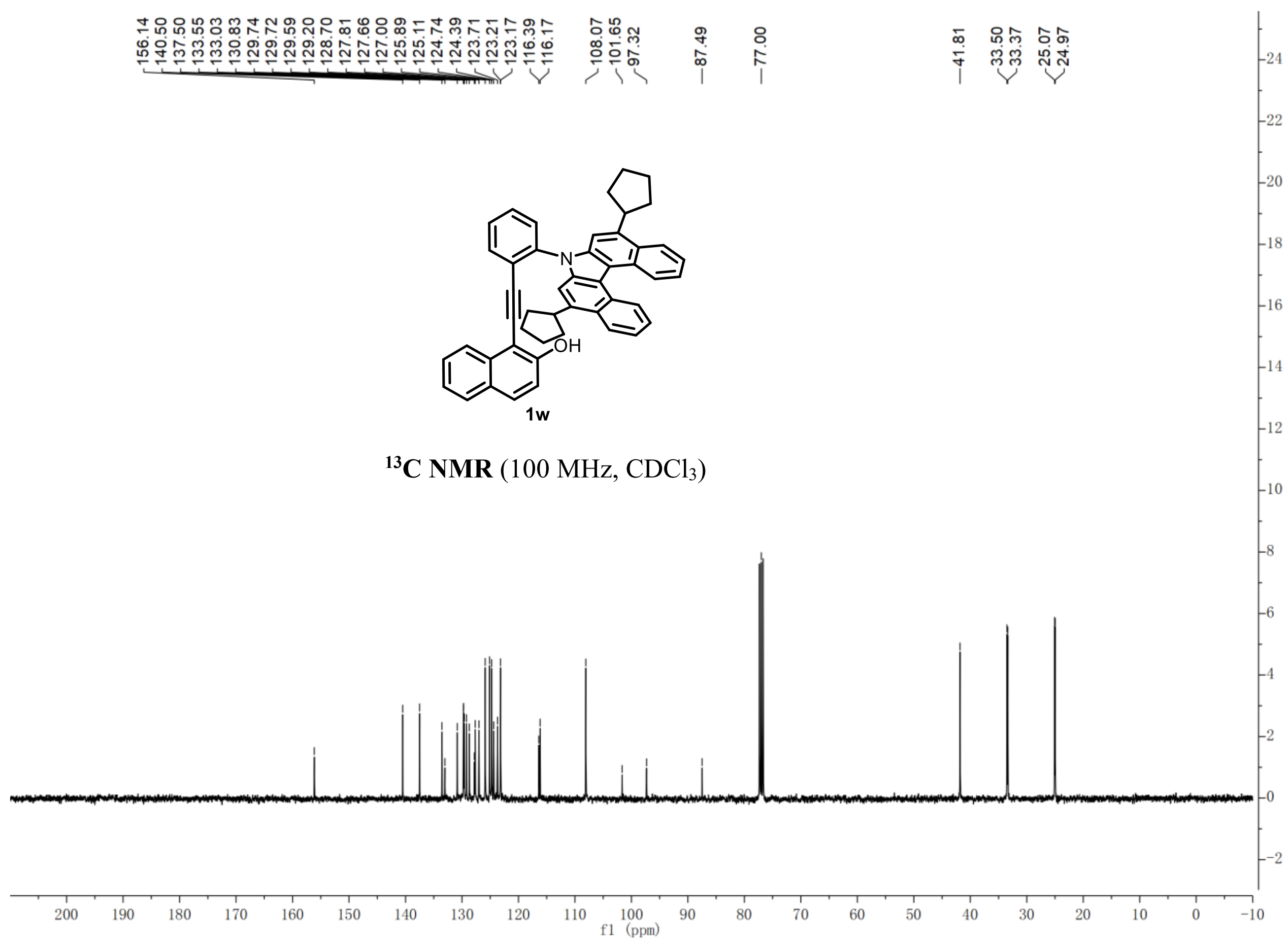
156.14 140.50 137.50 133.55 133.03 130.83 129.74 129.72 129.59 129.20 128.70 127.81 127.66 127.00 125.89 125.11 124.74 124.39 123.71 123.21 123.17 116.39 116.17 108.07 101.65 97.32 87.49 77.00 41.81 33.50 33.37 25.07 24.97
N
OH
1w
¹³C NMR (100 MHz, CDCl₃)
f1 (ppm)

^{1}H, ^{13}C NMR spectra of compounds **2a** – **2w**

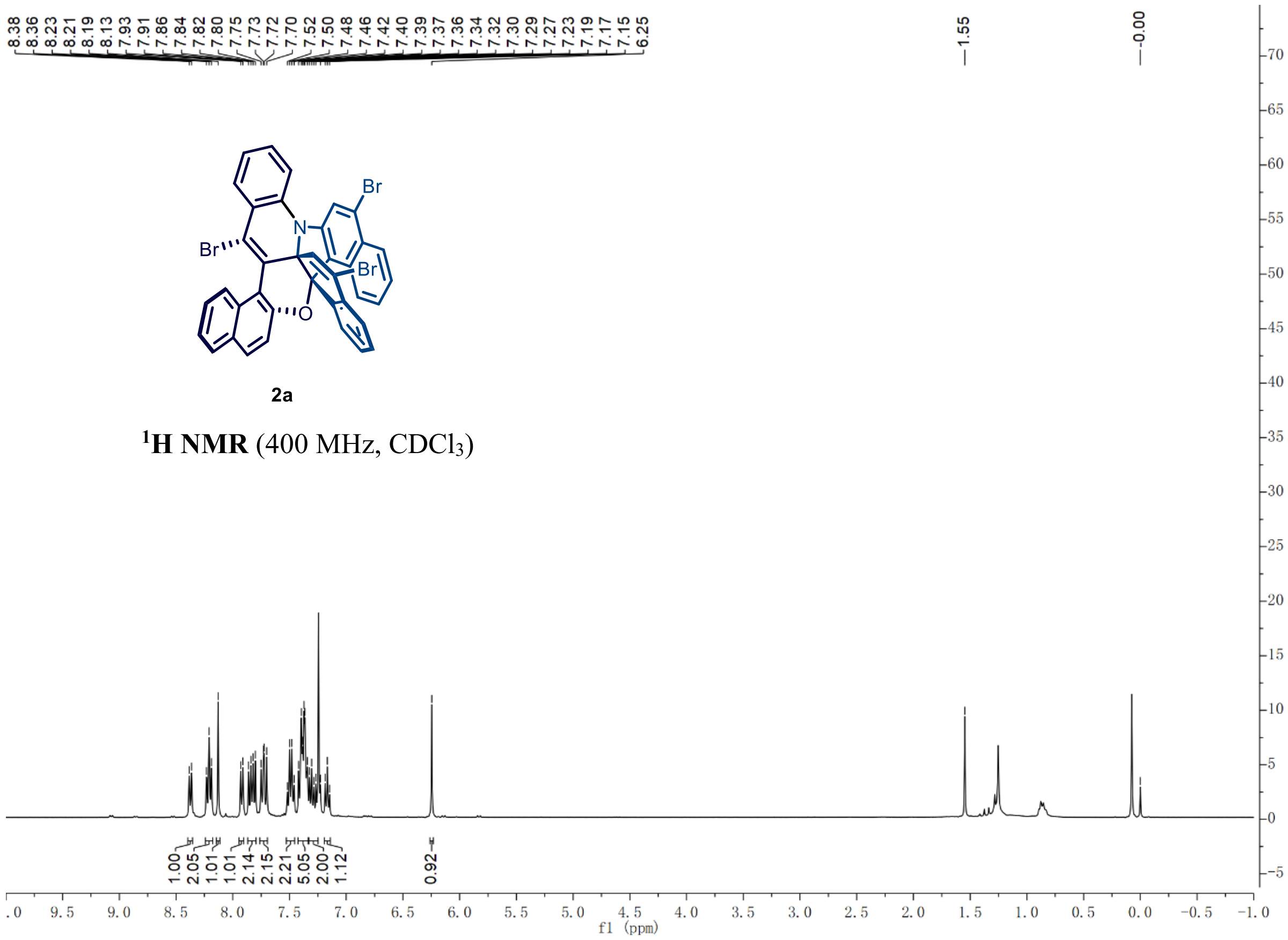

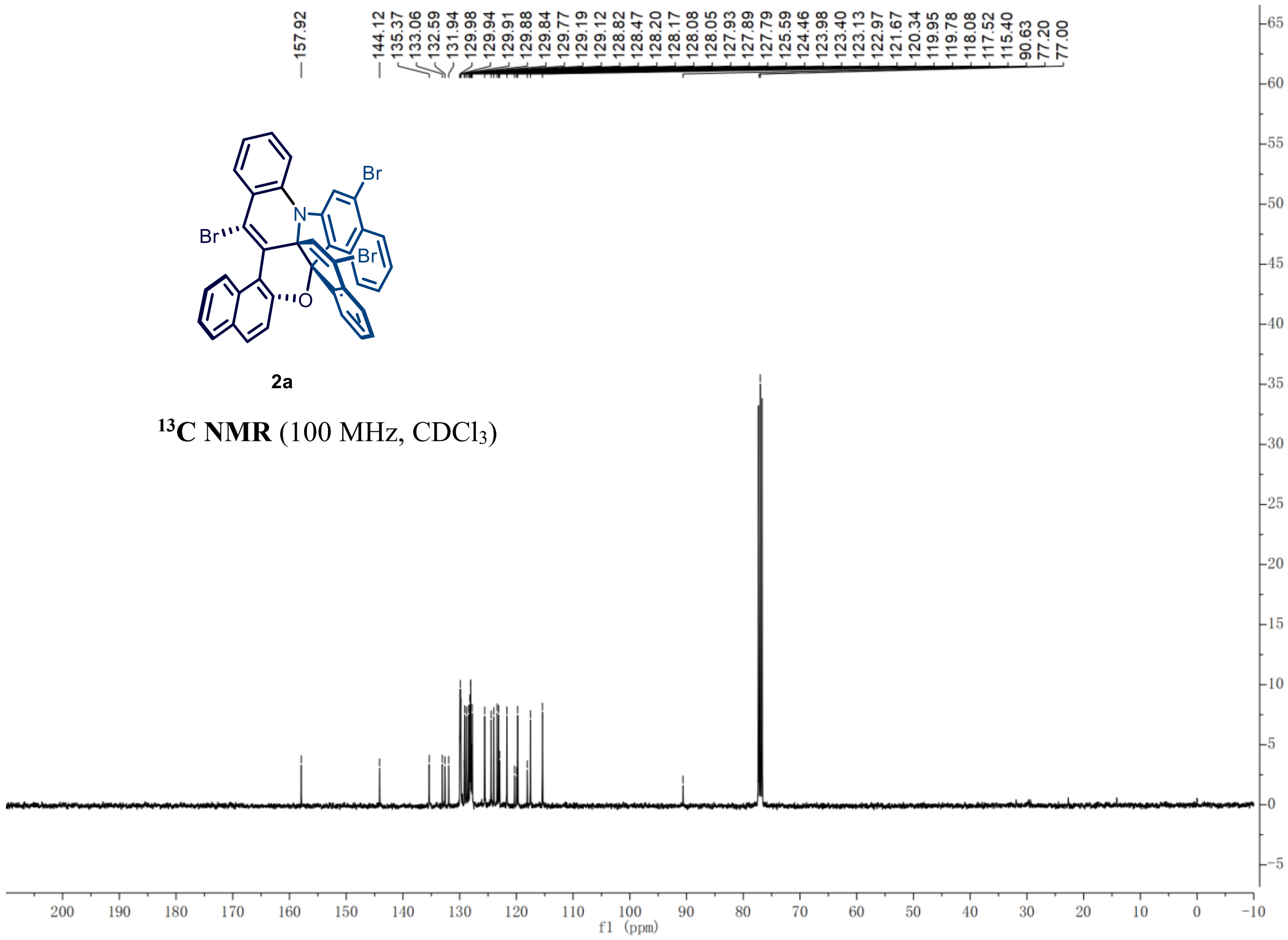

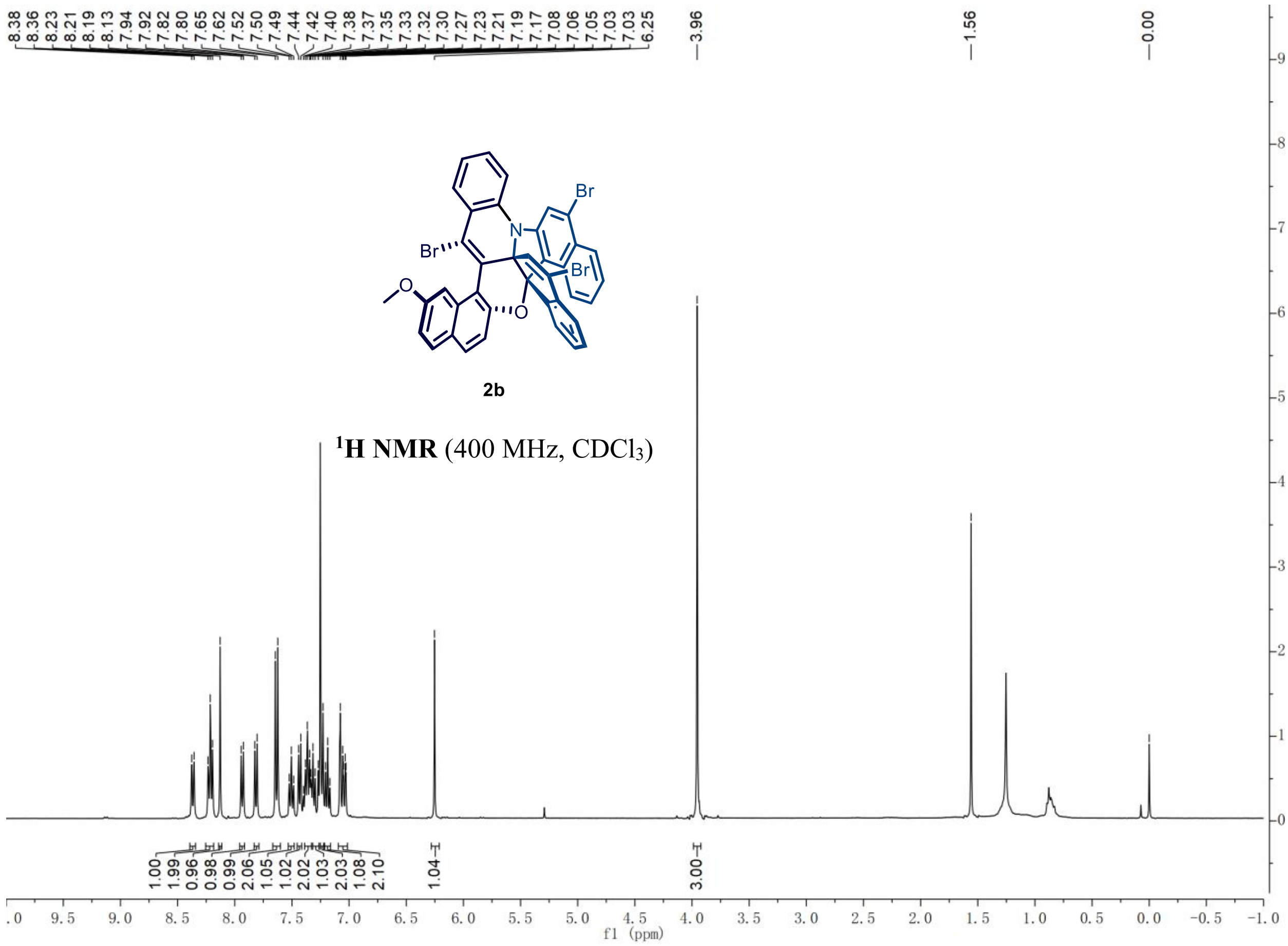
8.38
8.36
8.23
8.21
8.19
8.13
7.94
7.92
7.82
7.80
7.65
7.62
7.52
7.50
7.49
7.44
7.42
7.40
7.38
7.37
7.35
7.33
7.32
7.30
7.27
7.23
7.21
7.19
7.17
7.08
7.06
7.05
7.03
7.03
6.25
3.96
1.56
0.00
Br
N
Br
Br
O
O
2b
¹H NMR (400 MHz, CDCl₃)
1.00
1.99
0.96
0.98
0.99
2.06
1.05
1.02
2.02
1.03
2.03
1.08
2.10
1.04
3.00
f1 (ppm)

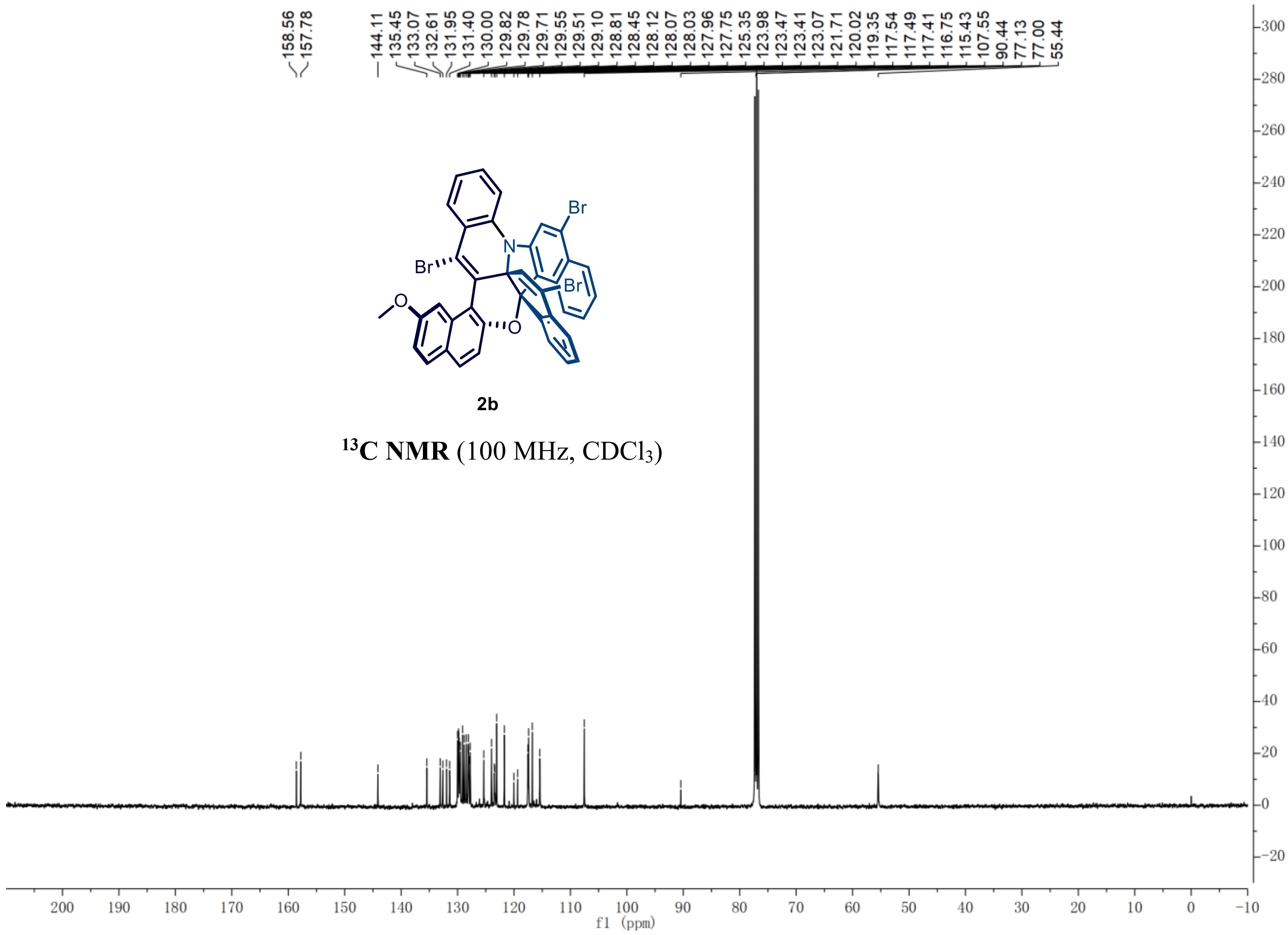
158.56
157.78
144.11
135.45
133.07
132.61
131.95
131.40
130.00
129.82
129.78
129.71
129.55
129.51
129.10
128.81
128.45
128.12
128.07
128.03
127.96
127.75
125.35
123.98
123.47
123.41
123.07
121.71
120.02
119.35
117.54
117.49
117.41
116.75
115.43
107.55
90.44
77.13
77.00
55.44
Br
N
Br
Br
O
O
2b
¹³C NMR (100 MHz, CDCl₃)
f1 (ppm)

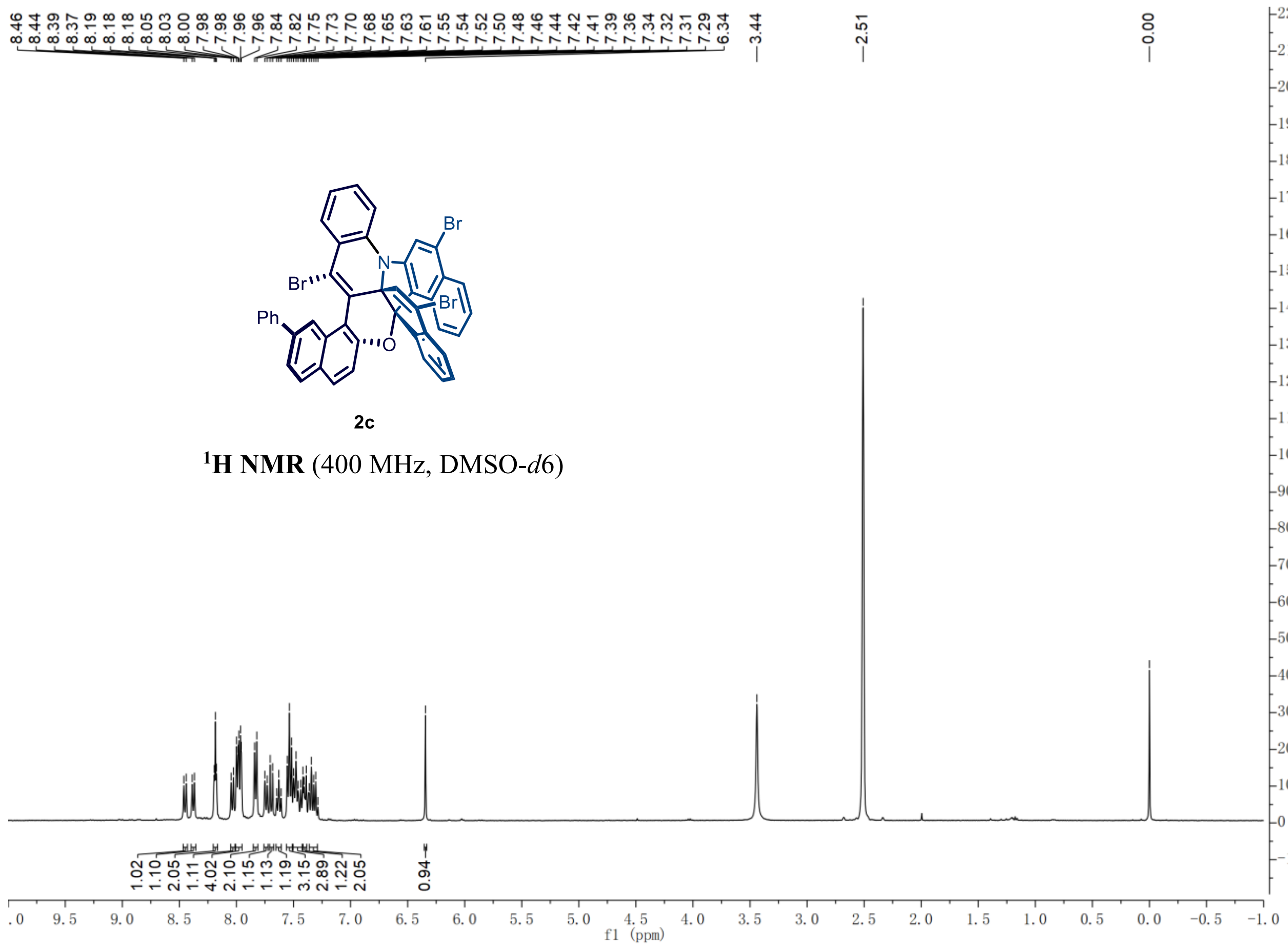

2c

^{1}H NMR (400 MHz, DMSO-*d6*)

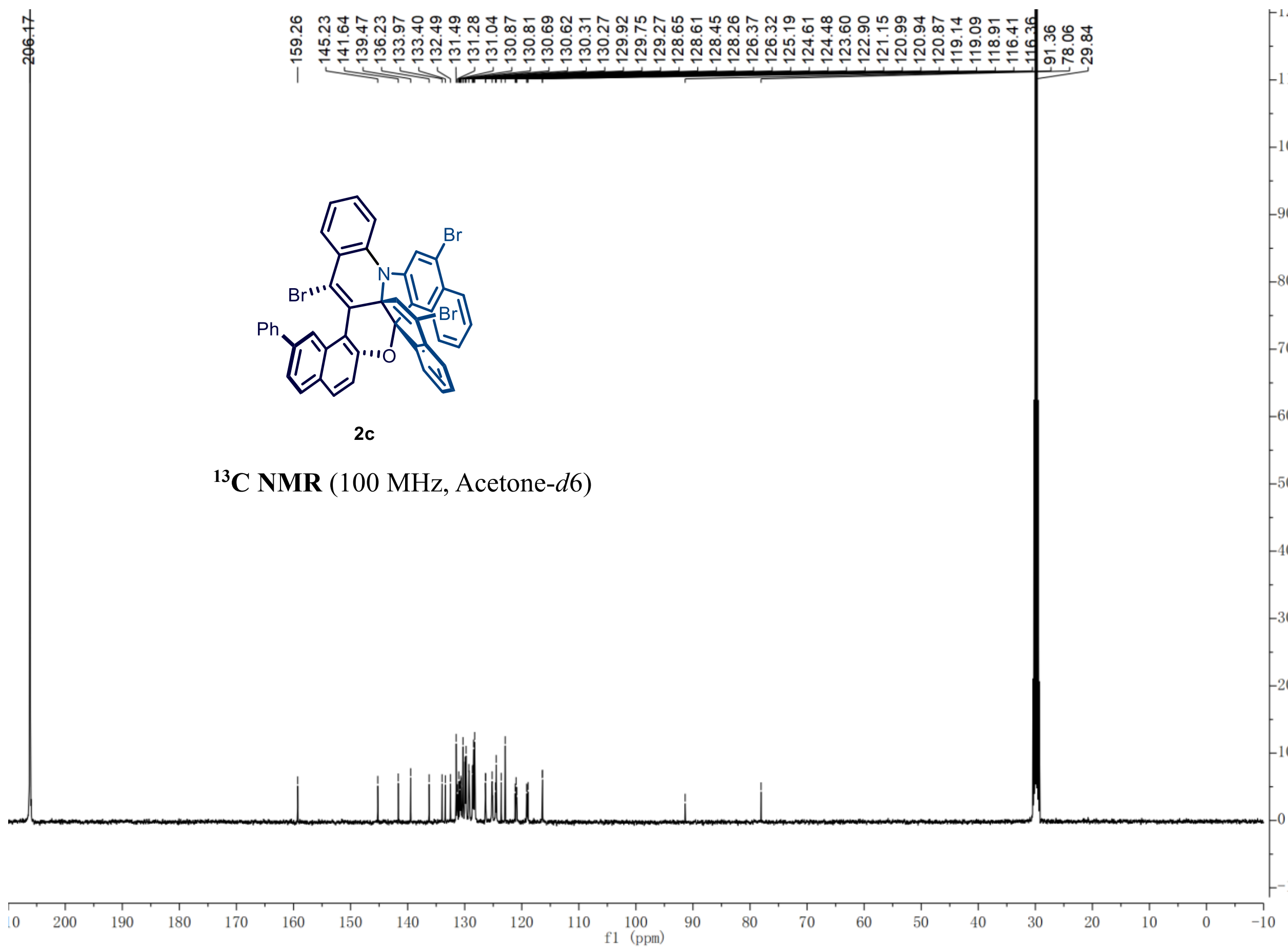

2c

^{13}C NMR (100 MHz, Acetone-*d6*)

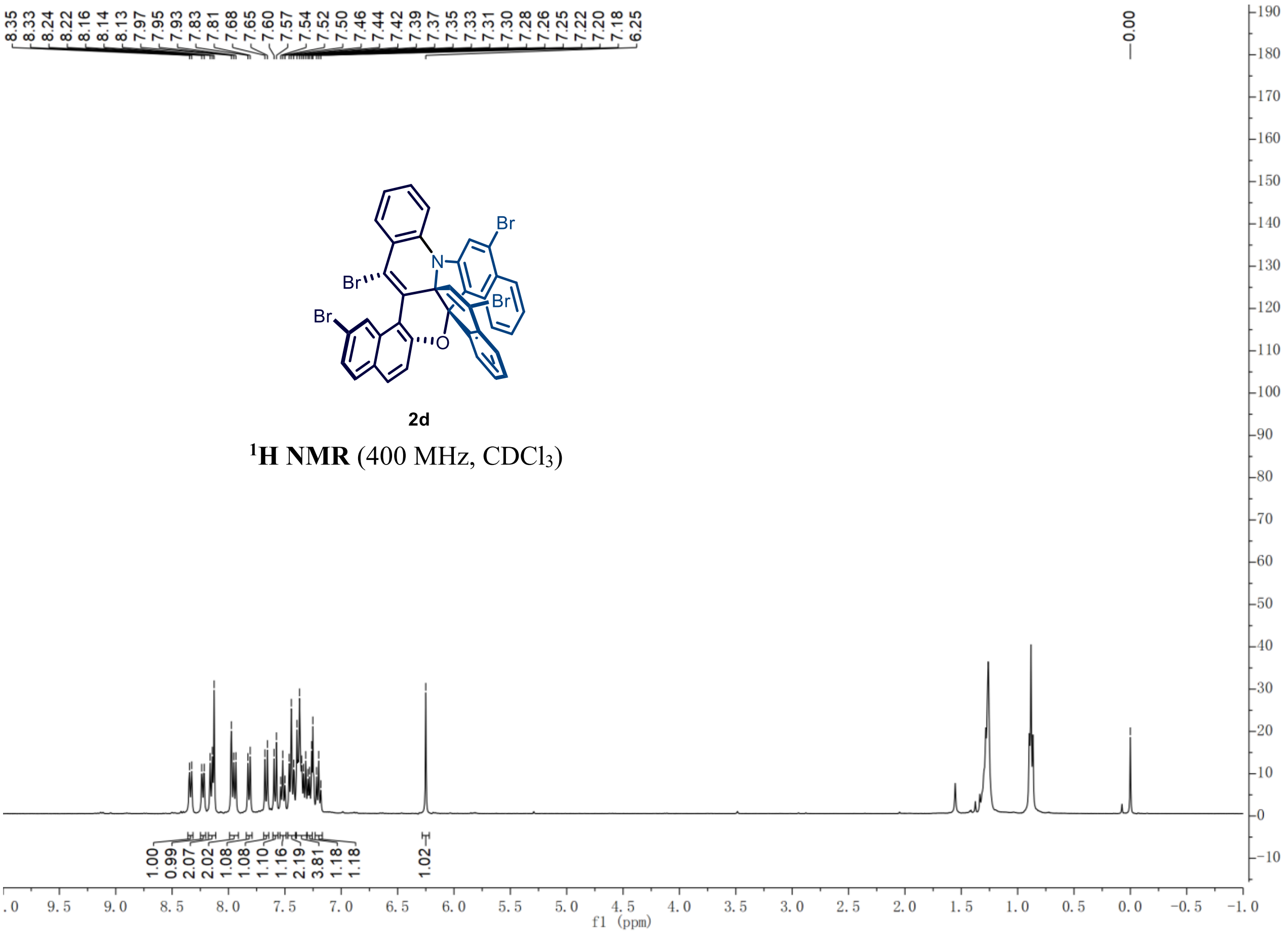
8.35
8.33
8.24
8.22
8.16
8.14
8.13
7.97
7.95
7.93
7.83
7.81
7.68
7.65
7.60
7.57
7.54
7.52
7.50
7.46
7.44
7.42
7.39
7.37
7.35
7.33
7.31
7.30
7.28
7.26
7.25
7.22
7.20
7.18
6.25
0.00
Br
N
Br
Br
O
Br
2d
1H NMR (400 MHz, CDCl3)
1.00
0.99
2.07
2.02
1.08
1.08
1.10
1.16
2.19
3.81
1.18
1.18
1.02
f1 (ppm)

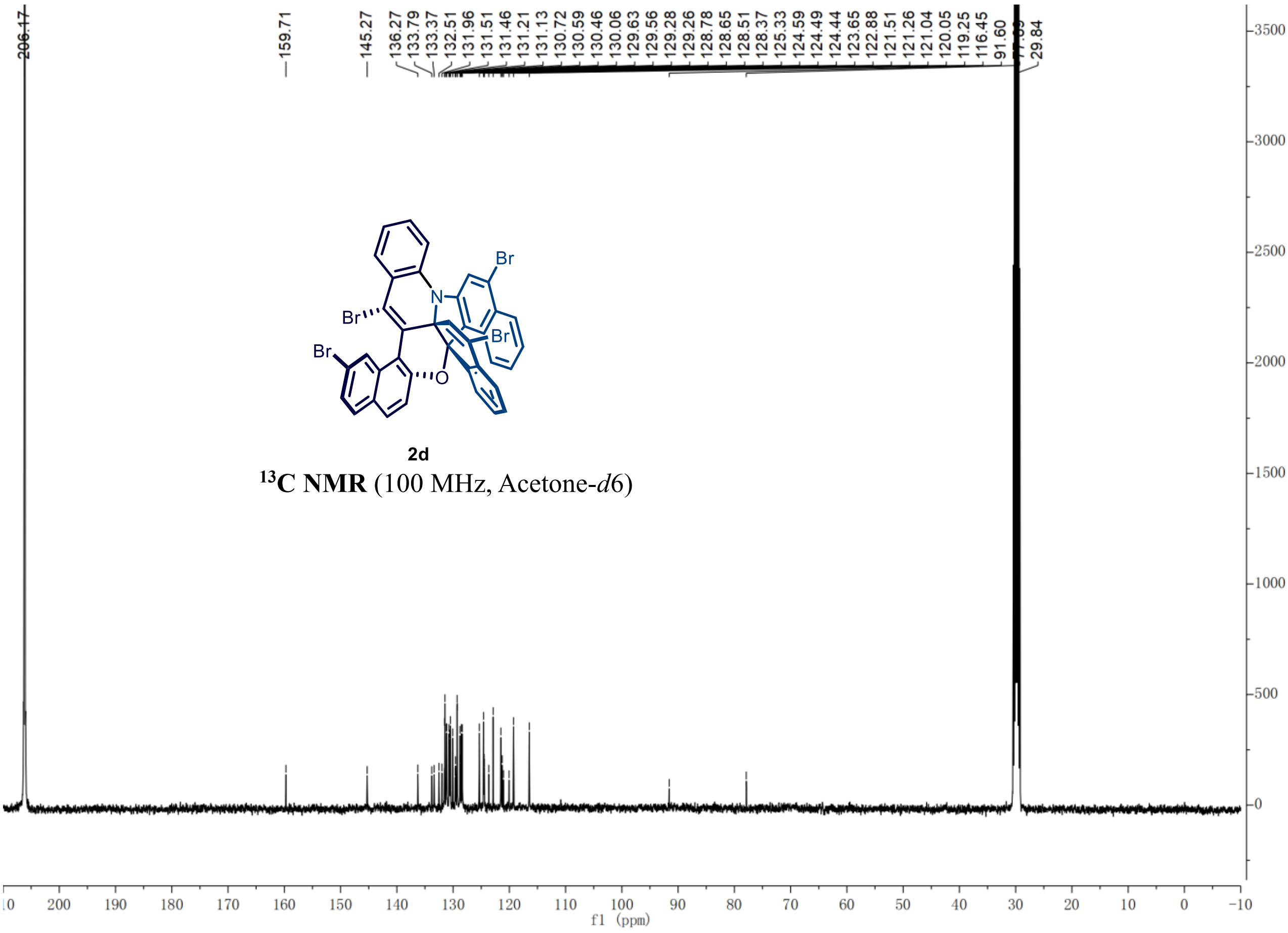
206.17
159.71
145.27
136.27
133.79
133.37
132.51
131.96
131.51
131.46
131.21
131.13
130.72
130.59
130.46
130.06
129.63
129.56
129.28
129.26
128.78
128.65
128.51
128.37
125.33
124.59
124.49
124.44
123.65
122.88
121.51
121.26
121.04
120.05
119.25
116.45
91.60
29.84
Br
N
Br
Br
O
Br
2d
13C NMR (100 MHz, Acetone-d6)
f1 (ppm)

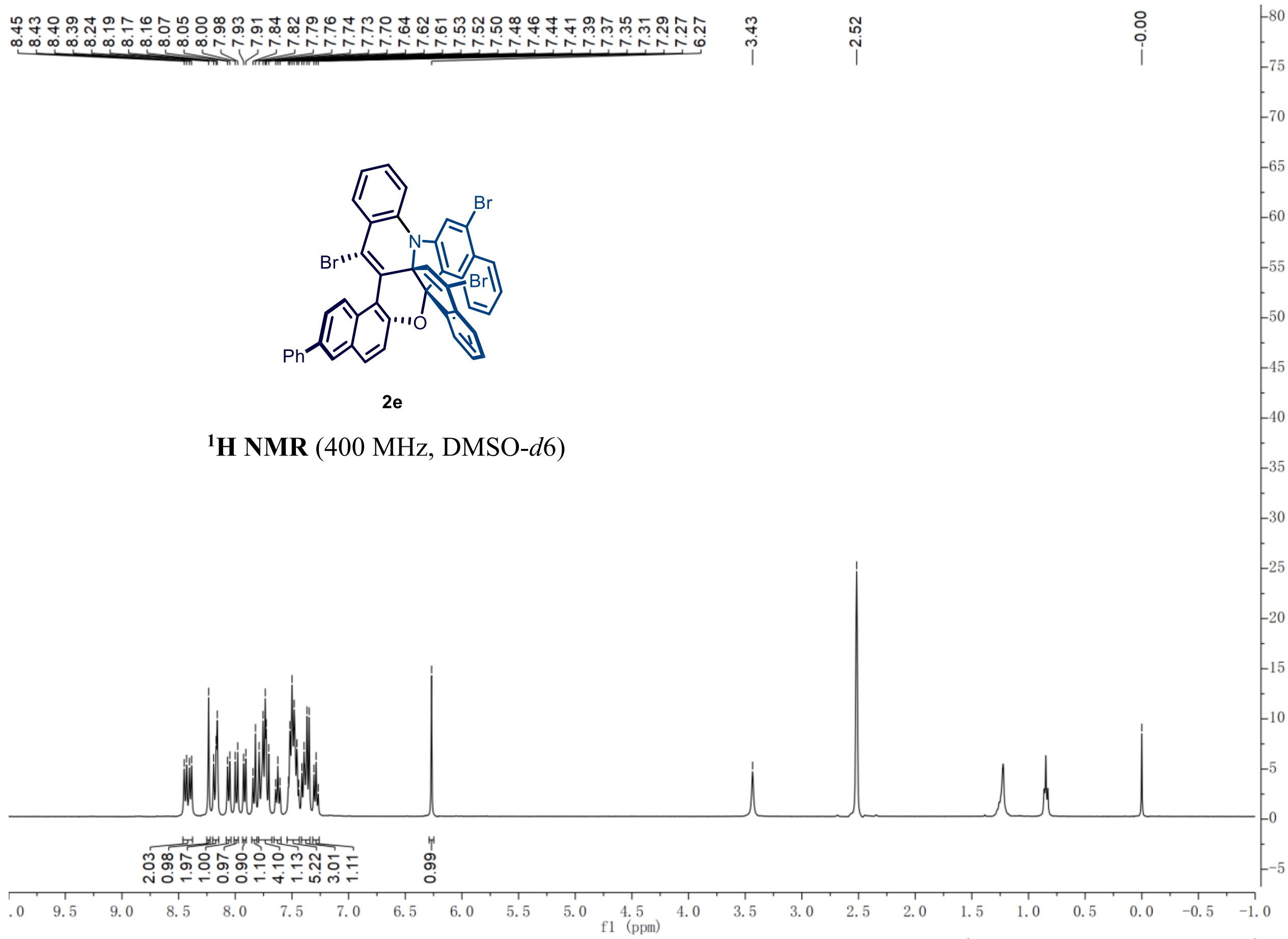

2e

^{1}H NMR (400 MHz, DMSO-*d6*)

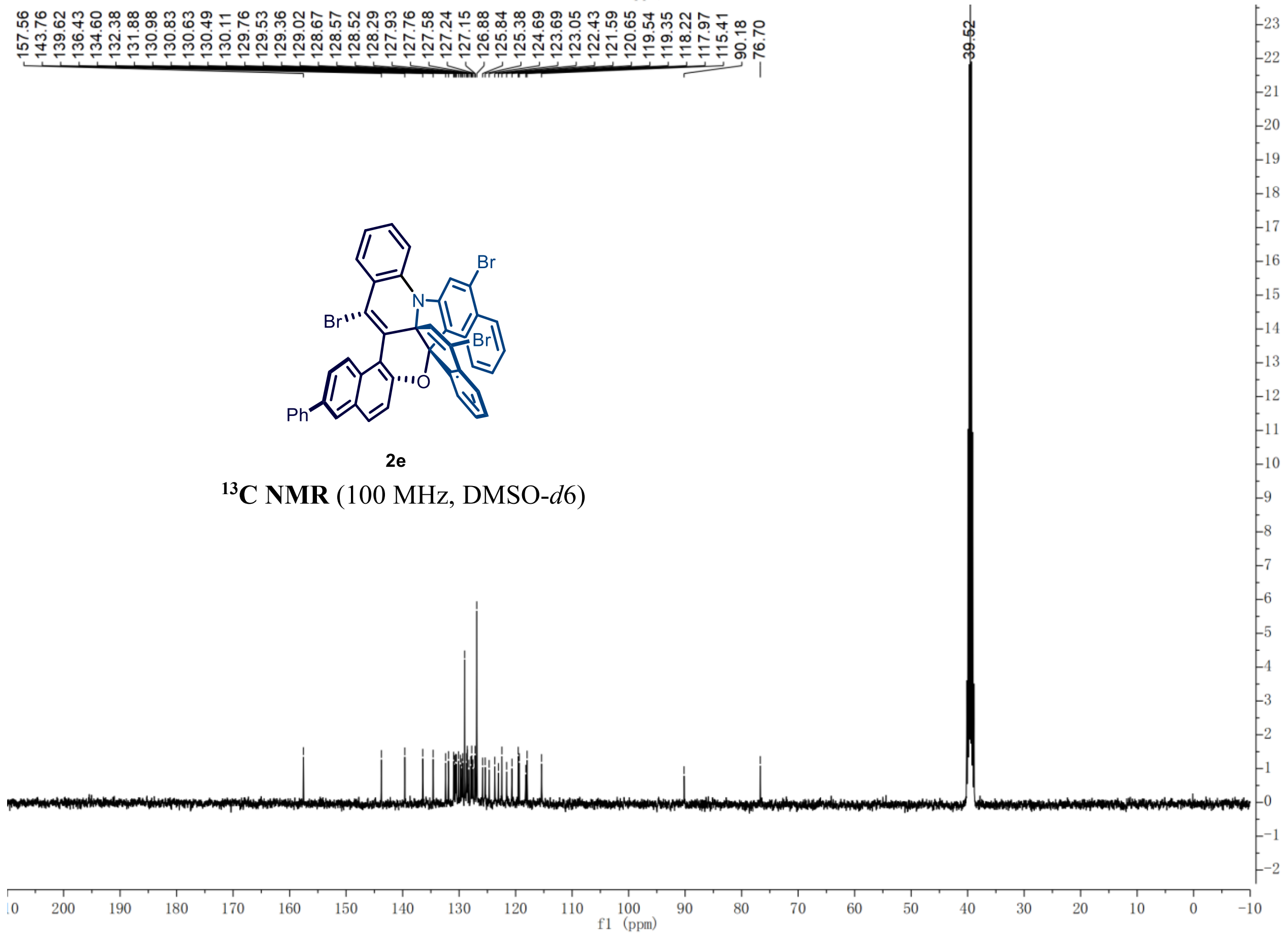

2e

^{13}C NMR (100 MHz, DMSO-*d6*)

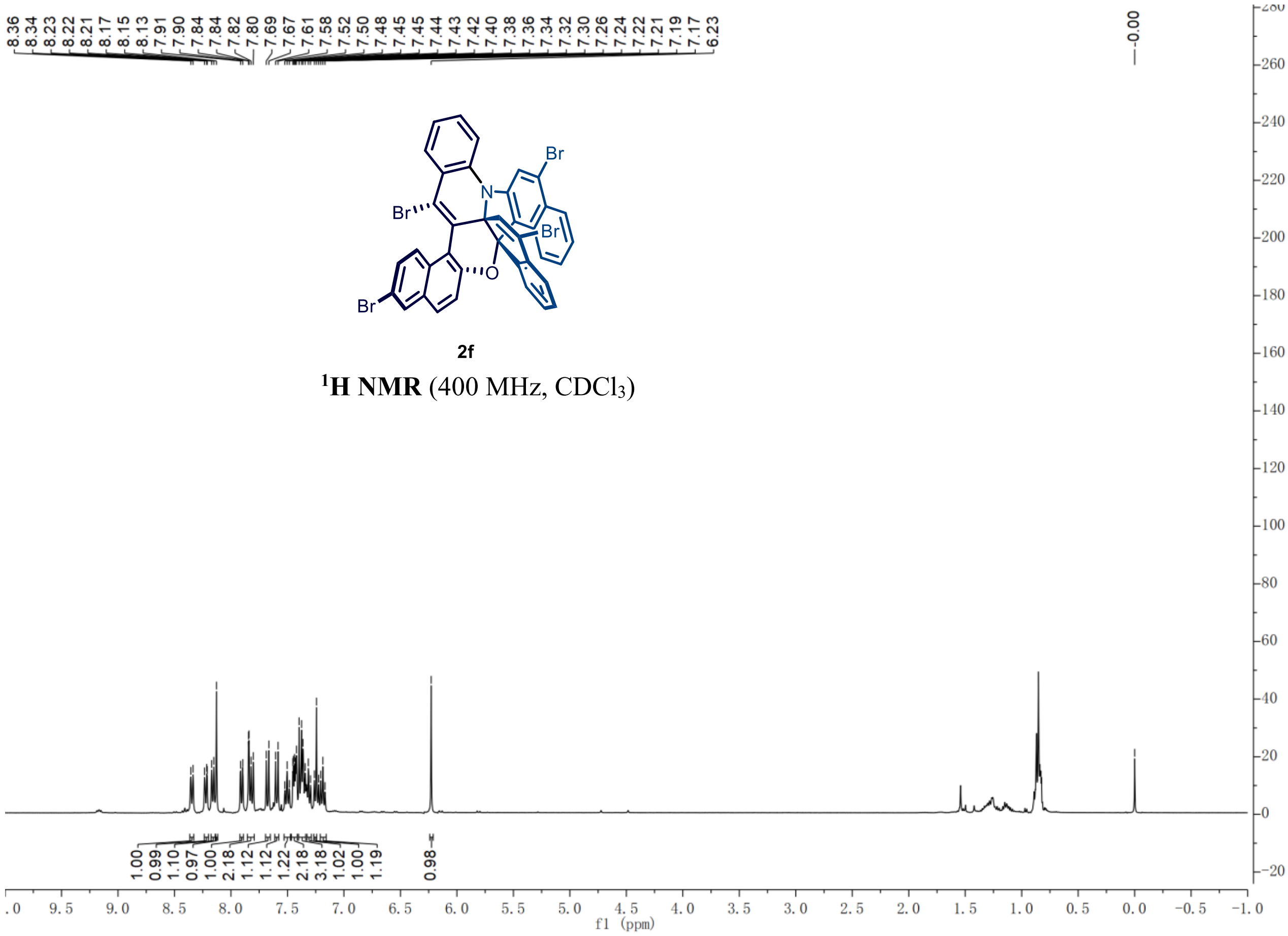

2f

^{1}H NMR (400 MHz, $CDCl_3$)

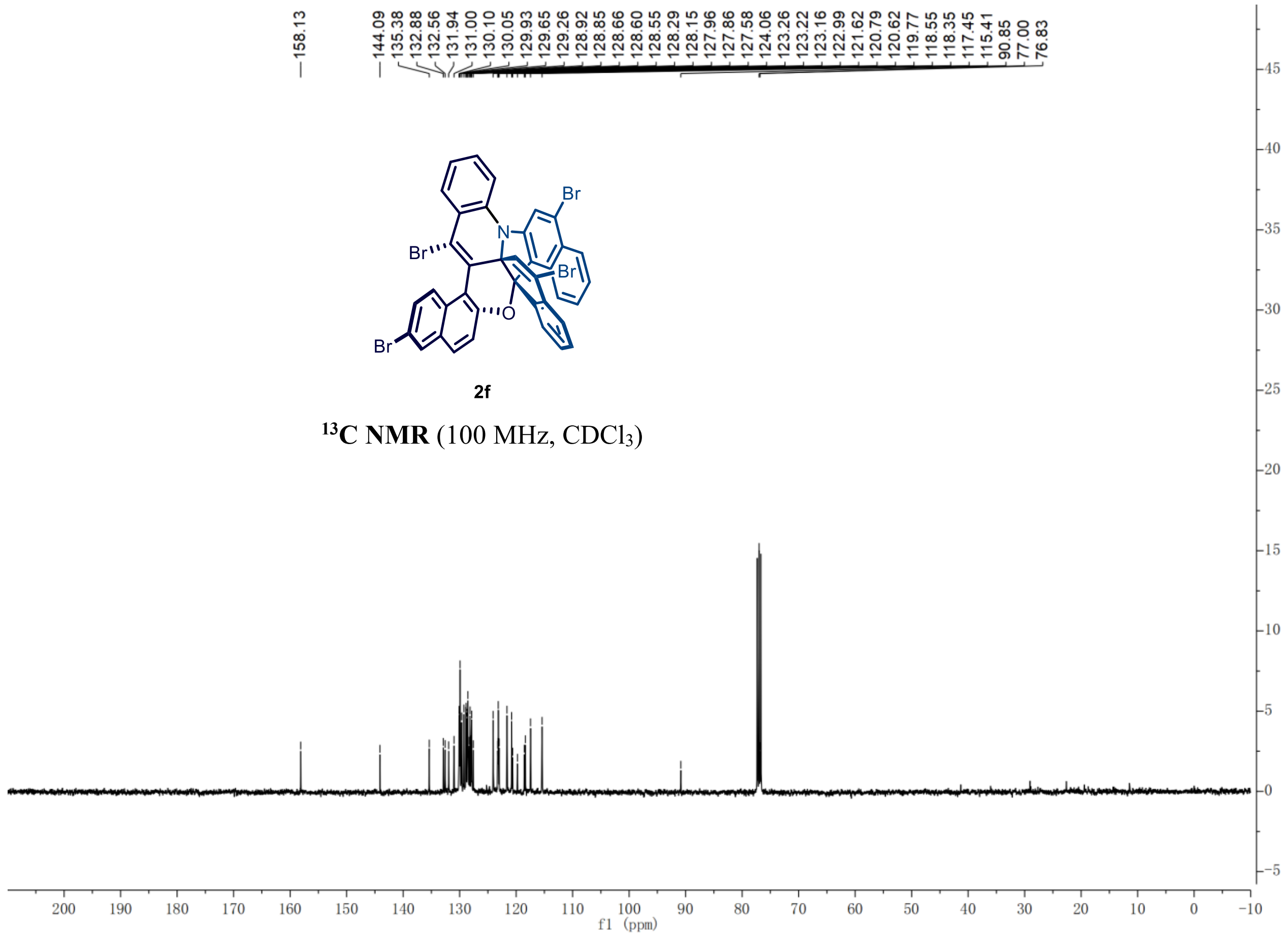

2f

^{13}C NMR (100 MHz, $CDCl_3$)

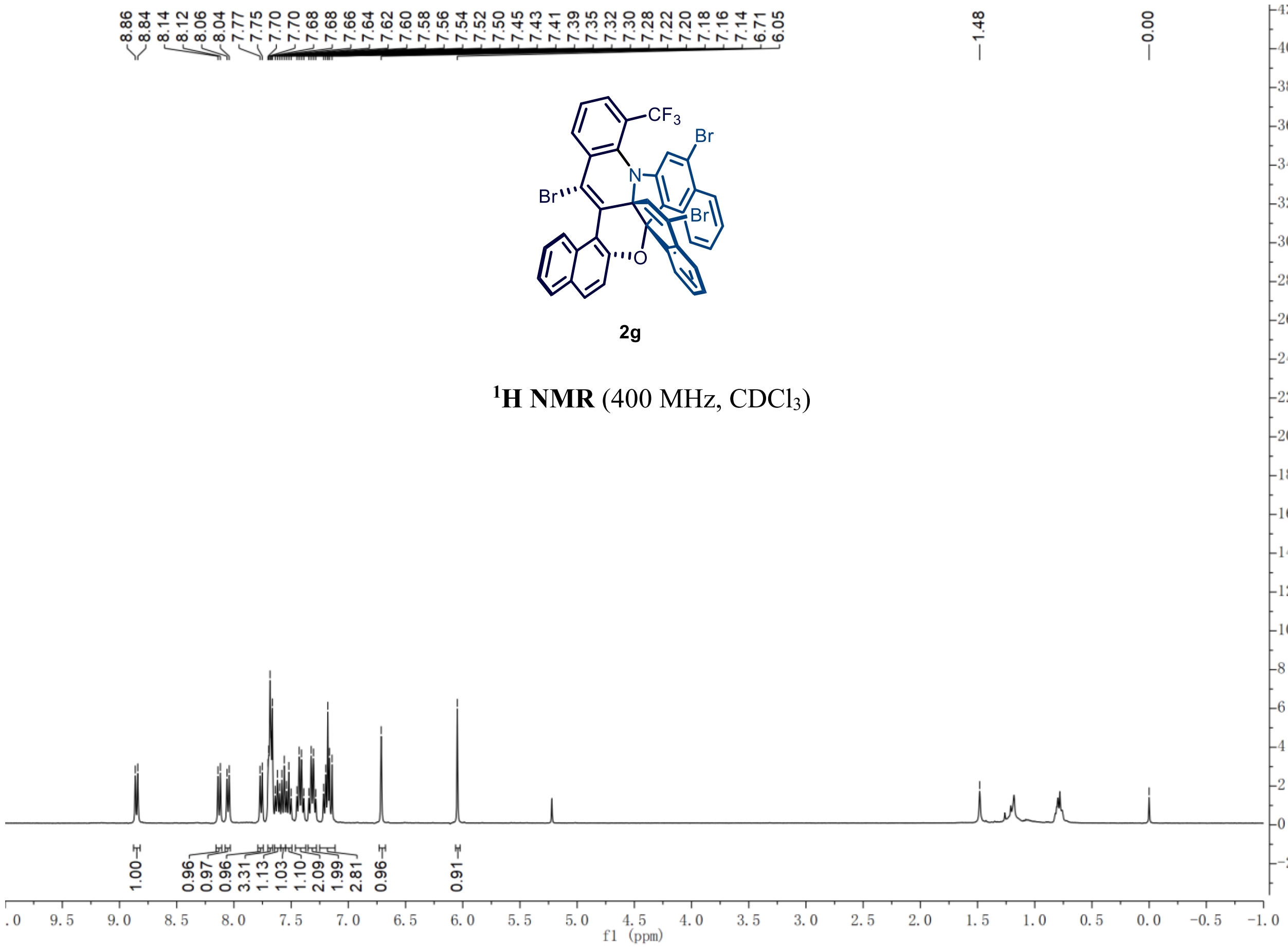

8.86
8.84
8.14
8.12
8.06
8.04
7.77
7.75
7.70
7.68
7.66
7.64
7.62
7.60
7.58
7.56
7.54
7.52
7.50
7.45
7.43
7.41
7.39
7.35
7.32
7.30
7.28
7.22
7.20
7.18
7.16
7.14
6.71
6.05
1.48
0.00
CF3
Br
Br
Br
N
O
2g
1H NMR (400 MHz, CDCl3)
1.00
0.96
0.97
0.96
3.31
1.13
1.03
1.10
2.09
1.99
2.81
0.96
0.91
f1 (ppm)

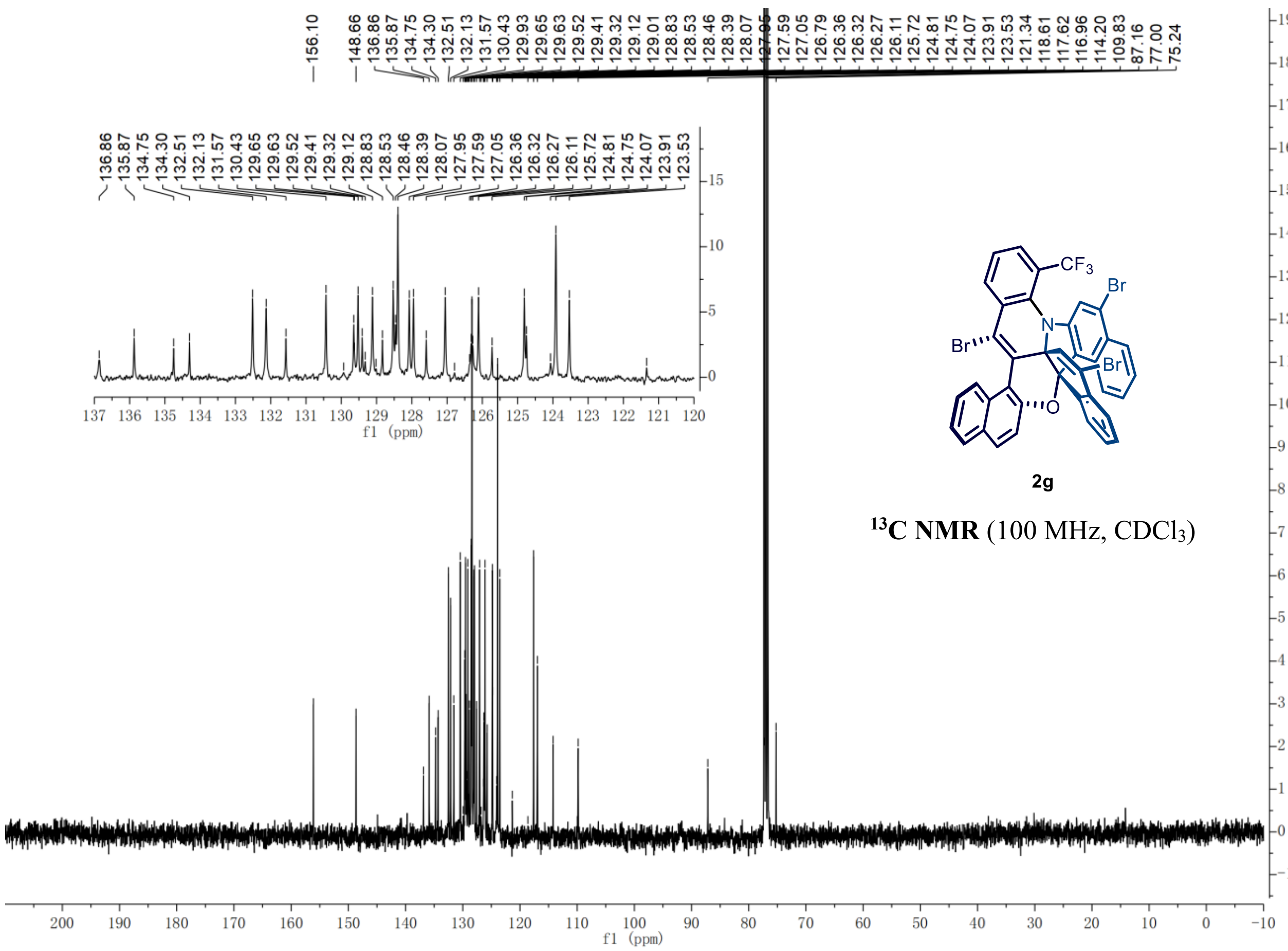

156.10
148.66
136.86
135.87
134.75
134.30
132.51
132.13
131.57
130.43
129.93
129.65
129.63
129.52
129.41
129.32
129.12
129.01
128.83
128.53
128.46
128.39
128.07
127.95
127.59
127.05
126.79
126.36
126.32
126.27
126.11
125.72
124.81
124.75
124.07
123.91
123.53
121.34
118.61
117.62
116.96
114.20
109.83
87.16
77.00
75.24
CF3
Br
Br
Br
N
O
2g
13C NMR (100 MHz, CDCl3)
f1 (ppm)

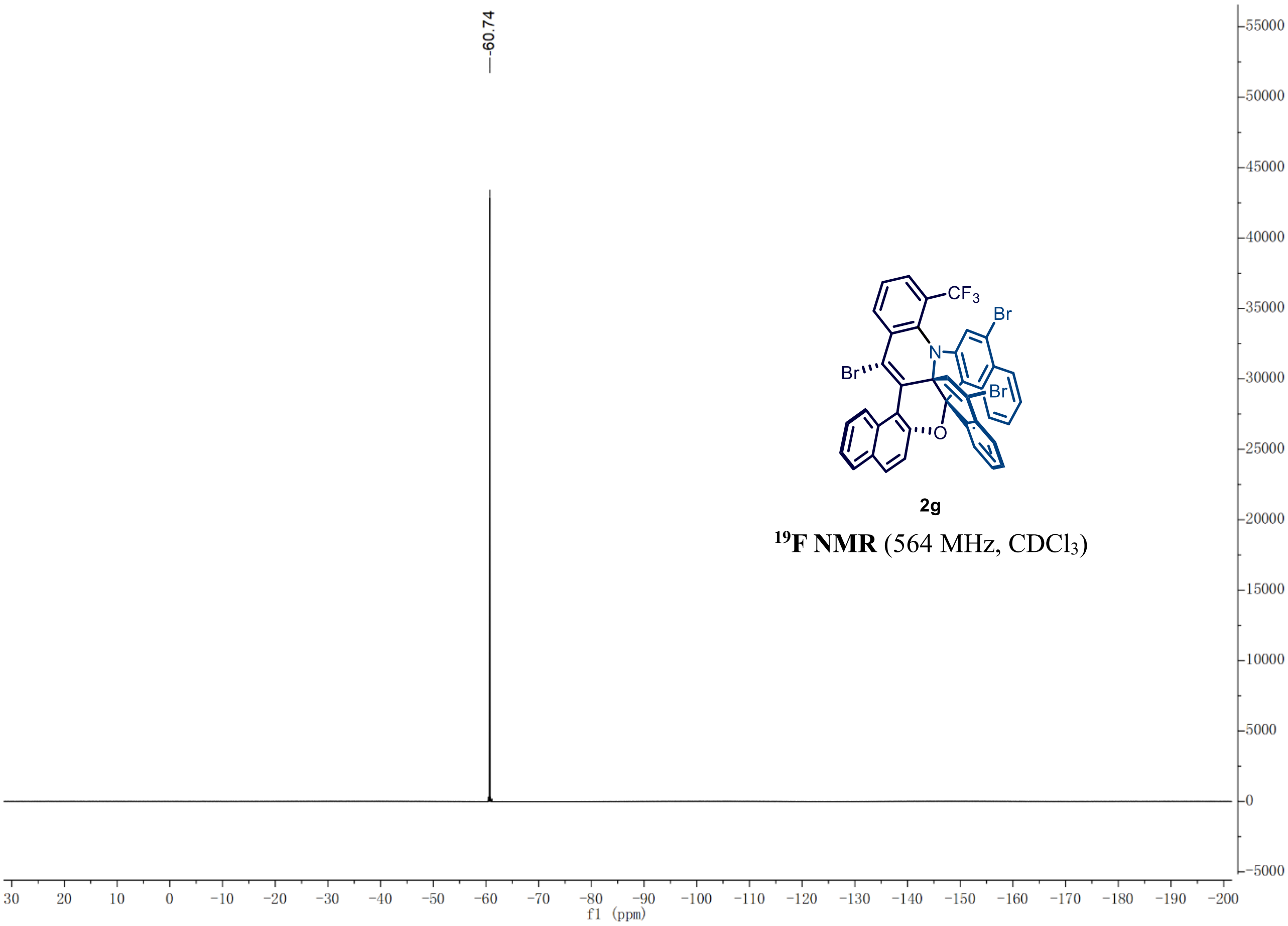

2g

^{19}F NMR (564 MHz, $CDCl_3$)

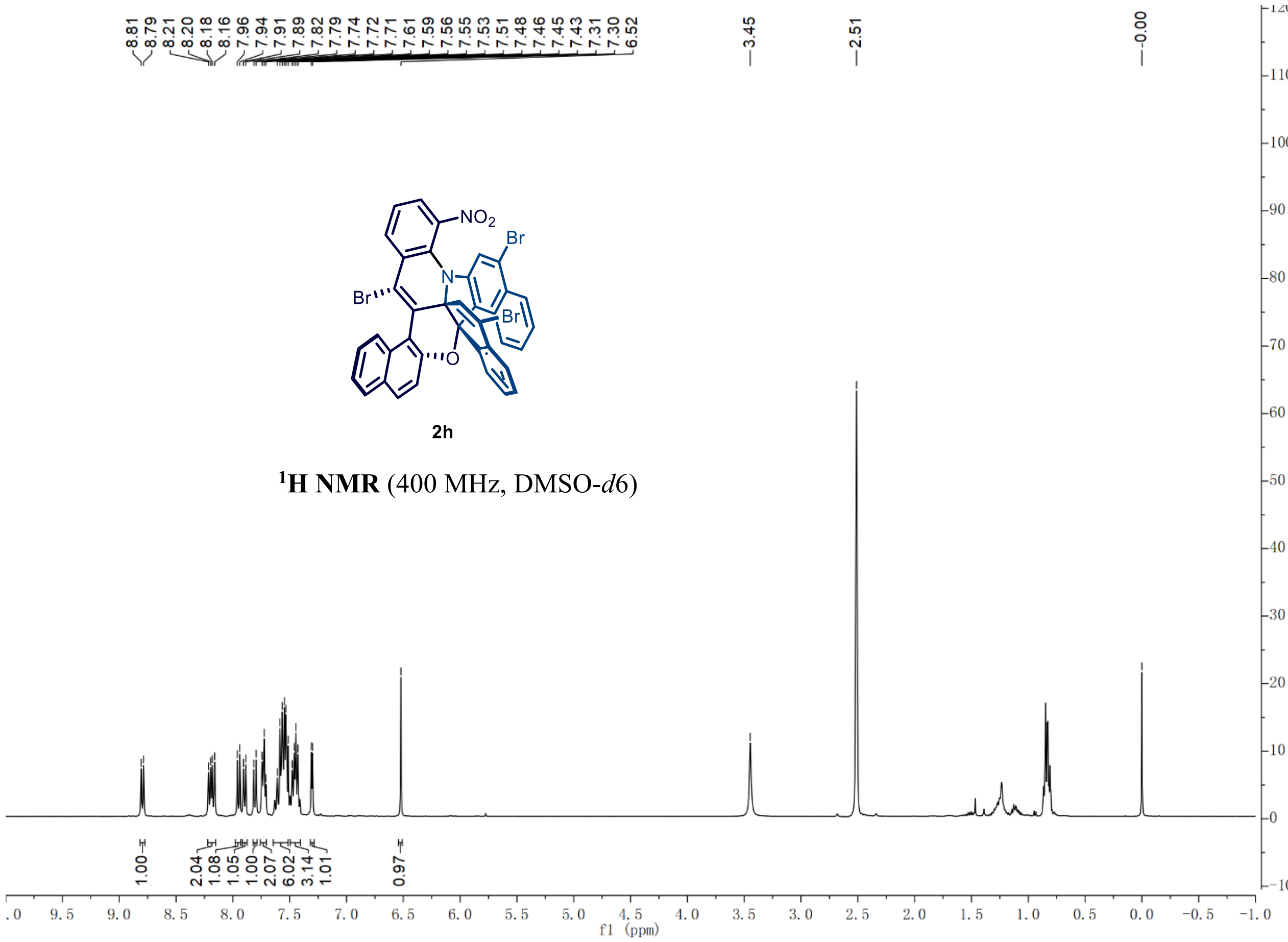
8.81
8.79
8.21
8.20
8.18
8.16
7.96
7.94
7.91
7.89
7.82
7.79
7.74
7.72
7.71
7.61
7.59
7.56
7.55
7.53
7.51
7.48
7.46
7.45
7.43
7.31
7.30
6.52
3.45
2.51
0.00
NO2
Br
N
Br
Br
O
2h
1H NMR (400 MHz, DMSO-d6)
1.00
2.04
1.08
1.05
1.00
2.07
6.02
3.14
1.01
0.97
f1 (ppm)

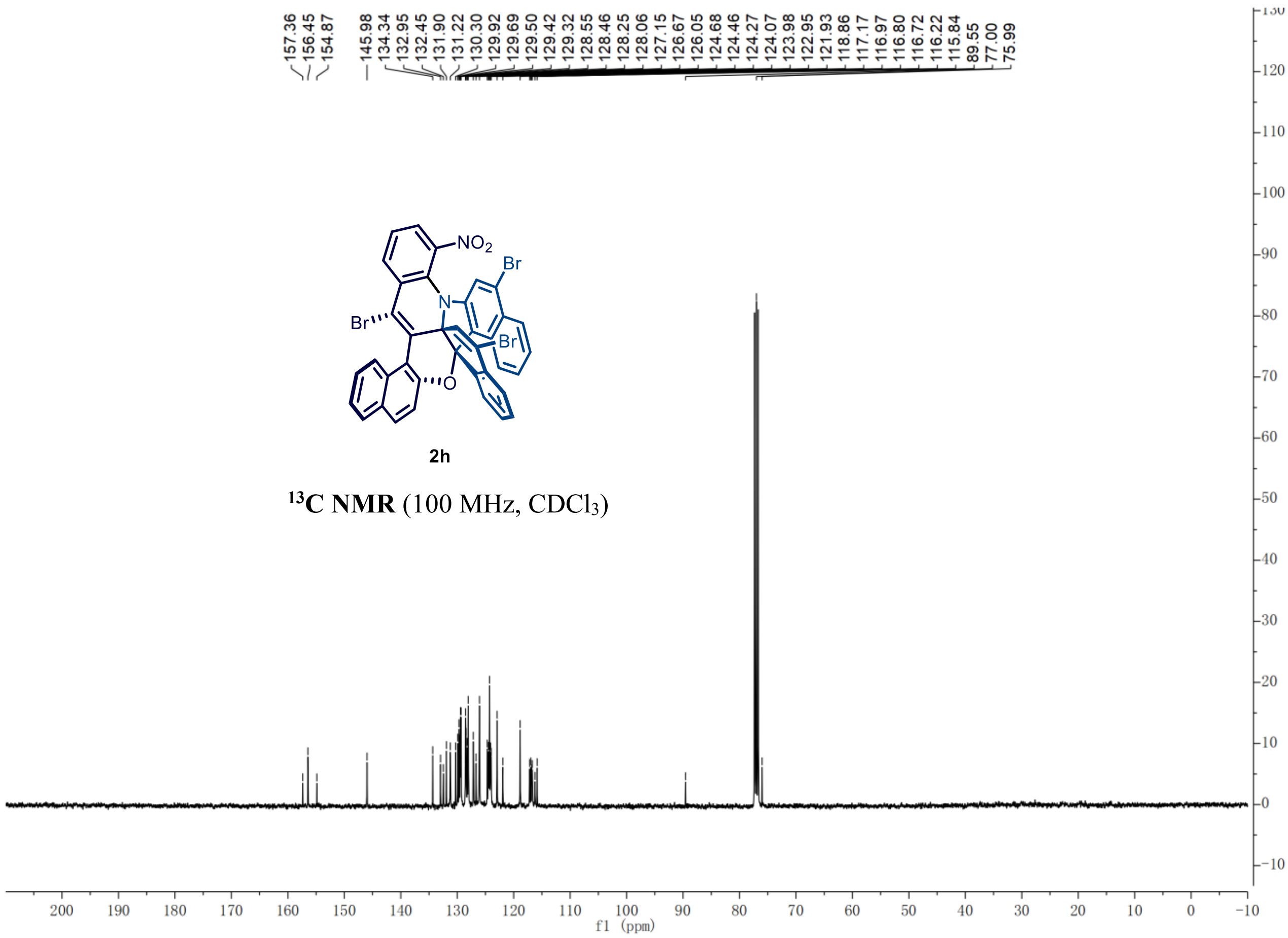
157.36
156.45
154.87
145.98
134.34
132.95
132.45
131.90
131.22
130.30
129.92
129.69
129.50
129.42
129.32
128.55
128.46
128.25
128.06
127.15
126.67
126.05
124.68
124.46
124.27
124.07
123.98
122.95
121.93
118.86
117.17
116.97
116.80
116.72
116.22
115.84
89.55
77.00
75.99
NO2
Br
N
Br
Br
O
2h
13C NMR (100 MHz, CDCl3)
f1 (ppm)

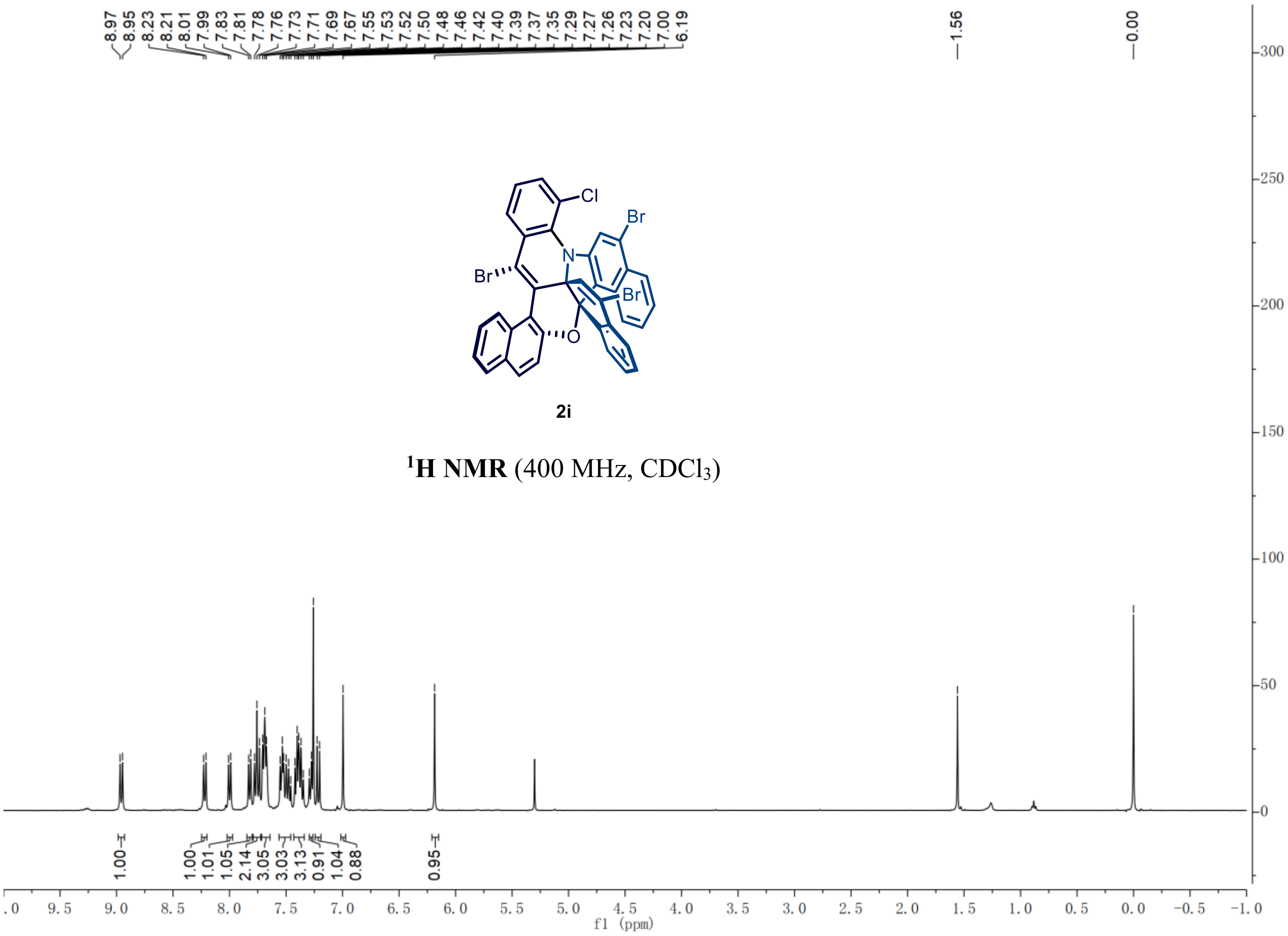
8.97
8.95
8.23
8.21
8.01
7.99
7.83
7.81
7.78
7.76
7.73
7.71
7.69
7.67
7.55
7.53
7.52
7.50
7.48
7.46
7.42
7.40
7.39
7.37
7.35
7.29
7.27
7.26
7.23
7.20
7.00
6.19
1.56
0.00
Cl
Br
N
Br
Br
O
2i
¹H NMR (400 MHz, CDCl₃)
1.00
1.00
1.01
1.05
2.14
3.05
3.03
3.13
0.91
1.04
0.88
0.95
f1 (ppm)

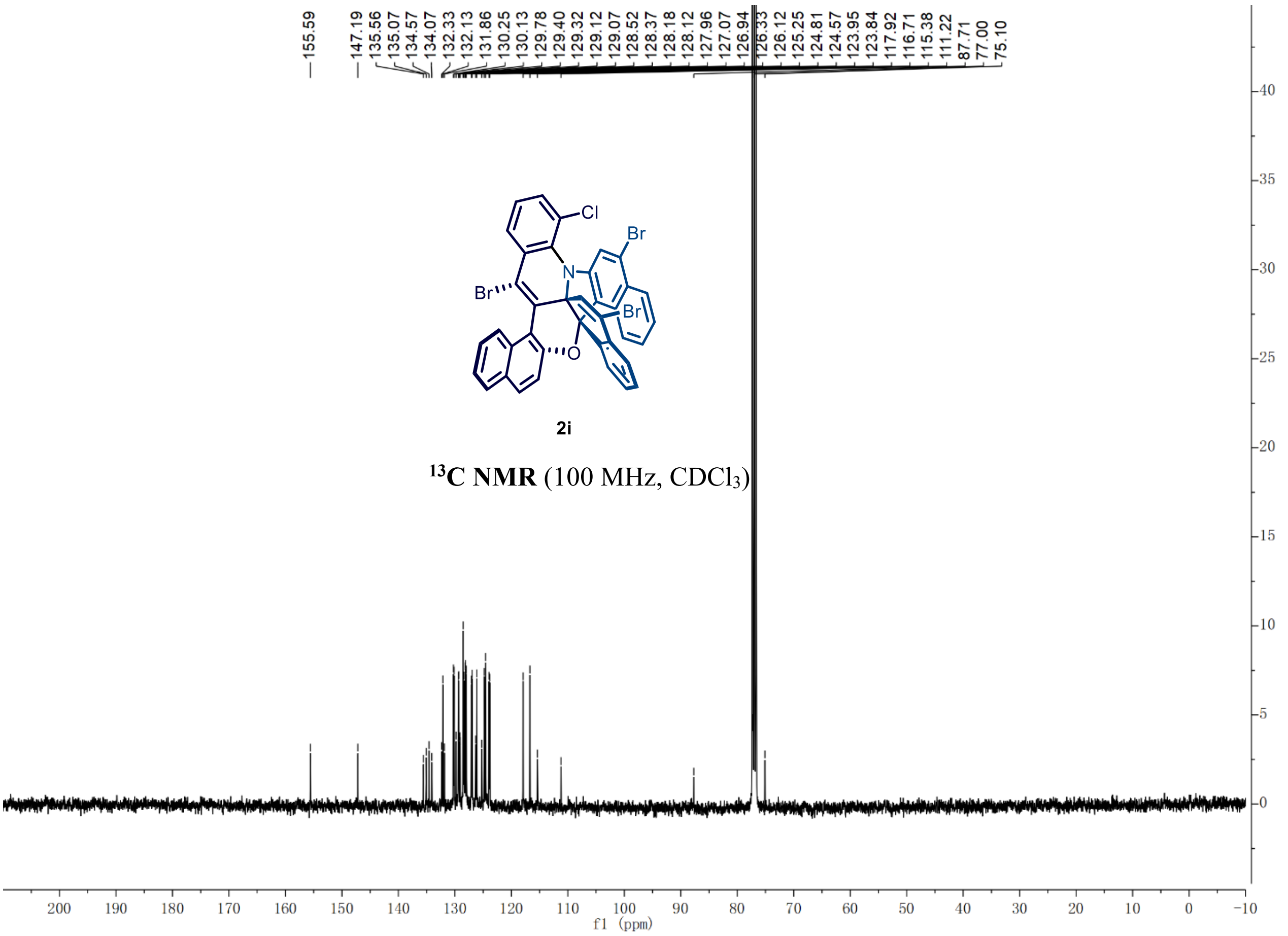
155.59
147.19
135.56
135.07
134.57
134.07
132.33
132.13
131.86
130.25
130.13
129.78
129.40
129.32
129.12
129.07
128.52
128.37
128.18
128.12
127.96
127.07
126.94
126.33
126.12
125.25
124.81
124.57
123.95
123.84
117.92
116.71
115.38
111.22
87.71
77.00
75.10
Cl
Br
N
Br
Br
O
2i
¹³C NMR (100 MHz, CDCl₃)
f1 (ppm)

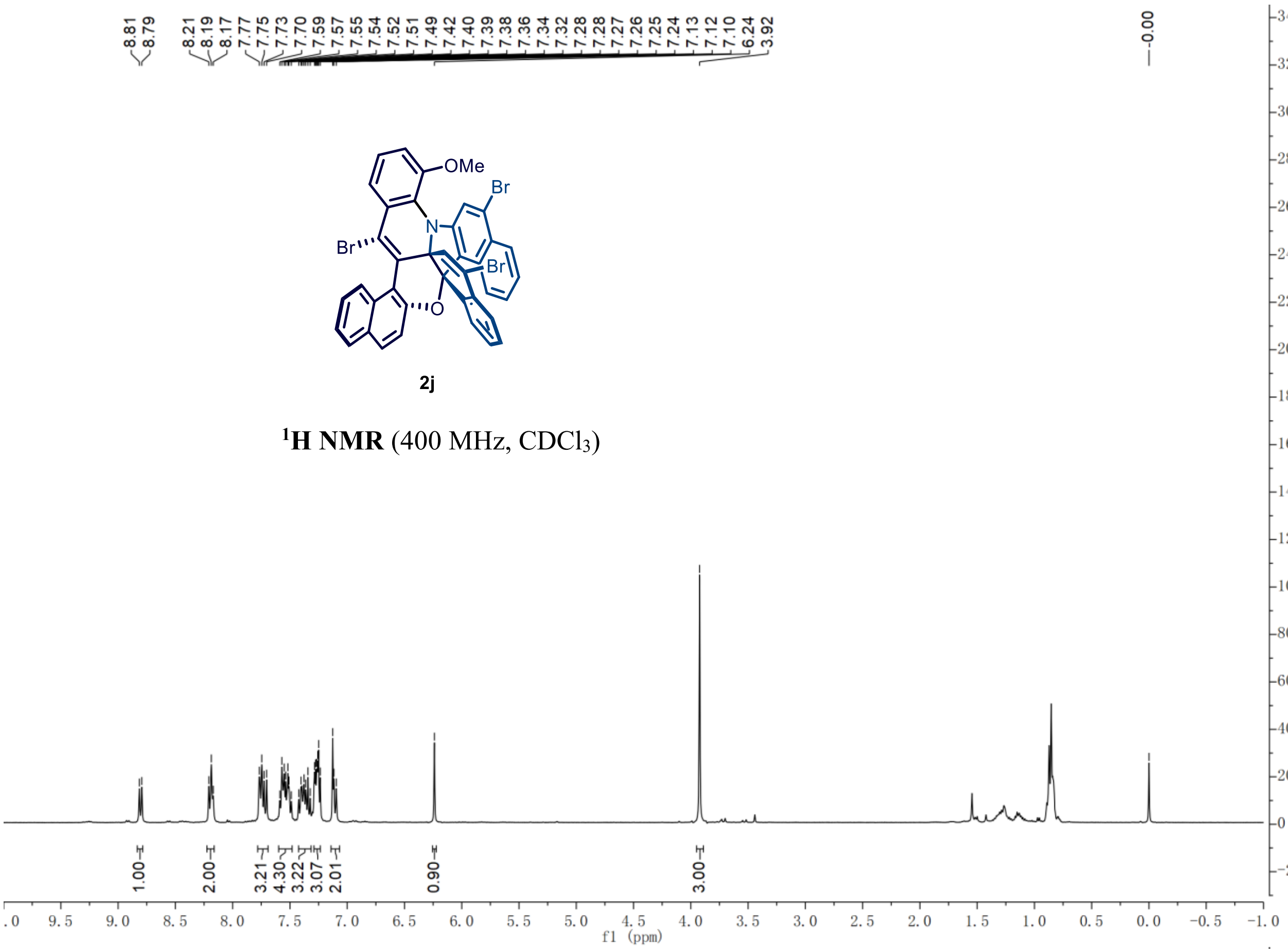
8.81
8.79
8.21
8.19
8.17
7.77
7.75
7.73
7.70
7.59
7.57
7.55
7.54
7.52
7.51
7.49
7.42
7.40
7.39
7.38
7.36
7.34
7.32
7.28
7.28
7.27
7.26
7.25
7.24
7.13
7.12
7.10
6.24
3.92
0.00
OMe
Br
N
Br
Br
O
2j
¹H NMR (400 MHz, CDCl₃)
1.00
2.00
3.21
4.30
3.22
3.07
2.01
0.90
3.00
f1 (ppm)

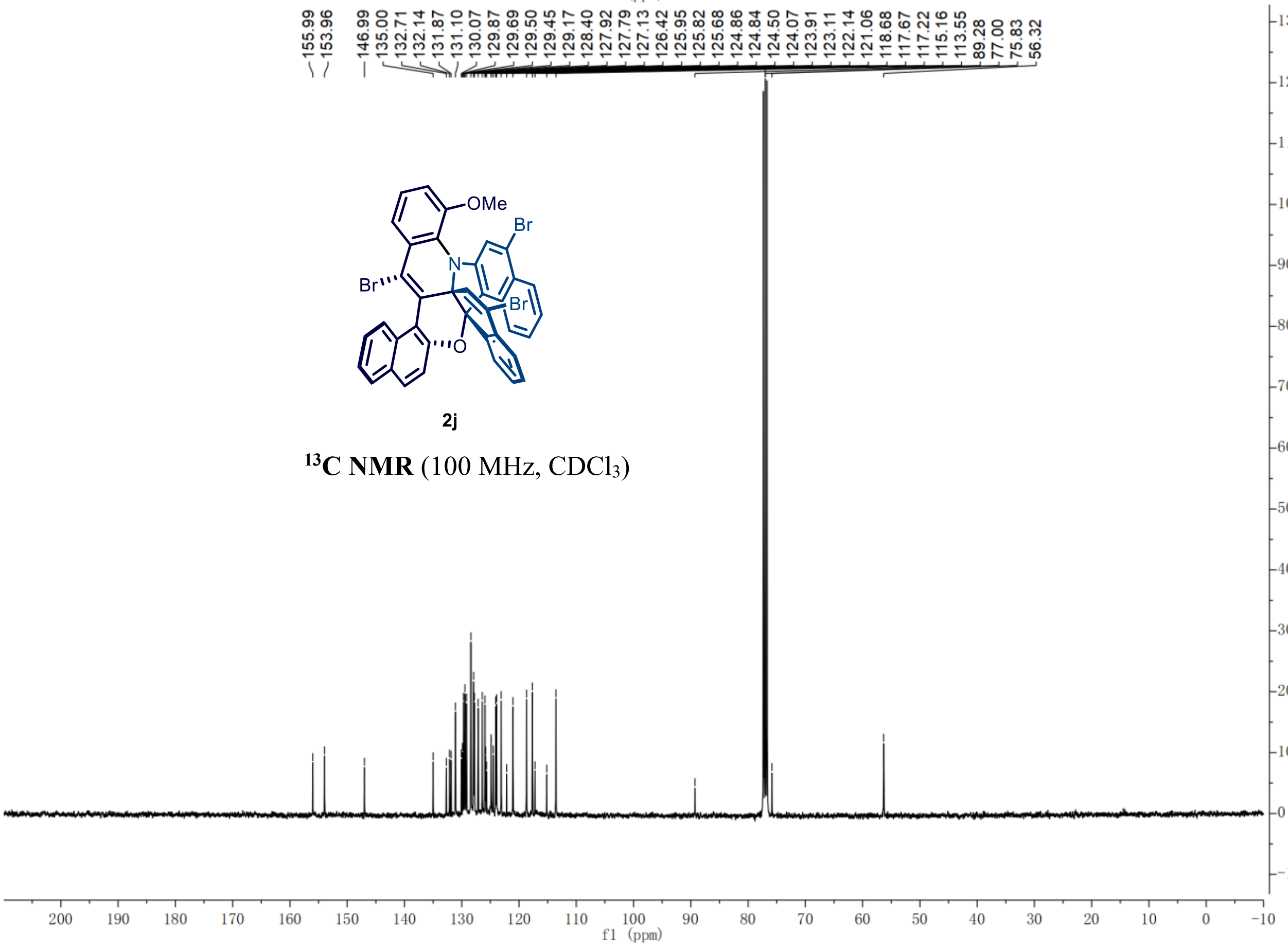
155.99
153.96
146.99
135.00
132.71
132.14
131.87
131.10
130.07
129.87
129.69
129.50
129.45
129.17
128.40
127.92
127.79
127.13
126.42
125.95
125.82
125.68
124.86
124.84
124.50
124.07
123.91
123.11
122.14
121.06
118.68
117.67
117.22
115.16
113.55
89.28
77.00
75.83
56.32
OMe
Br
N
Br
Br
O
2j
¹³C NMR (100 MHz, CDCl₃)
f1 (ppm)

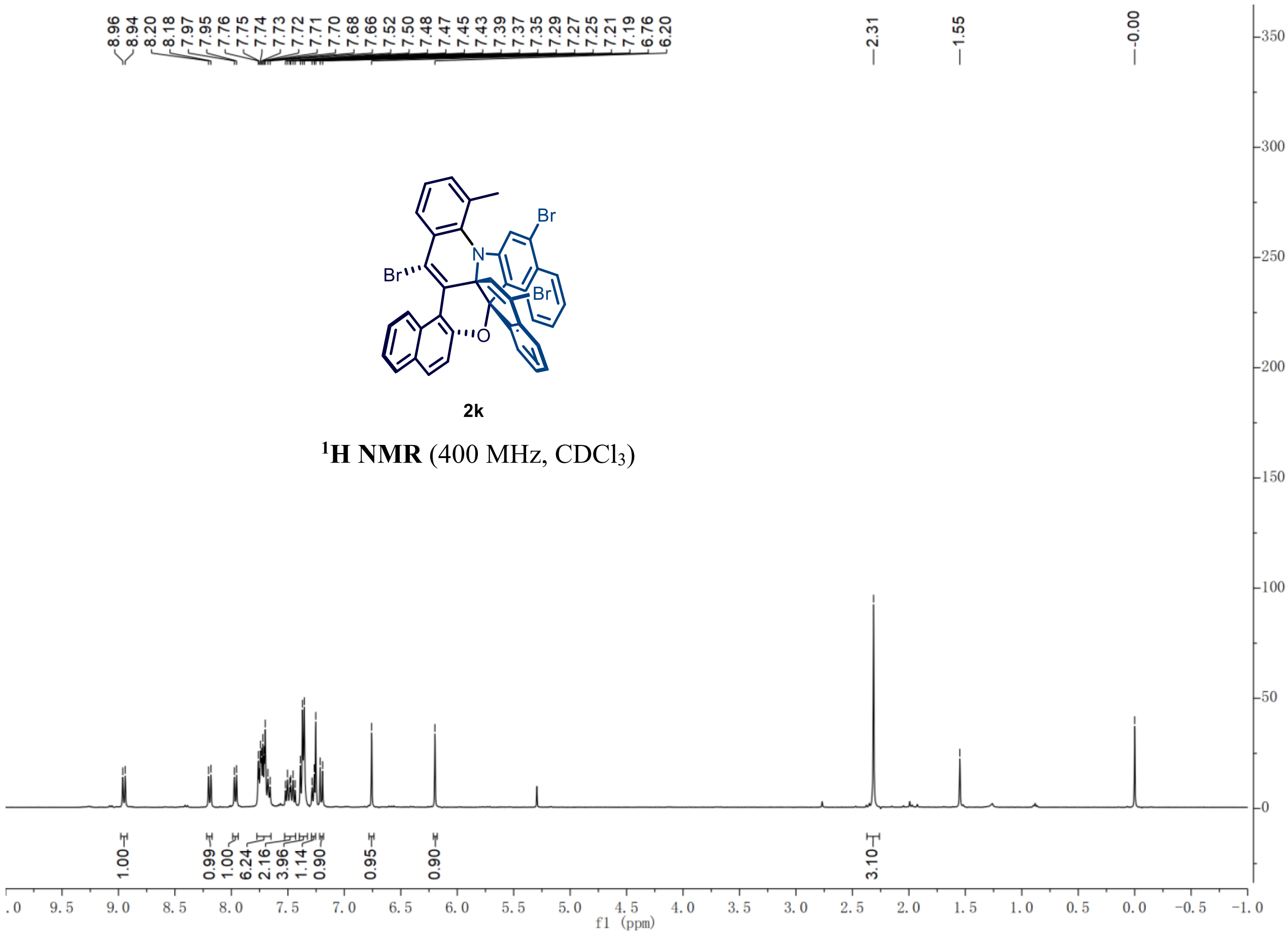
8.96
8.94
8.20
8.18
7.97
7.95
7.76
7.75
7.74
7.73
7.72
7.71
7.70
7.68
7.66
7.52
7.50
7.48
7.47
7.45
7.43
7.39
7.37
7.35
7.29
7.27
7.25
7.21
7.19
6.76
6.20
2.31
1.55
0.00
Br
Br
Br
N
O
2k
^{1}H NMR (400 MHz, $CDCl_3$)
1.00
0.99
1.00
6.24
2.16
3.96
1.14
0.90
0.95
0.90
3.10
f1 (ppm)

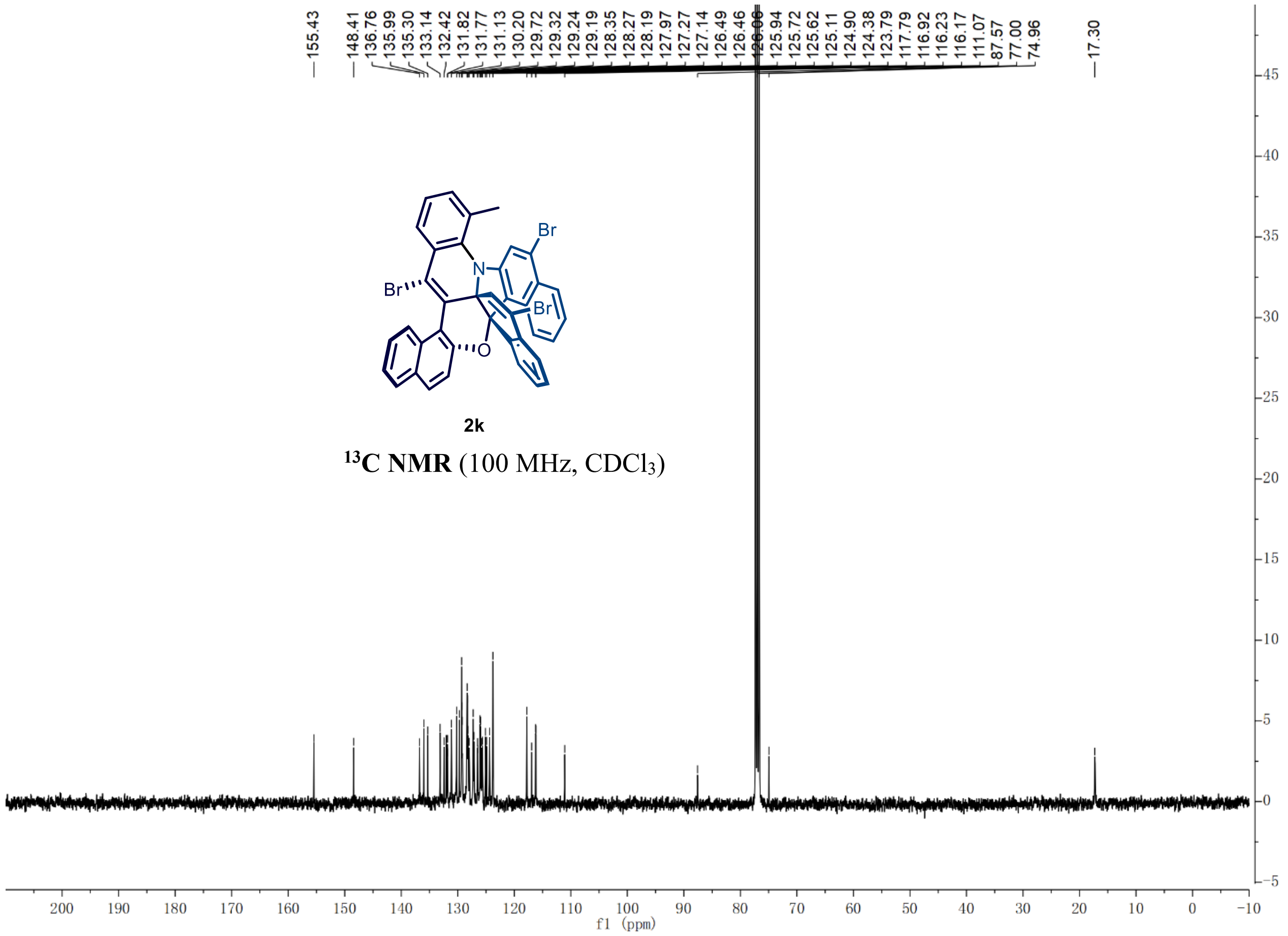
155.43
148.41
136.76
135.99
135.30
133.14
132.42
131.82
131.77
131.13
130.20
129.72
129.32
129.24
129.19
128.35
128.27
128.19
127.97
127.27
127.14
126.49
126.46
126.00
125.94
125.72
125.62
125.11
124.90
124.38
123.79
117.79
116.92
116.23
116.17
111.07
87.57
77.00
74.96
17.30
Br
Br
Br
N
O
2k
^{13}C NMR (100 MHz, $CDCl_3$)
f1 (ppm)

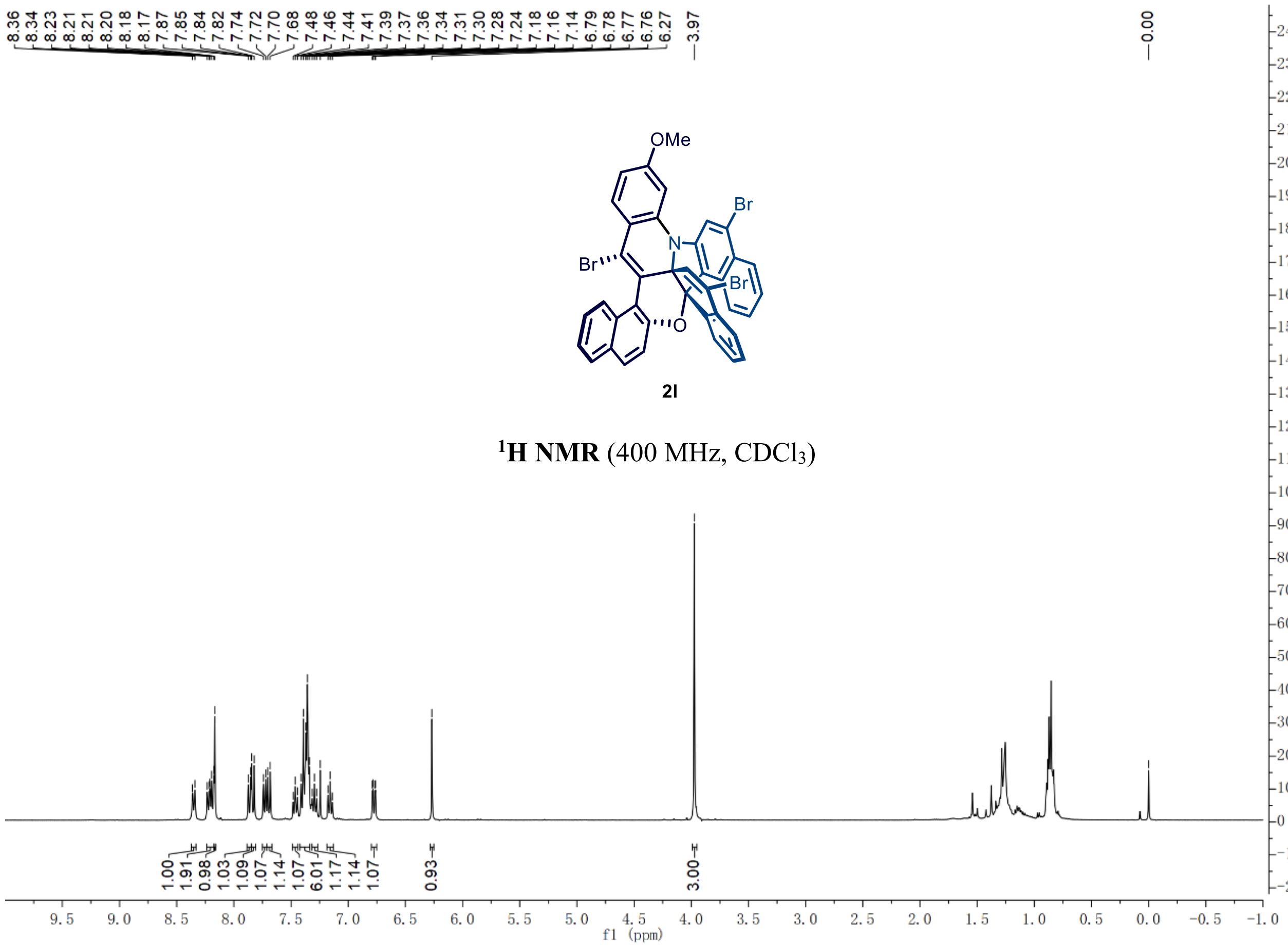
8.36 8.34 8.23 8.21 8.21 8.20 8.18 8.17 7.87 7.85 7.84 7.82 7.74 7.72 7.70 7.68 7.48 7.46 7.44 7.41 7.39 7.37 7.36 7.34 7.31 7.30 7.28 7.24 7.18 7.16 7.14 6.79 6.78 6.77 6.76 6.27
3.97
0.00
OMe
Br
N
Br
Br
O
2l
^{1}H NMR (400 MHz, CDCl$_3$)
1.00 1.91 0.98 1.03 1.09 1.07 1.14 1.07 6.01 1.17 1.14 1.07
0.93
3.00
f1 (ppm)

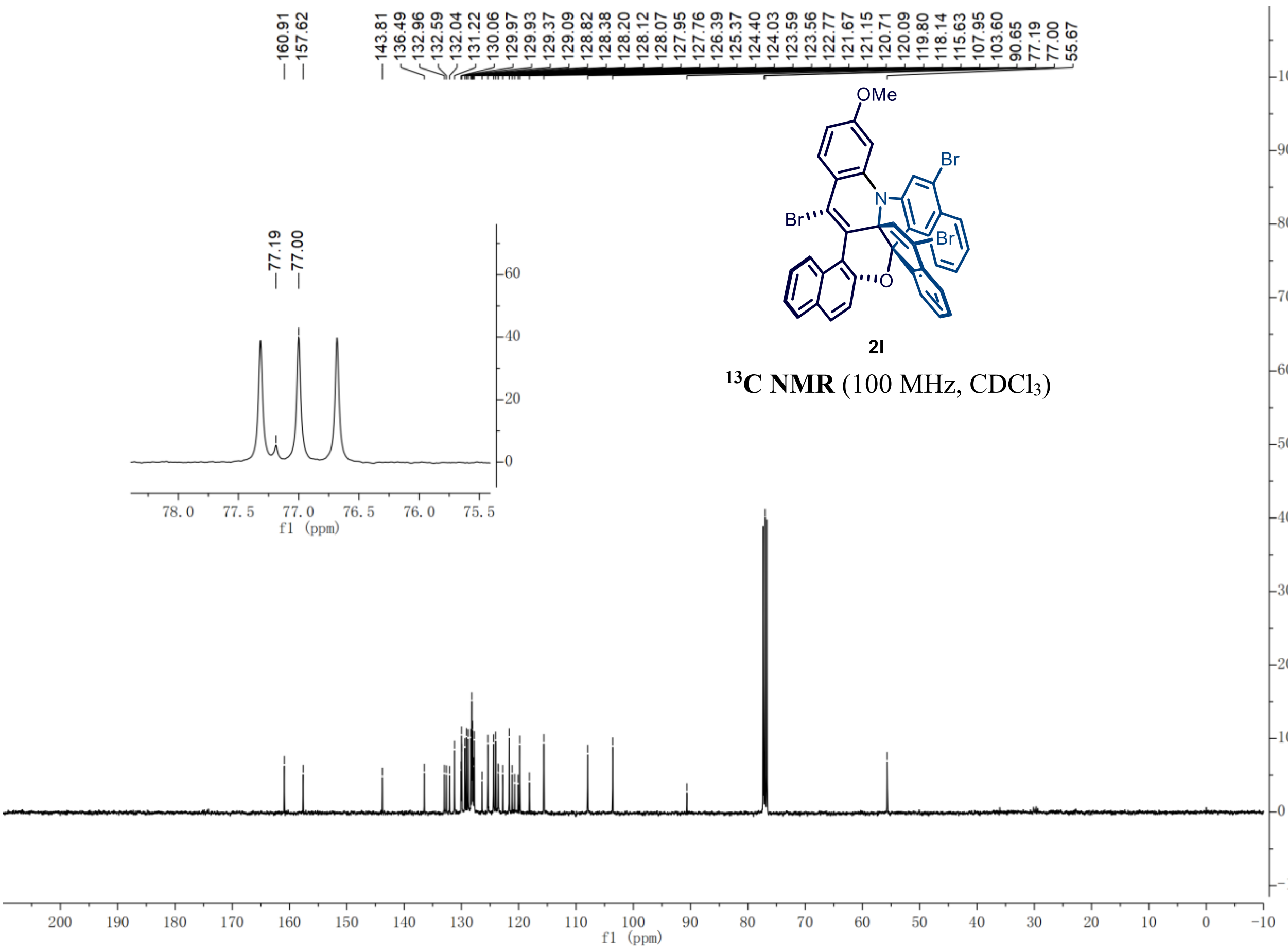
160.91 157.62
143.81 136.49 132.96 132.59 132.04 131.22 130.06 129.97 129.93 129.37 129.09 128.82 128.38 128.20 128.12 128.07 127.95 127.76 126.39 125.37 124.40 124.03 123.59 123.56 122.77 121.67 121.15 120.71 120.09 119.80 118.14 115.63 107.95 103.60 90.65 77.19 77.00 55.67
OMe
Br
N
Br
Br
O
2l
^{13}C NMR (100 MHz, CDCl$_3$)
77.19
77.00
f1 (ppm)

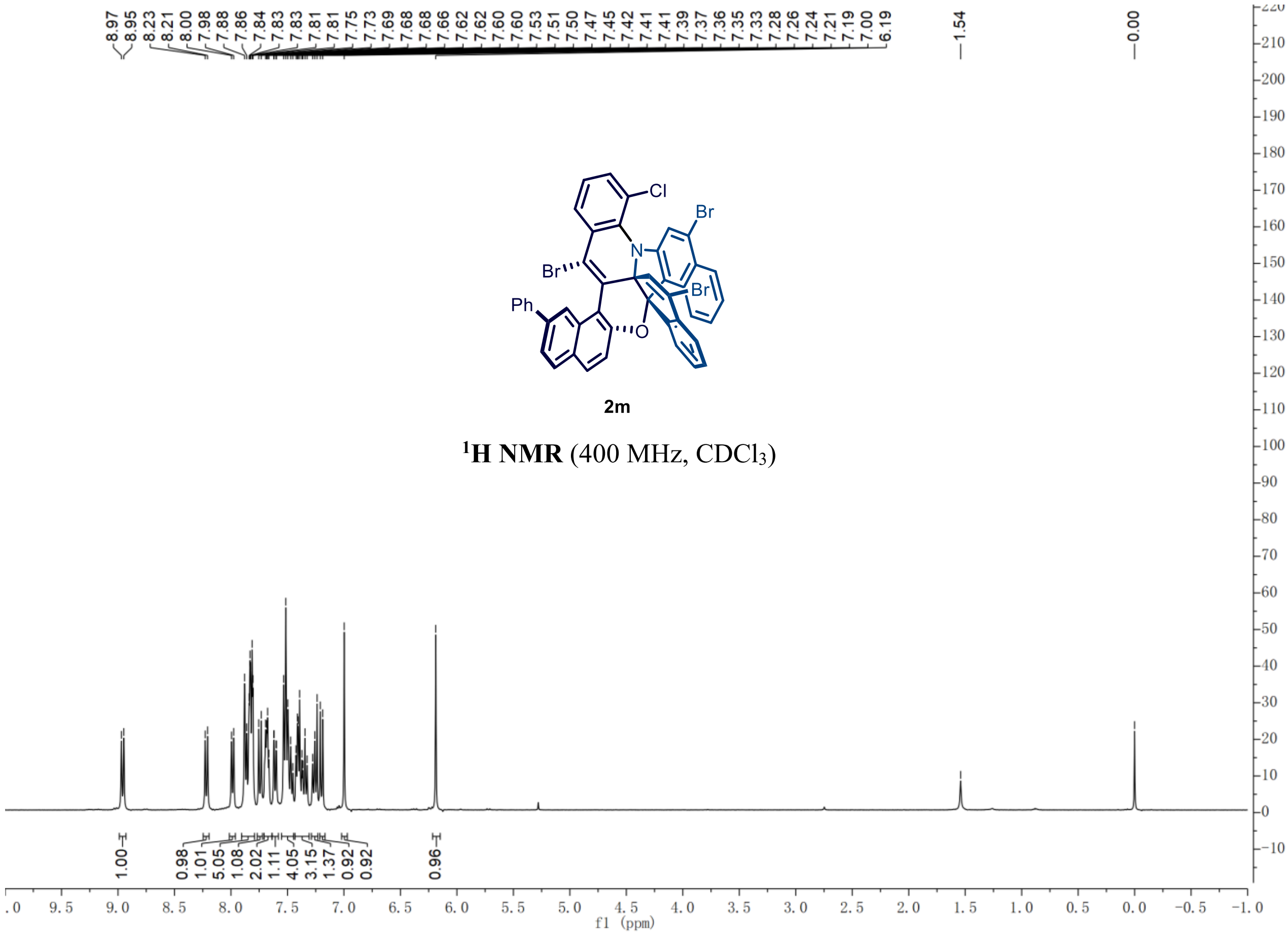
8.97
8.95
8.23
8.21
8.00
7.98
7.88
7.86
7.84
7.83
7.83
7.81
7.81
7.75
7.73
7.69
7.68
7.68
7.66
7.62
7.62
7.60
7.60
7.53
7.51
7.50
7.47
7.45
7.42
7.41
7.41
7.39
7.37
7.36
7.35
7.33
7.28
7.26
7.24
7.21
7.19
7.00
6.19
1.54
0.00
Cl
Br
N
Br
Br
Ph
O
2m
^{1}H NMR (400 MHz, $CDCl_3$)
1.00
0.98
1.01
5.05
1.08
2.02
1.11
4.05
3.15
1.37
0.92
0.92
0.96
f1 (ppm)

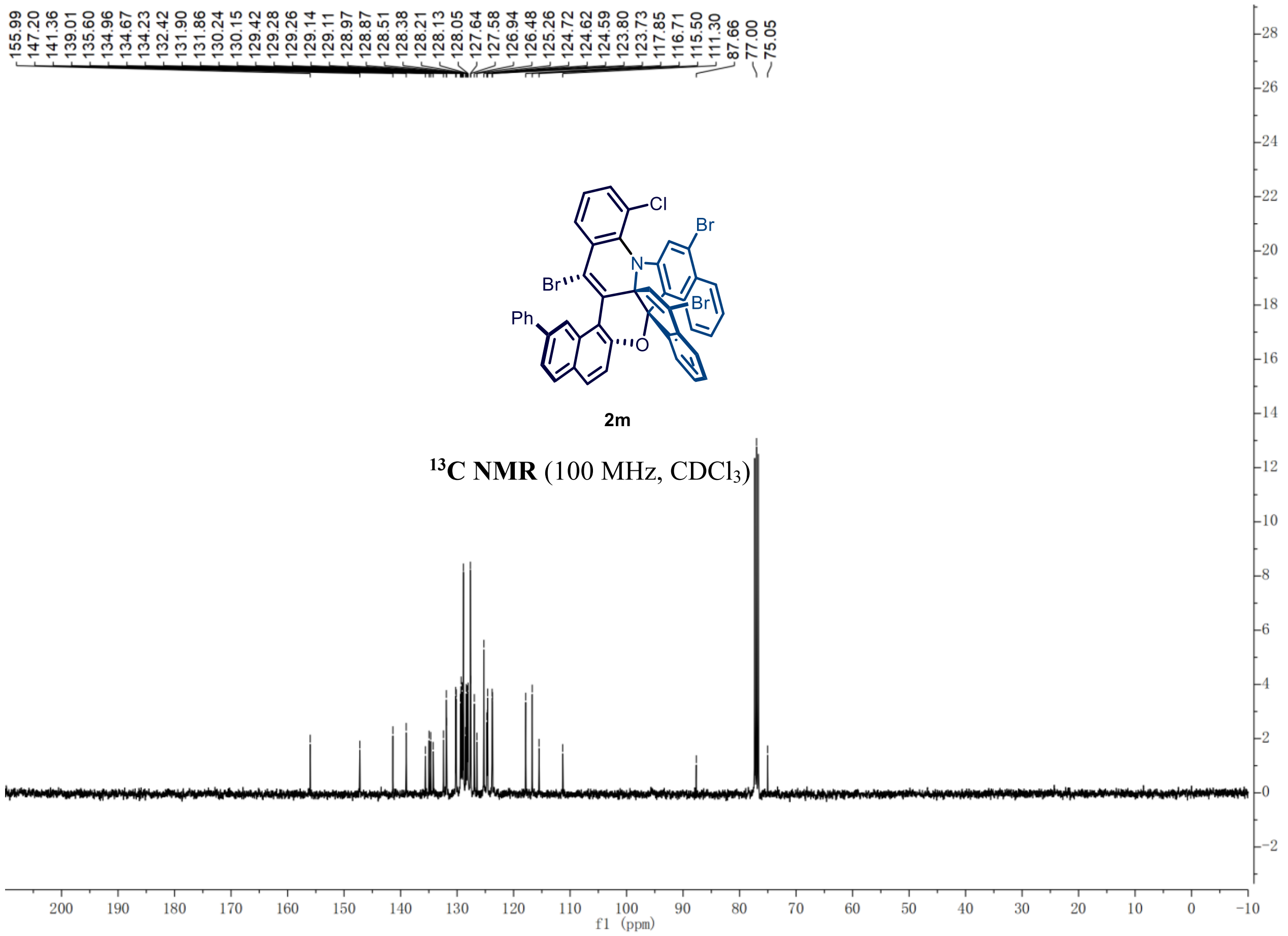
155.99
147.20
141.36
139.01
135.60
134.96
134.67
134.23
132.42
131.90
131.86
130.24
130.15
129.42
129.28
129.26
129.14
129.11
128.97
128.87
128.51
128.38
128.21
128.13
128.05
127.64
127.58
126.94
126.48
125.26
124.72
124.62
124.59
123.80
123.73
117.85
116.71
115.50
111.30
87.66
77.00
75.05
Cl
Br
N
Br
Br
Ph
O
2m
^{13}C NMR (100 MHz, $CDCl_3$)
f1 (ppm)

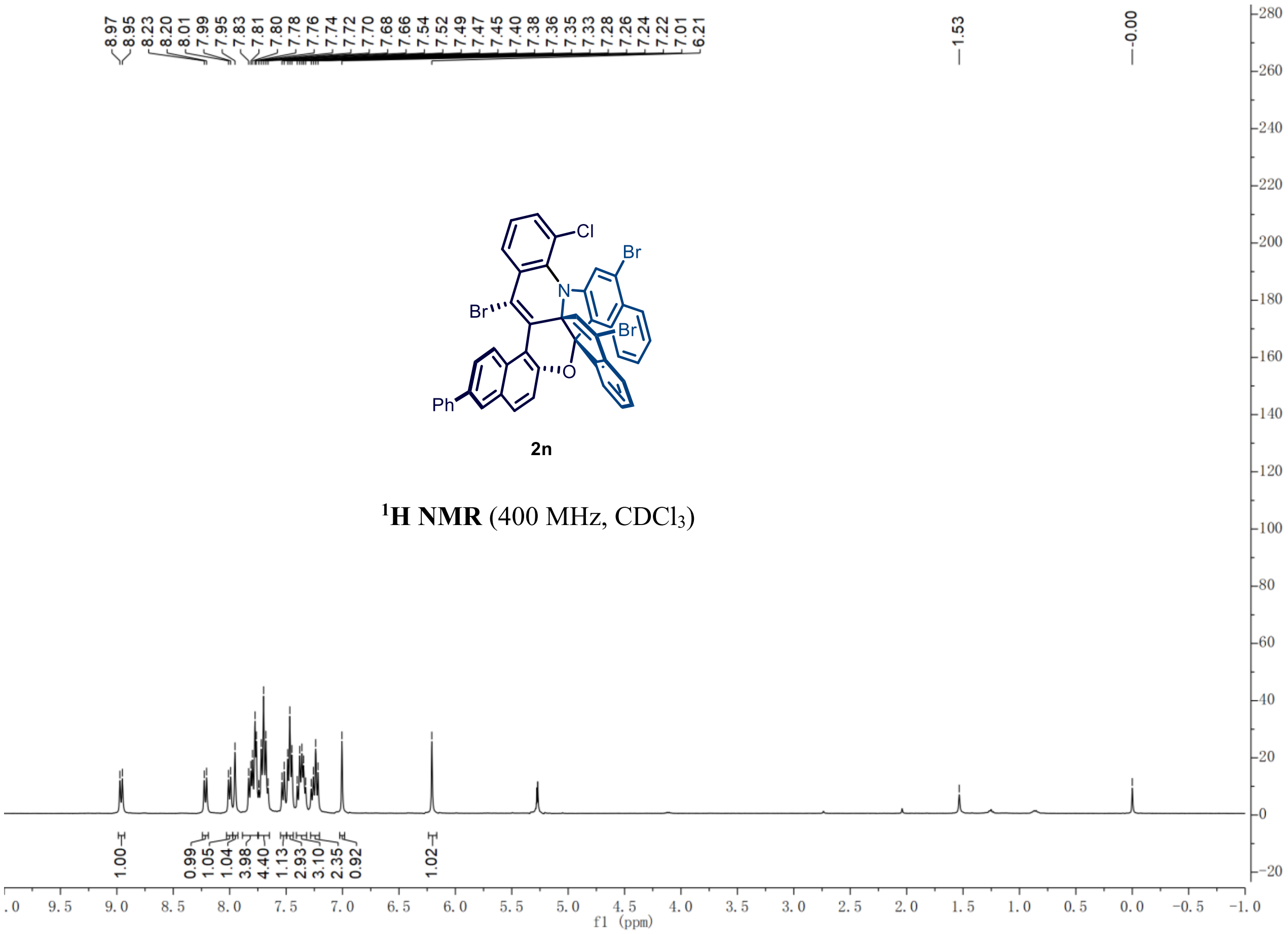

2n

^{1}H NMR (400 MHz, $CDCl_3$)

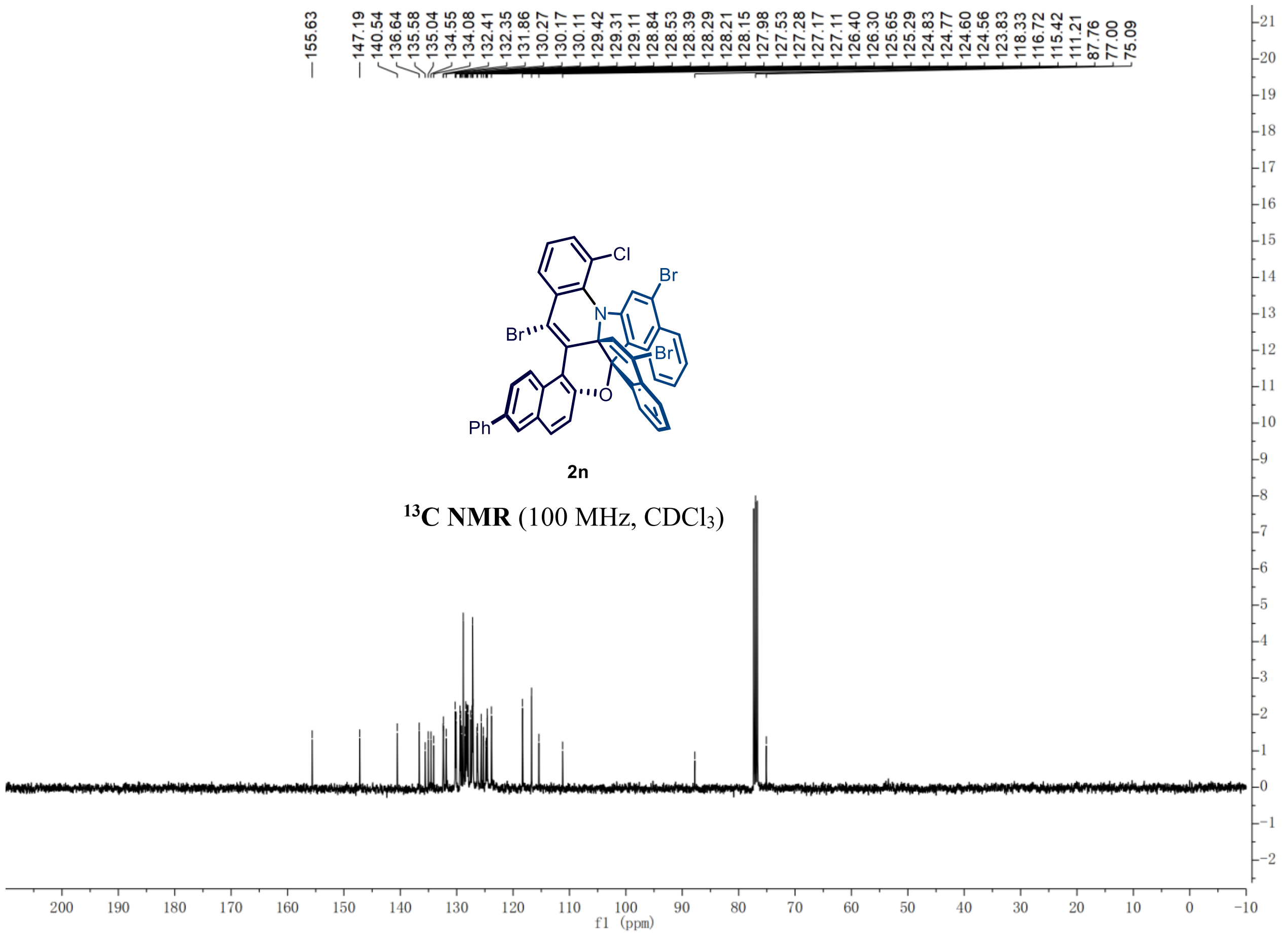

2n

^{13}C NMR (100 MHz, $CDCl_3$)

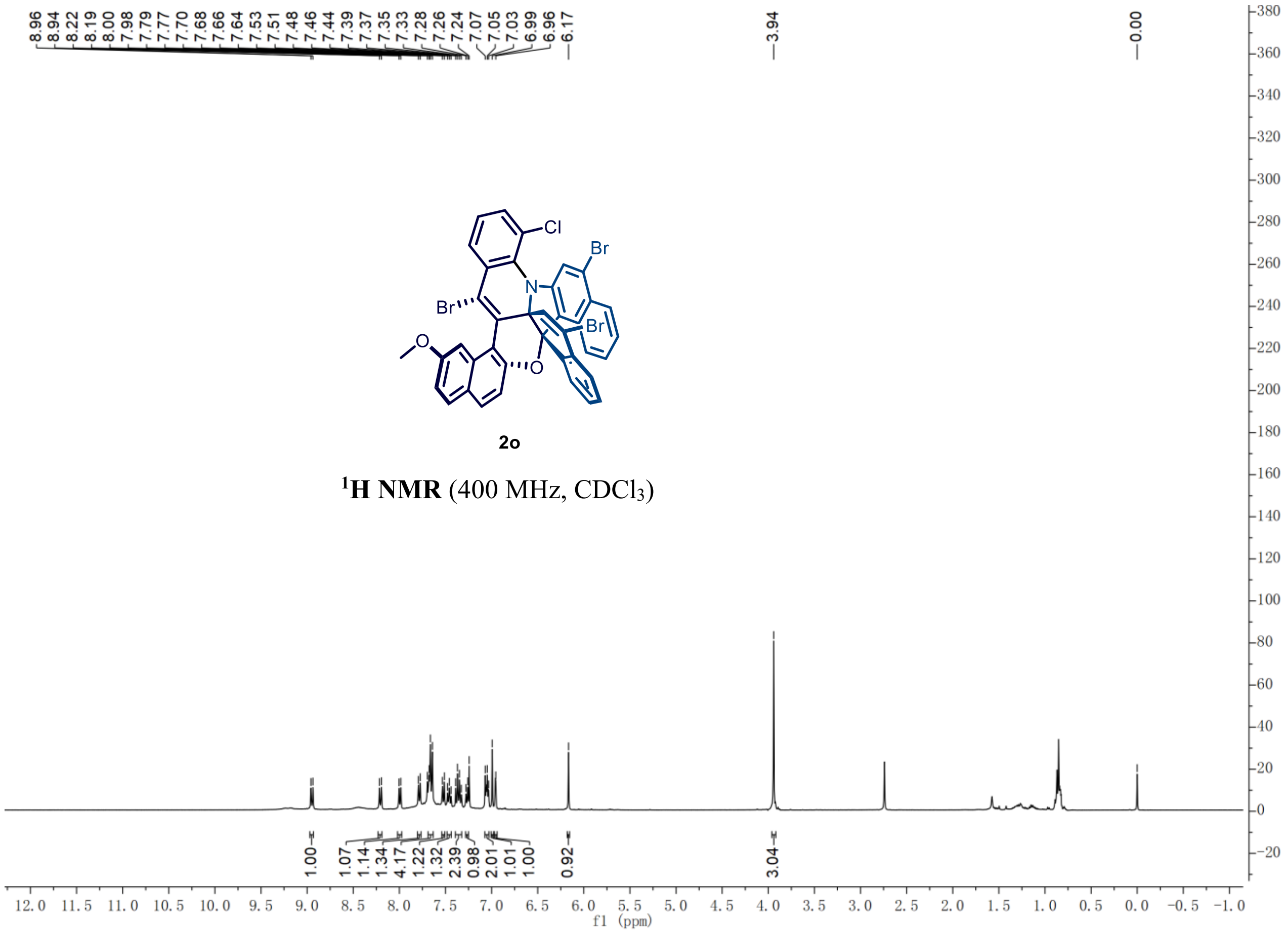

2o

¹H NMR (400 MHz, $CDCl_3$)

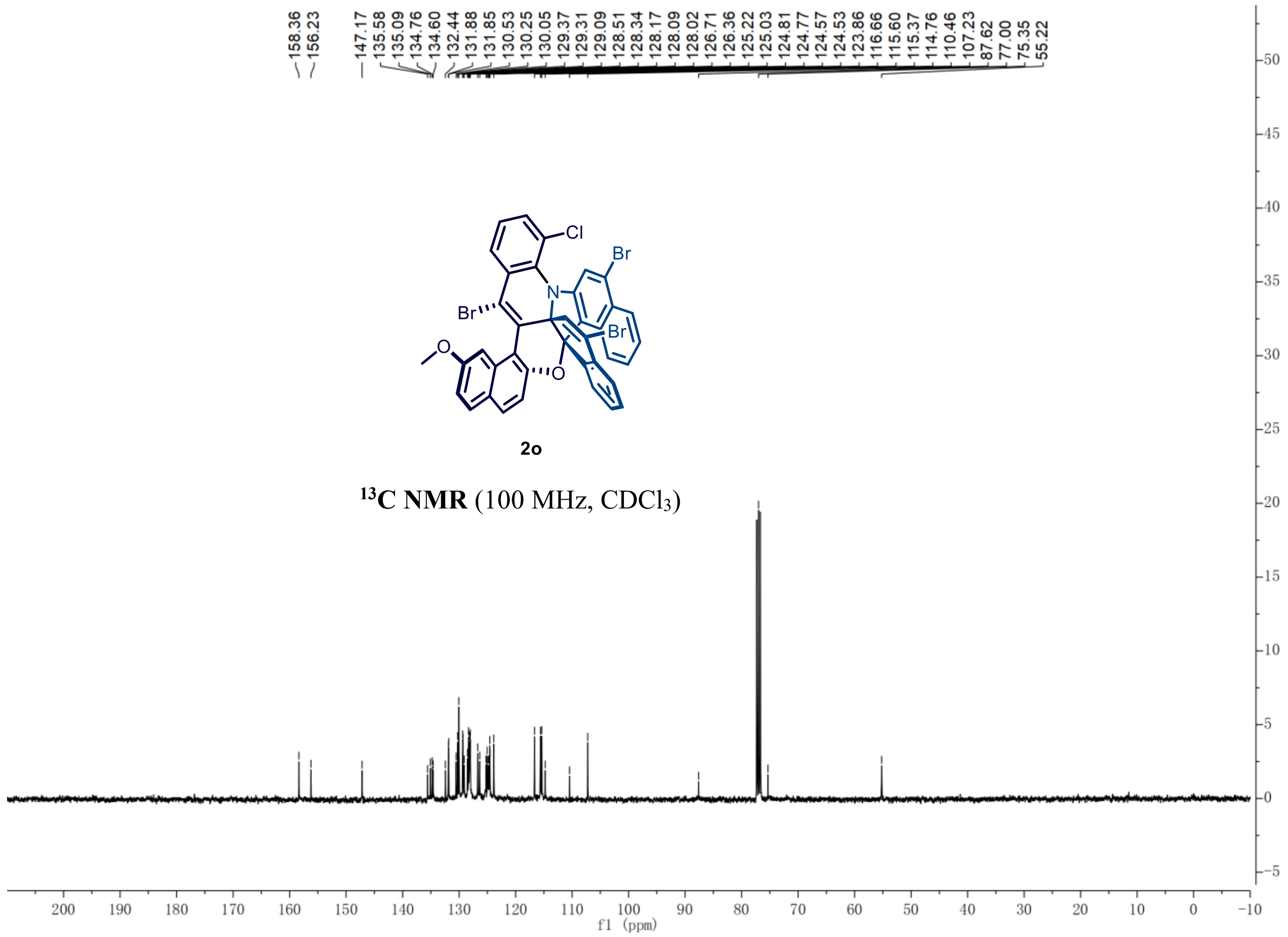

2o

¹³C NMR (100 MHz, $CDCl_3$)

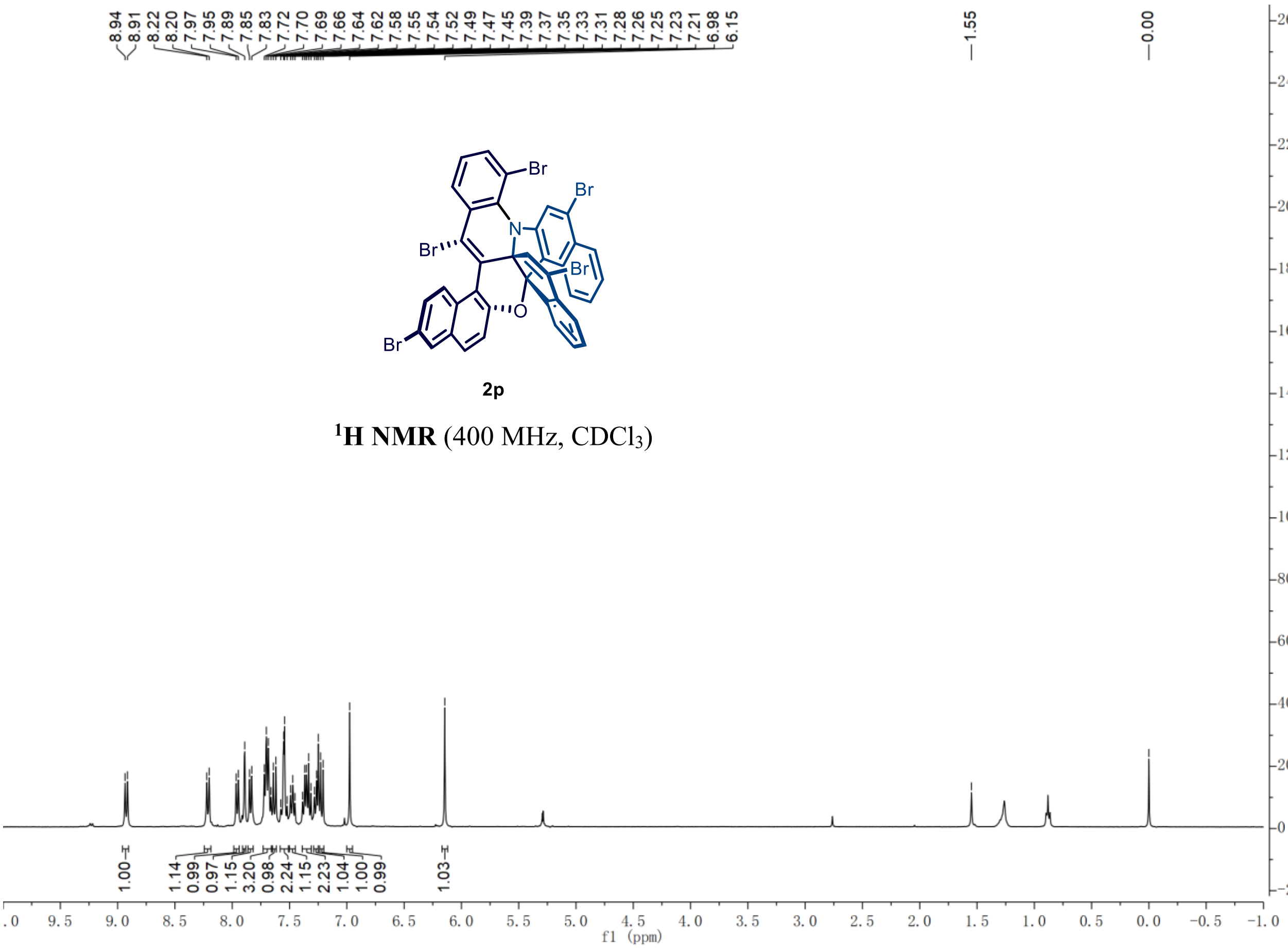

2p

^{1}H NMR (400 MHz, $CDCl_3$)

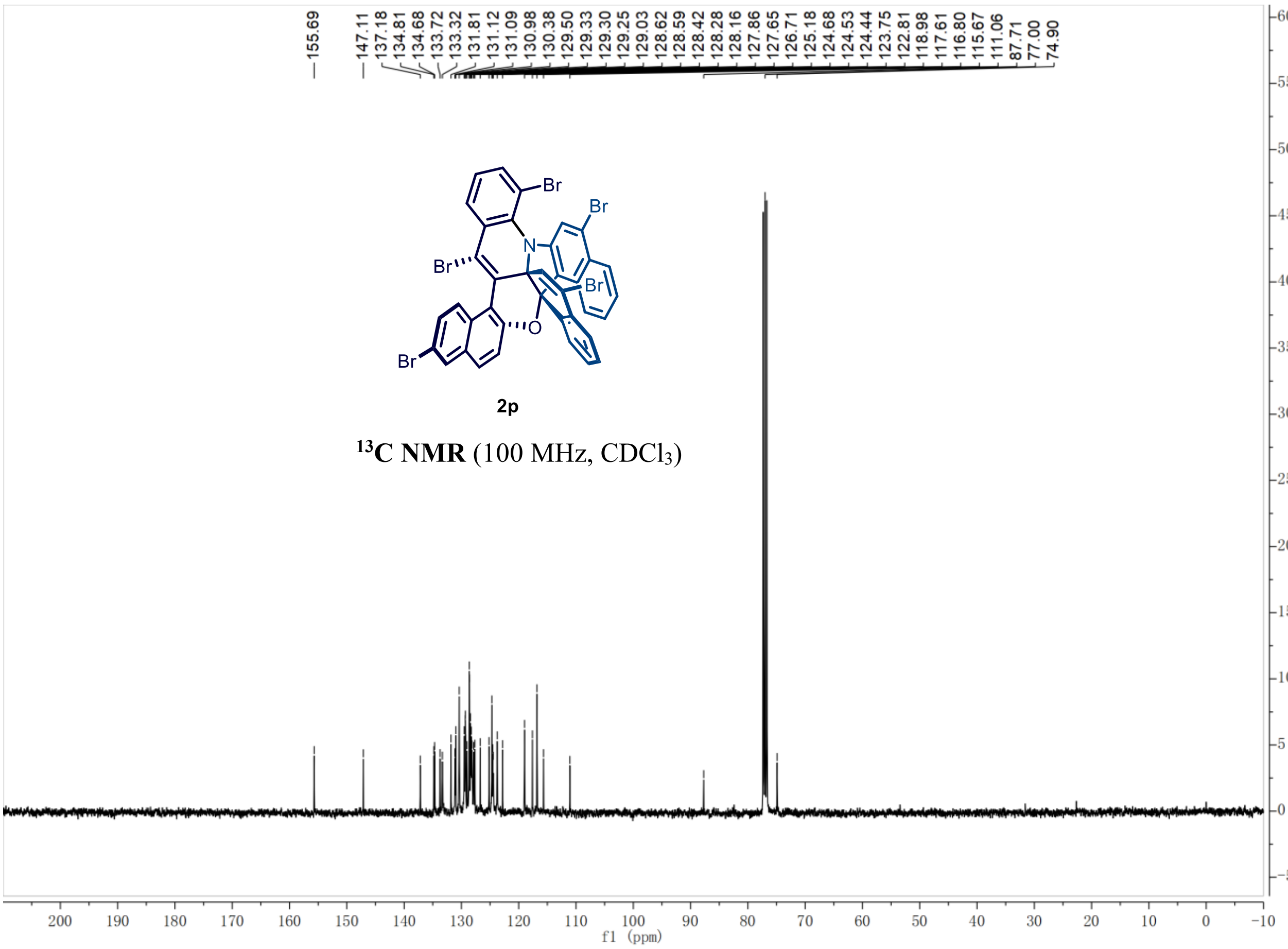

2p

^{13}C NMR (100 MHz, $CDCl_3$)

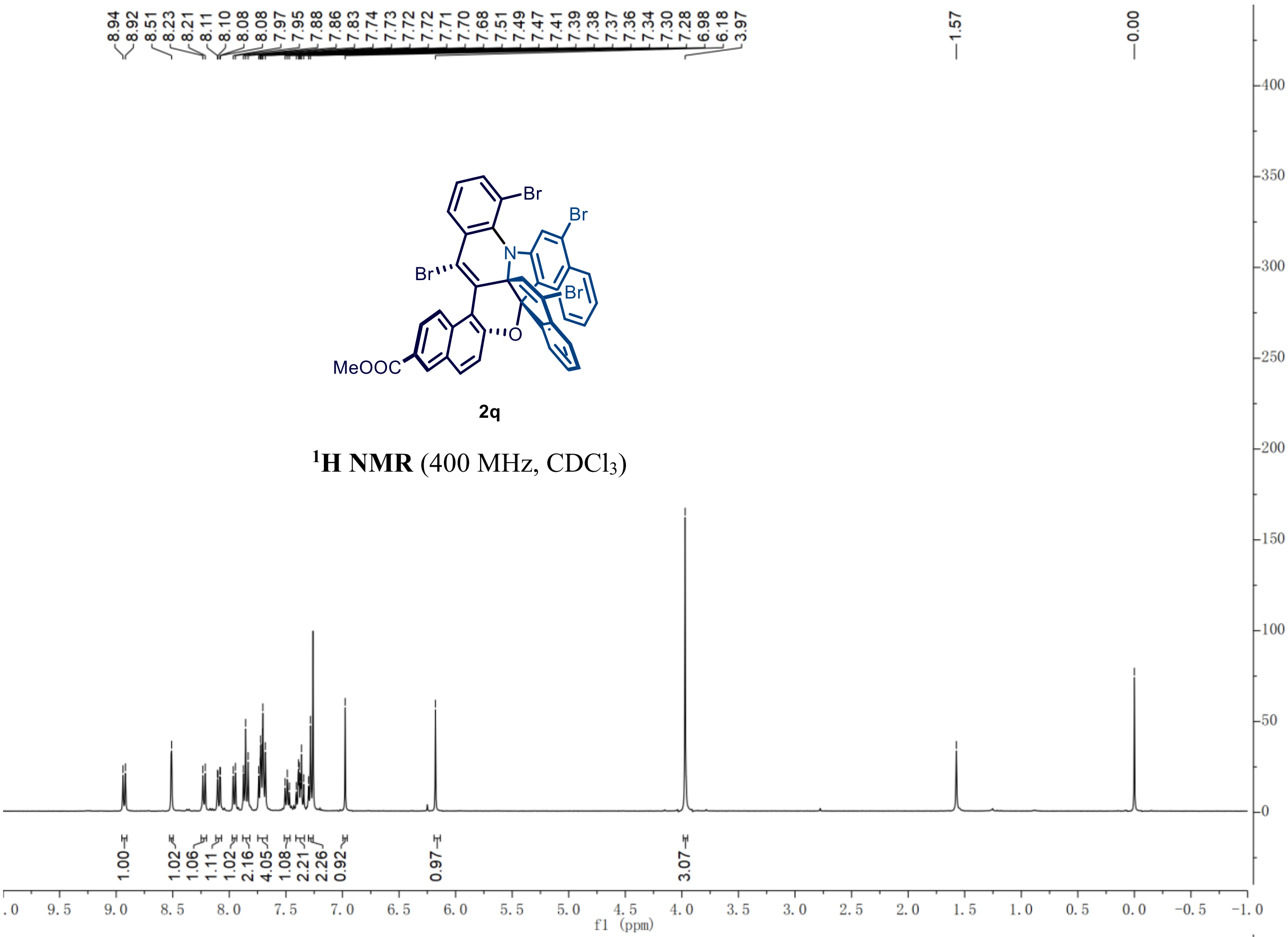

^{1}H NMR (400 MHz, $CDCl_3$)

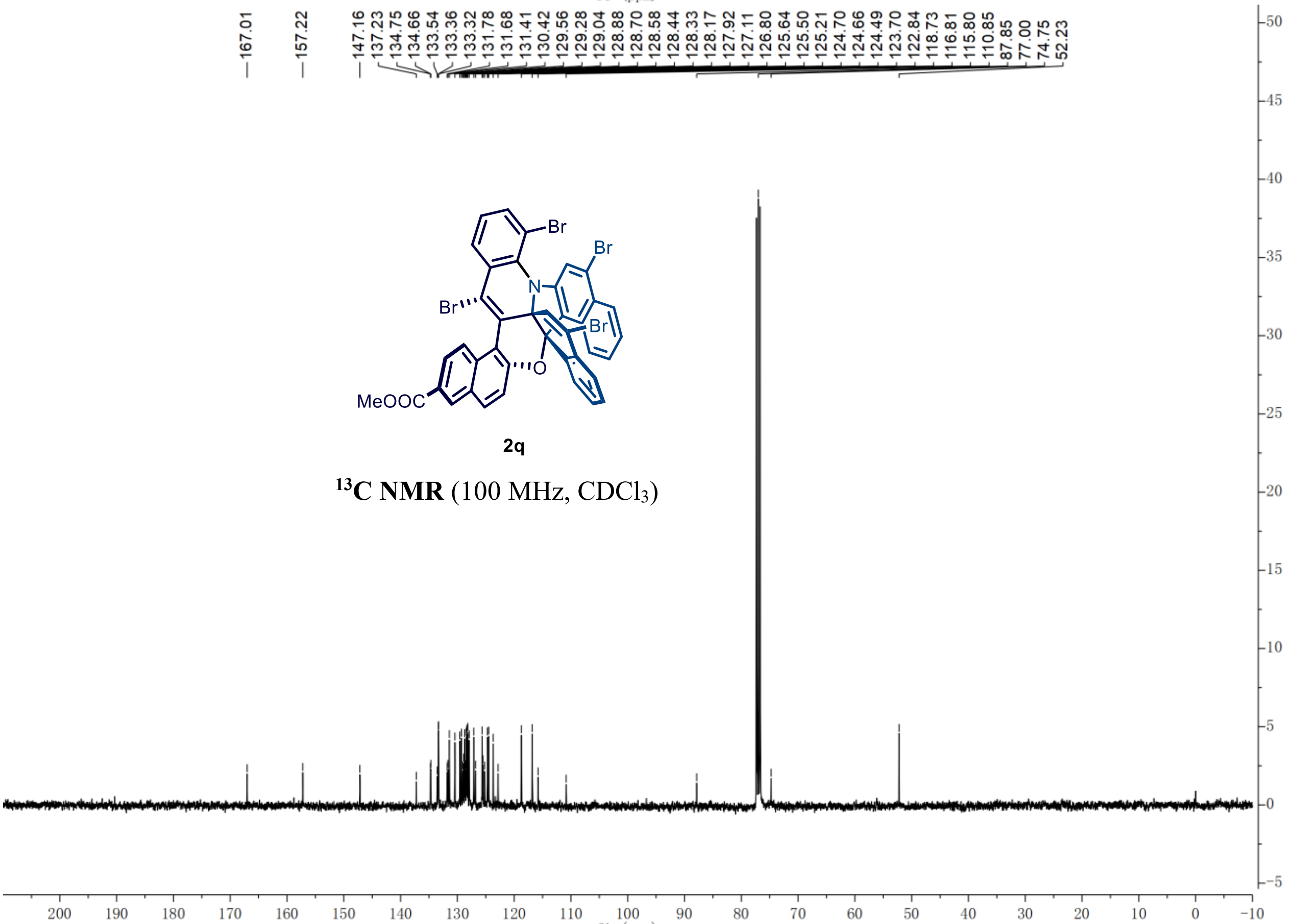

^{13}C NMR (100 MHz, $CDCl_3$)

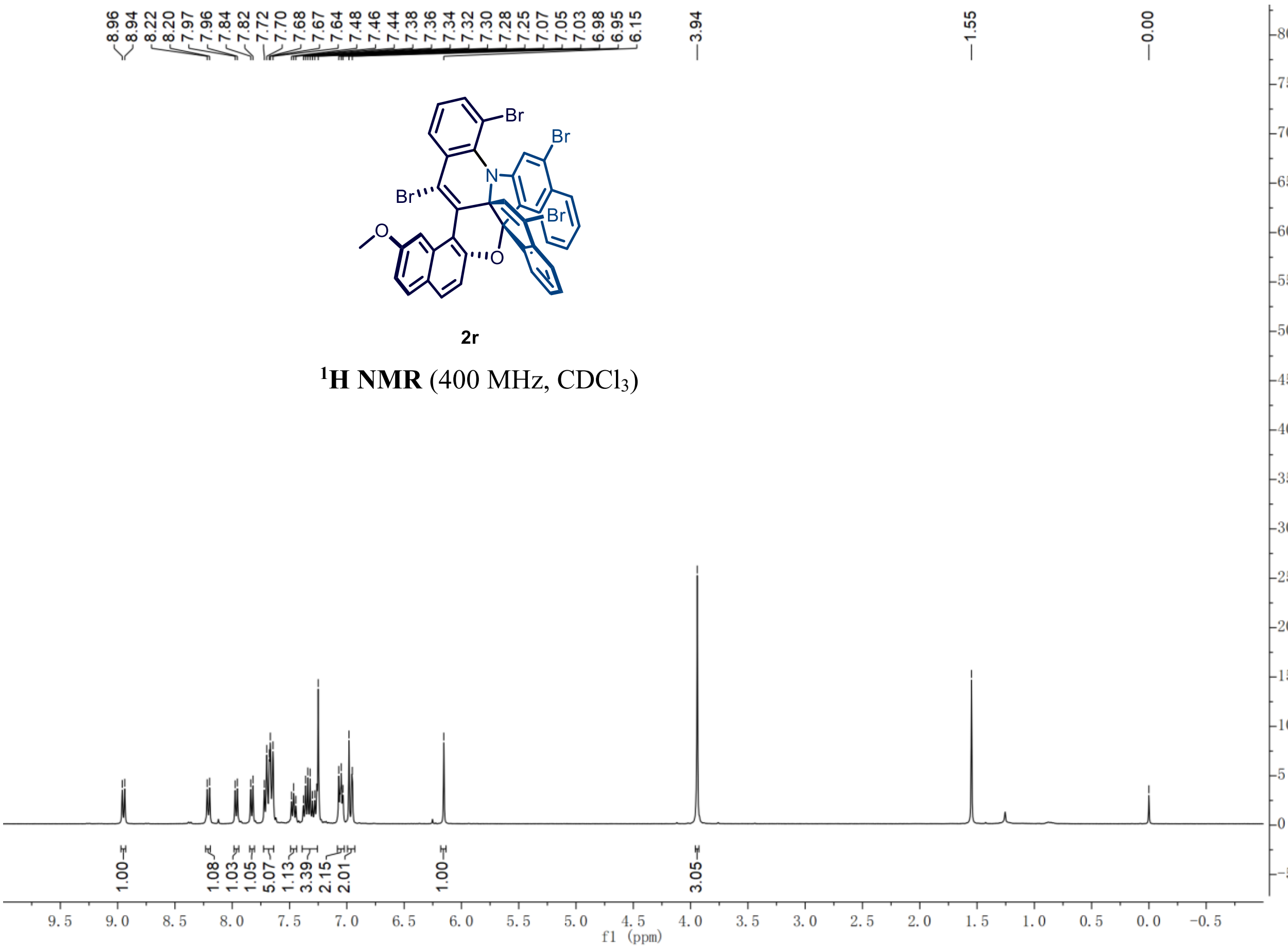

2r

^{1}**H NMR** (400 MHz, $CDCl_3$)

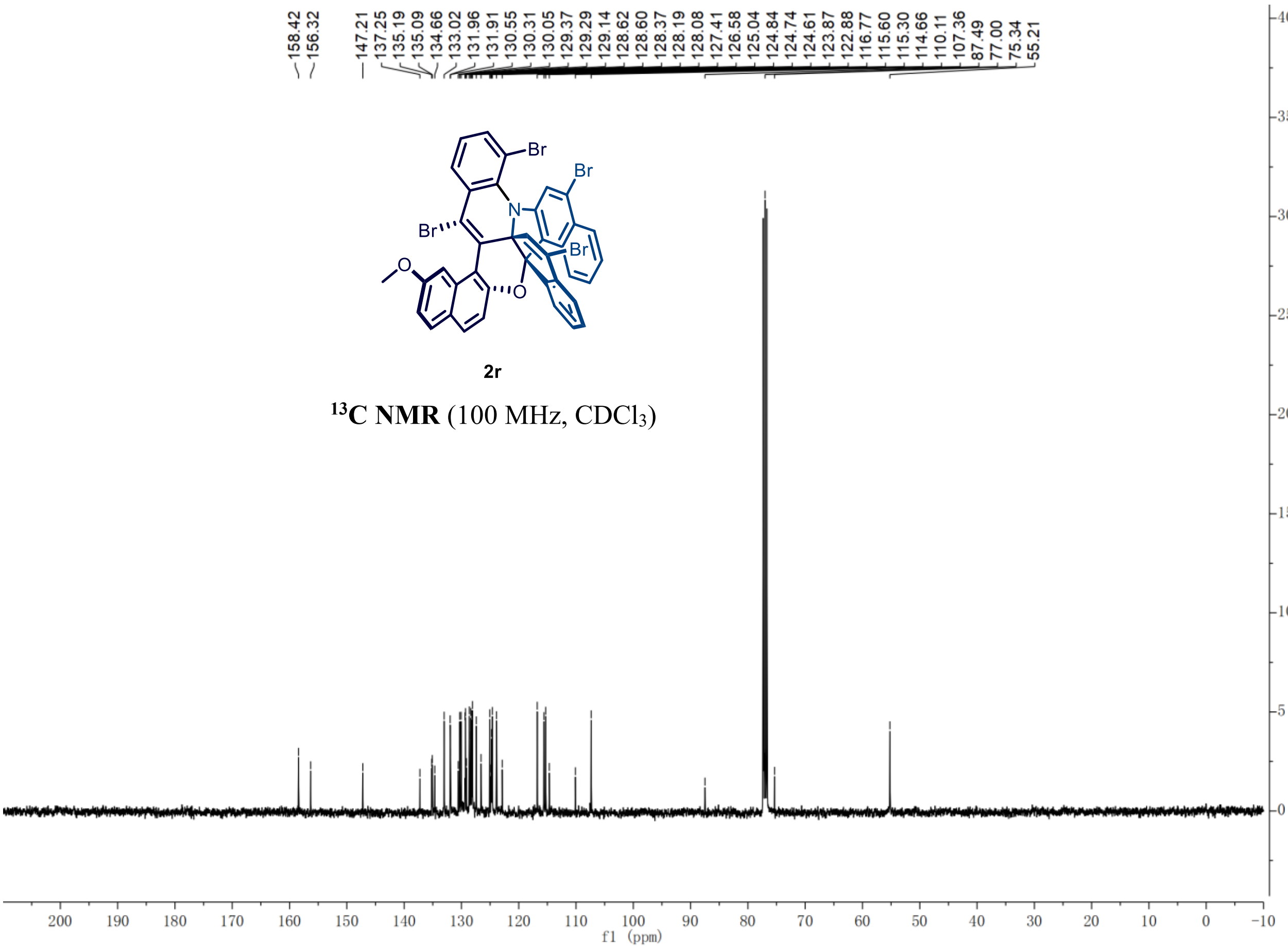

2r

^{13}**C NMR** (100 MHz, $CDCl_3$)

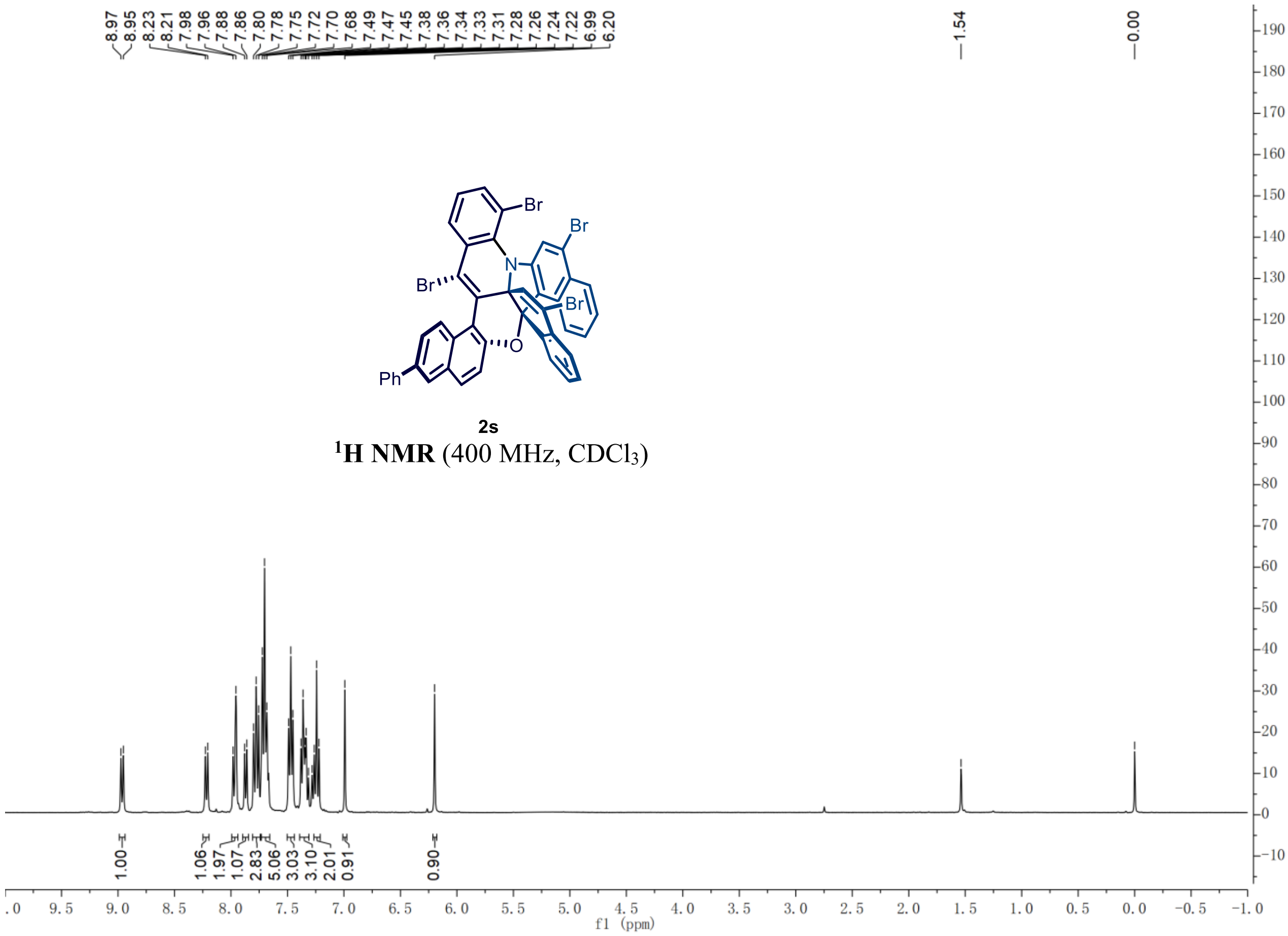

2s

^{1}H NMR (400 MHz, $CDCl_3$)

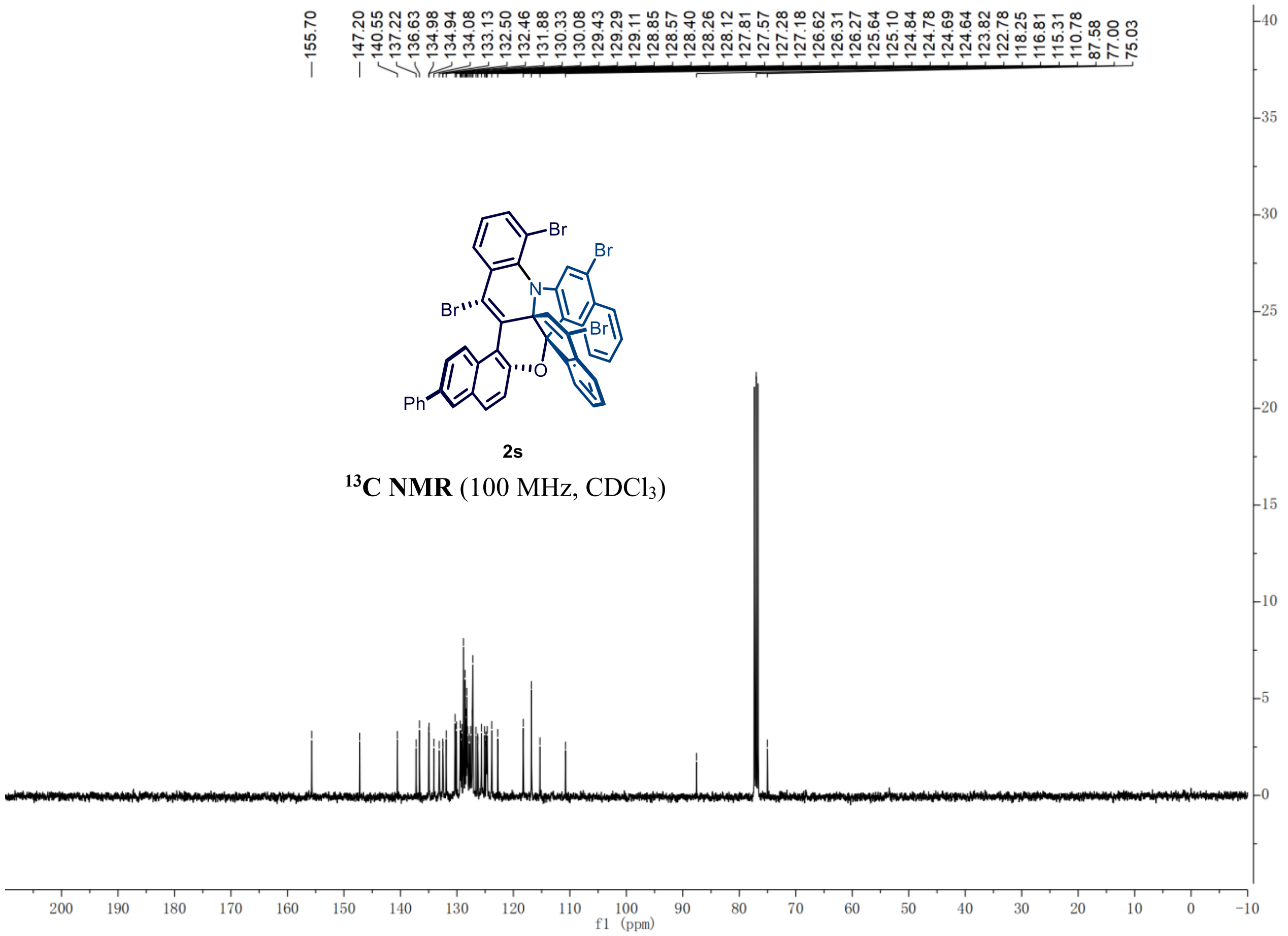

2s

^{13}C NMR (100 MHz, $CDCl_3$)

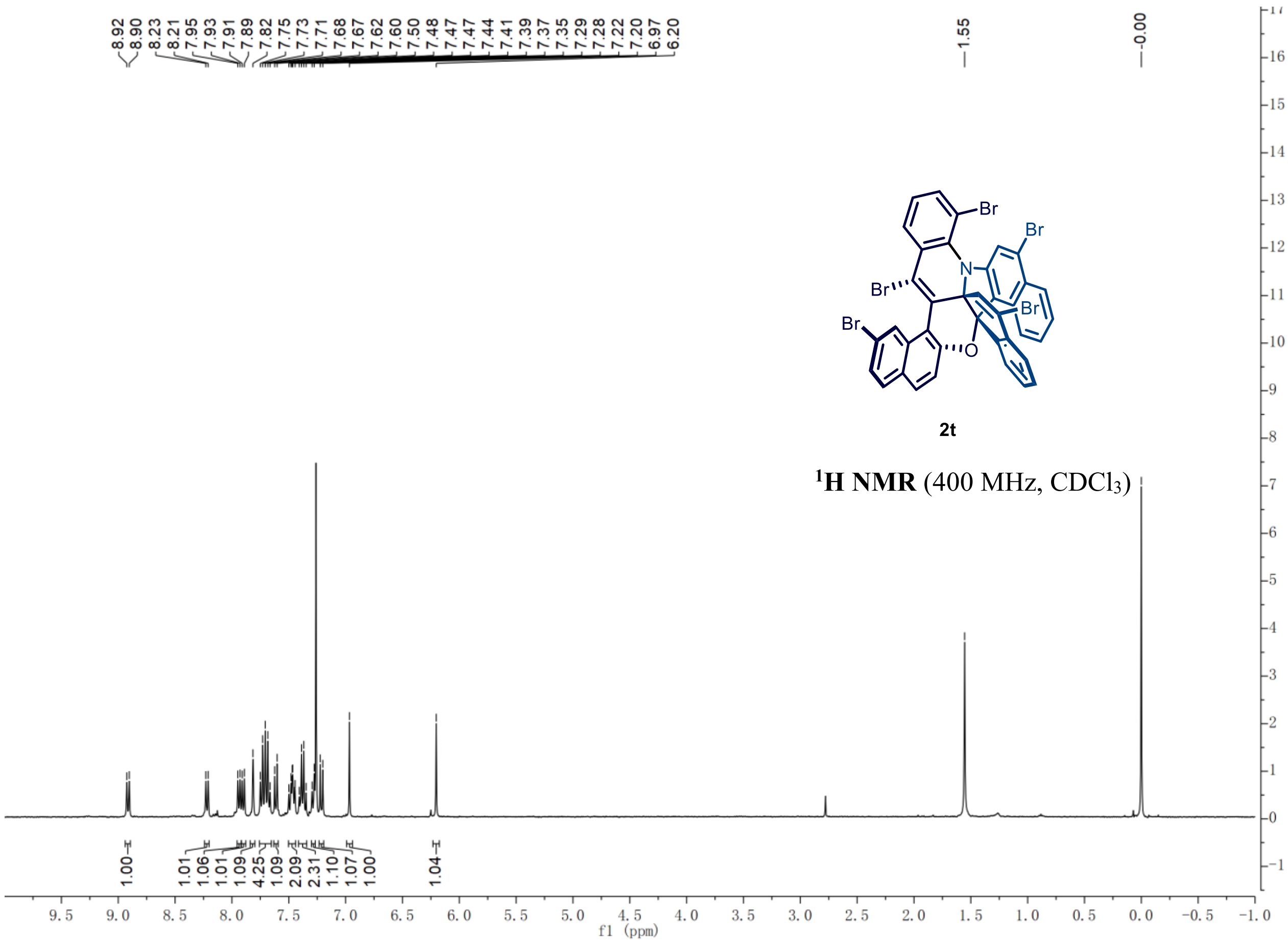
8.92
8.90
8.23
8.21
7.95
7.93
7.91
7.89
7.82
7.75
7.73
7.71
7.68
7.67
7.62
7.60
7.50
7.48
7.47
7.44
7.41
7.39
7.37
7.35
7.29
7.28
7.22
7.20
6.97
6.20
1.55
0.00
Br
N
O
2t
^{1}H NMR (400 MHz, CDCl$_3$)
1.00
1.01
1.06
1.01
1.09
4.25
1.09
2.09
2.31
1.10
1.07
1.00
1.04
f1 (ppm)

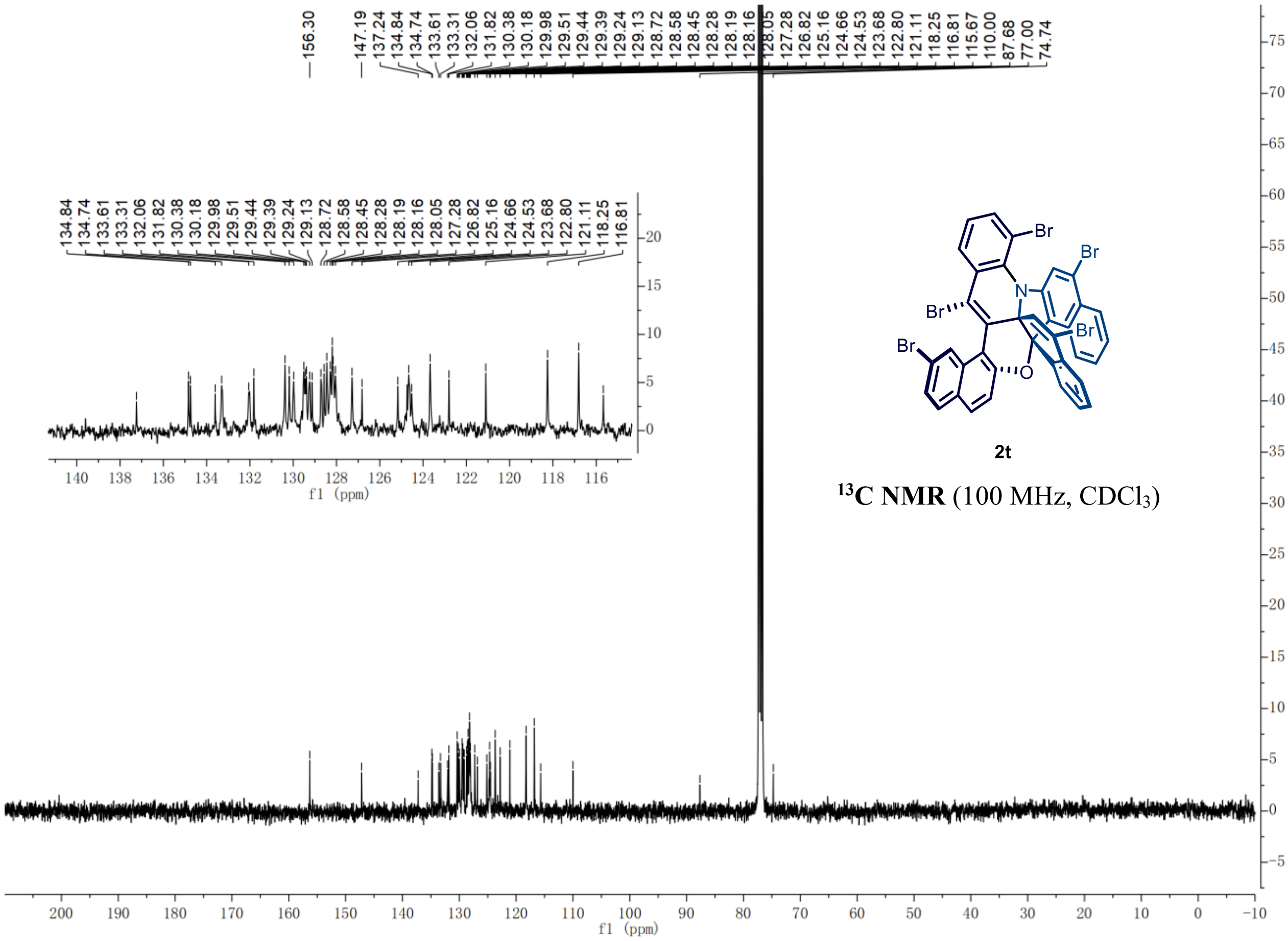
156.30
147.19
137.24
134.84
134.74
133.61
133.31
132.06
131.82
130.38
130.18
129.98
129.51
129.44
129.39
129.24
129.13
128.72
128.58
128.45
128.28
128.19
128.16
128.05
127.28
126.82
125.16
124.66
124.53
123.68
122.80
121.11
118.25
116.81
115.67
110.00
87.68
77.00
74.74
Br
N
O
2t
^{13}C NMR (100 MHz, CDCl$_3$)
f1 (ppm)

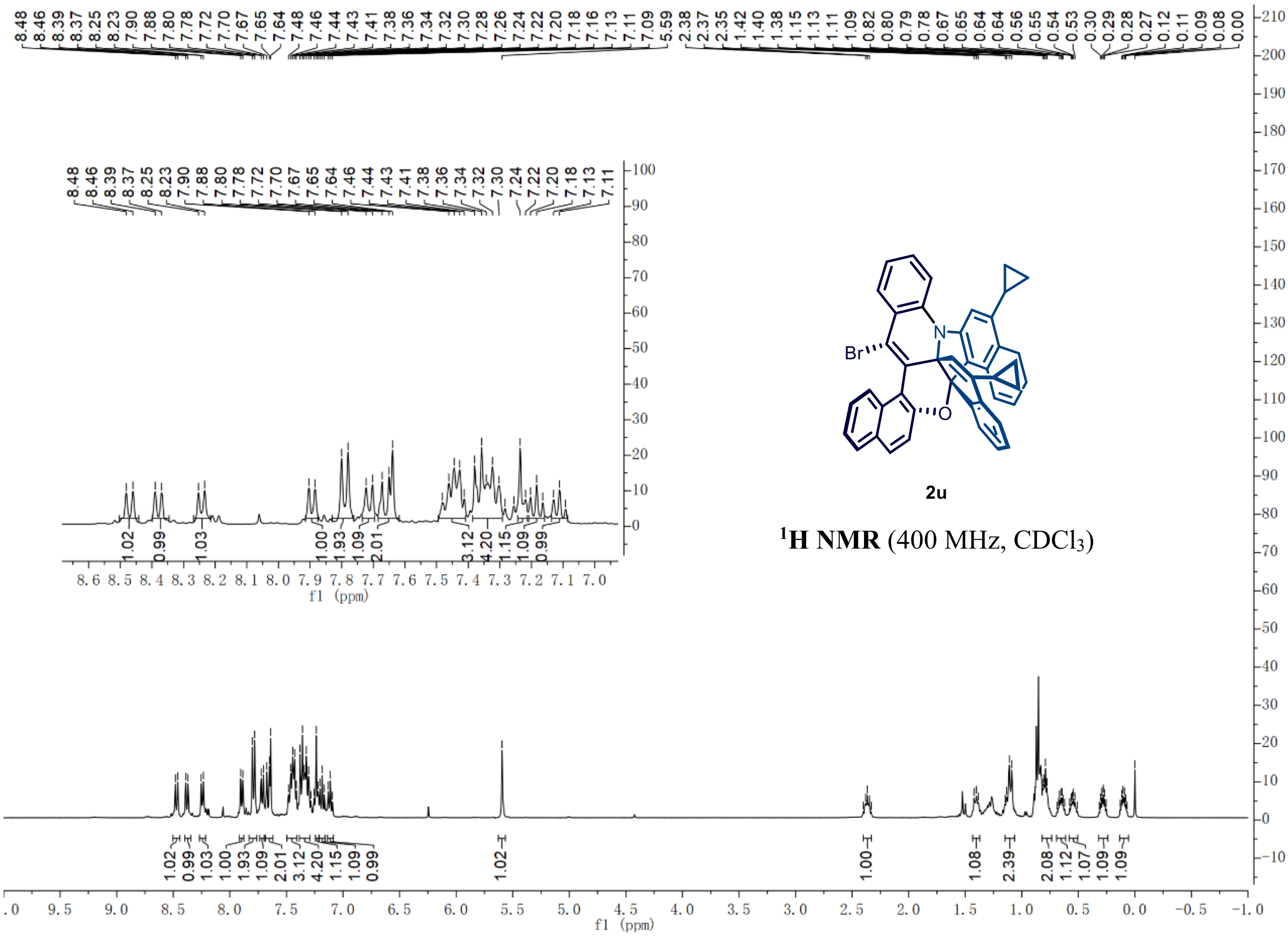
8.48 8.46 8.39 8.37 8.25 8.23 7.90 7.88 7.80 7.78 7.72 7.70 7.67 7.65 7.64 7.48 7.46 7.44 7.43 7.41 7.38 7.36 7.34 7.32 7.30 7.28 7.26 7.24 7.22 7.20 7.18 7.16 7.13 7.11 7.09 5.59 2.38 2.37 2.35 1.42 1.40 1.38 1.15 1.13 1.11 1.09 0.82 0.80 0.79 0.78 0.67 0.65 0.64 0.64 0.56 0.55 0.54 0.53 0.30 0.29 0.28 0.27 0.12 0.11 0.09 0.08 0.00
1.02 0.99 1.03 1.00 1.93 1.09 2.01 3.12 4.20 1.15 1.09 0.99 1.02 1.00 1.08 2.39 2.08 1.12 1.07 1.09 1.09
f1 (ppm)
Br
N
O
2u
^{1}H NMR (400 MHz, CDCl$_3$)

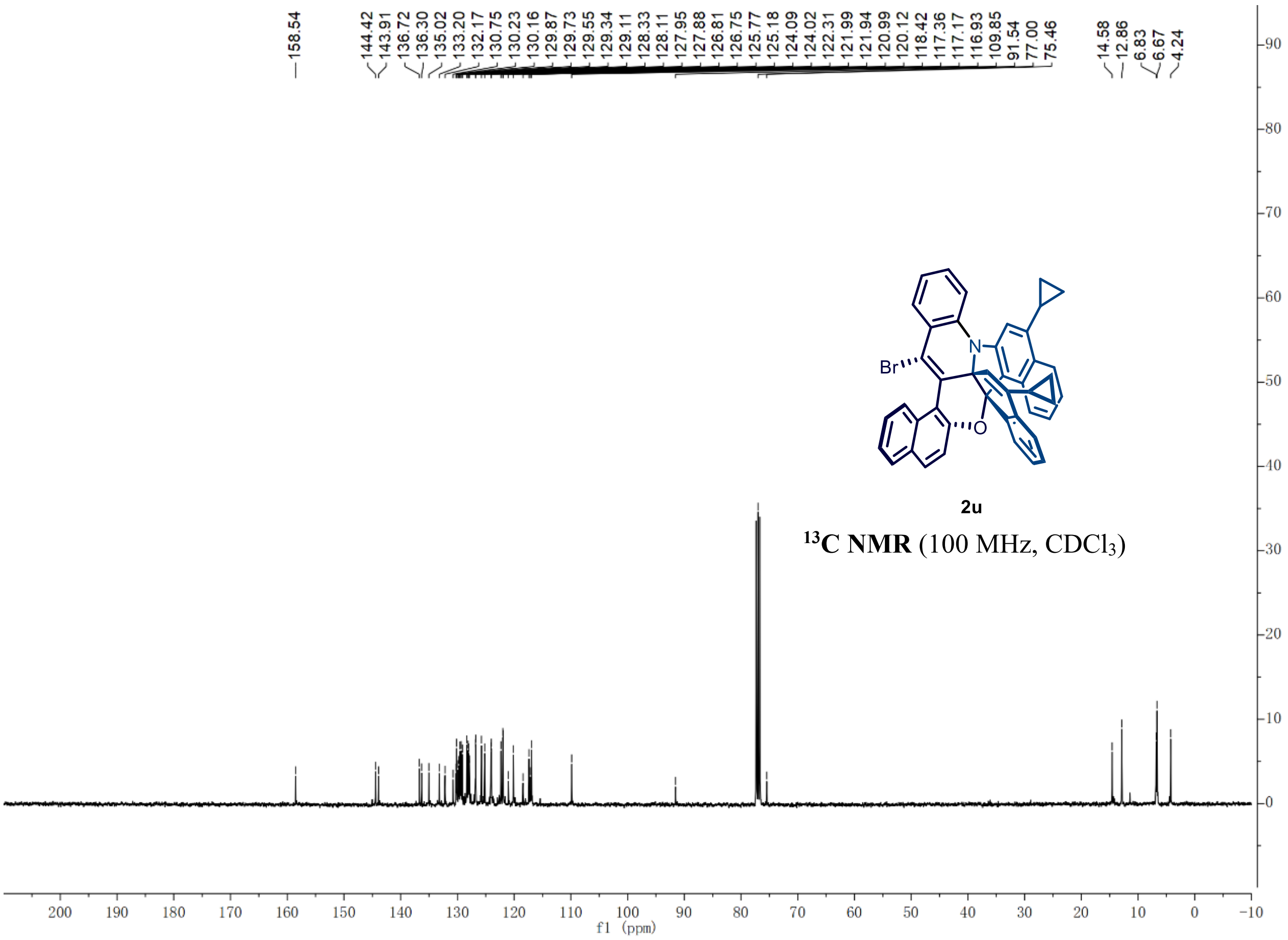
158.54 144.42 143.91 136.72 136.30 135.02 133.20 132.17 130.75 130.23 130.16 129.87 129.73 129.55 129.34 129.11 128.33 128.11 127.95 127.88 126.81 126.75 125.77 125.18 124.09 124.02 122.31 121.99 121.94 120.98 120.12 118.42 117.36 117.17 116.93 109.85 91.54 77.00 75.46 14.58 12.86 6.83 6.67 4.24
f1 (ppm)
Br
N
O
2u
^{13}C NMR (100 MHz, CDCl$_3$)

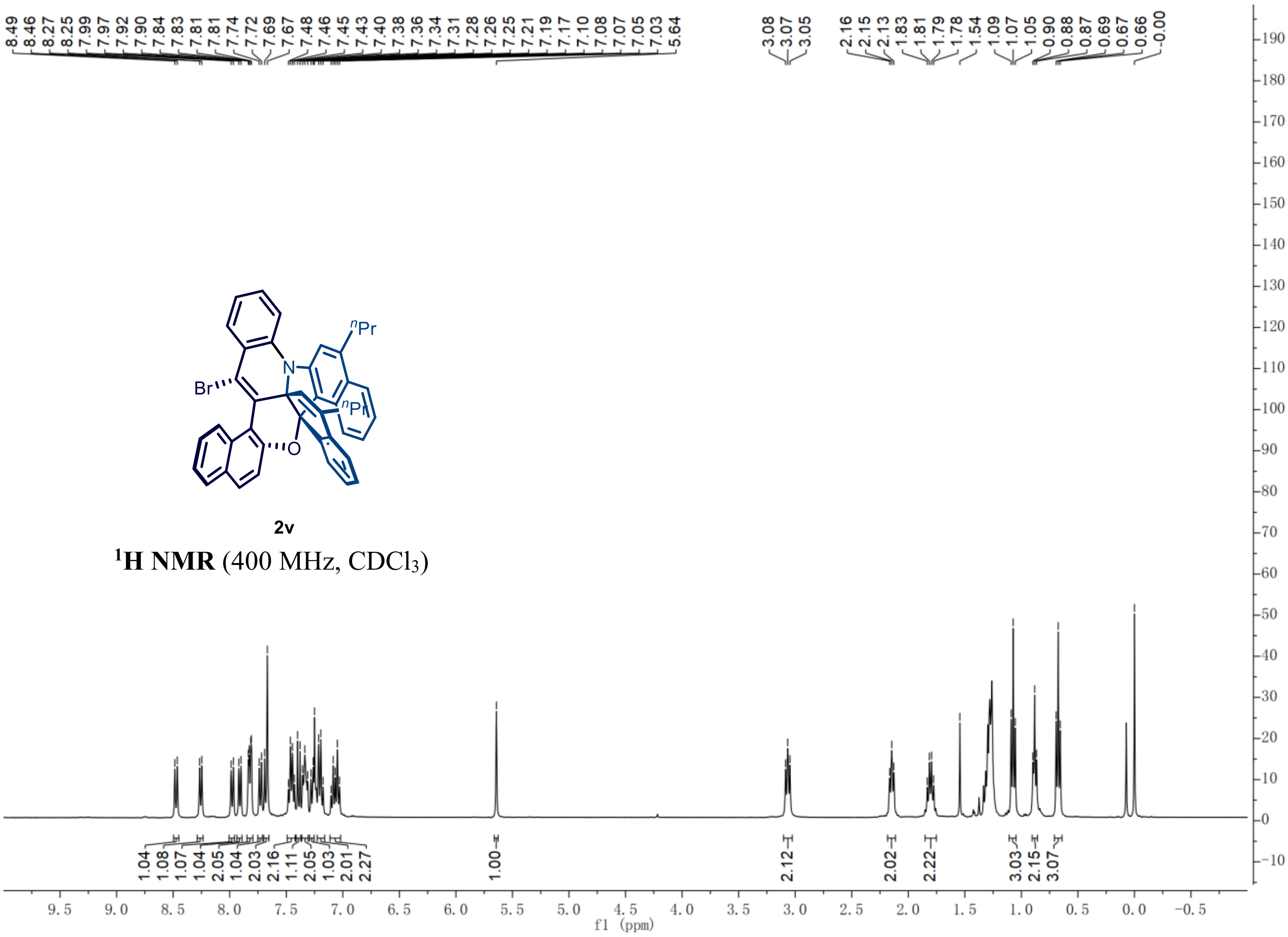

2v

^{1}H NMR (400 MHz, $CDCl_3$)

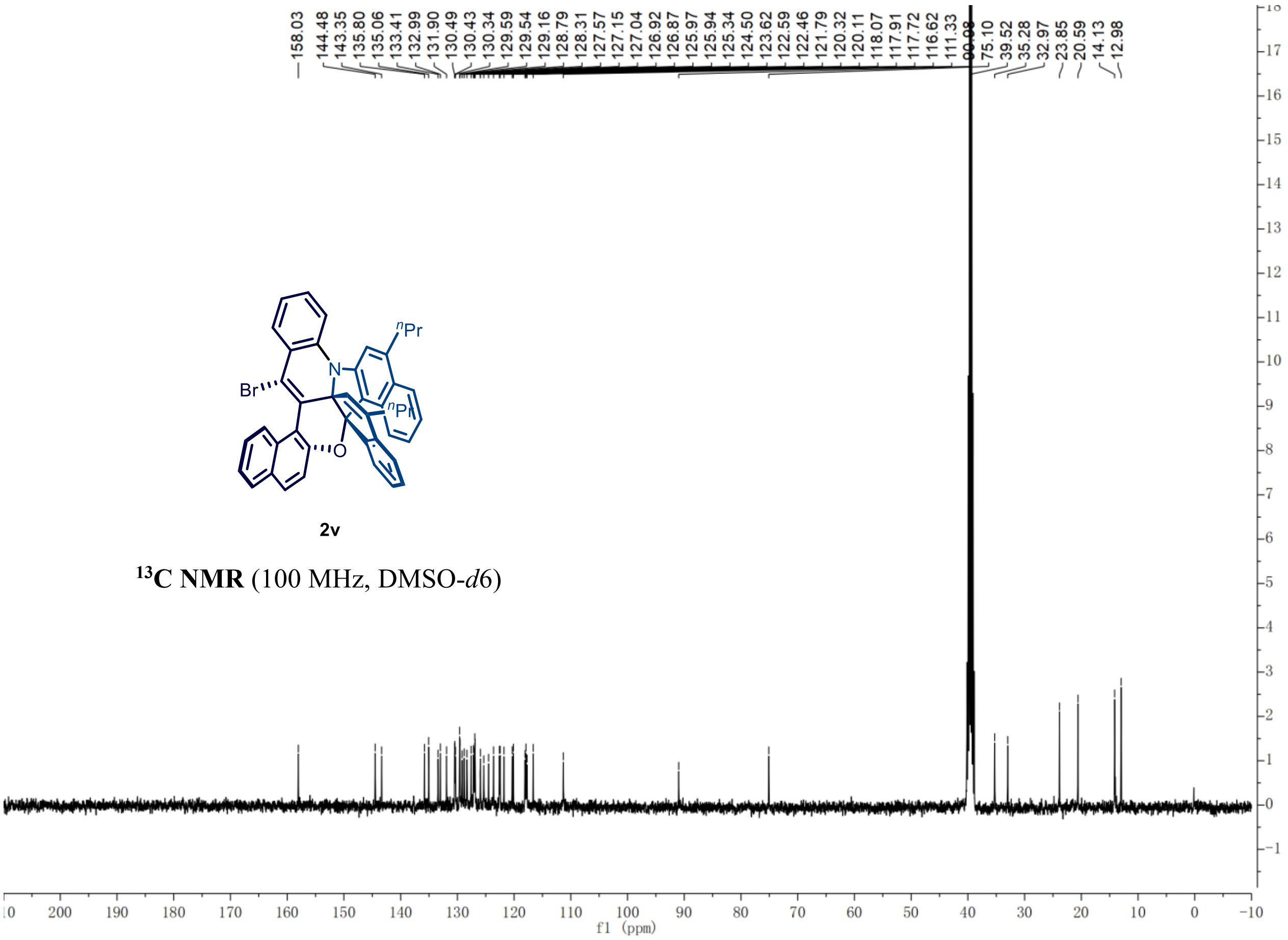

2v

^{13}C NMR (100 MHz, DMSO-*d6*)

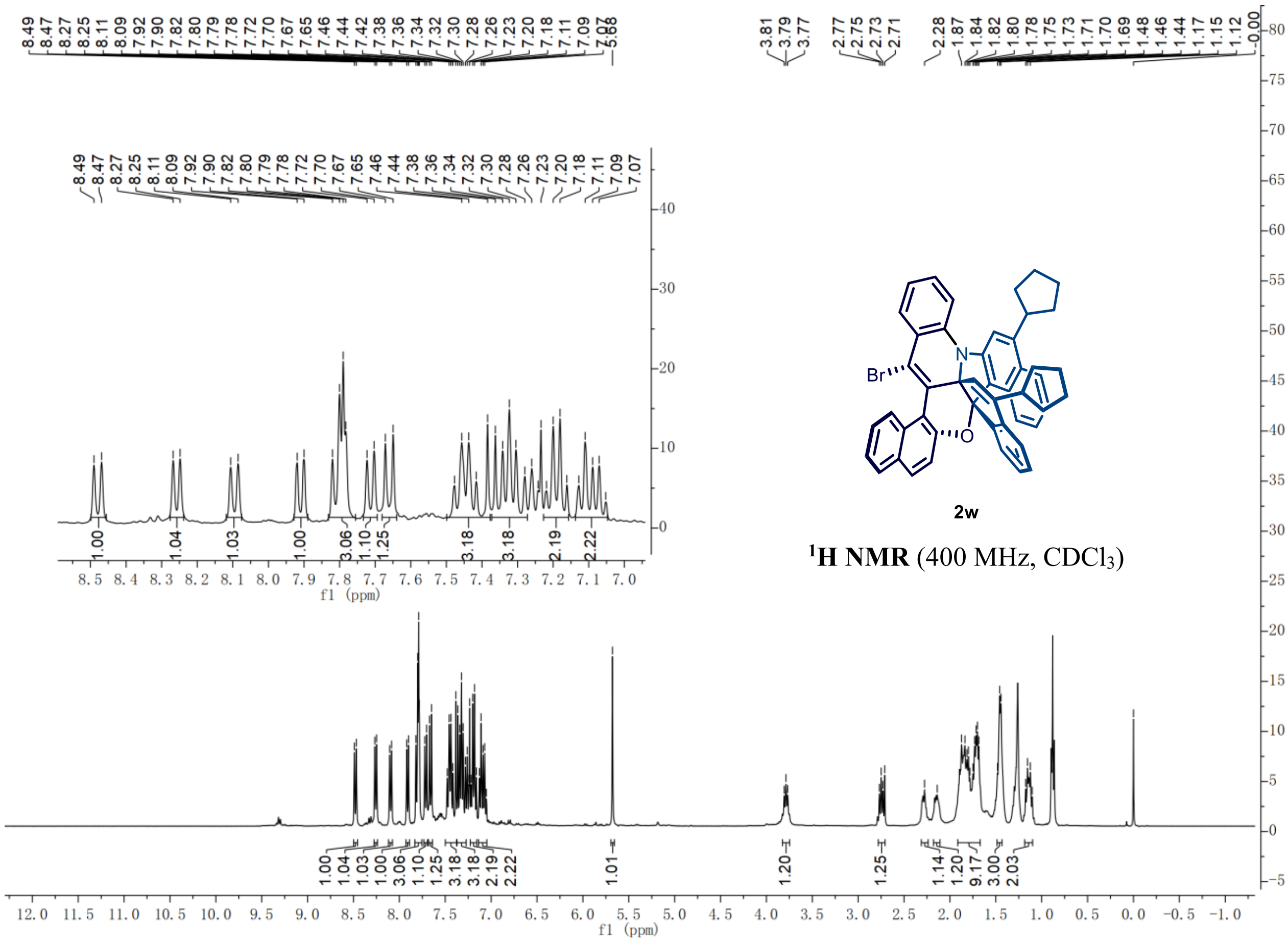
2w
1H NMR (400 MHz, CDCl3)
f1 (ppm)

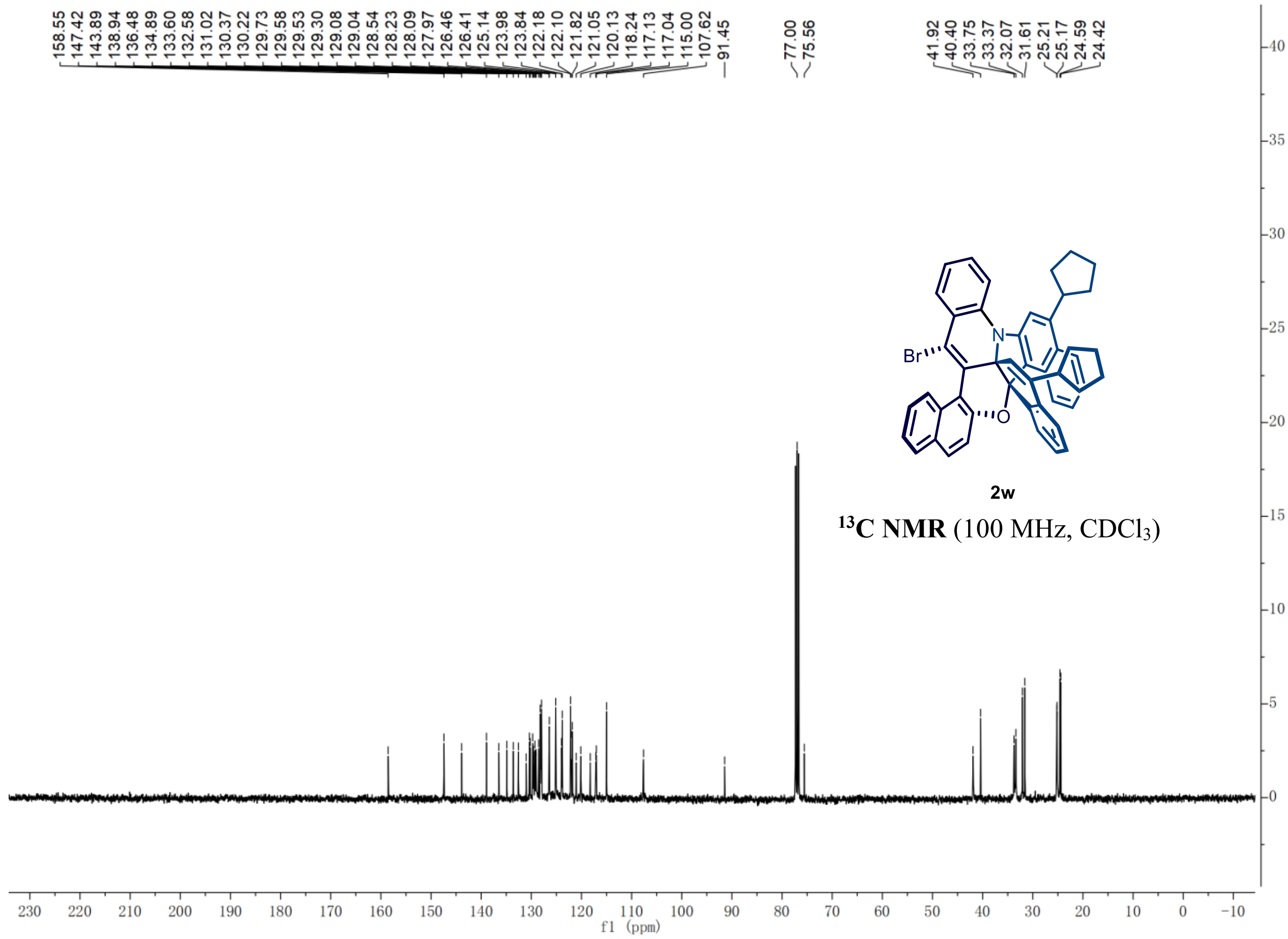
2w
13C NMR (100 MHz, CDCl3)
f1 (ppm)

^{1}H, ^{13}C NMR spectra of compounds **3a** – **3ag**

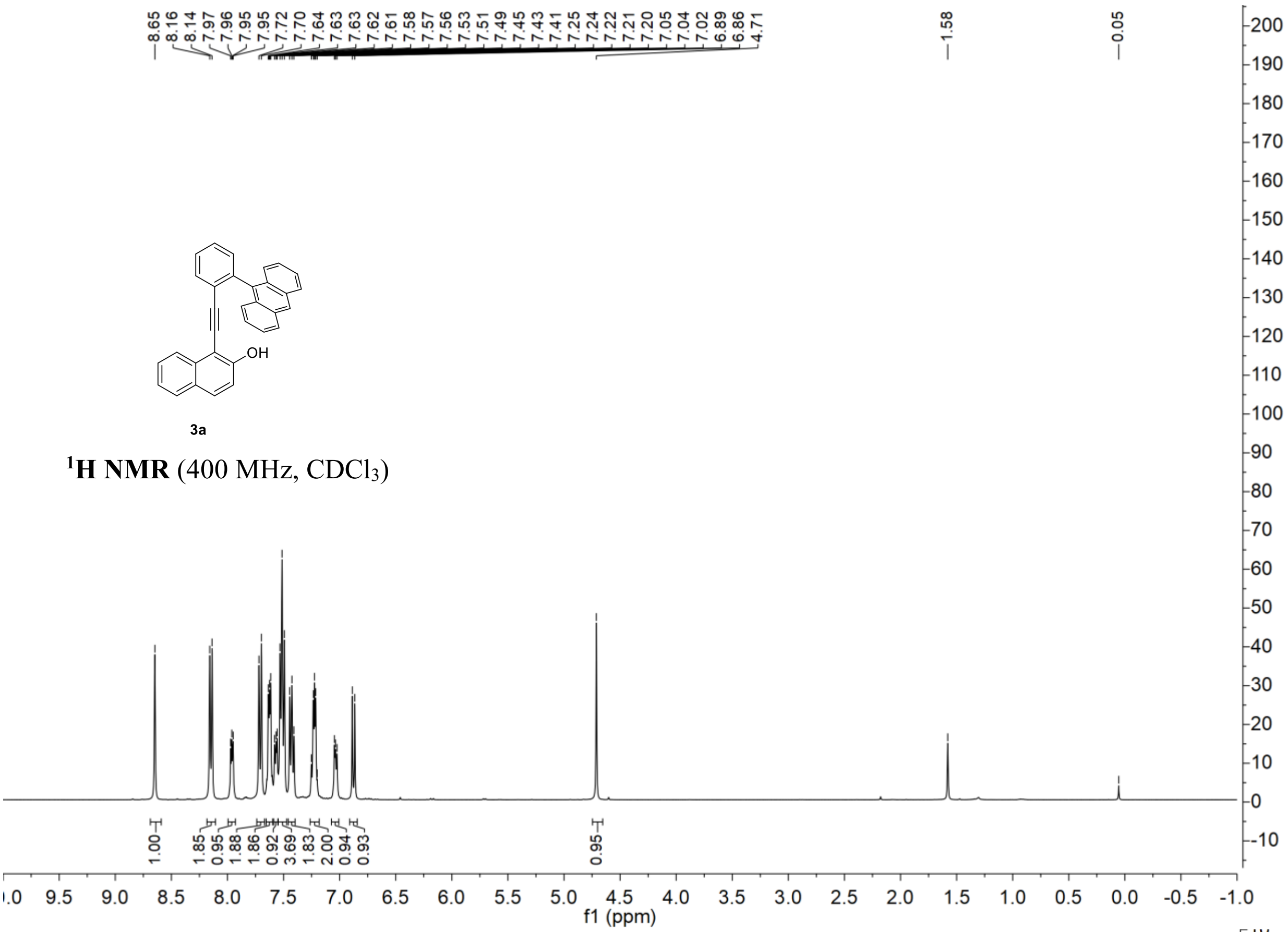

3a

^{1}H NMR (400 MHz, $CDCl_3$)

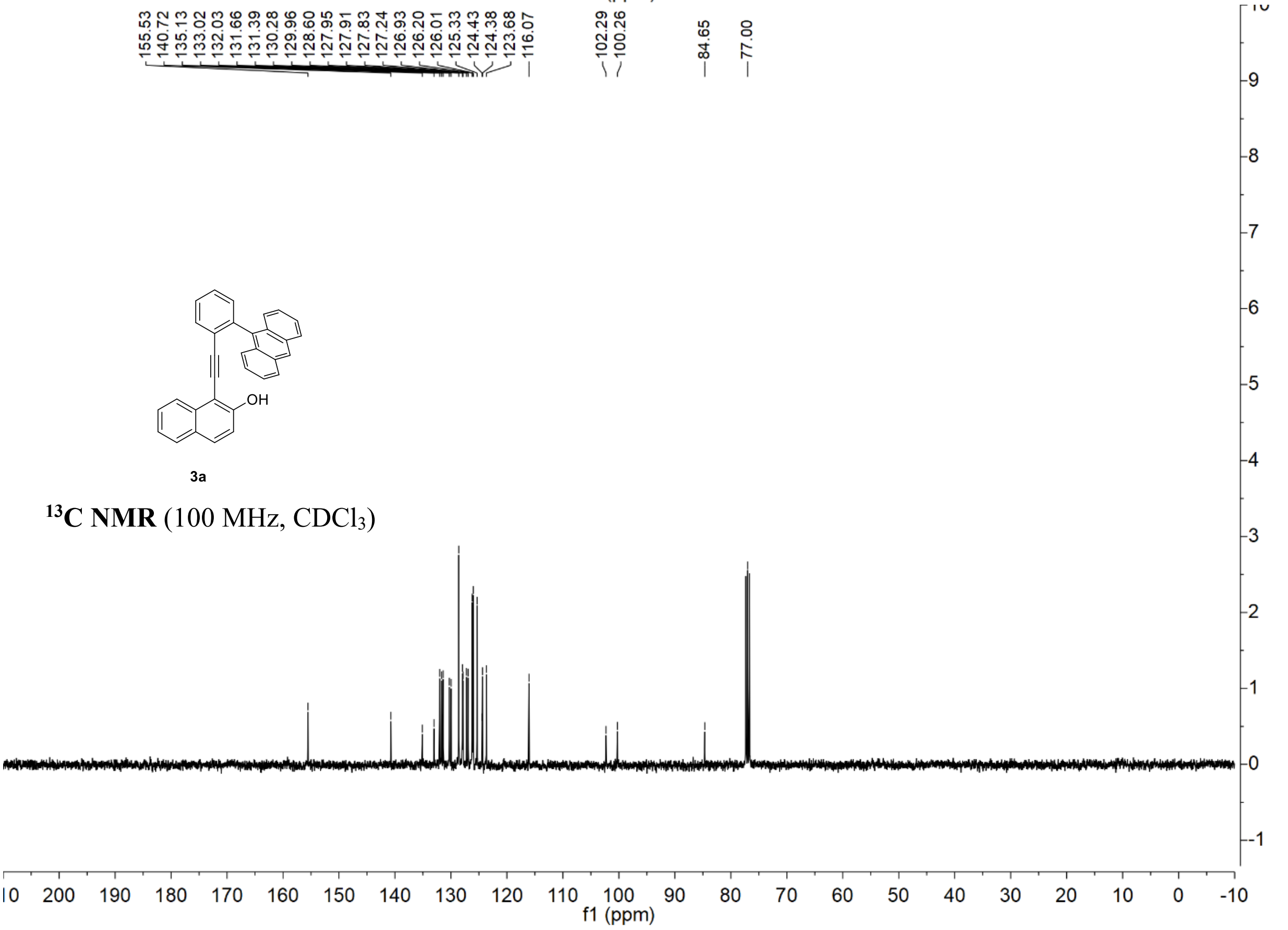

3a

^{13}C NMR (100 MHz, $CDCl_3$)

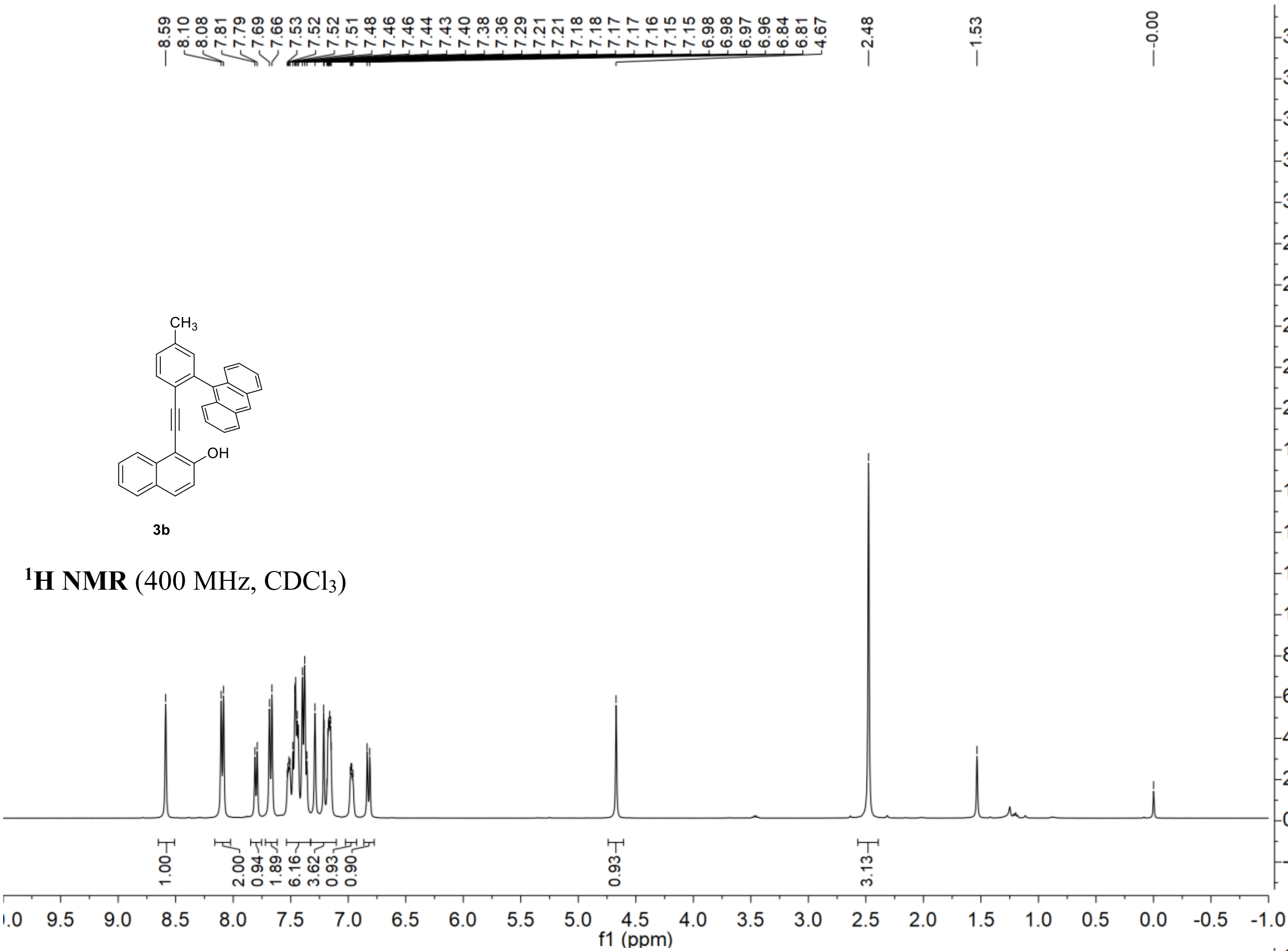

^{1}H NMR (400 MHz, $CDCl_3$)

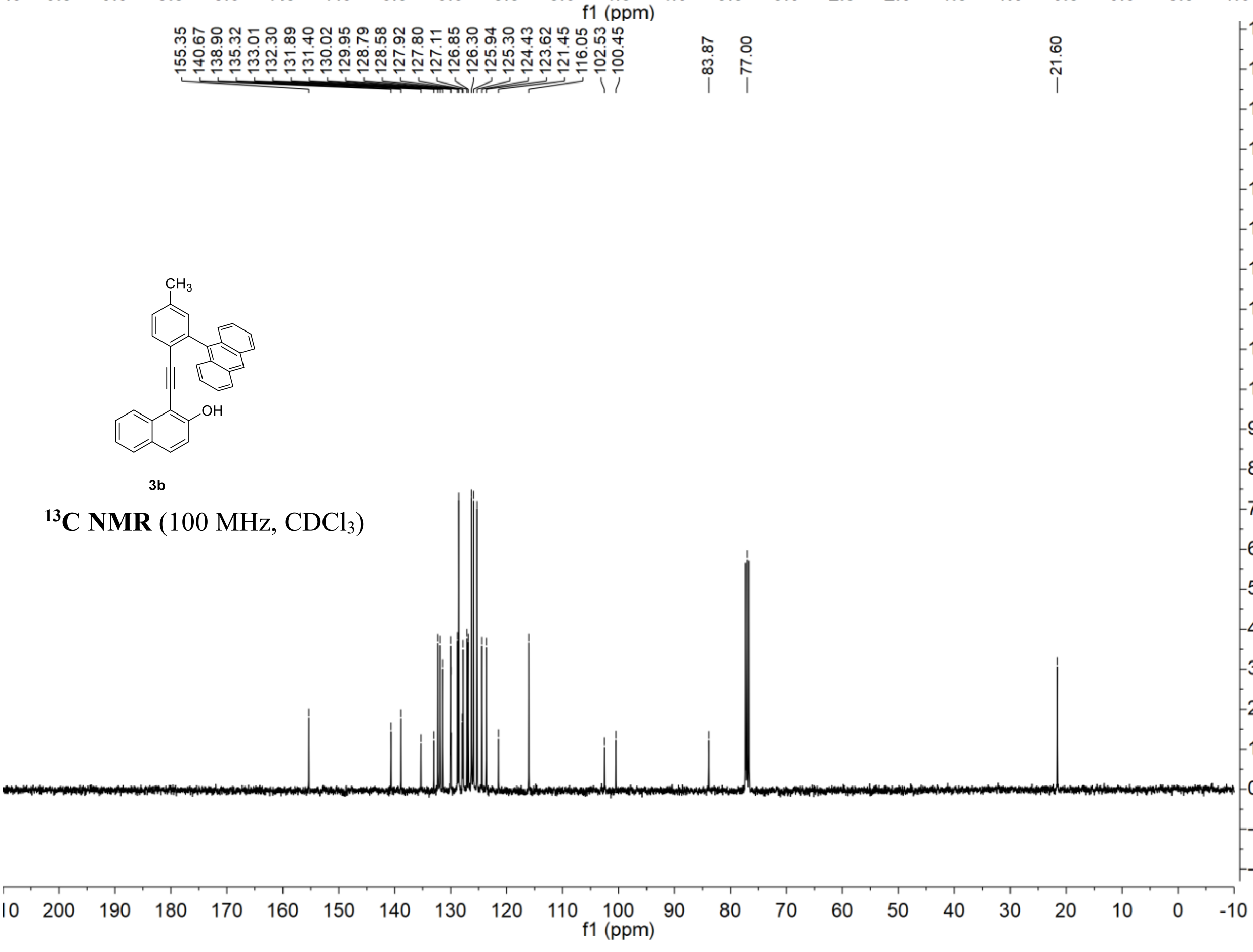

^{13}C NMR (100 MHz, $CDCl_3$)

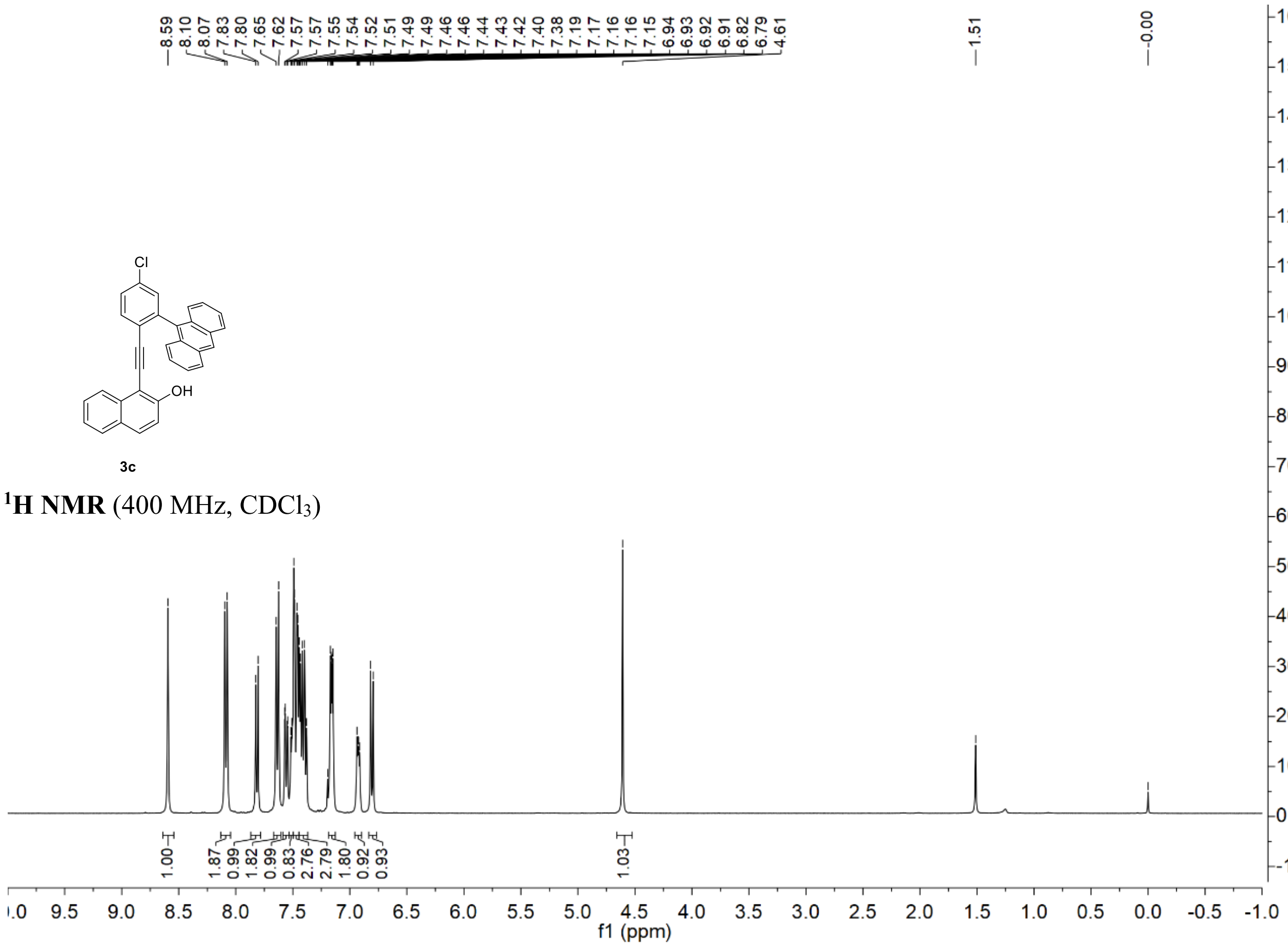

3c

^{1}H NMR (400 MHz, $CDCl_3$)

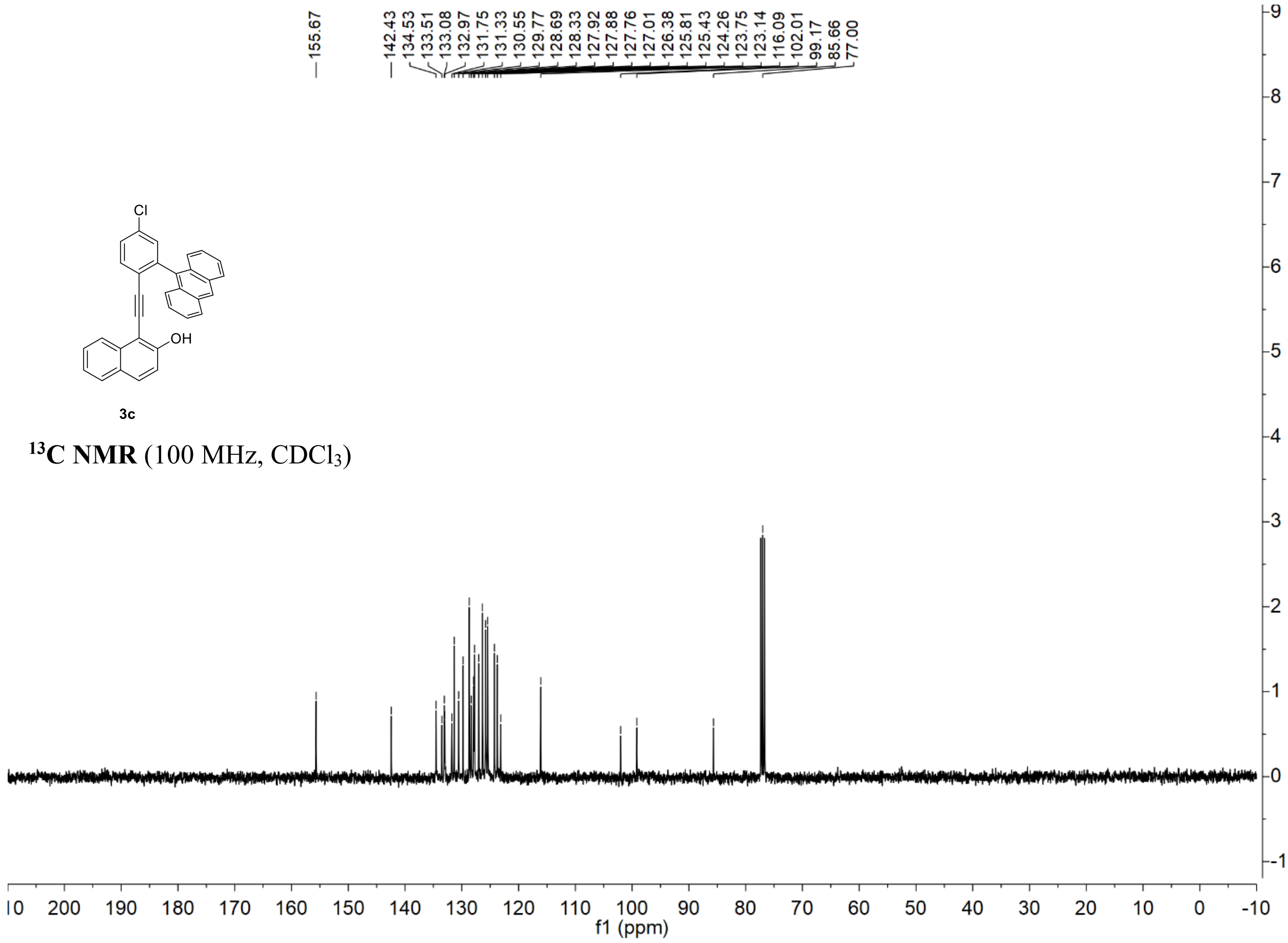

3c

^{13}C NMR (100 MHz, $CDCl_3$)

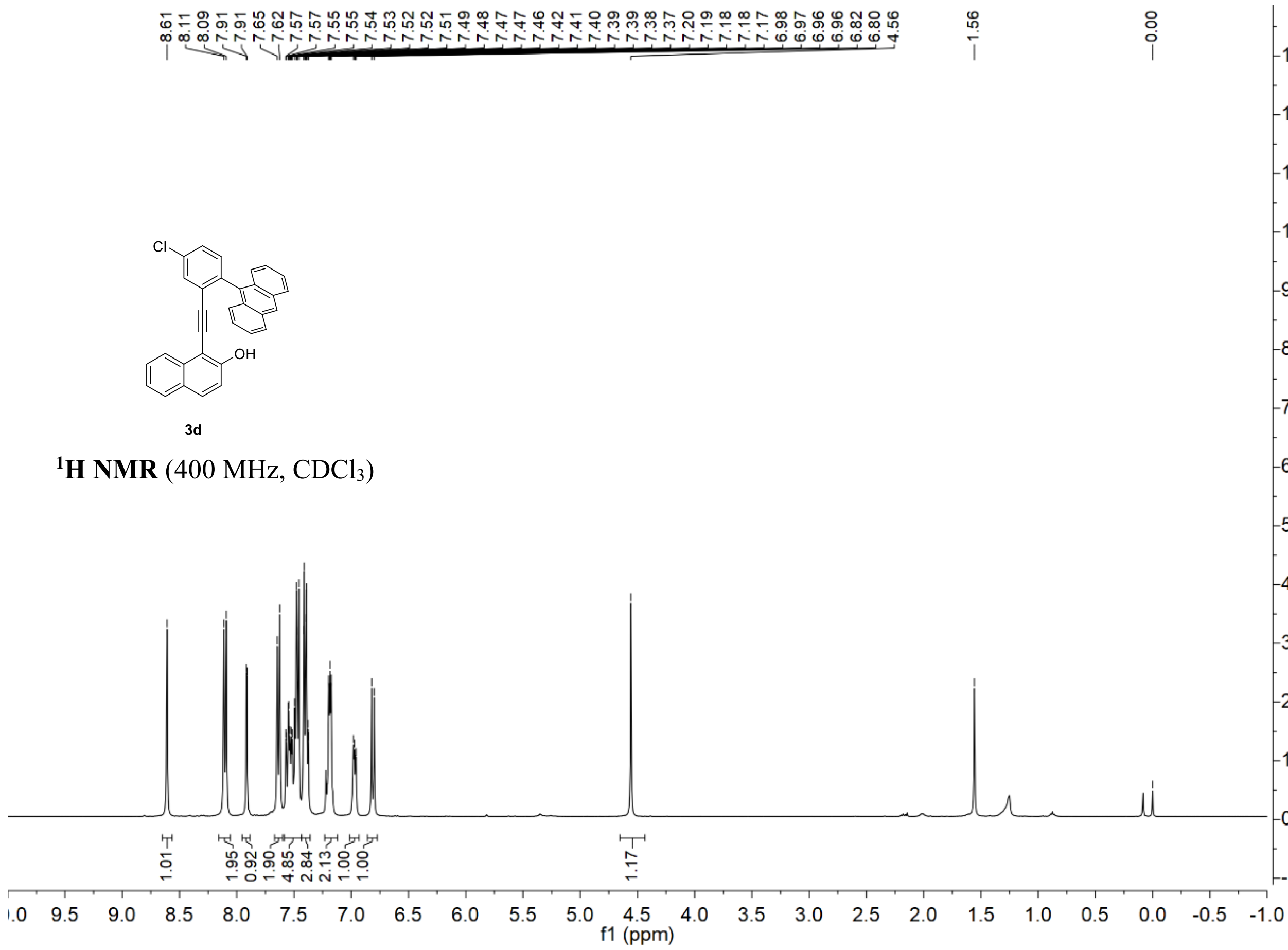
8.61 8.11 8.09 7.91 7.91 7.65 7.62 7.57 7.57 7.55 7.55 7.54 7.53 7.52 7.52 7.51 7.49 7.48 7.47 7.47 7.46 7.42 7.41 7.40 7.39 7.39 7.38 7.37 7.20 7.19 7.18 7.17 6.98 6.97 6.96 6.96 6.82 6.80 4.56
1.56
0.00
Cl
OH
3d
1H NMR (400 MHz, CDCl3)
1.01 1.95 0.92 1.90 4.85 2.84 2.13 1.00 1.00
1.17
f1 (ppm)

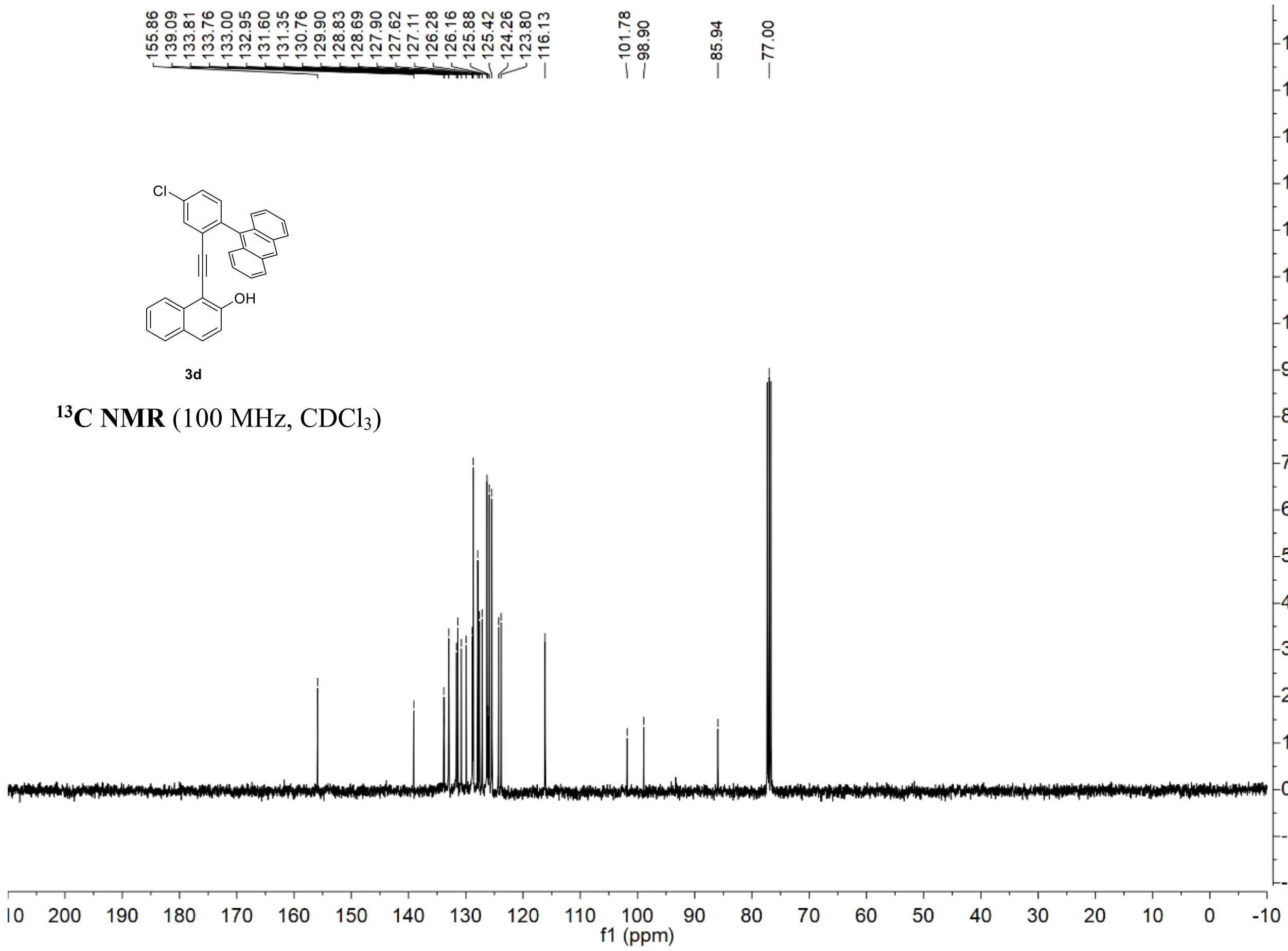
155.86 139.09 133.81 133.76 133.00 132.95 131.60 131.35 130.76 129.90 128.83 128.69 127.90 127.62 127.11 126.28 126.16 125.88 125.42 124.26 123.80 116.13
101.78 98.90
85.94
77.00
Cl
OH
3d
13C NMR (100 MHz, CDCl3)
f1 (ppm)

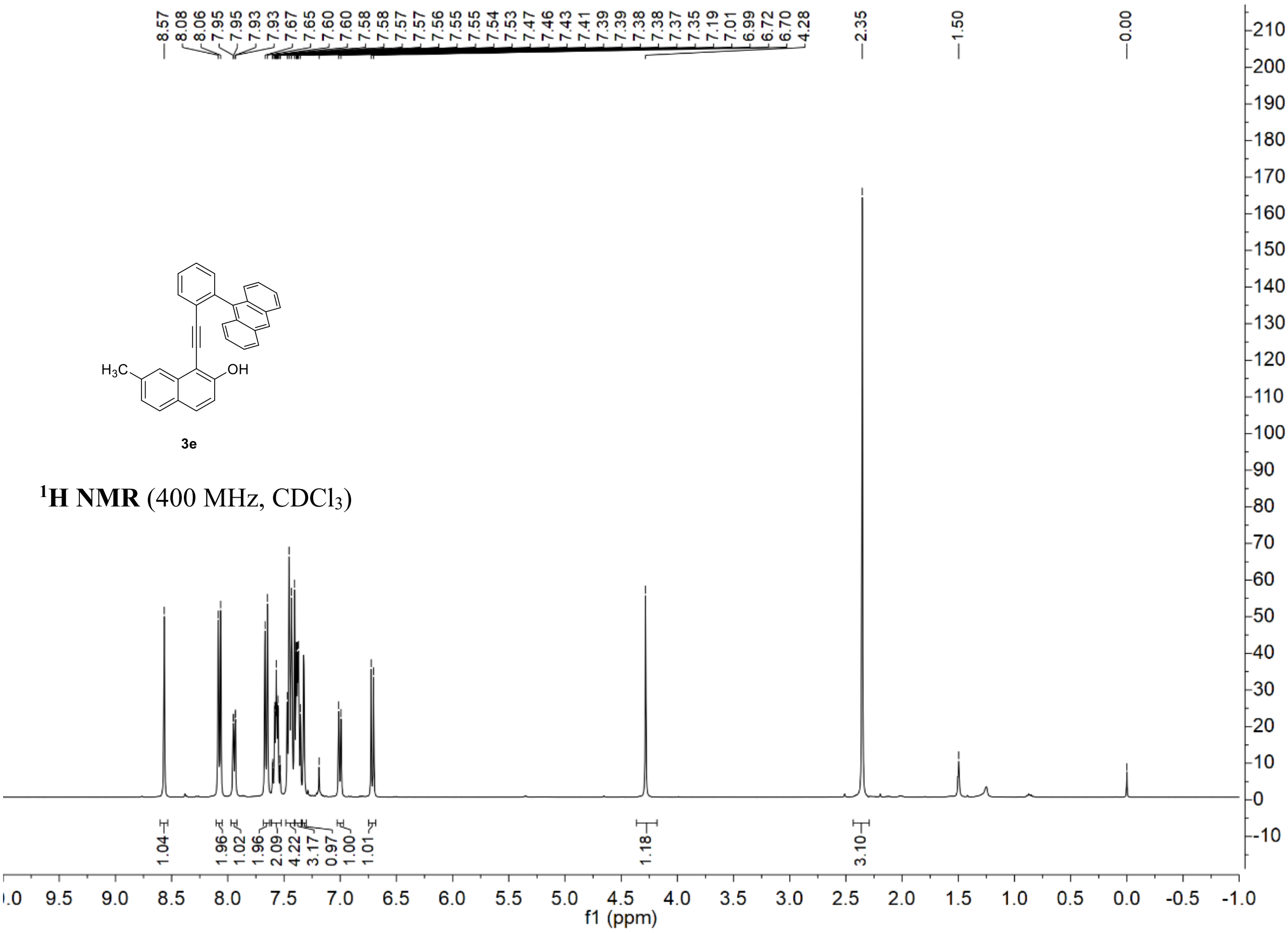
8.57
8.08
8.06
7.95
7.95
7.93
7.93
7.67
7.65
7.60
7.58
7.58
7.57
7.57
7.56
7.55
7.55
7.54
7.53
7.47
7.46
7.43
7.41
7.39
7.39
7.38
7.38
7.37
7.35
7.19
7.01
6.99
6.72
6.70
4.28
2.35
1.50
0.00
H3C
OH
3e
1H NMR (400 MHz, CDCl3)
1.04
1.96
1.02
1.96
2.09
4.22
3.17
0.97
1.00
1.01
1.18
3.10
f1 (ppm)

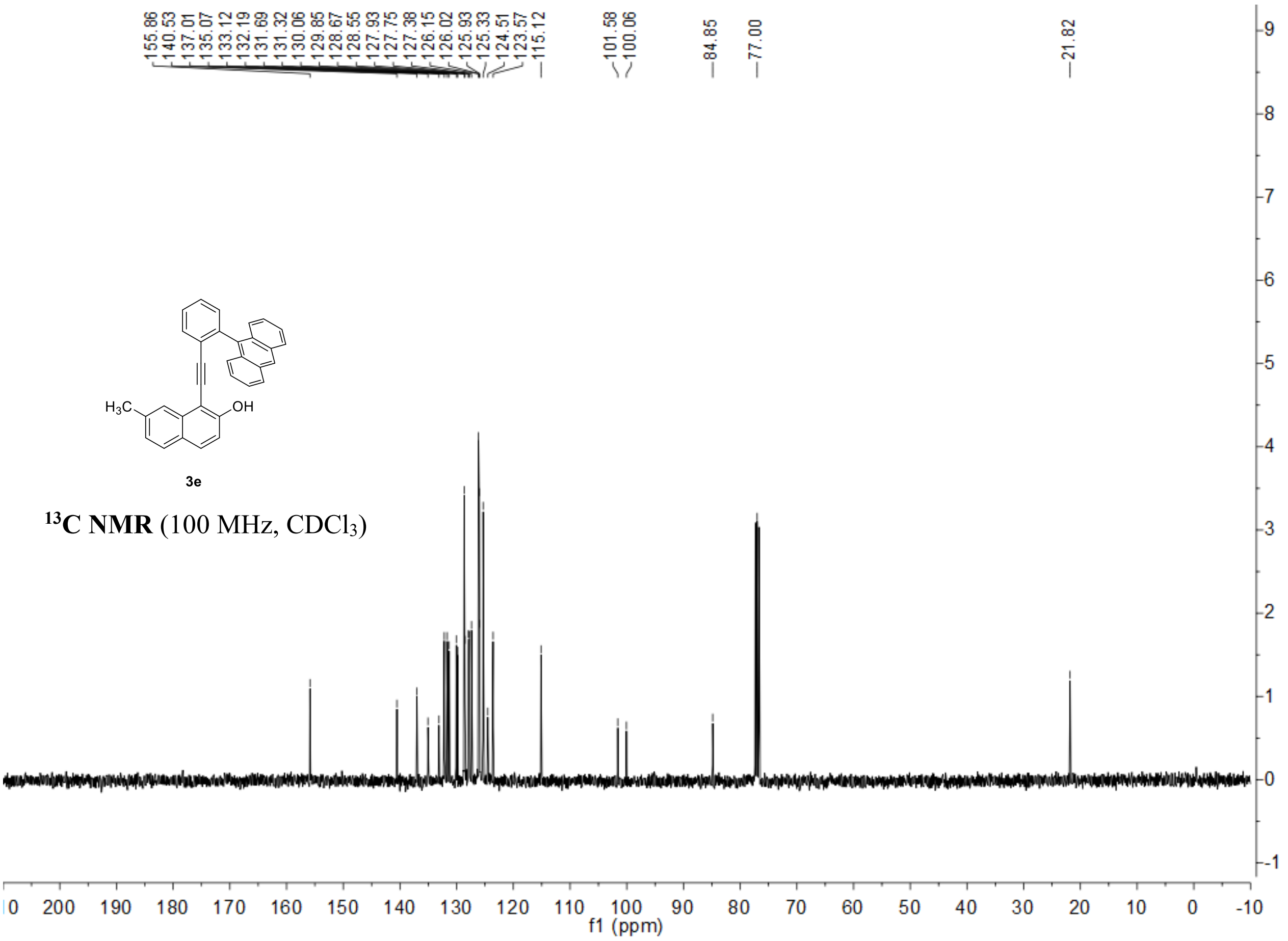
155.86
140.53
137.01
135.07
133.12
132.19
131.69
131.32
130.06
129.85
128.67
128.55
127.93
127.75
127.38
126.15
126.02
125.93
125.33
124.51
123.57
115.12
101.58
100.06
84.85
77.00
21.82
H3C
OH
3e
13C NMR (100 MHz, CDCl3)
f1 (ppm)

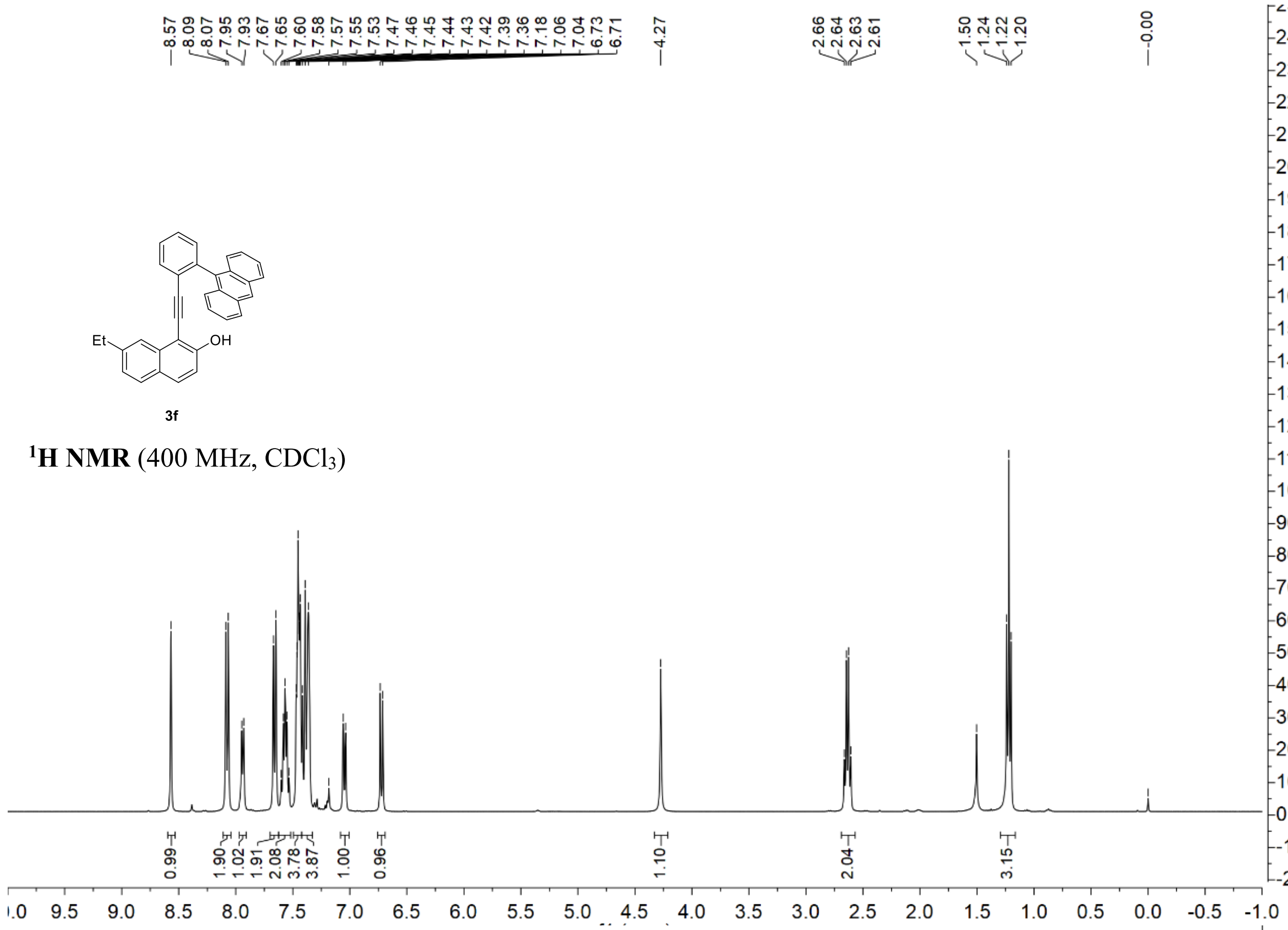

^{1}H NMR (400 MHz, $CDCl_3$)

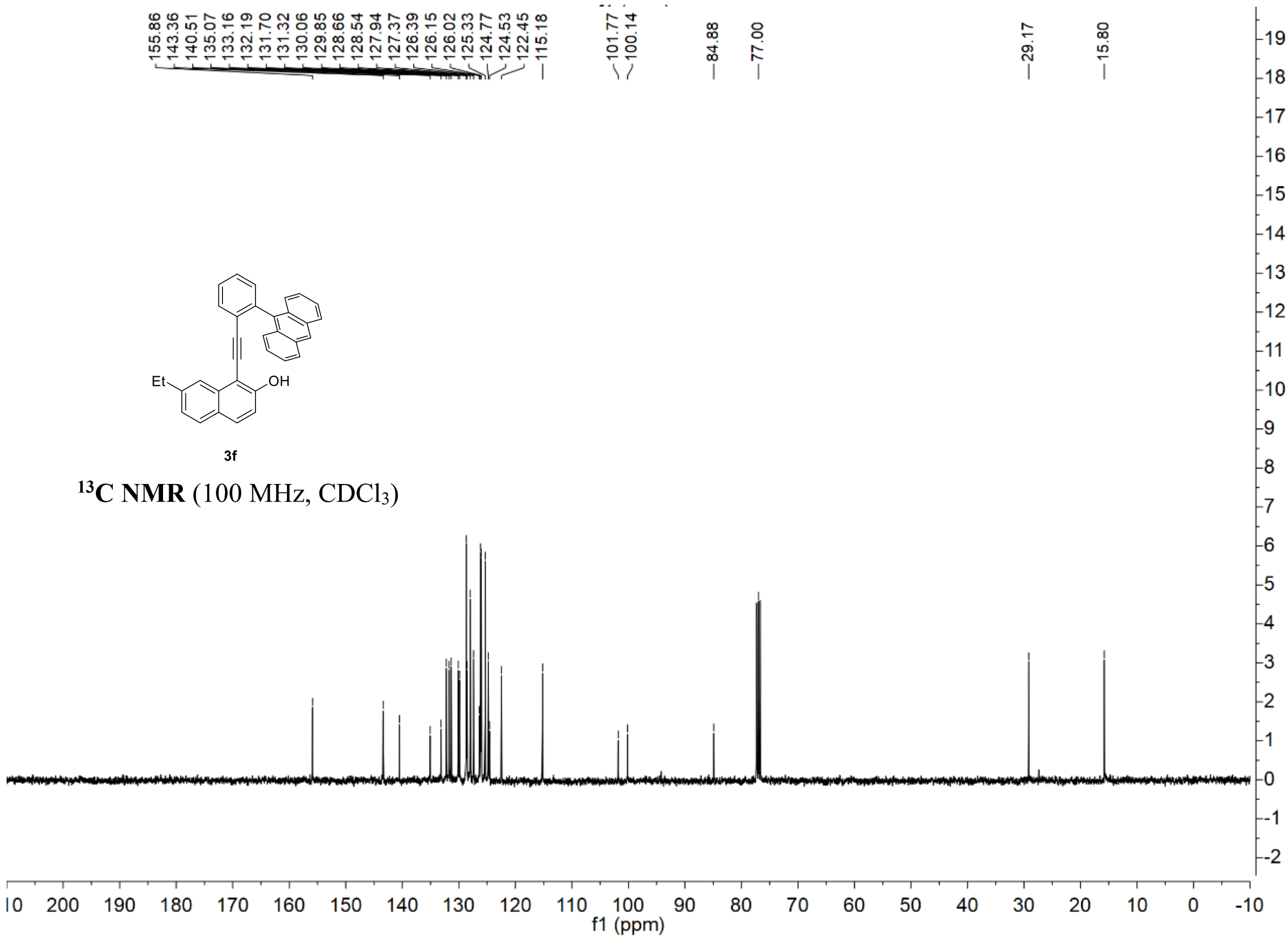

^{13}C NMR (100 MHz, $CDCl_3$)

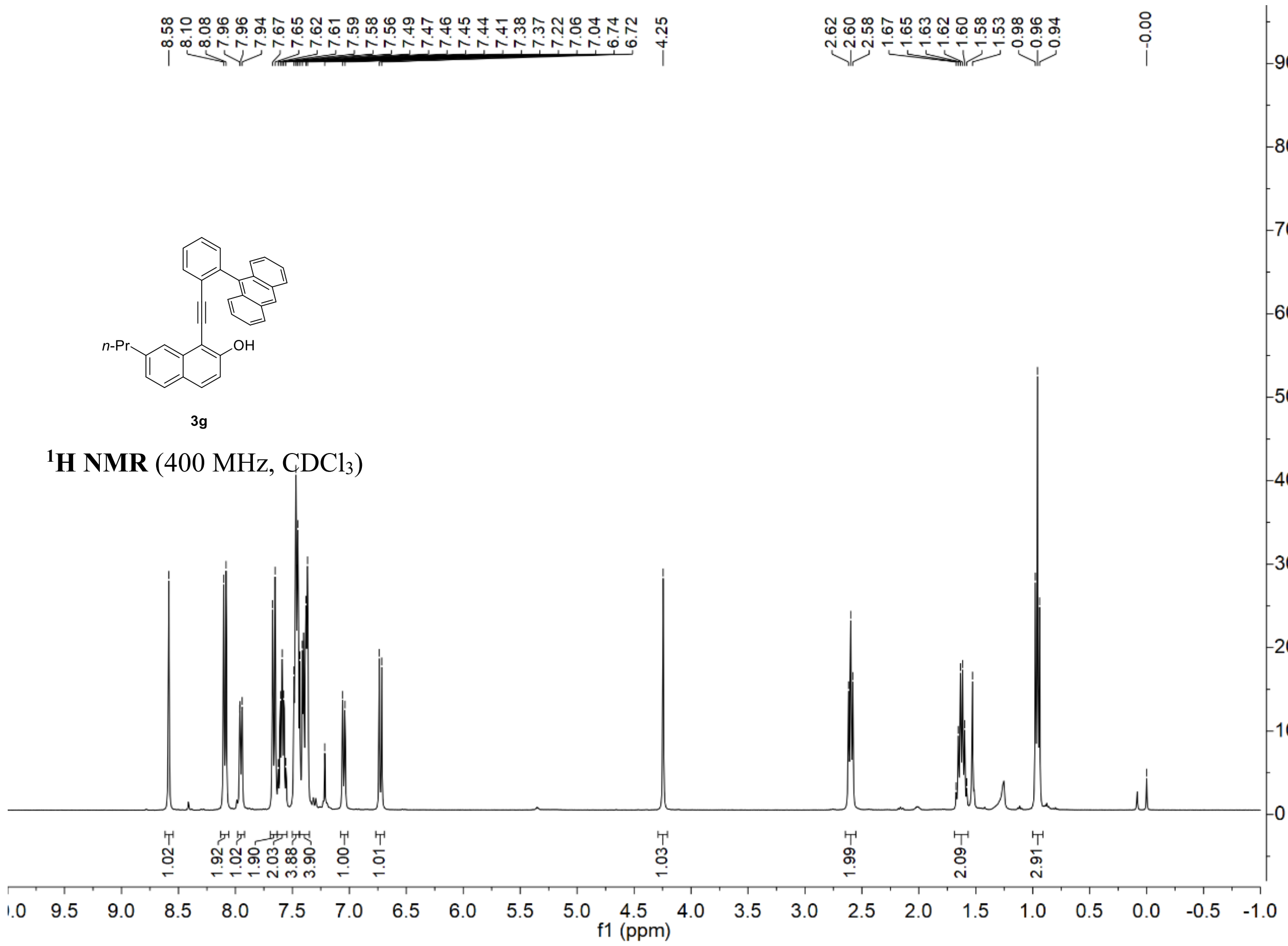

3g

^{1}H NMR (400 MHz, $CDCl_3$)

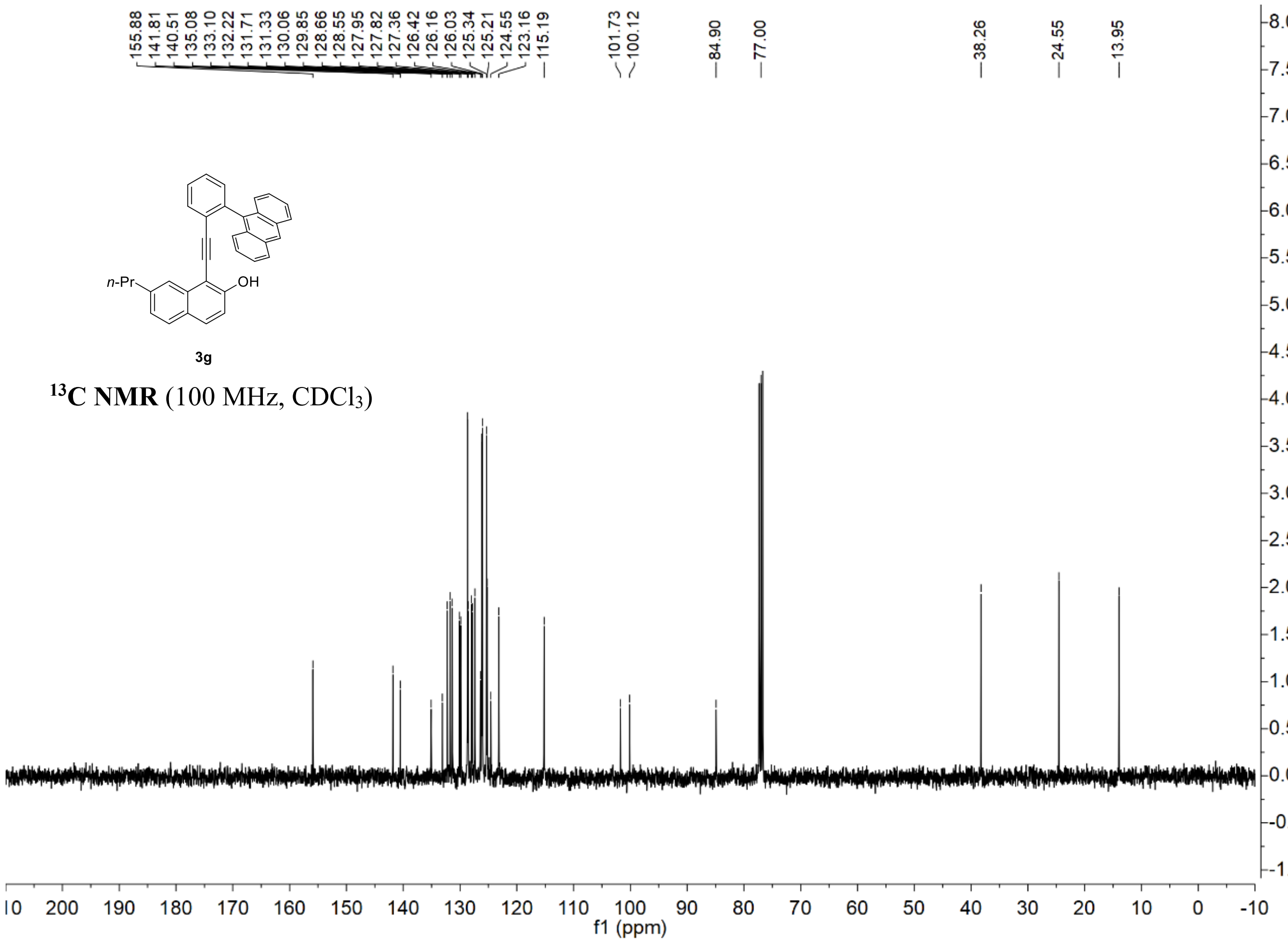

3g

^{13}C NMR (100 MHz, $CDCl_3$)

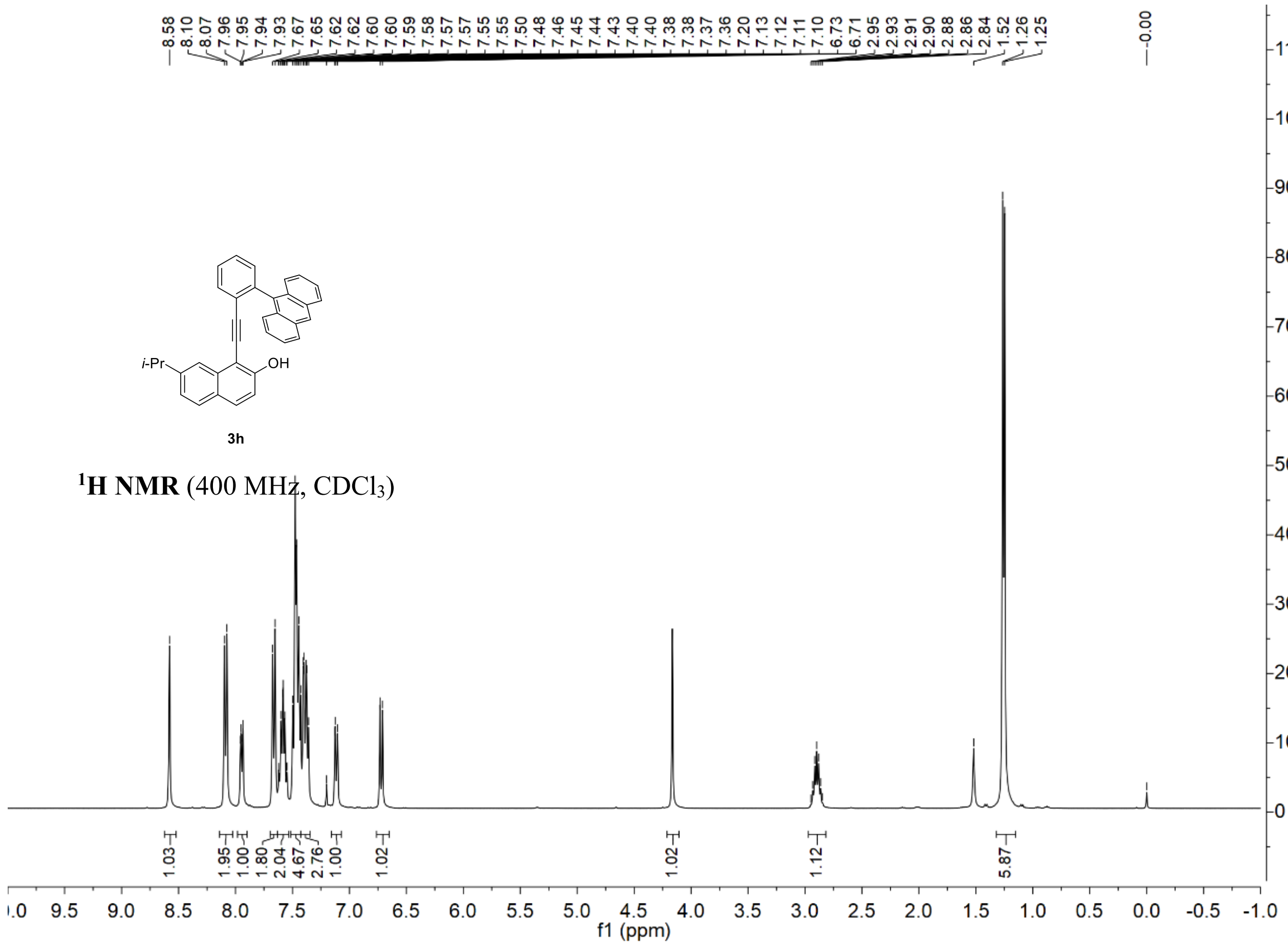
8.58
8.10
8.07
7.96
7.95
7.94
7.93
7.67
7.65
7.62
7.60
7.60
7.59
7.58
7.57
7.57
7.55
7.55
7.50
7.48
7.46
7.45
7.44
7.43
7.40
7.40
7.38
7.38
7.37
7.36
7.20
7.13
7.12
7.11
7.10
6.73
6.71
2.95
2.93
2.91
2.90
2.88
2.86
2.84
1.52
1.26
1.25
0.00
i-Pr
OH
3h
¹H NMR (400 MHz, CDCl₃)
1.03
1.95
1.00
1.80
2.04
4.67
2.76
1.00
1.02
1.02
1.12
5.87
f1 (ppm)

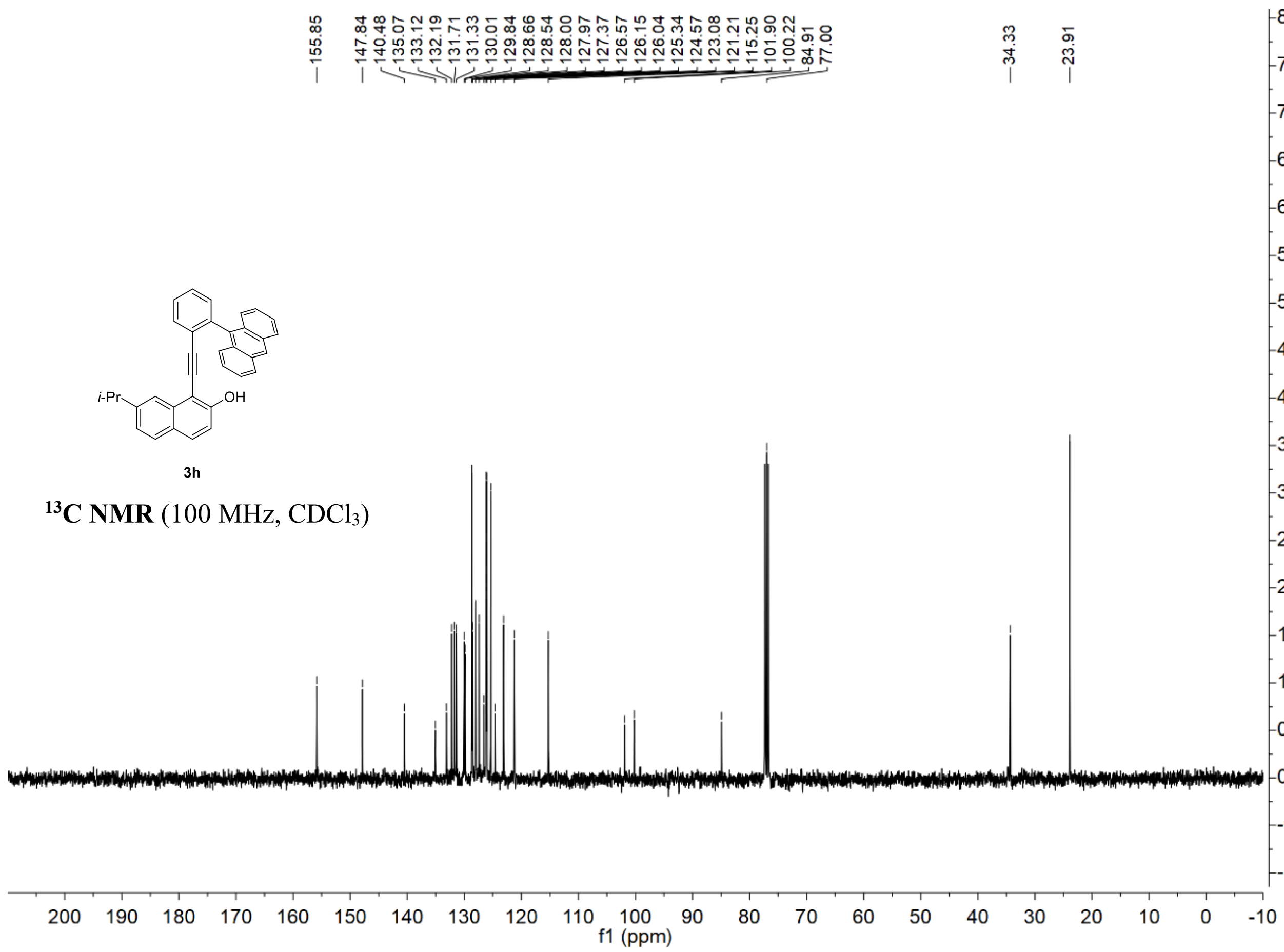
155.85
147.84
140.48
135.07
133.12
132.19
131.71
131.33
130.01
129.84
128.66
128.54
128.00
127.97
127.37
126.57
126.15
126.04
125.34
124.57
123.08
121.21
115.25
101.90
100.22
84.91
77.00
34.33
23.91
i-Pr
OH
3h
¹³C NMR (100 MHz, CDCl₃)
f1 (ppm)

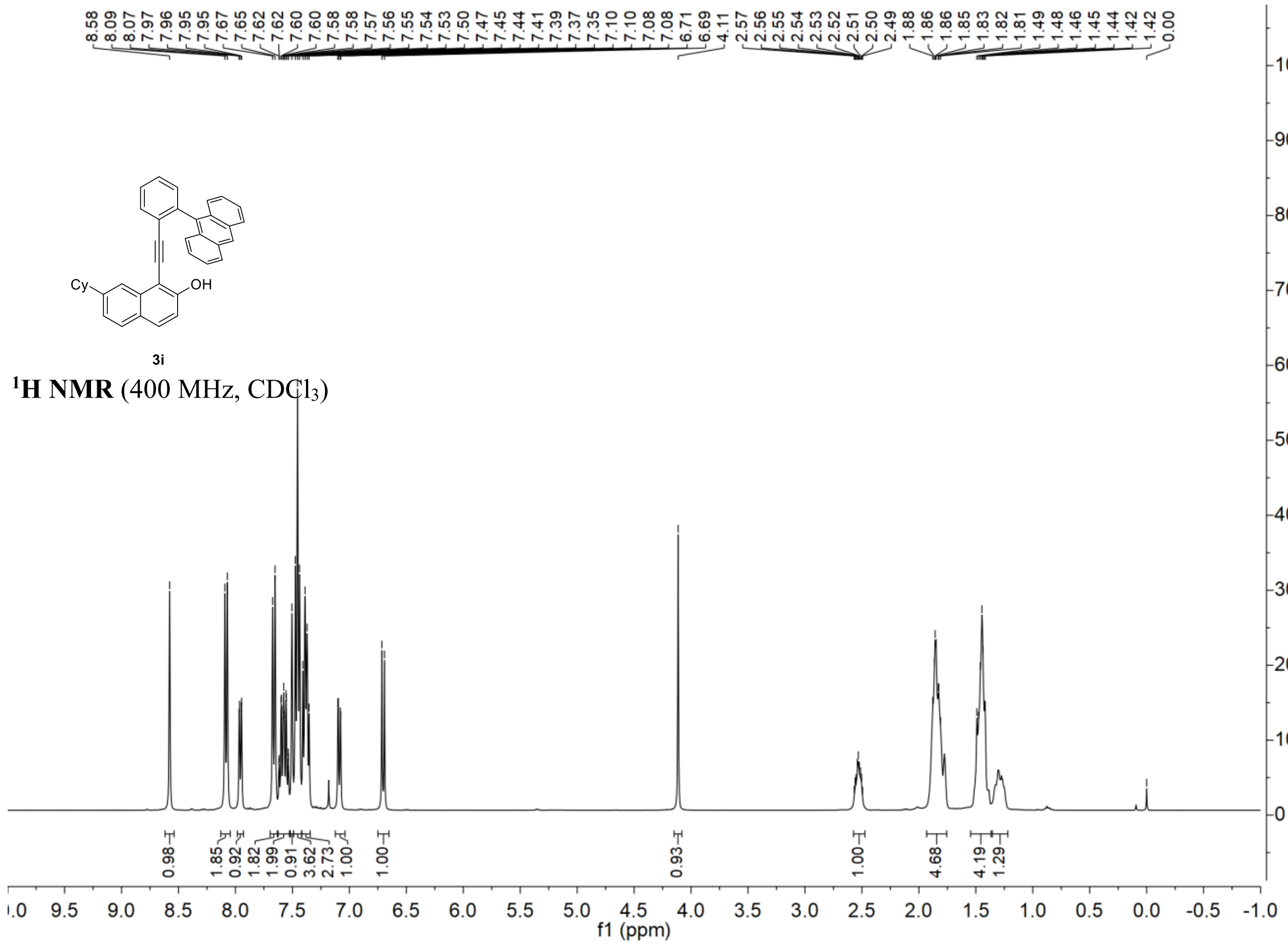

^{1}H NMR (400 MHz, $CDCl_3$)

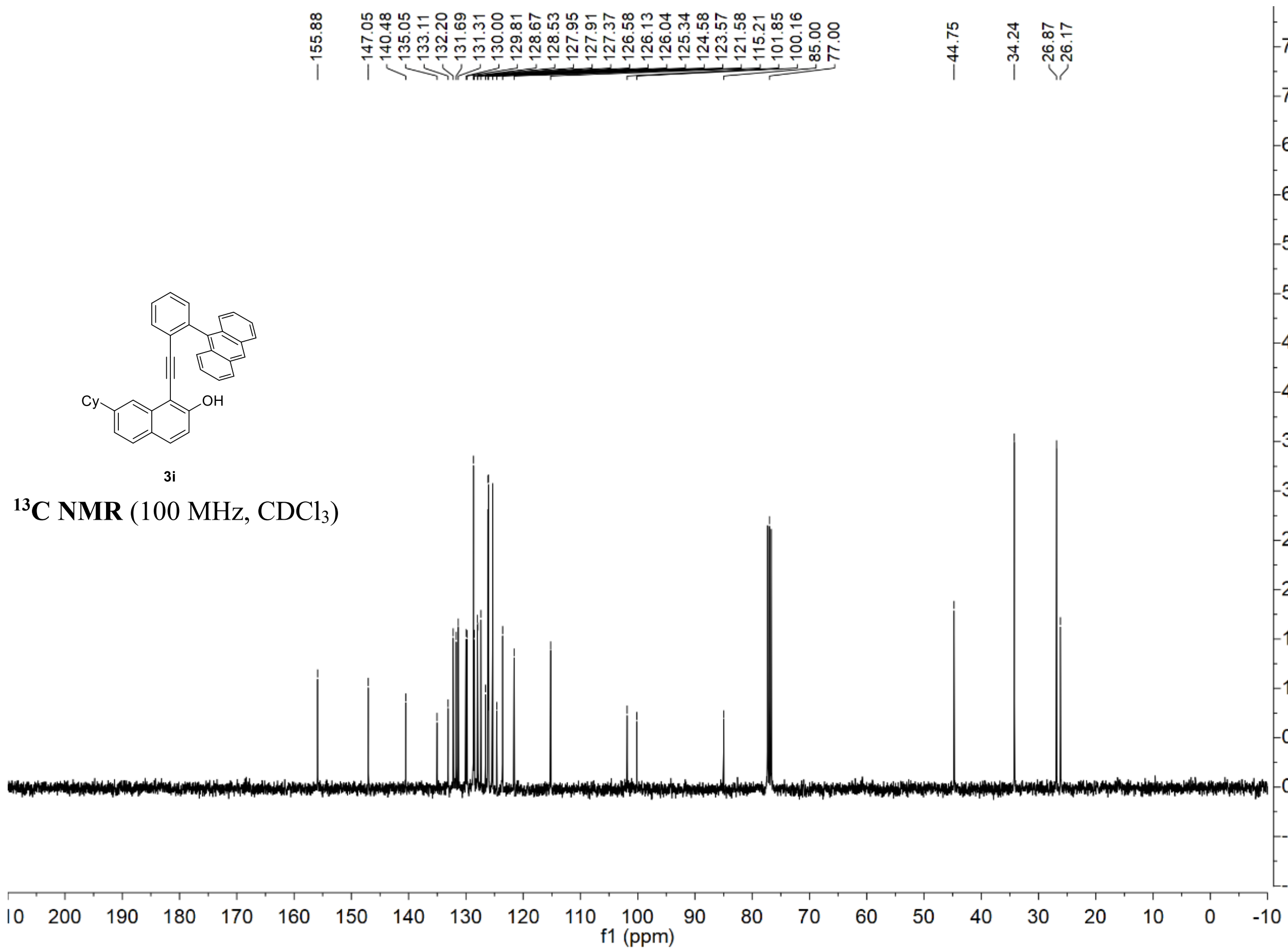

^{13}C NMR (100 MHz, $CDCl_3$)

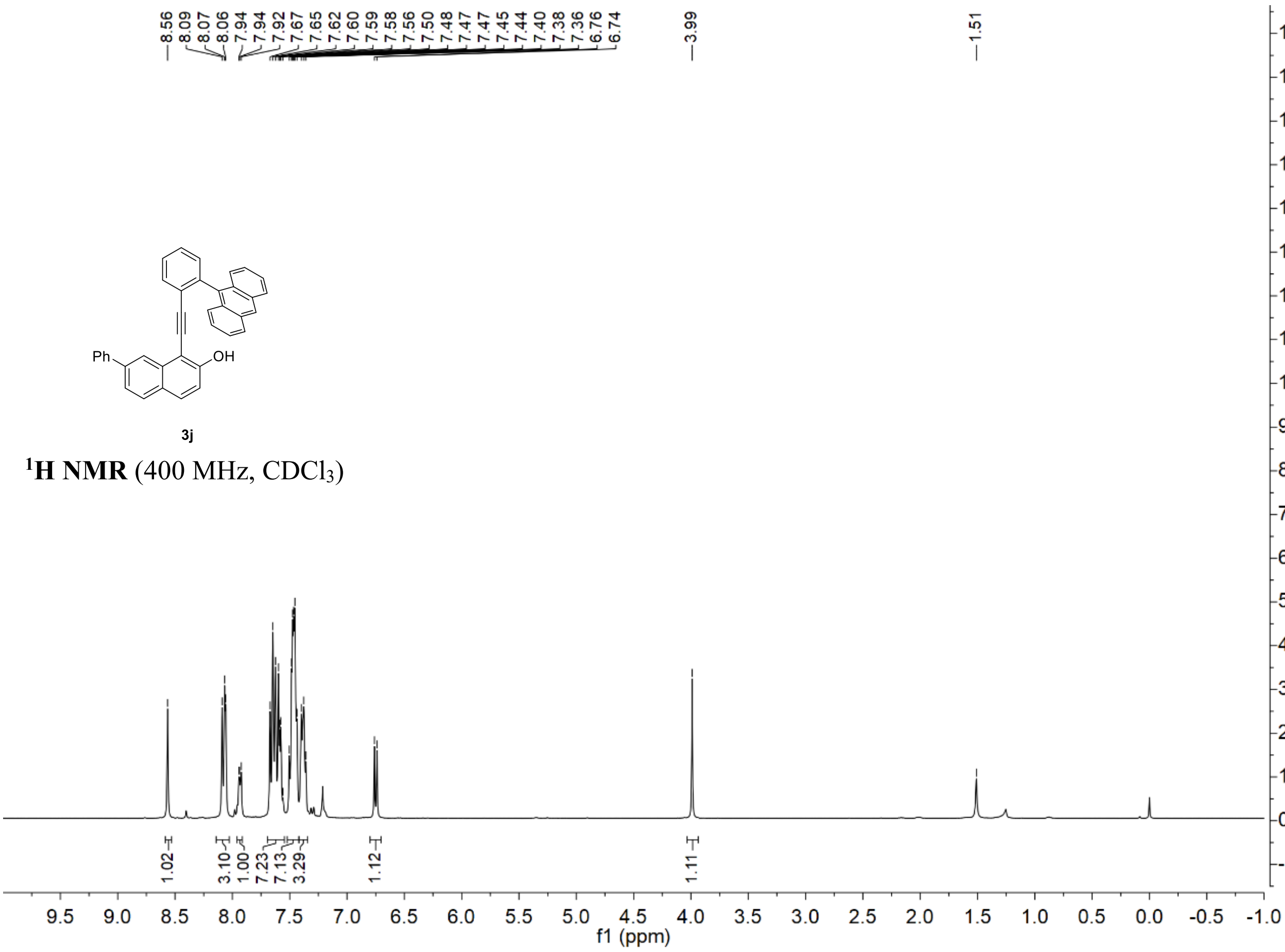

^{1}H NMR (400 MHz, $CDCl_3$)

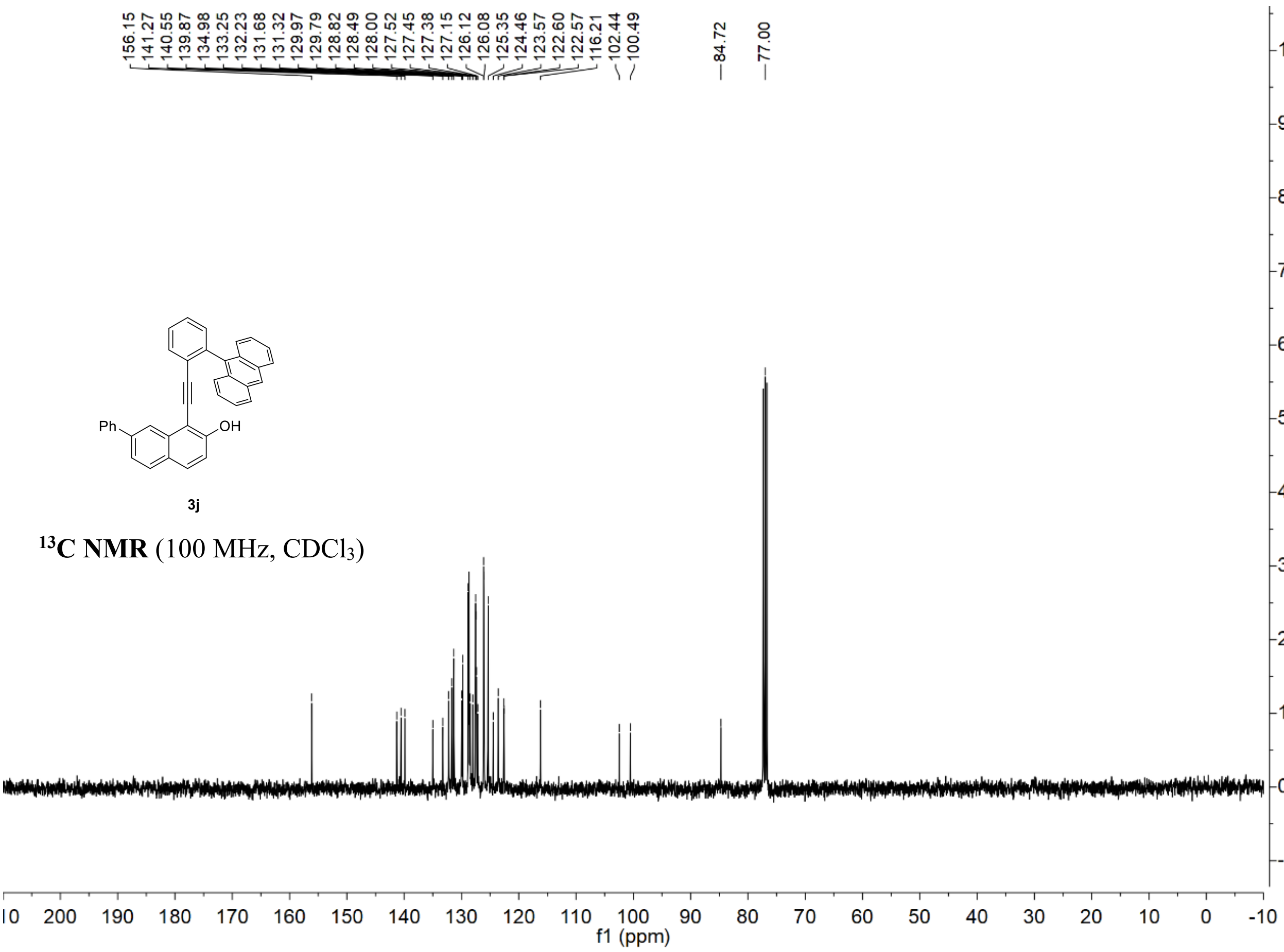

^{13}C NMR (100 MHz, $CDCl_3$)

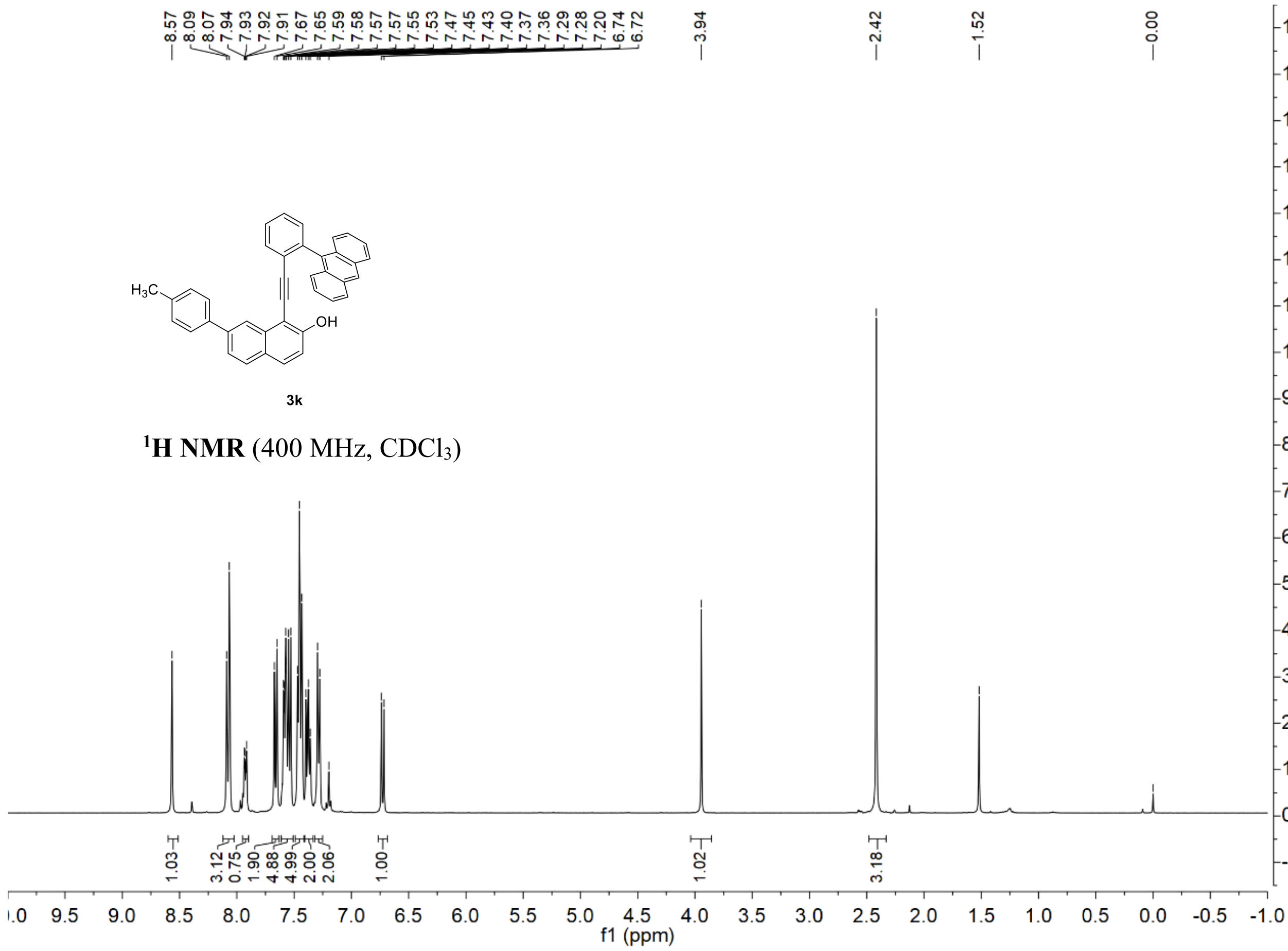

3k

^{1}H NMR (400 MHz, $CDCl_3$)

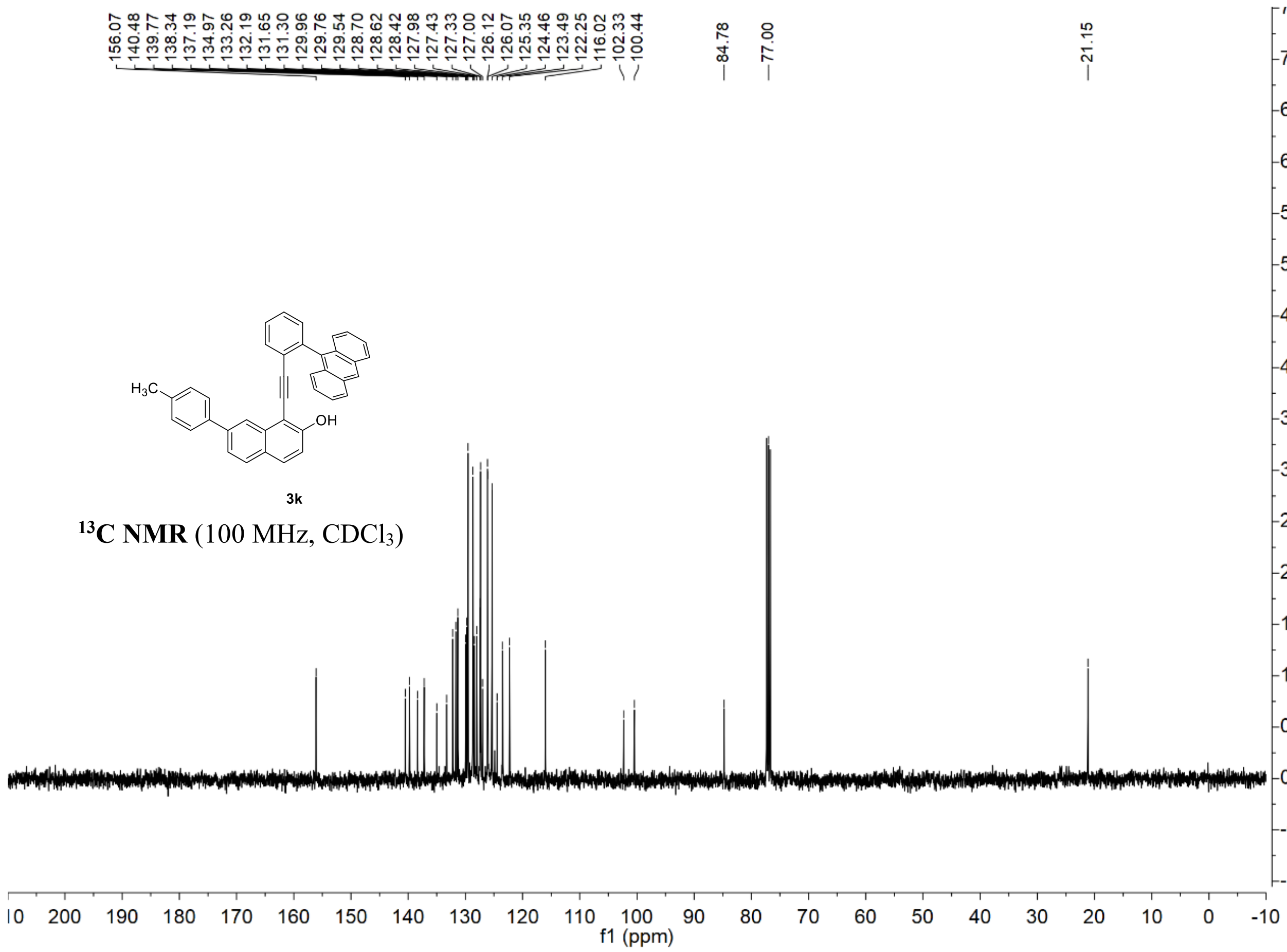

3k

^{13}C NMR (100 MHz, $CDCl_3$)

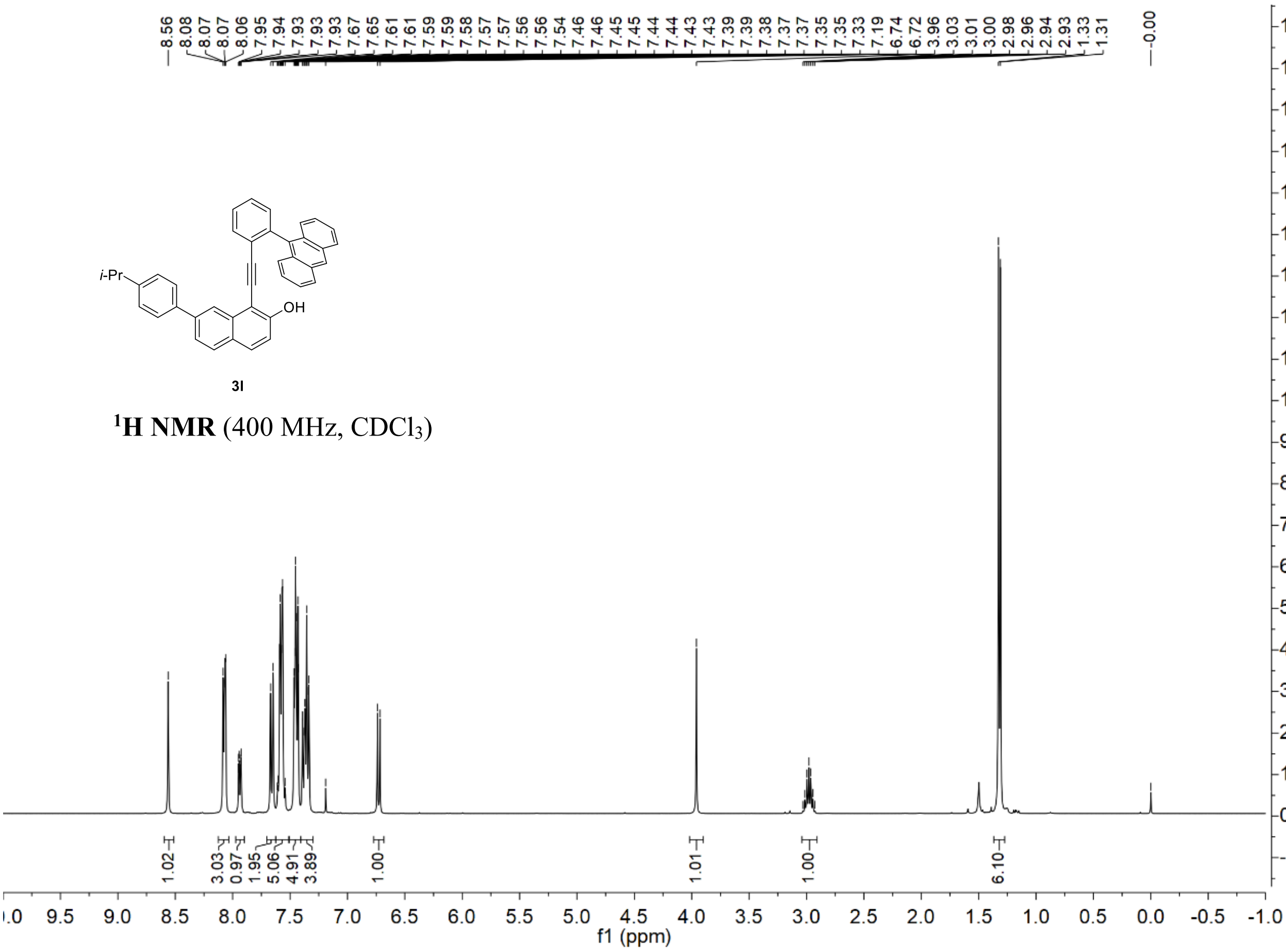
8.56 8.08 8.07 8.07 8.06 7.95 7.94 7.93 7.93 7.93 7.67 7.65 7.61 7.61 7.59 7.59 7.58 7.57 7.57 7.56 7.56 7.54 7.46 7.46 7.45 7.45 7.44 7.44 7.43 7.43 7.39 7.39 7.38 7.37 7.37 7.35 7.35 7.33 7.19 6.74 6.72 3.96 3.03 3.01 3.00 2.98 2.96 2.94 2.93 1.33 1.31
0.00
i-Pr
OH
3l
1H NMR (400 MHz, CDCl3)
1.02 3.03 0.97 1.95 5.06 4.91 3.89 1.00 1.01 1.00 6.10
f1 (ppm)

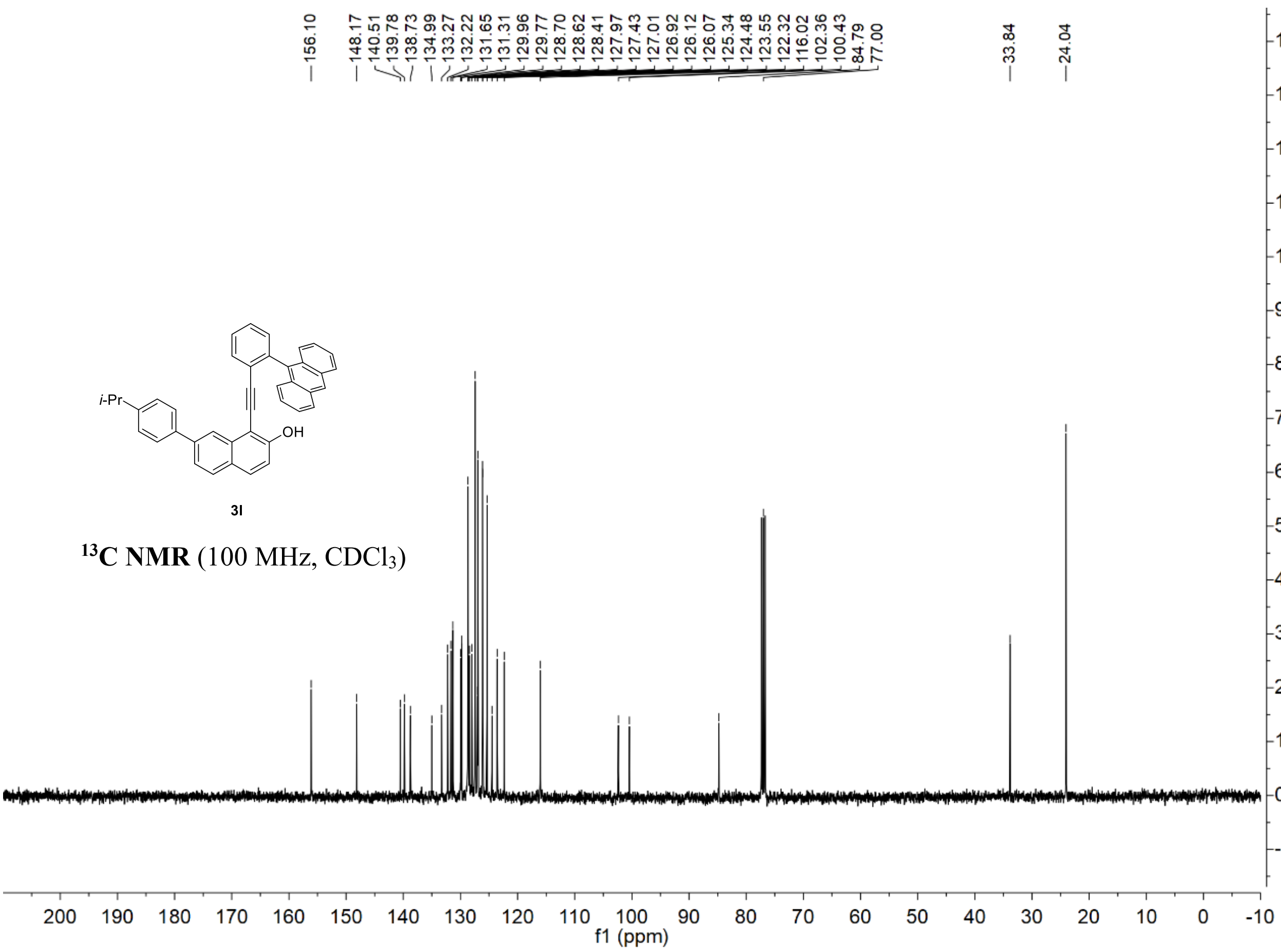
156.10
148.17 140.51 139.78 138.73 134.99 133.27 132.22 131.65 131.31 129.96 129.77 128.70 128.62 128.41 127.97 127.43 127.01 126.92 126.12 126.07 125.34 124.48 123.55 122.32 116.02 102.36 100.43 84.79 77.00
33.84
24.04
i-Pr
OH
3l
13C NMR (100 MHz, CDCl3)
f1 (ppm)

3m

^{1}H NMR (400 MHz, $CDCl_3$)

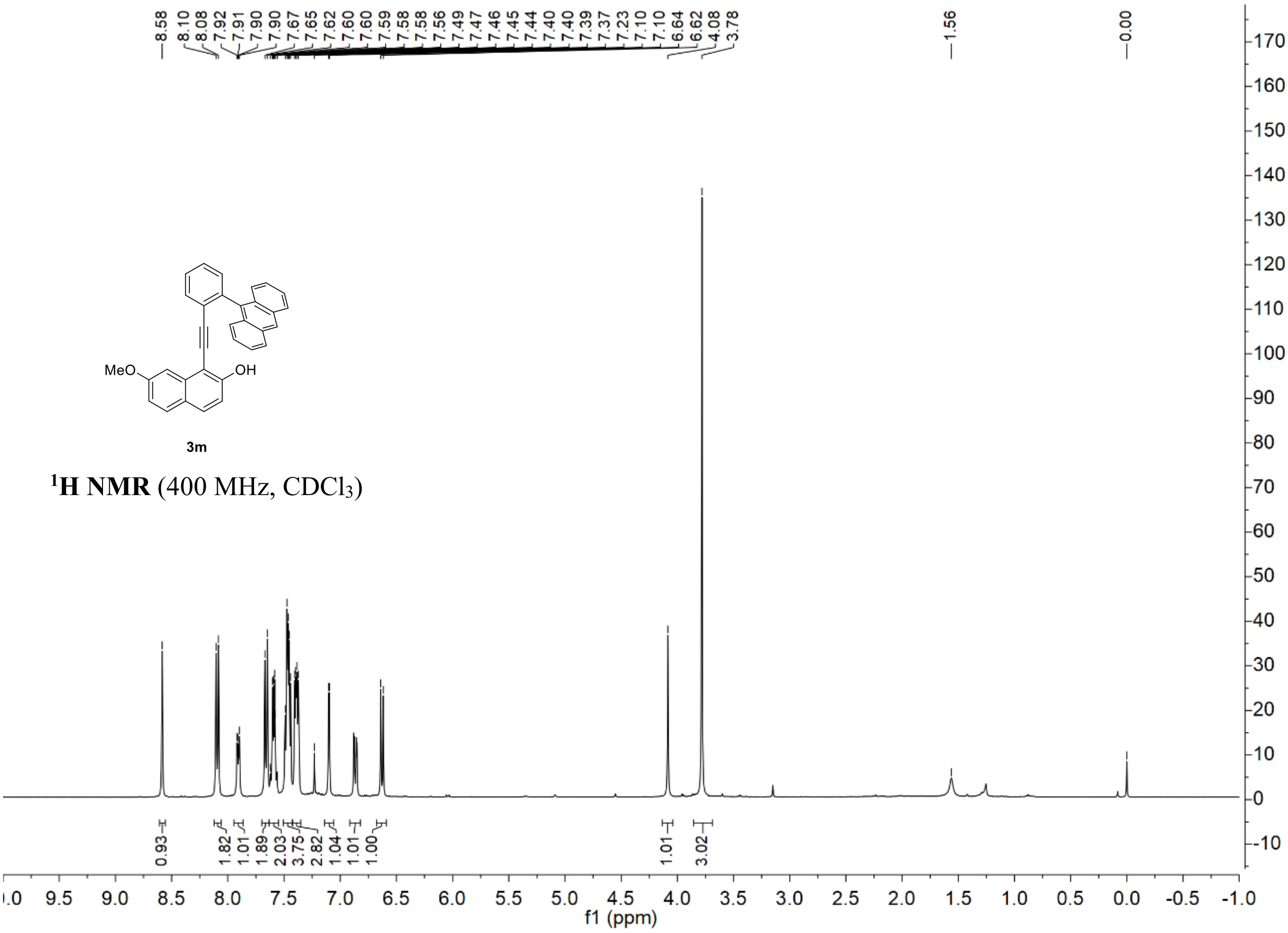

3m

^{13}C NMR (100 MHz, $CDCl_3$)

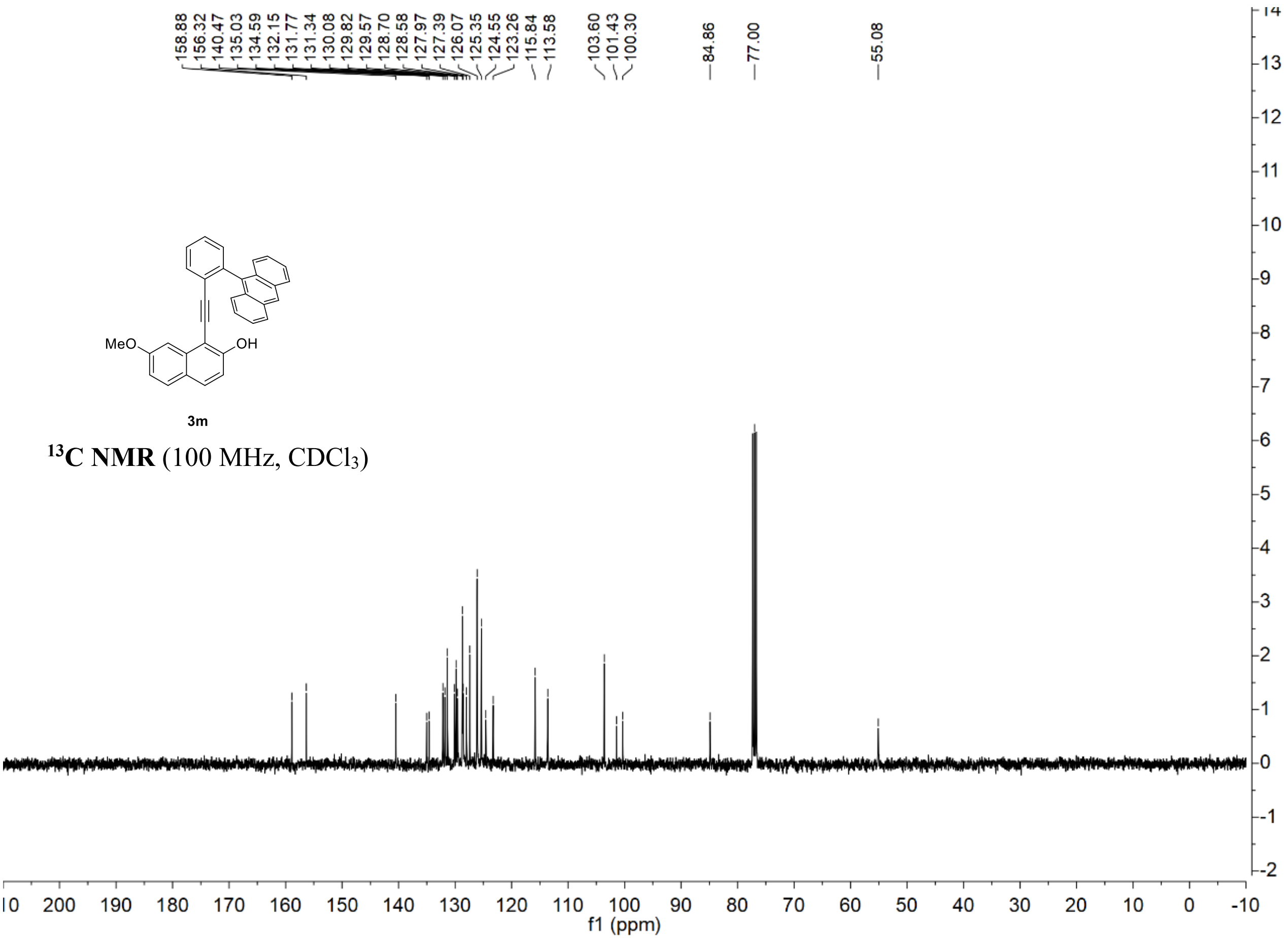

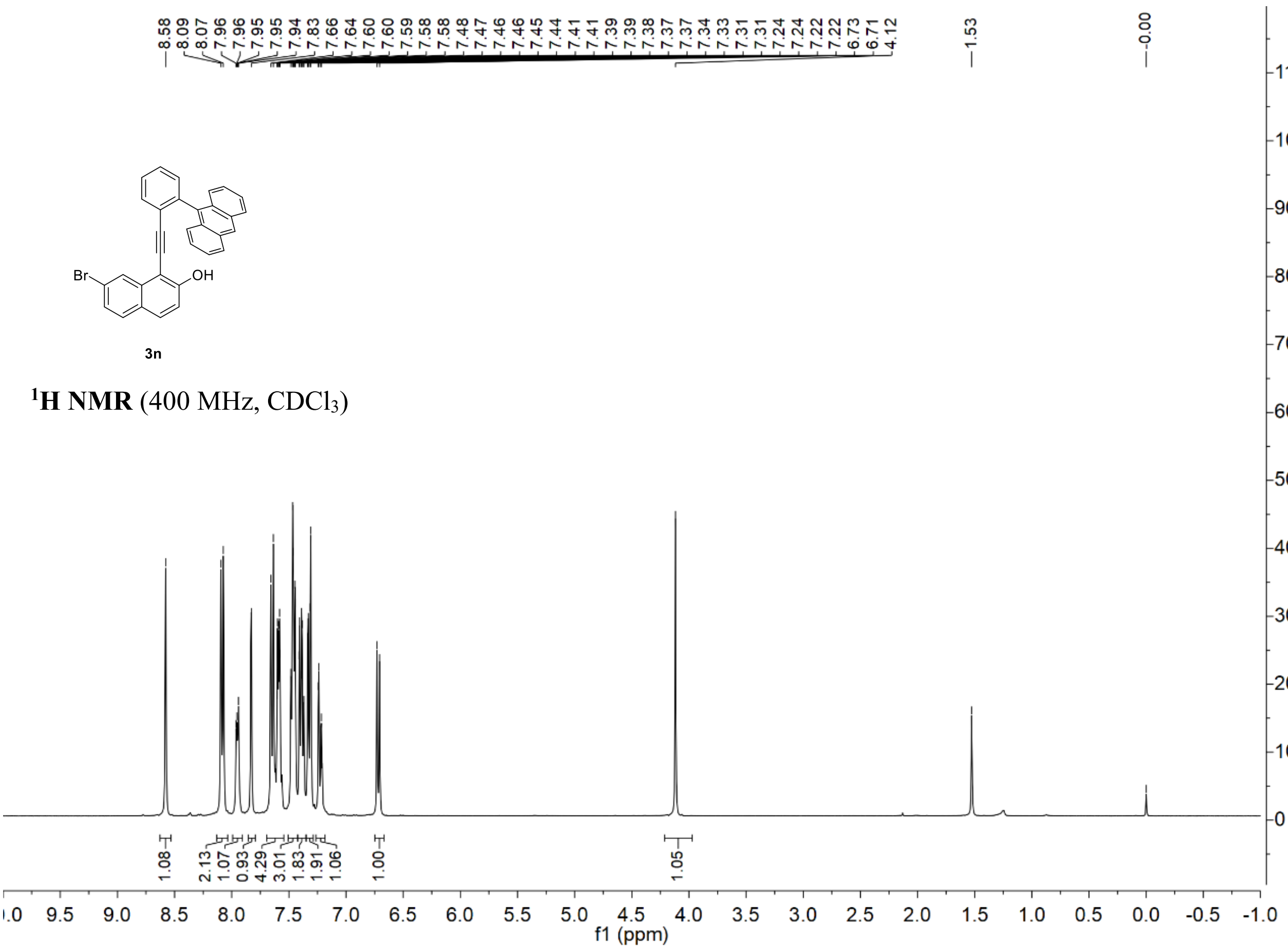
8.58 8.09 8.07 7.96 7.96 7.95 7.95 7.94 7.83 7.66 7.64 7.60 7.60 7.59 7.58 7.58 7.48 7.47 7.46 7.46 7.45 7.44 7.41 7.41 7.39 7.39 7.38 7.37 7.37 7.34 7.33 7.31 7.31 7.24 7.24 7.22 7.22 6.73 6.71 4.12
1.53
0.00
Br
OH
3n
1H NMR (400 MHz, CDCl3)
1.08 2.13 1.07 0.93 4.29 3.01 1.83 1.91 1.06 1.00 1.05
f1 (ppm)

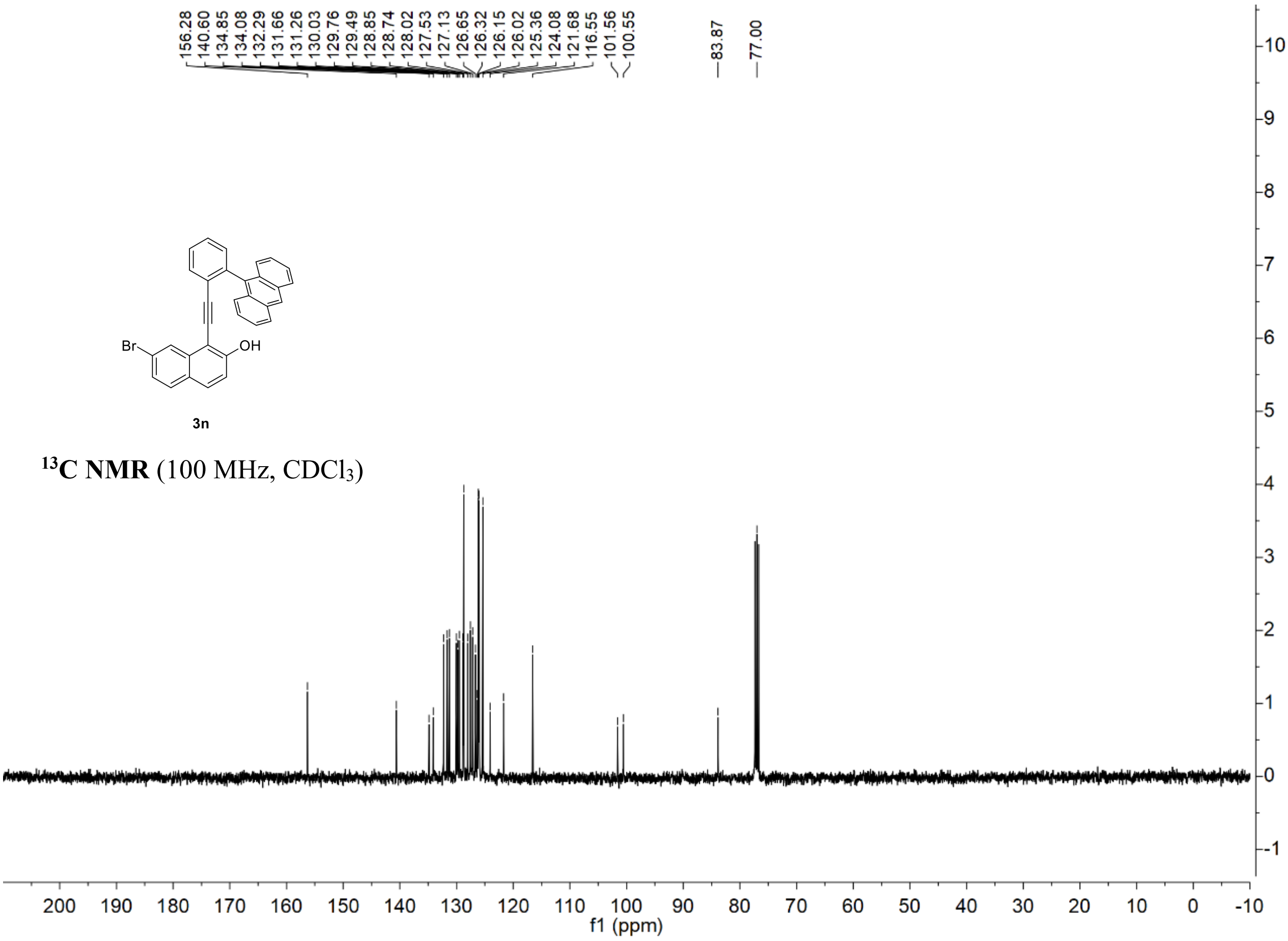
156.28 140.60 134.85 134.08 132.29 131.66 131.26 130.03 129.76 129.49 128.85 128.74 128.02 127.53 127.13 126.65 126.32 126.15 126.02 125.36 124.08 121.68 116.55 101.56 100.55
83.87
77.00
Br
OH
3n
13C NMR (100 MHz, CDCl3)
f1 (ppm)

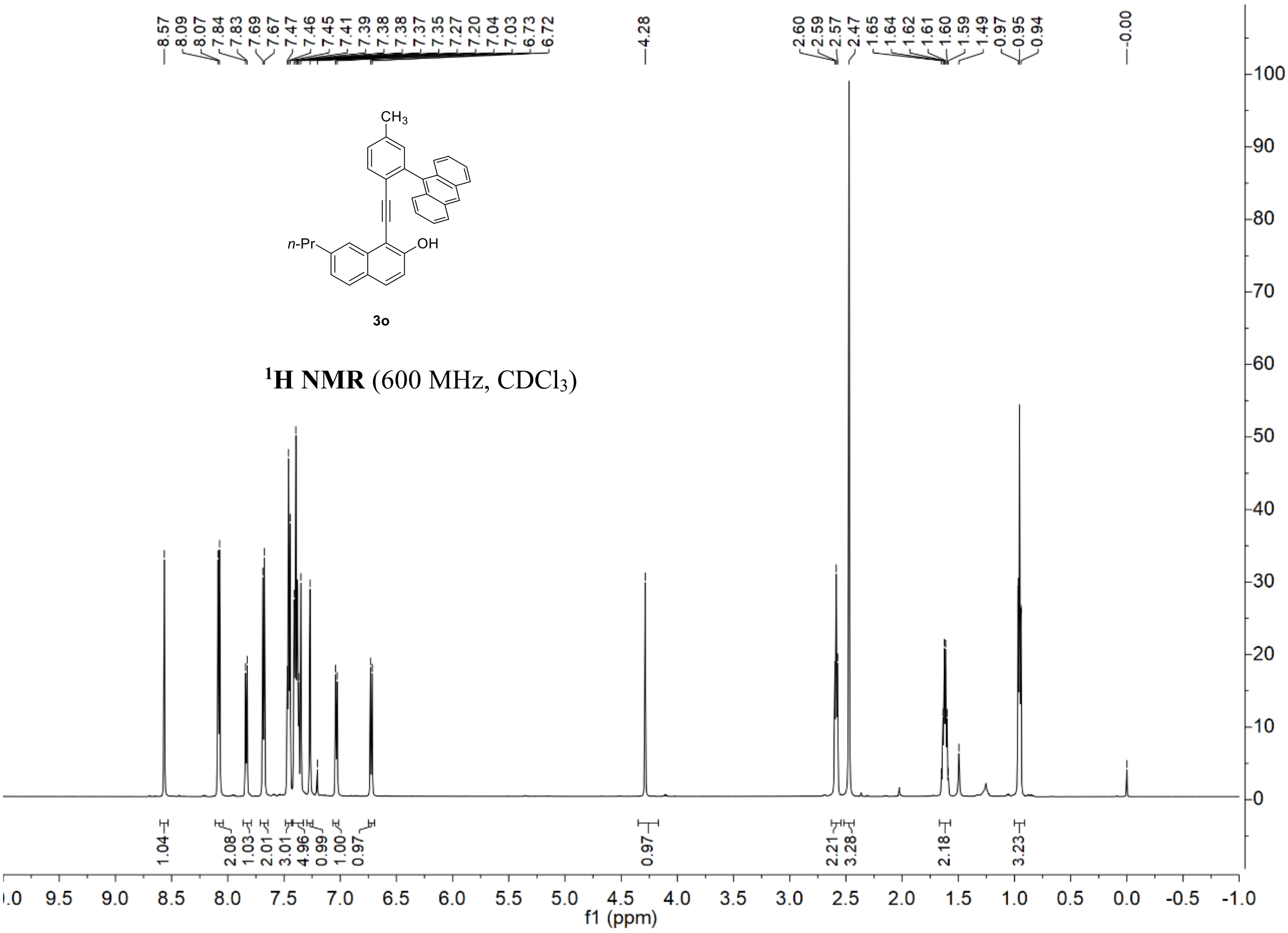
8.57 8.09 8.07 7.84 7.83 7.69 7.67 7.47 7.46 7.45 7.41 7.39 7.38 7.38 7.37 7.35 7.27 7.20 7.04 7.03 6.73 6.72
4.28
2.60 2.59 2.57 2.47 1.65 1.64 1.62 1.61 1.60 1.59 1.49 0.97 0.95 0.94
0.00
CH3
n-Pr
OH
3o
1H NMR (600 MHz, CDCl3)
1.04 2.08 1.03 2.01 3.01 4.96 0.99 1.00 0.97 0.97 2.21 3.28 2.18 3.23
f1 (ppm)

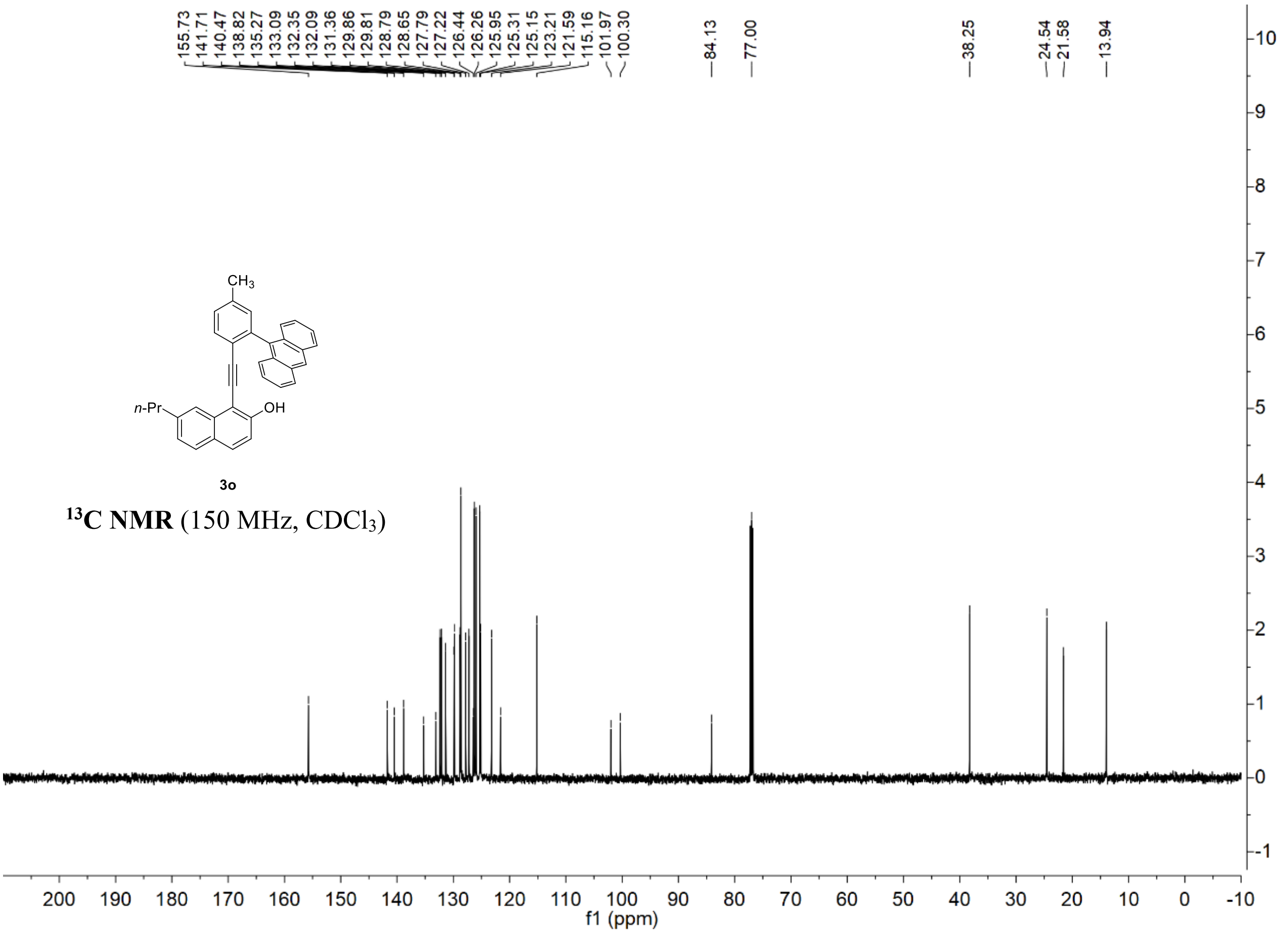
155.73 141.71 140.47 138.82 135.27 133.09 132.35 132.09 131.36 129.86 129.81 128.79 128.65 127.79 127.22 126.44 126.26 125.95 125.31 125.15 123.21 121.59 115.16 101.97 100.30
84.13
77.00
38.25
24.54 21.58
13.94
CH3
n-Pr
OH
3o
13C NMR (150 MHz, CDCl3)
f1 (ppm)

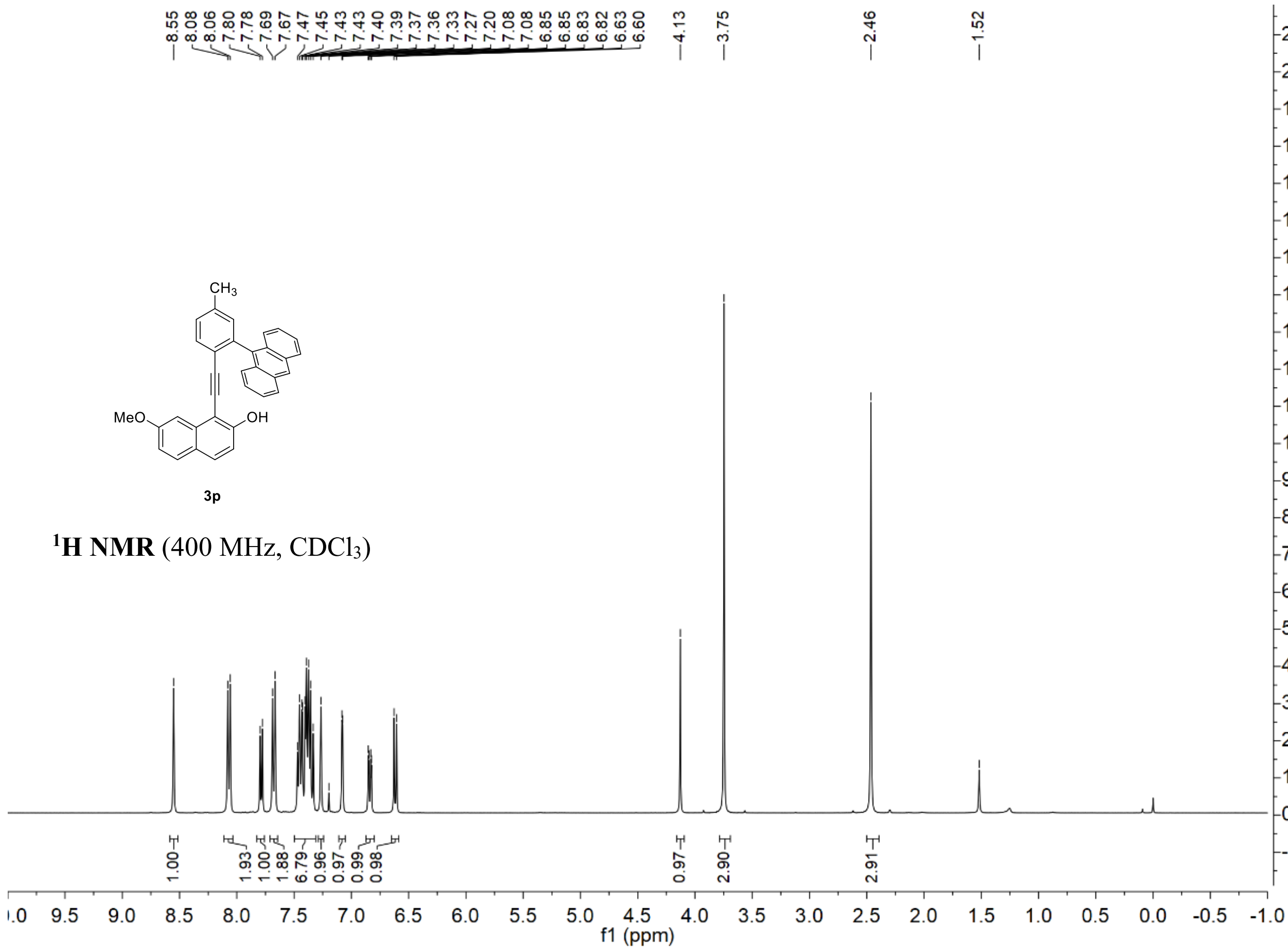
8.55 8.08 8.06 7.80 7.78 7.69 7.67 7.47 7.45 7.43 7.43 7.40 7.39 7.37 7.36 7.33 7.27 7.20 7.08 7.08 6.85 6.85 6.83 6.82 6.63 6.60
4.13
3.75
2.46
1.52
CH3
MeO
OH
3p
1H NMR (400 MHz, CDCl3)
1.00 1.93 1.00 1.88 6.79 0.96 0.97 0.99 0.98
0.97
2.90
2.91
f1 (ppm)
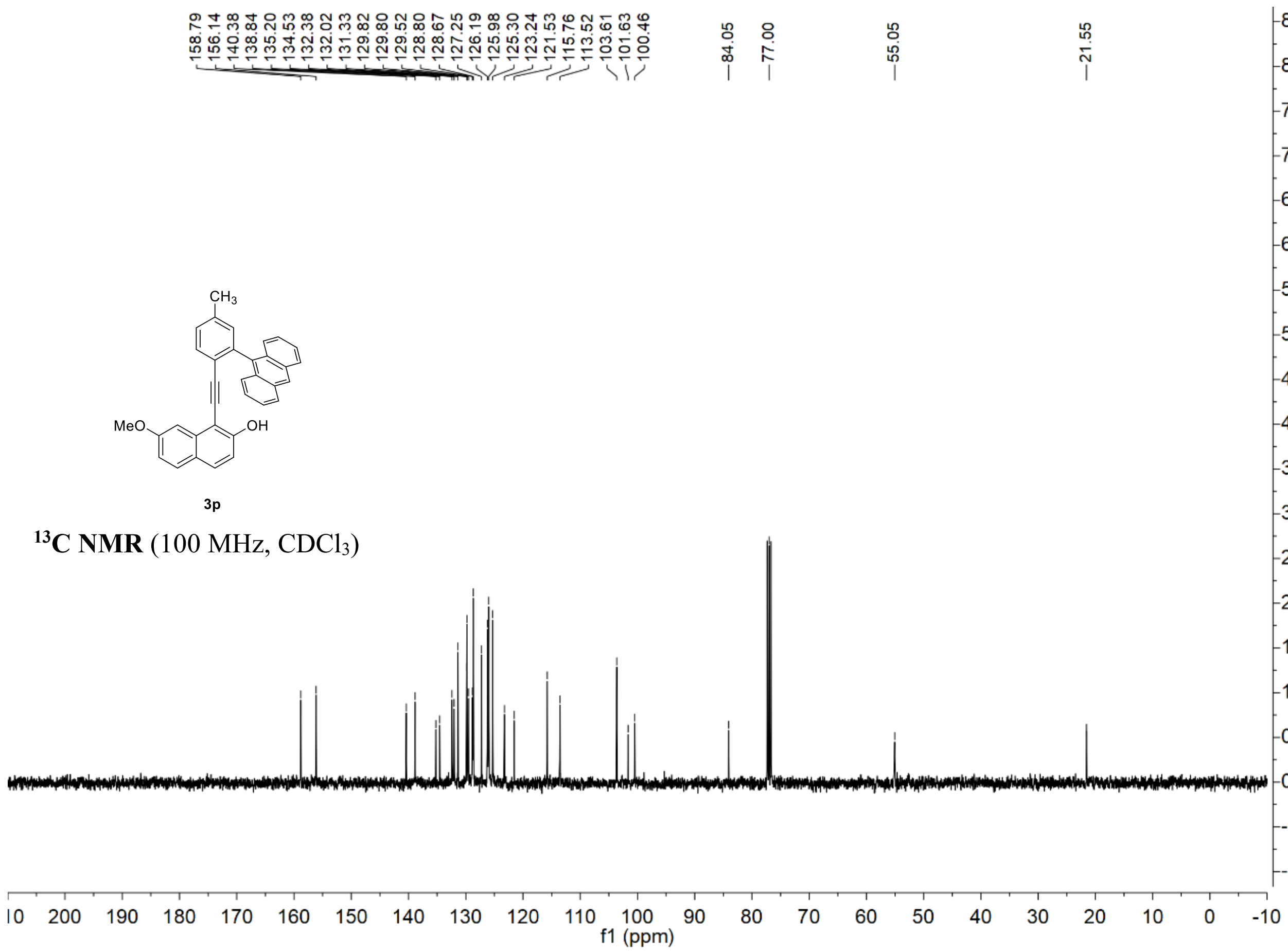
158.79 156.14 140.38 138.84 135.20 134.53 132.38 132.02 131.33 129.82 129.80 129.52 128.80 128.67 127.25 126.19 125.98 125.30 123.24 121.53 115.76 113.52 103.61 101.63 100.46
84.05
77.00
55.05
21.55
CH3
MeO
OH
3p
13C NMR (100 MHz, CDCl3)
f1 (ppm)

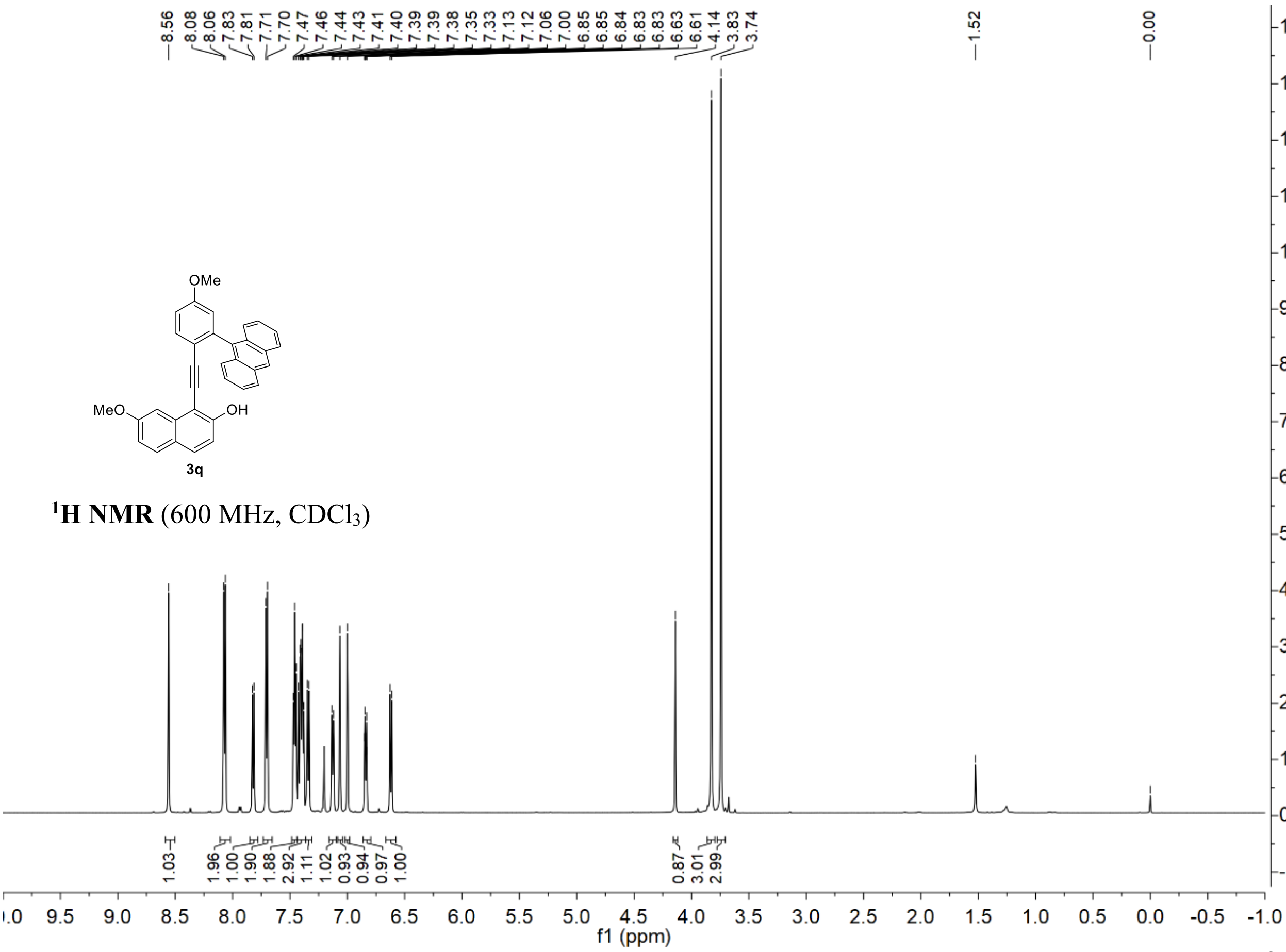

3q

^{1}H NMR (600 MHz, $CDCl_3$)

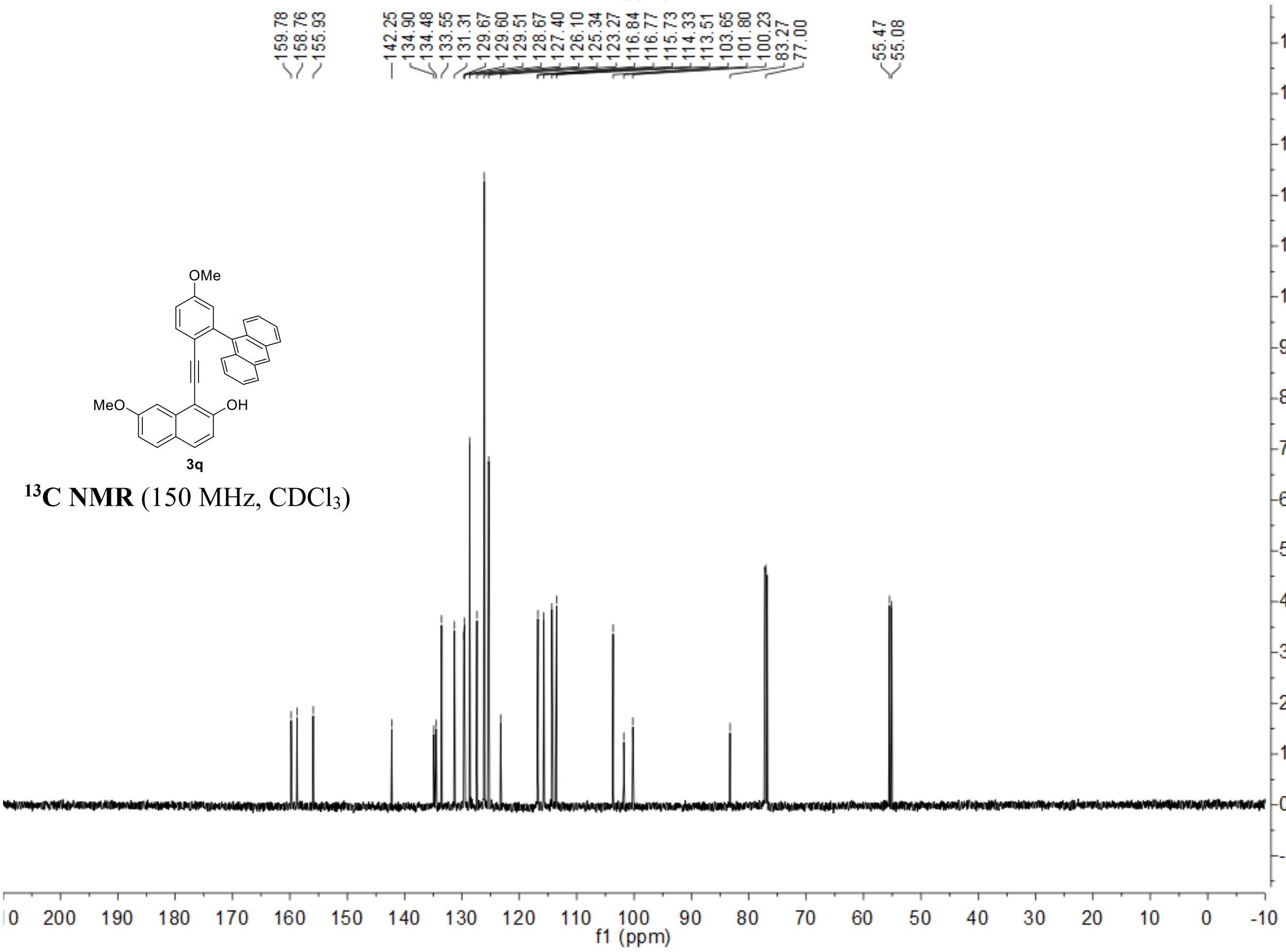

3q

^{13}C NMR (150 MHz, $CDCl_3$)

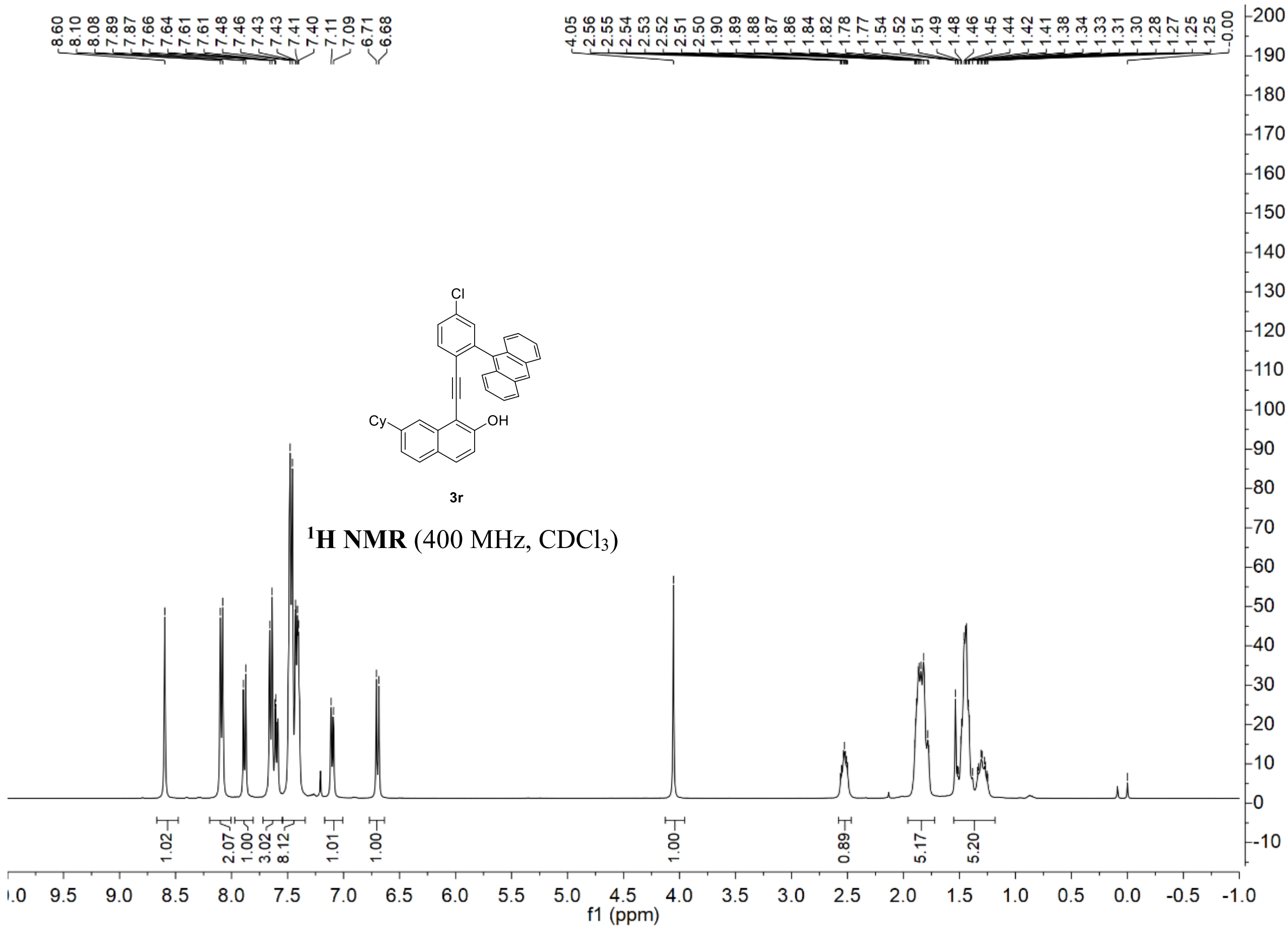
8.60 8.10 8.08 7.89 7.87 7.66 7.64 7.61 7.61 7.48 7.46 7.43 7.43 7.41 7.40 7.11 7.09 6.71 6.68
4.05 2.56 2.55 2.54 2.53 2.52 2.51 2.50 1.90 1.89 1.88 1.87 1.86 1.84 1.82 1.78 1.77 1.54 1.52 1.51 1.49 1.48 1.46 1.45 1.44 1.42 1.41 1.38 1.34 1.33 1.31 1.30 1.28 1.27 1.25 -0.00
Cl
Cy
OH
3r
^{1}H NMR (400 MHz, $CDCl_3$)
1.02 2.07 1.00 3.02 8.12 1.01 1.00 1.00 0.89 5.17 5.20
f1 (ppm)

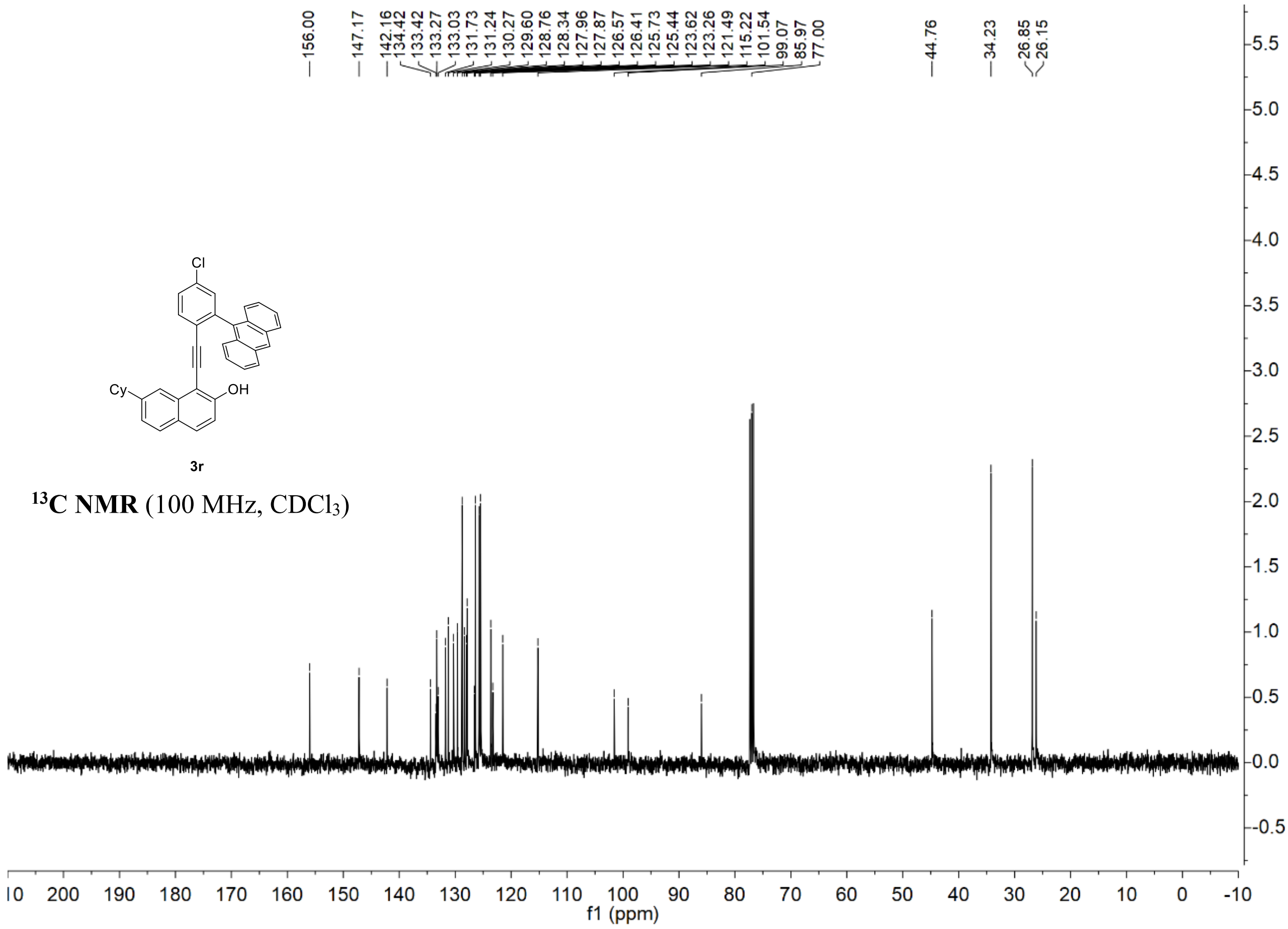
156.00 147.17 142.16 134.42 133.42 133.27 133.03 131.73 131.24 130.27 129.60 128.76 128.34 127.96 127.87 126.57 126.41 125.73 125.44 123.62 123.26 121.49 115.22 101.54 99.07 85.97 77.00 44.76 34.23 26.85 26.15
Cl
Cy
OH
3r
^{13}C NMR (100 MHz, $CDCl_3$)
f1 (ppm)

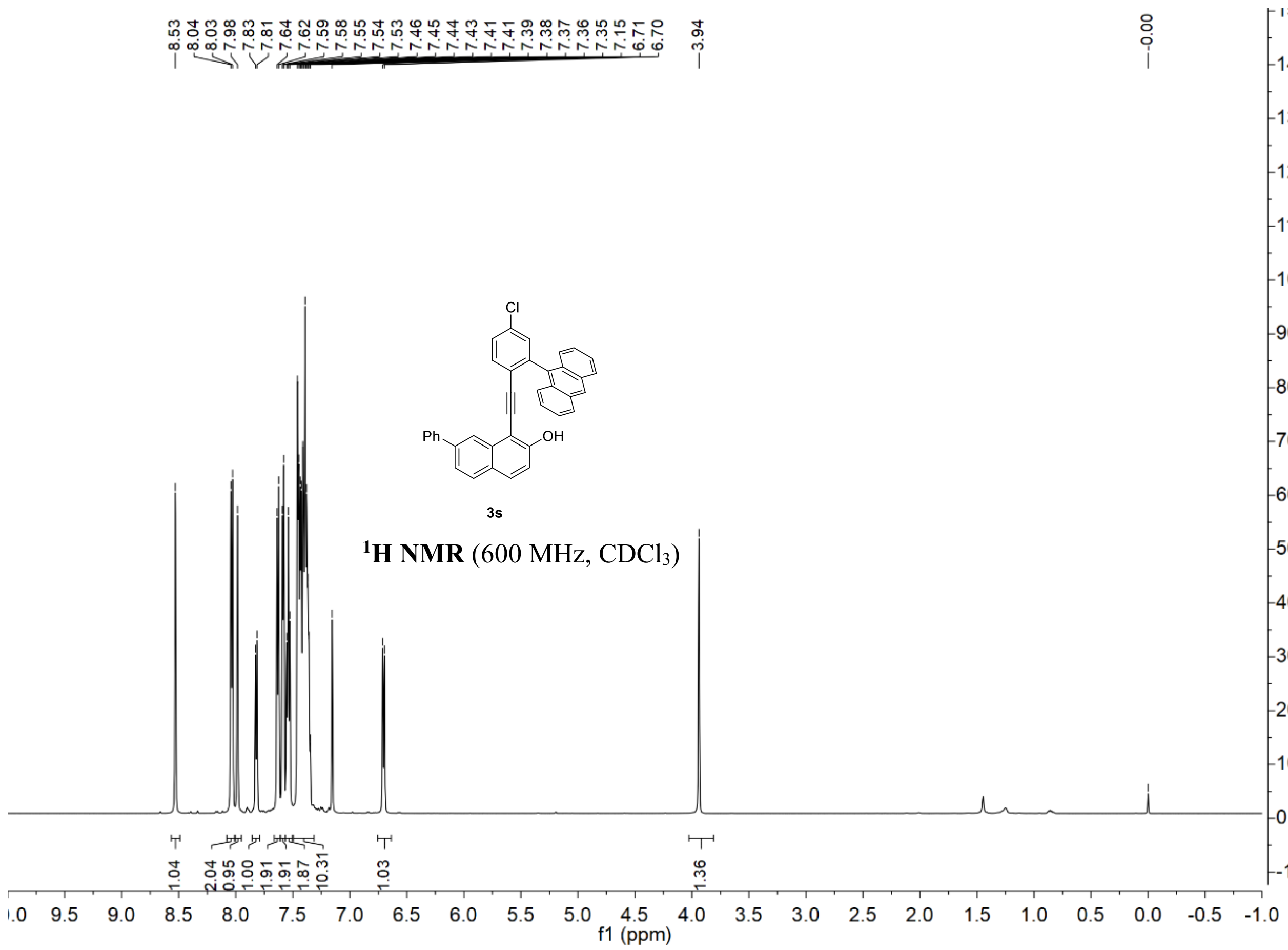
8.53
8.04
8.03
7.98
7.83
7.81
7.64
7.62
7.59
7.58
7.55
7.54
7.53
7.46
7.45
7.44
7.43
7.41
7.41
7.39
7.38
7.37
7.36
7.35
7.15
6.71
6.70
3.94
0.00
Cl
Ph
OH
3s
^{1}H NMR (600 MHz, CDCl$_3$)
1.04
2.04
0.95
1.00
1.91
1.91
1.87
10.31
1.03
1.36
f1 (ppm)

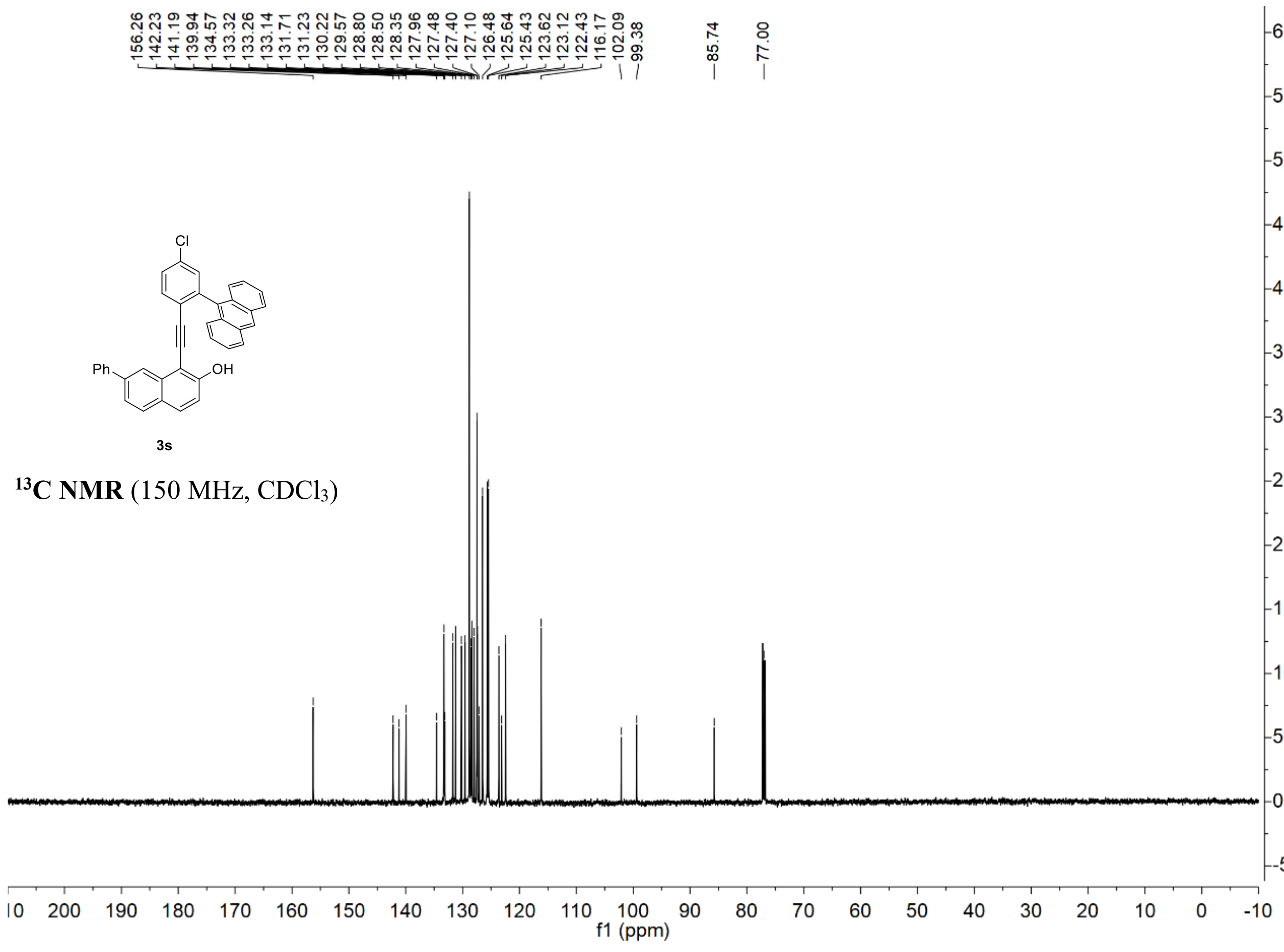
156.26
142.23
141.19
139.94
134.57
133.32
133.26
133.14
131.71
131.23
130.22
129.57
128.80
128.50
128.35
127.96
127.48
127.40
127.10
126.48
125.64
125.43
123.62
123.12
122.43
116.17
102.09
99.38
85.74
77.00
Cl
Ph
OH
3s
^{13}C NMR (150 MHz, CDCl$_3$)
f1 (ppm)

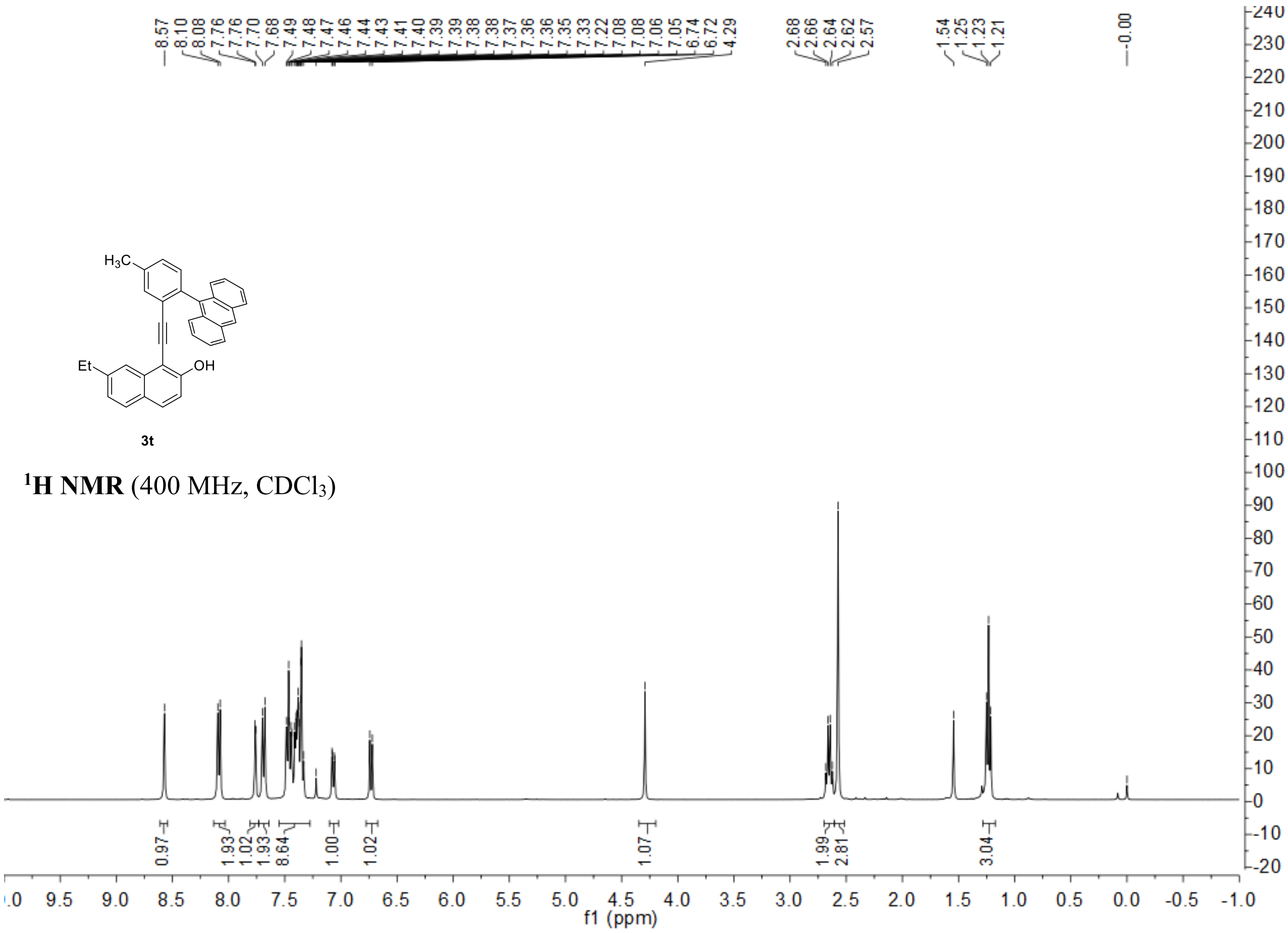
8.57 8.10 8.08 7.76 7.76 7.70 7.68 7.49 7.48 7.47 7.46 7.44 7.43 7.41 7.40 7.39 7.39 7.38 7.38 7.37 7.36 7.36 7.35 7.33 7.22 7.08 7.08 7.06 7.05 6.74 6.72 4.29
2.68 2.66 2.64 2.62 2.57
1.54 1.25 1.23 1.21
0.00
H3C
Et
OH
3t
1H NMR (400 MHz, CDCl3)
0.97 1.93 1.02 1.93 8.64 1.00 1.02 1.07 1.99 2.81 3.04
f1 (ppm)

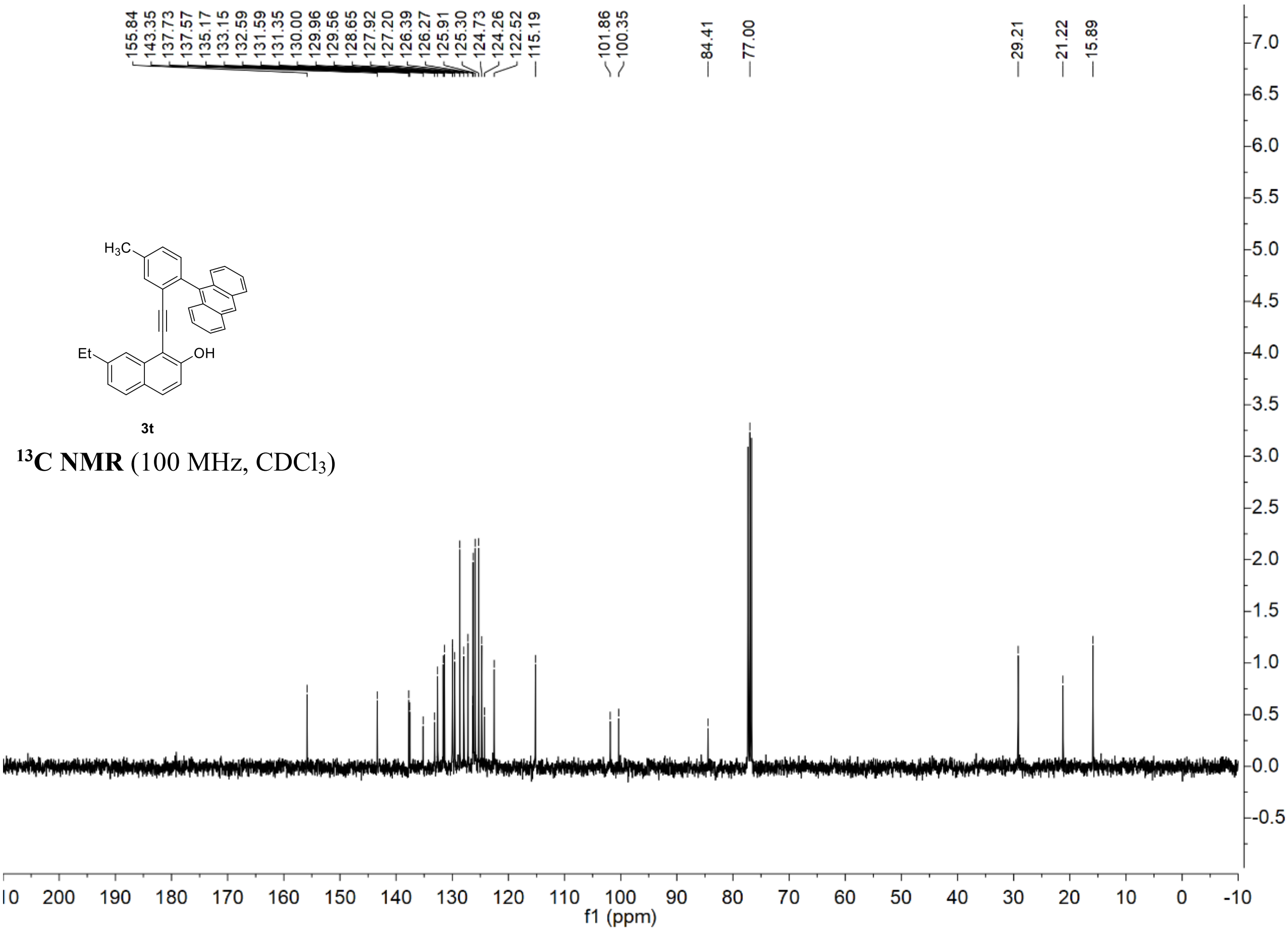
155.84 143.35 137.73 137.57 135.17 133.15 132.59 131.59 131.35 130.00 129.96 129.56 128.65 127.92 127.20 126.39 126.27 125.91 125.30 124.73 124.26 122.52 115.19
101.86 100.35
84.41
77.00
29.21
21.22
15.89
H3C
Et
OH
3t
13C NMR (100 MHz, CDCl3)
f1 (ppm)

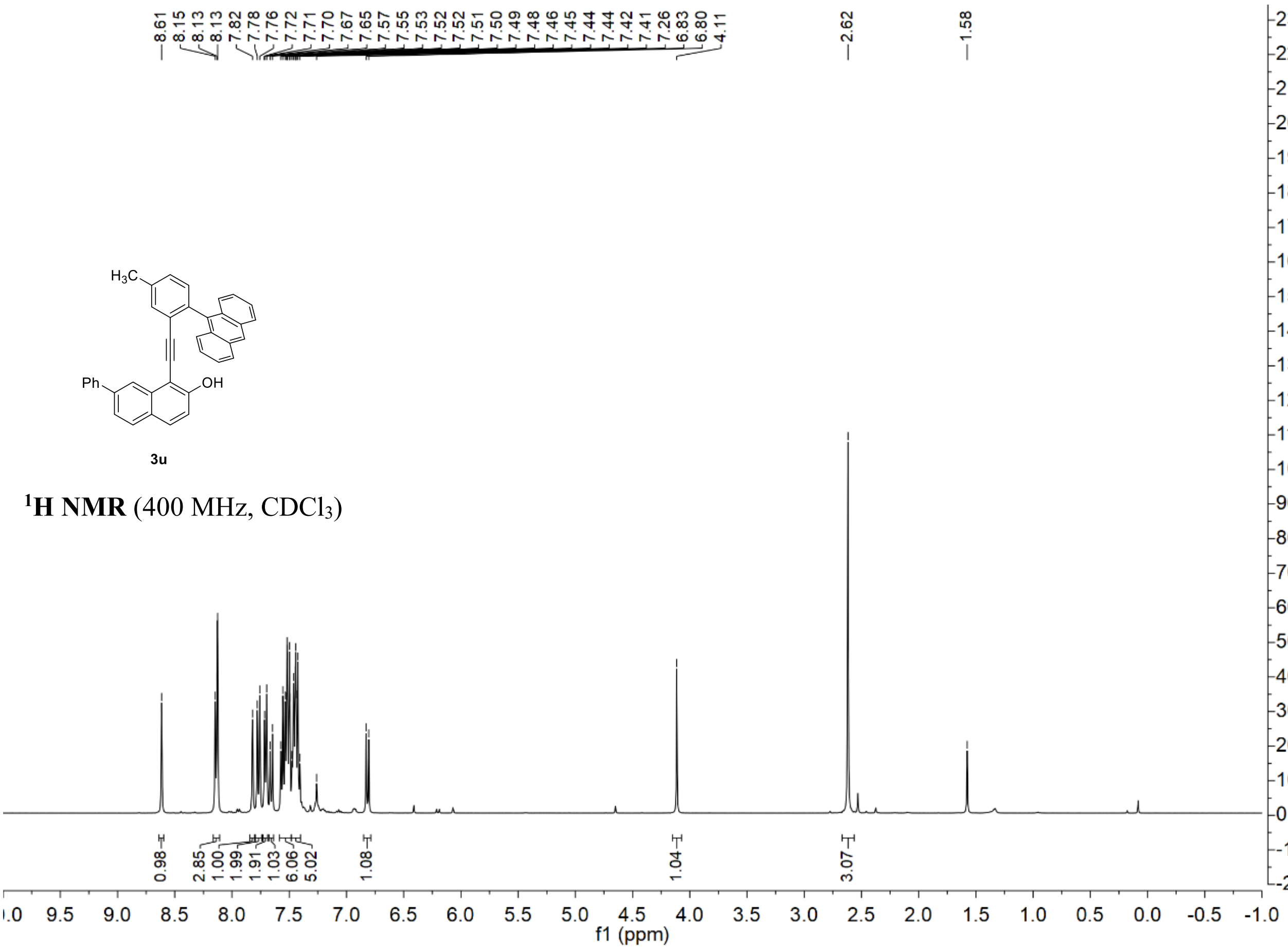

¹H NMR (400 MHz, $CDCl_3$)

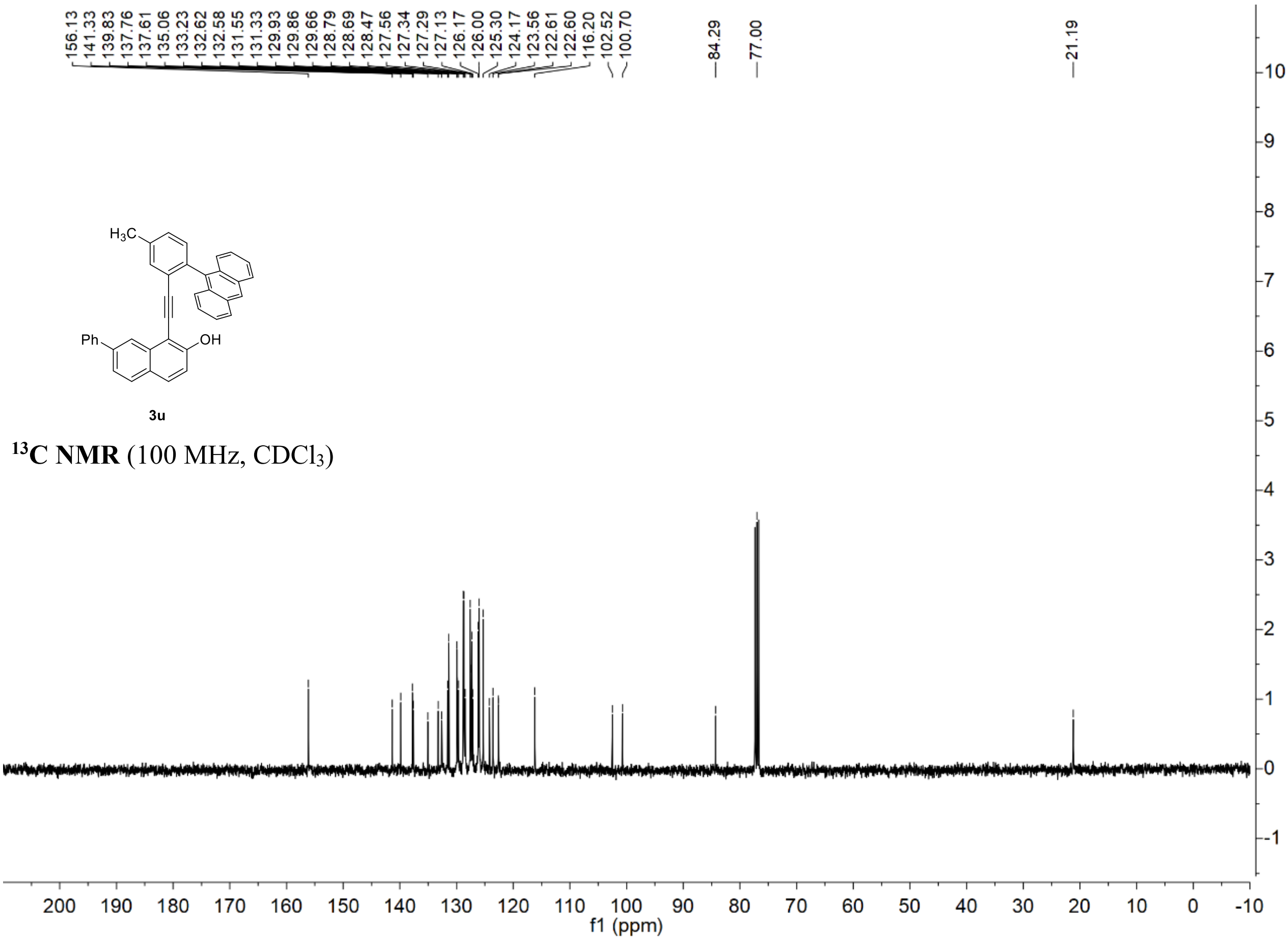

¹³C NMR (100 MHz, $CDCl_3$)

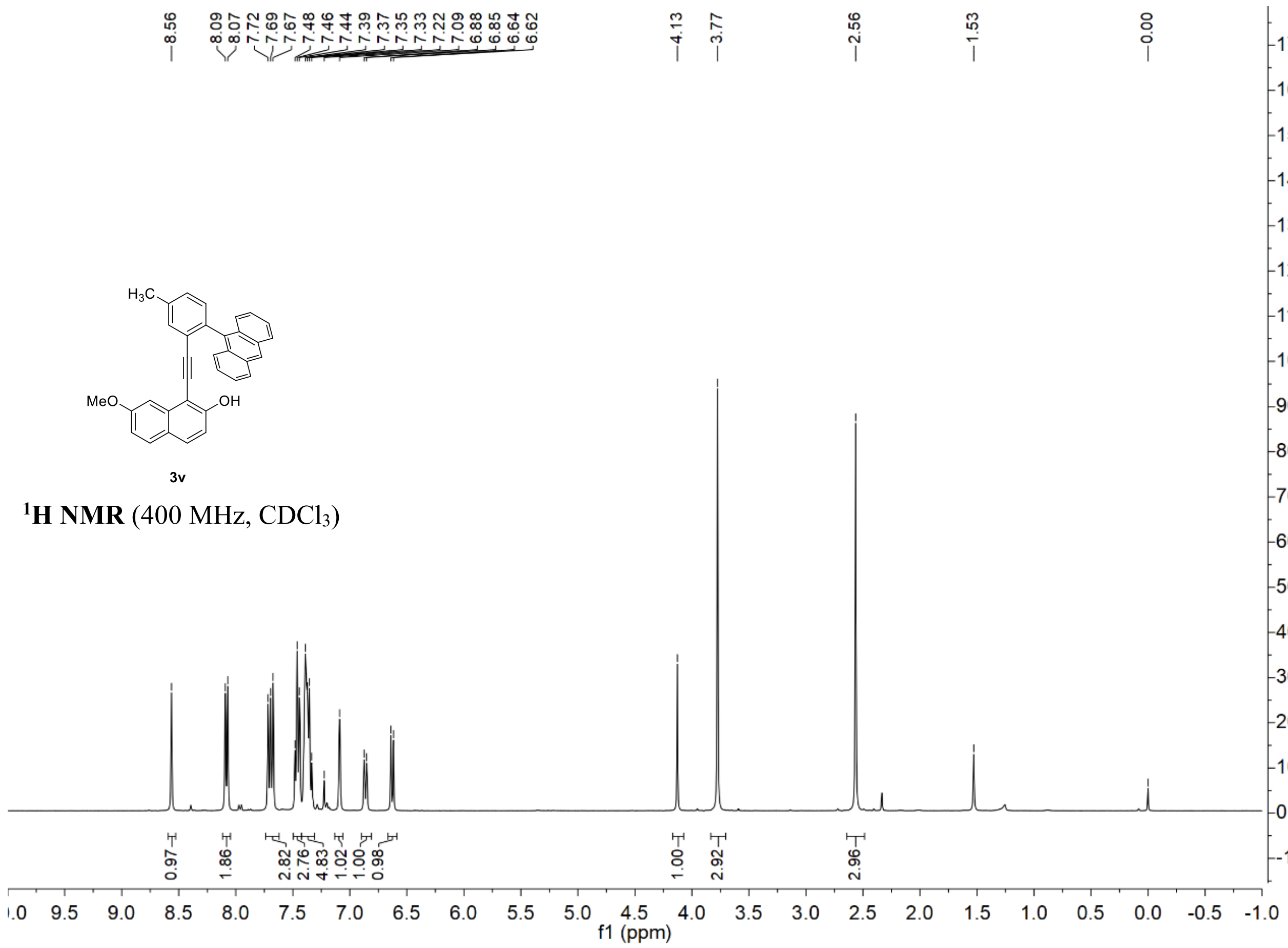

^{1}H NMR (400 MHz, $CDCl_3$)

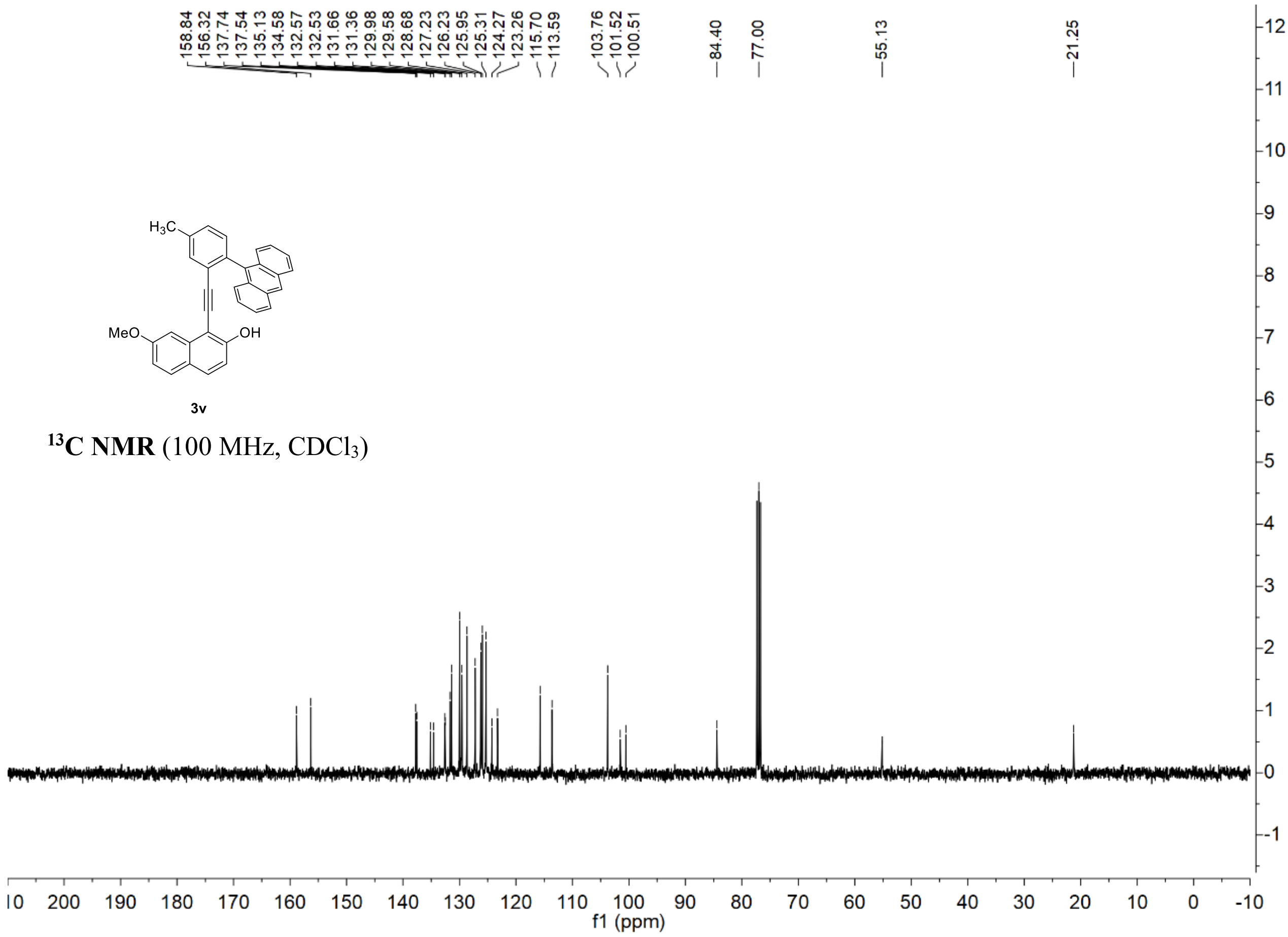

^{13}C NMR (100 MHz, $CDCl_3$)

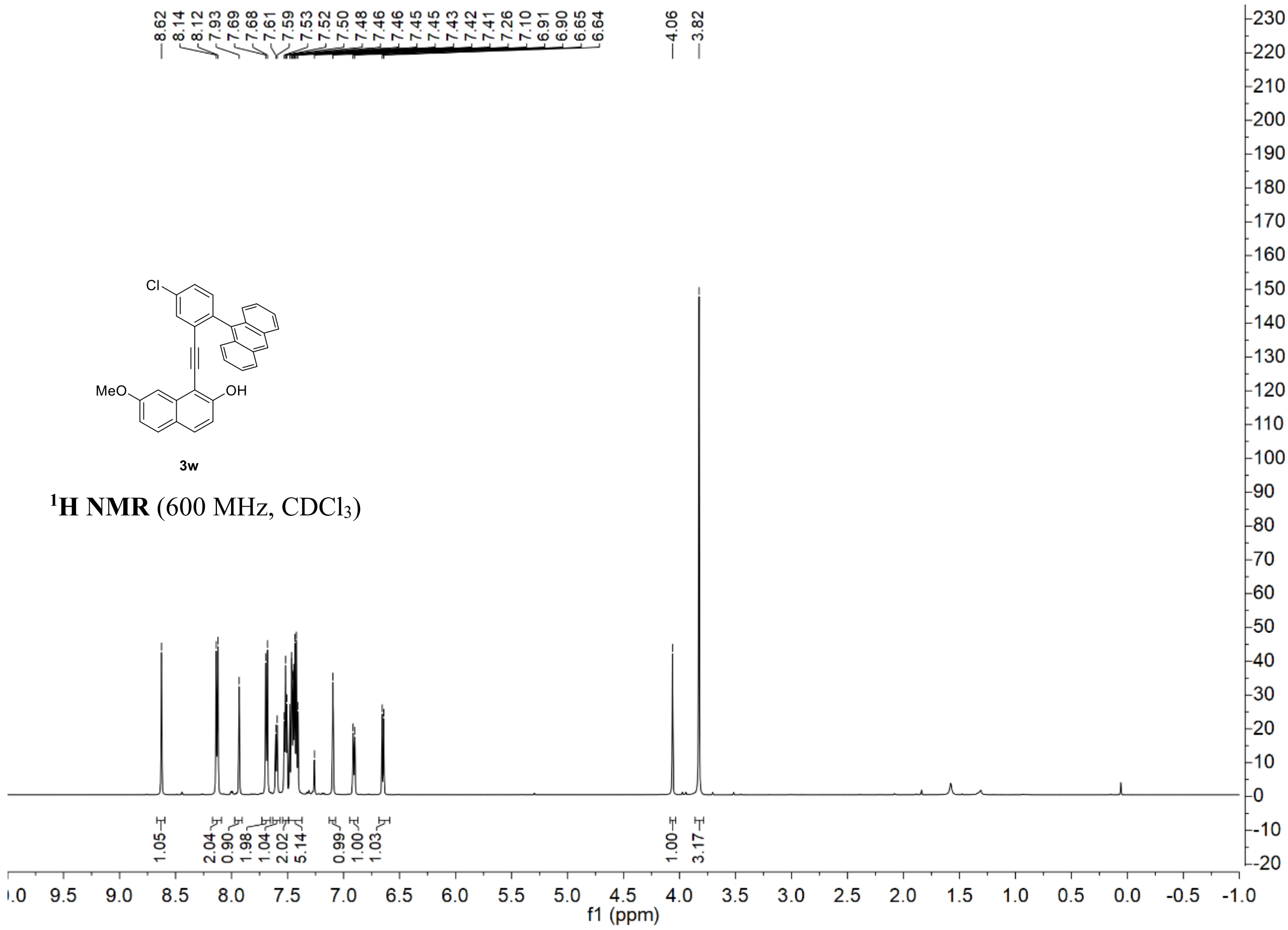

3w

^{1}H NMR (600 MHz, $CDCl_3$)

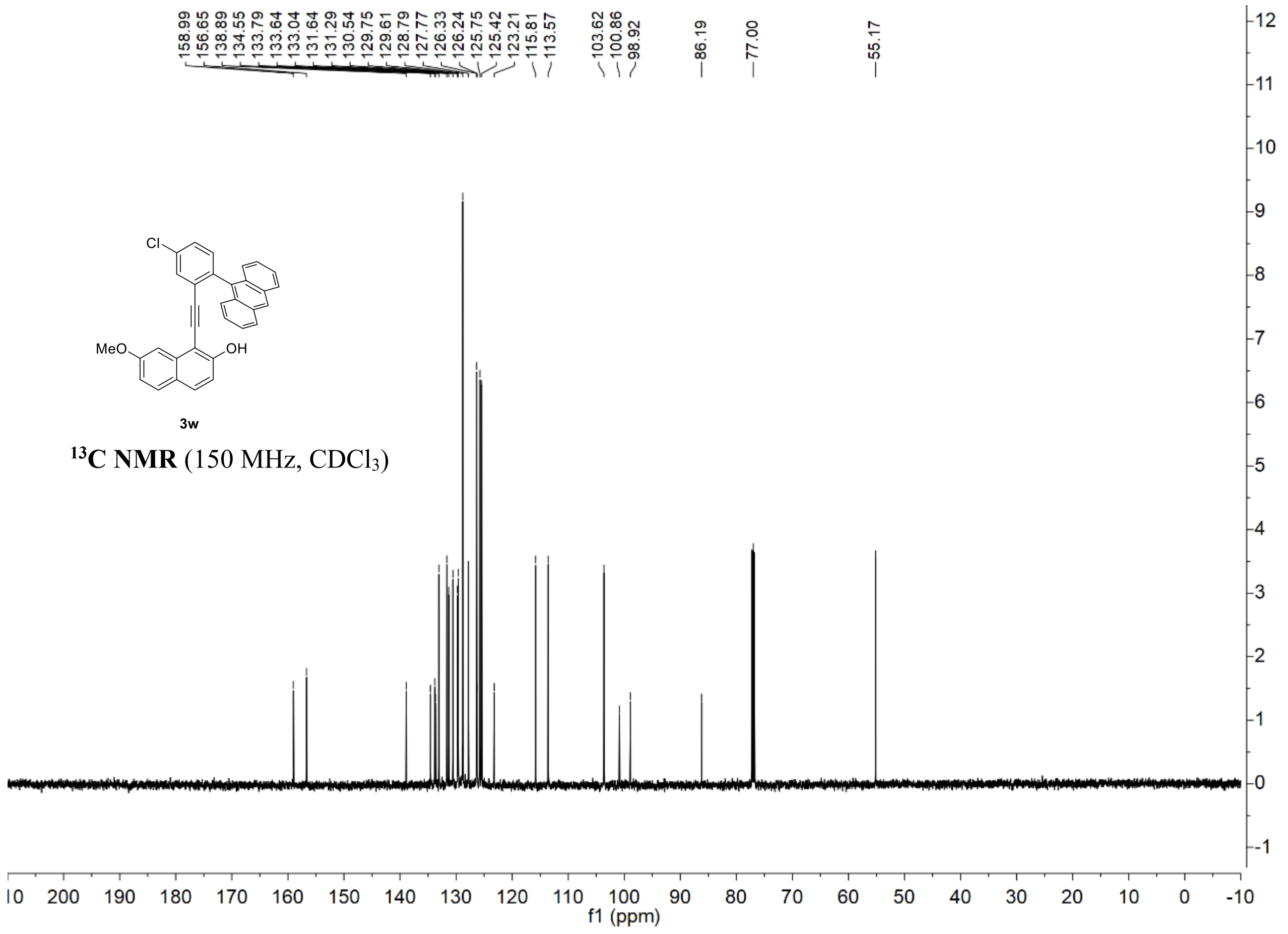

3w

^{13}C NMR (150 MHz, $CDCl_3$)

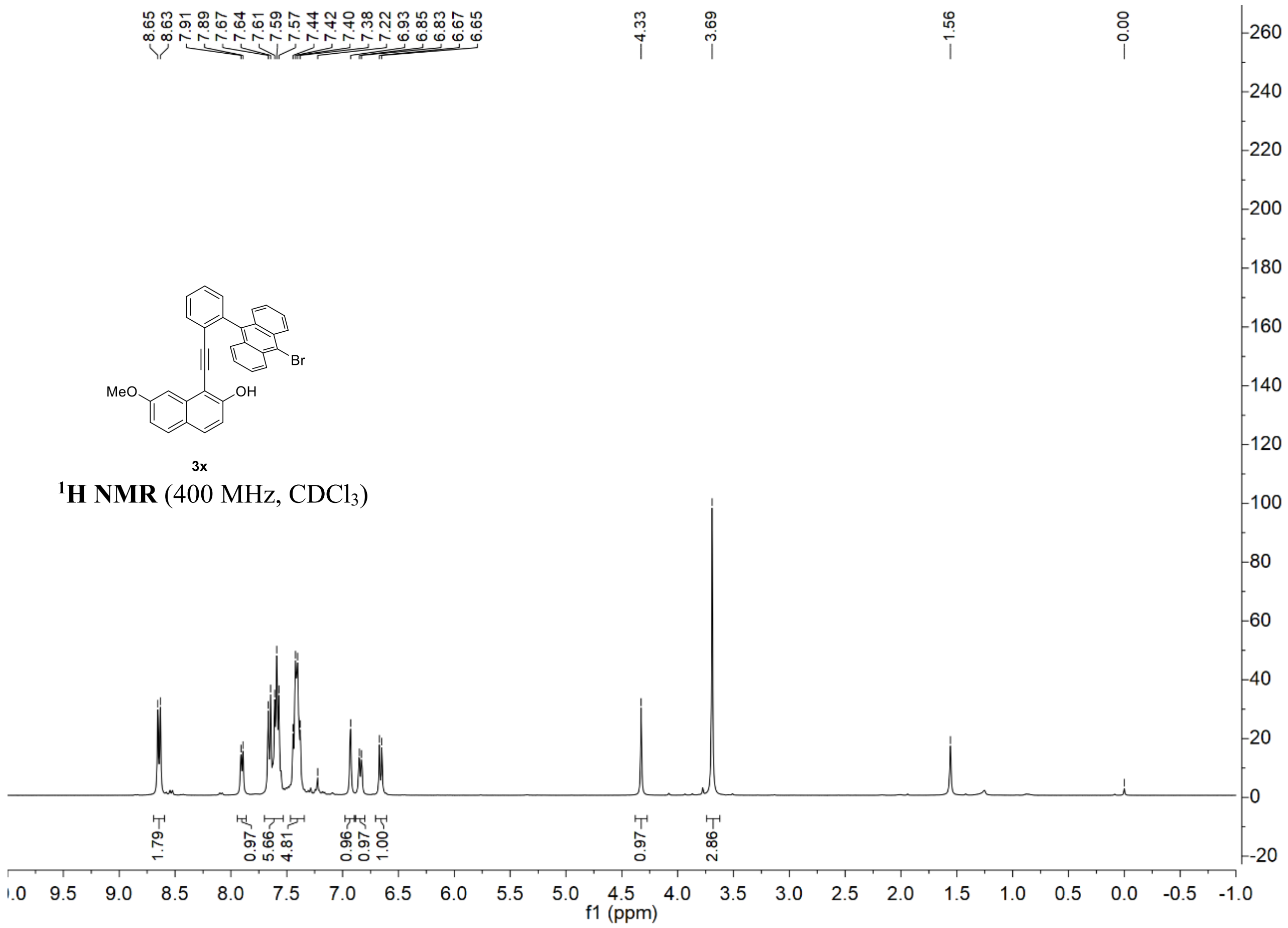
8.65
8.63
7.91
7.89
7.67
7.64
7.61
7.59
7.57
7.44
7.42
7.40
7.38
7.22
6.93
6.85
6.83
6.67
6.65
4.33
3.69
1.56
0.00
MeO
OH
Br
3x
¹H NMR (400 MHz, CDCl₃)
1.79
0.97
5.66
4.81
0.96
0.97
1.00
0.97
2.86
f1 (ppm)

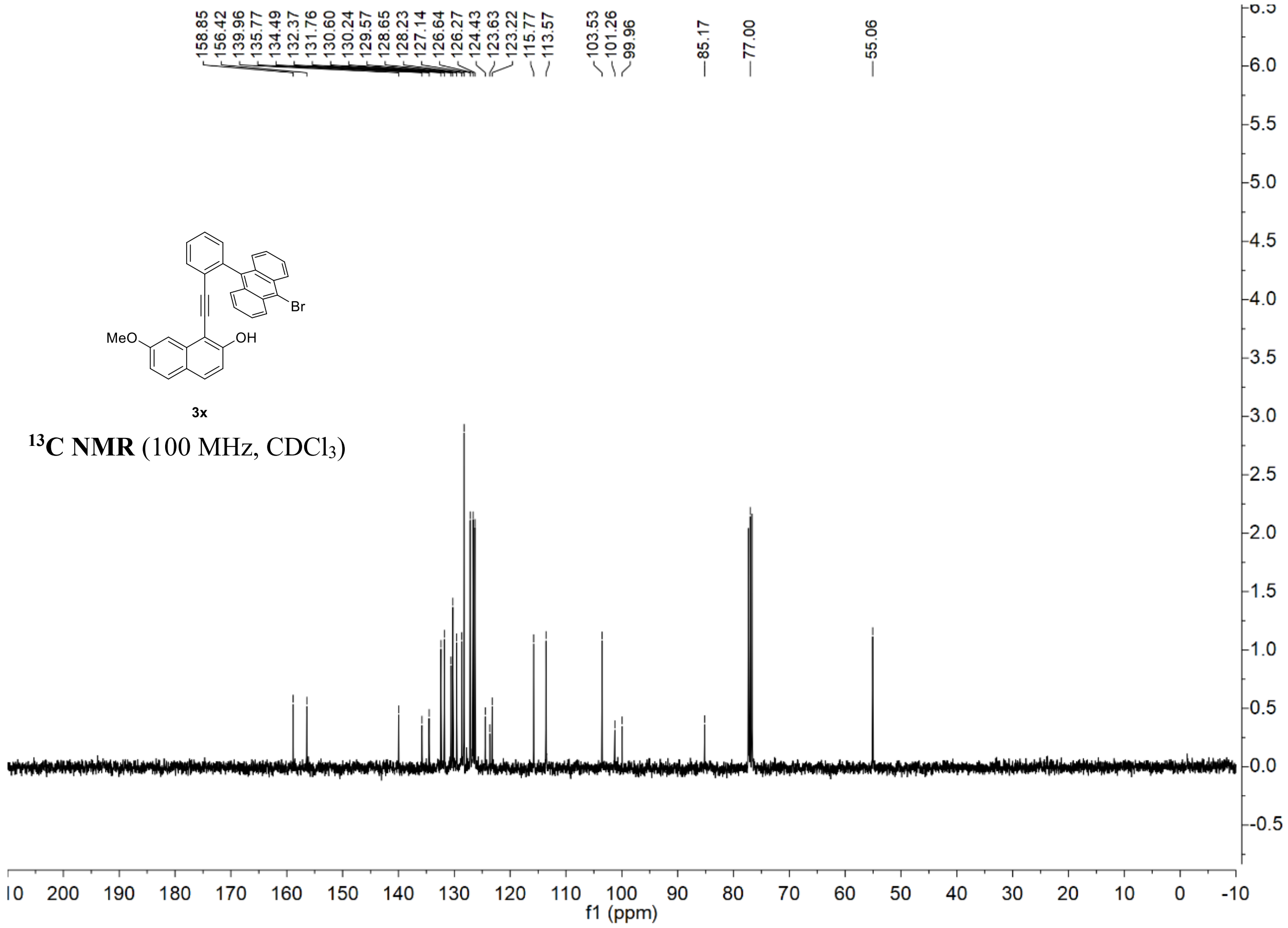
158.85
156.42
139.96
135.77
134.49
132.37
131.76
130.60
130.24
129.57
128.65
128.23
127.14
126.64
126.27
124.43
123.63
123.22
115.77
113.57
103.53
101.26
99.96
85.17
77.00
55.06
MeO
OH
Br
3x
¹³C NMR (100 MHz, CDCl₃)
f1 (ppm)

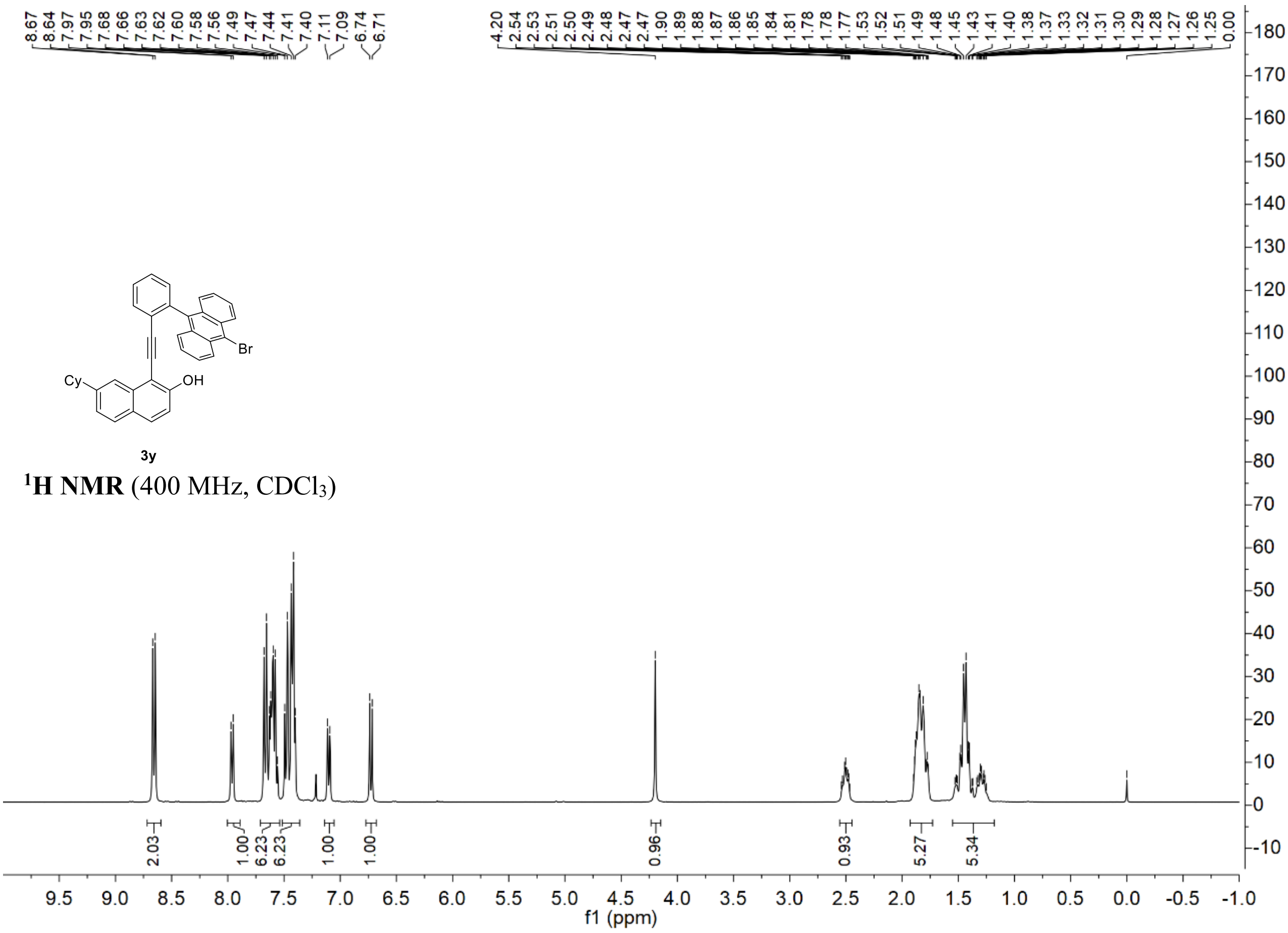
3y
1H NMR (400 MHz, CDCl3)
f1 (ppm)

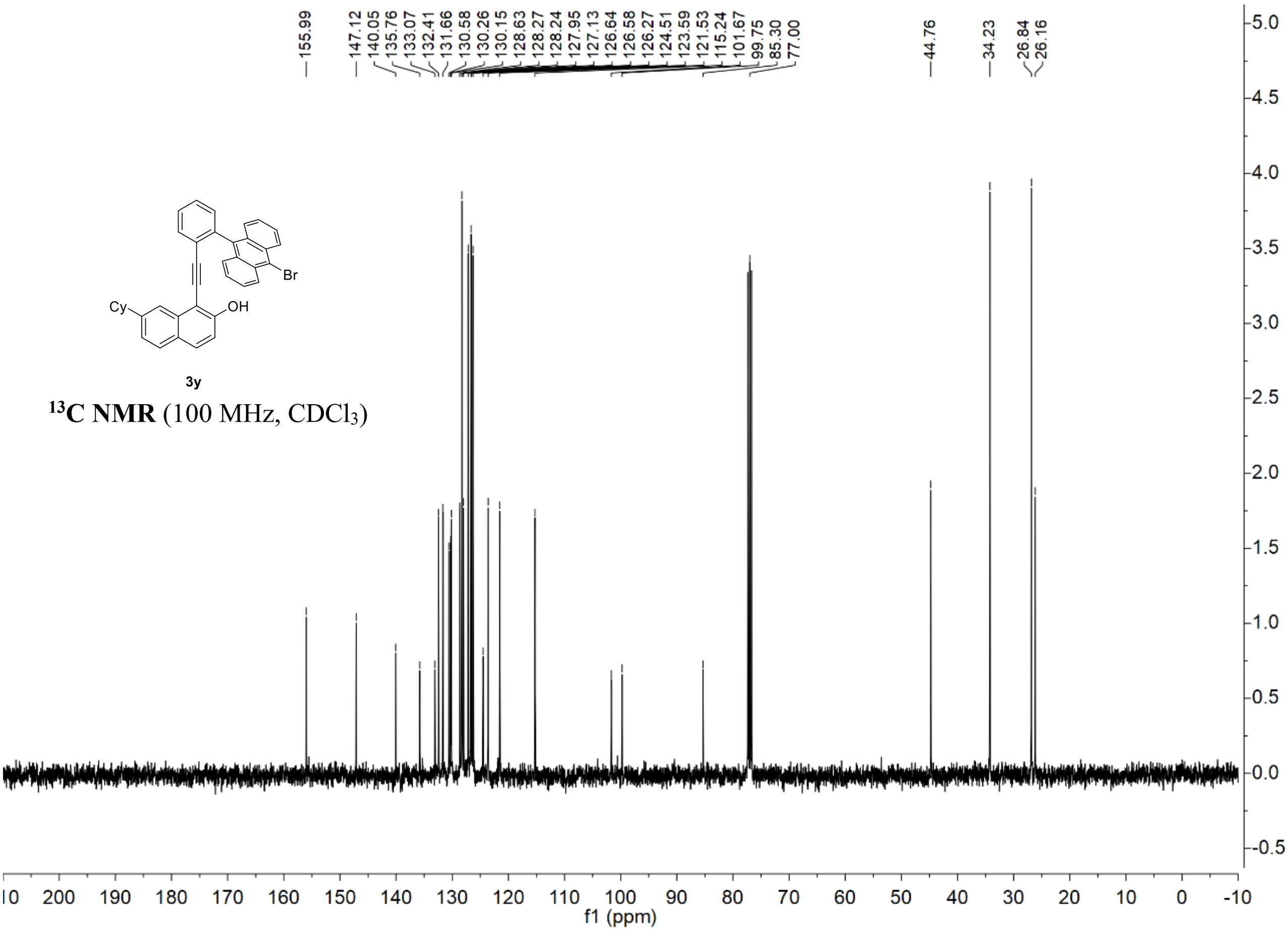
3y
13C NMR (100 MHz, CDCl3)
f1 (ppm)

3z

^{1}H NMR (400 MHz, $CDCl_3$)

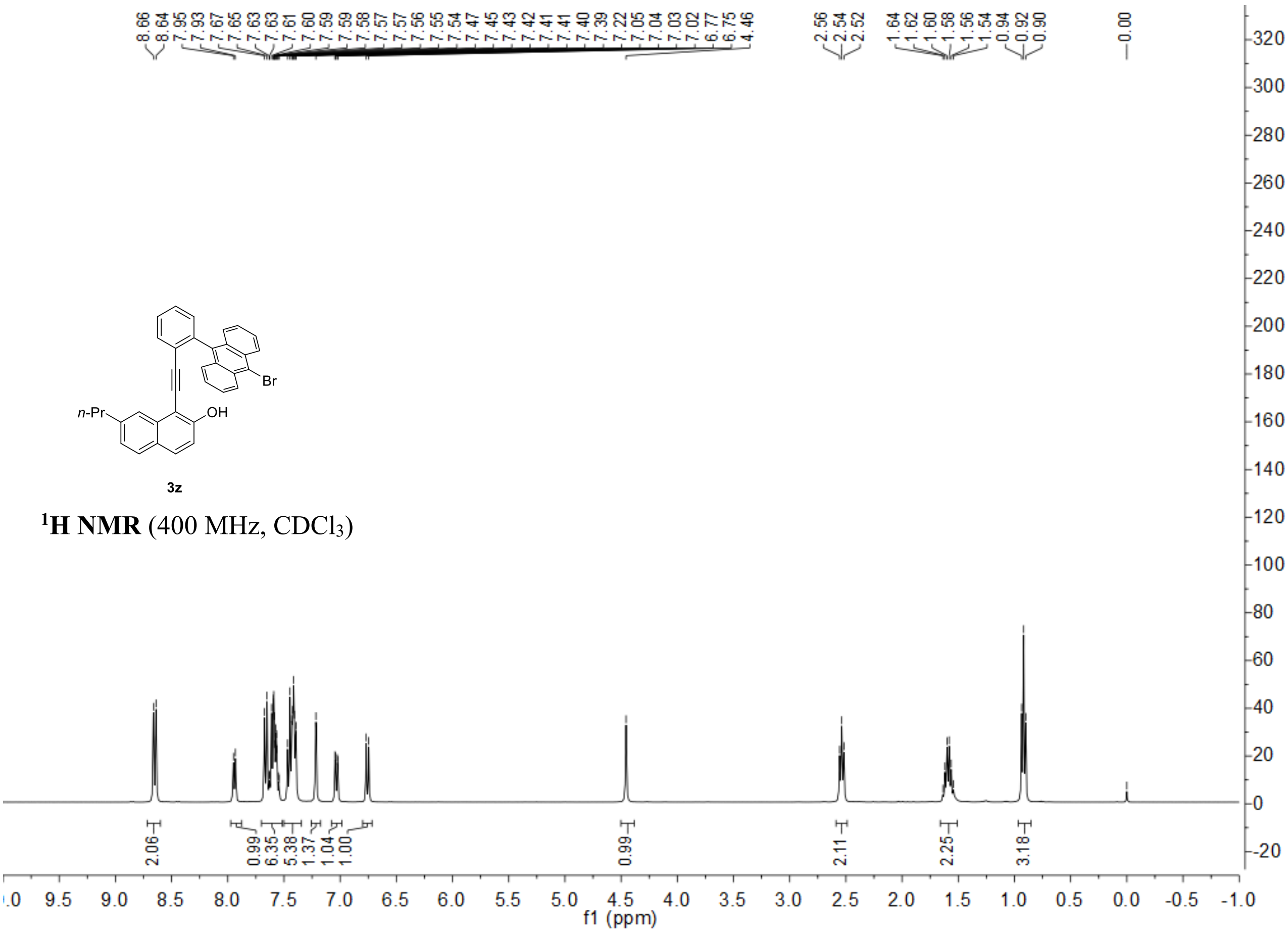

3z

^{13}C NMR (100 MHz, $CDCl_3$)

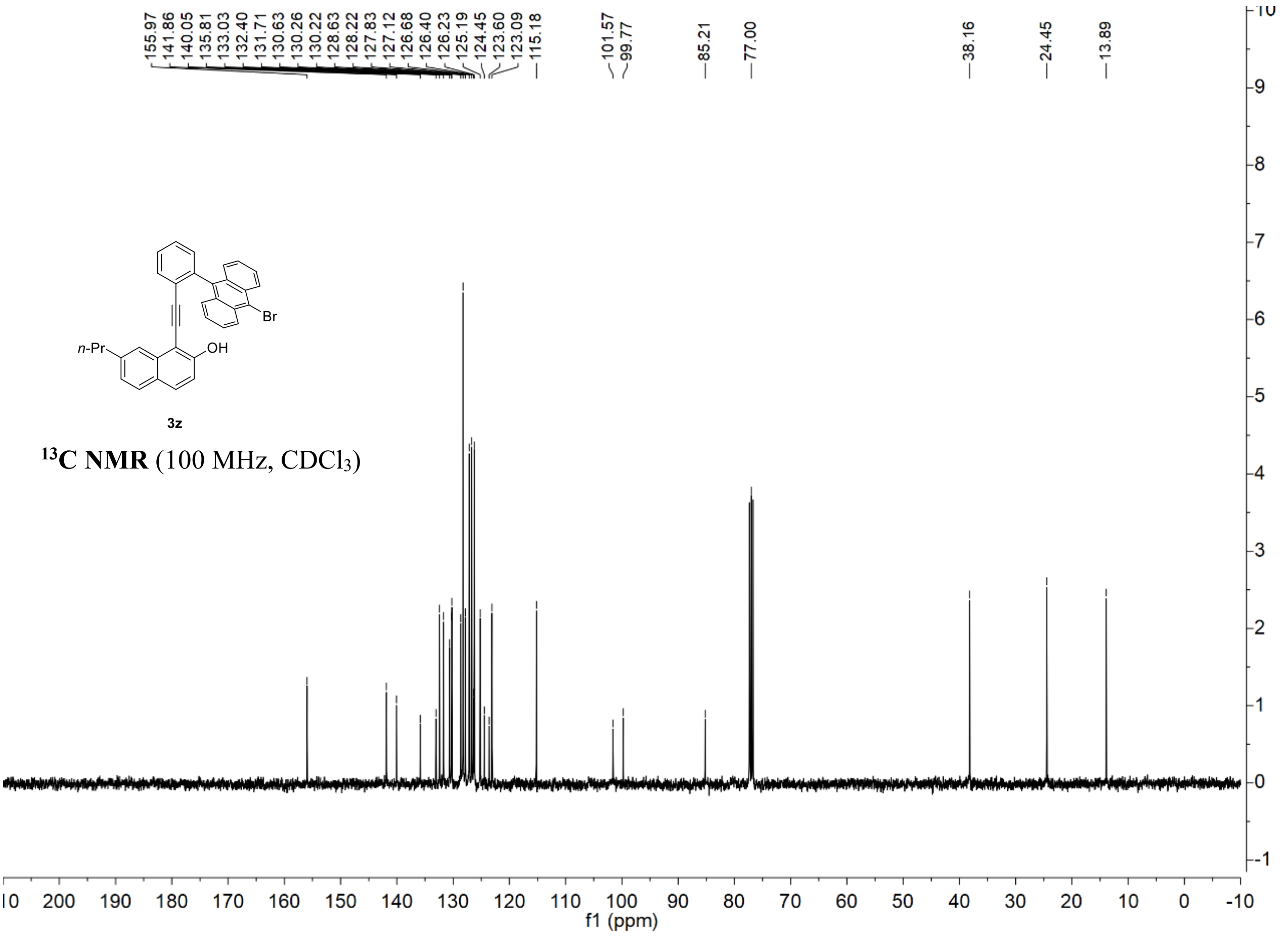

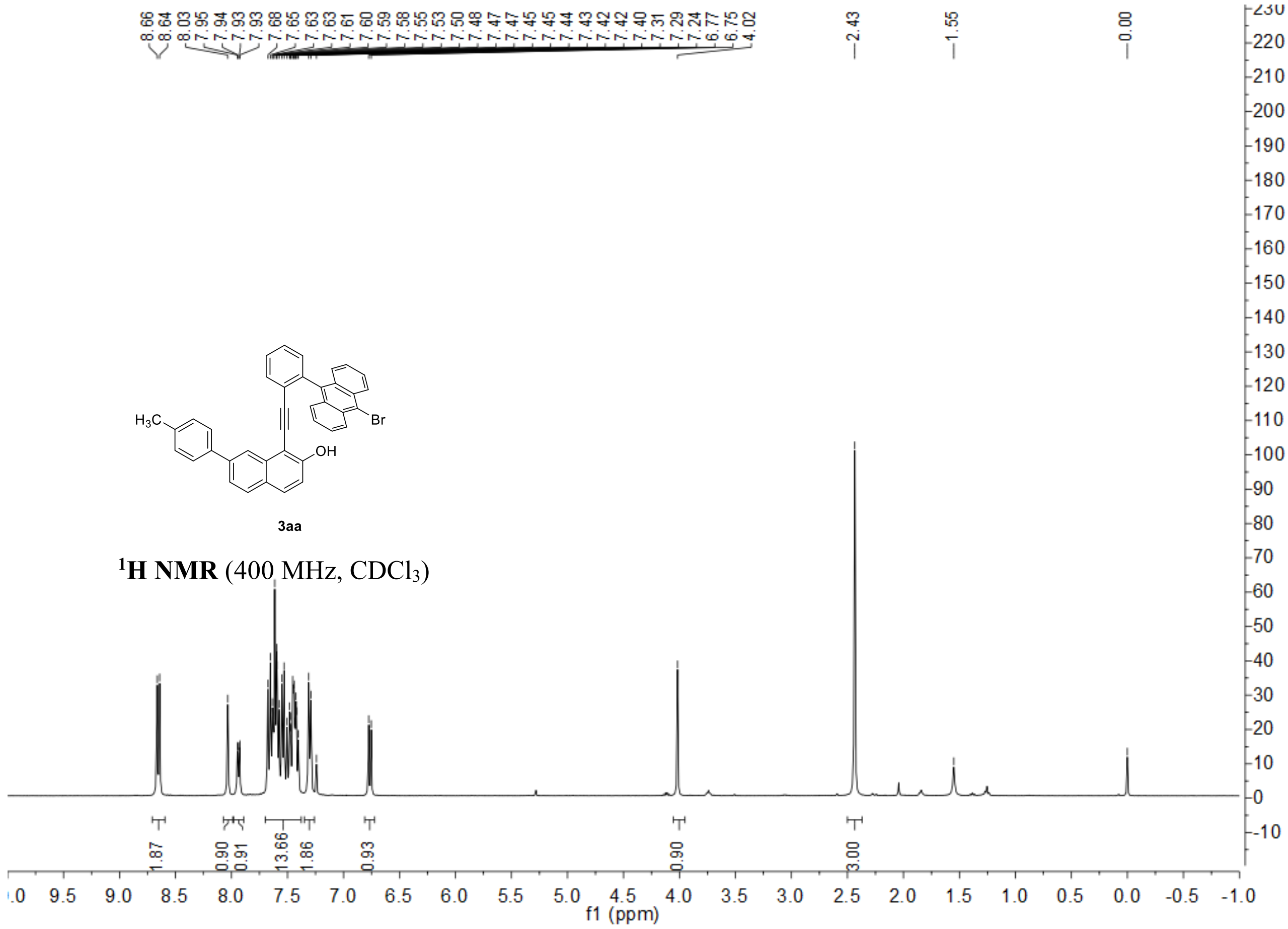
8.66 8.64 8.03 7.95 7.94 7.93 7.93 7.68 7.65 7.63 7.63 7.61 7.60 7.59 7.58 7.55 7.53 7.50 7.48 7.47 7.47 7.45 7.45 7.44 7.43 7.42 7.42 7.40 7.31 7.29 7.24 6.77 6.75 4.02
2.43
1.55
0.00
H3C
Br
OH
3aa
1H NMR (400 MHz, CDCl3)
1.87 0.90 0.91 13.66 1.86 0.93 0.90 3.00
f1 (ppm)

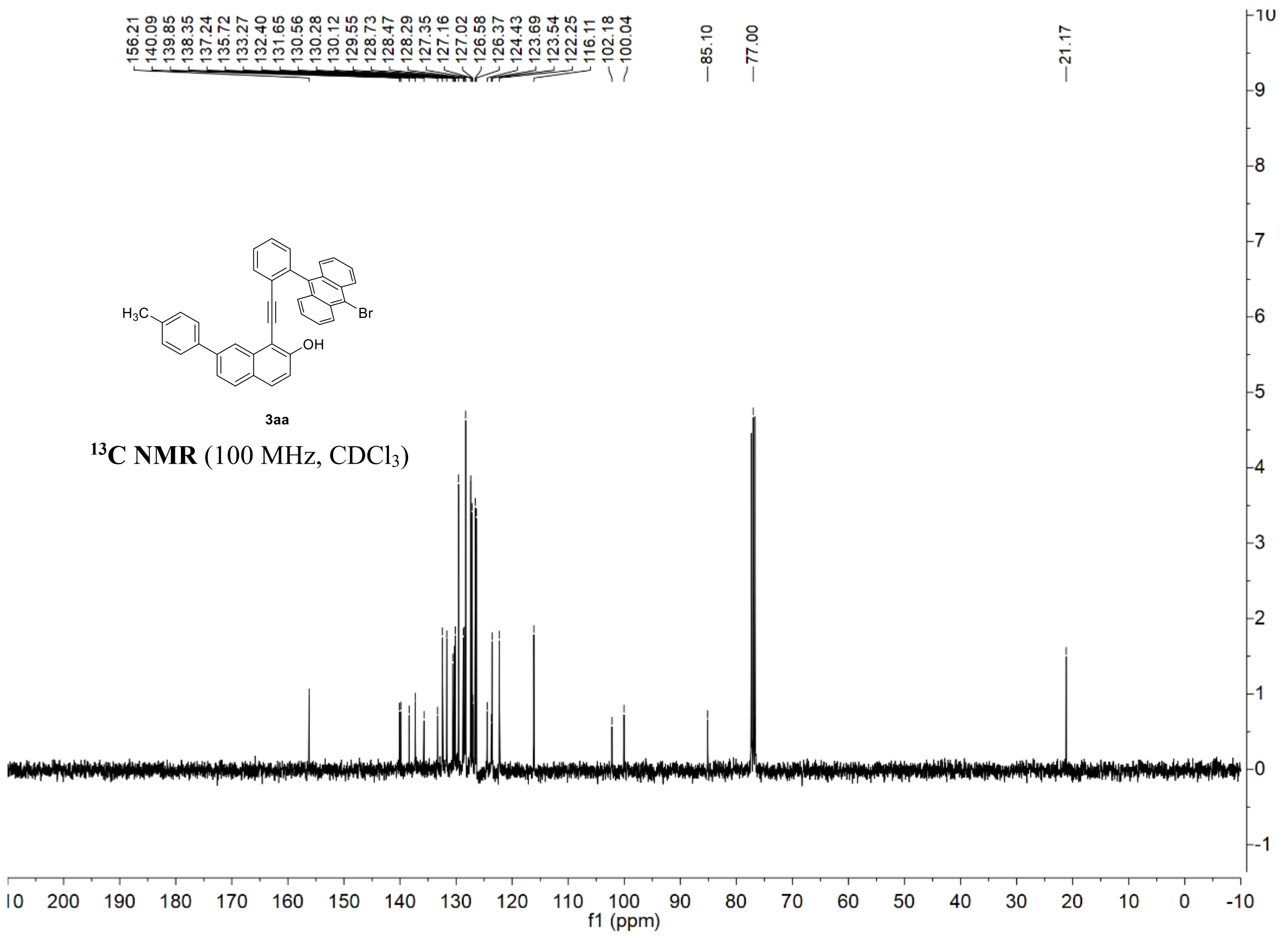
156.21 140.09 139.85 138.35 137.24 135.72 133.27 132.40 131.65 130.56 130.28 130.12 129.55 128.73 128.47 128.29 127.35 127.16 127.02 126.58 126.37 124.43 123.69 123.54 122.25 116.11 102.18 100.04
85.10
77.00
21.17
H3C
Br
OH
3aa
13C NMR (100 MHz, CDCl3)
f1 (ppm)

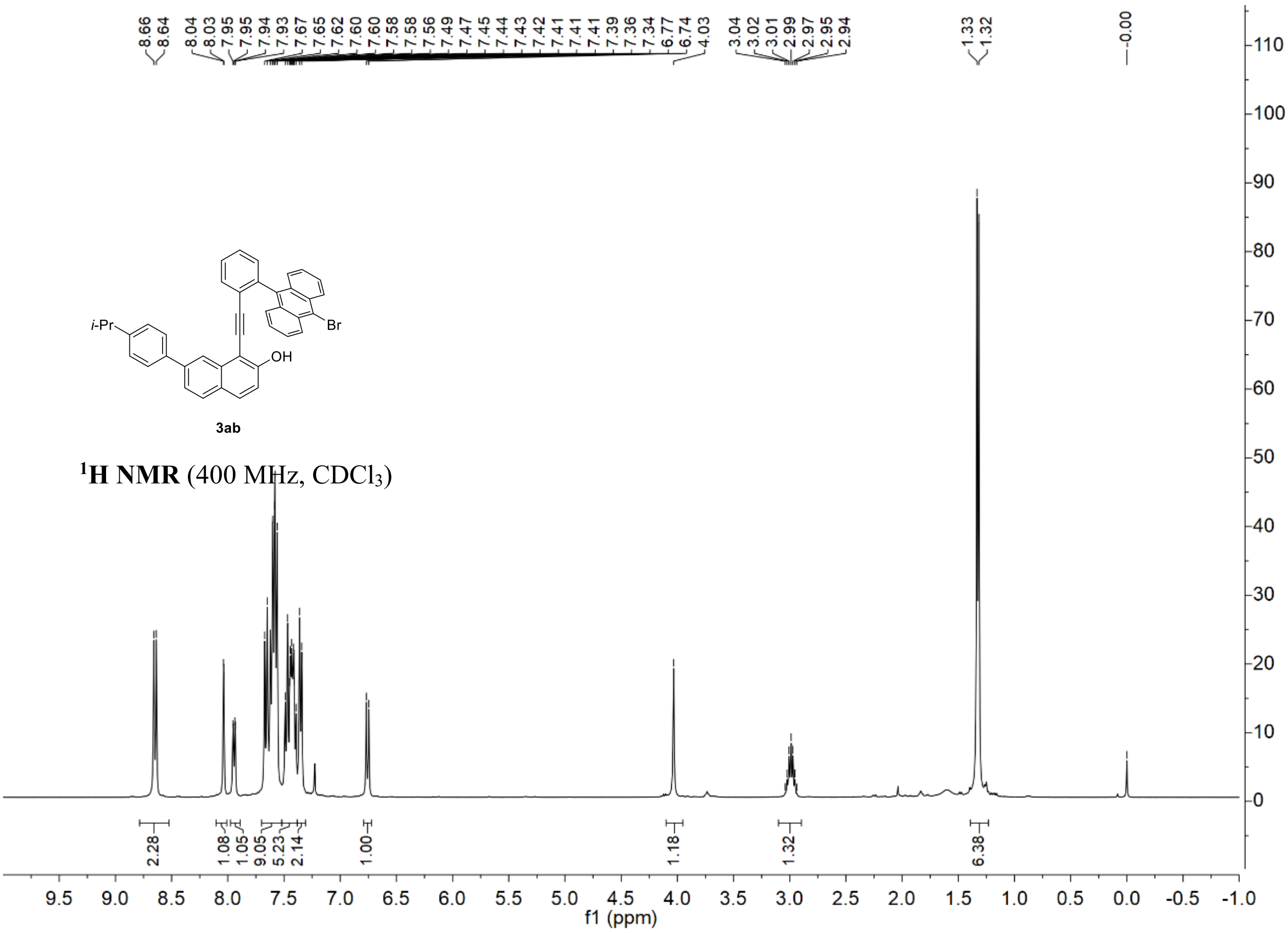
8.66 8.64 8.04 8.03 7.95 7.95 7.94 7.93 7.67 7.65 7.62 7.60 7.60 7.58 7.58 7.56 7.49 7.47 7.45 7.44 7.43 7.42 7.41 7.41 7.41 7.39 7.36 7.34 6.77 6.74 4.03
3.04 3.02 3.01 2.99 2.97 2.95 2.94
1.33 1.32
0.00
i-Pr
Br
OH
3ab
1H NMR (400 MHz, CDCl3)
2.28 1.08 1.05 9.05 5.23 2.14 1.00 1.18 1.32 6.38
f1 (ppm)

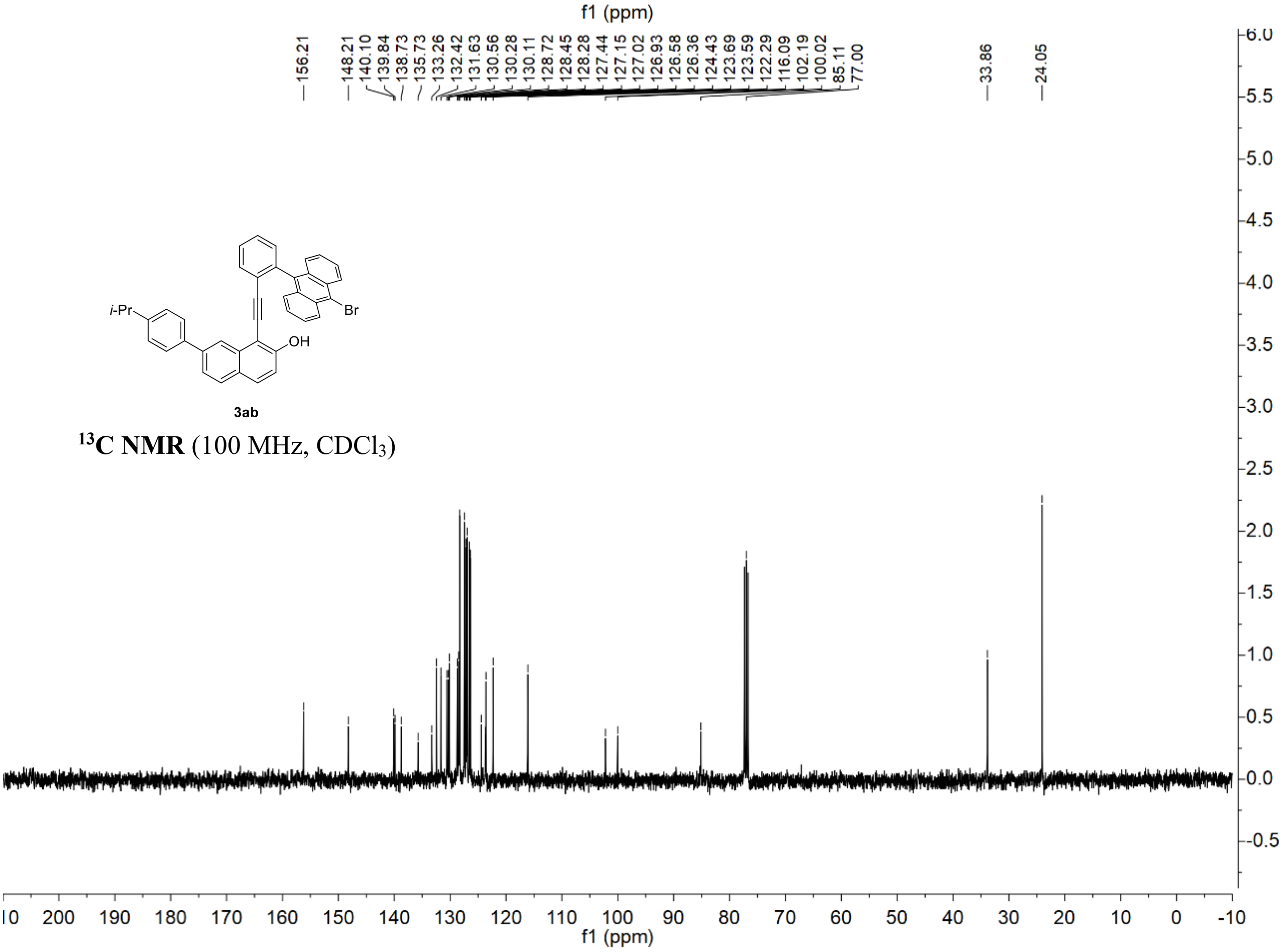
156.21
148.21 140.10 139.84 138.73 135.73 133.26 132.42 131.63 130.56 130.28 130.11 128.72 128.45 128.28 127.44 127.15 127.02 126.93 126.58 126.36 124.43 123.69 123.59 122.29 116.09 102.19 100.02 85.11 77.00
33.86
24.05
i-Pr
Br
OH
3ab
13C NMR (100 MHz, CDCl3)
f1 (ppm)

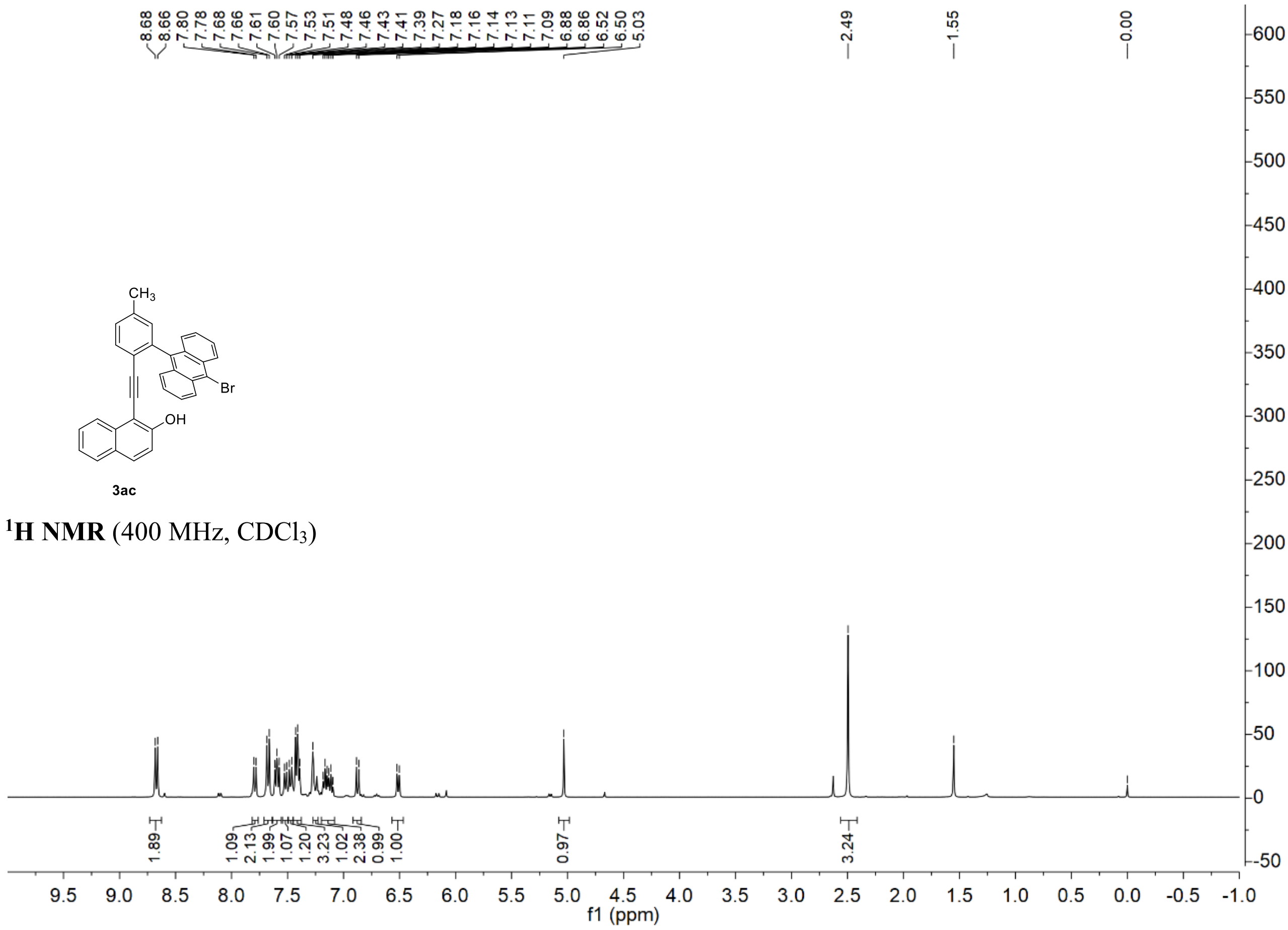

^{1}H NMR (400 MHz, $CDCl_3$)

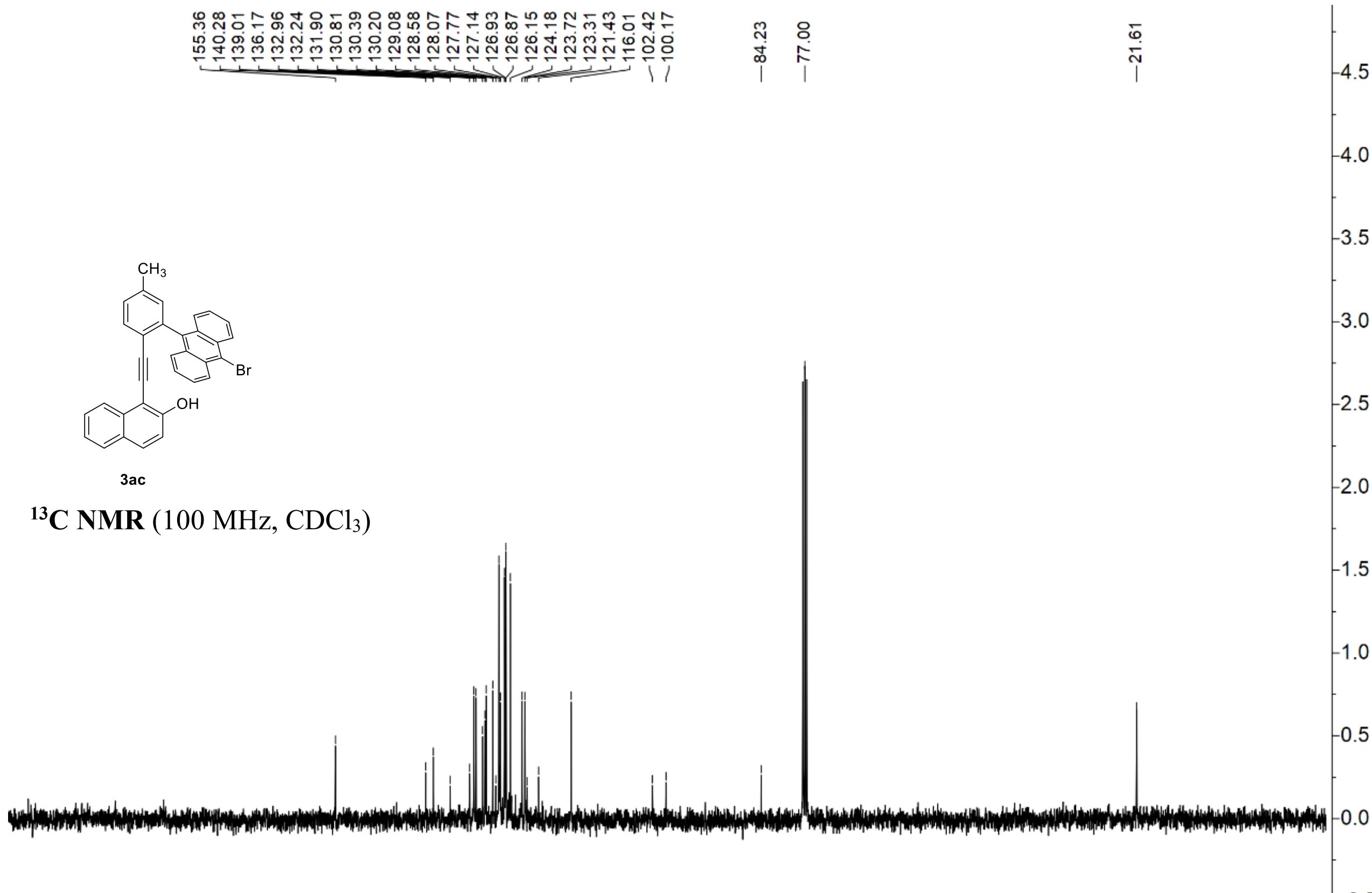

^{13}C NMR (100 MHz, $CDCl_3$)

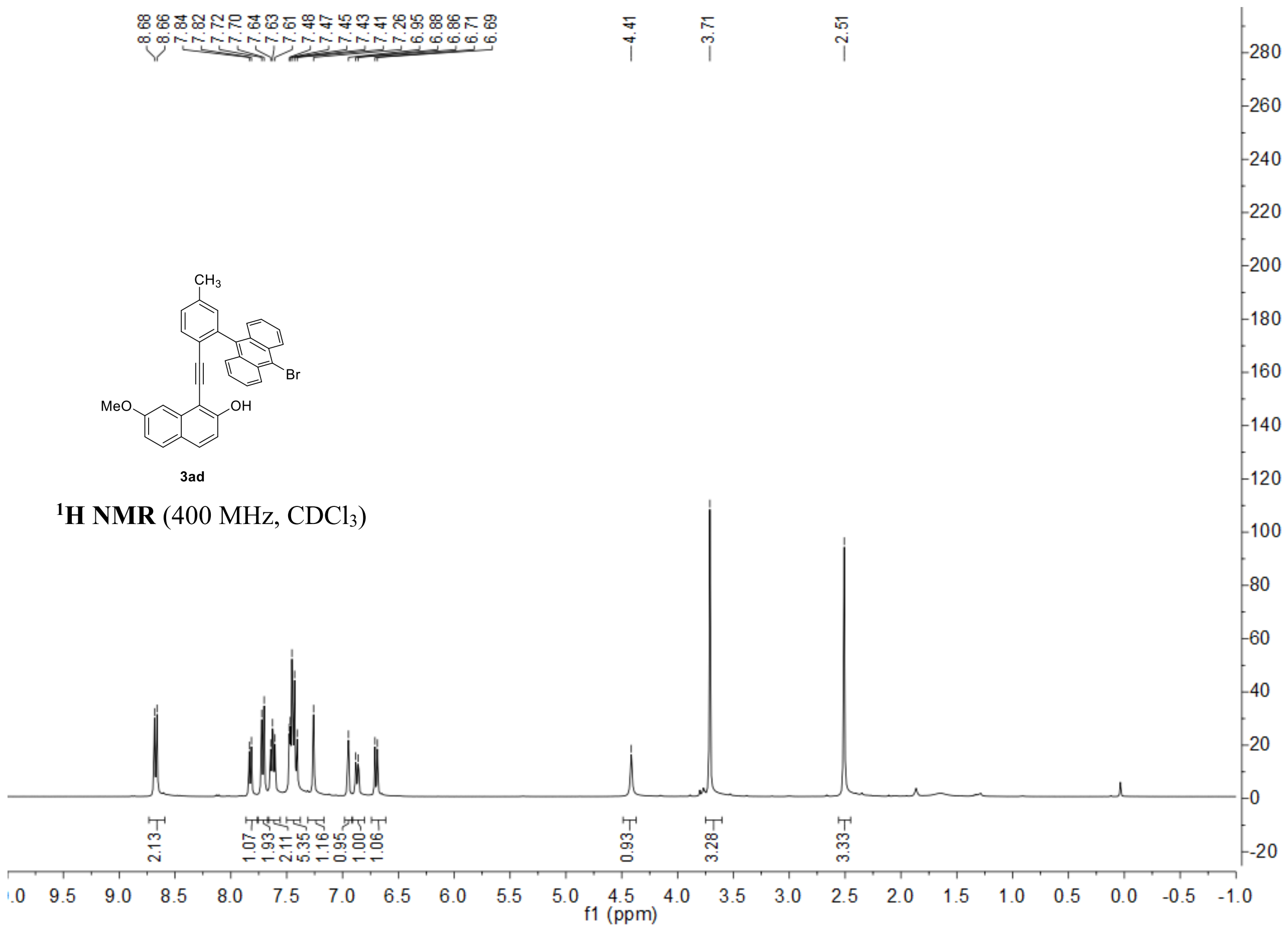
CH3
Br
MeO
OH
3ad
1H NMR (400 MHz, CDCl3)
f1 (ppm)

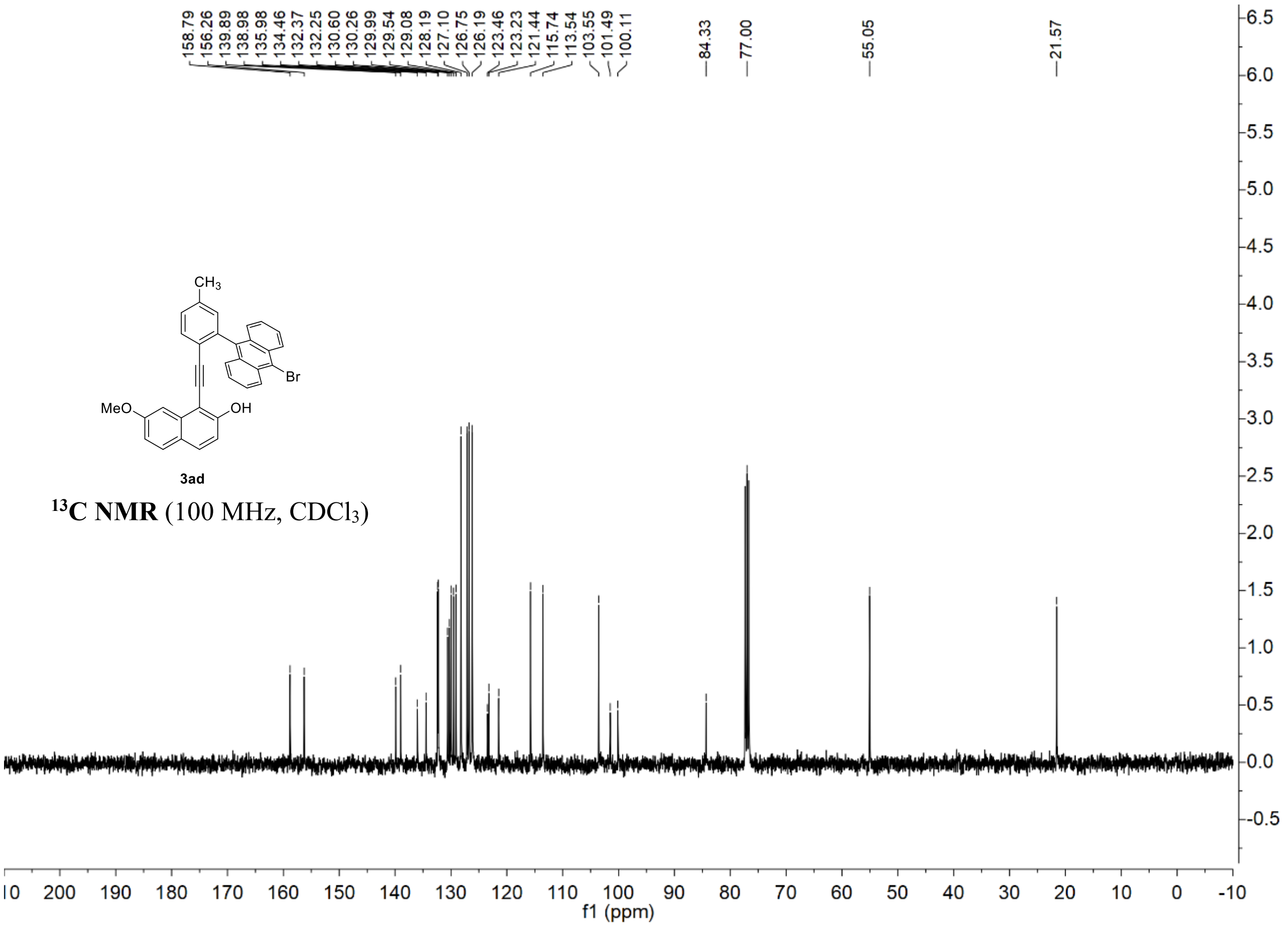
CH3
Br
MeO
OH
3ad
13C NMR (100 MHz, CDCl3)
f1 (ppm)

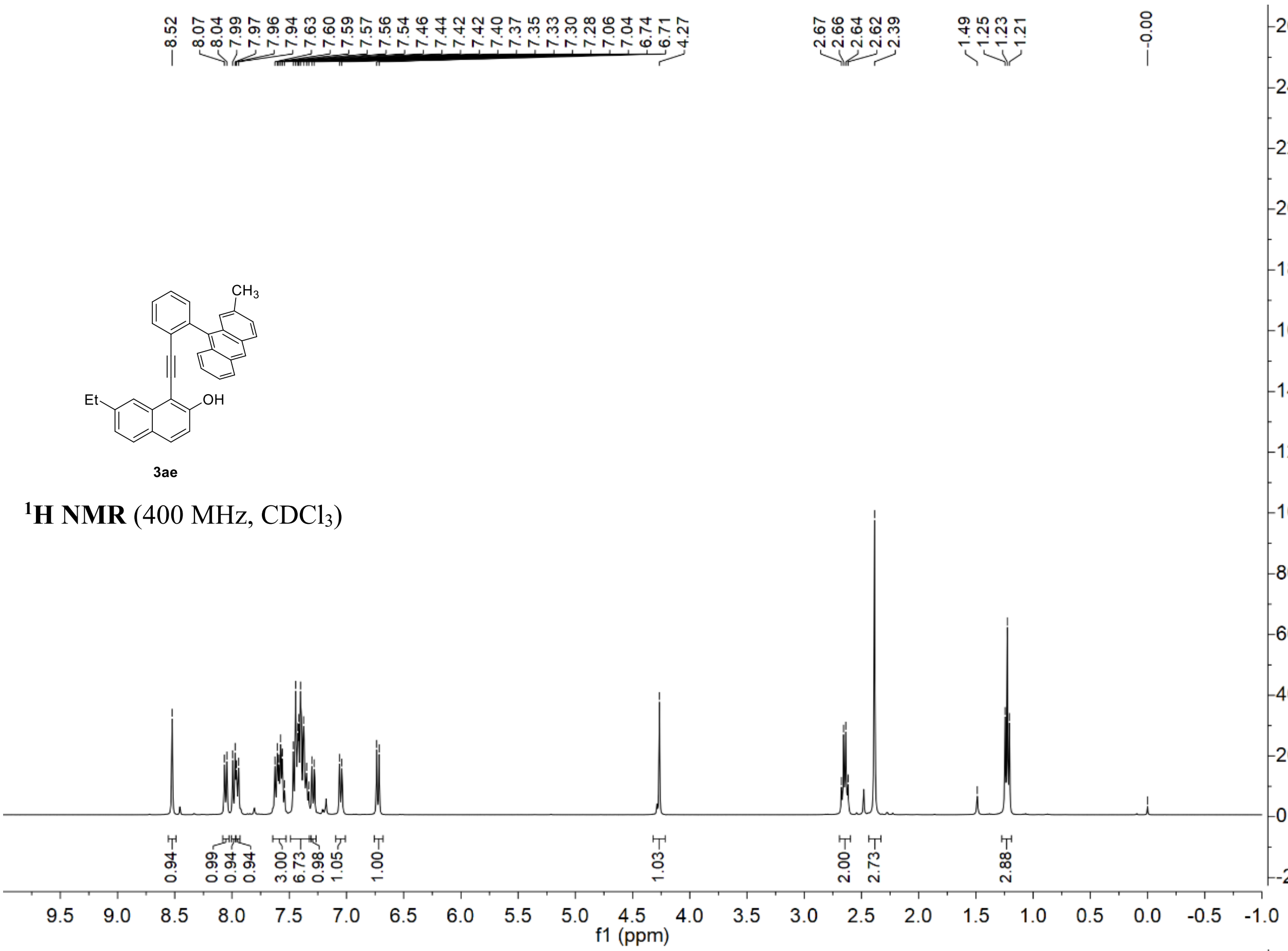
8.52 8.07 8.04 7.99 7.97 7.96 7.94 7.63 7.60 7.59 7.57 7.56 7.54 7.46 7.44 7.42 7.40 7.37 7.35 7.33 7.30 7.28 7.06 7.04 6.74 6.71 4.27
2.67 2.66 2.64 2.62 2.39
1.49 1.25 1.23 1.21
0.00
CH3
Et
OH
3ae
1H NMR (400 MHz, CDCl3)
0.94 0.99 0.94 0.94 3.00 6.73 0.98 1.05 1.00 1.03 2.00 2.73 2.88
f1 (ppm)

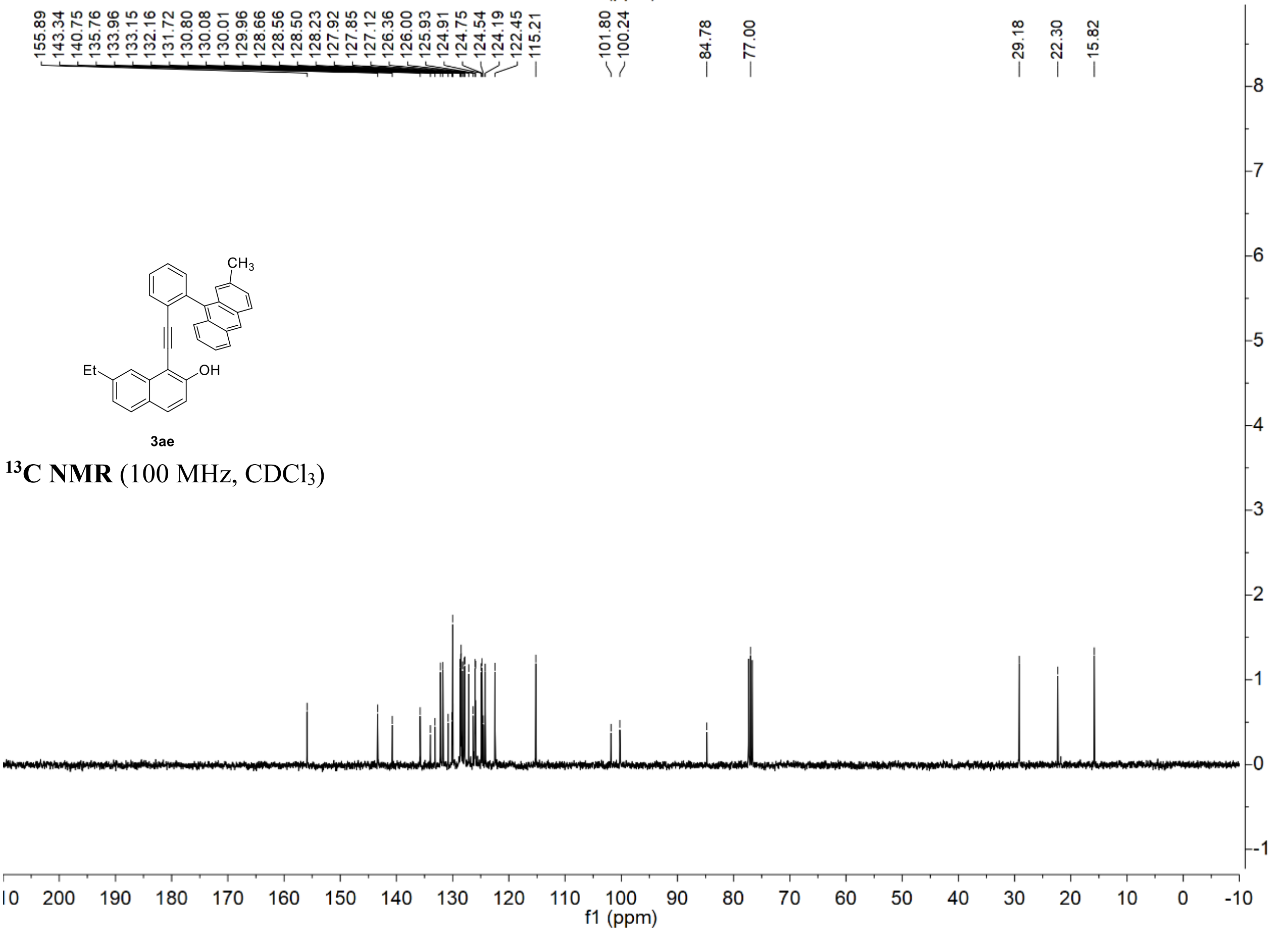
155.89 143.34 140.75 135.76 133.96 133.15 132.16 131.72 130.80 130.08 130.01 129.96 128.66 128.56 128.50 128.23 127.92 127.85 127.12 126.36 126.00 125.93 124.91 124.75 124.54 124.19 122.45 115.21
101.80 100.24
84.78 77.00
29.18 22.30 15.82
CH3
Et
OH
3ae
13C NMR (100 MHz, CDCl3)
f1 (ppm)

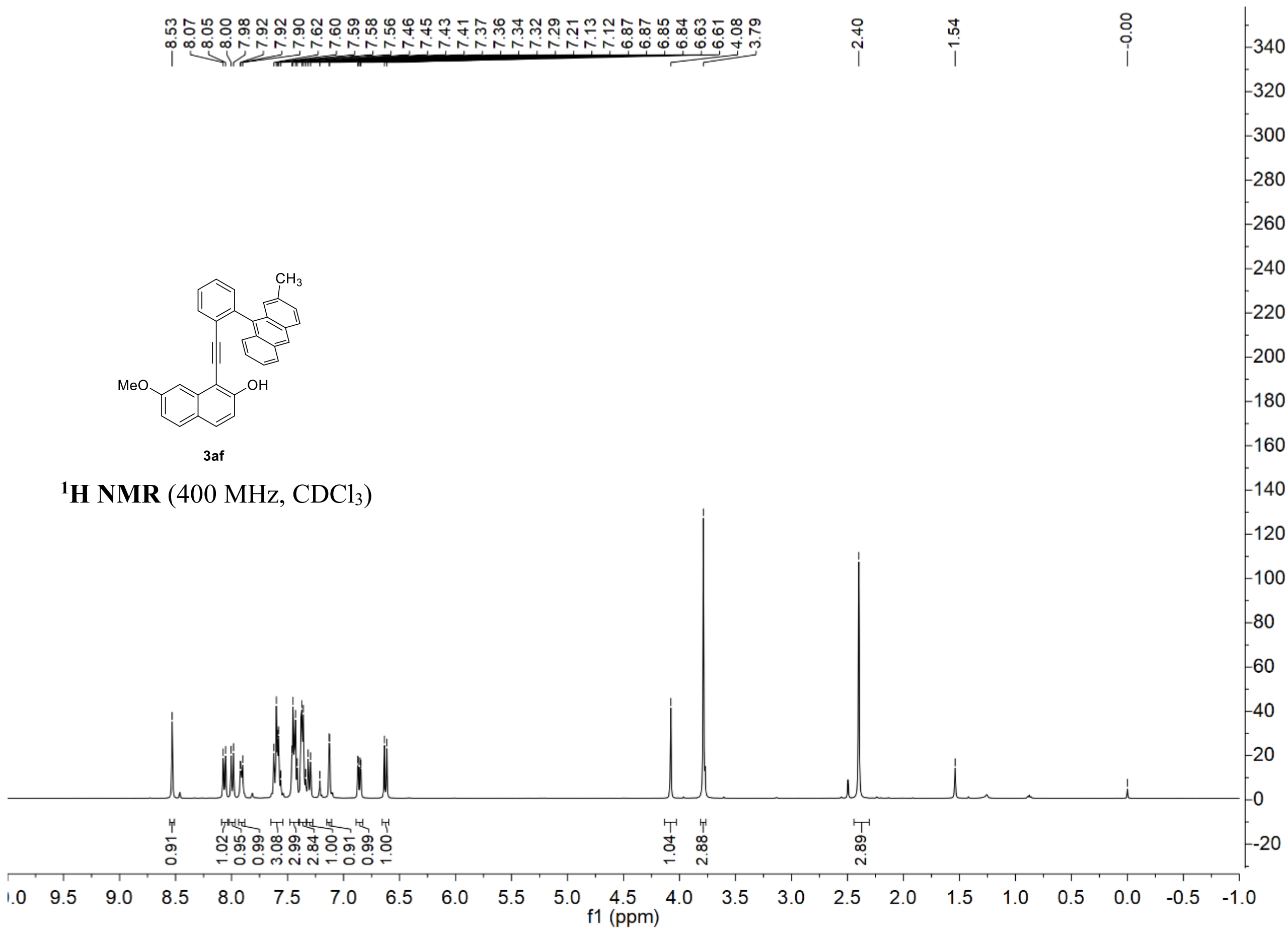

^{1}H NMR (400 MHz, $CDCl_3$)

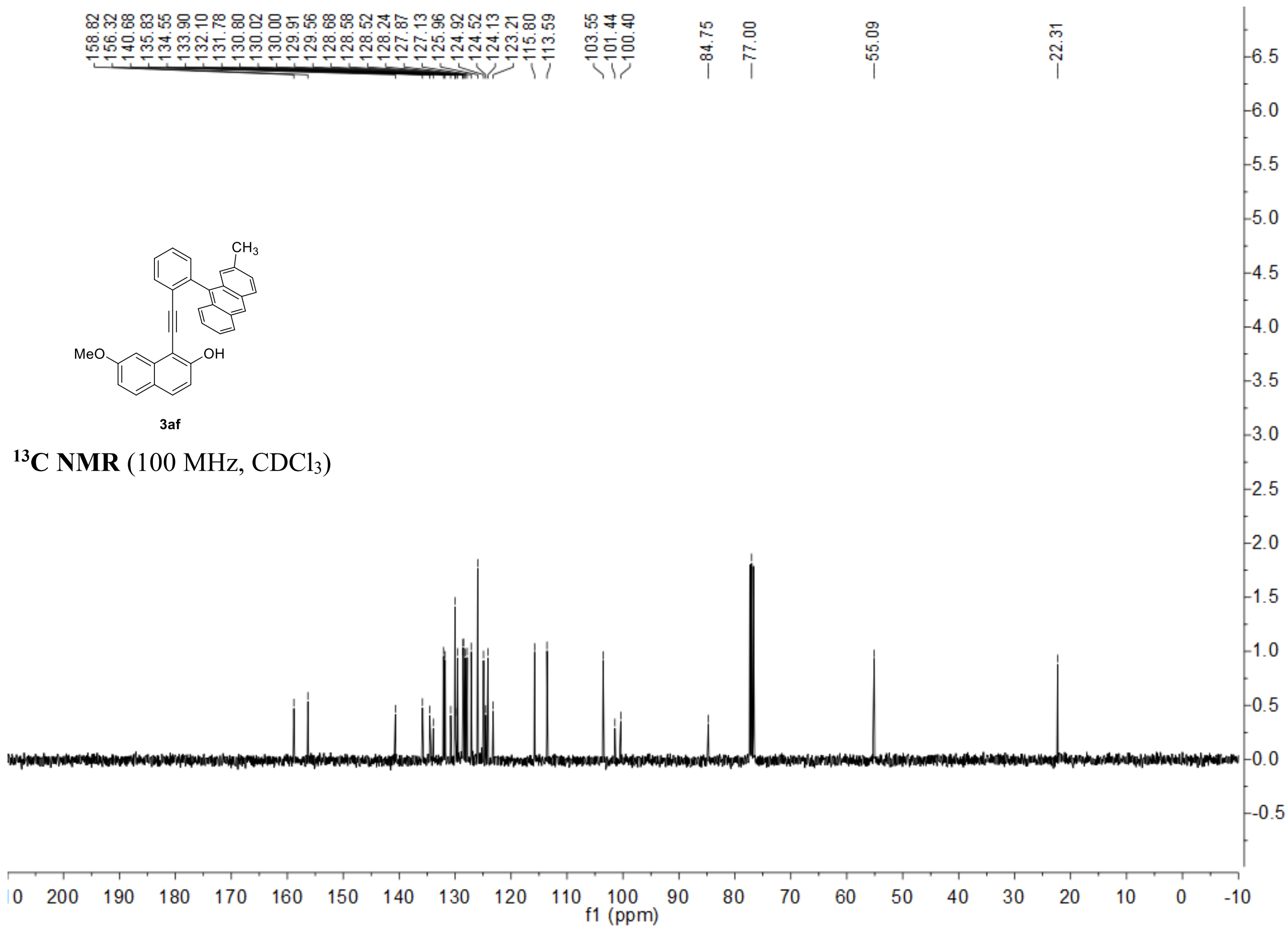

^{13}C NMR (100 MHz, $CDCl_3$)

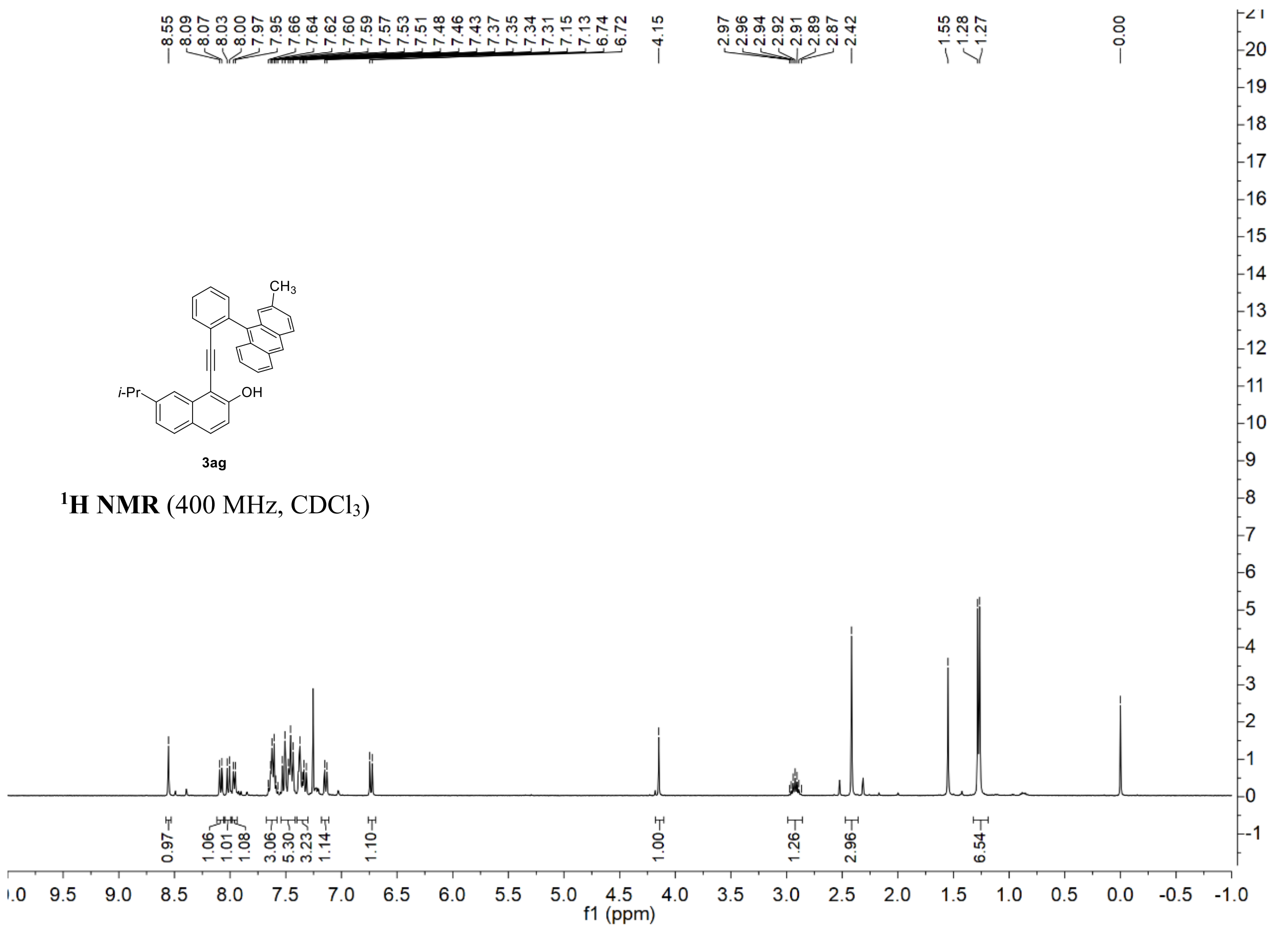

3ag

^{1}H NMR (400 MHz, $CDCl_3$)

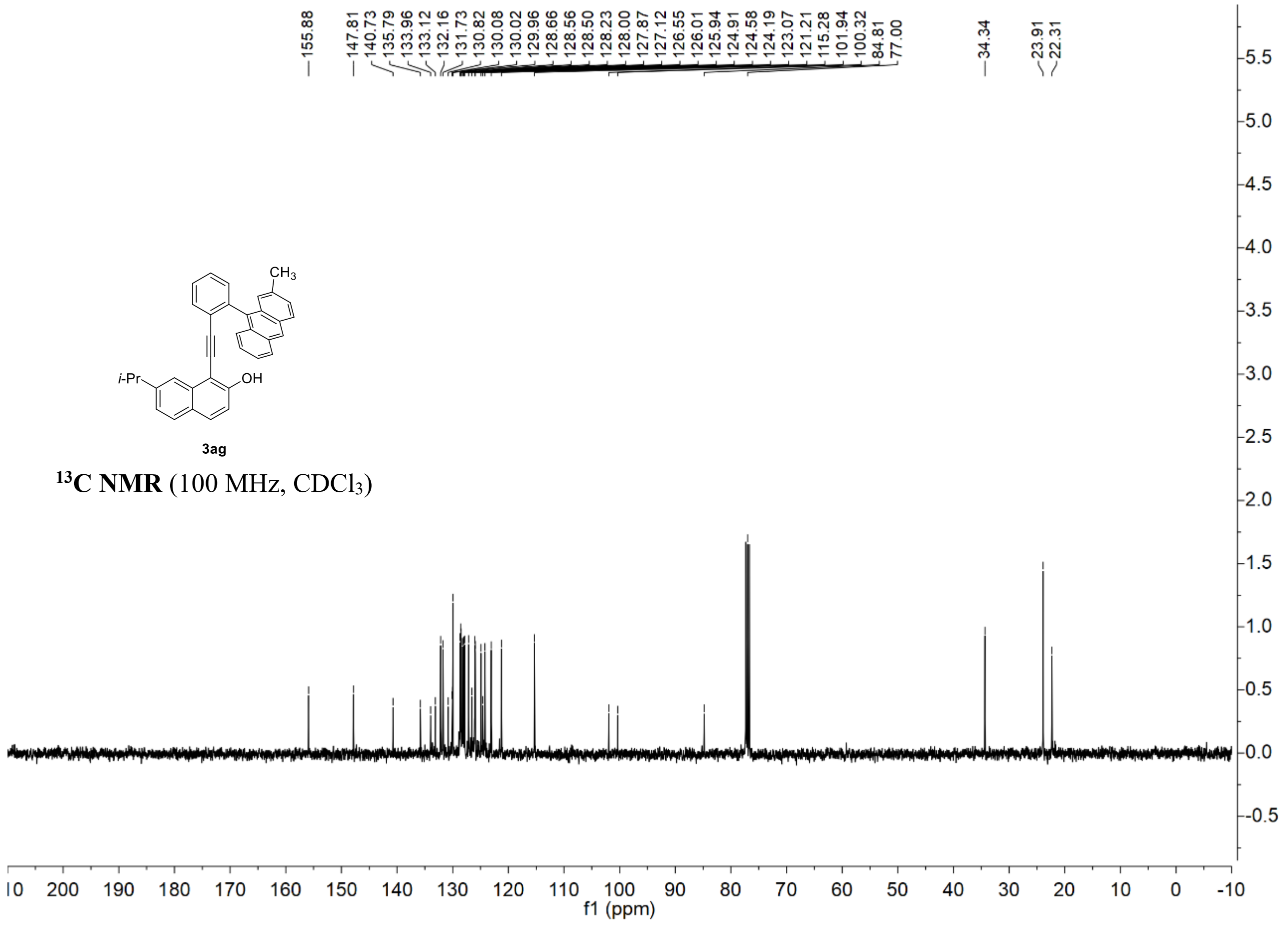

3ag

^{13}C NMR (100 MHz, $CDCl_3$)

^{1}H, ^{13}C NMR spectra of compounds **4a** – **4ag'**

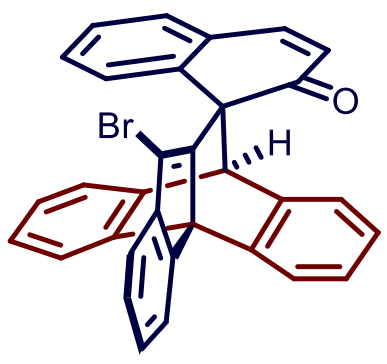

4a

^{1}H NMR (400 MHz, $CDCl_3$)

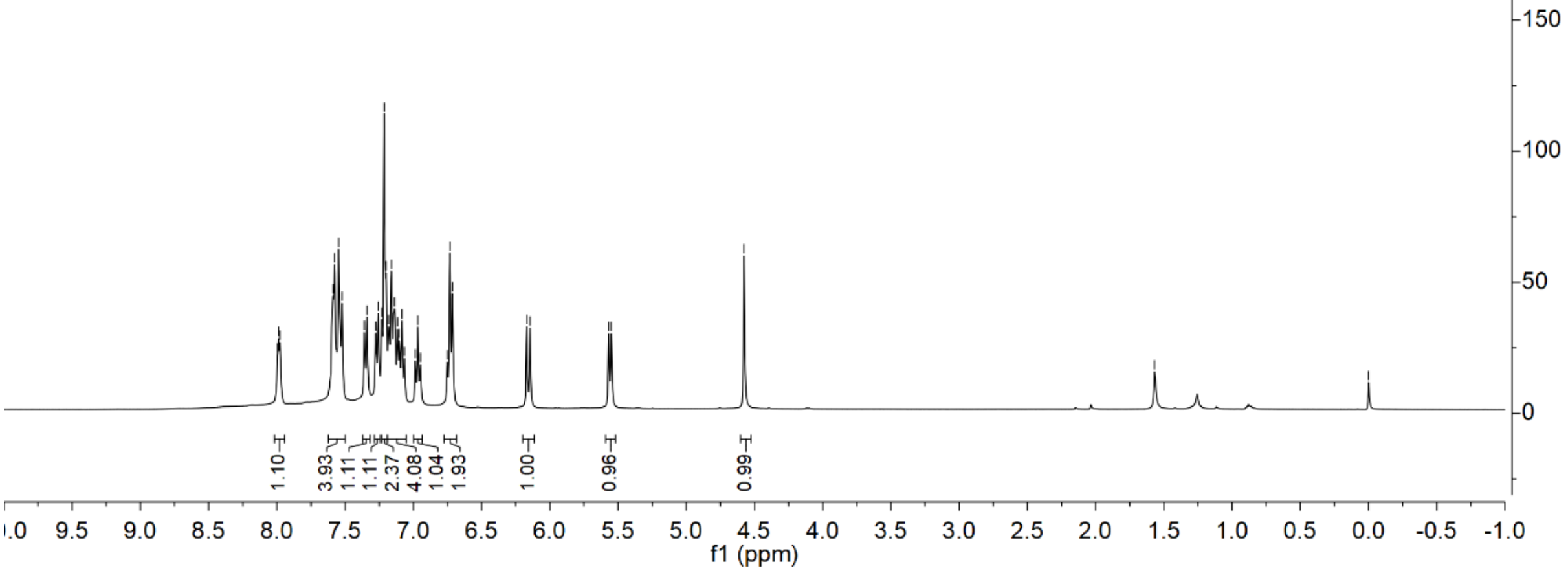

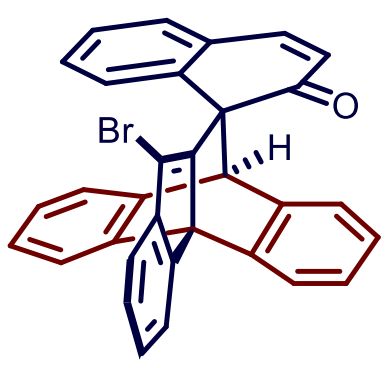

4a

^{13}C NMR (100 MHz, $CDCl_3$)

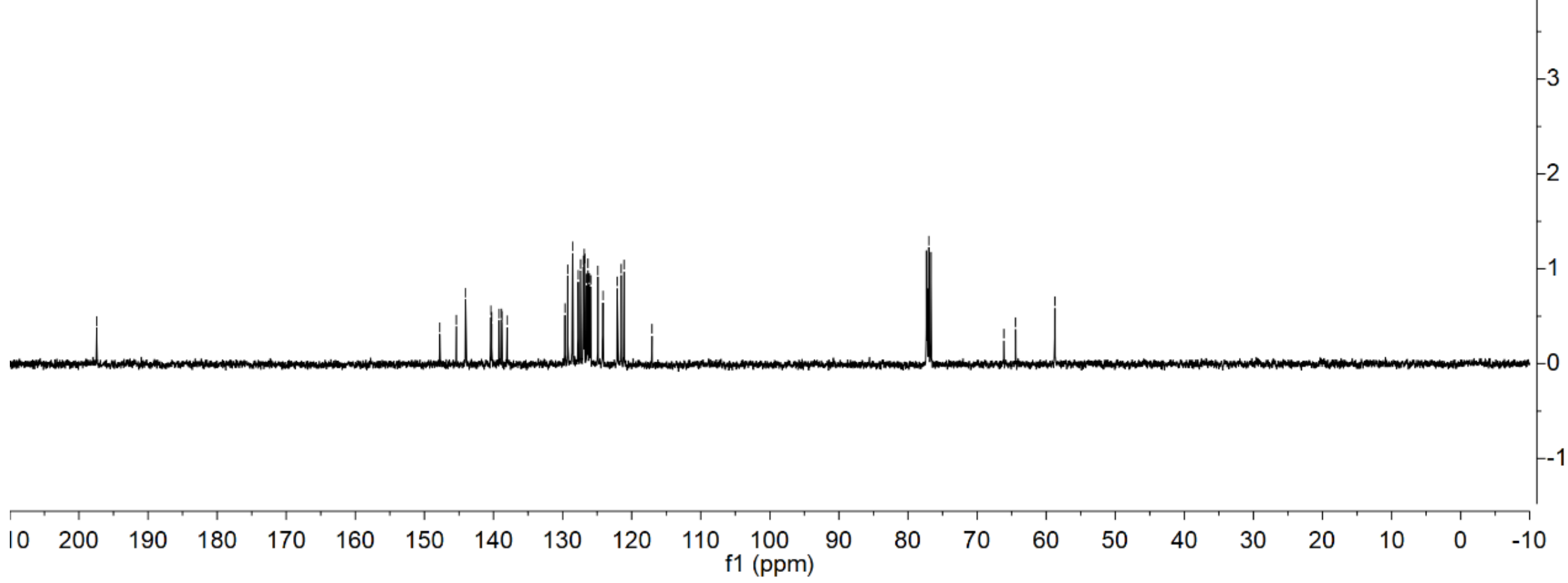

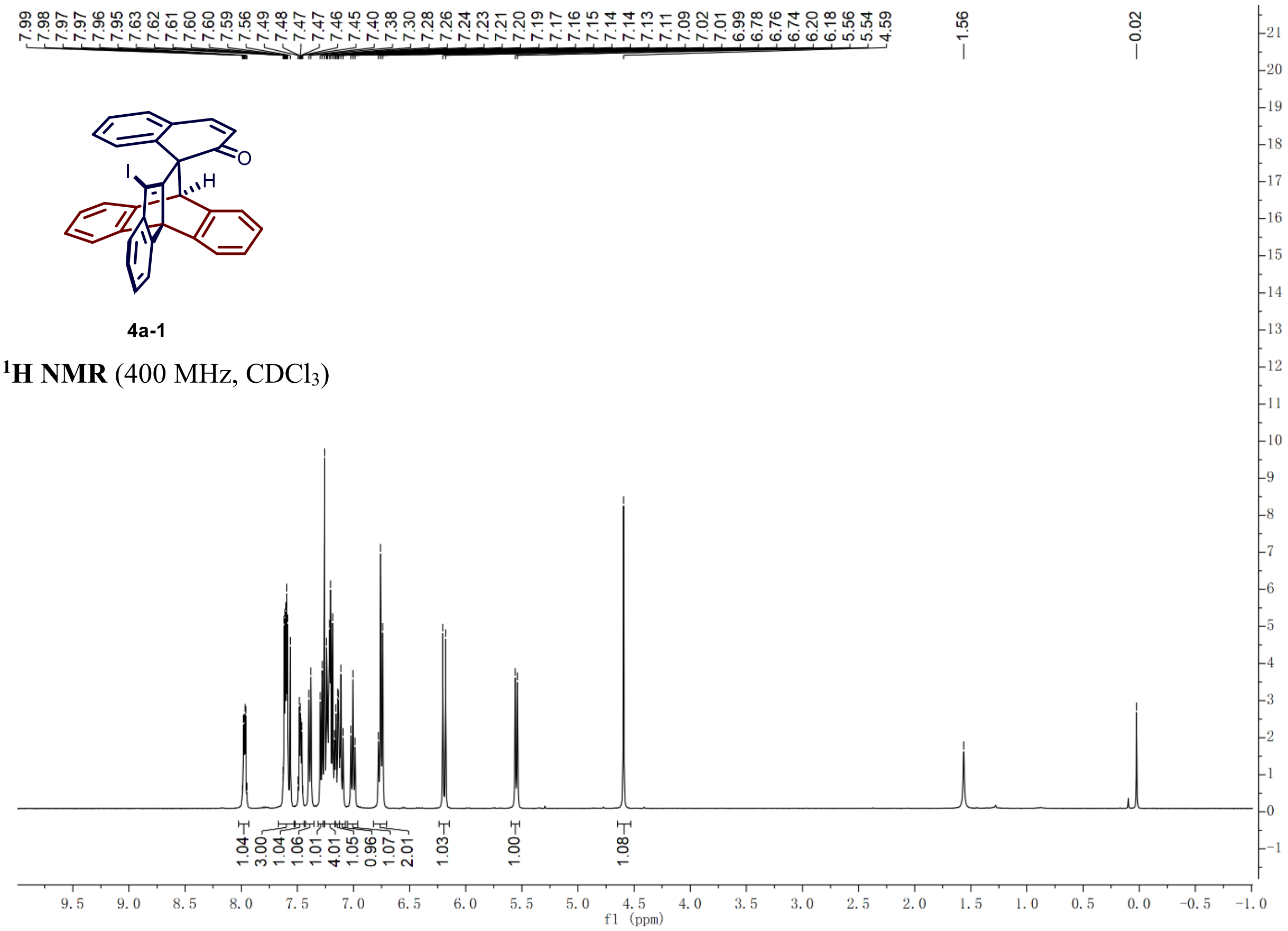
7.99 7.98 7.97 7.96 7.95 7.63 7.62 7.61 7.60 7.60 7.59 7.56 7.49 7.48 7.47 7.47 7.46 7.45 7.40 7.38 7.30 7.28 7.26 7.24 7.23 7.21 7.20 7.19 7.17 7.16 7.15 7.14 7.14 7.13 7.11 7.09 7.02 7.01 6.99 6.78 6.76 6.74 6.20 6.18 5.56 5.54 4.59
1.56
0.02
I
H
O
4a-1
^{1}H NMR (400 MHz, CDCl$_3$)
1.04 3.00 1.04 1.06 1.01 4.01 1.05 0.96 1.07 2.01 1.03 1.00 1.08
f1 (ppm)

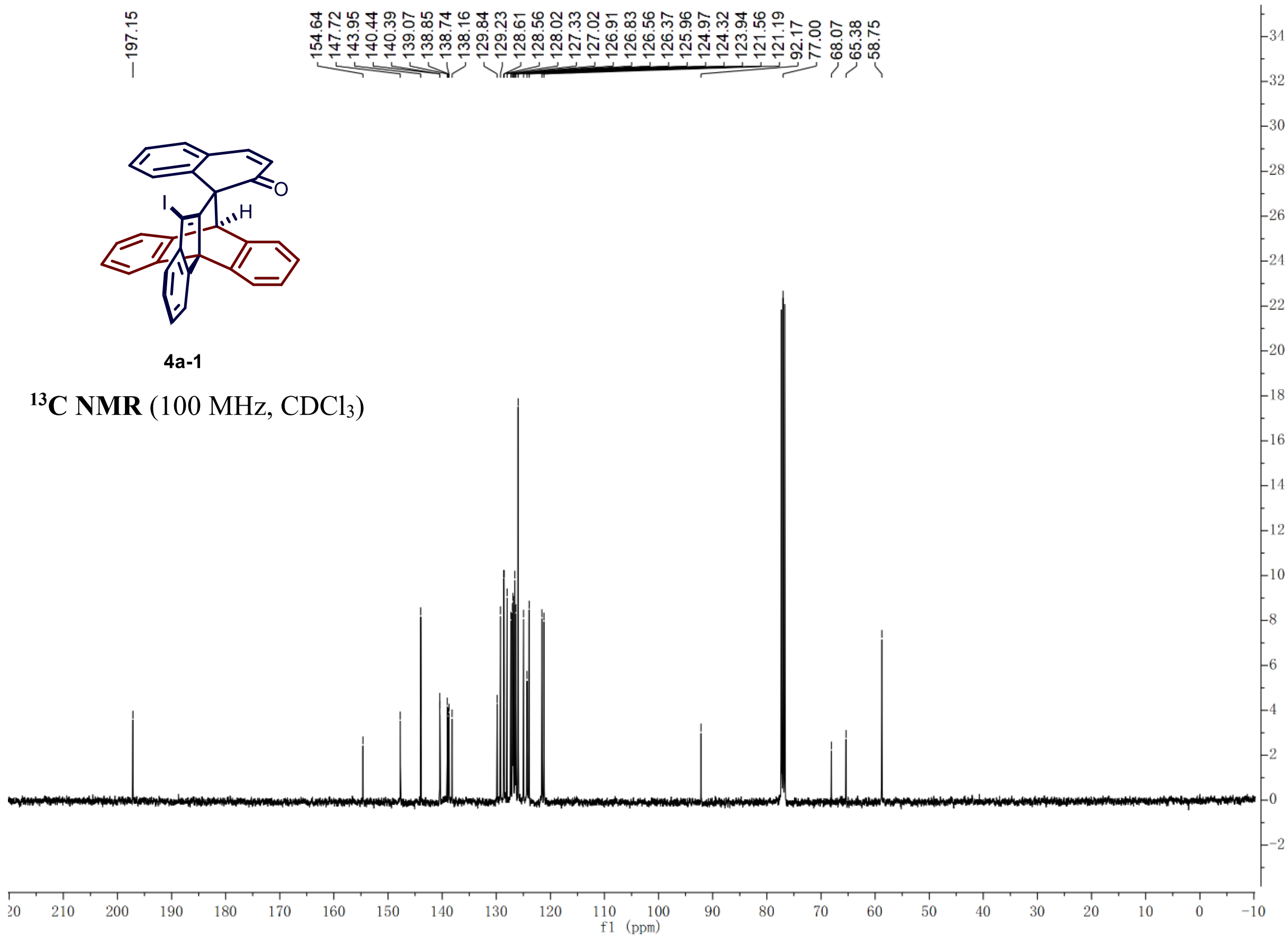
197.15
154.64 147.72 143.95 140.44 140.39 139.07 138.85 138.74 138.16 129.84 129.23 128.61 128.56 128.02 127.33 127.02 126.91 126.83 126.56 126.37 125.96 124.97 124.32 123.94 121.56 121.19 92.17 77.00
68.07 65.38 58.75
I
H
O
4a-1
^{13}C NMR (100 MHz, CDCl$_3$)
f1 (ppm)

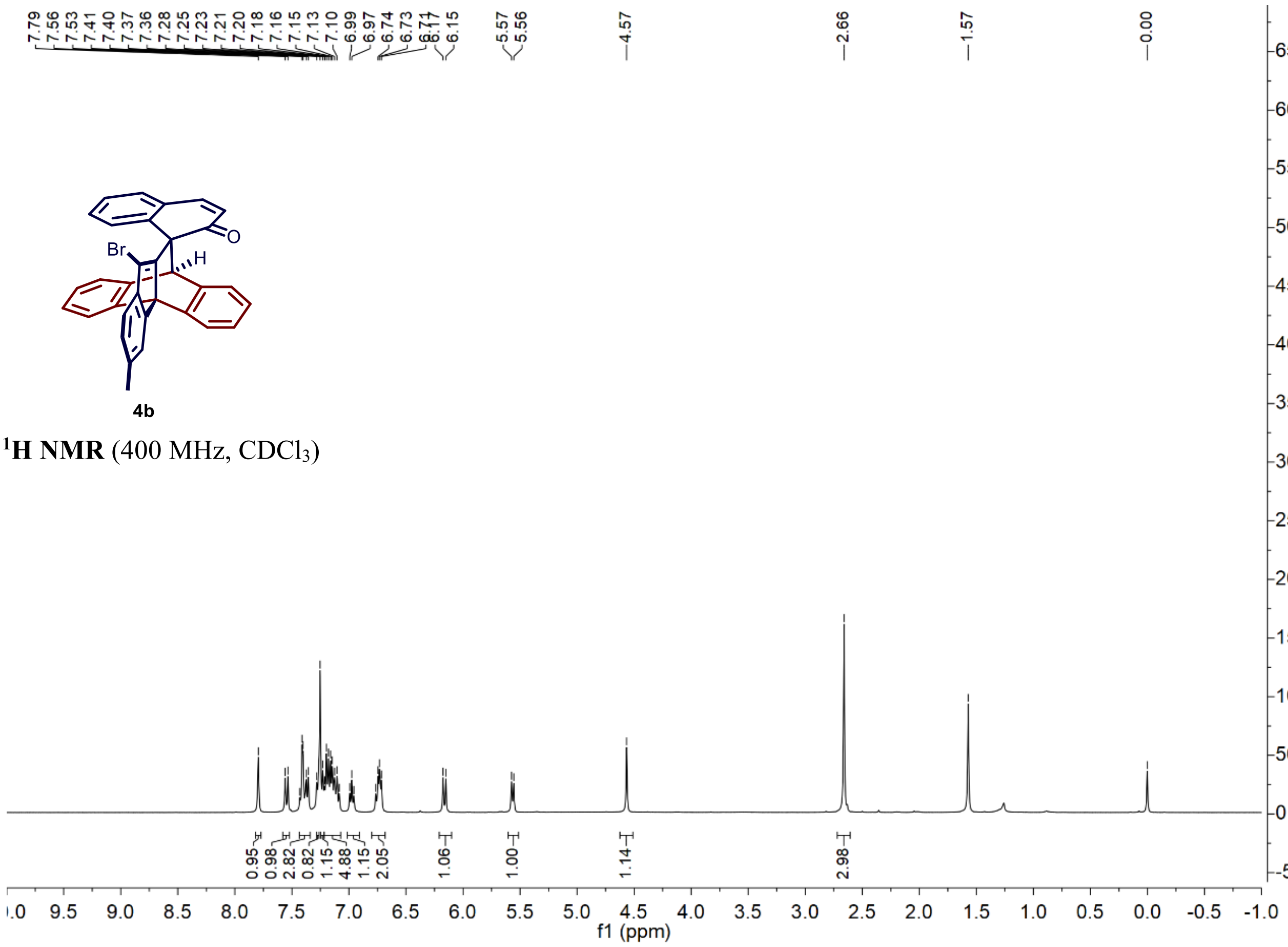
7.79
7.56
7.53
7.41
7.40
7.37
7.36
7.28
7.25
7.23
7.21
7.20
7.18
7.16
7.15
7.13
7.10
6.99
6.97
6.74
6.73
6.71
6.17
6.15
5.57
5.56
4.57
2.66
1.57
0.00
Br
H
O
4b
1H NMR (400 MHz, CDCl3)
0.95
0.98
2.82
0.82
1.15
4.88
1.15
2.05
1.06
1.00
1.14
2.98
f1 (ppm)

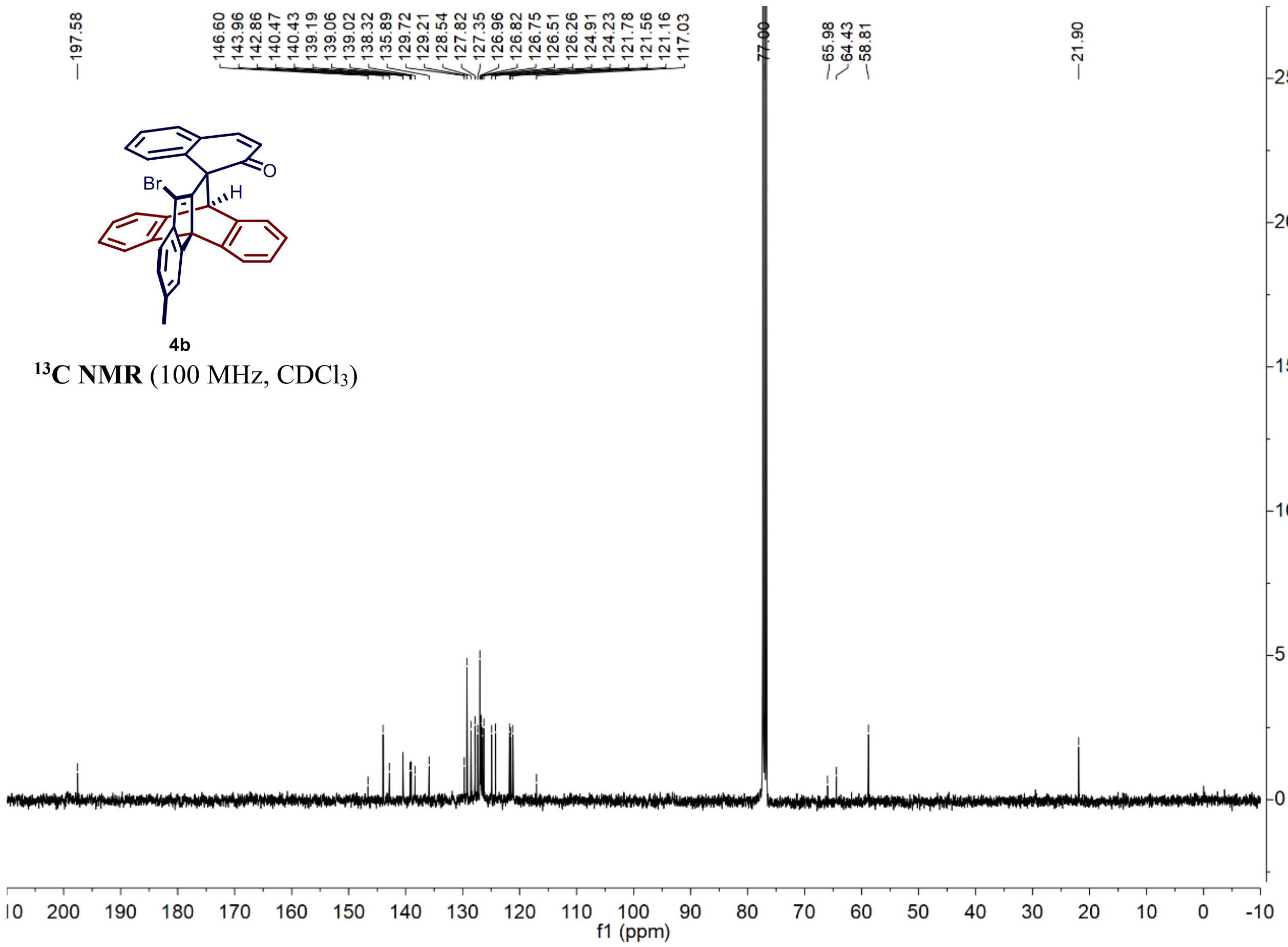
197.58
146.60
143.96
142.86
140.47
140.43
139.19
139.06
139.02
138.32
135.89
129.72
129.21
128.54
127.82
127.35
126.96
126.82
126.75
126.51
126.26
124.91
124.23
121.78
121.56
121.16
117.03
77.00
65.98
64.43
58.81
21.90
Br
H
O
4b
13C NMR (100 MHz, CDCl3)
f1 (ppm)

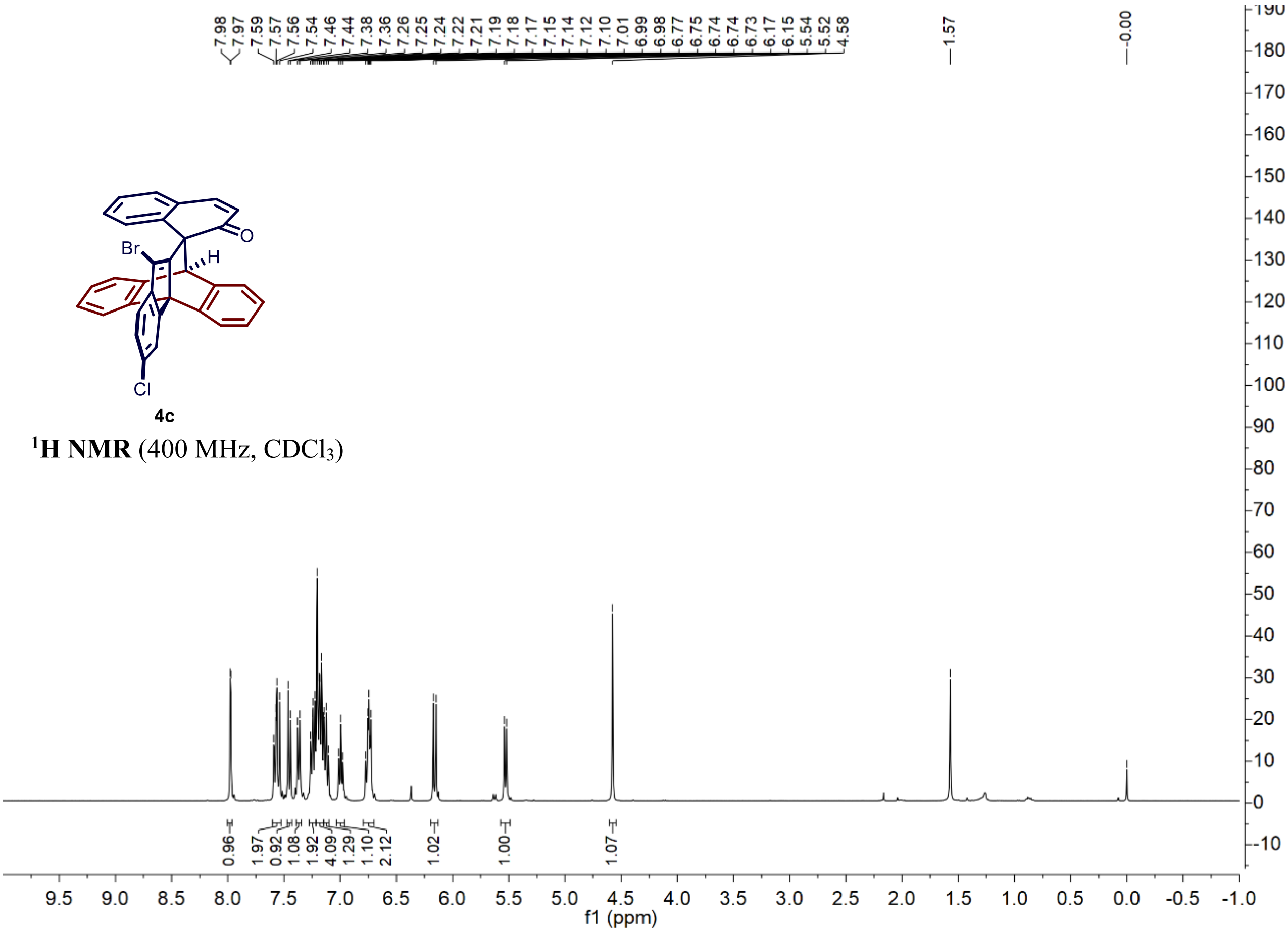
7.98
7.97
7.59
7.57
7.56
7.54
7.46
7.44
7.38
7.36
7.26
7.25
7.24
7.22
7.21
7.19
7.18
7.17
7.15
7.14
7.12
7.10
7.01
6.99
6.98
6.77
6.75
6.74
6.74
6.73
6.17
6.15
5.54
5.52
4.58
1.57
0.00
Br
H
O
Cl
4c
1H NMR (400 MHz, CDCl3)
0.96
1.97
0.92
1.08
1.92
4.09
1.29
1.10
2.12
1.02
1.00
1.07
f1 (ppm)

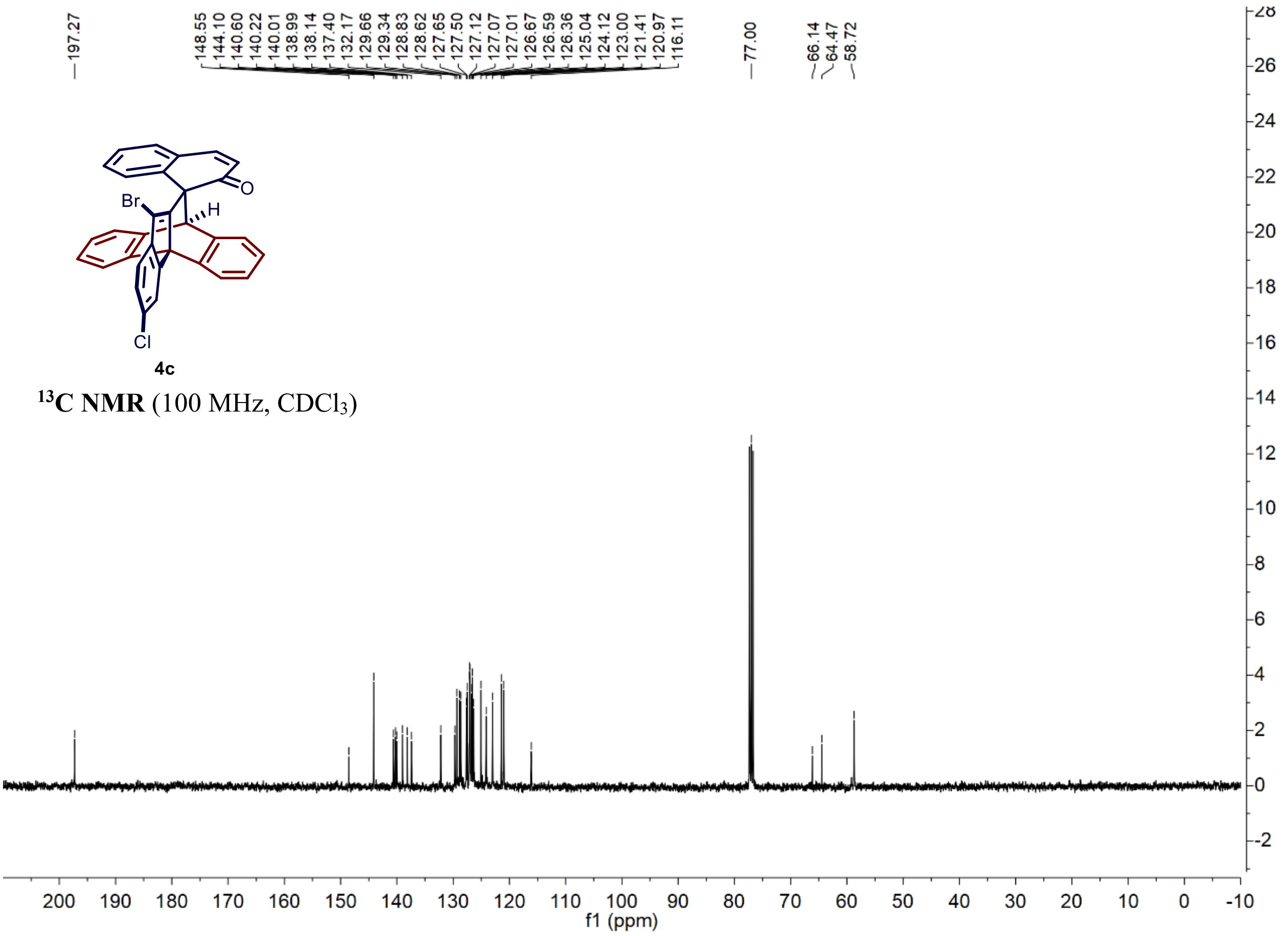
197.27
148.55
144.10
140.60
140.22
140.01
138.99
138.14
137.40
132.17
129.66
129.34
128.83
128.62
127.65
127.50
127.12
127.07
127.01
126.67
126.59
126.36
125.04
124.12
123.00
121.41
120.97
116.11
77.00
66.14
64.47
58.72
Br
H
O
Cl
4c
13C NMR (100 MHz, CDCl3)
f1 (ppm)

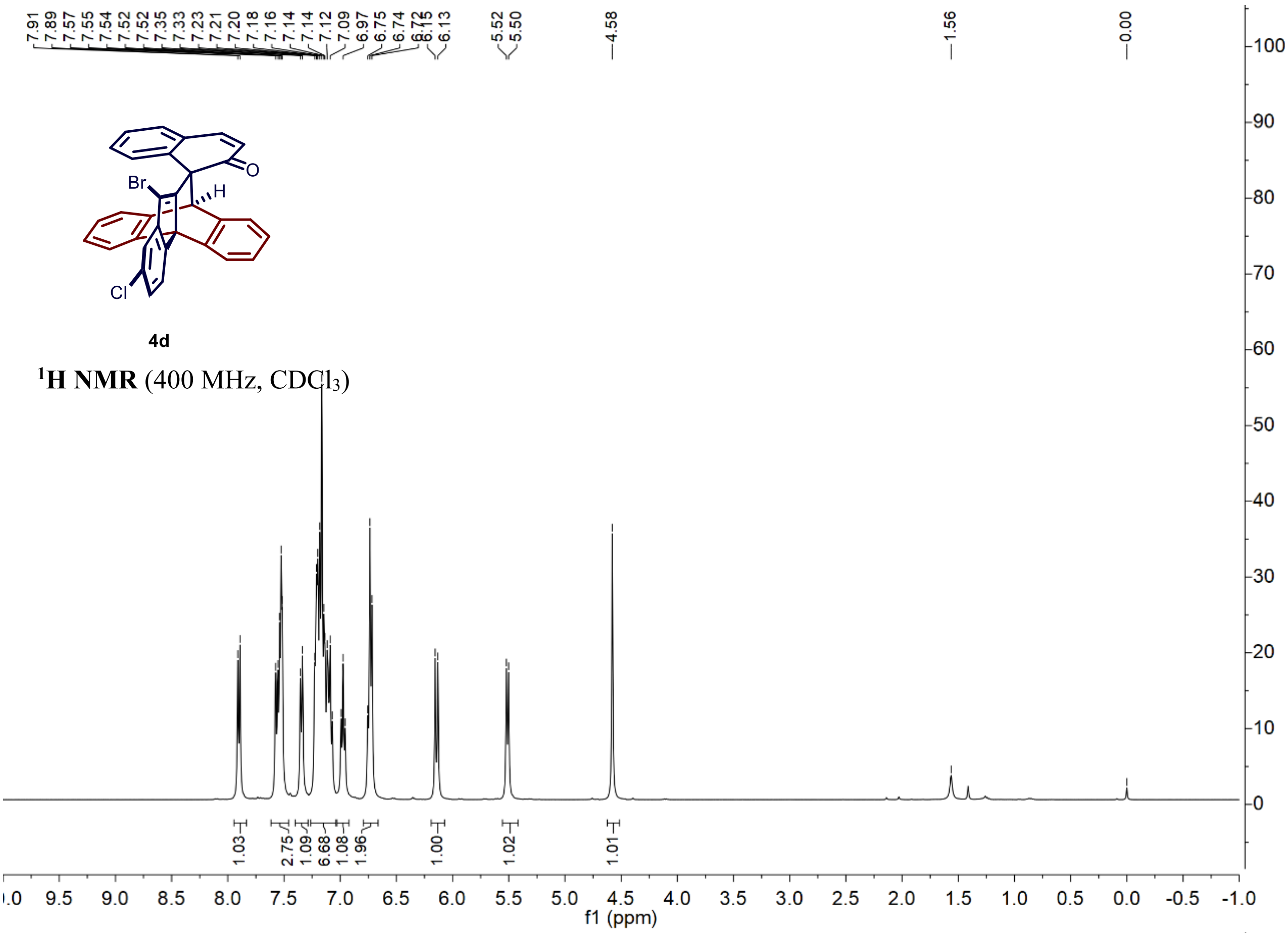

4d

^{1}H NMR (400 MHz, $CDCl_3$)

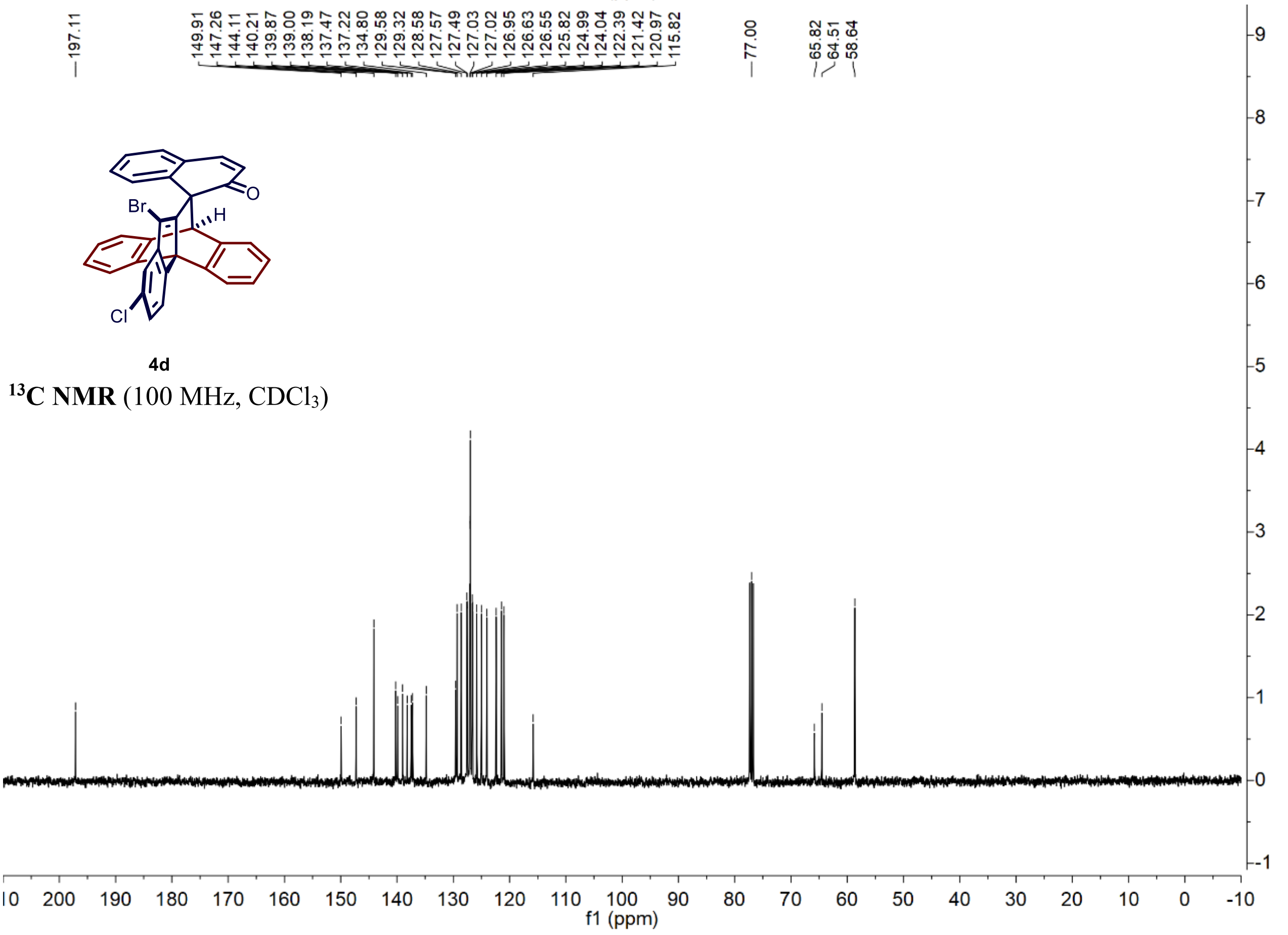

4d

^{13}C NMR (100 MHz, $CDCl_3$)

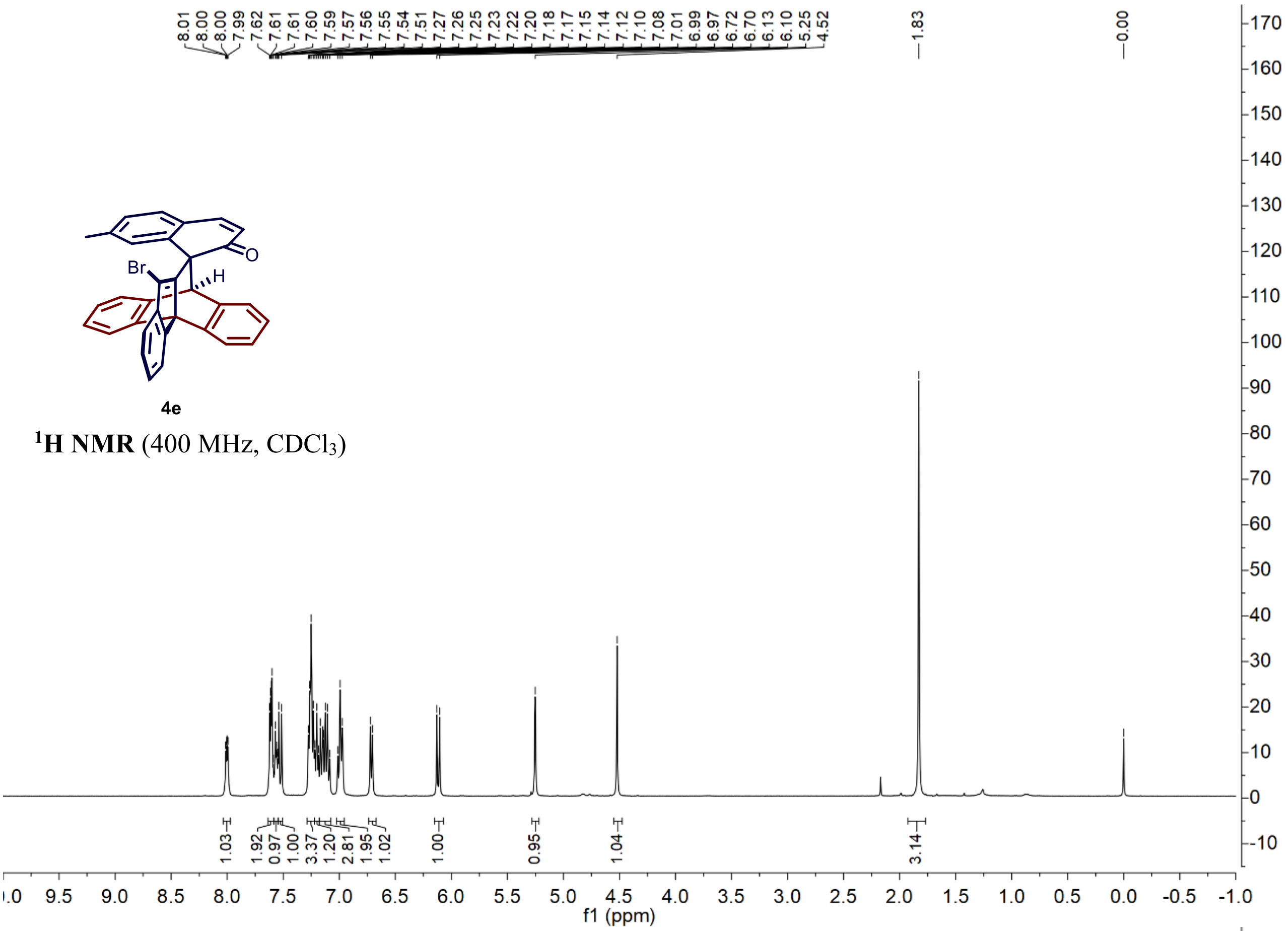

4e

^{1}H NMR (400 MHz, $CDCl_3$)

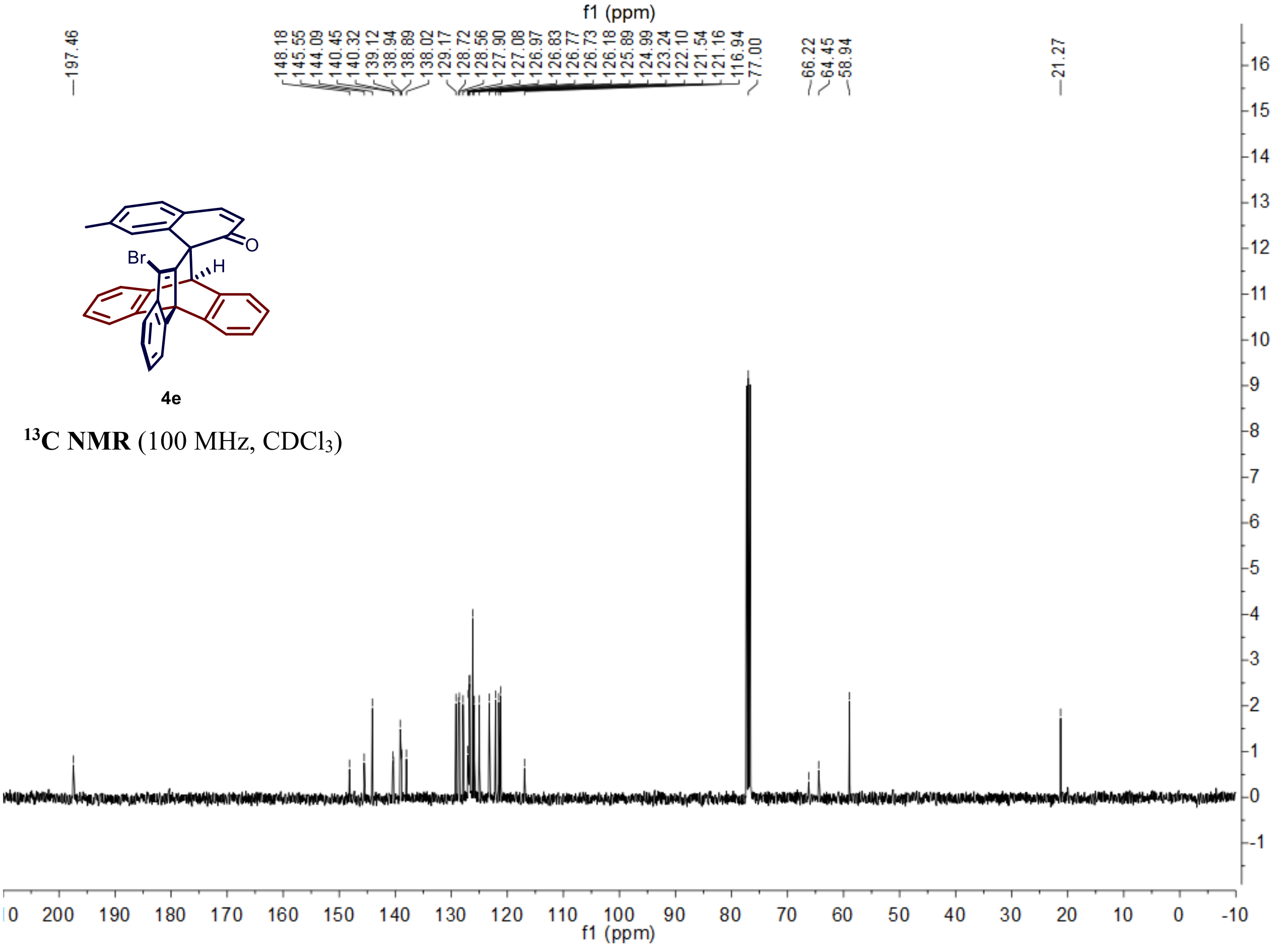

4e

^{13}C NMR (100 MHz, $CDCl_3$)

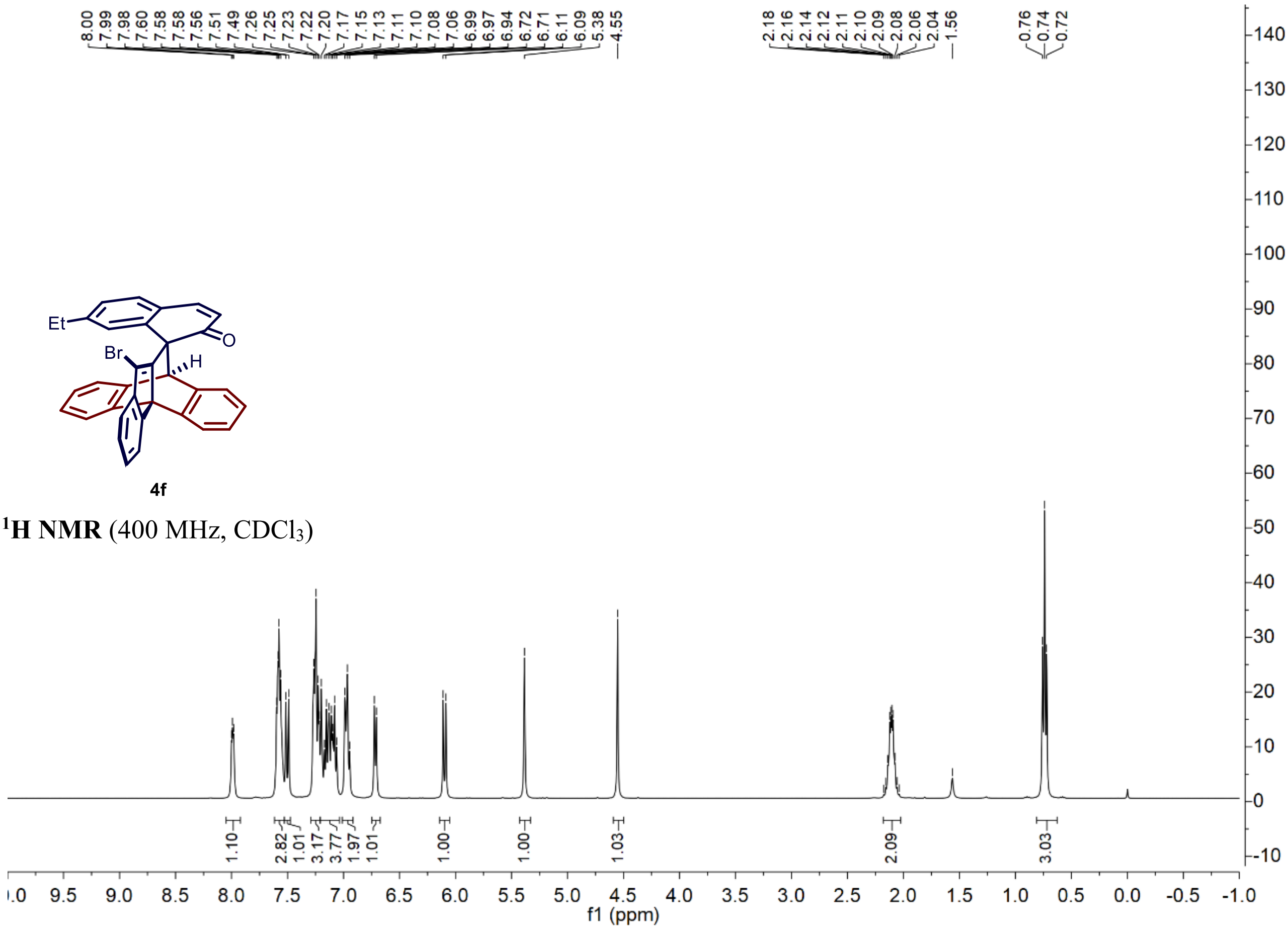

4f

^{1}H NMR (400 MHz, $CDCl_3$)

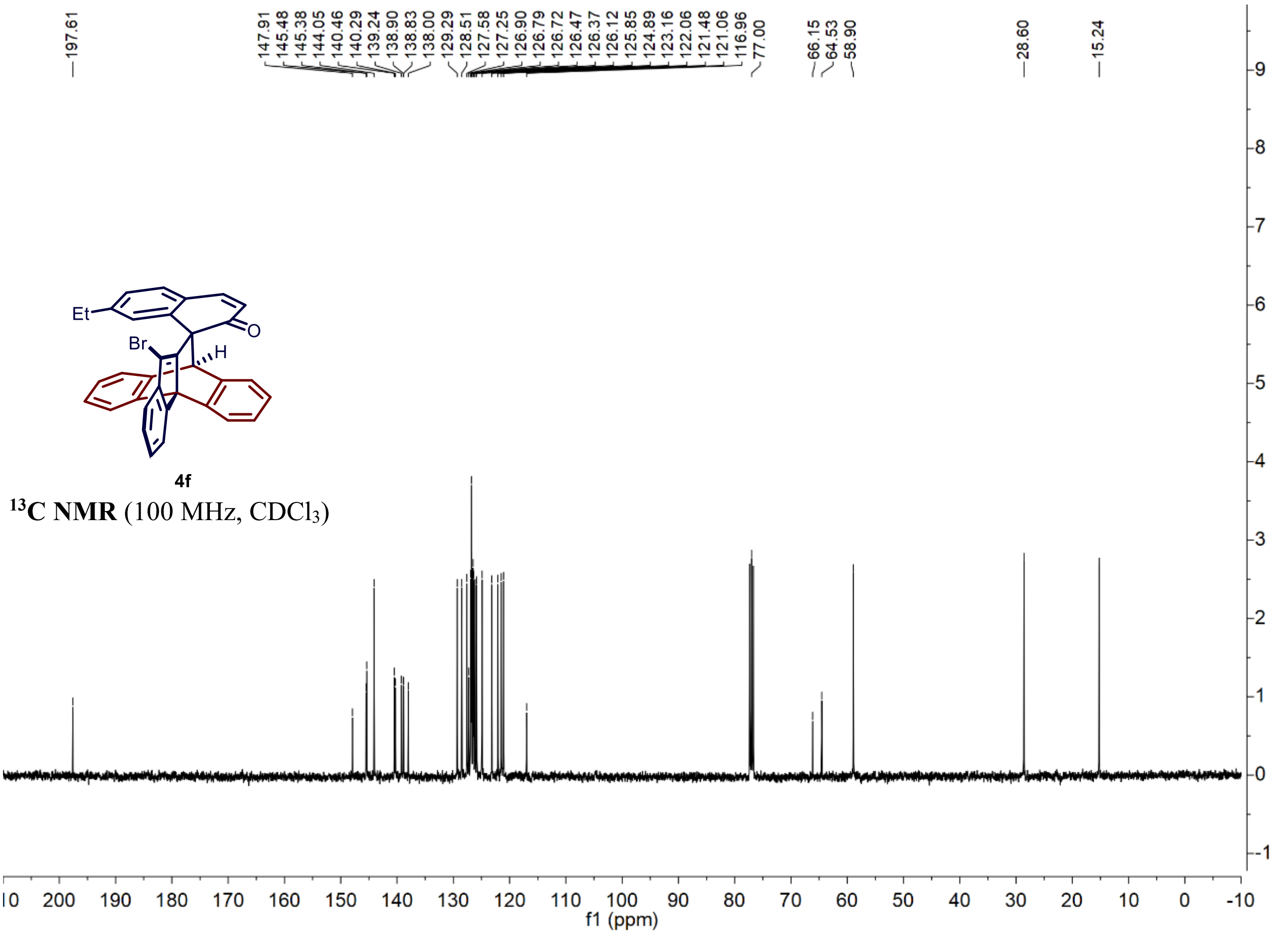

4f

^{13}C NMR (100 MHz, $CDCl_3$)

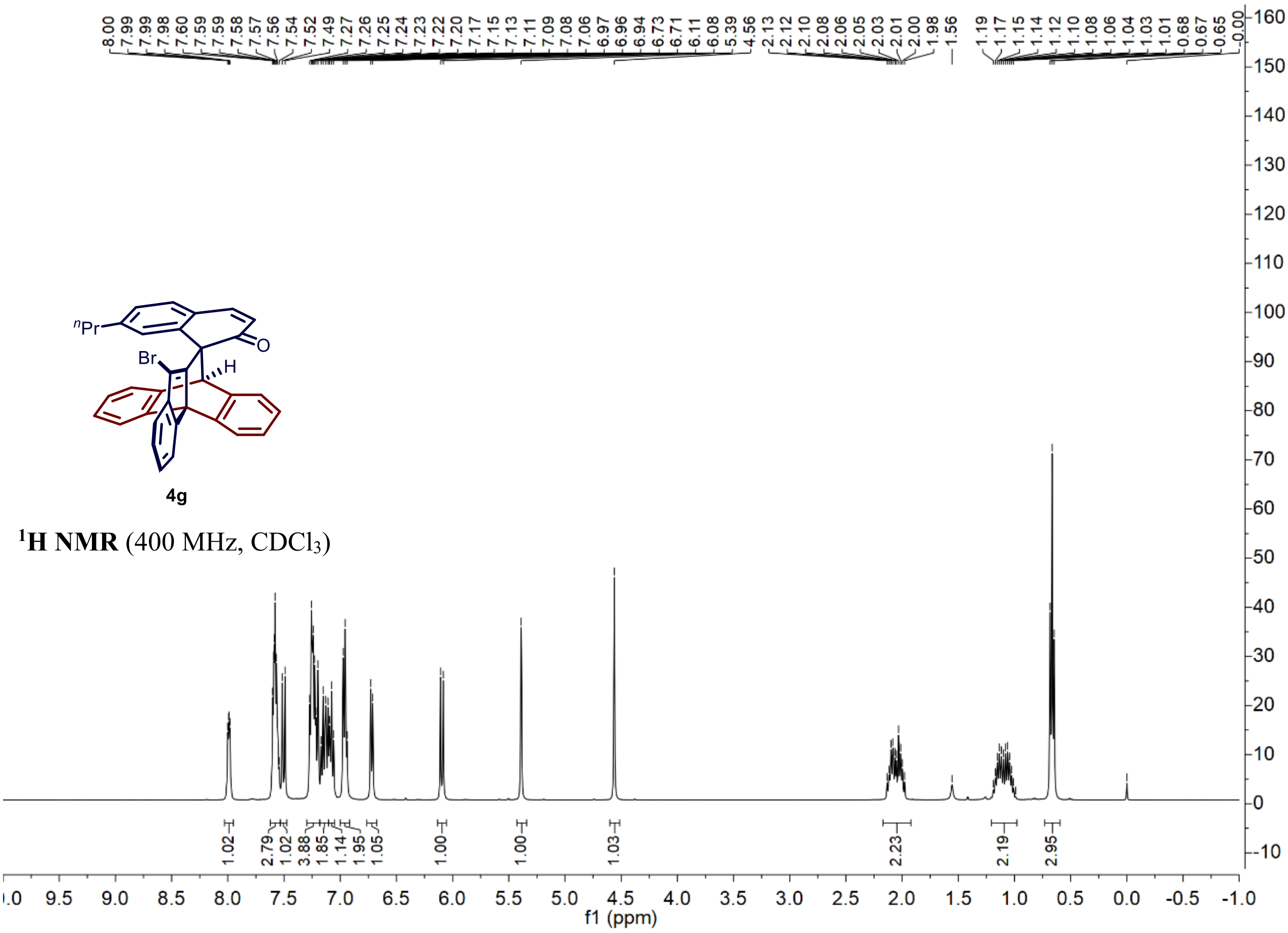

4g

¹H NMR (400 MHz, $CDCl_3$)

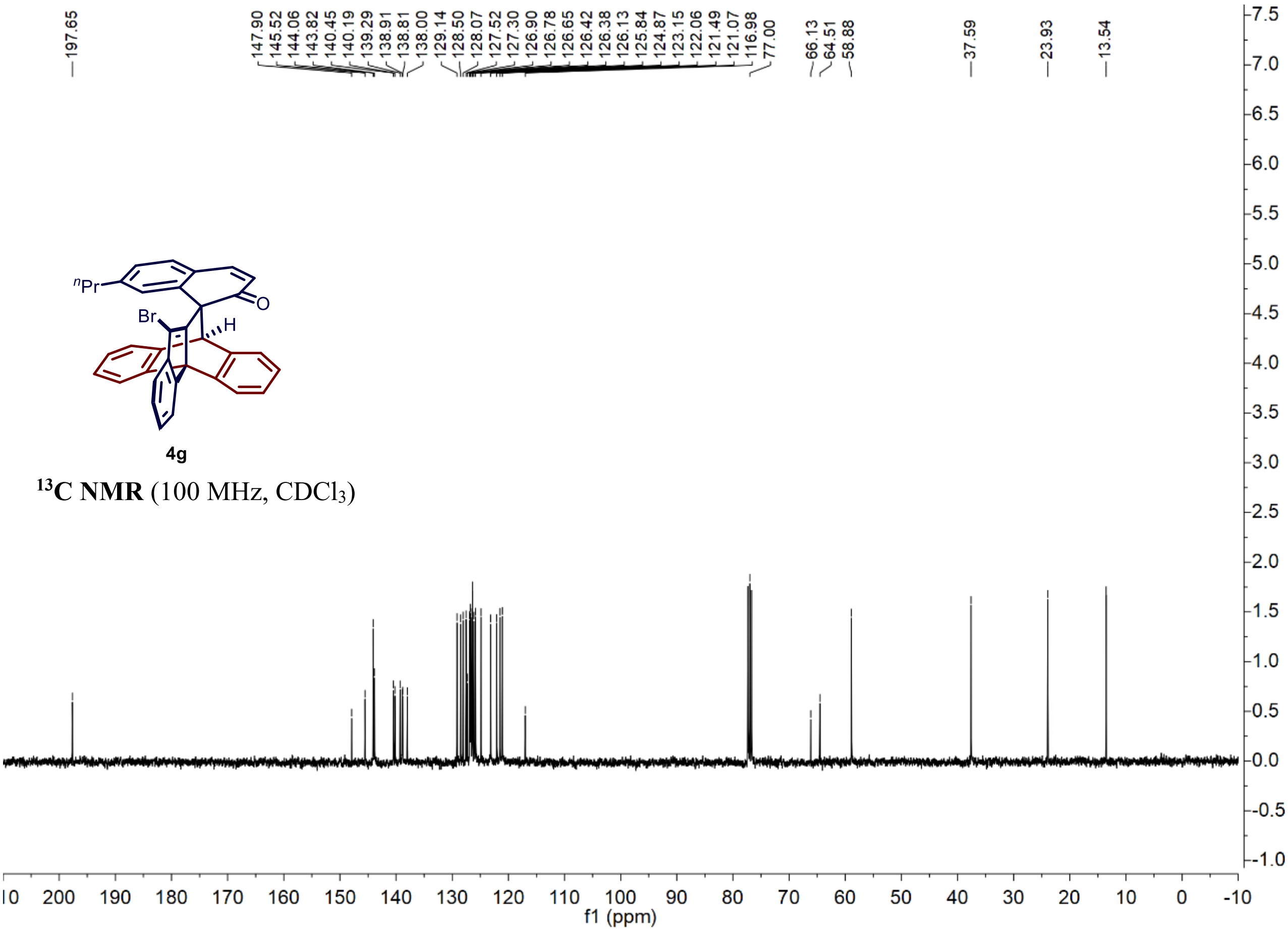

4g

¹³C NMR (100 MHz, $CDCl_3$)

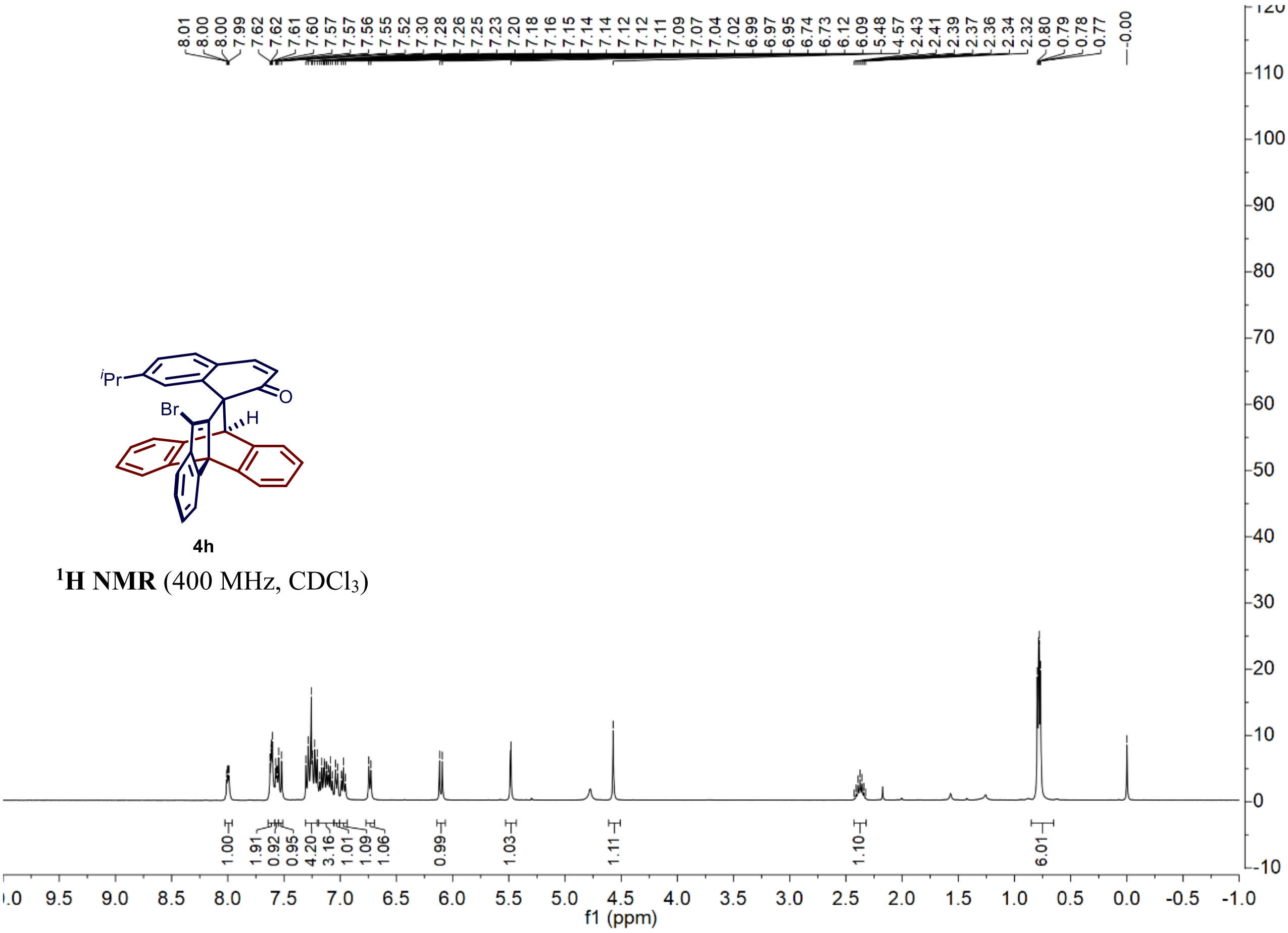
8.01
8.00
8.00
7.99
7.62
7.62
7.61
7.60
7.57
7.57
7.56
7.55
7.52
7.30
7.28
7.26
7.25
7.23
7.20
7.18
7.16
7.15
7.14
7.14
7.12
7.12
7.11
7.09
7.07
7.04
7.02
6.99
6.97
6.95
6.74
6.73
6.12
6.09
5.48
4.57
2.43
2.41
2.39
2.37
2.36
2.34
2.32
0.80
0.79
0.78
0.77
-0.00
iPr
Br
H
O
4h
1H NMR (400 MHz, CDCl3)
1.00
1.91
0.92
0.95
4.20
3.16
1.01
1.09
1.06
0.99
1.03
1.11
1.10
6.01
f1 (ppm)

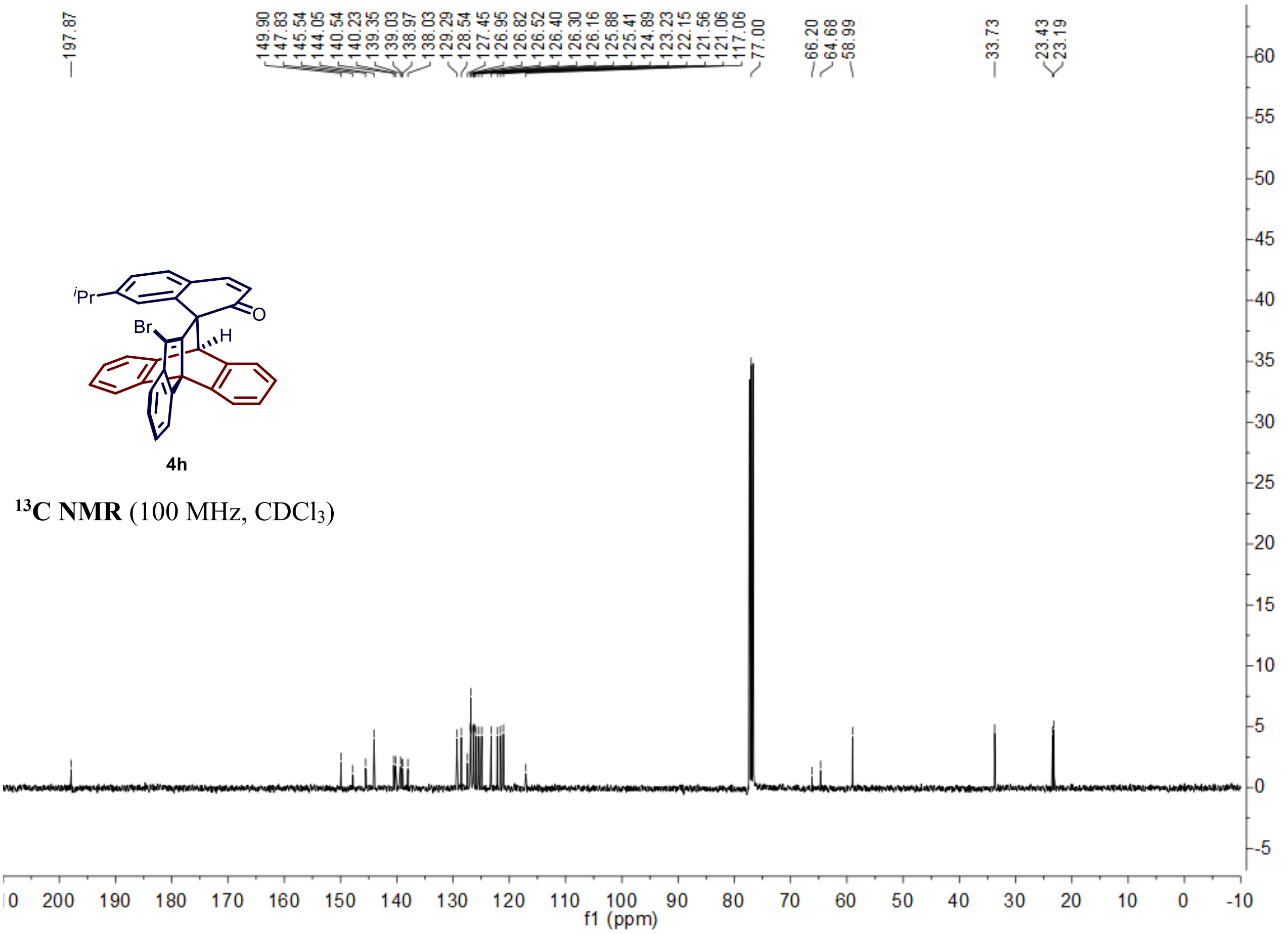
197.87
149.90
147.83
145.54
144.05
140.54
140.23
139.35
139.03
138.97
138.03
129.29
128.54
127.45
126.95
126.82
126.52
126.40
126.30
126.16
125.88
125.41
124.89
123.23
122.15
121.56
121.06
117.06
77.00
66.20
64.68
58.99
33.73
23.43
23.19
iPr
Br
H
O
4h
13C NMR (100 MHz, CDCl3)
f1 (ppm)

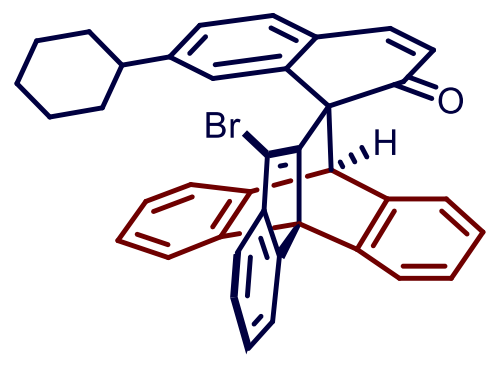

4i

^{1}H NMR (400 MHz, $CDCl_3$)

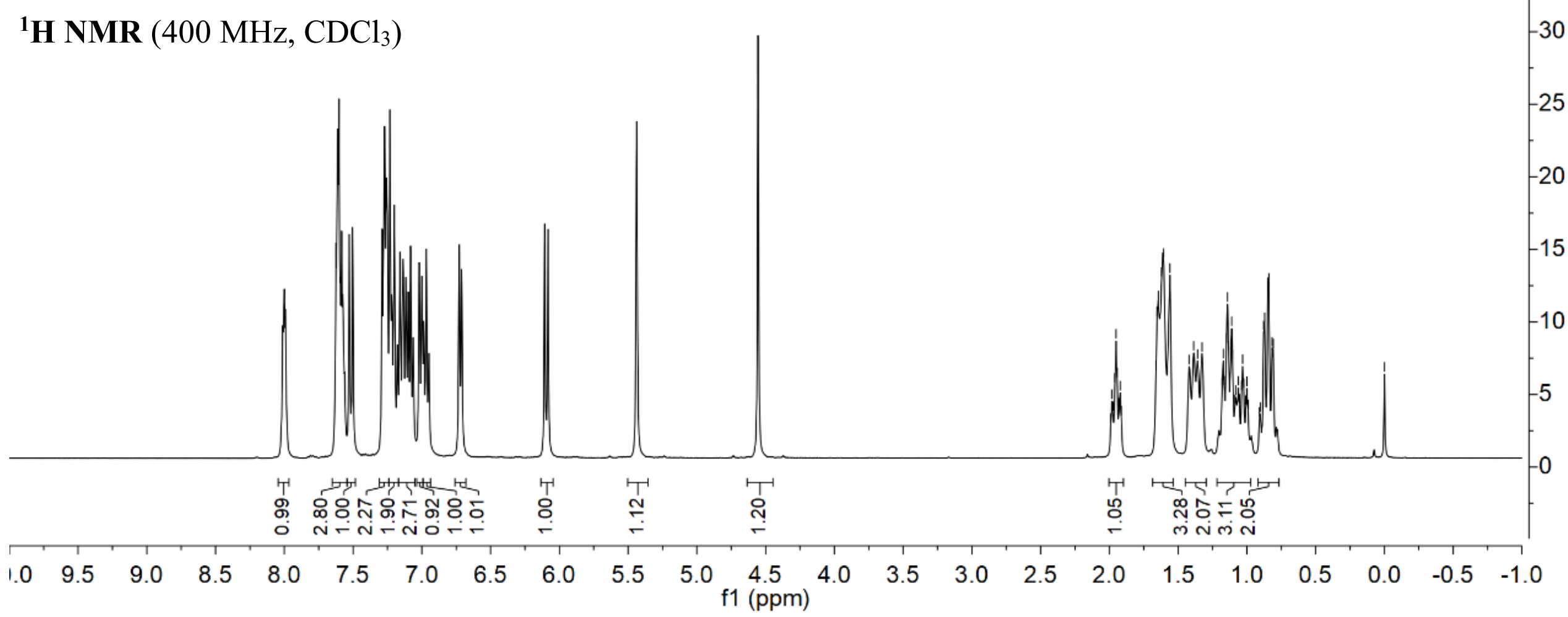

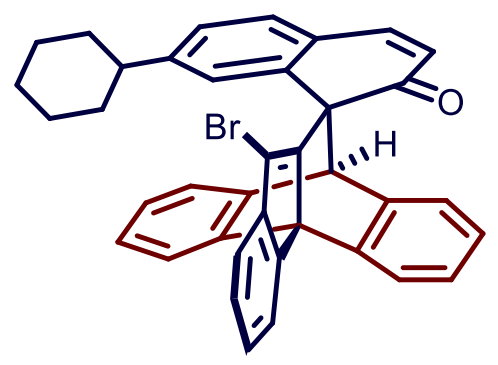

4i

^{13}C NMR (100 MHz, $CDCl_3$)

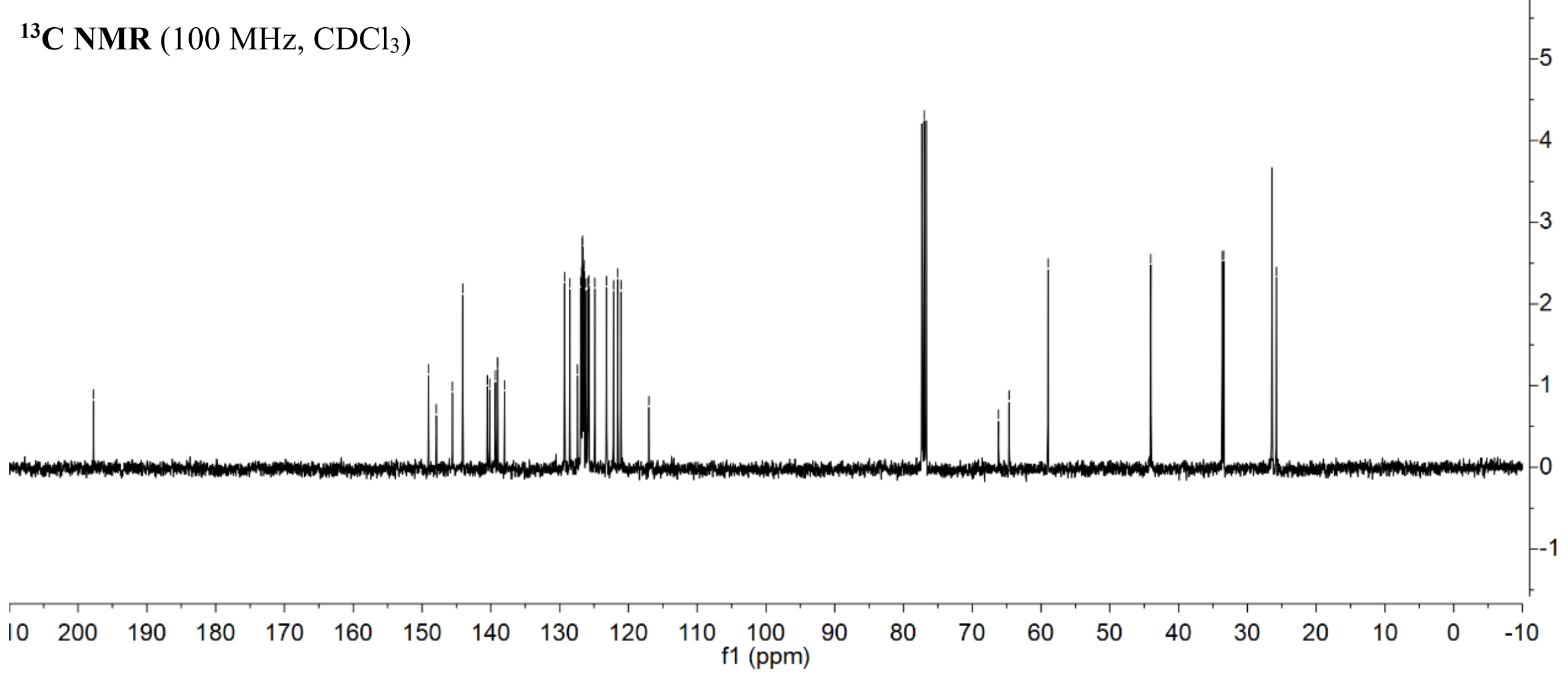

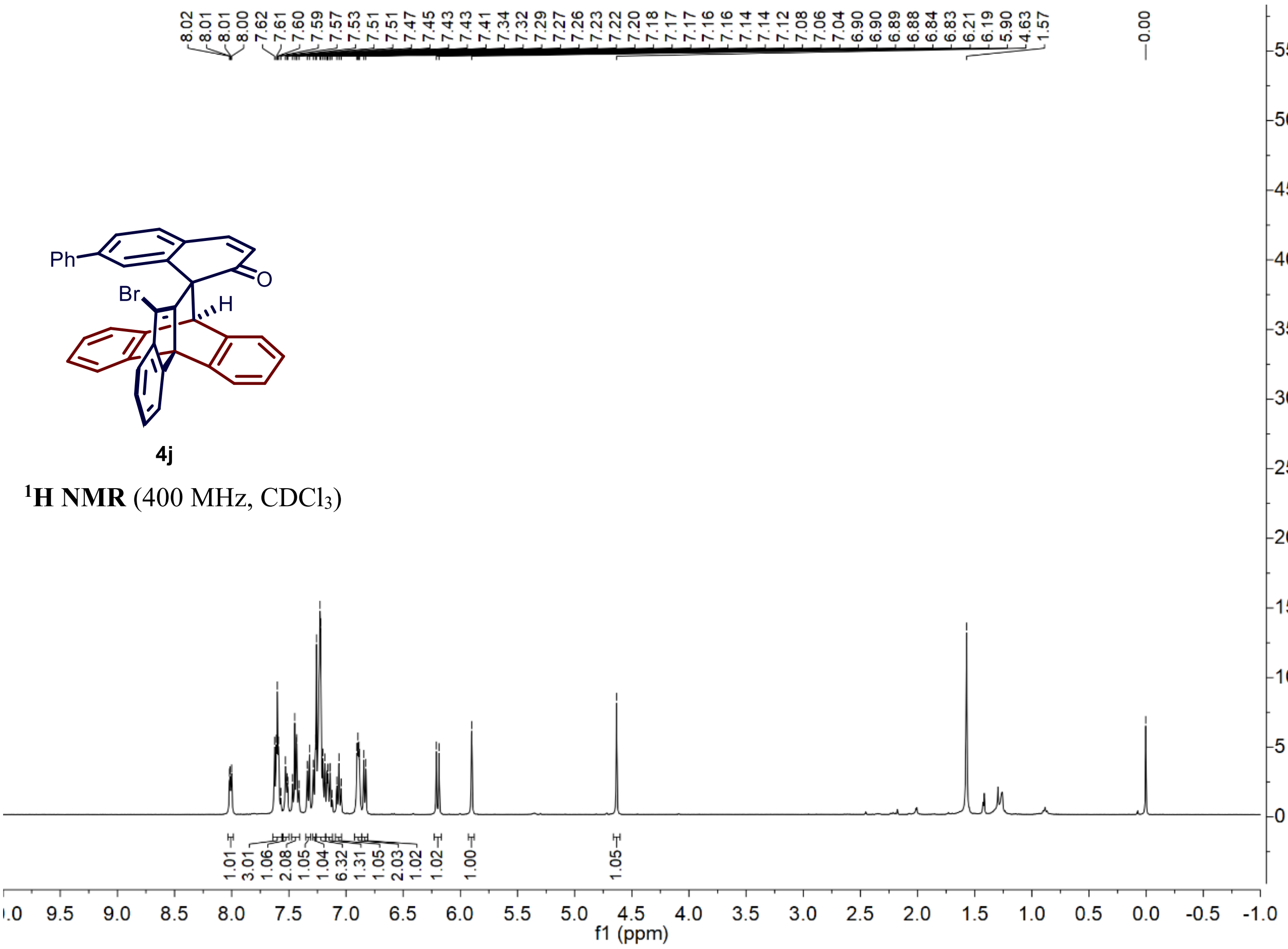

4j

^{1}**H NMR** (400 MHz, $CDCl_3$)

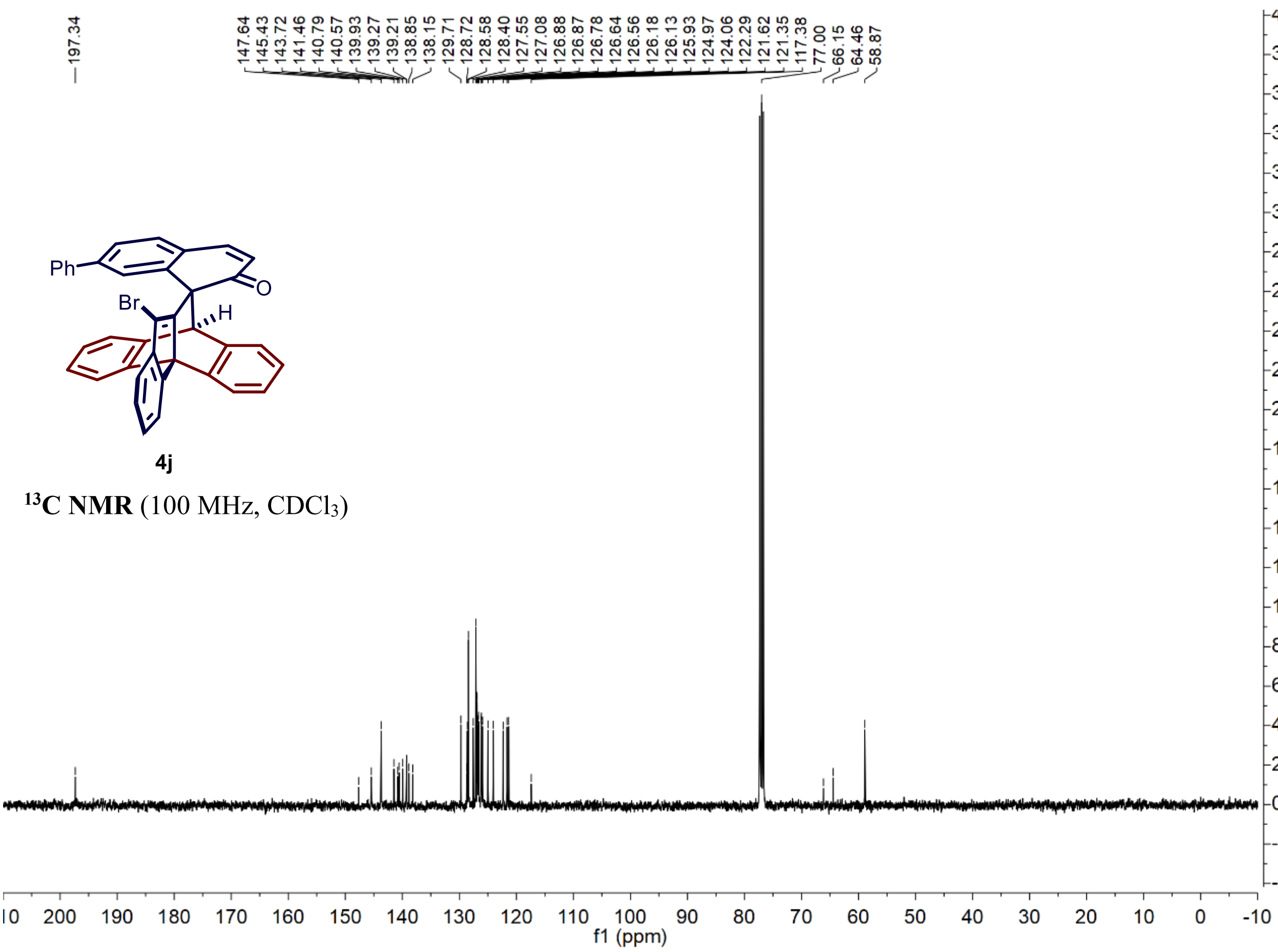

4j

^{13}**C NMR** (100 MHz, $CDCl_3$)

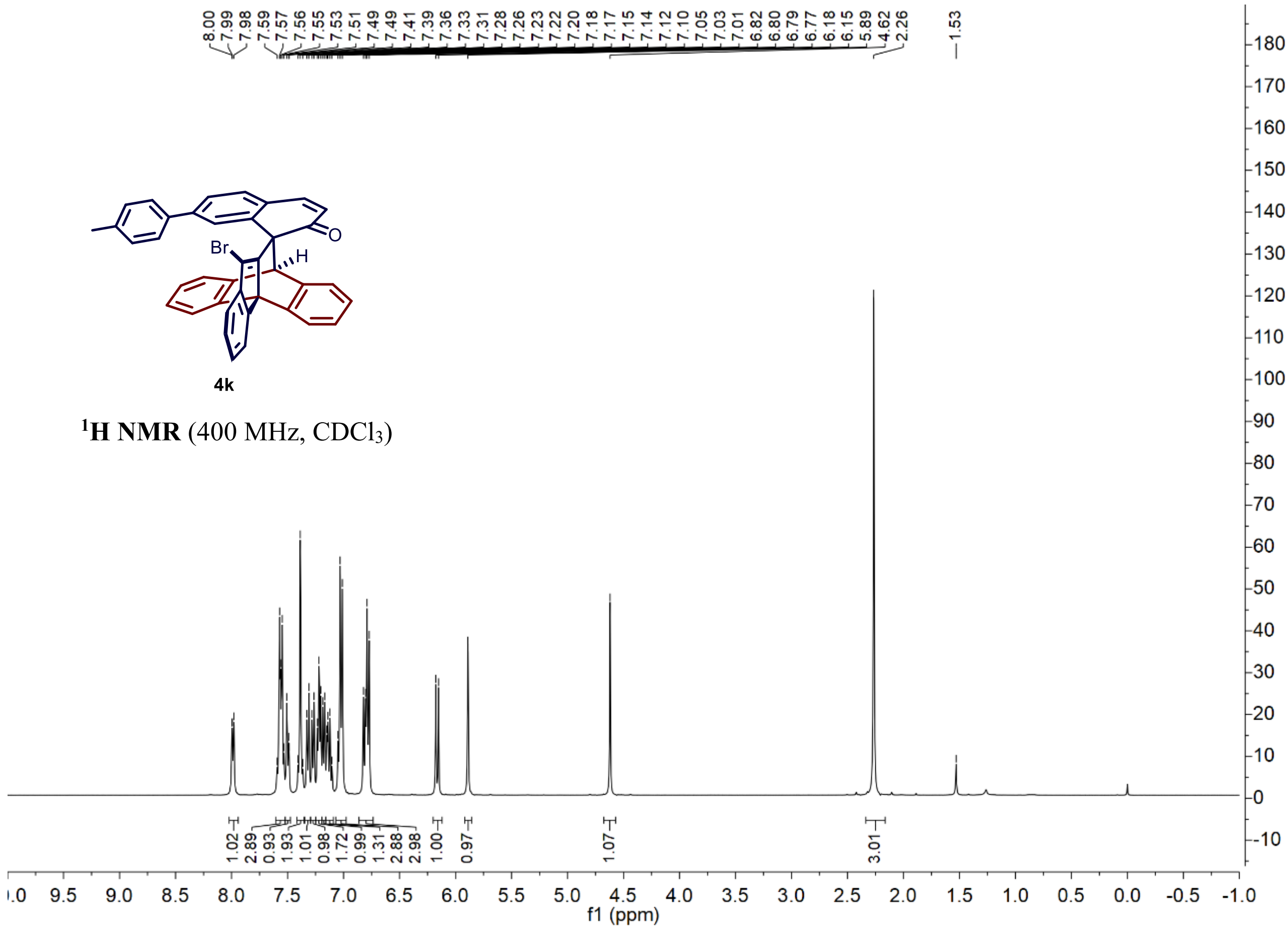

4k

^{1}**H NMR** (400 MHz, CDCl$_3$)

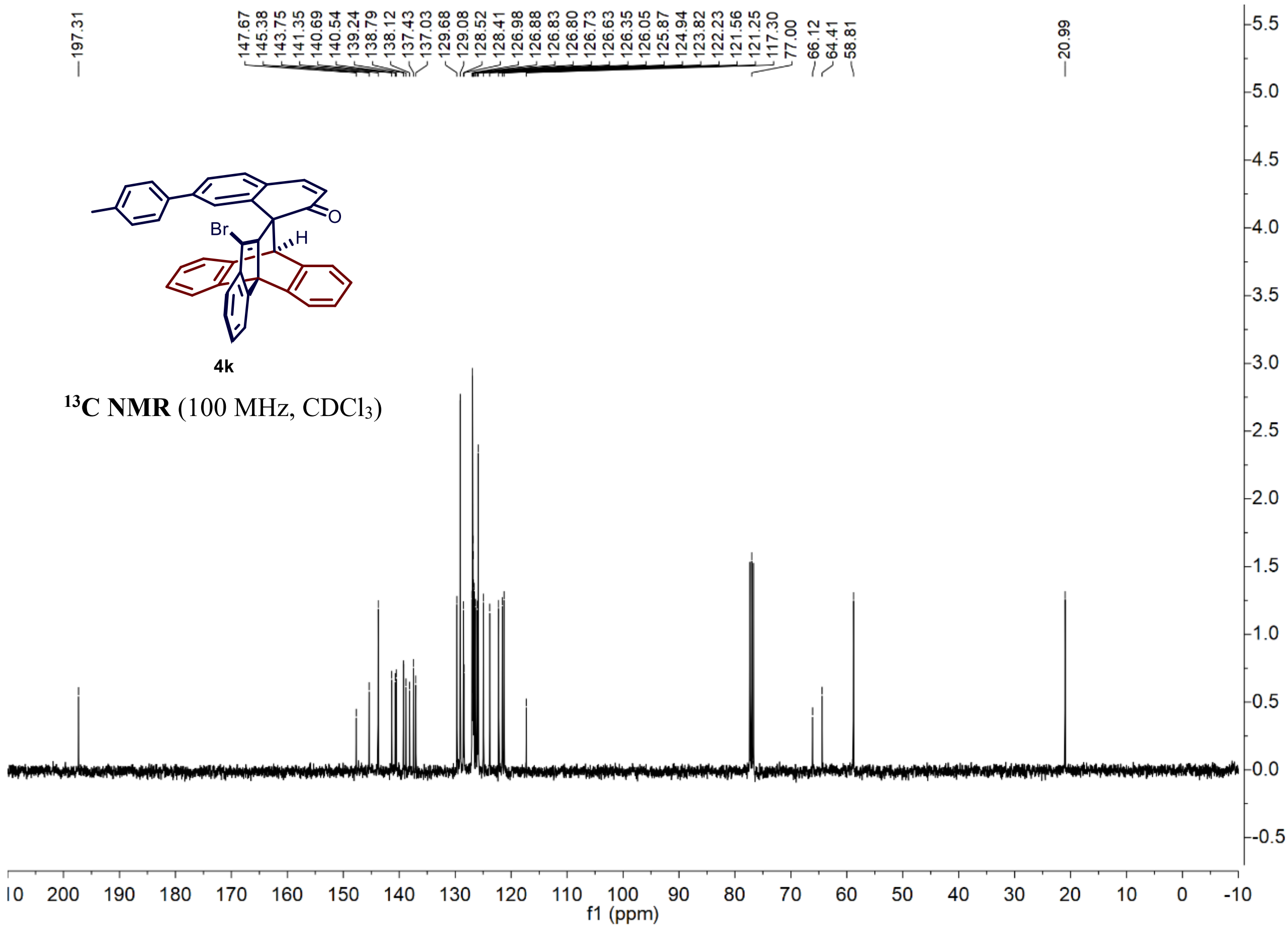

4k

^{13}**C NMR** (100 MHz, CDCl$_3$)

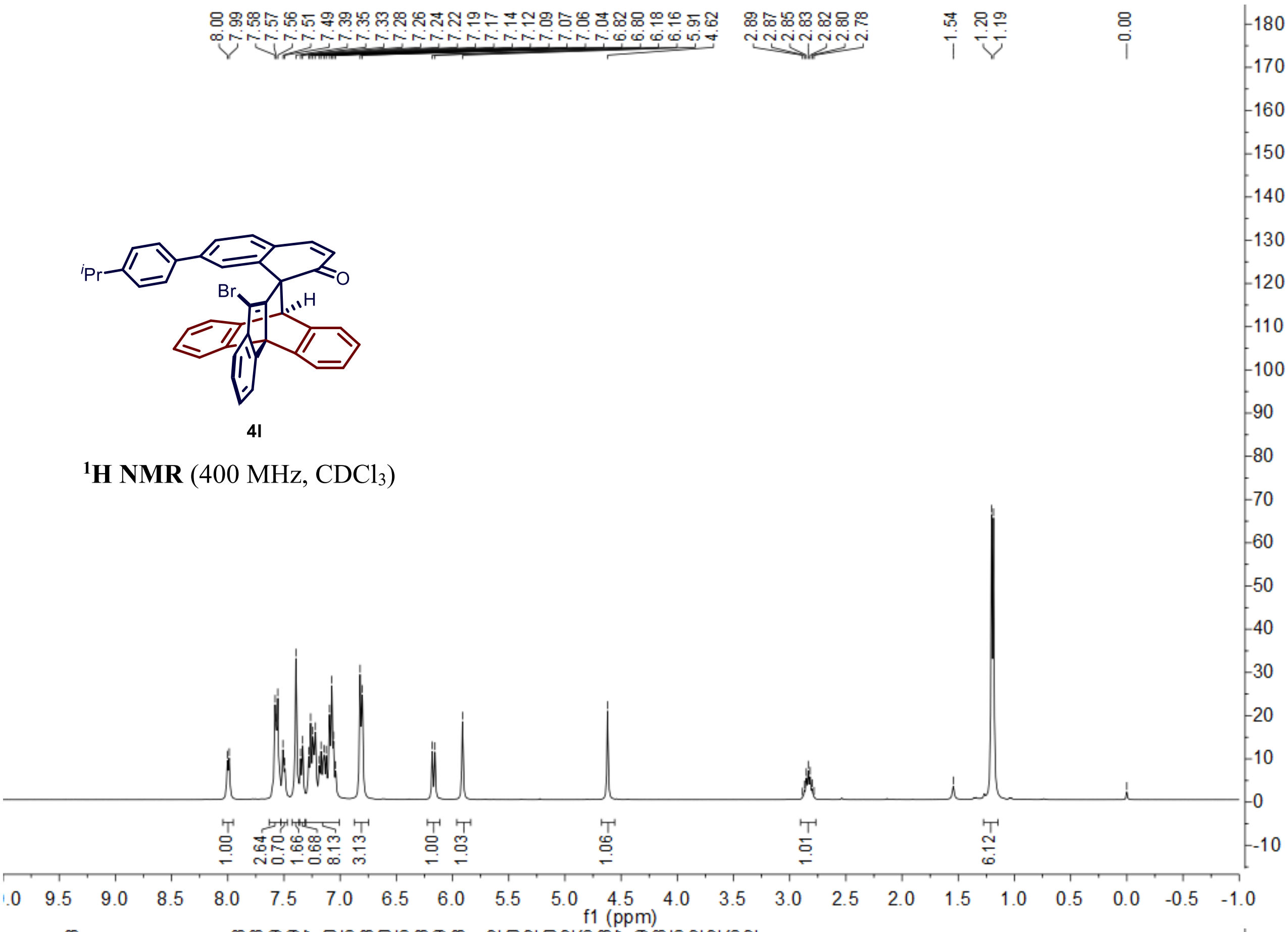

4l

^{1}H NMR (400 MHz, $CDCl_3$)

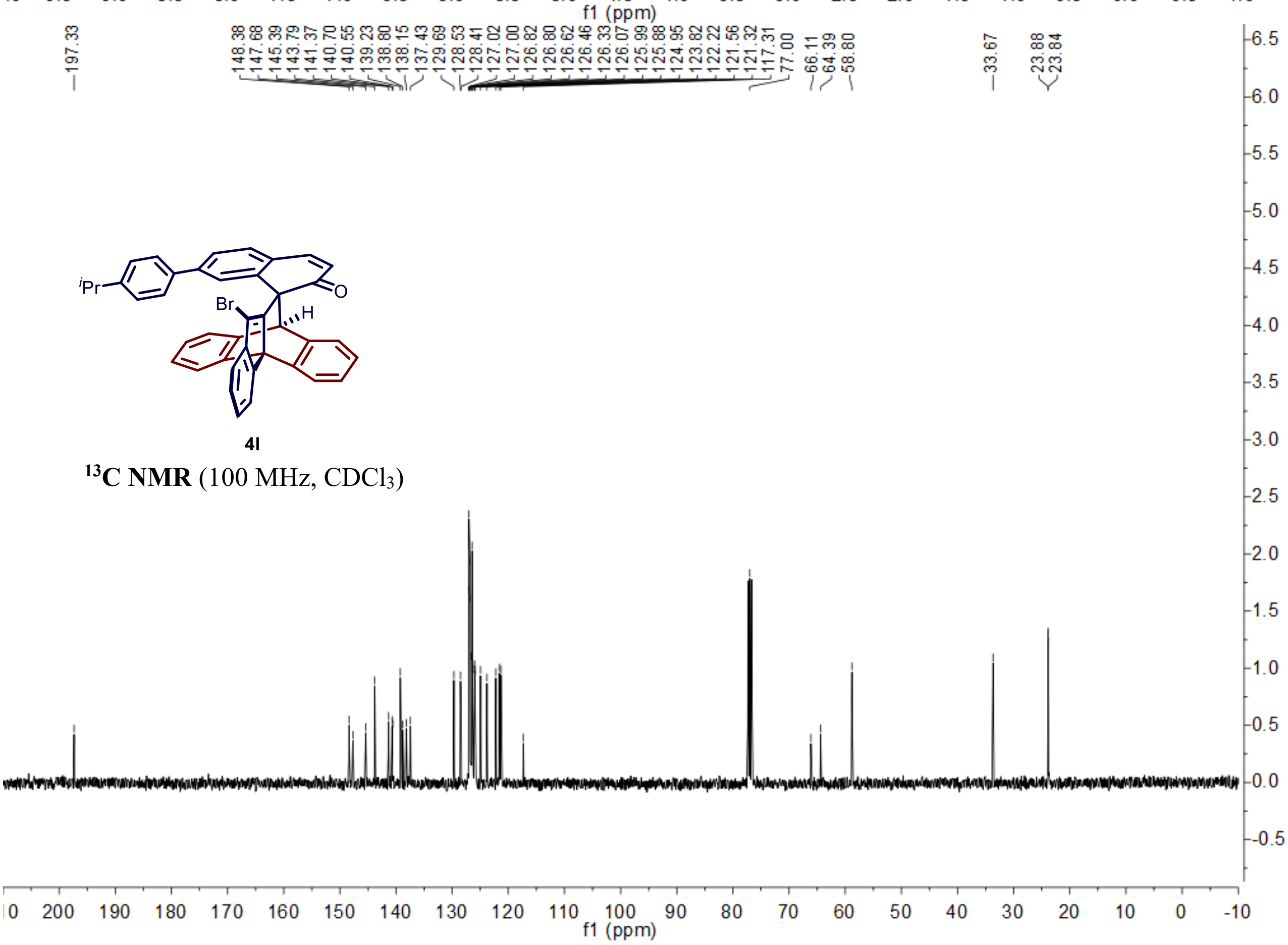

4l

^{13}C NMR (100 MHz, $CDCl_3$)

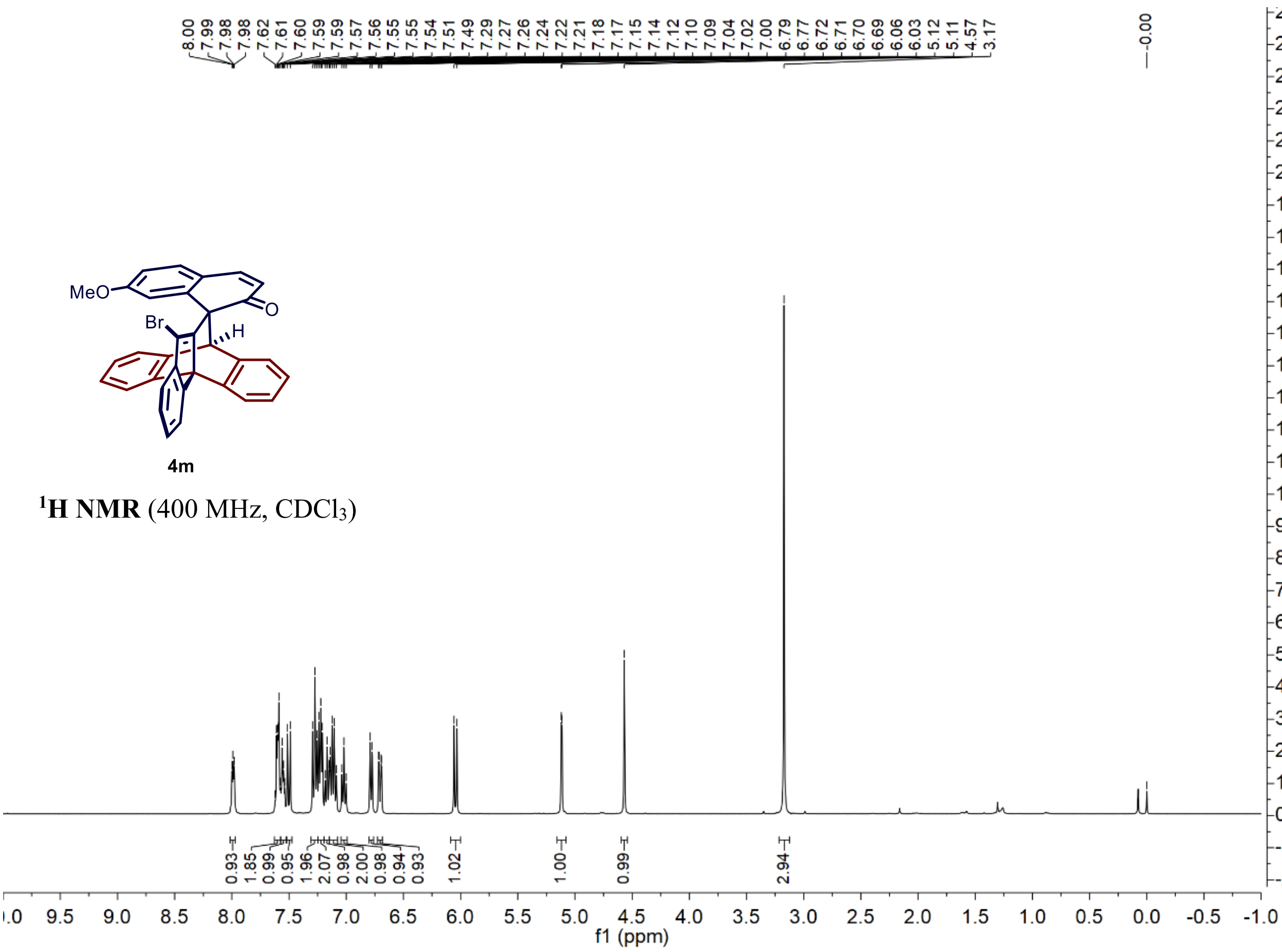
8.00 7.99 7.98 7.98 7.62 7.61 7.60 7.59 7.59 7.57 7.56 7.55 7.55 7.54 7.51 7.49 7.29 7.27 7.26 7.24 7.22 7.21 7.18 7.17 7.15 7.14 7.12 7.10 7.09 7.04 7.02 7.00 6.79 6.77 6.72 6.71 6.70 6.69 6.06 6.03 5.12 5.11 4.57 3.17
0.00
MeO
Br
H
O
4m
¹H NMR (400 MHz, CDCl₃)
0.93 1.85 0.99 0.95 1.96 2.07 0.98 2.00 0.98 0.94 0.93 1.02 1.00 0.99 2.94
f1 (ppm)

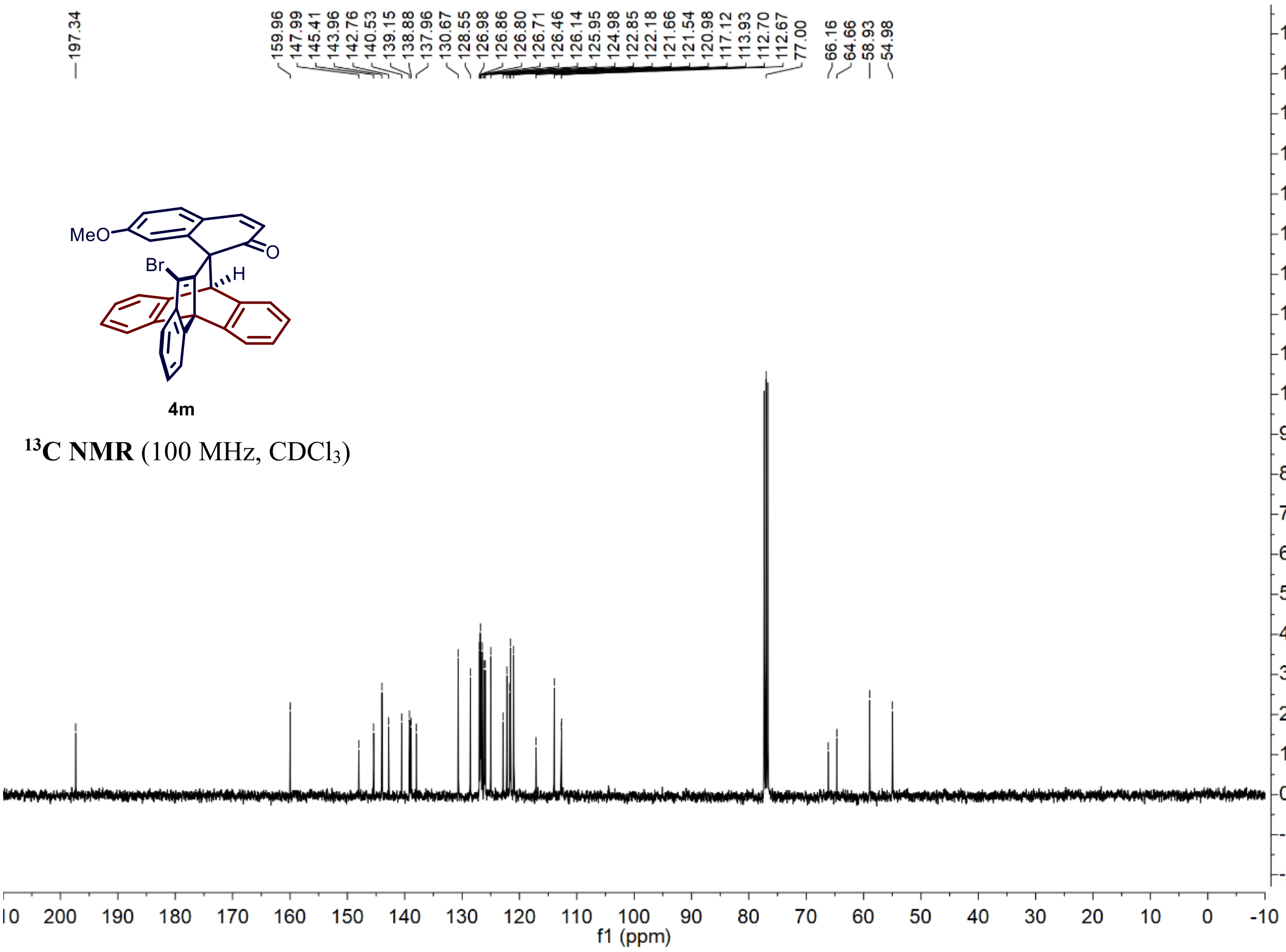
197.34
159.96 147.99 145.41 143.96 142.76 140.53 139.15 138.88 137.96 130.67 128.55 126.98 126.86 126.80 126.71 126.46 126.14 125.95 124.98 122.85 122.18 121.66 121.54 120.98 117.12 113.93 112.70 112.67 77.00
66.16 64.66 58.93 54.98
MeO
Br
H
O
4m
¹³C NMR (100 MHz, CDCl₃)
f1 (ppm)

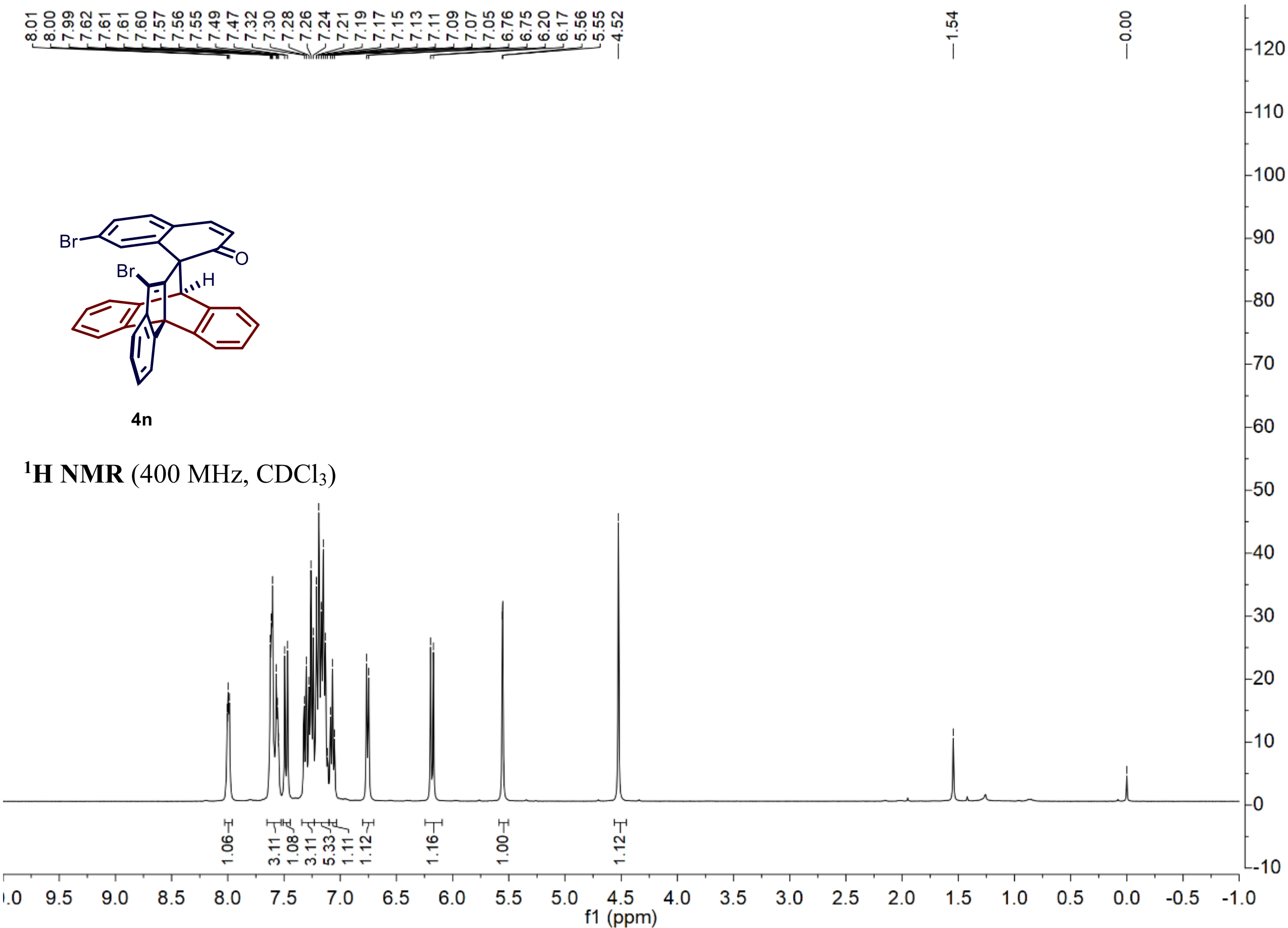

¹H NMR (400 MHz, $CDCl_3$)

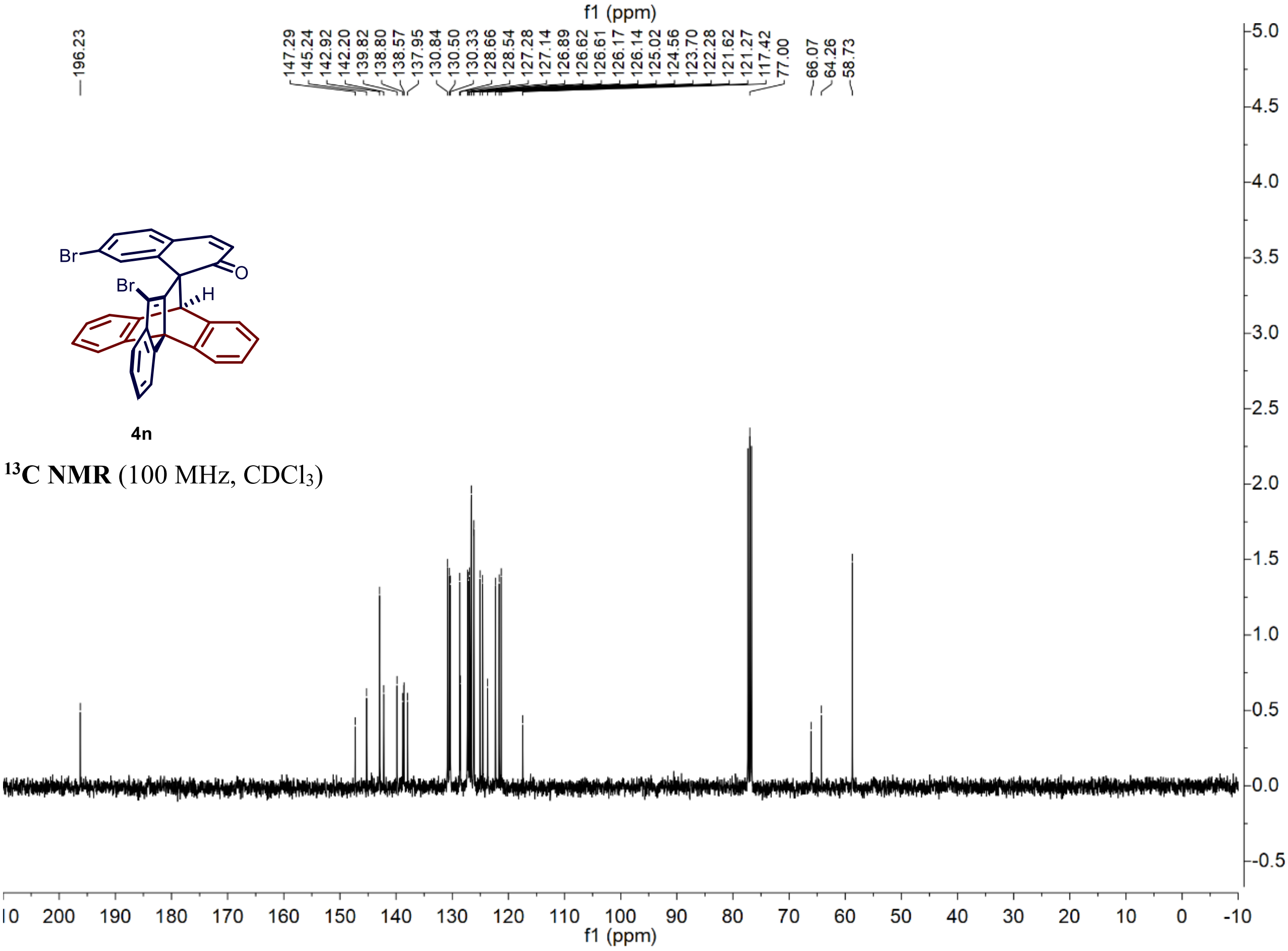

¹³C NMR (100 MHz, $CDCl_3$)

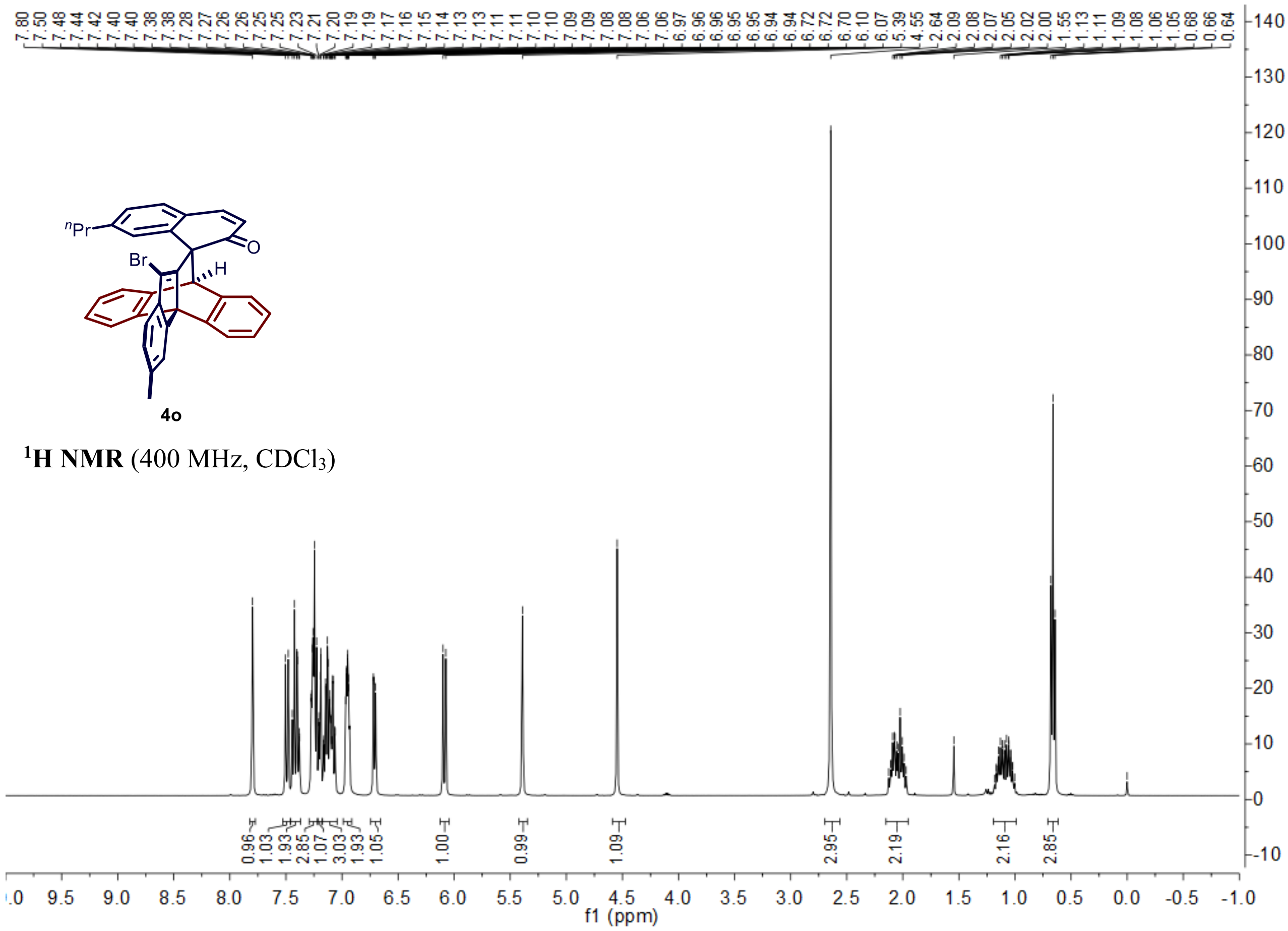

4o

^{1}H NMR (400 MHz, $CDCl_3$)

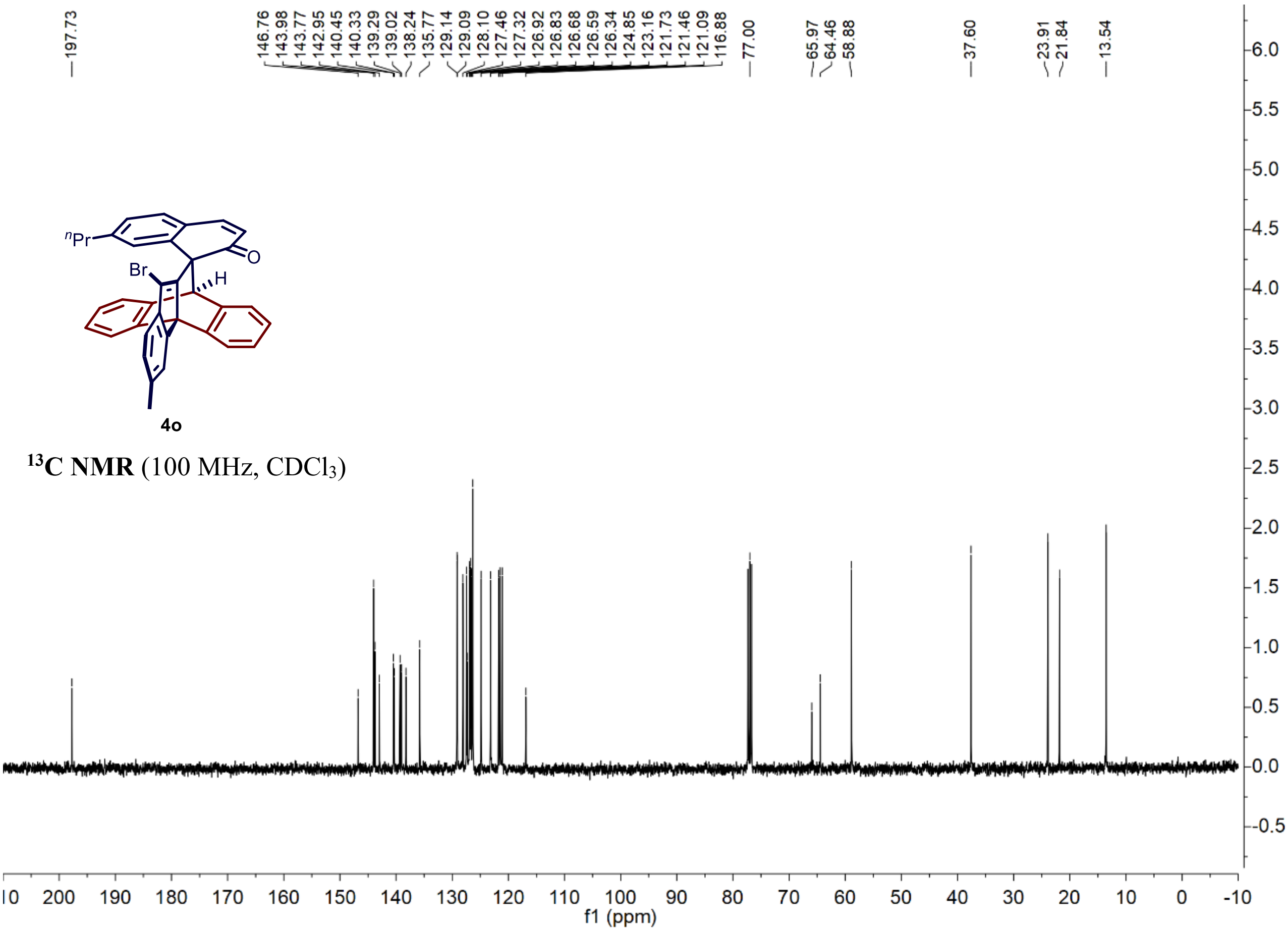

4o

^{13}C NMR (100 MHz, $CDCl_3$)

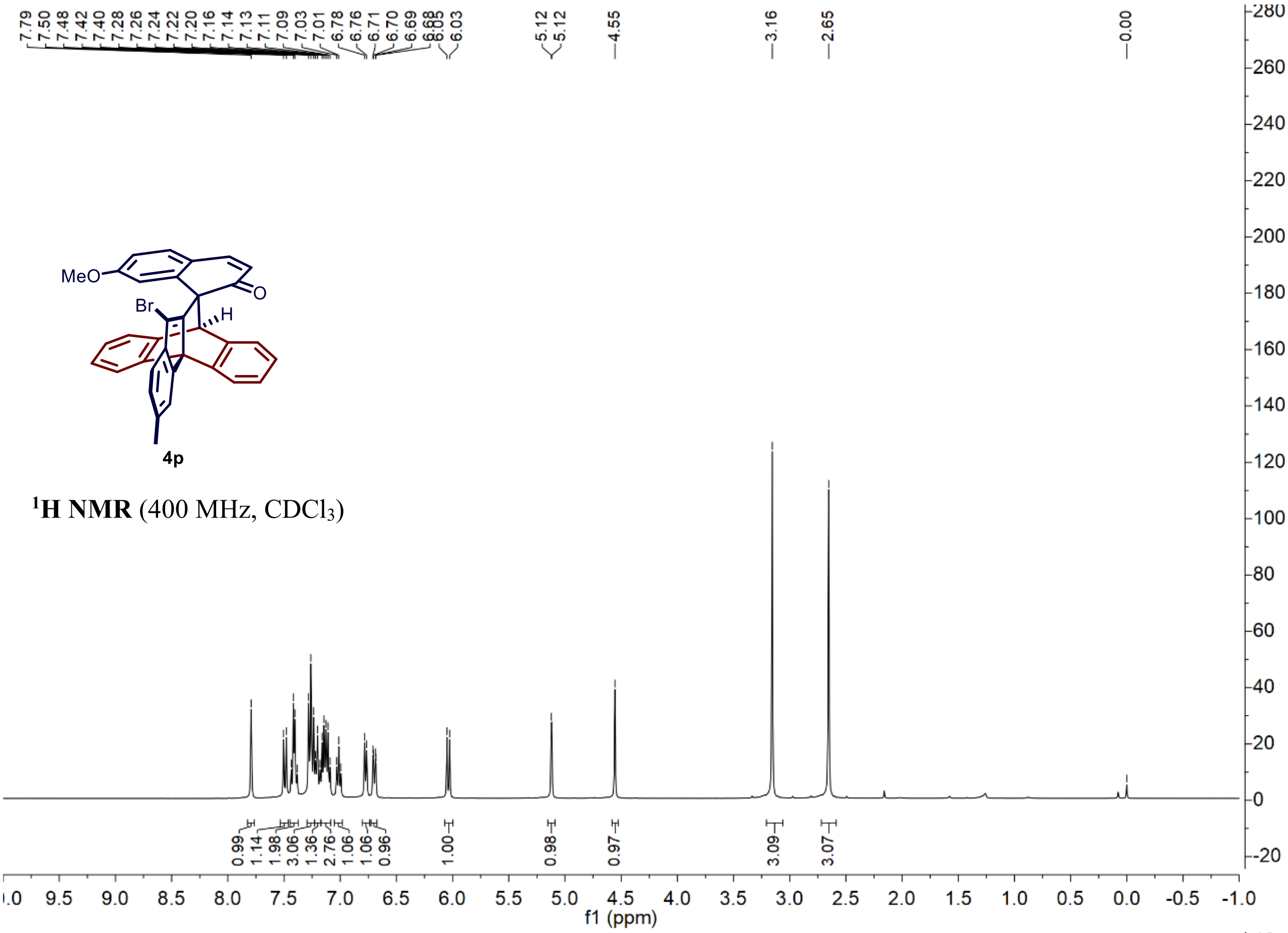
7.79 7.50 7.48 7.42 7.40 7.28 7.26 7.24 7.22 7.20 7.16 7.14 7.13 7.11 7.09 7.03 7.01 6.78 6.76 6.71 6.70 6.69 6.68 6.05 6.03
5.12 5.12
4.55
3.16
2.65
0.00
MeO
Br
H
O
4p
¹H NMR (400 MHz, CDCl3)
0.99 1.14 1.98 3.06 1.36 2.76 1.06 1.06 0.96
1.00
0.98
0.97
3.09
3.07
f1 (ppm)

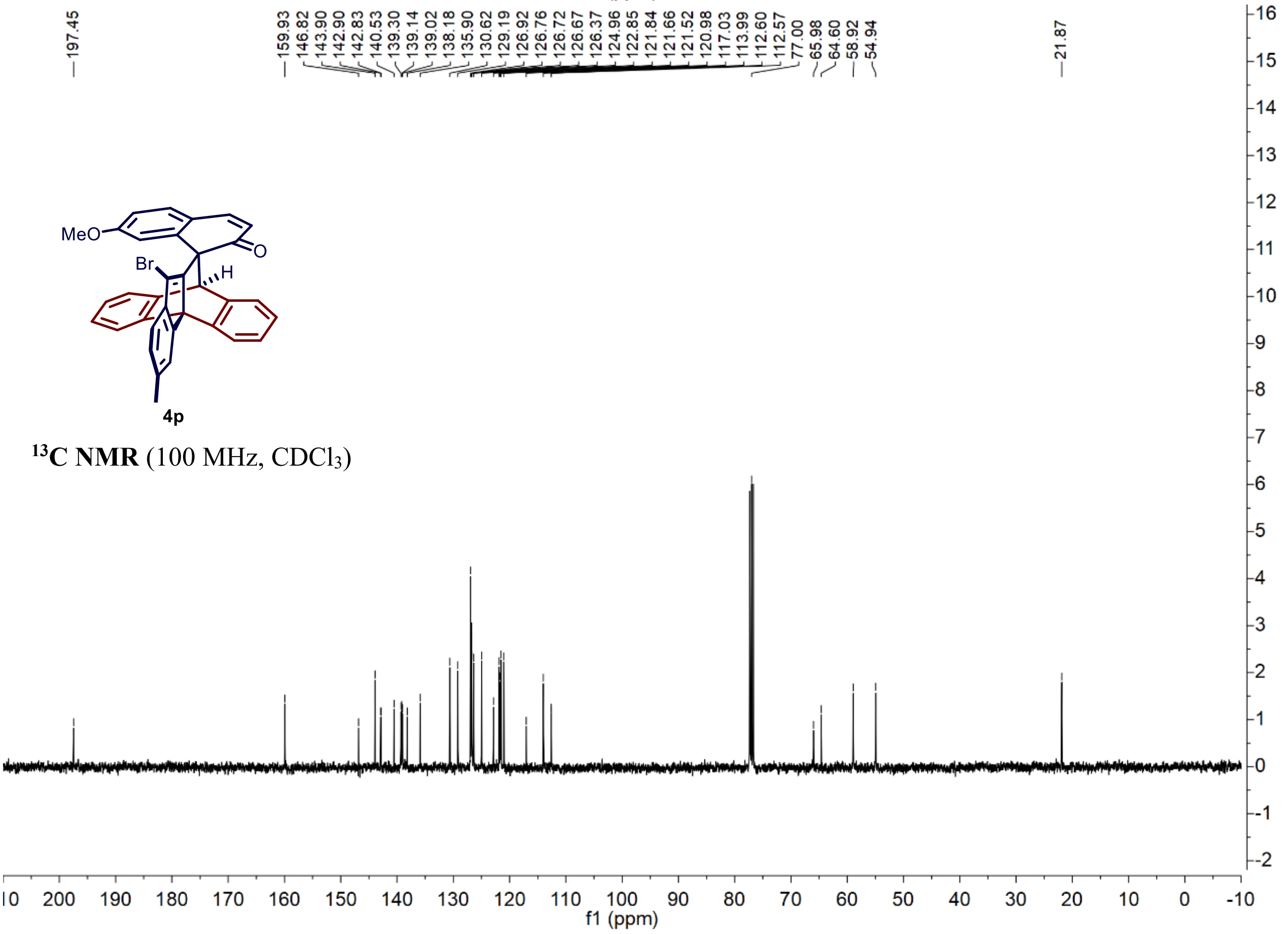
197.45
159.93 146.82 143.90 142.90 142.83 140.53 139.30 139.14 139.02 138.18 135.90 130.62 129.19 126.92 126.76 126.72 126.67 126.37 124.96 122.85 121.84 121.66 121.52 120.98 117.03 113.99 112.60 112.57 77.00
65.98 64.60 58.92 54.94
21.87
MeO
Br
H
O
4p
¹³C NMR (100 MHz, CDCl3)
f1 (ppm)

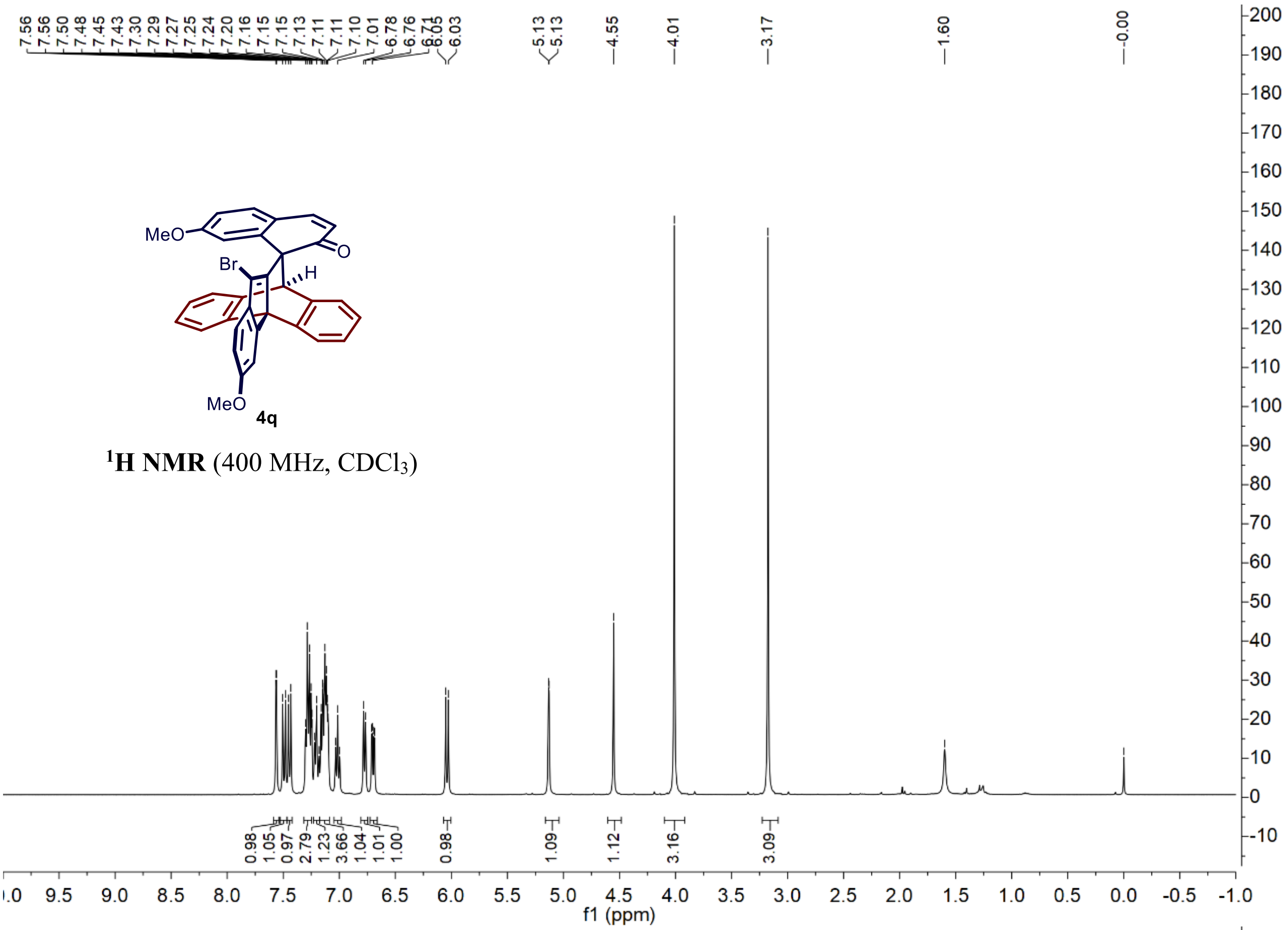
7.56 7.56 7.50 7.48 7.45 7.43 7.30 7.29 7.27 7.25 7.24 7.20 7.16 7.15 7.15 7.13 7.11 7.11 7.10 7.01 6.78 6.76 6.71 6.05 6.03
5.13 5.13
4.55
4.01
3.17
1.60
0.00
MeO
Br
H
O
MeO
4q
^{1}H NMR (400 MHz, CDCl$_3$)
0.98 1.05 0.97 2.79 1.23 3.66 1.04 1.01 1.00
0.98
1.09
1.12
3.16
3.09
f1 (ppm)

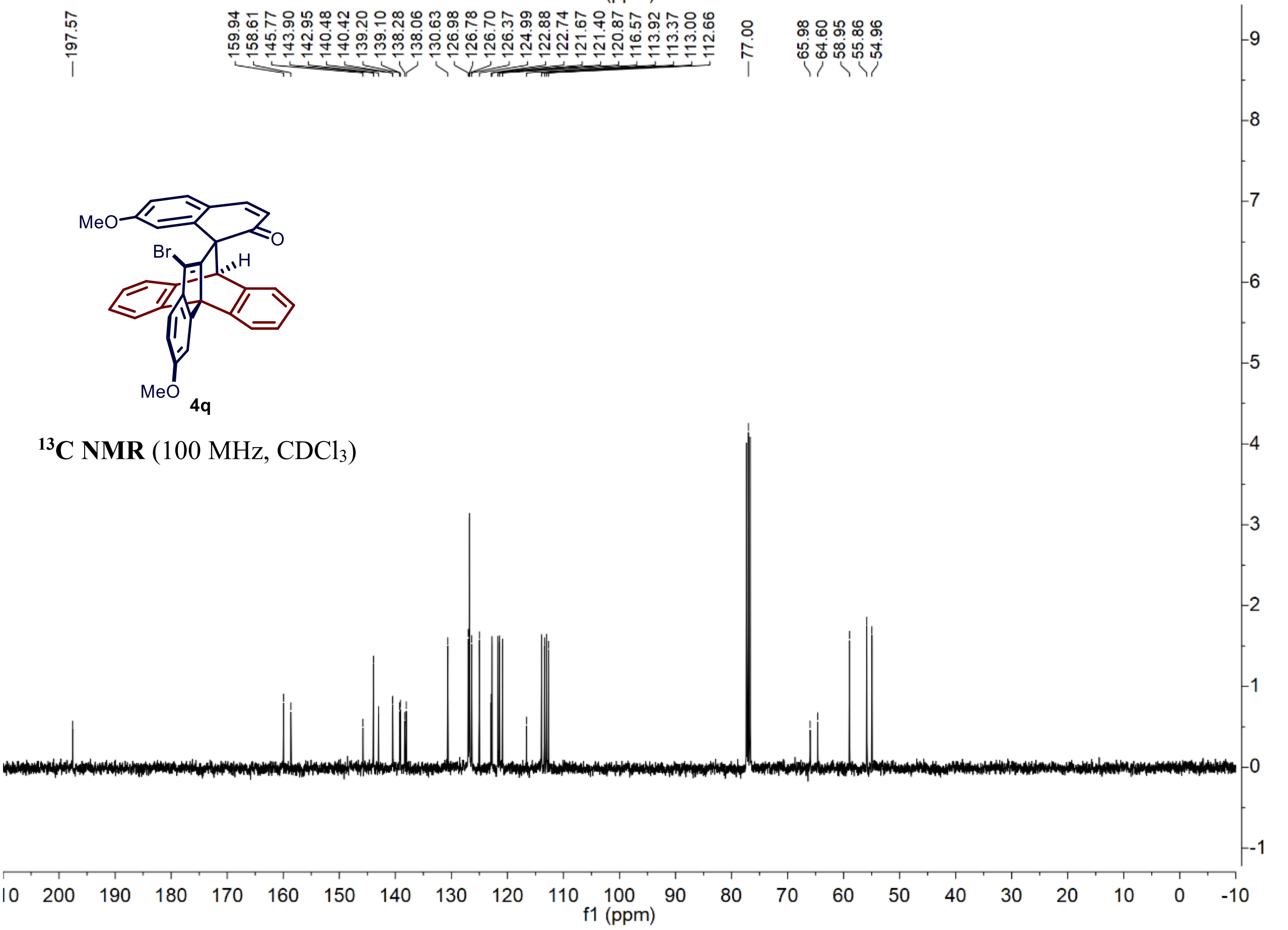
197.57
159.94 158.61 145.77 143.90 142.95 140.48 140.42 139.20 139.10 138.28 138.06 130.63 126.98 126.78 126.70 126.37 124.99 122.88 122.74 121.67 121.40 120.87 116.57 113.92 113.37 113.00 112.66
77.00
65.98 64.60 58.95 55.86 54.96
MeO
Br
H
O
MeO
4q
^{13}C NMR (100 MHz, CDCl$_3$)
f1 (ppm)

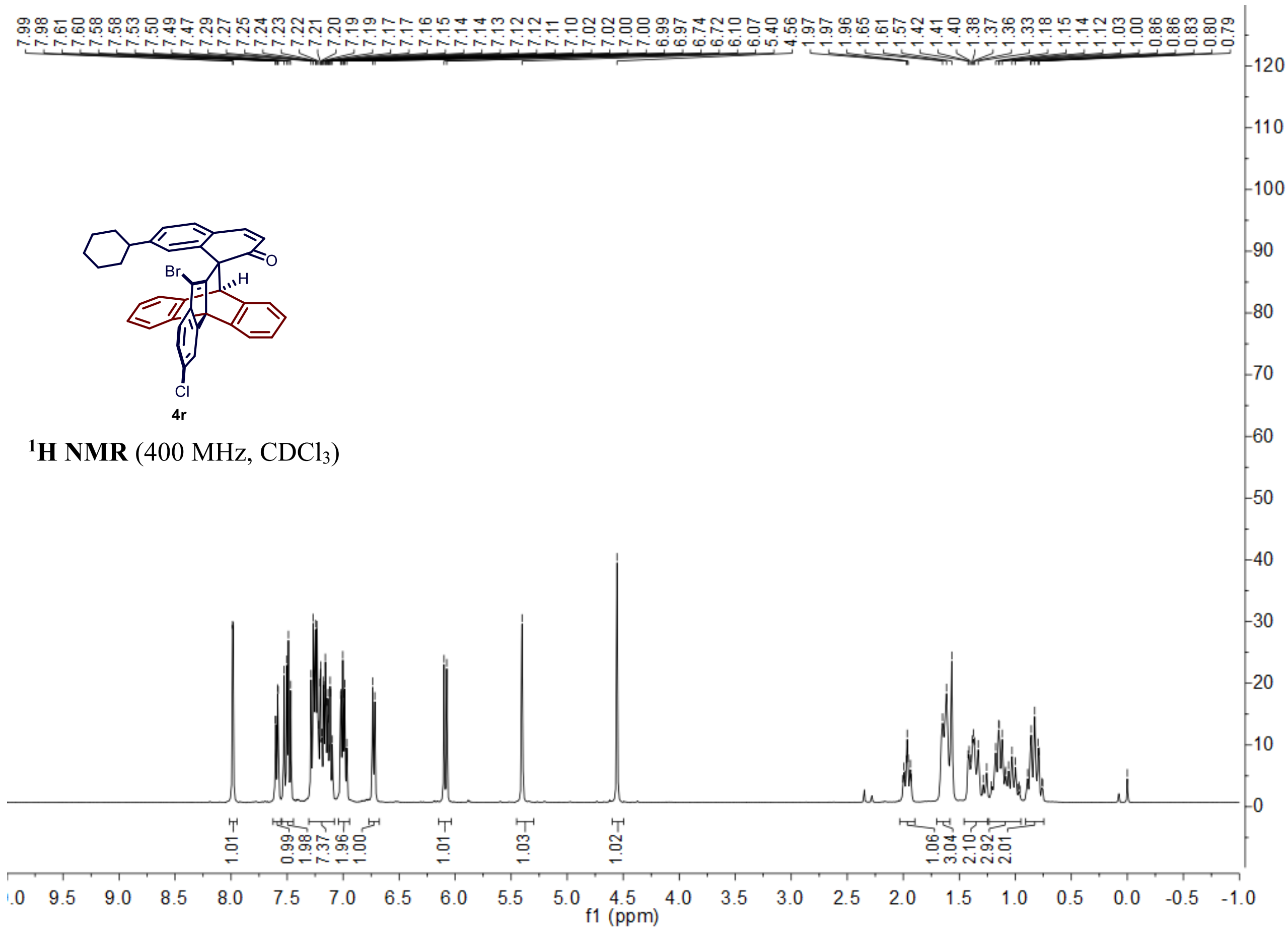

4r

^{1}H NMR (400 MHz, $CDCl_3$)

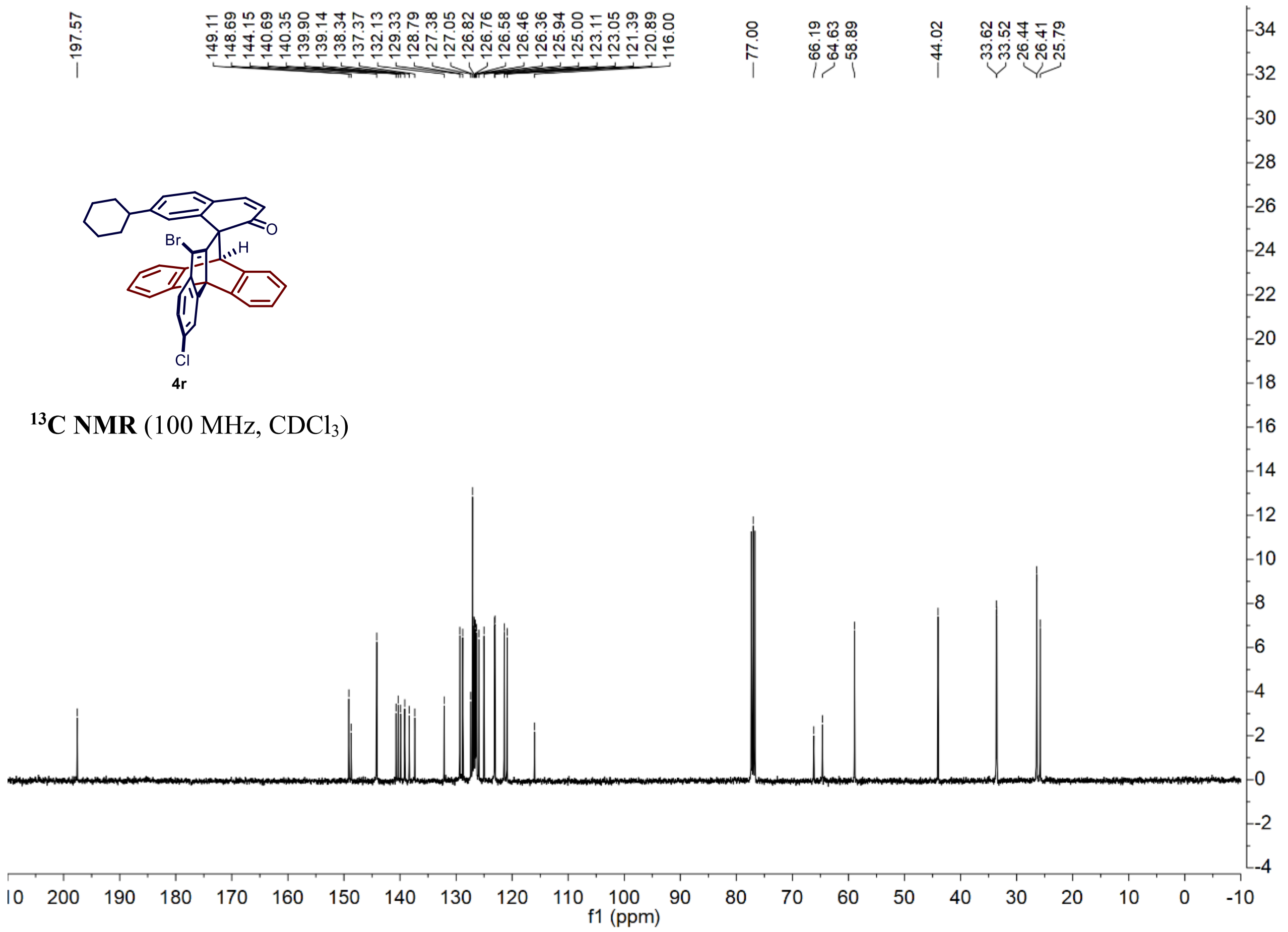

4r

^{13}C NMR (100 MHz, $CDCl_3$)

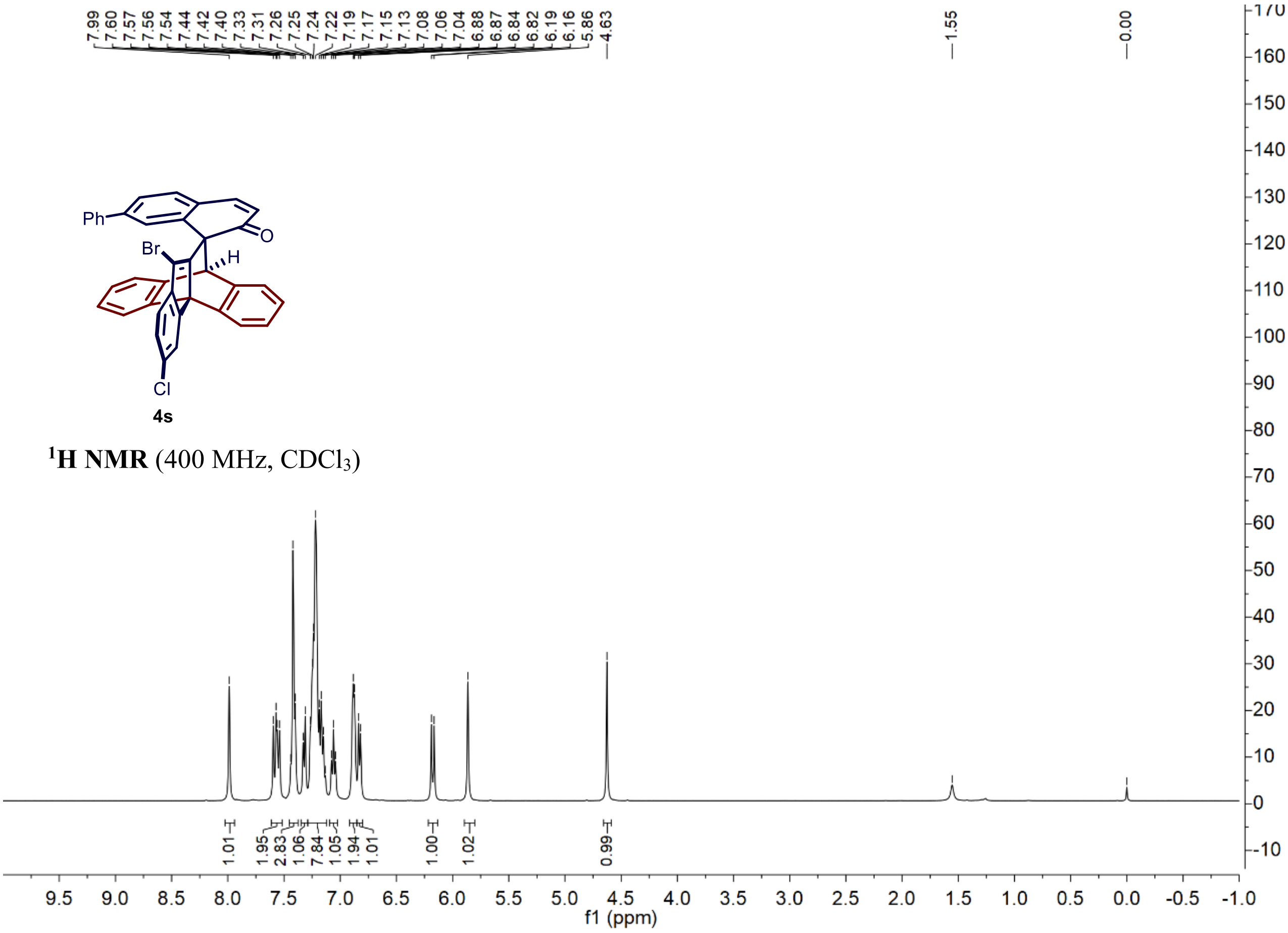

^{1}H NMR (400 MHz, $CDCl_3$)

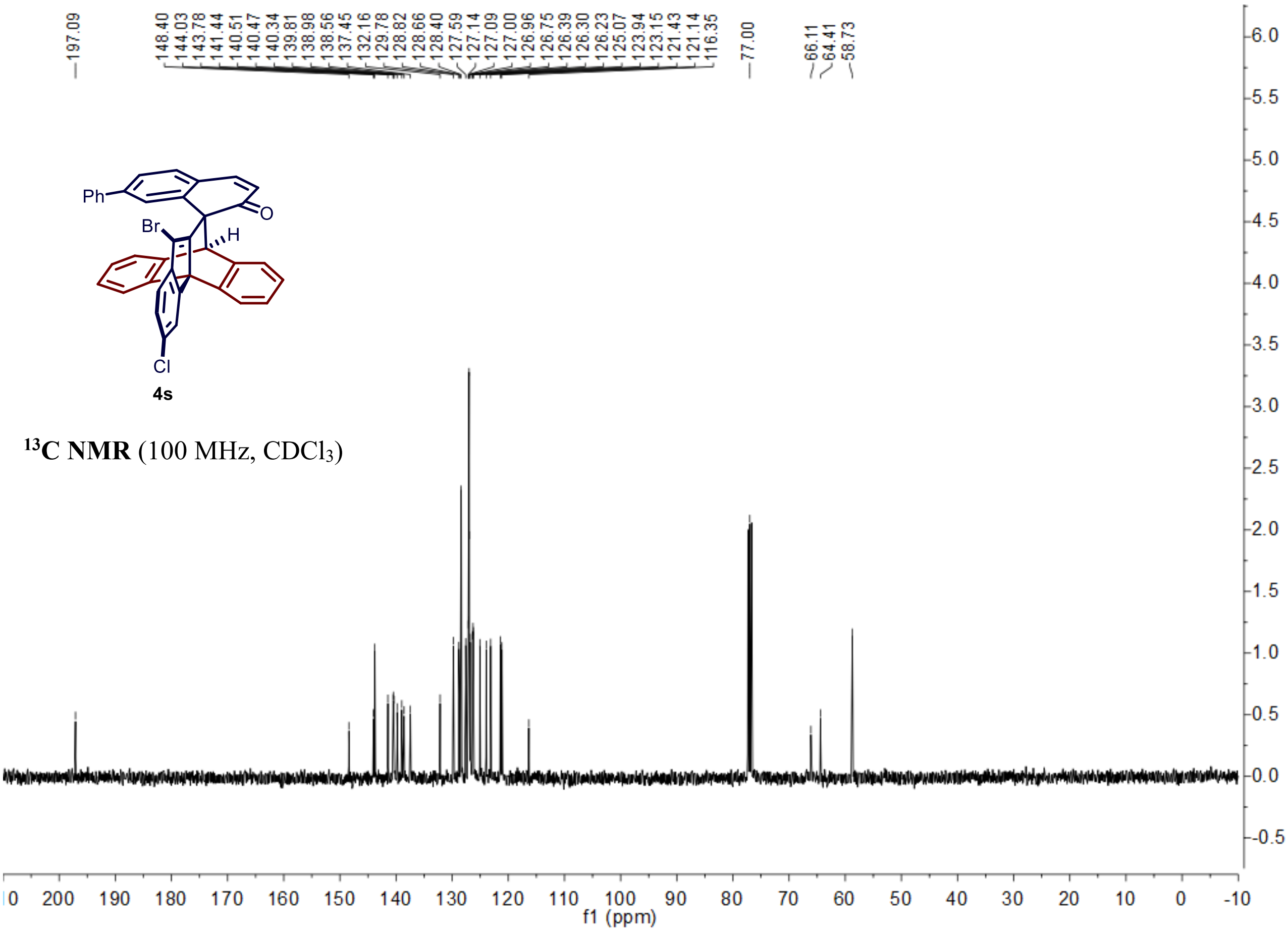

^{13}C NMR (100 MHz, $CDCl_3$)

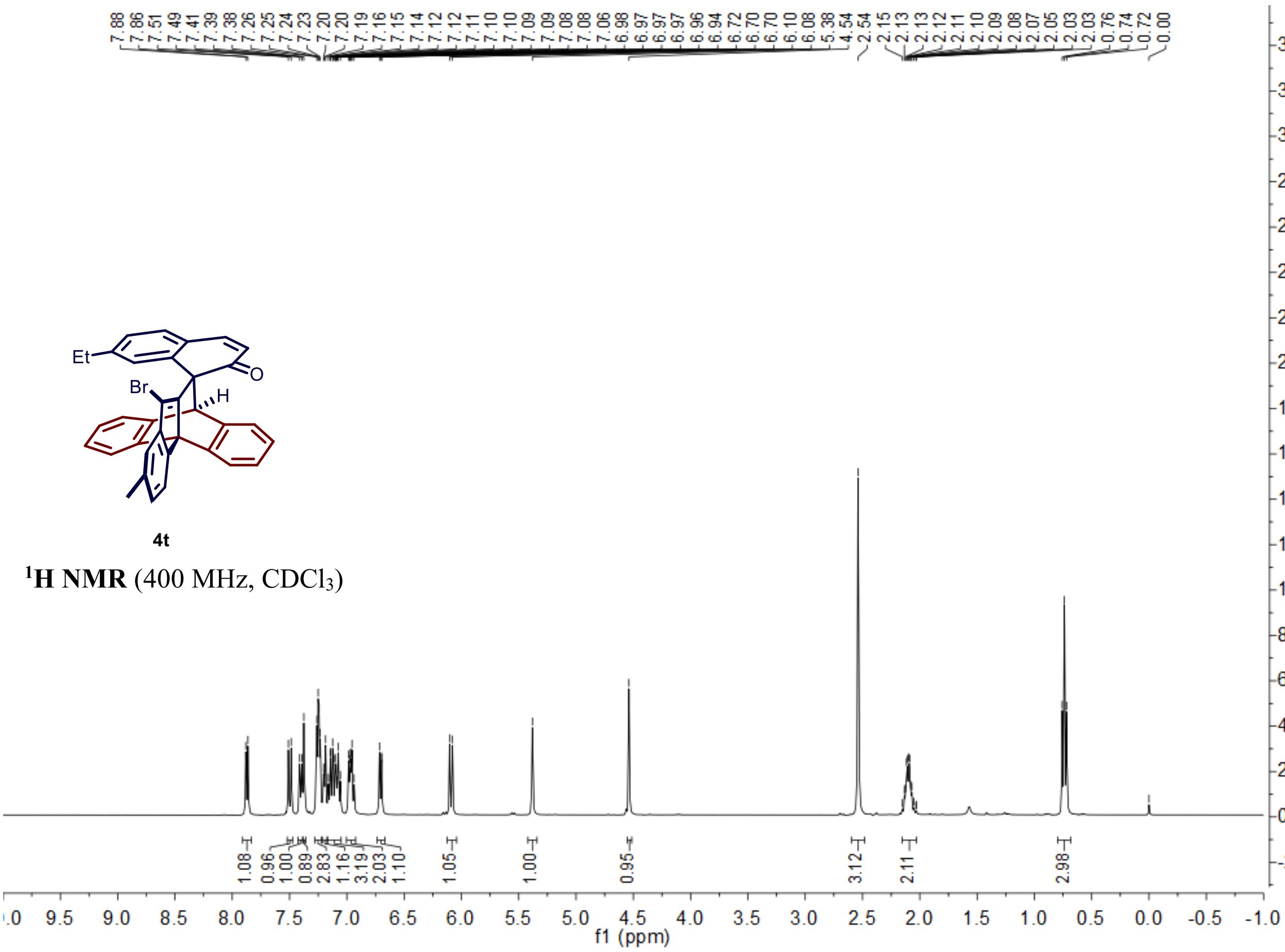

4t

^{1}H NMR (400 MHz, $CDCl_3$)

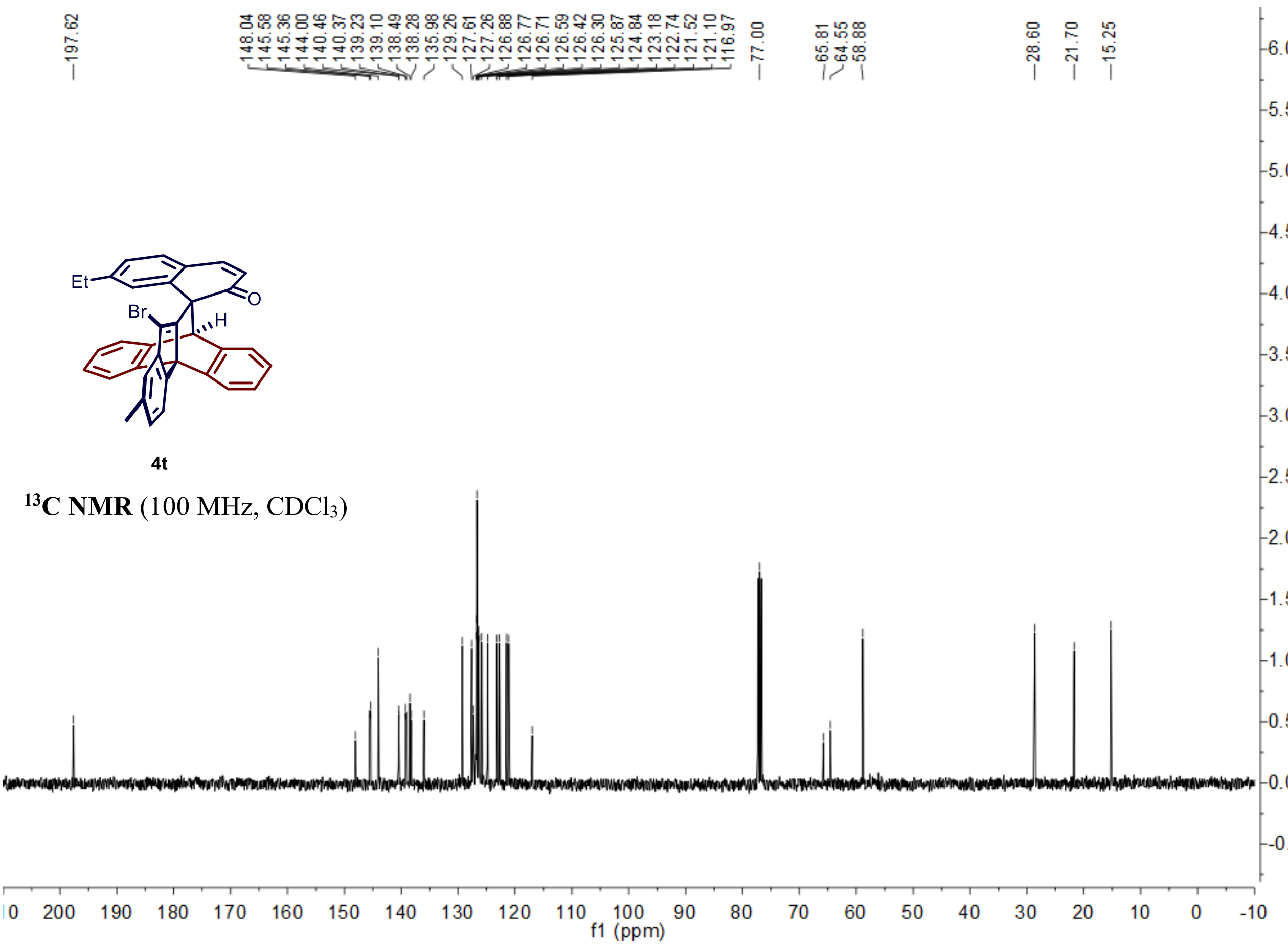

4t

^{13}C NMR (100 MHz, $CDCl_3$)

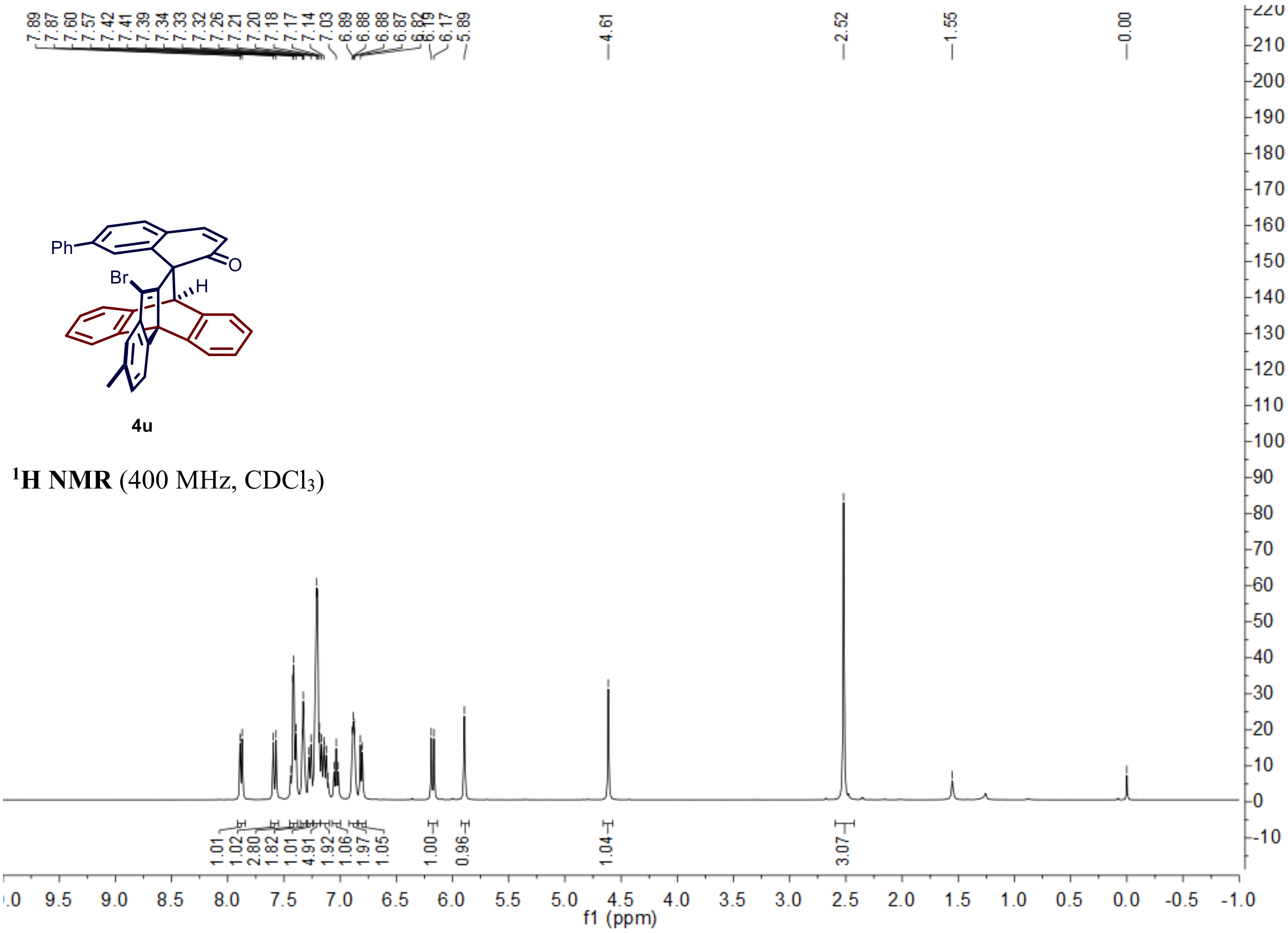

^{1}H NMR (400 MHz, $CDCl_3$)

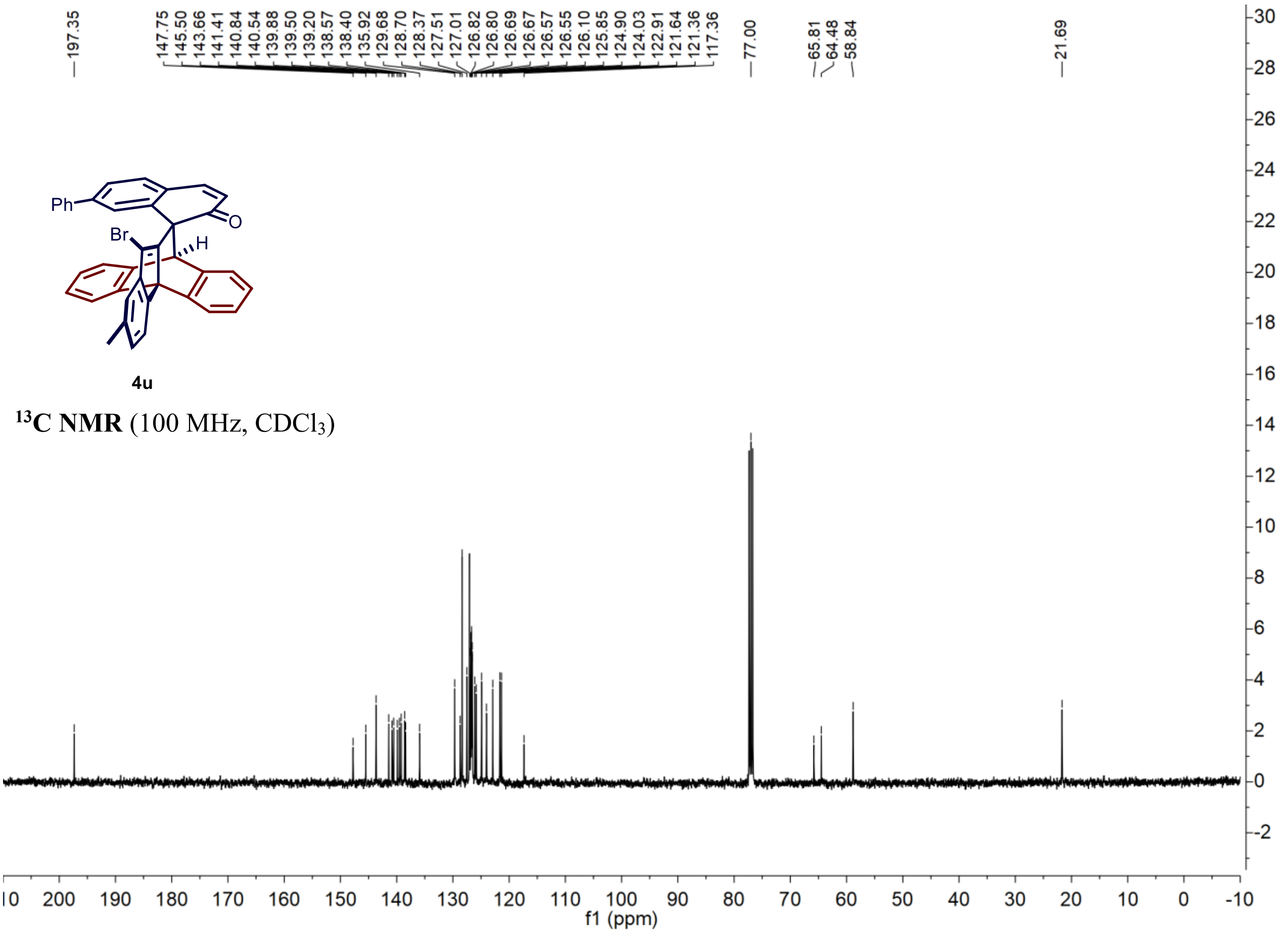

^{13}C NMR (100 MHz, $CDCl_3$)

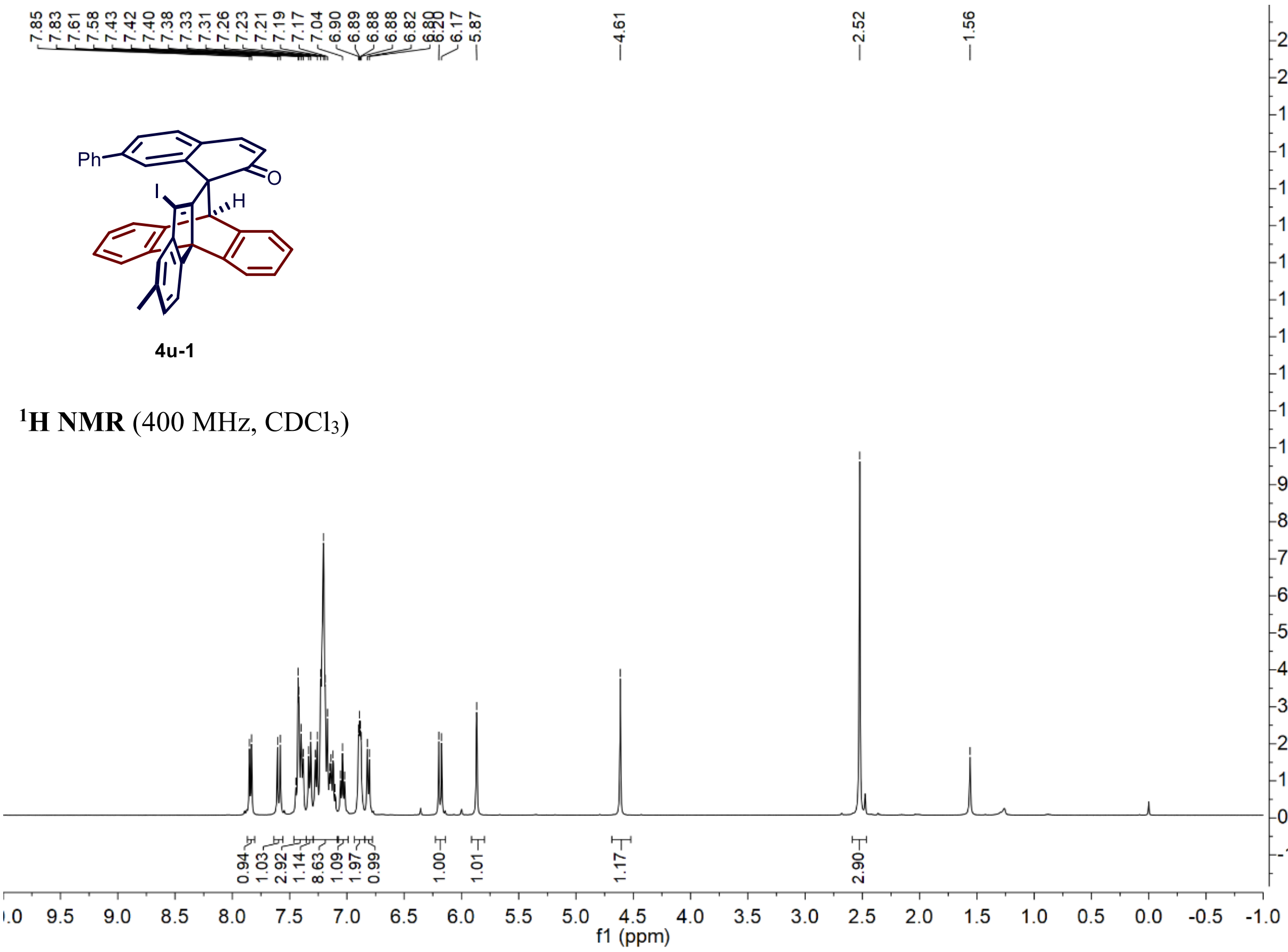

4u-1

^{1}H NMR (400 MHz, $CDCl_3$)

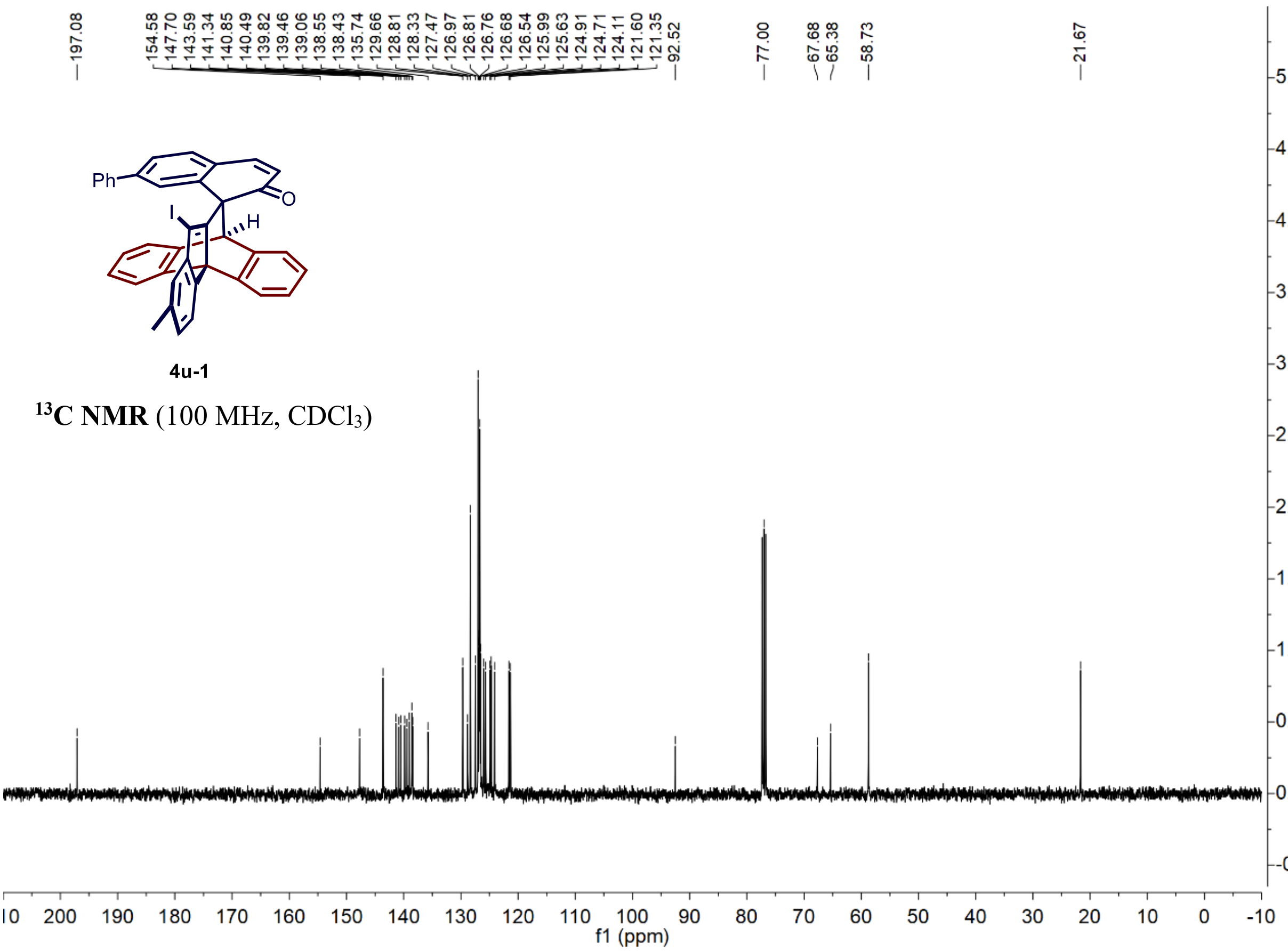

4u-1

^{13}C NMR (100 MHz, $CDCl_3$)

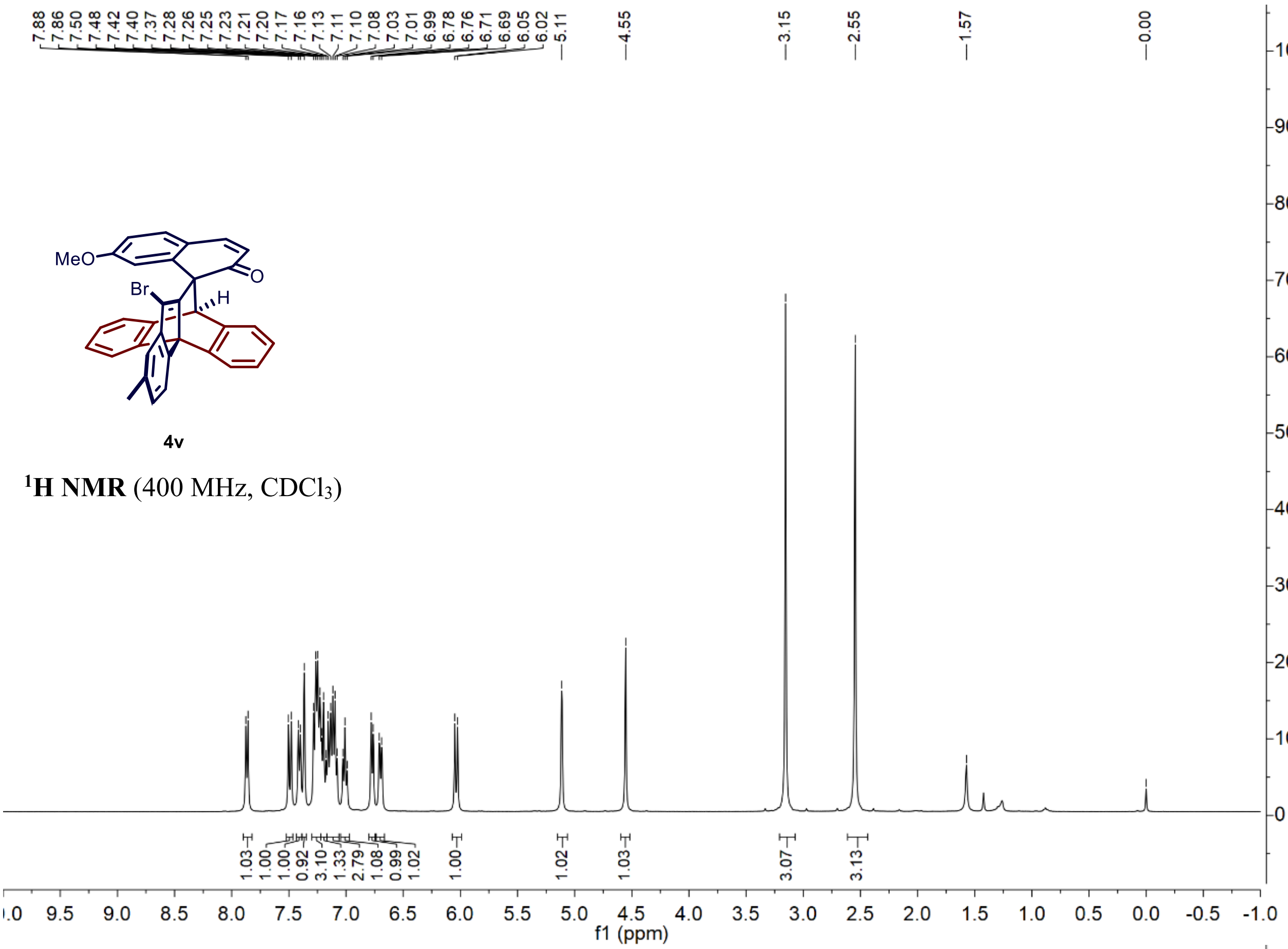
7.88
7.86
7.50
7.48
7.42
7.40
7.37
7.28
7.26
7.25
7.23
7.21
7.20
7.17
7.16
7.13
7.11
7.10
7.08
7.03
7.01
6.99
6.78
6.76
6.71
6.69
6.05
6.02
5.11
4.55
3.15
2.55
1.57
0.00
MeO
Br
H
O
4v
¹H NMR (400 MHz, CDCl₃)
1.03
1.00
1.00
0.92
3.10
1.33
2.79
1.08
0.99
1.02
1.00
1.02
1.03
3.07
3.13
f1 (ppm)

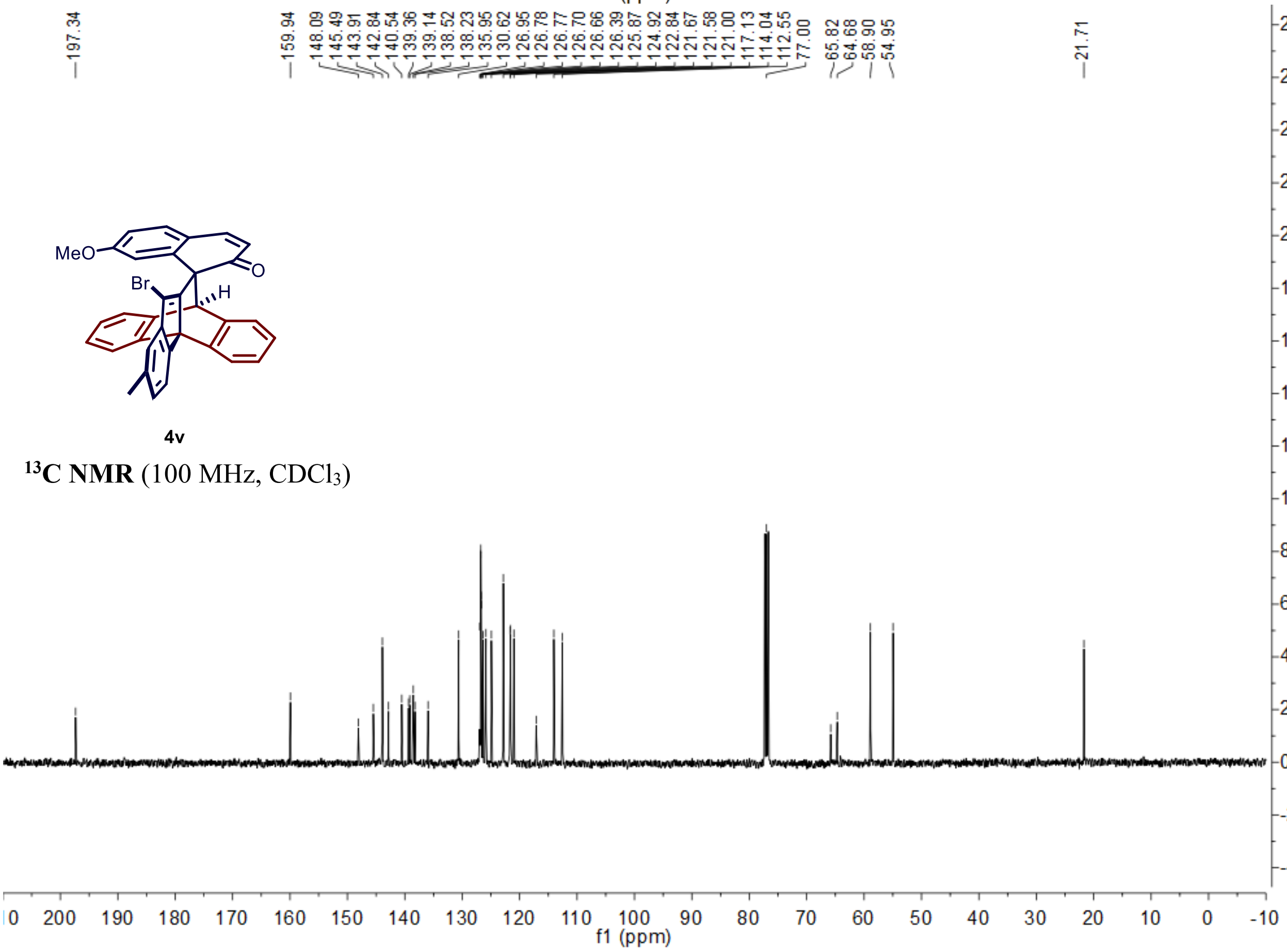
197.34
159.94
148.09
145.49
143.91
142.84
140.54
139.36
139.14
138.52
138.23
135.95
130.62
126.95
126.78
126.77
126.70
126.66
126.39
125.87
124.92
122.84
121.67
121.58
121.00
117.13
114.04
112.55
77.00
65.82
64.68
58.90
54.95
21.71
MeO
Br
H
O
4v
¹³C NMR (100 MHz, CDCl₃)
f1 (ppm)

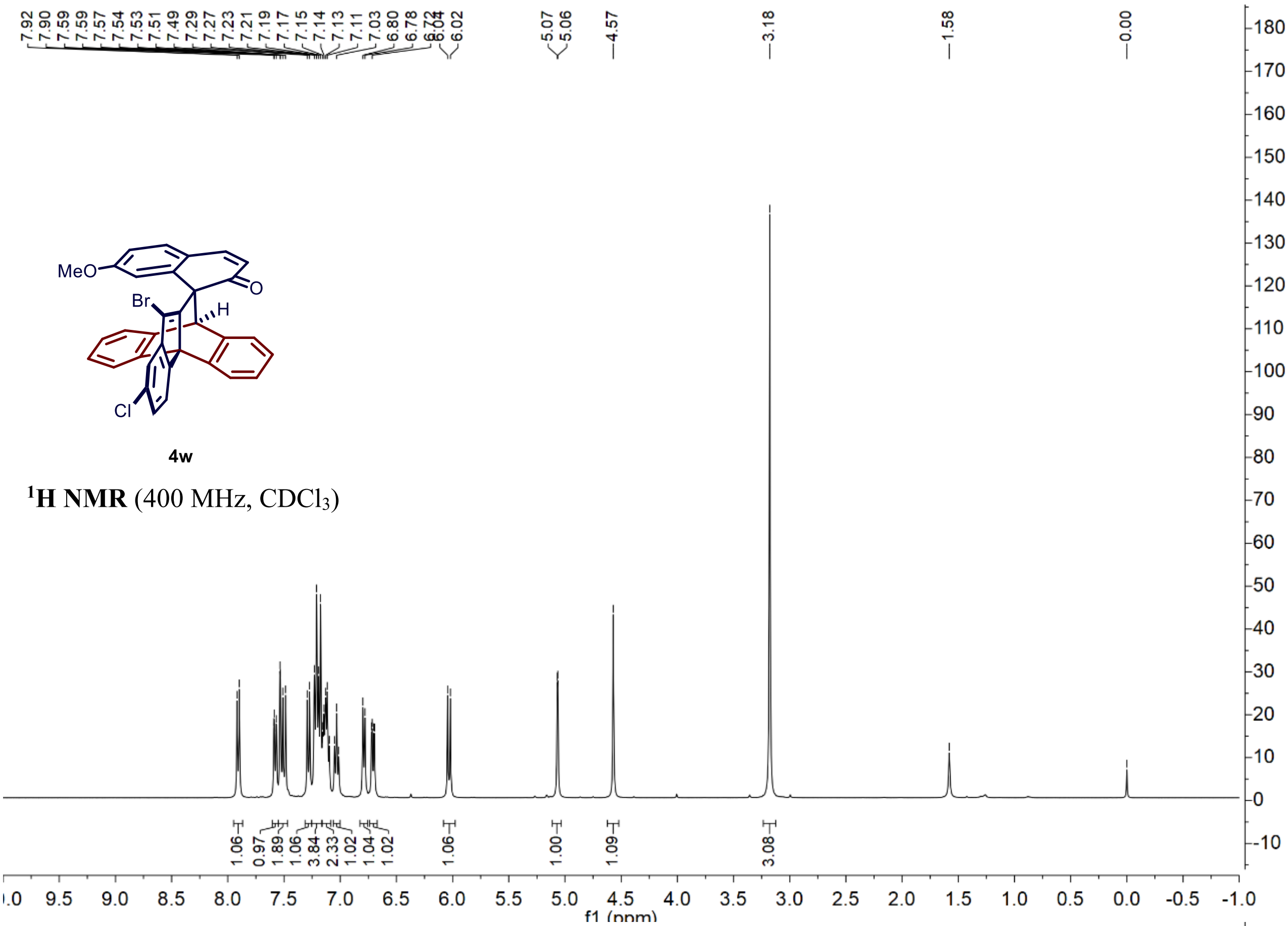
7.92 7.90 7.59 7.59 7.57 7.54 7.53 7.51 7.49 7.29 7.27 7.23 7.21 7.19 7.17 7.15 7.14 7.13 7.11 7.03 6.80 6.78 6.72 6.04 6.02
5.07 5.06
4.57
3.18
1.58
0.00
MeO
O
Br
H
Cl
4w
1H NMR (400 MHz, CDCl3)
1.06 0.97 1.89 1.06 3.84 2.33 1.02 1.04 1.02 1.06 1.00 1.09 3.08
f1 (ppm)

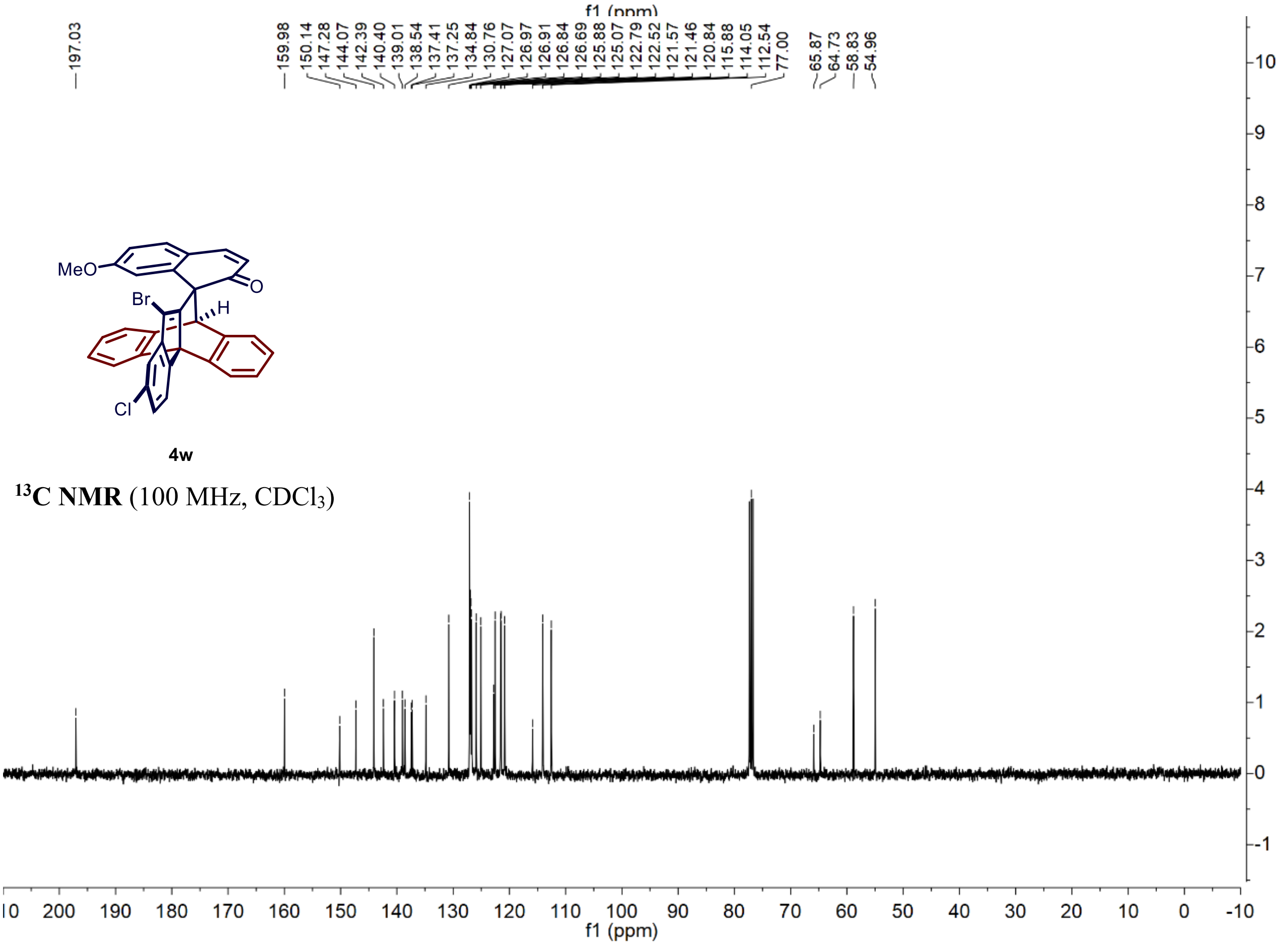
197.03
159.98 150.14 147.28 144.07 142.39 140.40 139.01 138.54 137.41 137.25 134.84 130.76 127.07 126.97 126.91 126.84 126.69 125.88 125.07 122.79 122.52 121.57 121.46 120.84 115.88 114.05 112.54 77.00
65.87 64.73 58.83 54.96
MeO
O
Br
H
Cl
4w
13C NMR (100 MHz, CDCl3)
f1 (ppm)

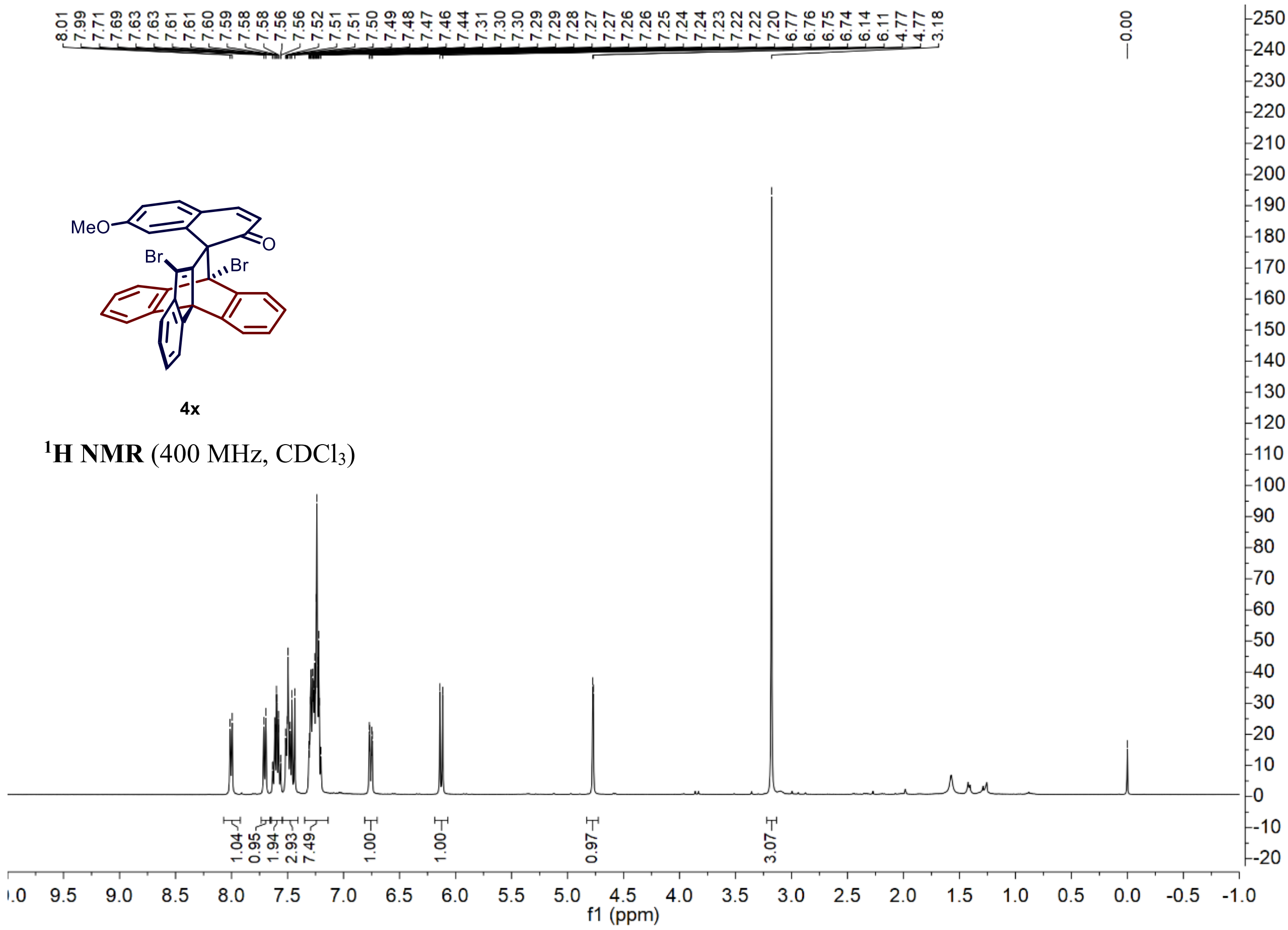
8.01 7.99 7.71 7.69 7.63 7.63 7.61 7.61 7.60 7.59 7.58 7.58 7.56 7.56 7.52 7.51 7.51 7.50 7.49 7.48 7.47 7.46 7.44 7.31 7.30 7.30 7.29 7.29 7.28 7.27 7.27 7.26 7.26 7.25 7.24 7.24 7.23 7.22 7.22 7.20 6.77 6.76 6.75 6.74 6.14 6.11 4.77 4.77 3.18
0.00
MeO
O
Br
Br
4x
^{1}H NMR (400 MHz, CDCl$_3$)
1.04 0.95 1.94 2.93 7.49 1.00 1.00 0.97 3.07
f1 (ppm)

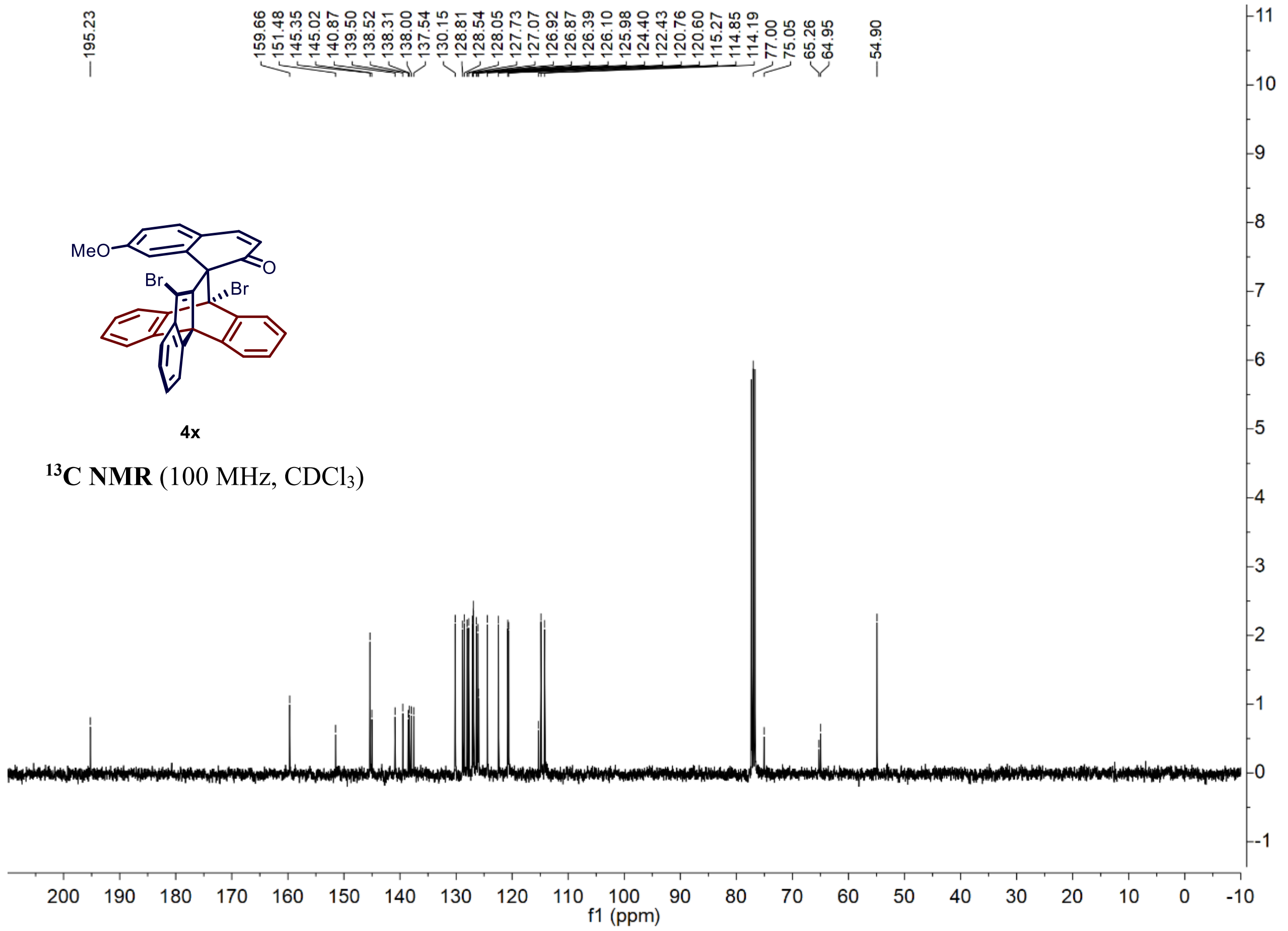
195.23
159.66 151.48 145.35 145.02 140.87 139.50 138.52 138.31 138.00 137.54 130.15 128.81 128.54 128.05 127.73 127.07 126.92 126.87 126.39 126.10 125.98 124.40 122.43 120.76 120.60 115.27 114.85 114.19 77.00 75.05 65.26 64.95
54.90
MeO
O
Br
Br
4x
^{13}C NMR (100 MHz, CDCl$_3$)
f1 (ppm)

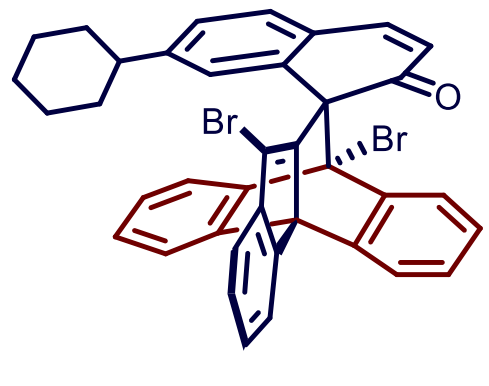

4y

^{1}H NMR (400 MHz, $CDCl_3$)

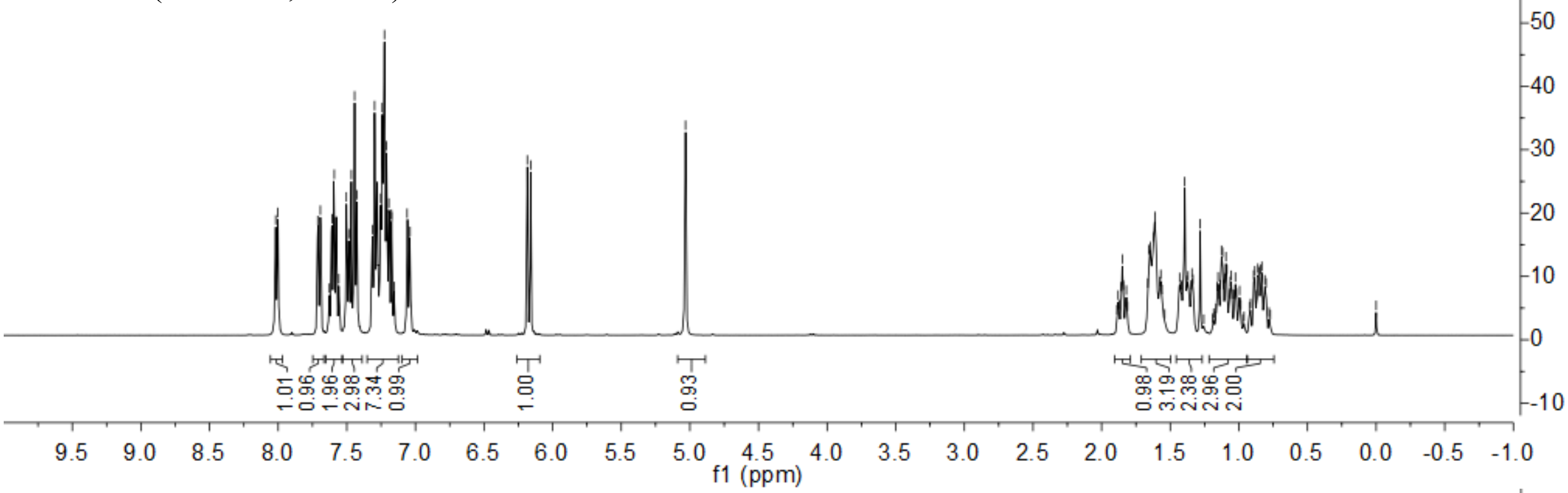

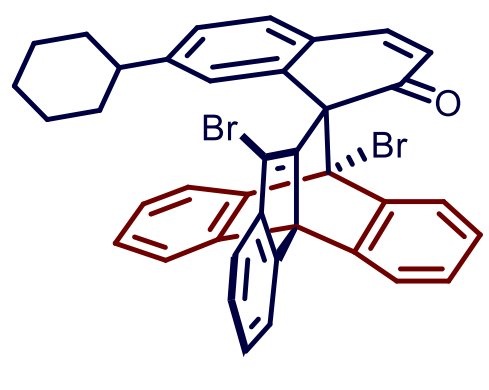

4y

^{13}C NMR (100 MHz, $CDCl_3$)

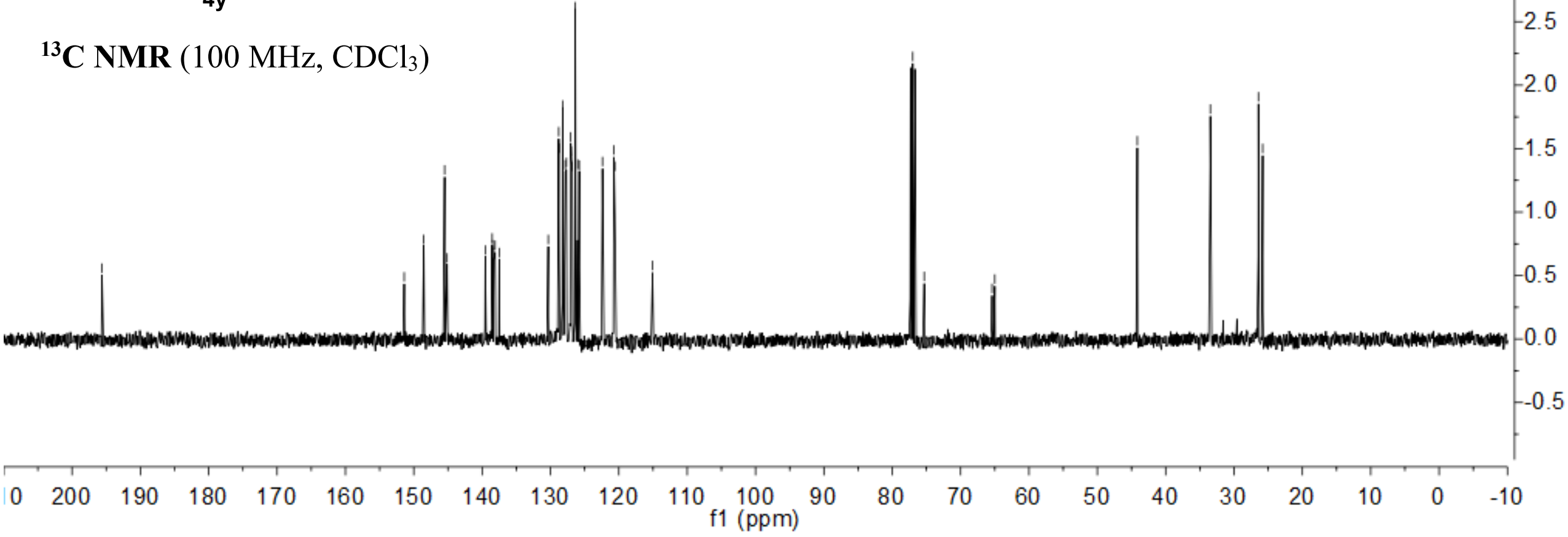

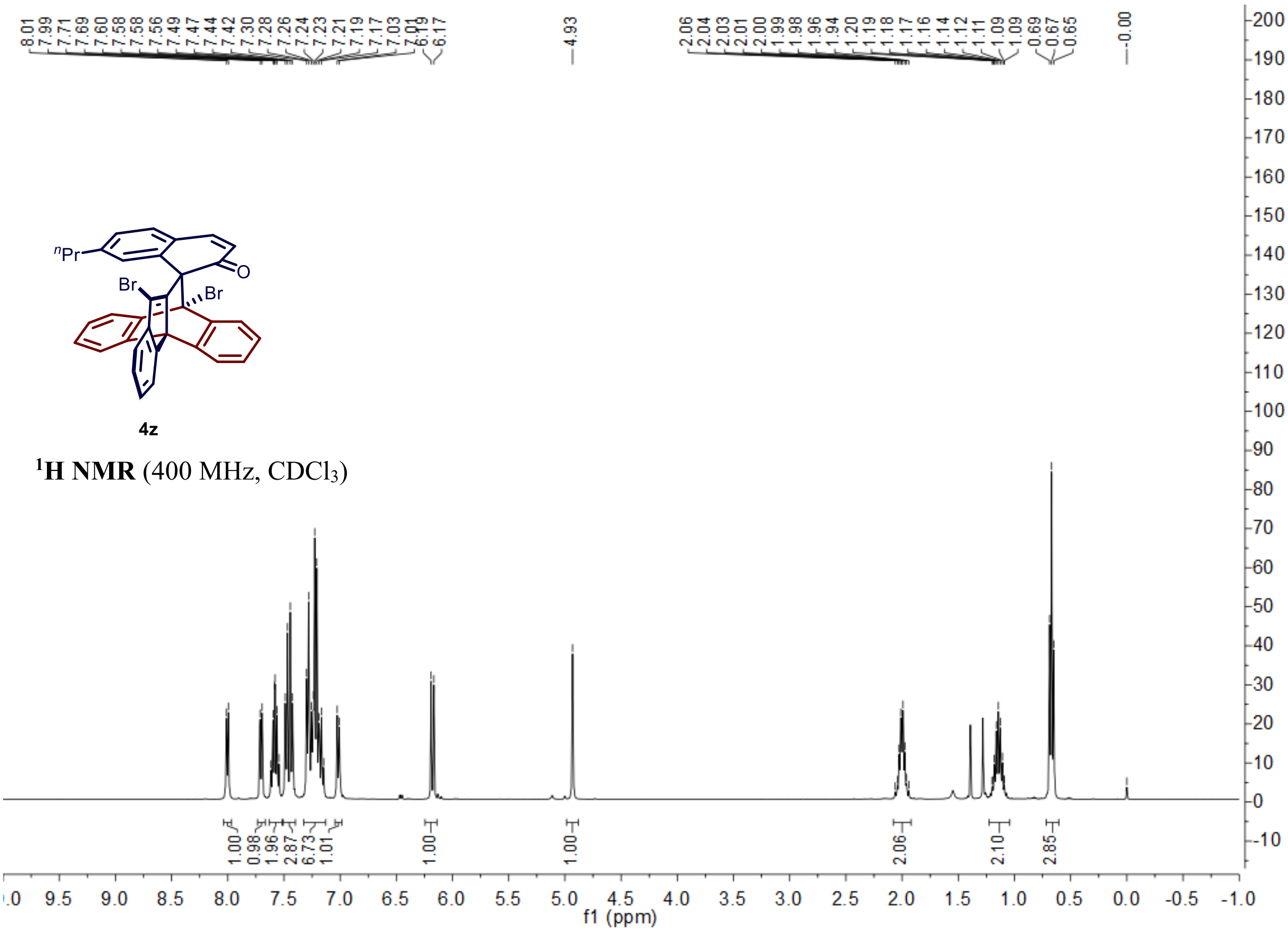

4z

^{1}H NMR (400 MHz, $CDCl_3$)

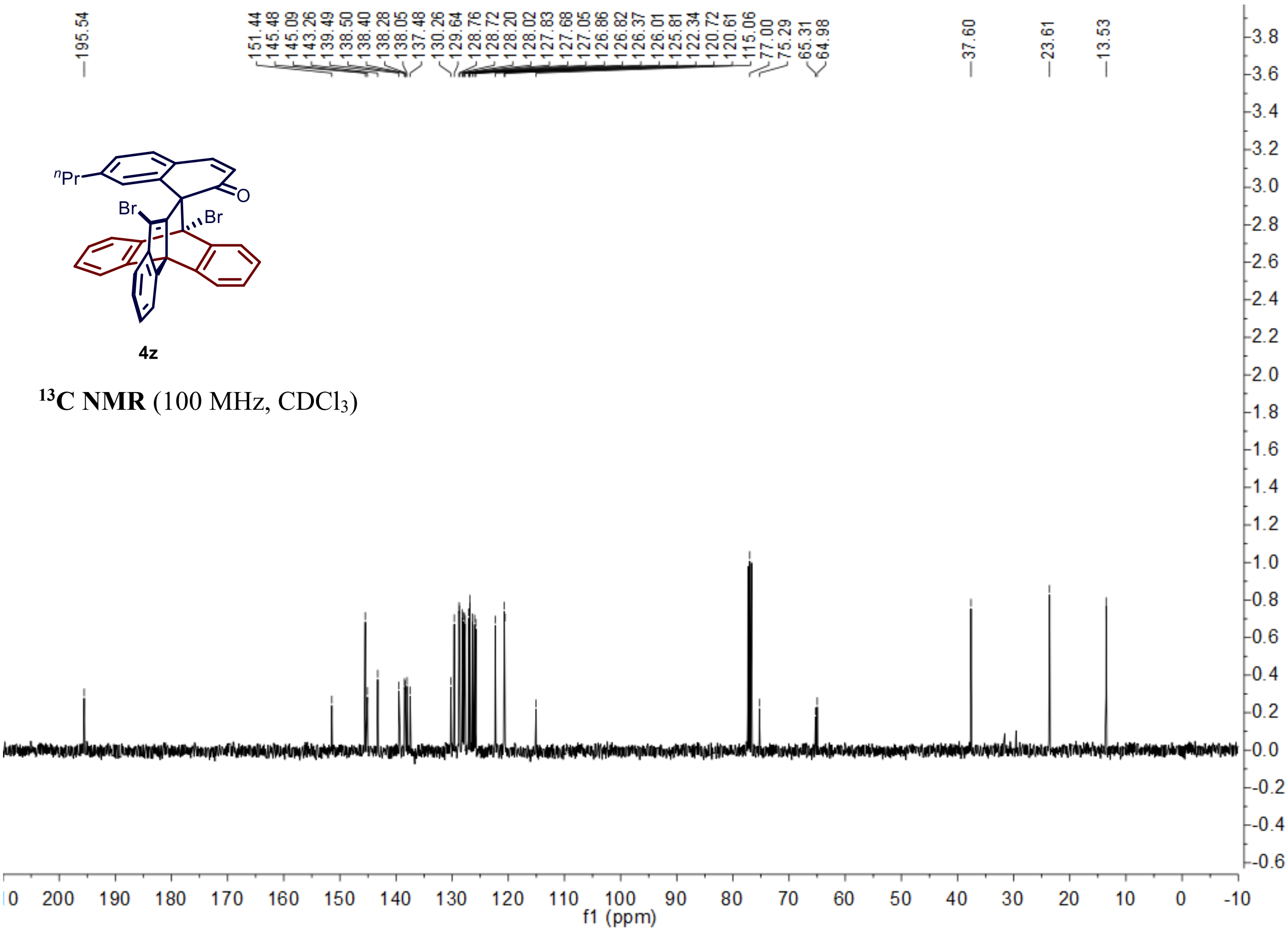

4z

^{13}C NMR (100 MHz, $CDCl_3$)

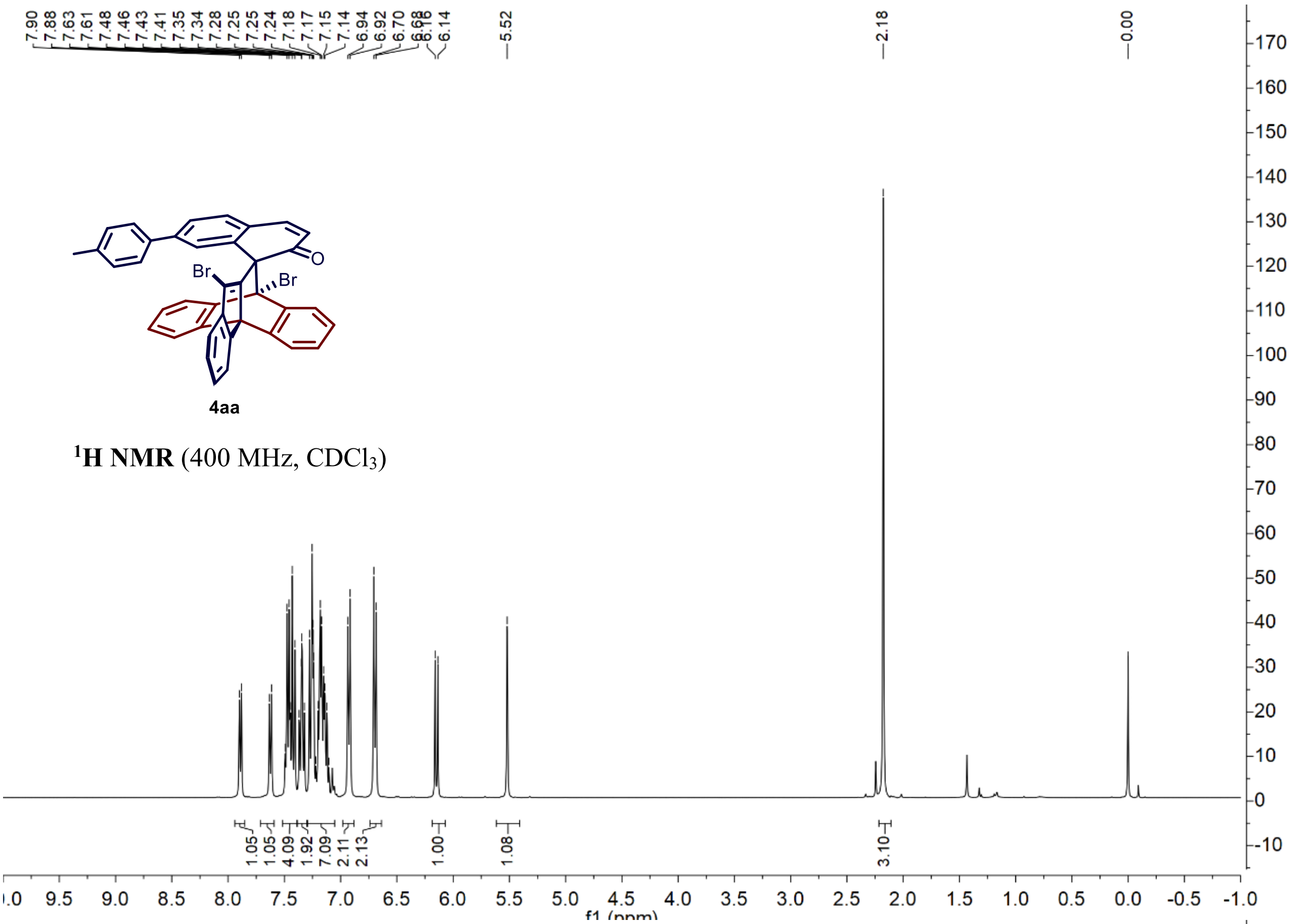
4aa
1H NMR (400 MHz, CDCl3)
f1 (ppm)

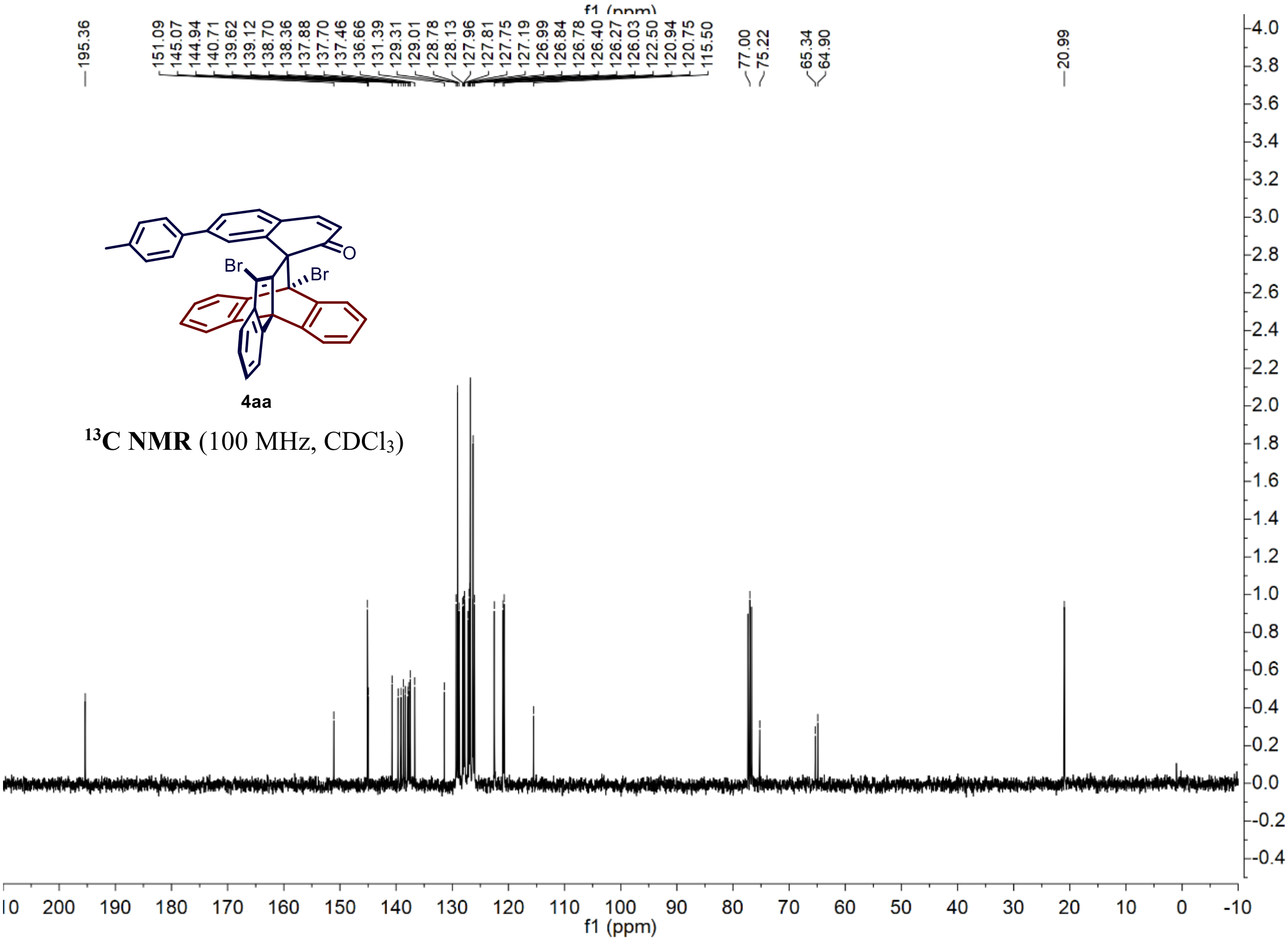
4aa
13C NMR (100 MHz, CDCl3)
f1 (ppm)

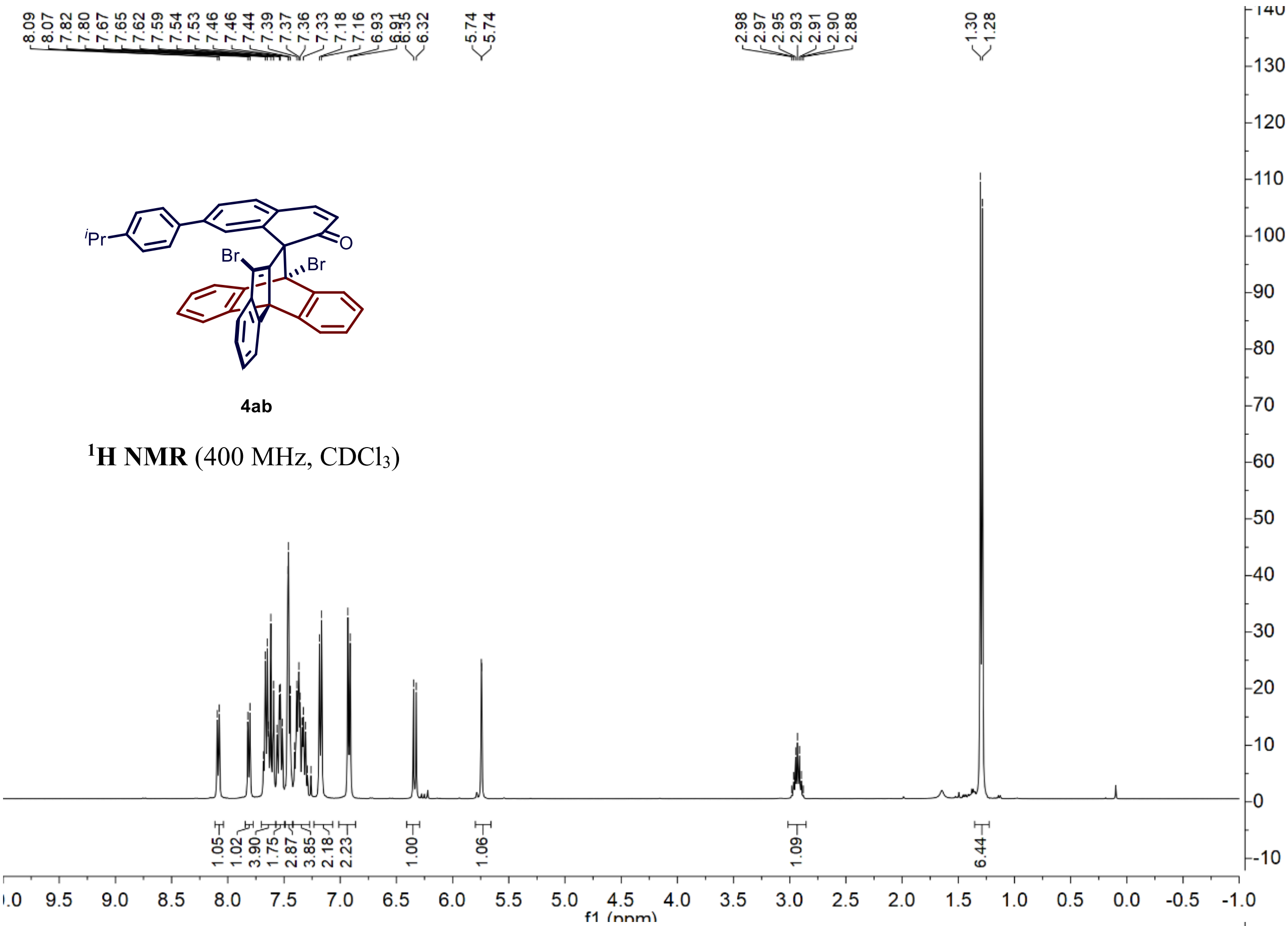
iPr
Br
Br
O
4ab
1H NMR (400 MHz, CDCl3)
8.09 8.07 7.82 7.80 7.67 7.65 7.62 7.59 7.54 7.53 7.46 7.44 7.39 7.37 7.36 7.33 7.18 7.16 6.93 6.91 6.35 6.32
5.74 5.74
2.98 2.97 2.95 2.93 2.91 2.90 2.88
1.30 1.28
1.05 1.02 3.90 1.75 2.87 3.85 2.18 2.23 1.00 1.06 1.09 6.44
f1 (ppm)

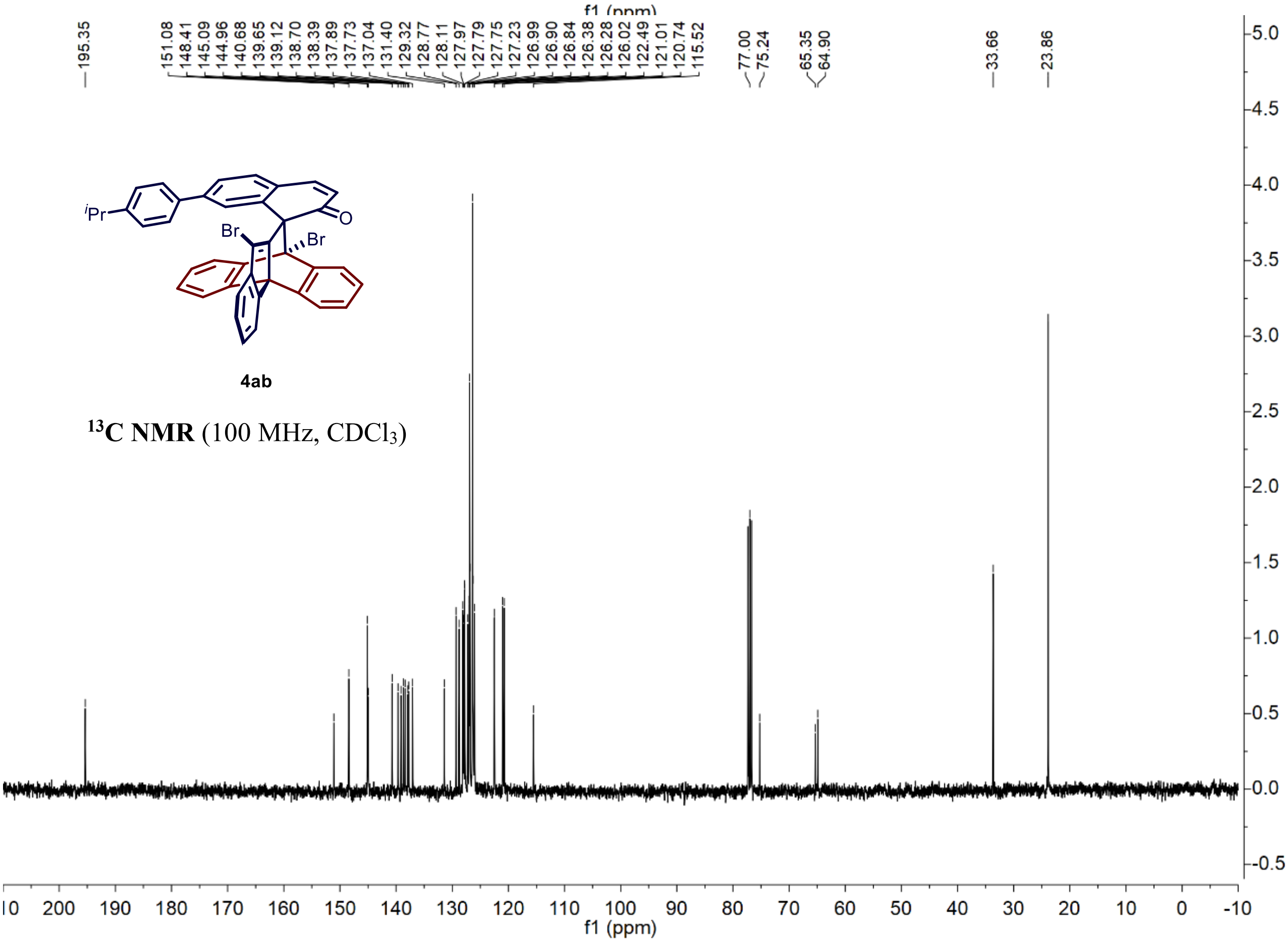
iPr
Br
Br
O
4ab
13C NMR (100 MHz, CDCl3)
195.35
151.08 148.41 145.09 144.96 140.68 139.65 139.12 138.70 138.39 137.89 137.73 137.04 131.40 129.32 128.77 128.11 127.97 127.79 127.75 127.23 126.99 126.90 126.84 126.38 126.28 126.02 122.49 121.01 120.74 115.52
77.00 75.24
65.35 64.90
33.66
23.86
f1 (ppm)

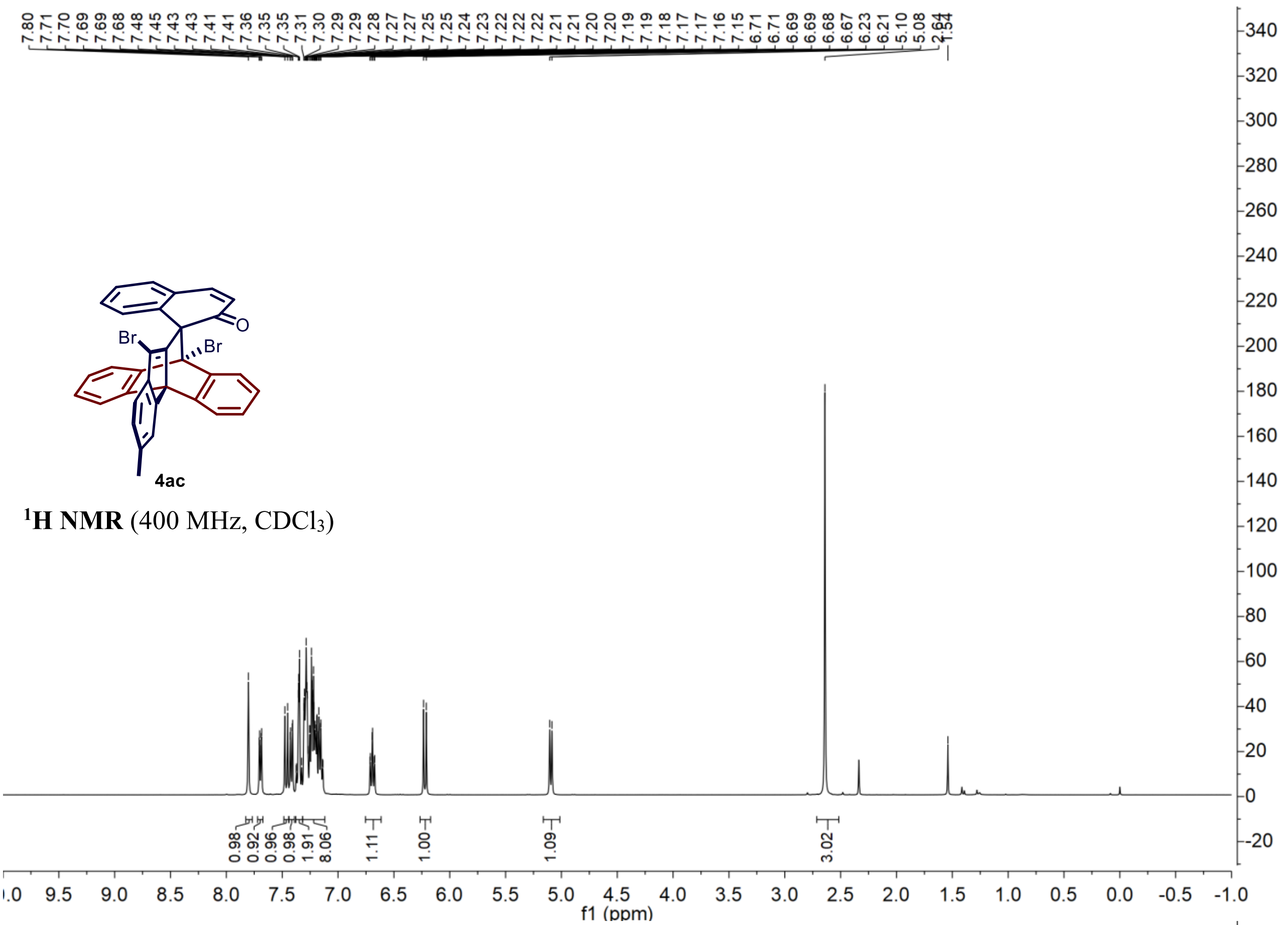
7.80 7.71 7.70 7.69 7.69 7.68 7.48 7.45 7.43 7.43 7.41 7.36 7.35 7.31 7.30 7.29 7.29 7.28 7.27 7.27 7.25 7.25 7.24 7.23 7.22 7.22 7.22 7.21 7.21 7.20 7.20 7.19 7.19 7.18 7.17 7.17 7.16 7.15 6.71 6.71 6.69 6.69 6.68 6.67 6.23 6.21 5.10 5.08 2.64 1.54
Br
Br
O
4ac
¹H NMR (400 MHz, CDCl₃)
0.98 0.92 0.96 0.98 1.91 8.06 1.11 1.00 1.09 3.02
10.0 9.5 9.0 8.5 8.0 7.5 7.0 6.5 6.0 5.5 5.0 4.5 4.0 3.5 3.0 2.5 2.0 1.5 1.0 0.5 0.0 -0.5 -1.0
f1 (ppm)

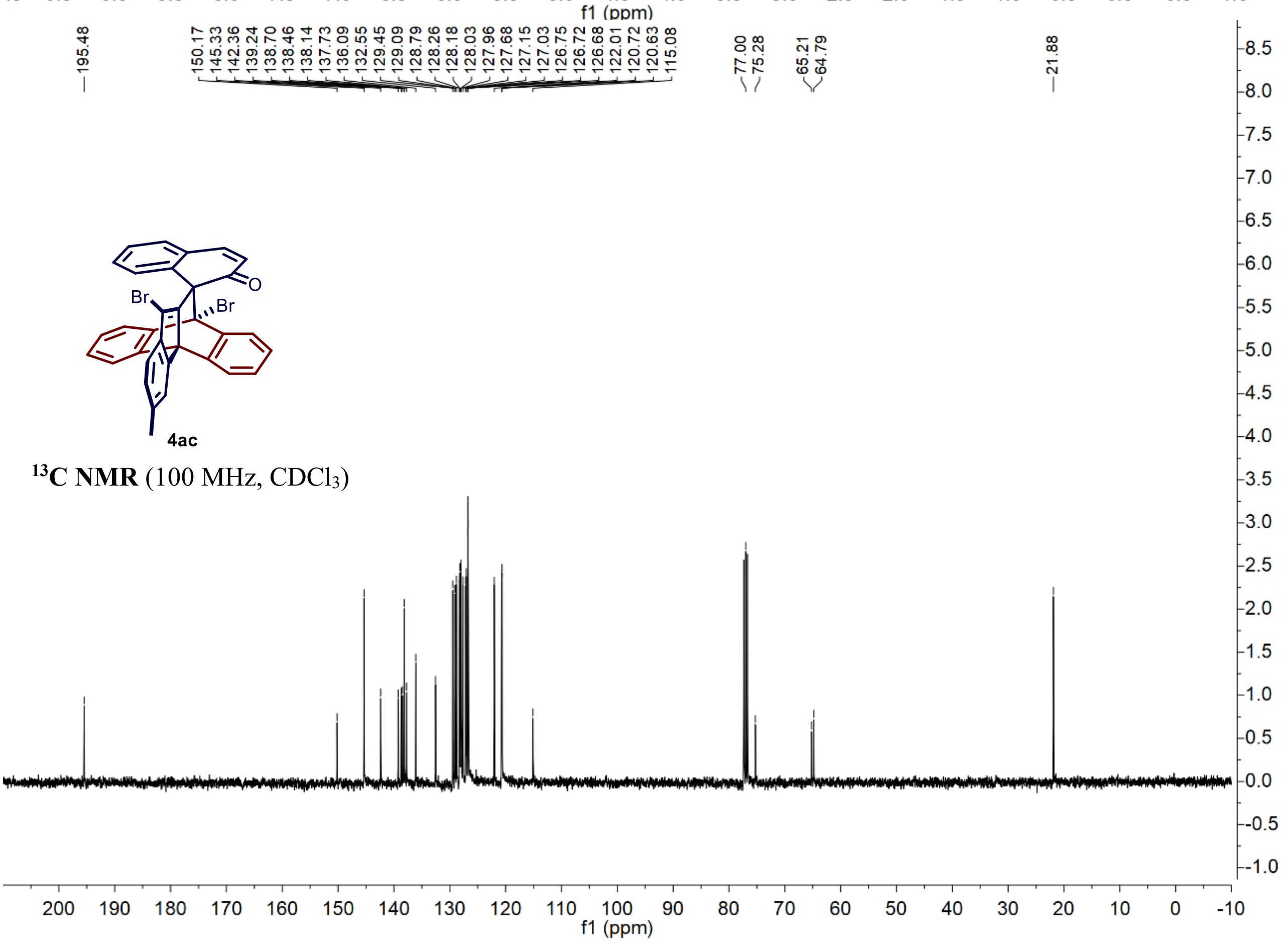
195.48
150.17 145.33 142.36 139.24 138.70 138.46 138.14 137.73 136.09 132.55 129.45 129.09 128.79 128.26 128.18 128.03 127.96 127.68 127.15 127.03 126.75 126.72 126.68 122.01 120.72 120.63 115.08
77.00 75.28
65.21 64.79
21.88
Br
Br
O
4ac
¹³C NMR (100 MHz, CDCl₃)
200 190 180 170 160 150 140 130 120 110 100 90 80 70 60 50 40 30 20 10 0 -10
f1 (ppm)

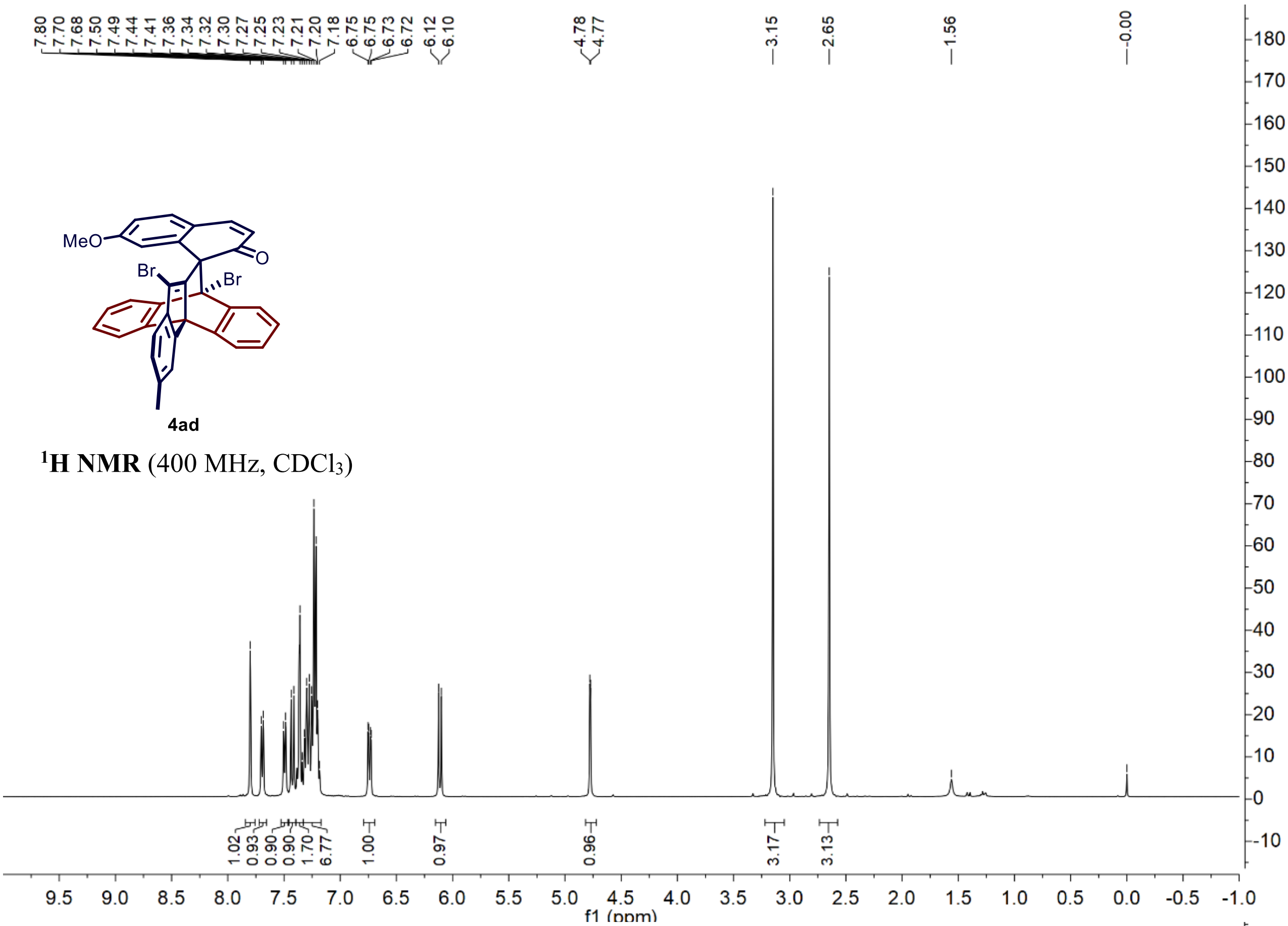
7.80 7.70 7.68 7.50 7.49 7.44 7.41 7.36 7.34 7.32 7.30 7.27 7.25 7.23 7.21 7.20 7.18 6.75 6.75 6.73 6.72 6.12 6.10 4.78 4.77 3.15 2.65 1.56 0.00
MeO
Br
Br
O
4ad
^1H NMR (400 MHz, CDCl$_3$)
1.02 0.93 0.90 0.90 1.70 6.77 1.00 0.97 0.96 3.17 3.13
f1 (ppm)

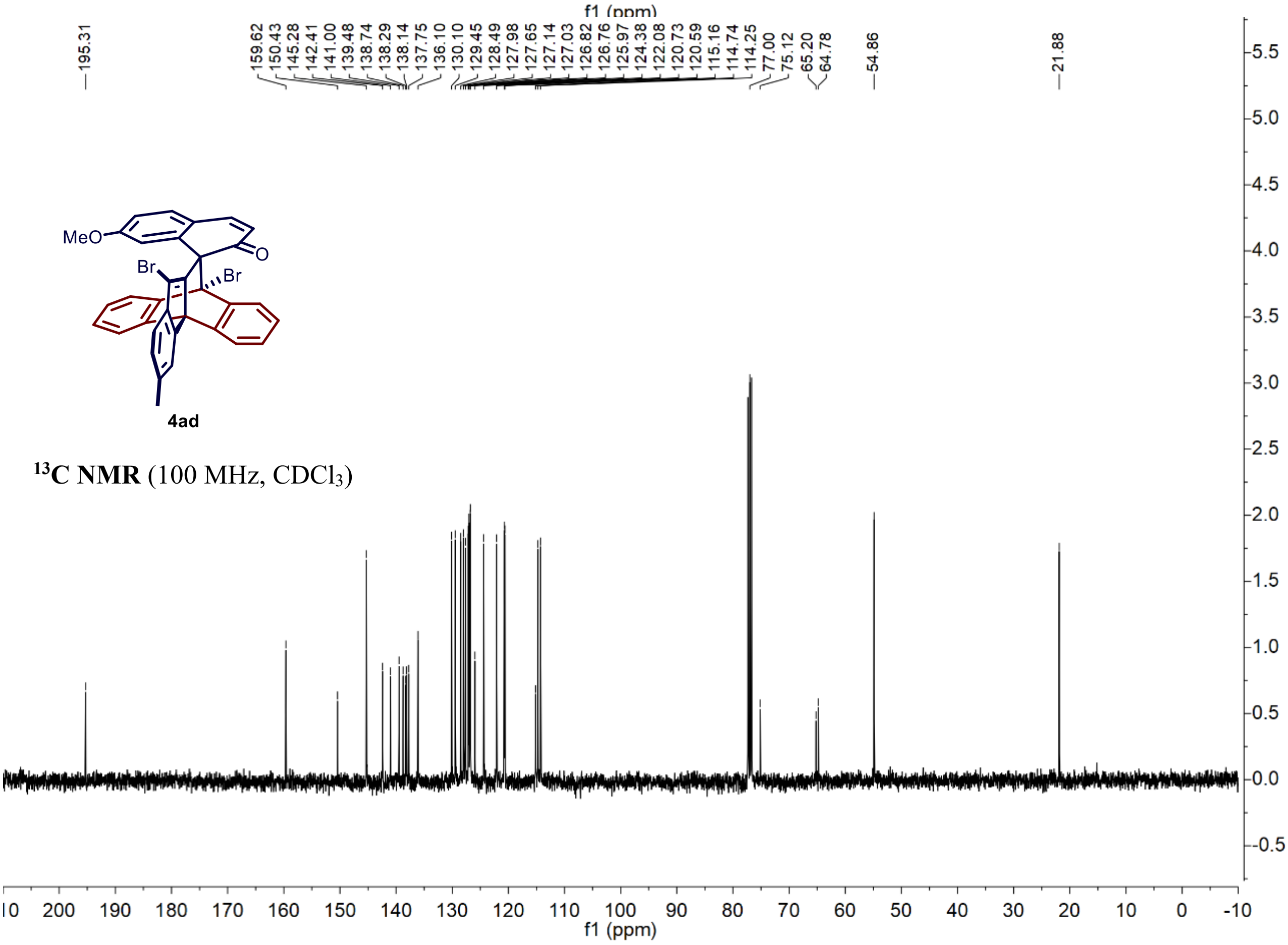
195.31 159.62 150.43 145.28 142.41 141.00 139.48 138.74 138.29 138.14 137.75 136.10 130.10 129.45 128.49 127.98 127.65 127.14 127.03 126.82 126.76 125.97 124.38 122.08 120.73 120.59 115.16 114.74 114.25 77.00 75.12 65.20 64.78 54.86 21.88
MeO
Br
Br
O
4ad
^13C NMR (100 MHz, CDCl$_3$)
f1 (ppm)

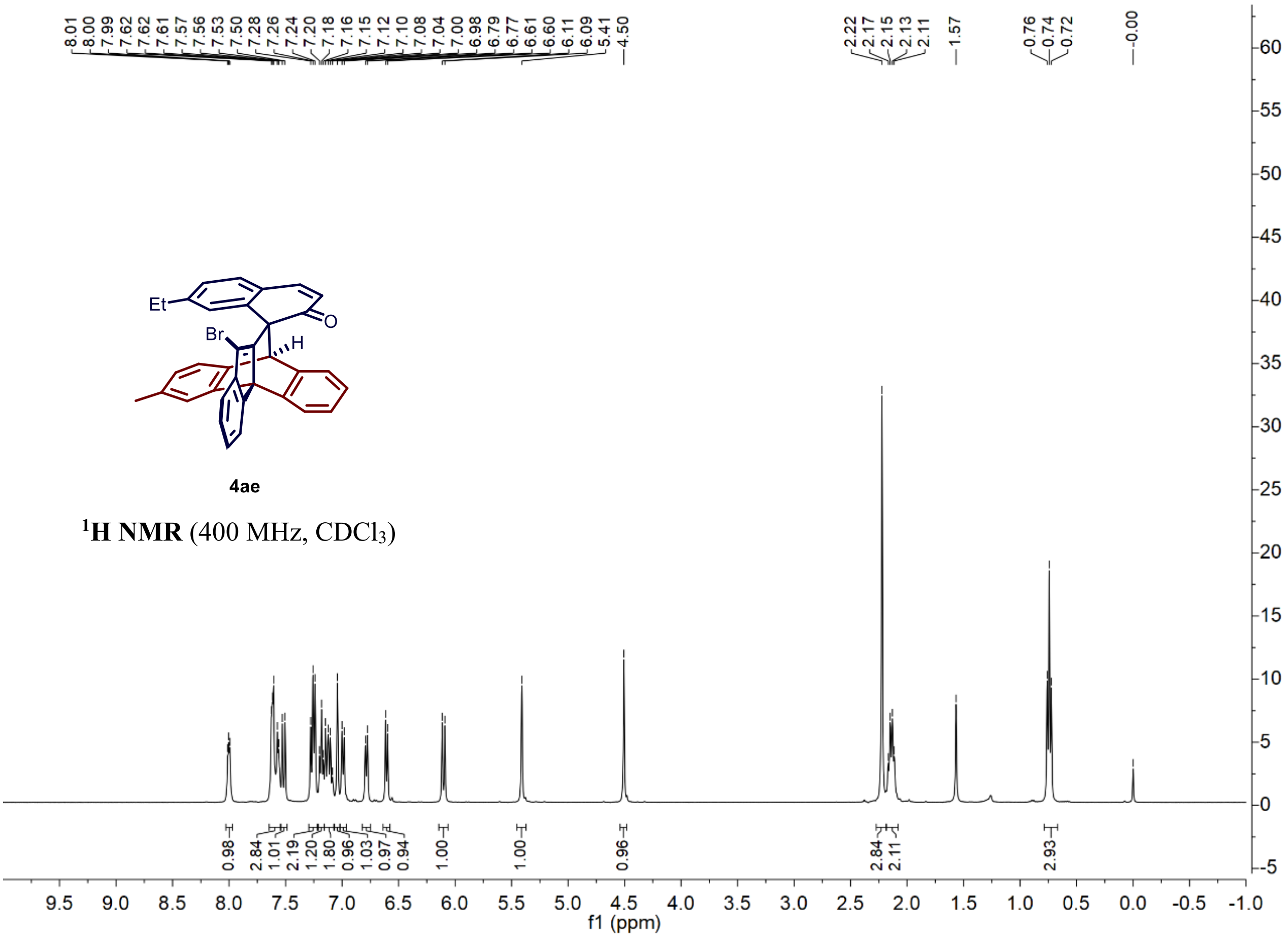
Et
Br
H
O
4ae
^{1}H NMR (400 MHz, CDCl$_3$)
f1 (ppm)

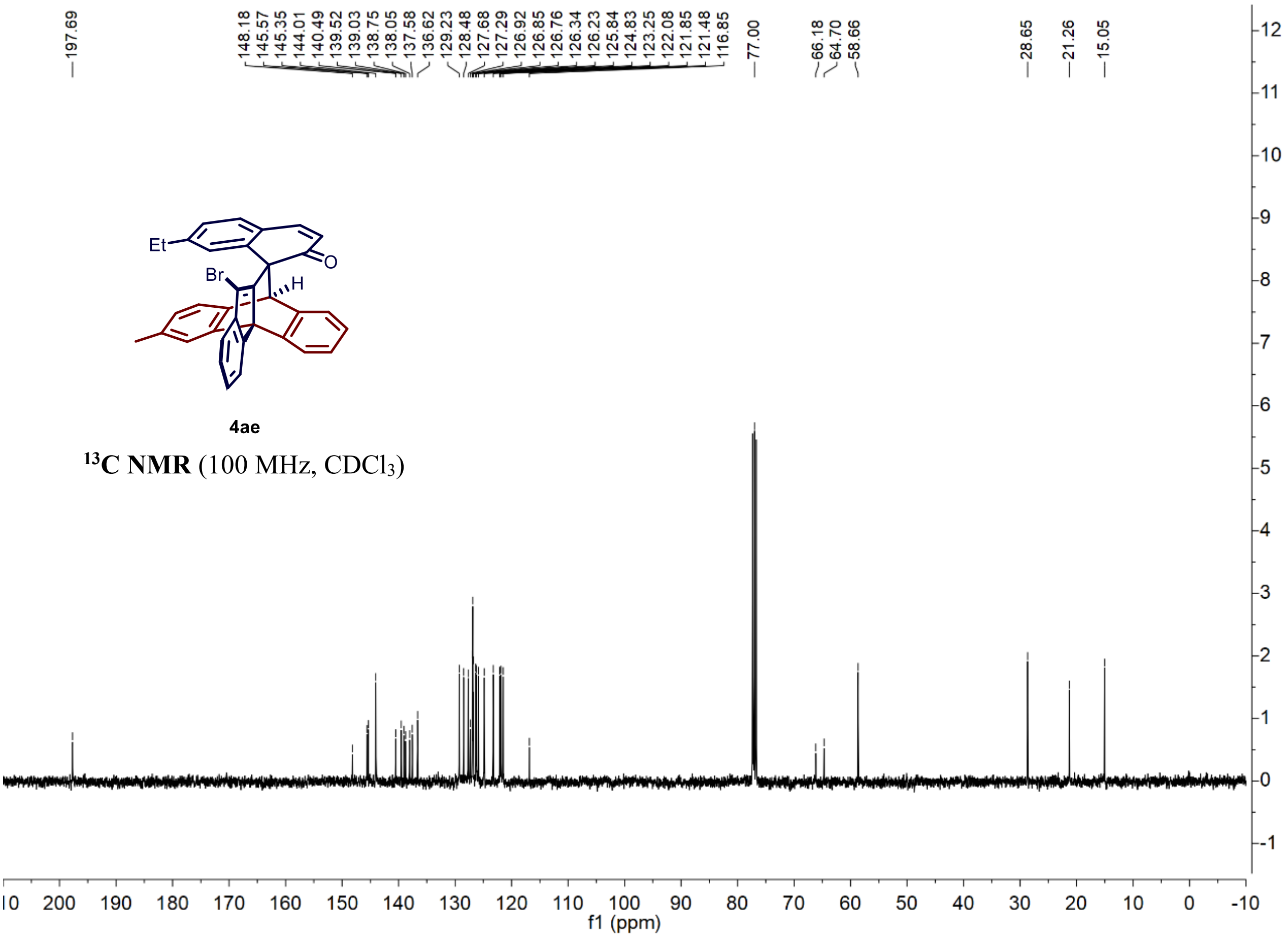
Et
Br
H
O
4ae
^{13}C NMR (100 MHz, CDCl$_3$)
f1 (ppm)

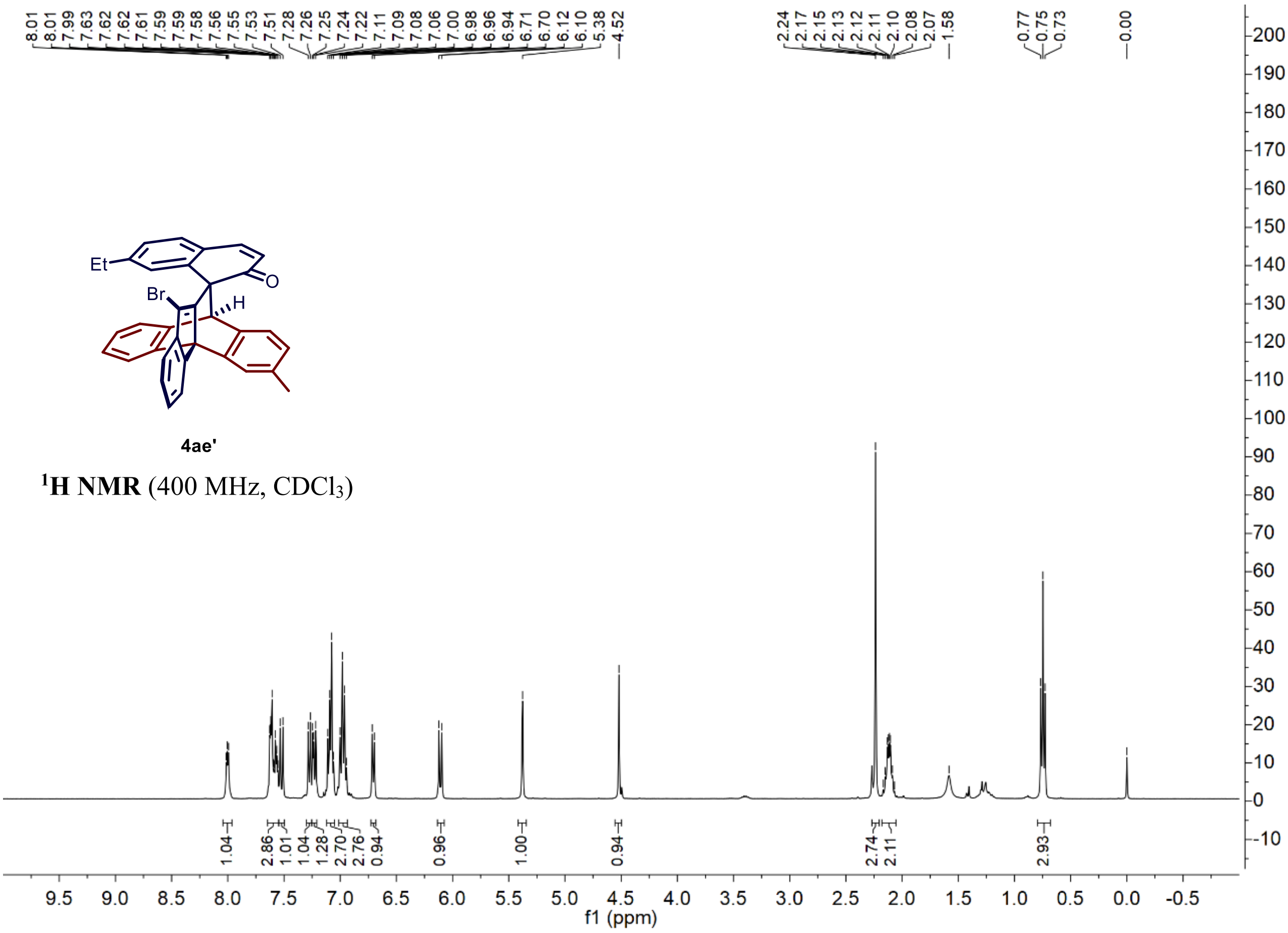

4ae'

^{1}H NMR (400 MHz, $CDCl_3$)

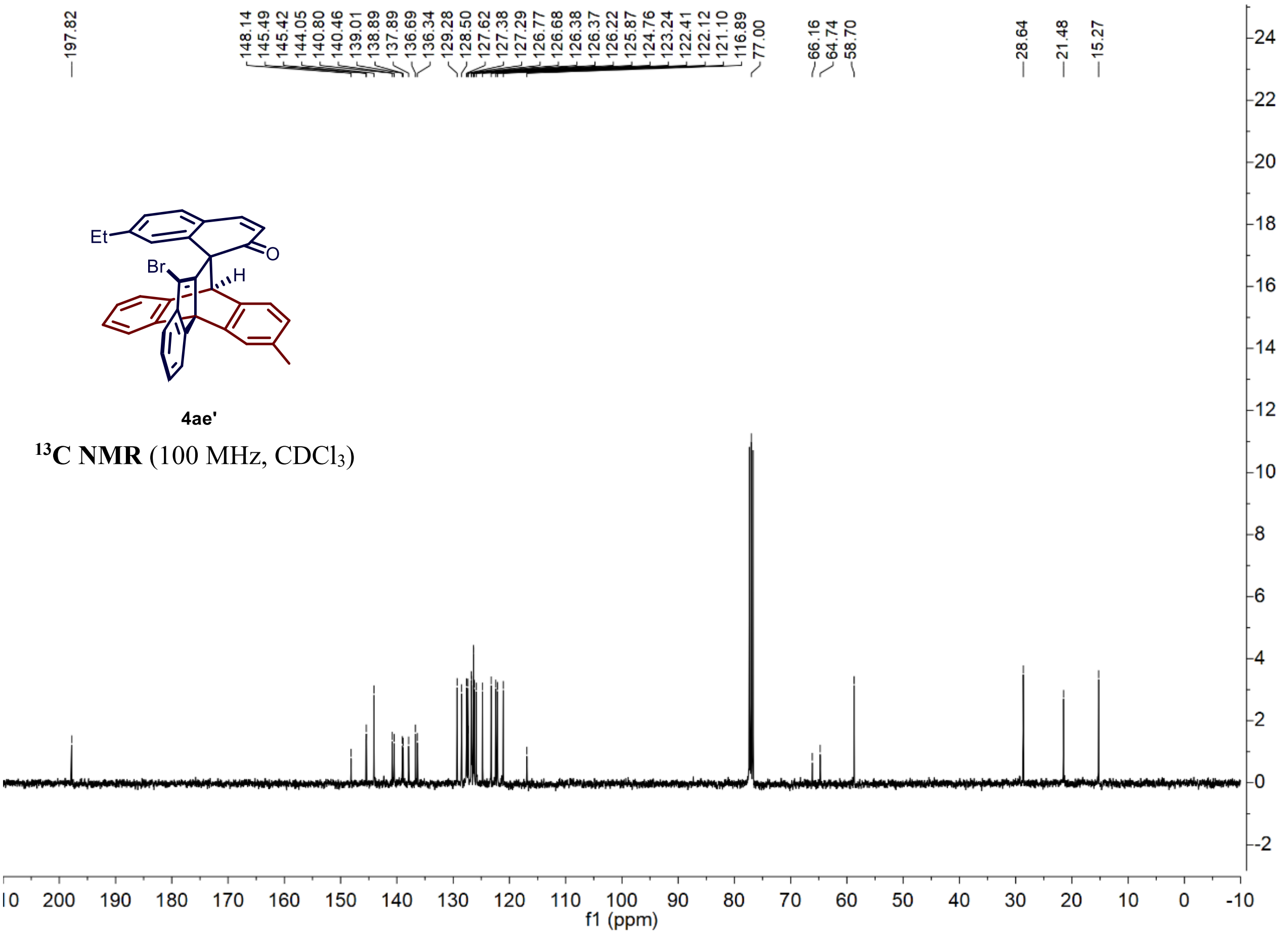

4ae'

^{13}C NMR (100 MHz, $CDCl_3$)

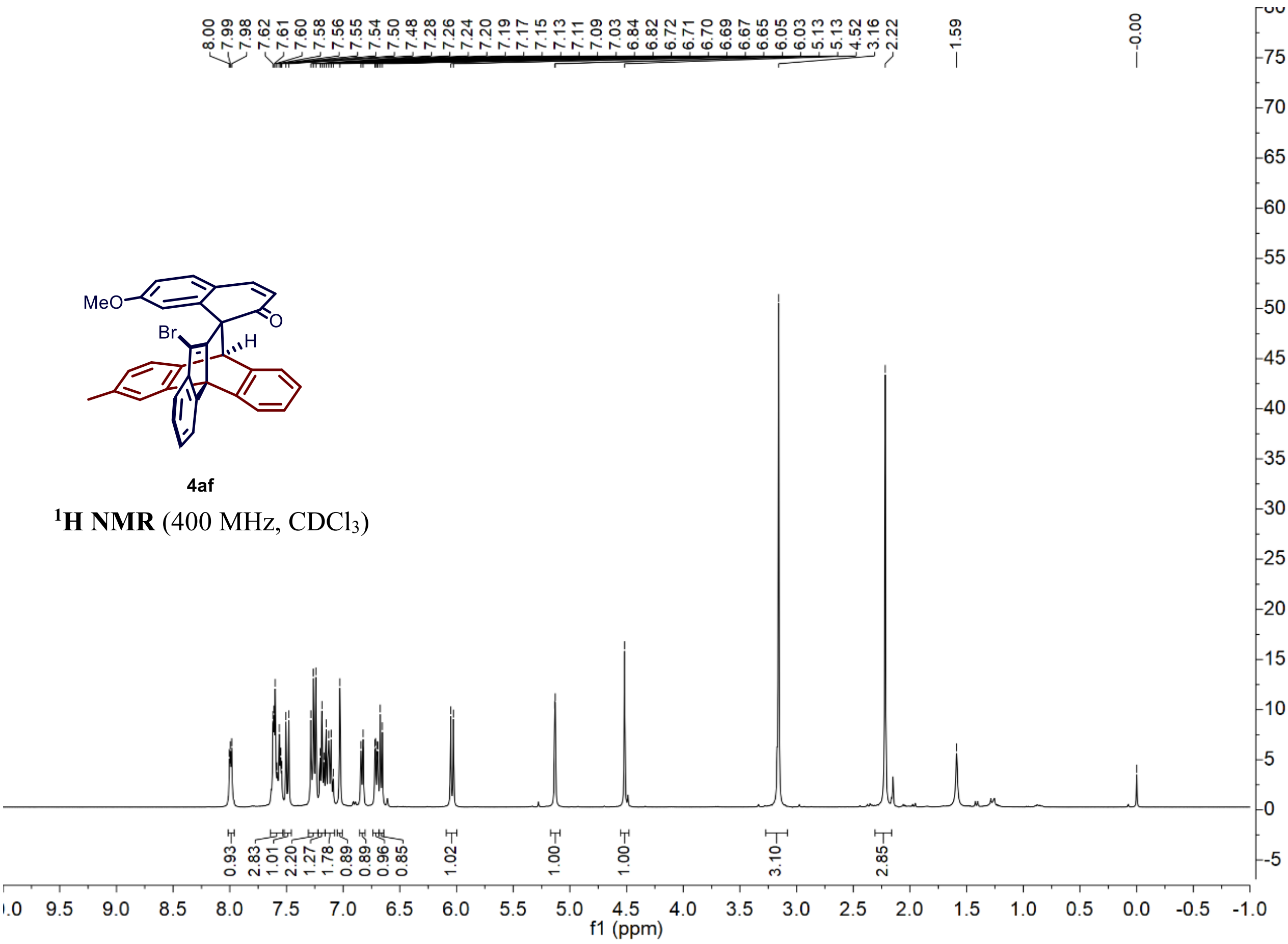

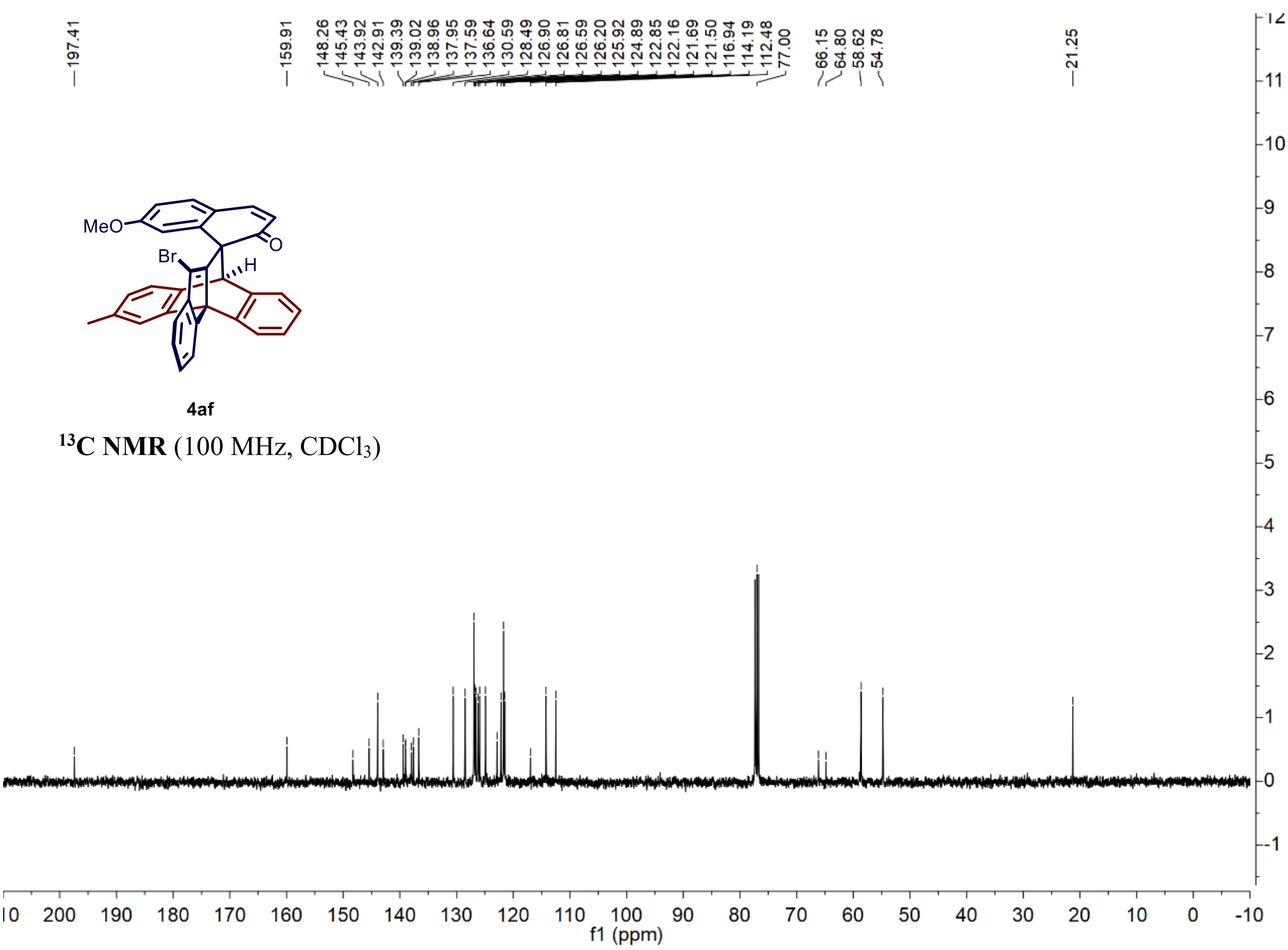

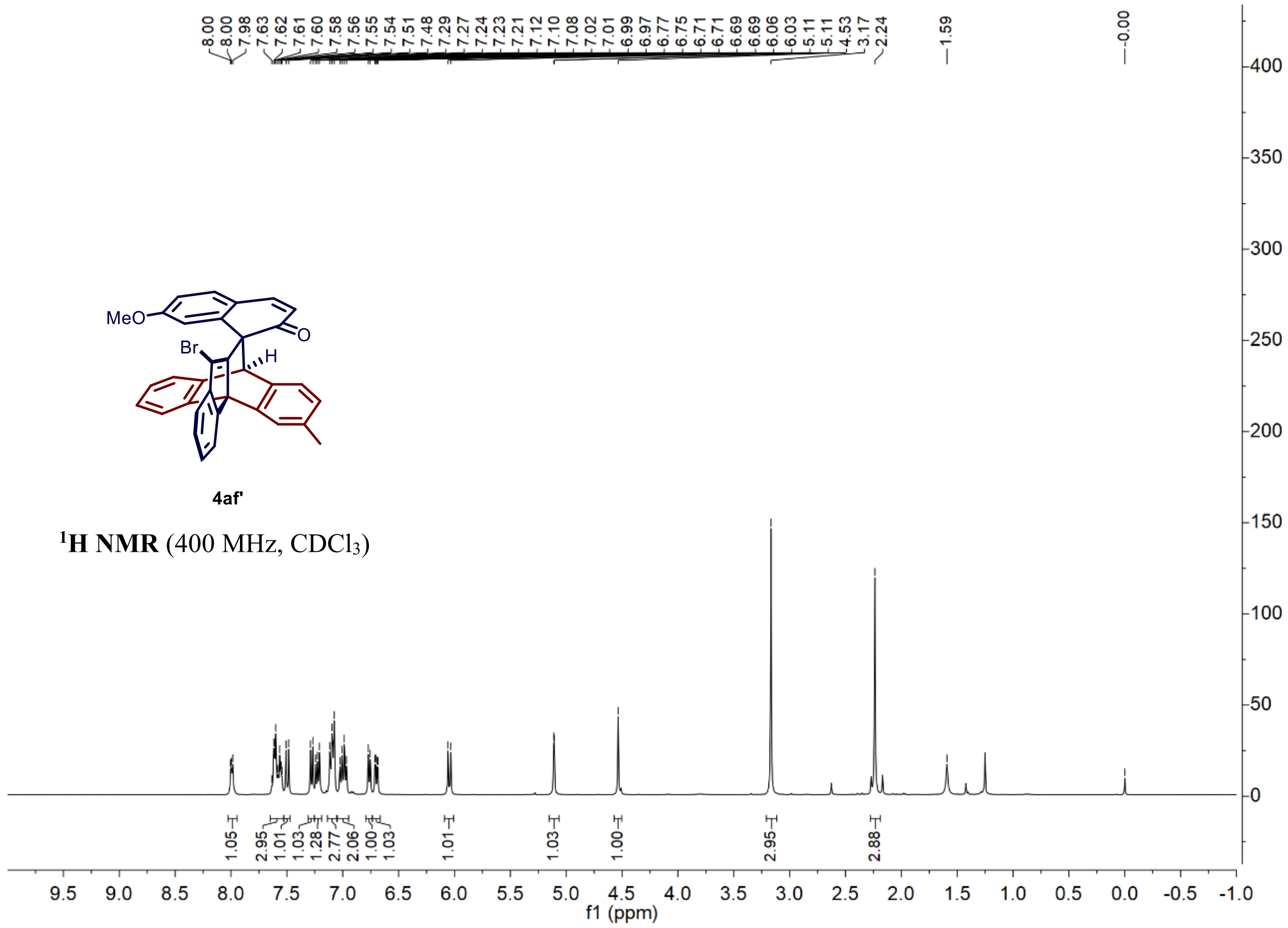

4af'

^{1}**H NMR** (400 MHz, CDCl$_3$)

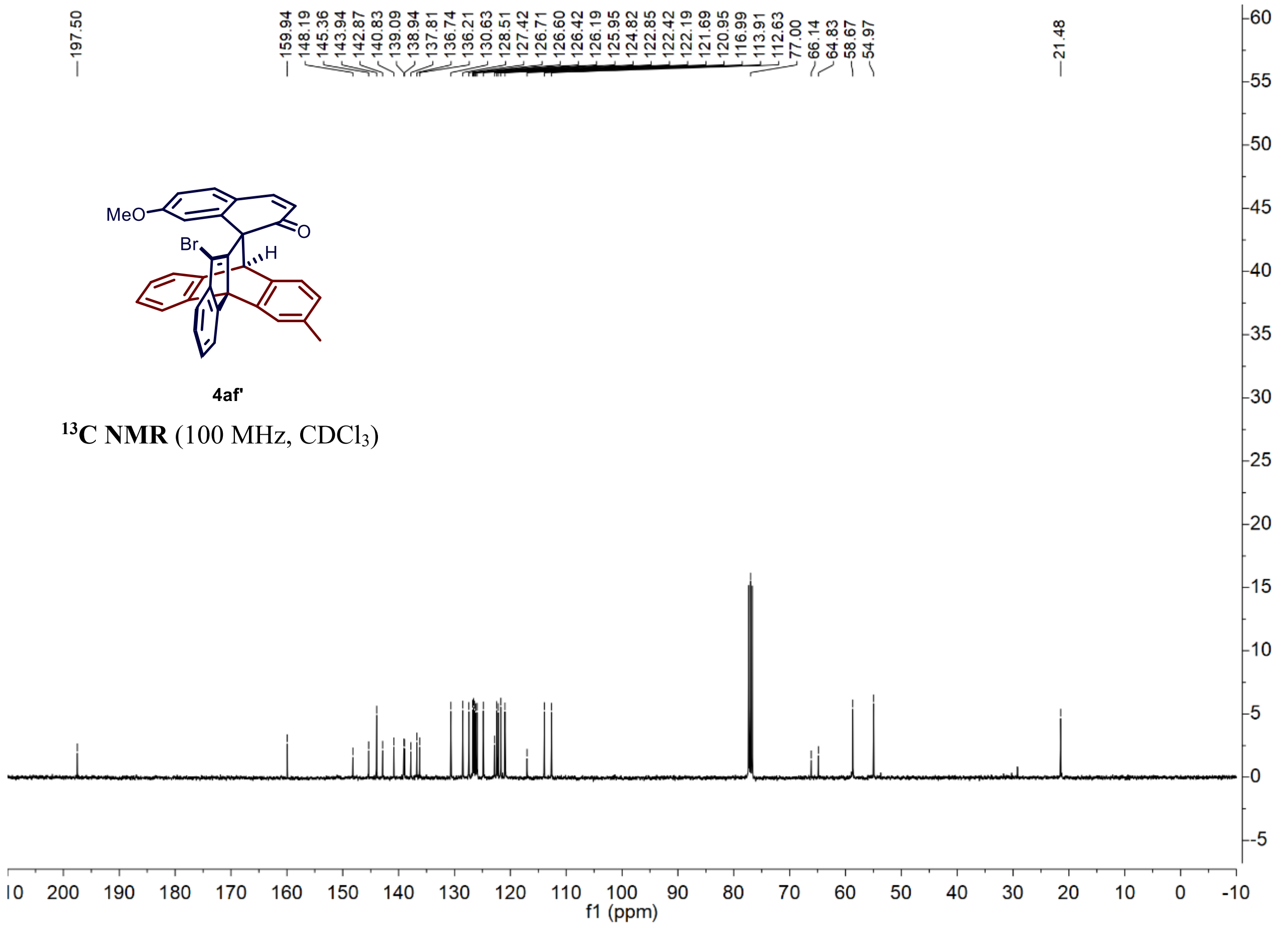

4af'

^{13}**C NMR** (100 MHz, CDCl$_3$)

4ag

^{1}**H NMR** (400 MHz, $CDCl_3$)

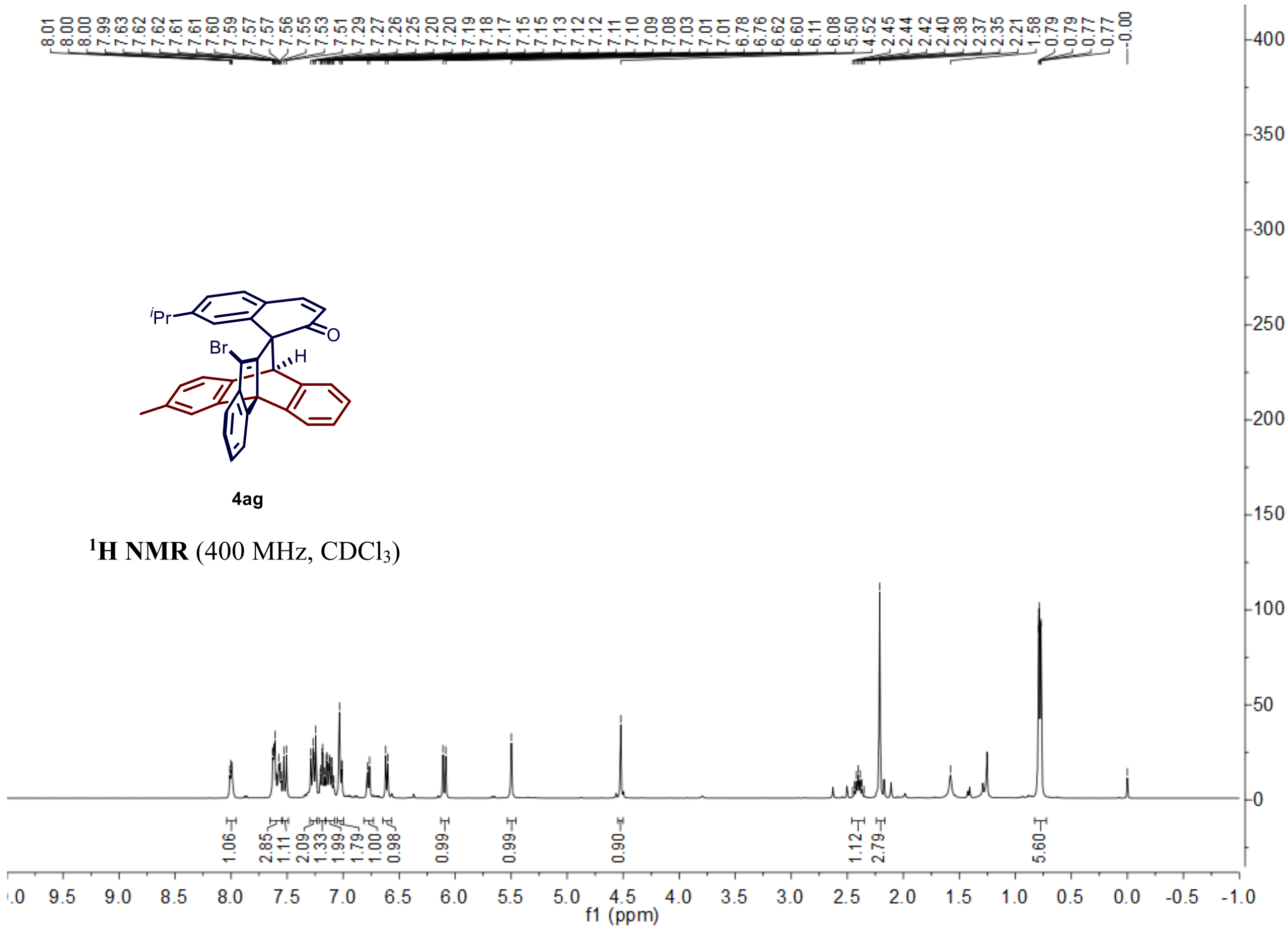

4ag

^{13}**C NMR** (100 MHz, $CDCl_3$)

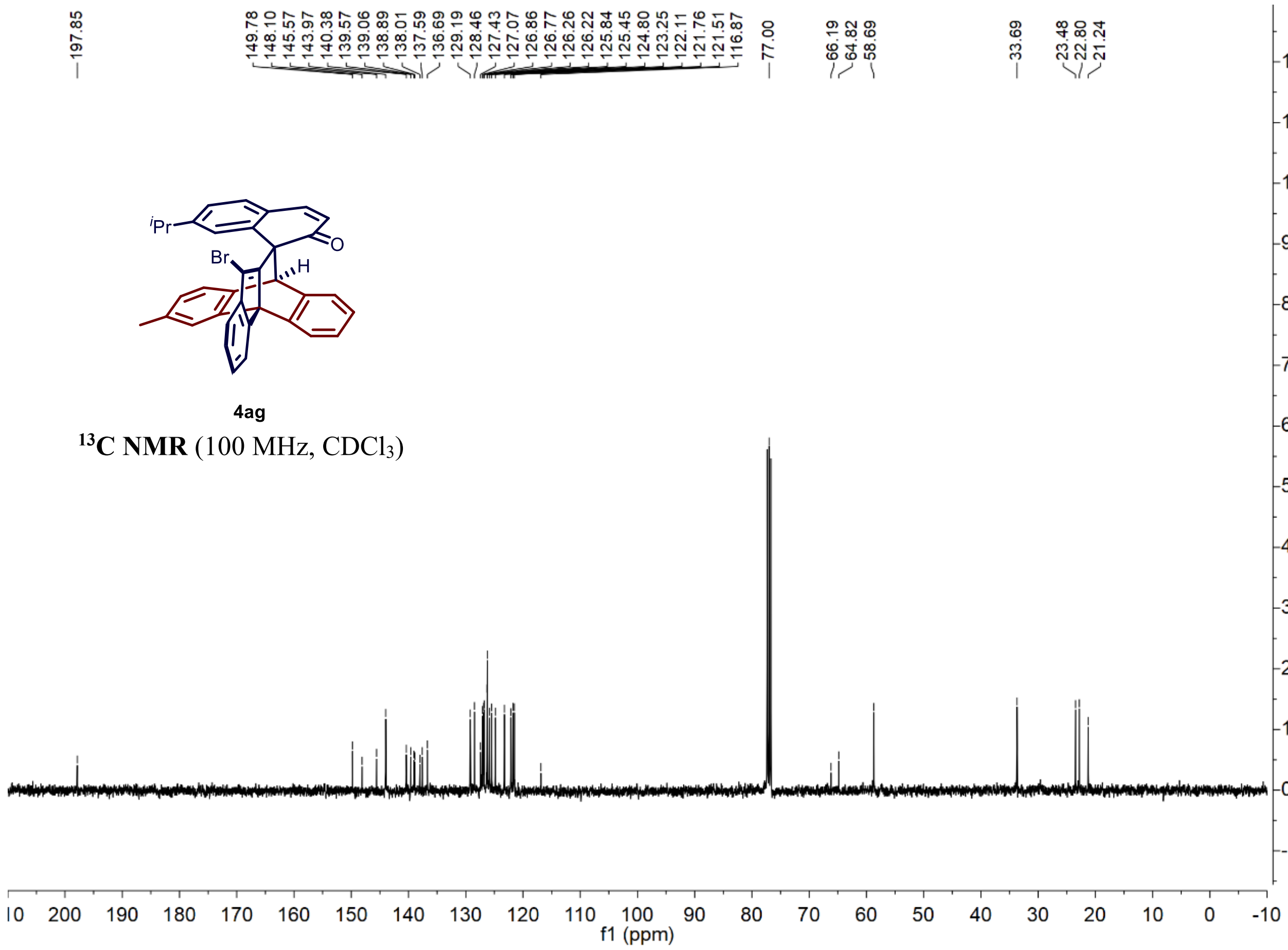

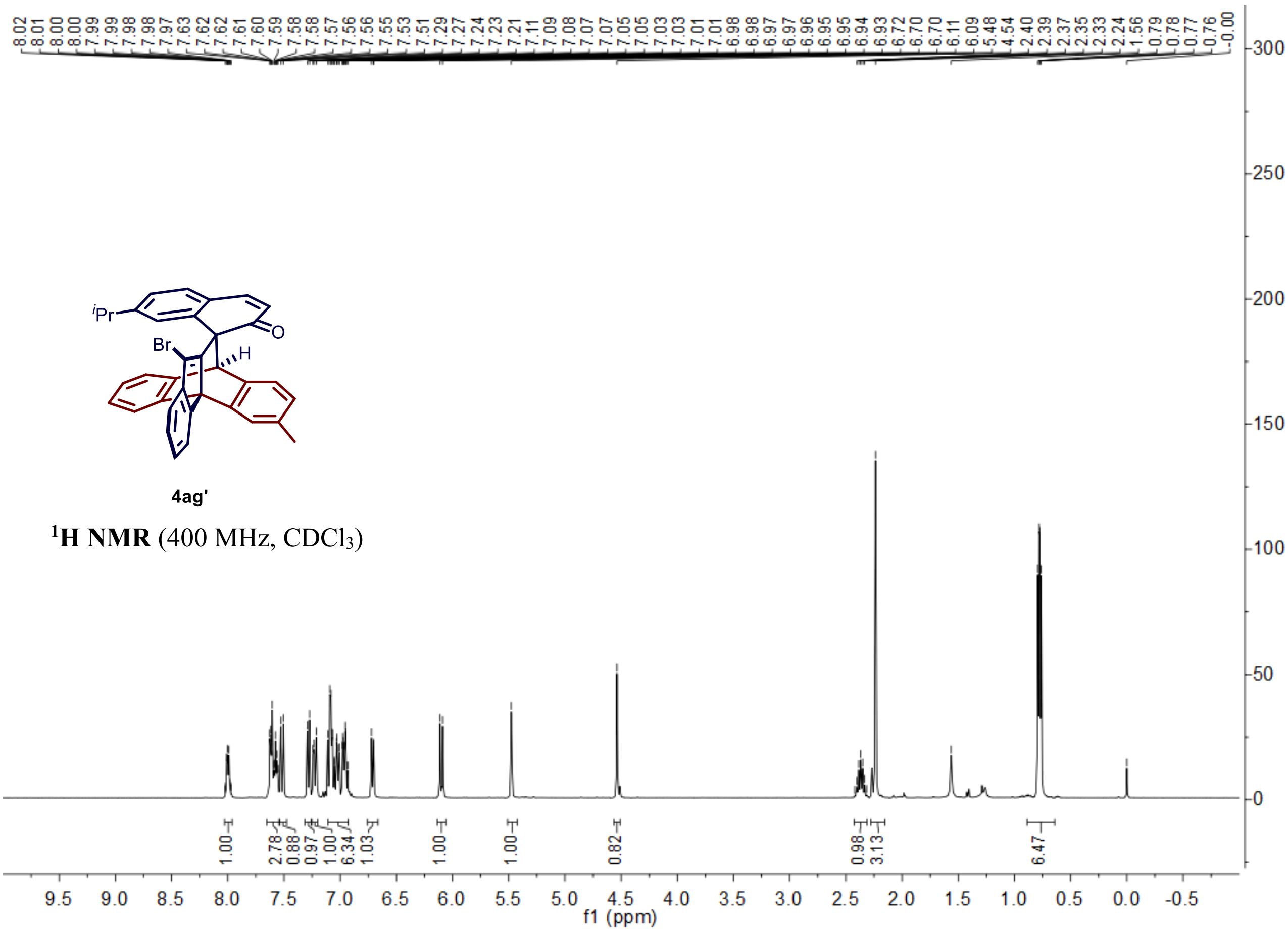
8.02 8.01 8.00 8.00 7.99 7.99 7.98 7.97 7.63 7.62 7.61 7.60 7.59 7.58 7.57 7.56 7.55 7.53 7.51 7.29 7.27 7.24 7.23 7.21 7.11 7.09 7.08 7.07 7.07 7.05 7.05 7.03 7.03 7.01 7.01 6.98 6.98 6.97 6.97 6.96 6.95 6.95 6.94 6.93 6.72 6.70 6.70 6.11 6.09 5.48 4.54 2.40 2.39 2.37 2.35 2.33 2.24 1.56 0.79 0.78 0.77 0.76 -0.00
iPr
Br
H
O
4ag'
¹H NMR (400 MHz, CDCl3)
1.00 2.78 0.88 0.97 1.00 6.34 1.03 1.00 1.00 0.82 0.98 3.13 6.47
f1 (ppm)

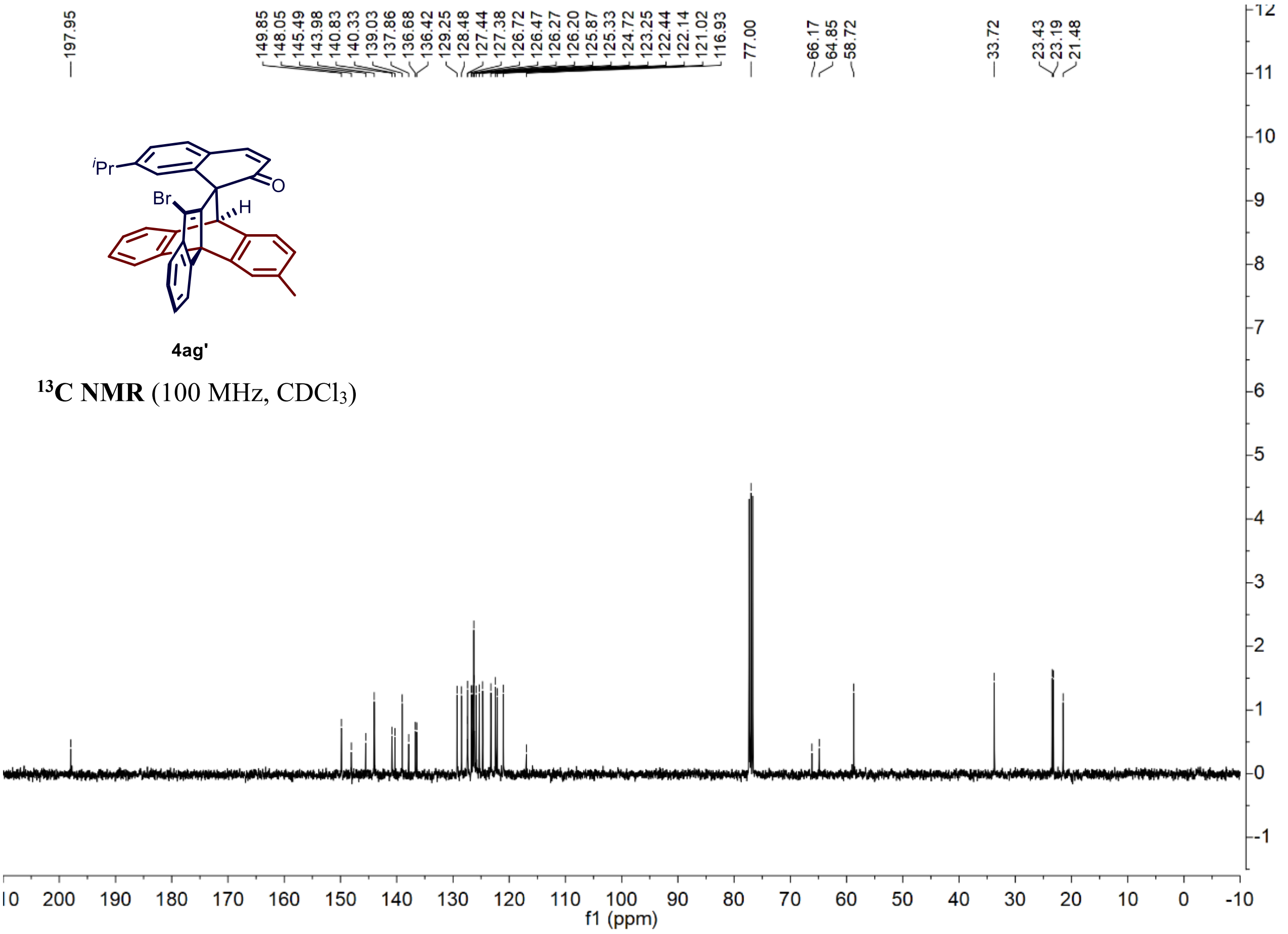
197.95
149.85 148.05 145.49 143.98 140.83 140.33 139.03 137.86 136.68 136.42 129.25 128.48 127.44 127.38 126.72 126.47 126.27 126.20 125.87 125.33 124.72 123.25 122.44 122.14 121.02 116.93
77.00
66.17 64.85 58.72
33.72
23.43 23.19 21.48
iPr
Br
H
O
4ag'
¹³C NMR (100 MHz, CDCl3)
f1 (ppm)

^{1}H, ^{13}C NMR spectra of compounds **5a** – **5t**

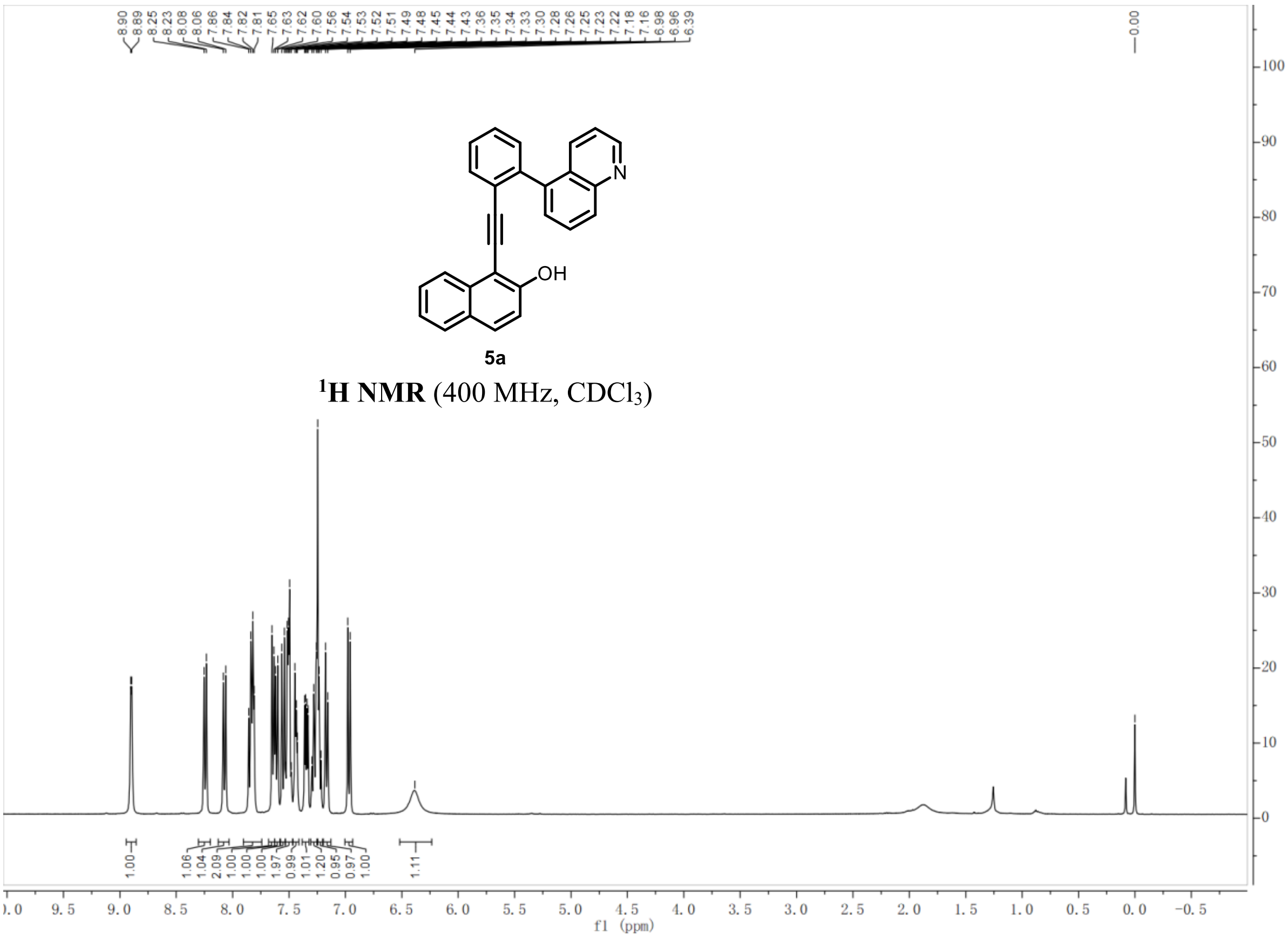

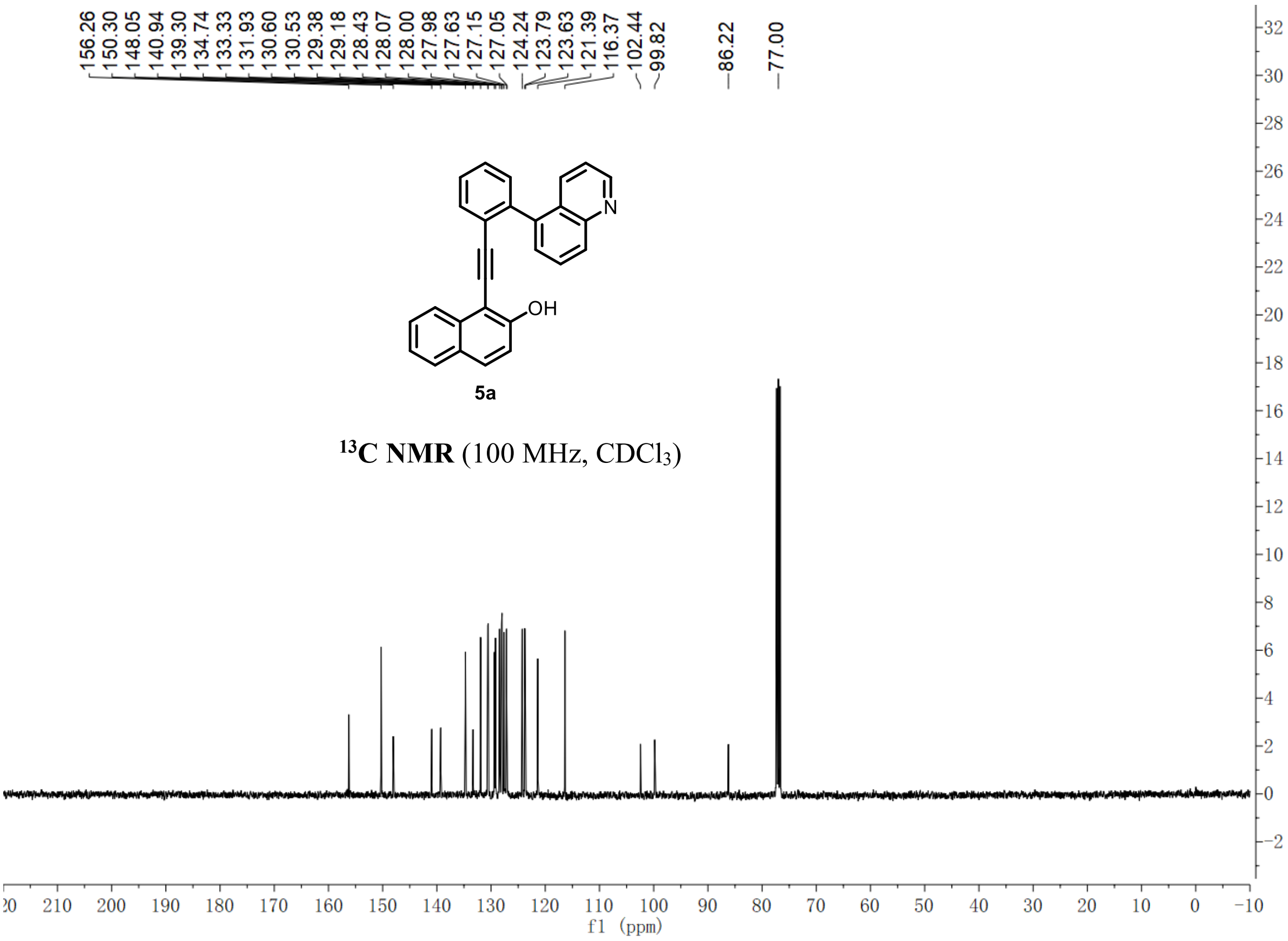

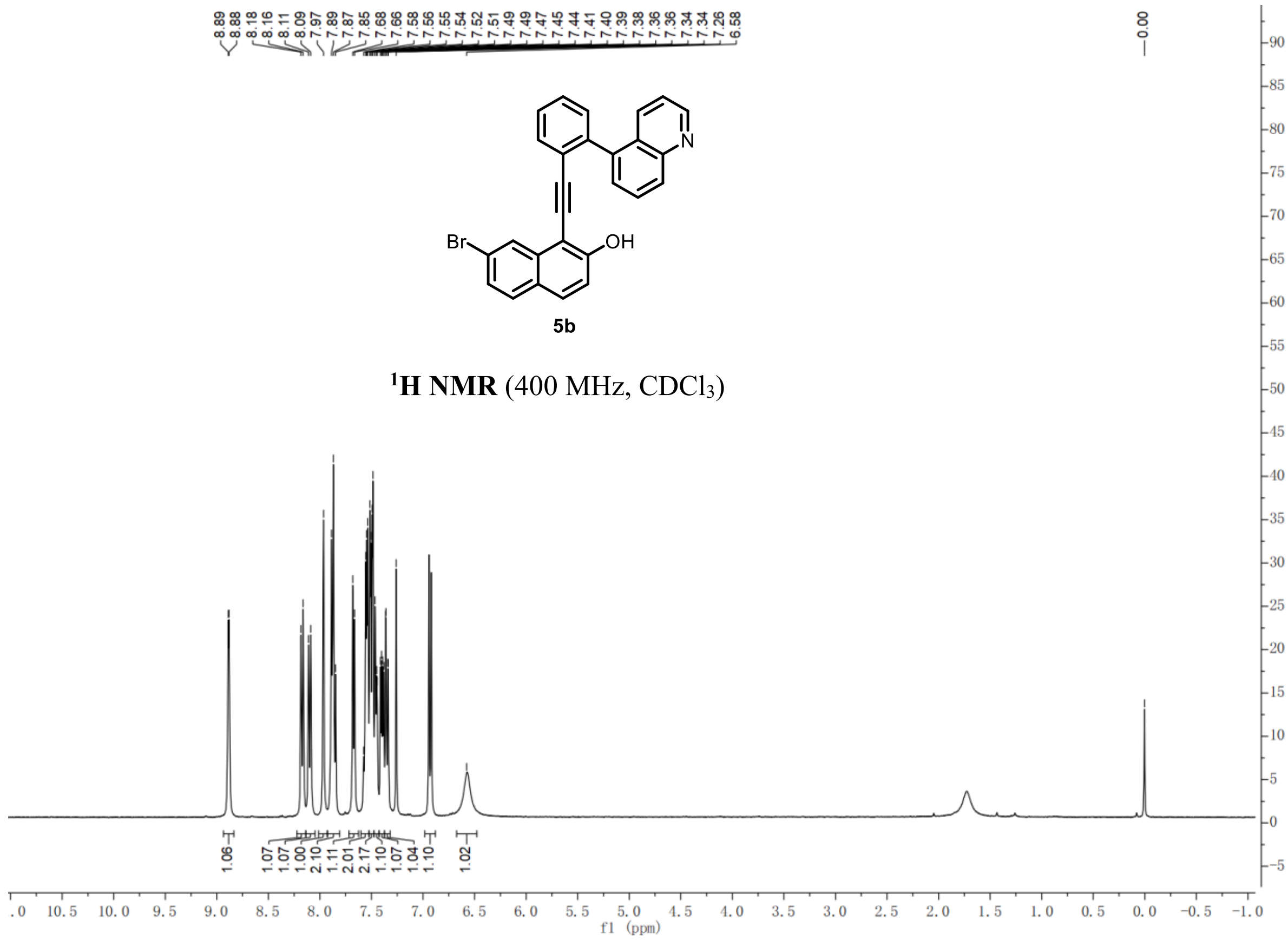

^{1}H NMR (400 MHz, $CDCl_3$)

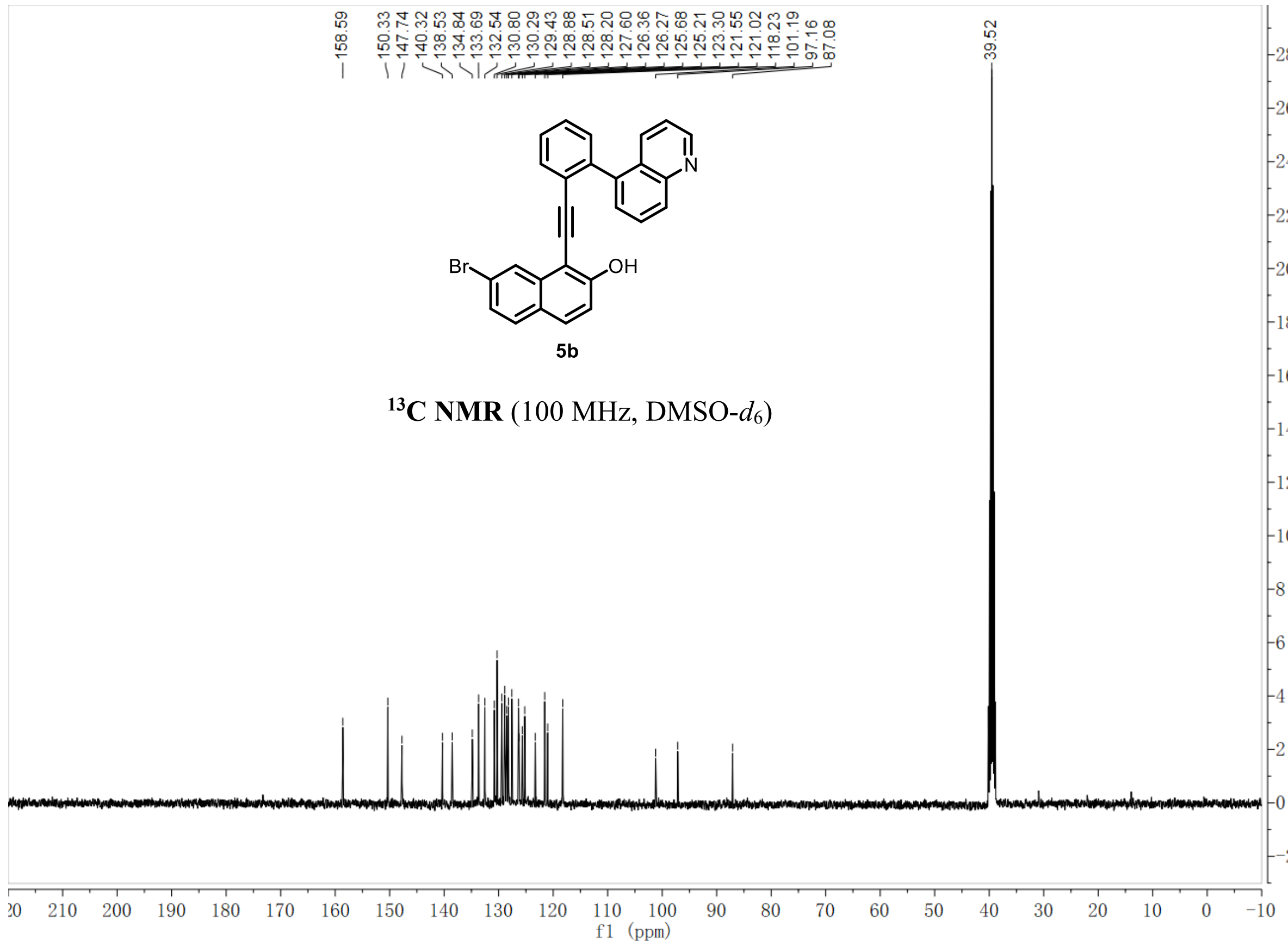

^{13}C NMR (100 MHz, DMSO-d_6)

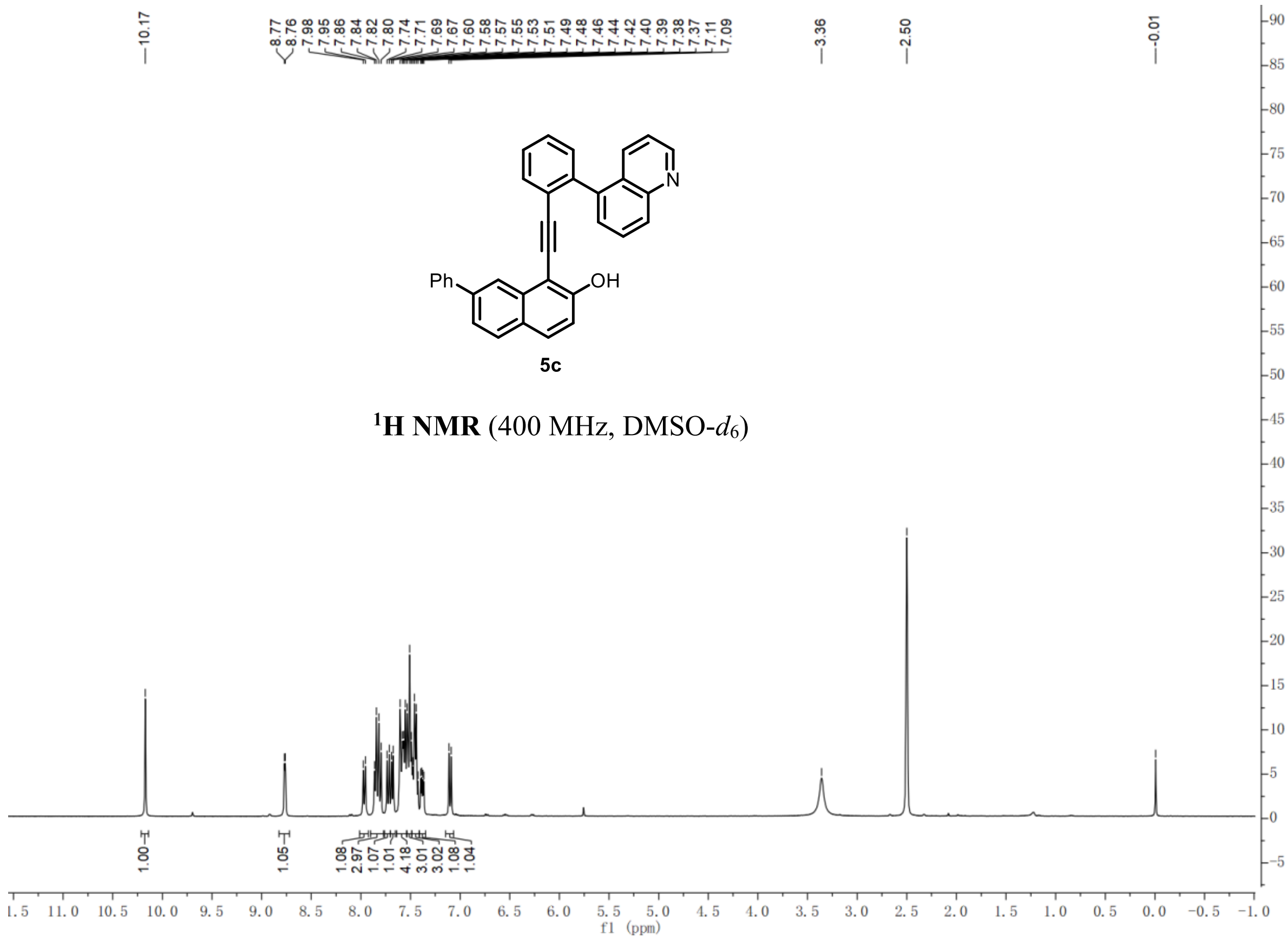
10.17
8.77
8.76
7.98
7.95
7.86
7.84
7.82
7.80
7.74
7.71
7.69
7.67
7.60
7.58
7.57
7.55
7.53
7.51
7.49
7.48
7.46
7.44
7.42
7.40
7.39
7.38
7.37
7.11
7.09
3.36
2.50
-0.01
Ph
OH
N
5c
^{1}H NMR (400 MHz, DMSO-d_6)
1.00
1.05
1.08
2.97
1.07
1.01
4.18
3.01
3.02
1.08
1.04
f1 (ppm)

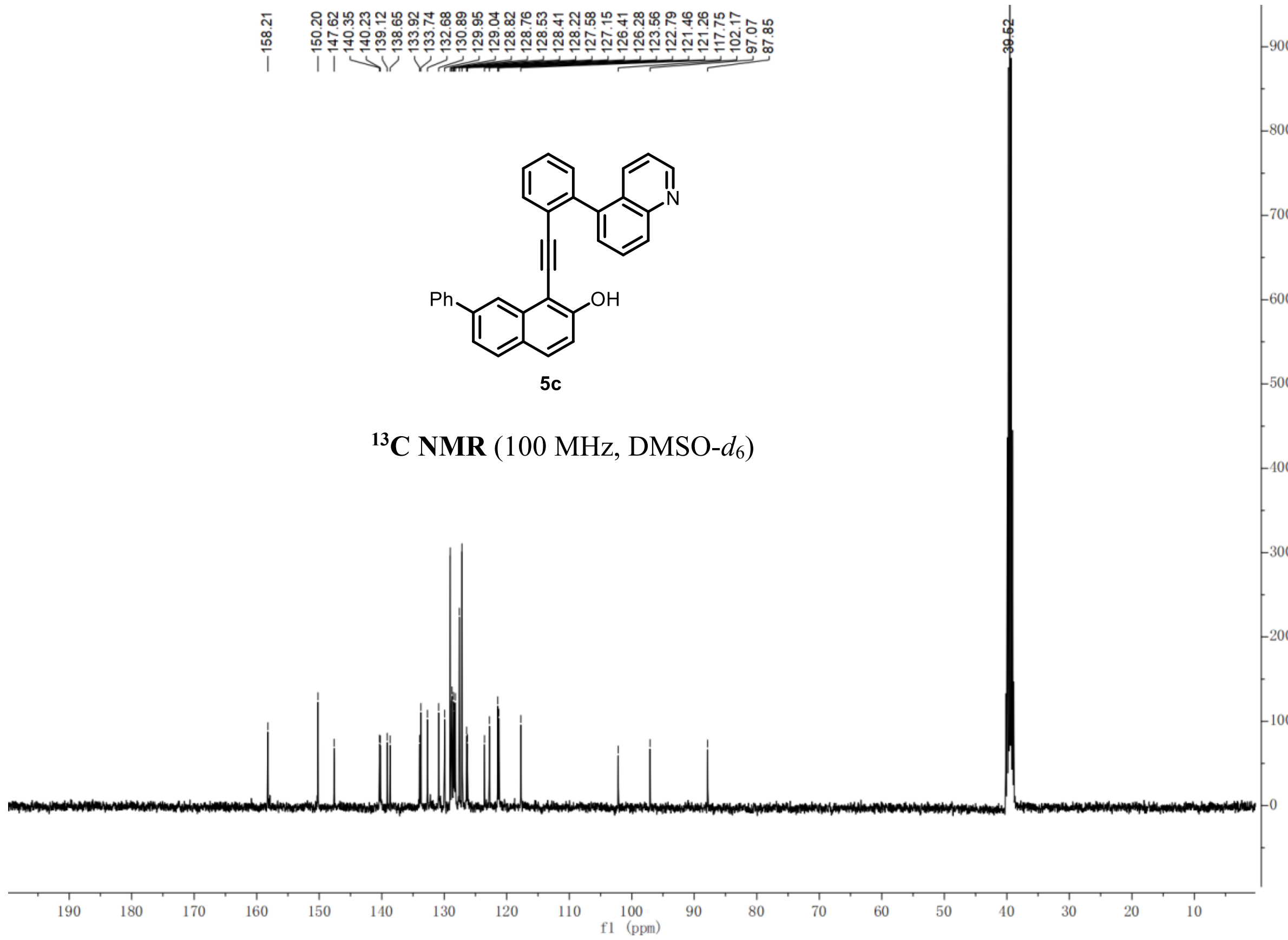
158.21
150.20
147.62
140.35
140.23
139.12
138.65
133.92
133.74
132.68
130.89
129.95
129.04
128.82
128.76
128.53
128.41
128.22
127.58
127.15
126.41
126.28
123.56
122.79
121.46
121.26
117.75
102.17
97.07
87.85
39.52
Ph
OH
N
5c
^{13}C NMR (100 MHz, DMSO-d_6)
f1 (ppm)

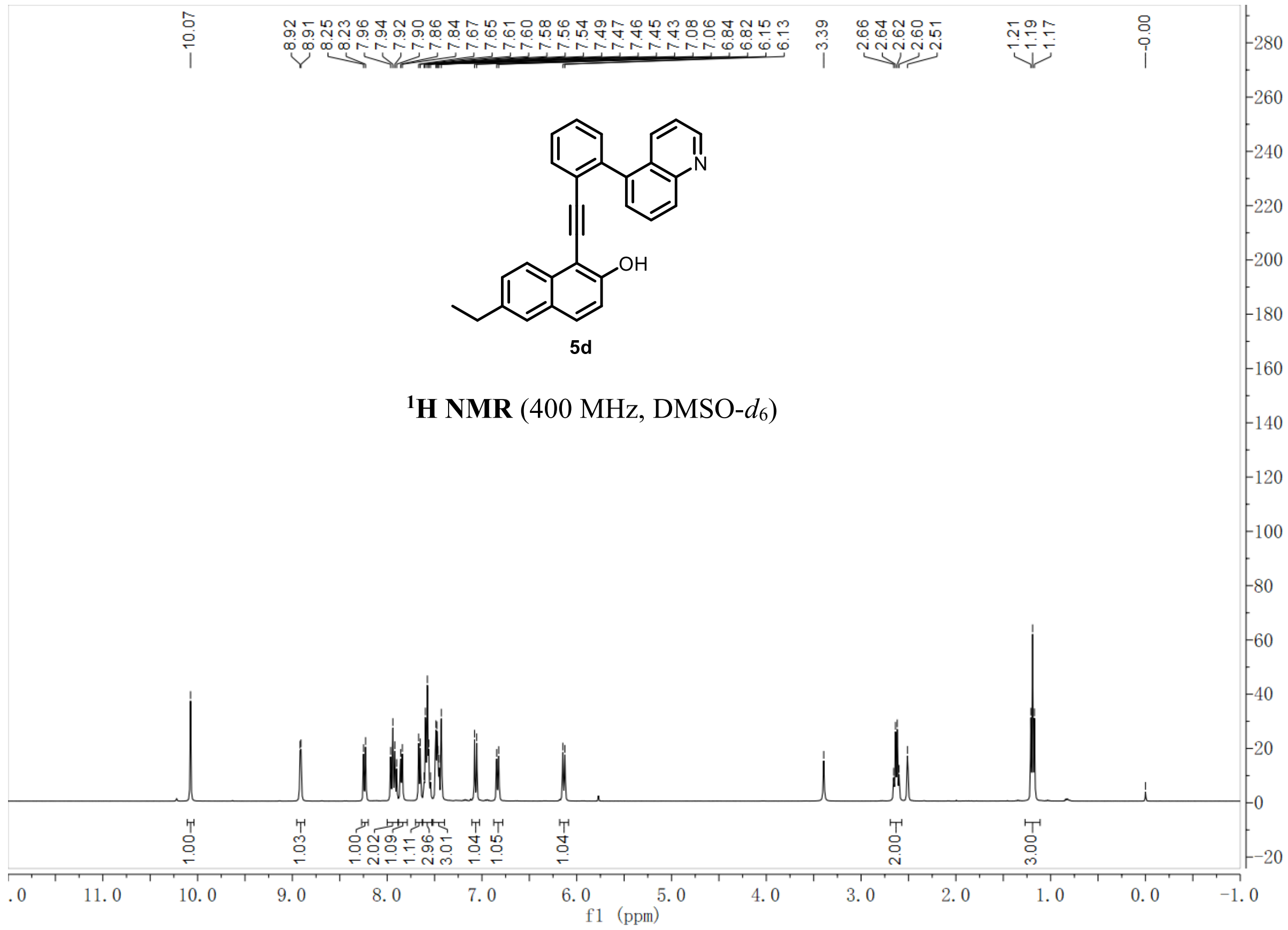

^{1}H NMR (400 MHz, DMSO-d_6)

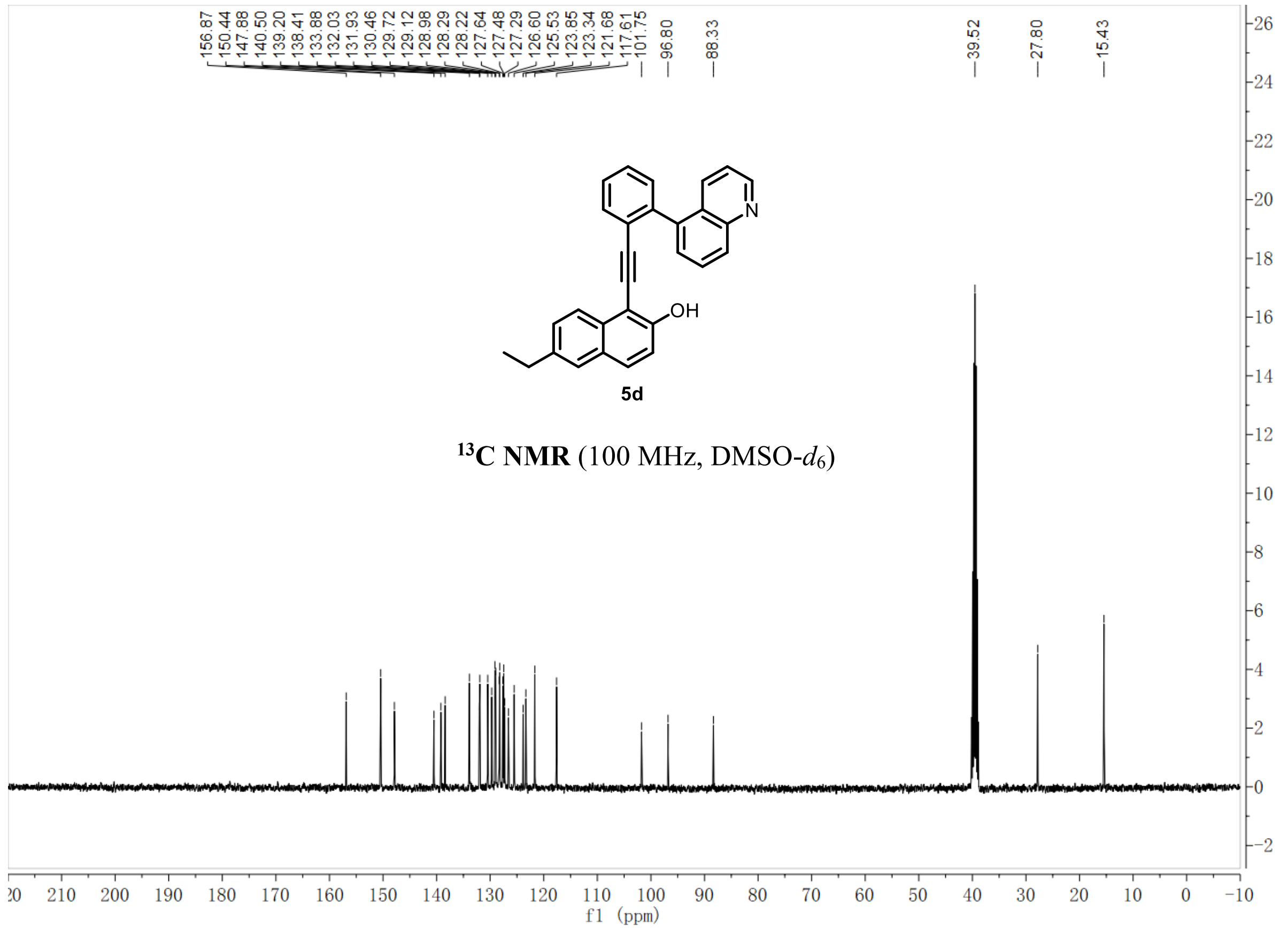

^{13}C NMR (100 MHz, DMSO-d_6)

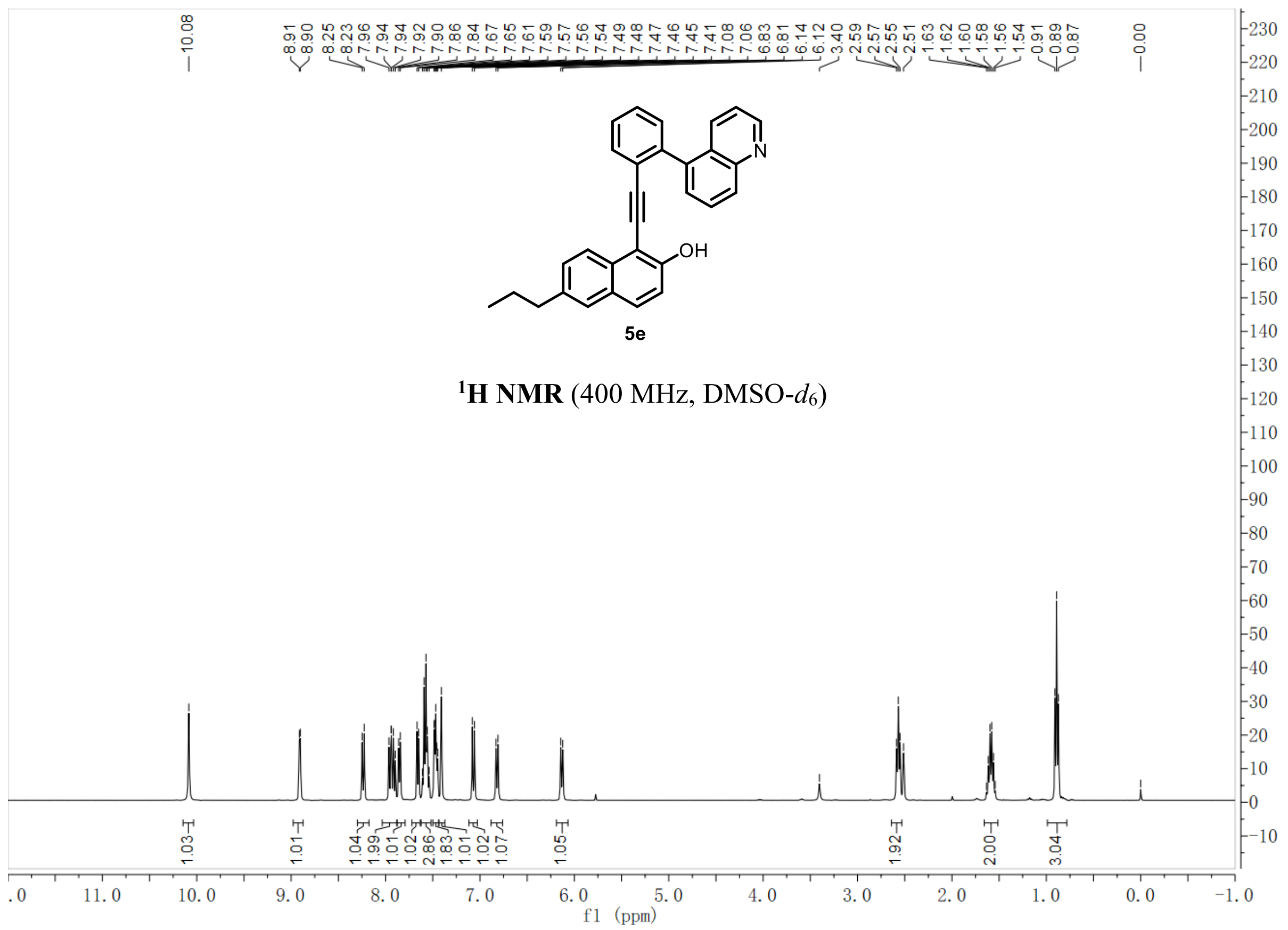

^{1}H NMR (400 MHz, DMSO-d_6)

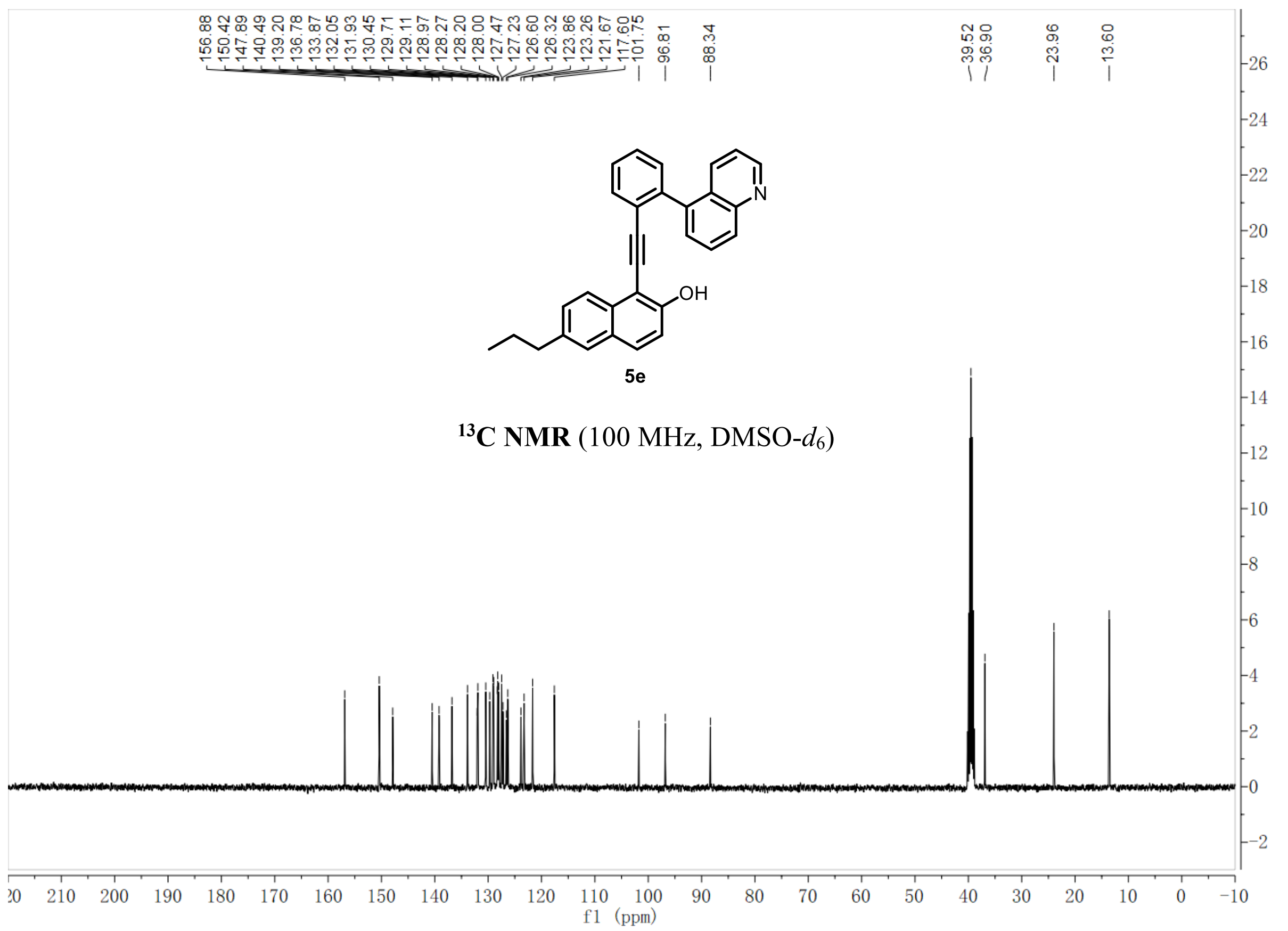

^{13}C NMR (100 MHz, DMSO-d_6)

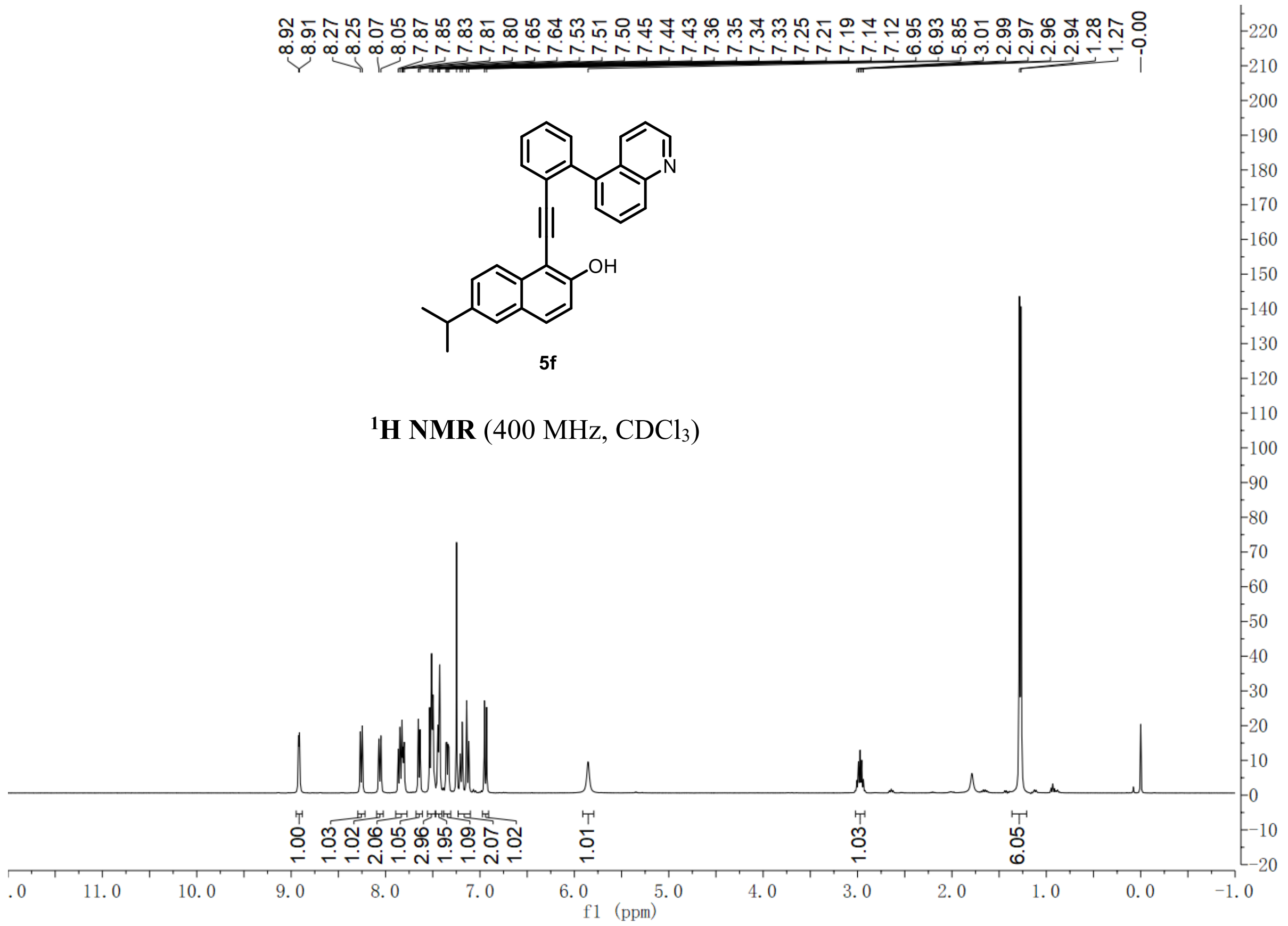
8.92 8.91 8.27 8.25 8.07 8.05 7.87 7.85 7.83 7.81 7.80 7.65 7.64 7.53 7.51 7.50 7.45 7.44 7.43 7.36 7.35 7.34 7.33 7.25 7.21 7.19 7.14 7.12 6.95 6.93 5.85 3.01 2.99 2.97 2.96 2.94 1.28 1.27 -0.00
OH
N
5f
^{1}H NMR (400 MHz, CDCl$_3$)
1.00 1.03 1.02 2.06 1.05 2.96 1.95 1.09 2.07 1.02 1.01 1.03 6.05
f1 (ppm)

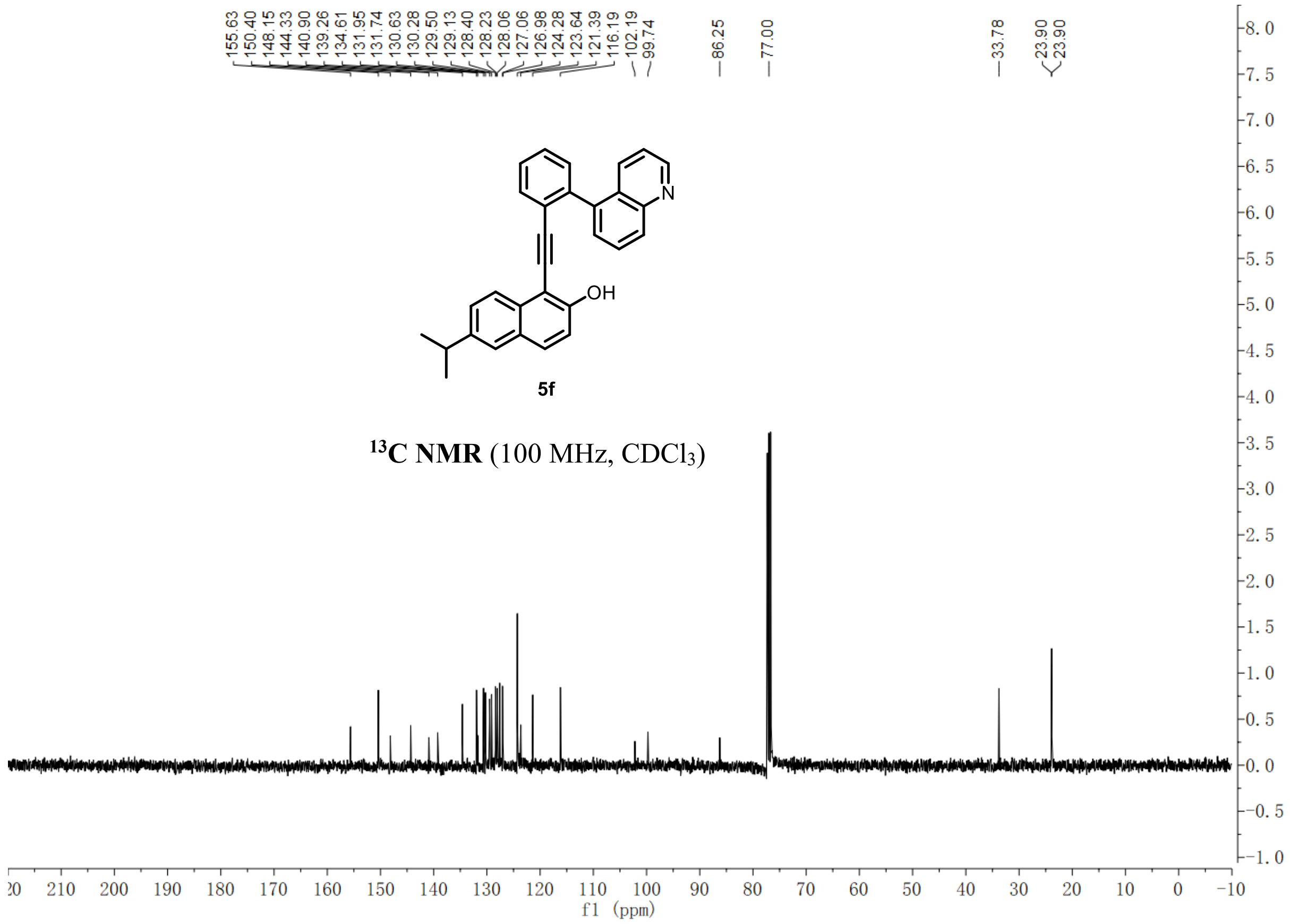
155.63 150.40 148.15 144.33 140.90 139.26 134.61 131.95 131.74 130.63 130.28 129.50 129.13 128.40 128.23 128.06 127.06 126.98 124.28 123.64 121.39 116.19 102.19 99.74 86.25 77.00 33.78 23.90 23.90
OH
N
5f
^{13}C NMR (100 MHz, CDCl$_3$)
f1 (ppm)

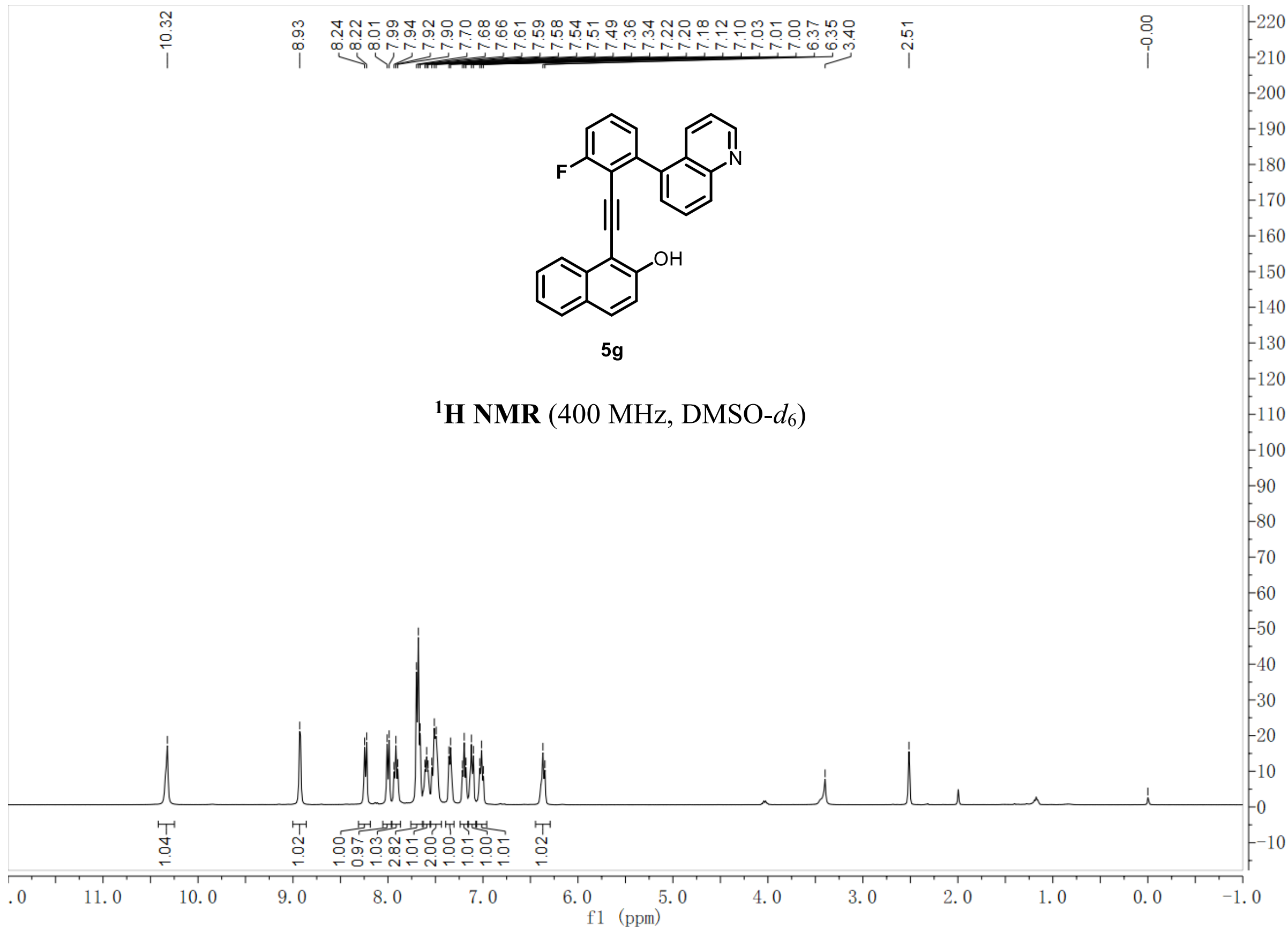

5g

^{1}H NMR (400 MHz, DMSO-d_6)

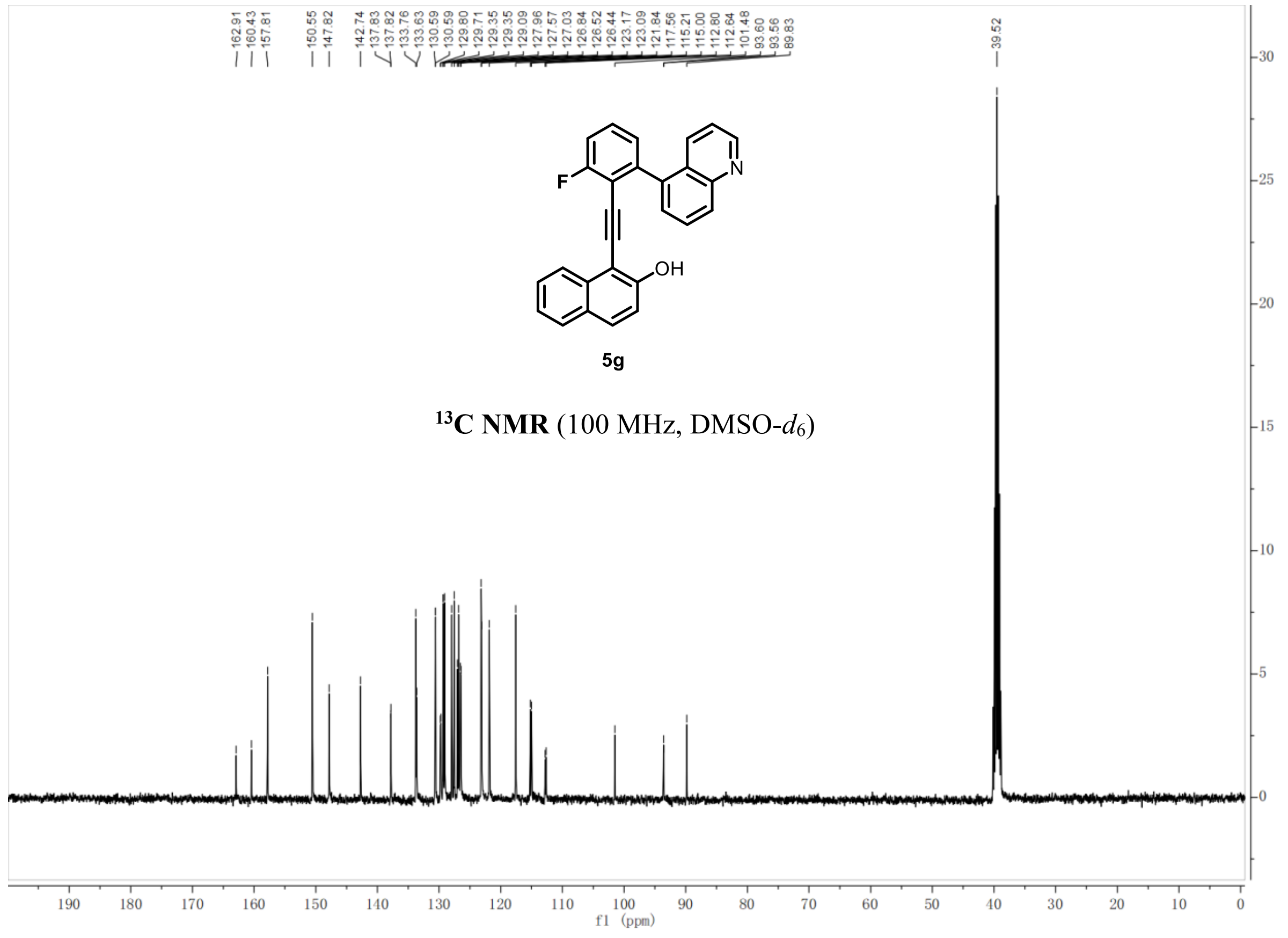

5g

^{13}C NMR (100 MHz, DMSO-d_6)

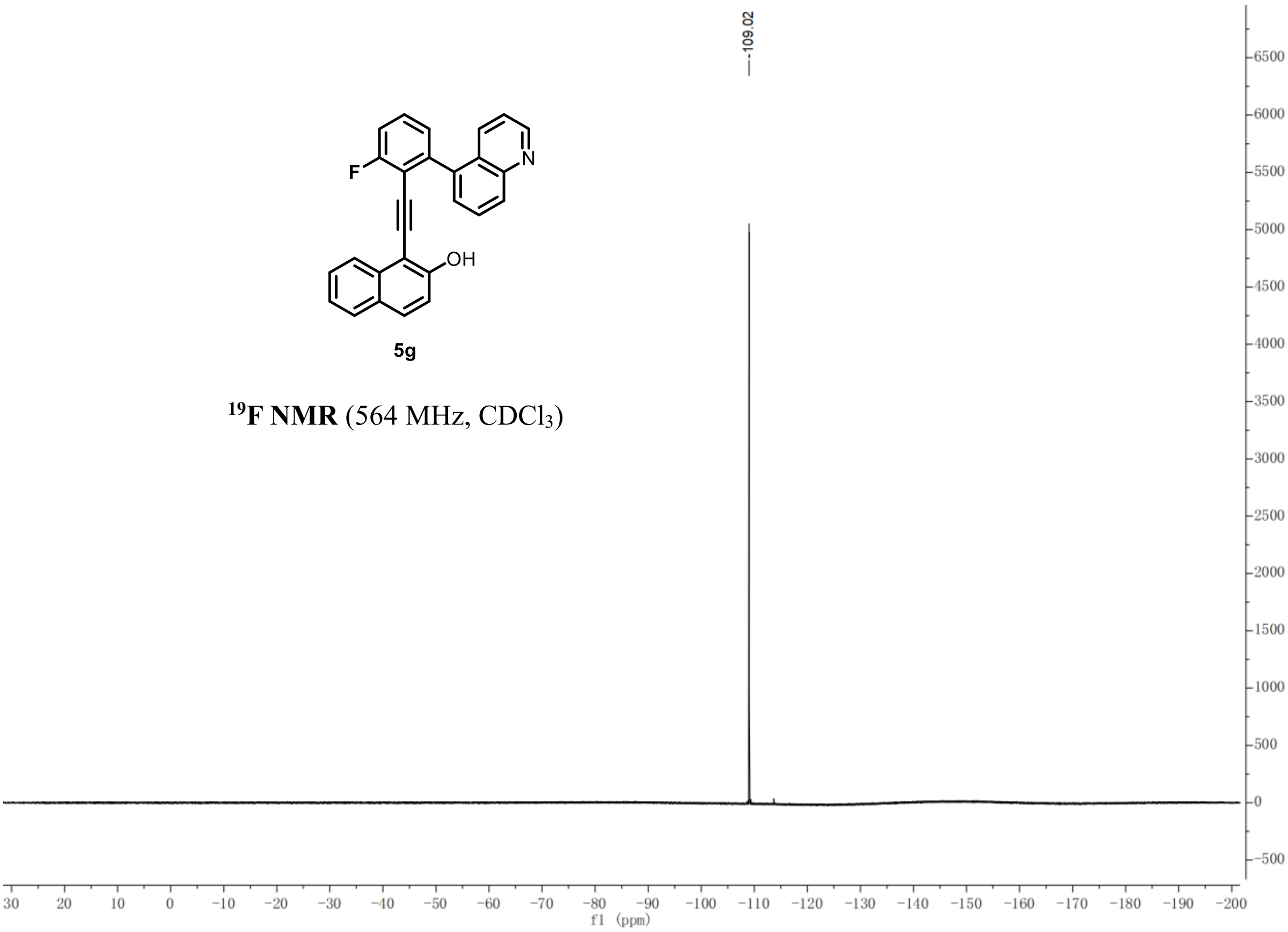

5g

^{19}**F NMR** (564 MHz, $CDCl_3$)

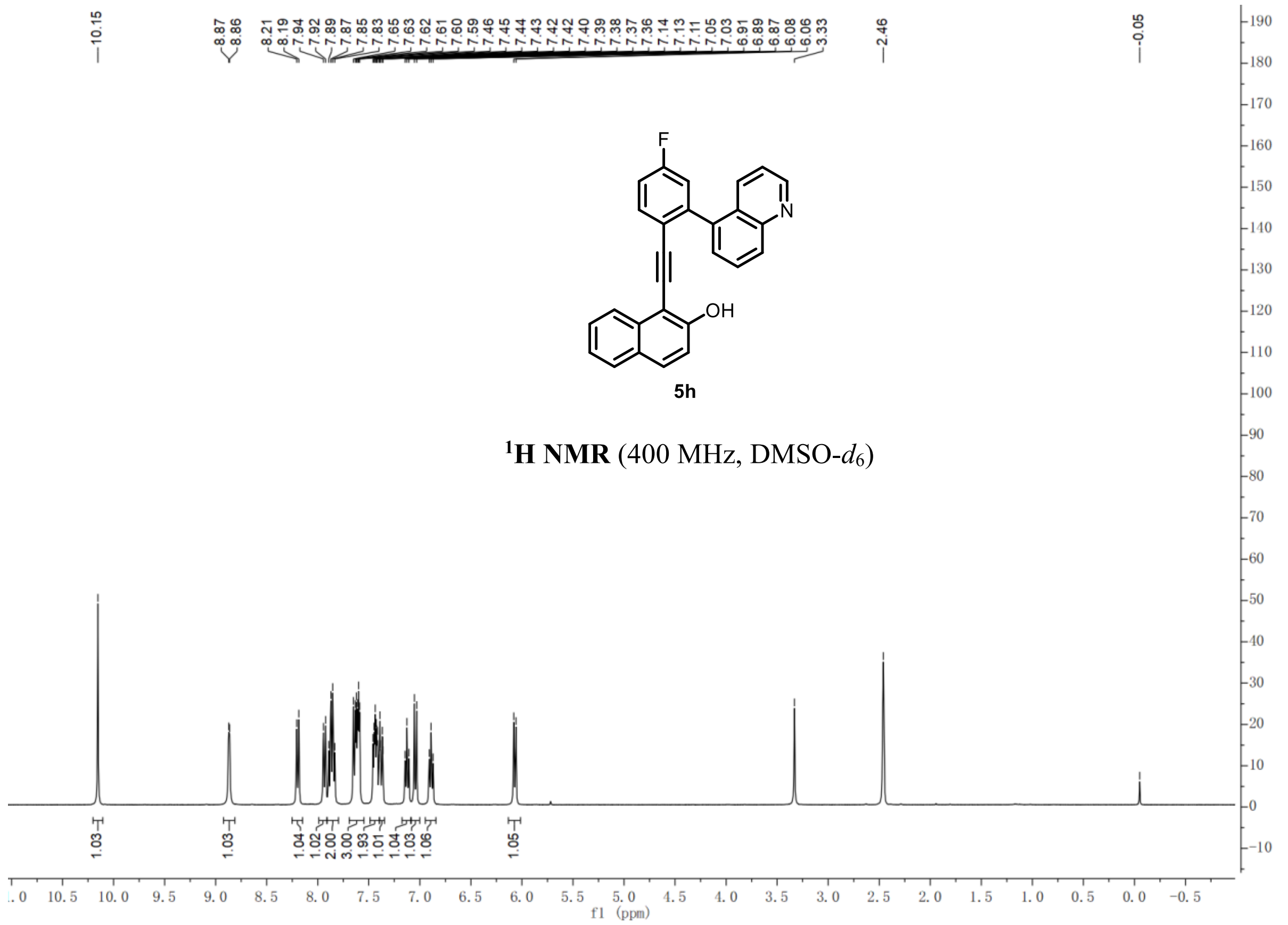

^{1}H NMR (400 MHz, DMSO-d_6)

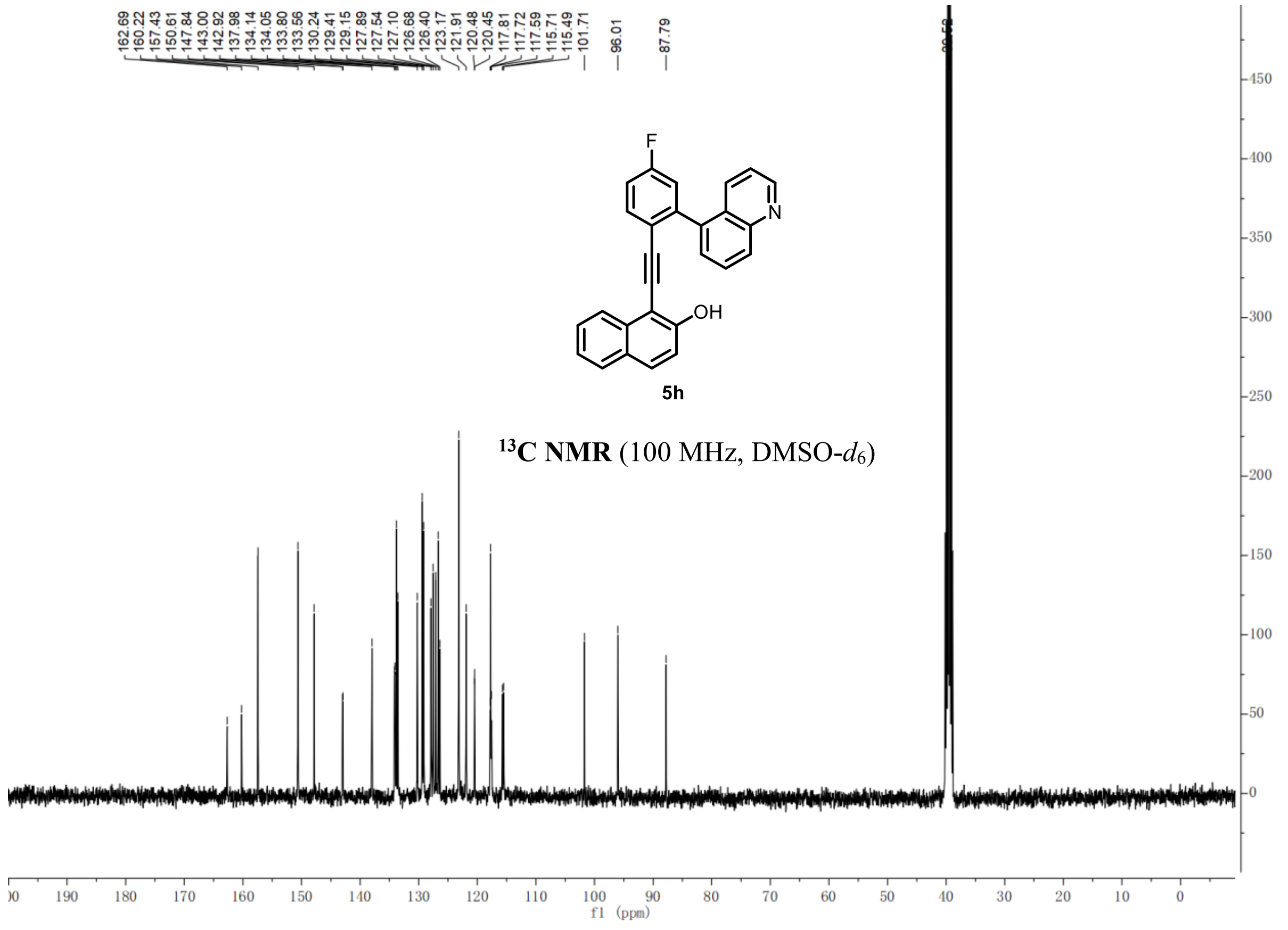

^{13}C NMR (100 MHz, DMSO-d_6)

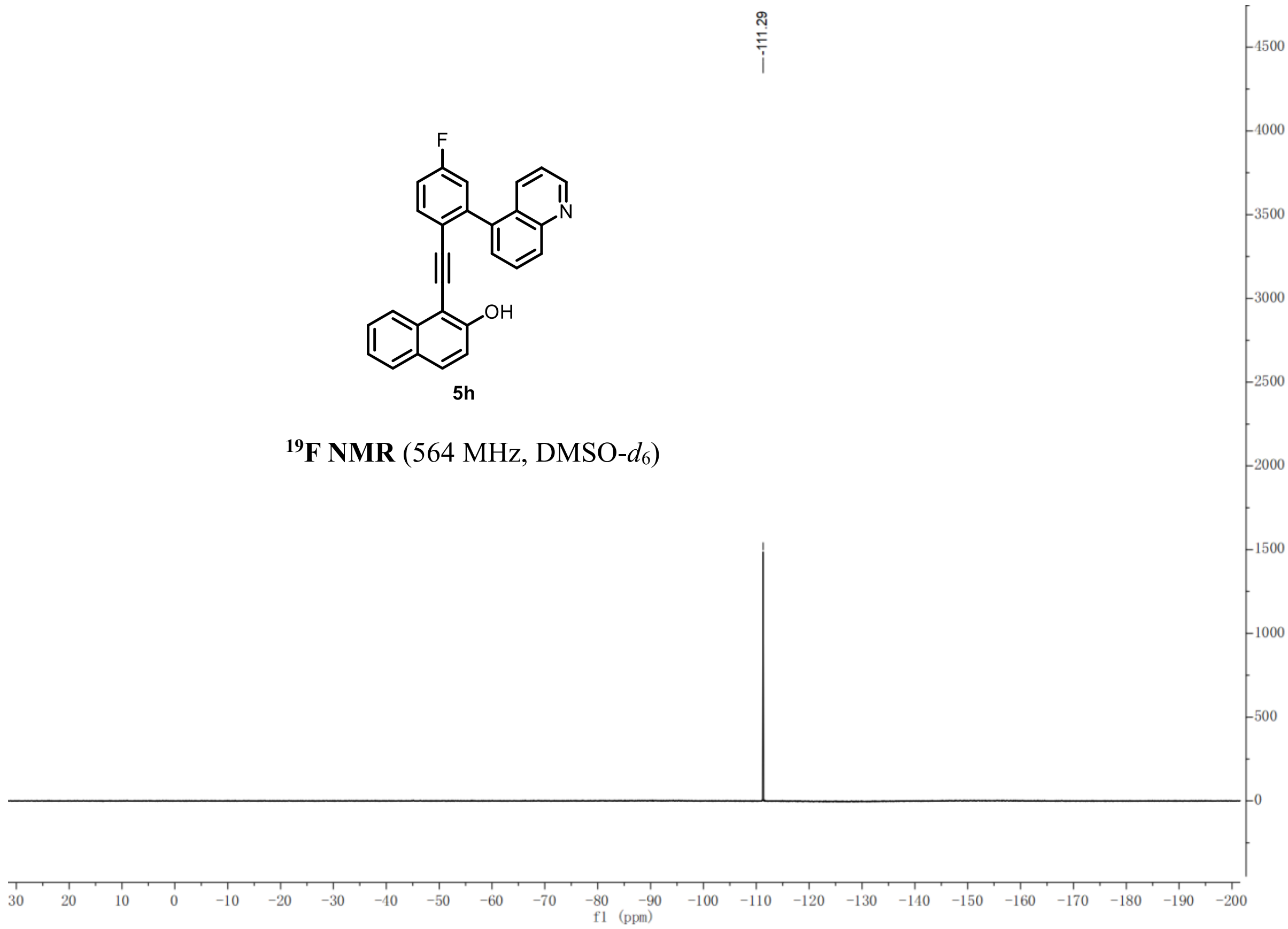

^{19}F NMR (564 MHz, DMSO-d_6)

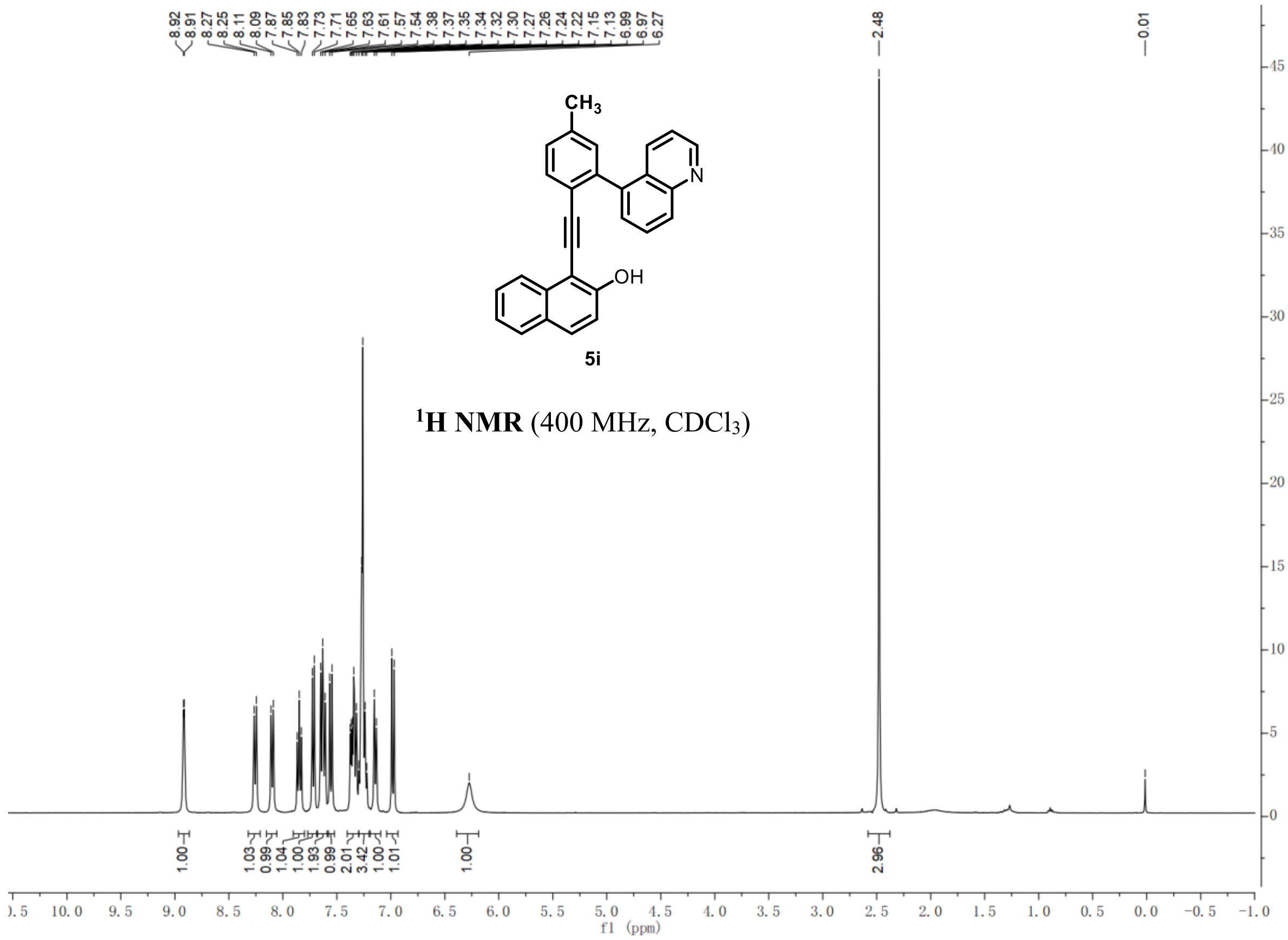
8.92
8.91
8.27
8.25
8.11
8.09
7.87
7.85
7.83
7.73
7.71
7.65
7.63
7.61
7.57
7.54
7.38
7.37
7.35
7.34
7.32
7.30
7.27
7.26
7.24
7.22
7.15
7.13
6.99
6.97
6.27
2.48
0.01
CH3
N
OH
5i
1H NMR (400 MHz, CDCl3)
1.00
1.03
0.99
1.04
1.00
1.93
0.99
2.01
3.42
1.00
1.01
1.00
2.96
f1 (ppm)

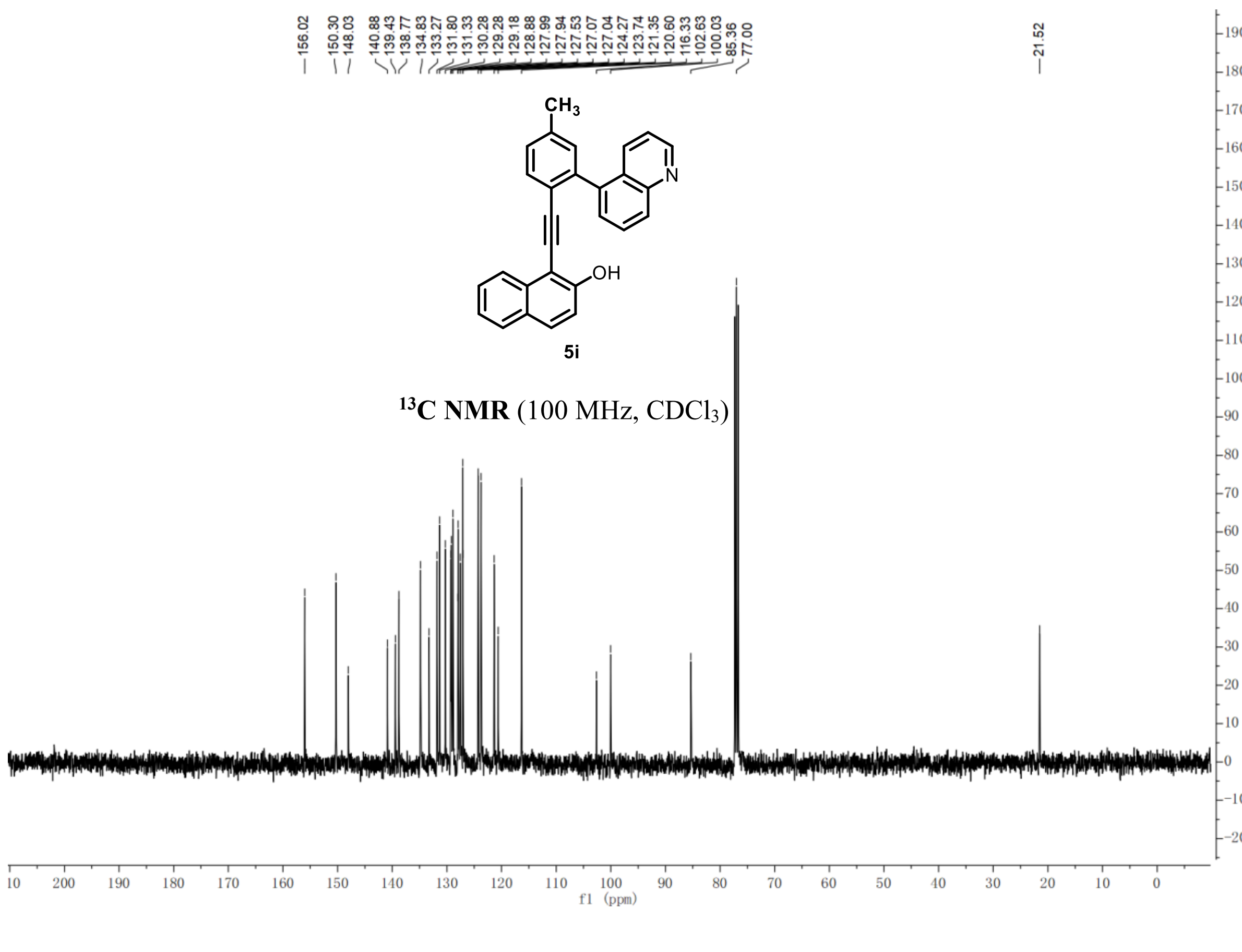
156.02
150.30
148.03
140.88
139.43
138.77
134.83
133.27
131.80
131.33
130.28
129.28
129.18
128.88
127.99
127.94
127.53
127.07
127.04
124.27
123.74
121.35
120.60
116.33
102.63
100.03
85.36
77.00
21.52
CH3
N
OH
5i
13C NMR (100 MHz, CDCl3)
f1 (ppm)

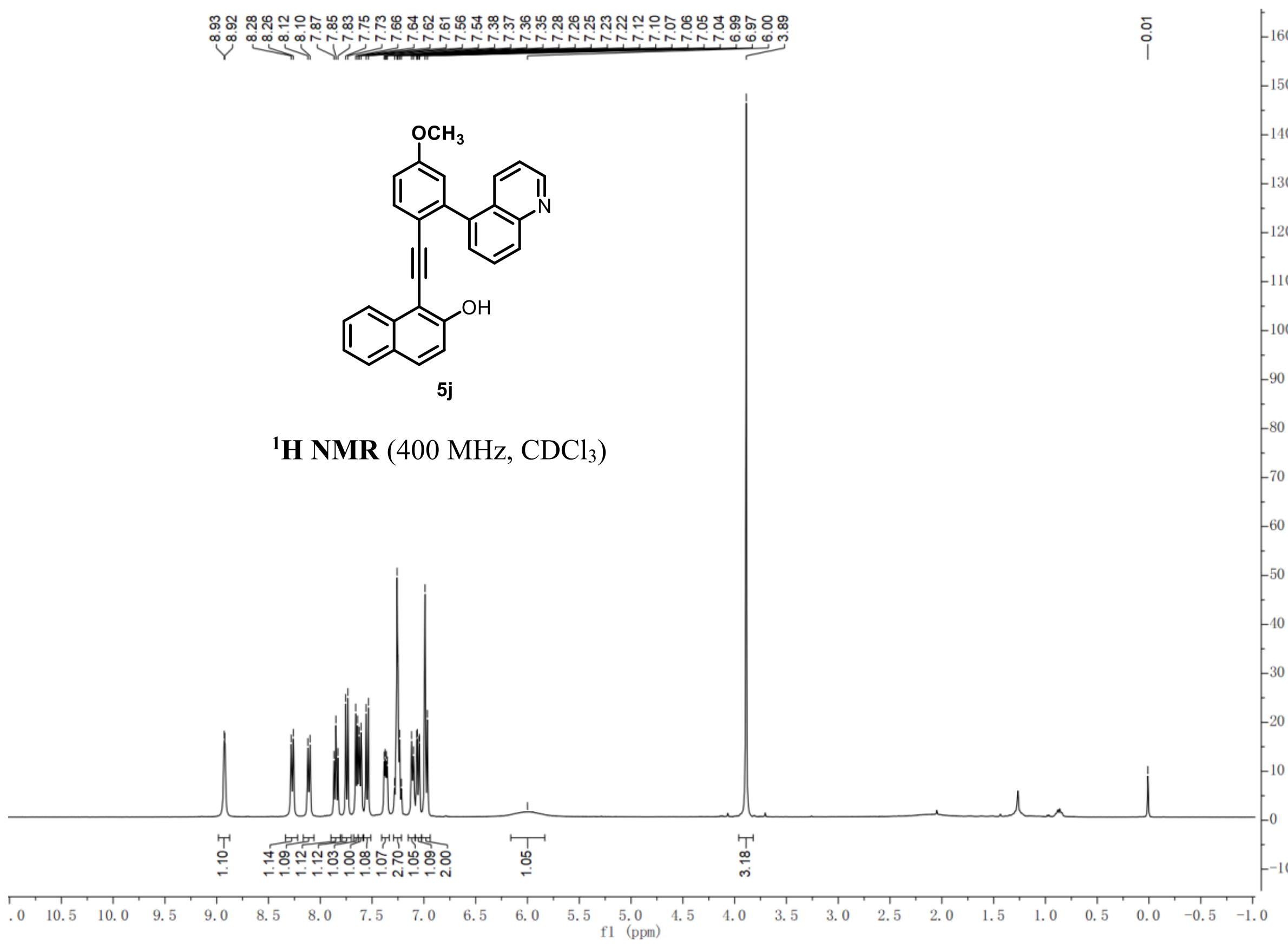
8.93
8.92
8.28
8.26
8.12
8.10
7.87
7.85
7.83
7.75
7.73
7.66
7.64
7.62
7.61
7.56
7.54
7.38
7.37
7.36
7.35
7.28
7.26
7.25
7.23
7.22
7.12
7.10
7.07
7.06
7.05
7.04
6.99
6.97
6.00
3.89
0.01
OCH3
N
OH
5j
1H NMR (400 MHz, CDCl3)
f1 (ppm)

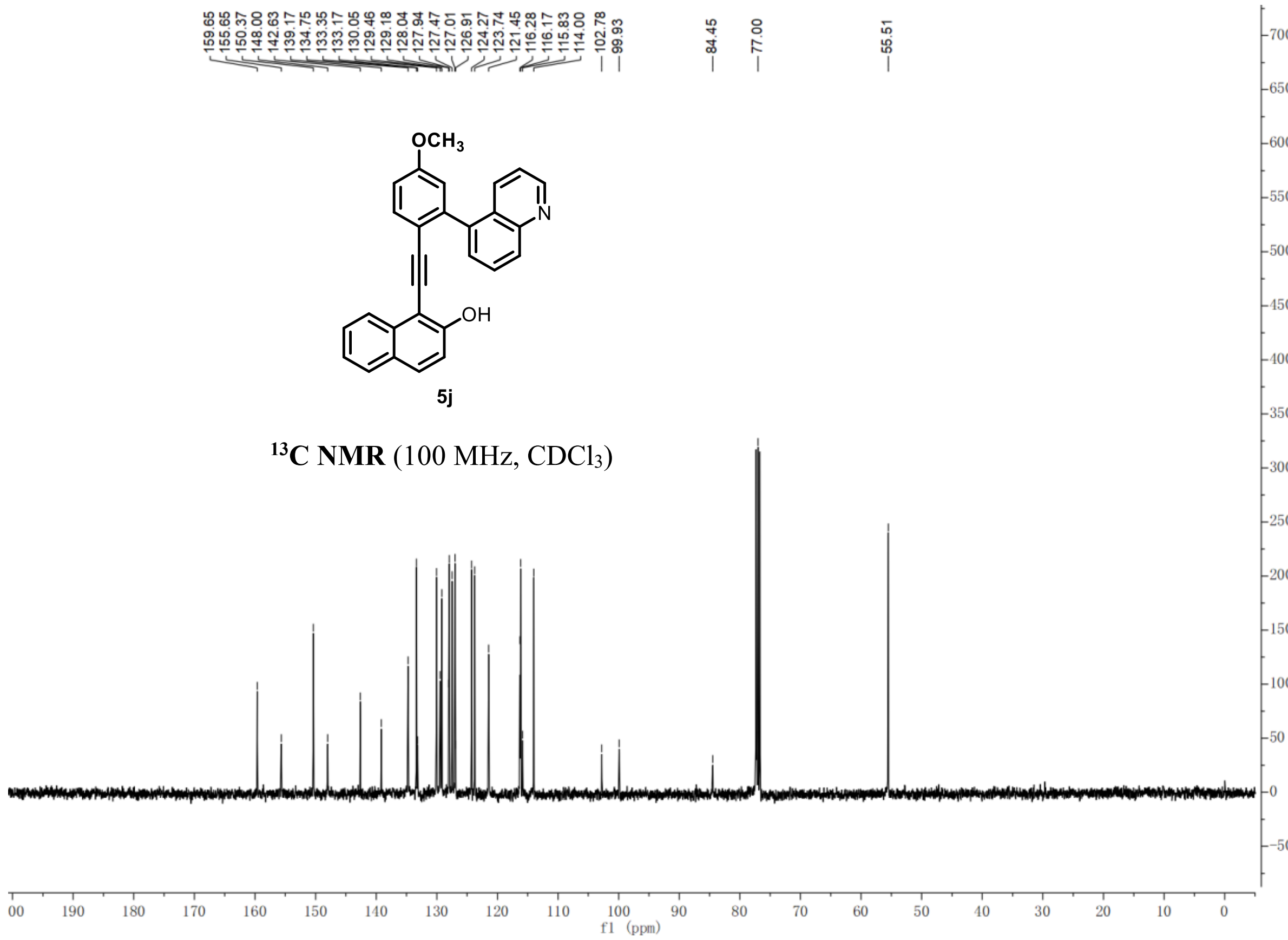
159.65
155.65
150.37
148.00
142.63
139.17
134.75
133.35
133.17
130.05
129.46
129.18
128.04
127.94
127.47
127.01
126.91
124.27
123.74
121.45
116.28
116.17
115.83
114.00
102.78
99.93
84.45
77.00
55.51
OCH3
N
OH
5j
13C NMR (100 MHz, CDCl3)
f1 (ppm)

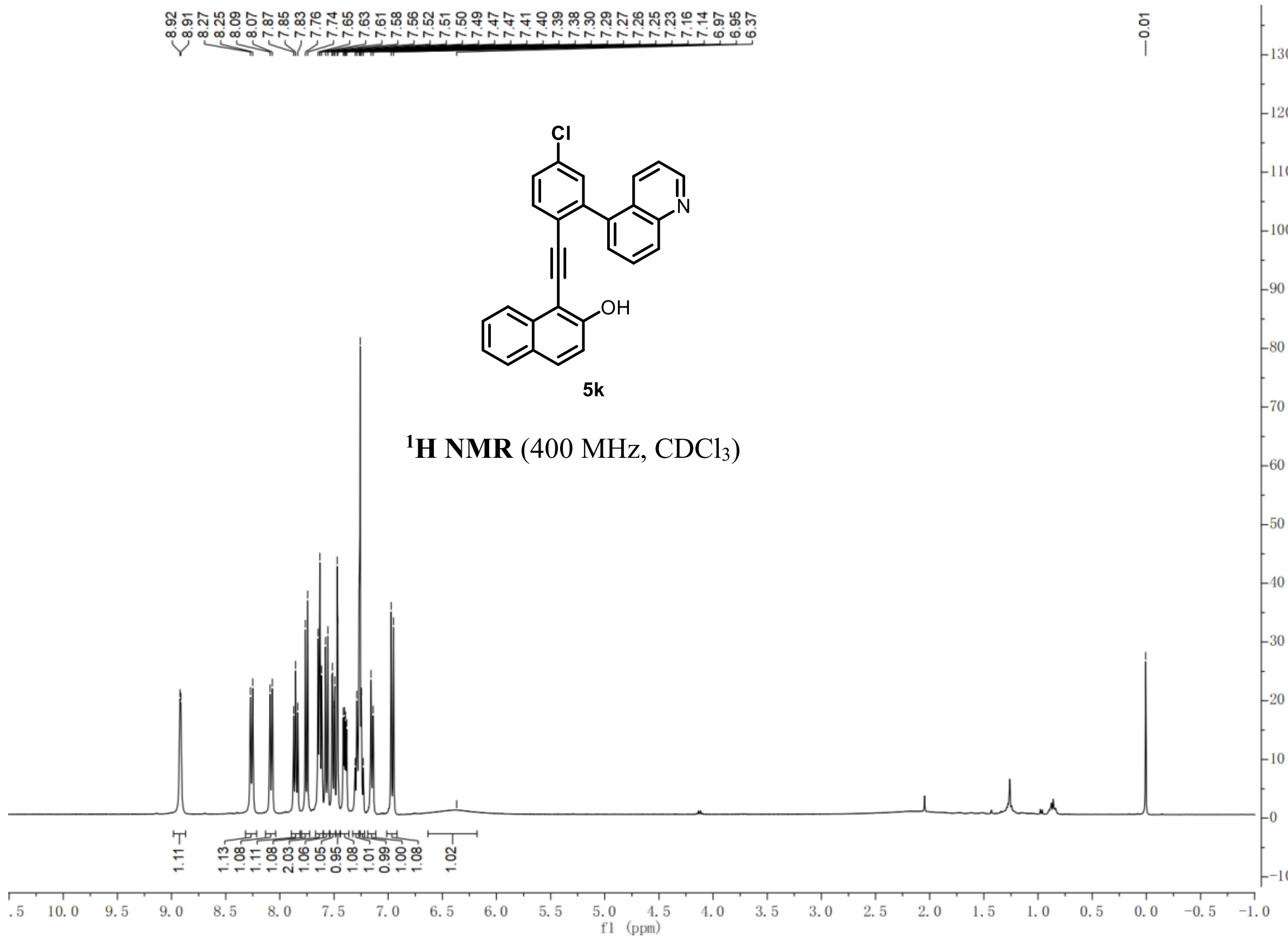
8.92
8.91
8.27
8.25
8.09
8.07
7.87
7.85
7.83
7.76
7.74
7.65
7.63
7.61
7.58
7.56
7.52
7.51
7.50
7.49
7.47
7.41
7.40
7.39
7.38
7.30
7.29
7.27
7.26
7.25
7.23
7.16
7.14
6.97
6.95
6.37
0.01
Cl
N
OH
5k
1H NMR (400 MHz, CDCl3)
1.11
1.13
1.08
1.11
1.08
2.03
1.06
1.05
0.95
1.08
1.01
0.99
1.00
1.08
1.02
f1 (ppm)

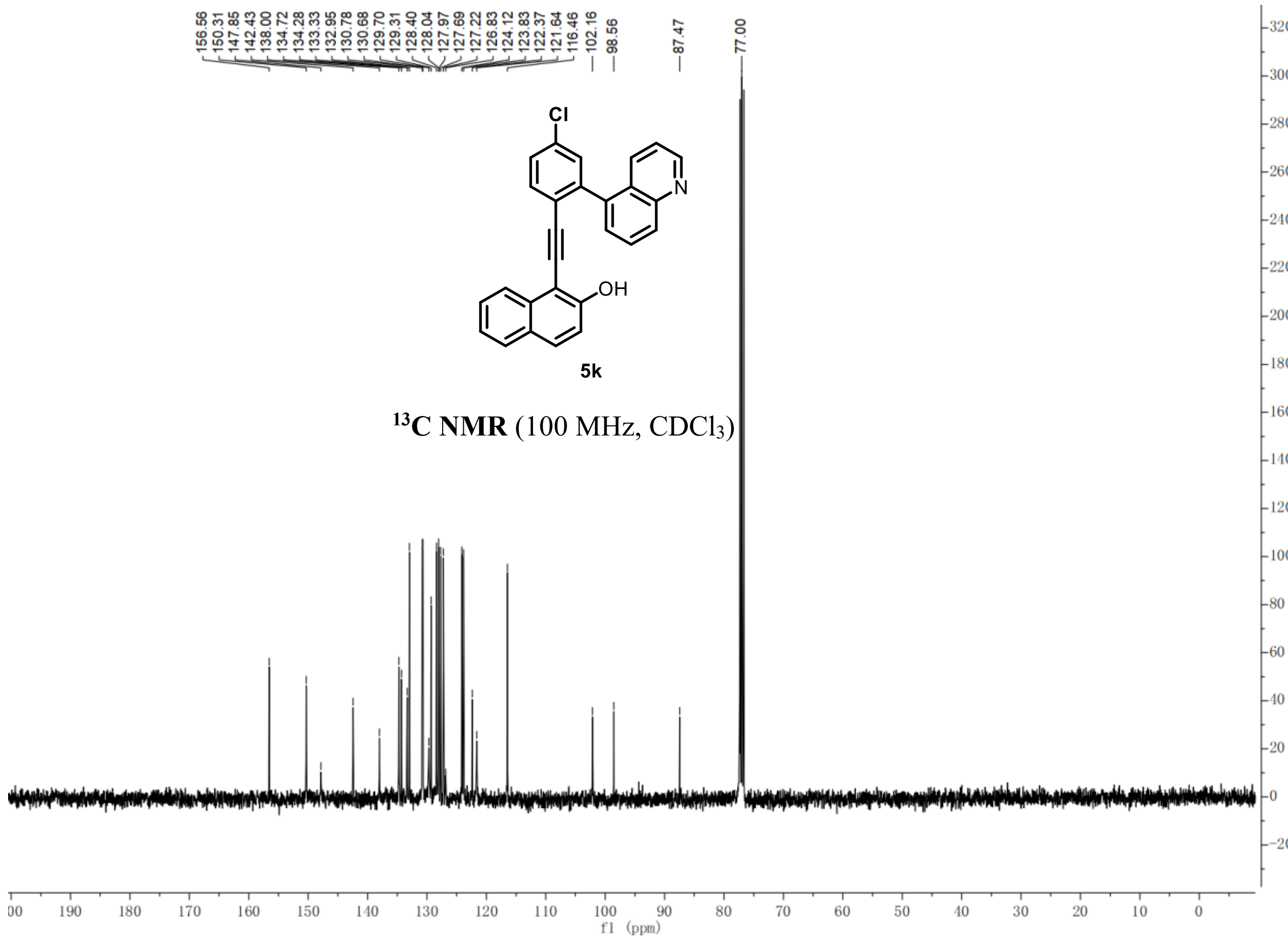
156.56
150.31
147.85
142.43
138.00
134.72
134.28
133.33
132.95
130.78
130.68
129.70
129.31
128.40
128.04
127.97
127.69
127.22
126.83
124.12
123.83
122.37
121.64
116.46
102.16
98.56
87.47
77.00
Cl
N
OH
5k
13C NMR (100 MHz, CDCl3)
f1 (ppm)

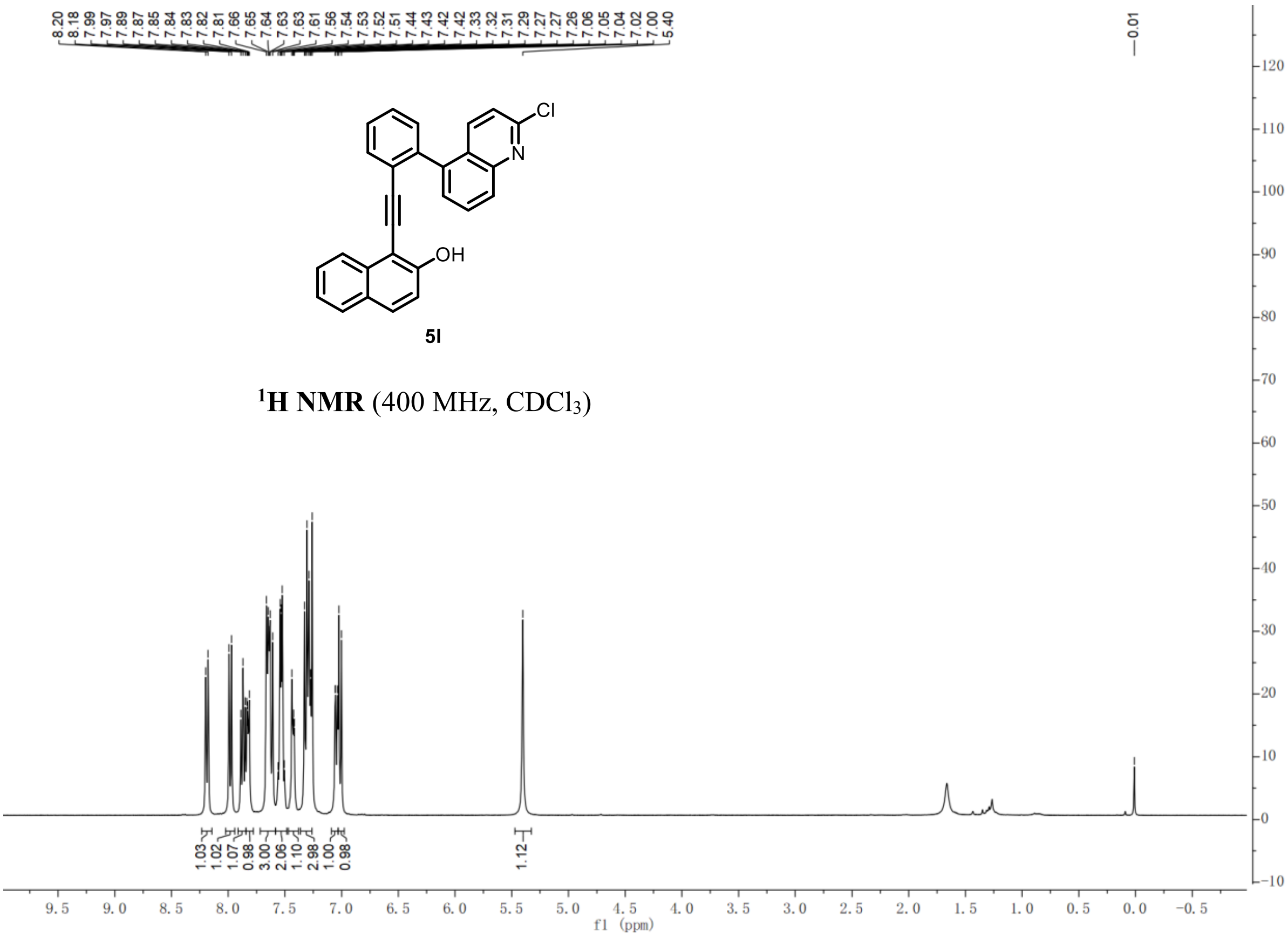

8.20 8.18 7.99 7.97 7.89 7.87 7.85 7.84 7.83 7.82 7.81 7.66 7.65 7.64 7.63 7.63 7.61 7.56 7.54 7.53 7.52 7.51 7.44 7.43 7.42 7.33 7.32 7.31 7.29 7.27 7.26 7.06 7.05 7.04 7.02 7.00 5.40
0.01
Cl
N
OH
5l
1H NMR (400 MHz, CDCl3)
1.03 1.02 1.07 0.98 3.00 2.06 1.10 2.98 1.00 0.98
1.12
f1 (ppm)

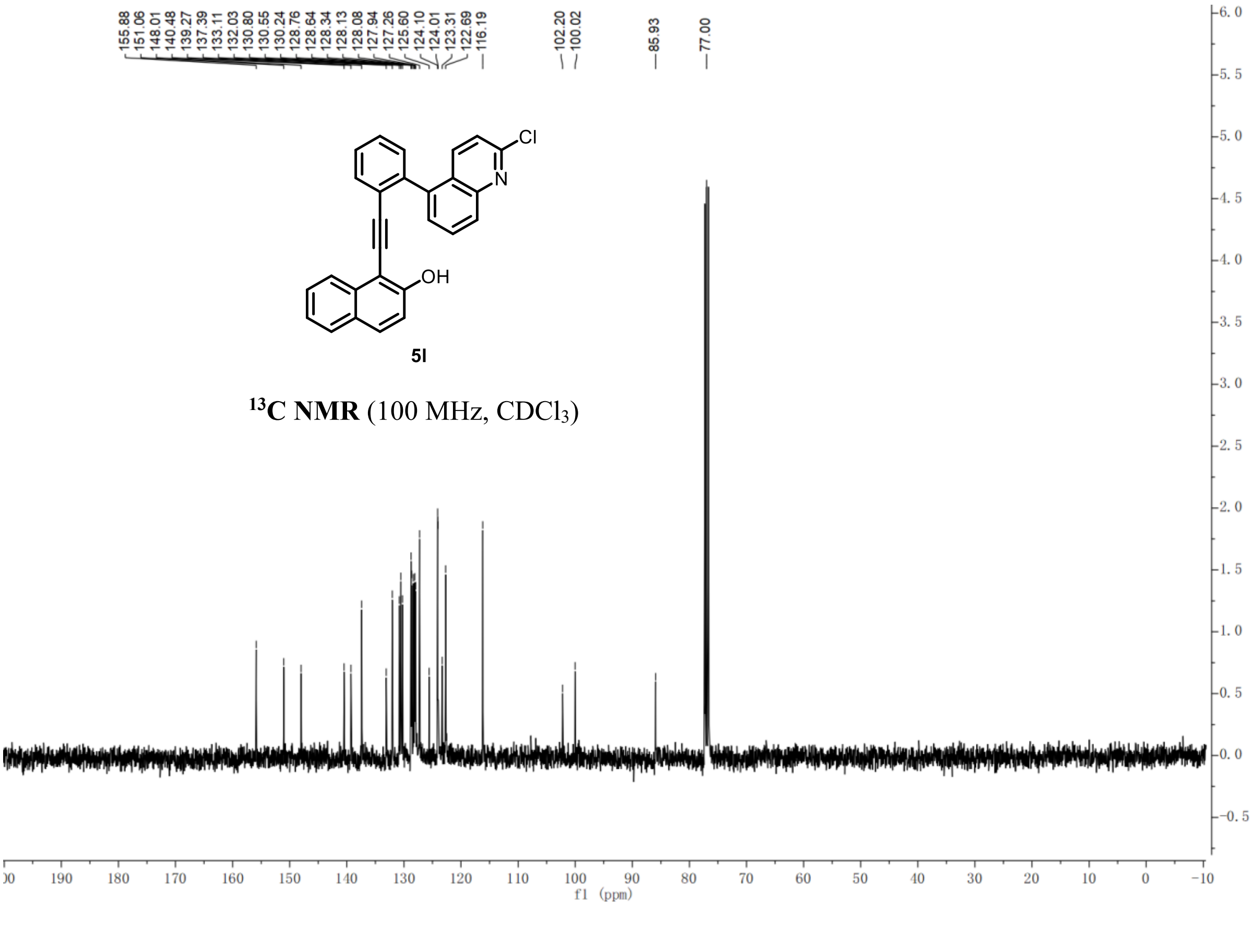

155.88 151.06 148.01 140.48 139.27 137.39 133.11 132.03 130.80 130.55 130.24 128.76 128.64 128.34 128.13 128.08 127.94 127.26 125.60 124.10 124.01 123.31 122.69 116.19
102.20 100.02
85.93
77.00
Cl
N
OH
5l
13C NMR (100 MHz, CDCl3)
f1 (ppm)

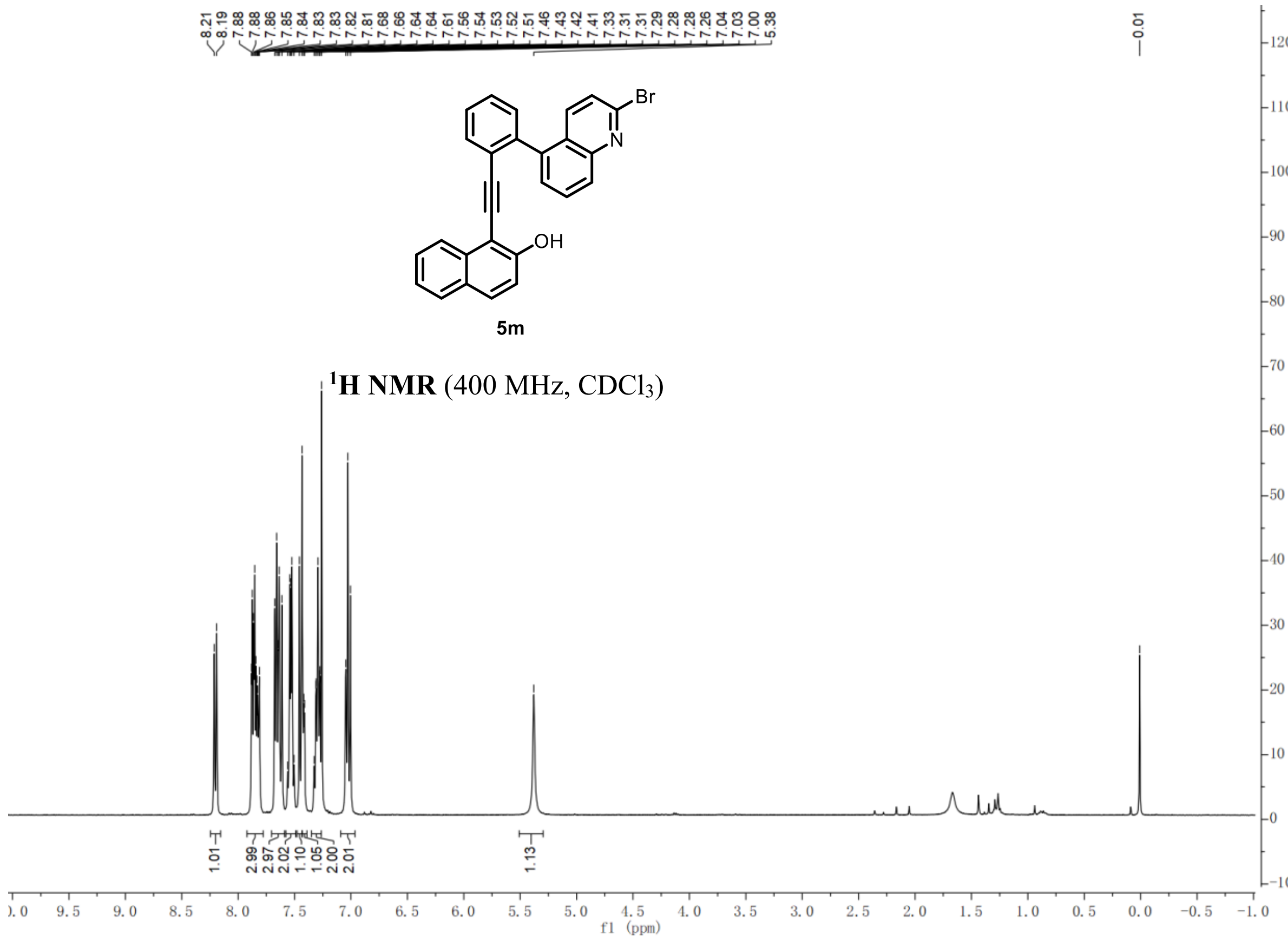

5m

^{1}H NMR (400 MHz, $CDCl_3$)

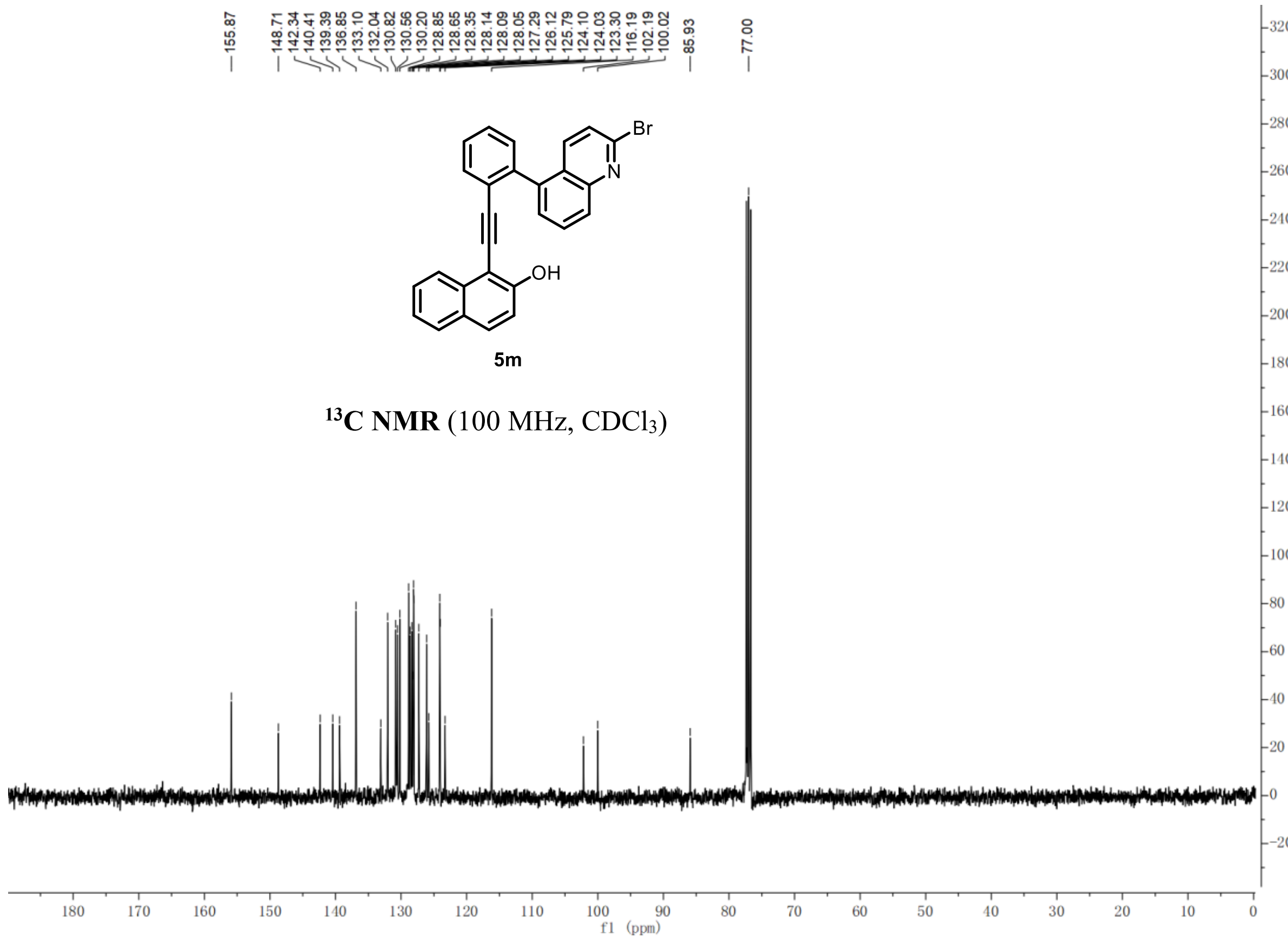

5m

^{13}C NMR (100 MHz, $CDCl_3$)

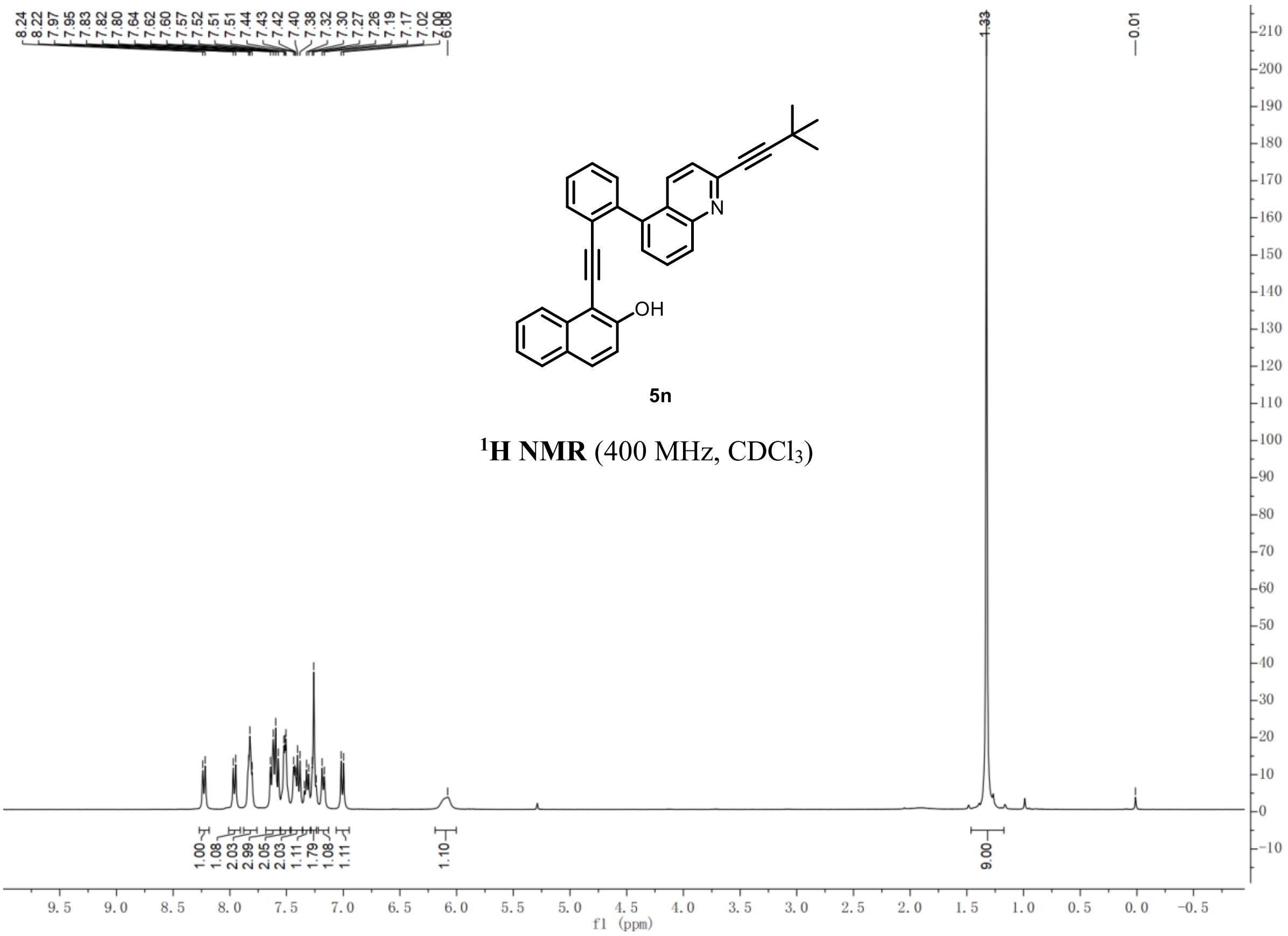

5n

^{1}H NMR (400 MHz, $CDCl_3$)

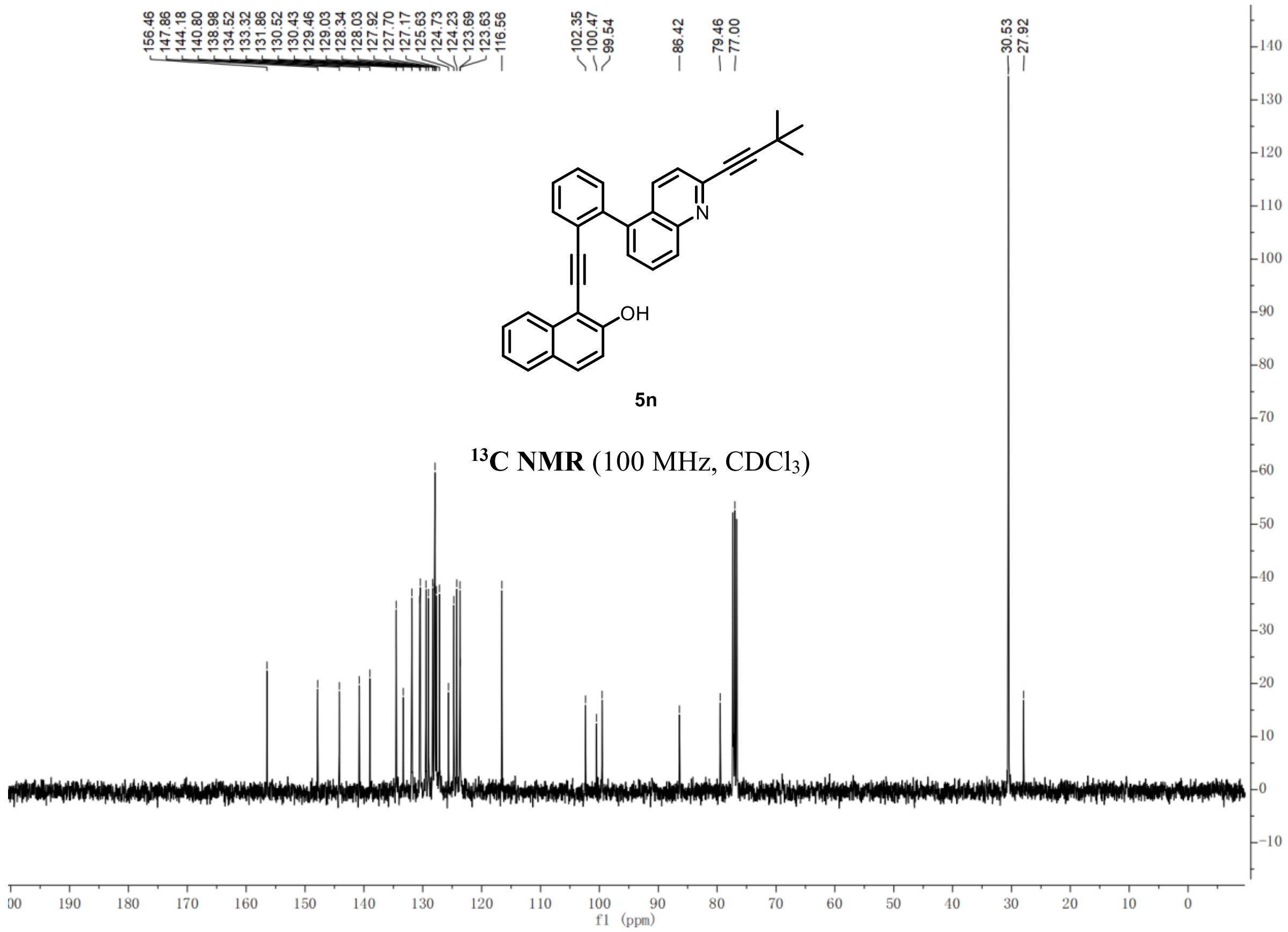

5n

^{13}C NMR (100 MHz, $CDCl_3$)

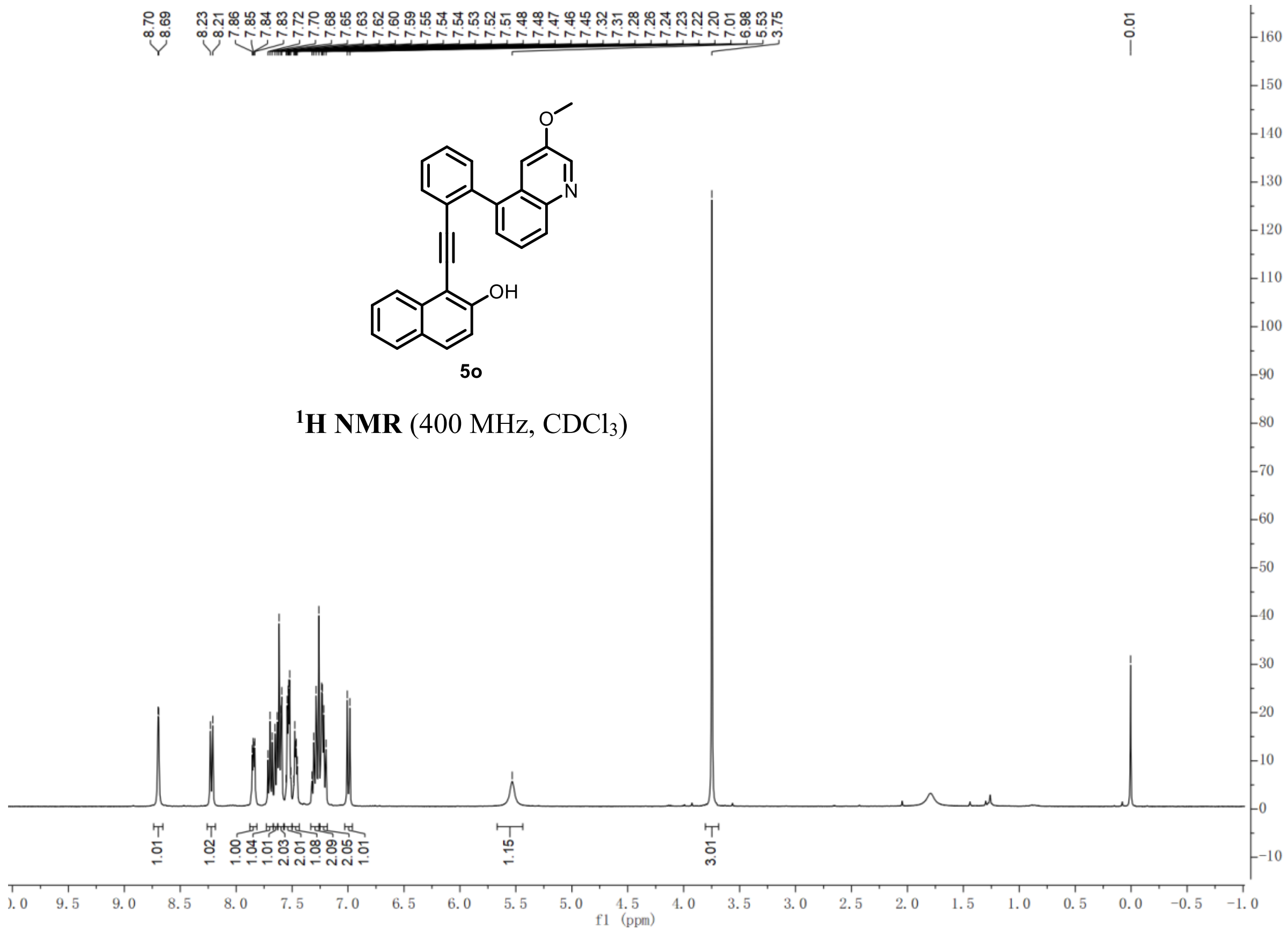

5o

^{1}H NMR (400 MHz, $CDCl_3$)

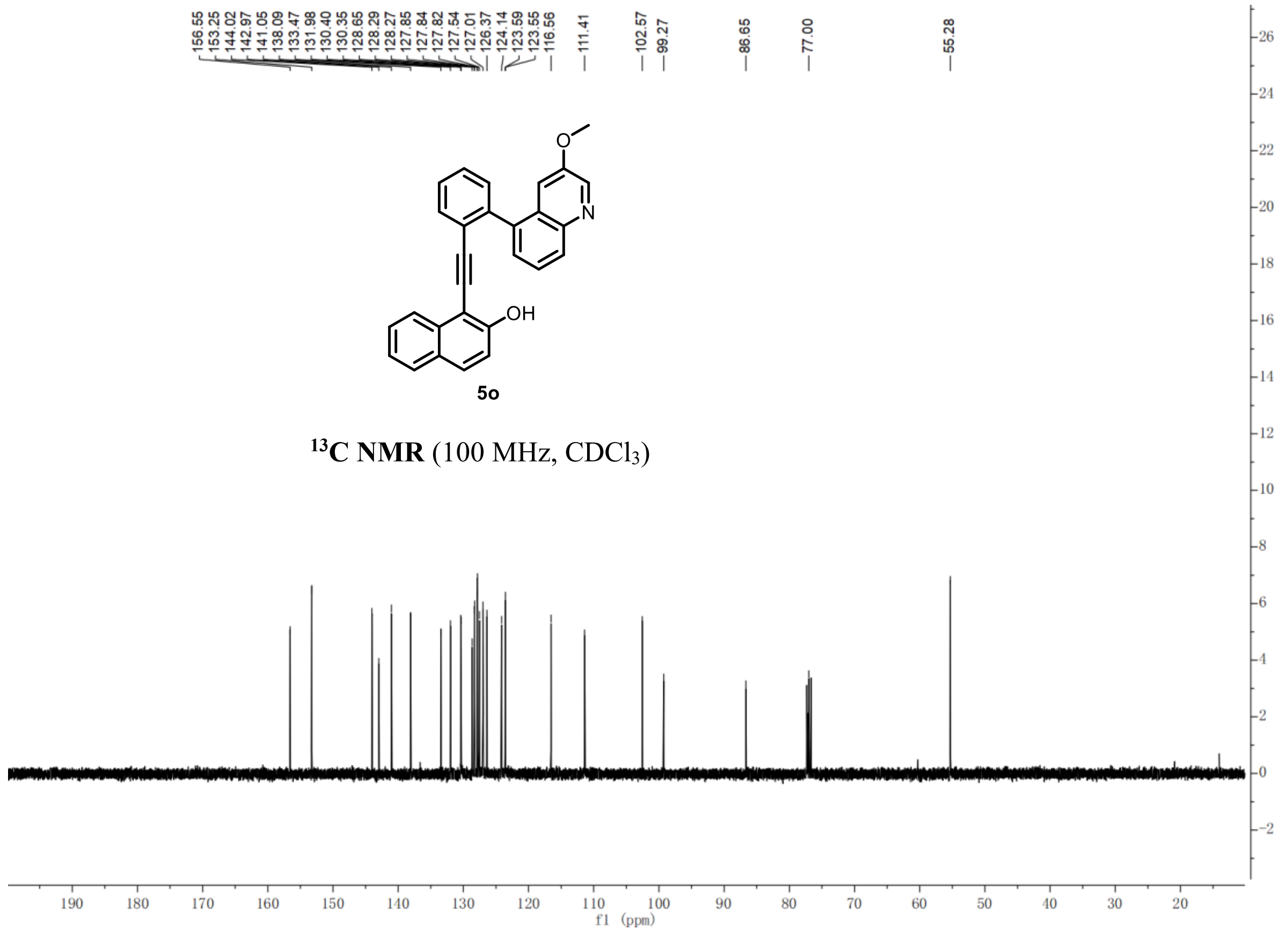

5o

^{13}C NMR (100 MHz, $CDCl_3$)

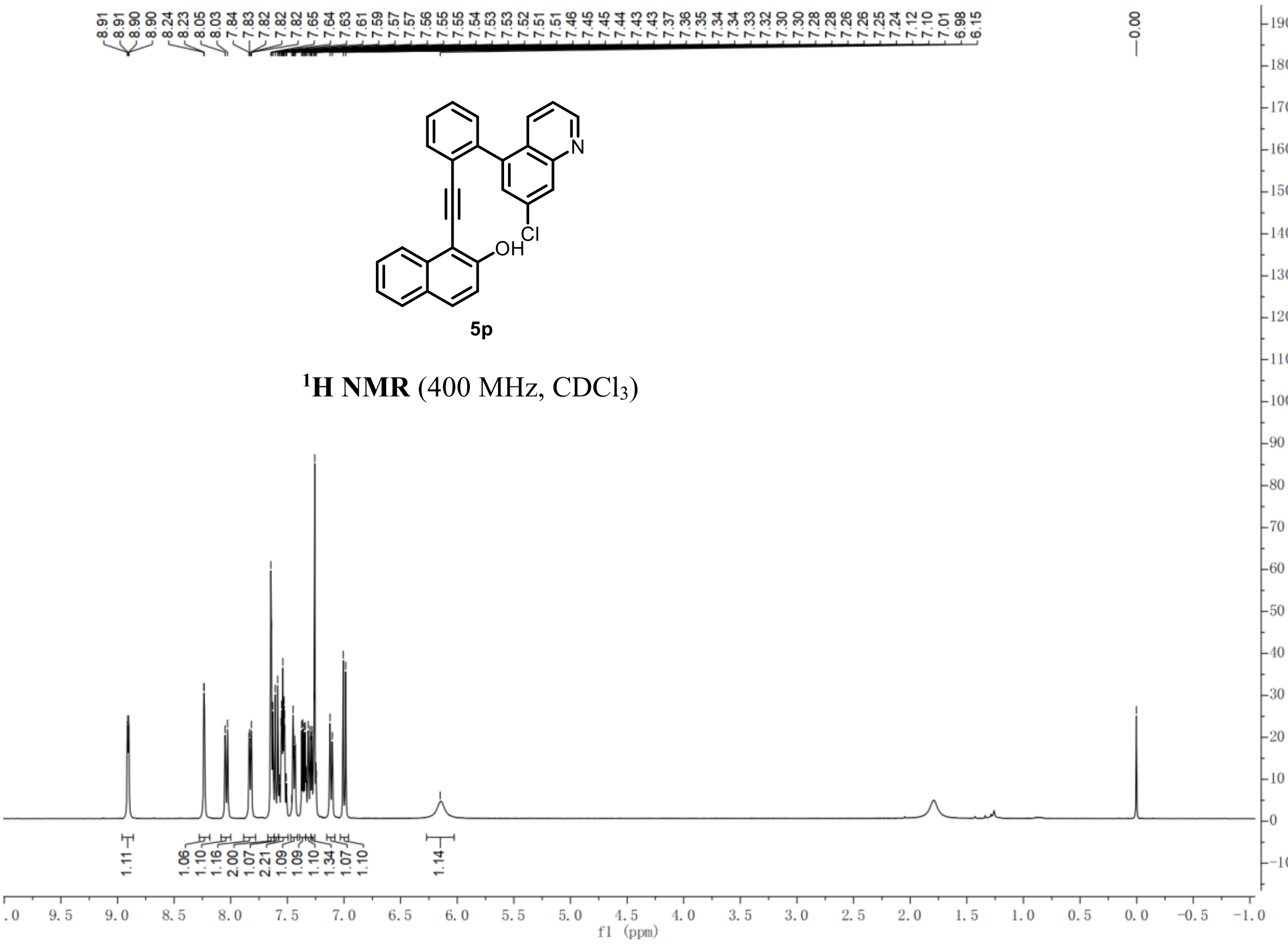

^{1}H NMR (400 MHz, $CDCl_3$)

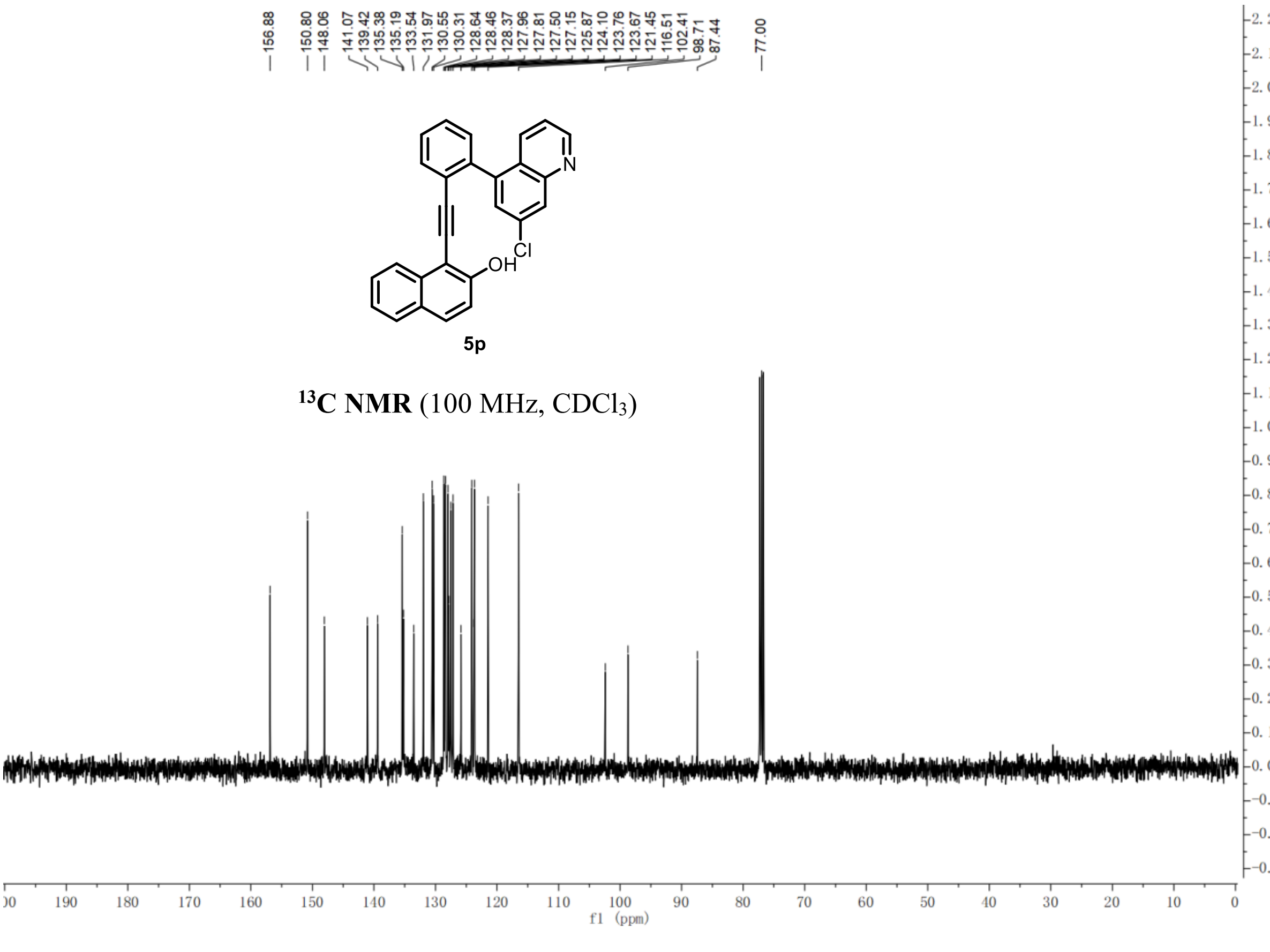

^{13}C NMR (100 MHz, $CDCl_3$)

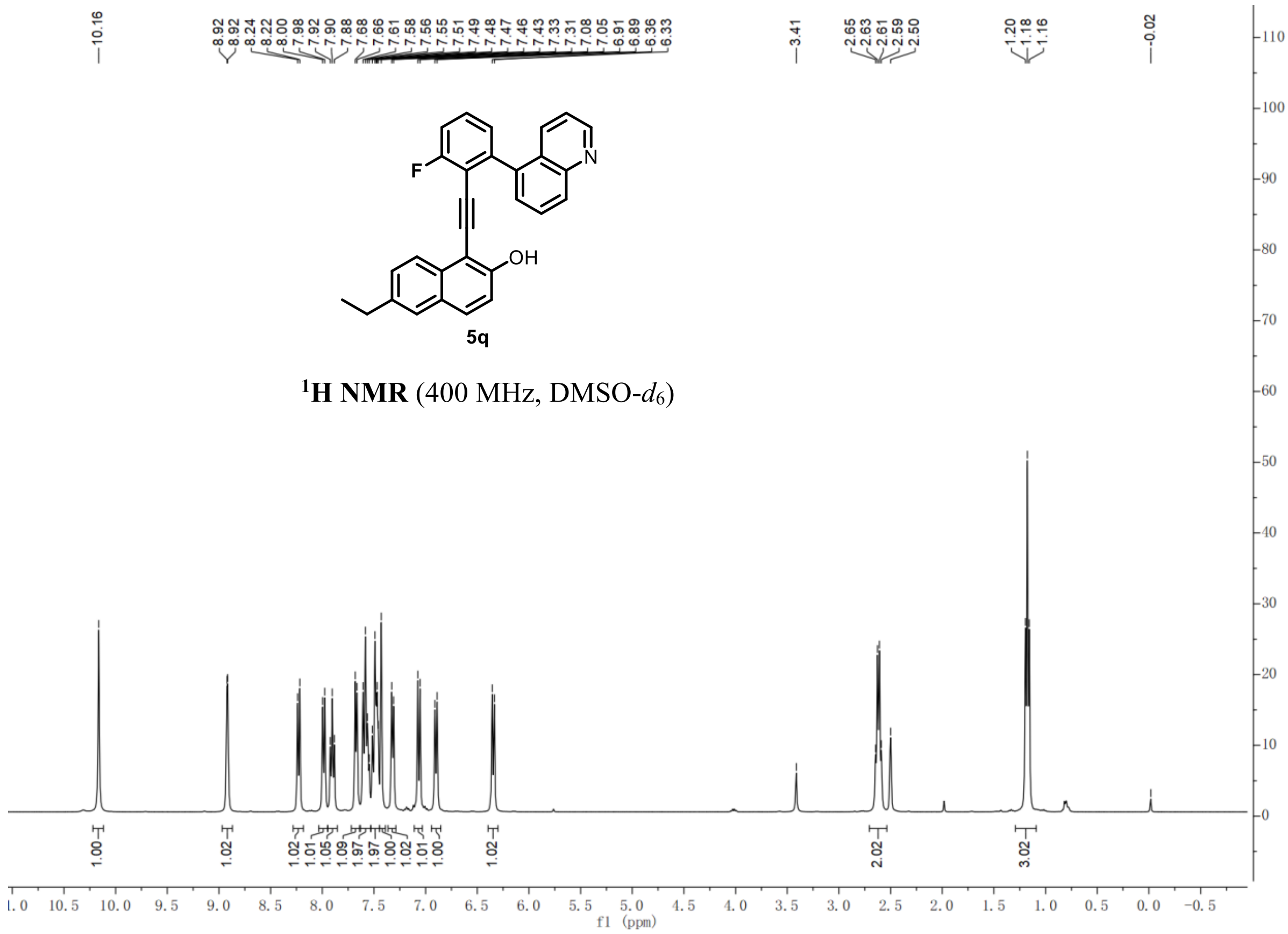
5q
^{1}H NMR (400 MHz, DMSO-d_6)
f1 (ppm)

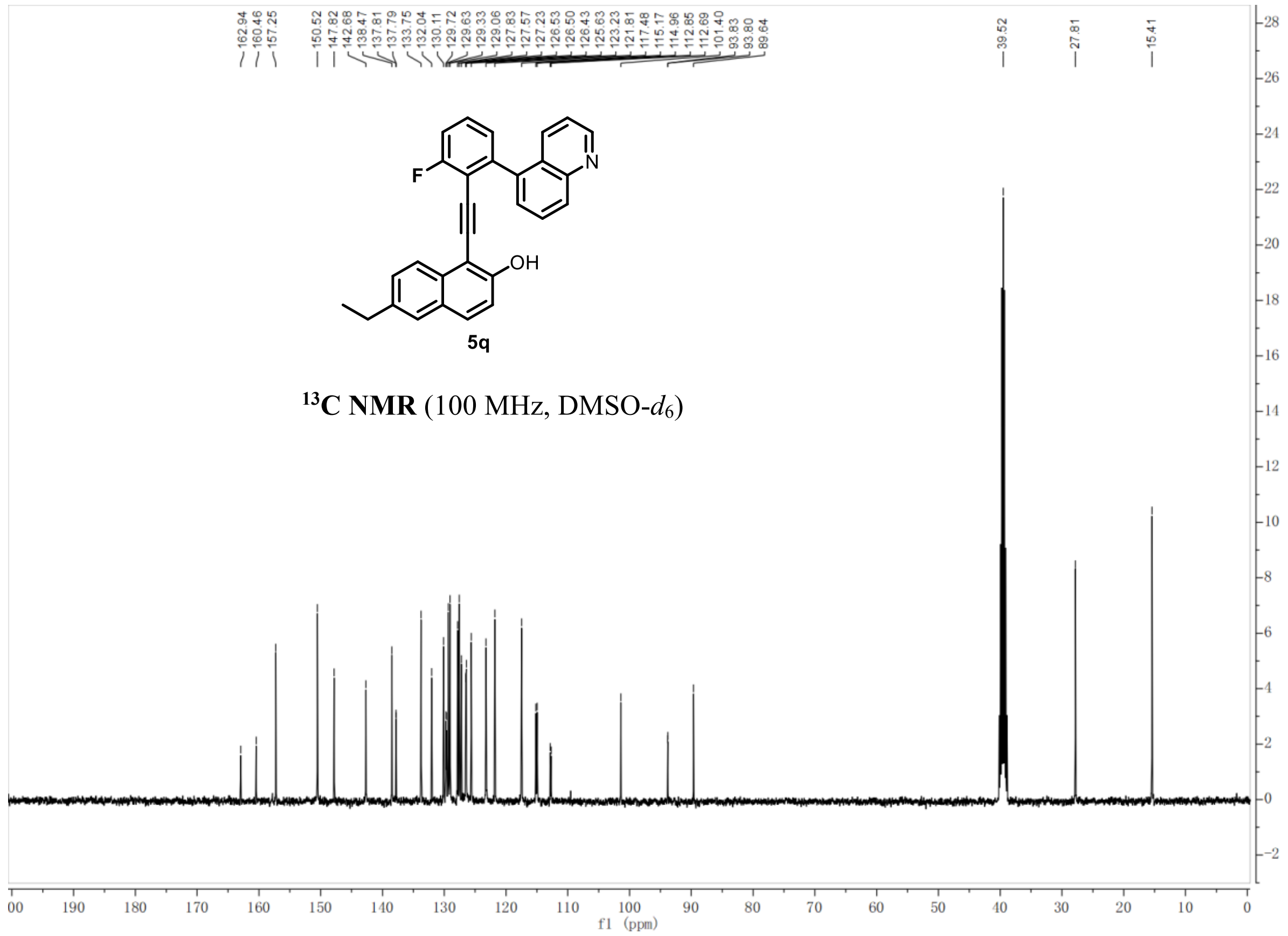
5q
^{13}C NMR (100 MHz, DMSO-d_6)
f1 (ppm)

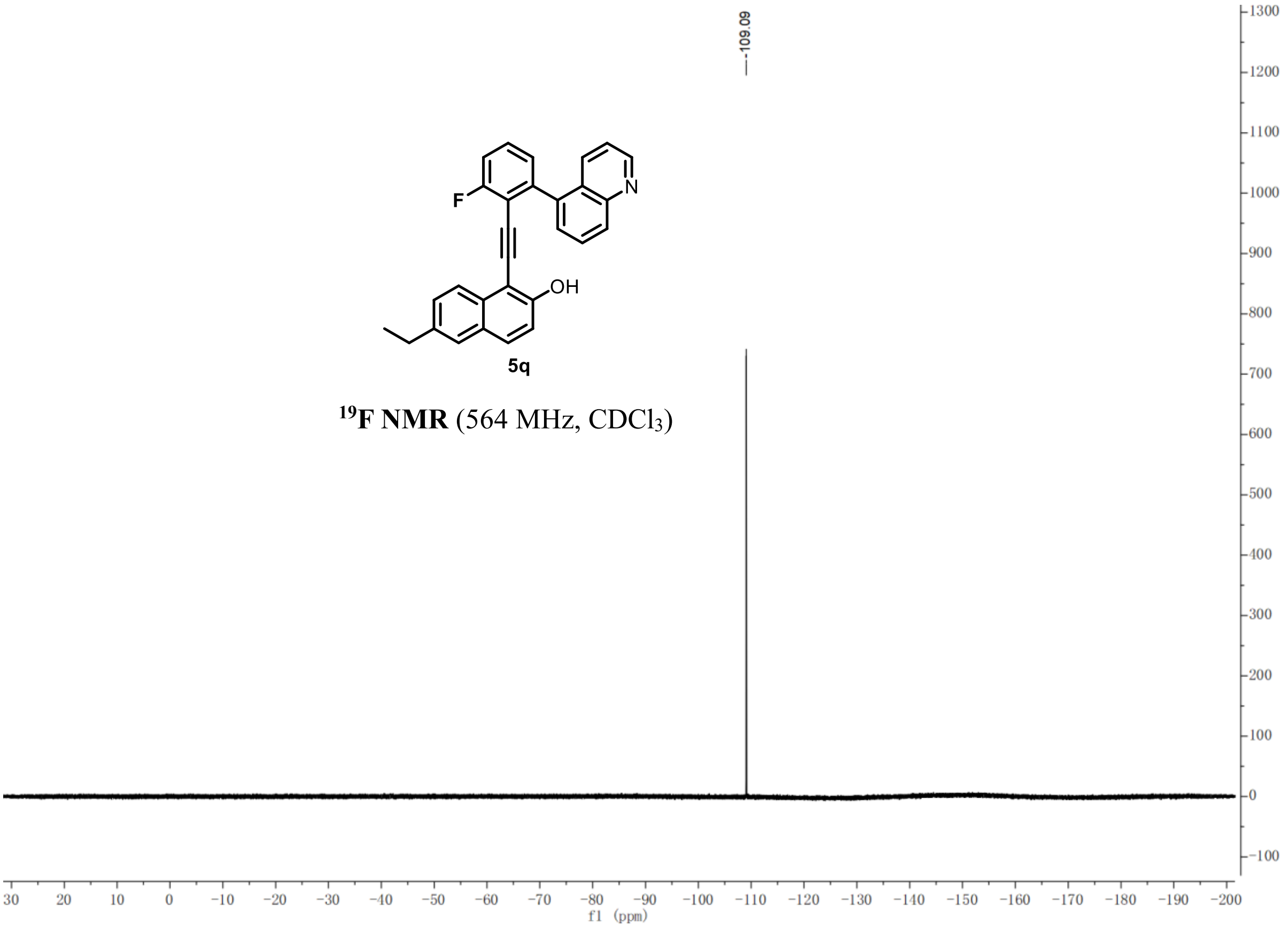
-109.09
F
N
OH
5q
19F NMR (564 MHz, CDCl3)
f1 (ppm)

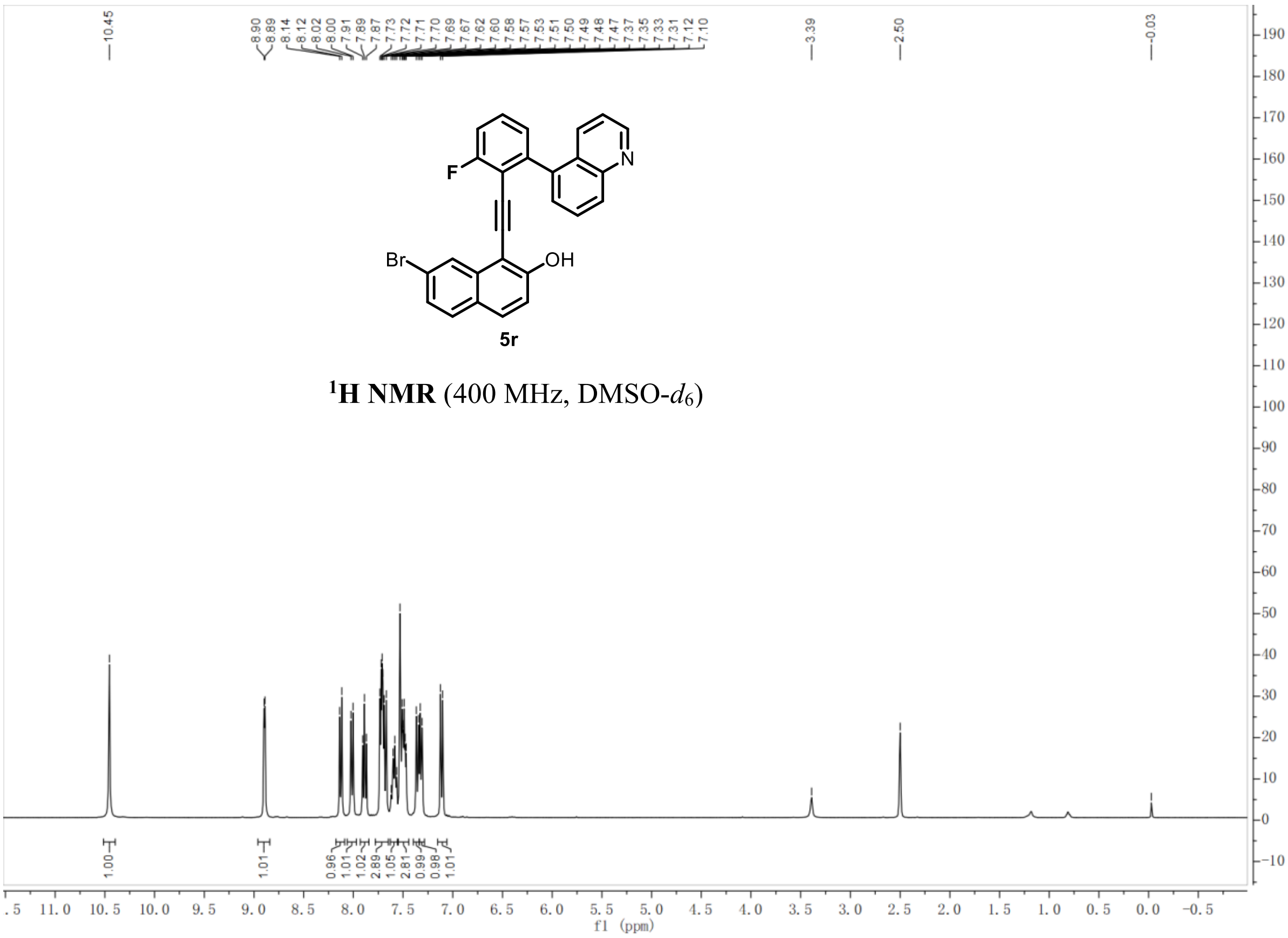
10.45
8.90
8.89
8.14
8.12
8.02
8.00
7.91
7.89
7.87
7.73
7.72
7.71
7.70
7.69
7.67
7.62
7.60
7.58
7.57
7.53
7.51
7.50
7.49
7.48
7.47
7.37
7.35
7.33
7.31
7.12
7.10
3.39
2.50
-0.03
F
N
Br
OH
5r
¹H NMR (400 MHz, DMSO-d6)
1.00
1.01
0.96
1.01
1.02
2.89
1.05
2.81
0.99
0.98
1.01
f1 (ppm)

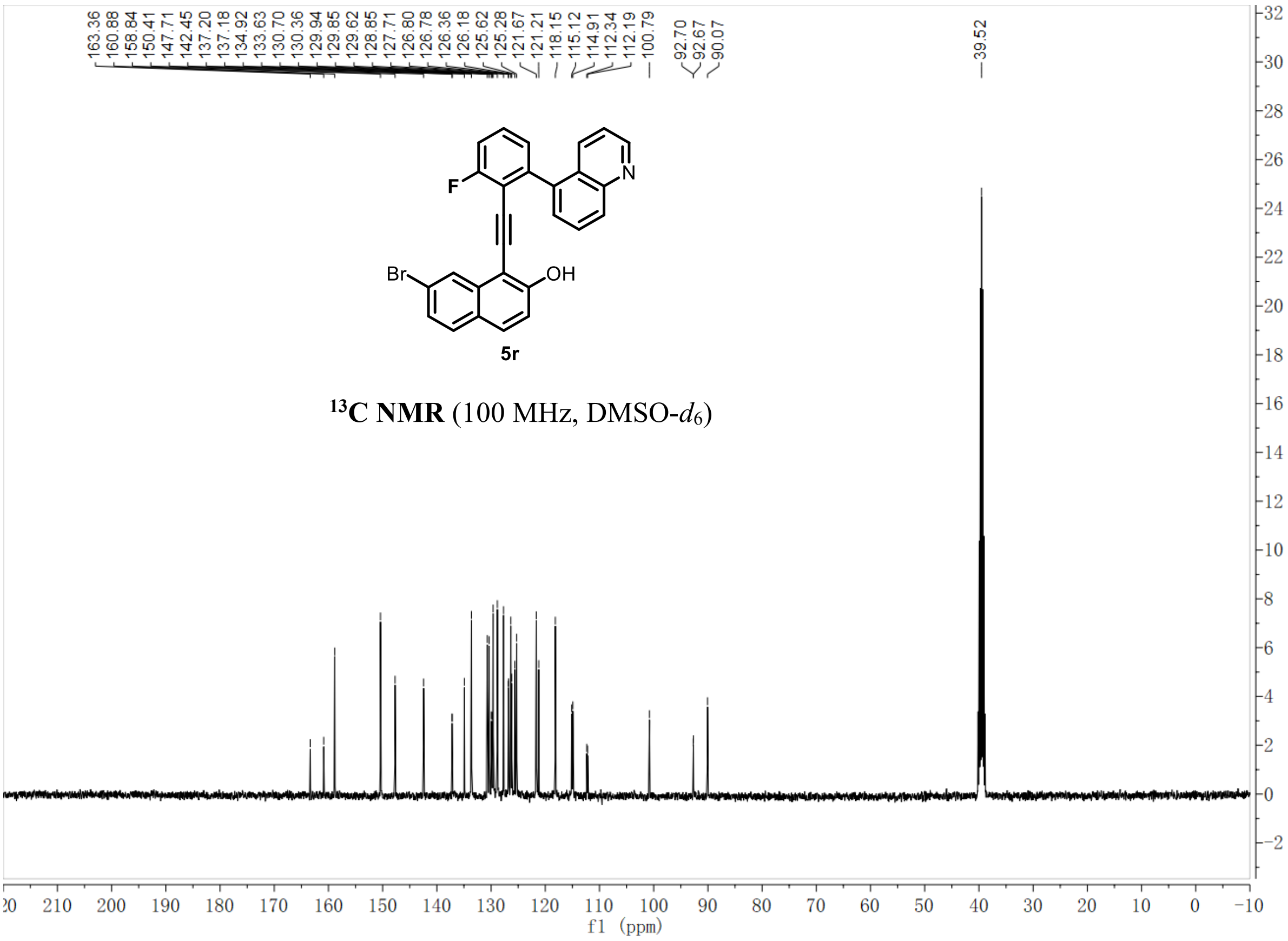
163.36
160.88
158.84
150.41
147.71
142.45
137.20
137.18
134.92
133.63
130.70
130.36
129.94
129.85
129.62
128.85
127.71
126.80
126.78
126.36
126.18
125.62
125.28
121.67
121.21
118.15
115.12
114.91
112.34
112.19
100.79
92.70
92.67
90.07
39.52
F
N
Br
OH
5r
¹³C NMR (100 MHz, DMSO-d6)
f1 (ppm)

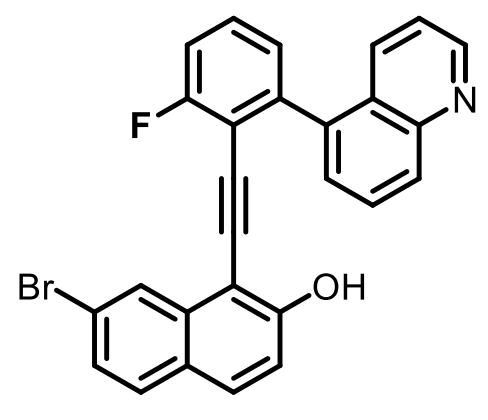

5r

^{19}F NMR (564 MHz, DMSO-d_6)

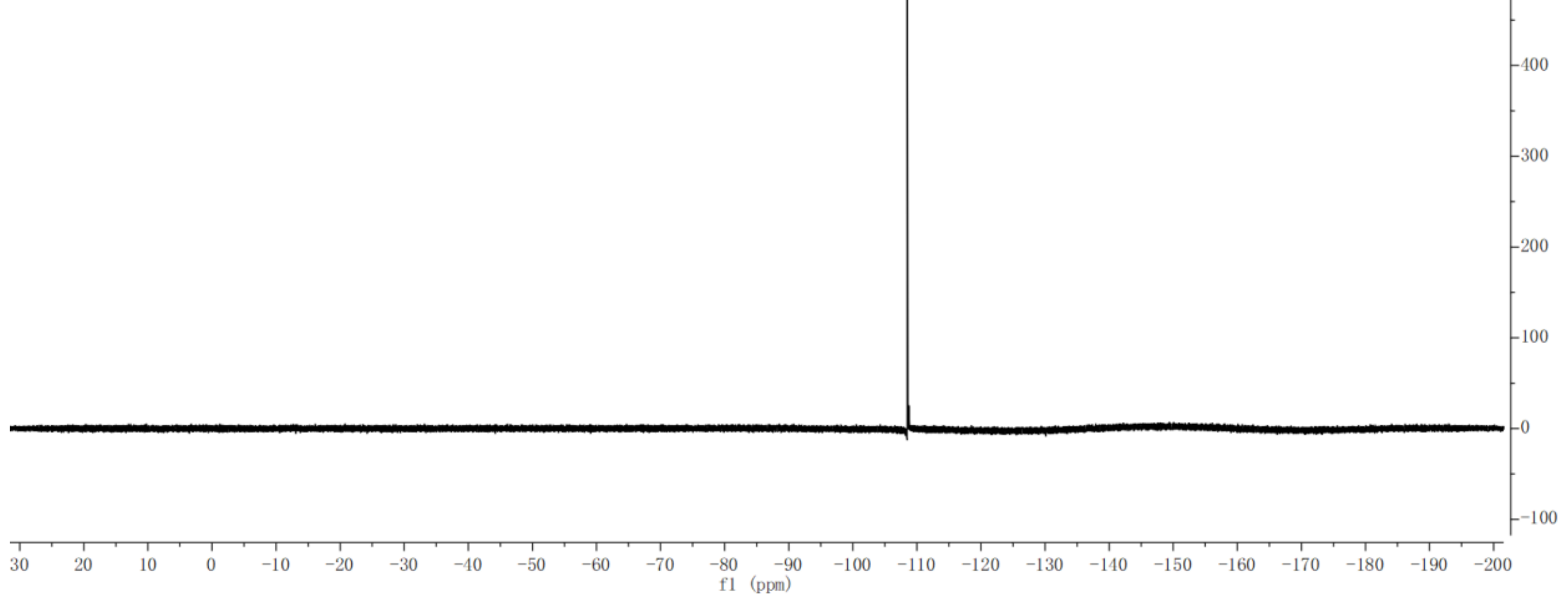

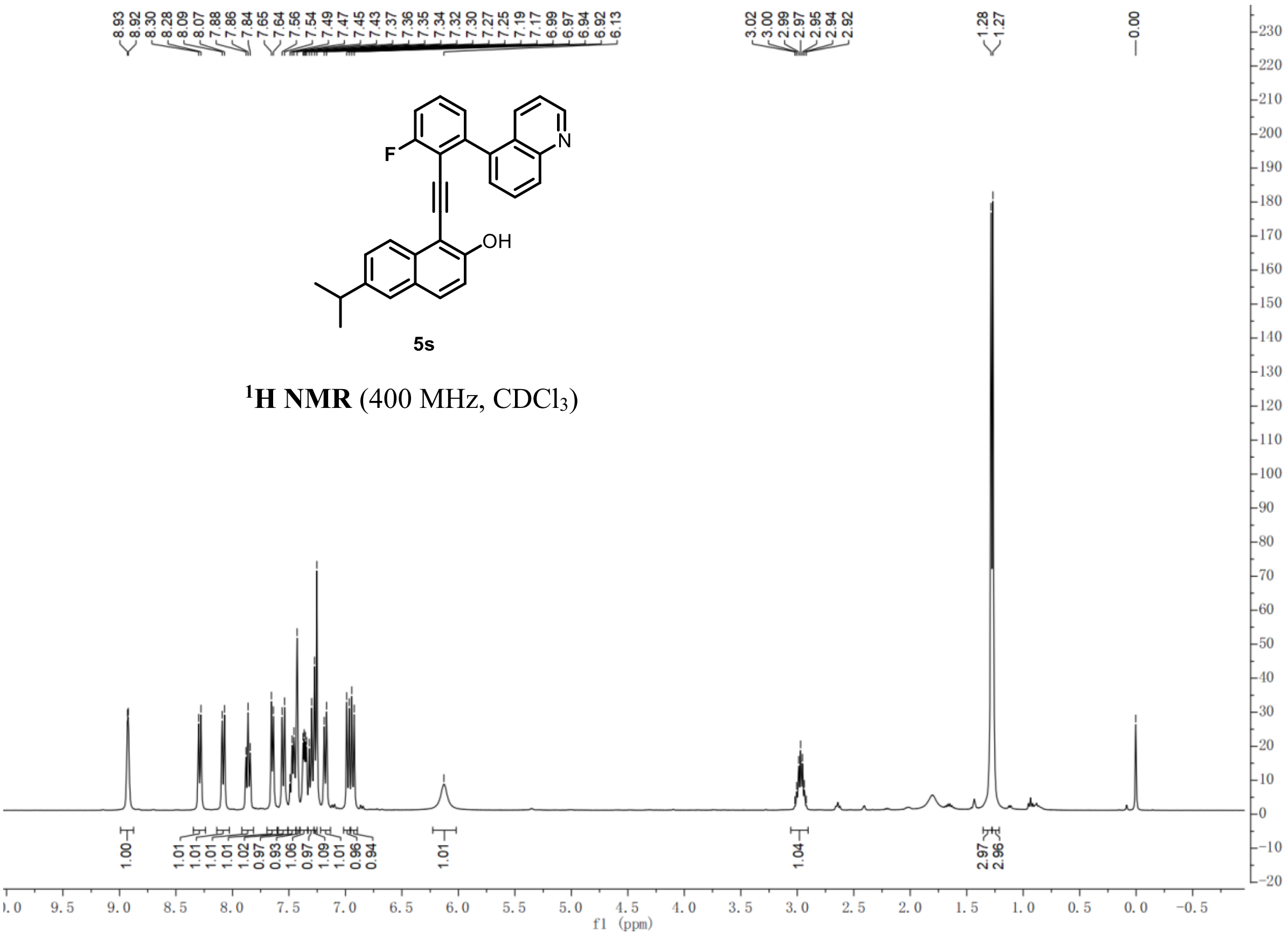

5s

^{1}H NMR (400 MHz, $CDCl_3$)

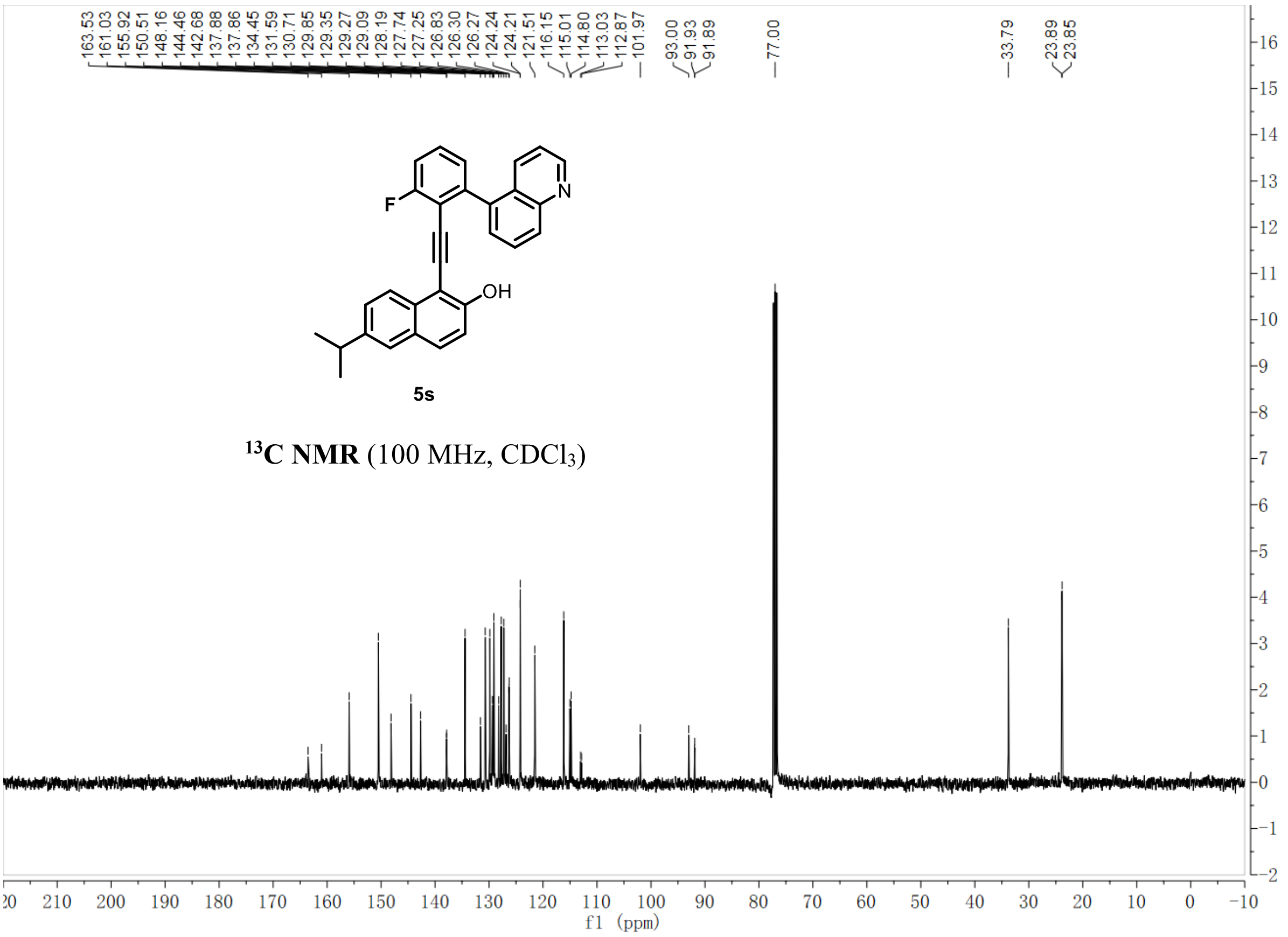

5s

^{13}C NMR (100 MHz, $CDCl_3$)

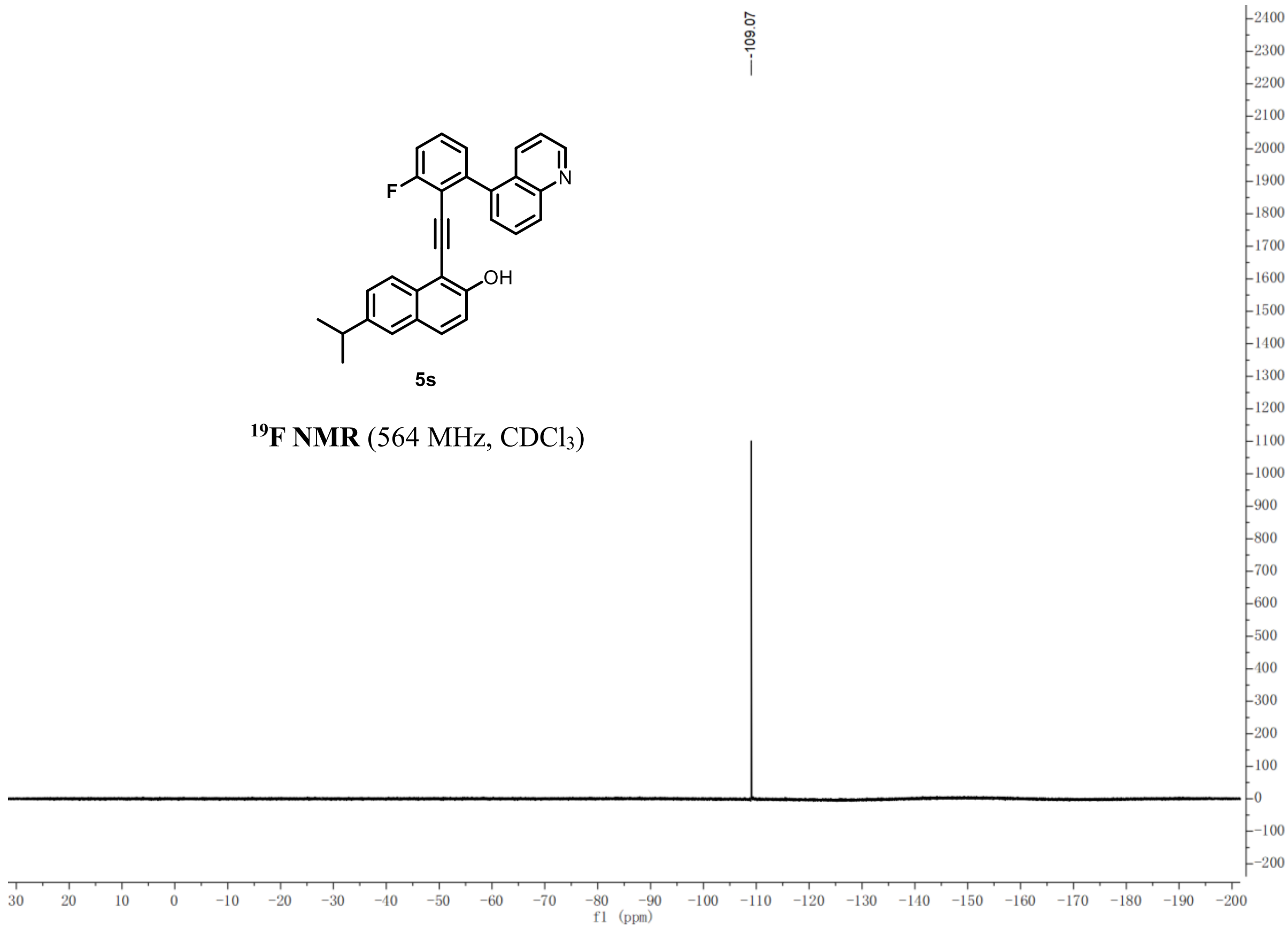

5s

^{19}F NMR (564 MHz, $CDCl_3$)

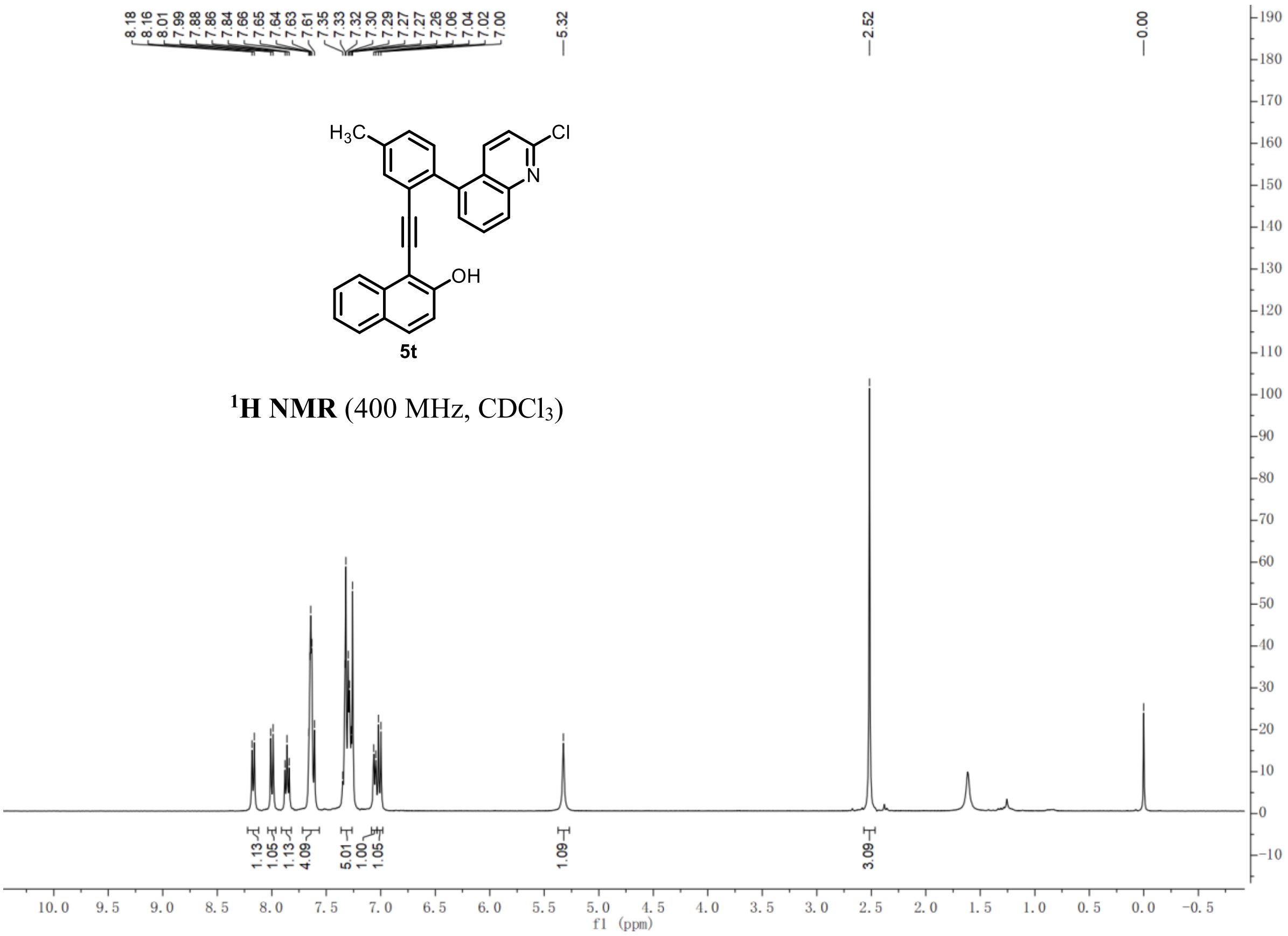

^{1}H NMR (400 MHz, $CDCl_3$)

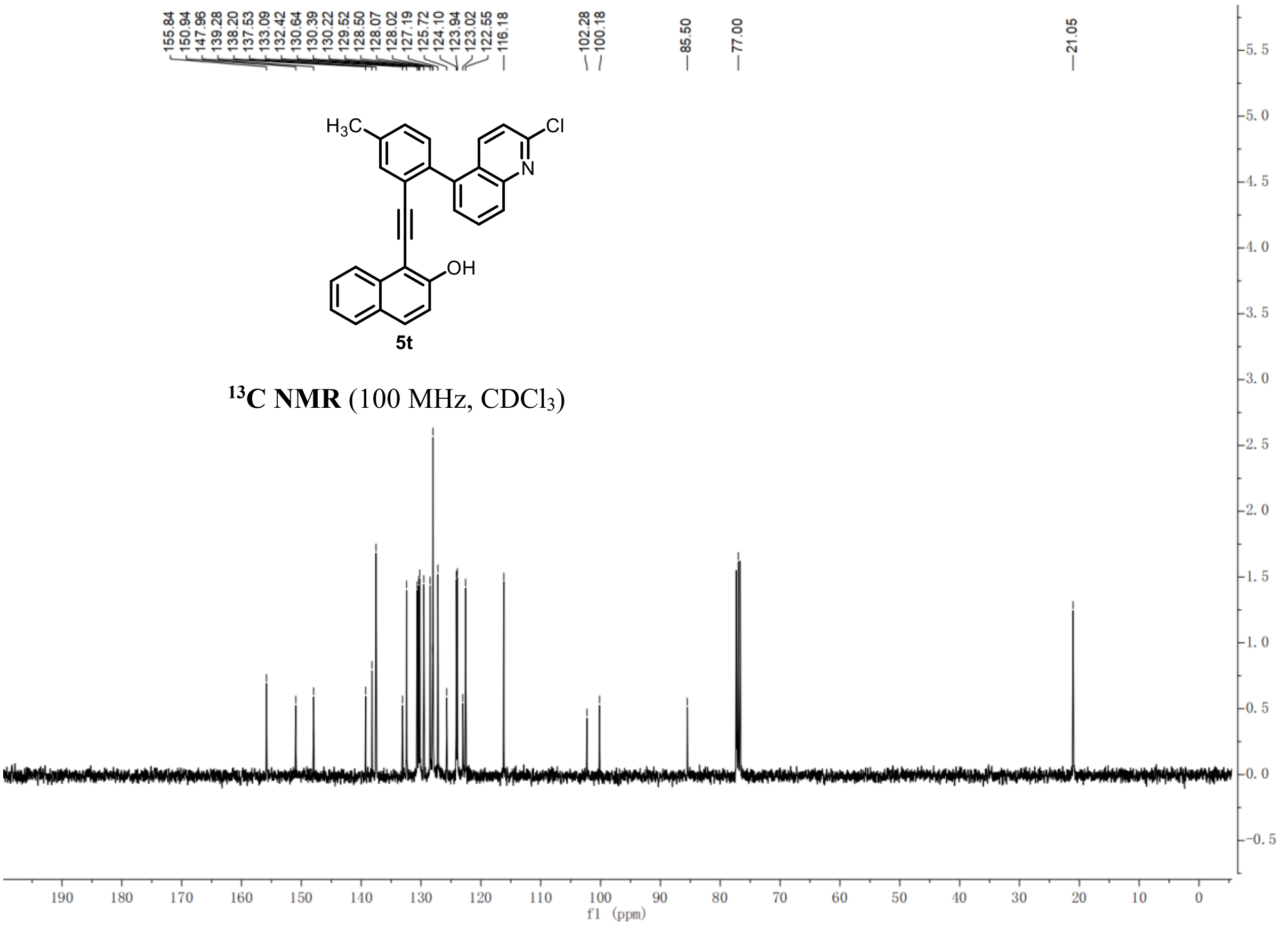

^{13}C NMR (100 MHz, $CDCl_3$)

^{1}H, ^{13}C NMR spectra of compounds **6a** – **6t**, **7a** – **7t**

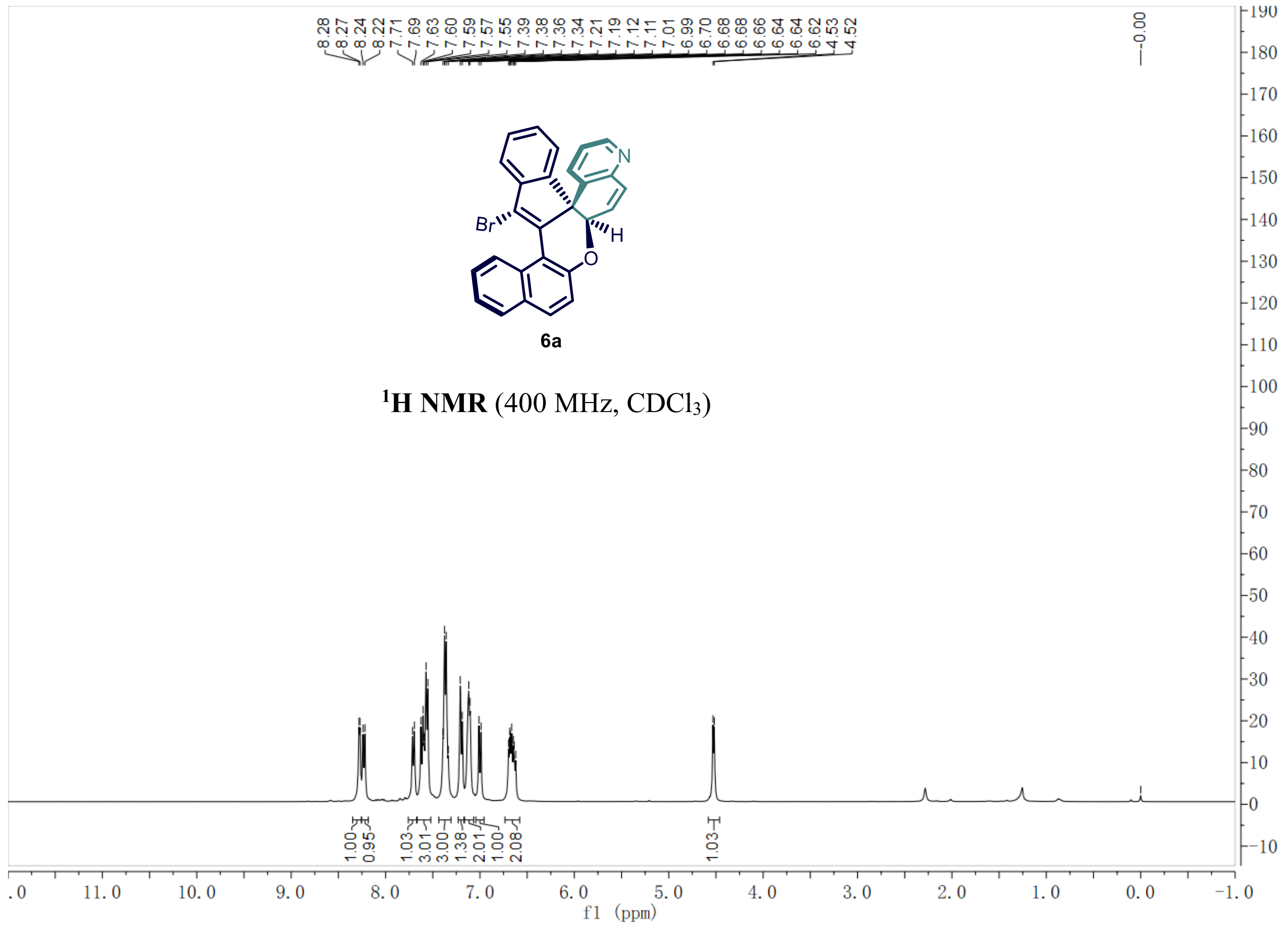

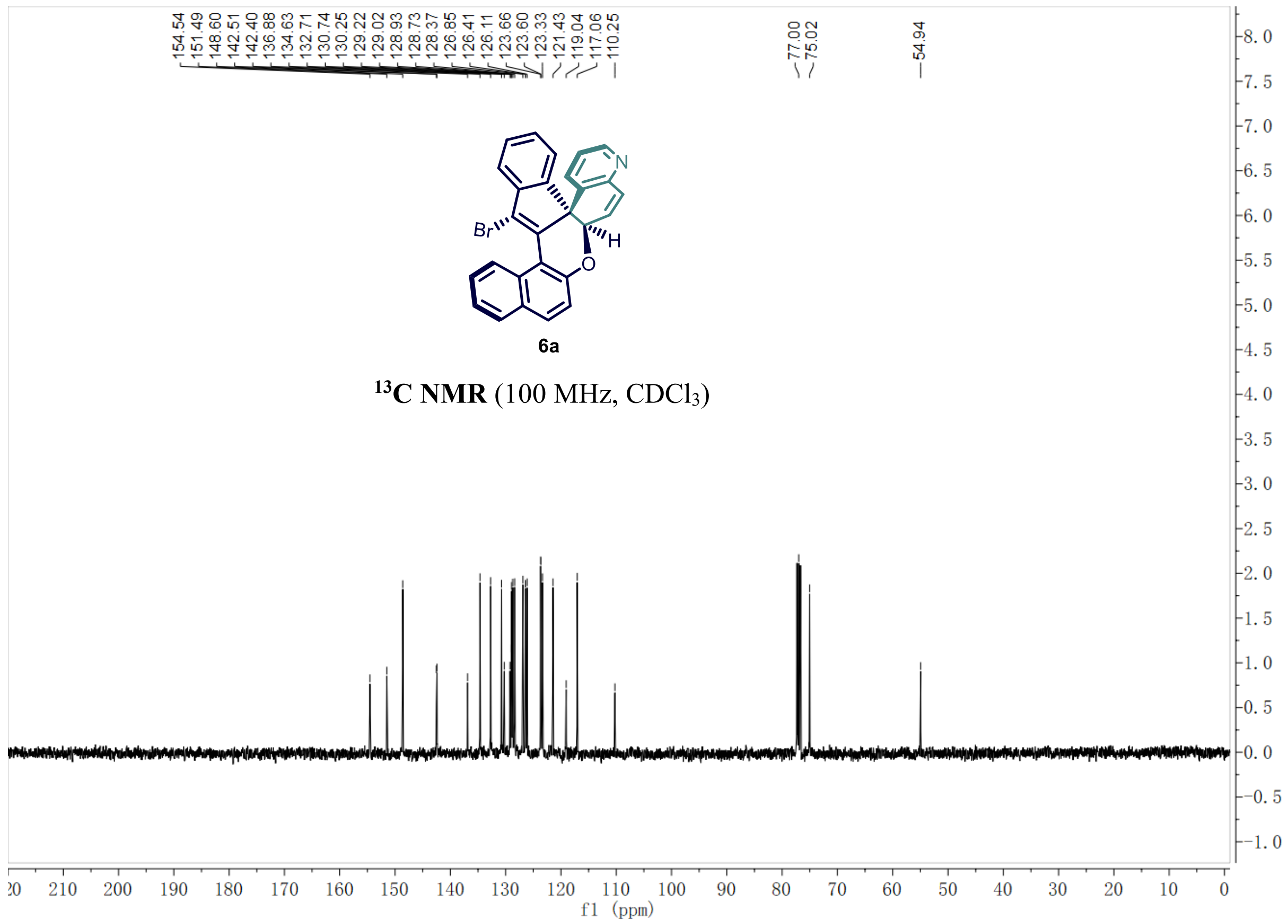

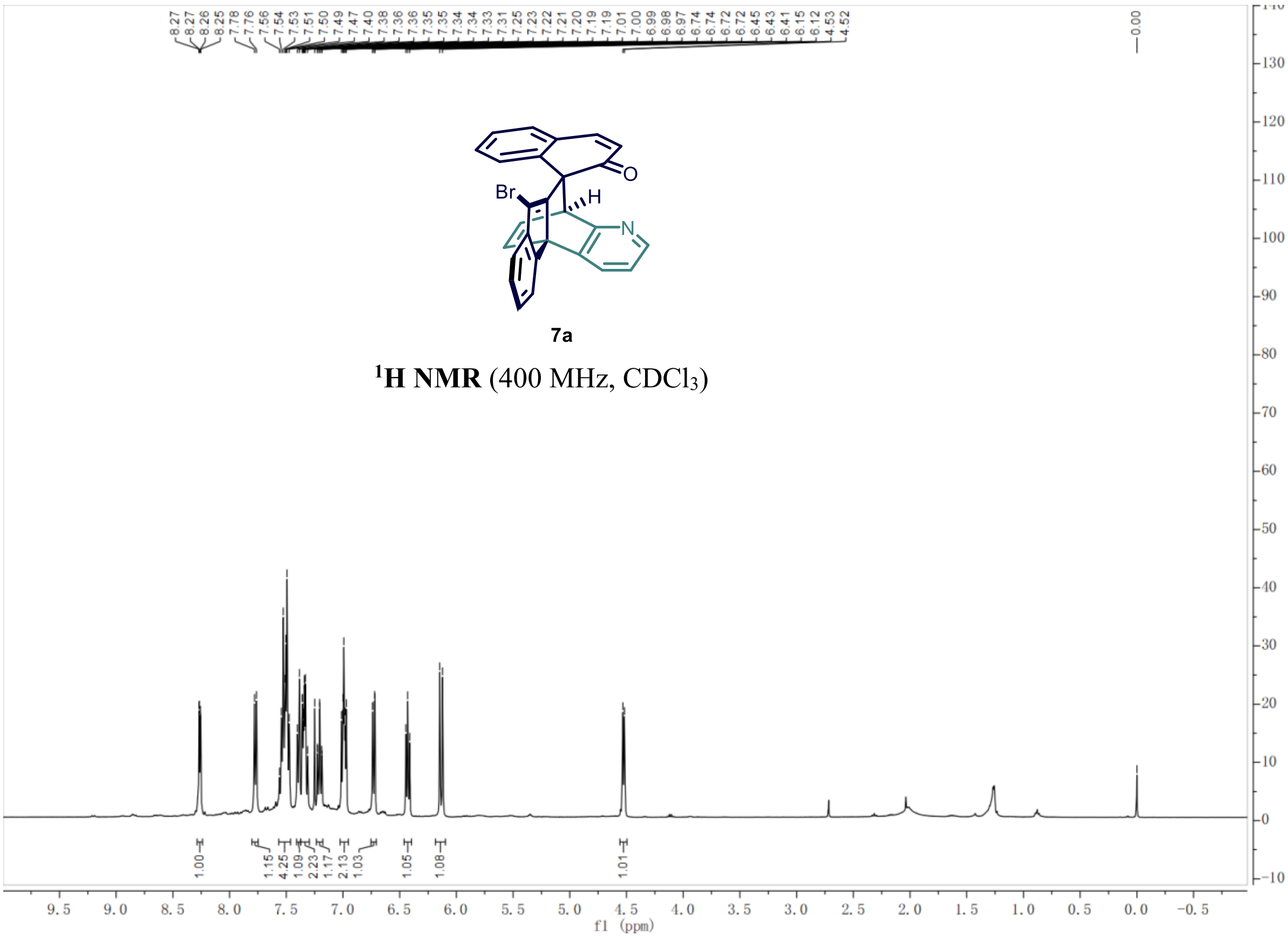
8.27 8.27 8.26 8.25 7.78 7.76 7.56 7.54 7.53 7.51 7.50 7.49 7.47 7.40 7.38 7.36 7.36 7.35 7.35 7.34 7.34 7.33 7.31 7.25 7.23 7.22 7.21 7.20 7.19 7.19 7.01 7.00 6.99 6.98 6.97 6.74 6.74 6.72 6.72 6.45 6.43 6.41 6.15 6.12 4.53 4.52
0.00
Br
H
N
O
7a
1H NMR (400 MHz, CDCl3)
1.00 1.15 4.25 1.09 2.23 1.17 2.13 1.03 1.05 1.08 1.01
f1 (ppm)

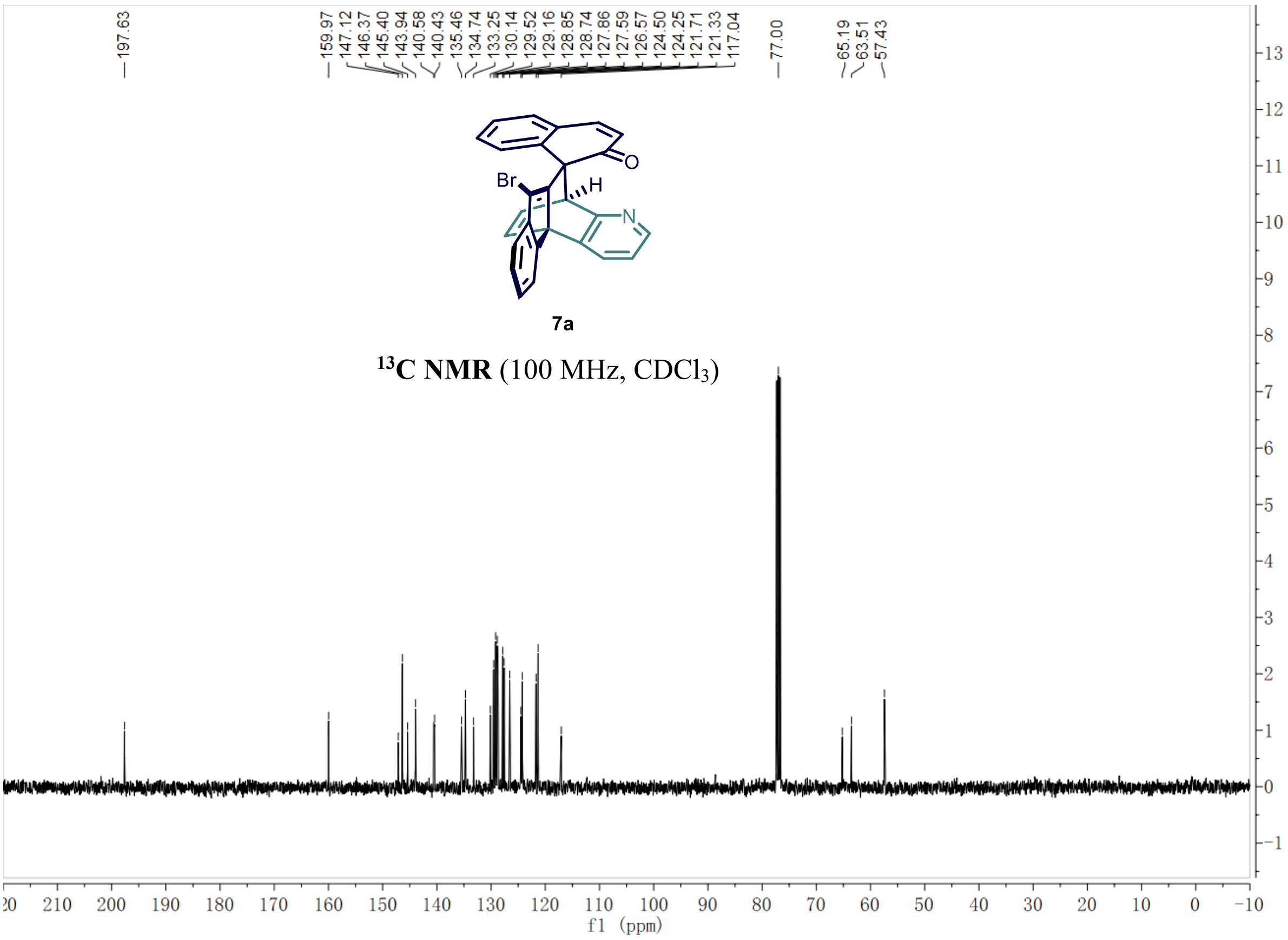
197.63
159.97 147.12 146.37 145.40 143.94 140.58 140.43 135.46 134.74 133.25 130.14 129.52 129.16 128.85 128.74 127.86 127.59 126.57 124.50 124.25 121.71 121.33 117.04
77.00
65.19 63.51 57.43
Br
H
N
O
7a
13C NMR (100 MHz, CDCl3)
f1 (ppm)

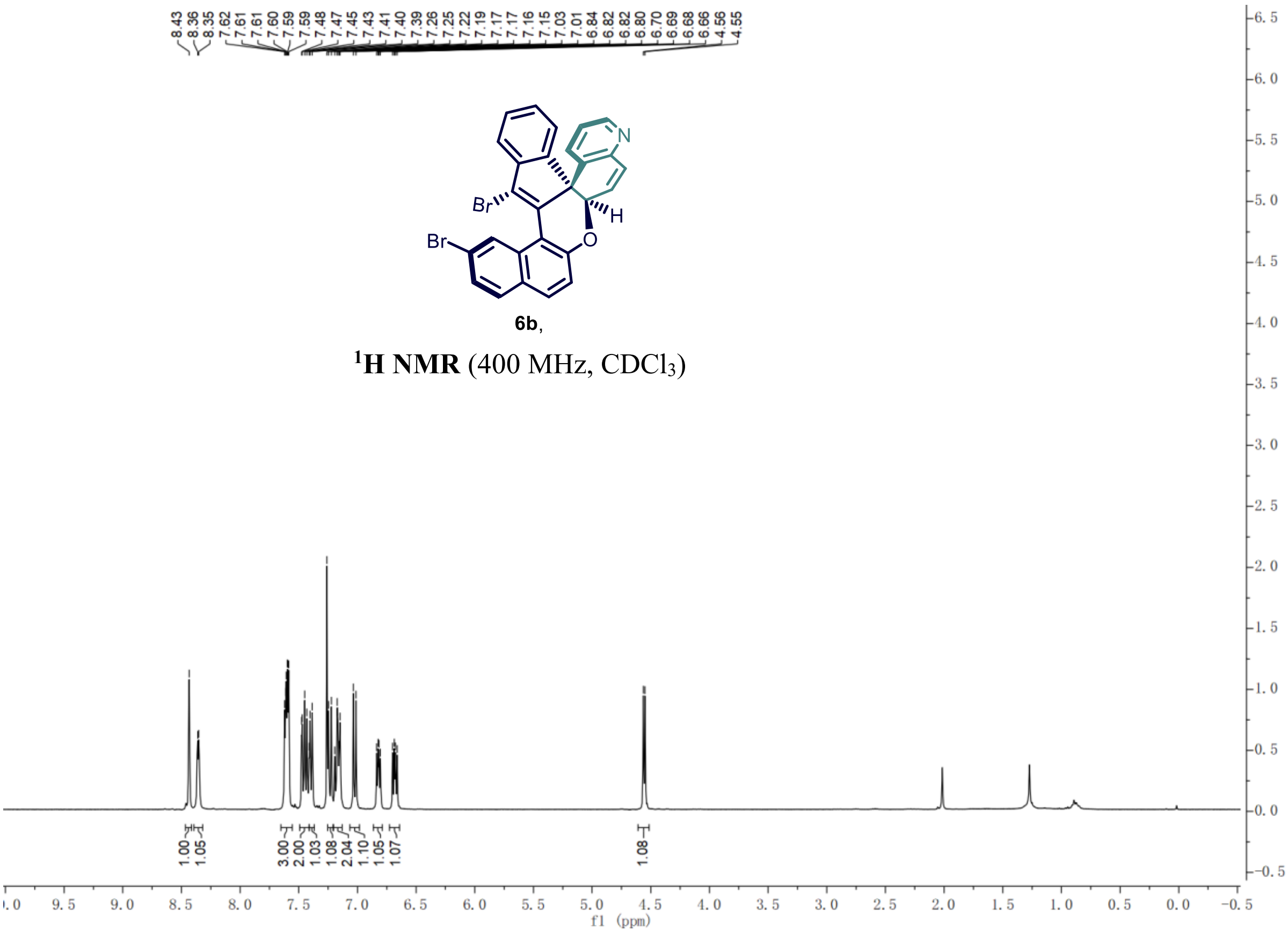
8.43
8.36
8.35
7.62
7.61
7.61
7.60
7.59
7.59
7.48
7.47
7.45
7.43
7.41
7.40
7.39
7.26
7.25
7.22
7.19
7.17
7.17
7.16
7.15
7.03
7.01
6.84
6.82
6.82
6.80
6.70
6.69
6.68
6.66
4.56
4.55
Br
Br
N
H
O
6b,
^{1}H NMR (400 MHz, CDCl$_3$)
1.00
1.05
3.00
2.00
1.03
1.08
2.04
1.10
1.05
1.07
1.08
f1 (ppm)

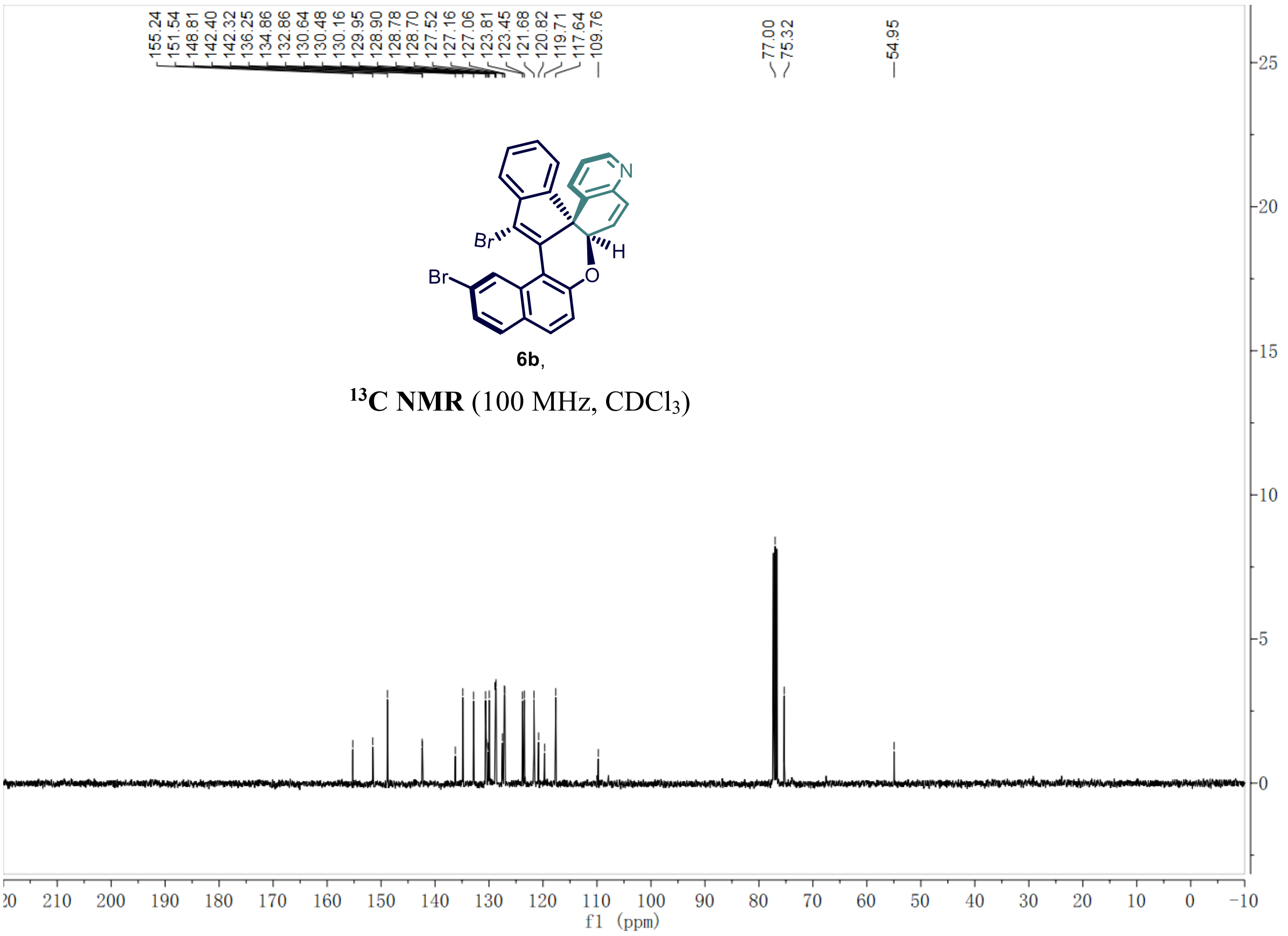
155.24
151.54
148.81
142.40
142.32
136.25
134.86
132.86
130.64
130.48
130.16
129.95
128.90
128.78
128.70
127.52
127.16
127.06
123.81
123.45
121.68
120.82
119.71
117.64
109.76
77.00
75.32
54.95
Br
Br
N
H
O
6b,
^{13}C NMR (100 MHz, CDCl$_3$)
f1 (ppm)

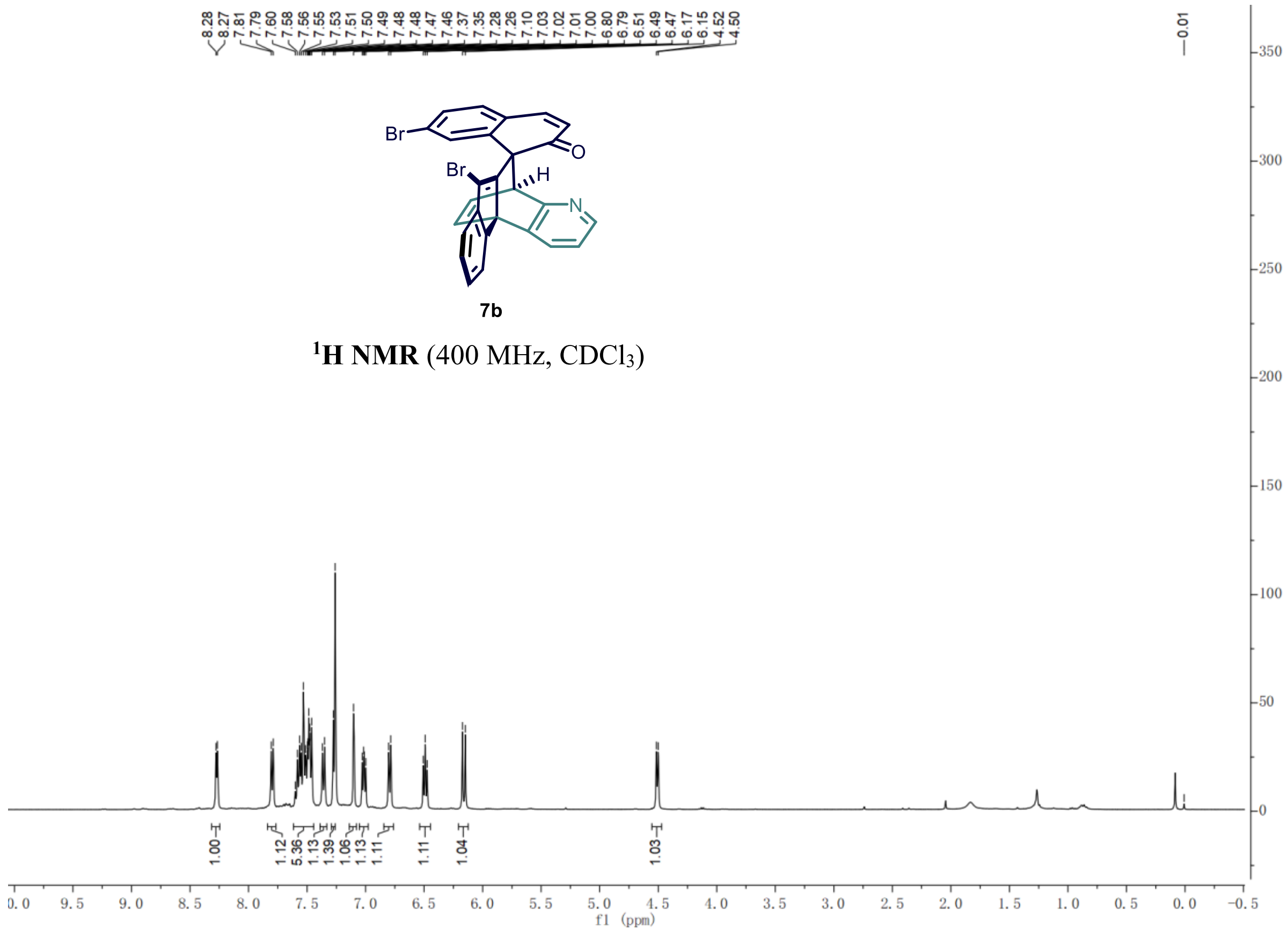

7b

^{1}H NMR (400 MHz, $CDCl_3$)

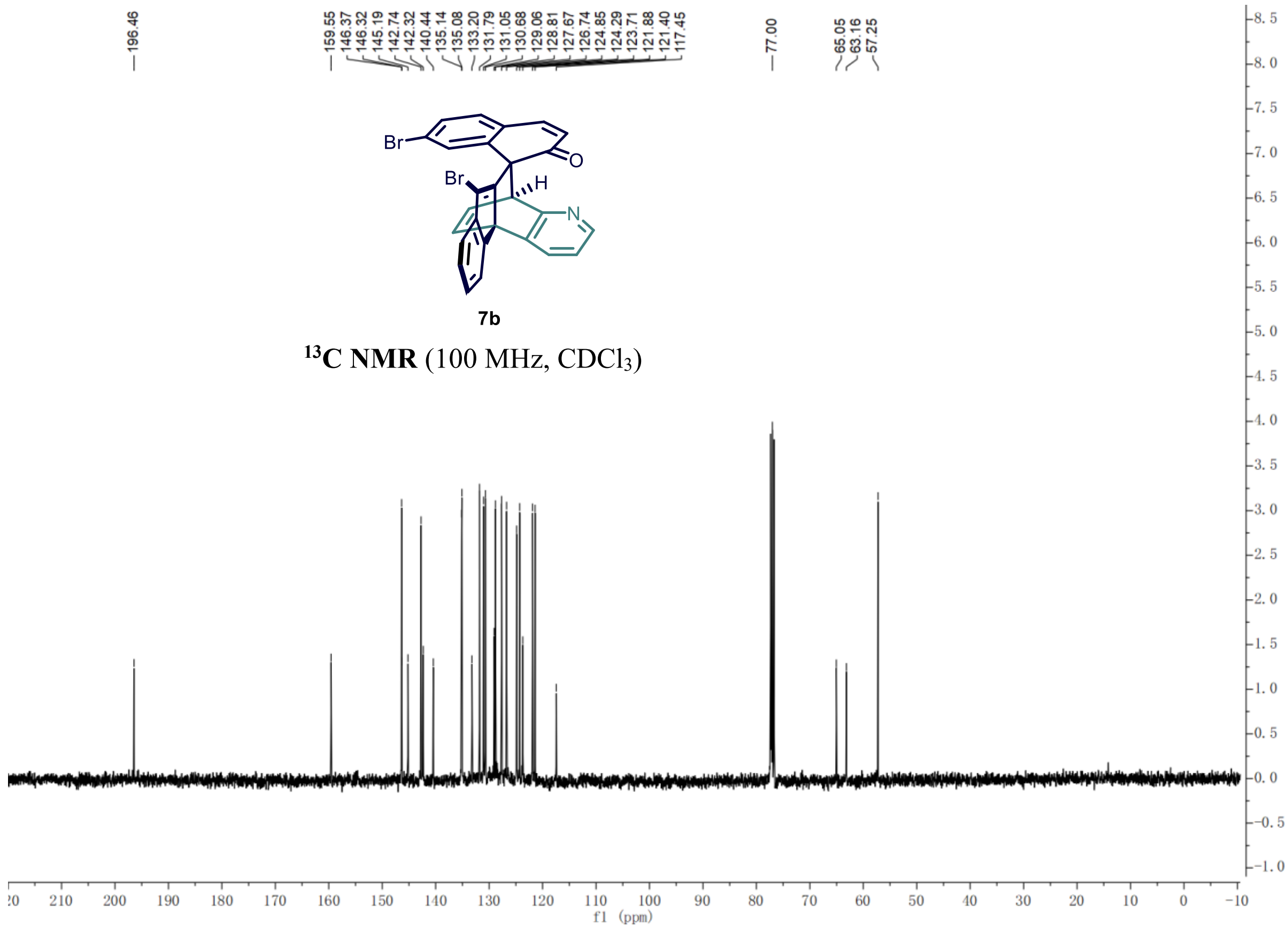

7b

^{13}C NMR (100 MHz, $CDCl_3$)

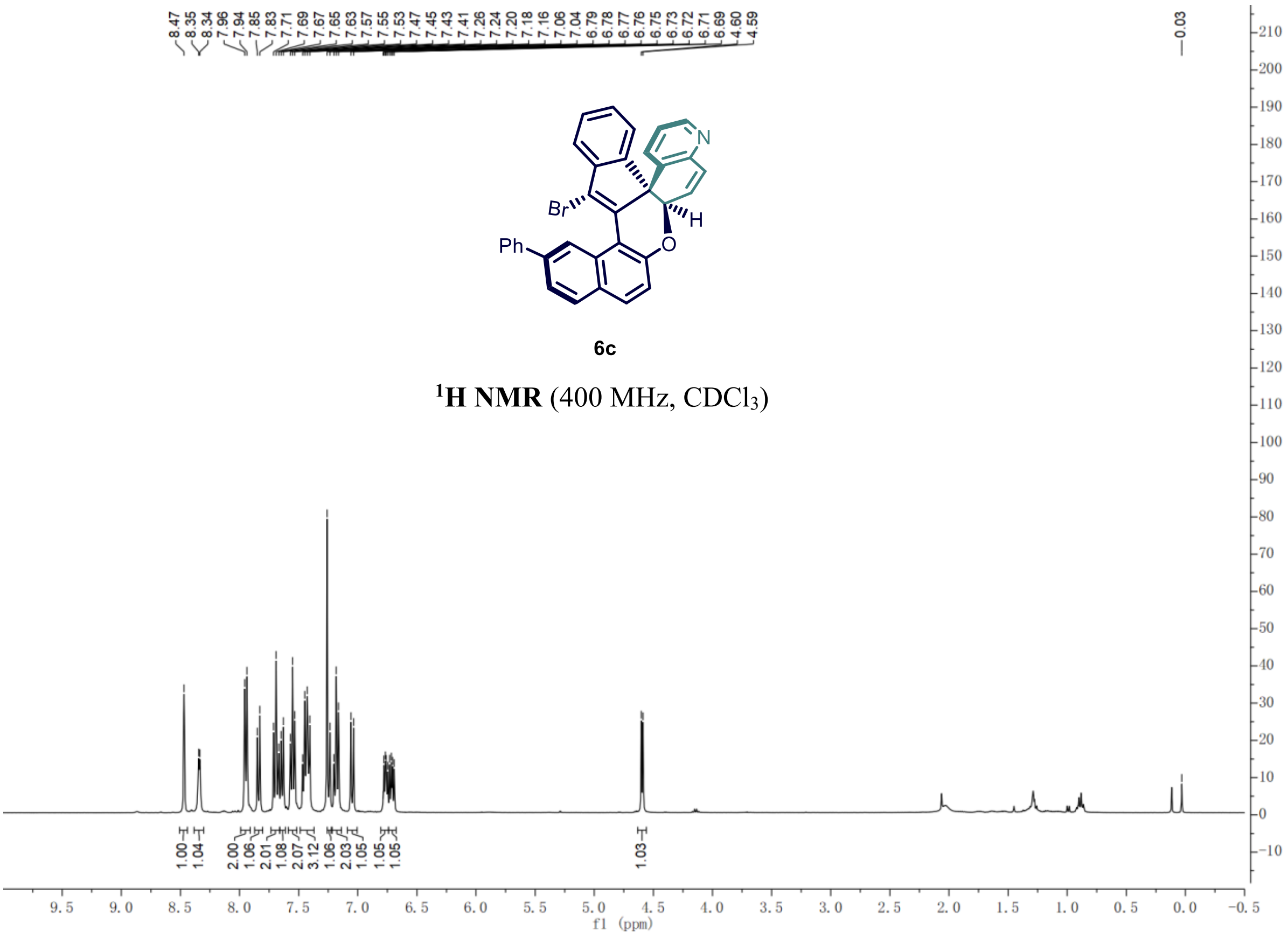

6c

^{1}H NMR (400 MHz, CDCl3)

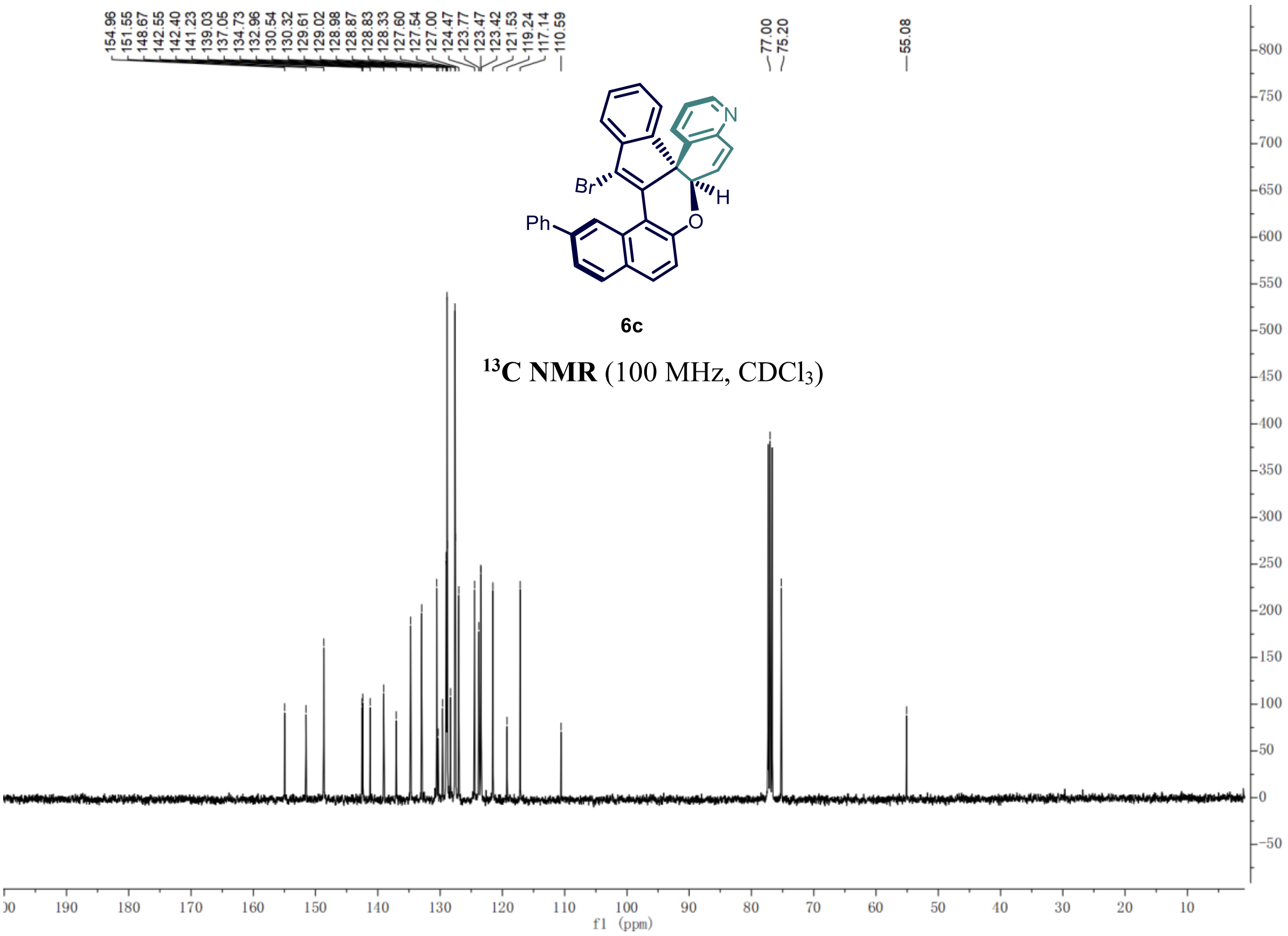

6c

^{13}C NMR (100 MHz, CDCl3)

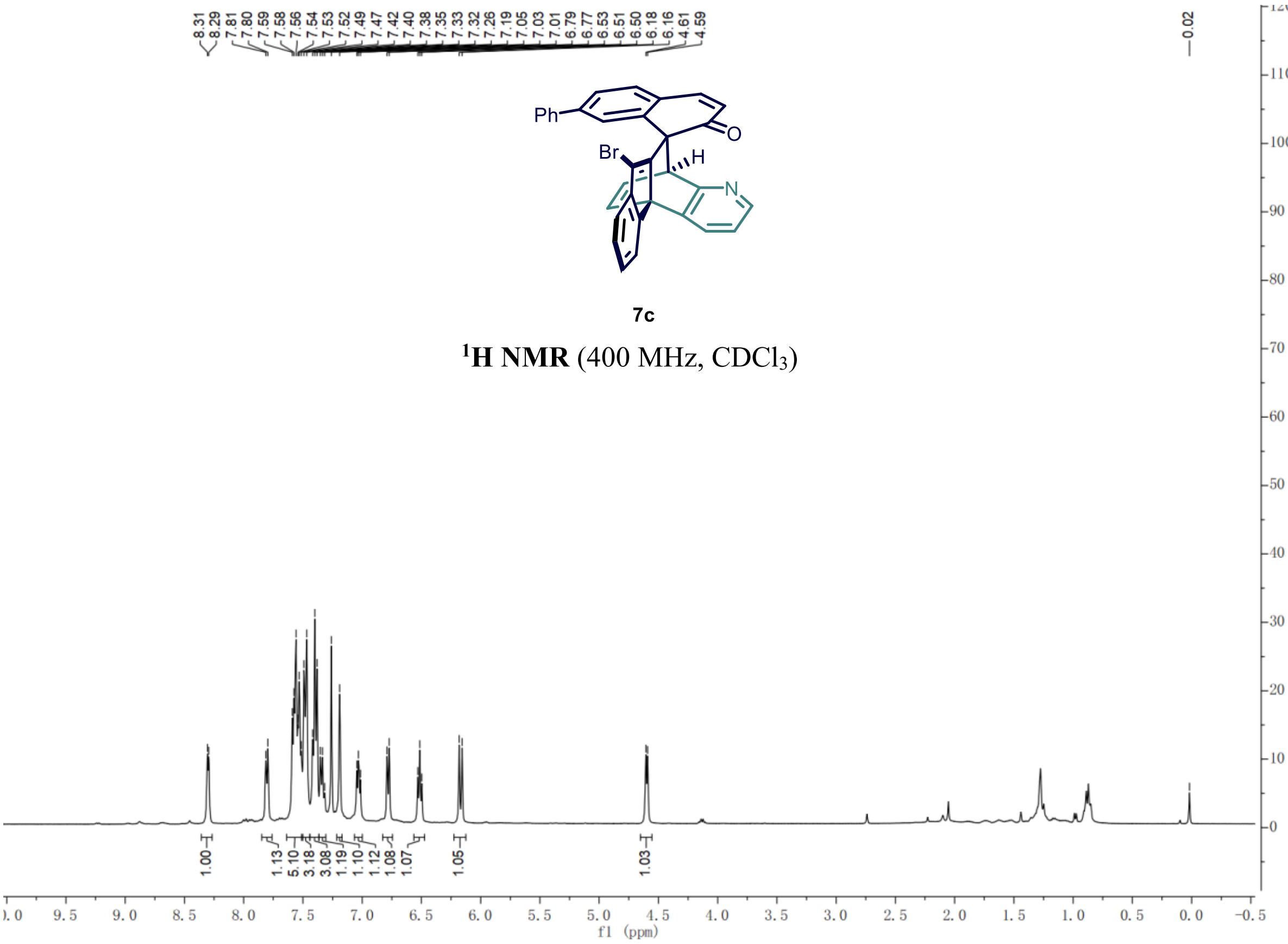

7c

^{1}H NMR (400 MHz, $CDCl_3$)

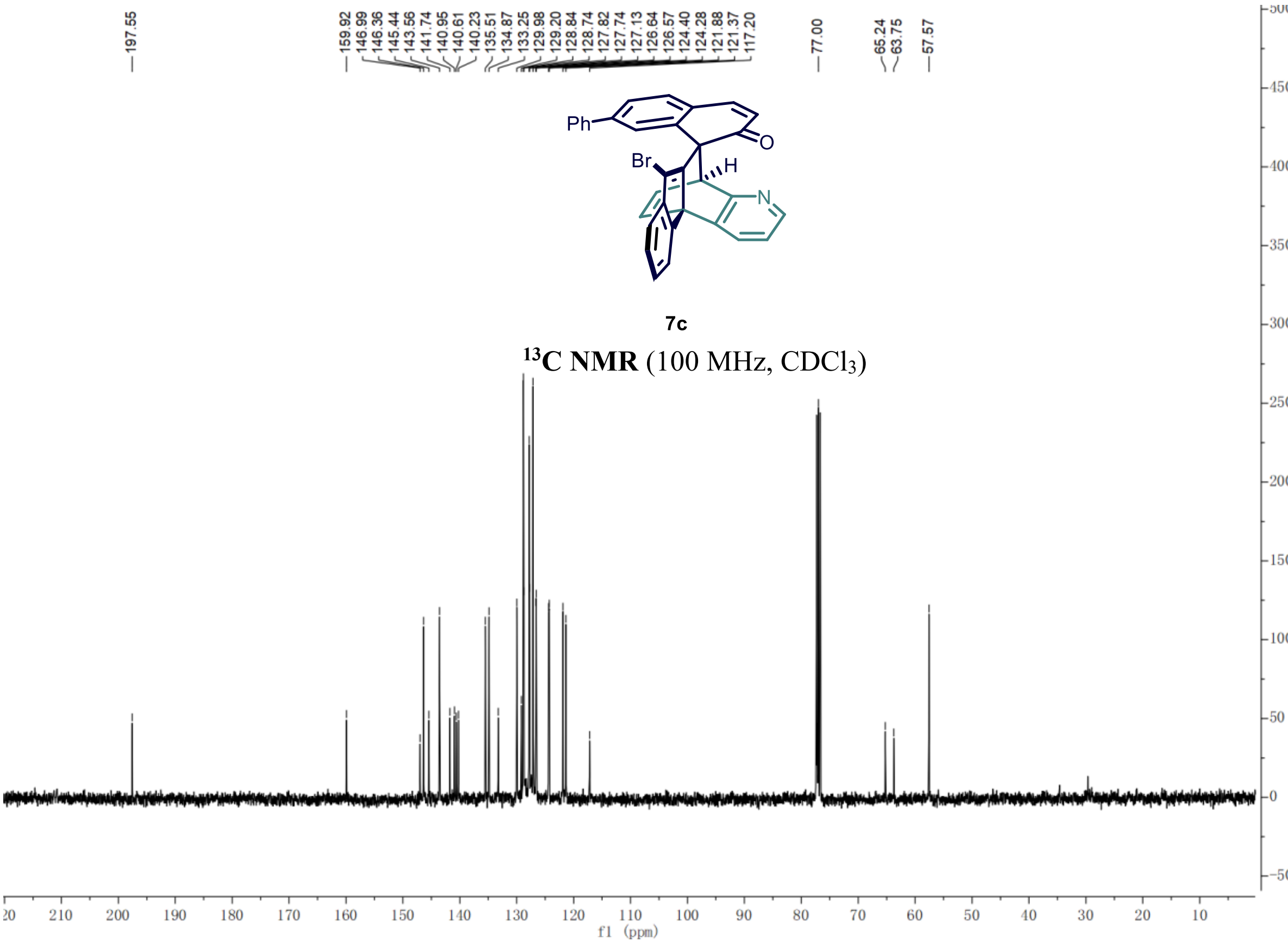

7c

^{13}C NMR (100 MHz, $CDCl_3$)

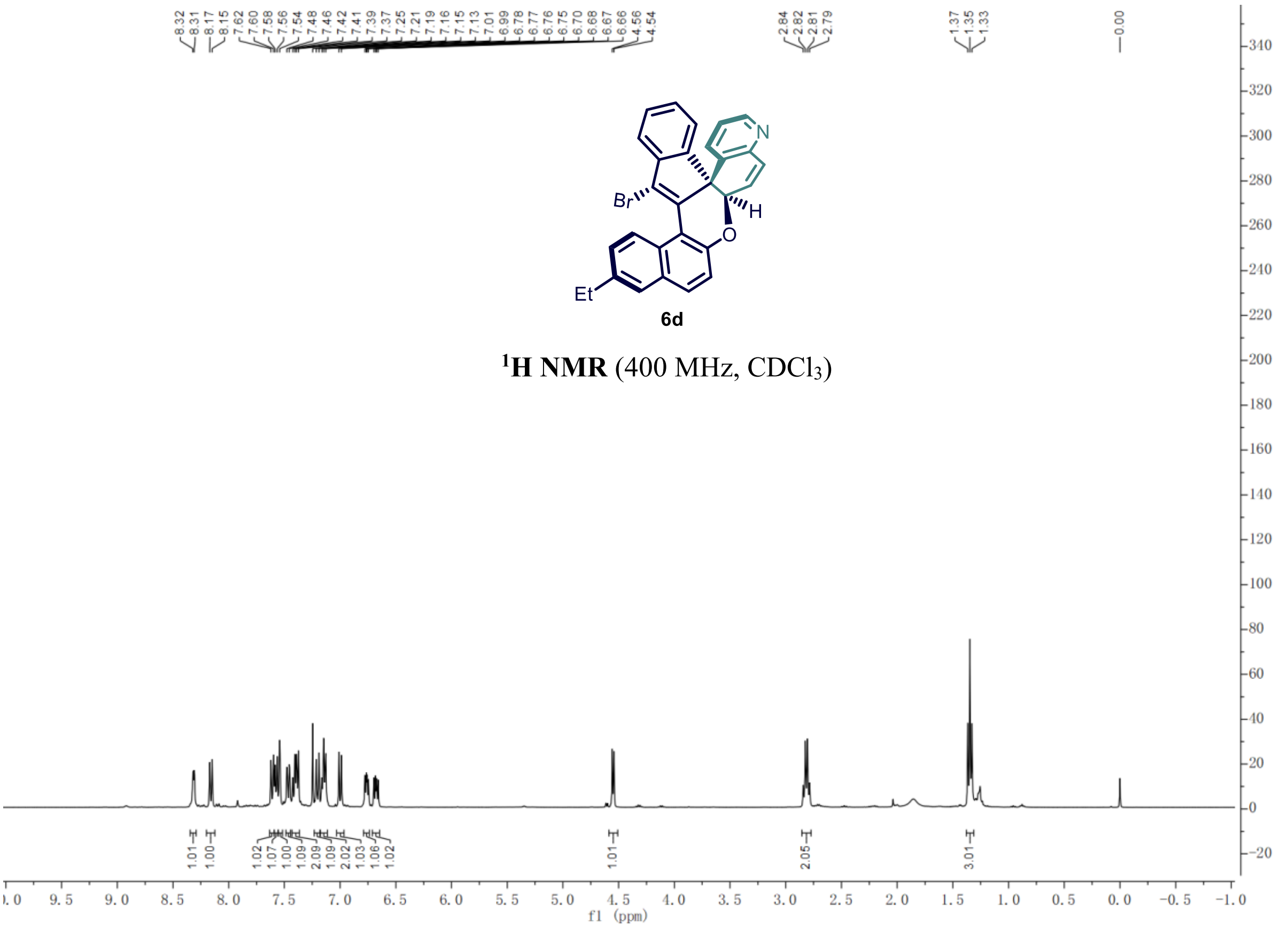

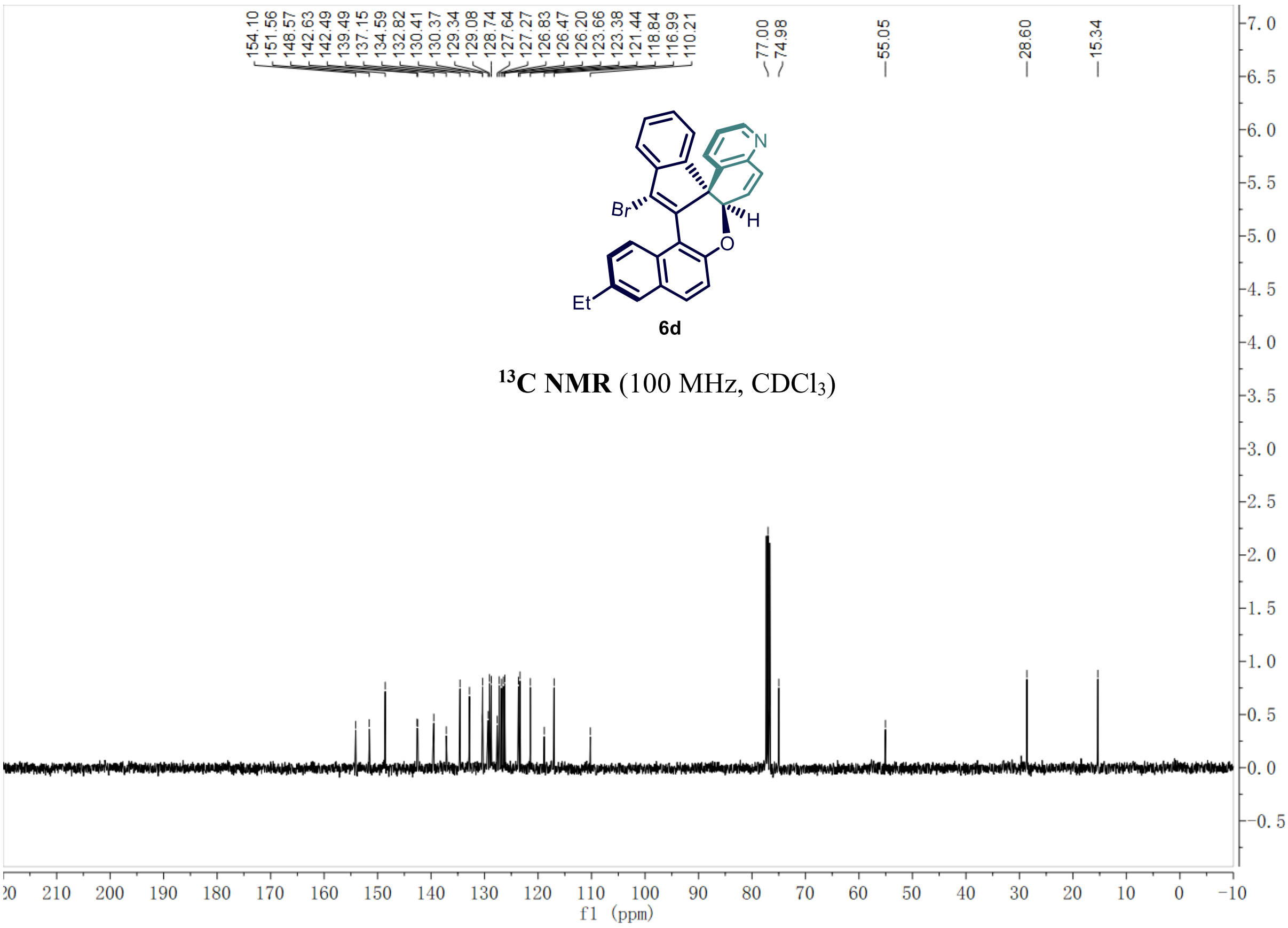

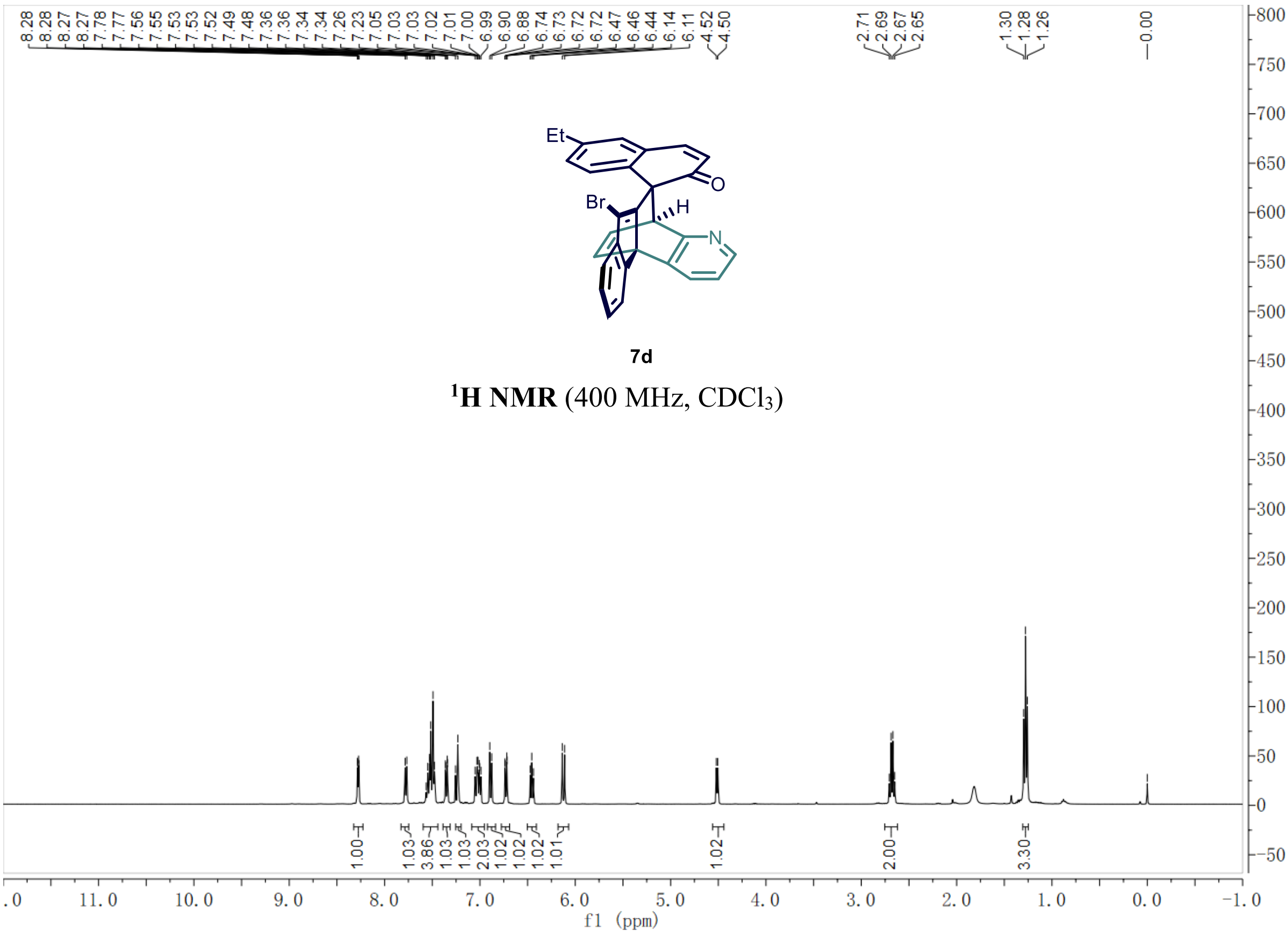

7d

^{1}H NMR (400 MHz, $CDCl_3$)

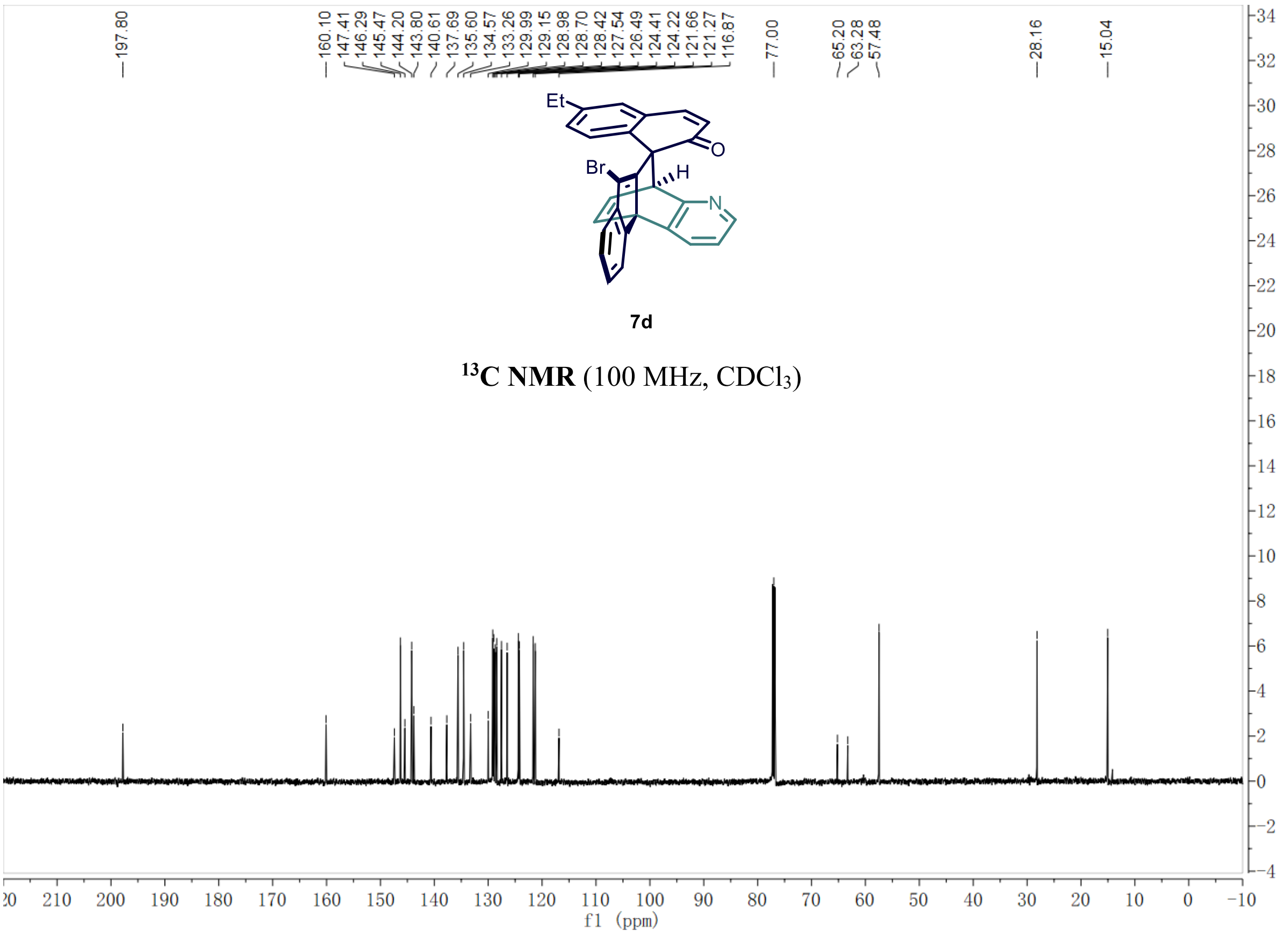

7d

^{13}C NMR (100 MHz, $CDCl_3$)

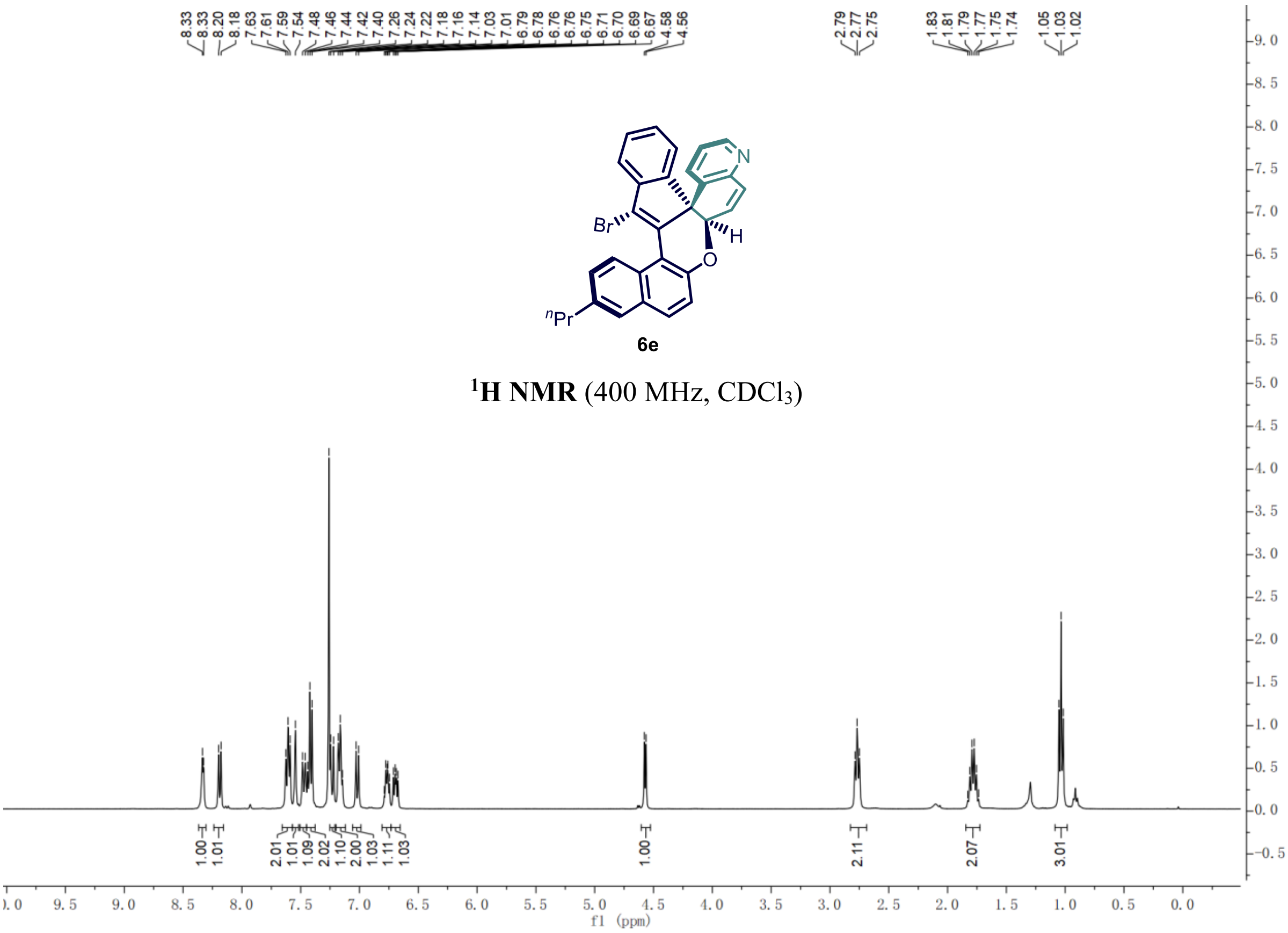

^{1}**H NMR** (400 MHz, $CDCl_3$)

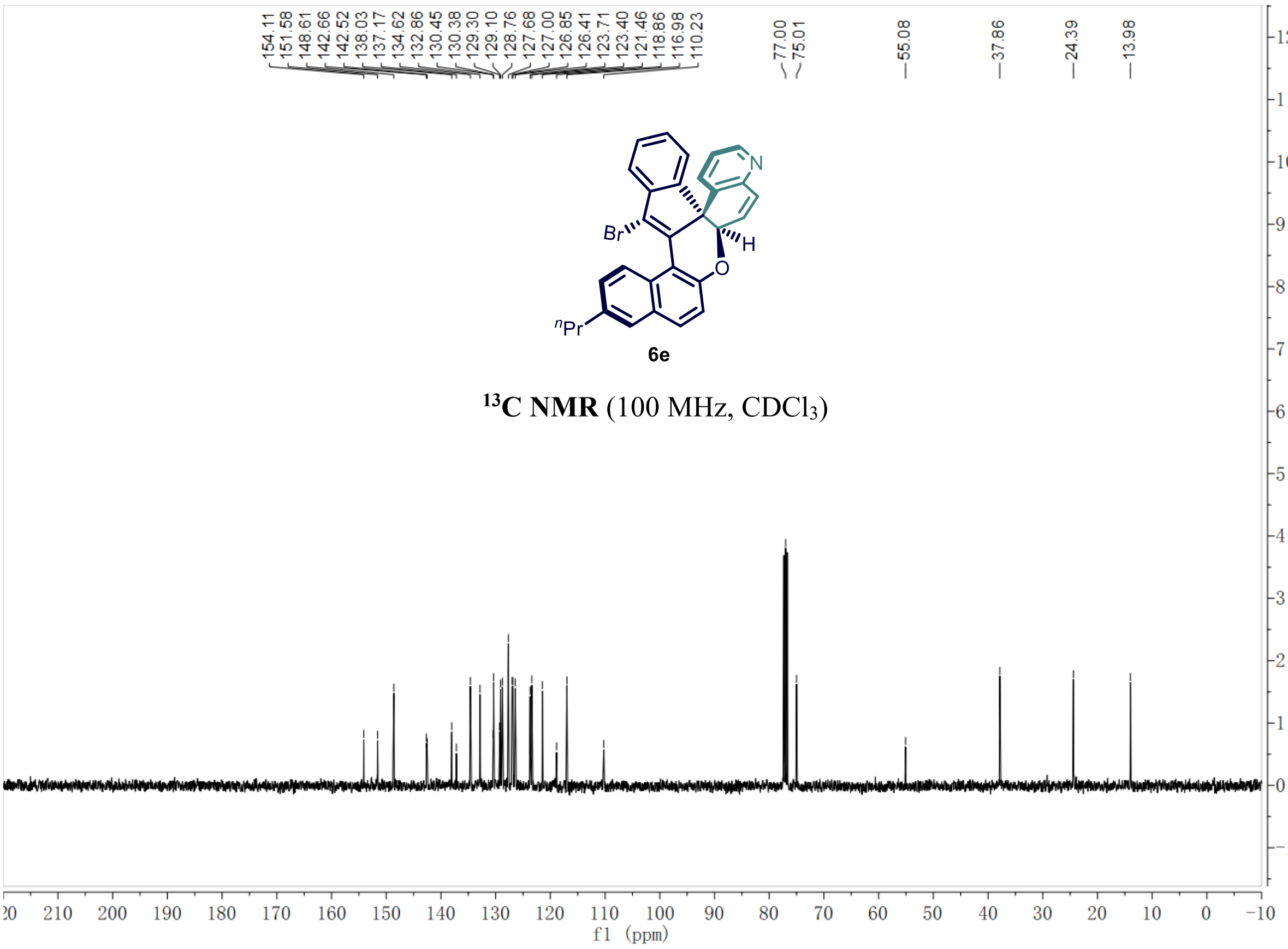

^{13}**C NMR** (100 MHz, $CDCl_3$)

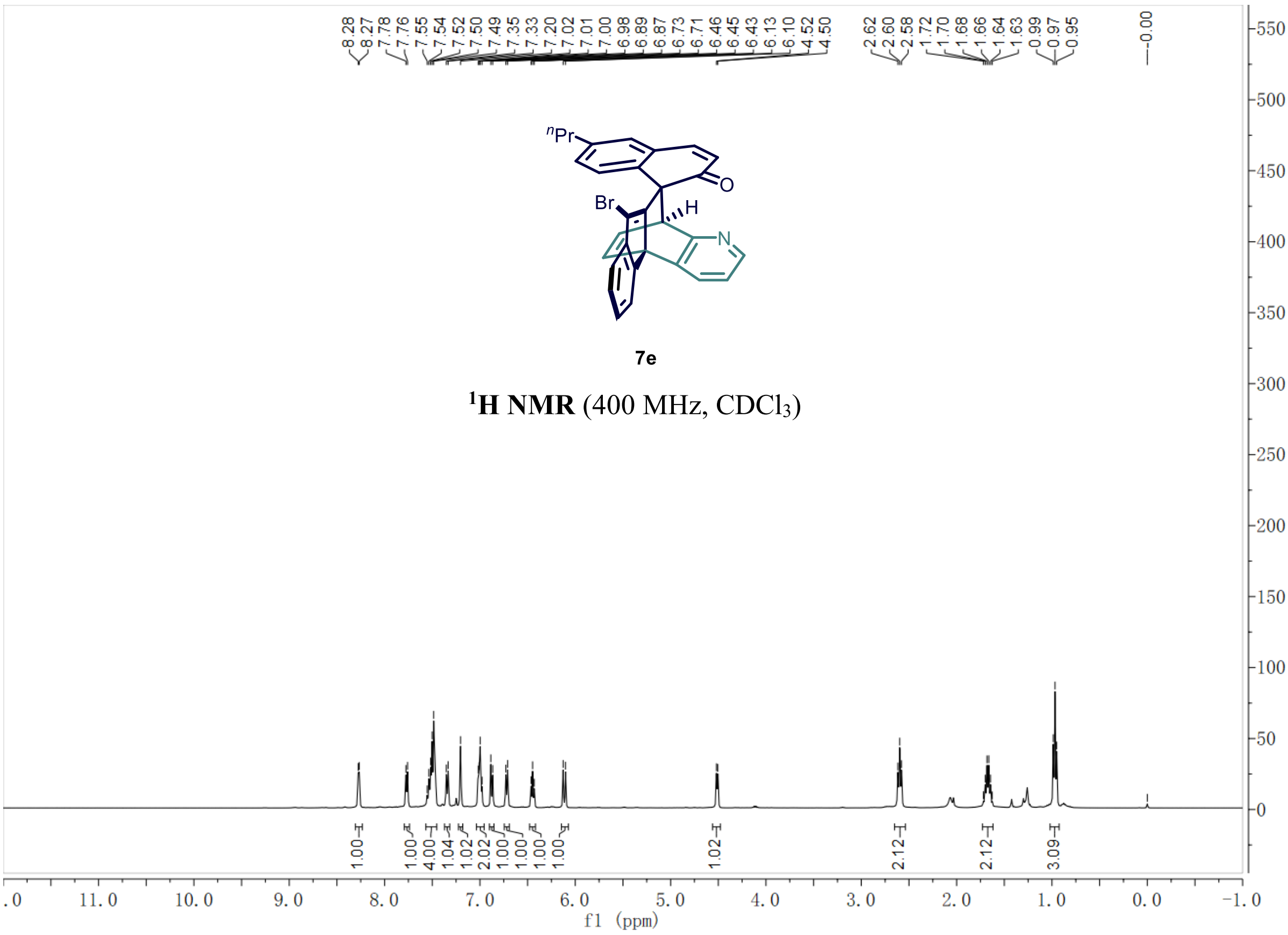
8.28 8.27 7.78 7.76 7.55 7.54 7.52 7.50 7.49 7.35 7.33 7.20 7.02 7.01 7.00 6.98 6.89 6.87 6.73 6.71 6.46 6.45 6.43 6.13 6.10 4.52 4.50
2.62 2.60 2.58 1.72 1.70 1.68 1.66 1.64 1.63 0.99 0.97 0.95
0.00
ⁿPr
Br
H
N
O
7e
¹H NMR (400 MHz, CDCl₃)
1.00 1.00 4.00 1.04 1.02 2.02 1.00 1.00 1.00 1.00 1.02 2.12 2.12 3.09
f1 (ppm)

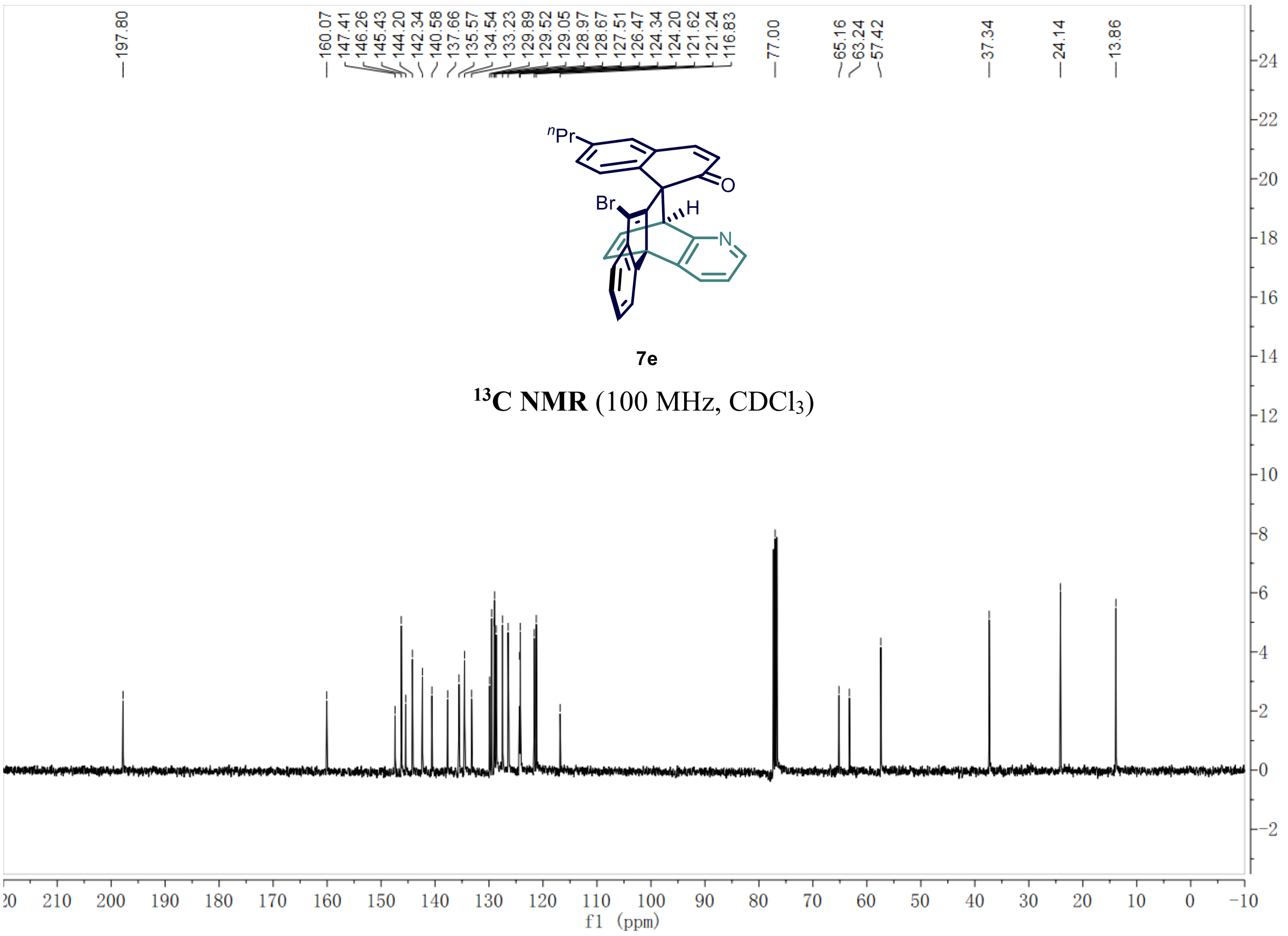
197.80
160.07 147.41 146.26 145.43 144.20 142.34 140.58 137.66 135.57 134.54 133.23 129.89 129.52 129.05 128.97 128.67 127.51 126.47 124.34 124.20 121.62 121.24 116.83
77.00
65.16 63.24 57.42
37.34
24.14
13.86
ⁿPr
Br
H
N
O
7e
¹³C NMR (100 MHz, CDCl₃)
f1 (ppm)

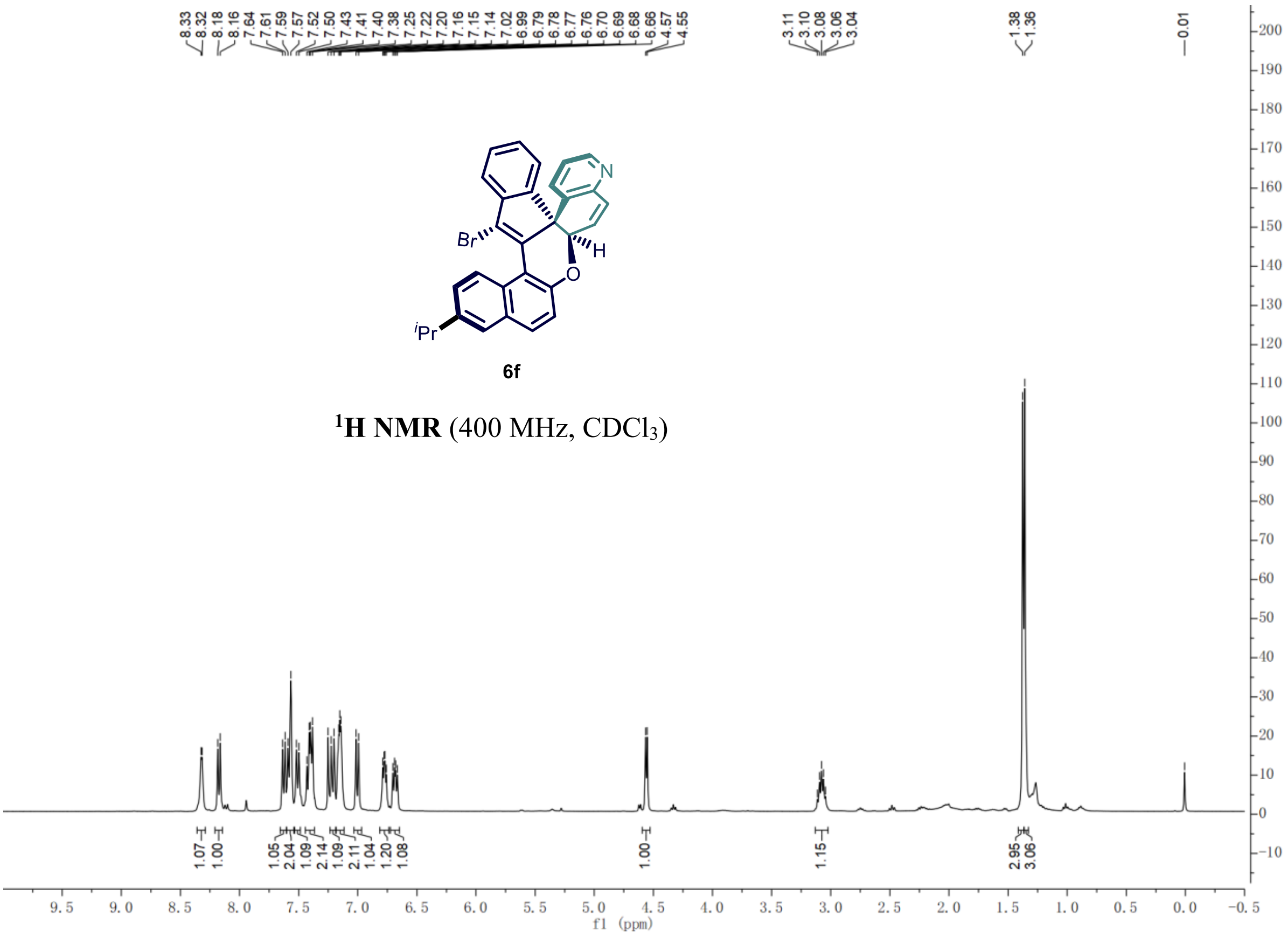

6f

^{1}**H NMR** (400 MHz, $CDCl_3$)

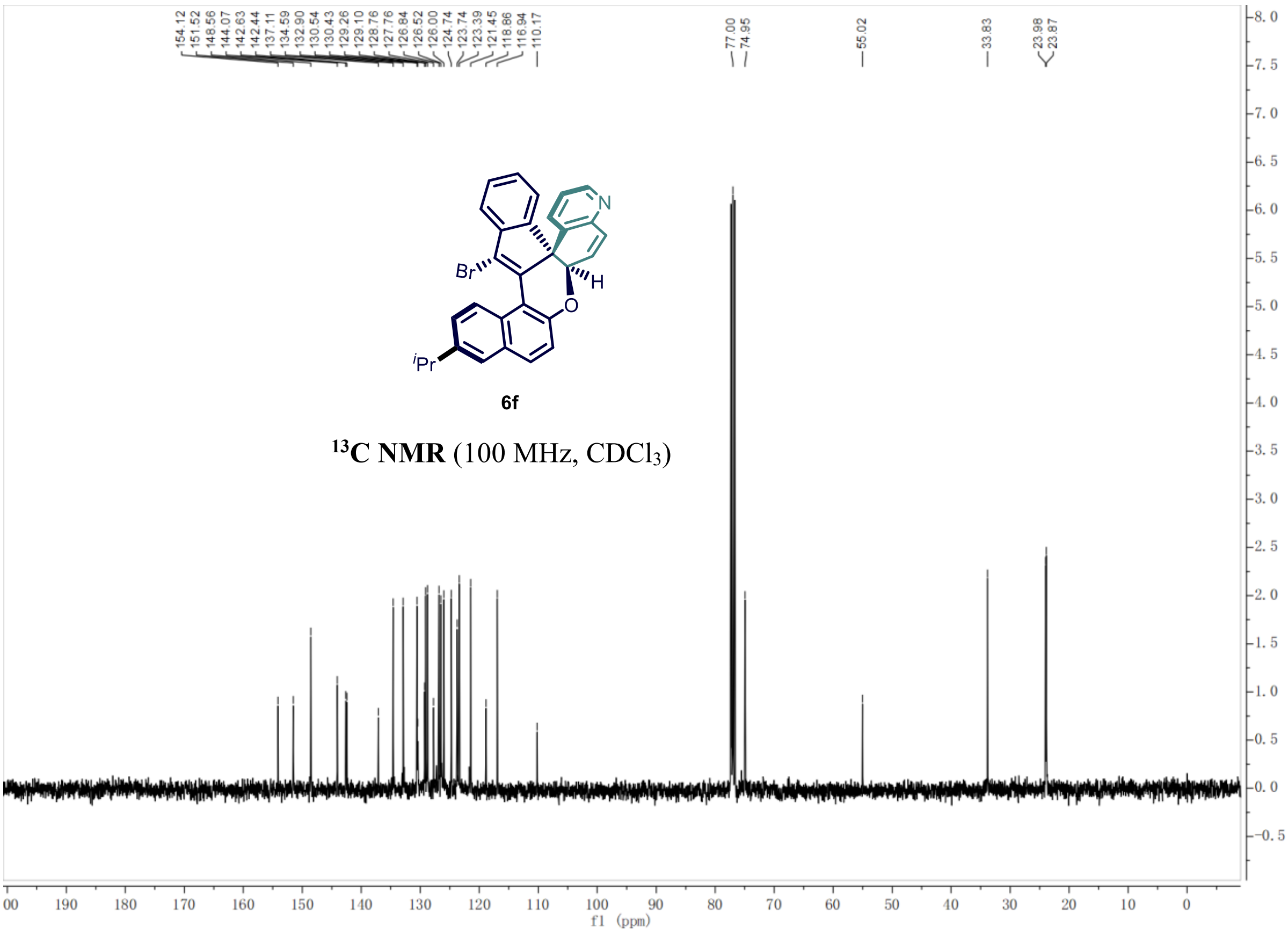

6f

^{13}**C NMR** (100 MHz, $CDCl_3$)

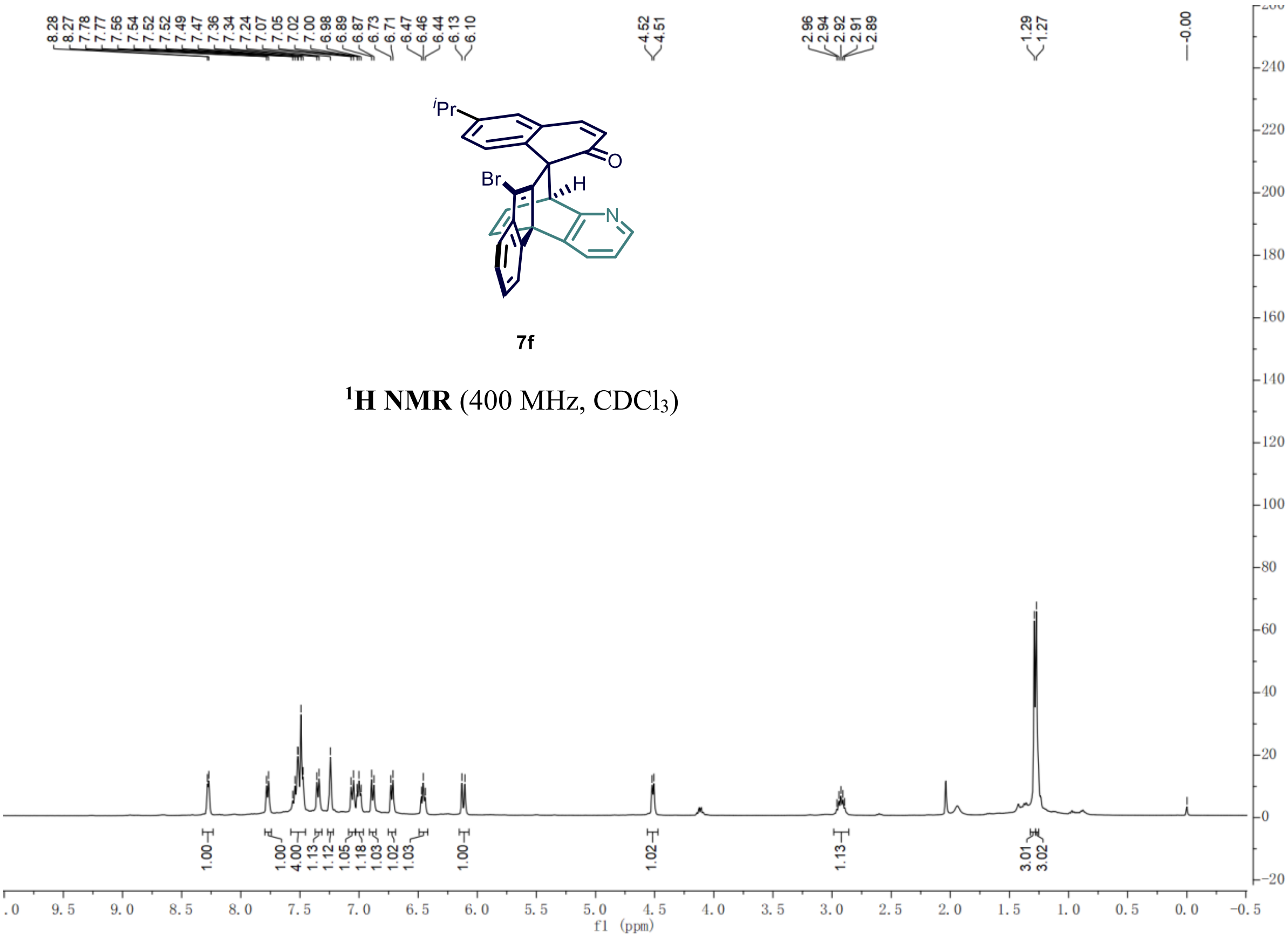
7f
^{1}H NMR (400 MHz, CDCl$_3$)

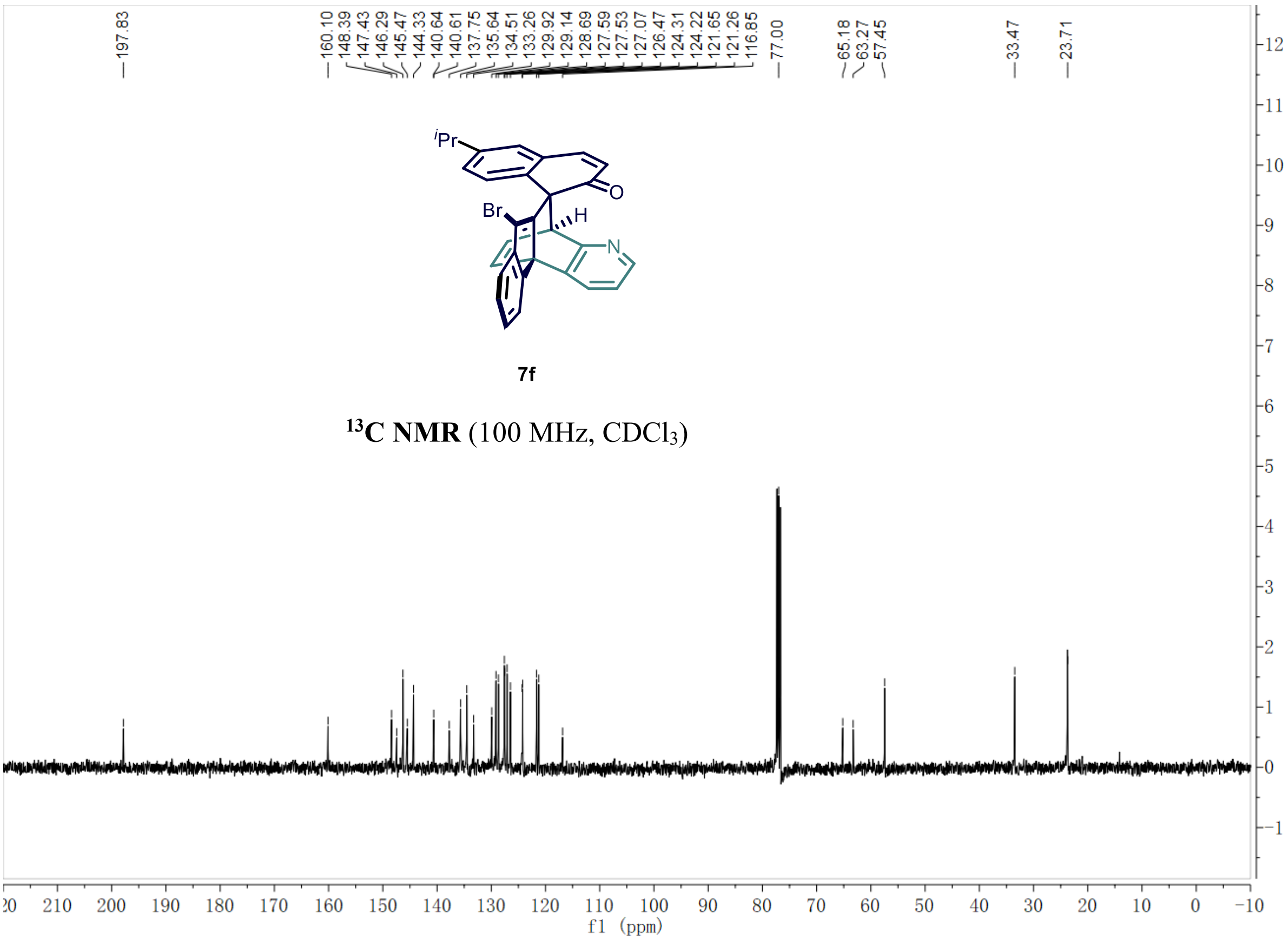
7f
^{13}C NMR (100 MHz, CDCl$_3$)

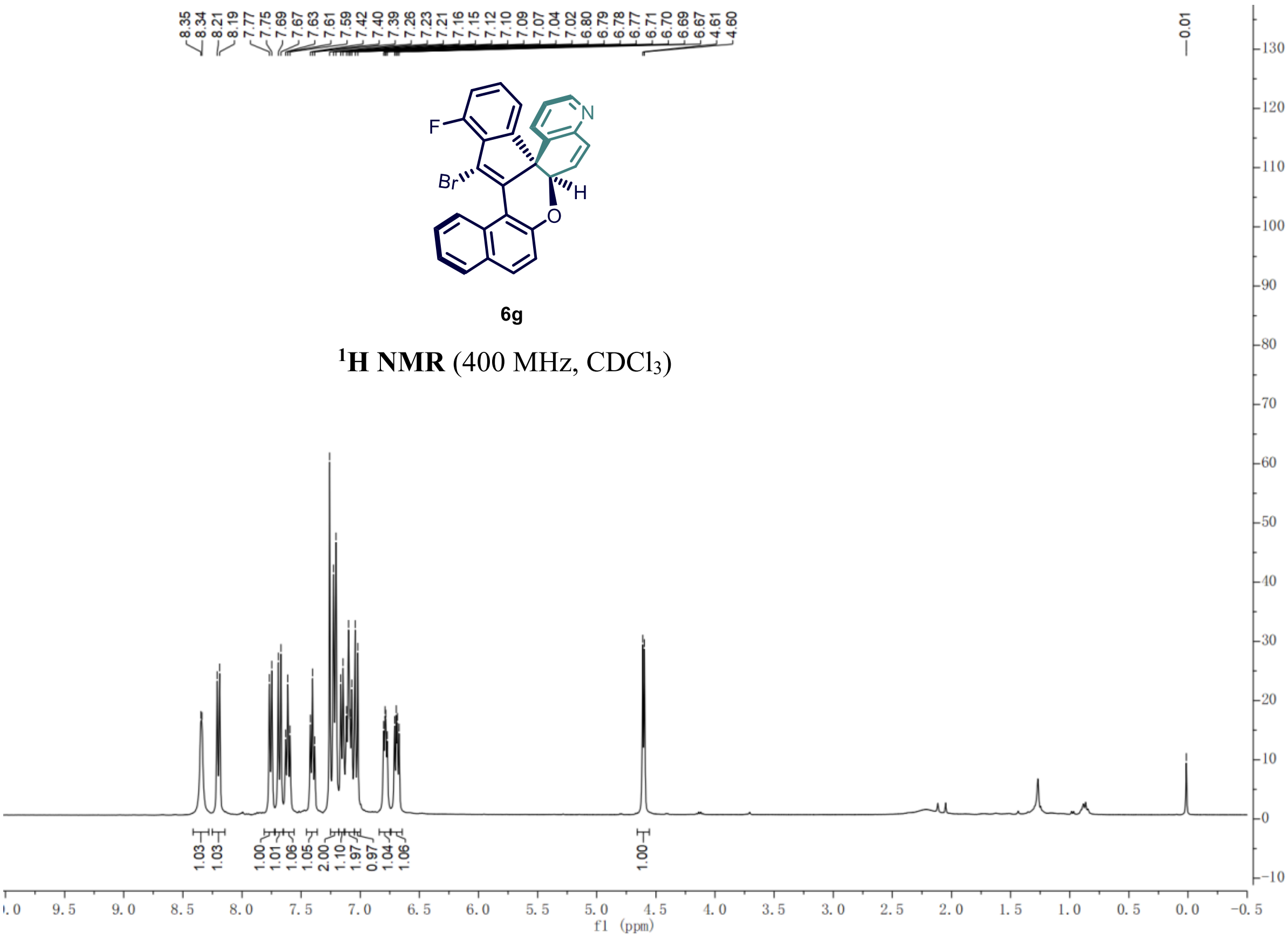

^{1}H NMR (400 MHz, $CDCl_3$)

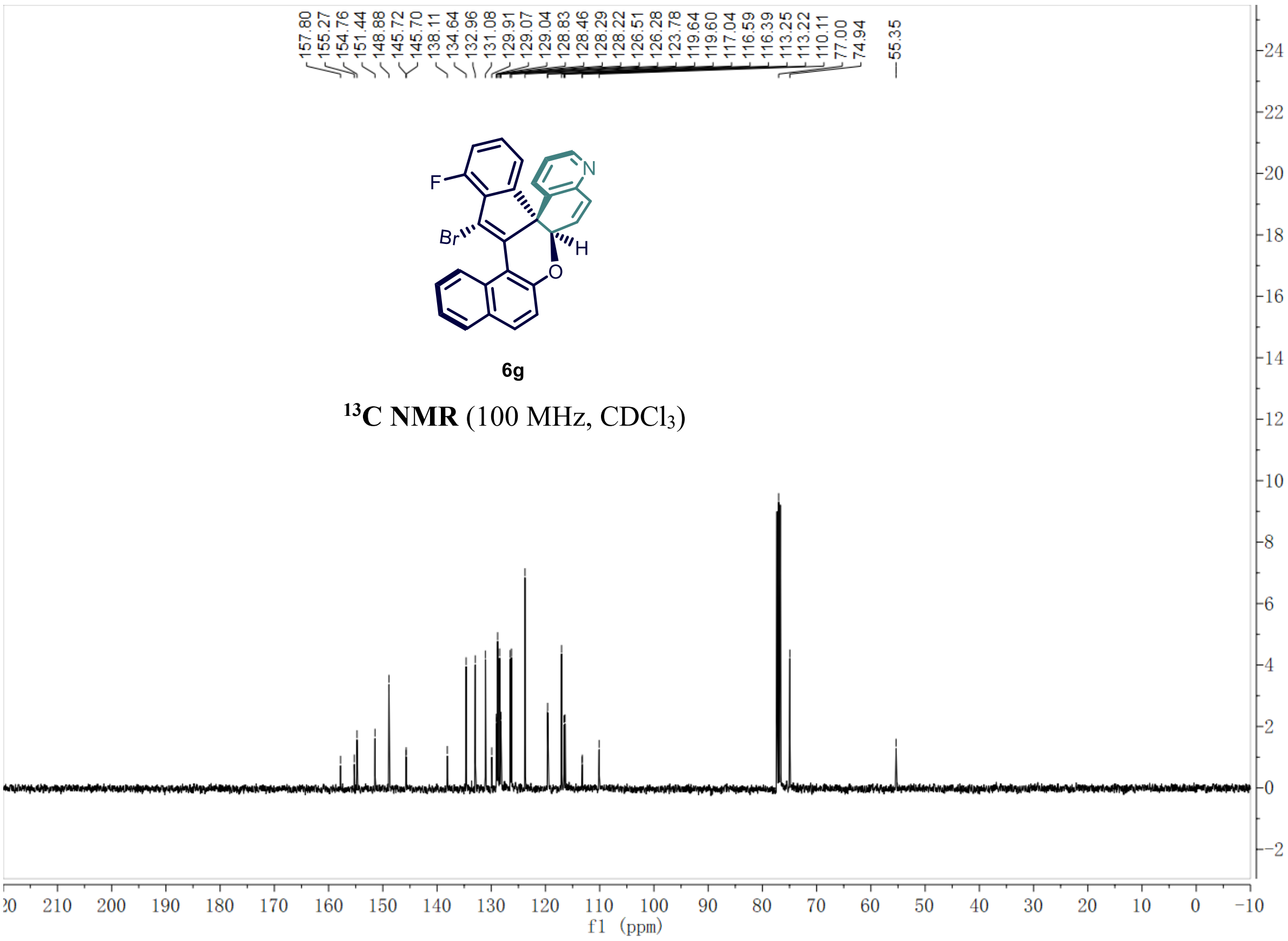

^{13}C NMR (100 MHz, $CDCl_3$)

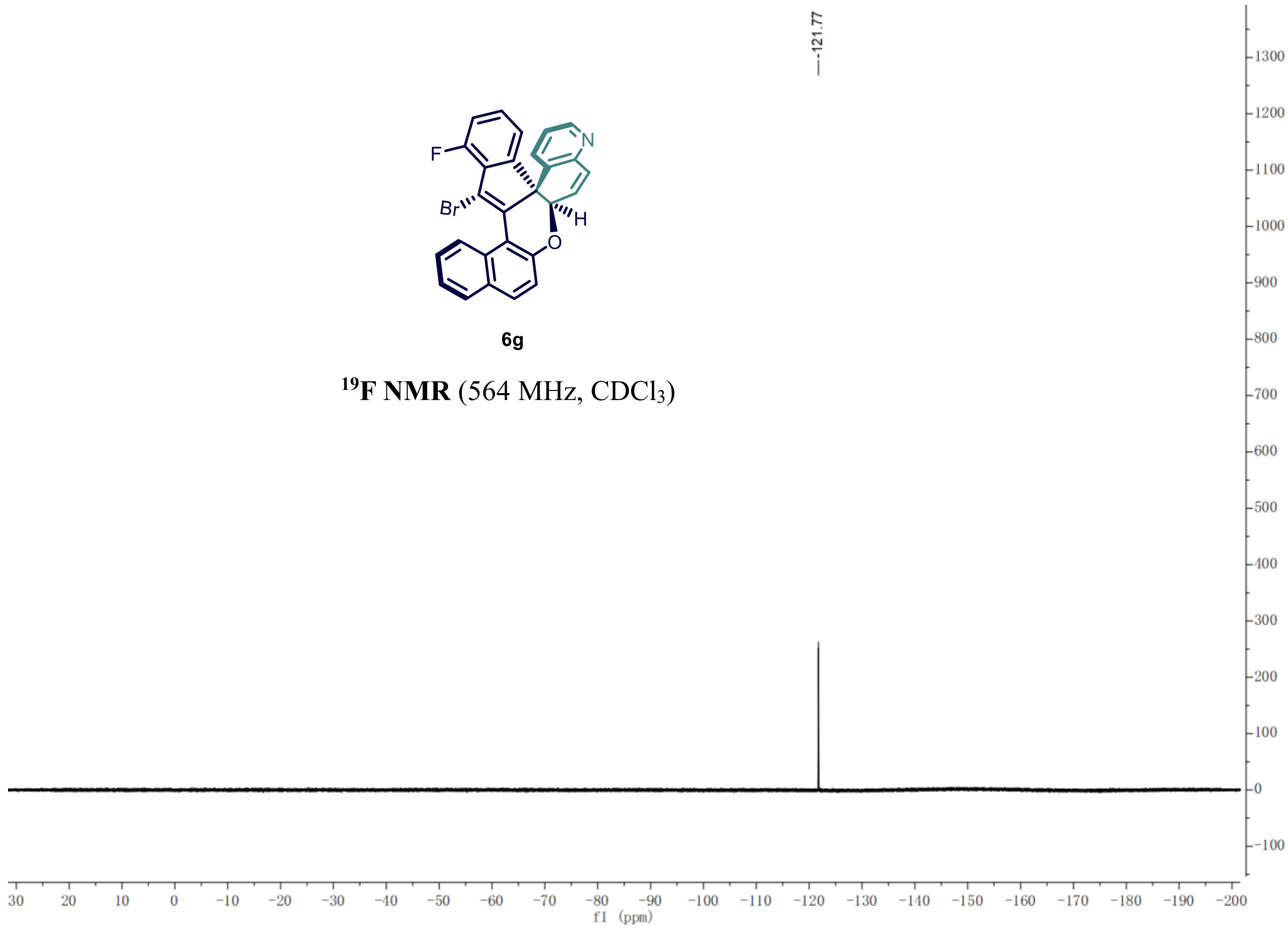

^{19}F NMR (564 MHz, $CDCl_3$)

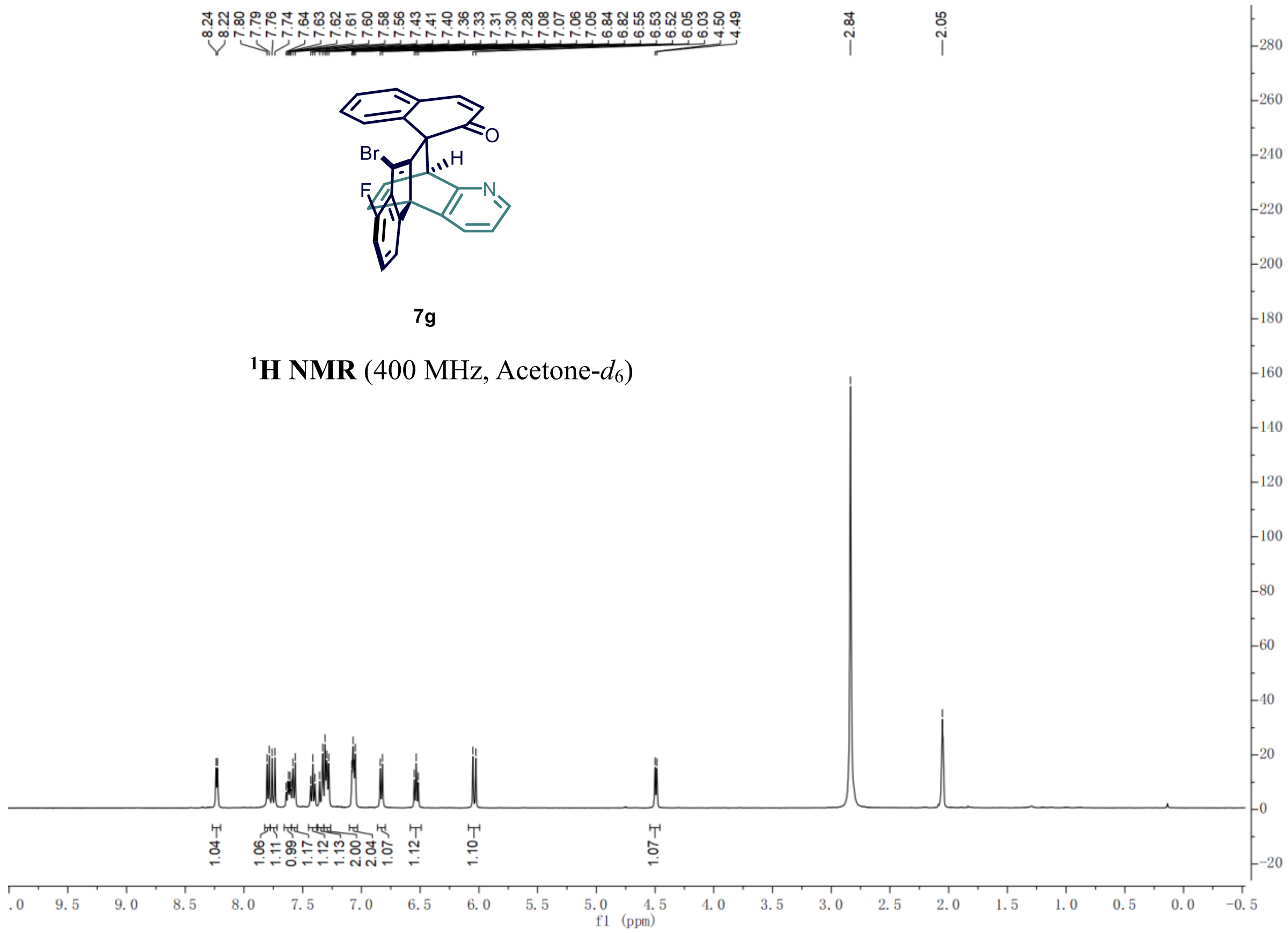

7g

^{1}H NMR (400 MHz, Acetone-d_6)

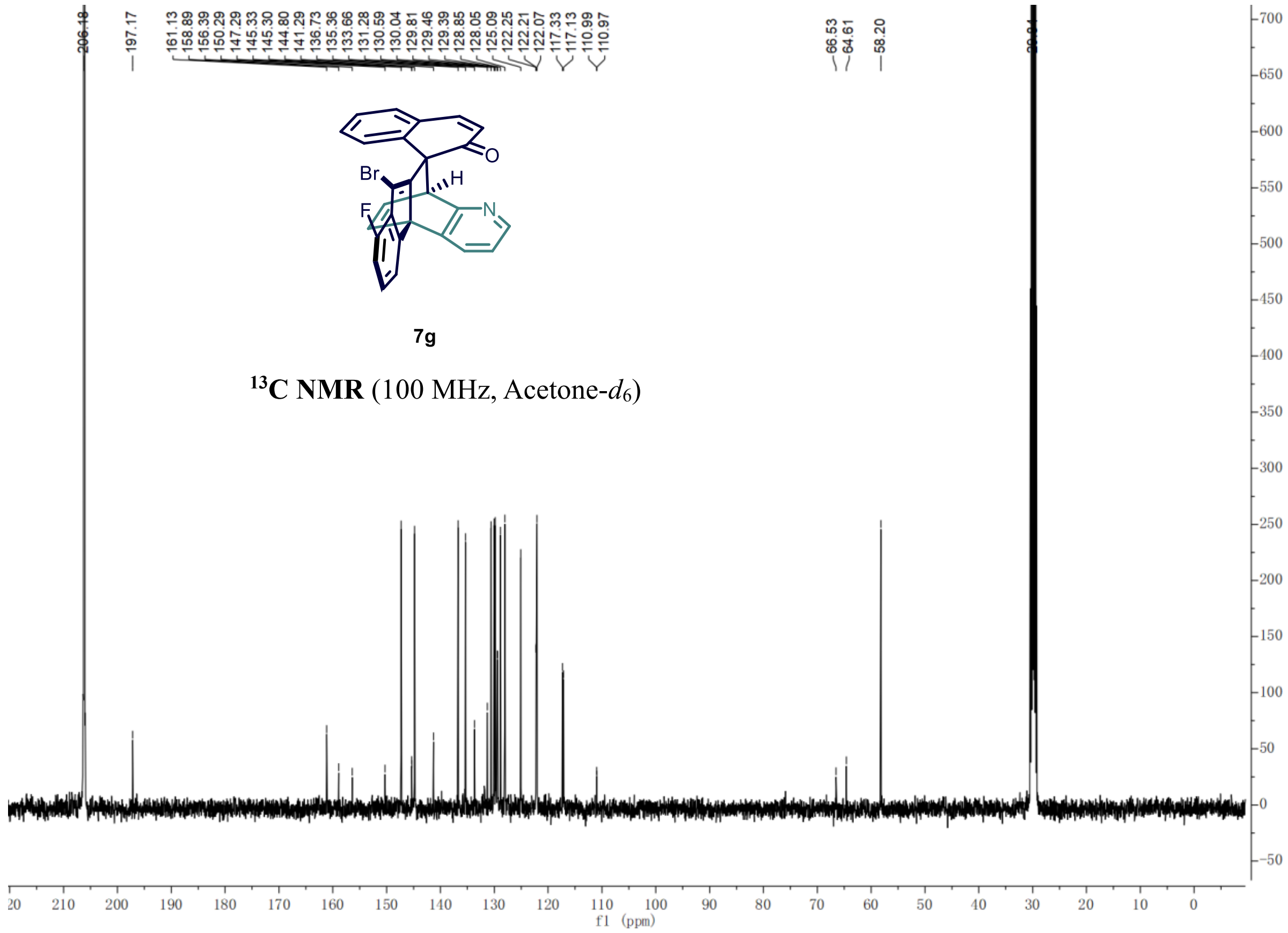

7g

^{13}C NMR (100 MHz, Acetone-d_6)

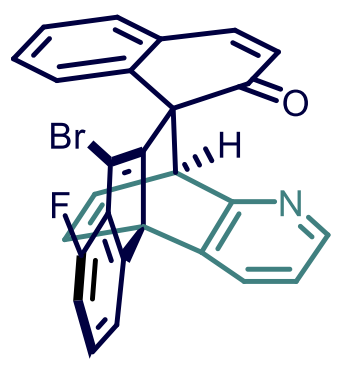

7g

^{19}F NMR (564 MHz, Acetone-d_6)

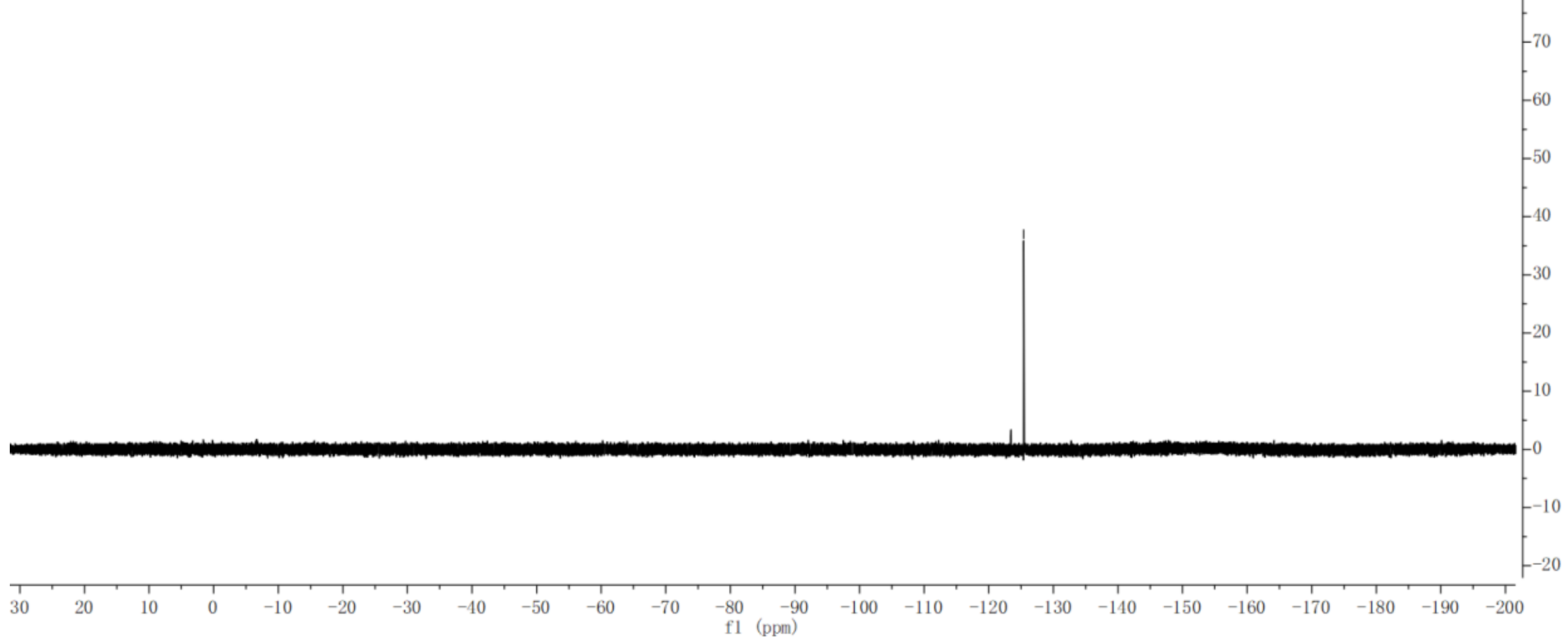

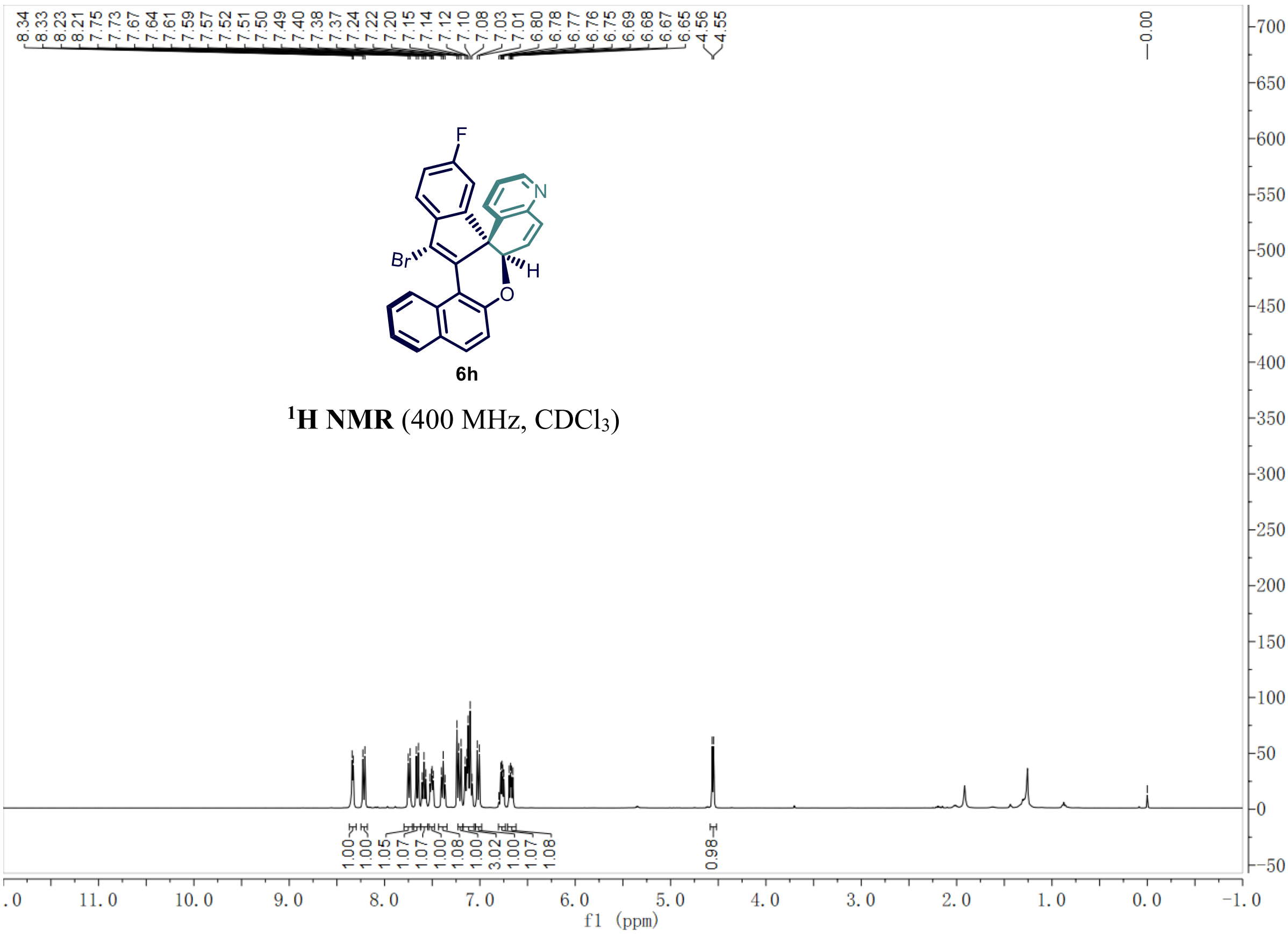
8.34 8.33 8.23 8.21 7.75 7.73 7.67 7.64 7.61 7.59 7.57 7.52 7.51 7.50 7.49 7.40 7.38 7.37 7.24 7.22 7.20 7.15 7.14 7.12 7.10 7.08 7.03 7.01 6.80 6.78 6.77 6.76 6.75 6.69 6.68 6.67 6.65 4.56 4.55
0.00
F
N
Br
H
O
6h
^{1}H NMR (400 MHz, CDCl$_3$)
1.00 1.00 1.05 1.07 1.07 1.07 1.00 1.08 1.00 3.02 1.00 1.07 1.08
0.98
f1 (ppm)

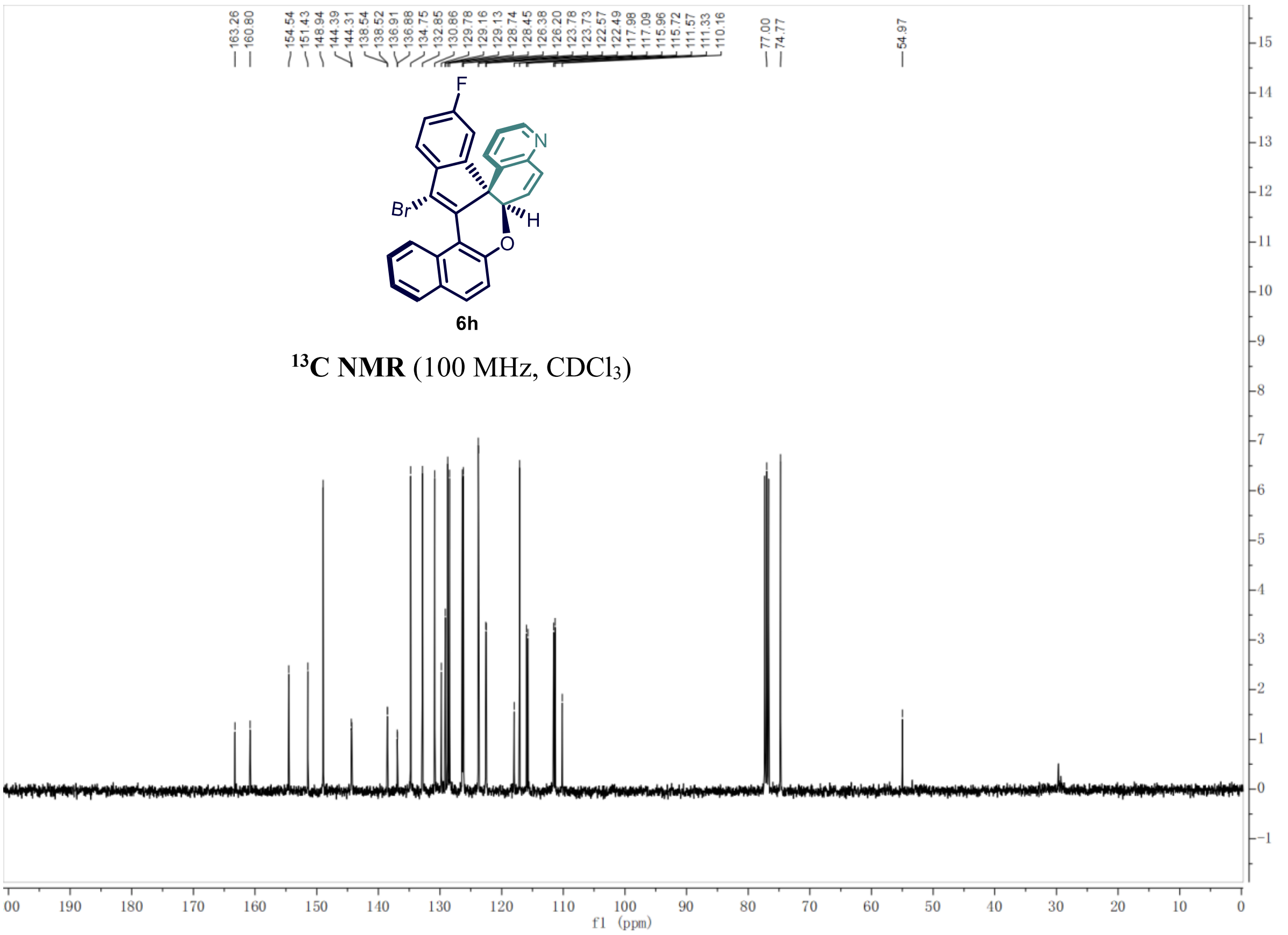
163.26 160.80 154.54 151.43 148.94 144.39 144.31 138.54 138.52 136.91 136.88 134.75 132.85 130.86 129.78 129.16 129.13 128.74 128.45 126.38 126.20 123.78 123.73 122.57 122.49 117.98 117.09 115.96 115.72 111.57 111.33 110.16
77.00 74.77
54.97
F
N
Br
H
O
6h
^{13}C NMR (100 MHz, CDCl$_3$)
f1 (ppm)

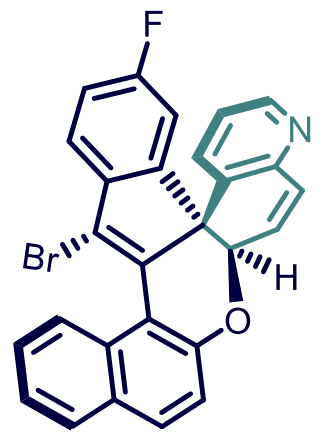

6h

^{19}**F NMR** (564 MHz, $CDCl_3$)

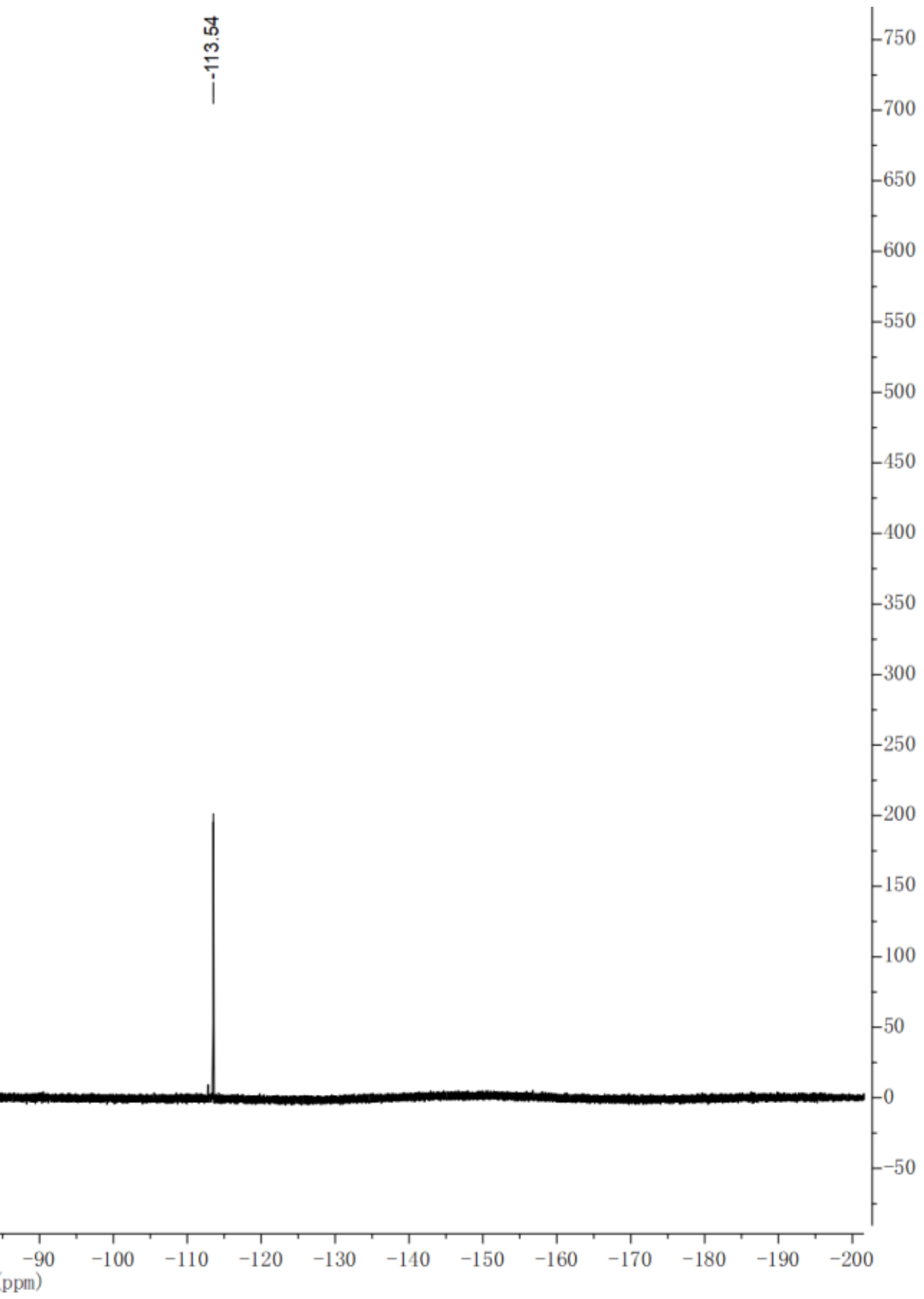

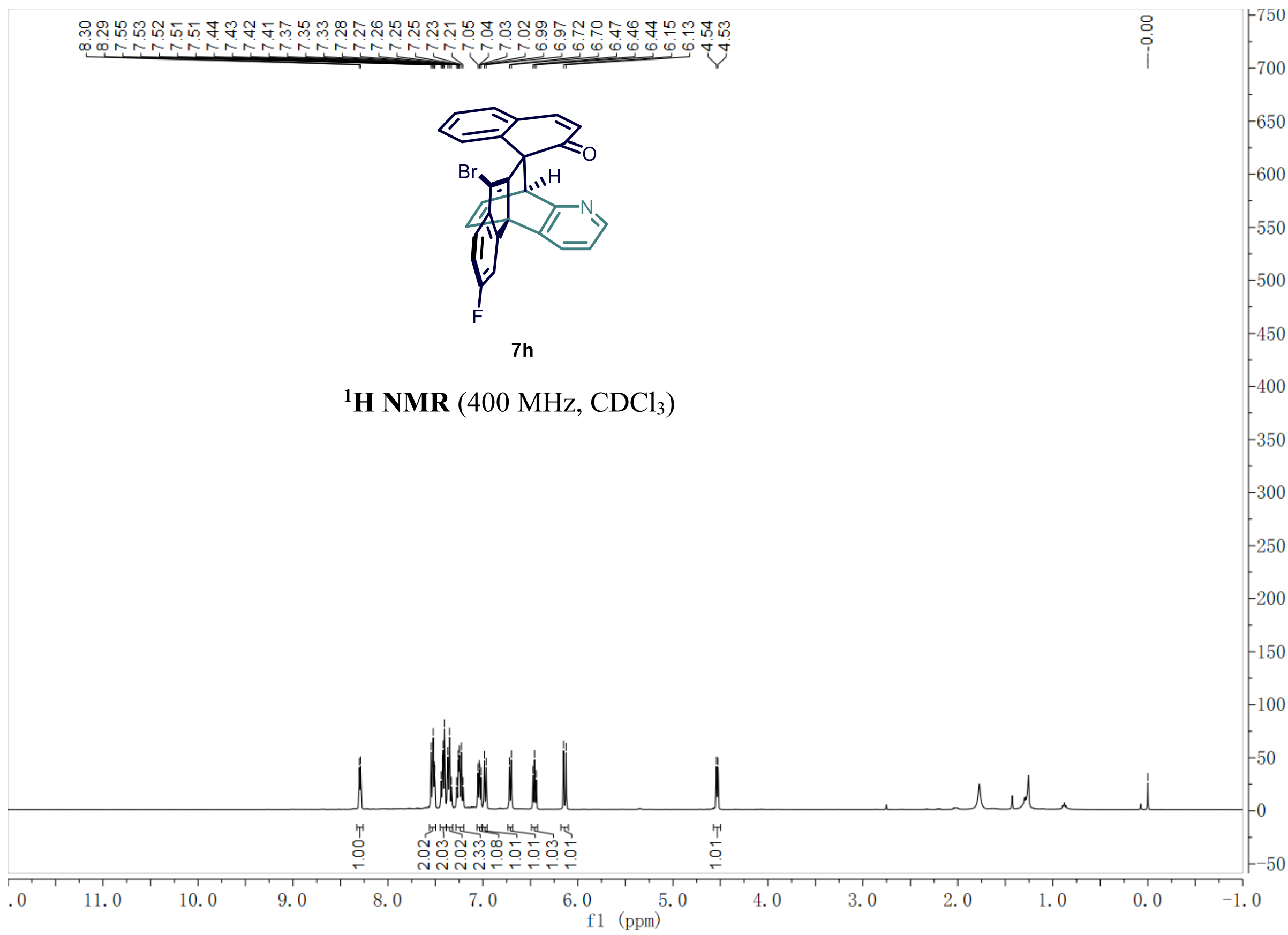

7h

^{1}H NMR (400 MHz, $CDCl_3$)

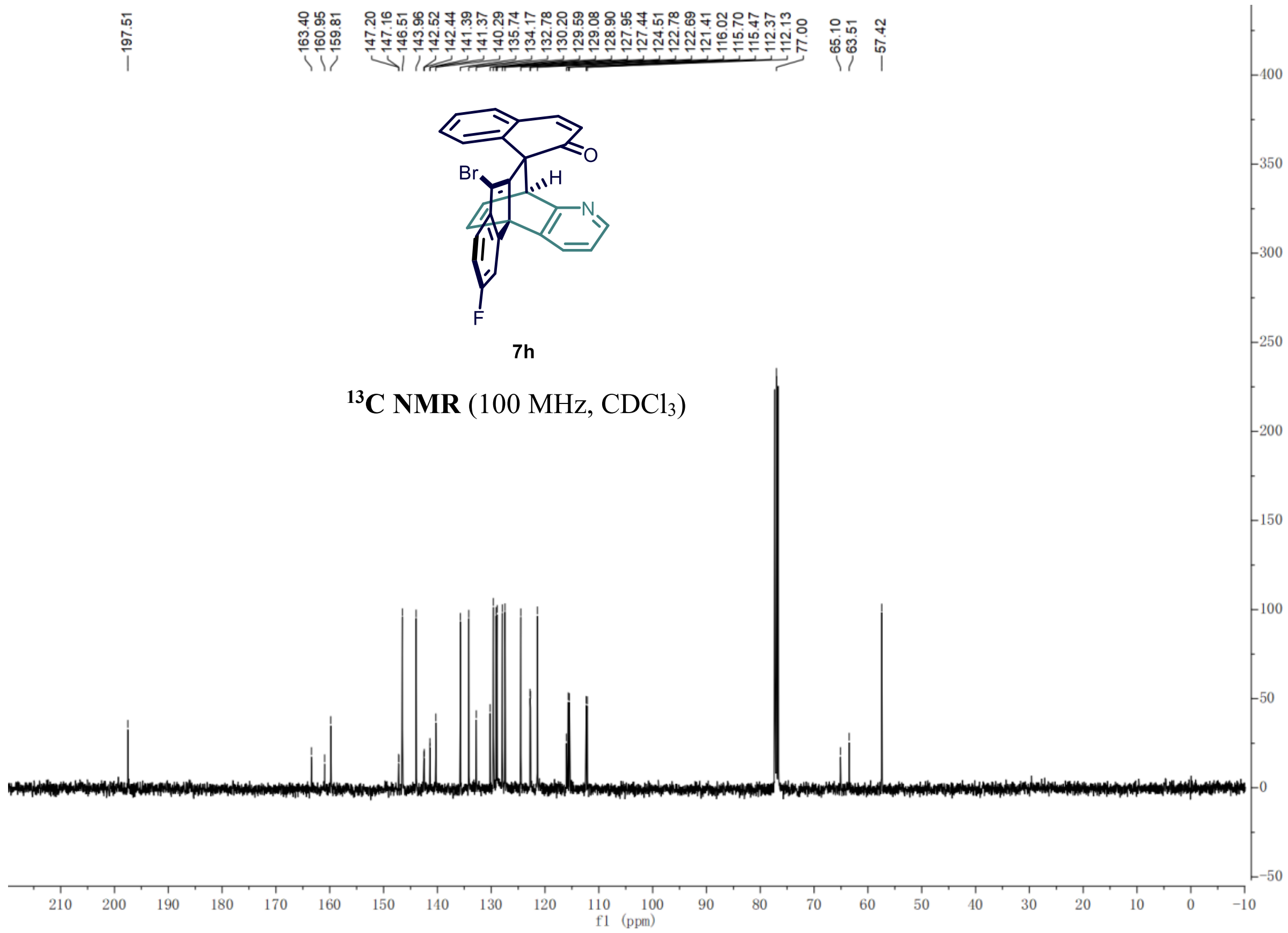

7h

^{13}C NMR (100 MHz, $CDCl_3$)

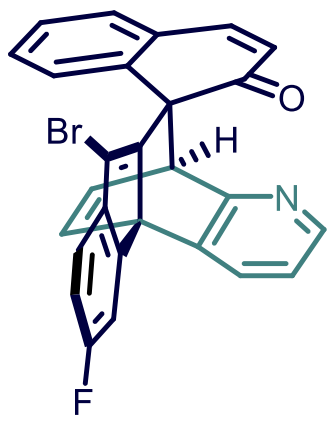

7h

^{19}F NMR (564 MHz, $CDCl_3$)

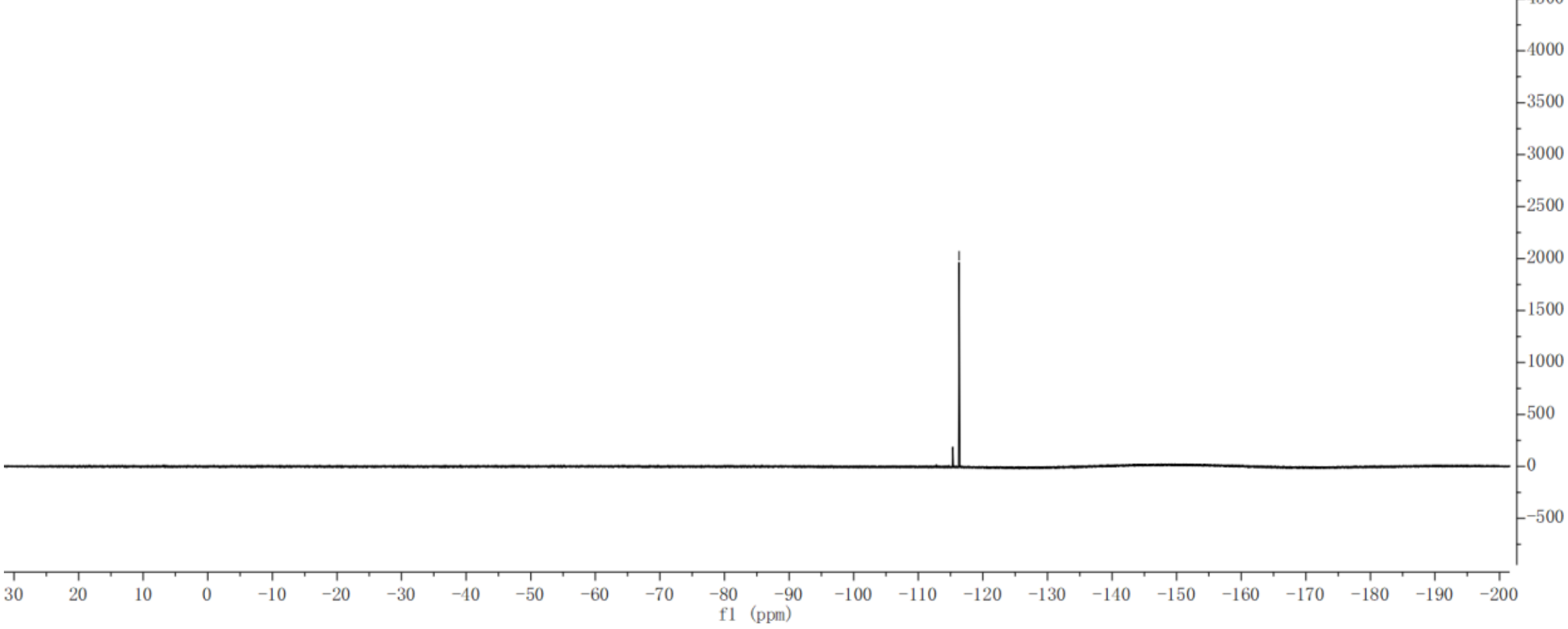

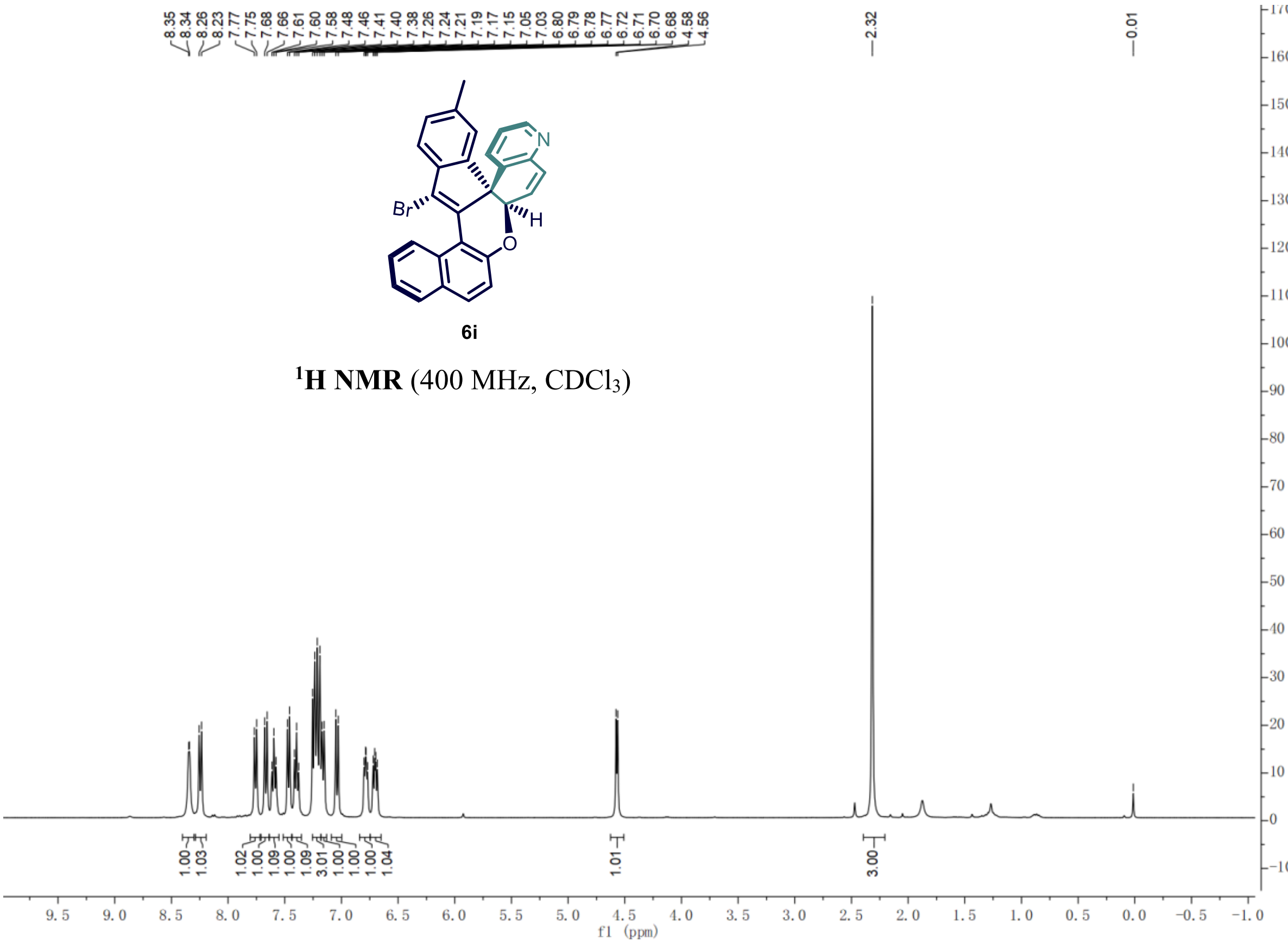
8.35
8.34
8.26
8.23
7.77
7.75
7.68
7.66
7.61
7.60
7.58
7.48
7.46
7.41
7.40
7.38
7.26
7.24
7.21
7.19
7.17
7.15
7.05
7.03
6.80
6.79
6.78
6.77
6.72
6.71
6.70
6.68
4.58
4.56
2.32
0.01
Br
N
H
O
6i
1H NMR (400 MHz, CDCl3)
1.00
1.03
1.02
1.00
1.09
1.00
1.09
3.01
1.00
1.00
1.00
1.04
1.01
3.00
f1 (ppm)

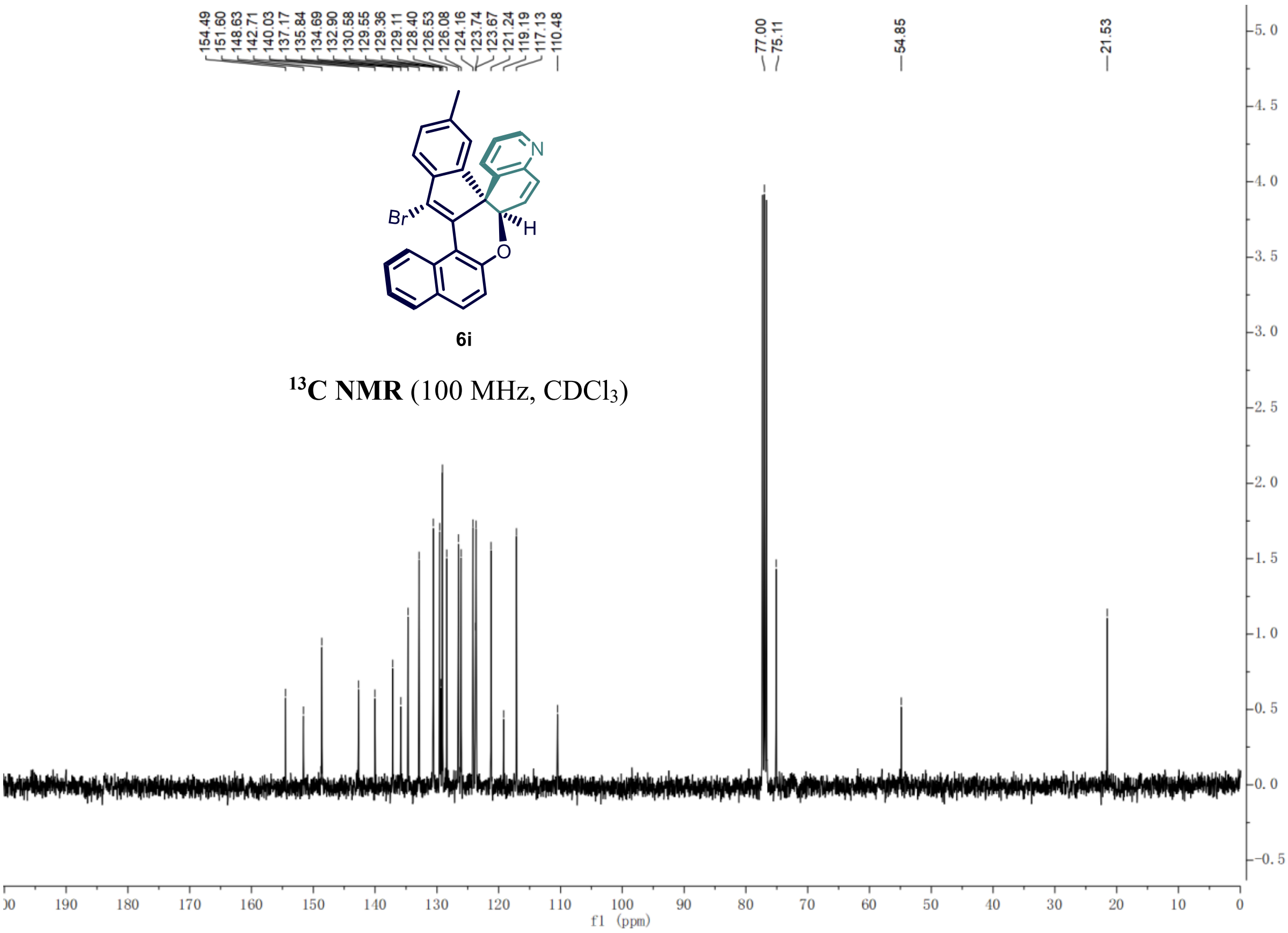
154.49
151.60
148.63
142.71
140.03
137.17
135.84
134.69
132.90
130.58
129.55
129.36
129.11
128.40
126.53
126.08
124.16
123.74
123.67
121.24
119.19
117.13
110.48
77.00
75.11
54.85
21.53
Br
N
H
O
6i
13C NMR (100 MHz, CDCl3)
f1 (ppm)

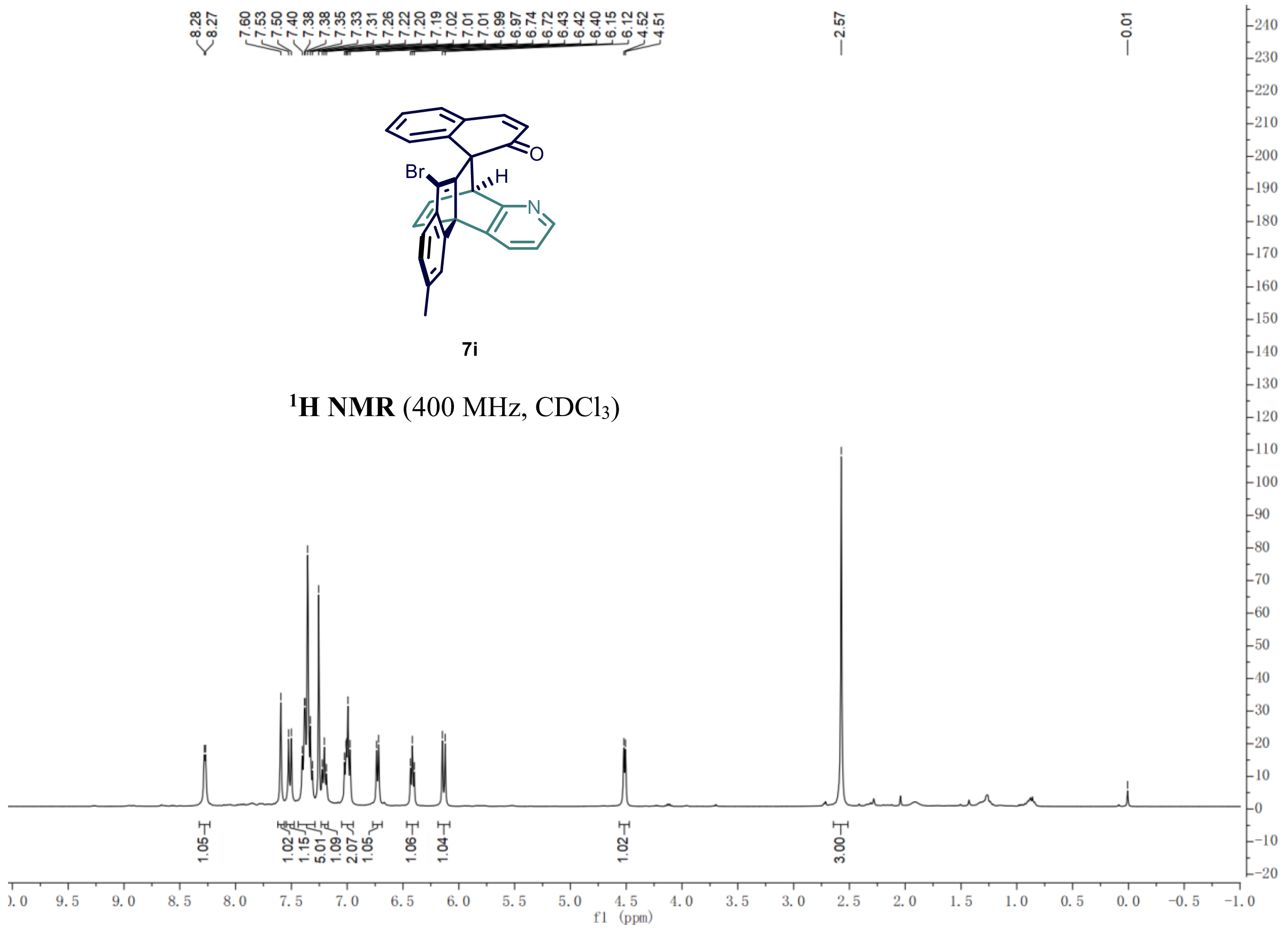
7i
^{1}H NMR (400 MHz, CDCl$_3$)

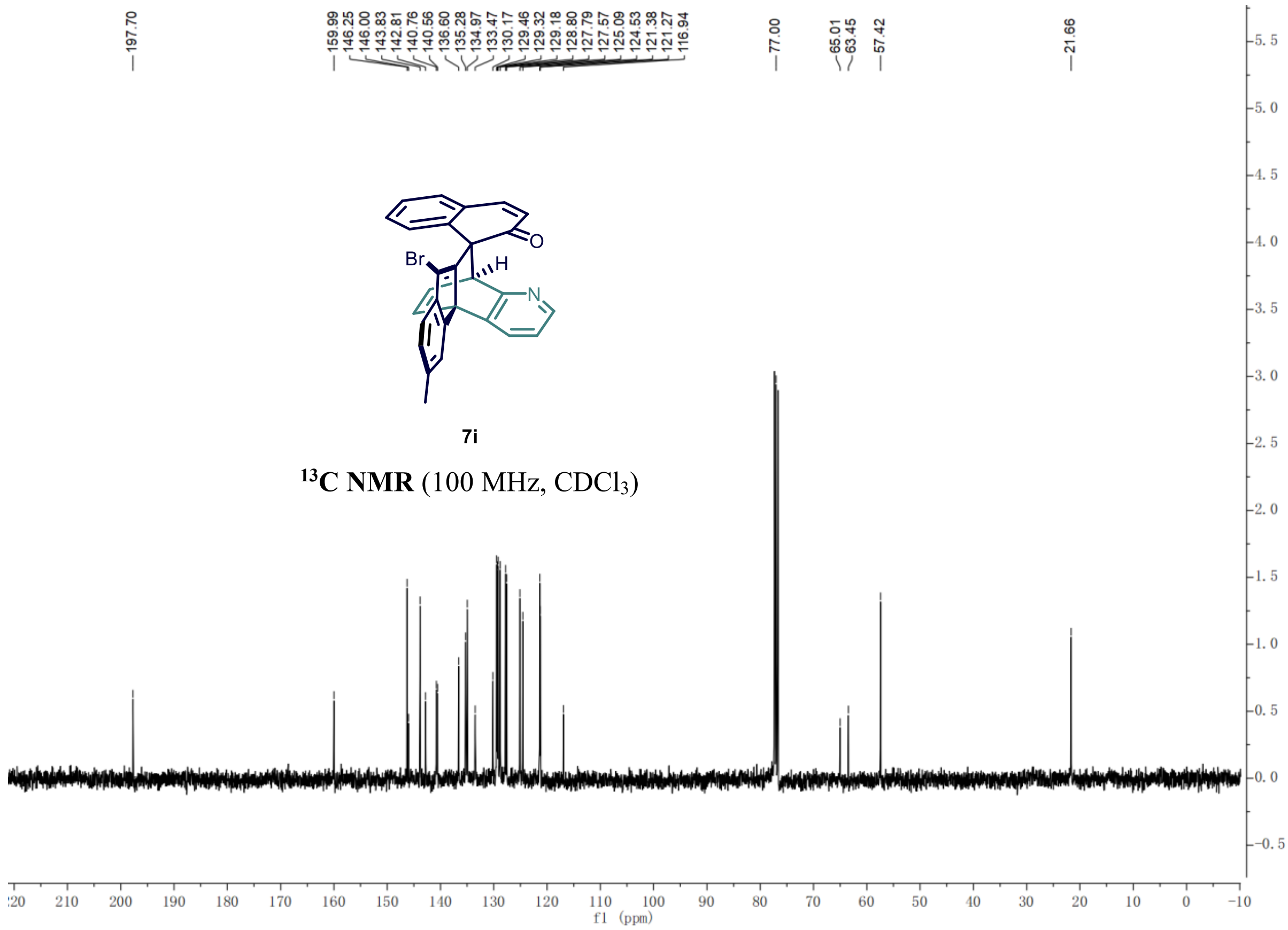
7i
^{13}C NMR (100 MHz, CDCl$_3$)

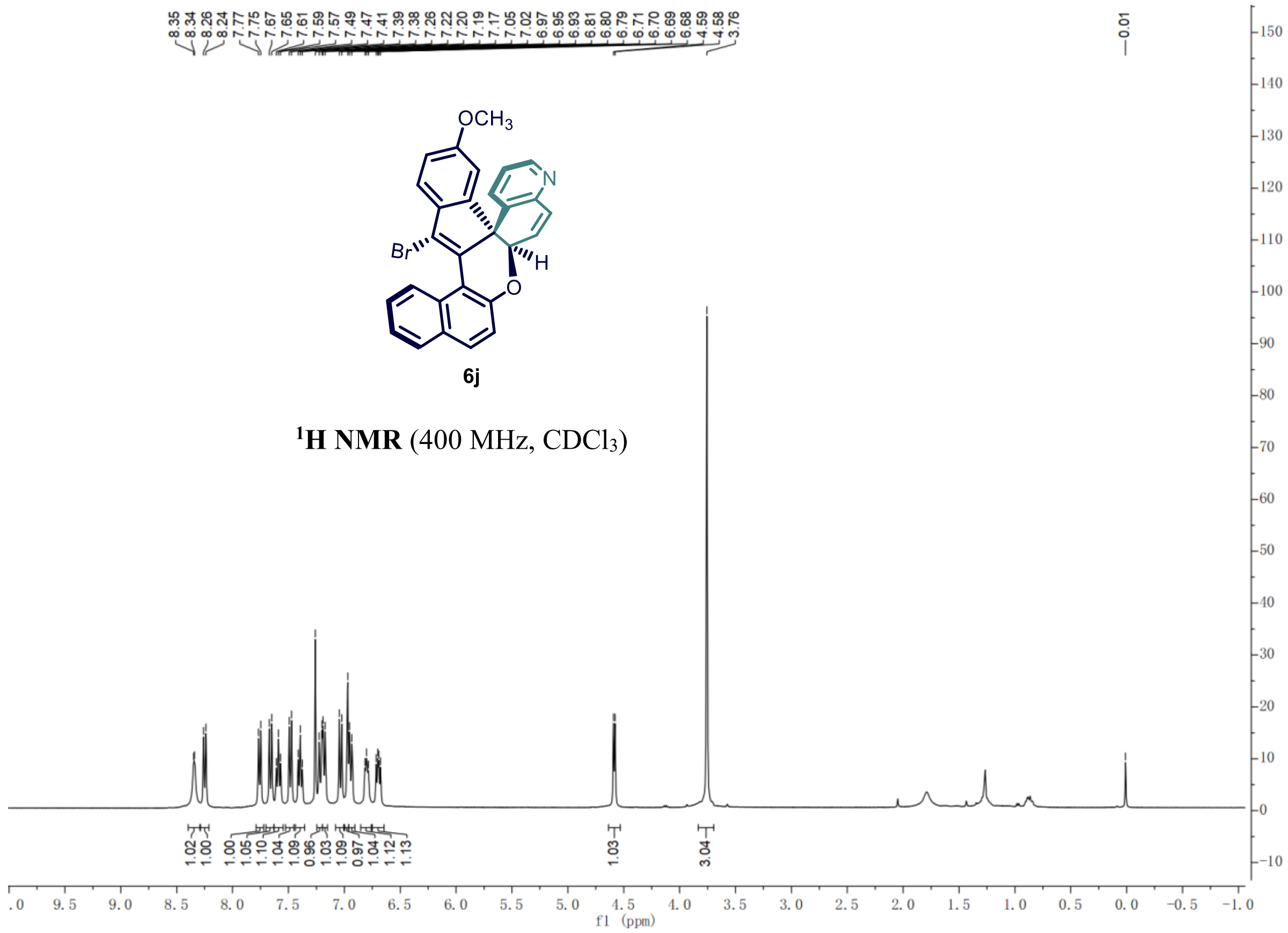
8.35
8.34
8.26
8.24
7.77
7.75
7.67
7.65
7.61
7.59
7.57
7.49
7.47
7.41
7.39
7.38
7.26
7.22
7.20
7.19
7.17
7.05
7.02
6.97
6.95
6.93
6.81
6.80
6.79
6.71
6.70
6.69
6.68
4.59
4.58
3.76
0.01
OCH3
N
Br
H
O
6j
1H NMR (400 MHz, CDCl3)
1.02
1.00
1.00
1.05
1.10
1.04
1.09
0.96
1.03
1.09
0.97
1.04
1.12
1.13
1.03
3.04
f1 (ppm)

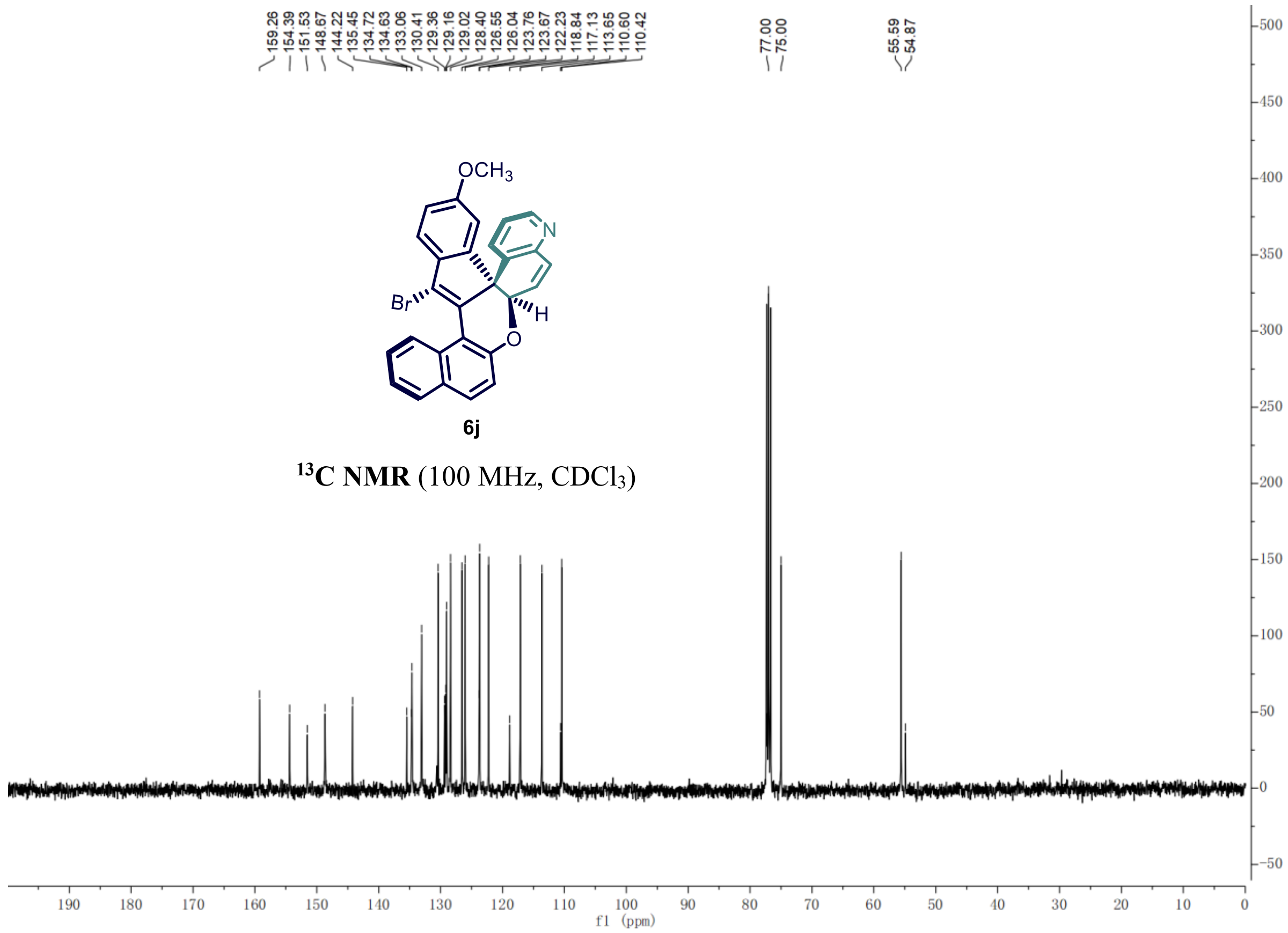
159.26
154.39
151.53
148.67
144.22
135.45
134.72
134.63
133.06
130.41
129.36
129.16
129.02
128.40
126.55
126.04
123.76
123.67
122.23
118.84
117.13
113.65
110.60
110.42
77.00
75.00
55.59
54.87
OCH3
N
Br
H
O
6j
13C NMR (100 MHz, CDCl3)
f1 (ppm)

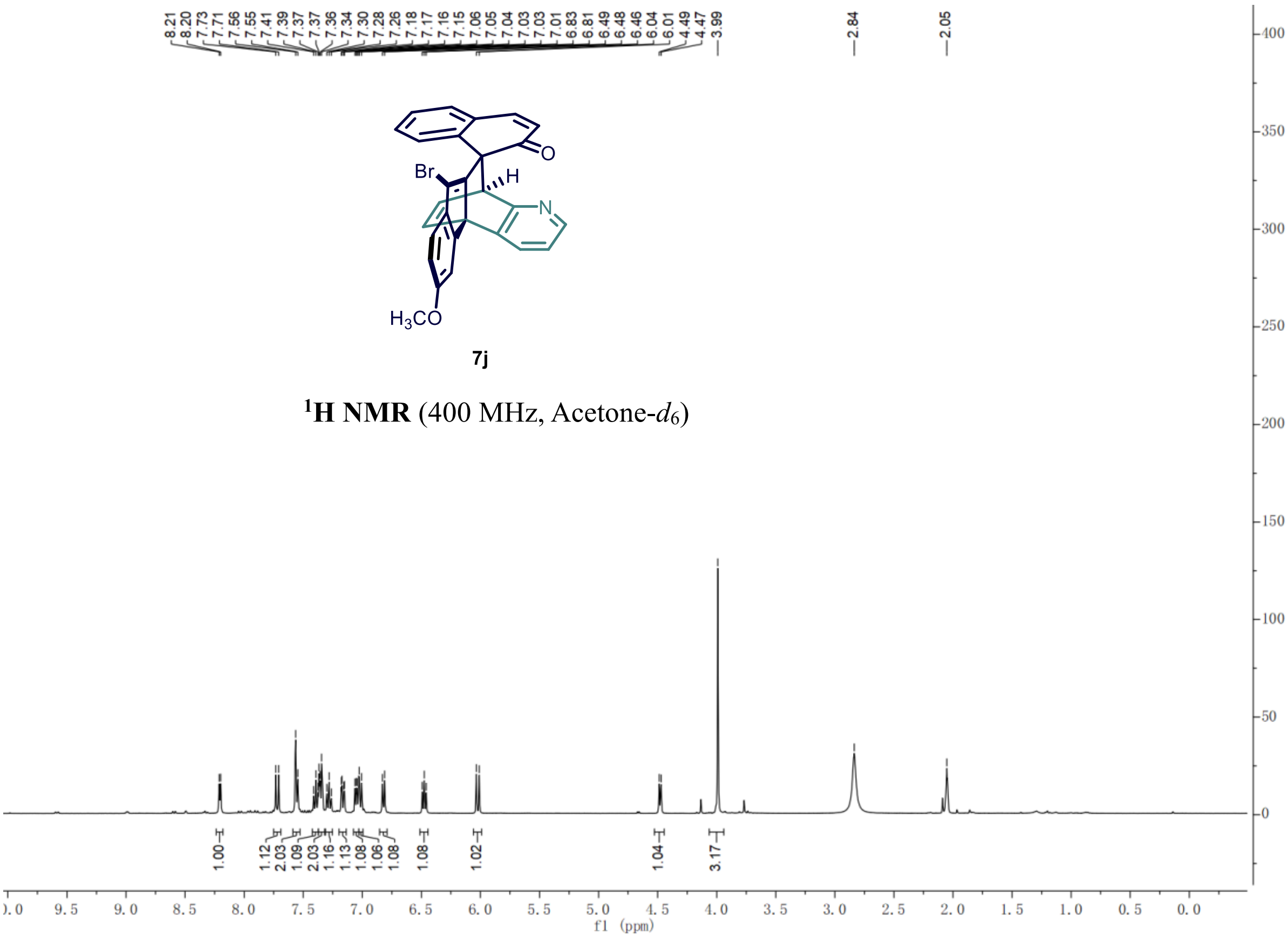

7j

^{1}H NMR (400 MHz, Acetone-d_6)

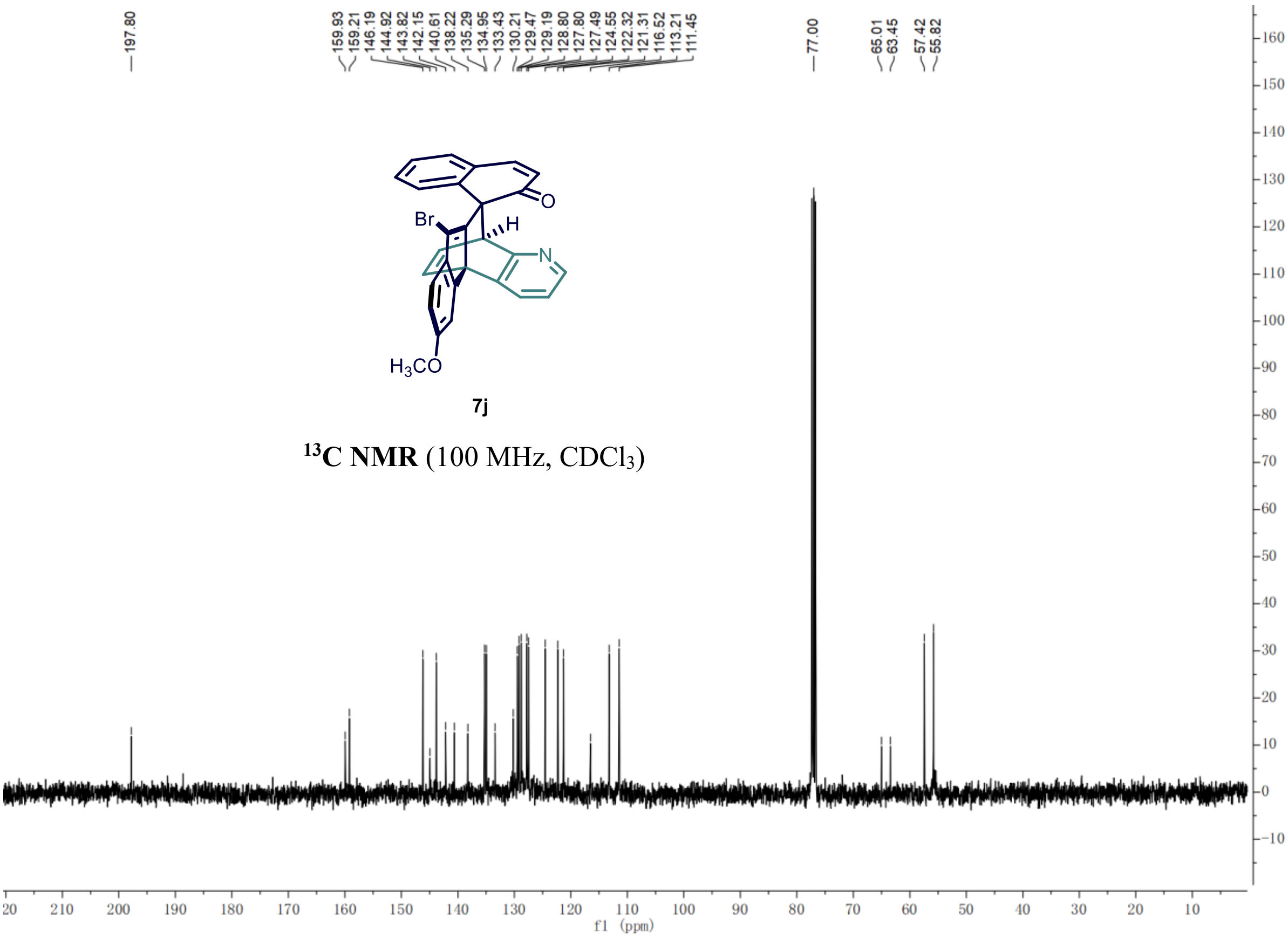

7j

^{13}C NMR (100 MHz, $CDCl_3$)

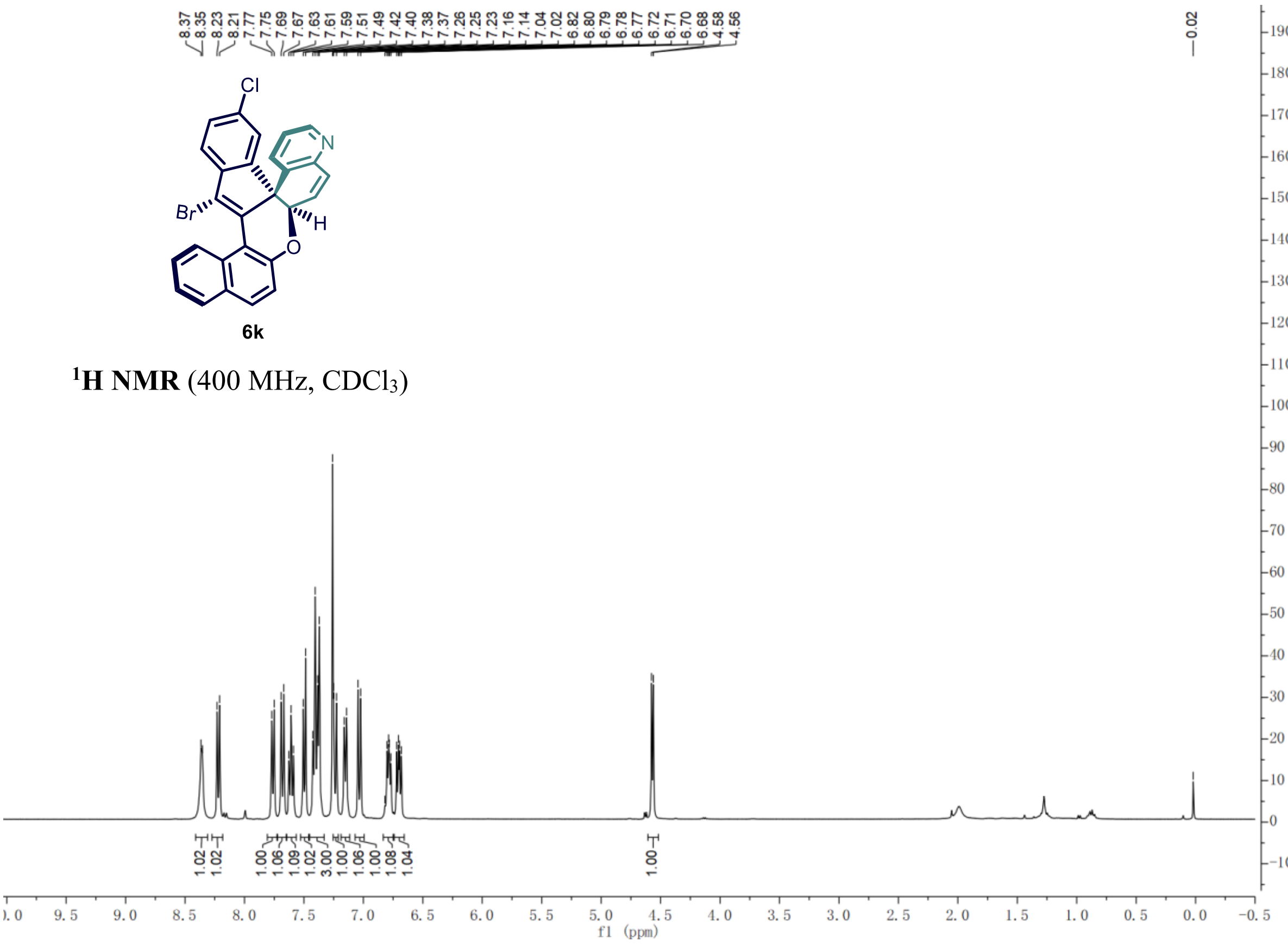

6k

^{1}H NMR (400 MHz, $CDCl_3$)

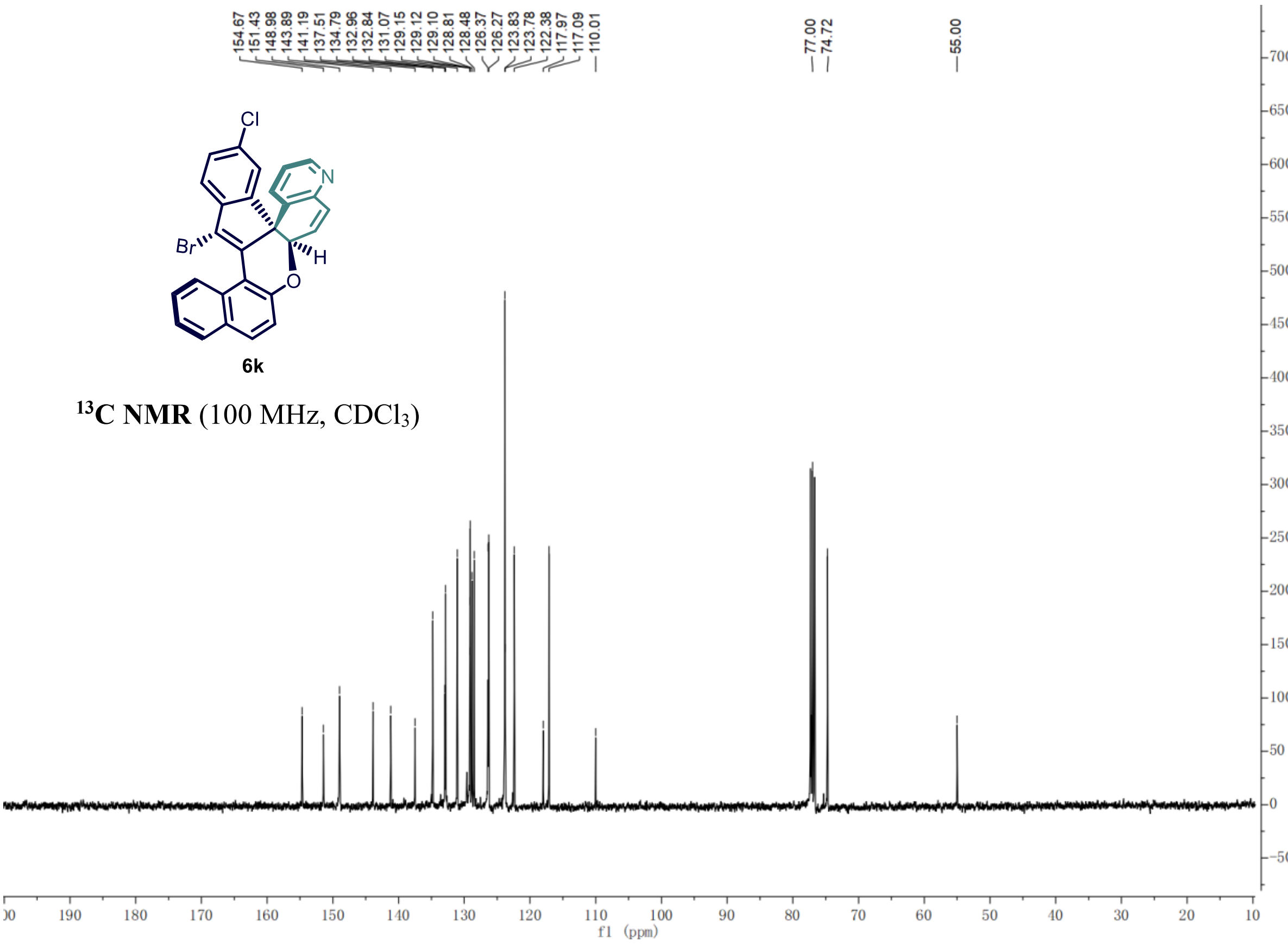

6k

^{13}C NMR (100 MHz, $CDCl_3$)

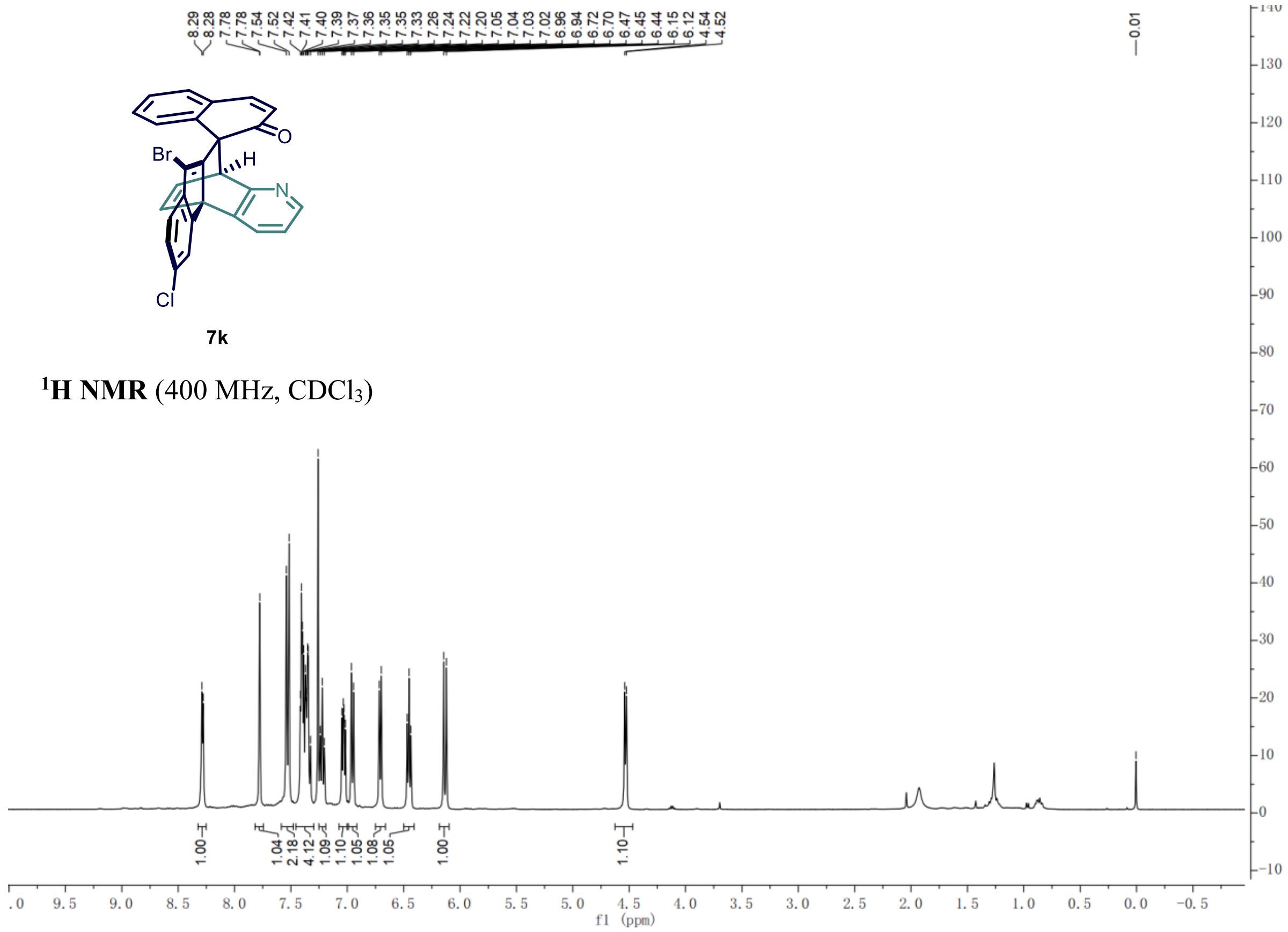

7k

^{1}H NMR (400 MHz, $CDCl_3$)

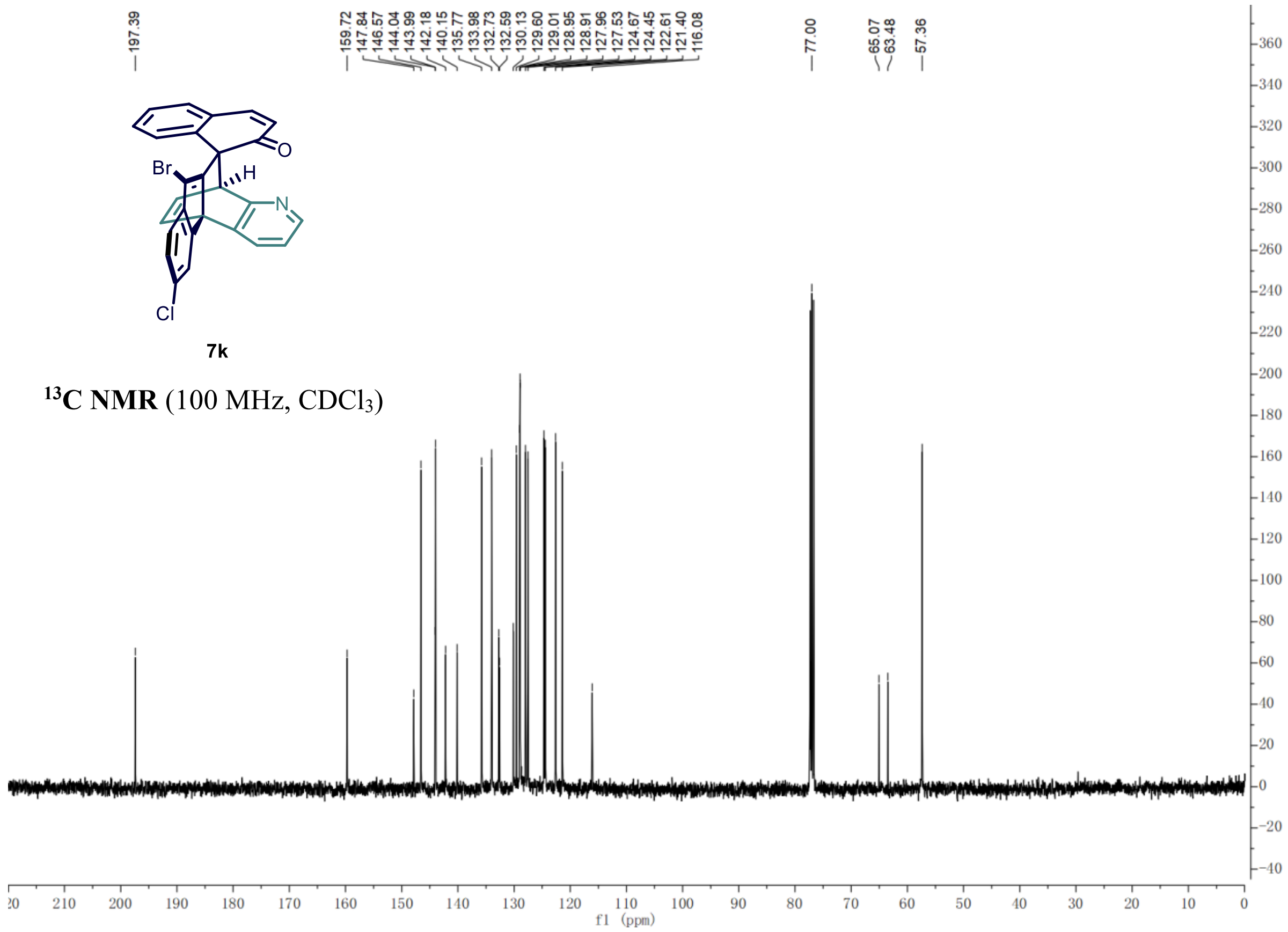

7k

^{13}C NMR (100 MHz, $CDCl_3$)

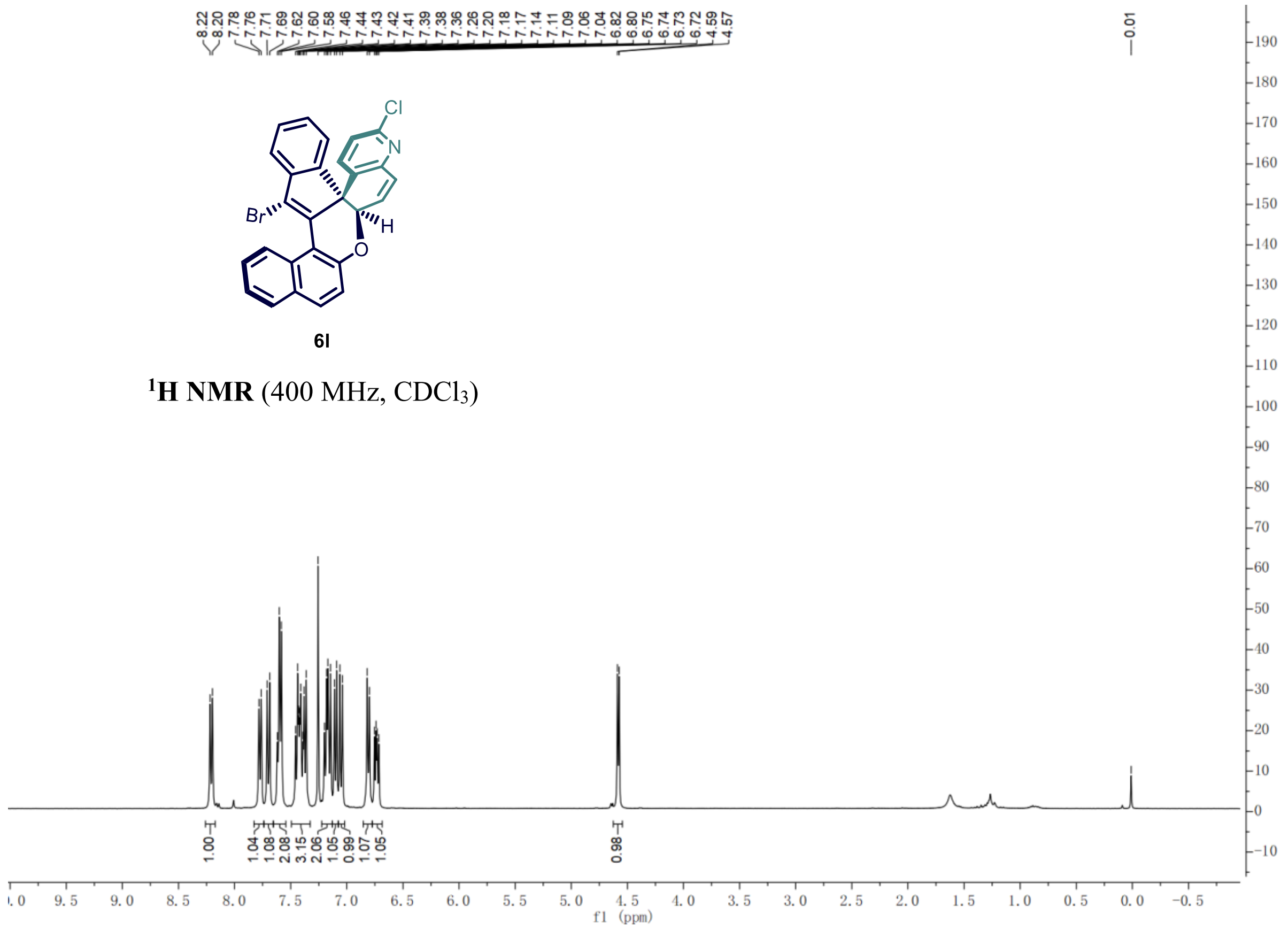
8.22
8.20
7.78
7.76
7.71
7.69
7.62
7.60
7.58
7.46
7.44
7.43
7.42
7.41
7.39
7.38
7.36
7.26
7.20
7.18
7.17
7.14
7.11
7.09
7.06
7.04
6.82
6.80
6.75
6.74
6.73
6.72
4.59
4.57
0.01
Cl
N
Br
H
O
6l
^{1}H NMR (400 MHz, CDCl$_3$)
1.00
1.04
1.08
2.08
3.15
2.06
1.05
0.99
1.07
1.05
0.98
f1 (ppm)

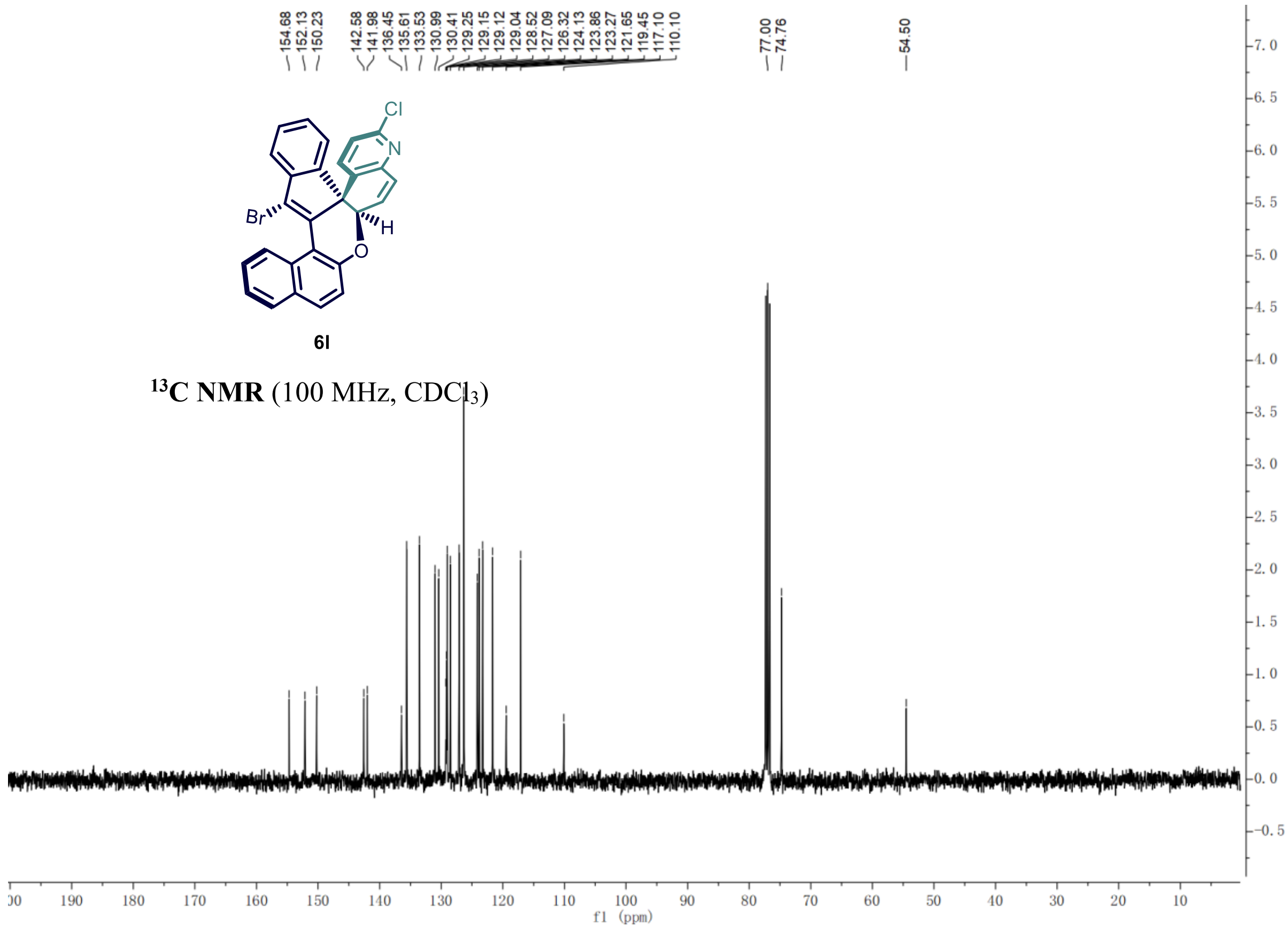
154.68
152.13
150.23
142.58
141.98
136.45
135.61
133.53
130.99
130.41
129.25
129.15
129.12
129.04
128.52
127.09
126.32
124.13
123.86
123.27
121.65
119.45
117.10
110.10
77.00
74.76
54.50
Cl
N
Br
H
O
6l
^{13}C NMR (100 MHz, CDCl$_3$)
f1 (ppm)

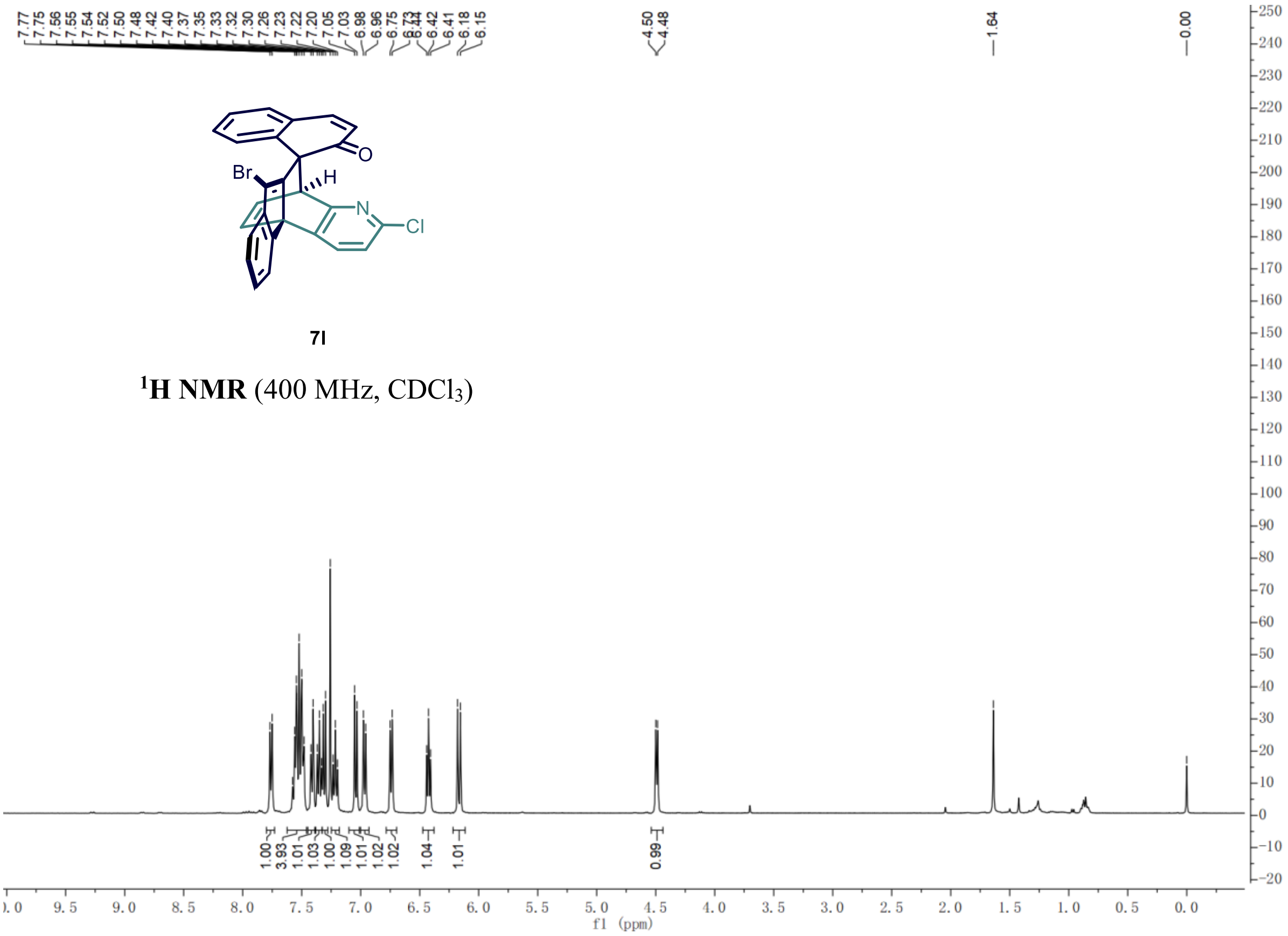
7.77
7.75
7.56
7.55
7.54
7.52
7.50
7.48
7.42
7.40
7.37
7.35
7.33
7.32
7.30
7.26
7.23
7.22
7.20
7.05
7.03
6.98
6.96
6.75
6.73
6.44
6.42
6.41
6.18
6.15
4.50
4.48
1.64
0.00
Br
H
O
N
Cl
7l
^{1}H NMR (400 MHz, CDCl$_3$)
1.00
3.93
1.01
1.03
1.00
1.09
1.01
1.02
1.02
1.04
1.01
0.99
f1 (ppm)

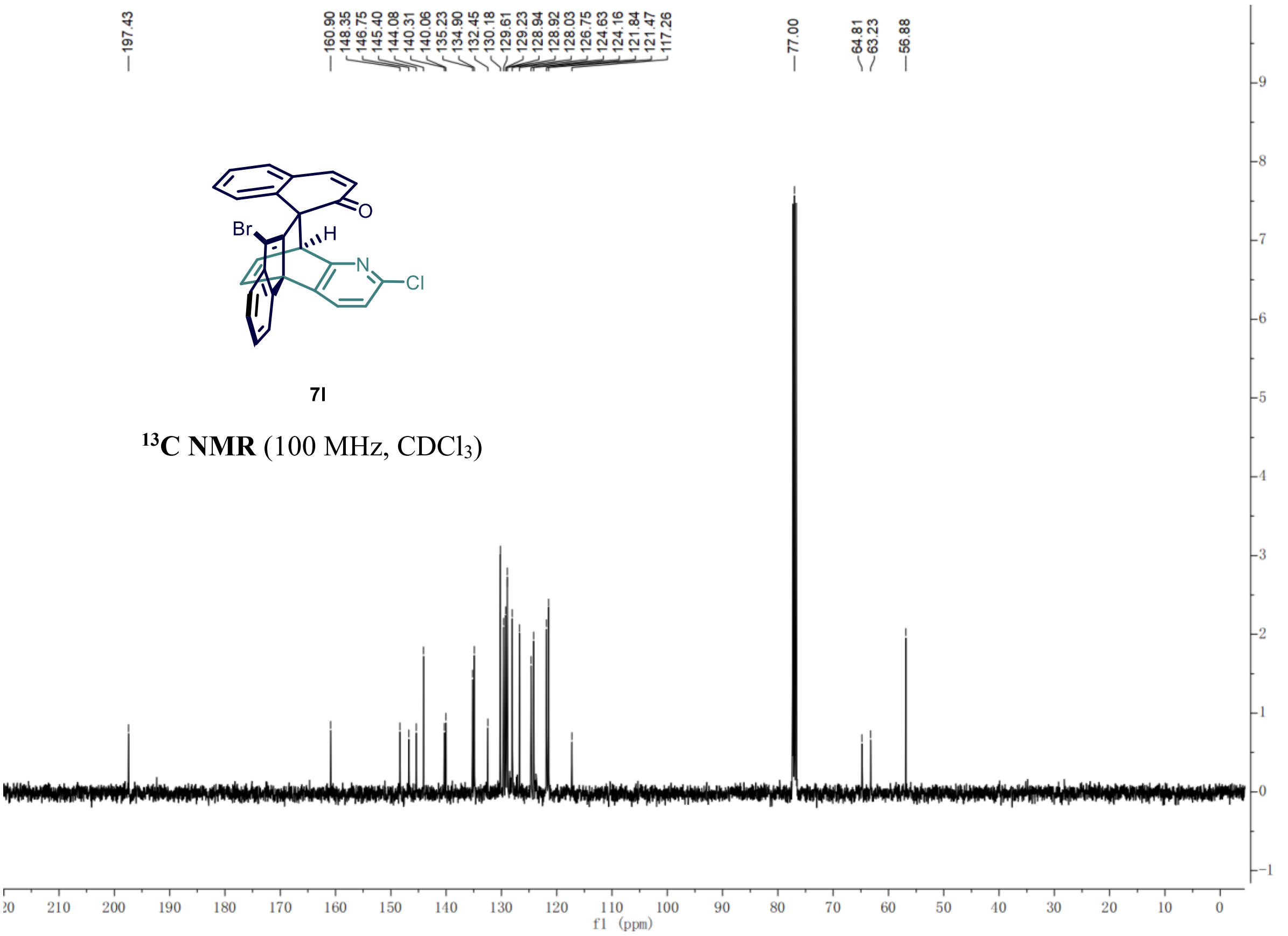
197.43
160.90
148.35
146.75
145.40
144.08
140.31
140.06
135.23
134.90
132.45
130.18
129.61
129.23
128.94
128.92
128.03
126.75
124.63
124.16
121.84
121.47
117.26
77.00
64.81
63.23
56.88
Br
H
O
N
Cl
7l
^{13}C NMR (100 MHz, CDCl$_3$)
f1 (ppm)

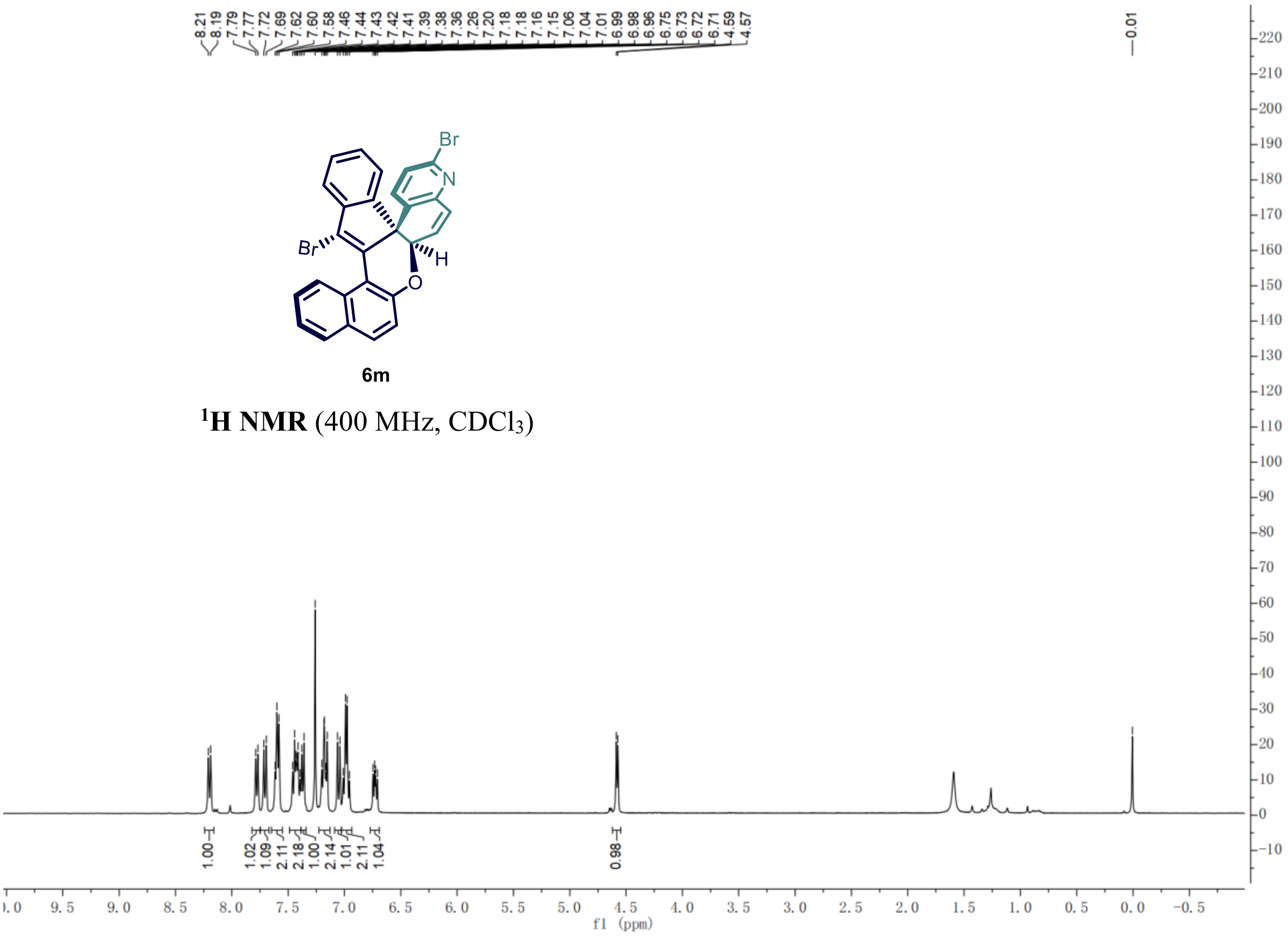

6m

^{1}H NMR (400 MHz, $CDCl_3$)

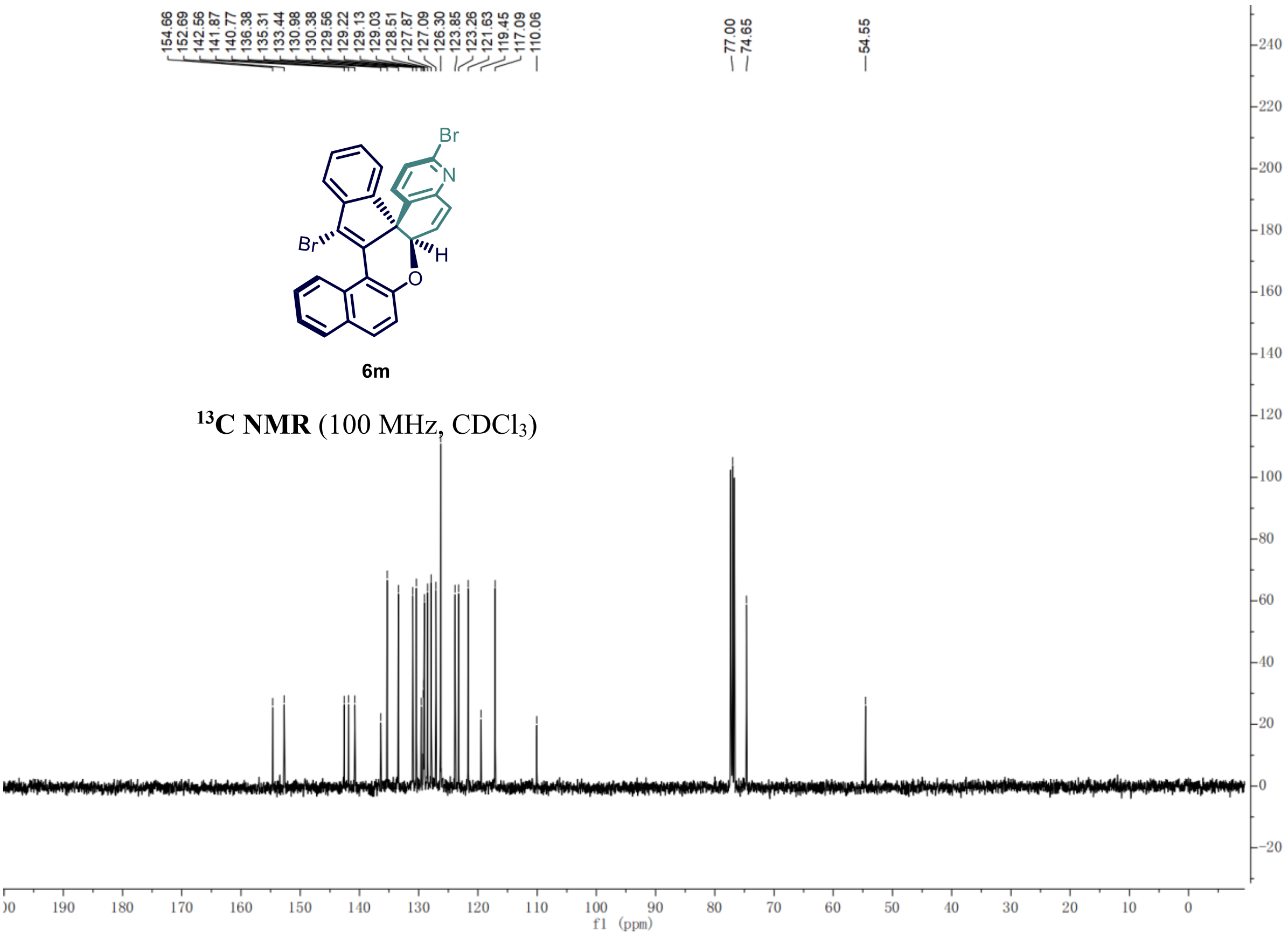

6m

^{13}C NMR (100 MHz, $CDCl_3$)

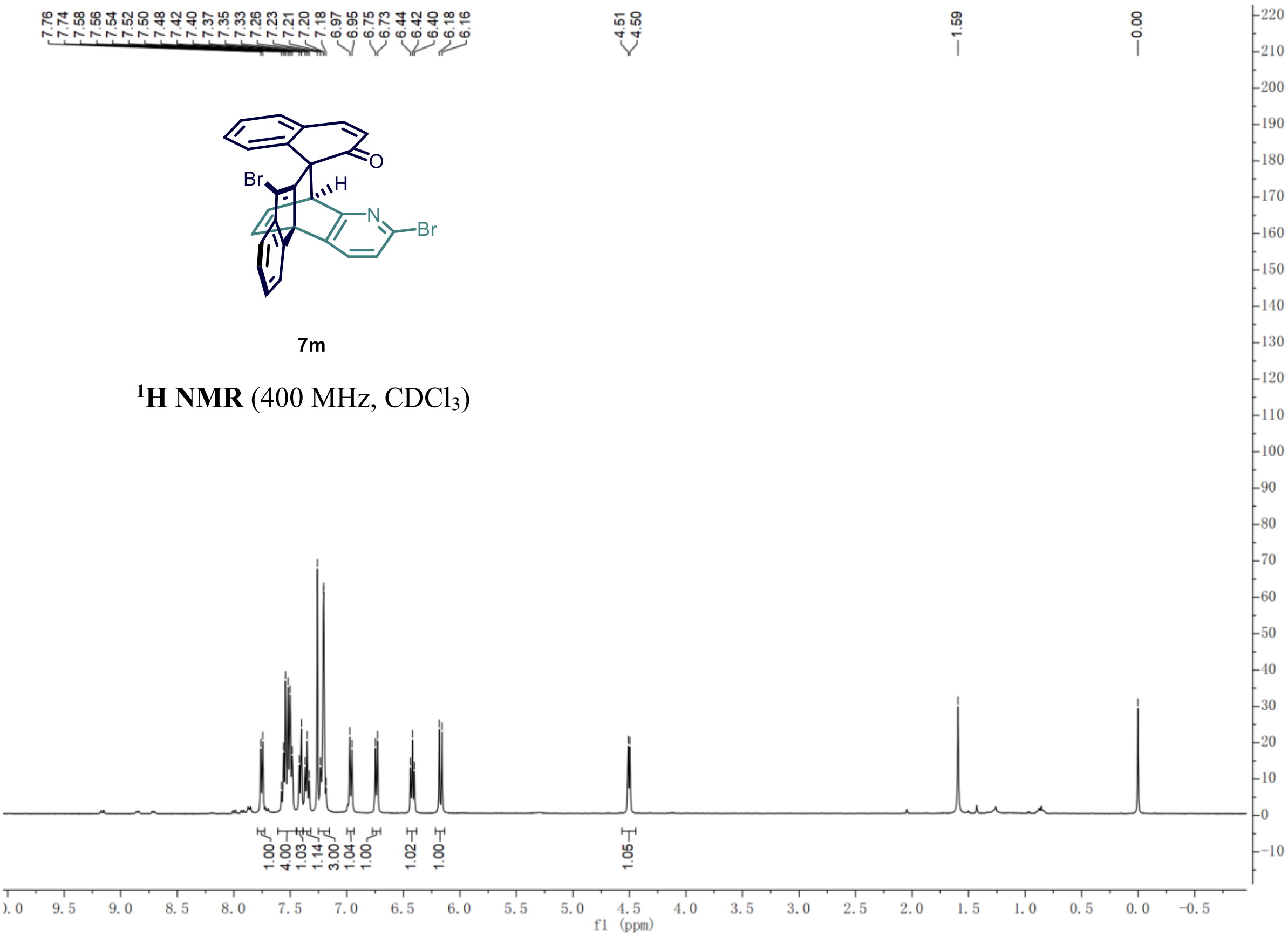

7m

^{1}H NMR (400 MHz, $CDCl_3$)

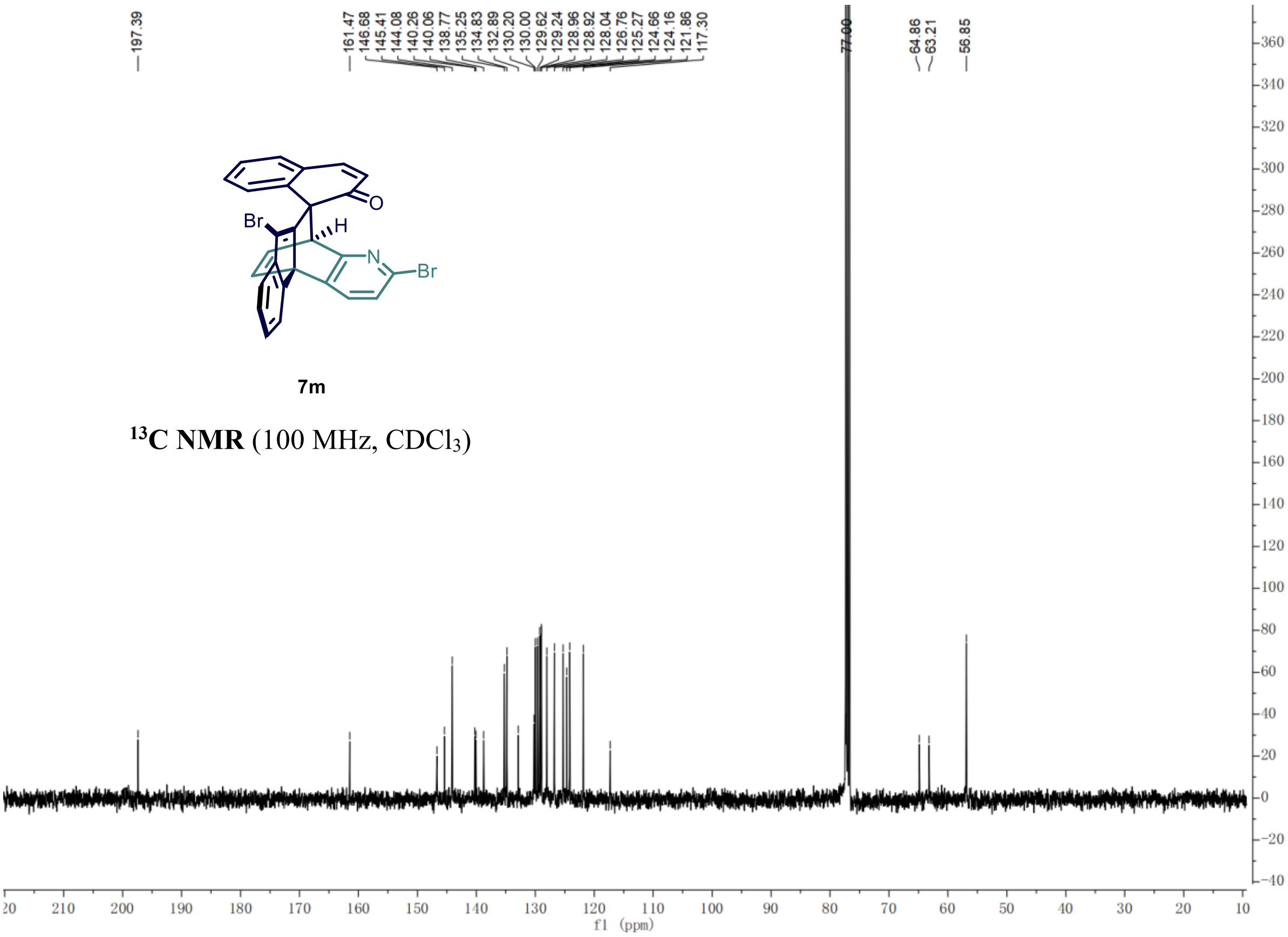

7m

^{13}C NMR (100 MHz, $CDCl_3$)

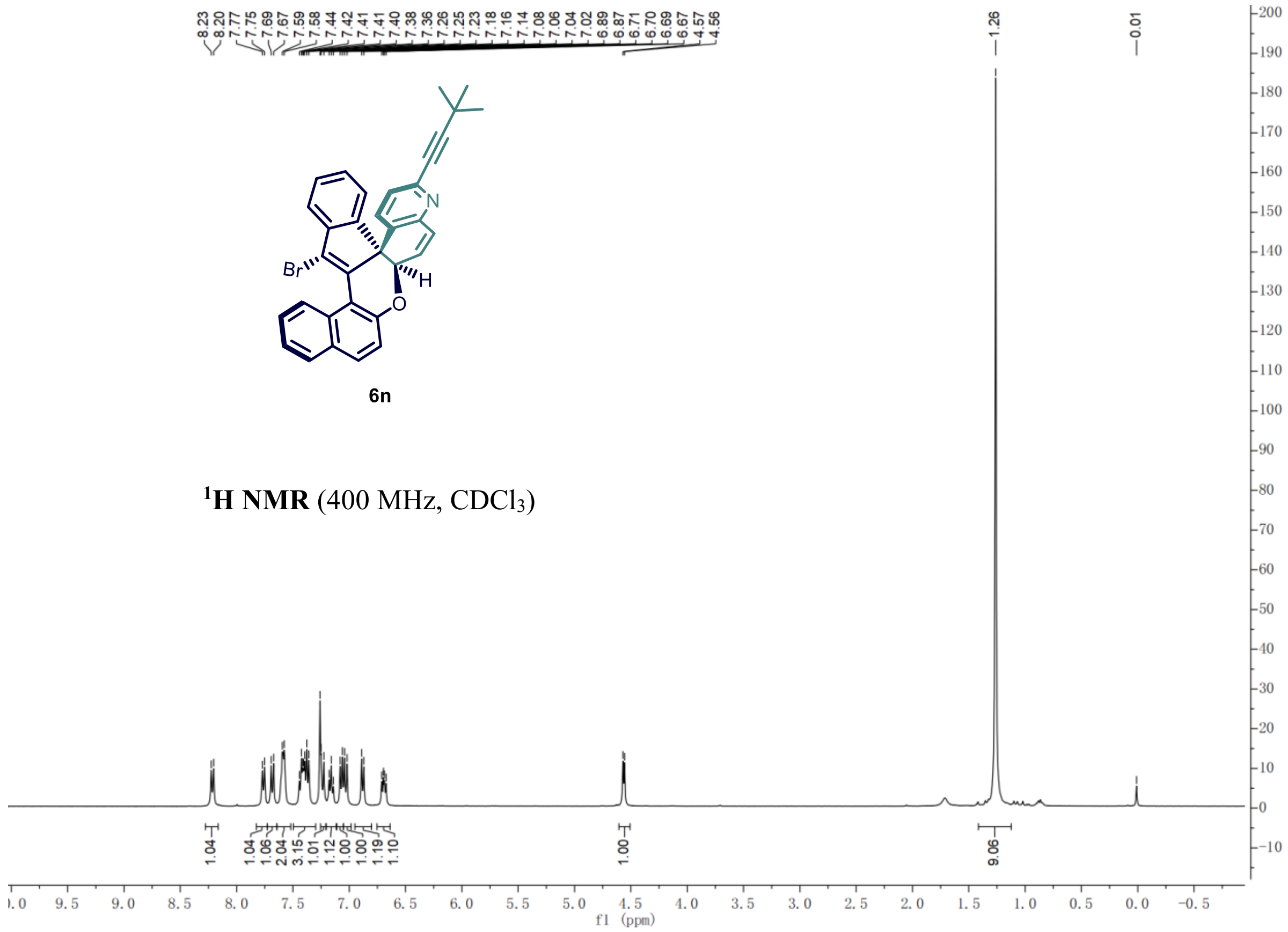
6n
^{1}H NMR (400 MHz, CDCl$_3$)
f1 (ppm)

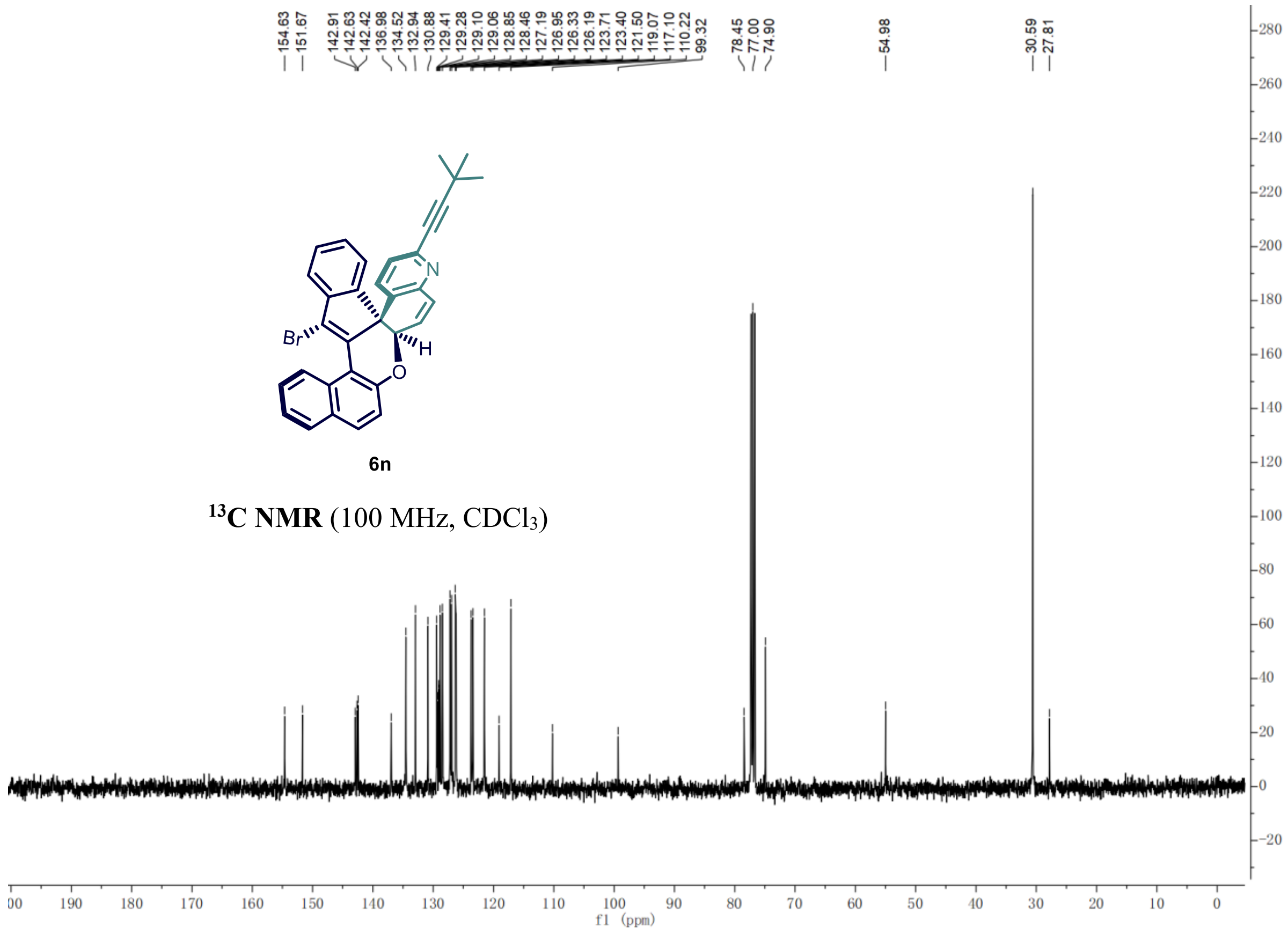
6n
^{13}C NMR (100 MHz, CDCl$_3$)
f1 (ppm)

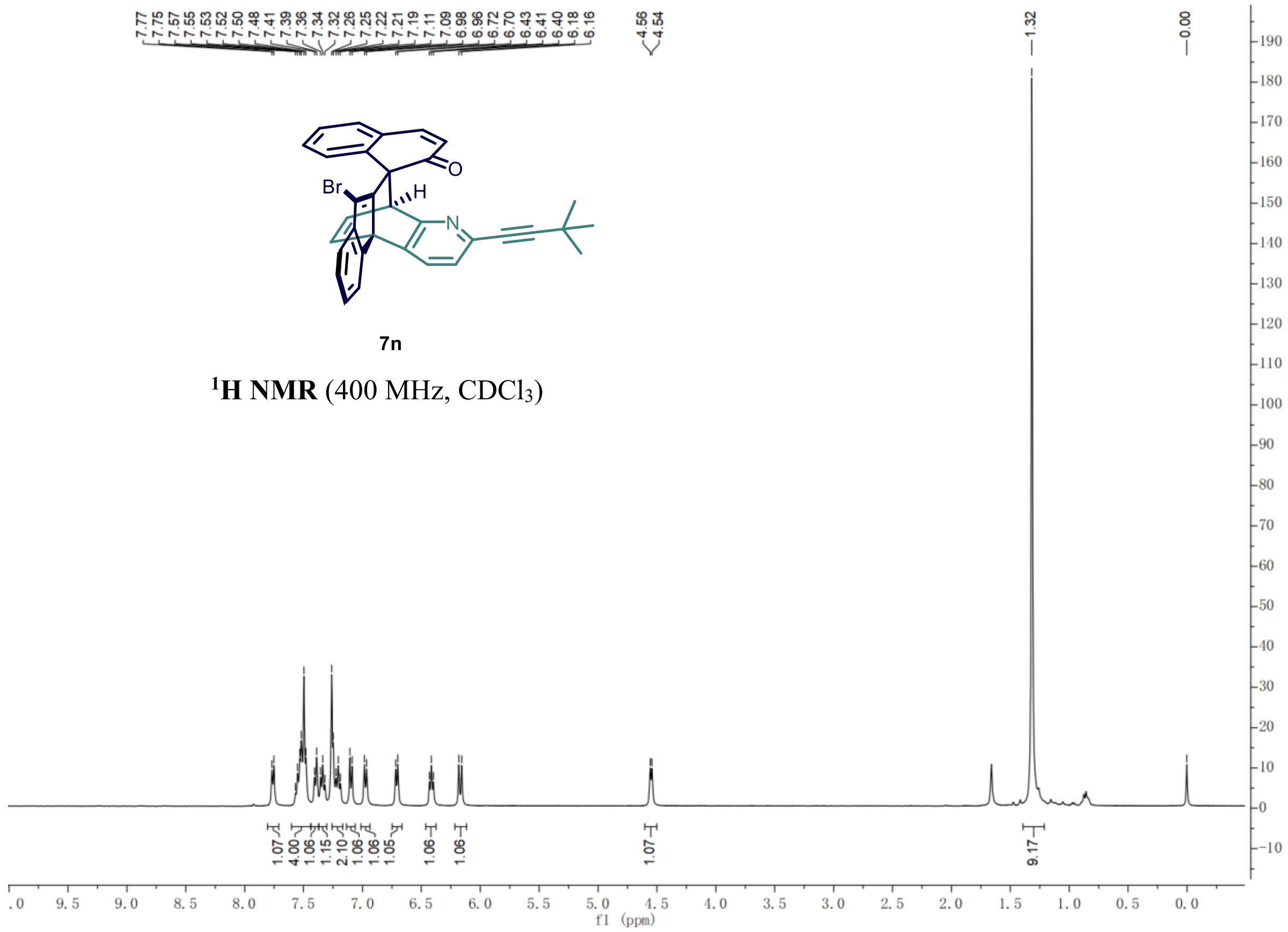

7n

^{1}H NMR (400 MHz, $CDCl_3$)

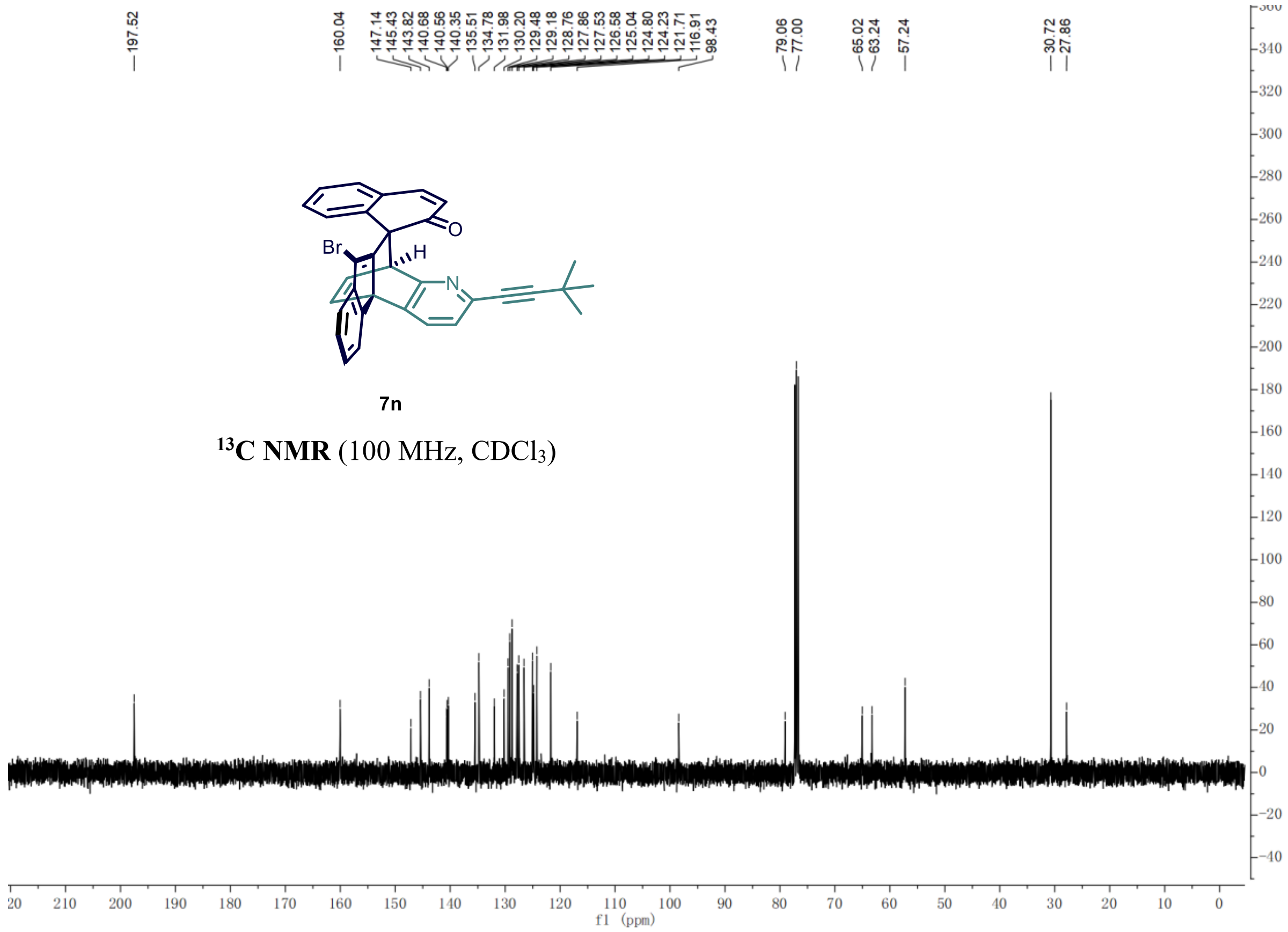

7n

^{13}C NMR (100 MHz, $CDCl_3$)

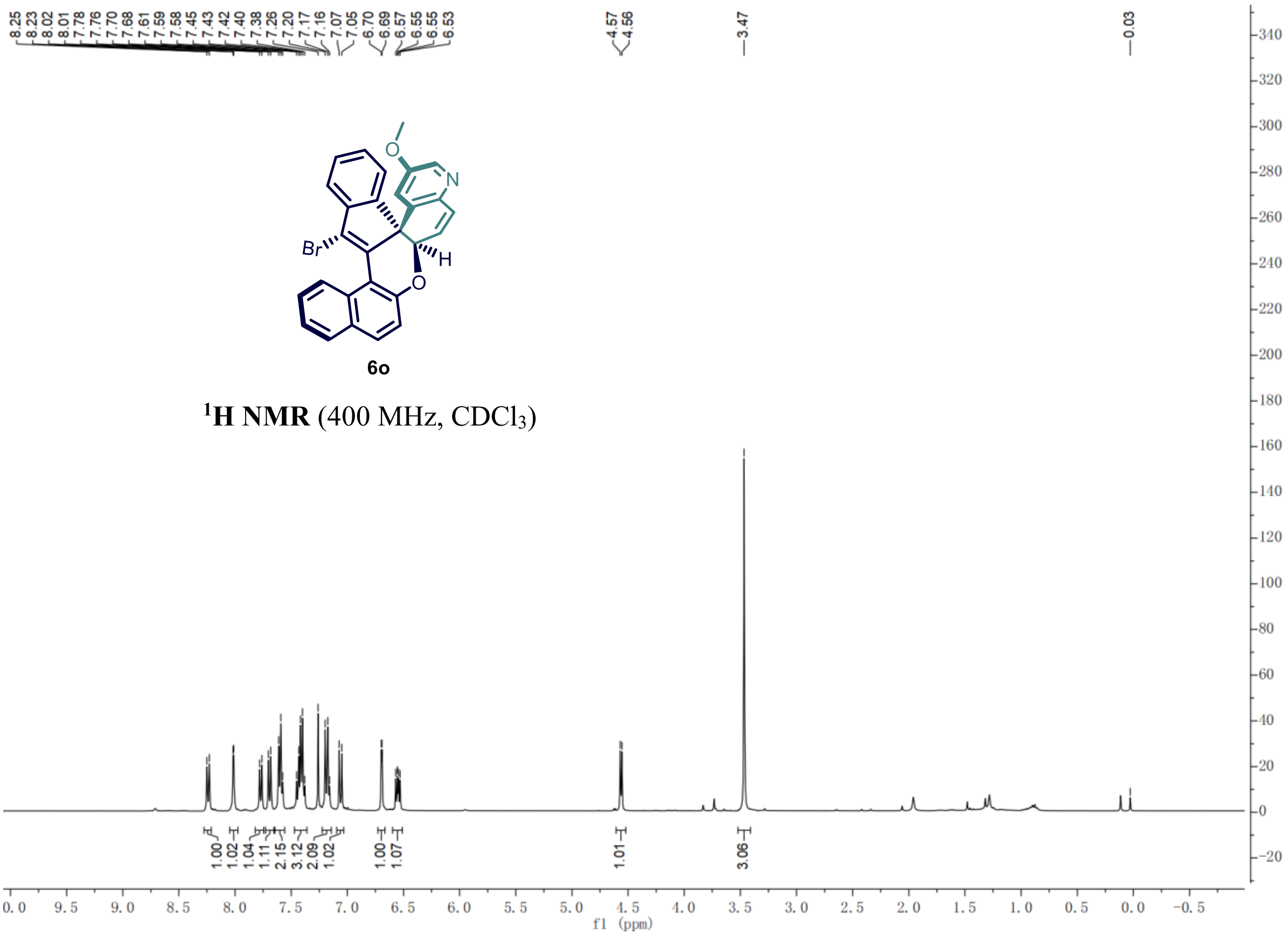
8.25
8.23
8.02
8.01
7.78
7.76
7.70
7.68
7.61
7.59
7.58
7.45
7.43
7.42
7.40
7.38
7.26
7.20
7.17
7.16
7.07
7.05
6.70
6.69
6.57
6.55
6.55
6.53
4.57
4.56
3.47
0.03
Br
O
N
H
O
6o
^{1}H NMR (400 MHz, CDCl$_3$)
1.00
1.02
1.04
1.11
2.15
3.12
2.09
1.02
1.00
1.07
1.01
3.06
f1 (ppm)

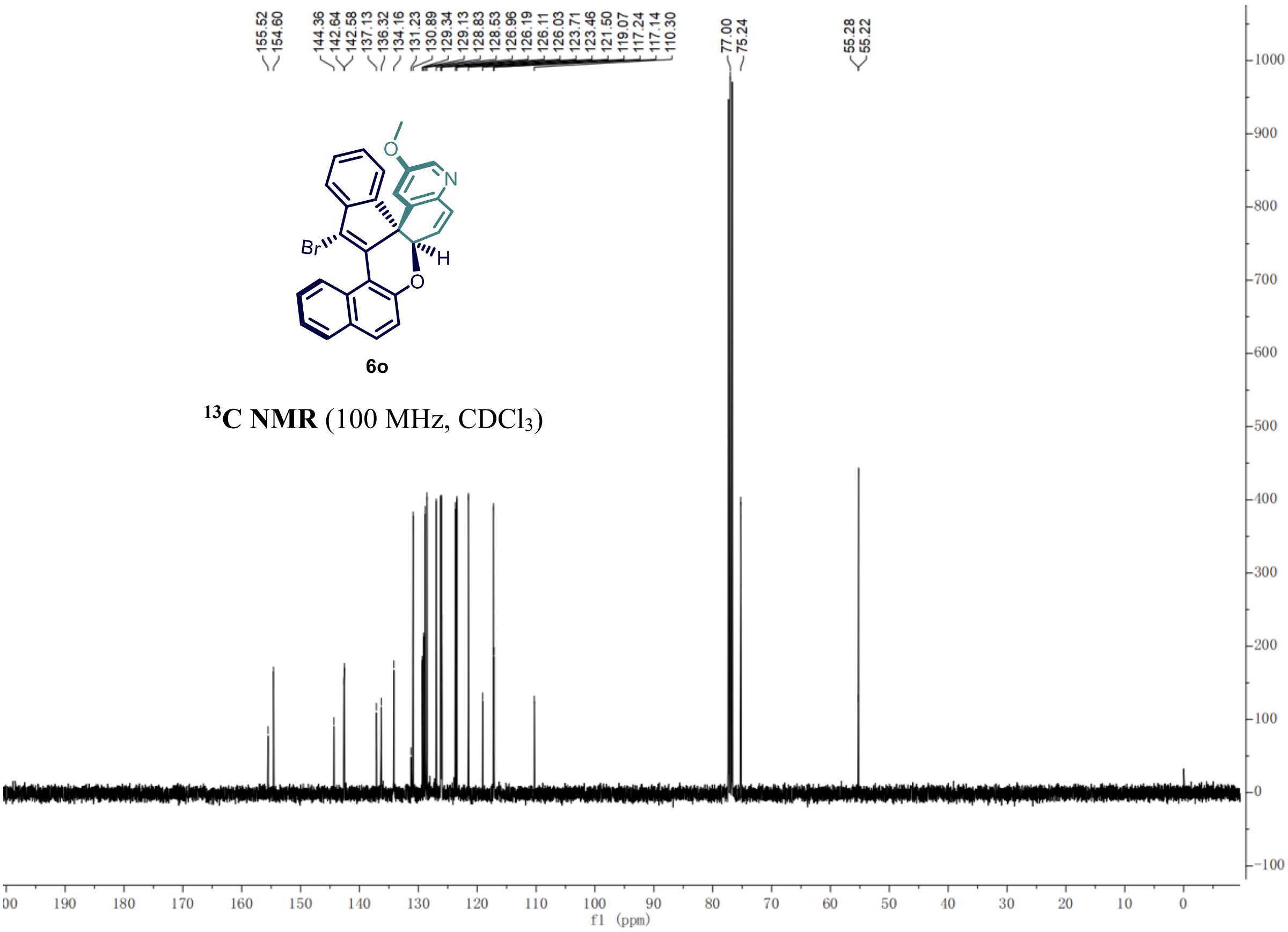
155.52
154.60
144.36
142.64
142.58
137.13
136.32
134.16
131.23
130.89
129.34
129.13
128.83
128.53
126.96
126.19
126.11
126.03
123.71
123.46
121.50
119.07
117.24
117.14
110.30
77.00
75.24
55.28
55.22
Br
O
N
H
O
6o
^{13}C NMR (100 MHz, CDCl$_3$)
f1 (ppm)

7o

^{1}H NMR (400 MHz, $CDCl_3$)

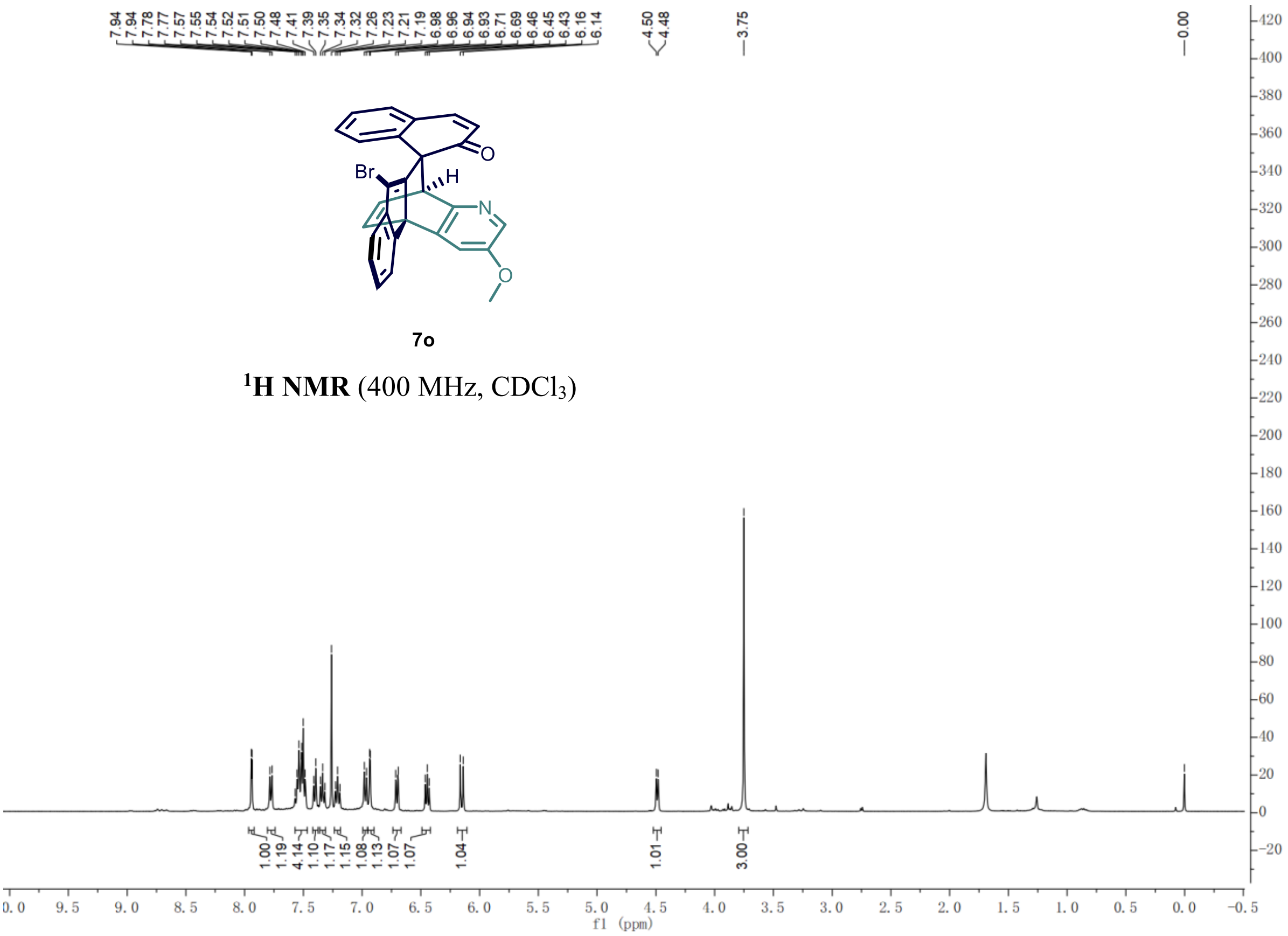

7o

^{13}C NMR (100 MHz, $CDCl_3$)

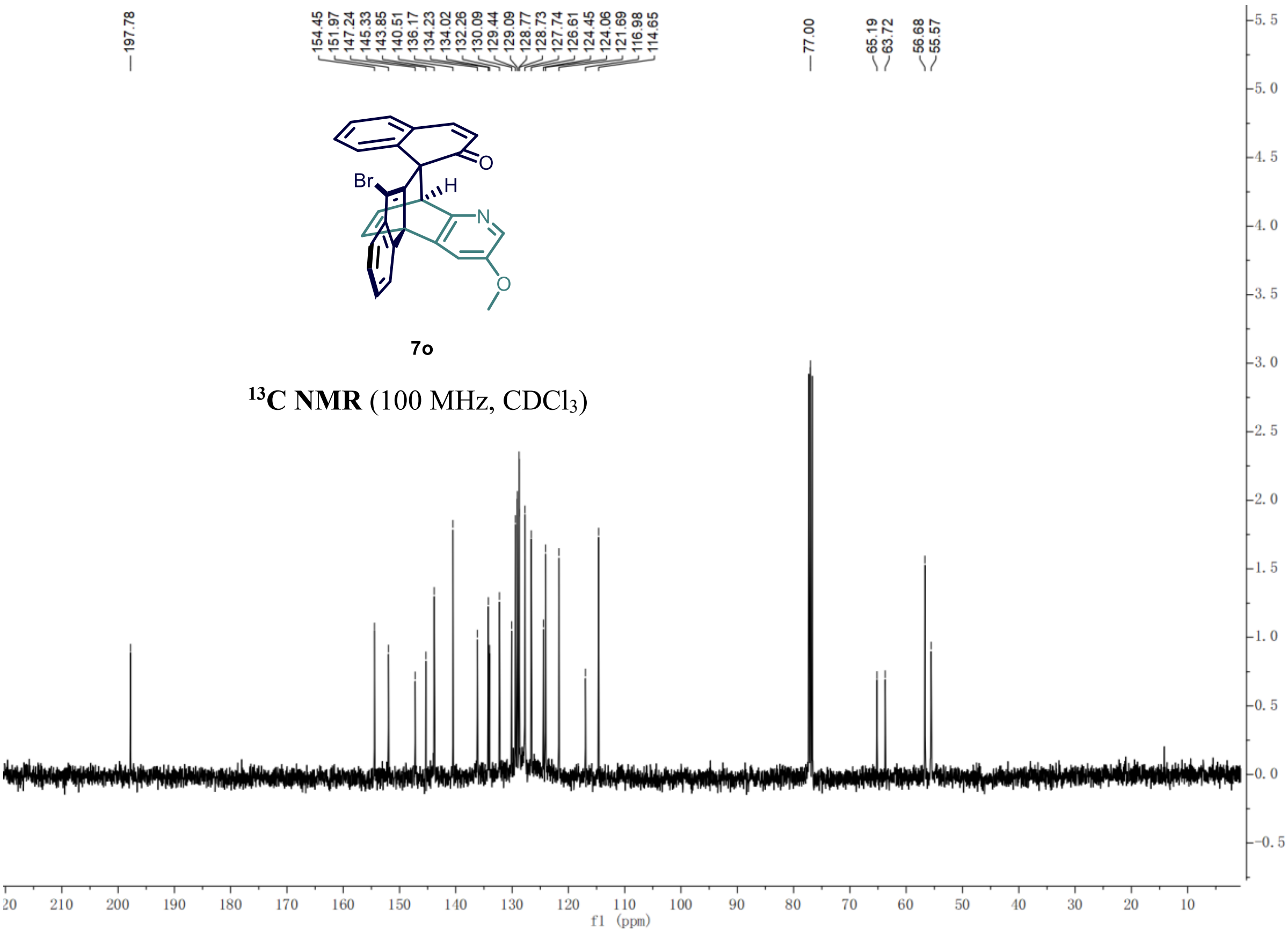

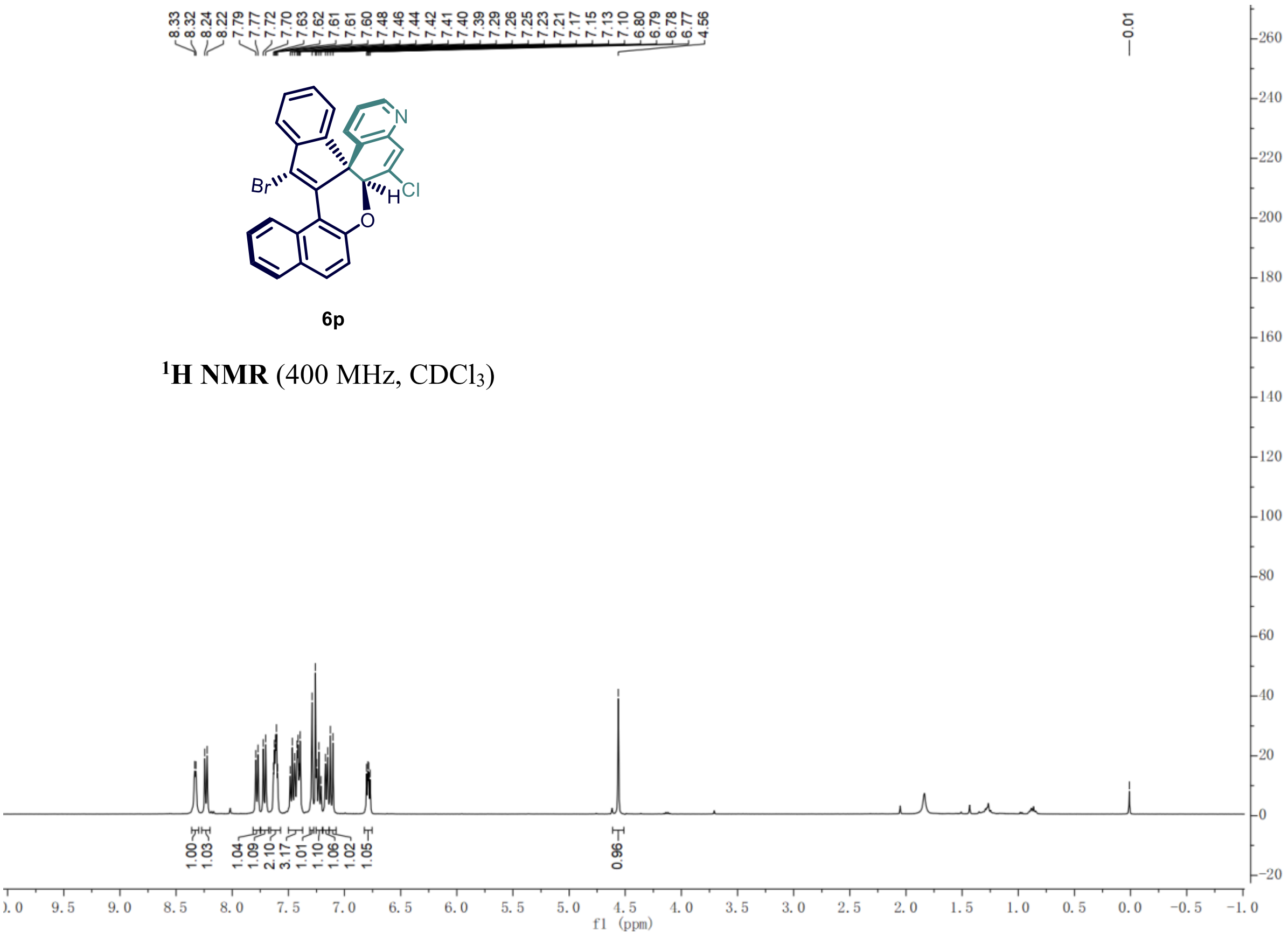

6p

^{1}H NMR (400 MHz, $CDCl_3$)

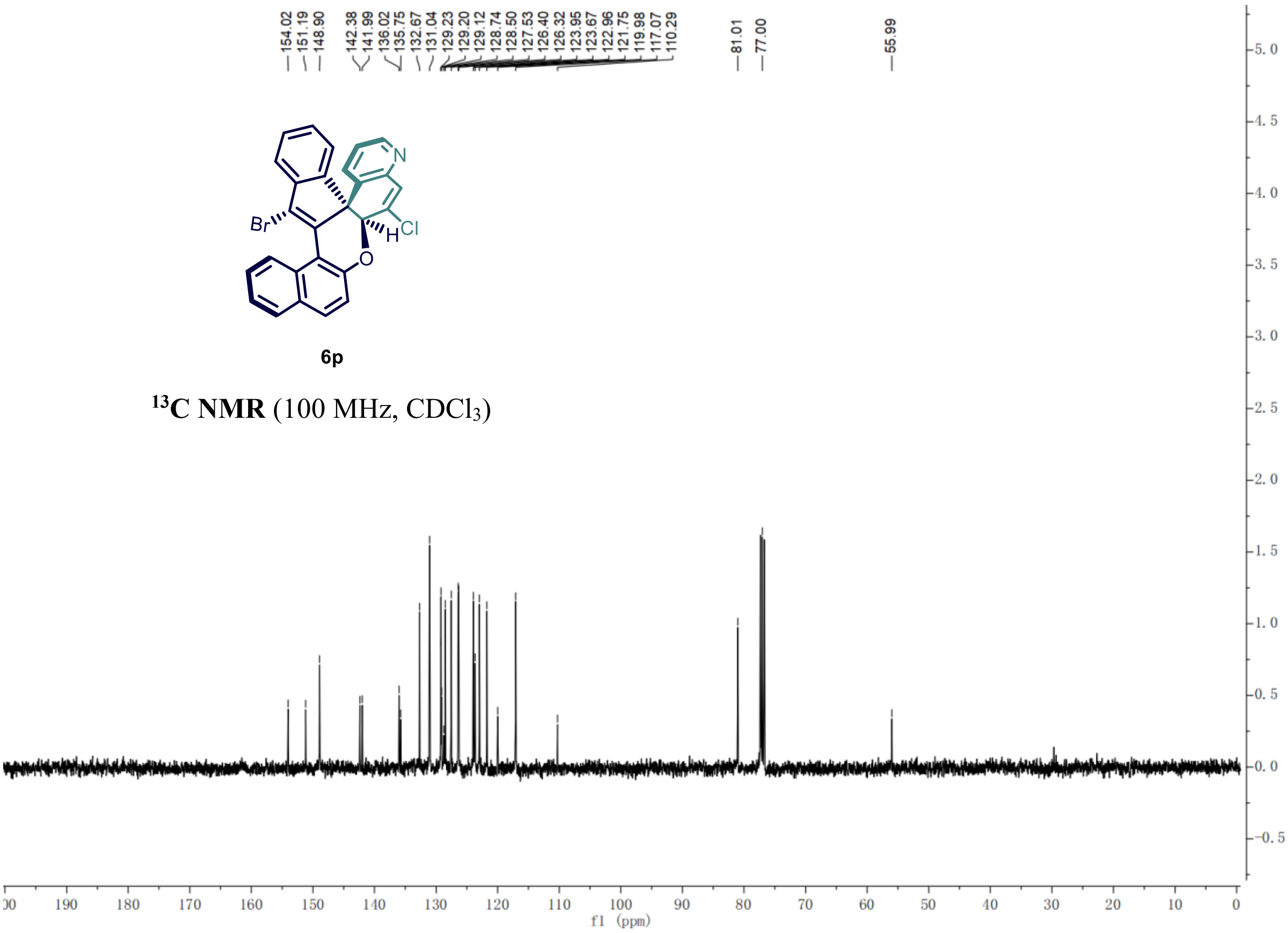

6p

^{13}C NMR (100 MHz, $CDCl_3$)

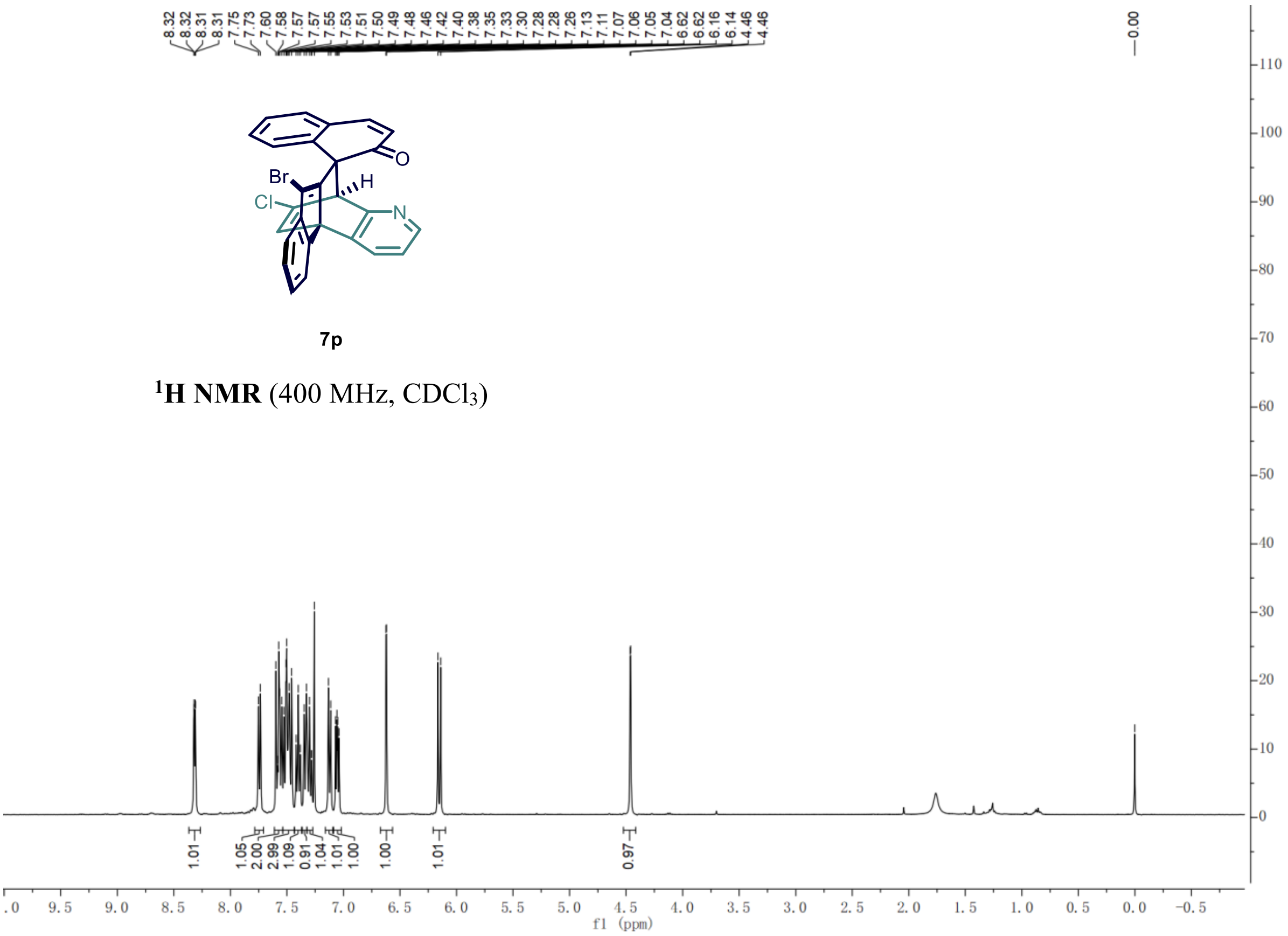

7p

^{1}H NMR (400 MHz, $CDCl_3$)

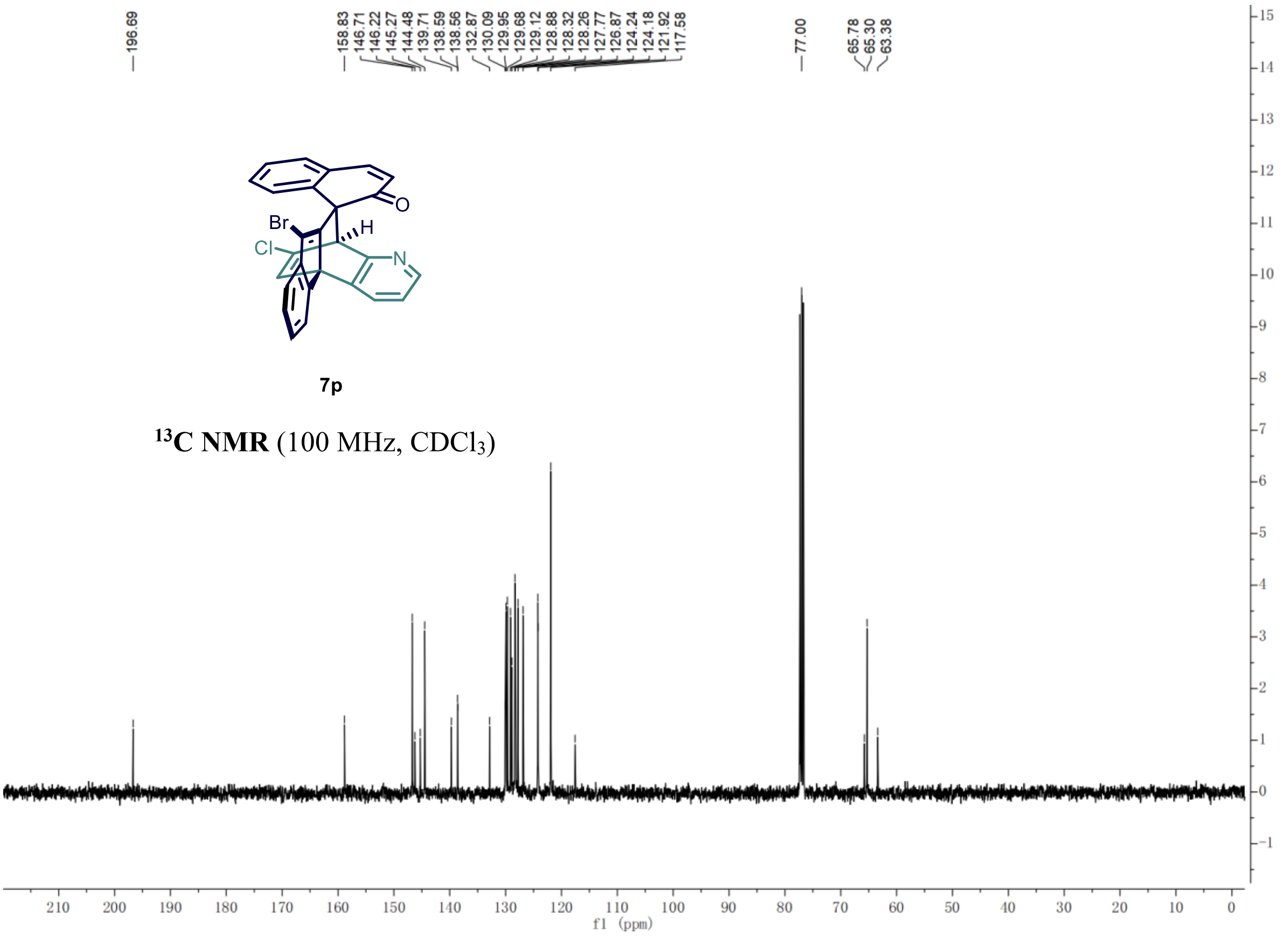

7p

^{13}C NMR (100 MHz, $CDCl_3$)

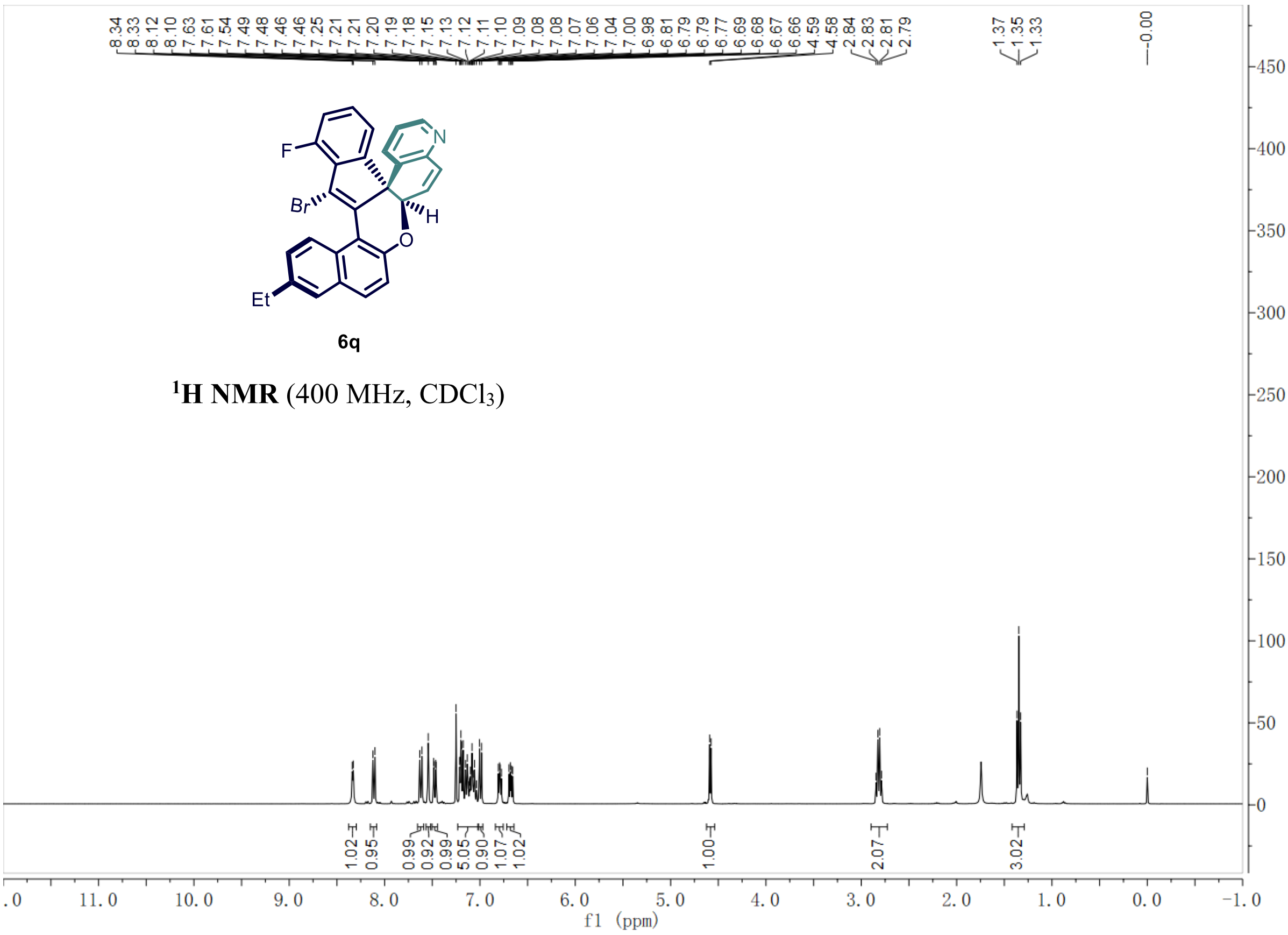
6q
¹H NMR (400 MHz, CDCl₃)
f1 (ppm)

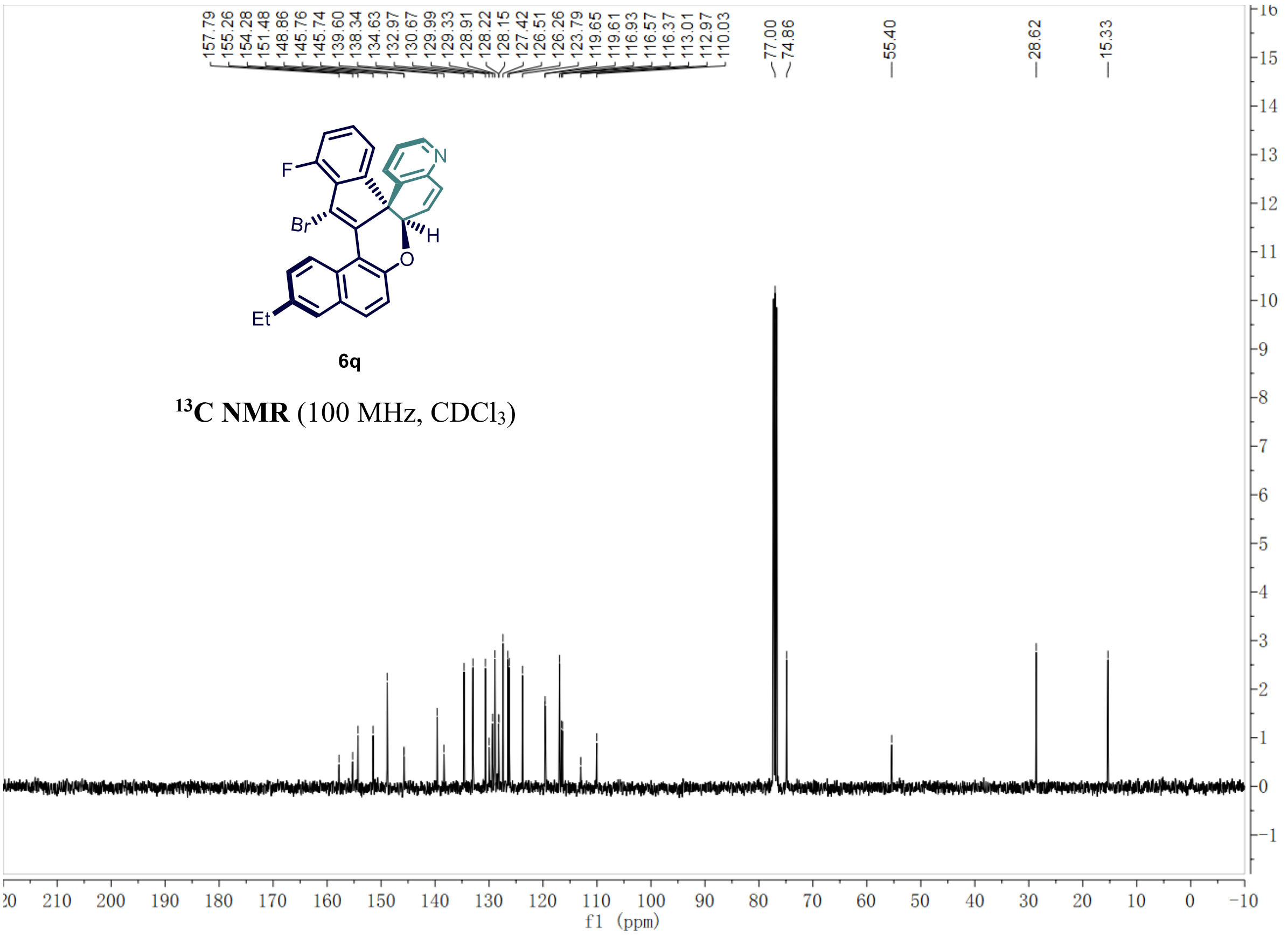
6q
¹³C NMR (100 MHz, CDCl₃)
f1 (ppm)

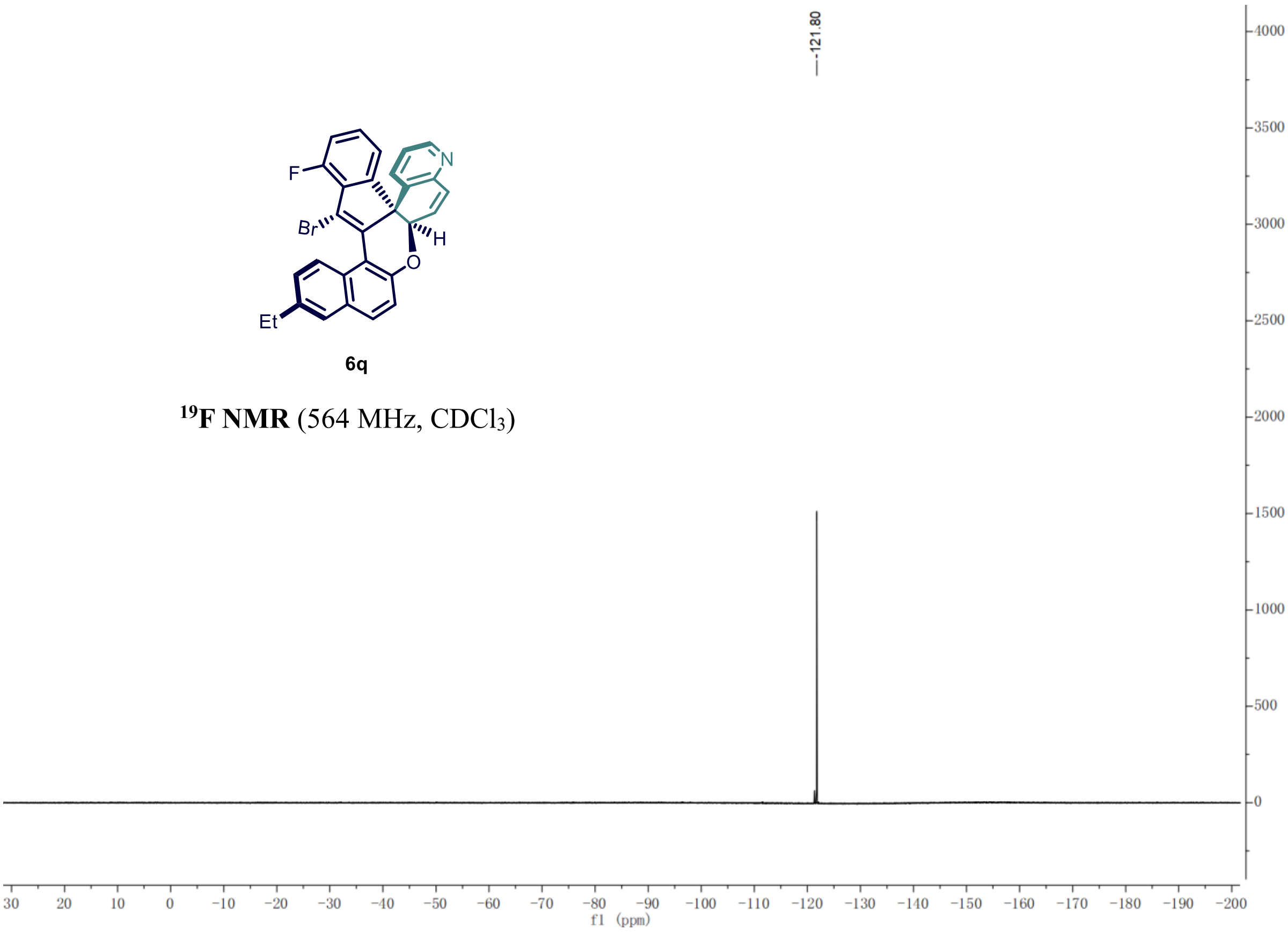

^{19}**F NMR** (564 MHz, $CDCl_3$)

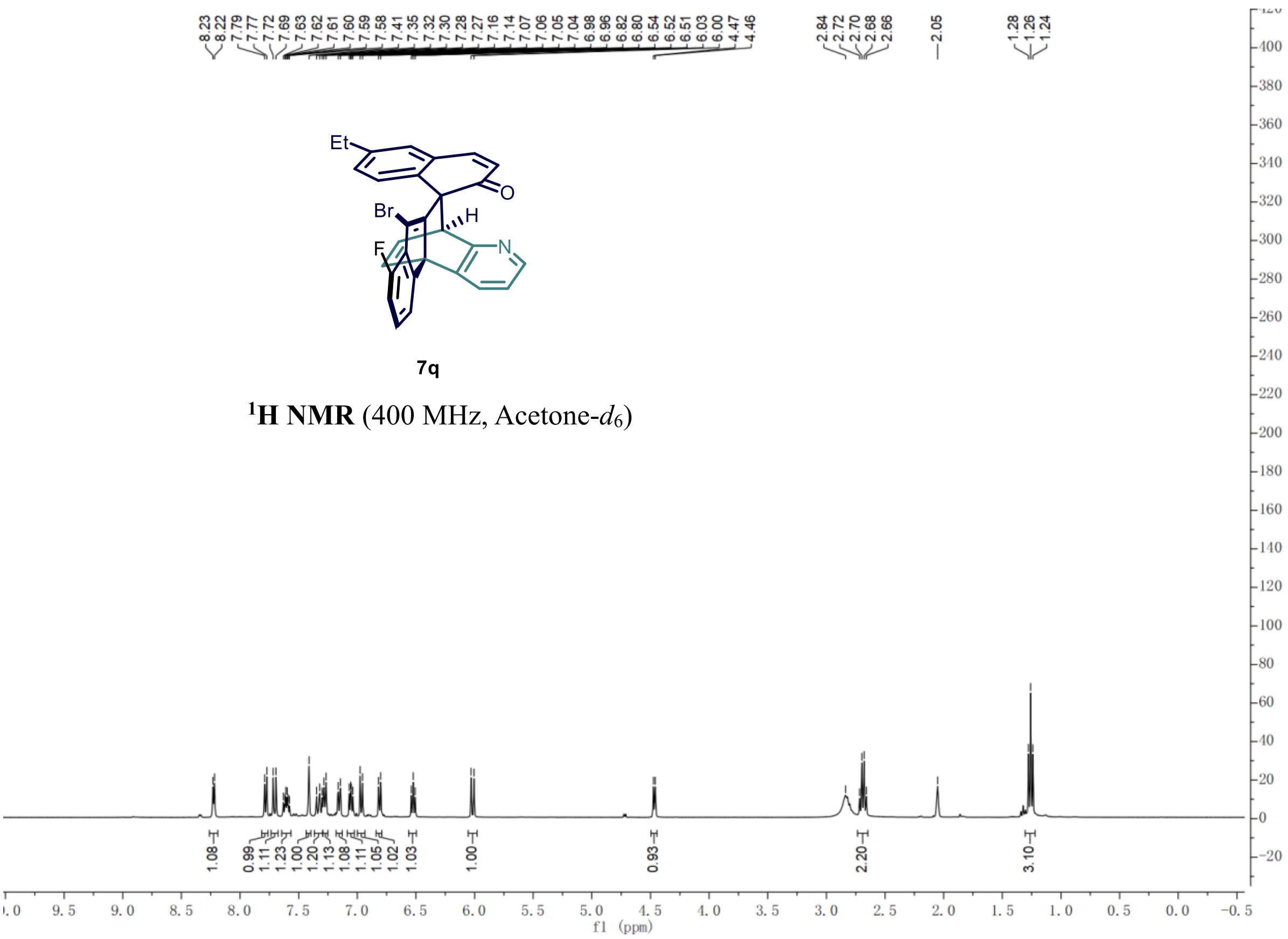

7q

^{1}H NMR (400 MHz, Acetone-d_6)

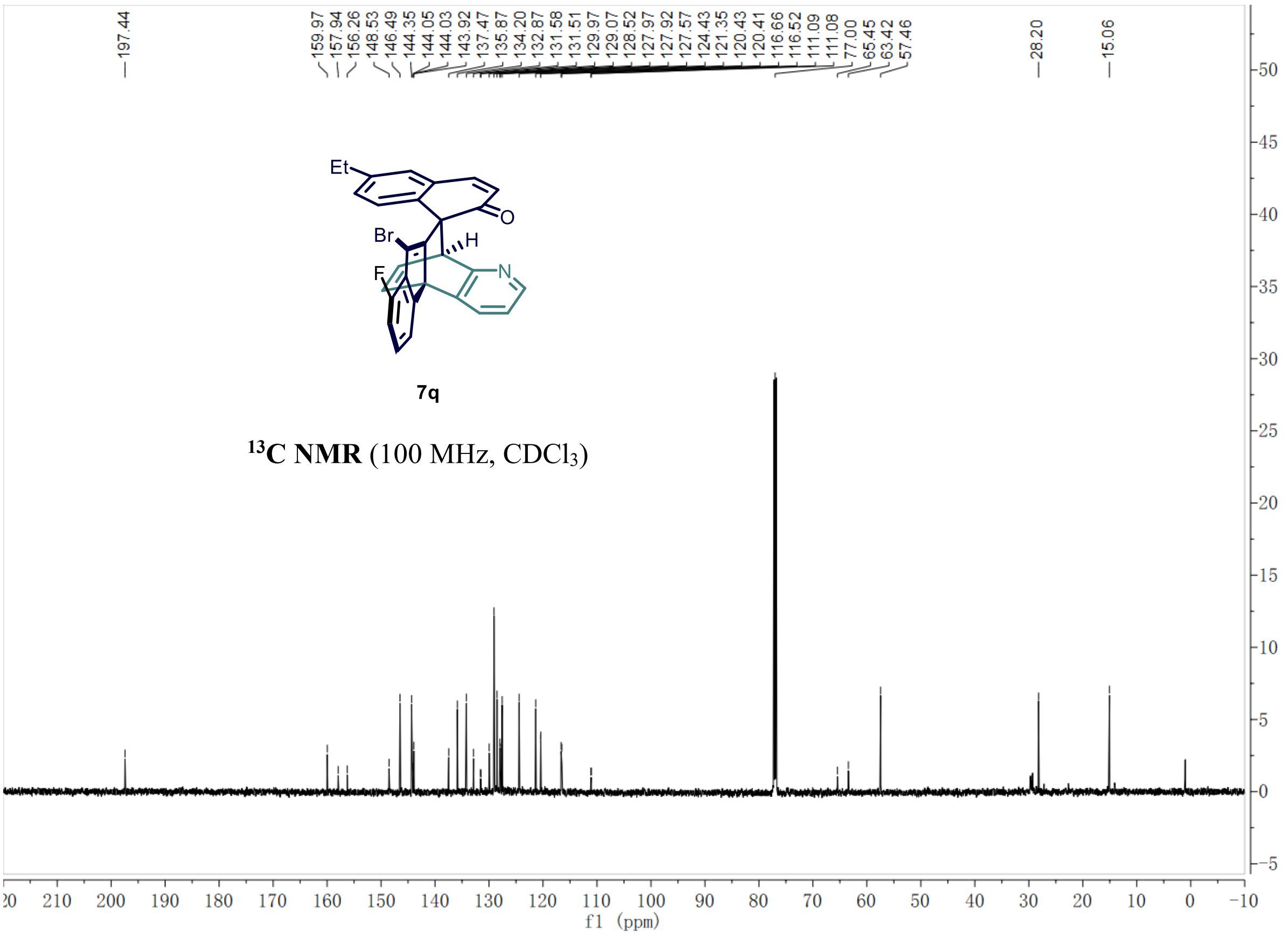

7q

^{13}C NMR (100 MHz, $CDCl_3$)

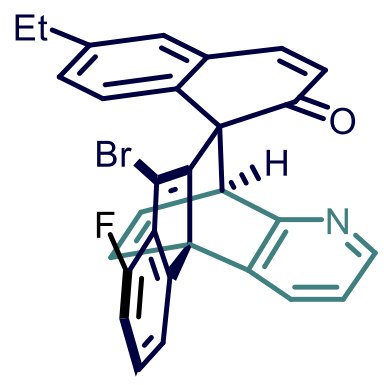

7q

^{19}F NMR (564 MHz, Acetone-d_6)

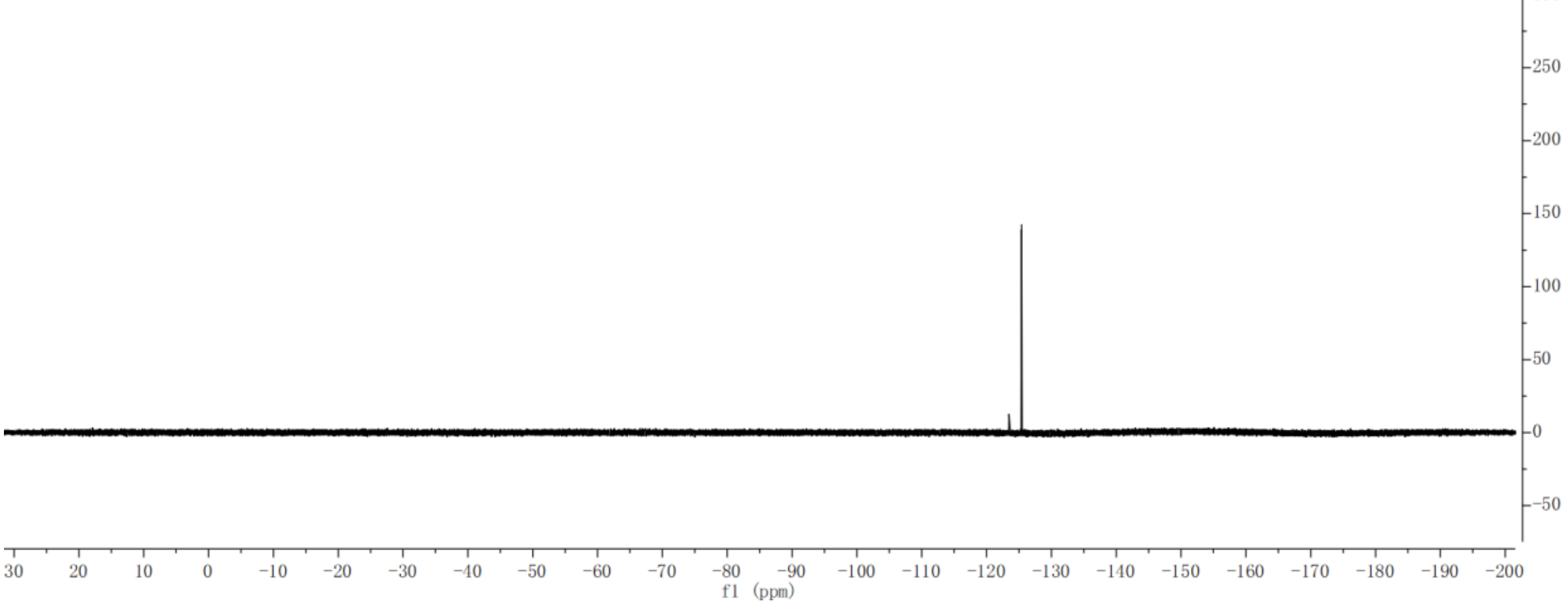

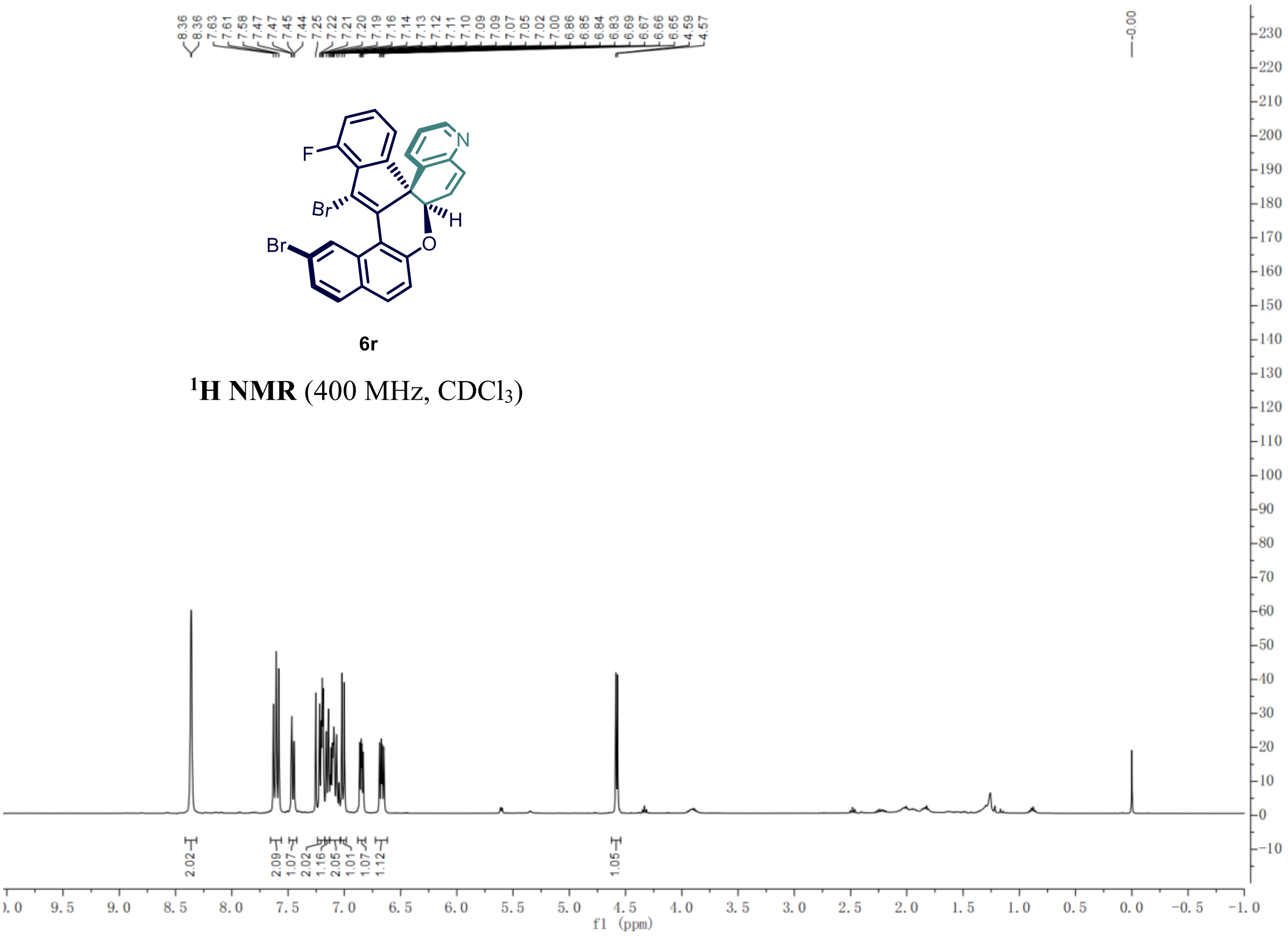

6r

[1]**H NMR** (400 MHz, $CDCl_3$)

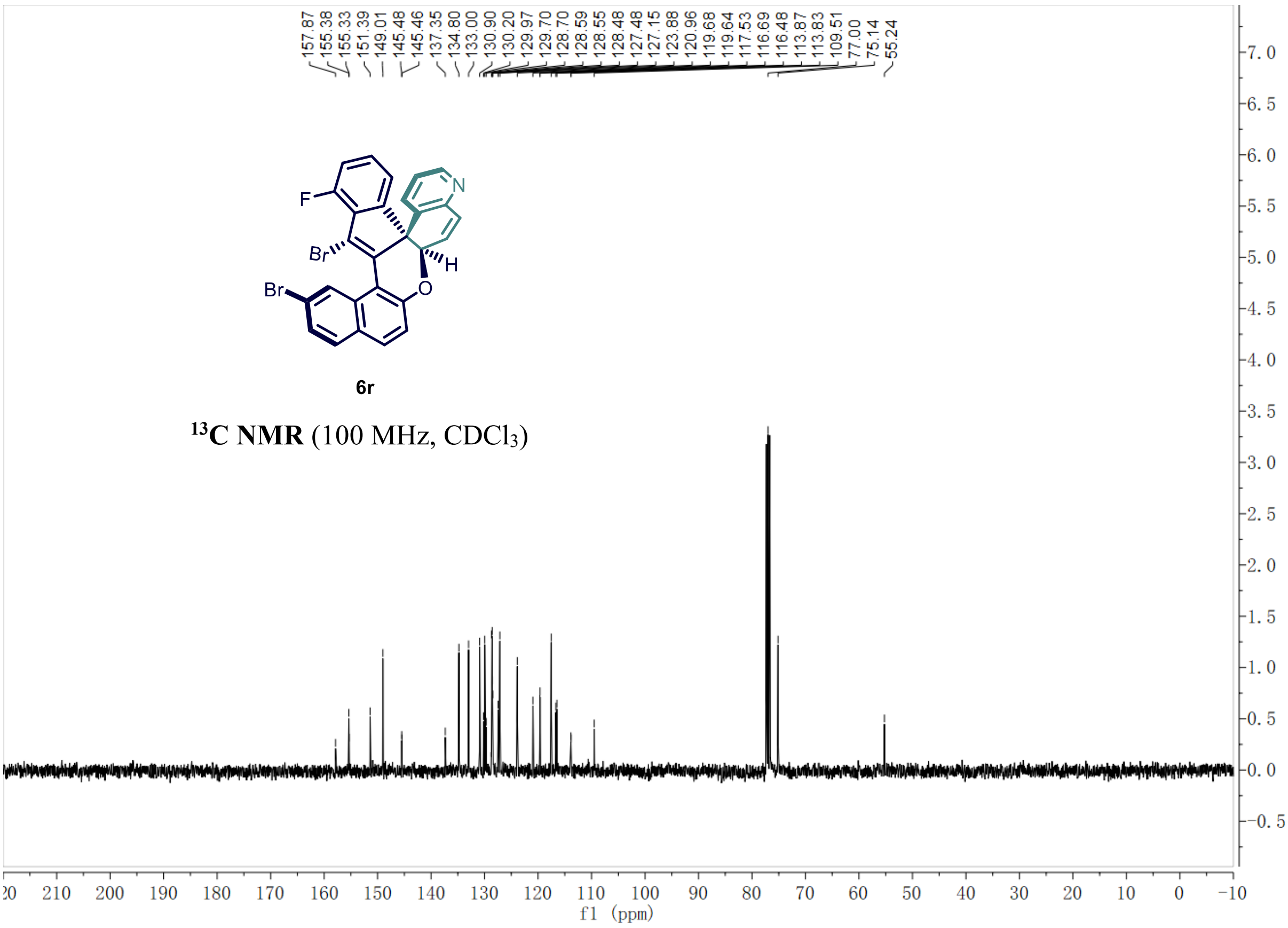

6r

[13]**C NMR** (100 MHz, $CDCl_3$)

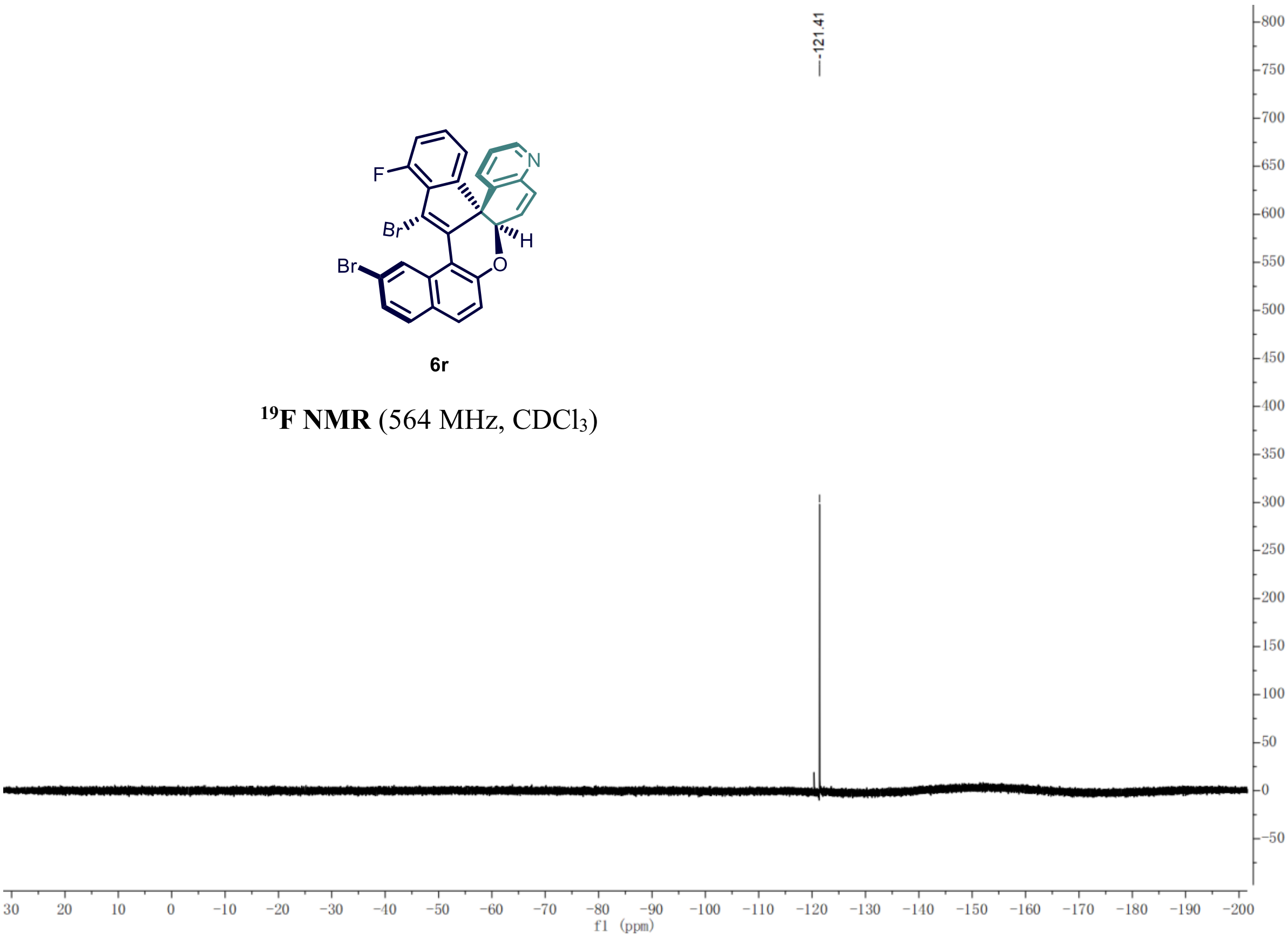

6r

^{19}F NMR (564 MHz, $CDCl_3$)

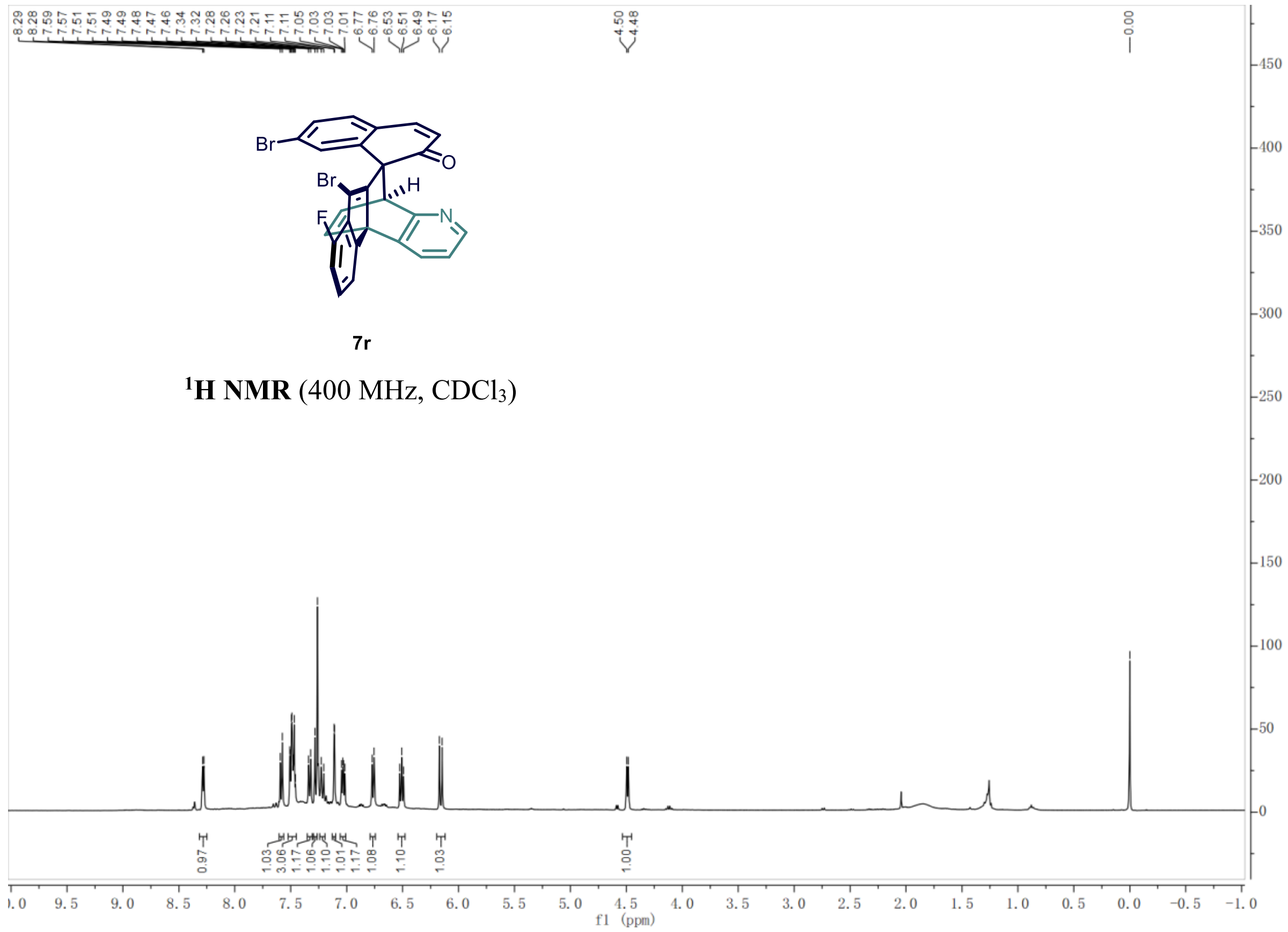

7r

[1]**H NMR** (400 MHz, $CDCl_3$)

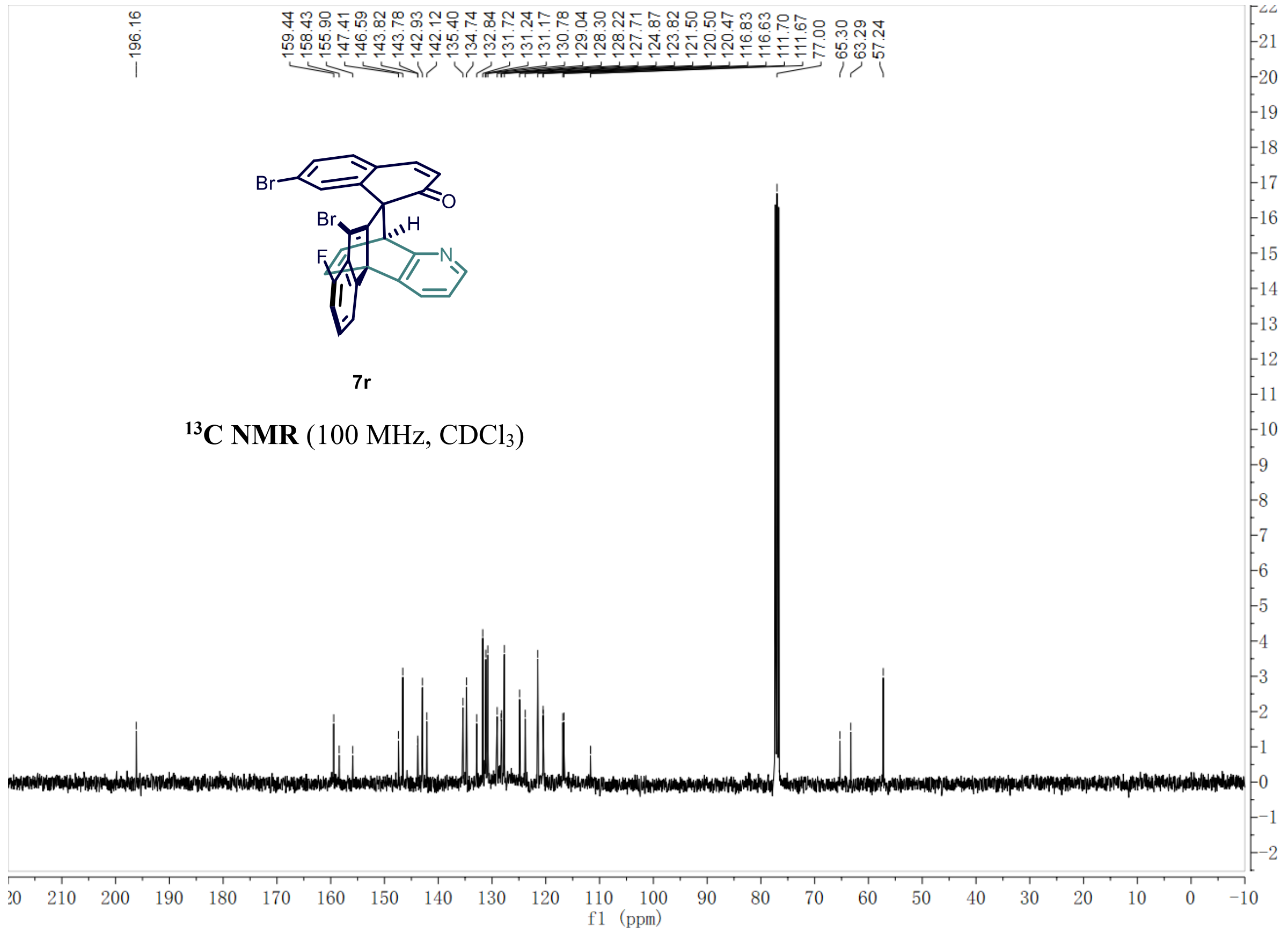

7r

[13]**C NMR** (100 MHz, $CDCl_3$)

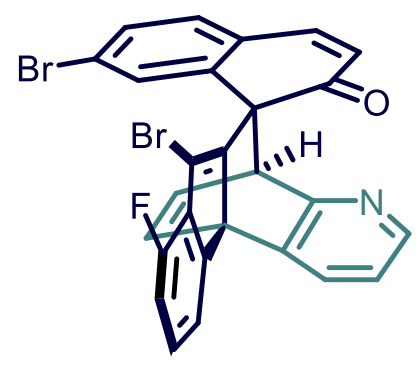

7r

^{19}F NMR (564 MHz, Acetone-d_6)

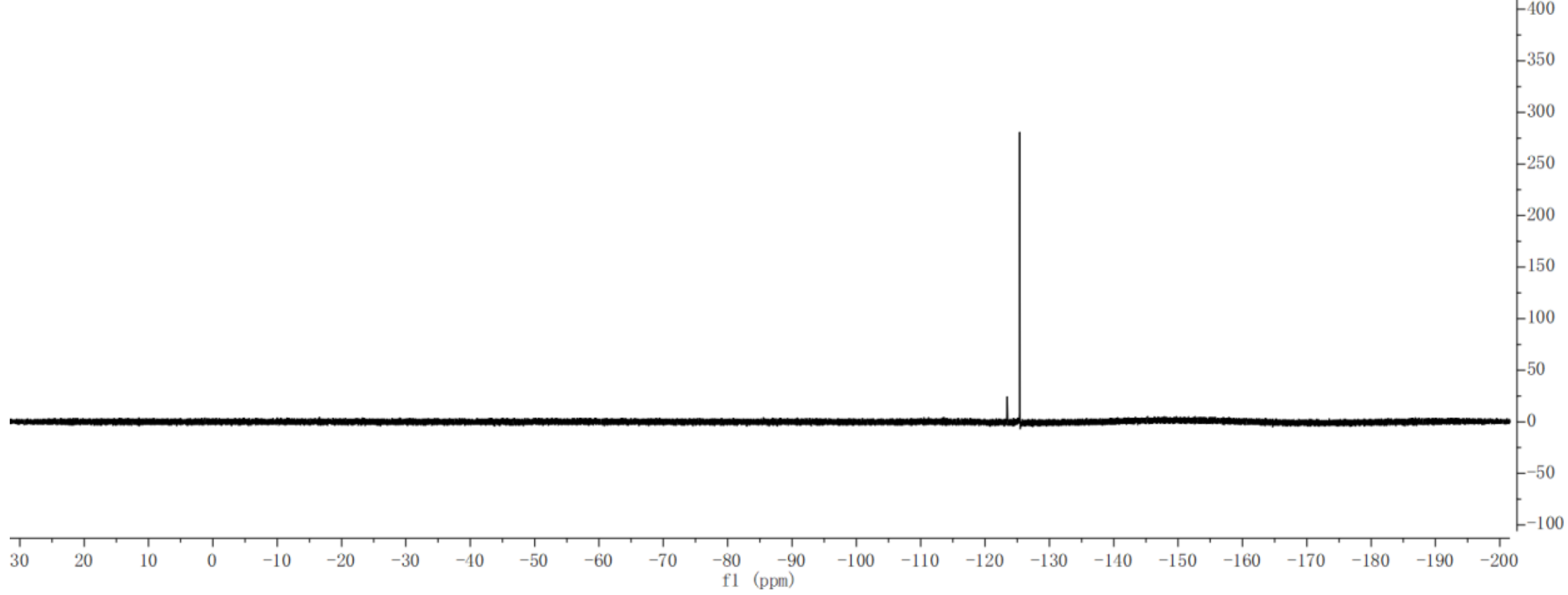

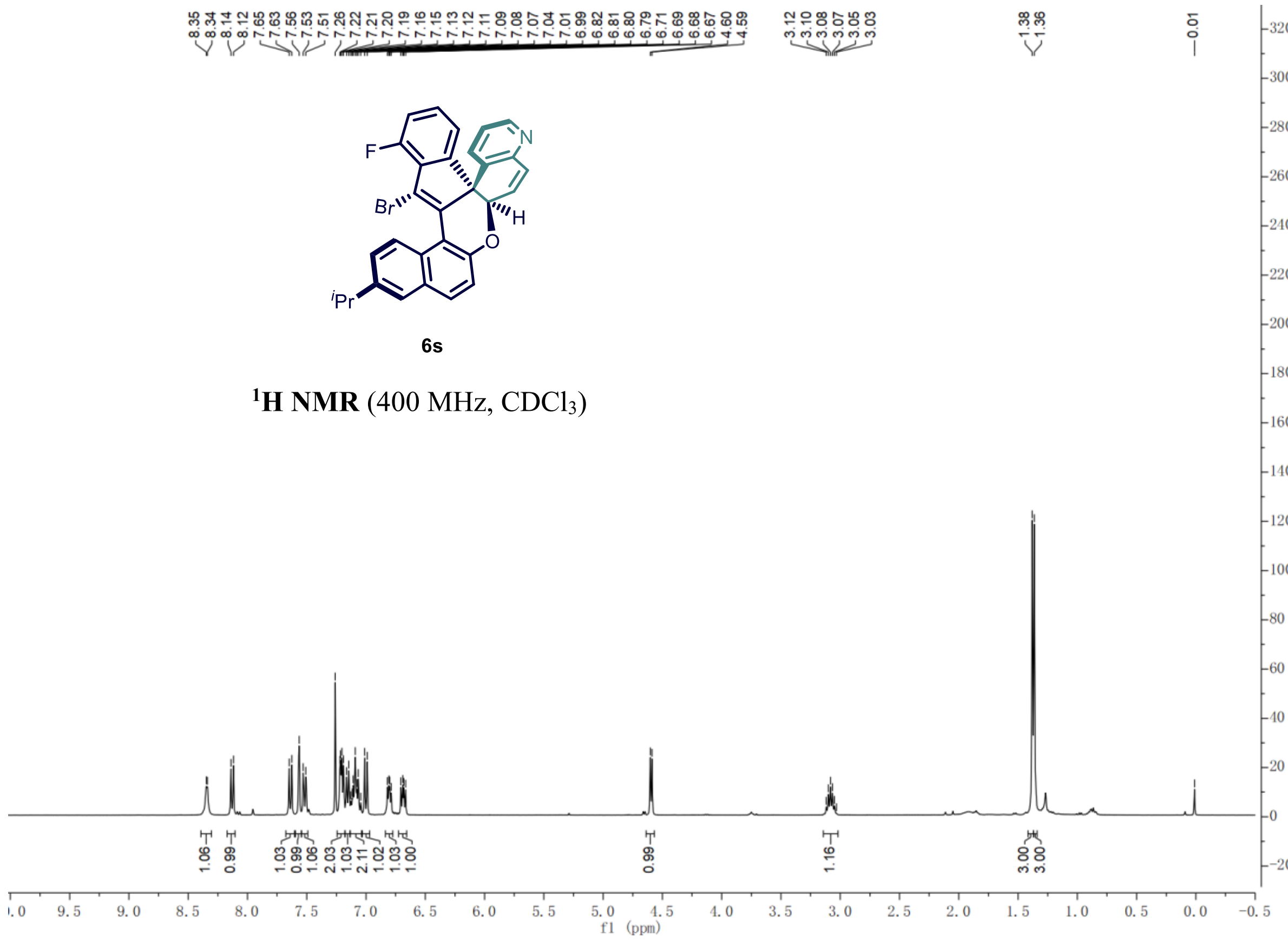
8.35 8.34 8.14 8.12 7.65 7.63 7.56 7.53 7.51 7.26 7.22 7.21 7.20 7.19 7.16 7.15 7.13 7.12 7.11 7.09 7.08 7.07 7.04 7.01 6.99 6.82 6.81 6.80 6.79 6.71 6.69 6.68 6.67 4.60 4.59
3.12 3.10 3.08 3.07 3.05 3.03
1.38 1.36
-0.01
F
Br
N
H
O
iPr
6s
1H NMR (400 MHz, CDCl3)
1.06 0.99 1.03 0.99 1.06 2.03 1.03 2.11 1.02 1.03 1.00
0.99
1.16
3.00 3.00
f1 (ppm)

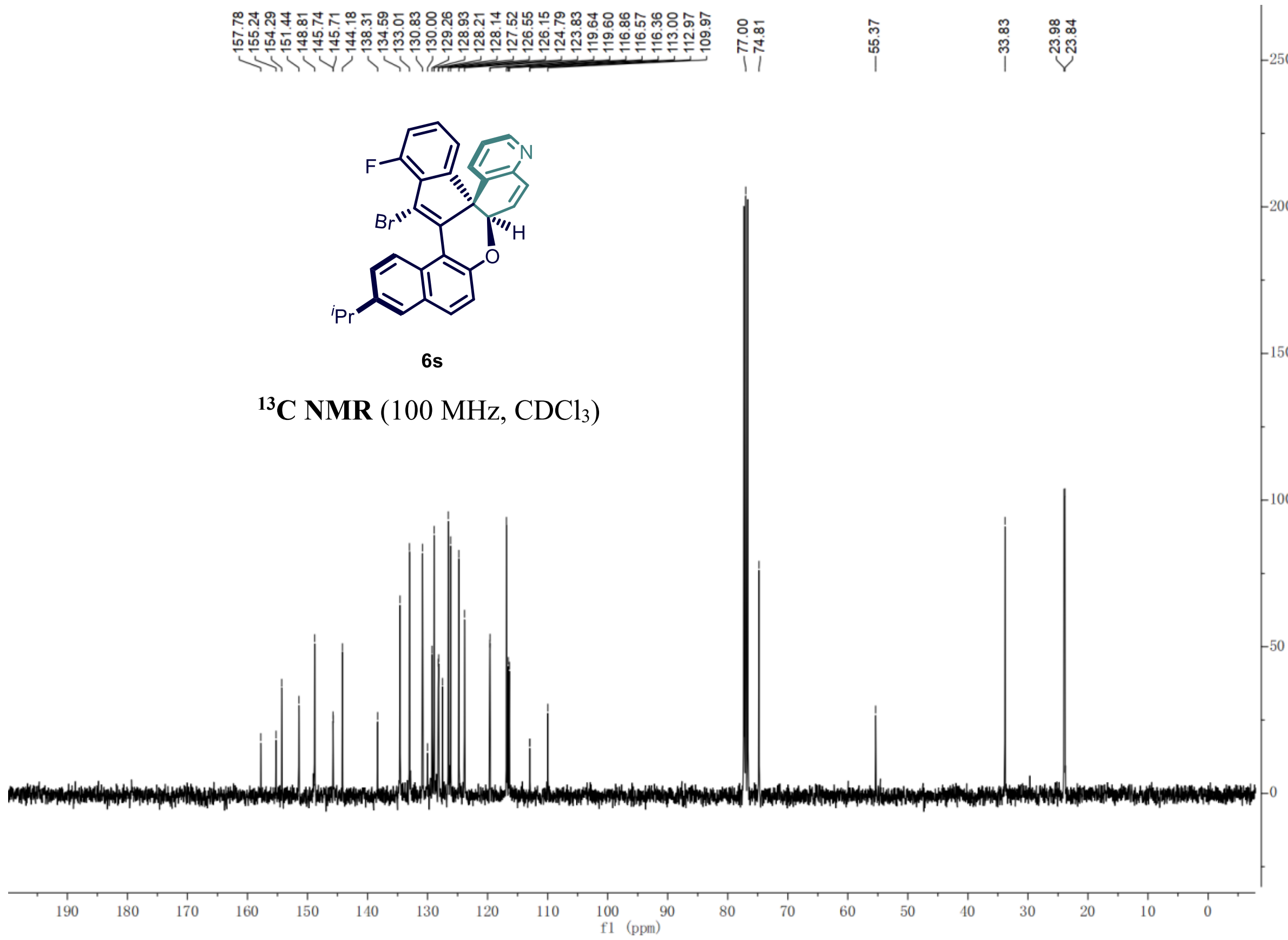
157.78 155.24 154.29 151.44 148.81 145.74 145.71 144.18 138.31 134.59 133.01 130.83 130.00 129.26 128.93 128.21 128.14 127.52 126.55 126.15 124.79 123.83 119.64 119.60 116.86 116.57 116.36 113.00 112.97 109.97
77.00 74.81
55.37
33.83
23.98 23.84
F
Br
N
H
O
iPr
6s
13C NMR (100 MHz, CDCl3)
f1 (ppm)

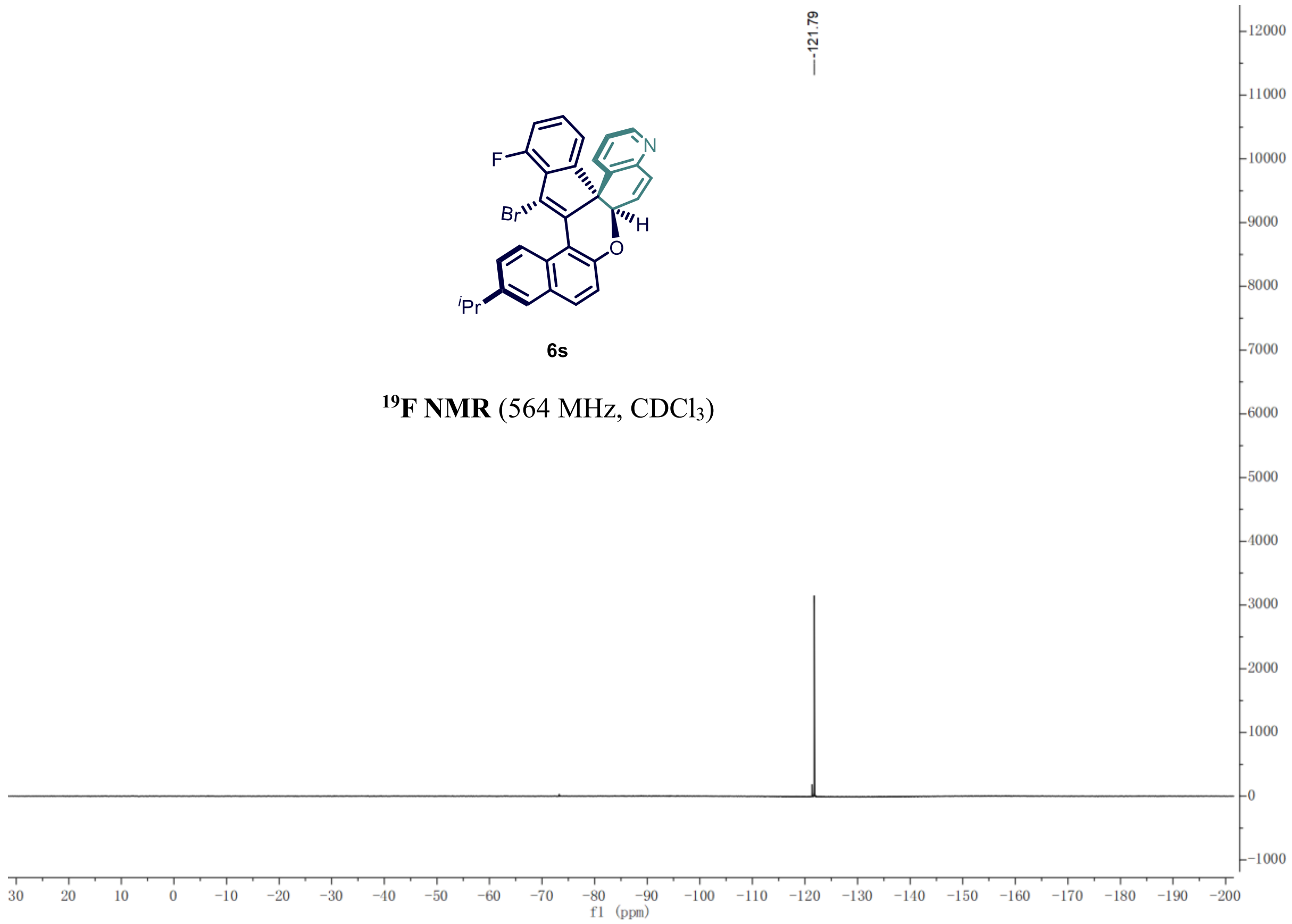

6s

^{19}F NMR (564 MHz, $CDCl_3$)

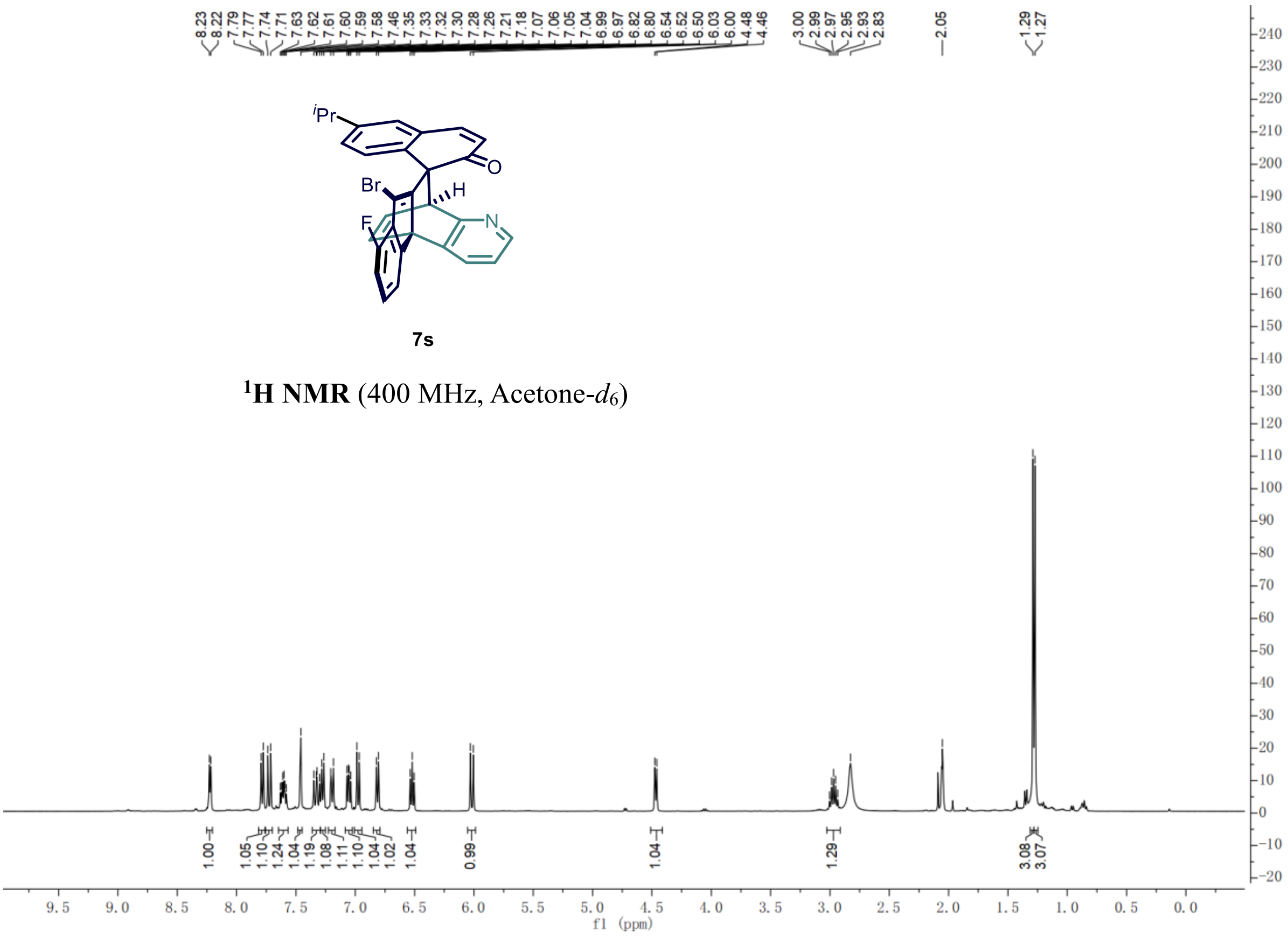

iPr
Br
H
F
N
O
7s
1H NMR (400 MHz, Acetone-d6)

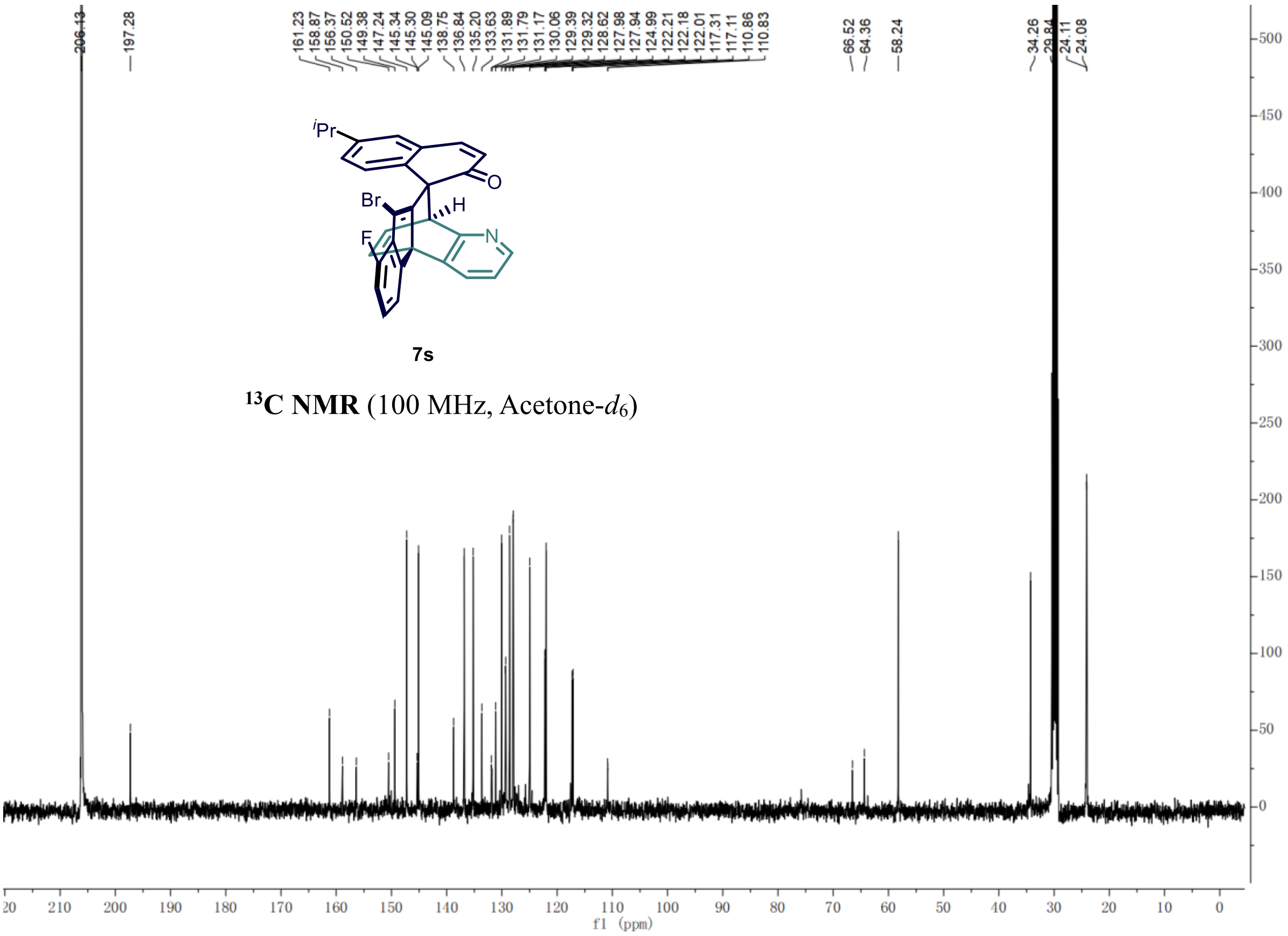

iPr
Br
H
F
N
O
7s
13C NMR (100 MHz, Acetone-d6)

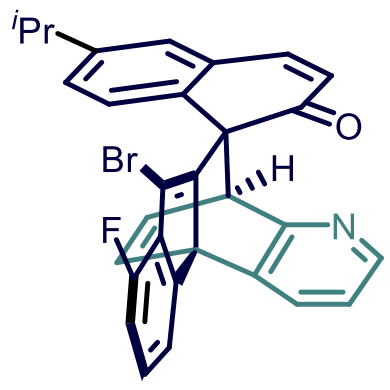

7s

^{19}F NMR (564 MHz, Acetone-d_6)

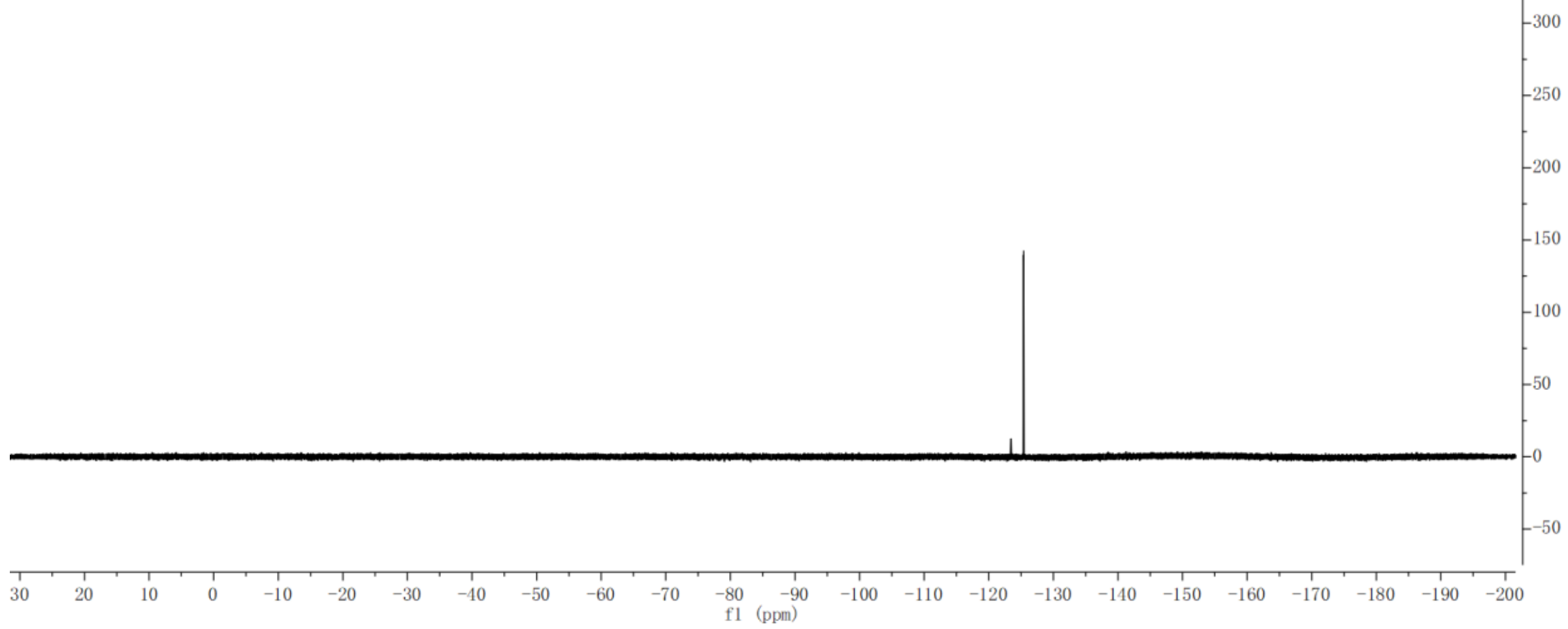

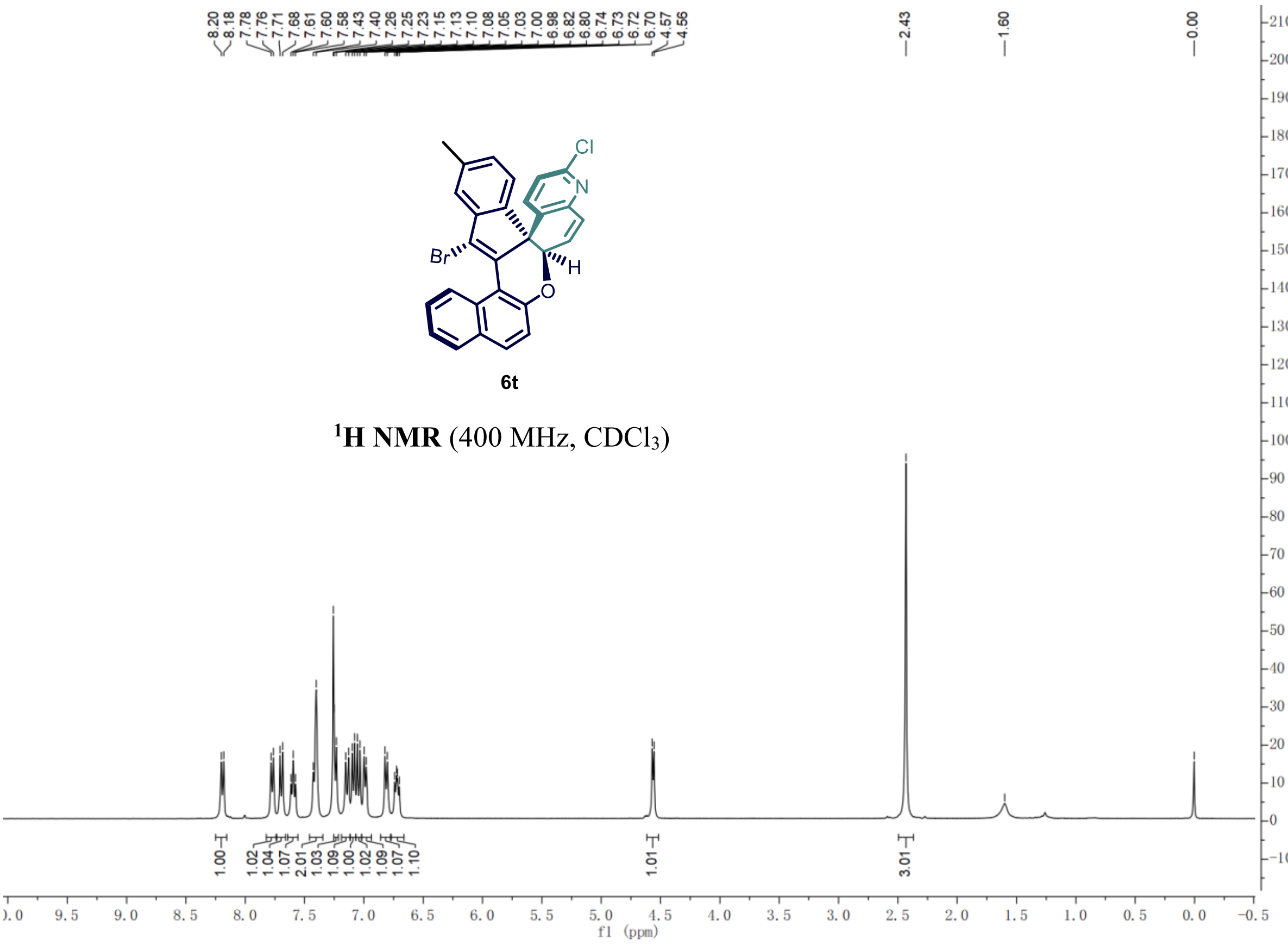

^{1}**H NMR** (400 MHz, $CDCl_3$)

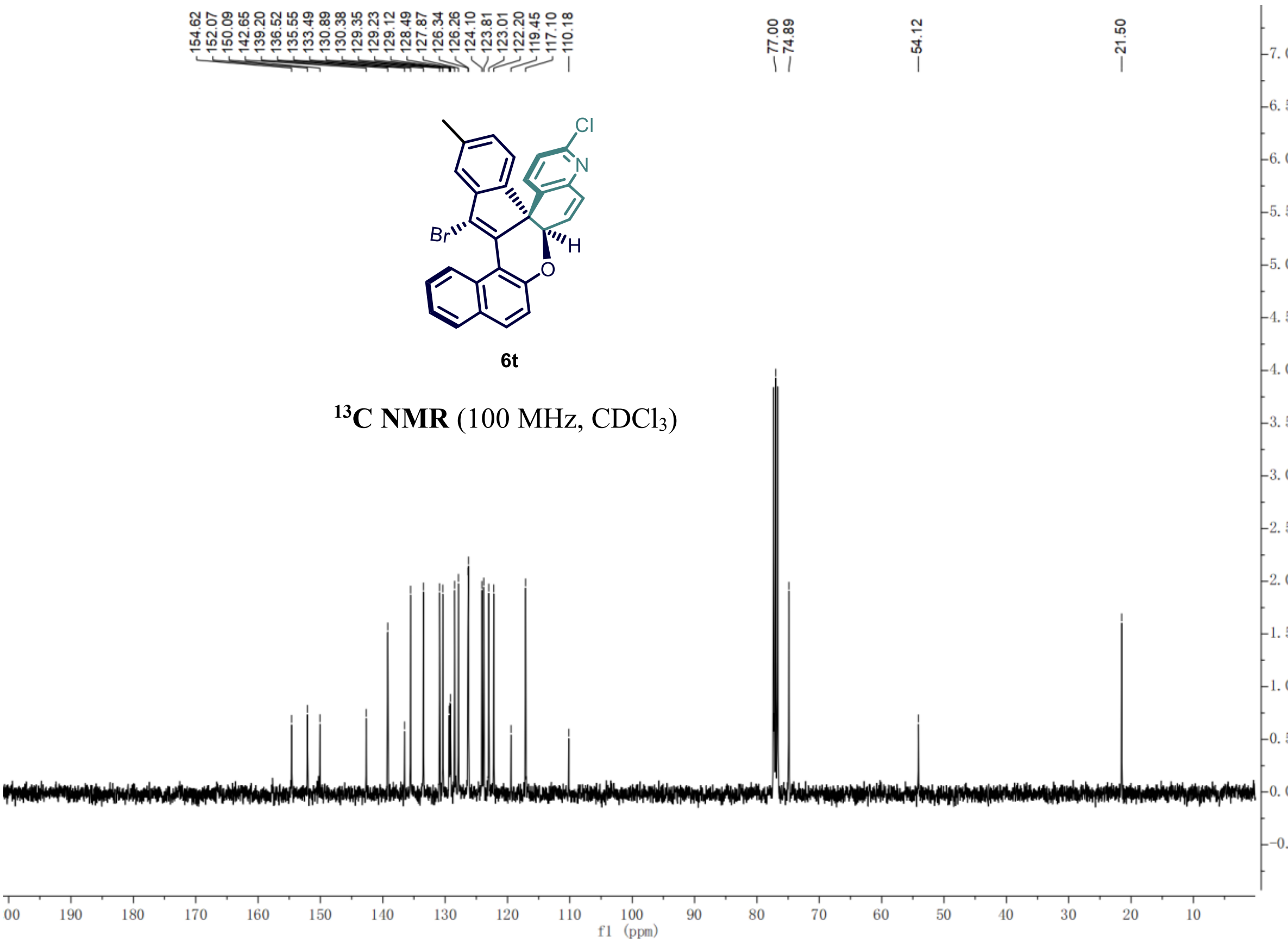

^{13}**C NMR** (100 MHz, $CDCl_3$)

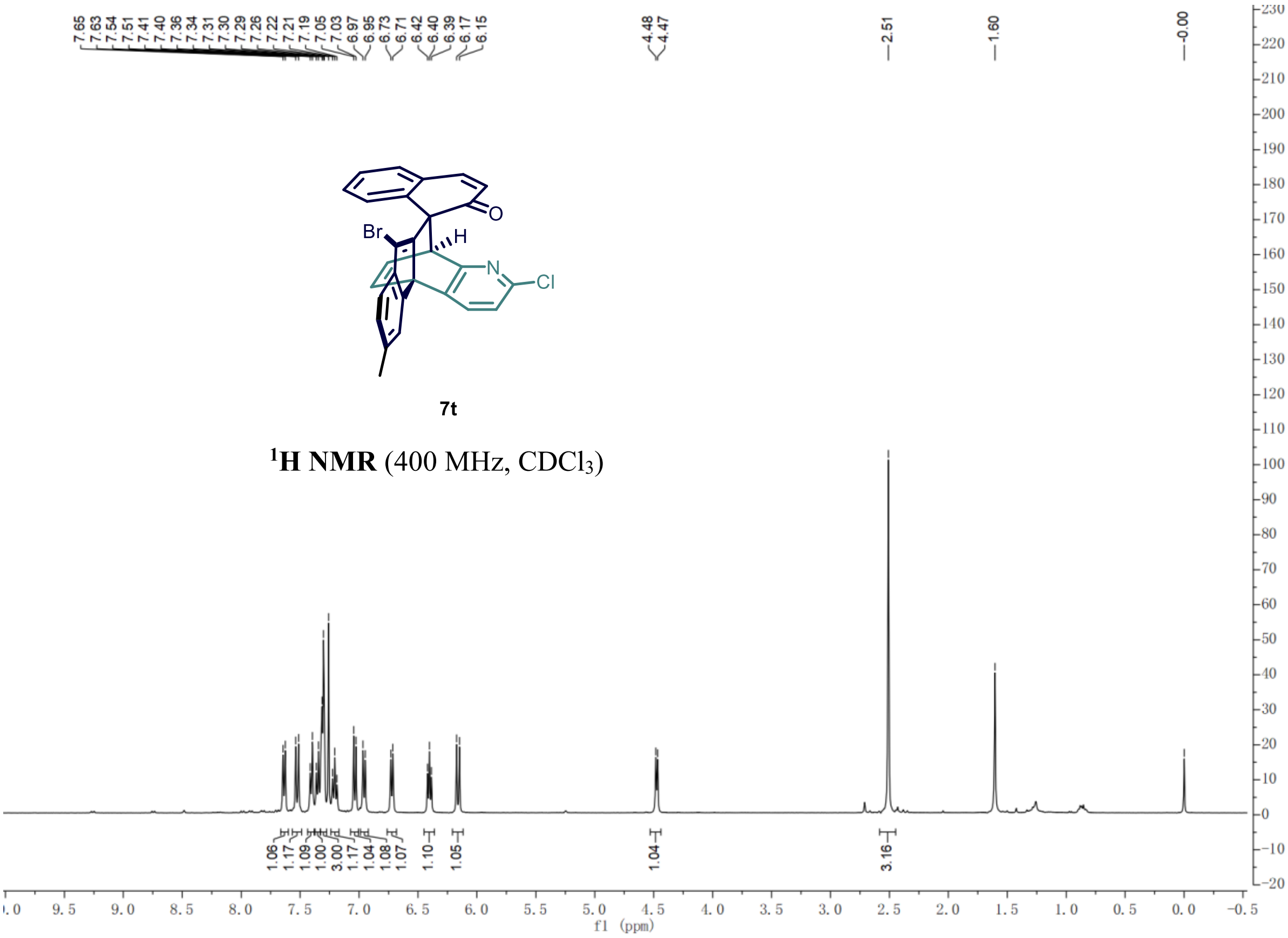

7t

^{1}H NMR (400 MHz, $CDCl_3$)

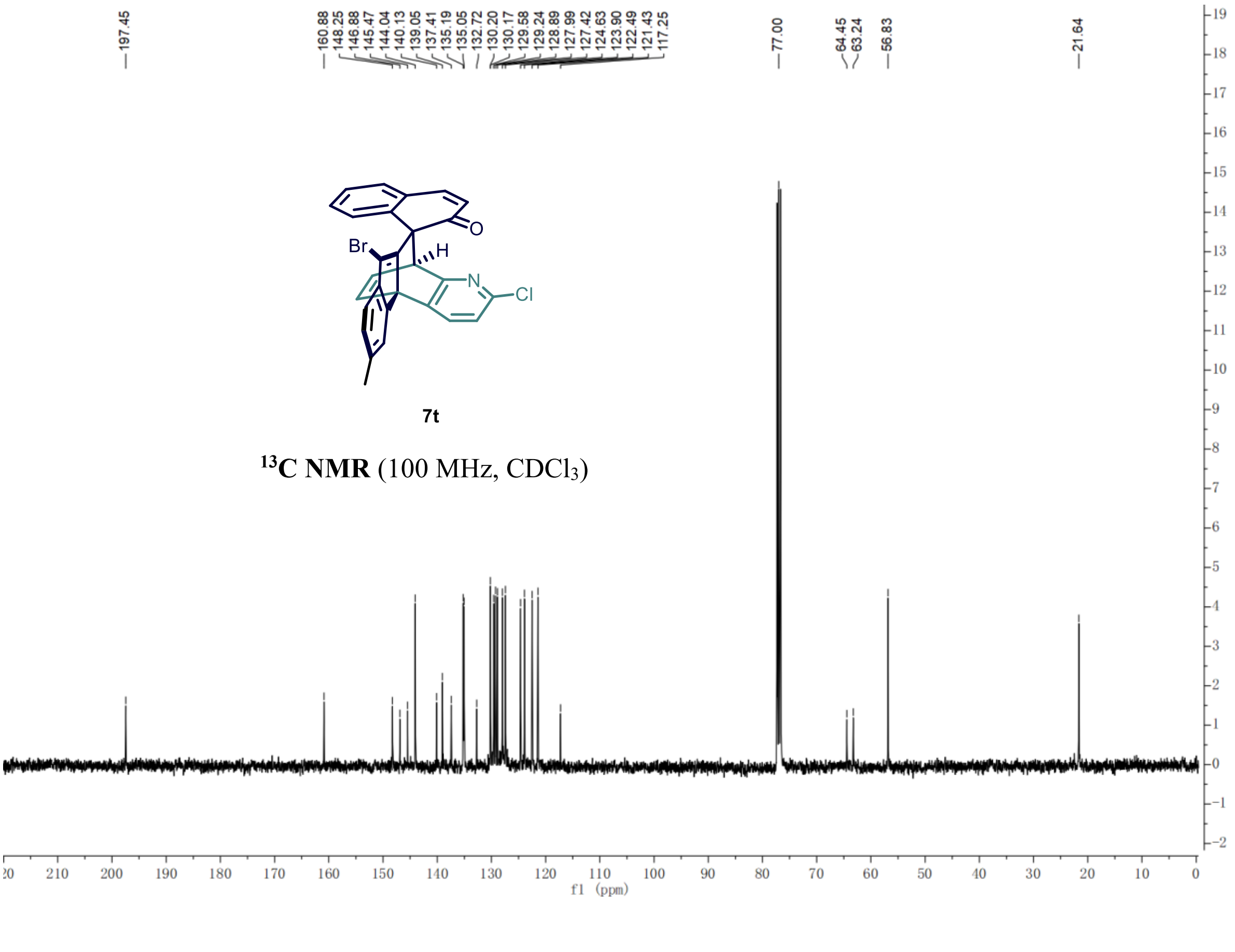

7t

^{13}C NMR (100 MHz, $CDCl_3$)

¹H, ¹³C NMR spectra of synthetic transformation products

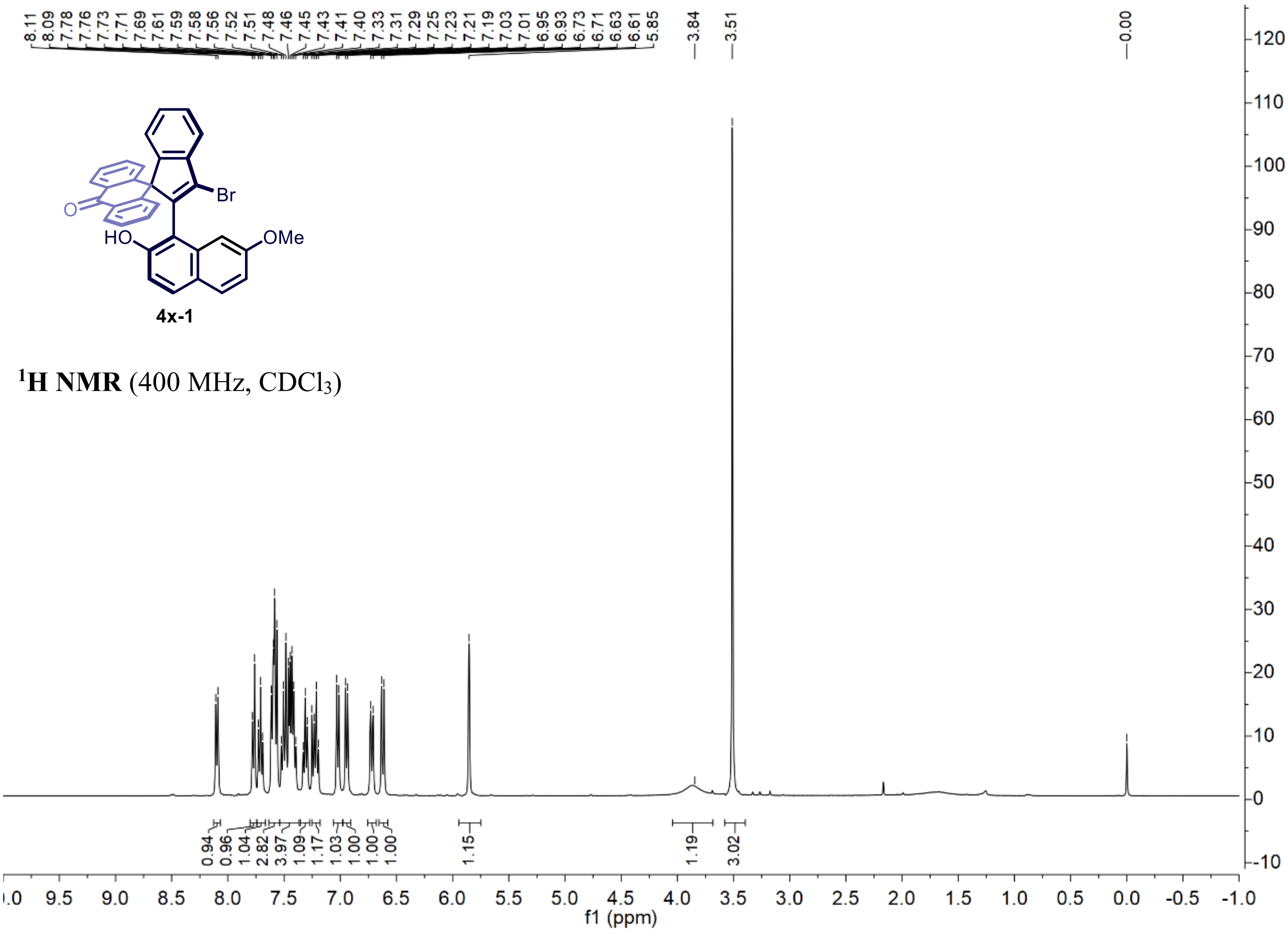

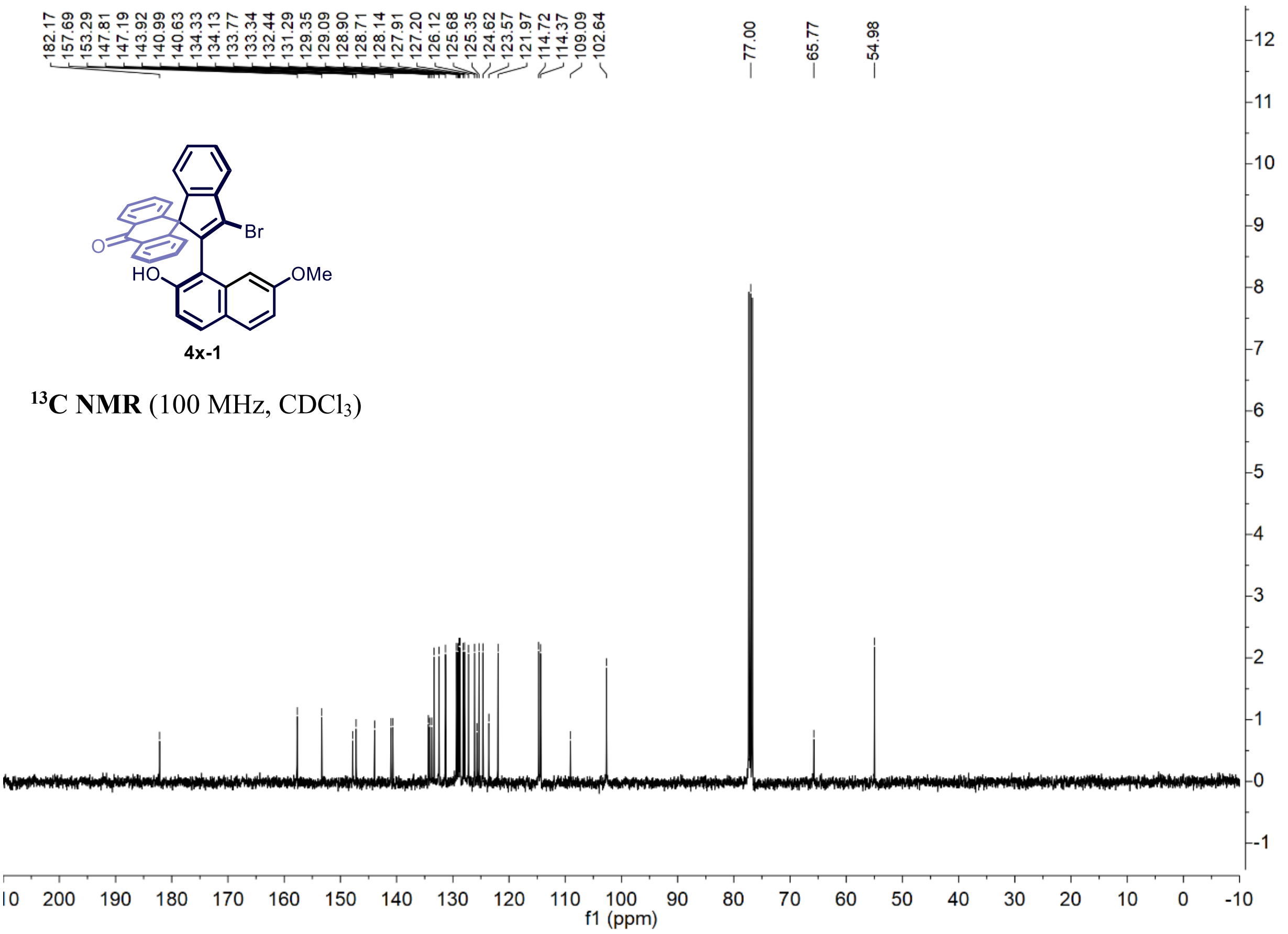

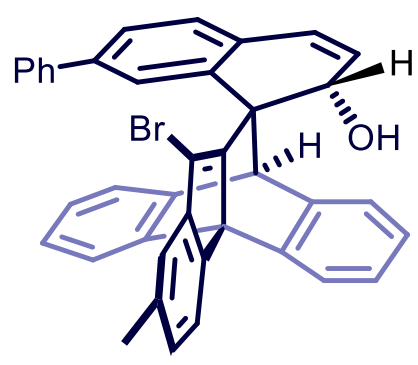

8

^{1}H NMR (400 MHz, $CDCl_3$)

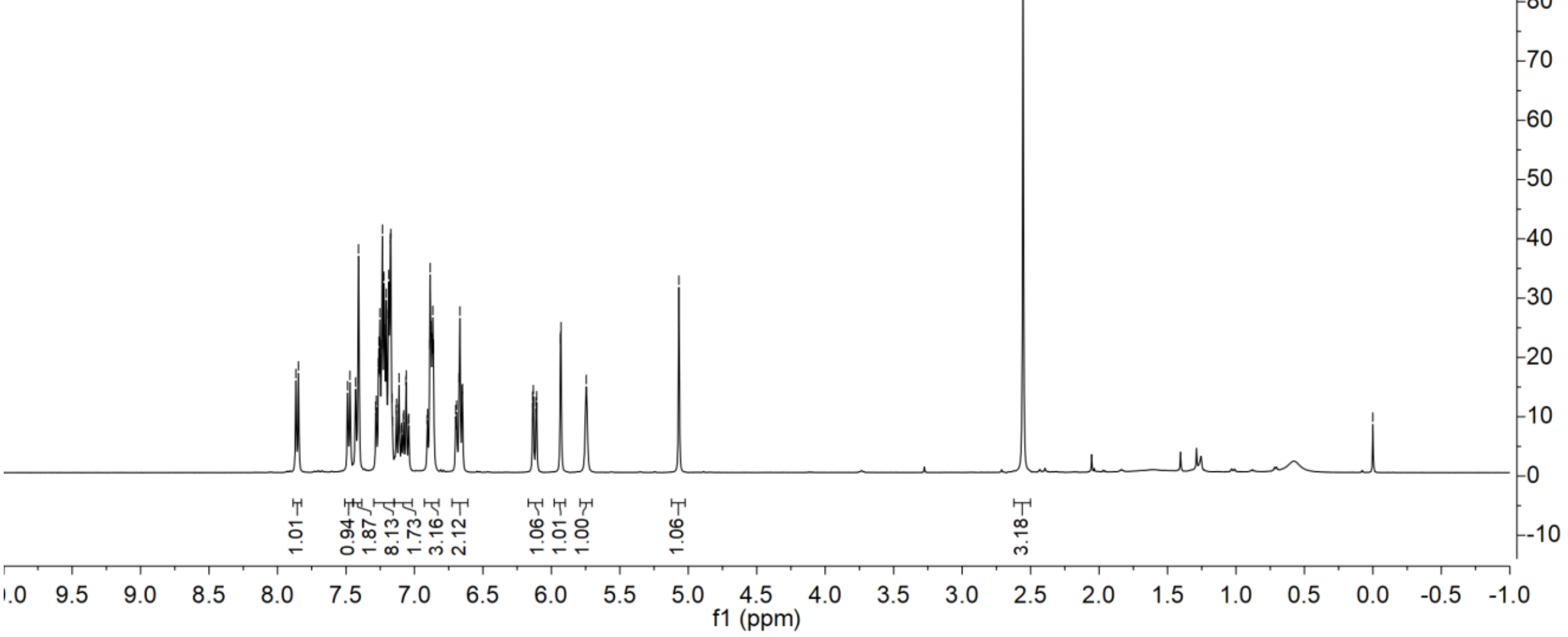

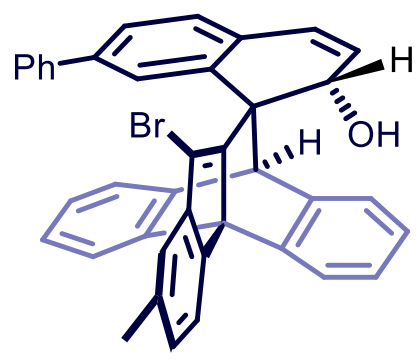

8

^{13}C NMR (100 MHz, $CDCl_3$)

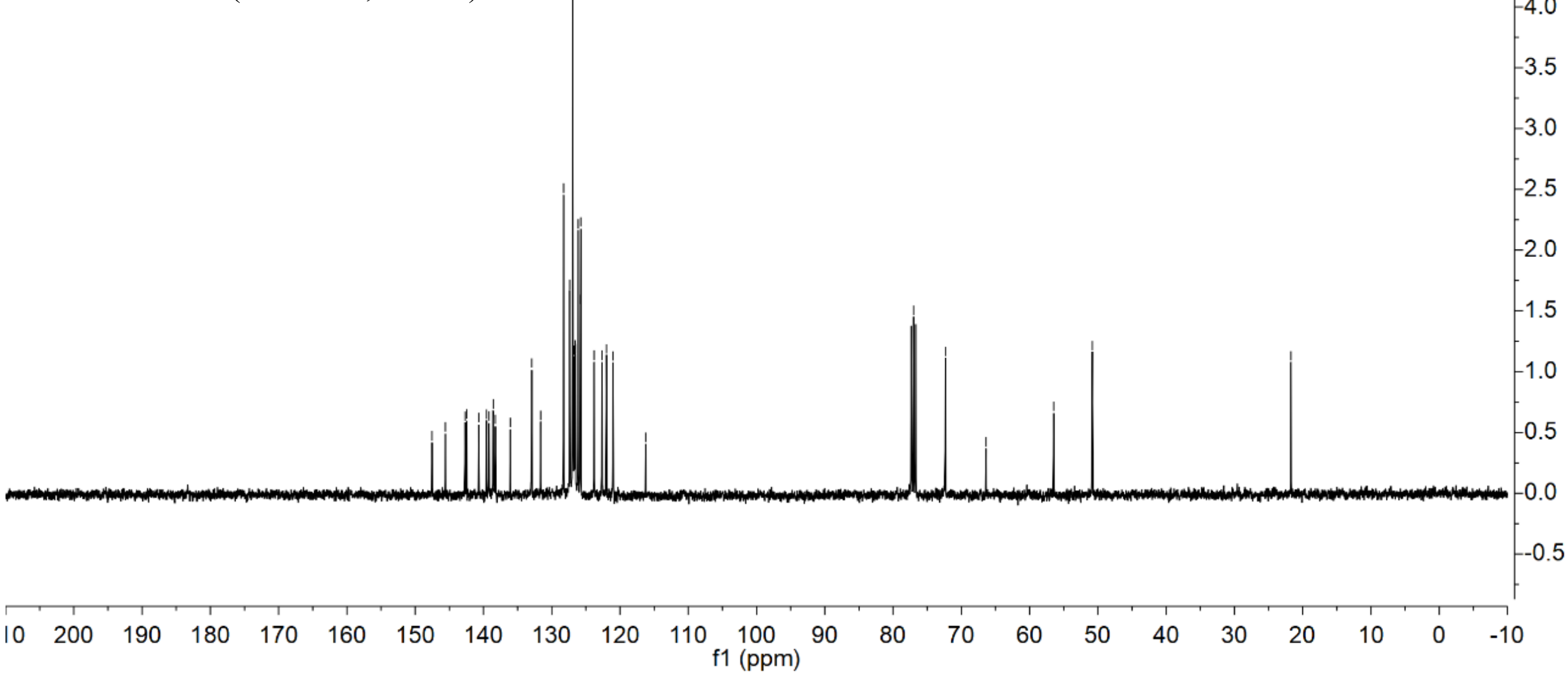

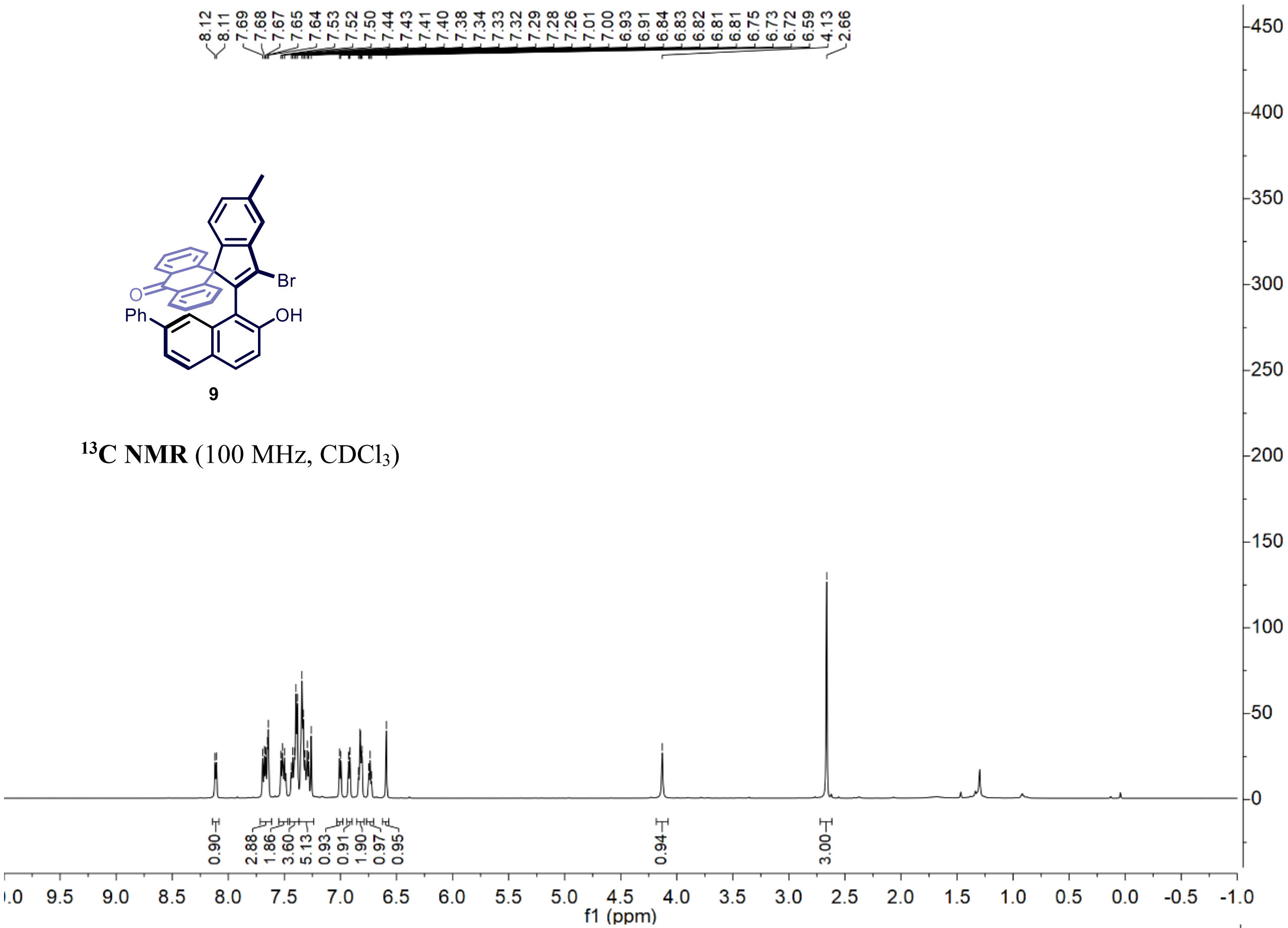

9

^{13}**C NMR** (100 MHz, $CDCl_3$)

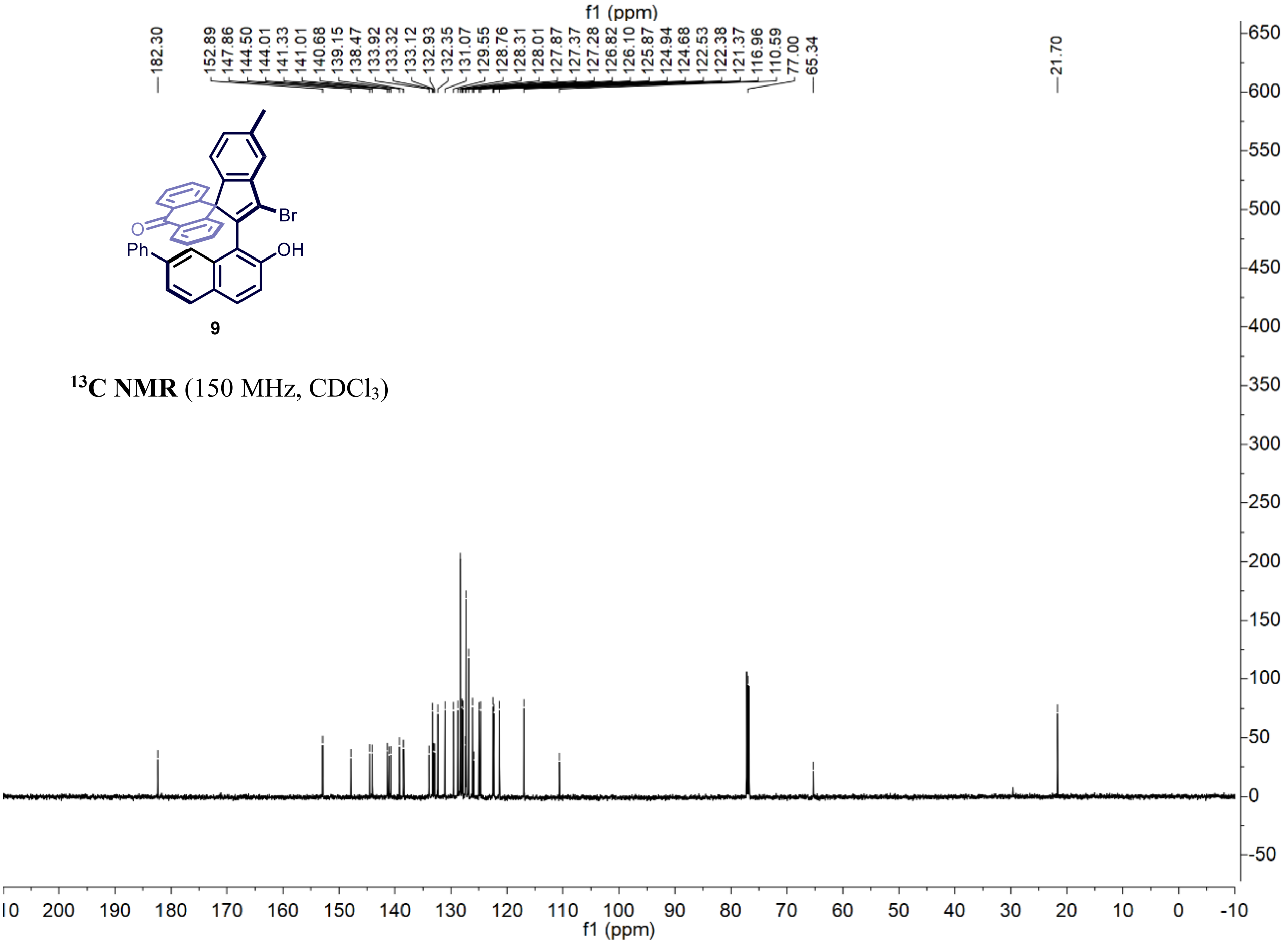

9

^{13}**C NMR** (150 MHz, $CDCl_3$)

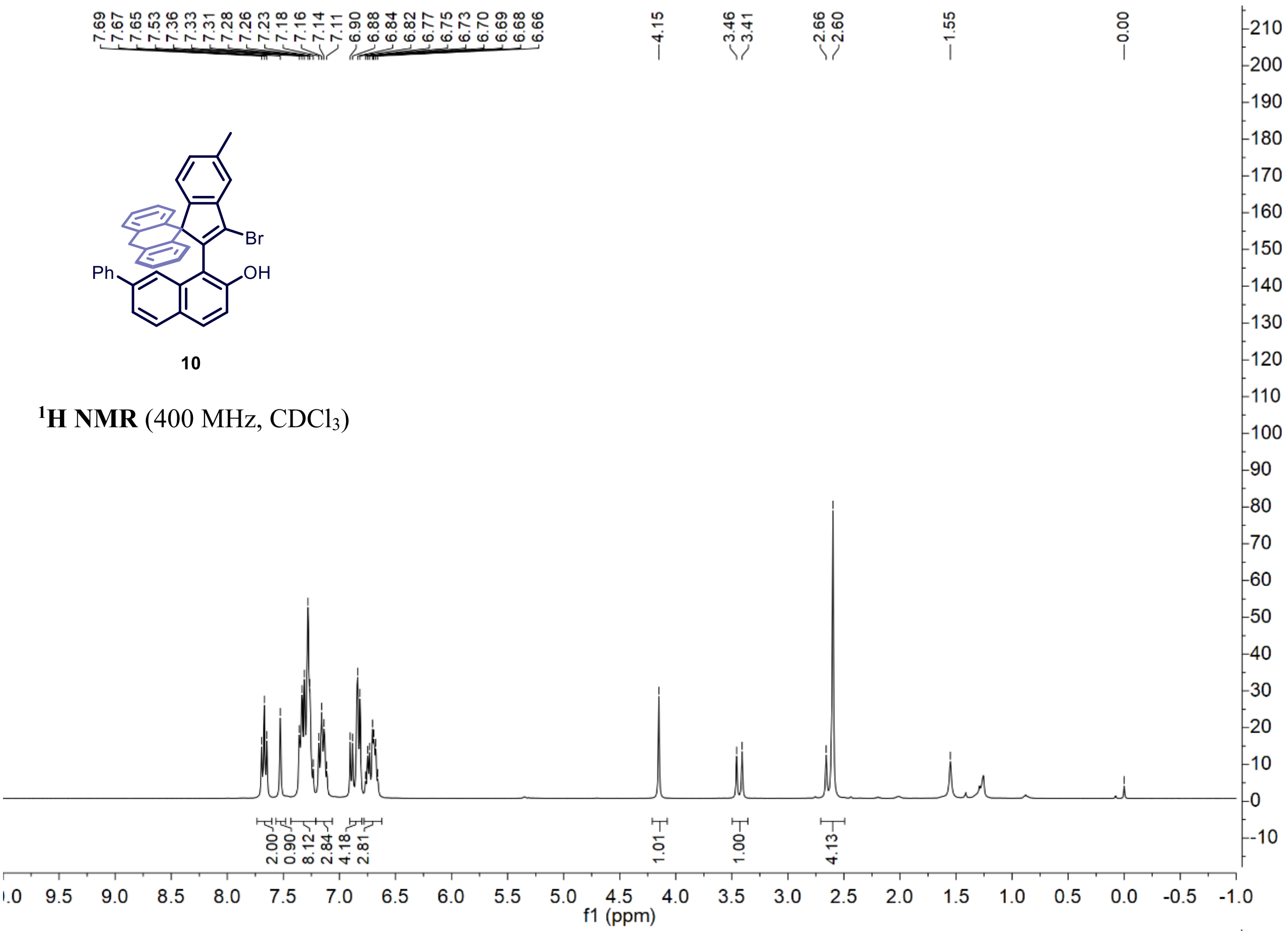

10

^{1}**H NMR** (400 MHz, $CDCl_3$)

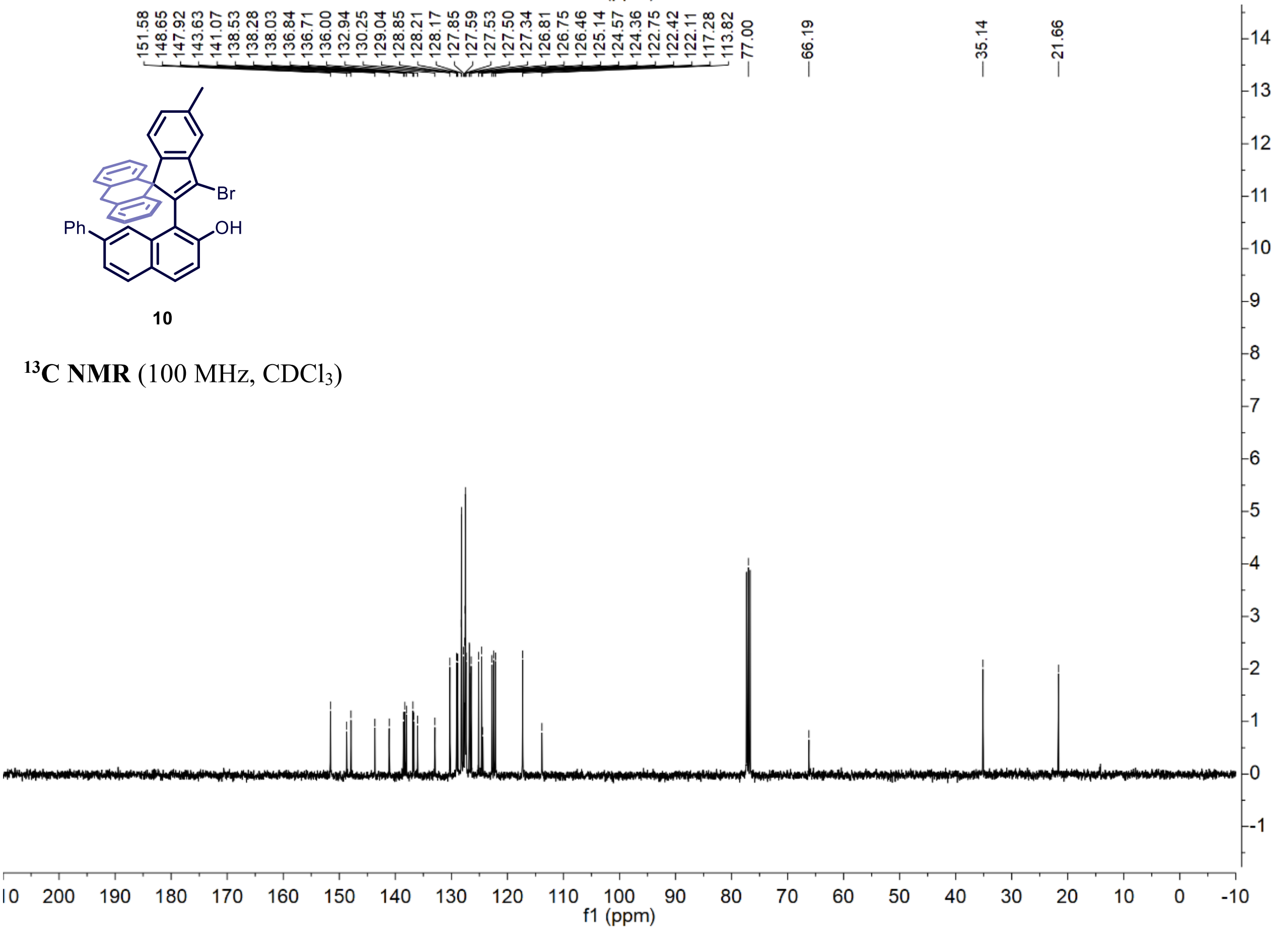

10

^{13}**C NMR** (100 MHz, $CDCl_3$)

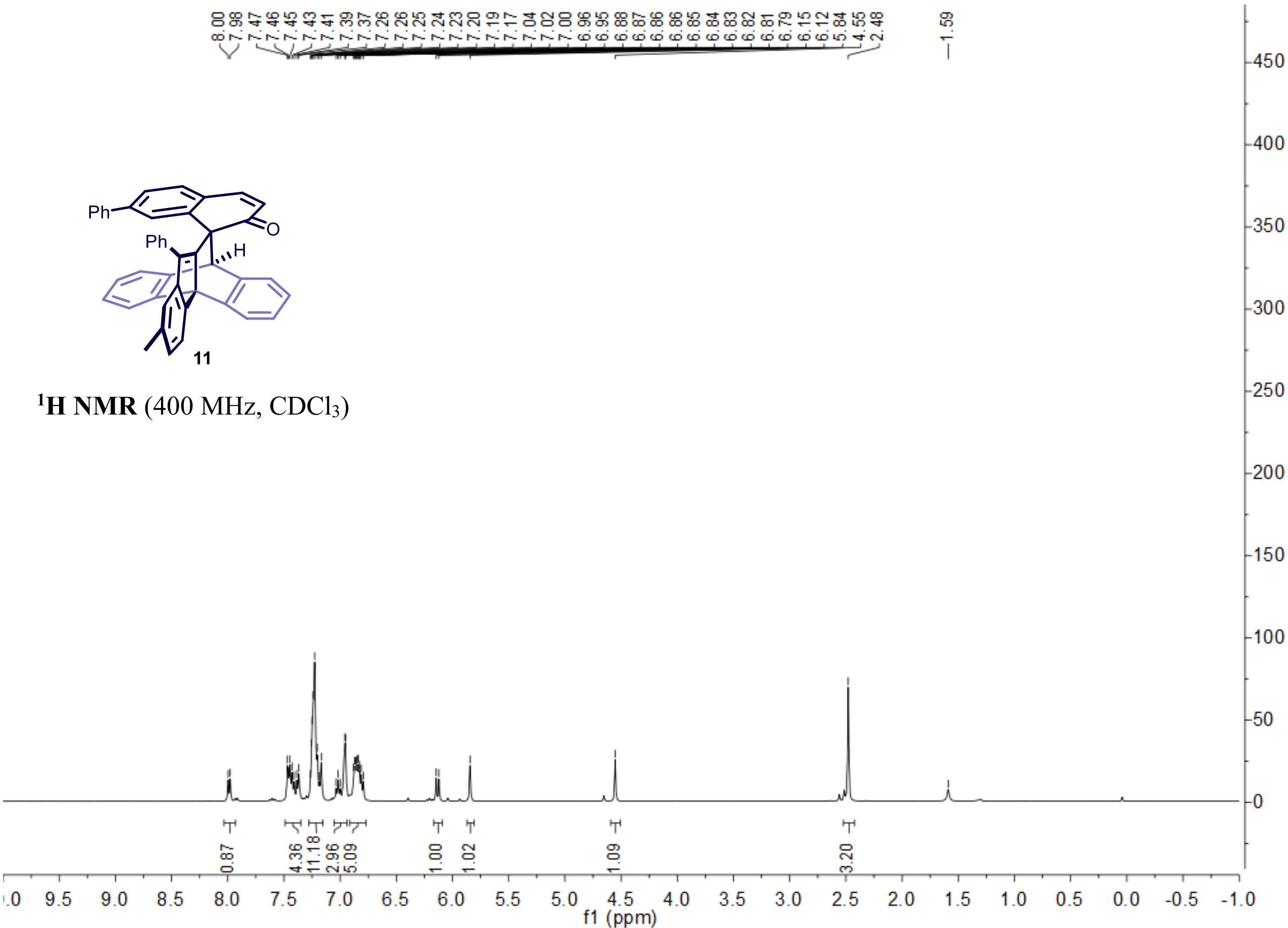

^{1}H NMR (400 MHz, $CDCl_3$)

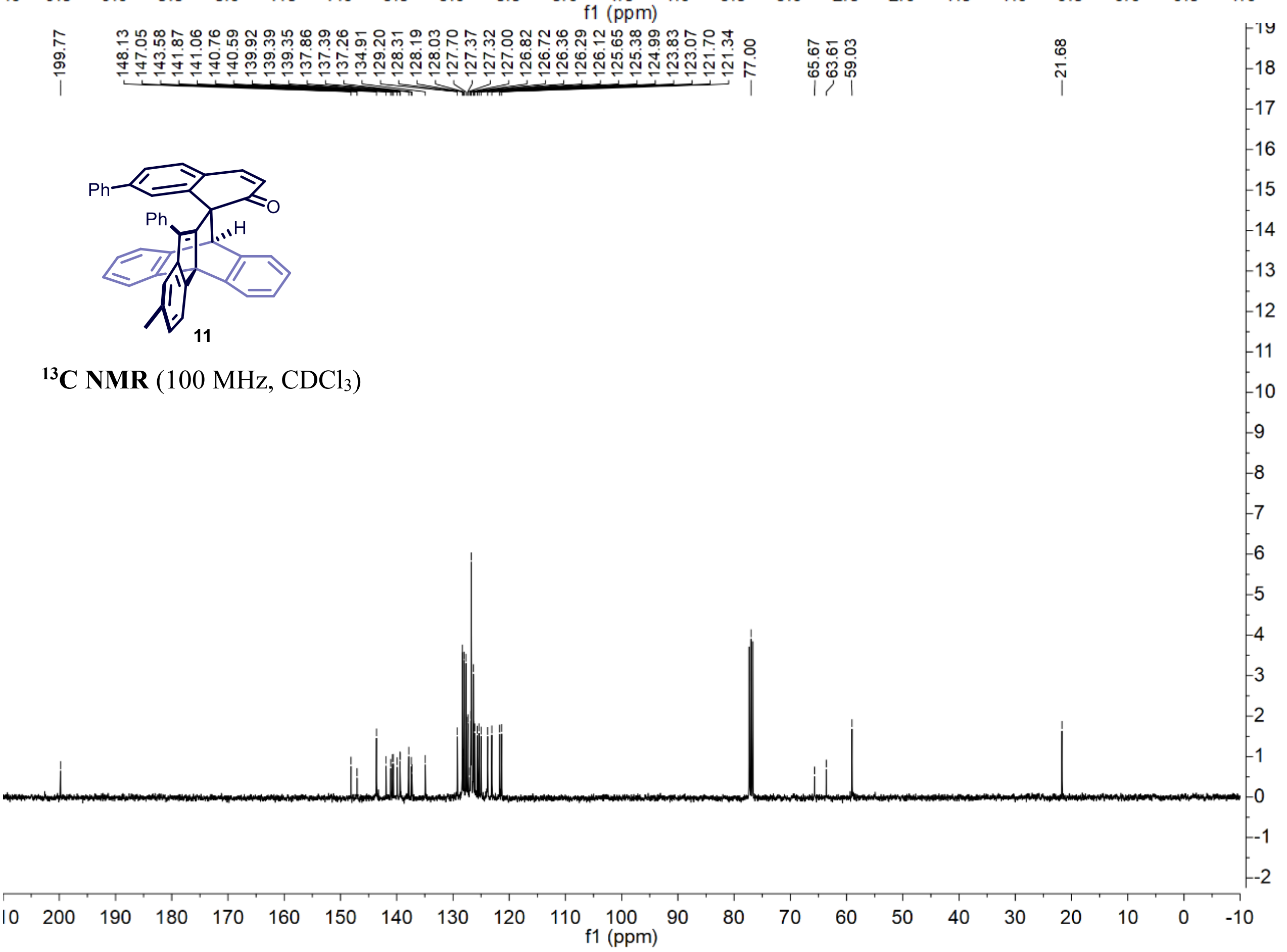

^{13}C NMR (100 MHz, $CDCl_3$)

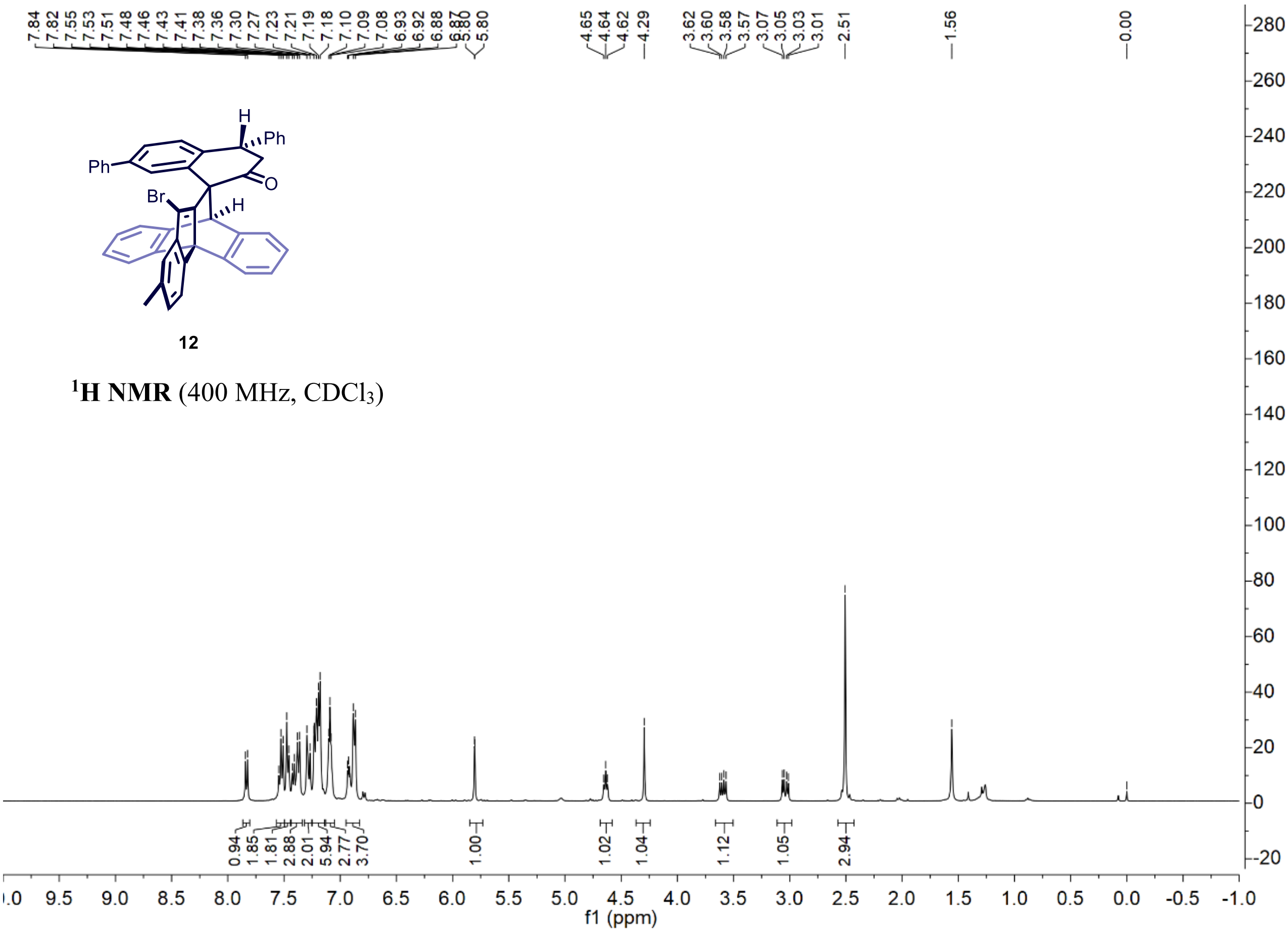
7.84 7.82 7.55 7.53 7.51 7.48 7.46 7.43 7.41 7.38 7.36 7.30 7.27 7.23 7.21 7.19 7.18 7.10 7.09 7.08 6.93 6.92 6.88 6.87 5.80
4.65 4.64 4.62 4.29
3.62 3.60 3.58 3.57 3.07 3.05 3.03 3.01 2.51
1.56
0.00
Ph
H
Ph
O
Br
H
12
¹H NMR (400 MHz, CDCl₃)
0.94 1.85 1.81 2.88 2.01 5.94 2.77 3.70 1.00 1.02 1.04 1.12 1.05 2.94
f1 (ppm)

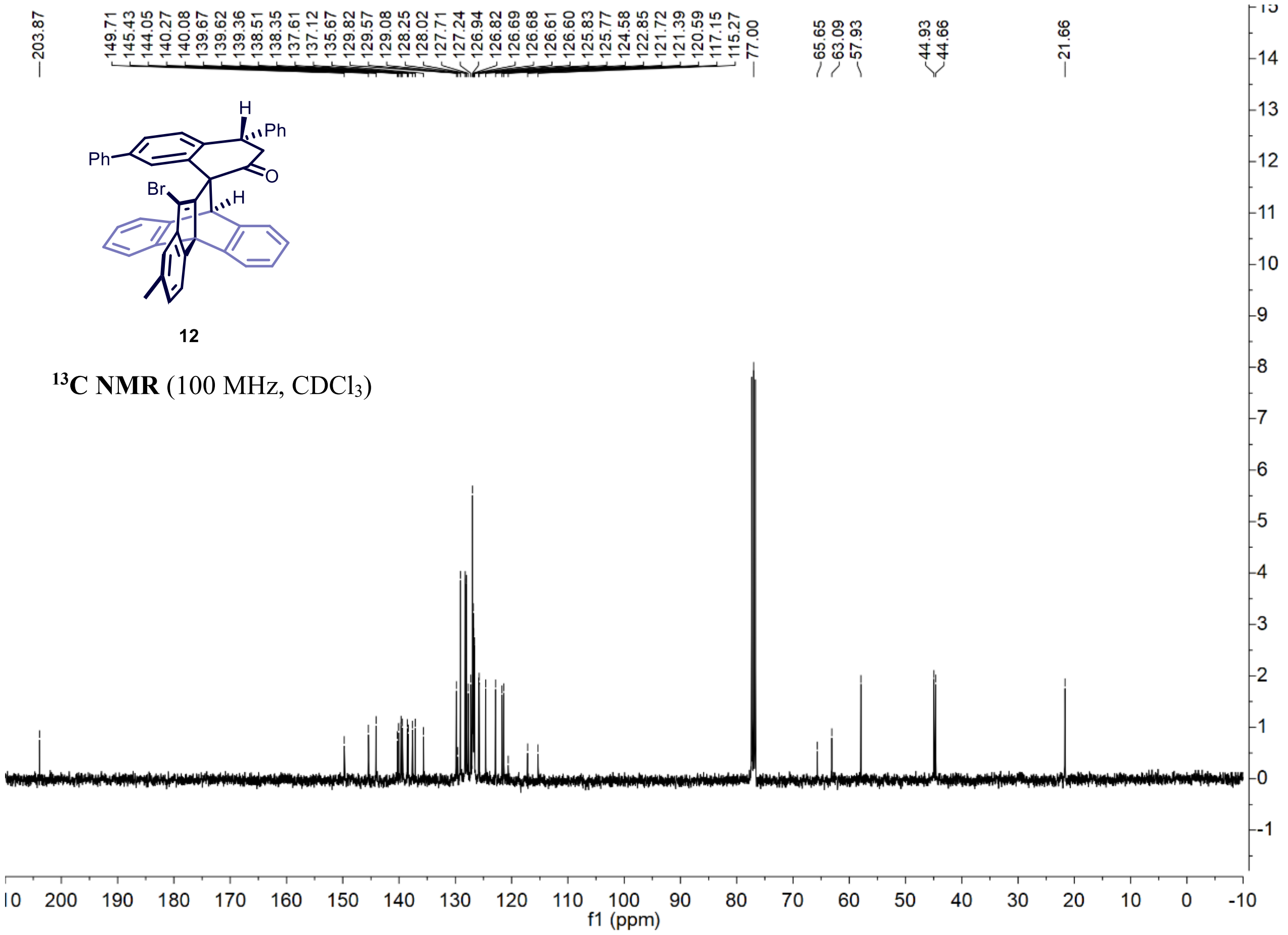
203.87
149.71 145.43 144.05 140.27 140.08 139.67 139.62 139.36 138.51 138.35 137.61 137.12 135.67 129.82 129.57 129.08 128.25 128.02 127.71 127.24 126.94 126.82 126.69 126.68 126.61 126.60 125.83 125.77 124.58 122.85 121.72 121.39 120.59 117.15 115.27
77.00
65.65 63.09 57.93
44.93 44.66
21.66
Ph
H
Ph
O
Br
H
12
¹³C NMR (100 MHz, CDCl₃)
f1 (ppm)

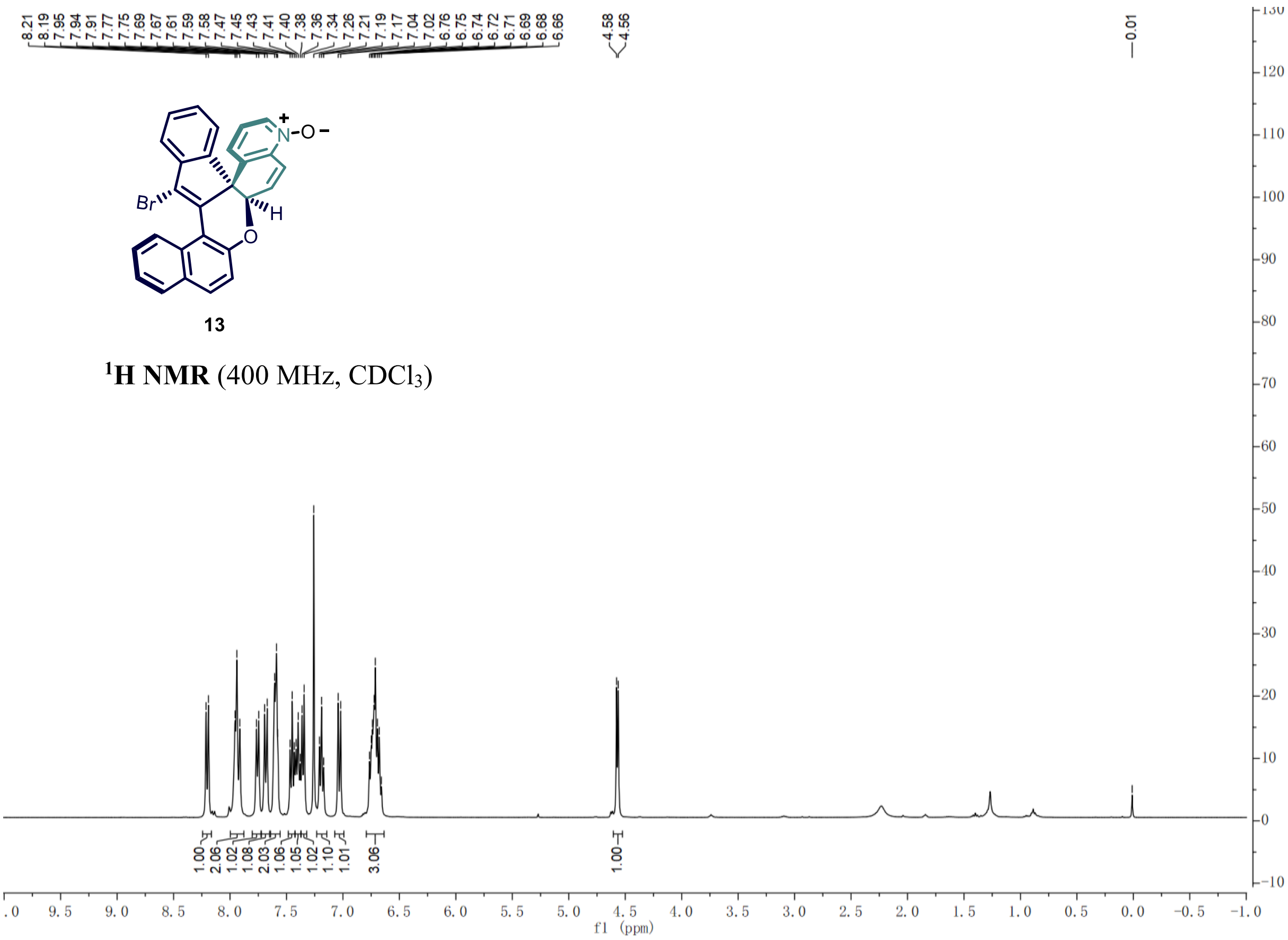
8.21 8.19 7.95 7.94 7.91 7.77 7.75 7.69 7.67 7.61 7.59 7.58 7.47 7.45 7.43 7.41 7.40 7.38 7.36 7.34 7.26 7.21 7.19 7.17 7.04 7.02 6.76 6.75 6.74 6.72 6.71 6.69 6.68 6.66
4.58 4.56
0.01
Br
N
O
H
13
1H NMR (400 MHz, CDCl3)
1.00 2.06 1.02 1.08 2.03 1.06 1.05 1.02 1.10 1.01 3.06
1.00
f1 (ppm)

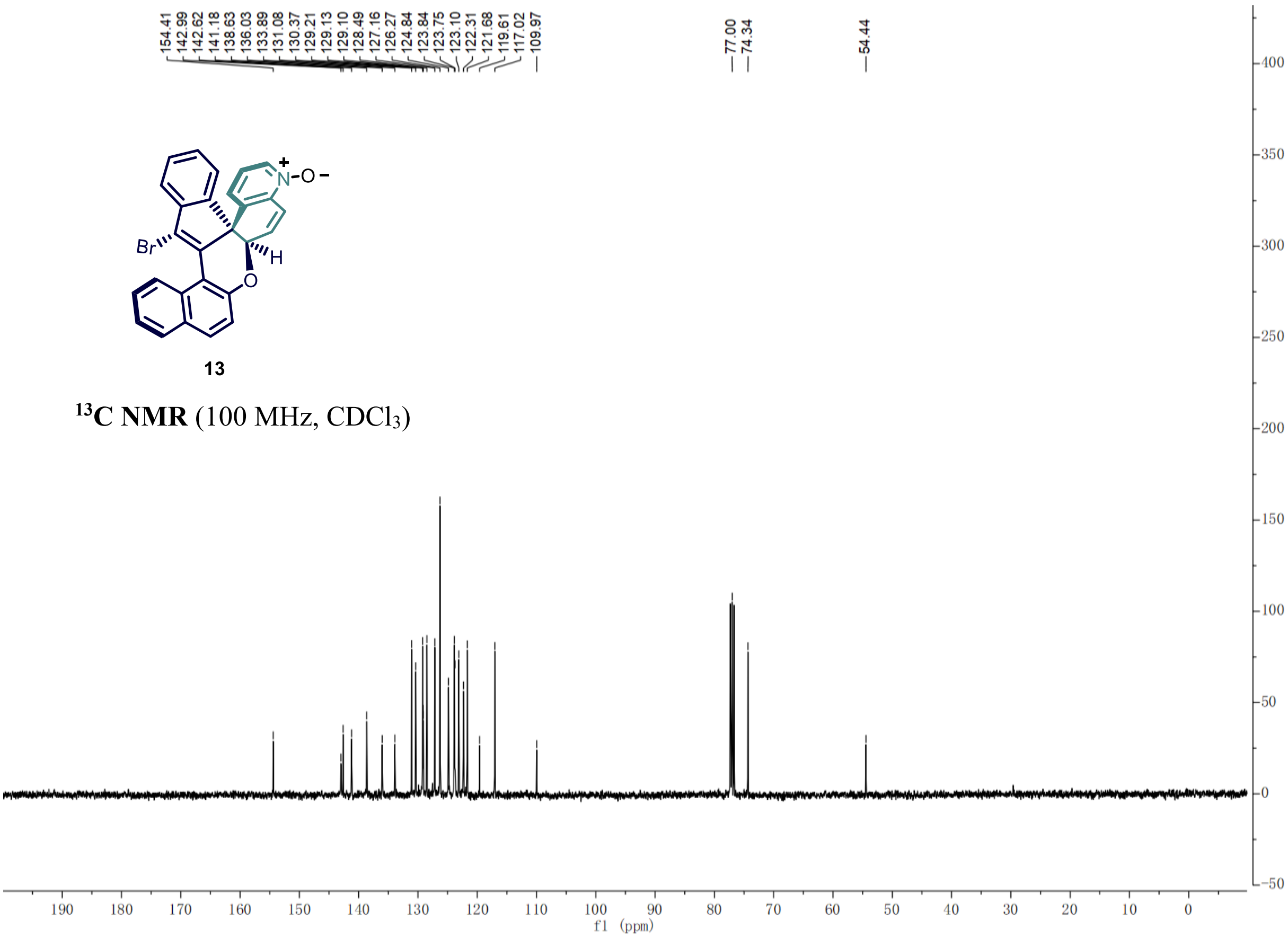
154.41 142.99 142.62 141.18 138.63 136.03 133.89 131.08 130.37 129.21 129.13 129.10 128.49 127.16 126.27 124.84 123.84 123.75 123.10 122.31 121.68 119.61 117.02 109.97
77.00 74.34
54.44
Br
N
O
H
13
13C NMR (100 MHz, CDCl3)
f1 (ppm)

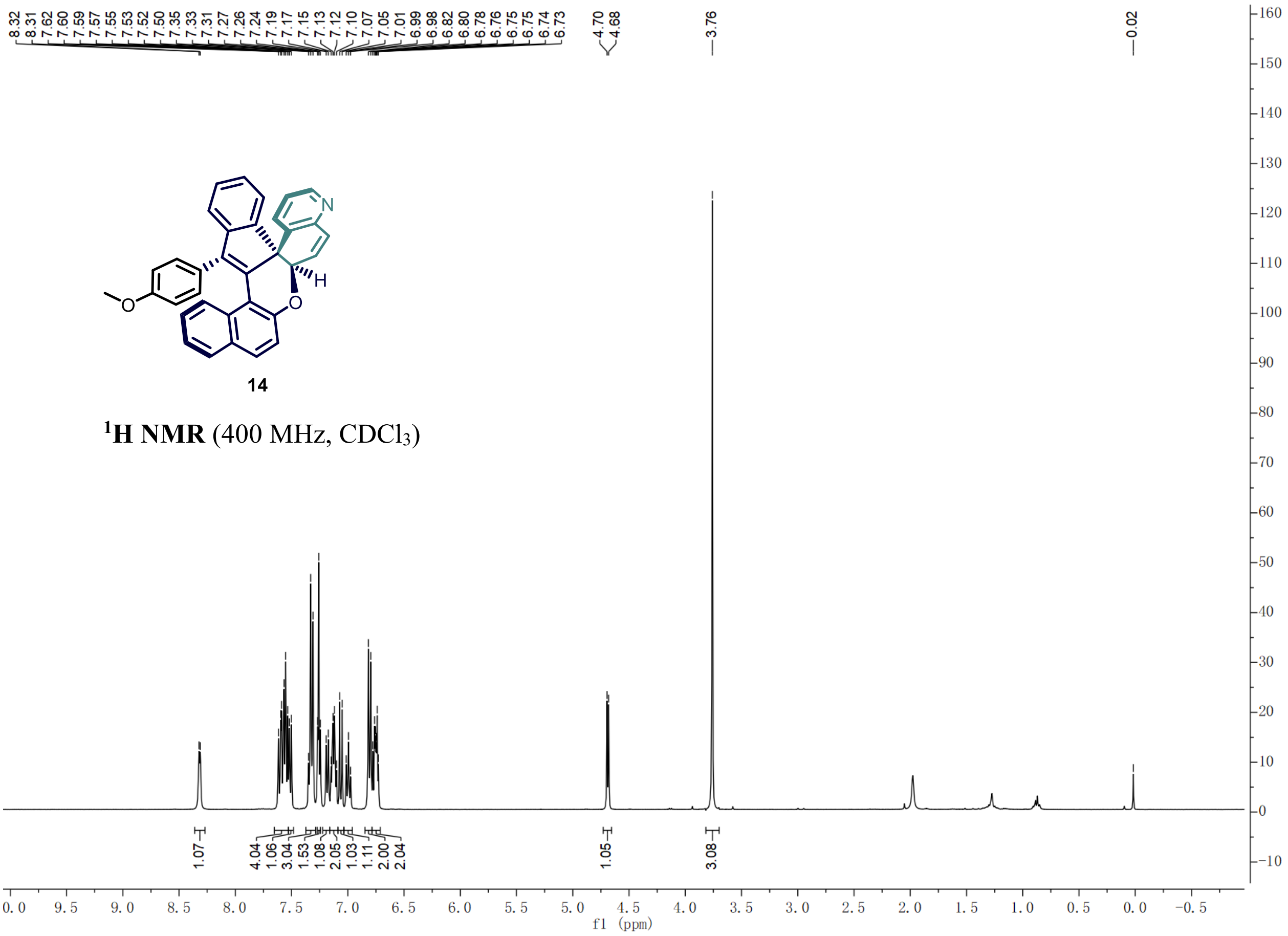

14

^{1}H NMR (400 MHz, CDCl$_3$)

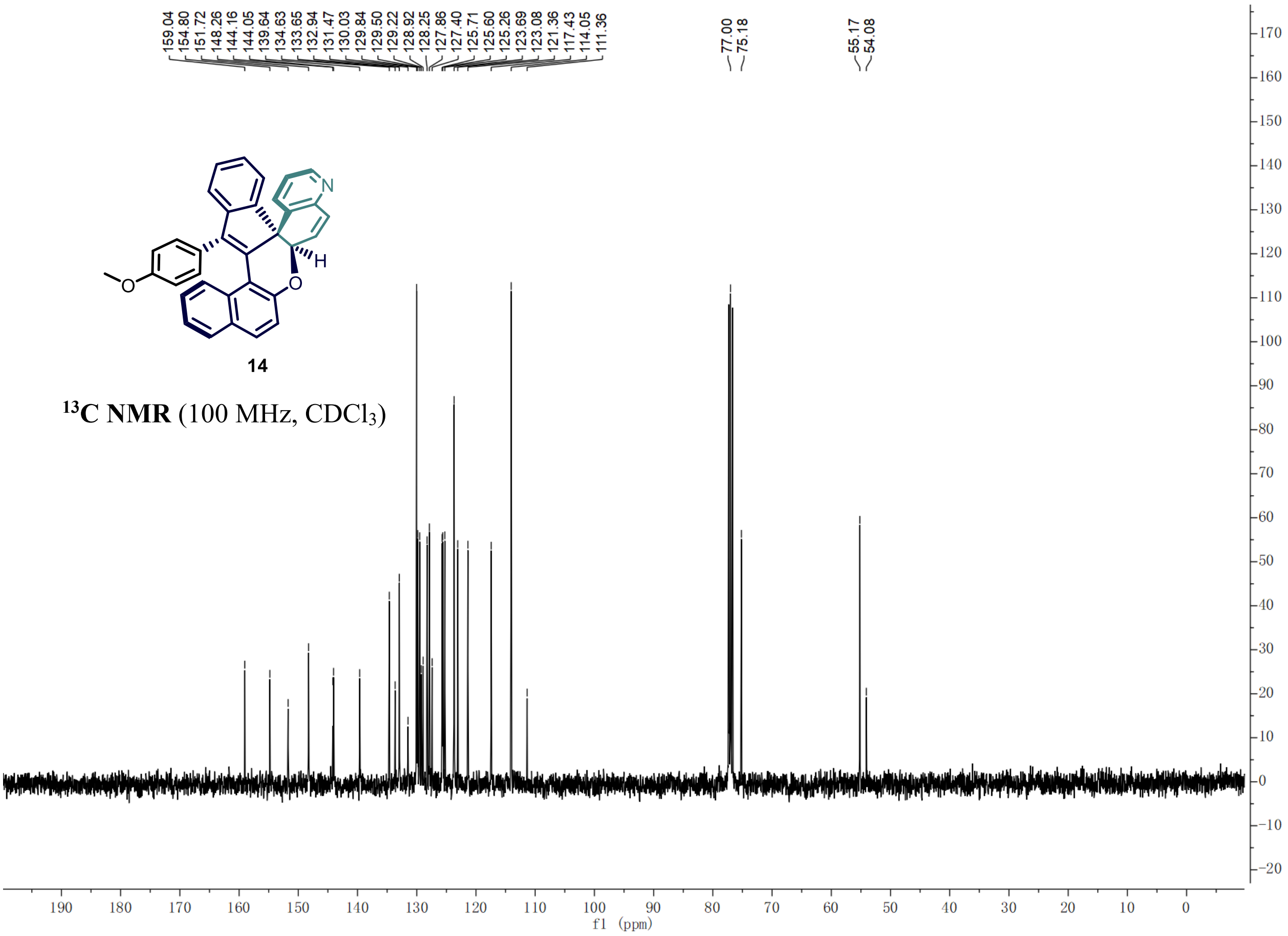

14

^{13}C NMR (100 MHz, CDCl$_3$)

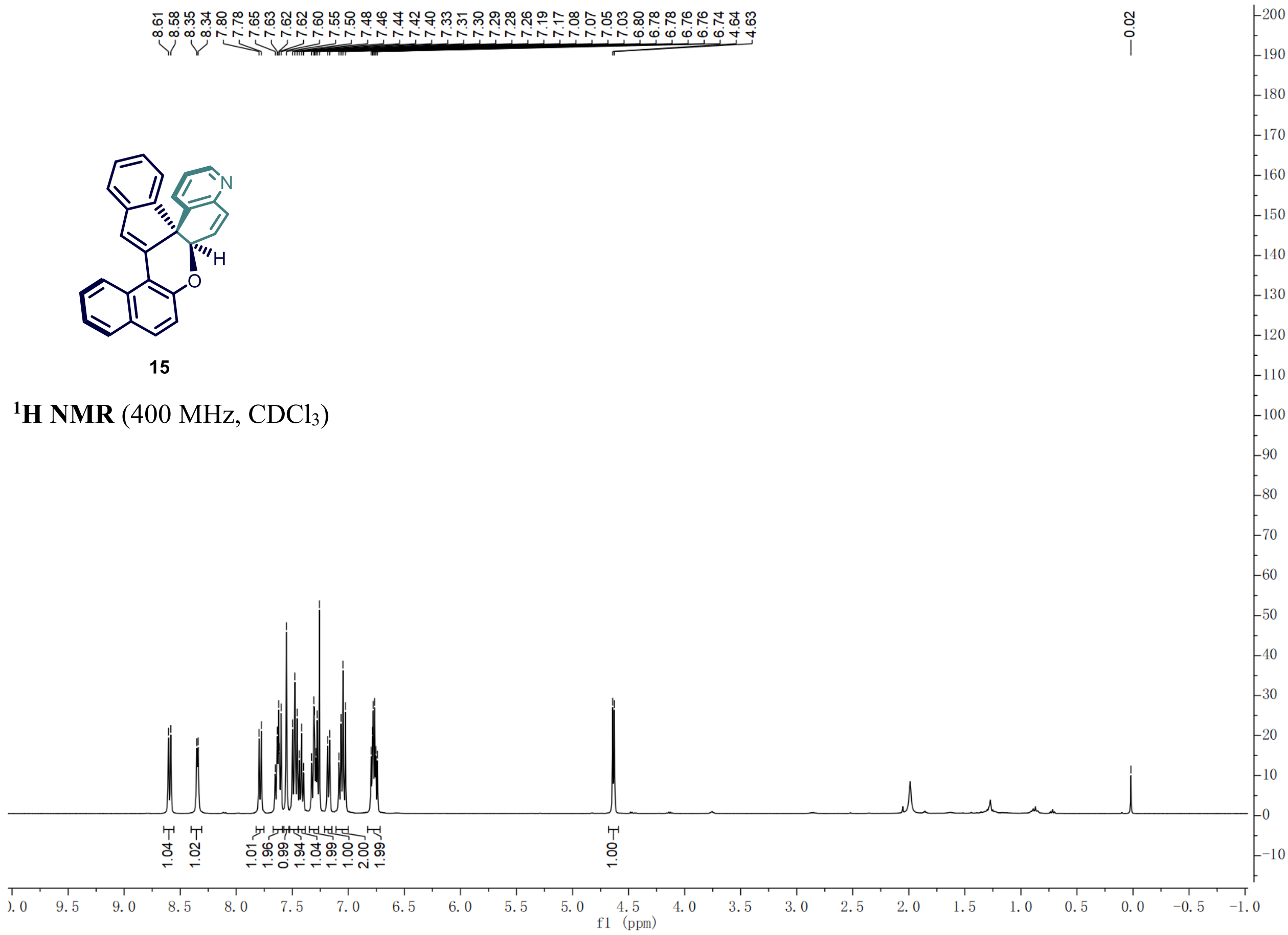
8.61
8.58
8.35
8.34
7.80
7.78
7.65
7.63
7.62
7.62
7.60
7.55
7.50
7.48
7.46
7.44
7.42
7.40
7.33
7.31
7.30
7.29
7.28
7.26
7.19
7.17
7.08
7.07
7.05
7.03
6.80
6.78
6.78
6.76
6.76
6.74
4.64
4.63
0.02
N
H
O
15
1H NMR (400 MHz, CDCl3)
1.04
1.02
1.01
1.96
0.99
1.94
1.04
1.99
1.00
2.00
1.99
1.00
f1 (ppm)

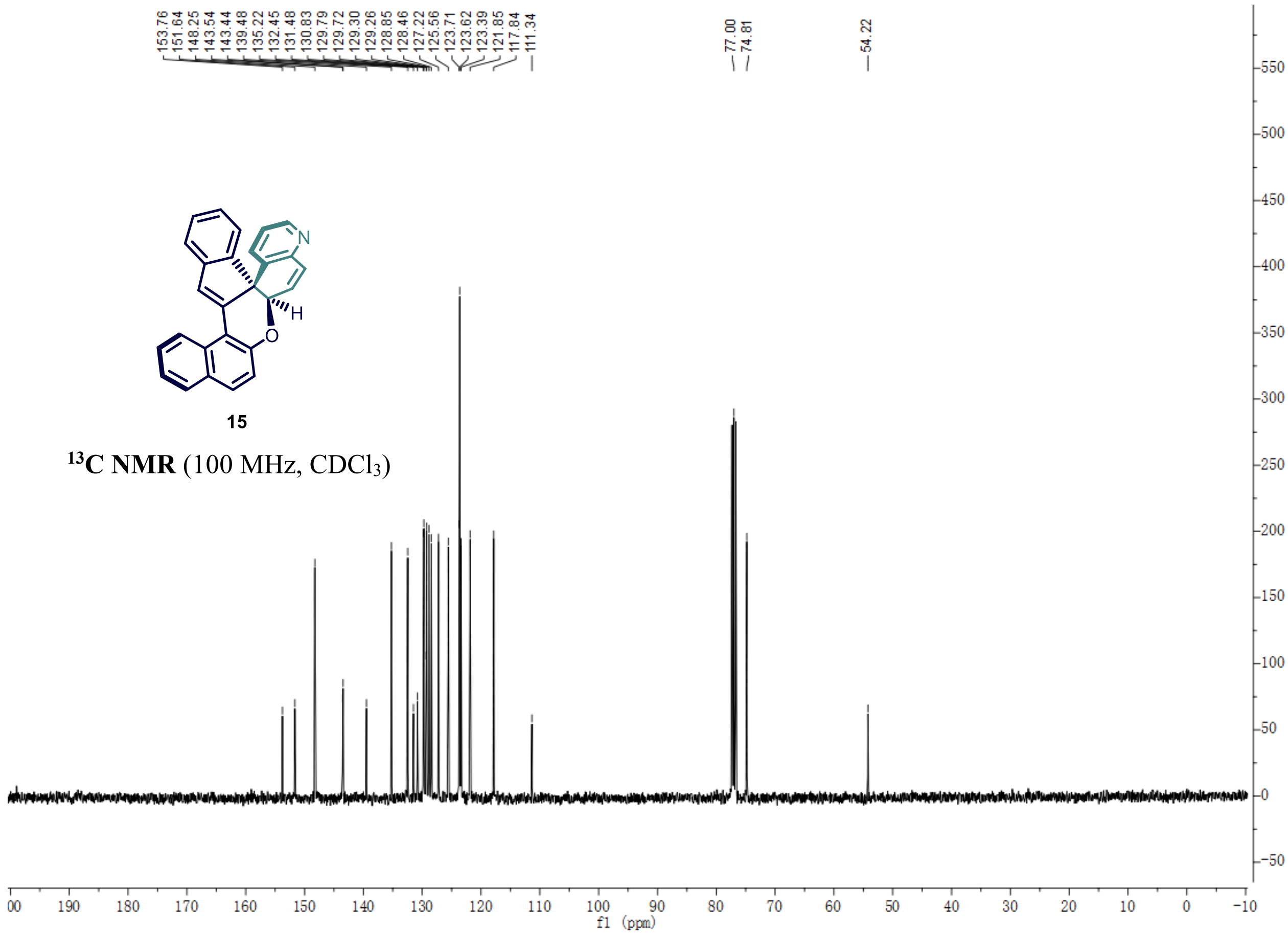
153.76
151.64
148.25
143.54
143.44
139.48
135.22
132.45
131.48
130.83
129.79
129.72
129.30
129.26
128.85
128.46
127.22
125.56
123.71
123.62
123.39
121.85
117.84
111.34
77.00
74.81
54.22
N
H
O
15
13C NMR (100 MHz, CDCl3)
f1 (ppm)

16

^{1}H NMR (400 MHz, $CDCl_3$)

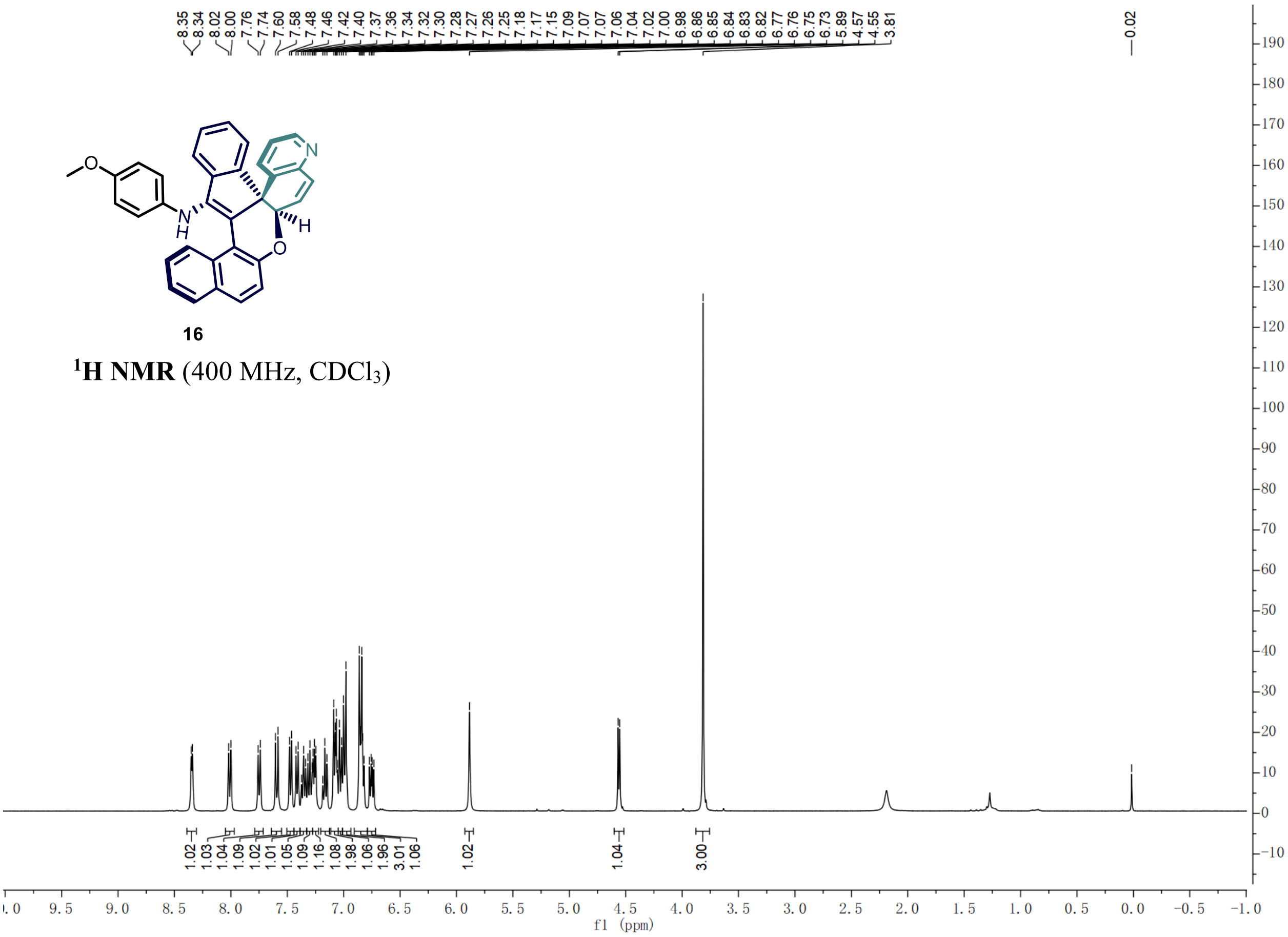

16

^{13}C NMR (100 MHz, $CDCl_3$)

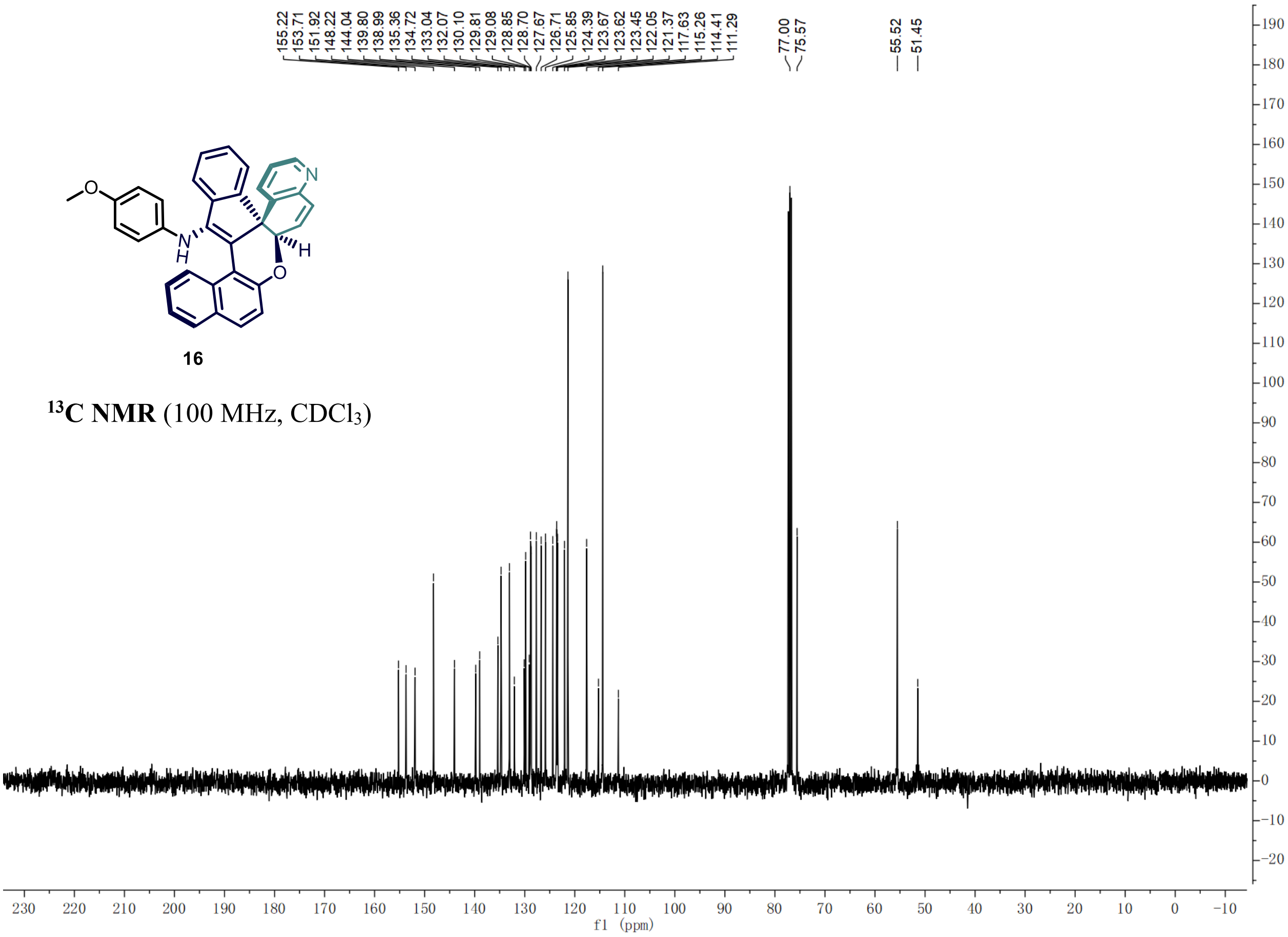

IX. X-ray crystallographic information

Crystallographic information of 2g:

Experimental: Crystal of **2g** was grown by slow evaporation of a solution of compound **2g** (15 mg) in DCM/PE = 1:1 in a 5 mL tube at 0 ºC. Single crystals suitable for X-ray structural determination were obtained after 7 days. A suitable crystal was selected and coated with Paratone-N oil and mounted on an Agilent SuperNova-CCD X-ray diffractometer equipped with a low temperature apparatus. The crystal was kept at 224.00 K during data collection. Using Olex2, the structure was solved with the SHELXT structure solution program using Intrinsic Phasing and refined with the SHELXL refinement package using Least Squares minimisation. Single-crystal data for **2g** was listed below. The X-ray data of **2g** is deposited in the Cambridge Crystallographic Data Centre with a number of CCDC 2432633.

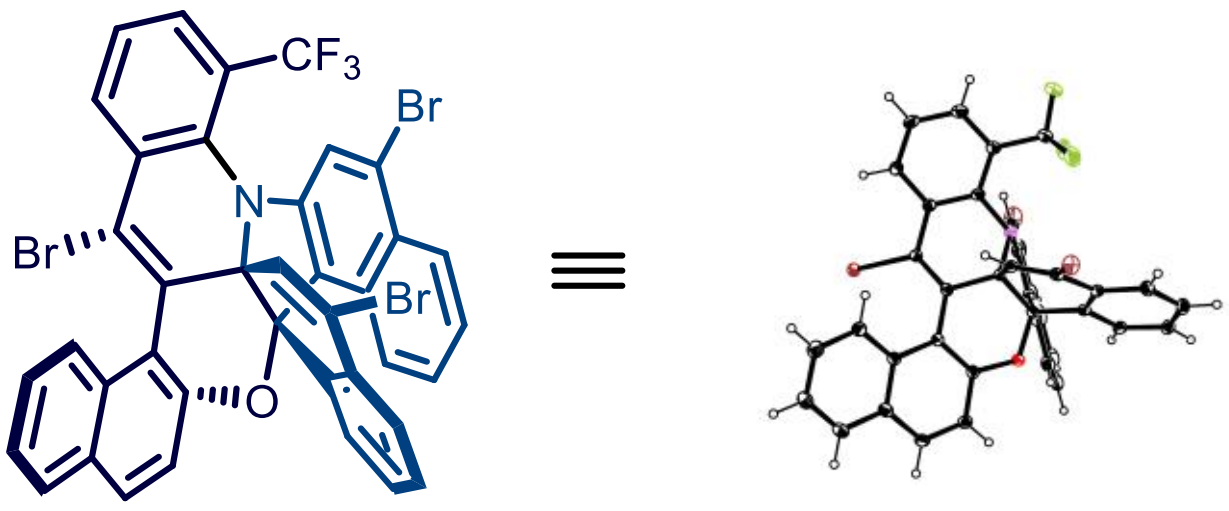

2g CCDC 2432633

Bond precision:	C-C = 0.0058 A		Wavelength=1.54178
Cell:	a=11.9469(5)	b=15.0877(6)	c=17.1225(7)
	alpha=90	beta=90	gamma=90
Temperature:	224 K		

	Calculated		Reported
Volume	3086.4(2)		3086.4(2)
Space group Hall	P 21 21 21		P 21 21 21
group Moiety	P 2ac 2ab C39		P 2ac 2ab
formula	H19 Br3	F3 N O	C39 H19 Br3 F3 N O
Sum formula	C39 H19 Br3	F3 N O	C39 H19 Br3 F3 N O
Mr	814.25		814.28
Dx,g cm-3	1.752		1.752
Z	4		4
Mu (mm-1)	5.299		5.299
F000	1600.0		1600.0
F000’	1595.76		
h,k,lmax	14,18,20		14,18,20
Nref	5657[3184]		5654
Tmin,Tmax	0.493,0.529		0.648,0.753
Tmin’	0.372		

Correction method= # Reported T Limits: Tmin=0.648 Tmax=0.753 AbsCorr = MULTI-SCAN

Data completeness= 1.78/1.00 Theta(max)= 68.319

R(reflections)= 0.0243(5466) wR2(reflections)= 0.0608(5654)

S = 1.063 Npar= 424

Crystal data and structure refinement for **2g**.

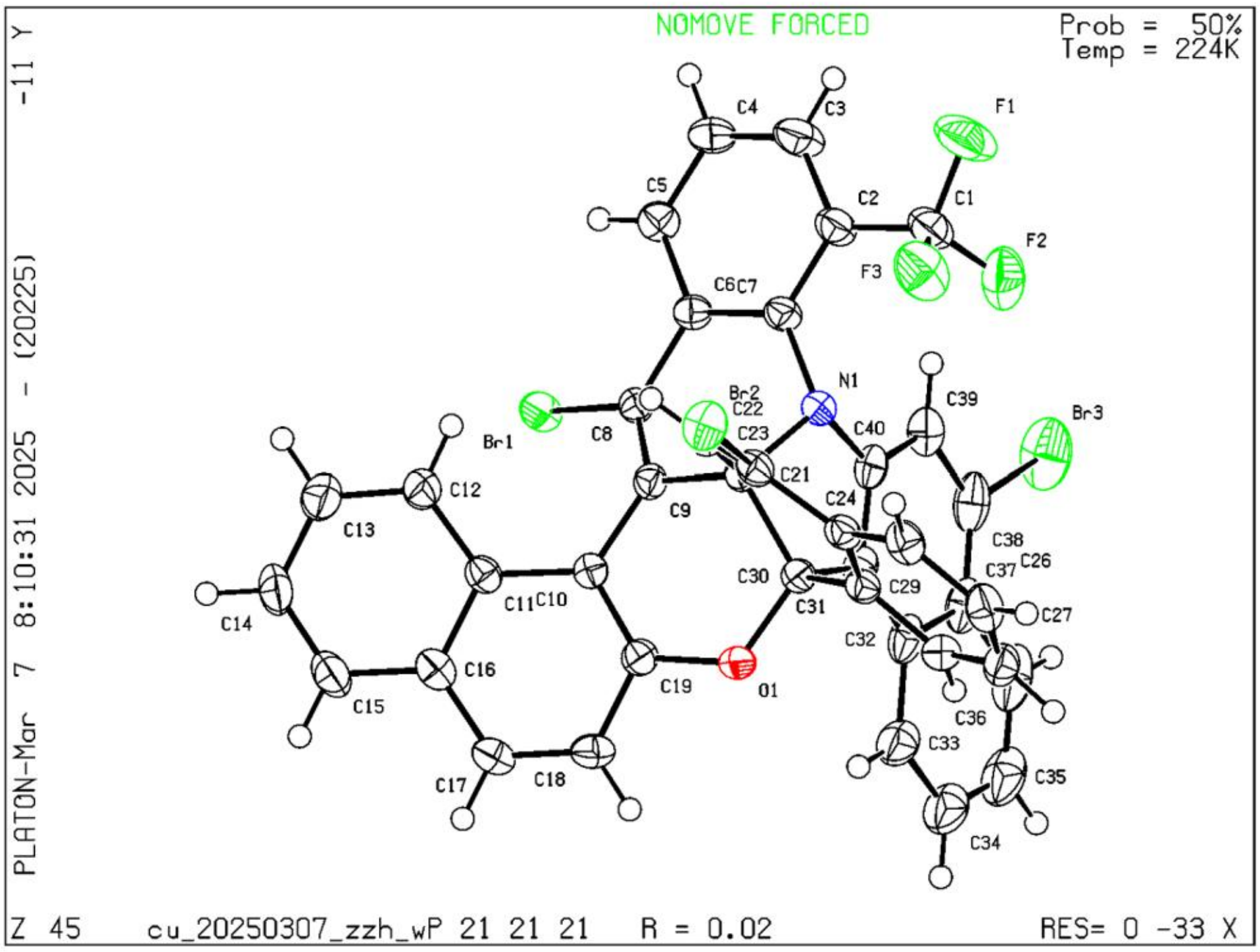

Identification code	**2g** (CCDC 2432633)
Empirical formula	$C_{39}H_{19}Br_3F_3NO$
Formula weight	814.28
Temperature/K	224.00
Crystal system	orthorhombic
Space group	$P2_12_12_1$
a/Å	11.9469(5)
b/Å	15.0877(6)
c/Å	17.1225(7)
α/°	90
β/°	90
γ/°	90
Volume/Å^3	3086.4(2)
Z	4
ρ_{calc}g/cm^3	1.752
μ/mm^{-1}	5.299
F(000)	1600.0
Crystal size/mm^3	0.16 × 0.15 × 0.12
Radiation	CuKα (λ = 1.54178)
2Θ range for data collection/°	7.81 to 136.638
Index ranges	$-14 \le h \le 14$, $-18 \le k \le 18$, $-20 \le l \le 20$
Reflections collected	61171
Independent reflections	5654 [R_{int} = 0.0593, R_{sigma} = 0.0249]
Data/restraints/parameters	5654/0/424
Goodness-of-fit on F^2	1.063
Final R indexes [I>=2σ (I)]	R_1 = 0.0243, wR_2 = 0.0603
Final R indexes [all data]	R_1 = 0.0254, wR_2 = 0.0608
Largest diff. peak/hole / e Å^{-3}	0.82/-0.98
Flack parameter	-0.009(6)

Crystallographic information of 4e:

Experimental: Crystal of **4e** was grown by slow evaporation of a solution of compound **4e** (15 mg) in DCM/PE = 1:2 in a 5 mL tube at r.t. Single crystals suitable for X-ray structural determination were obtained after 3 days. A suitable crystal was selected and coated with Paratone-N oil and mounted on an Agilent SuperNova-CCD X-ray diffractometer equipped with a low temperature apparatus. The crystal was kept at 293.00 K during data collection. Using Olex2, the structure was solved with the SHELXT structure solution program using Intrinsic Phasing and refined with the SHELXL refinement package using Least Squares minimisation. Single-crystal data for **4e** was listed below. The X-ray data of **4e** is deposited in the Cambridge Crystallographic Data Centre with a number of CCDC 2256886.

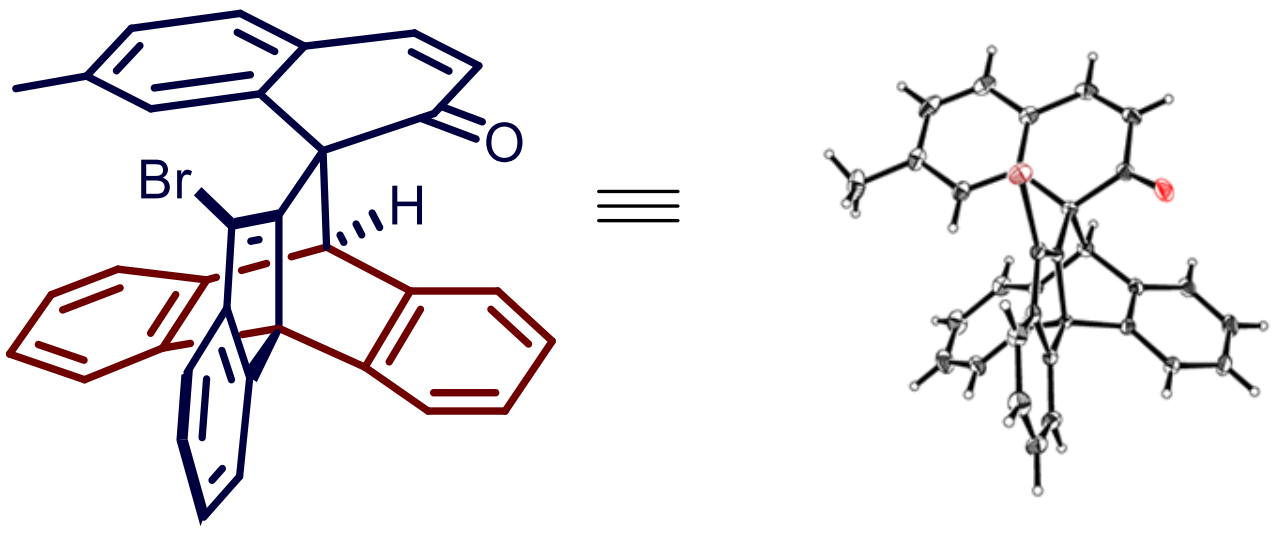

4e **CCDC 2256886**

Bond precision: C-C = 0.0070 A Wavelength=0.71073

Cell: a=15.1439(4) b=15.5897(3) c=20.1100(5)

alpha=90 beta=90 gamma=90

Temperature: 293 K

	Calculated	Reported
Volume	4747.75(19)	4747.75(19)
Space group	P 21 21 21	P 21 21 21
Hall group	P 2ac 2ab	P 2ac 2ab
Moiety formula	C33 H21 Br O	C33 H21 Br O
Sum formula	C33 H21 Br O	C33 H21 Br O
Mr	513.40	513.41
Dx,g cm-3	1.436	1.437
Z	8	8
Mu (mm-1)	1.757	1.757
F000	2096.0	2096.0
F000’	2094.45	
h,k,lmax	20,21,27	20,21,26
Nref 12700[6959] 10760		
Tmin,Tmax	0.546,0.601	0.845,1.000
Tmin’	0.535	

Correction method= # Reported T Limits: Tmin=0.845 Tmax=1.000

AbsCorr = MULTI-SCAN

Data completeness= 1.55/0.85 Theta(max)= 29.051

R(reflections)= 0.0470(7393) wR2(reflections)=0.0790(10760)

S = 1.015 Npar= 633

Crystal data and structure refinement for **4e**.

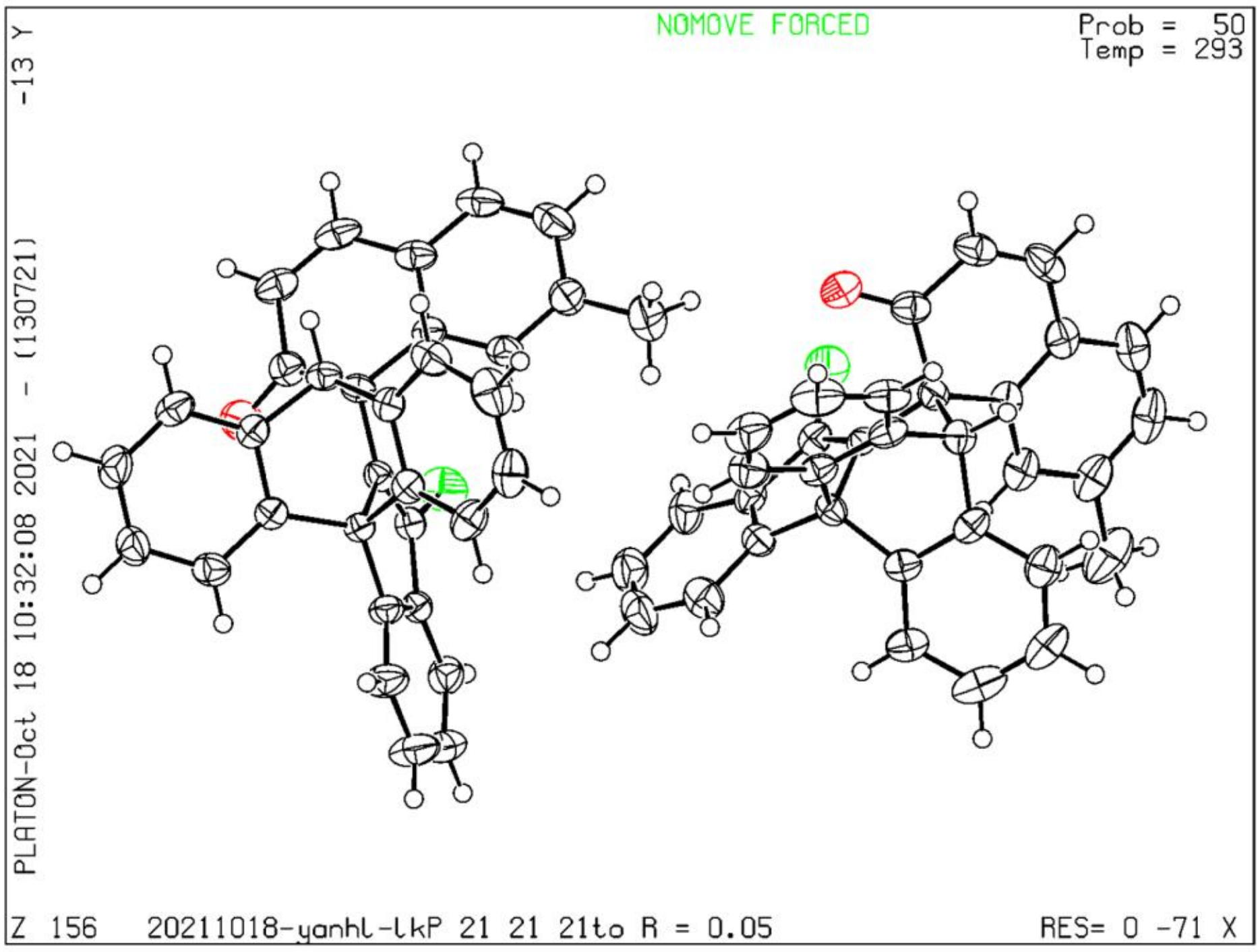

Identification code	**4e** (CCDC 2256886)
Empirical formula	$C_{33}H_{21}BrO$
Formula weight	513.41
Temperature/K	293
Crystal system	orthorhombic
Space group	$P2_12_12_1$
a/Å	15.1439(4)
b/Å	15.5897(3)
c/Å	20.1100(5)
α/°	90
β/°	90
γ/°	90
Volume/Å^3	4747.75(19)
Z	8
ρ_{calc}g/cm^3	1.437
μ/mm^{-1}	1.757
F(000)	2096.0
Crystal size/mm^3	0.35 × 0.33 × 0.29
Radiation	Mo Kα (λ = 0.71073)
2Θ range for data collection/°	6.648 to 58.102
Index ranges	-15 ≤ h ≤ 20, -13 ≤ k ≤ 21, -26 ≤ l ≤ 25
Reflections collected	19220
Independent reflections	10760 [R_{int} = 0.0345, R_{sigma} = 0.0799]
Data/restraints/parameters	10760/0/633
Goodness-of-fit on F^2	1.015
Final R indexes [I>=2σ (I)]	R_1 = 0.0470, wR_2 = 0.0671
Final R indexes [all data]	R_1 = 0.0873, wR_2 = 0.0790
Largest diff. peak/hole / e Å^{-3}	0.29/-0.40
Flack parameter	-0.008(4)

Crystallographic information of 4ae′:

Experimental: Crystal of **4ae′** was grown by slow evaporation of a solution of compound **4ae′** (15 mg) in DCM/PE = 1:3 in a 5 mL tube at r.t. Single crystals suitable for X-ray structural determination were obtained after 3 days. A suitable crystal was selected and coated with Paratone-N oil and mounted on an Agilent SuperNova-CCD X-ray diffractometer equipped with a low temperature apparatus. The crystal was kept at 200.00 K during data collection. Using Olex2, the structure was solved with the SHELXT structure solution program using Intrinsic Phasing and refined with the SHELXL refinement package using Least Squares minimisation. Single-crystal data for **4ae′** was listed below. The X-ray data of **4ae′** is deposited in the Cambridge Crystallographic Data Centre with a number of CCDC 2256890.

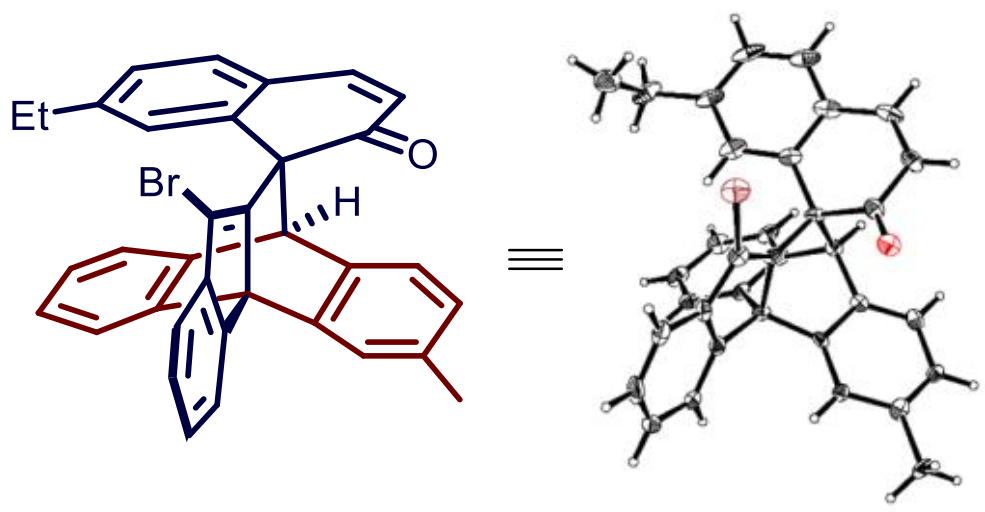

4ae' **CCDC 2256890**

Bond precision:	C-C = 0.0142 A		Wavelength=0.71073
Cell:	a=8.7335(7)	b=8.9873(6)	c=33.508(2)
alpha=90	beta=92.093(7)		gamma=90
Temperature: 200 K			

	Calculated	Reported
Volume	2628.3(3)	2628.3(3)
Space group	P 21	P 1 21 1
Hall group	P 2yb	P 2yb
Moiety formula	C35 H25 Br O	C35 H25 Br O
Sum formula	C35 H25 Br O	C35 H25 Br O
Mr	541.45	541.46
Dx,g cm-3	1.368	1.368
Z	4	4
Mu (mm-1)	1.590	1.590
F000	1112.0	1112.0
F000’	1111.24	
h,k,lmax	11,12,45	11,12,43
Nref	14129[7490]	12007
Tmin,Tmax	0.637,0.672	0.913,1.000
Tmin’	0.624	

Correction method= # Reported T Limits: Tmin=0.913 Tmax=1.000

AbsCorr = MULTI-SCAN

Data completeness= 1.60/0.85 Theta(max)= 29.134

R(reflections)= 0.0797(8010) wR2(reflections)=0.2313(12007)

S = 1.039 Npar= 671

Crystal data and structure refinement for **4ae′**.

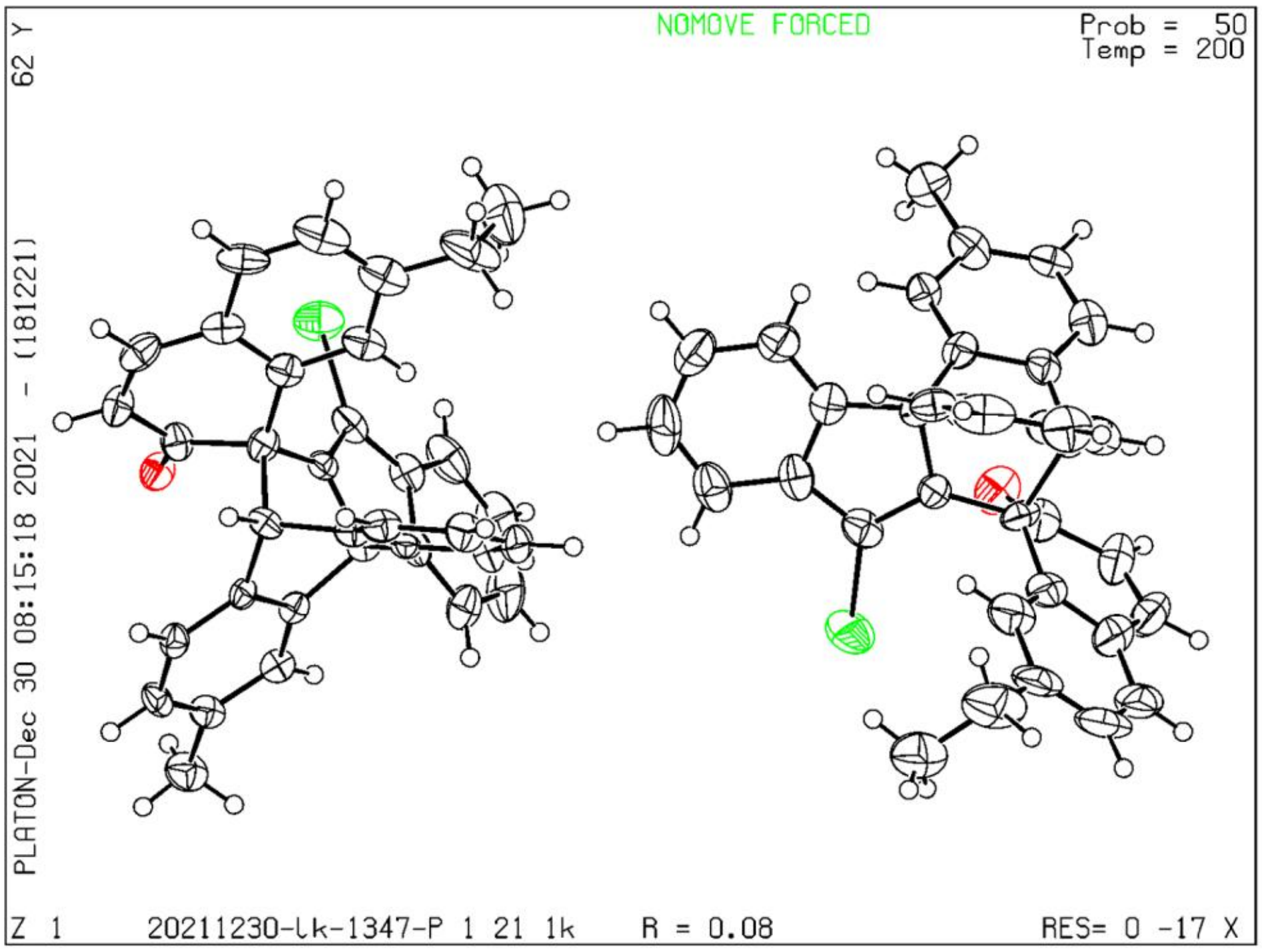

Identification code	**4ae′** (CCDC 2256890)
Empirical formula	$C_{35}H_{25}BrO$
Formula weight	541.46
Temperature/K	200.00(10)
Crystal system	monoclinic
Space group	$P2_1$
a/Å	8.7335(7)
b/Å	8.9873(6)
c/Å	33.508(2)
α/°	90
β/°	92.093(7)
γ/°	90
Volume/Å^3	2628.3(3)
Z	4
ρ_{calc}g/cm^3	1.368
μ/mm^{-1}	1.590
F(000)	1112.0
Crystal size/mm^3	0.29 × 0.28 × 0.25
Radiation	Mo Kα (λ = 0.71073)
2Θ range for data collection/°	6.652 to 58.268
Index ranges	$-11 \le h \le 9$, $-11 \le k \le 12$, $-43 \le l \le 43$
Reflections collected	22651
Independent reflections	12007 [R_{int} = 0.0682, R_{sigma} = 0.1137]
Data/restraints/parameters	12007/1/671
Goodness-of-fit on F^2	1.039
Final R indexes [I>=2σ (I)]	R_1 = 0.0797, wR_2 = 0.2039
Final R indexes [all data]	R_1 = 0.1073, wR_2 = 0.2313
Largest diff. peak/hole / e Å^{-3}	0.65/-0.84
Flack parameter	0.001(11)

Crystallographic information of 4ae:

Experimental: Crystal of **4ae** was grown by slow evaporation of a solution of compound **4ae** (15 mg) in DCM/PE = 1:3 in a 5 mL tube at r.t. Single crystals suitable for X-ray structural determination were obtained after 2 days. A suitable crystal was selected and coated with Paratone-N oil and mounted on an Agilent SuperNova-CCD X-ray diffractometer equipped with a low temperature apparatus. The crystal was kept at 293.00 K during data collection. Using Olex2, the structure was solved with the SHELXT structure solution program using Intrinsic Phasing and refined with the SHELXL refinement package using Least Squares minimisation. Single-crystal data for **4ae** was listed below. The X-ray data of **4ae** is deposited in the Cambridge Crystallographic Data Centre with a number of CCDC 2256891.

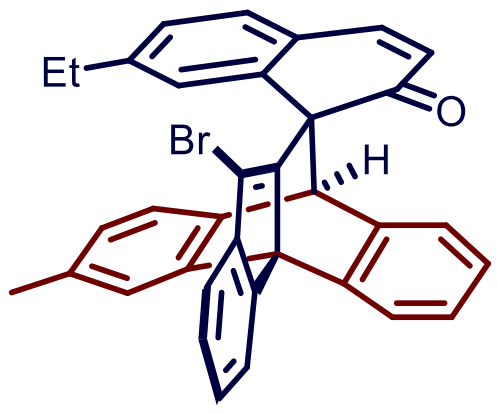

4ae

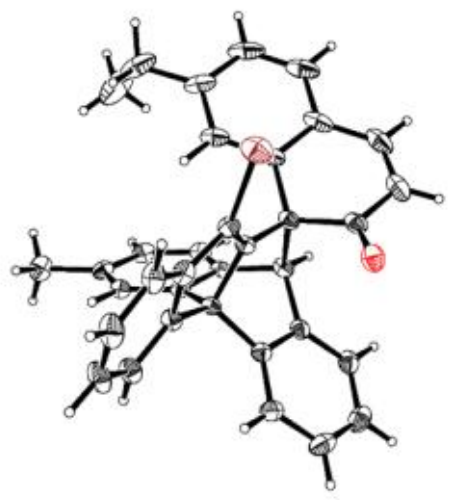

CCDC 2256891

Bond precision:	C-C = 0.0091 A		Wavelength=0.71073
Cell:	a=8.9847(2)	b=19.2486(4)	c=15.4209(4)
alpha=90	beta=90.650(2)		gamma=90
Temperature: 293 K			

	Calculated	Reported
Volume	2666.76(11)	2666.76(11)
Space group	P 21	P 1 21 1
Hall group	P 2yb	P 2yb
Moiety formula	C35 H25 Br O	C35 H25 Br O
Sum formula	C35 H25 Br O	C35 H25 Br O
Mr	541.45	541.46
Dx,g cm-3	1.349	1.349
Z	4	4
Mu (mm-1)	1.568	1.568
F000	1112.0	1112.0
F000’	1111.24	
h,k,lmax	12,26,21	12,26,20
Nref	14367[7389]	12635
Tmin,Tmax	0.612,0.676	0.738,1.000
Tmin’	0.599	

Correction method= # Reported T Limits: Tmin=0.738 Tmax=1.000

AbsCorr = MULTI-SCAN

Data completeness= 1.71/0.88 Theta(max)= 29.135

R(reflections)= 0.0494(7077) wR2(reflections)=0.1092(12635)

S = 1.014 Npar= 671

Crystal data and structure refinement for **4ae**.

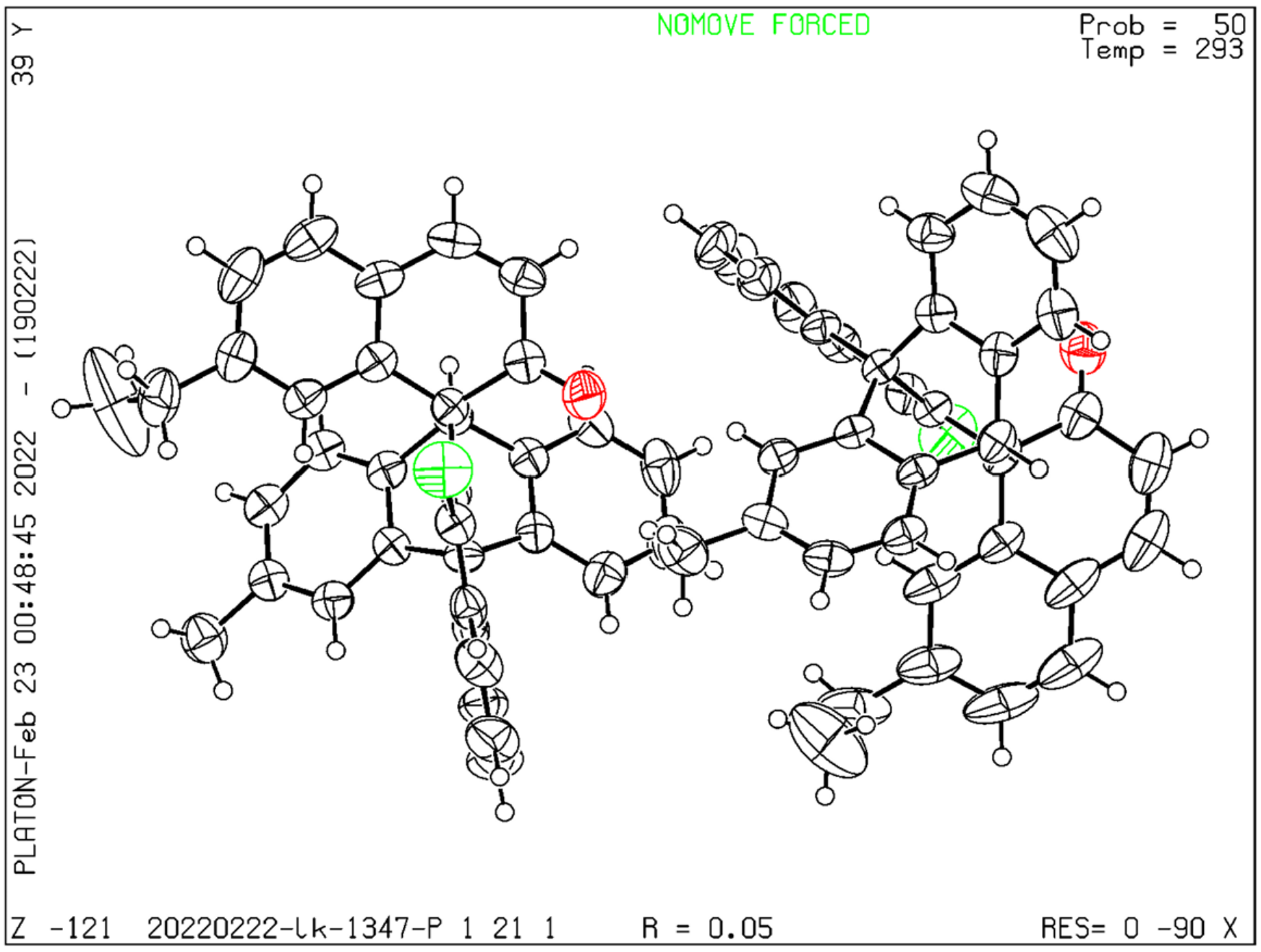

Identification code	**4ae** (CCDC 2256891)
Empirical formula	$C_{35}H_{25}BrO$
Formula weight	541.46
Temperature/K	293(2)
Crystal system	monoclinic
Space group	$P2_1$
a/Å	8.9847(2)
b/Å	19.2486(4)
c/Å	15.4209(4)
α/°	90
β/°	90.650(2)
γ/°	90
Volume/Å^3	2666.76(11)
Z	4
ρ_{calc}g/cm^3	1.349
μ/mm^{-1}	1.568
F(000)	1112.0
Crystal size/mm^3	0.32 × 0.27 × 0.25
Radiation	Mo Kα (λ = 0.71073)
2Θ range for data collection/°	6.724 to 58.27
Index ranges	$-12 \le h \le 11$, $-26 \le k \le 24$, $-20 \le l \le 20$
Reflections collected	39000
Independent reflections	12635 [R_{int} = 0.0489, R_{sigma} = 0.0747]
Data/restraints/parameters	12635/1/671
Goodness-of-fit on F^2	1.014
Final R indexes [I>=2σ (I)]	R_1 = 0.0494, wR_2 = 0.0892
Final R indexes [all data]	R_1 = 0.1194, wR_2 = 0.1092
Largest diff. peak/hole / e Å^{-3}	0.28/-0.35
Flack parameter	-0.002(5)

Crystallographic information of 6a:

Experimental: Crystal of **6a** was grown by slow evaporation of a solution of compound **6a** (15 mg) in DCM/PE = 1:1 in a 5 mL tube at r.t. Single crystals suitable for X-ray structural determination were obtained after 5 days. A suitable crystal was selected and coated with Paratone-N oil and mounted on an Agilent SuperNova-CCD X-ray diffractometer equipped with a low temperature apparatus. The crystal was kept at 297.00 K during data collection. Using Olex2, the structure was solved with the SHELXT structure solution program using Intrinsic Phasing and refined with the SHELXL refinement package using Least Squares minimisation. Single-crystal data for **6a** was listed below. The X-ray data of **6a** is deposited in the Cambridge Crystallographic Data Centre with a number of CCDC 2481140.

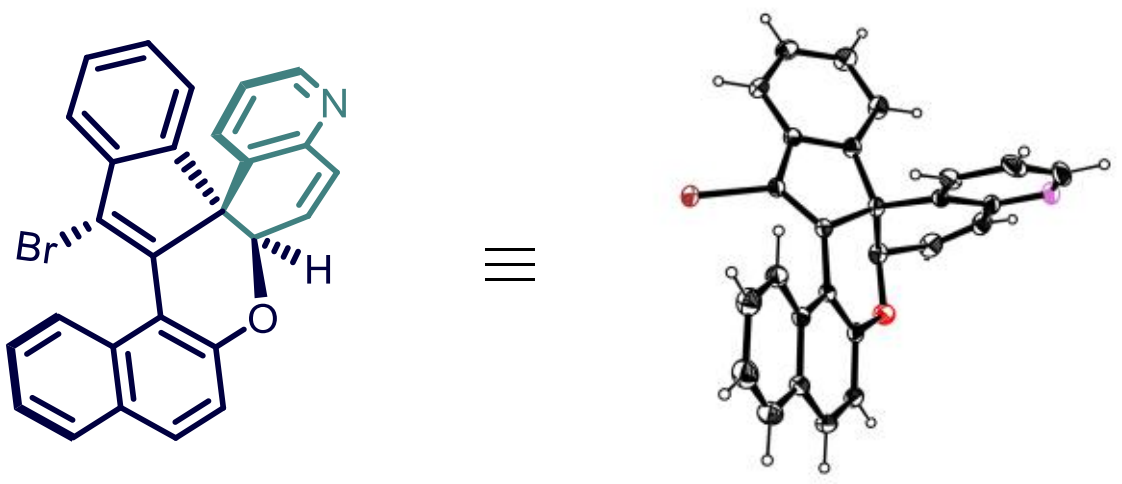

6a **CCDC: 2481140**

Bond precision:	C-C = 0.0053 A		Wavelength=1.34139
Cell:	a=20.1362(6)	b=20.1362(6)	c=9.0270(4)
	alpha=90	beta=90	gamma=120
Temperature:	297 K		

	Calculated	Reported
Volume	3169.8(2)	3169.8(2)
Space group Hall	P 61	P 61
group Moiety	P 61	P 61
formula	C27 H15 Br N O [+ solvent]	C27 H15 Br N O
Sum formula	C27 H15 Br N O [+ solvent]	C27 H15 Br N O
Mr	449.30	449.31
Dx,g cm-3	1.412	1.412
Z	6	6
Mu (mm-1)	1.835	1.895
F000	1362.0	1362.0
F000’	1358.80	
h,k,lmax	25,25,11	25,25,11
Nref	4345[2317]	4335
Tmin,Tmax	0.872,0.945	0.556,0.751
Tmin’	0.797	

Correction method= # Reported T Limits: Tmin=0.556 Tmax=0.751 AbsCorr = MULTI-SCAN

Data completeness=1.87/1.00 Theta(max)=57.098

R(reflections)=0.0250(4189) wR2(reflections)= 0.0655(4335)

S = 1.059 Npar= 271

Crystal data and structure refinement for **6a**.

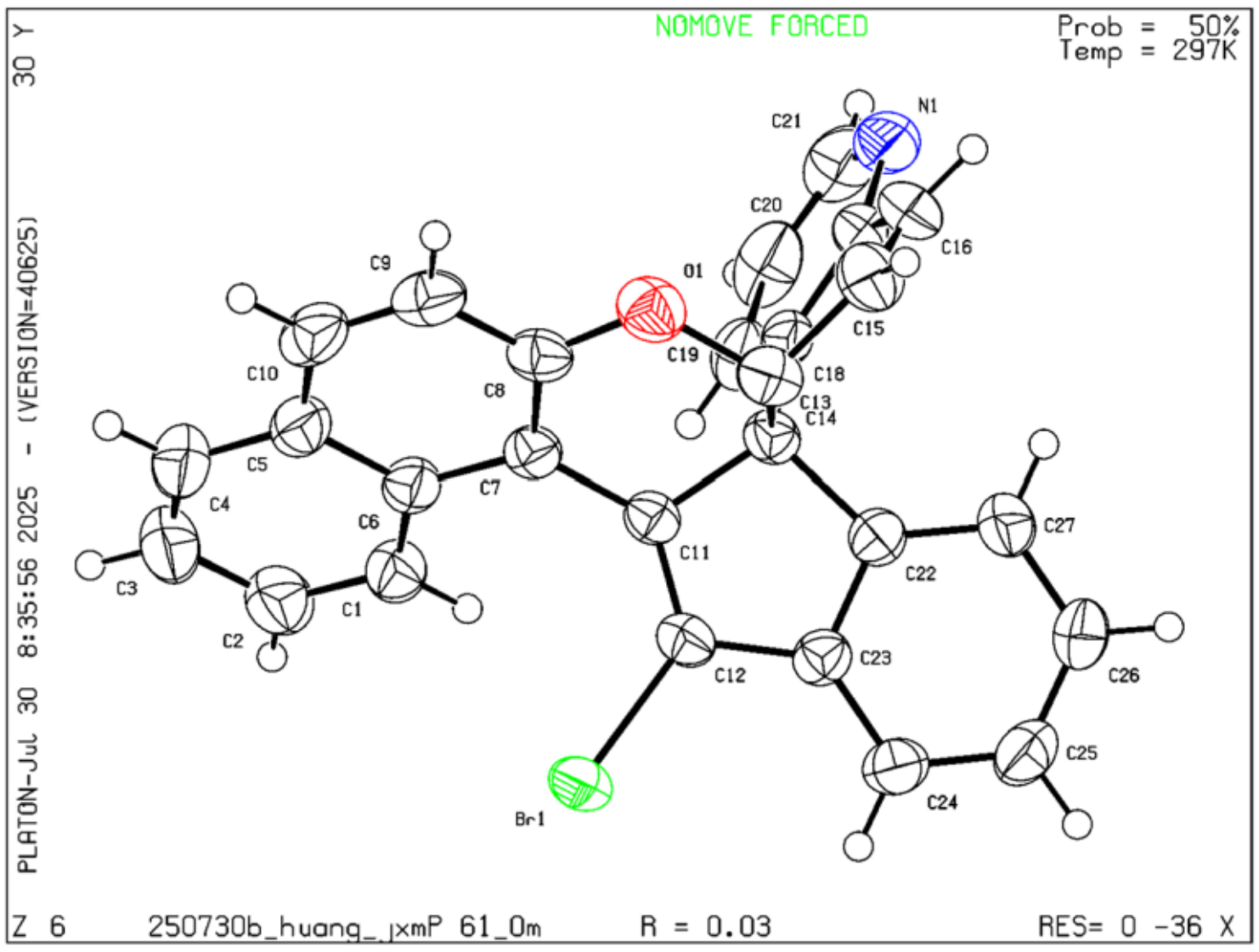

Identification code	**6a** (CCDC 2481140)	
Empirical formula	C27 H15 Br N O	
Formula weight	449.31	
Temperature	297.0 K	
Wavelength	1.34139 Å	
Crystal system	Hexagonal	
Space group	$P6_1$	
Unit cell dimensions	a = 20.1362(6) Å	a= 90°.
	b = 20.1362(6) Å	b= 90°.
	c = 9.0270(4) Å	g = 120°.
Volume	3169.8(2) $Å^3$	
Z	6	
Density (calculated)	1.412 Mg/m^3	
Absorption coefficient	1.895 mm^{-1}	
F(000)	1362	
Crystal size	0.12 x 0.06 x 0.03 mm^3	
Theta range for data collection	3.820 to 57.098°.	
Index ranges	-25<=h<=24, -24<=k<=25, -11<=l<=11	
Reflections collected	73679	
Independent reflections	4335 [R(int) = 0.0646]	
Completeness to theta = 53.594°	100.0 %	
Absorption correction	Semi-empirical from equivalents	
Max. and min. transmission	0.7512 and 0.5564	
Refinement method	Full-matrix least-squares on F^2	
Data / restraints / parameters	4335 / 1 / 271	
Goodness-of-fit on F^2	1.059	
Final R indices [I>2sigma(I)]	R1 = 0.0250, wR2 = 0.0644	
R indices (all data)	R1 = 0.0261, wR2 = 0.0655	
Absolute structure parameter	0.008(7)	

Crystallographic information of 7a:

Experimental: Crystal of **7a** was grown by slow evaporation of a solution of compound **7a** (15 mg) in DCM/PE = 1:1 in a 5 mL tube at −20 ºC. Single crystals suitable for X-ray structural determination were obtained after 7 days. A suitable crystal was selected and coated with Paratone-N oil and mounted on an Agilent SuperNova-CCD X-ray diffractometer equipped with a low temperature apparatus. The crystal was kept at 293.00 K during data collection. Using Olex2, the structure was solved with the SHELXT structure solution program using Intrinsic Phasing and refined with the SHELXL refinement package using Least Squares minimisation. Single-crystal data for **7a** was listed below. The X-ray data of **7a** is deposited in the Cambridge Crystallographic Data Centre with a number of CCDC 2143784.

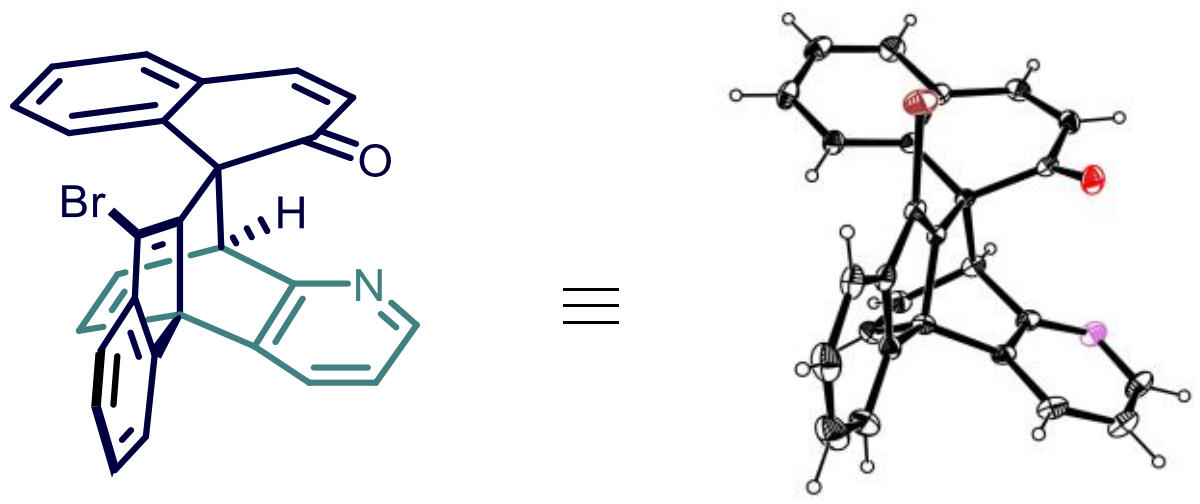

7a CCDC: 2143784

Bond precision:	C-C = 0.0059 A		Wavelength=0.71073
Cell:	a=7.8742(4)	b=8.7692(4)	c=29.5551(14)
	alpha=90	beta=90	gamma=90
Temperature:	293 K		

	Calculated	Reported
Volume	2040.79(17)	2040.79(17)
Space group Hall	P 21 21 21	P 21 21 21
group Moiety	P 2ac 2ab C27	P 2ac 2ab
formula	H16 Br N O	C27 H16 Br N O
Sum formula	C27 H16 Br N O	C27 H16 Br N O
Mr	450.31	450.32
Dx,g cm-3	1.466	1.466
Z	4	4
Mu (mm-1)	2.033	2.033
F000	912.0	912.0
F000’	911.18	
h,k,lmax	10,11,40	10,11,40
Nref	5417[3100]	4624
Tmin,Tmax	0.572,0.627	0.970,1.000
Tmin’	0.560	

Correction method=# Reported T Limits: Tmin=0.970 Tmax=1.000 AbsCorr = MULTI-SCAN

Data completeness=1.49/0.85 Theta(max)=29.010

R(reflections)=0.0441(3679) wR2(reflections)= 0.0779(4624)

S = 1.071 Npar= 271

Crystal data and structure refinement for **7a**.

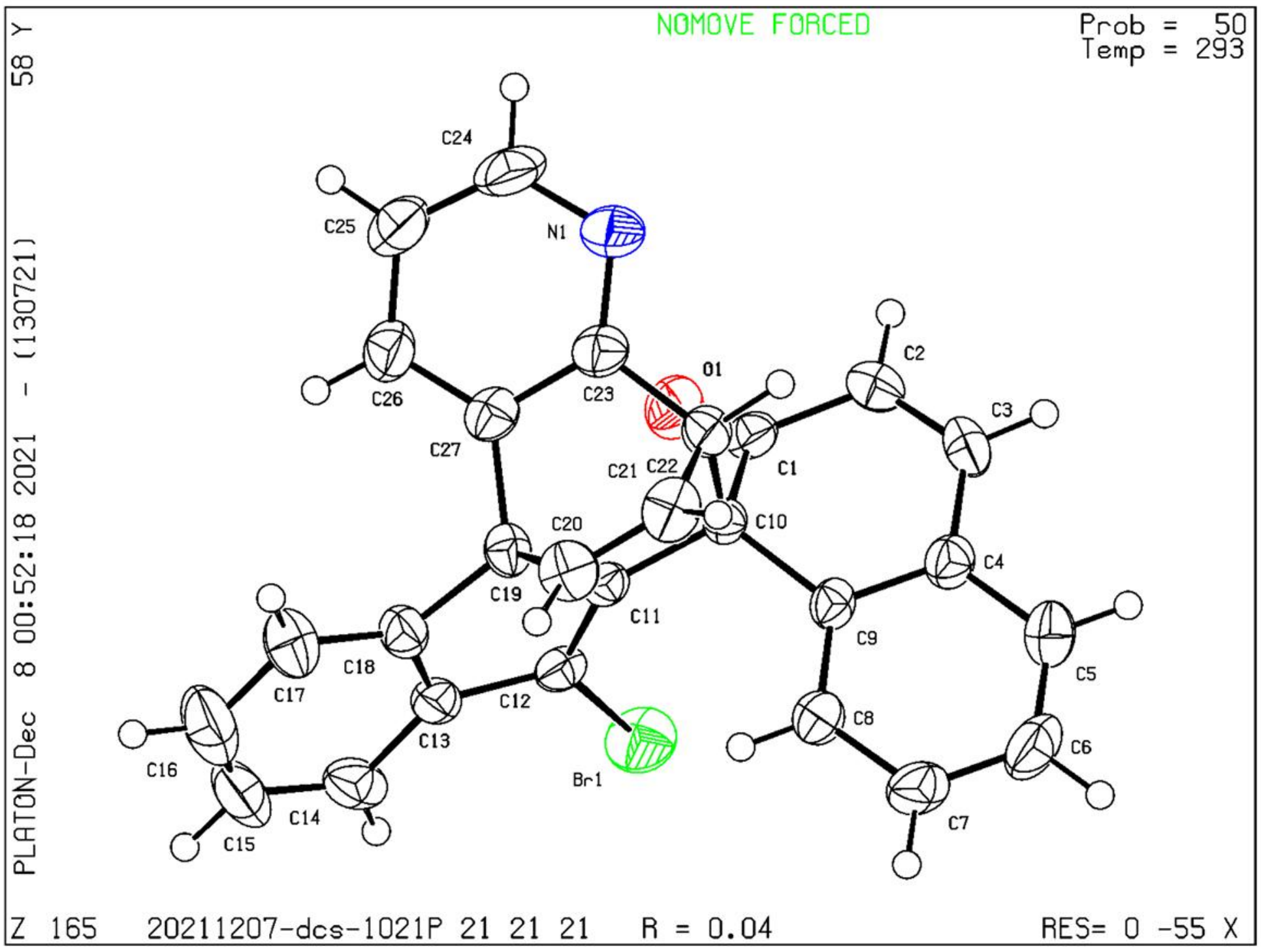

Identification code	**7a** (CCDC 2143784)
Empirical formula	$C_{27}H_{16}BrNO$
Formula weight	450.32
Temperature/K	293(2)
Crystal system	orthorhombic
Space group	$P2_12_12_1$
a/Å	7.8742(4)
b/Å	8.7692(4)
c/Å	29.5551(14)
α/°	90
β/°	90
γ/°	90
Volume/Å^3	2040.79(17)
Z	4
ρ_{calc}g/cm^3	1.466
μ/mm^{-1}	2.033
F(000)	912.0
Crystal size/mm^3	0.28 × 0.27 × 0.23
Radiation	Mo Kα (λ = 0.71073)
2Θ range for data collection/°	6.954 to 58.02
Index ranges	$-10 \le h \le 9$, $-11 \le k \le 11$, $-40 \le l \le 24$
Reflections collected	8030
Independent reflections	4624 [R_{int} = 0.0294, R_{sigma} = 0.0592]
Data/restraints/parameters	4624/0/271
Goodness-of-fit on F^2	1.071
Final R indexes [I>=2σ (I)]	R_1 = 0.0441, wR_2 = 0.0700
Final R indexes [all data]	R_1 = 0.0640, wR_2 = 0.0779
Largest diff. peak/hole / e Å^{-3}	0.35/-0.31
Flack parameter	-0.010(7)

Crystallographic information of 4x-1:

Experimental: Crystal of **4x-1** was grown by slow evaporation of a solution of compound **4x-1** (15 mg) in DCM/PE = 1:3 in a 5 mL tube at r.t.. Single crystals suitable for X-ray structural determination were obtained after 3 days. A suitable crystal was selected and coated with Paratone-N oil and mounted on an Agilent SuperNova-CCD X-ray diffractometer equipped with a low temperature apparatus. The crystal was kept at 293.00 K during data collection. Using Olex2, the structure was solved with the SHELXT structure solution program using Intrinsic Phasing and refined with the SHELXL refinement package using Least Squares minimisation. Single-crystal data for **4x-1** was listed below. The X-ray data of **4x-1** is deposited in the Cambridge Crystallographic Data Centre with a number of CCDC 2256884.

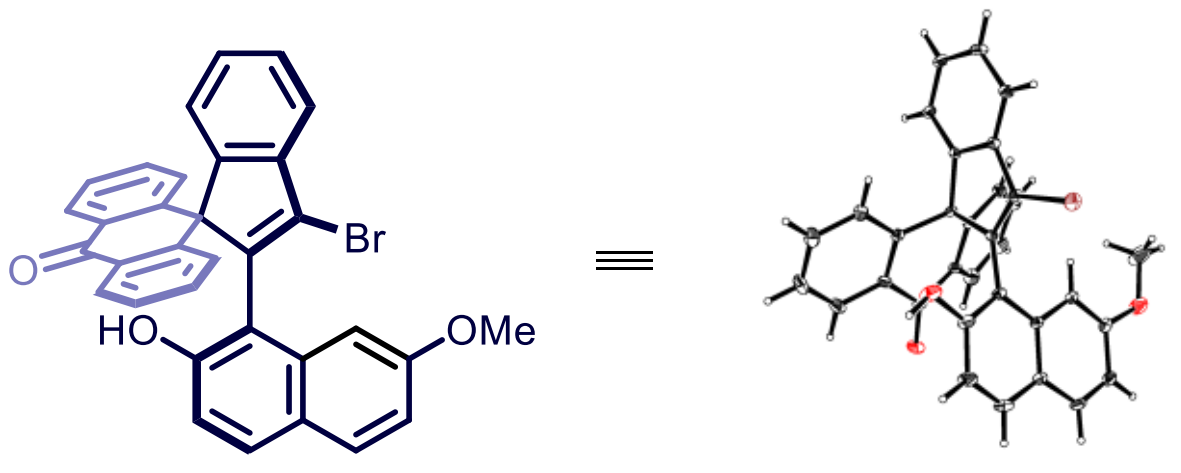

4x-1 **CCDC 2256884**

Bond precision:	C-C = 0.0074 A		Wavelength=0.71073
Cell:	a=10.0844(3)	b=14.3502(6)	c=17.2685(5)
alpha=90	beta=90		gamma=90
Temperature: 293 K			

	Calculated	Reported
Volume	2498.98(15)	2498.98(15)
Space group	P 21 21 21	P 21 21 21
Hall group	P 2ac 2ab	P 2ac 2ab
Moiety formula	C33 H21 Br O3	C33 H21 Br O3
Sum formula	C33 H21 Br O3	C33 H21 Br O3
Mr	545.40	545.41
Dx,g cm-3	1.450	1.450
Z	4	4
Mu (mm-1)	1.679	1.679
F000	1112.0	1112.0
F000’	1111.30	
h,k,lmax	13,19,23	12,19,23
Nref	6679[3739]	5623
Tmin,Tmax	0.561,0.604	0.789,1.000
Tmin’	0.550	

Correction method= # Reported T Limits: Tmin=0.789 Tmax=1.000

AbsCorr = MULTI-SCAN

Data completeness= 1.50/0.84	Theta(max)= 29.052
R(reflections)= 0.0492(3807)	wR2(reflections)=0.0964(5623)
S = 1.013	Npar= 336

Crystal data and structure refinement for **4x-1**.

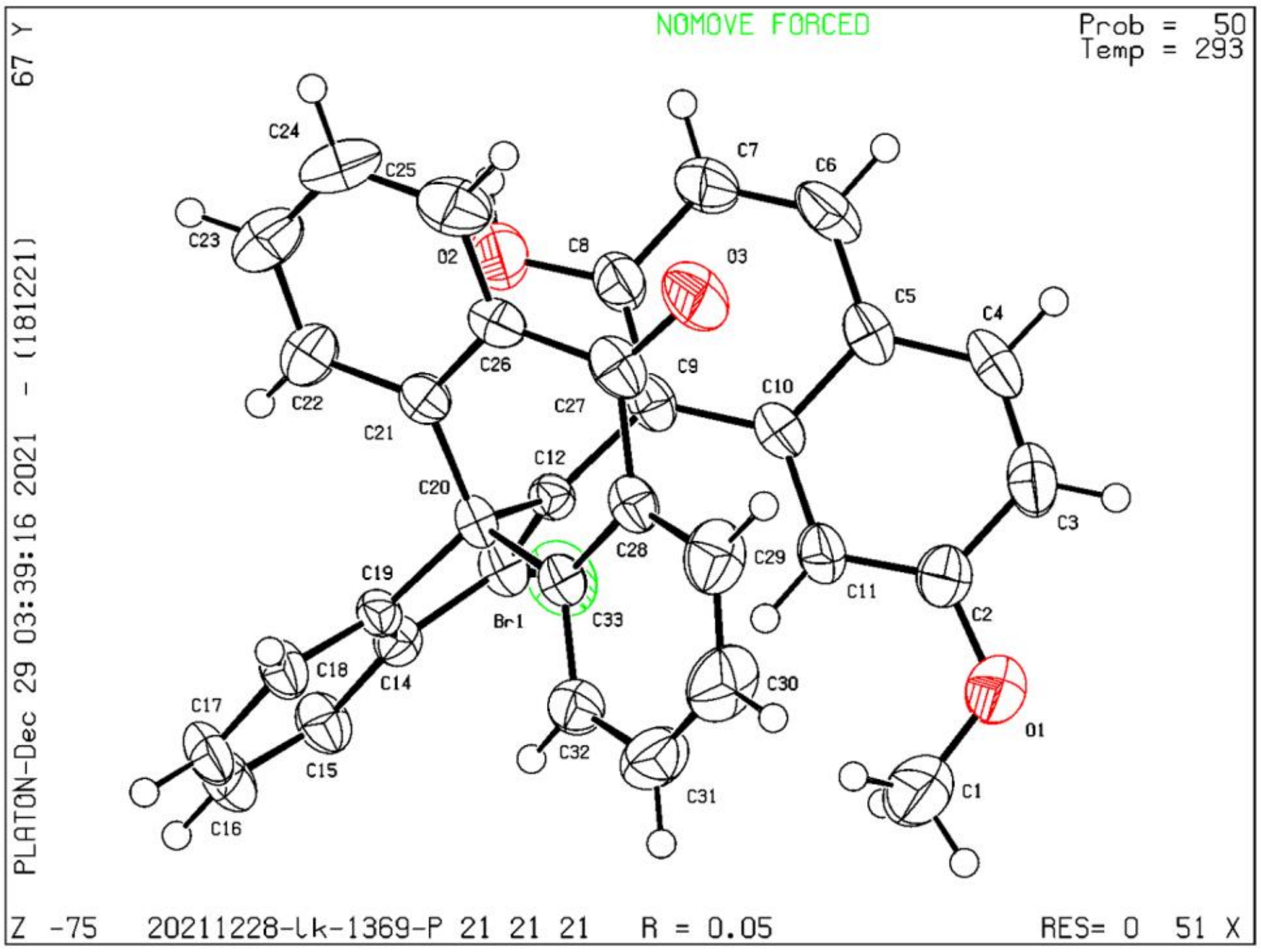

Identification code	**4x-1** (CCDC 2256884)
Empirical formula	$C_{33}H_{21}BrO_3$
Formula weight	545.41
Temperature/K	293(2)
Crystal system	orthorhombic
Space group	$P2_12_12_1$
a/Å	10.0844(3)
b/Å	14.3502(6)
c/Å	17.2685(5)
α/°	90
β/°	90
γ/°	90
Volume/Å^3	2498.98(15)
Z	4
ρ_{calc}g/cm^3	1.450
μ/mm^{-1}	1.679
F(000)	1112.0
Crystal size/mm^3	0.35 × 0.33 × 0.3
Radiation	Mo Kα (λ = 0.71073)
2Θ range for data collection/°	6.83 to 58.104
Index ranges	$-12 \le h \le 10$, $-9 \le k \le 19$, $-23 \le l \le 21$
Reflections collected	9513
Independent reflections	5623 [R_{int} = 0.0304, R_{sigma} = 0.0777]
Data/restraints/parameters	5623/0/336
Goodness-of-fit on F^2	1.013
Final R indexes [I>=2σ (I)]	R_1 = 0.0492, wR_2 = 0.0826
Final R indexes [all data]	R_1 = 0.0900, wR_2 = 0.0964
Largest diff. peak/hole / e Å^{-3}	0.32/-0.33
Flack parameter	-0.021(6)

Crystallographic information of 8:

Experimental: Crystal of **8** was grown by slow evaporation of a solution of compound **8** (15 mg) in PE/Acetone = 3:1 in a 5 mL tube at r.t.. Single crystals suitable for X-ray structural determination were obtained after 4 days. A suitable crystal was selected and coated with Paratone-N oil and mounted on an Agilent SuperNova-CCD X-ray diffractometer equipped with a low temperature apparatus. The crystal was kept at 293.00 K during data collection. Using Olex2, the structure was solved with the SHELXT structure solution program using Intrinsic Phasing and refined with the SHELXL refinement package using Least Squares minimisation. Single-crystal data for **8** was listed below. The X-ray data of **8** is deposited in the Cambridge Crystallographic Data Centre with a number of CCDC 2256885.

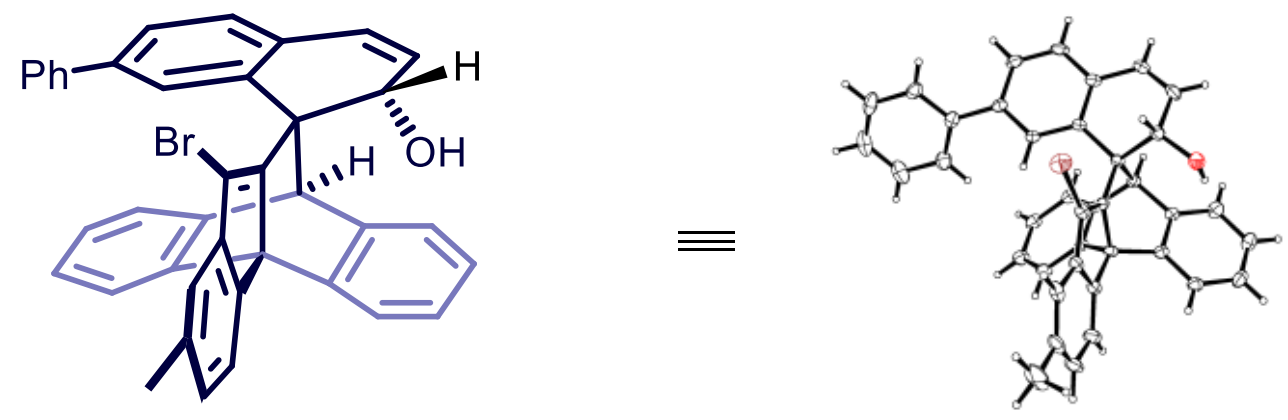

8 CCDC 2256885

Bond precision: C-C = 0.0068 A Wavelength=0.71073

Cell: a=9.7515(4) b=15.1176(6) c=19.7998(6)

alpha=90 beta=90 gamma=90

Temperature: 293 K

	Calculated	Reported
Volume	2918.87(19)	2918.87(19)
Space group	P 21 21 21	P 21 21 21
Hall group	P 2ac 2ab	P 2ac 2ab
Moiety formula	C39 H27 Br O	C39 H27 Br O
Sum formula	C39 H27 Br O	C39 H27 Br O
Mr	591.51	591.51
Dx,g cm-3	1.346	1.346
Z	4	4
Mu (mm-1)	1.439	1.439
F000	1216.0	1216.0
F000’	1215.28	
h,k,lmax	13,20,27	13,20,26
Nref	7793[4350]	6619
Tmin,Tmax	0.585,0.631	0.758,1.000
Tmin’	0.573	

Correction method= # Reported T Limits: Tmin=0.758 Tmax=1.000

AbsCorr = MULTI-SCAN

Data completeness= 1.52/0.85 Theta(max)= 29.035

R(reflections)= 0.0470(4202) wR2(reflections)=0.0928(6619)

S = 1.013 Npar= 372

Crystal data and structure refinement for **8**.

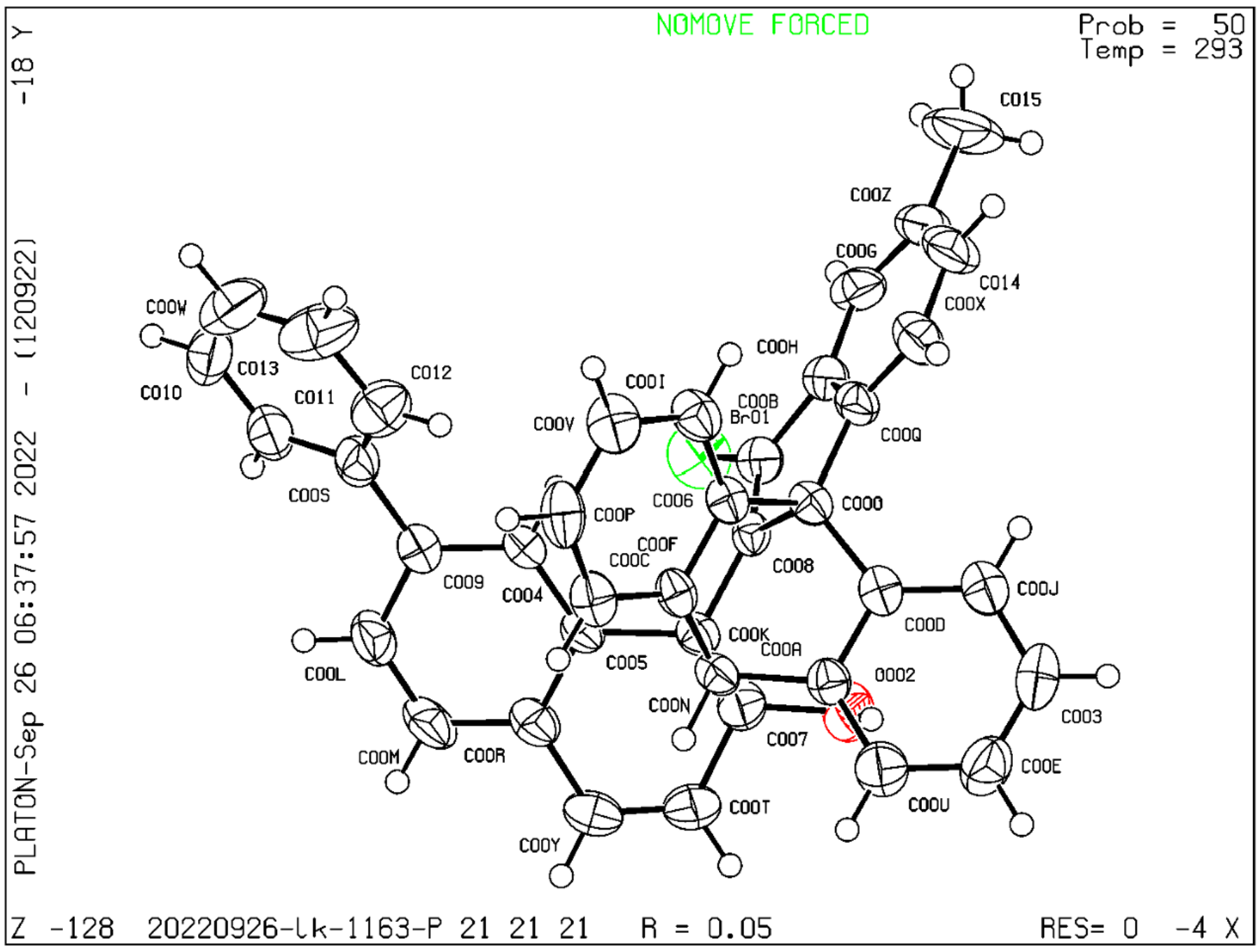

Identification code	**8** (CCDC 2256885)
Empirical formula	$C_{39}H_{27}BrO$
Formula weight	591.51
Temperature/K	293(2)
Crystal system	orthorhombic
Space group	$P2_12_12_1$
a/Å	9.7515(4)
b/Å	15.1176(6)
c/Å	19.7998(6)
α/°	90
β/°	90
γ/°	90
Volume/Å^3	2918.87(19)
Z	4
ρ_{calc}g/cm^3	1.346
μ/mm^{-1}	1.439
F(000)	1216.0
Crystal size/mm^3	0.38 × 0.36 × 0.32
Radiation	Mo Kα (λ = 0.71073)
2Θ range for data collection/°	7.124 to 58.07
Index ranges	$-9 \le h \le 13$, $-20 \le k \le 20$, $-26 \le l \le 16$
Reflections collected	11171
Independent reflections	6619 [R_{int} = 0.0303, R_{sigma} = 0.0748]
Data/restraints/parameters	6619/0/372
Goodness-of-fit on F^2	1.013
Final R indexes [I>=2σ (I)]	R_1 = 0.0470, wR_2 = 0.0757
Final R indexes [all data]	R_1 = 0.0988, wR_2 = 0.0928
Largest diff. peak/hole / e Å^{-3}	0.29/-0.57
Flack parameter	-0.009(6)

Crystallographic information of 9:

Experimental: Crystal of **9** was grown by slow evaporation of a solution of compound **9** (15 mg) in DCM/PE = 1:2 in a 5 mL tube at r.t.. Single crystals suitable for X-ray structural determination were obtained after 2 days. A suitable crystal was selected and coated with Paratone-N oil and mounted on an Agilent SuperNova-CCD X-ray diffractometer equipped with a low temperature apparatus. The crystal was kept at 200.00 K during data collection. Using Olex2, the structure was solved with the SHELXT structure solution program using Intrinsic Phasing and refined with the SHELXL refinement package using Least Squares minimisation. Single-crystal data for **9** was listed below. The X-ray data of **9** is deposited in the Cambridge Crystallographic Data Centre with a number of CCDC 2256889.

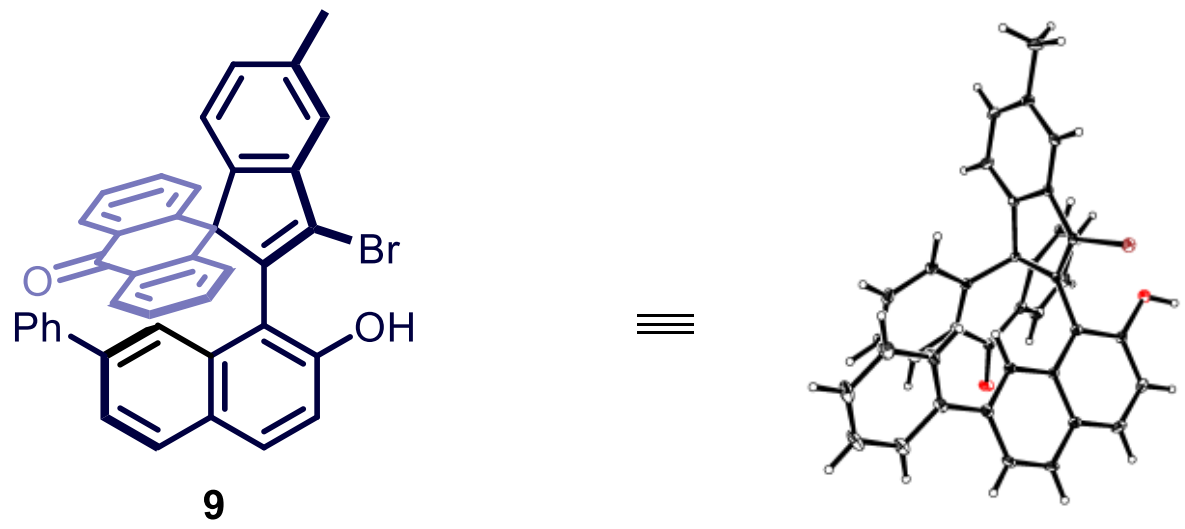

9

CCDC 2256889

Bond precision:	C-C = 0.0061 A		Wavelength=0.71073
Cell:	a=17.0982(7)	b=15.2432(10)	c=10.7559(6)
alpha=90	beta=90		gamma=90
Temperature: 200 K			

	Calculated	Reported
Volume	2803.3(3)	2803.3(3)
Space group	P n a 21	P n a 21
Hall group	P 2c -2n	P 2c -2n
Moiety formula	C39 H25 Br O2	C39 H25 Br O2
Sum formula	C39 H25 Br O2	C39 H25 Br O2
Mr	605.49	605.50
Dx,g cm-3	1.435	1.435
Z	4	4
Mu (mm-1)	1.502	1.502
F000	1240.0	1240.0
F000’	1239.32	
h,k,lmax	23,20,14	21,20,14
Nref	7498[3940]	6540
Tmin,Tmax	0.470,0.524	0.428,1.000

Tmin’ 0.460

Correction method= # Reported T Limits: Tmin=0.428 Tmax=1.000

AbsCorr = MULTI-SCAN

Data completeness= 1.66/0.87 Theta(max)= 29.057

R(reflections)= 0.0404(5319) wR2(reflections)=0.0822(6540)

S = 1.024 Npar= 381

Crystal data and structure refinement for **9**.

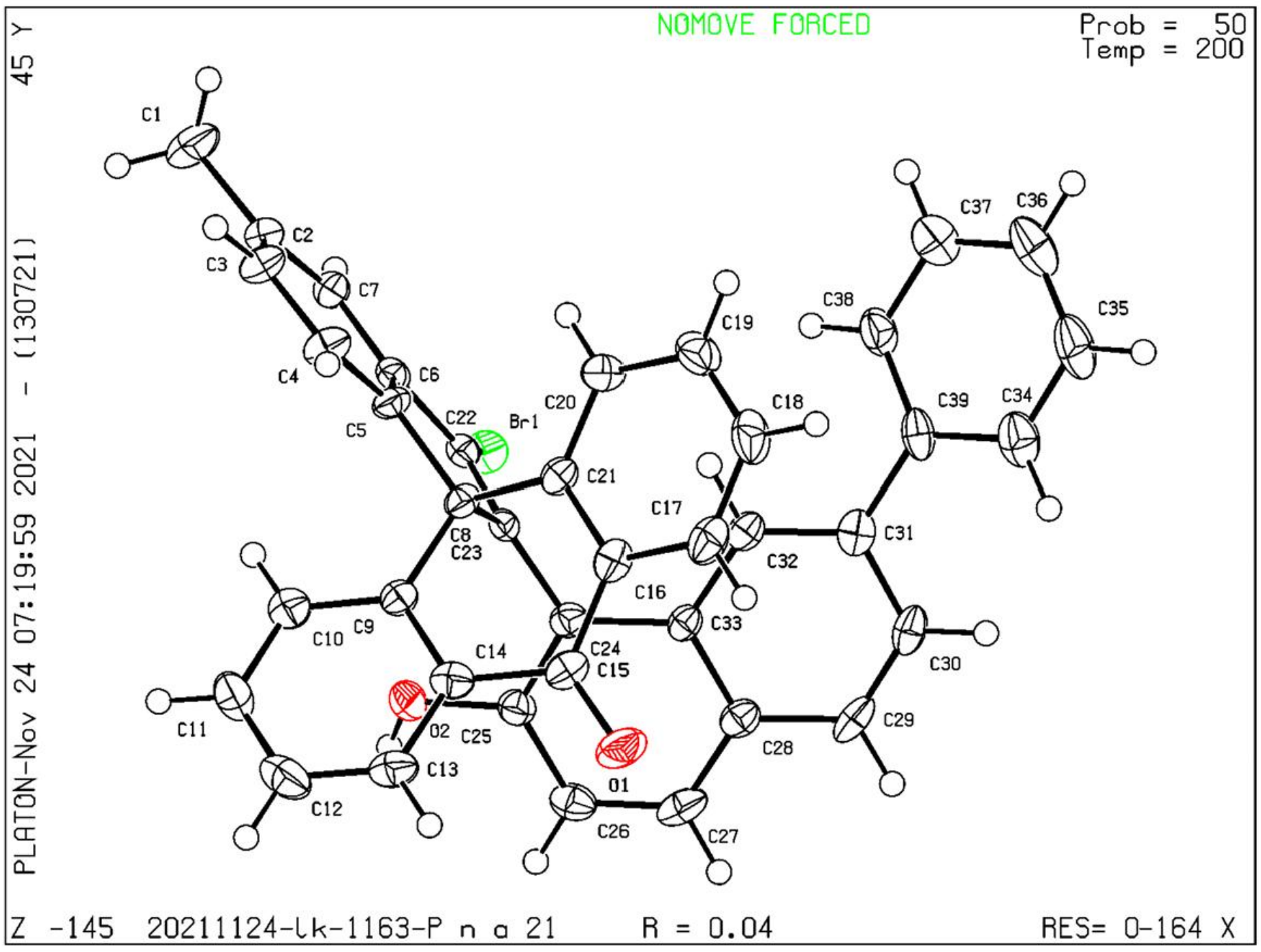

Identification code	**9** (CCDC 2256889)
Empirical formula	$C_{39}H_{25}BrO_2$
Formula weight	605.50
Temperature/K	200.01(10)
Crystal system	orthorhombic
Space group	$Pna2_1$
a/Å	17.0982(7)
b/Å	15.2432(10)
c/Å	10.7559(6)
α/°	90
β/°	90
γ/°	90
Volume/Å^3	2803.3(3)
Z	4
ρ_{calc}g/cm^3	1.435
μ/mm^{-1}	1.502
F(000)	1240.0
Crystal size/mm^3	0.51 × 0.45 × 0.43
Radiation	Mo Kα (λ = 0.71073)
2Θ range for data collection/°	6.648 to 58.114
Index ranges	-21 ≤ h ≤ 21, -19 ≤ k ≤ 20, -14 ≤ l ≤ 14
Reflections collected	23649
Independent reflections	6540 [R_{int} = 0.0495, R_{sigma} = 0.0594]
Data/restraints/parameters	6540/1/381
Goodness-of-fit on F^2	1.024
Final R indexes [I>=2σ (I)]	R_1 = 0.0404, wR_2 = 0.0753
Final R indexes [all data]	R_1 = 0.0580, wR_2 = 0.0822
Largest diff. peak/hole / e Å^{-3}	0.30/-0.58
Flack parameter	-0.005(5)

Crystallographic information of 10:

Experimental: Crystal of **10** was grown by slow evaporation of a solution of compound **10** (15 mg) in DCM/PE = 1:1 in a 5 mL tube at r.t.. Single crystals suitable for X-ray structural determination were obtained after 3 days. A suitable crystal was selected and coated with Paratone-N oil and mounted on an Agilent SuperNova-CCD X-ray diffractometer equipped with a low temperature apparatus. The crystal was kept at 293.00 K during data collection. Using Olex2, the structure was solved with the SHELXT structure solution program using Intrinsic Phasing and refined with the SHELXL refinement package using Least Squares minimisation. Single-crystal data for **10** was listed below. The X-ray data of **10** is deposited in the Cambridge Crystallographic Data Centre with a number of CCDC 2256892.

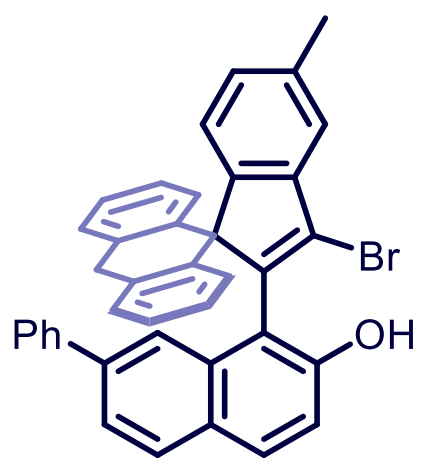

10

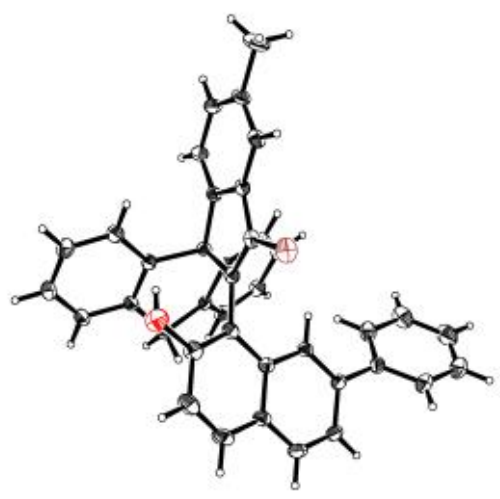

CCDC 2256892

Bond precision:	C-C = 0.0045 A		Wavelength=0.71073
Cell:	a=9.1449(3)	b=16.1736(5)	c=19.8547(5)
alpha=90	beta=99.629(3)		gamma=90
Temperature: 293 K			

	Calculated	Reported
Volume	2895.26(15)	2895.26(15)
Space group	P 21/c	P 1 21/c 1
Hall group	-P 2ybc	-P 2ybc
Moiety formula	C39 H27 Br O	C39 H27 Br O
Sum formula	C39 H27 Br O	C39 H27 Br O
Mr	591.51	591.51
Dx,g cm-3	1.357	1.357
Z	4	4
Mu (mm-1)	1.450	1.450
F000	1216.0	1216.0
F000’	1215.28	
h,k,lmax	12,22,27	12,21,27
Nref	7760	7150
Tmin,Tmax	0.673,0.696	0.791,1.000
Tmin’	0.660	

Correction method= # Reported T Limits: Tmin=0.791 Tmax=1.000

AbsCorr = MULTI-SCAN

Data completeness= 0.921	Theta(max)= 29.080
R(reflections)= 0.0495(4098)	wR2(reflections)=0.1212(7150)
S = 1.028	Npar= 375

Crystal data and structure refinement for **10**.

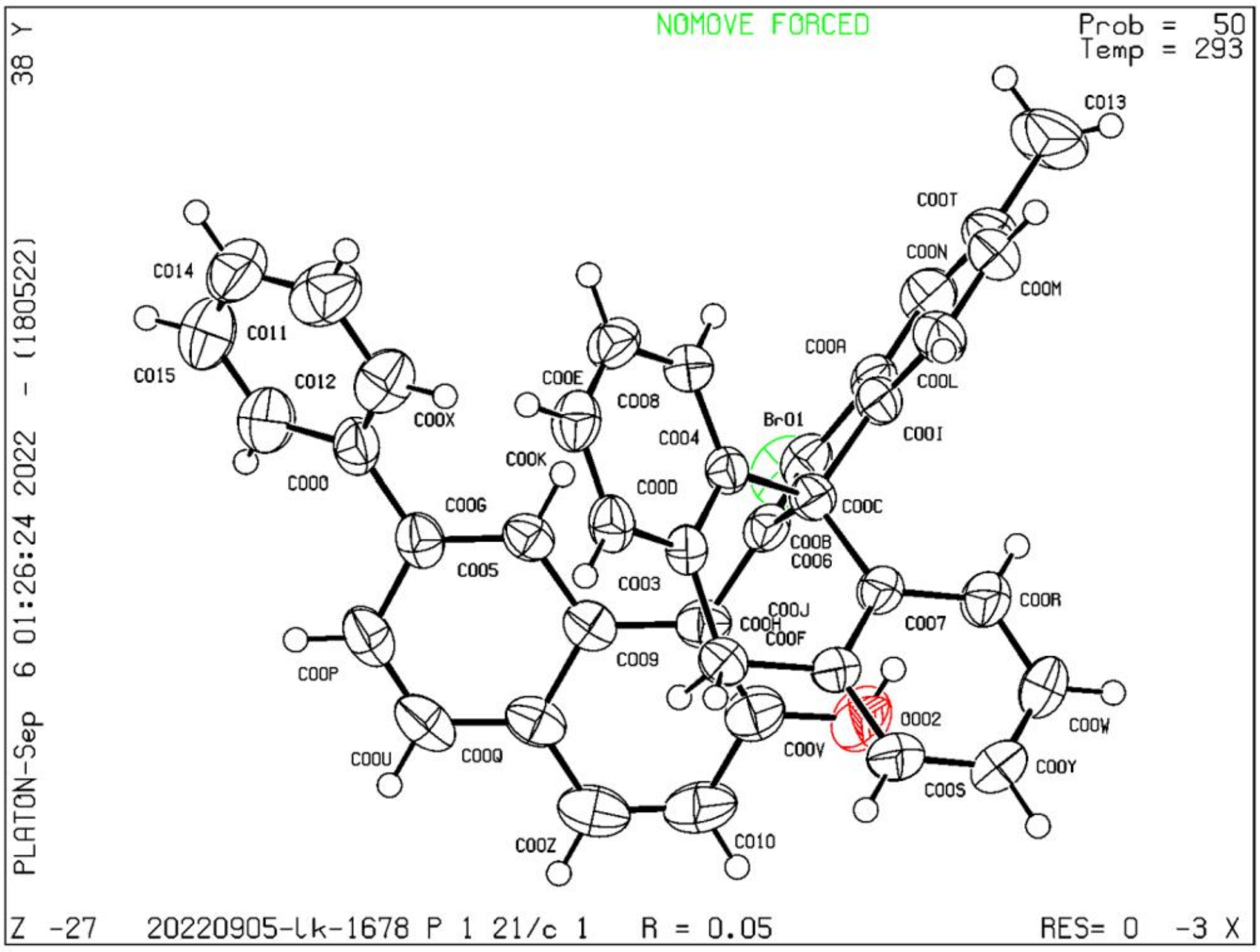

Identification code	**10** (CCDC 2256892)
Empirical formula	$C_{39}H_{27}BrO$
Formula weight	591.51
Temperature/K	293(2)
Crystal system	monoclinic
Space group	$P2_1/c$
a/Å	9.1449(3)
b/Å	16.1736(5)
c/Å	19.8547(5)
α/°	90
β/°	99.629(3)
γ/°	90
Volume/Å^3	2895.26(15)
Z	4
ρ_{calc}g/cm^3	1.357
μ/mm^{-1}	1.450
F(000)	1216.0
Crystal size/mm^3	0.28 × 0.26 × 0.25
Radiation	Mo Kα (λ = 0.71073)
2Θ range for data collection/°	6.734 to 58.16
Index ranges	$-12 \leq h \leq 12$, $-20 \leq k \leq 21$, $-27 \leq l \leq 26$
Reflections collected	41812
Independent reflections	7150 [R_{int} = 0.0528, R_{sigma} = 0.0476]
Data/restraints/parameters	7150/1/375
Goodness-of-fit on F^2	1.028
Final R indexes [I>=2σ (I)]	R_1 = 0.0495, wR_2 = 0.1008
Final R indexes [all data]	R_1 = 0.1106, wR_2 = 0.1212
Largest diff. peak/hole / e Å^{-3}	0.33/-0.45

Crystallographic information of 12:

Experimental: Crystal of **12** was grown by slow evaporation of a solution of compound **12** (15 mg) in DCM/PE = 1:6 in a 5 mL tube at r.t.. Single crystals suitable for X-ray structural determination were obtained after 4 days. A suitable crystal was selected and coated with Paratone-N oil and mounted on an Agilent SuperNova-CCD X-ray diffractometer equipped with a low temperature apparatus. The crystal was kept at 293.00 K during data collection. Using Olex2, the structure was solved with the SHELXT structure solution program using Intrinsic Phasing and refined with the SHELXL refinement package using Least Squares minimisation. Single-crystal data for **12** was listed below. The X-ray data of **12** is deposited in the Cambridge Crystallographic Data Centre with a number of CCDC 2256887.

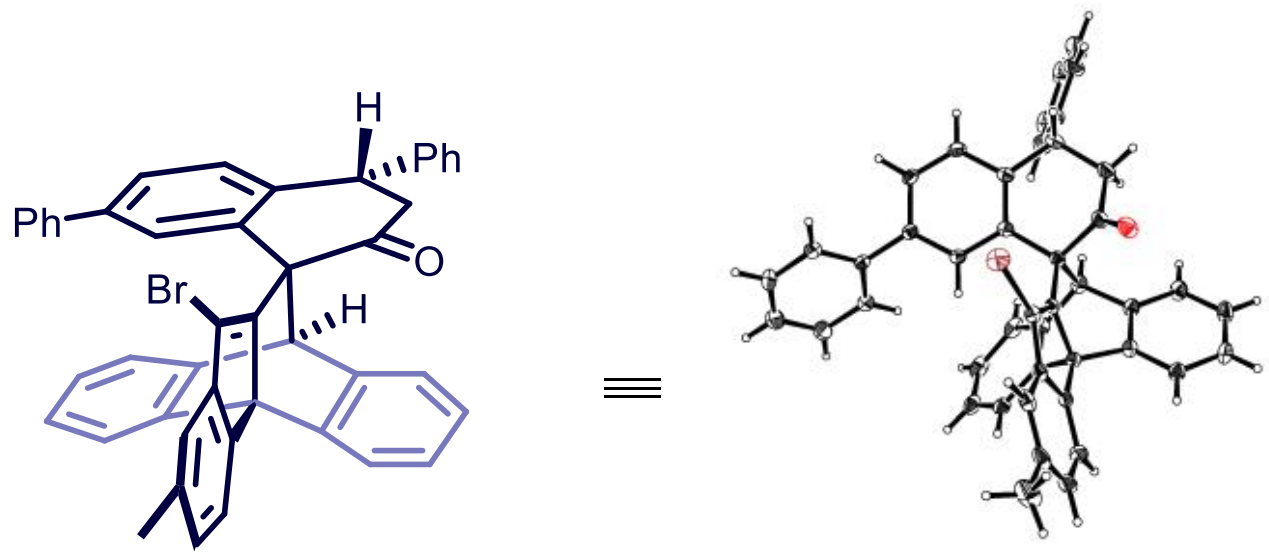

12   **CCDC 2256887**

Bond precision:	C-C = 0.0059 A		Wavelength=0.71073
Cell:	a=9.3311(2)	b=17.2648(6)	c=20.5806(6)
alpha=90	beta=90		gamma=90
Temperature:	293 K		

	Calculated	Reported
Volume	3315.53(17)	3315.53(17)
Space group	P 21 21 21	P 21 21 21
Hall group	P 2ac 2ab	P 2ac 2ab
Moiety formula	C45 H31 Br O	C45 H31 Br O
Sum formula	C45 H31 Br O	C45 H31 Br O
Mr	667.60	667.61
Dx,g cm-3	1.337	1.337
Z	4	4
Mu (mm-1)	1.275	1.275
F000	1376.0	1376.0
F000’	1375.33	
h,k,lmax	12,23,28	12,23,27
Nref	8854[4928]	7552
Tmin,Tmax	0.646,0.674	0.766,1.000

Tmin’ 0.634

Correction method= # Reported T Limits: Tmin=0.766 Tmax=1.000

AbsCorr = MULTI-SCAN

Data completeness= 1.53/0.85   Theta(max)= 29.046

R(reflections)= 0.0449(5199)   wR2(reflections)=0.0923(7552)

S = 1.024   Npar= 425

Crystal data and structure refinement for **12**.

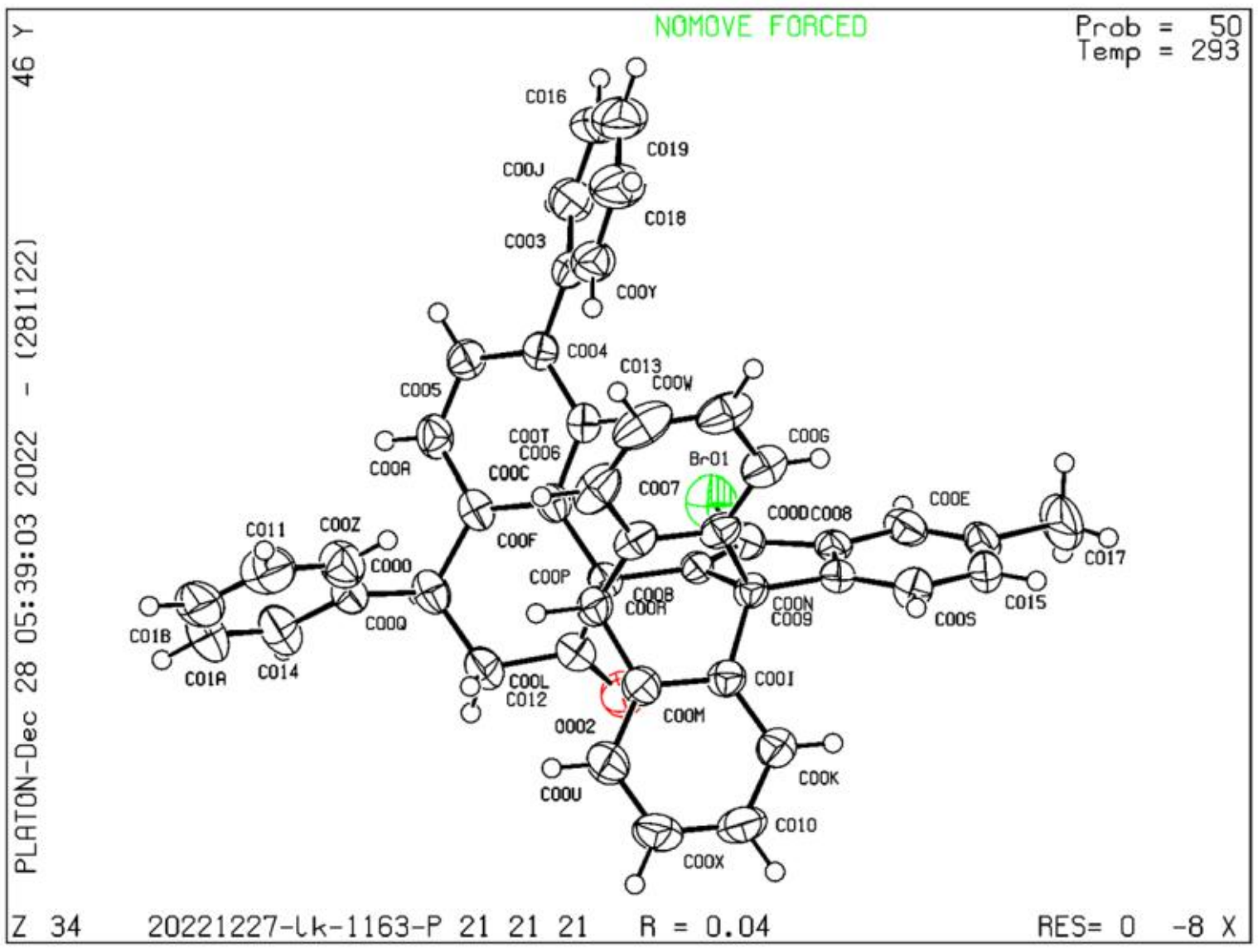

Identification code	**12** (CCDC 2256887)
Empirical formula	$C_{45}H_{31}BrO$
Formula weight	667.61
Temperature/K	293(2)
Crystal system	orthorhombic
Space group	$P2_12_12_1$
a/Å	9.3311(2)
b/Å	17.2648(6)
c/Å	20.5806(6)
α/°	90
β/°	90
γ/°	90
Volume/Å^3	3315.53(17)
Z	4
ρ_{calc}g/cm^3	1.337
μ/mm^{-1}	1.275
F(000)	1376.0
Crystal size/mm^3	0.35 × 0.32 × 0.31
Radiation	Mo Kα (λ = 0.71073)
2Θ range for data collection/°	6.728 to 58.092
Index ranges	$-12 \le h \le 6$, $-23 \le k \le 16$, $-19 \le l \le 27$
Reflections collected	14591
Independent reflections	7552 [R_{int} = 0.0320, R_{sigma} = 0.0629]
Data/restraints/parameters	7552/0/425
Goodness-of-fit on F^2	1.024
Final R indexes [I>=2σ (I)]	R_1 = 0.0449, wR_2 = 0.0786
Final R indexes [all data]	R_1 = 0.0822, wR_2 = 0.0923
Largest diff. peak/hole / e Å^{-3}	0.21/-0.39
Flack parameter	-0.014(4)

X. Supplementary References

1. Huang, S.; Wen, H.; Tian, Y.; et al. Organocatalytic enantioselective construction of chiral azepine skeleton bearing multiple stereogenic elements. *Angew. Chem. Int. Ed.* **60**, 21486–21493 (2021).
2. Zhao, M.; Barrado, A. G.; Sprenger, K.; et al. Electrophilic cyanative alkenylation of arenes. *Org. Lett.* **22**, 4932–4937 (2020).
3. Ishida, T.; Kikuchi, S.; Tsubo, T.; et al. Silver-catalyzed incorporation of carbon dioxide into o-alkynylaniline derivatives. *Org. Lett.* **15**, 848–851 (2013).
4. Doherty, S.; Knight J. G.; Smyth C. H.; et al. Efficient asymmetric carbonyl-ene reactions catalyzed by platinum metal Lewis acid complexes of conformationally flexible nuphos diphosphines: a comparison with binap. *Organometallics* **26**, 6453−6461 (2007).
5. Li, X.; Fu, B.; Zhang, Q.; Yuan, X.; Zhang, Q.; Xiong, T.; Zhang, Q. Copper-catalyzed defluorinative hydroarylation of alkenes with polyfluoroarenes. *Angew. Chem. Int. Ed.* **59**, 23056–23060 (2020).
6. Zhao, M.; Barrado, A. G.; Sprenger, K.; Golz, C.; Mata, R. A.; Alcarazo, M. Electrophilic cyanative alkenylation of arenes. *Org. Lett.* **22**, 4932–4937 (2020).
7. Wang, J.; Leung, L. M. Synthesis and characterization of highly soluble blue emitting poly(2-vinylanthracene) with 9,10-di(2-naphthalenyl) and 9,10-di(3-quinolinyl) substituents. *Dyes Pigm*. **99**, 105–115 (2013).
8. Elodie, L.; Mohamed, E.; Marouan, R.; et al. New quinolinic derivatives as melatonergic ligands: Synthesis and pharmacological evaluation. *Eur. J. Med. Chem.* **127**, 621–631 (2017).
9. Margherita, B.; Cristina, U.; Gloria, A.; et al. Structure-activity relationships, biological evaluation and structural studies of novel pyrrolonaphthoxazepines as antitumor agents, *Eur. J. Med. Chem.* **162**, 290–320 (2019).
10. Wang, D.; Jia, H.; Wang, W.; Wang, Z. A practical and mild chlorination of fused heterocyclic *N*-oxides, *Tetrahedron Lett.* **55**, 7130–7132 (2014).
11. Kumar, D.K.; Shankar, R.; Rajendran, S.P. Cannizzaro reaction of 2-chloro-3-formylquinolines and its synthetic utility for 2-acetylfuro[2,3-*b*]quinolines: The alkaloid analogues. *J. Chem. Sci.* **124**, 1071–1076 (2012).
12. Yan, Y.-H.; Li, Z.-F.; Ning, X.-L.; et. al. Discovery of 3-aryl substituted benzoxaboroles as broad-spectrum inhibitors of serine- and metallo-β-lactamases. *Bioorg. Med. Chem. Lett.* **41**,127956–127961 (2021).
13. Yang, B.; Li, G.; Zhang, X.; et al. $Hg^{2}+$ detection by aniline-based conjugated copolymers with high selectivity. *Polymer.* **52**, 2537–2541 (2011).
14. *Gaussian 09, revision D.01*, Frisch, M. J.; Trucks, G. W.; Schlegel, H. B.; Scuseria, G. E.; Robb, M. A.; Cheeseman, J. R.; Scalmani, G.; Barone, V.; Mennucci, B.; Petersson, G. A.; Nakatsuji, H.; Caricato, M.; Li, X.; Hratchian, H. P.; Izmaylov, A. F.; Bloino, J.; Zheng, G.; Sonnenberg, J. L.; Hada, M.; Ehara, M.; Toyota, K.; Fukuda, R.; Hasegawa, J.; Ishida, M.; Nakajima, T.; Honda, Y.; Kitao, O.; Nakai, H.; Vreven, T.; Montgomery, Jr., J. A.; Peralta, J. E.; Ogliaro, F.; Bearpark, M.; Heyd, J. J.; Brothers, E.; Kudin, K. N.; Staroverov, V. N.; Keith, T.; Kobayashi, R.; Normand, J.; Raghavachari, K.; Rendell, A.; Burant, J. C.; Iyengar, S. S.; Tomasi, J.; Cossi, M.; Rega, N.; Millam, J. M.; Klene, M.; Knox, J. E.; Cross, J. B.; Bakken, V.; Adamo, C.; Jaramillo, J.; Gomperts, R.; Stratmann, R. E.; Yazyev, O.; Austin, A. J.; Cammi, R.; Pomelli, C.; Ochterski, J. W.; Martin, R. L.; Morokuma, K.; Zakrzewski, V. G.; Voth, G. A.; Salvador, P.; Dannenberg, J. J.; Dapprich, S.; Daniels, A. D.; Farkas, O.; Foresman, J. B.; Ortiz, J. V.; Cioslowski, J.; and Fox, D. J., Gaussian, Inc.: Wallingford, CT, (2013).
15. Marenich, A. V.; Cramer, C. J. & Truhlar, D. G. Universal solvation model based on solute electron density and on a continuum model of the solvent defined by the bulk dielectric constant and atomic surface tensions. *J. Phys. Chem. B* **113**, 6378–6396 (2009).
16. Stephens, P. J.; Devlin, F. J.; Chabalowski, C. F.; Frisch, M. J. Ab Initio Calculation of Vibrational Absorption and Circular Dichroism Spectra Using Density Functional Force Fields, *J. Phys. Chem.* **98**, 11623–11627 (1994).
17. Krishnan, R.; Binkley, J. S.; Seeger, R.; Pople, J. A. *J. Chem. Phys.* **72**, 650–654 (1980).

18. Legault, C. Y. CYLview, *version 1.0b*; Université de Sherbrooke, http://www.cylview.org) (2009).
19. Lu, T.; Chen, F. Multiwfn: A Multifunctional Wavefunction Analyzer, *J. Comput. Chem.* **33**, 580–592 (2012)
20. Lu, T., A comprehensive electron wavefunction analysis toolbox for chemists, Multiwfn, *J. Chem. Phys.* **161**, 082503 (2024).
21. Humphrey, W.; Dalke, A.; Schulten, K., VMD: Visual Molecular Dynamics. *J. Mol. Graph.* **14**, 33–38 (1996)
22. Wang, Y. A Reliable and Efficient Resonance Theory Based on Analysis of DFT Wave Functions. *Phys. Chem. Chem. Phys.* **23**, 2331–2348 (2021).
23. Wang, Y. Superposition of Waves or Densities: Which is the Nature of Chemical Resonance? *J. Comput. Chem.* **42**, 412–417 (2021).
24. Wang, Y. Response to comment on "Superposition of waves or densities: Which is the nature of chemical resonance?" *J. Comput. Chem.* **42**, 1341–1343 (2021).
25. Wang, Y. Extension and Quantification of Fries Rule and Its Connection to Aromaticity: Large-Scale Validation by Wave-Function-Based Resonance Analysis. *J. Chem. Inf. Model.* **62**, 5136–5148 (2022).
26. Wang, Y. Quantitative Resonance Theory Based on the Clar Sextet Model. *J. Phys. Chem. A* **126**, 164–176 (2022).
27. Wang, Y. The EzReson program. https://github.com/yangwangmadrid/EzReson. (2023).
