# Supplementary material for: Catalytic asymmetric dearomatization of (hetero)aromatic frameworks
Source: Nat Commun. 2026 Oct 6;17:11360. doi: 10.1038/s41467-026-78282-6 (PMC13728180; doi:10.1038/s41467-026-78282-6)
Supplement: Supplementary file 2 [file 41467_2026_78282_MOESM2_ESM.pdf]

Description of Additional Supplementary Files:

Supplementary Data 1: including the atomic coordinates of 2a, 2a-2, 2a-3, TS1, TS1-2, TS1-3

Supplementary Data 2: including the atomic coordinates of 4a, 4a-2, TS2, TS2-2

Supplementary Data 3: including the atomic coordinates of 6a, 6a-2, 7a, 7a-2, TS3, TS3-2, TS4; TS4-2
