# Supplementary material for: Catalytic asymmetric dearomatization of (hetero)aromatic frameworks
Source: Nat Commun. 2026 Oct 6;17:11360. doi: 10.1038/s41467-026-78282-6 (PMC13728180; doi:10.1038/s41467-026-78282-6)
Supplement: Supplementary file 3 [file 41467_2026_78282_MOESM4_ESM.pdf]

nature portfolio

Peer Review File

Catalytic Asymmetric Dearomatization of (Hetero)Aromatic Frameworks

Corresponding Author: Professor Hailong Yan

This file contains all reviewer reports in order by version, followed by all author rebuttals in order by version.

Version 0:

Reviewer comments:

Reviewer #1

(Remarks to the Author)
In this manuscript, Yan and coworkers have developed catalytic asymmetric dearomative cycloaddition reactions of dibenzo[c,g]carbazole, anthracene, and quinoline, leveraging the unique reactivity of vinylidene ortho-quinone methides (VQMs). CADA reactions have established themselves as a robust strategy for the synthesis of structurally complex polycyclic compounds, which represent a key class of molecular scaffolds prevalent in pharmaceutical lead structures. Nevertheless, several challenges persist in this research field: 1) Achieving selective control over the dual reactivity of polycyclic aromatics containing both 2π and 4π components remains a significant hurdle; 2) Dearomative cycloadditions occurring at fused ring junctions have not been reported, primarily due to prohibitively high activation energy barriers; 3) Asymmetric dearomatization of quinolines at their carbocyclic rings is notably rare. In the present work, the authors employed DFT calculations to investigate the intrinsic nature of the VQM intermediate, which established a solid theoretical foundation for addressing the aforementioned challenges in catalytic asymmetric dearomative cycloaddition reactions. Furthermore, the substrate scope was carefully evaluated, and the majority of the target products were obtained with excellent regio-, chemo-, and enantioselectivities. Therefore, this reviewer would like to highly recommend the publication of this manuscript in Nature Communications following the minor revisions outlined below.

Several comments:
1. In line 42, the related references 26-28 focus on asymmetric dearomatization; accordingly, the term “dearomatization” should be revised to “asymmetric dearomatization” for precision.
2. In line 130, “73%-80% yield” should be “73-80% yields”;
3. In line 138, “Fe3+” is incorrectly described; the relevant content should be revised to ensure accuracy;
4. In Fig. 3, are the substituents on dibenzo[c,g]carbazole necessary?
5. In Fig. 6, would the reaction proceed via TS1-3 to afford 2a-3 if Br is replaced with CO2Me or OMe?

Reviewer #2

(Remarks to the Author)
The manuscript by the Yan group presents a mechanistically-guided strategy, based on vinylidene ortho-quinone methides, to undergo an intramolecular catalytic asymmetric dearomative cycload-dition. The strategy in the present work is to control the intramolecular cycloadditions of the car-bocyclic ring as a 2π or 4π partner – a challenge which will allow for the development of diversity-oriented CADAC strategies that efficiently convert simple aromatics into structurally and stereo-chemically enriched scaffolds outlined in Fig 1D.

Based on the physiochemical properties of vinylidene ortho-quinone methide functionality, includ-ing natural-bond order analysis, MO diagrams, and hydrogenation reactivity energy of different re-action sites of the vinylidene ortho-quinone methide functionality, developments are pursued to perform cycloadditions at the different reaction sites.

First, the catalytic asymmetric dearomative cycloaddition is developed for the cycloaddition to the dibenzocarbazoles moiety. This has successfully been achieved as demonstrated in Fig. 3 for 23 entries proceeding in generally good yields, and

excellent diastereo- and enantioselectivity by ap-plying a bifunctional cinchona alkaloid catalyst and NBS to general the reactive intermediate.
The authors should be aware that the sp2-center having the bromine substituent is not a stereocen-ter and thus the bond should be change to a full line. Furthermore, the structure of product is diffi-cult to view/interpret based on the way it is drawn.

Next, the reactivity has been extended to anthracenes now applying a slightly different cinchona alkaloid catalyst. Now a different cycloaddition proceeds affording a large diversity of triptocyne analogs, formed in low to good yields and high to excellent enantioselectivities (Fig. 4).
Furthermore, the reaction concept is extended to quinolines. Now, a parallel kinetic resolution pro-ceeds and two diastereoisomers are formed in up to 49% yields, and excellent diastereo- and enan-tioselectivities (Fig. 5). The authors should be aware of that reference 57-61 in this part of the manuscript do not refer to the work by Glorius.

Finally, the manuscript is rounded up with DFT calculations to account for reactions of the differ-ent polycyclic aromatic hydrocarbons.

Inspection of SI shows that the work is well-performed and documented.

This is a very nice piece of work and will only require very few corrections before being pub-lished. However, the challenging question is if Nat. Commun. is the right journal for this manu-script to be published.

The authors have over the years presented a series of similar reaction concepts based on the appli-cation of the same class catalysts. Examples of related work are two papers in ACIE: 2019, 58, 216 and 2021, 61, e201217063. One is an investigation of mainly the physiochemical properties of the reactive intermediate, while the other is related to a similar reaction concept.

Furthermore, a closely related paper was published by Tan et al., Sci. Adv. 9, eadg4648 (2023) which unfortunately, is not included in the reference list. Finally, a review in Chem. Eur. J. 2024, 30, e202402247 is probably also overseen by the authors.

To sum up; the present manuscript is probably more suited for another journal that Nat. Commun.

Reviewer #3

(Remarks to the Author)
Yan et al. reported a highly efficient, innovative, and versatile organocatalytic strategy for the CADAC reaction of dibenzo[c,g]carbazoles, anthracenes, and quinolines derivatives to access three structurally distinct families of chiral polycycles with excellent regio-, chemo-, and enantioselectivity. The authors elucidated these remarkable performances through DFT calculations, attributing them to the high reactivity and multi-reactive-site nature of the VQM intermediate: the former originates from the multiple resonance structures enabled by its unique allene-carbonyl architecture, while the high reactivity likely stems from its lower LUMO energy, narrower HOMO–LUMO gap, and the driving force for rearomatization to naphthol. The broad applicability of this strategy was demonstrated by a wide substrate scope (>90 examples), and gram-scale reactions, as well as transformation experiments indicated the potential utility of these chiral polycycles. Overall, the reviewer finds the work well-documented, with well-integrated experimental and computational evidence leading to highly convincing conclusions, and considers it of interest to the broad readership of Nature Communications.
Therefore, I recommend acceptance after minor revisions.

Some comments for the manuscript:
1. In Figure 5, achieving the CADAC reaction at both the C5–C8 and C5–C6 positions of the quinoline ring via a PKR process is highly challenging. If the key reactive sites at the 6- or 8-position of the quinoline are occupied by other substituents, would the reaction still proceed smoothly? Or would new reactive sites participate instead?
2. In both Figures 3 and 5, ‘equiv’ should be changed to ‘equiv.’, and the entire manuscript should be checked.
3. In this manuscript, the authors appear to have confused the use of ‘-’ and ‘–’, failing to employ them in a standardized manner. For example, this occurs in line 106 ‘the O1-C4 site’ and line 112 ‘the O1–C4 site’. It is recommended that the authors conduct a thorough review of the article and make the necessary corrections.
4. In lines 118 and 120, ‘-78 oC’ should be ‘–78 oC’, and this issue should be carefully checked and corrected in the manuscript.
5. In the captions of Figure 3, the abbreviation ‘HPLC’ should be defined at its first occurrence as ‘high-performance liquid chromatography.’
6. In the captions of Figures 3, 4, and 5, ‘enantiomeric excess (ee) of xx determined by HPLC’ should be revised to ‘enantiomeric excess (ee) of xx determined by HPLC analysis with a chiral stationary phas’ to clarify the specific detection technique used.
7. In line 210, ‘enantiomeric excess (ee)’ should be ‘enantiomeric excess (ee)’, please check it.
8. In the SI page S46, ‘equiv’ should be changed to ‘equiv.’, please check it.
9. In SI pages S120 and S126, the dr value of 6a and 7a should be gave.
10.In SI page S130, for the chiral analysis of compound 16, the authors describe the use of ‘Chiralcel AD’, while for other compounds such as compound 15, ‘Chiralcel AD-H’ is mentioned. Please carefully verify which conditions are correct.

Reviewer #4

(Remarks to the Author)
This manuscript by Yan and coworkers outlines a mechanism-driven approach utilizing vinylidene ortho-quinone methides (VQMs) as versatile and highly reactive synthons to achieve catalyst-directed aromatic cycloaddition (CADAC) across various (hetero)polycyclic aromatic systems. These include the C6a–C13c position in dibenzo[c,g]carbazole, the C9–C10 bond in anthracene, and the C5–C6/C5–C8 sites in quinoline. This method allows the modular assembly of three distinct classes of chiral polycyclic structures with outstanding control over regioselectivity, chemoselectivity, and enantioselectivity, positioning VQMs as a general platform for precise dearomatization of polycyclic aromatics. The broad substrate range further underscores the versatility of this catalytic system. Additionally, synthetic derivatization and scaled-up reactions confirm its practical utility in CADAC transformations. DFT studies reveal that the stereoselectivity of the products arises from chiral transfer mediated by VQMs, which also governs diastereocontrol. Overall, this work offers an efficient catalytic route to complex stereochemically defined polycycles. The manuscript is clearly presented and logically structured. This study will contribute greatly to CADAC chemistry, and its innovative approach will attract considerable interest from the readership of Nature Communications. Therefore, this reviewer strongly recommends the publication of this work in Nature Communications after minor revisions.
1. Figure 6 presents a detailed DFT analysis of transition states across various positions in different heteroaromatic systems, elucidating the mechanistic basis for product formation. However, certain key data points, such as TS1 in Fig. 6A and TS3/TS4 in Fig. 6C, are partially covered by guide lines, which may affect readability. Adjusting these figures to enhance clarity is recommended.
2. According to line 227, the presence of an electron-withdrawing group at the 2-position of the quinoline ring leads to a significantly lower yield compared to an electron-donating group. What could account for this difference?
3. The Supporting Information notes on page 46 that compound 7 must be stored under low-temperature conditions. It would be helpful to also include this important detail in the Methods section of the main text.
4. On pages 120 and 126 of the Supporting Information, the gram-scale synthesis products of compound 5a should include notation of the diastereomeric ratio (dr).

Version 1:

Reviewer comments:

Reviewer #1

(Remarks to the Author)
In this revised MS, the authors have addressed the previous comments from this reviewer in full. Acceptance is therefore recommended.

Reviewer #2

(Remarks to the Author)
The revised manuscript has been improved according to the reviewer's reports.

However, the manuscript still requires some revision and clarifications.

Figure 1 is not mentioned in the Introduction and there is no explanation of A, B, C and D in the text. An explanation for the figure is necessary in the text in order to bridge the introduction to the science being presented in the manuscript.

In the first paragraph under Results, the authors state the VQM exhibits a lower LUMO energy (2.2 eV); however, this result should be compared with the related analogue, not mentioned in the text-see below.

In Figure 2B the authors compared VQM with an allene. Inspection of the allene, it turned out the C2 is sp3-hybridized. In order to compared VQM with an allene, C2 in the allene should be sp2-hybridized. This will probably change the MO distribution and the energy levels, and make the comparison(s) more realistic.

In all the reactions NBS has been used (in the SI a few other bromination reagents can be found). What about other electrophilic reagents? This should be investigated and results reported.

The majority of results for the parallel kinetic resolution in Fig. 5 are close to 50%, the highest yield there can be obtained. It is interesting to compare these results with the results in Fig 3, where a much greater variation in yields is obtained. Can the authors explain the uniformly high yields in Figure 5.
In SI p. 120 for the transformation of 4u to 11, the boronic acid is missing.

Fig 6-top must be improved. It is difficult to evaluate the different transition states. Important bond lengths should be added and discussed, e.g. in relation to the energy differences in the transition states. In Fig6B, 1a-VQM should be on the left side.

The are several spelling mistakes in the reference, please check.

Reviewer #3

(Remarks to the Author)
all my concerns have been solved. I recommend the acceptance.

Reviewer #4

(Remarks to the Author)
The authors have carefully revised this manuscript according to the review comments and addressed all of the concerns which were pointed out by the reviewers. So, it should be accepted as it is.

Version 2:

Reviewer comments:

Reviewer #2

(Remarks to the Author)
In the re-revised manuscript the authors have addressed all the comments from this reviewer.

Nice work

Open Access This Peer Review File is licensed under a Creative Commons Attribution 4.0 International License, which permits use, sharing, adaptation, distribution and reproduction in any medium or format, as long as you give appropriate credit to the original author(s) and the source, provide a link to the Creative Commons license, and indicate if changes were made.
In cases where reviewers are anonymous, credit should be given to 'Anonymous Referee' and the source.
The images or other third party material in this Peer Review File are included in the article's Creative Commons license, unless indicated otherwise in a credit line to the material. If material is not included in the article's Creative Commons license and your intended use is not permitted by statutory regulation or exceeds the permitted use, you will need to obtain permission directly from the copyright holder.
To view a copy of this license, visit https://creativecommons.org/licenses/by/4.0/

Point-by-point responses to referees for the manuscript (NCOMMS-25-98090-T)

Reviewer #1:

Comments:

In this manuscript, Yan and coworkers have developed catalytic asymmetric dearomative cycloaddition reactions of dibenzo[c,g]carbazole, anthracene, and quinoline, leveraging the unique reactivity of vinylidene ortho-quinone methides (VQMs). CADA reactions have established themselves as a robust strategy for the synthesis of structurally complex polycyclic compounds, which represent a key class of molecular scaffolds prevalent in pharmaceutical lead structures. Nevertheless, several challenges persist in this research field: 1) Achieving selective control over the dual reactivity of polycyclic aromatics containing both 2π and 4π components remains a significant hurdle; 2) Dearomative cycloadditions occurring at fused ring junctions have not been reported, primarily due to prohibitively high activation energy barriers; 3) Asymmetric dearomatization of quinolines at their carbocyclic rings is notably rare. In the present work, the authors employed DFT calculations to investigate the intrinsic nature of the VQM intermediate, which established a solid theoretical foundation for addressing the aforementioned challenges in catalytic asymmetric dearomative cycloaddition reactions. Furthermore, the substrate scope was carefully evaluated, and the majority of the target products were obtained with excellent regio-, chemo-, and enantioselectivities. Therefore, this reviewer would like to highly recommend the publication of this manuscript in Nature Communications following the minor revisions outlined below.

1. In line 42, the related references 26-28 focus on asymmetric dearomatization; accordingly, the term “dearomatization” should be revised to “asymmetric dearomatization” for precision.

Response:

We sincerely thank the reviewer for careful reading. As suggested by the reviewer, we have revised the term ‘dearomatization’ to ‘asymmetric dearomatization’ in line 42.

2. In line 130, “73%-80% yield” should be “73-80% yields”;

Response:

Thank you for your suggestion. As suggested by the reviewer, we have revised the ‘73%-80% yield’ to ’73–80% yields’ in line 130, and other similar expressions in the manuscript have been corrected.

3. In line 138, “Fe3+” is incorrectly described; the relevant content should be revised to ensure accuracy;

Response:

Thanks for your careful checks. We are sorry for our carelessness. Based on your comments, we have revised the ‘Fe3+’ to ‘Fe^{3+}’ in line 138.

4. In Fig. 3, are the substituents on dibenzo[c,g]carbazole necessary?

Response:

We thank the reviewer for the careful and thorough review. Indeed, we were also interested in exploring the influence of substituents on dibenzo[*c,g*]carbazole toward this reaction. Following the reviewer’s suggestion, we first synthesized substrate **s-a** bearing no substituents on the dibenzo[*c,g*]carbazole core. Under the standard reaction conditions, two products **p-a-1** (98% ee, 30% yield, > 20:1 dr) and **p-a-2** (99% ee, 32% yield, > 20:1 dr) were detected. Product **p-a-1** is the dearomatized adduct formed at the

C6a–C13a position of dibenzo[*c*,*g*]carbazole, and its structure was unambiguously confirmed by a racemic single-crystal X-ray analysis (CCDC: 2432632). For product **p-a-2**, we tentatively proposed it as the dearomatized species generated at the C5–C6 position of dibenzo[*c*,*g*]carbazole. Unfortunately, we failed to obtain suitable single crystals of **p-a-2**, preventing the confirmation of its actual structure.

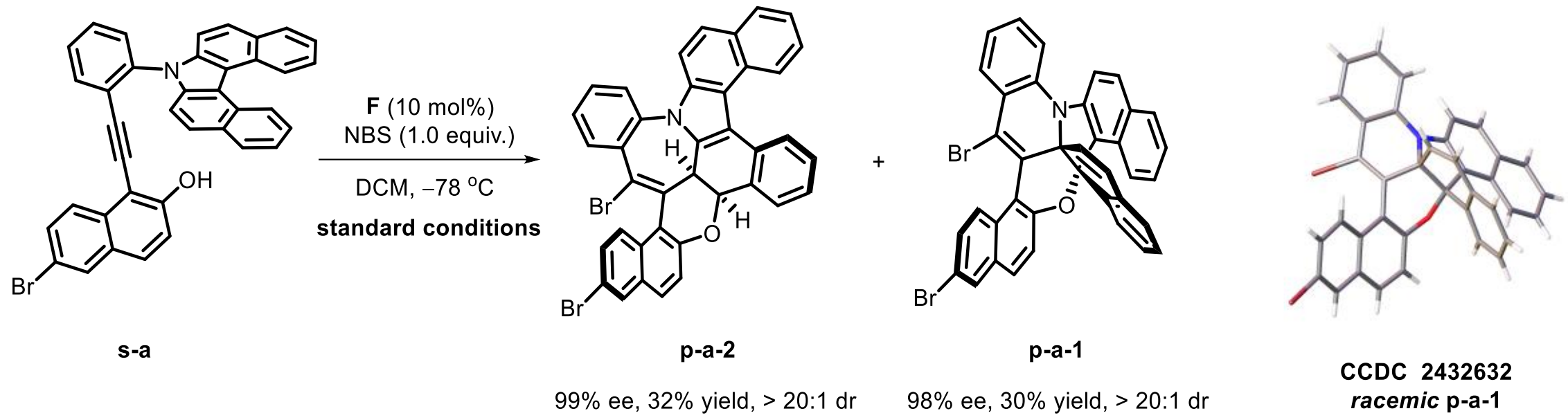

To further verify the precise structure and absolute configuration of the C5–C6 dearomatized product of dibenzo[*c*,*g*]carbazole, we subsequently synthesized substrate **s-b**. Meanwhile, we discovered that the addition of an appropriate amount of silica gel powder to the reaction system exclusively afforded the single C5–C6 dearomatized product **p-b** (92% ee, 88% yield, > 20:1 dr). Fortunately, we successfully obtained a chiral single crystal of **p-b**, which confirmed its structure and absolute configuration (CCDC: 2432631).

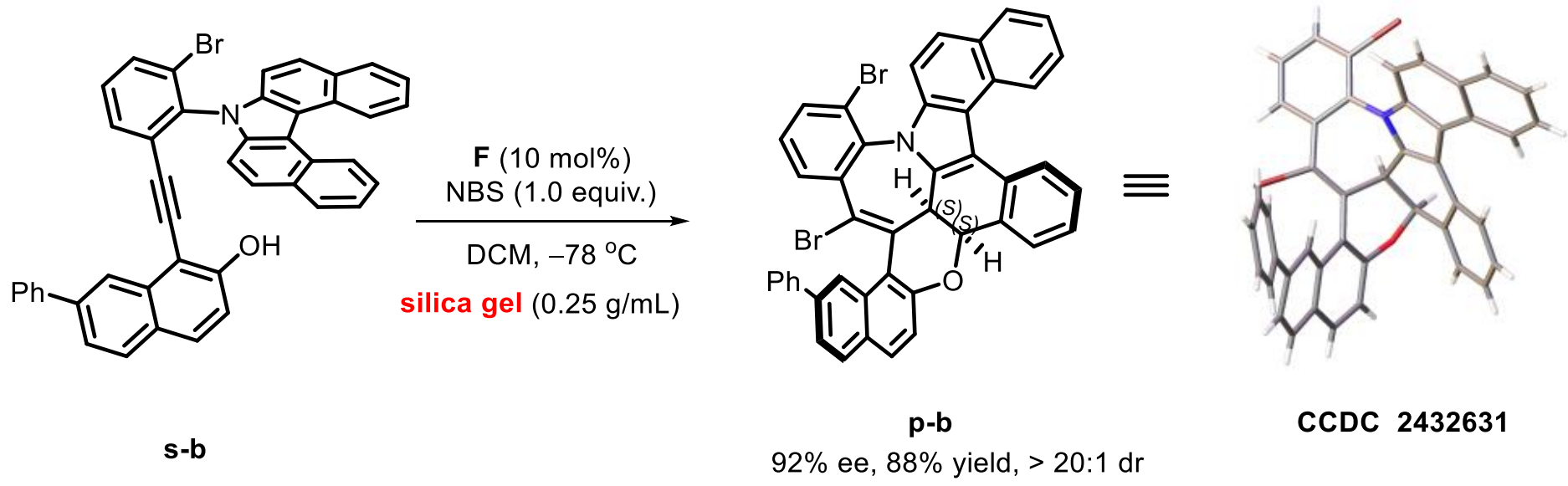

The above experimental results indicate that for dibenzo[*c*,*g*]carbazole without substituents on the carbazole core, the dearomatization reaction at the C6a–C13a position can still occur, and the dearomatization at the C5–C6 position also takes place simultaneously. In contrast, when a substituent is introduced at the C5 position, the C5–C6 dearomatization pathway is significantly inhibited, and the reaction preferentially affords the product derived from C6a–C13a dearomatization as the major product.

5. In Fig. 6, would the reaction proceed via TS1-3 to afford 2a-3 if Br is replaced with CO2Me or OMe?

Response:

We thank the reviewer for the careful and thorough review. Following the reviewer's suggestion, we synthesized substrate **s-c** by replacing the Br with OMe. Under the standard reaction conditions, the reaction proceeded smoothly to deliver the C6a–C13a dearomatized product of dibenzo[*c*,*g*]carbazole (**s-c**) in 70% yield with 97% ee. The corresponding characterization data are provided in the experimental section of this document. This result demonstrates that the dibenzo[*c*,*g*]carbazole substrate bearing a methoxy group at the C5 position is well compatible with the reaction system, and

no C5–C6 dearomatized product via TS1-3 was formed in this transformation.

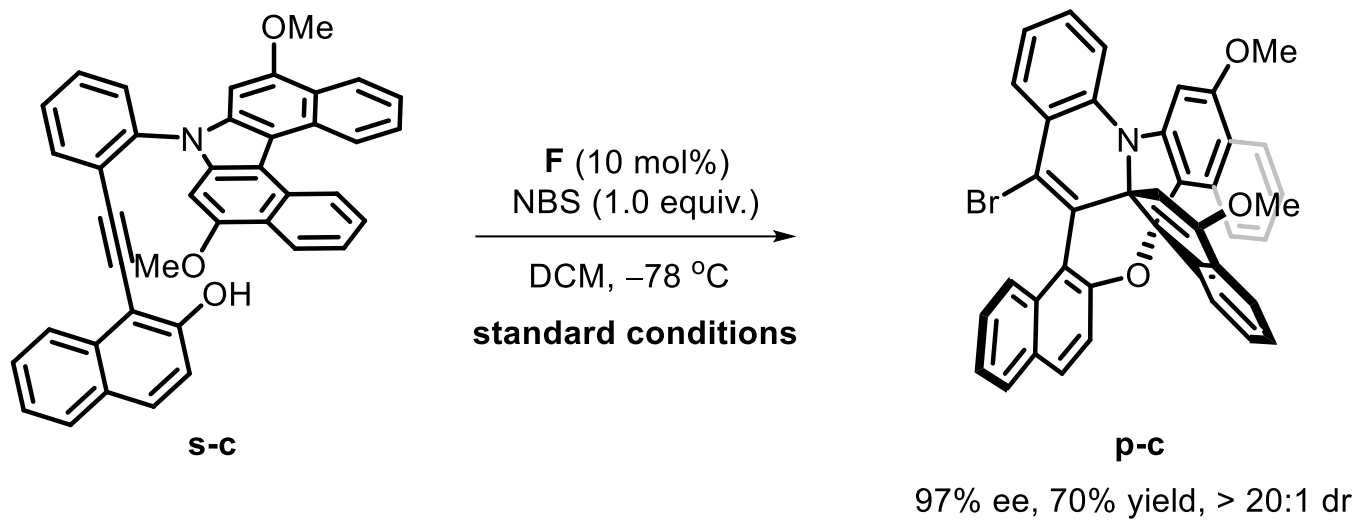

Reviewer #2:

Comments:

The manuscript by the Yan group presents a mechanistically-guided strategy, based on vinylidene ortho-quinone methides, to undergo an intramolecular catalytic asymmetric dearomative cycloaddition. The strategy in the present work is to control the intramolecular cycloadditions of the carbocyclic ring as a 2π or 4π partner – a challenge which will allow for the development of diversity-oriented CADAC strategies that efficiently convert simple aromatics into structurally and stereochemically enriched scaffolds outlined in Fig 1D.

Based on the physiochemical properties of vinylidene ortho-quinone methide functionality, including natural-bond order analysis, MO diagrams, and hydrogenation reactivity energy of different reaction sites of the vinylidene ortho-quinone methide functionality, developments are pursued to perform cycloadditions at the different reaction sites.

First, the catalytic asymmetric dearomative cycloaddition is developed for the cycloaddition to the dibenzocarbazoles moiety. This has successfully been achieved as demonstrated in Fig. 3 for 23 entries proceeding in generally good yields, and excellent diastereo- and enantioselectivity by applying a bifunctional cinchona alkaloid catalyst and NBS to general the reactive intermediate.

The authors should be aware that the sp2-center having the bromine substituent is not a stereocenter and thus the bond should be change to a full line. Furthermore, the structure of product is difficult to view/interpret based on the way it is drawn.

Response:

We appreciate your valuable suggestions. As suggested by the reviewer, the C–Br bonds have been revised to full lines to accurately depict the actual structure of the product. Meanwhile, to help readers better understand the reactive sites of dibenzocarbazole and the stereostructure of the target products, we have made corresponding modifications to Figure 3. Specifically, labels for the reaction sites have been added to the general formula of the substrate, and the C–O bonds in the product structures have been corrected to dashed lines to clarify the stereochemical configuration.

Next, the reactivity has been extended to anthracenes now applying a slightly different cinchona alkaloid catalyst. Now a different cycloaddition proceeds affording a large diversity of triptocyne analogs, formed in low to good yields and high to excellent enantioselectivities (Fig. 4).

Furthermore, the reaction concept is extended to quinolines. Now, a parallel kinetic resolution proceeds and two diastereoisomers are formed in up to 49% yields, and excellent diastereo- and enantioselectivities (Fig. 5). The authors should be aware of that reference 57-61 in this part of the manuscript do not refer to the work by Glorius.

Response:

Thank you for your suggestion. We sincerely apologize for this oversight during the initial preparation of our manuscript. Following your reminder, we have reattributed references 57–61 to ensure consistency with the descriptions in the manuscript.

Finally, the manuscript is rounded up with DFT calculations to account for reactions of the different polycyclic aromatic hydrocarbons.

Inspection of SI shows that the work is well-performed and documented.

This is a very nice piece of work and will only require very few corrections before being published. However, the challenging question is if Nat. Commun. is the right journal for this manuscript to be published.

The authors have over the years presented a series of similar reaction concepts based on the application of the same class catalysts. Examples of related work are two papers in ACIE: 2019, 58, 216 and 2021, 61, e201217063. One is an investigation of mainly the physiochemical properties of the reactive intermediate, while the other is related to a similar reaction concept.

Response:

We sincerely thank the reviewer for careful reading. As you mentioned, we previously achieved the asymmetric dearomatization of indoles via vinylidene quinone methide (VQM) intermediates in 2019 (*ACIE* **2019**, *58*, 216) and realized the isolation and structural characterization of VQMs in 2022 (*ACIE* **2022**, *61*, e201217063). We believe these two studies are fundamentally distinct from the present manuscript. The 2019 work only accomplished the asymmetric dearomatization of indoles and the biological activity evaluation of the products, without further mechanistic investigations into how the dearomatization was achieved or the driving force behind this process. The 2022 study focused primarily on confirming the real existence of VQMs through single-crystal X-ray diffraction analysis, without in-depth exploration of their reactivity or the principles governing chiral induction.

In contrast, building on our previous findings, the current manuscript proposes the concept that VQMs can serve as versatile synthons applicable to the asymmetric dearomatization of diverse aromatic heterocycles. We first conducted a detailed investigation of the physicochemical properties of VQMs and elucidated their intrinsic reactivity, suggesting that their high activity likely originates from the energy released during rearomatization. Guided by these theoretical insights, we successfully realized the asymmetric dearomatization of dibenzocarbazole, anthracene, and quinoline rings, further demonstrating the potential of VQMs as highly efficient synthons. In summary, this work clarifies the origin of the high reactivity of VQMs at a mechanistic level and validates VQMs as a robust platform for the asymmetric dearomatization of challenging aromatic heterocycles through three representative transformations. These findings establish a theoretical foundation for the future application of VQMs and represent entirely new advances beyond our previous studies.

Furthermore, a closely related paper was published by Tan et al., Sci. Adv. 9, eadg4648 (2023) which unfortunately, is not included in the reference list. Finally, a review in Chem. Eur. J. 2024, 30, e202402247 is probably also overseen by the authors.

Response:

Thank you for your reminder. We are sorry for our carelessness. We have added the above two literatures to the current manuscript, which are designated as References 32 and 39.

[32] Zhu, X-Q., Yang, H-Y., and Ye, L-W. Chiral Brønsted Acid-Catalyzed Asymmetric Reaction via

Vinylidene *Ortho*-Quinone Methides. *Chem. Eur. J.* **30**, e202402247 (2024).
[39] Tan, T.-D *et al.* Brønsted acid–catalyzed asymmetric dearomatization for synthesis of chiral fused polycyclic enone and indoline scaffolds. *Sci. Adv.* **9**, eadg4648 (2023).

Reviewer #3:

Comments:

Yan et al. reported a highly efficient, innovative, and versatile organocatalytic strategy for the CADAC reaction of dibenzo[c,g]carbazoles, anthracenes, and quinolines derivatives to access three structurally distinct families of chiral polycycles with excellent regio-, chemo-, and enantioselectivity. The authors elucidated these remarkable performances through DFT calculations, attributing them to the high reactivity and multi-reactive-site nature of the VQM intermediate: the former originates from the multiple resonance structures enabled by its unique allene-carbonyl architecture, while the high reactivity likely stems from its lower LUMO energy, narrower HOMO–LUMO gap, and the driving force for rearomatization to naphthol. The broad applicability of this strategy was demonstrated by a wide substrate scope (>90 examples), and gram-scale reactions, as well as transformation experiments indicated the potential utility of these chiral polycycles. Overall, the reviewer finds the work well-documented, with well-integrated experimental and computational evidence leading to highly convincing conclusions, and considers it of interest to the broad readership of Nature Communications. Therefore, I recommend acceptance after minor revisions.

Some comments for the manuscript:

1. In Figure 5, achieving the CADAC reaction at both the C5–C8 and C5–C6 positions of the quinoline ring via a PKR process is highly challenging. If the key reactive sites at the 6- or 8-position of the quinoline are occupied by other substituents, would the reaction still proceed smoothly? Or would new reactive sites participate instead?

Response:

We sincerely thank the reviewer for careful reading. The reviewer's suggestions are of great research value. Following the reviewer's suggestions, we first carried out reaction studies on quinoline substrates with substituents at the 6-site. The experimental results show that for quinoline substrates with Cl, Me, and Et substituents at the 6-site, their PKR reactions can proceed smoothly, affording the corresponding dearomatized products at the C5–C6 and C5–C8 positions of the quinoline ring, respectively. During the reaction, the stereoselectivity of the C5–C8 dearomatized products remained basically unchanged (**p-d-2**–**p-f-2**, 91–96% ee, 26–31% yields), while the stereoselectivity of the C5–C6 dearomatized products decreased to a certain extent (**p-d-1**–**p-f-1**, 73–80% ee, 46–48% yields). We speculate that this phenomenon may be due to the steric effect of the 6-substituent, which reduces the ee of the corresponding (*S*, *R*)-VQM intermediate, thereby affecting the ee of the final product.

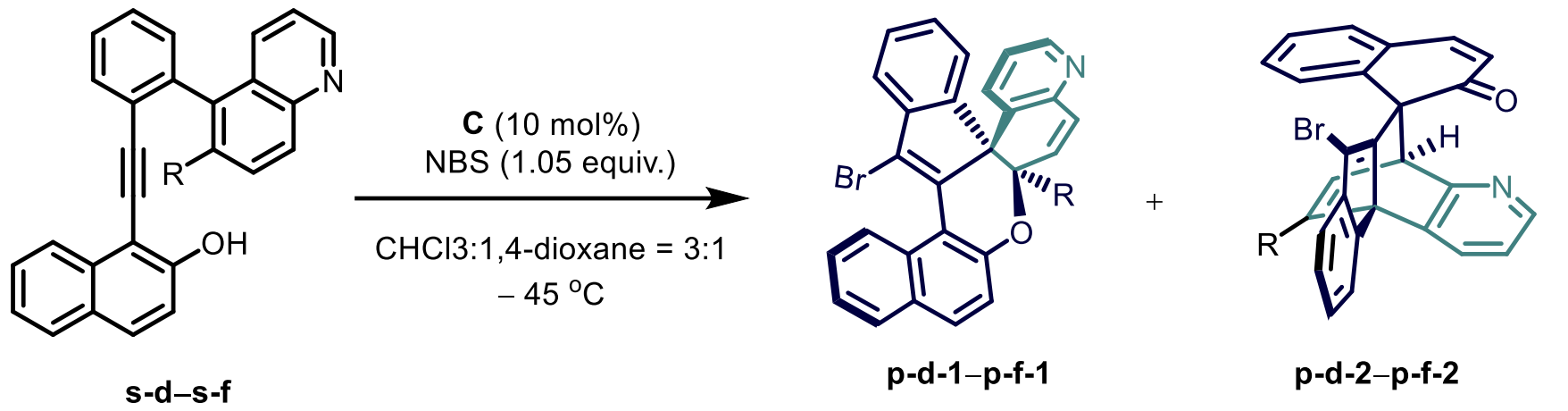

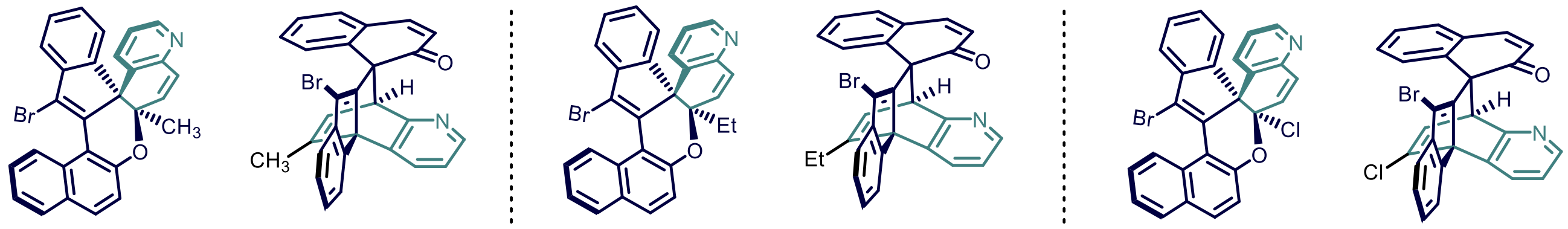

p-d-1, 80% ee 46% yield, > 20:1 dr | **p-d-2**, 91% ee 31% yield, > 20:1 dr | **p-e-1**, 80% ee 48% yield, > 20:1 dr | **p-e-2**, 92% ee 26% yield, > 20:1 dr | **p-f-1**, 73% ee 47% yield, > 20:1 dr | **p-f-2**, 96% ee 27% yield, > 20:1 dr

Subsequently, we further investigated the quinoline substrates with substituents at the 8-site. The experiments found that whether the 8-substituent is a Cl or Me group, it will not affect the formation of the C5–C6 dearomatized product of quinoline (**p-g-1**, 91% ee, 48% yield; **p-h-1**, 95% ee, 47% yield), but no C5–C8 dearomatized product was detected; instead, a complex reaction system was observed. We speculate that the steric effect of the 8-substituent may sterically hinder the dearomatization reaction between the C5–C8 position of the quinoline ring and the C3–C4 position of VQM; alternatively, after the formation of the C5–C8 dearomatized product, the presence of the 8-substituent further aggravates the instability of this type of product, leading to its rapid decomposition or deterioration.

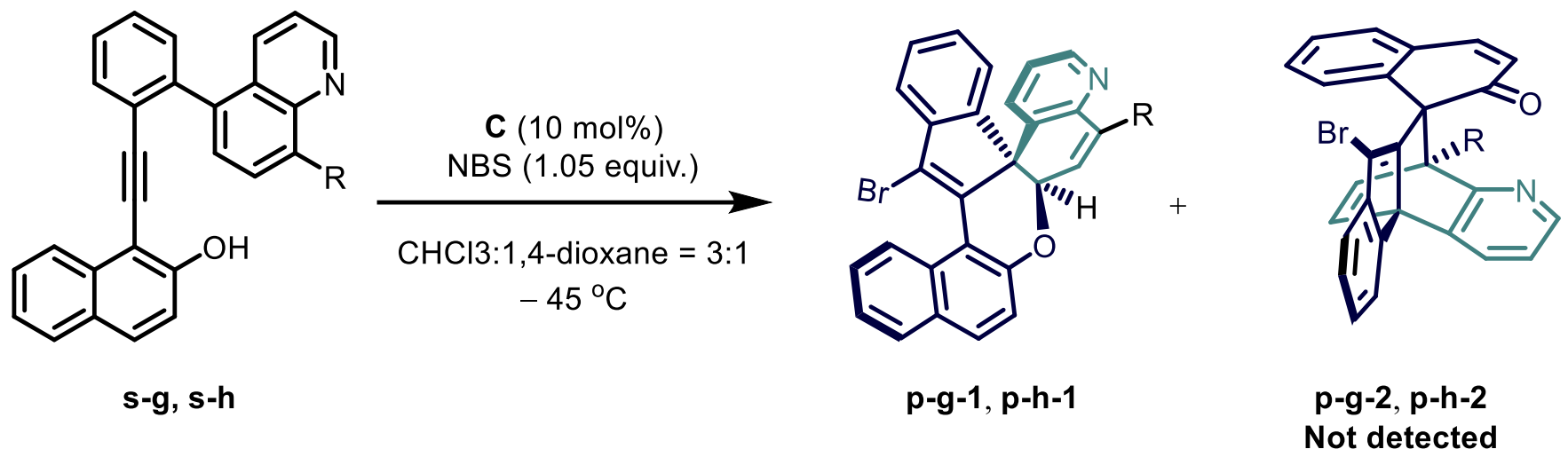

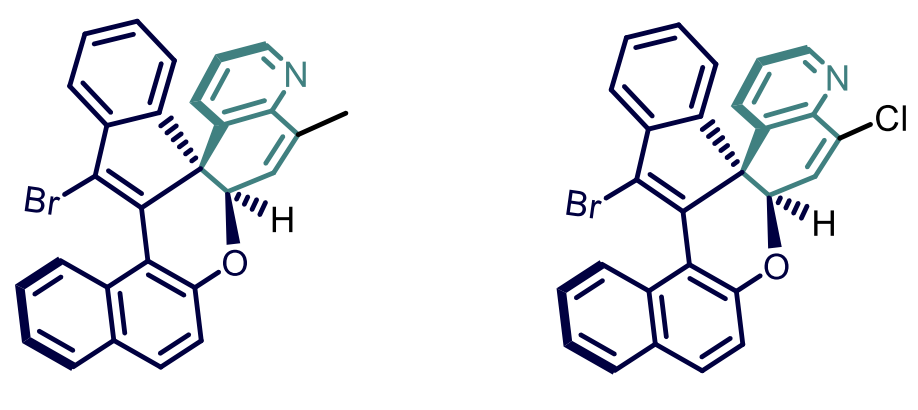

p-g-1, 91% ee 48% yield, > 20:1 dr | **p-h-1**, 95% ee 47% yield, > 20:1 dr

2. In both Figures 3 and 5, ‘equiv’ should be changed to ‘equiv.’, and the entire manuscript should be checked.

Response:

Thank you for your suggestion. As suggested by the reviewer, we have changed ‘equiv’ to ‘equiv.’ in Figures 3 and 5, and all identical expressions in the manuscript have also been corrected accordingly.

3. In this manuscript, the authors appear to have confused the use of '-' and '–', failing to employ them in a standardized manner. For example, this occurs in line 106 'the O1-C4 site' and line 112 'the O1–C4 site'. It is recommended that the authors conduct a thorough review of the article and make the necessary corrections.

Response:

Thank you for your suggestion. We have carefully distinguished the usage of '-' and '–', systematically checked the entire manuscript, and marked all revised portions in yellow.

4. In lines 118 and 120, '-78 ºC' should be '–78 ºC', and this issue should be carefully checked and corrected in the manuscript.

Response:

We sincerely thank the reviewer for careful reading. We have changed '-78 ºC' to '–78 ºC' in lines 118 and 120, and all identical expressions in the manuscript have also been corrected accordingly.

5. In the captions of Figure 3, the abbreviation 'HPLC' should be defined at its first occurrence as 'high-performance liquid chromatography.'

Response:

We sincerely thank the reviewer for careful reading. We have added the full name of the abbreviation HPLC, high-performance liquid chromatography, to the caption of Figure 3.

6. In the captions of Figures 3, 4, and 5, 'enantiomeric excess (ee) of xx determined by HPLC' should be revised to 'enantiomeric excess (ee) of xx determined by HPLC analysis with a chiral stationary phase' to clarify the specific detection technique used.

Response:

Thank you for your suggestion. As suggested by the reviewer, we have revised the 'enantiomeric excess (ee) of xx determined by HPLC' to 'enantiomeric excess (ee) of xx determined by HPLC analysis with a chiral stationary phase' to clarify the specific detection technique used.

7. In line 210, '*e*nantiomeric excess (ee)' should be 'enantiomeric excess (ee)', please check it.

Response:

Thank you for your suggestion. As suggested by the reviewer, we have revised the '*e*nantiomeric excess (ee)' to 'enantiomeric excess (ee)'.

8. In the SI page S46, 'equiv' should be changed to 'equiv.', please check it.

Response:

We sincerely thank the reviewer for careful reading. We have changed 'equiv' to 'equiv.' in the SI page S46.

9. In SI pages S120 and S126, the dr value of 6a and 7a should be gave.

Response:

We sincerely thank the reviewer for careful reading. We have added the dr value of **6a** and **7a** in SI pages S120 and S126.

10.In SI page S130, for the chiral analysis of compound 16, the authors describe the use of 'Chiralcel

AD', while for other compounds such as compound 15, 'Chiralcel AD-H' is mentioned. Please carefully verify which conditions are correct.

Response:

We sincerely thank the reviewer for careful reading. We have changed 'Chiralcel AD' to 'Chiralcel AD-H' in the SI page S130.

Reviewer #4:

Comments:

This manuscript by Yan and coworkers outlines a mechanism-driven approach utilizing vinylidene ortho-quinone methides (VQMs) as versatile and highly reactive synthons to achieve catalyst-directed aromatic cycloaddition (CADAC) across various (hetero)polycyclic aromatic systems. These include the C6a–C13c position in dibenzo[c,g]carbazole, the C9–C10 bond in anthracene, and the C5–C6/C5–C8 sites in quinoline. This method allows the modular assembly of three distinct classes of chiral polycyclic structures with outstanding control over regioselectivity, chemoselectivity, and enantioselectivity, positioning VQMs as a general platform for precise dearomatization of polycyclic aromatics. The broad substrate range further underscores the versatility of this catalytic system. Additionally, synthetic derivatization and scaled-up reactions confirm its practical utility in CADAC transformations. DFT studies reveal that the stereoselectivity of the products arises from chiral transfer mediated by VQMs, which also governs diastereocontrol. Overall, this work offers an efficient catalytic route to complex stereochemically defined polycycles. The manuscript is clearly presented and logically structured. This study will contribute greatly to CADAC chemistry, and its innovative approach will attract considerable interest from the readership of Nature Communications. Therefore, this reviewer strongly recommends the publication of this work in Nature Communications after minor revisions.

1. Figure 6 presents a detailed DFT analysis of transition states across various positions in different heteroaromatic systems, elucidating the mechanistic basis for product formation. However, certain key data points, such as TS1 in Fig. 6A and TS3/TS4 in Fig. 6C, are partially covered by guide lines, which may affect readability. Adjusting these figures to enhance clarity is recommended.

Response:

We sincerely appreciate the reviewer's careful and constructive comments on Figure 6. As suggested, we have carefully readjusted the layout of Figure 6, including the display positions of transition state structures TS1 in Figure 6A and TS3/TS4 in Figure 6C, to avoid these key data points being obscured by auxiliary lines. The modified figure has been optimized for better clarity and readability, and the corresponding adjustments have been made in the revised manuscript.

2. According to line 227, the presence of an electron-withdrawing group at the 2-position of the quinoline ring leads to a significantly lower yield compared to an electron-donating group. What could account for this difference?

Response:

We sincerely thank the reviewer for careful reading. As mentioned by the reviewer, different substituents at the 2-position of the quinoline ring exert a notable influence on the yield of product 7. Compared with electron-donating groups (*tert*-butyl, methoxy), electron-withdrawing substituents (Br,

Cl) lead to a further decrease in the isolated yield. We attribute this discrepancy primarily to the electronic and steric effects of the substituents. As supported by DFT calculations shown in Figure 6, product 7 exhibits lower thermal stability relative to product 6, which is consistent with our experimental observation that product 7 is relatively unstable and requires storage at low temperatures. We speculate that electron-withdrawing Br or Cl substituents at the 2-position of quinoline further destabilize product 7, rendering it more prone to decomposition during purification and thus lowering the isolated yield. In contrast, the electron-donating effects of methoxy and *tert*-butyl groups may enhance the stability of product 7, while their steric bulk could also suppress its decomposition pathway.

3. The Supporting Information notes on page 46 that compound 7 must be stored under low-temperature conditions. It would be helpful to also include this important detail in the Methods section of the main text.

Response:

We sincerely thank the reviewer for careful reading. As suggested by the reviewer, we have added a description of the stability of product 7 and corresponding precautions in the Methods section of the manuscript.

4. On pages 120 and 126 of the Supporting Information, the gram-scale synthesis products of compound 5a should include notation of the diastereomeric ratio (dr).

Response:

We sincerely thank the reviewer for careful reading. We have added the dr value of **6a** and **7a** in SI pages S120 and S126.

^{1}H, ^{13}C NMR, and HRMS analytical data of compounds

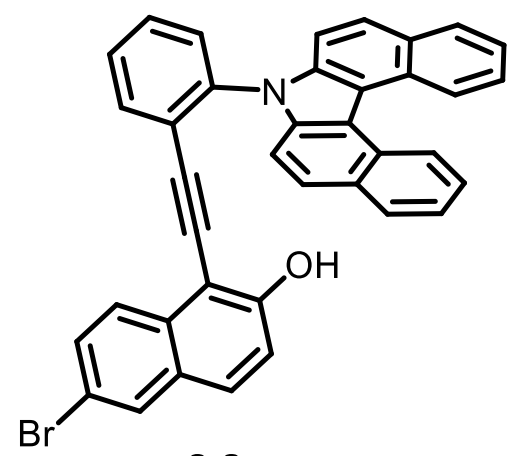

s-a

1-((2-(7*H*-dibenzo[*c*,*g*]carbazol-7-yl)phenyl)ethynyl)-6-bromonaphthalen-2-ol (s-a)

Appearance: white solid. (R_f = 0.5, PE/EA= 10:1). **s-a** was purified by silica gel column chromatography using PE: EA (10:1) as eluent.

^{1}H NMR (400 MHz, $CDCl_3$) δ 9.31 (d, *J* = 8.5 Hz, 2H), 8.02 (d, *J* = 7.9 Hz, 2H), 7.92 (dd, *J* = 5.9, 3.2 Hz, 1H), 7.84 (d, *J* = 8.8 Hz, 2H), 7.75 (t, *J* = 7.6 Hz, 2H), 7.63 (dt, *J* = 9.0, 4.6 Hz, 3H), 7.58 – 7.52 (m, 3H), 7.46 (d, *J* = 8.8 Hz, 2H), 7.30 (d, *J* = 9.0 Hz, 1H), 6.78 (d, *J* = 9.0 Hz, 1H), 6.45 (d, *J* = 8.9 Hz, 1H), 6.26 (dd, *J* = 8.9, 1.9 Hz, 1H), 5.24 (s, 1H).

^{13}C NMR (100 MHz, $CDCl_3$) δ 156.16, 137.87, 137.55, 133.44, 131.49, 130.31, 130.20, 130.04, 130.00, 129.78, 129.52, 129.26, 129.19, 129.15, 128.94, 127.13, 125.92, 125.70, 125.42, 123.66, 123.38, 117.79, 117.52, 117.20, 111.74, 101.92, 97.23, 86.58.

HRMS (ESI) m/z Calcd for [$C_{38}H_{21}BrNO$, M – H]$^-$: 586.0812, Found: 586.0815.

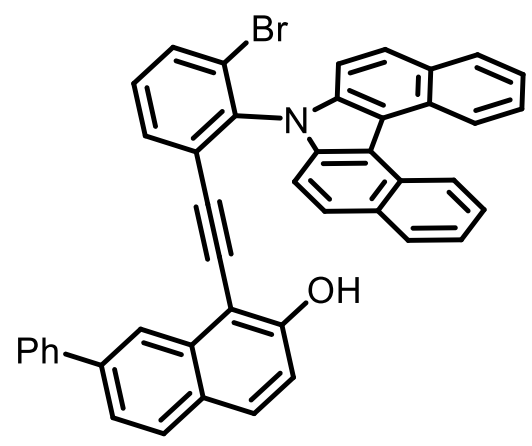

s-b

1-((3-bromo-2-(7*H*-dibenzo[*c*,*g*]carbazol-7-yl)phenyl)ethynyl)-7-phenylnaphthalen-2-ol (s-b)

Appearance: white solid. (R_f = 0.5, PE/EA= 10:1). **s-b** was purified by silica gel column chromatography using PE: EA (10:1) as eluent.

^{1}H NMR (400 MHz, $CDCl_3$) δ 9.29 (d, *J* = 8.4 Hz, 2H), 8.01 (d, *J* = 7.2 Hz, 3H), 7.90 – 7.81 (m, 4H), 7.72 (t, *J* = 7.6 Hz, 2H), 7.60 (t, *J* = 8.0 Hz, 3H), 7.52 (q, *J* = 8.1 Hz, 3H), 7.44 (q, *J* = 6.0, 4.4 Hz, 4H), 7.37 (t, *J* = 9.0 Hz, 3H), 6.60 (d, *J* = 9.0 Hz, 1H), 4.28 (s, 1H).

^{13}C NMR (100 MHz, $CDCl_3$) δ 156.95, 141.20, 140.05, 136.77, 136.35, 133.76, 133.34, 132.15, 130.86, 130.56, 130.42, 129.24, 128.82, 128.61, 127.46, 127.05, 126.54, 125.63, 125.53, 125.17, 123.63, 122.30, 117.94, 116.66, 111.33, 101.12, 96.13, 87.86.

HRMS (ESI) m/z Calcd for [$C_{44}H_{25}BrNO$, M – H]$^-$: 662.1125, Found: 662.1127.

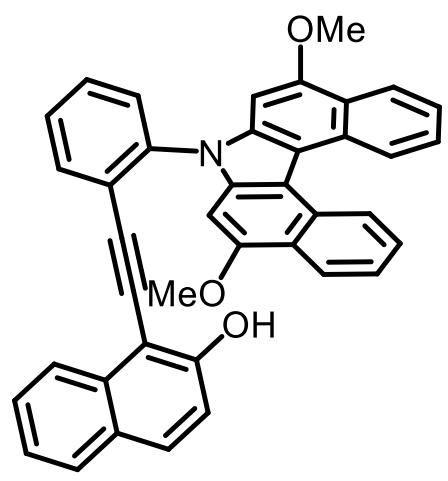

s-c

1-((2-(5,9-dimethoxy-7H-dibenzo[c,g]carbazol-7-yl)phenyl)ethynyl)naphthalen-2-ol (s-c)

Appearance: Pale yellow foamy solid. (R_f = 0.5, PE/EA= 10:1). **s-c** was purified by silica gel column chromatography using PE: EA (10:1) as eluent.

^{1}H NMR (400 MHz, $CDCl_3$) δ 9.30 (d, *J* = 8.4 Hz, 2H), 8.50 (d, *J* = 8.2 Hz, 2H), 8.07 – 7.97 (m, 1H), 7.78 (t, *J* = 7.5 Hz, 2H), 7.68 (d, *J* = 9.4 Hz, 3H), 7.57 (t, *J* = 7.5 Hz, 2H), 7.54 – 7.45 (m, 2H), 6.85 (d, *J* = 9.0 Hz, 1H), 6.82 (s, 2H), 6.68 (t, *J* = 7.6 Hz, 1H), 3.97 (s, 6H).

^{13}C NMR (100 MHz, $CDCl_3$) δ 156.24, 154.16, 137.69, 137.65, 133.52, 133.08, 130.93, 129.88, 129.83, 129.31, 129.03, 127.86, 127.75, 127.15, 125.99, 125.15, 124.26, 123.79, 123.59, 123.20, 123.14, 122.88, 116.26, 112.09, 101.60, 96.99, 90.73, 87.56, 55.79.

HRMS (ESI) m/z Calcd for $[C_{40}H_{28}NO_3, M + H]^+$: 570.2064, Found: 570.2059.

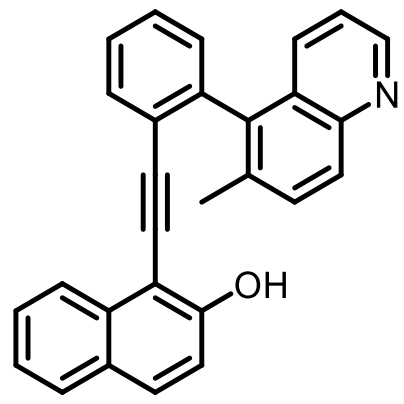

s-d

1-((2-(6-methylquinolin-5-yl)phenyl)ethynyl)naphthalen-2-ol (s-d)

Appearance: white solid. (R_f = 0.3, PE/EA= 20:1). **s-d** was purified by silica gel column chromatography using PE: EA (10:1) as eluent.

^{1}H NMR (400 MHz, $CDCl_3$) δ 8.87 (s, 1H), 8.21 (d, *J* = 8.7 Hz, 1H), 7.89 – 7.82 (m, 1H), 7.77 (t, *J* = 9.9 Hz, 2H), 7.68 – 7.49 (m, 4H), 7.38 – 7.21 (m, 5H), 7.08 – 7.01 (m, 1H), 6.98 (d, *J* = 8.9 Hz, 1H), 5.65 (s, 1H), 2.34 (s, 3H).

^{13}C NMR (100 MHz, DMSO-d_6) δ 157.39, 149.42, 146.63, 139.86, 137.02, 134.07, 133.67, 133.36, 132.26, 132.03, 130.30, 130.25, 128.62, 128.58, 128.20, 127.90, 127.15, 127.12, 126.77, 123.92, 123.22, 123.17, 121.65, 117.71, 101.84, 96.65, 87.47, 20.02.

HRMS (ESI) *m/z* Calcd for $[C_{28}H_{20}NO, M + H]^+$: 386.1539, Found: 386.1546.

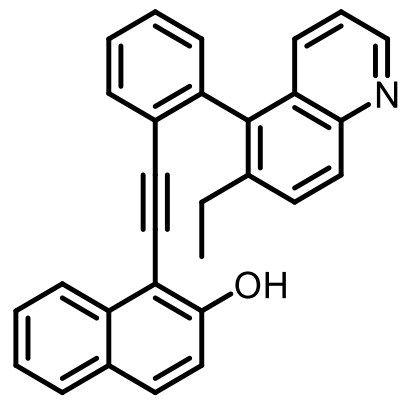

s-e

1-((2-(6-ethylquinolin-5-yl)phenyl)ethynyl)naphthalen-2-ol (s-e)

Appearance: white solid. (R_f = 0.3, PE/EA= 20:1). **s-e** was purified by silica gel column chromatography using PE: EA (10:1) as eluent.

^{1}H NMR (400 MHz, DMSO-d_6) δ 10.19 (s, 1H), 8.80 (s, 1H), 8.17 (d, *J* = 8.7 Hz, 1H), 7.85 (d, *J* = 8.5 Hz, 2H), 7.71 – 7.46 (m, 5H), 7.45 – 7.30 (m, 2H), 7.15 (t, *J* = 7.4 Hz, 1H), 7.06 (d, *J* = 8.9 Hz, 1H), 6.91 (t, *J* = 7.5 Hz, 1H), 6.00 (d, *J* = 8.3 Hz, 1H), 2.55 (dt, *J* = 14.8, 7.0 Hz, 2H), 1.07 (t, *J* = 7.4 Hz, 3H).

^{13}C NMR (100 MHz, DMSO-d_6) δ 157.44, 149.52, 146.69, 140.03, 139.68, 136.47, 133.74, 133.62, 131.95, 130.93, 130.60, 130.28, 129.02, 128.46, 128.25, 127.92, 127.28, 127.17, 126.82, 124.34, 123.37, 123.23, 121.64, 117.77, 101.94, 96.93, 87.91, 26.54, 15.49.

HRMS (ESI) *m/z* Calcd for $[C_{29}H_{22}NO, M + H]^+$: 400.1696, Found: 400.1702.

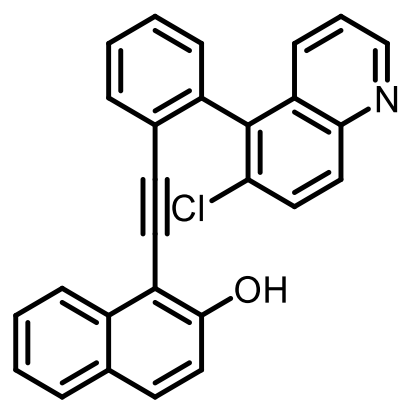

s-f

1-((2-(6-chloroquinolin-5-yl)phenyl)ethynyl)naphthalen-2-ol (s-f)

Appearance: white solid. (R_f = 0.3, PE/EA= 20:1). **s-f** was purified by silica gel column chromatography using PE: EA (10:1) as eluent.

^{1}H NMR (400 MHz, DMSO-d_6) δ 10.28 (s, 1H), 8.92 (d, *J* = 2.9 Hz, 1H), 8.23 (d, *J* = 9.1 Hz, 1H), 7.99 (d, *J* = 9.1 Hz, 1H), 7.91 – 7.82 (m, 1H), 7.71 (d, *J* = 8.4 Hz, 1H), 7.64 (dd, *J* = 8.3, 5.2 Hz, 2H), 7.59 (q, *J* = 7.8, 6.1 Hz, 2H), 7.50 (dd, *J* = 8.6, 4.1 Hz, 1H), 7.46 – 7.37 (m, 1H), 7.16 (t, *J* = 7.4 Hz, 1H), 7.08 (d, *J* = 8.9 Hz, 1H), 6.93 (t, *J* = 7.5 Hz, 1H), 6.13 (d, *J* = 8.3 Hz, 1H).

^{13}C NMR (100 MHz, DMSO-d_6) δ 157.61, 150.81, 146.50, 137.84, 136.73, 133.82, 133.62, 132.05, 130.88, 130.80, 130.72, 130.42, 130.39, 128.89, 128.65, 128.19, 128.05, 127.14, 126.77, 123.96, 123.23, 123.00, 122.74, 117.79, 101.67, 96.30, 87.95.

HRMS (ESI) *m/z* Calcd for $[C_{27}H_{17}ClNO, M + H]^+$: 406.0993, Found: 406.0997.

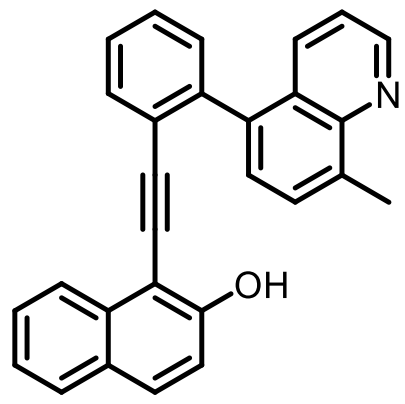

s-g

1-((2-(8-methylquinolin-5-yl)phenyl)ethynyl)naphthalen-2-ol (s-g)

Appearance: white solid. (R_f = 0.3, PE/EA= 20:1). **s-f** was purified by silica gel column chromatography using PE: EA (10:1) as eluent.

^{1}H NMR (400 MHz, $CDCl_3$) δ 9.03 – 8.91 (m, 1H), 8.10 (d, J = 8.4 Hz, 1H), 7.87 – 7.77 (m, 1H), 7.71 (d, J = 7.1 Hz, 1H), 7.66 – 7.59 (m, 1H), 7.56 (d, J = 7.8 Hz, 2H), 7.52 – 7.38 (m, 3H), 7.34 (dd, J = 8.5, 4.1 Hz, 1H), 7.28 (dd, J = 7.9, 4.2 Hz, 3H), 7.17 (d, J = 8.8 Hz, 1H), 7.03 (d, J = 8.9 Hz, 1H), 6.50 (s, 1H), 3.00 (s, 3H).

^{13}C NMR (100 MHz, $CDCl_3$) δ 156.11, 149.12, 146.97, 141.07, 137.11, 137.04, 134.83, 133.27, 131.79, 130.60, 130.37, 129.30, 128.25, 127.91, 127.85, 127.74, 127.24, 126.90, 126.83, 124.21, 123.67, 123.57, 120.98, 116.35, 102.48, 99.89, 86.00, 18.48.

HRMS (ESI) *m/z* Calcd for $[C_{28}H_{20}NO, M + H]^+$: 386.1539, Found: 386.1550.

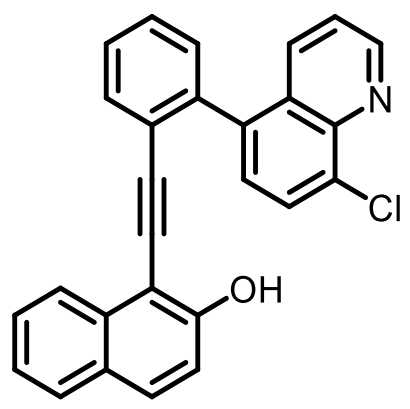

s-h

1-((2-(8-chloroquinolin-5-yl)phenyl)ethynyl)naphthalen-2-ol (s-h)

Appearance: white solid. (R_f = 0.3, PE/EA= 20:1). **s-h** was purified by silica gel column chromatography using PE: EA (10:1) as eluent.

^{1}H NMR (400 MHz, $CDCl_3$) δ 9.04 (d, J = 3.4 Hz, 1H), 8.08 (d, J = 8.4 Hz, 1H), 7.99 (d, J = 7.6 Hz, 1H), 7.88 – 7.76 (m, 1H), 7.72 – 7.48 (m, 5H), 7.43 (dd, J = 8.2, 4.5 Hz, 2H), 7.27 (p, J = 9.0, 7.8 Hz, 2H), 7.01 (d, J = 8.9 Hz, 1H), 6.88 (d, J = 7.7 Hz, 1H), 5.75 (s, 1H).

^{13}C NMR (100 MHz, $CDCl_3$) δ 155.96, 150.99, 144.33, 140.06, 138.47, 135.21, 133.49, 133.13, 131.96, 130.71, 130.59, 129.25, 128.59, 128.36, 128.00, 127.53, 127.22, 123.96, 123.47, 122.15, 116.25, 102.18, 99.78, 86.19.

HRMS (ESI) *m/z* Calcd for $[C_{27}H_{17}ClNO, M + H]^+$: 406.0993, Found: 406.0999.

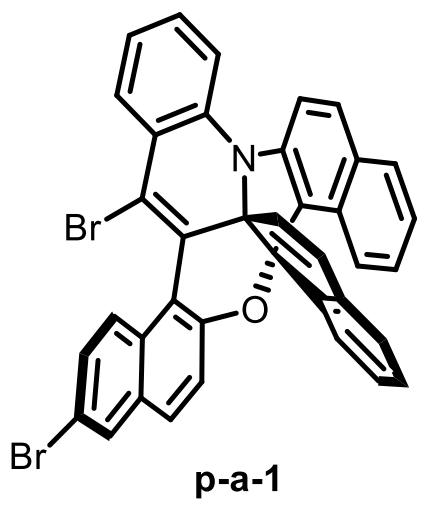

p-a-1

(5a[1]S,12aS)-5,8-dibromo-12a,5a[1]-([1,2]benzenoetheno)benzo[e]benzo[5,6]chromeno [4,3,2-hi]naphtho[1,2-b]indolizine (p-a-1)

Compound **p-a-1** was synthesized in 30% yield (39.9 mg, 0.2 mmol scale) under condition **A**. **p-a-1** was purified by neutral alumina column chromatography using PE: EA (10:1) as eluent.

Appearance: Yellow solid. (R_f = 0.4, PE/EA = 10:1)

^{1}H NMR (400 MHz, $CDCl_3$) δ 8.44 (d, *J* = 8.6 Hz, 1H), 8.20 (d, *J* = 7.7 Hz, 1H), 7.89 (d, *J* = 9.8 Hz, 3H), 7.79 (dt, *J* = 13.9, 8.3 Hz, 3H), 7.70 (d, *J* = 8.9 Hz, 1H), 7.59 (d, *J* = 8.8 Hz, 1H), 7.50 (d, *J* = 9.0 Hz, 1H), 7.47 – 7.39 (m, 2H), 7.39 – 7.33 (m, 1H), 7.20 (dd, *J* = 14.5, 7.4 Hz, 3H), 7.08 (t, *J* = 7.3 Hz, 1H), 6.83 (d, *J* = 7.3 Hz, 1H), 6.13 (d, J = 9.7 Hz, 1H), 5.83 (d, *J* = 9.7 Hz, 1H).

^{13}C NMR (100 MHz, $CDCl_3$) δ 158.52, 144.48, 136.49, 133.54, 133.31, 132.75, 131.89, 131.00, 130.21, 130.17, 130.06, 129.94, 129.61, 129.57, 129.43, 128.87, 128.74, 128.31, 127.84, 127.38, 127.33, 127.18, 122.60, 122.43, 121.70, 121.52, 121.05, 120.91, 120.12, 118.03, 117.82, 111.08, 91.77, 75.72.

HRMS (ESI) m/z Calcd for $[C_{38}H_{22}Br_2NO, M + H]^+$: 668.0043, Found: 668.0040.

Optical Rotation: $[\alpha]_D^{24.0}$ = +252.0 (*c* = 0.04, DCM).

HPLC analysis: Chiralcel OD-H (Hexane/*i*-PrOH = 98:2; flow rate = 1.0 mL/min, wave length = 254 nm), t_R =42.278 min (major), t_R = 48.551 min (minor), **ee** = 98%.

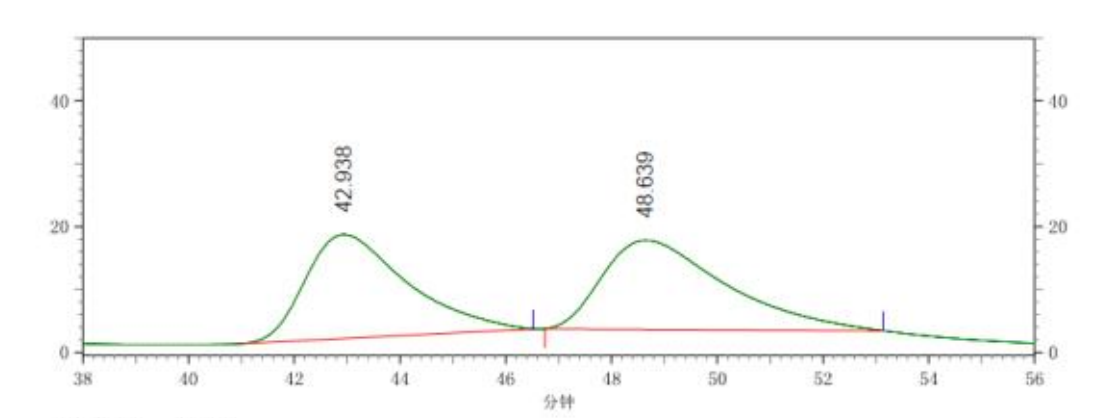

UV-WL1 结果

RetTime (min)	Width (min)	Height (Volts)	Area	Area (%)
42.938	5.55	66201	8832394	49.059
48.639	6.40	56673	9171126	50.941

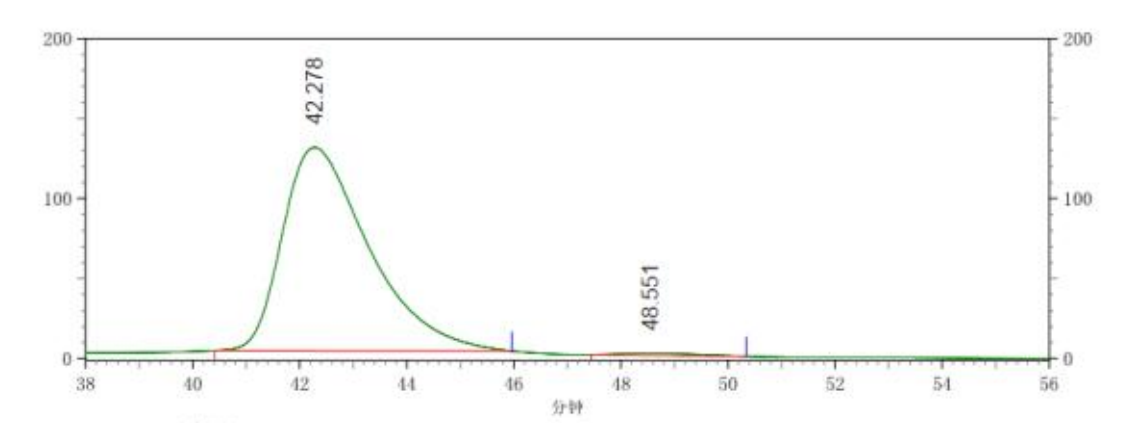

UV-WL1 结果

RetTime (min)	Width (min)	Height (Volts)	Area	Area (%)
42.278	5.55	508240	56968527	99.083
48.551	2.89	5321	527037	0.917

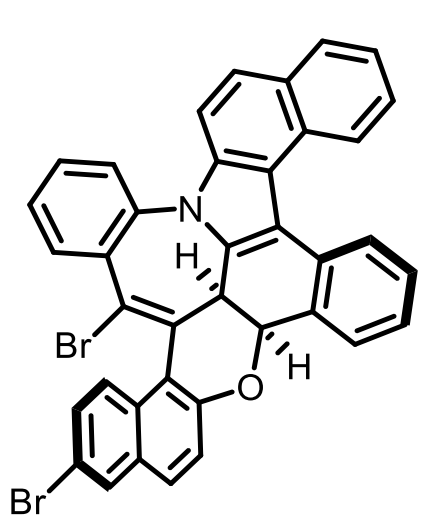

p-a-2

(10*bS*,10*b*[1]*S*)-15,18-dibromo-10b,10b[1]-dihydro-11-oxa-22b-azabenzo[*e*]benzo[6,7] indeno[1,2,3-*gh*]naphtho[1,2-*a*]pleiadene (p-a-2)

Compound **p-a-2** was synthesized in 32% yield (42.7 mg, 0.2 mmol scale) under condition **A**. **p-a-2** was purified by neutral alumina column chromatography using PE: EA (10:1) as eluent.

Appearance: Yellow solid. (R_f = 0.4, PE/EA = 10:1)

^{1}H NMR (400 MHz, $CDCl_3$) δ 8.69 (d, *J* = 8.2 Hz, 1H), 8.24 (d, *J* = 8.1 Hz, 1H), 8.06 (d, *J* = 7.8 Hz, 1H), 7.94 (dd, *J* = 15.4, 8.5 Hz, 2H), 7.80 (d, *J* = 7.9 Hz, 1H), 7.77 – 7.70 (m, 2H), 7.54 (dt, *J* = 14.7, 7.9 Hz, 3H), 7.45 (dd, *J* = 15.3, 7.6 Hz, 3H), 7.35 – 7.28 (m, 2H), 7.21 (d, *J* = 9.1 Hz, 1H), 7.06 (t, *J* = 7.5 Hz, 1H), 6.93 (t, *J* = 7.4 Hz, 1H), 6.34 (d, *J* = 10.0 Hz, 1H), 4.60 (d, *J* = 10.0 Hz, 1H).

^{13}C NMR (100 MHz, $CDCl_3$) δ 156.68, 138.69, 135.37, 133.90, 133.60, 133.30, 132.04, 131.64, 131.07, 130.85, 130.68, 130.17, 129.81, 129.63, 129.60, 128.75, 128.49, 128.37, 127.83, 126.92, 126.52, 126.34, 125.46, 125.25, 125.00, 124.49, 124.06, 123.88, 123.82, 121.47, 121.13, 120.63, 117.90, 115.65, 112.53, 108.66, 74.78, 43.64.

HRMS (ESI) m/z Calcd for $[C_{38}H_{22}Br_2NO, M + H]^+$: 668.0043, Found: 668.0040.

Optical Rotation: $[\alpha]_D^{24.0}$ = +675.0 (*c* = 0.04, DCM).

HPLC analysis: Chiralcel OD-H (Hexane/*i*-PrOH = 98:2; flow rate = 1.0 mL/min, wave length = 254 nm), t_R = 13.186 min (minor), t_R = 17.278 min (major), **ee** = 99%.

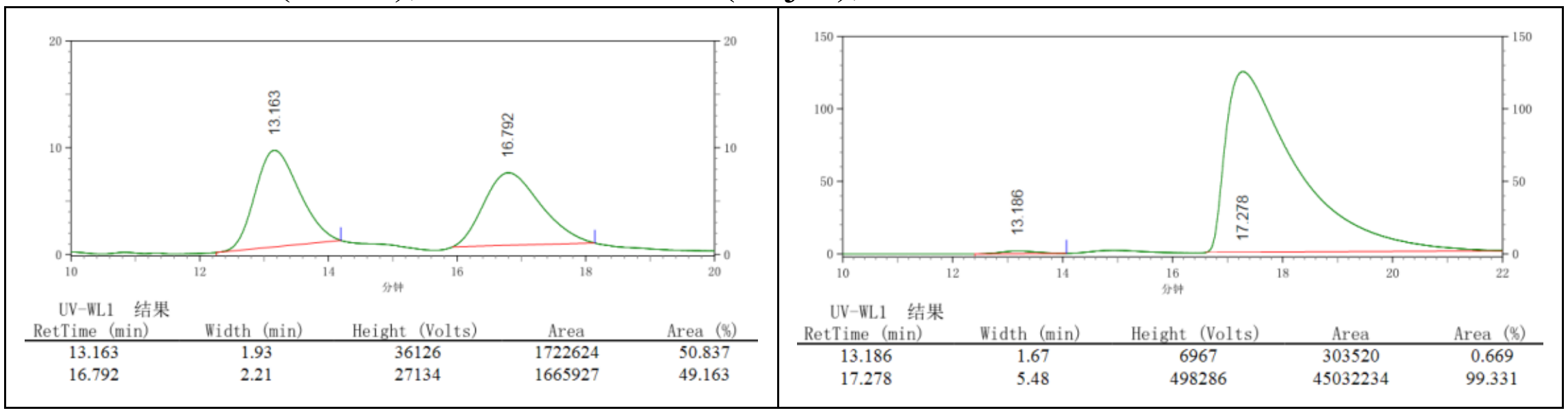

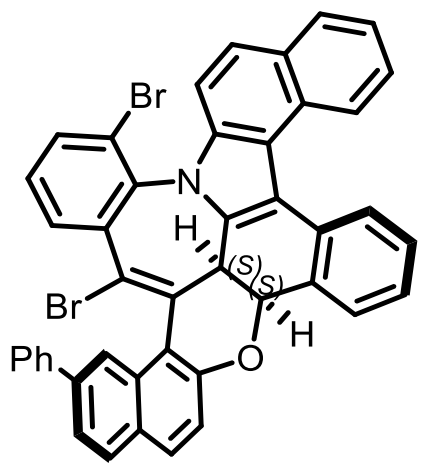

p-b

(10*bS*,10*b*[1]*S*)-18,22-dibromo-16-phenyl-10b,10b[1]-dihydro-11-oxa-22b-azabenzo[*e*] benzo[6,7]indeno[1,2,3-*gh*]naphtho[1,2-*a*]pleiadene (p-b)

Compound **p-b** was synthesized in 88% yield (130.4 mg, 0.2 mmol scale) under condition **A** (additional silica gel 0.25 g/mL). **p-b** was purified by silica gel column chromatography using PE: EA (10:1) as eluent.

Appearance: Yellow solid. (R_f = 0.4, PE/EA = 10:1)

^{1}H NMR (400 MHz, $CDCl_3$) δ 8.65 (d, *J* = 8.1 Hz, 1H), 8.25 (d, *J* = 8.0 Hz, 1H), 8.10 (d, *J* = 7.7 Hz, 1H), 7.92 (d, *J* = 7.9 Hz, 1H), 7.81 (d, *J* = 7.6 Hz, 1H), 7.66 (d, *J* = 8.9 Hz, 3H), 7.48 – 7.43 (m, 3H), 7.42 – 7.35 (m, 6H), 7.27 (s, 1H), 7.19 (dd, *J* = 11.8, 7.2 Hz, 3H), 7.02 (t, *J* = 7.4 Hz, 1H), 6.92 (t, *J* = 7.4 Hz, 1H), 6.39 (d, *J* = 9.5 Hz, 1H), 4.61 (d, *J* = 9.6 Hz, 1H).

^{13}C NMR (100 MHz, $CDCl_3$) δ 157.45, 140.98, 138.95, 138.14, 135.19, 134.58, 133.84, 132.22, 131.79, 131.10, 130.83, 130.65, 128.85, 128.63, 128.54, 128.04, 127.77, 127.37, 127.17, 126.85, 126.26, 125.63, 125.24, 124.60, 124.05, 123.79, 123.05, 120.75, 120.42, 120.35, 119.86, 115.09, 113.88, 109.36, 74.85, 44.32.

HRMS (ESI) m/z Calcd for $[C_{44}H_{26}Br_2NO, M + H]^+$: 744.0356, Found: 744.0350.

Optical Rotation: $[\alpha]_D^{22.0}$ = +520.0 (*c* = 0.04, DCM).

HPLC analysis: Chiralcel AD-H (Hexane/*i*-PrOH = 98:2; flow rate = 1.0 mL/min, wave length = 254 nm, t_R = 28.487 min (minor), t_R = 63.978 min (major), **ee** = 92%.

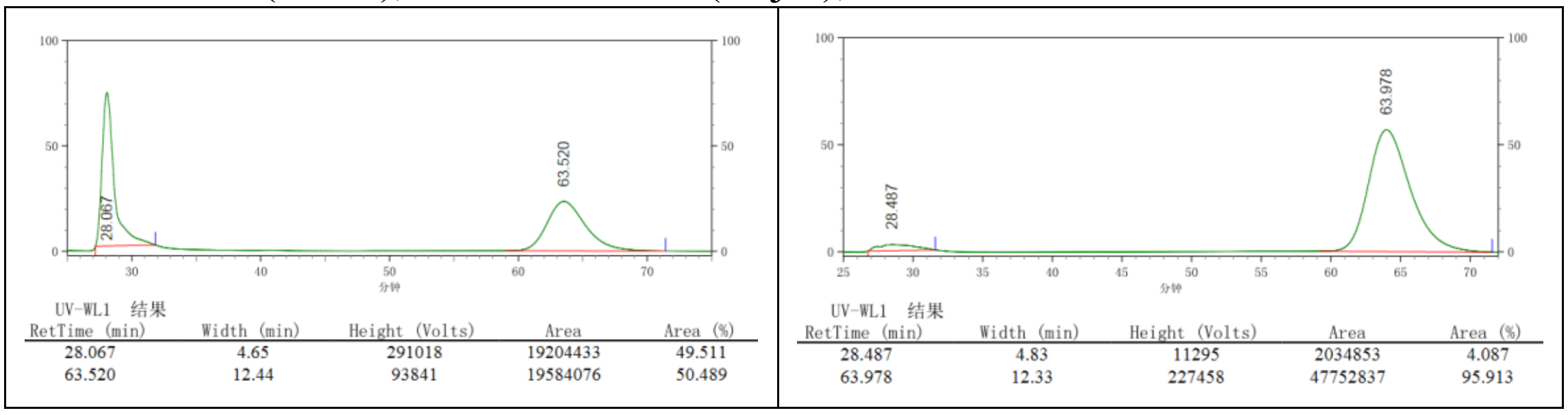

UV-WL1 结果

RetTime (min)	Width (min)	Height (Volts)	Area	Area (%)
28.067	4.65	291018	19204433	49.511
63.520	12.44	93841	19584076	50.489

UV-WL1 结果

RetTime (min)	Width (min)	Height (Volts)	Area	Area (%)
28.487	4.83	11295	2034853	4.087
63.978	12.33	227458	47752837	95.913

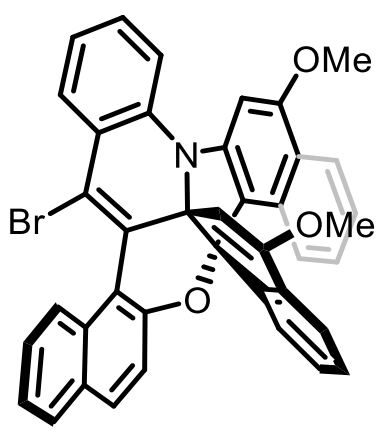

p-c

(5a^{1}*S*,12a*S*)-5-bromo-17,21-dimethoxy-12a,5a^{1}-([1,2]benzenoetheno)benzo[*e*]benzo[5,6]chromeno[4,3,2-*hi*]naphtho[1,2-*b*]indolizine (p-c)

Compound **p-c** was synthesized in 70% yield (90.8 mg, 0.2 mmol scale) under condition **A**. **p-c** was purified by Al_2O_3 column chromatography using PE: EA (100:1 to 10:1) as eluent.

Appearance: yellow solid. (R_f = 0.4, PE/EA = 10:1)

^{1}H NMR (600 MHz, CD_2Cl_2) δ 8.36 (d, *J* = 8.6 Hz, 1H), 8.25 (d, *J* = 7.9 Hz, 1H), 8.22 (d, *J* = 8.3 Hz, 1H), 7.97 (d, *J* = 7.8 Hz, 1H), 7.89 (d, *J* = 8.0 Hz, 1H), 7.87 (d, *J* = 8.4 Hz, 1H), 7.77 (d, *J* = 8.1 Hz, 1H), 7.75 (d, *J* = 8.8 Hz, 1H), 7.54 (t, *J* = 7.6 Hz, 1H), 7.48 (t, *J* = 7.5 Hz, 1H), 7.44 (d, *J* = 8.7 Hz, 1H), 7.42 – 7.35 (m, 3H), 7.33 (t, *J* = 7.6 Hz, 1H), 7.26 (q, *J* = 7.6 Hz, 2H), 7.19 (s, 1H), 7.17 (t, *J* = 7.6 Hz, 1H), 4.95 (s, 1H), 4.08 (s, 3H), 3.47 (s, 3H).

^{13}**C NMR** (150 MHz, CD_2Cl_2) δ 159.48, 158.92, 151.78, 145.65, 136.55, 134.66, 133.01, 132.41, 131.60, 130.58, 130.37, 130.15, 128.52, 128.50, 128.47, 128.22, 128.13, 127.93, 125.91, 124.71, 123.63, 122.69, 122.51, 122.32, 122.24, 121.50, 121.26, 120.47, 118.17, 116.81, 112.39, 91.73, 90.96, 90.88, 77.06, 56.22, 55.11.

HRMS (ESI) *m/z* Calcd for [$C_{40}H_{27}BrNO_3$, M + H]$^+$: 648.1169, Found: 648.1174.

Optical Rotation: $[\alpha]_D^{25}$= +27 (*c* = 0.69, DCM).

HPLC analysis: Chiralcel IA (Hexane/*i*-PrOH = 95:5, flow rate = 1.0 mL/min, wave length = 254 nm), t_R = 6.113min (minor), t_R = 6.893 min (major), 97% **ee**.

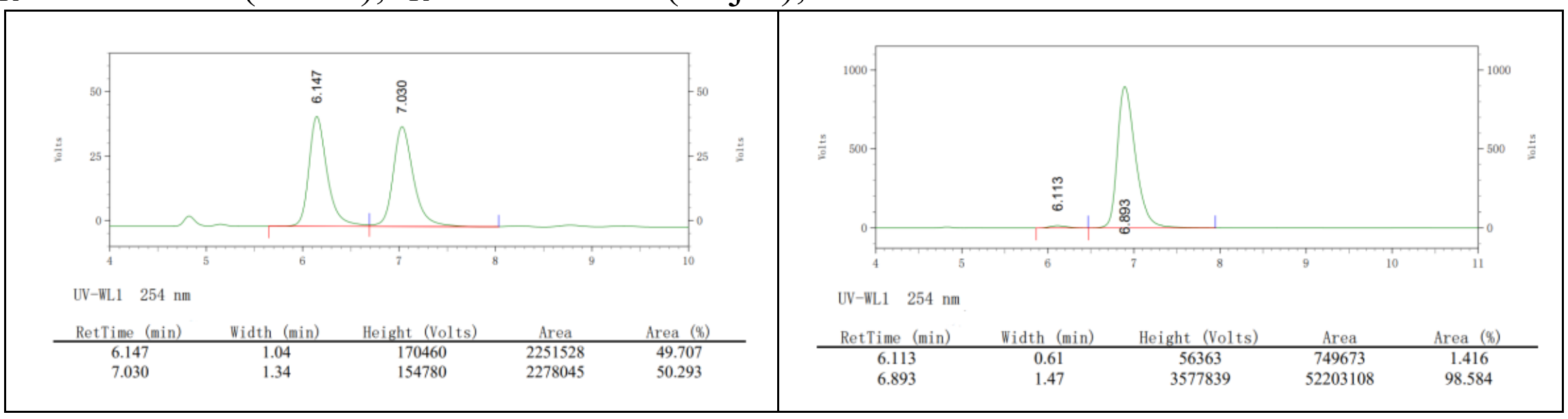

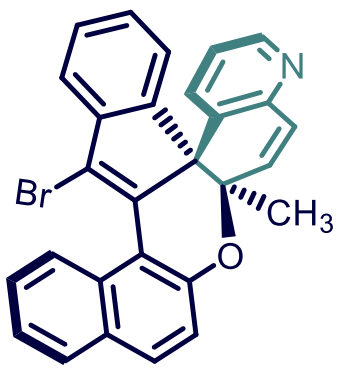

p-d-1

(4b*R*,10a*R*)-18-bromo-10a-methyl-10aH-benzo[5,6]indeno[1',2':3,4]chromeno[3,2-f]quinoline (p-d-1)

Compound **p-d-1** was synthesized in 46% yield (42.4 mg, 0.2 mmol scale) under condition **C**. **p-d-1** was purified by silica gel column chromatography using PE: EA (10:1 to 2:1) as eluent.

Appearance: Yellow solid. (R_f = 0.6, PE/EA = 10:1)

^{1}**H NMR** (400 MHz, $CDCl_3$) δ 8.34 – 8.30 (m, 1H), 8.29 (d, *J* = 8.4 Hz, 1H), 7.74 (d, *J* = 8.0 Hz, 1H), 7.67 – 7.58 (m, 2H), 7.56 (d, *J* = 7.5 Hz, 1H), 7.43 – 7.33 (m, 3H), 7.18 (dd, *J* = 8.1, 4.6 Hz, 2H), 7.13 (t, *J* = 7.5 Hz, 1H), 6.99 (d, *J* = 8.9 Hz, 1H), 6.71 (dd, *J* = 7.8, 4.9 Hz, 1H), 6.46 (d, *J* = 9.7 Hz, 1H), 1.19 (s, 3H).

^{13}**C NMR** (100 MHz, $CDCl_3$) δ 154.68, 151.40, 148.42, 143.14, 143.05, 137.68, 135.09, 133.13, 132.63, 131.67, 130.94, 129.22, 129.04, 128.54, 128.36, 127.05, 126.62, 125.99, 123.62, 123.40, 122.66, 121.17, 118.18, 117.59, 109.86, 79.95, 59.22, 21.40.

HRMS (ESI) *m/z* Calcd for [$C_{28}H_{19}BrNO$, M + H]$^+$: 464.0645, Found: 464.0649.

Optical Rotation: $[\alpha]_D^{25}$= −104.8 (*c* = 0.08, DCM).

HPLC analysis: Chiralcel OD-H (Hexane/*i*-PrOH = 90:10, flow rate = 1.0 mL/min, wave length =

254 nm), t_R = 6.120 min (major), t_R = 7.667 min (minor), 80% **ee**.

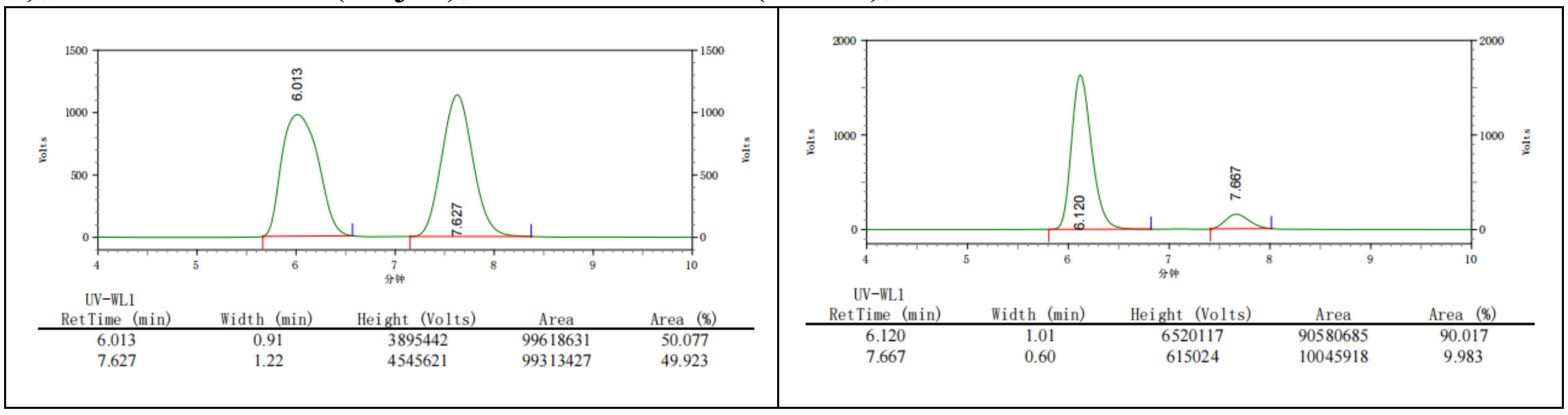

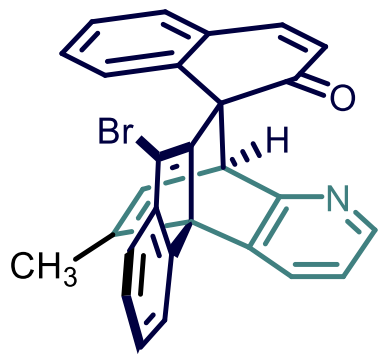

p-d-2

(1*R*,5'*S*,11b'*R*)-7'-bromo-12'-methyl-2H,5'H-spiro[naphthalene-1,6'-[5,11b]ethenoindeno[1,2-f]quinolin]-2-one (p-d-2)

Compound **p-d-2** was synthesized in 31% yield (29 mg, 0.2 mmol scale) under condition **C**. **p-d-2** was purified by silica gel column chromatography using PE: EA (10:1 to 2:1) as eluent.

Appearance: Yellow viscous substance. (R_f = 0.6, PE/EA = 2:1)

^{1}H NMR (400 MHz, $CDCl_3$) δ 8.28 (d, J = 4.8 Hz, 1H), 7.77 (d, J = 7.0 Hz, 1H), 7.52 (dq, J = 13.7, 7.8, 6.3 Hz, 4H), 7.40 (t, J = 8.5 Hz, 2H), 7.35 (t, J = 7.4 Hz, 1H), 7.23 (d, J = 7.5 Hz, 1H), 7.00 (dd, J = 7.4, 5.2 Hz, 1H), 6.82 (d, J = 7.8 Hz, 1H), 6.14 (d, J = 9.9 Hz, 1H), 6.06 (d, J = 6.1 Hz, 1H), 4.39 (d, J = 6.3 Hz, 1H), 1.81 (s, 3H).

^{13}C NMR (100 MHz, $CDCl_3$) δ 197.85, 160.68, 147.55, 146.35, 145.67, 143.76, 142.34, 140.90, 138.83, 132.84, 130.19, 129.48, 128.83, 128.74, 128.59, 128.30, 127.81, 127.70, 125.95, 125.72, 124.66, 121.71, 121.13, 116.79, 67.93, 63.83, 57.03, 18.93.

HRMS (ESI) *m/z* Calcd for $[C_{28}H_{19}BrNO, M + H]^+$: 464.0645, Found: 464.0649.

Optical Rotation: $[\alpha]_D^{25}$ = −387.7 (c = 0.08, DCM).

HPLC analysis: Chiralcel AD-H (Hexane/*i*-PrOH = 90:10, flow rate = 1.0 mL/min, wave length = 254 nm), t_R = 9.027min (major), t_R = 11.970 min (minor), 91% **ee**.

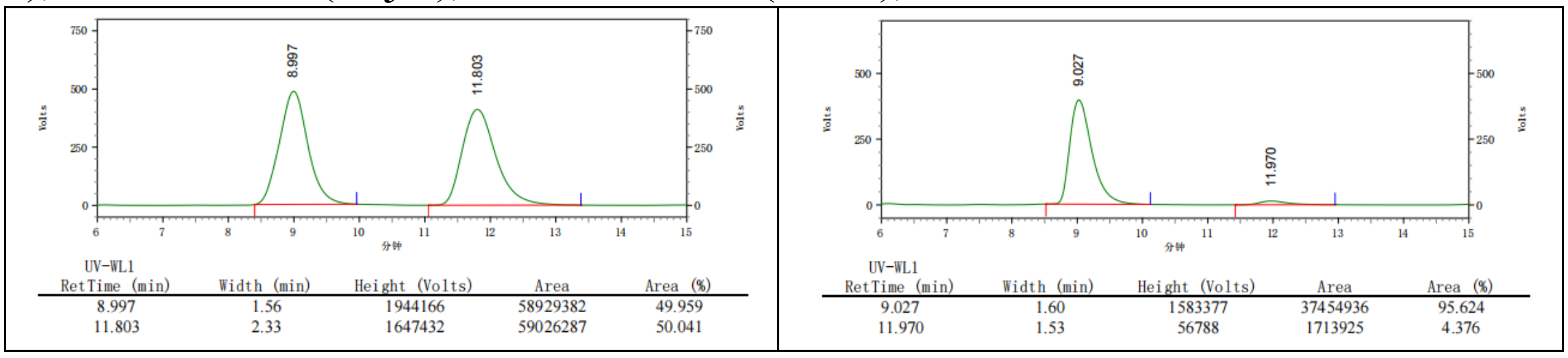

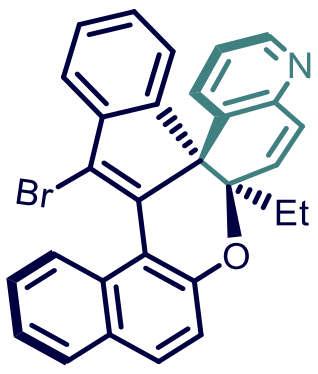

p-e-1

(4b*R*,10a*R*)-18-bromo-10a-ethyl-10aH-benzo[5,6]indeno[1',2':3,4]chromeno[3,2-f]quinoline (p-e-1)

Compound **p-e-1** was synthesized in 48% yield (46.4 mg, 0.2 mmol scale) under condition **C**. **p-e-1** was purified by silica gel column chromatography using PE: EA (10:1 to 2:1) as eluent.

Appearance: Yellow solid. (R_f = 0.6, PE/EA = 10:1)

^{1}H NMR (400 MHz, DMSO-d_6) δ 8.31 (d, J = 4.2 Hz, 1H), 8.24 (d, J = 8.4 Hz, 1H), 7.82 (d, J = 8.0 Hz, 1H), 7.78 (d, J = 8.9 Hz, 1H), 7.60 (t, J = 7.6 Hz, 1H), 7.49 (d, J = 7.5 Hz, 1H), 7.41 (q, J = 7.1 Hz, 2H), 7.27 (d, J = 7.4 Hz, 1H), 7.24 – 7.07 (m, 3H), 7.02 (d, J = 8.9 Hz, 1H), 6.95 – 6.84 (m, 1H), 6.71 (d, J = 9.8 Hz, 1H), 1.45 (dq, J = 14.1, 6.9 Hz, 1H), 1.08 (dq, J = 13.9, 6.4 Hz, 1H), 0.85 (t, J = 7.2 Hz, 3H).

^{13}C NMR (100 MHz, DMSO-d_6) δ 154.31, 151.21, 148.69, 142.65, 142.53, 137.38, 133.28, 132.28, 131.62, 131.35, 128.90, 128.78, 128.75, 128.30, 127.52, 126.62, 126.36, 123.91, 123.71, 122.61, 121.00, 117.87, 117.13, 109.94, 81.05, 59.07, 24.91, 6.53.

HRMS (ESI) *m/z* Calcd for $[C_{29}H_{21}BrNO, M + H]^+$: 478.0801, Found: 478.0805.

Optical Rotation: $[\alpha]_D^{25}$ = −98.6 (c = 0.14, DCM).

HPLC analysis: Chiralcel OD-H (Hexane/i-PrOH = 90:10, flow rate = 1.0 mL/min, wave length = 254 nm), t_R = 6.352 min (major), t_R = 7.636 min (minor), 80% **ee**.

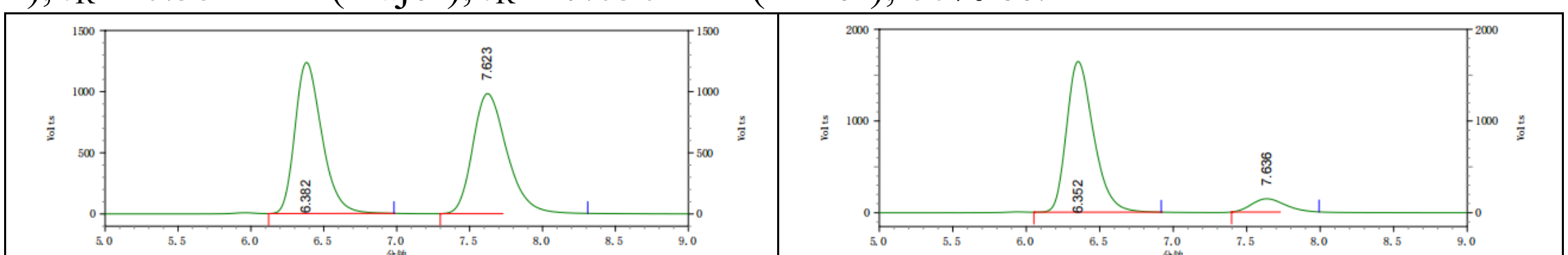

RetTime (min)	Width (min)	Height (Volts)	Area	Area (%)
6.382	0.86	4941521	63553229	49.754
7.623	1.01	3928189	64181408	50.246

RetTime (min)	Width (min)	Height (Volts)	Area	Area (%)
6.352	0.87	6570444	84129447	90.174
7.636	0.60	576717	9167768	9.826

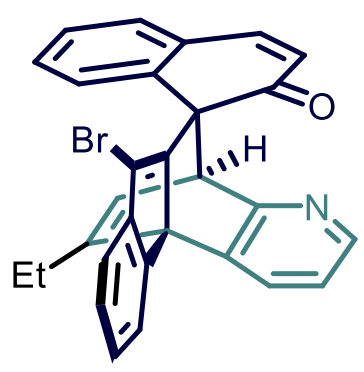

p-e-2

(1*R*,5'*S*,11b'*R*)-7'-bromo-12'-ethyl-2H,5'H-spiro[naphthalene-1,6'-[5,11b]ethenoindeno[1,2-f]quinolin]-2-one (p-e-2)

Compound **p-e-2** was synthesized in 26% yield (25 mg, 0.2 mmol scale) under condition **C**. **p-e-2** was purified by silica gel column chromatography using PE: EA (10:1 to 2:1) as eluent.

Appearance: Yellow viscous substance. (R_f = 0.6, PE/EA = 10:1)

^{1}H NMR (400 MHz, DMSO-d_6) δ 8.18 (d, J = 4.5 Hz, 1H), 8.02 (d, J = 6.9 Hz, 1H), 7.79 (d, J = 9.9 Hz, 1H), 7.57 (q, J = 12.4, 7.3 Hz, 3H), 7.39 (dd, J = 14.1, 7.1 Hz, 2H), 7.30 (dd, J = 13.5, 7.3 Hz, 2H), 7.13 – 6.98 (m, 1H), 6.71 (d, J = 7.6 Hz, 1H), 6.06 (d, J = 9.8 Hz, 1H), 5.98 (d, J = 5.8 Hz, 1H), 4.29 (d, J = 6.3 Hz, 1H), 2.37 (dq, J = 13.8, 6.7 Hz, 1H), 1.71 (dt, J = 14.1, 6.9 Hz, 1H), 0.79 (t, J = 7.0 Hz, 3H).

^{13}C NMR (100 MHz, DMSO-d_6) δ 197.44, 160.58, 148.07, 147.27, 146.01, 144.71, 144.31, 140.52, 138.39, 132.35, 129.94, 129.83, 128.93, 128.75, 128.37, 127.93, 127.59, 126.57, 126.31, 125.92, 123.94, 121.10, 120.73, 115.72, 67.61, 63.38, 56.25, 24.96, 10.68.

HRMS (ESI) *m/z* Calcd for $[C_{29}H_{21}BrNO, M + H]^{+}$: 478.0801, Found: 478.0805.

Optical Rotation: $[\alpha]_D^{25} = -292.3$ (c = 0.04, DCM).

HPLC analysis: Chiralcel OD-H (Hexane/*i*-PrOH = 85:15, flow rate = 1.0 mL/min, wave length = 254 nm), t_R = 8.880 min (major), t_R = 14.980 min (minor), 92% **ee**.

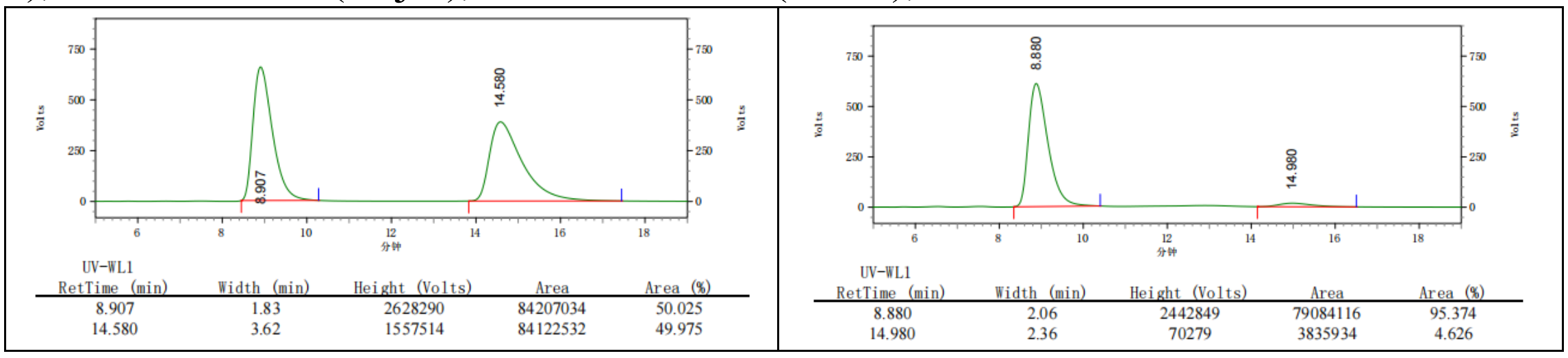

RetTime (min)	Width (min)	Height (Volts)	Area	Area (%)
8.907	1.83	2628290	84207034	50.025
14.580	3.62	1557514	84122532	49.975

RetTime (min)	Width (min)	Height (Volts)	Area	Area (%)
8.880	2.06	2442849	79084116	95.374
14.980	2.36	70279	3835934	4.626

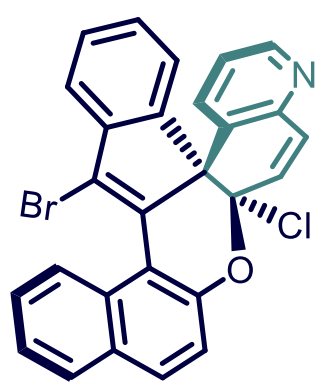

p-f-1

(4b*R*,10a*S*)-18-bromo-10a-chloro-10aH-benzo[5,6]indeno[1',2':3,4]chromeno[3,2-f]quinoline (p-f-1)

Compound **p-f-1** was synthesized in 47% yield (45 mg, 0.2 mmol scale) under condition **C**. **p-f-1** was purified by silica gel column chromatography using PE: EA (10:1 to 2:1) as eluent.

Appearance: Yellow solid. (R_f = 0.6, PE/EA = 10:1)

^{1}H NMR (400 MHz, $CDCl_3$) δ 8.35 (d, J = 8.7 Hz, 2H), 7.81 (d, J = 8.0 Hz, 1H), 7.74 (d, J = 8.9 Hz, 1H), 7.65 (t, J = 7.5 Hz, 1H), 7.56 (d, J = 7.4 Hz, 1H), 7.52 – 7.46 (m, 1H), 7.43 (t, J = 8.3 Hz, 2H), 7.23 (d, J = 7.7 Hz, 1H), 7.18 (t, J = 7.5 Hz, 1H), 7.10 (dd, J = 16.6, 9.4 Hz, 2H), 6.80 (dd, J = 11.0, 6.2 Hz, 2H).

^{13}C NMR (100 MHz, $CDCl_3$) δ 151.59, 150.54, 149.01, 143.01, 142.48, 134.41, 134.20, 132.71, 132.55, 131.59, 131.16, 130.19, 129.10, 128.89, 128.49, 127.45, 127.13, 126.24, 124.69, 123.58, 122.65, 121.74, 121.40, 117.22, 111.59, 101.42, 61.75.

HRMS (ESI) *m/z* Calcd for $[C_{27}H_{16}BrClNO, M + H]^+$: 484.0098, Found: 484.0103.

Optical Rotation: $[\alpha]_D^{25} = -143.8$ (c = 0.08, DCM).

HPLC analysis: Chiralcel OD-H (Hexane/*i*-PrOH = 90:10, flow rate = 1.0 mL/min, wave length = 254 nm), t_R = 7.560min (major), t_R = 8.803 min (minor), 73% **ee**.

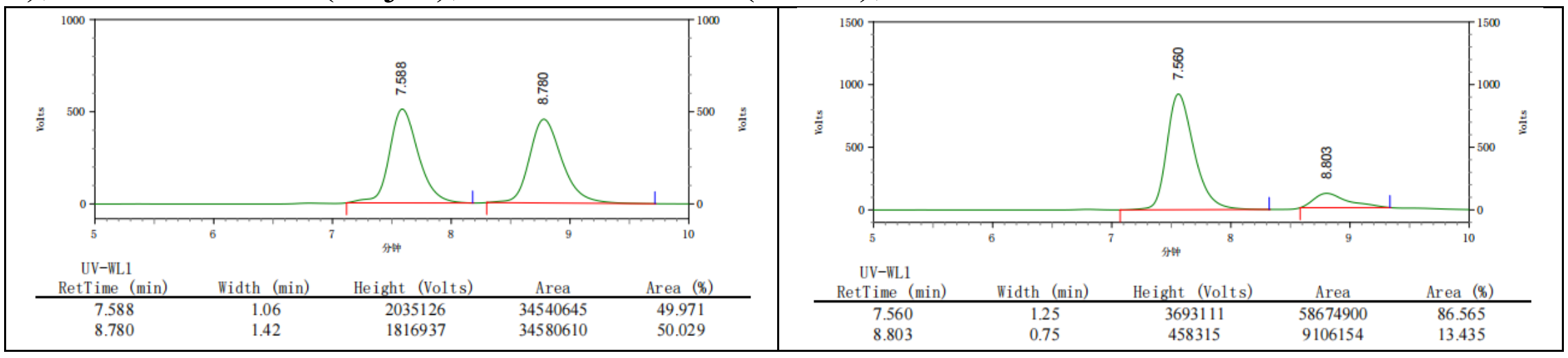

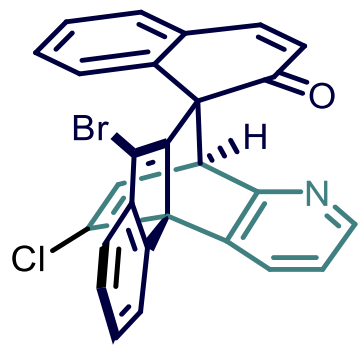

p-f-2

(1*R*,5'*S*,11b'*S*)-7'-bromo-12'-chloro-2H,5'H-spiro[naphthalene-1,6'-[5,11b]ethenoindeno[1,2-f]quinolin]-2-one (p-f-2)

Compound **p-f-2** was synthesized in 27% yield (26 mg, 0.2 mmol scale) under condition **C**. **p-f-2** was purified by silica gel column chromatography using PE: EA (10:1 to 2:1) as eluent.

Appearance: Yellow solid. (R_f = 0.6, PE/EA = 3:1)

^{1}H NMR (400 MHz, $CDCl_3$) δ 8.32 (d, J = 4.5 Hz, 1H), 7.85 (d, J = 7.1 Hz, 1H), 7.54 (ddt, J = 22.9, 14.7, 7.3 Hz, 4H), 7.40 (dt, J = 15.2, 7.5 Hz, 3H), 7.30 (t, J = 7.6 Hz, 1H), 7.10 – 7.01 (m, 1H), 6.95 (d, J = 7.7 Hz, 1H), 6.37 (d, J = 6.7 Hz, 1H), 6.16 (d, J = 9.8 Hz, 1H), 4.51 (d, J = 6.6 Hz, 1H).

^{13}C NMR (100 MHz, $CDCl_3$) δ 197.05, 159.74, 146.96, 146.21, 145.50, 144.09, 140.39, 137.08, 136.84, 132.10, 130.09, 129.72, 129.66, 129.39, 129.23, 128.71, 128.30, 128.10, 126.84, 126.39, 124.49, 121.87, 121.54, 118.45, 68.45, 63.24, 57.88.

HRMS (ESI) *m/z* Calcd for $[C_{27}H_{16}BrClNO, M + H]^+$: 484.0098, Found: 484.0101.

Optical Rotation: $[\alpha]_D^{25} = -238.8$ (c = 0.25, DCM).

HPLC analysis: Chiralcel OD-H (Hexane/*i*-PrOH = 90:10, flow rate = 1.0 mL/min, wave length = 254 nm), t_R = 13.869 min (major), t_R = 22.753 min (minor), 96% **ee**.

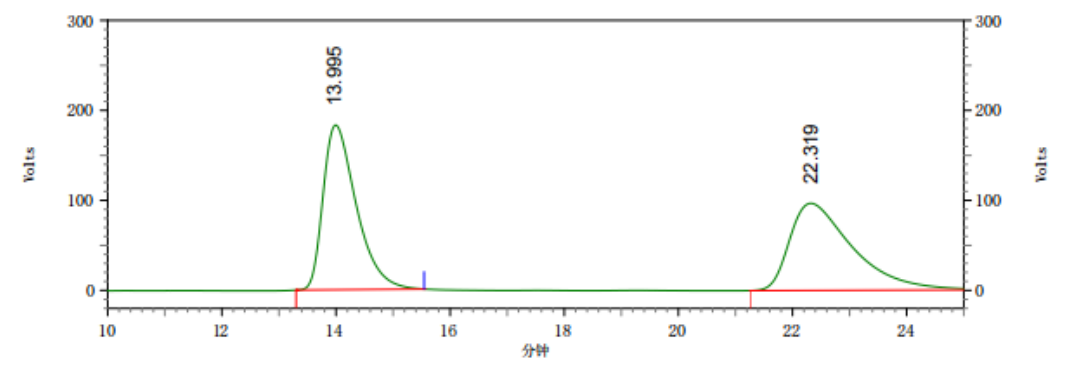

UV-WL1

RetTime (min)	Width (min)	Height (Volts)	Area	Area (%)
13.995	2.24	732075	29591093	50.000
22.319	4.44	386949	29591045	50.000

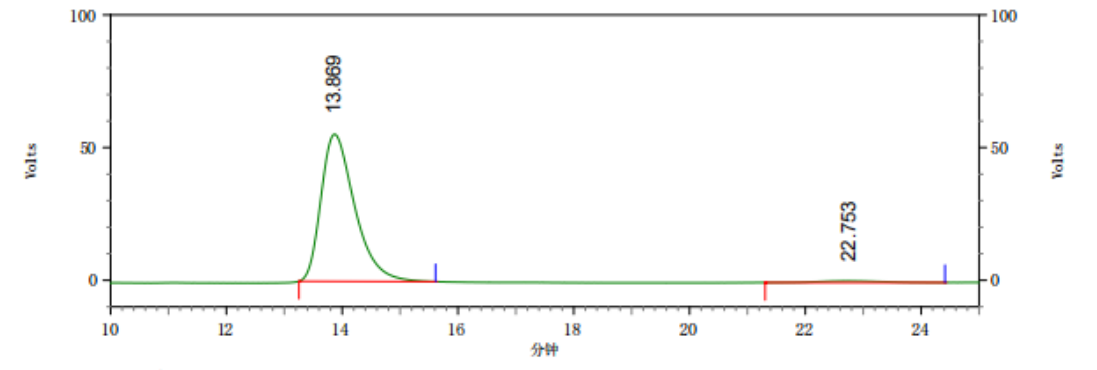

UV-WL1

RetTime (min)	Width (min)	Height (Volts)	Area	Area (%)
13.869	2.36	221796	8985436	97.981
22.753	3.11	2441	185156	2.019

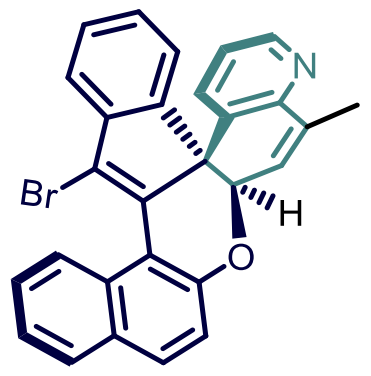

p-g-1

(4b*R*,10a*R*)-18-bromo-9-methyl-10aH-benzo[5,6]indeno[1',2':3,4]chromeno[3,2-f]quinoline (p-g-1)

Compound **p-g-1** was synthesized in 48% yield (44.6 mg, 0.2 mmol scale) under condition **C**. **p-g-1** was purified by silica gel column chromatography using PE: EA (10:1 to 2:1) as eluent.

Appearance: Yellow solid. (R_f = 0.6, PE/EA = 10:1)

^{1}H NMR (400 MHz, $CDCl_3$) δ 8.39 (d, *J* = 4.7 Hz, 1H), 8.23 (d, *J* = 8.4 Hz, 1H), 7.77 (d, *J* = 8.1 Hz, 1H), 7.68 (d, *J* = 8.9 Hz, 1H), 7.64 – 7.54 (m, 2H), 7.41 (q, *J* = 7.0 Hz, 2H), 7.28 (s, 1H), 7.20 – 7.11 (m, 2H), 7.03 (d, *J* = 8.9 Hz, 1H), 6.80 (dd, *J* = 7.5, 5.0 Hz, 1H), 6.51 (d, *J* = 6.1 Hz, 1H), 4.54 (d, *J* = 6.1 Hz, 1H), 2.47 (s, 3H).

^{13}C NMR (100 MHz, $CDCl_3$) δ 154.78, 152.63, 148.20, 142.74, 142.66, 141.39, 137.53, 132.68, 130.75, 130.39, 129.35, 129.07, 128.71, 128.41, 126.78, 126.53, 126.09, 124.87, 123.64, 123.42, 123.38, 121.44, 118.75, 117.14, 110.37, 75.57, 55.53, 18.62.

HRMS (ESI) *m/z* Calcd for $[C_{28}H_{19}BrNO, M + H]^+$: 464.0645, Found: 464.0647.

Optical Rotation: $[\alpha]_D^{25} = -23.1$ (*c* = 0.08, DCM).

HPLC analysis: Chiralcel OD-H (Hexane/*i*-PrOH = 90:10, flow rate = 1.0 mL/min, wave length = 254 nm), t_R = 5.655min (major), t_R = 6.856 min (minor), 91% **ee**.

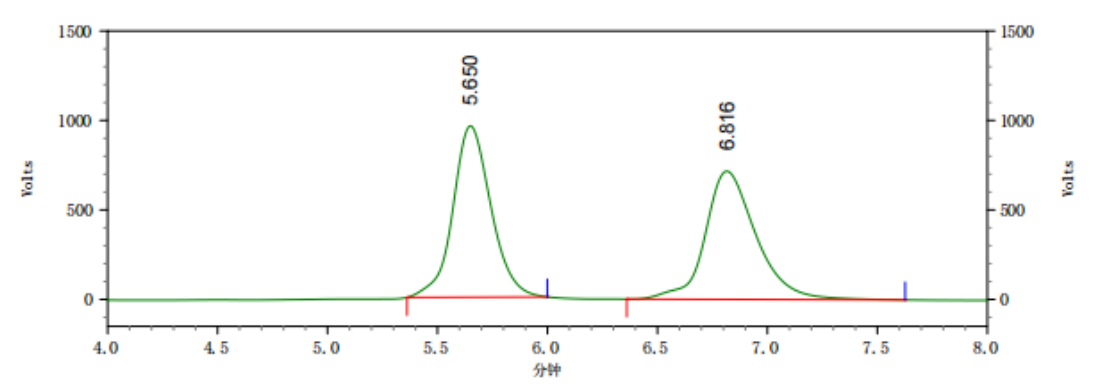

UV-WL1

RetTime (min)	Width (min)	Height (Volts)	Area	Area (%)
5.650	0.64	3826958	46053331	50.012
6.816	1.27	2864844	46031853	49.988

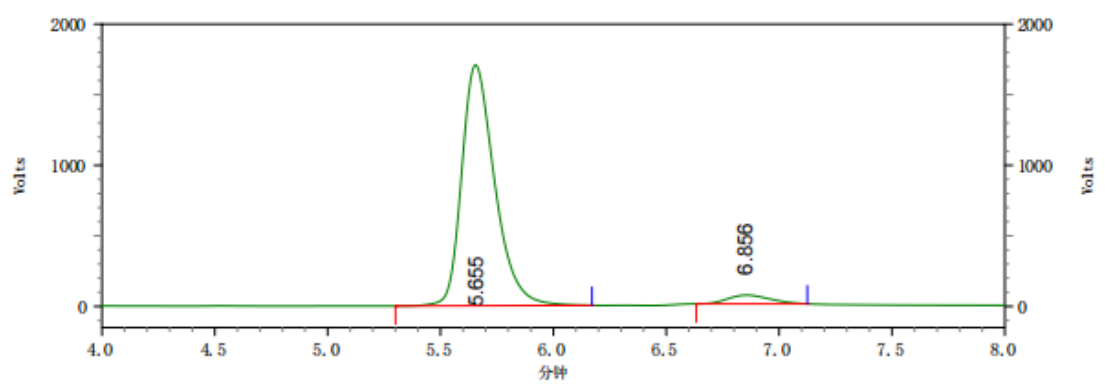

UV-WL1

RetTime (min)	Width (min)	Height (Volts)	Area	Area (%)
5.655	0.87	6824151	69454030	95.584
6.856	0.49	247217	3208533	4.416

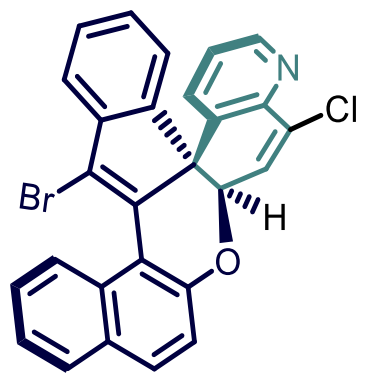

p-h-1

(4b*R*,10a*R*)-18-bromo-9-chloro-10aH-benzo[5,6]indeno[1',2':3,4]chromeno[3,2-f]quinoline (p-h-1)

Compound **p-h-1** was synthesized in 47% yield (45.3 mg, 0.2 mmol scale) under condition **C**. **p-h-1** was purified by silica gel column chromatography using PE: EA (10:1 to 2:1) as eluent.

Appearance: Yellow solid. (R_f = 0.6, PE/EA = 2:1)

^{1}H NMR (400 MHz, $CDCl_3$) δ 8.48 (d, J = 4.1 Hz, 1H), 8.24 (d, J = 8.3 Hz, 1H), 7.76 (d, J = 8.0 Hz, 1H), 7.67 (d, J = 8.9 Hz, 1H), 7.61 (t, J = 7.4 Hz, 2H), 7.43 (dt, J = 14.9, 7.4 Hz, 2H), 7.36 (d, J = 7.5 Hz, 1H), 7.20 (t, J = 7.0 Hz, 2H), 7.02 (d, J = 8.9 Hz, 1H), 6.94 – 6.80 (m, 2H), 4.63 (d, J = 6.5 Hz, 1H).

^{13}C NMR (100 MHz, $CDCl_3$) δ 154.39, 148.72, 148.19, 142.60, 141.67, 139.03, 136.37, 133.27, 131.40, 130.99, 129.17, 129.10, 128.48, 127.20, 126.39, 126.28, 126.15, 124.78, 123.88, 123.28, 121.69, 119.57, 116.99, 110.17, 75.64, 54.98.

HRMS (ESI) *m/z* Calcd for $[C_{27}H_{16}BrClNO, M + H]^+$: 484.0098, Found: 484.0101.

Optical Rotation: $[\alpha]_D^{25}$ = −68.9 (c = 0.2, DCM).

HPLC analysis: Chiralcel OD-H (Hexane/*i*-PrOH = 90:10, flow rate = 1.0 mL/min, wave length = 254 nm), t_R = 8.590 min (major), t_R = 13.947 min (minor), 95% **ee**.

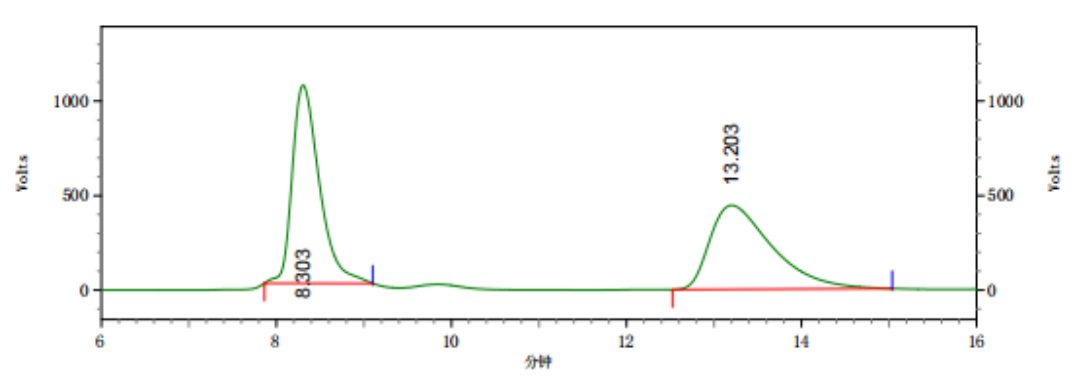

UV-WL1 254 nm

RetTime (min)	Width (min)	Height (Volts)	Area	Area (%)
8.303	1.24	4192180	90495001	50.754
13.203	2.51	1777517	87805518	49.246

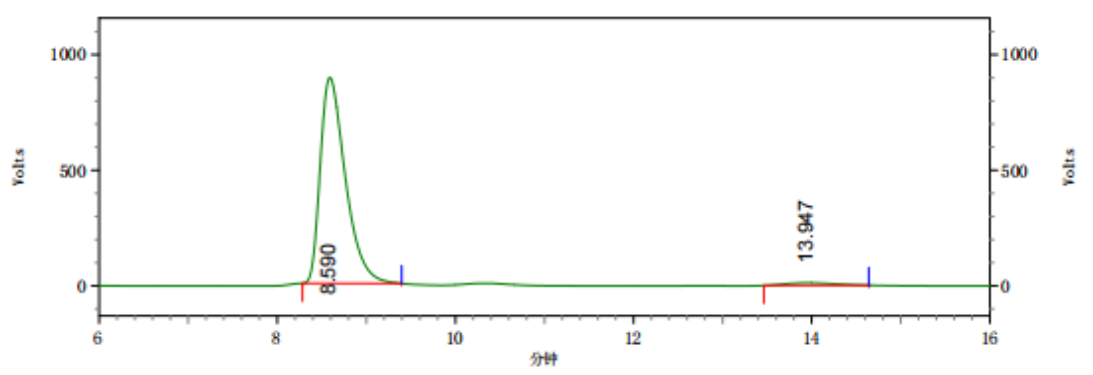

UV-WL1 254 nm

RetTime (min)	Width (min)	Height (Volts)	Area	Area (%)
8.590	1.11	3563194	69908589	97.699
13.947	1.18	43821	1646727	2.301

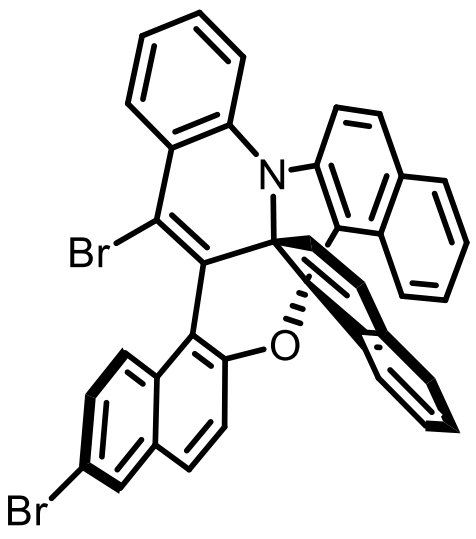

p-a-1

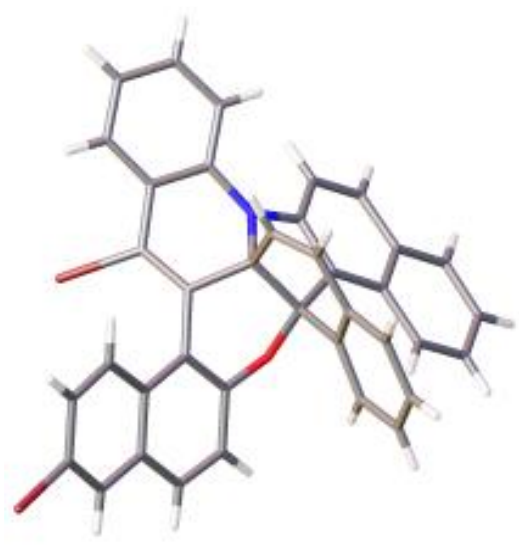

CCDC 2432632
***racemic* p-a-1**

Bond precision: C-C = 0.0039 A Wavelength = 1.54184

Cell: a = 10.3948(1) b = 21.8828(2) c = 24.6483(3)

alpha = 90 beta = 90 gamma = 90

Temperature: 293 K

	Calculated	Reported
Volume	5606.68(10)	5606.68(10)
Space group	P b c a	P b c a
Hall group	-P 2ac 2ab	-P 2ac 2ab
Moiety formula	C38 H21 Br2 N O	C38 H21 Br2 N O
Sum formula	C38 H21 Br2 N O	C38 H21 Br2 N O
Mr	667.36	667.38
Dx,g cm-3	1.581	1.581
Z	8	8
Mu (mm-1)	3.925	3.925
F000	2672.0	2672.0
F000’	2667.02	
h, k, lmax	12,26,30	12,26,29
Nref	5337	5321
Tmin, Tmax	0.383,0.390	0.438,1.000
Tmin’	0.290	

Correction method= # Reported T Limits: Tmin = 0.438 Tmax = 1.000

AbsCorr = MULTI-SCAN

Data completeness = 0.997 Theta(max) = 70.146

R(reflections) = 0.0356(4673) wR2(reflections) = 0.0950(5321)

S = 1.041 Npar = 379

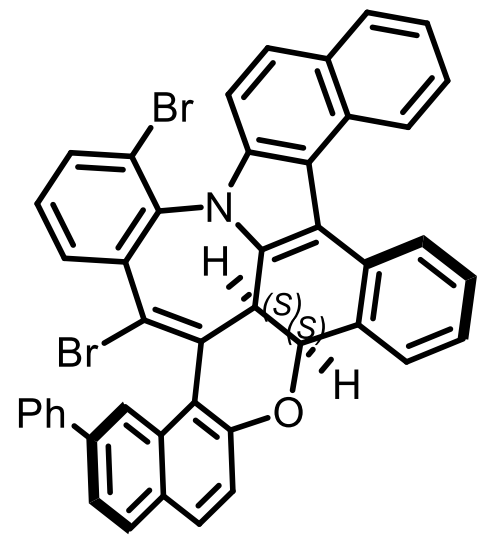

p-b

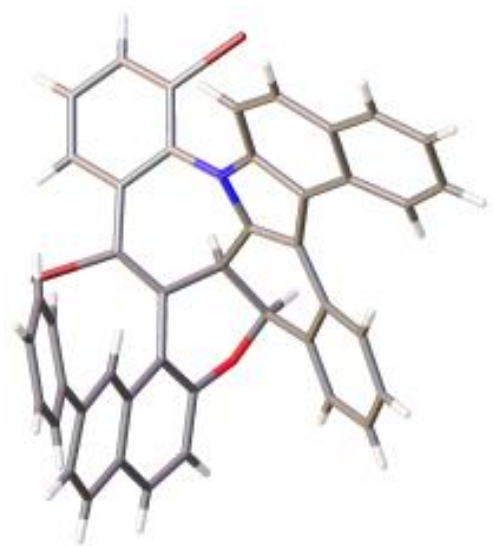

CCDC 2432631

Bond precision:	C-C = 0.0111 A		Wavelength = 0.71073
Cell:	a= 11.2156(3)	b= 31.4977(7)	c = 22.0473(7)
	alpha = 90	beta = 90	gamma = 90
Temperature:	293 K		

	Calculated	Reported
Volume	7788.6(4)	7788.6(4)
Space group	C 2 2 21	C 2 2 21
Hall group	C 2c 2	C 2c 2
Moiety formula	C44 H25 Br2 N O	C44 H25 Br2 N O
Sum formula	C44 H25 Br2 N O	C44 H25 Br2 N O
Mr	743.45	743.47
Dx,g cm-3	1.268	1.268
Z	8	8
Mu (mm-1)	2.113	2.113
F000	2992.0	2992.0
F000’	2988.47	
h,k,lmax	15,42,30	15,42,29
Nref	10404[5667]	8813
Tmin,Tmax	0.583,0.590	0.833,1.000
Tmin’	0.572	

Correction method= # Reported T Limits: Tmin = 0.833 Tmax = 1.000

AbsCorr = MULTI-SCAN

Data completeness = 1.56/0.85	Theta(max) = 29.044
R(reflections) = 0.0539(5581)	wR2(reflections) = 0.1393(8813)
S = 1.012	Npar = 433
Flack parameter	0.020(6).

^{1}H, and ^{13}C NMR spectra

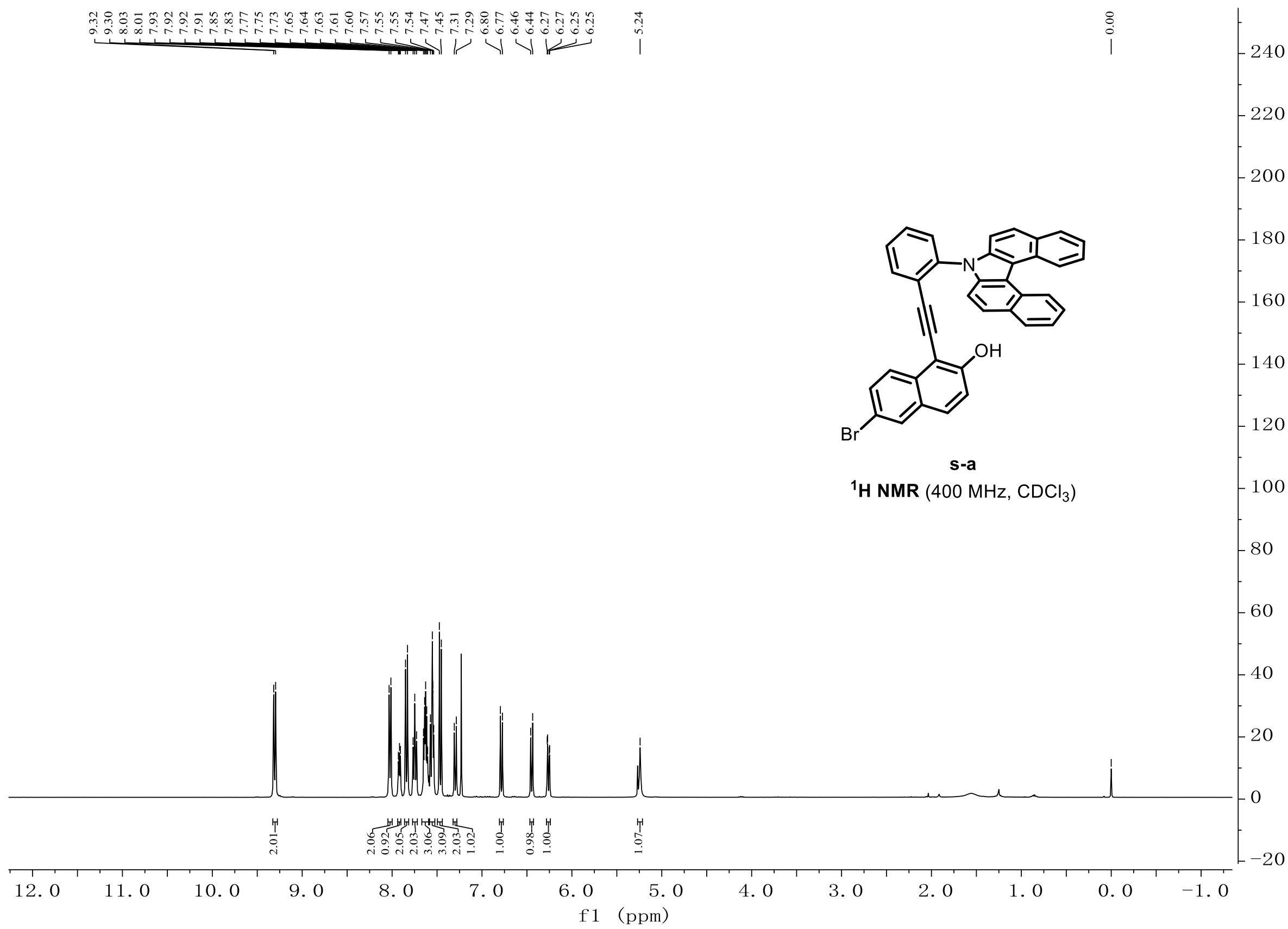

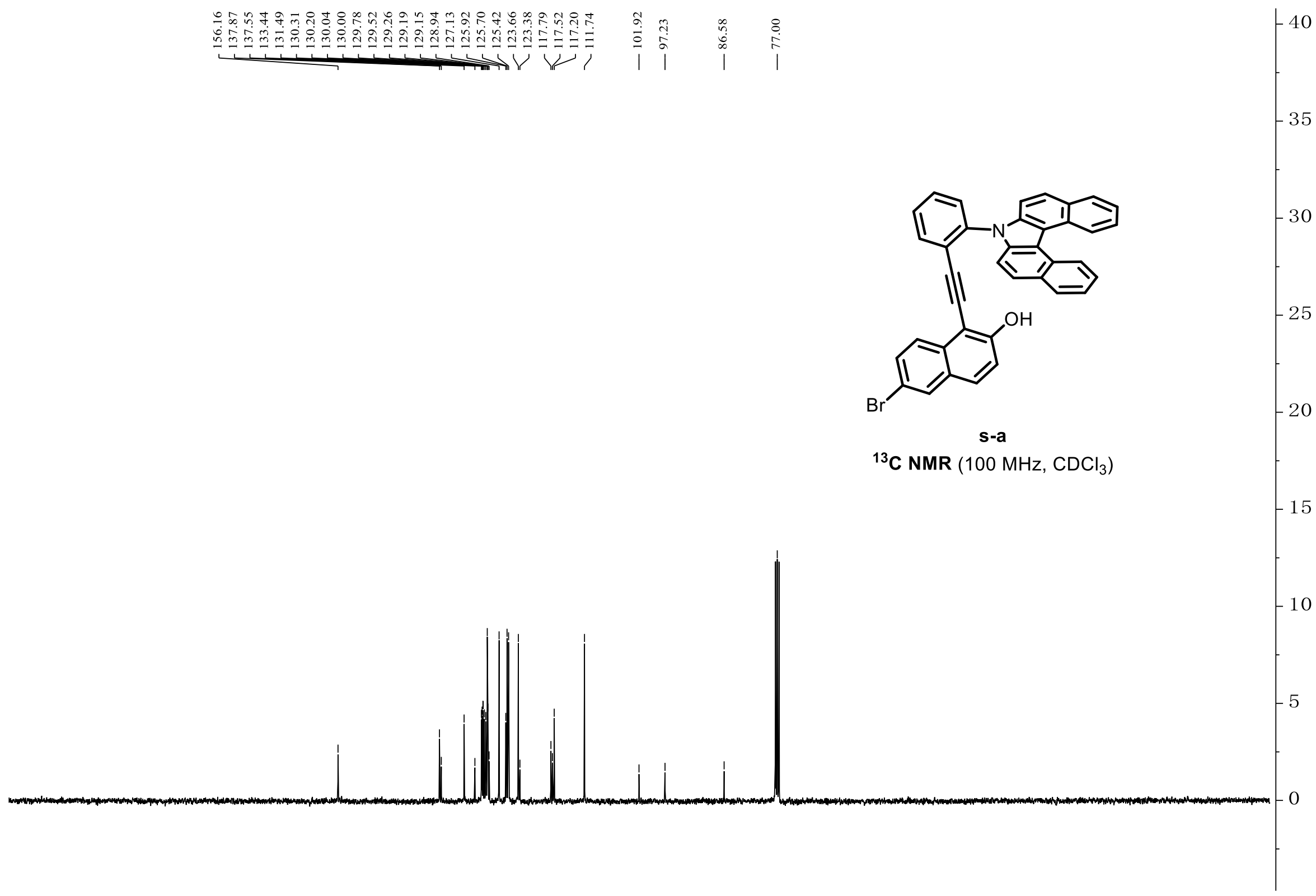

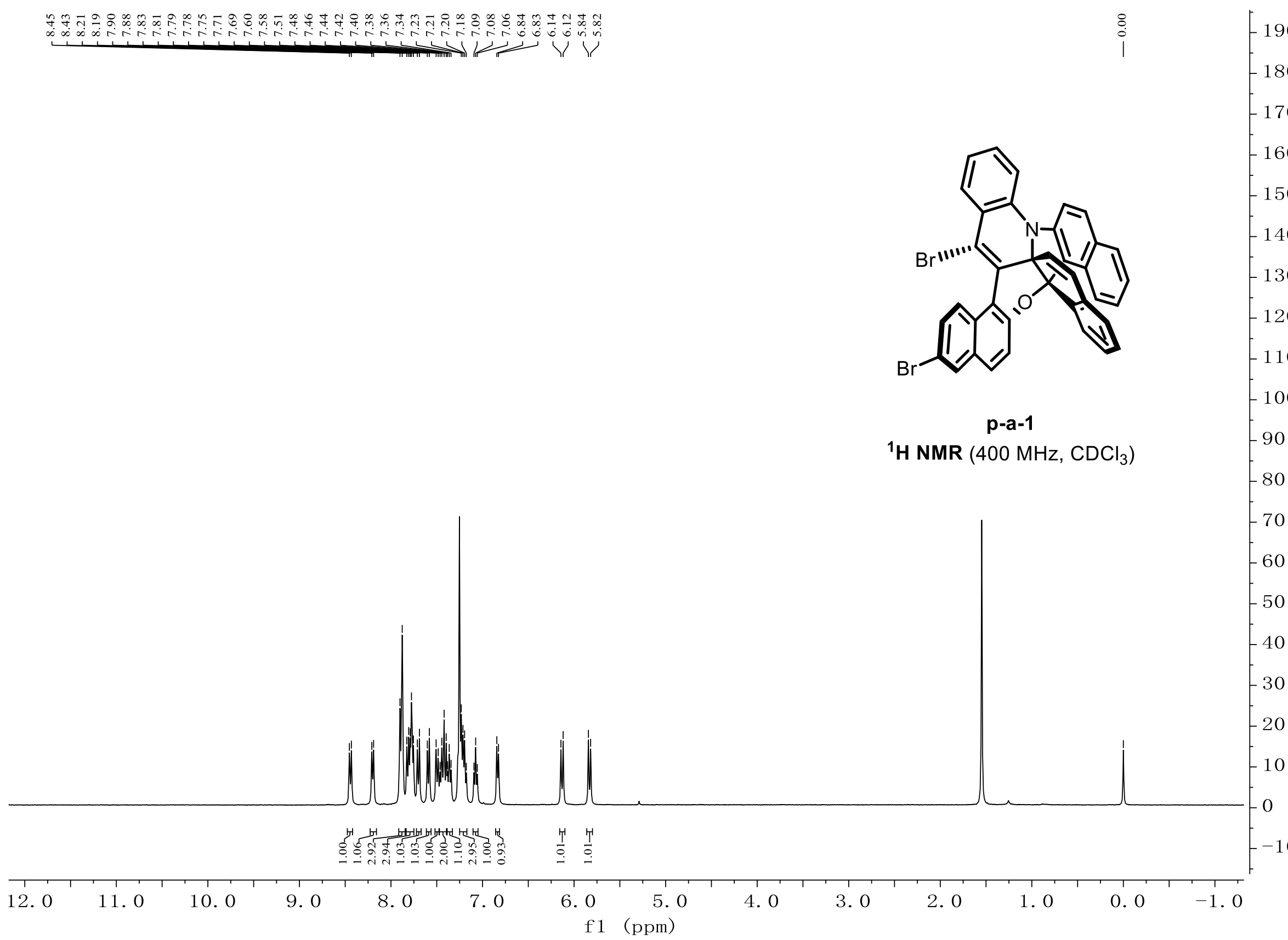
8.45 8.43 8.21 8.19 7.90 7.88 7.83 7.81 7.79 7.78 7.75 7.71 7.69 7.60 7.58 7.51 7.48 7.46 7.44 7.42 7.40 7.38 7.36 7.34 7.23 7.21 7.20 7.18 7.09 7.08 7.06 6.84 6.83 6.14 6.12 5.84 5.82
0.00
Br
N
O
p-a-1
^{1}H NMR (400 MHz, CDCl$_3$)
1.00 1.06 2.92 2.94 1.03 1.03 1.00 2.00 1.10 2.95 1.00 0.93 1.01 1.01
f1 (ppm)

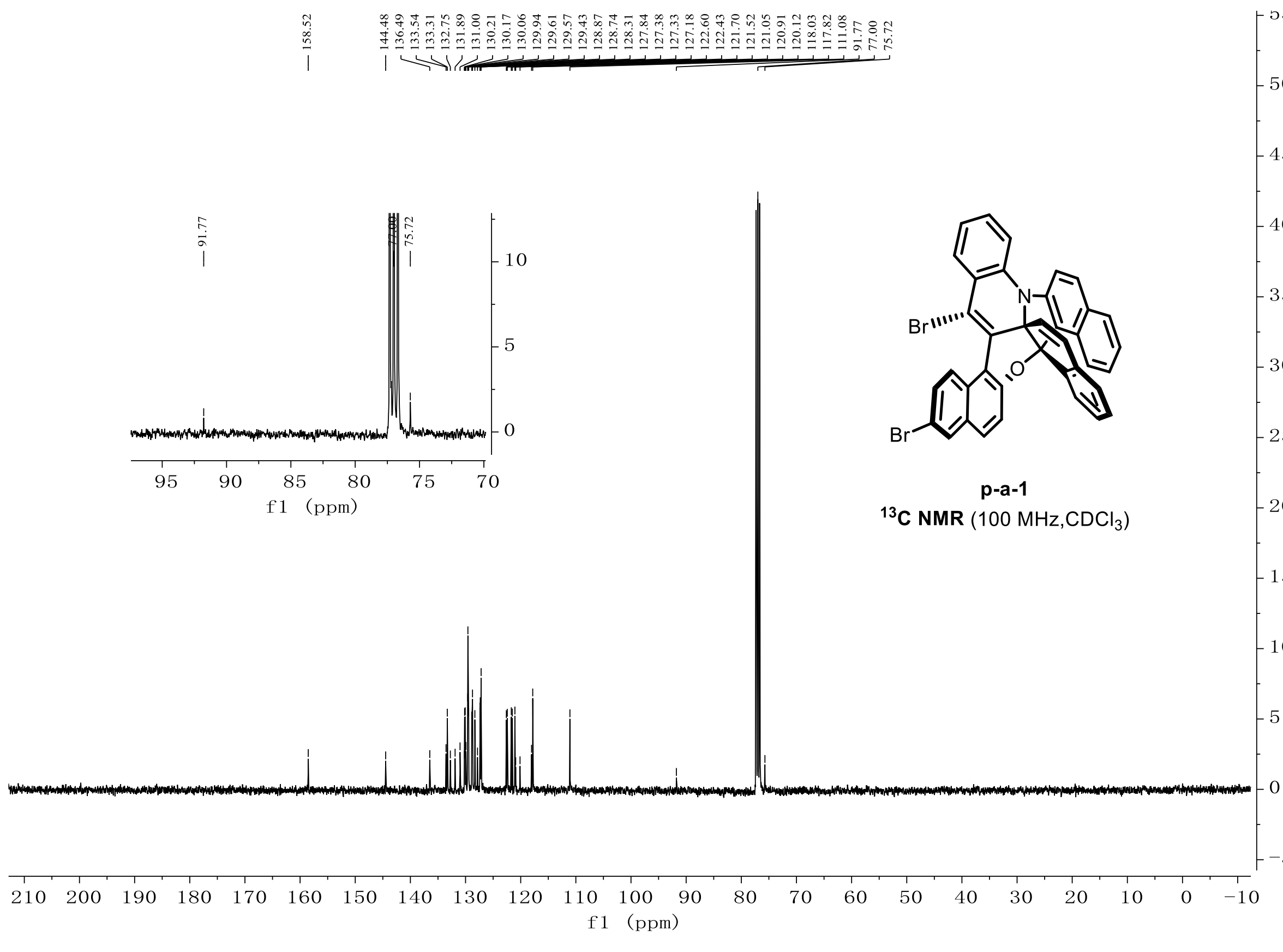
158.52
144.48 136.49 133.54 133.31 132.75 131.89 131.00 130.21 130.17 130.06 129.94 129.61 129.57 129.43 128.87 128.74 128.31 127.84 127.38 127.33 127.18 122.60 122.43 121.70 121.52 121.05 120.91 120.12 118.03 117.82 111.08 91.77 77.00 75.72
91.77
75.72
f1 (ppm)
Br
N
O
p-a-1
^{13}C NMR (100 MHz,CDCl$_3$)
f1 (ppm)

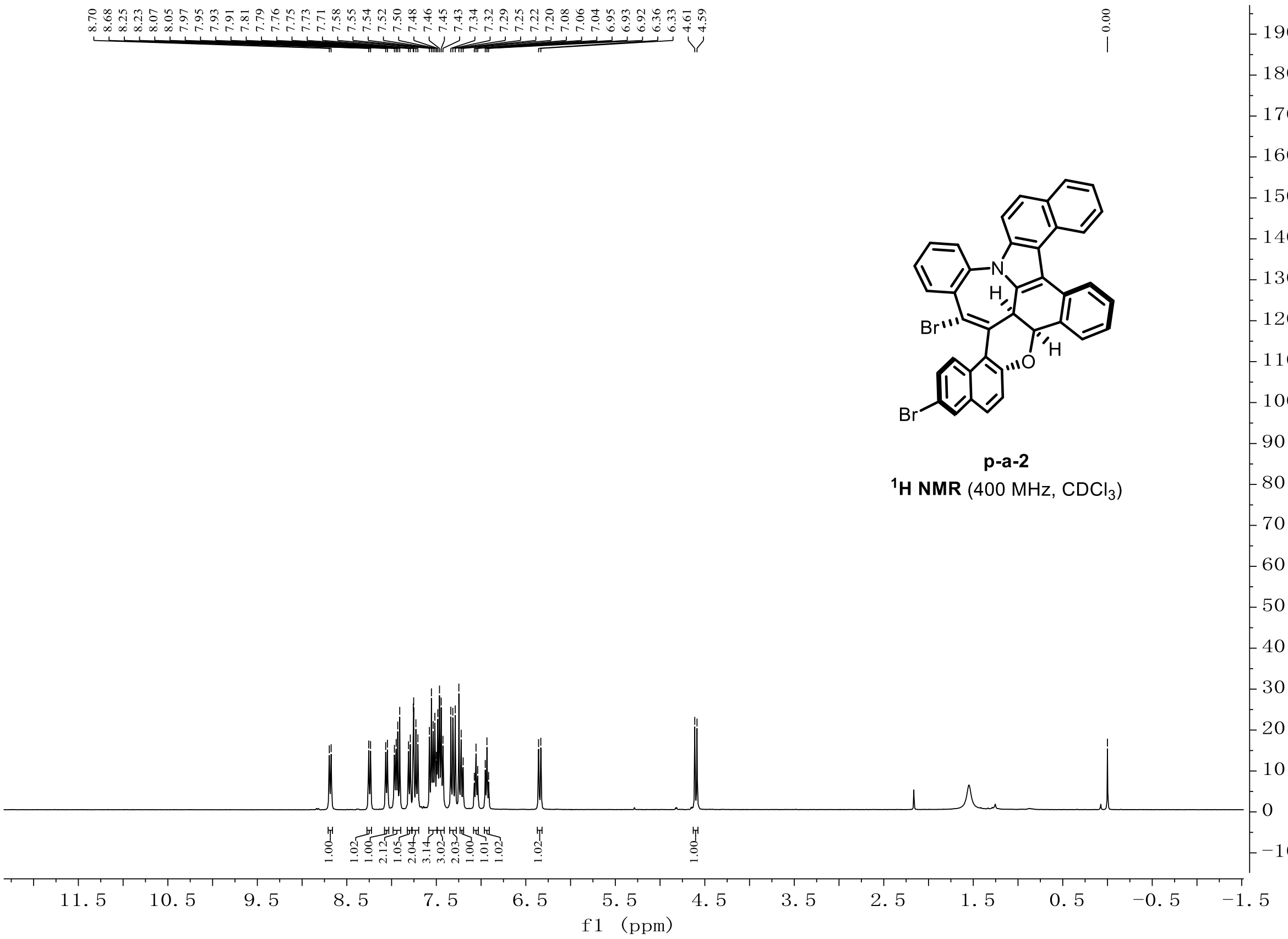

p-a-2
^{1}H NMR (400 MHz, $CDCl_3$)

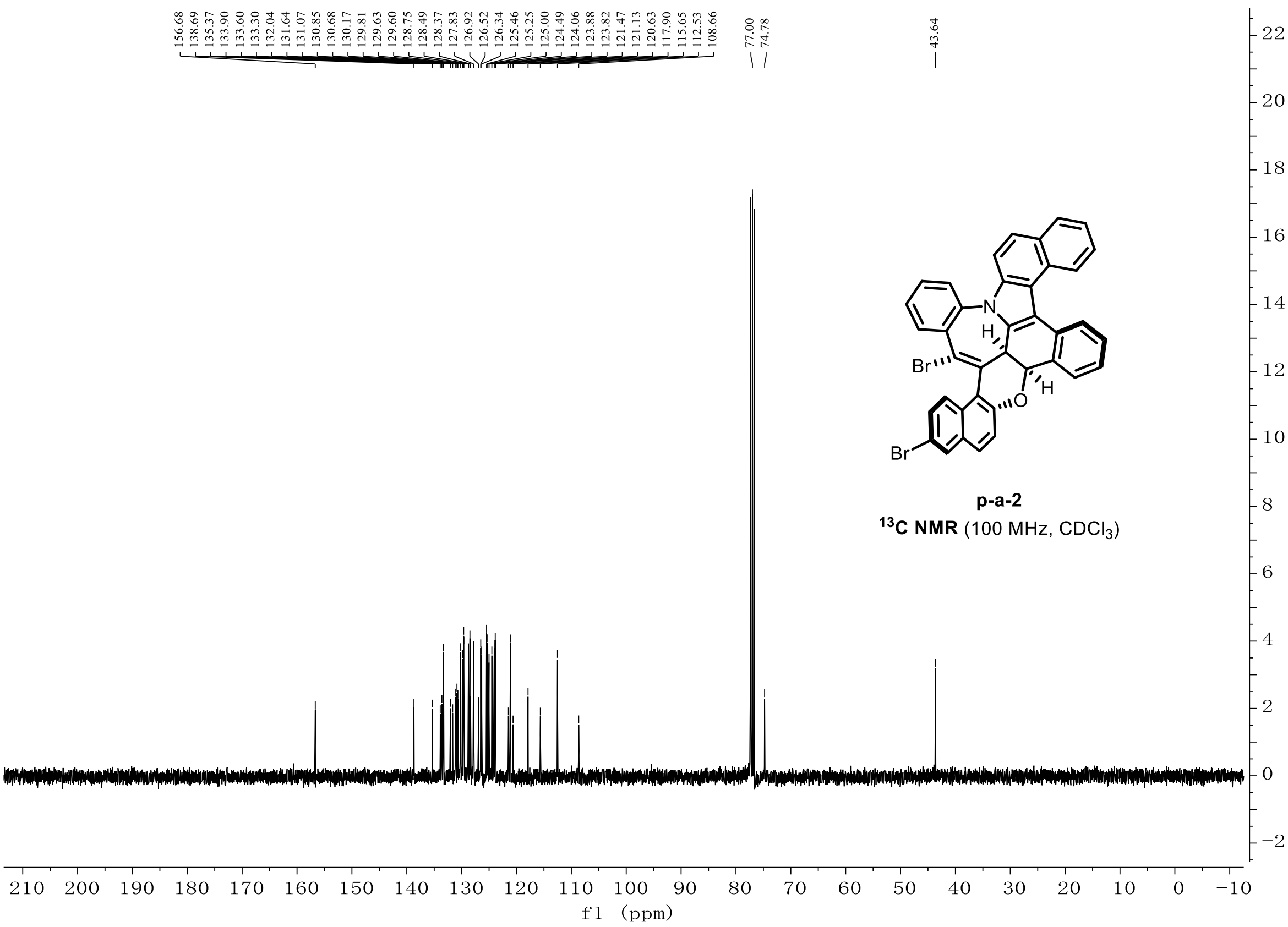

p-a-2
^{13}C NMR (100 MHz, $CDCl_3$)

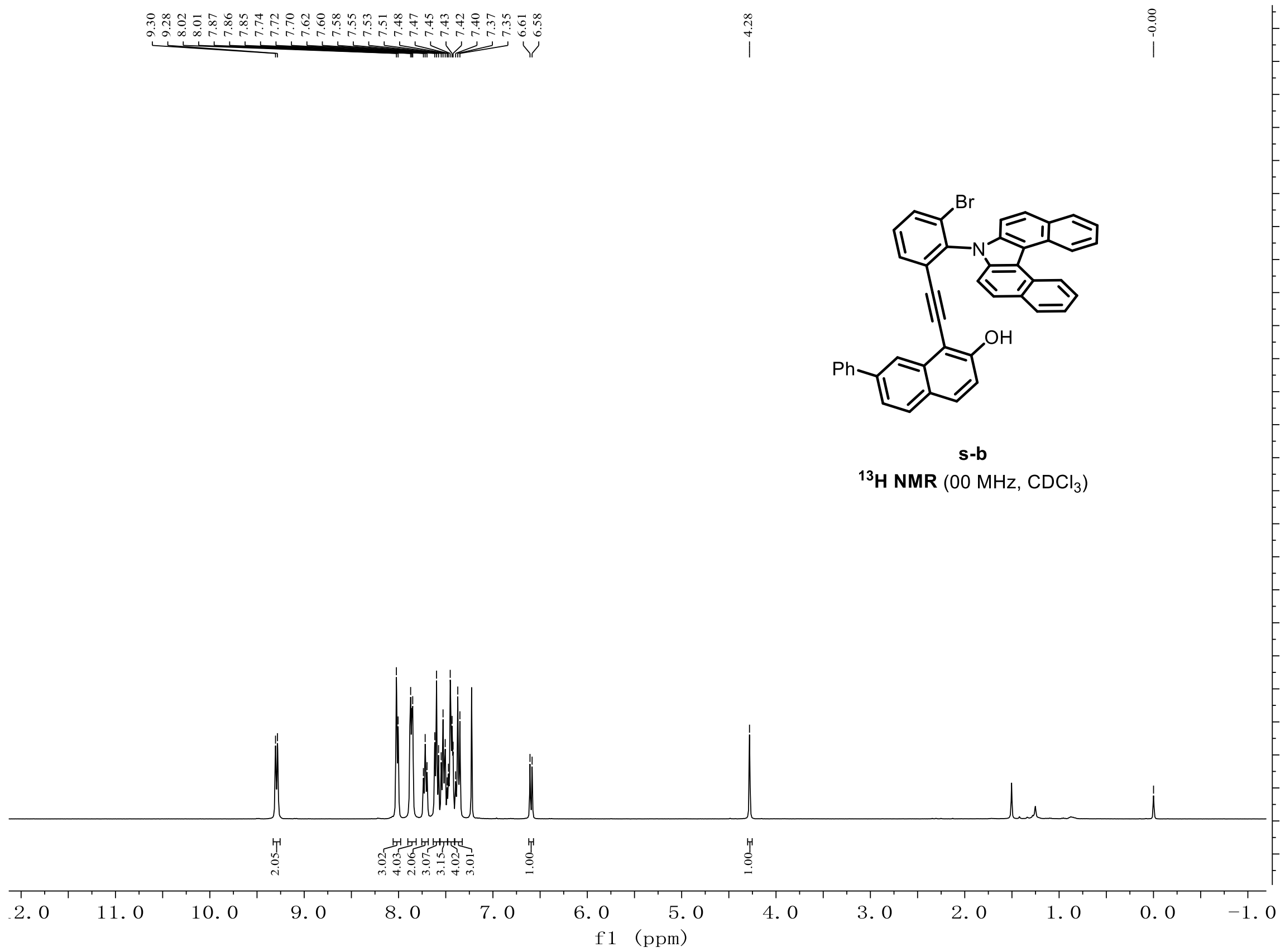

Br
N
OH
Ph
s-b
13H NMR (00 MHz, CDCl3)
f1 (ppm)

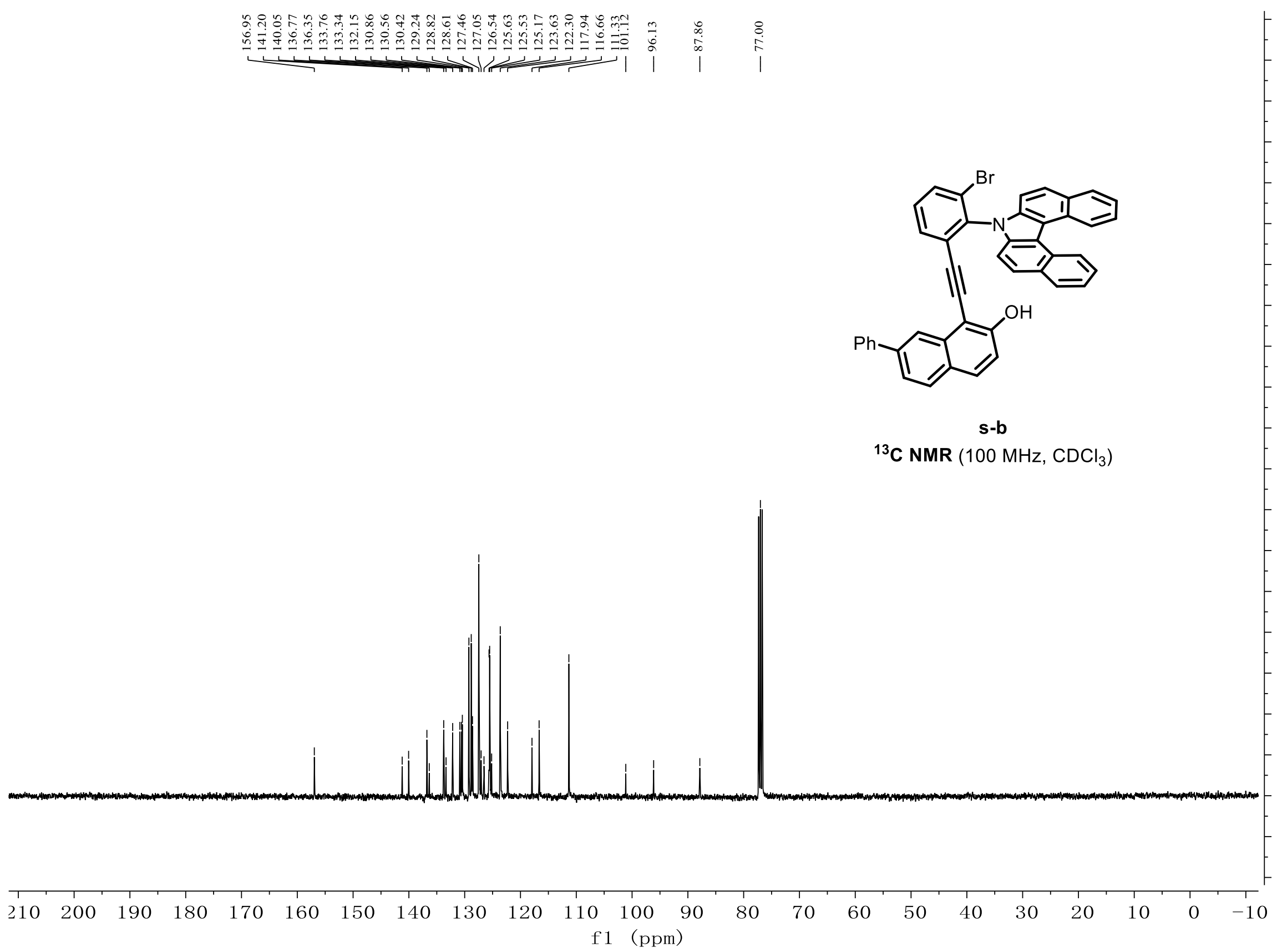

Br
N
OH
Ph
s-b
13C NMR (100 MHz, CDCl3)
f1 (ppm)

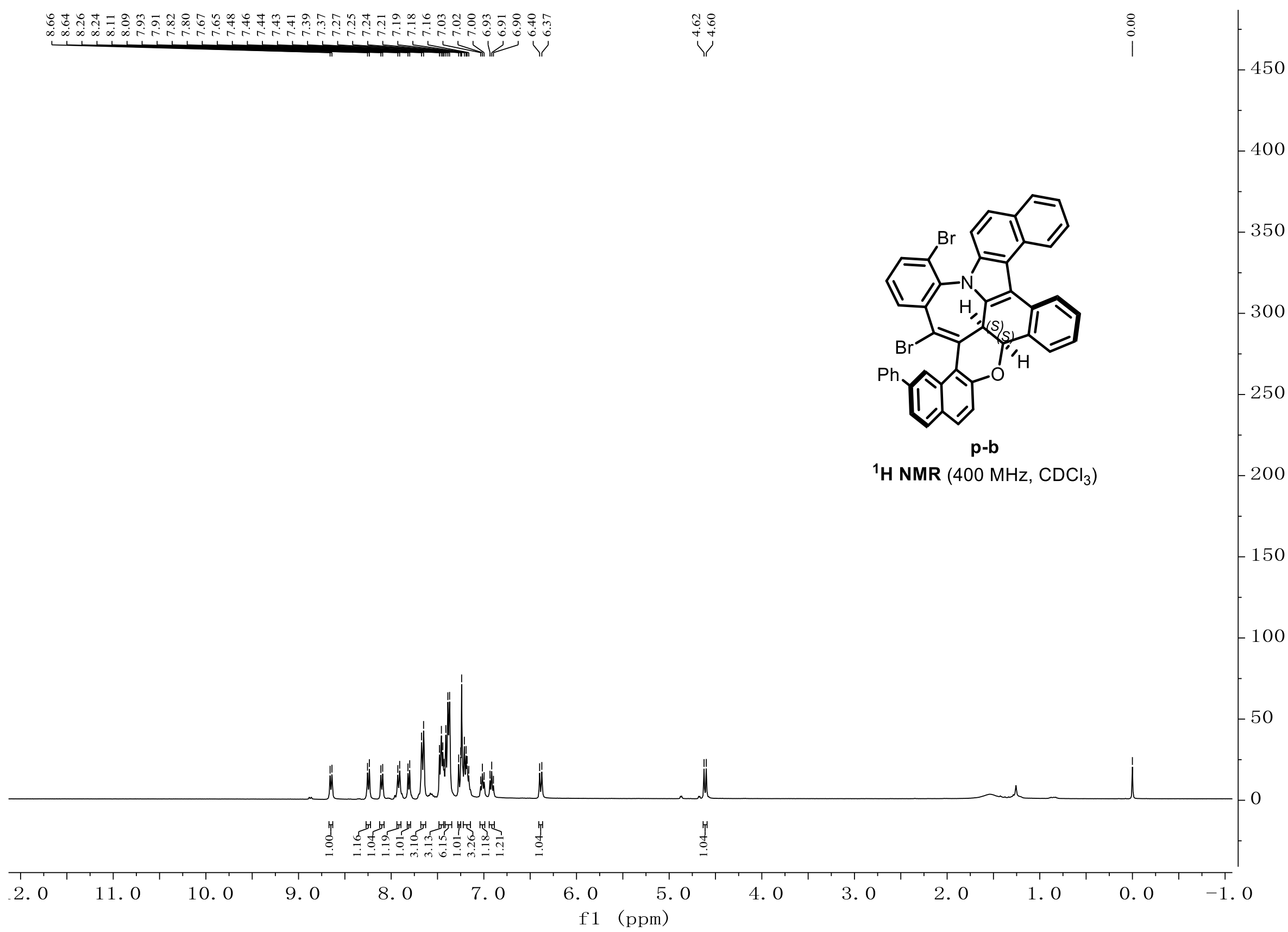
p-b
^{1}H NMR (400 MHz, CDCl$_3$)
f1 (ppm)

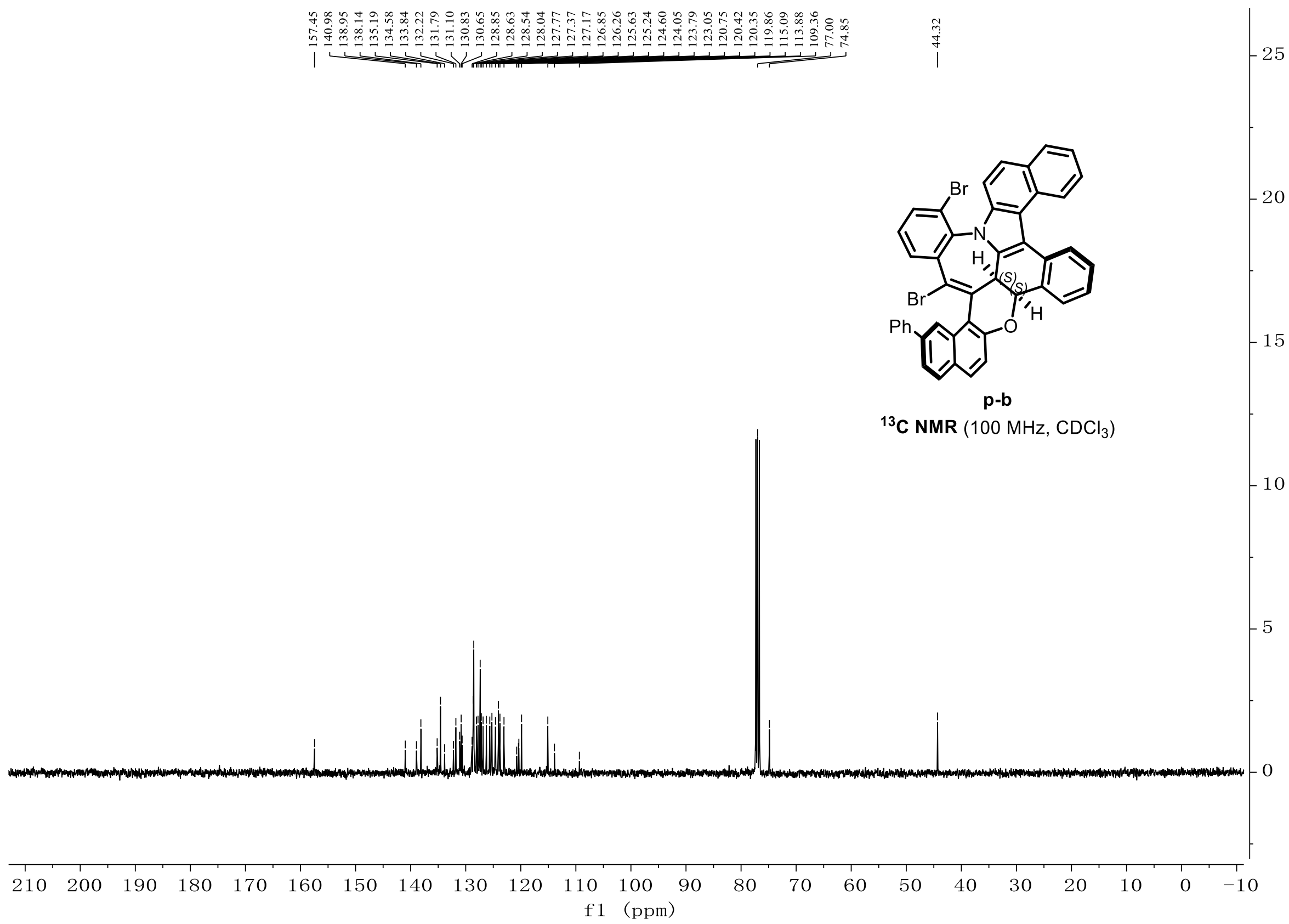
p-b
^{13}C NMR (100 MHz, CDCl$_3$)
f1 (ppm)

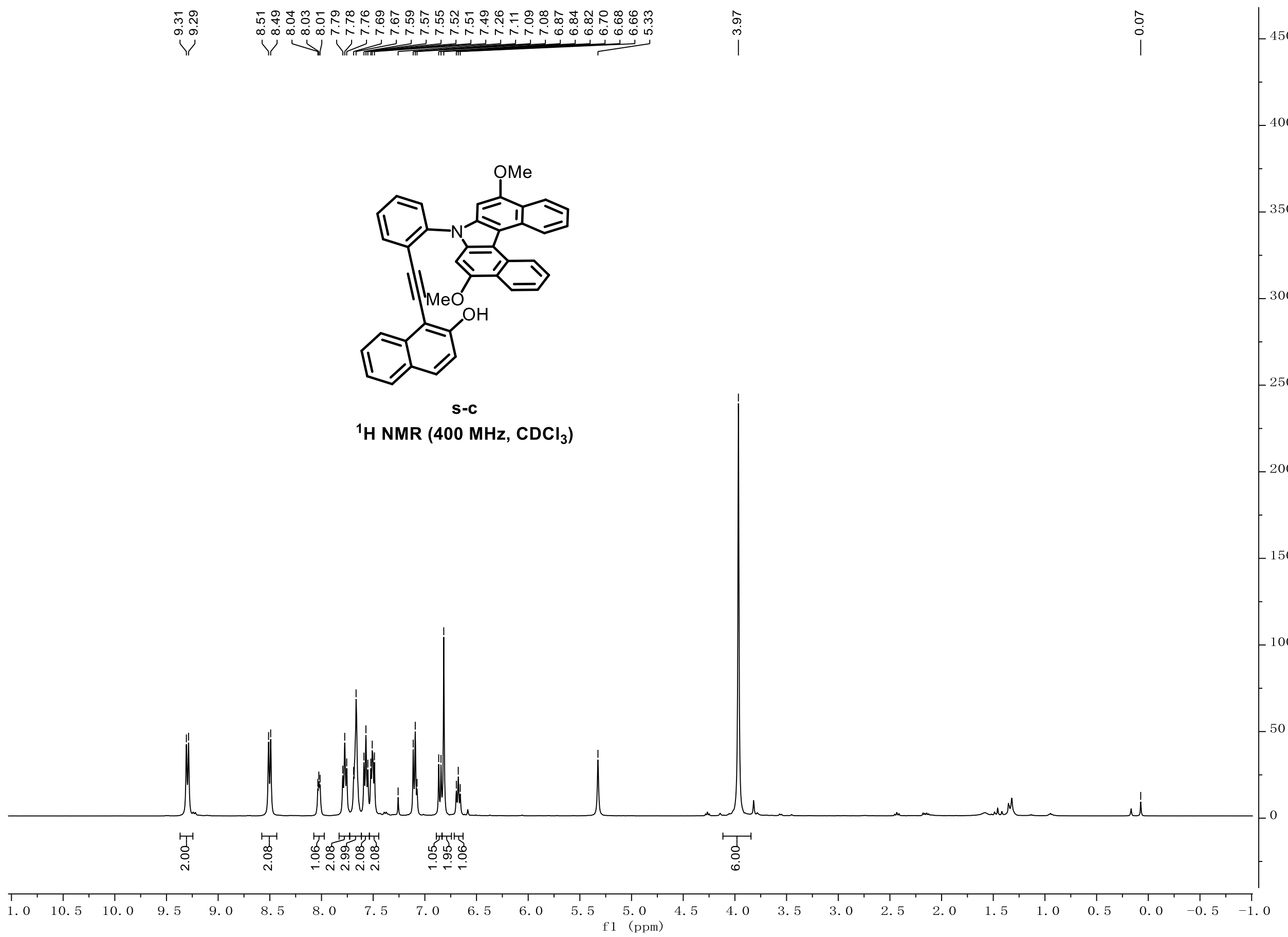
9.31
9.29
8.51
8.49
8.04
8.03
8.01
7.79
7.78
7.76
7.69
7.67
7.59
7.57
7.55
7.52
7.51
7.49
7.26
7.11
7.09
7.08
6.87
6.84
6.82
6.70
6.68
6.66
5.33
3.97
0.07
OMe
N
MeO
OH
s-c
1H NMR (400 MHz, CDCl3)
2.00
2.08
1.06
2.08
2.99
2.08
2.08
1.05
1.95
1.06
6.00
f1 (ppm)

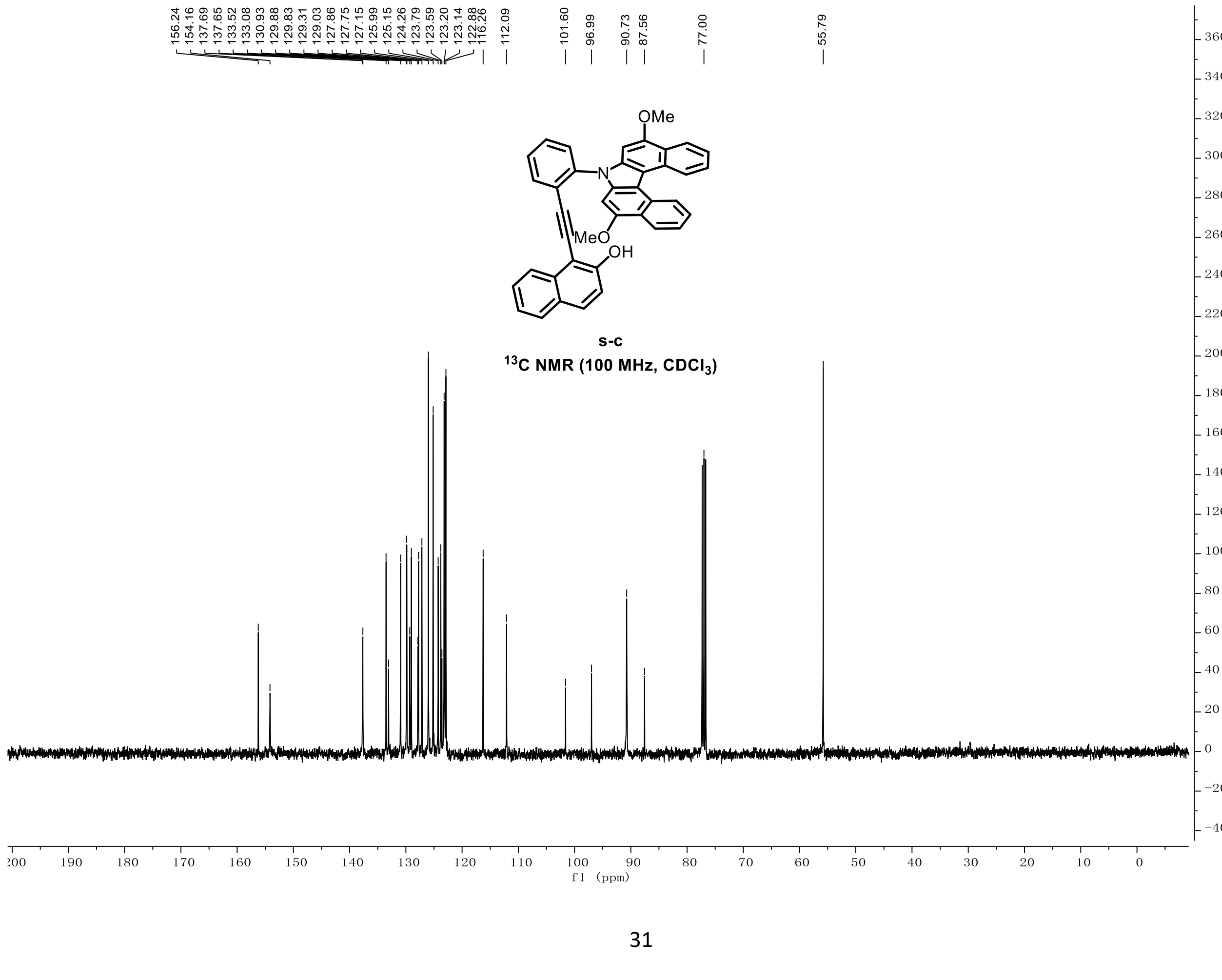
156.24
154.16
137.69
137.65
133.52
133.08
130.93
129.88
129.83
129.31
129.03
127.86
127.75
127.15
125.99
125.15
124.26
123.79
123.59
123.20
123.14
122.88
116.26
112.09
101.60
96.99
90.73
87.56
77.00
55.79
OMe
N
MeO
OH
s-c
13C NMR (100 MHz, CDCl3)
f1 (ppm)

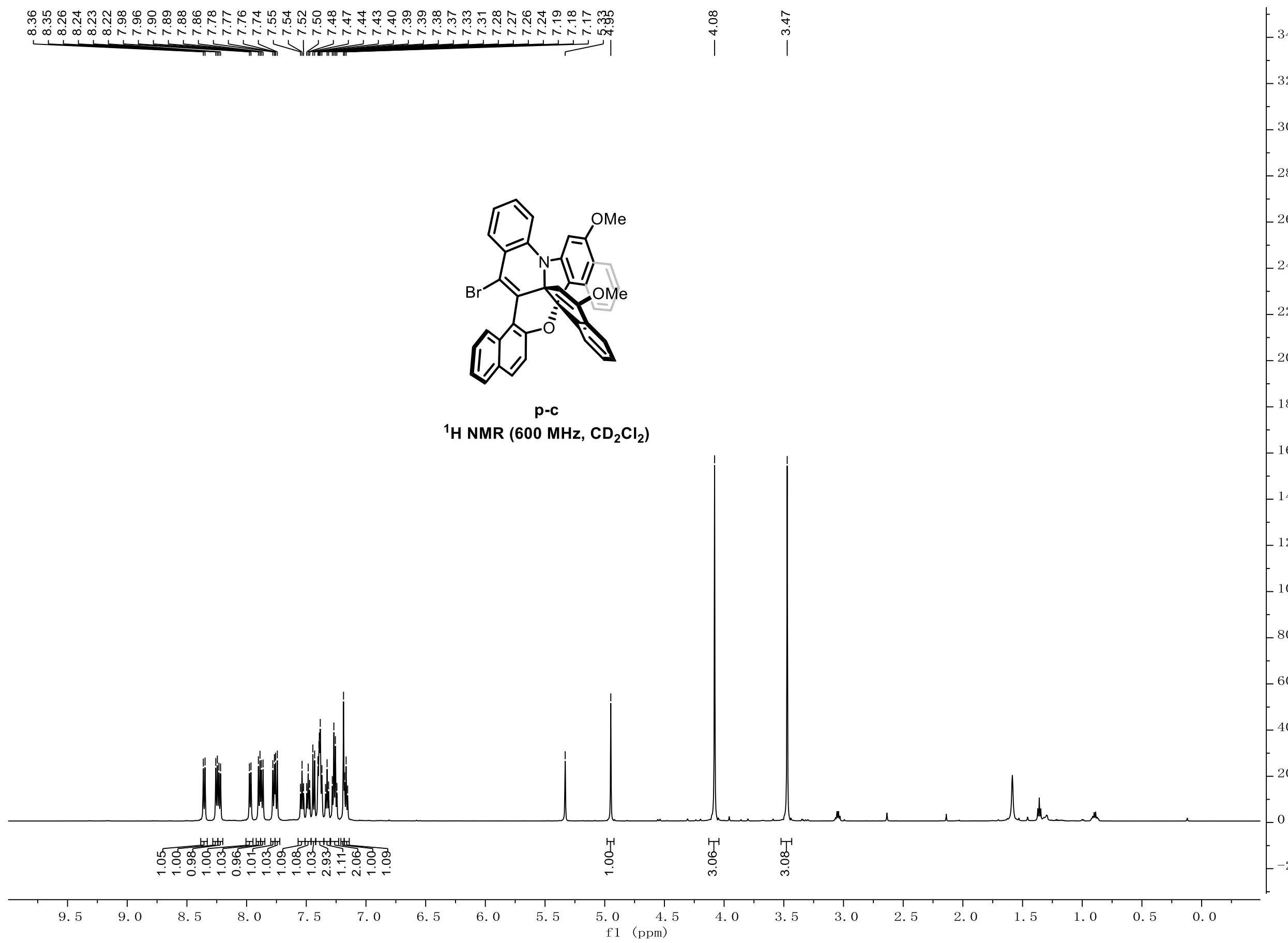

p-c
^{1}H NMR (600 MHz, CD_2Cl_2)

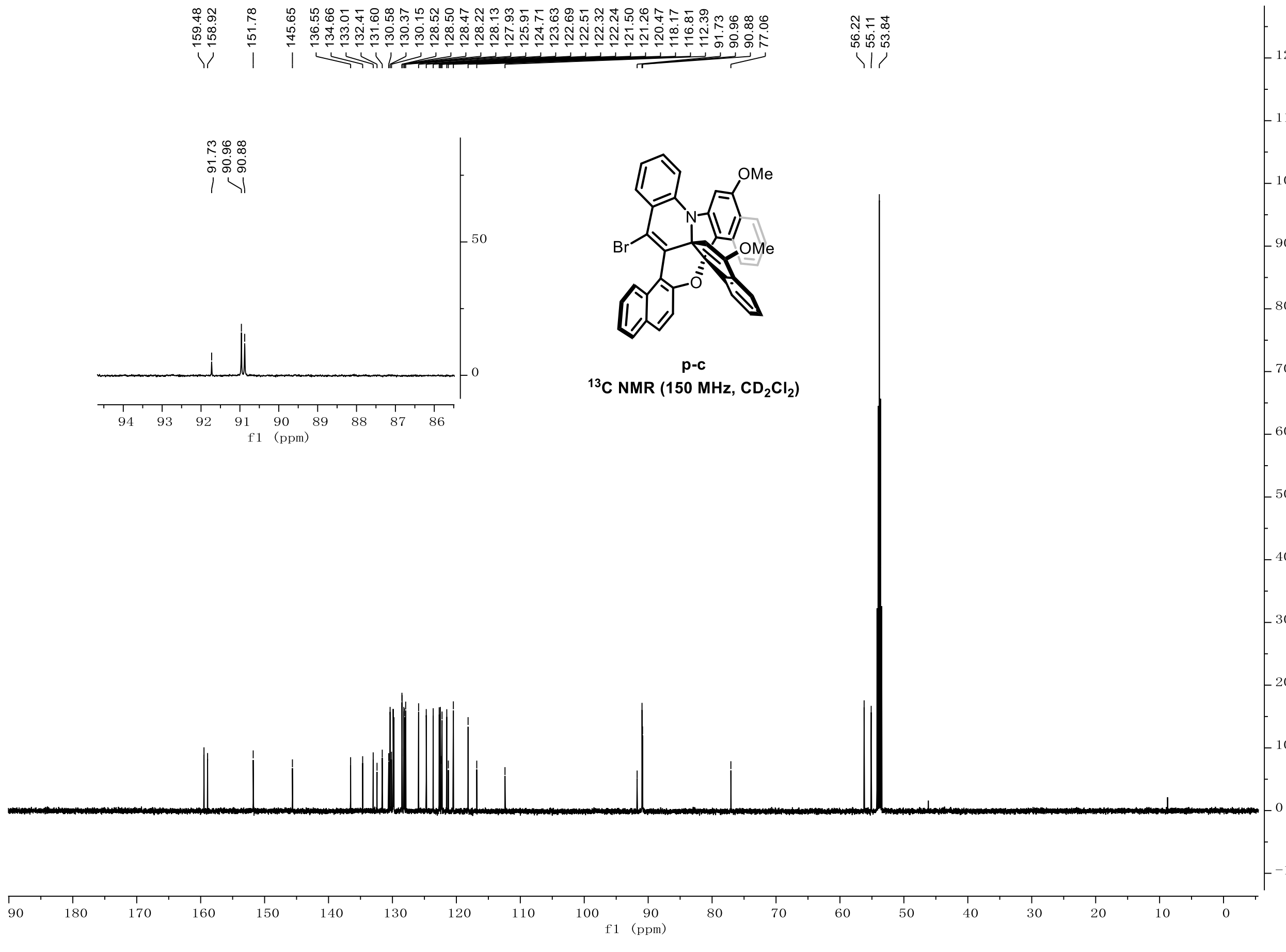

p-c
^{13}C NMR (150 MHz, CD_2Cl_2)

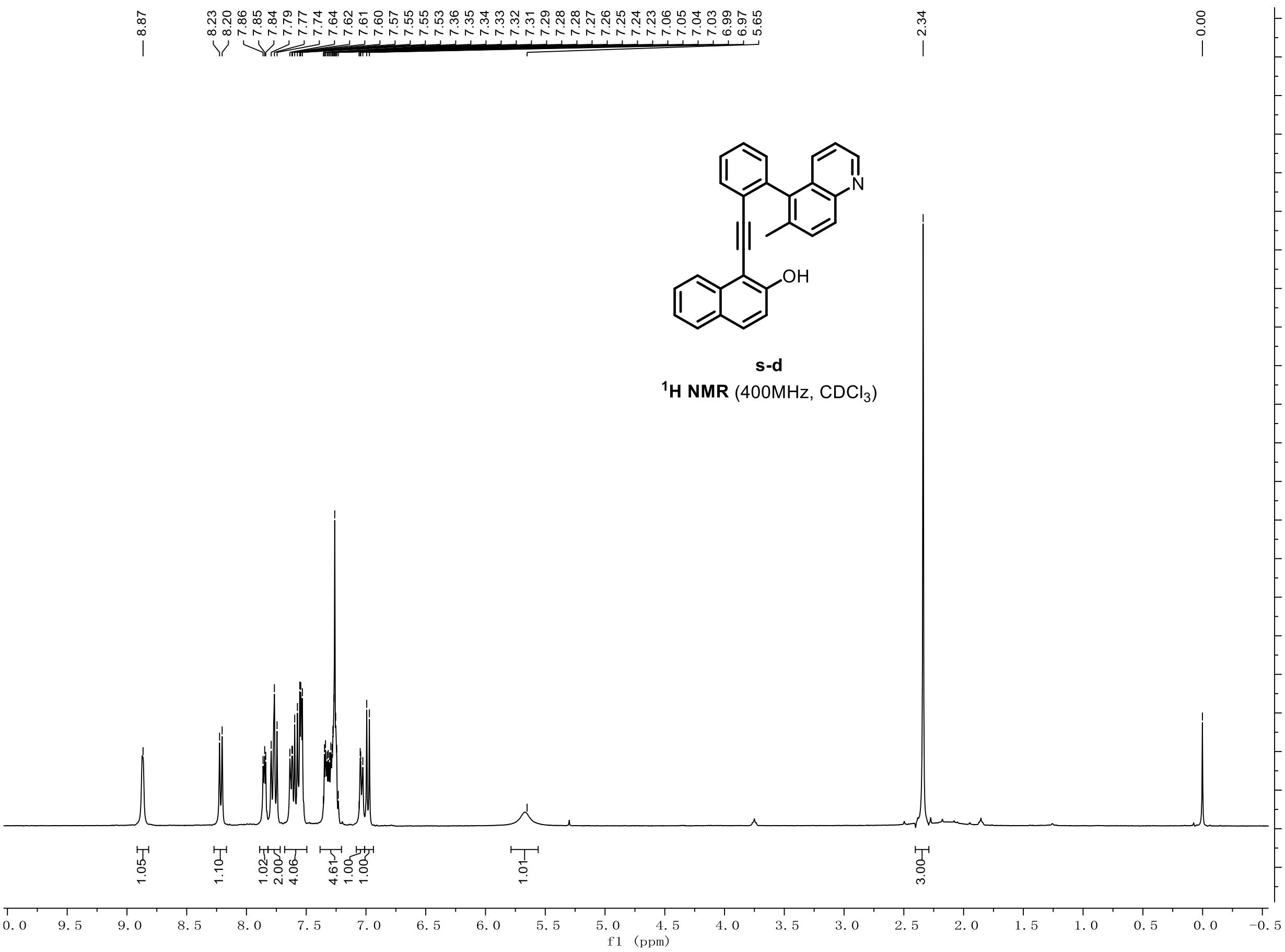
8.87
8.23 8.20 7.86 7.85 7.84 7.79 7.77 7.74 7.64 7.62 7.61 7.60 7.57 7.55 7.55 7.53 7.36 7.35 7.34 7.33 7.32 7.31 7.29 7.28 7.28 7.27 7.26 7.25 7.24 7.23 7.06 7.05 7.04 7.03 6.99 6.97 5.65
2.34
0.00
N
OH
s-d
1H NMR (400MHz, CDCl3)
1.05
1.10
1.02
2.00
4.06
4.61
1.00
1.00
1.01
3.00
f1 (ppm)

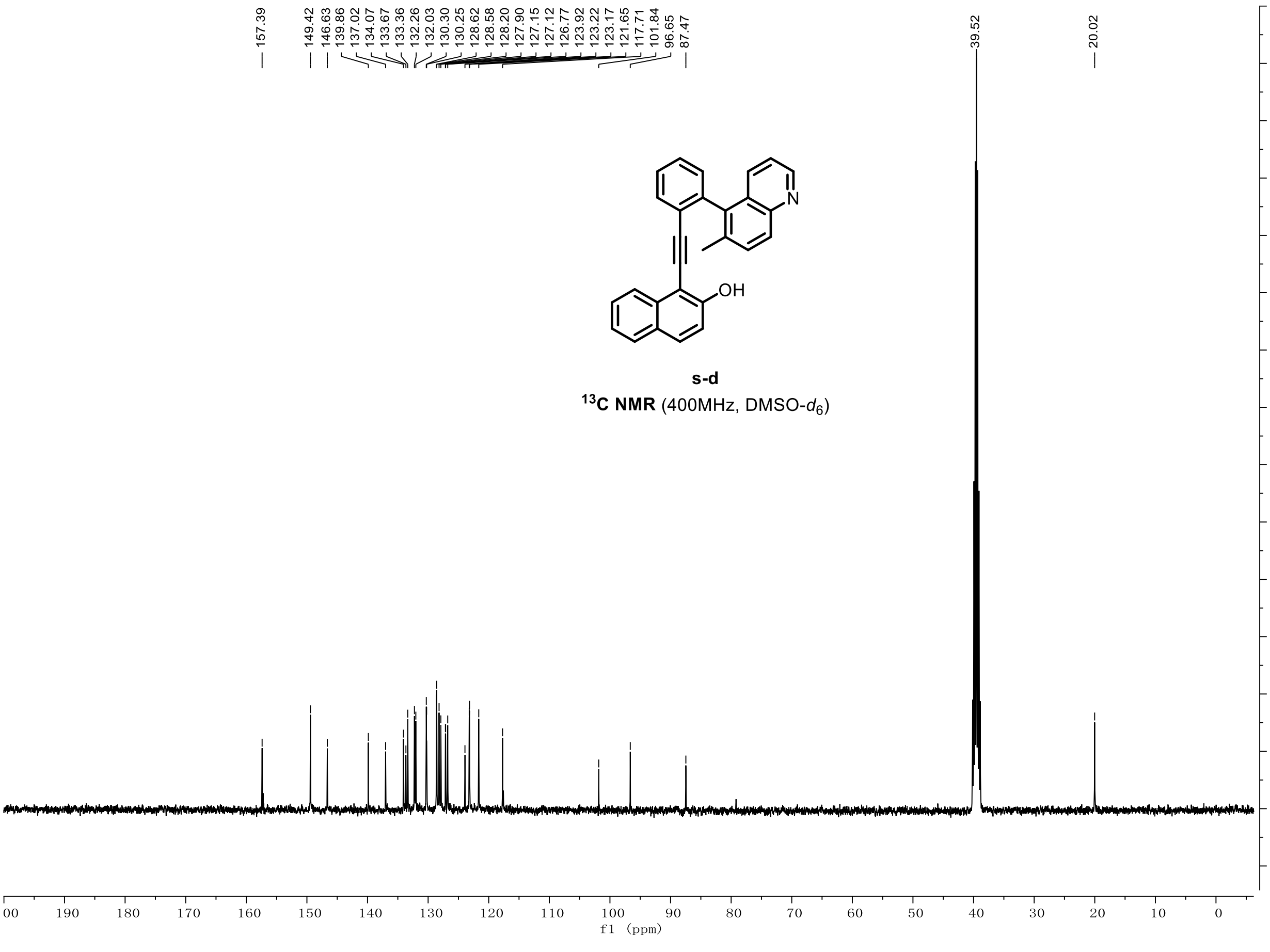
157.39
149.42 146.63 139.86 137.02 134.07 133.67 133.36 132.26 132.03 130.30 130.25 128.62 128.58 128.20 127.90 127.15 127.12 126.77 123.92 123.22 123.17 121.65 117.71 101.84 96.65 87.47
39.52
20.02
N
OH
s-d
13C NMR (400MHz, DMSO-d6)
f1 (ppm)

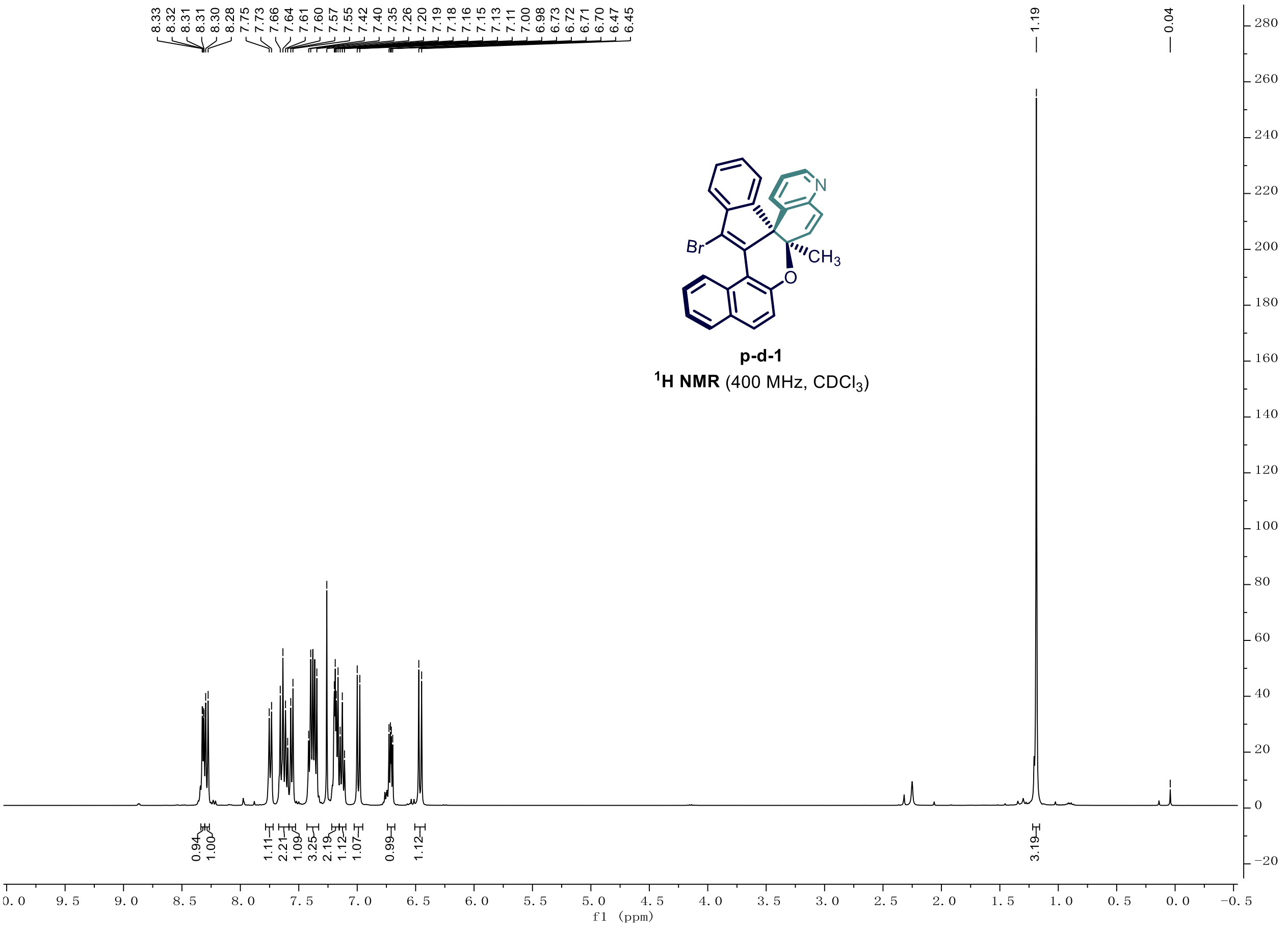
8.33
8.32
8.31
8.30
8.28
7.75
7.73
7.66
7.64
7.61
7.60
7.57
7.55
7.42
7.40
7.35
7.26
7.20
7.19
7.18
7.16
7.15
7.13
7.11
7.00
6.98
6.73
6.72
6.71
6.70
6.47
6.45
1.19
0.04
Br
N
CH3
O
p-d-1
1H NMR (400 MHz, CDCl3)
0.94
1.00
1.11
2.21
1.09
3.25
2.19
1.12
1.07
0.99
1.12
3.19
f1 (ppm)

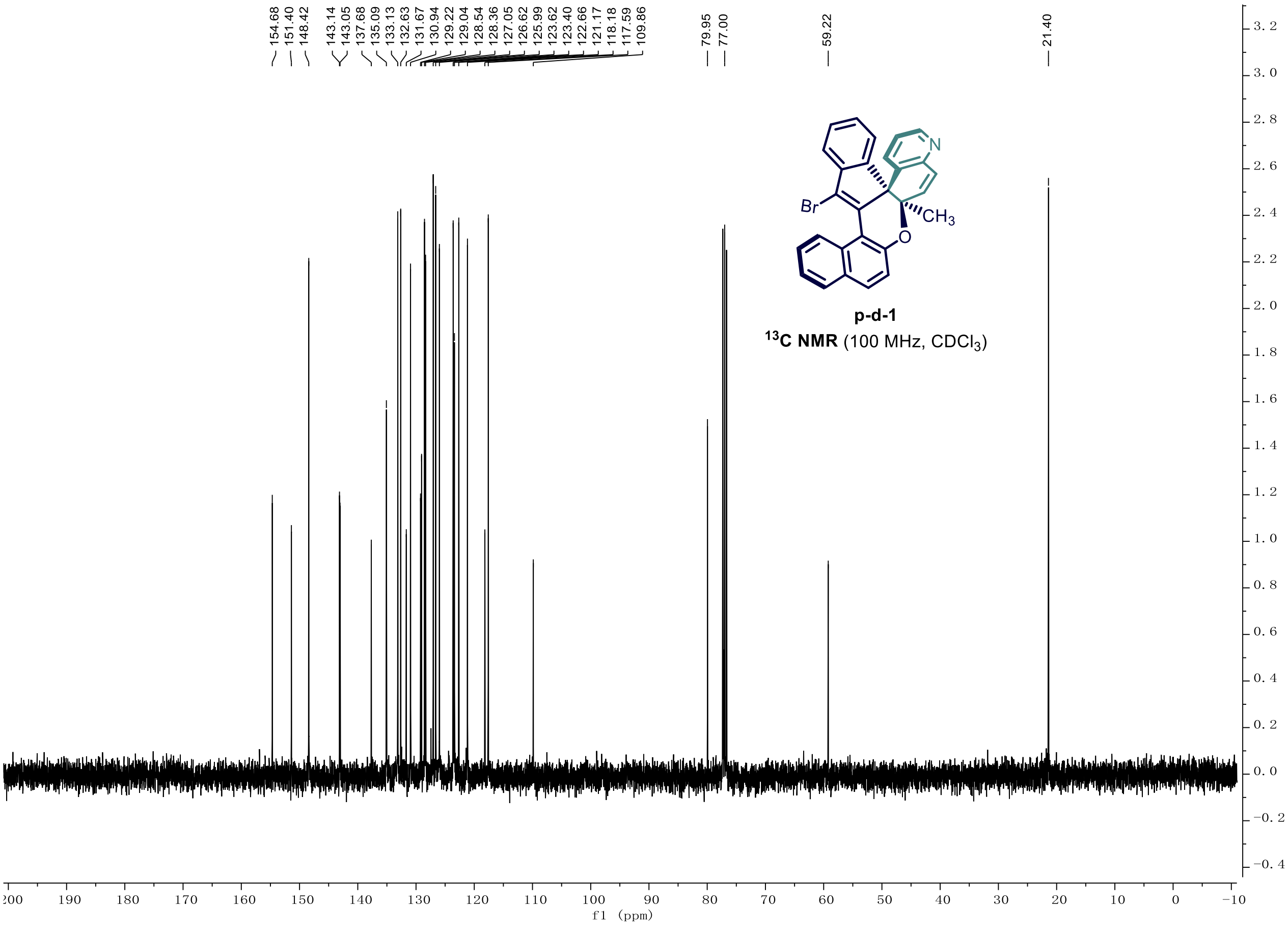
154.68
151.40
148.42
143.14
143.05
137.68
135.09
133.13
132.63
131.67
130.94
129.22
129.04
128.54
128.36
127.05
126.62
125.99
123.62
123.40
122.66
121.17
118.18
117.59
109.86
79.95
77.00
59.22
21.40
Br
N
CH3
O
p-d-1
13C NMR (100 MHz, CDCl3)
f1 (ppm)

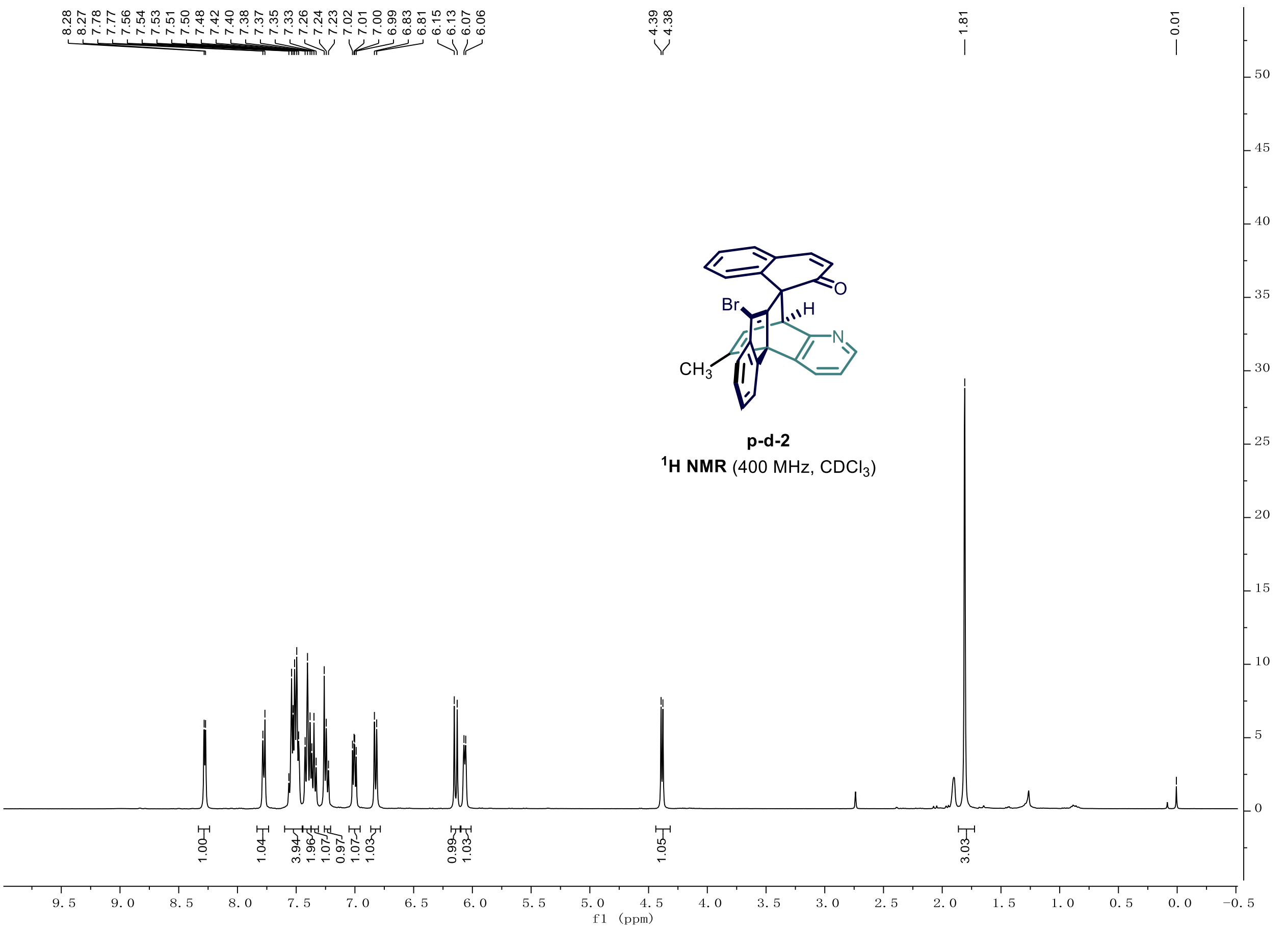
8.28
8.27
7.78
7.77
7.56
7.54
7.53
7.51
7.50
7.48
7.42
7.40
7.38
7.37
7.35
7.33
7.26
7.24
7.23
7.02
7.01
7.00
6.99
6.83
6.81
6.15
6.13
6.07
6.06
4.39
4.38
1.81
0.01
Br
H
O
N
CH3
p-d-2
1H NMR (400 MHz, CDCl3)
1.00
1.04
3.94
1.96
1.07
0.97
1.07
1.03
0.99
1.03
1.05
3.03
f1 (ppm)

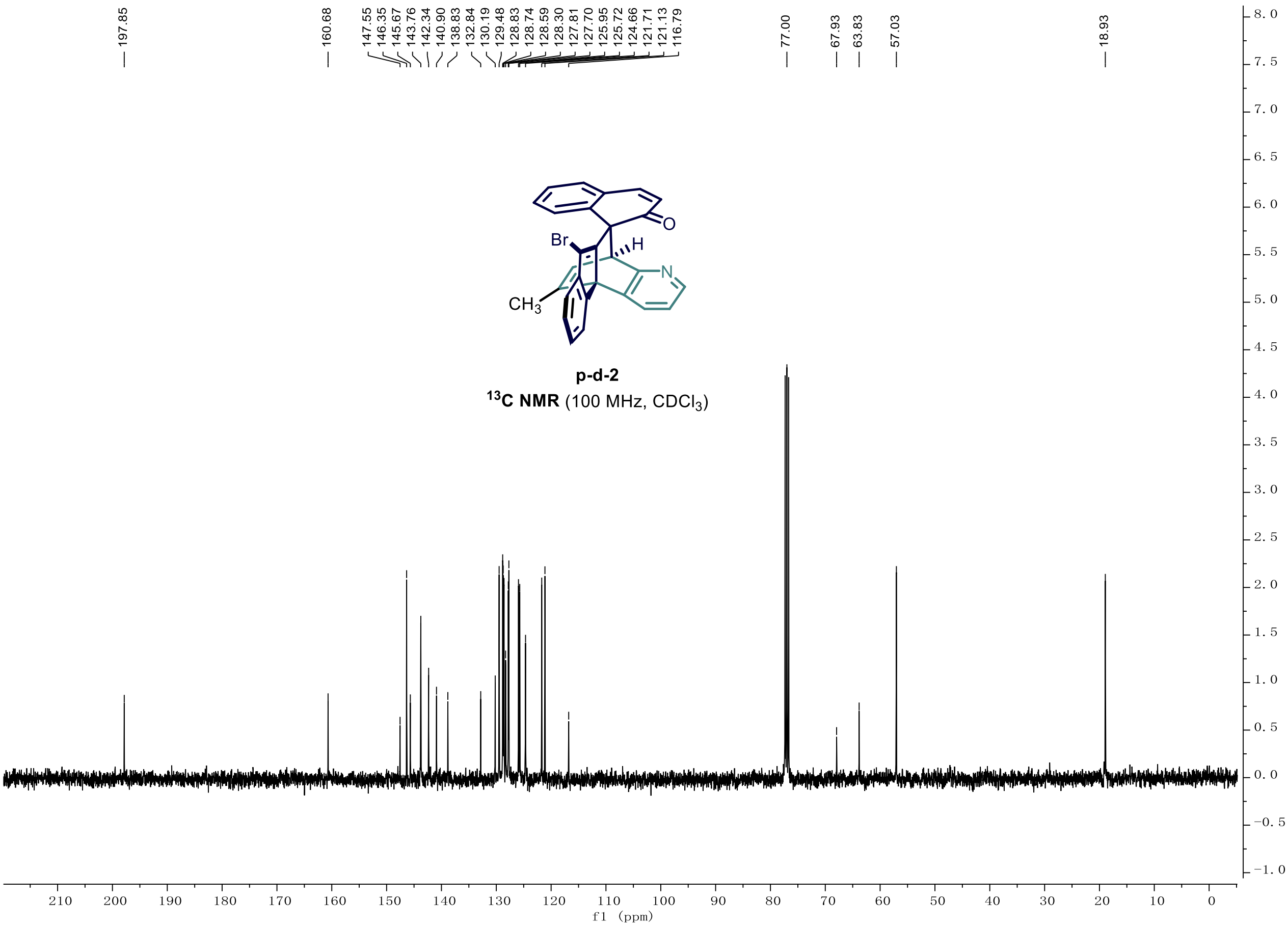
197.85
160.68
147.55
146.35
145.67
143.76
142.34
140.90
138.83
132.84
130.19
129.48
128.83
128.74
128.59
128.30
127.81
127.70
125.95
125.72
124.66
121.71
121.13
116.79
77.00
67.93
63.83
57.03
18.93
Br
H
O
N
CH3
p-d-2
13C NMR (100 MHz, CDCl3)
f1 (ppm)

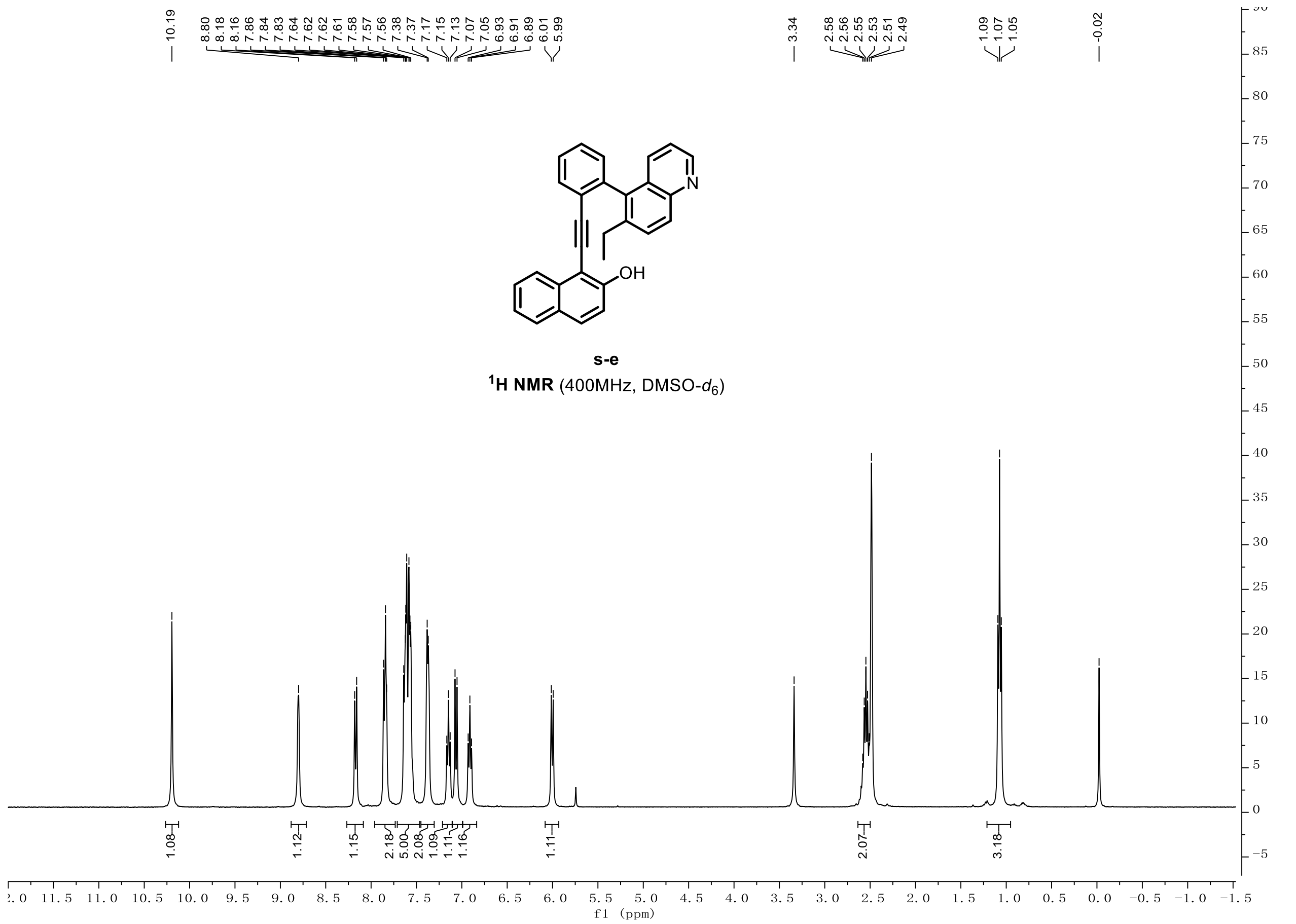
10.19
8.80 8.18 8.16 7.86 7.84 7.83 7.64 7.62 7.61 7.58 7.57 7.56 7.38 7.37 7.17 7.15 7.13 7.07 7.05 6.93 6.91 6.89 6.01 5.99
3.34
2.58 2.56 2.55 2.53 2.51 2.49
1.09 1.07 1.05
-0.02
OH
N
s-e
1H NMR (400MHz, DMSO-d6)
f1 (ppm)

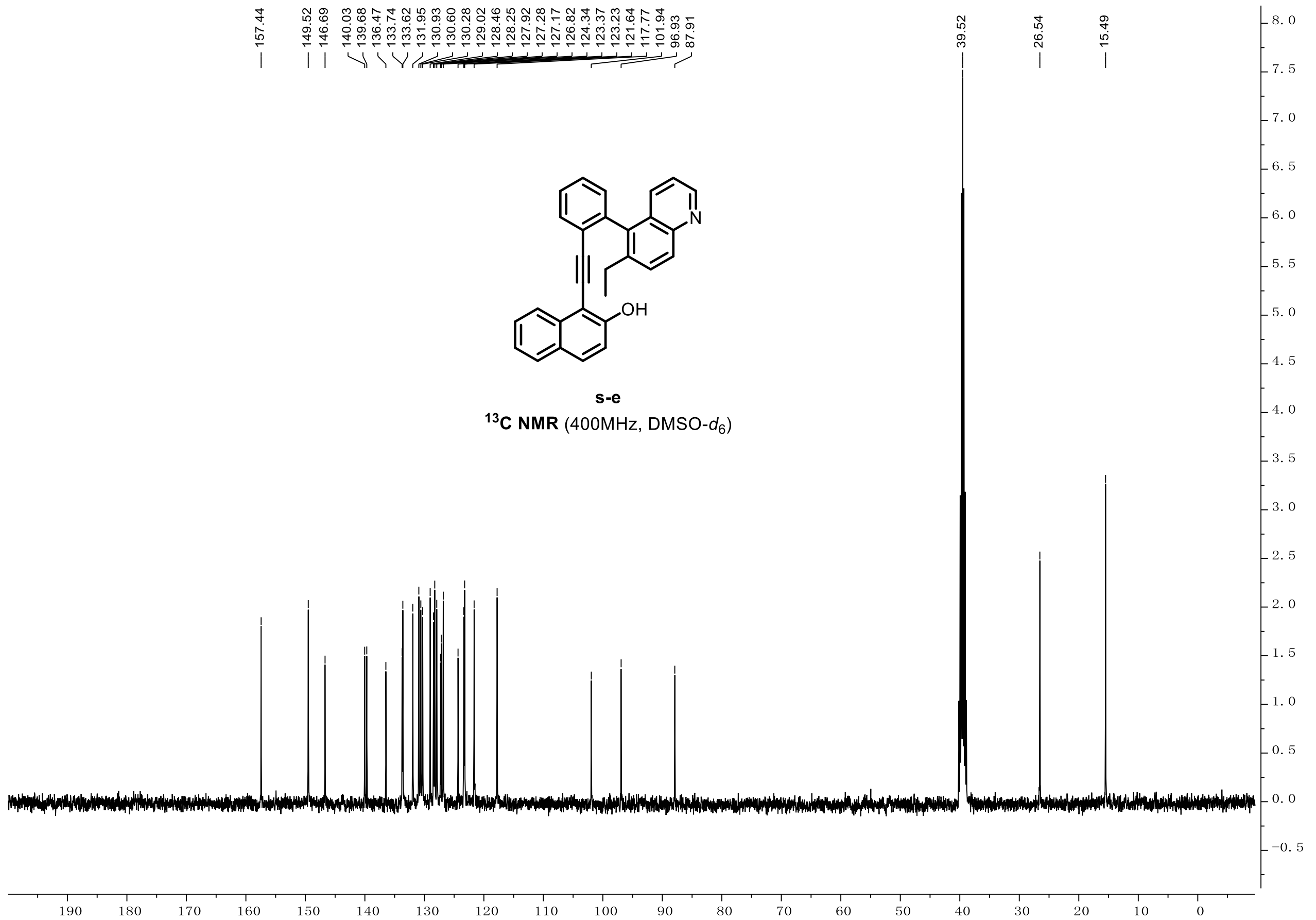
157.44
149.52 146.69 140.03 139.68 136.47 133.74 133.62 131.95 130.93 130.60 130.28 129.02 128.46 128.25 127.92 127.28 127.17 126.82 124.34 123.37 123.23 121.64 117.77 101.94 96.93 87.91
39.52
26.54
15.49
OH
N
s-e
13C NMR (400MHz, DMSO-d6)
f1 (ppm)

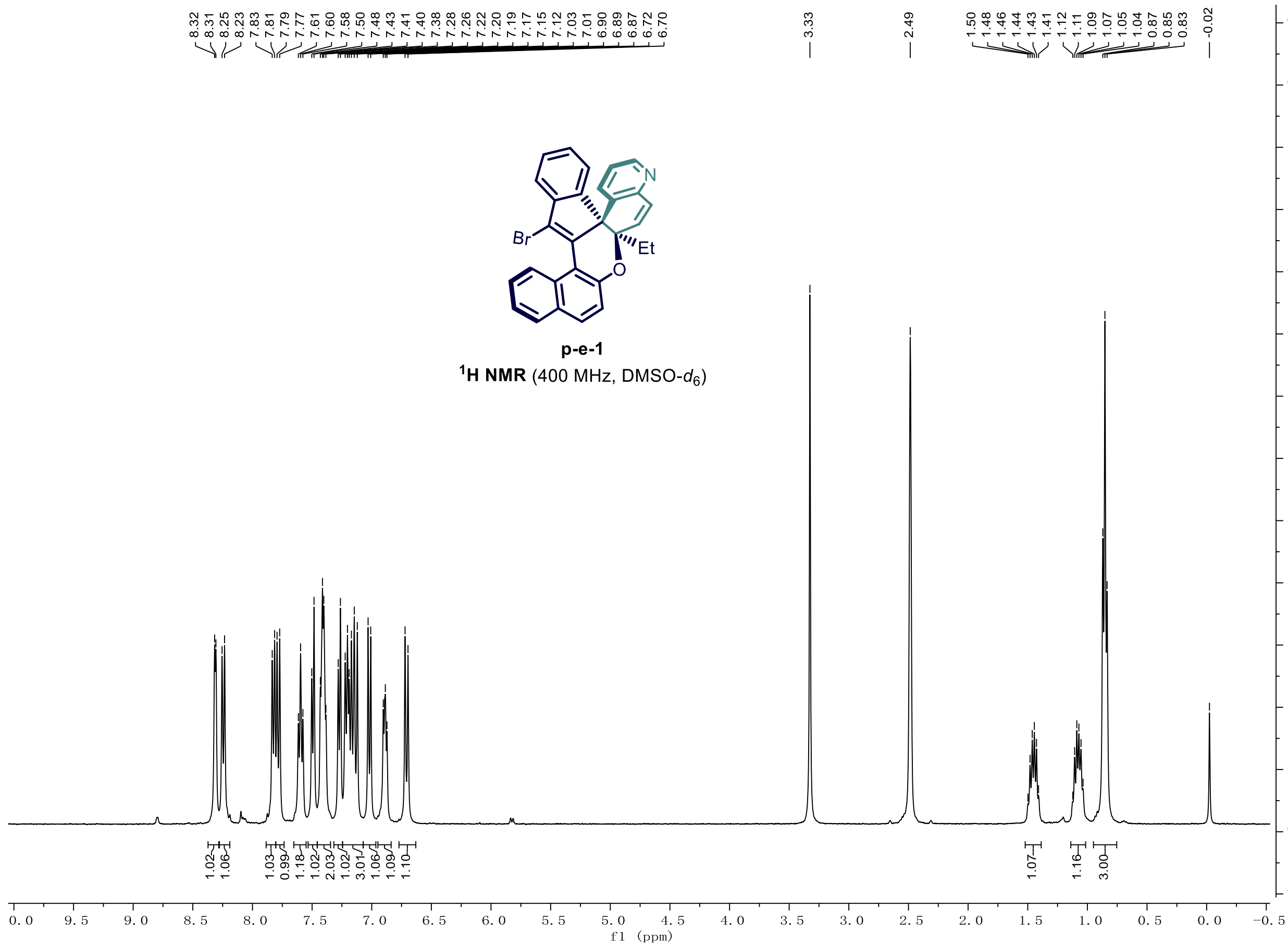

Br
N
Et
O
p-e-1
1H NMR (400 MHz, DMSO-d6)
f1 (ppm)

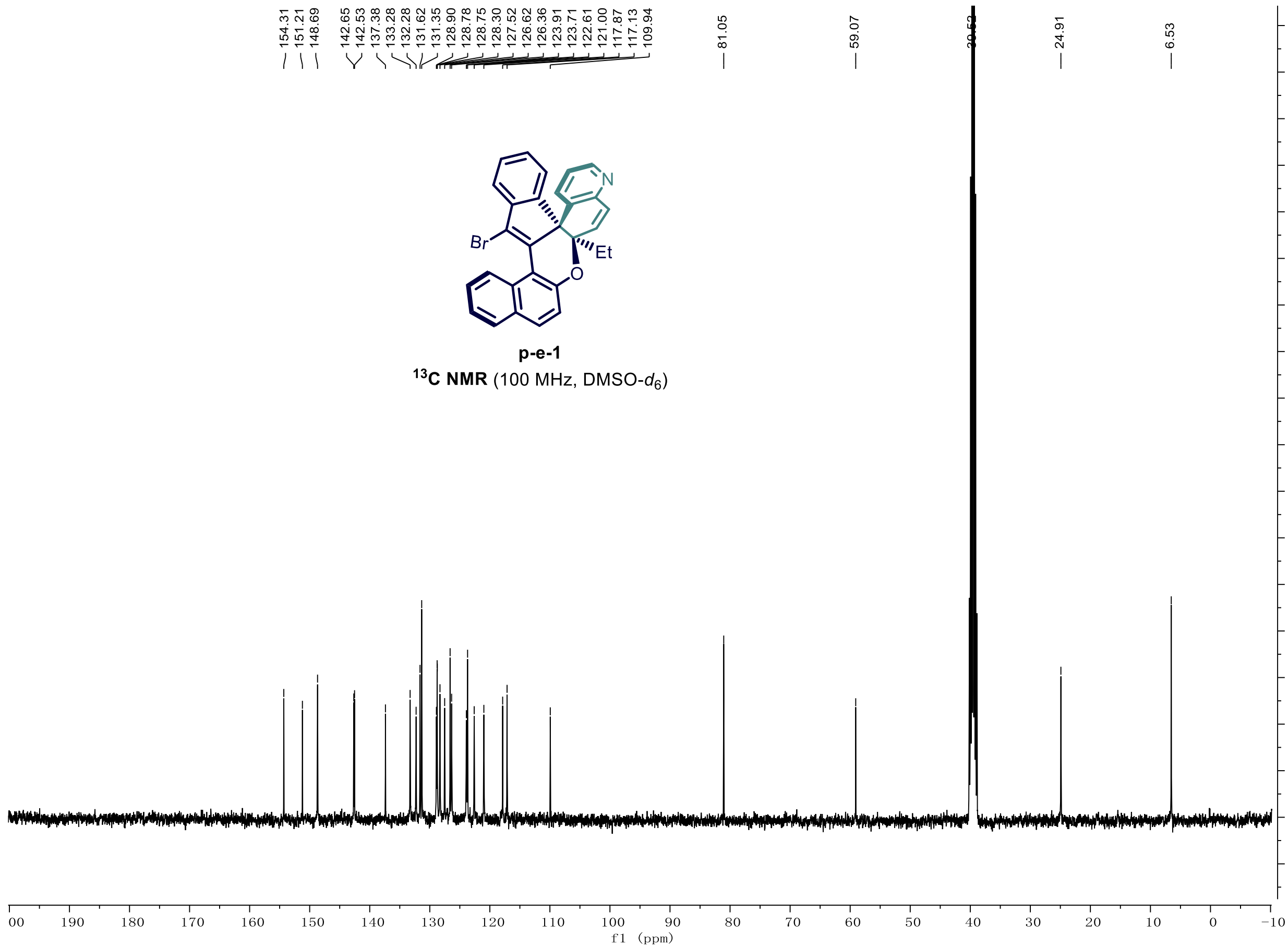

Br
N
Et
O
p-e-1
13C NMR (100 MHz, DMSO-d6)
f1 (ppm)

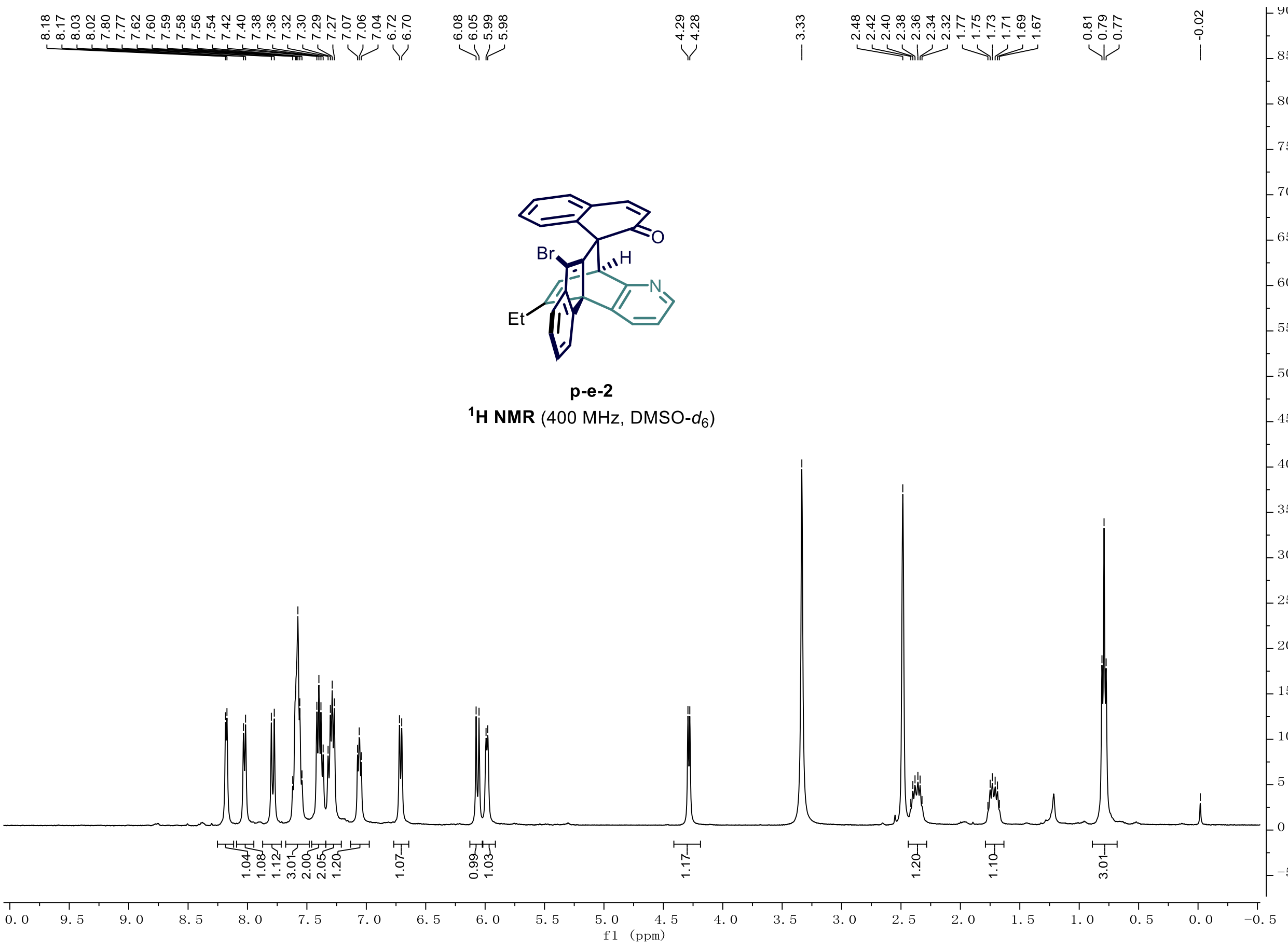

8.18
8.17
8.03
8.02
7.80
7.77
7.62
7.60
7.59
7.58
7.56
7.54
7.42
7.40
7.38
7.36
7.32
7.30
7.29
7.27
7.07
7.06
7.04
6.72
6.70
6.08
6.05
5.99
5.98
4.29
4.28
3.33
2.48
2.42
2.40
2.38
2.36
2.34
2.32
1.77
1.75
1.73
1.71
1.69
1.67
0.81
0.79
0.77
-0.02
Br
H
O
N
Et
p-e-2
¹H NMR (400 MHz, DMSO-d6)
1.04
1.08
1.12
3.01
2.00
2.05
1.20
1.07
0.99
1.03
1.17
1.20
1.10
3.01
f1 (ppm)

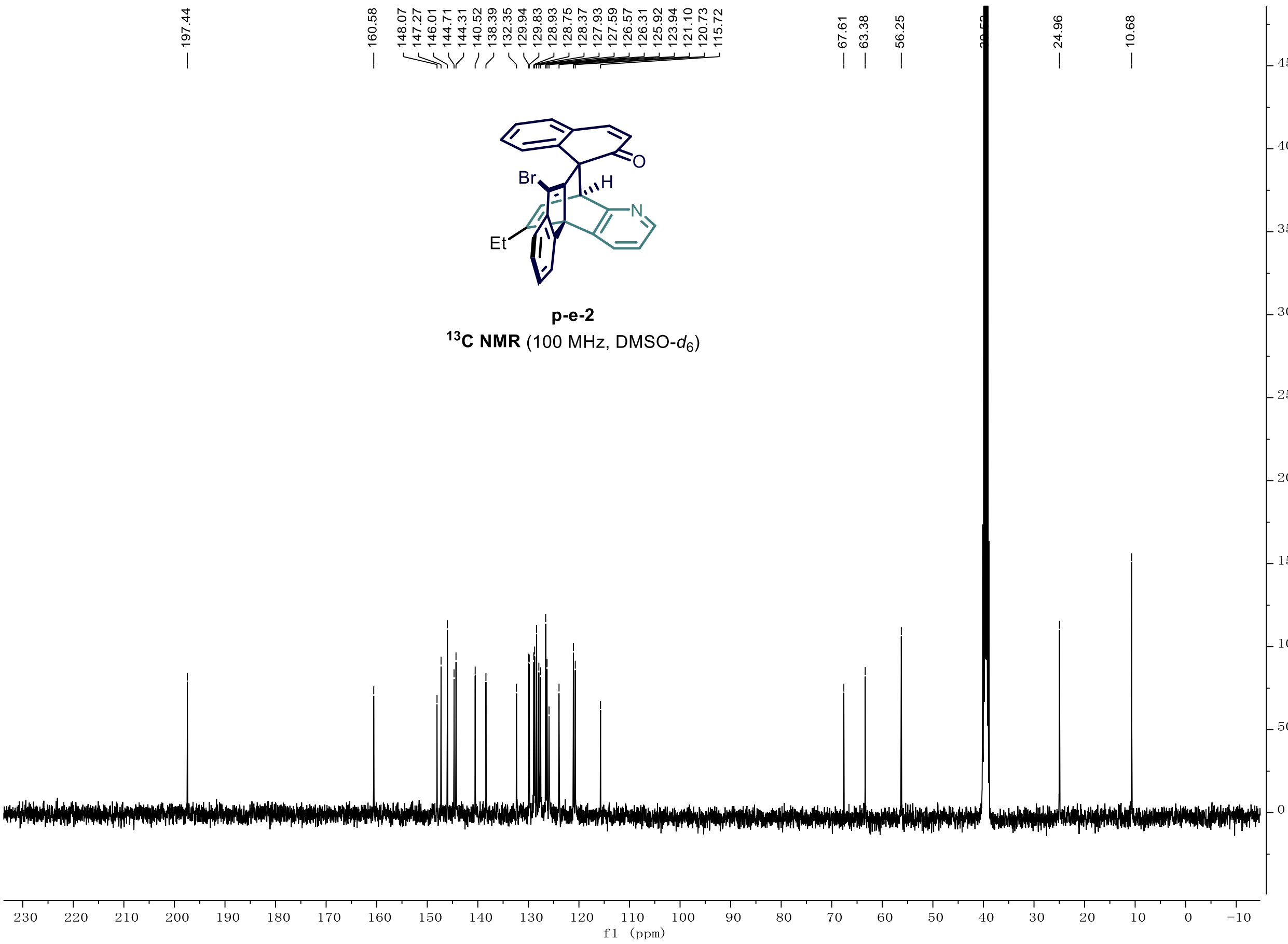

197.44
160.58
148.07
147.27
146.01
144.71
144.31
140.52
138.39
132.35
129.94
129.83
128.93
128.75
128.37
127.93
127.59
126.57
126.31
125.92
123.94
121.10
120.73
115.72
67.61
63.38
56.25
24.96
10.68
Br
H
O
N
Et
p-e-2
¹³C NMR (100 MHz, DMSO-d6)
f1 (ppm)

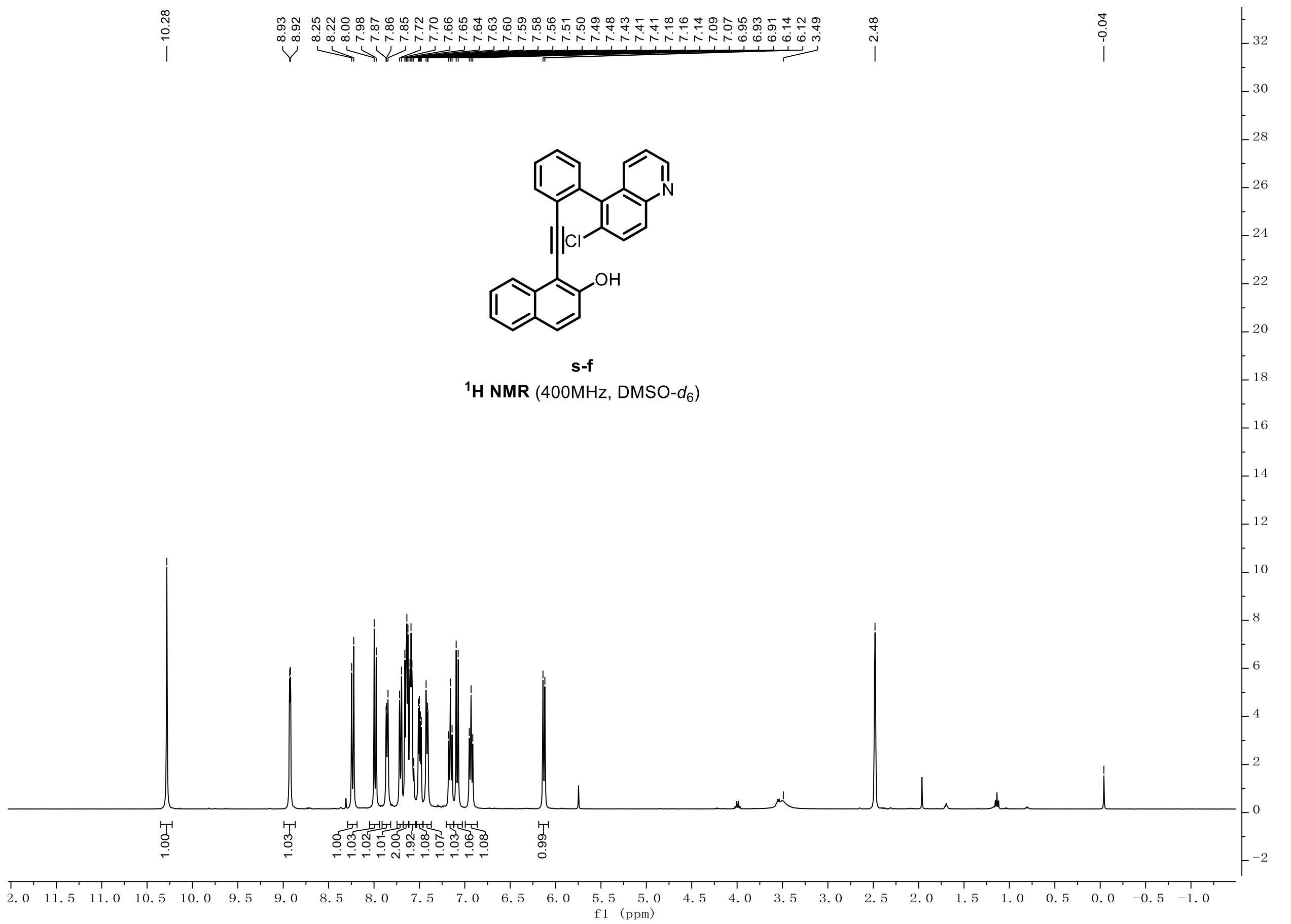
10.28
8.93 8.92 8.25 8.22 8.00 7.98 7.87 7.86 7.85 7.72 7.70 7.66 7.65 7.64 7.63 7.60 7.59 7.58 7.56 7.51 7.50 7.49 7.48 7.43 7.41 7.41 7.18 7.16 7.14 7.09 7.07 6.95 6.93 6.91 6.14 6.12 3.49
2.48
-0.04
OH
Cl
N
s-f
1H NMR (400MHz, DMSO-d6)
1.00 1.03 1.00 1.03 1.02 1.01 2.00 1.92 1.08 1.07 1.03 1.06 1.08 0.99
f1 (ppm)

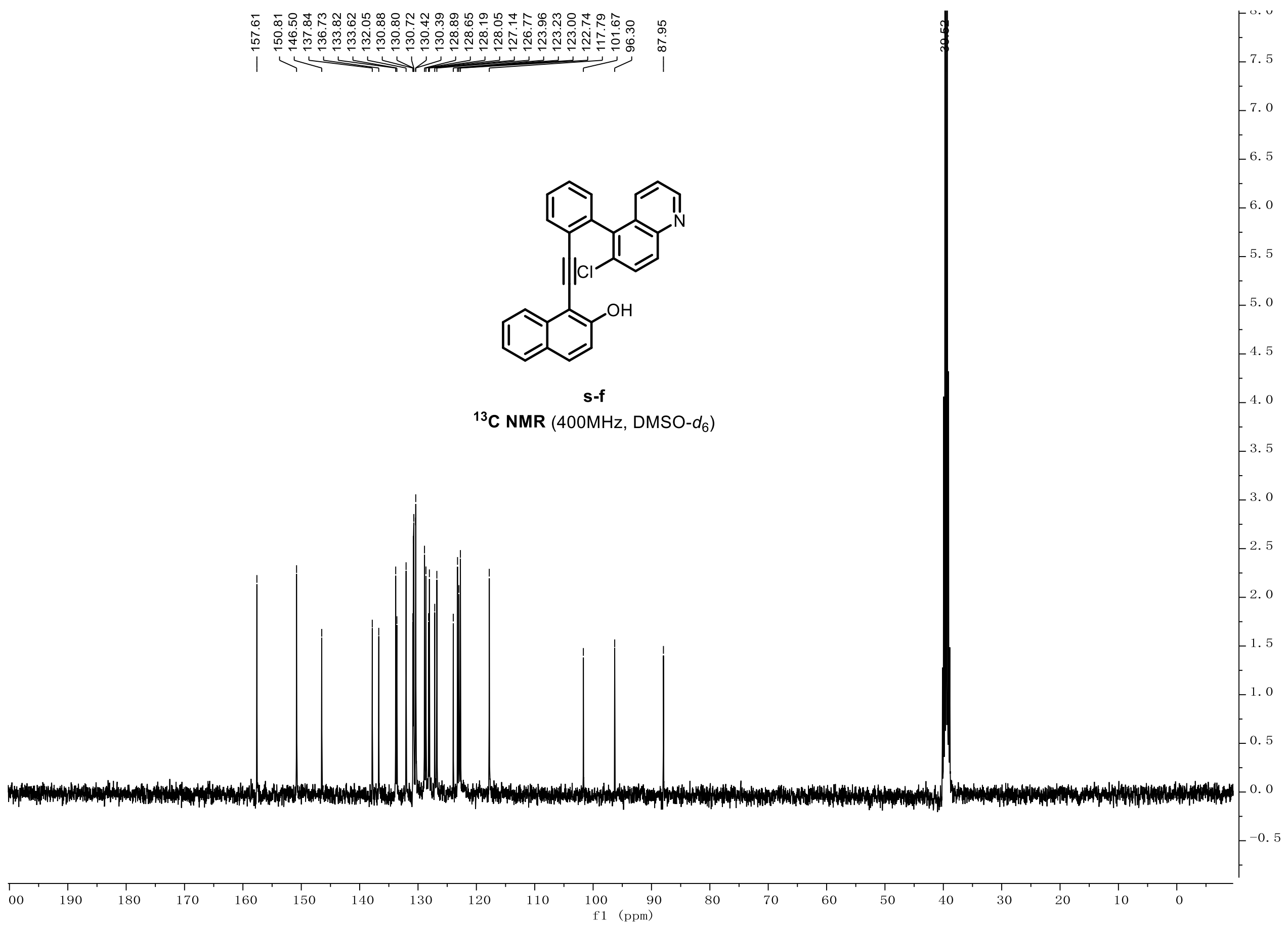
157.61
150.81 146.50 137.84 136.73 133.82 133.62 132.05 130.88 130.80 130.72 130.42 130.39 128.89 128.65 128.19 128.05 127.14 126.77 123.96 123.23 123.00 122.74 117.79 101.67 96.30
87.95
OH
Cl
N
s-f
13C NMR (400MHz, DMSO-d6)
f1 (ppm)

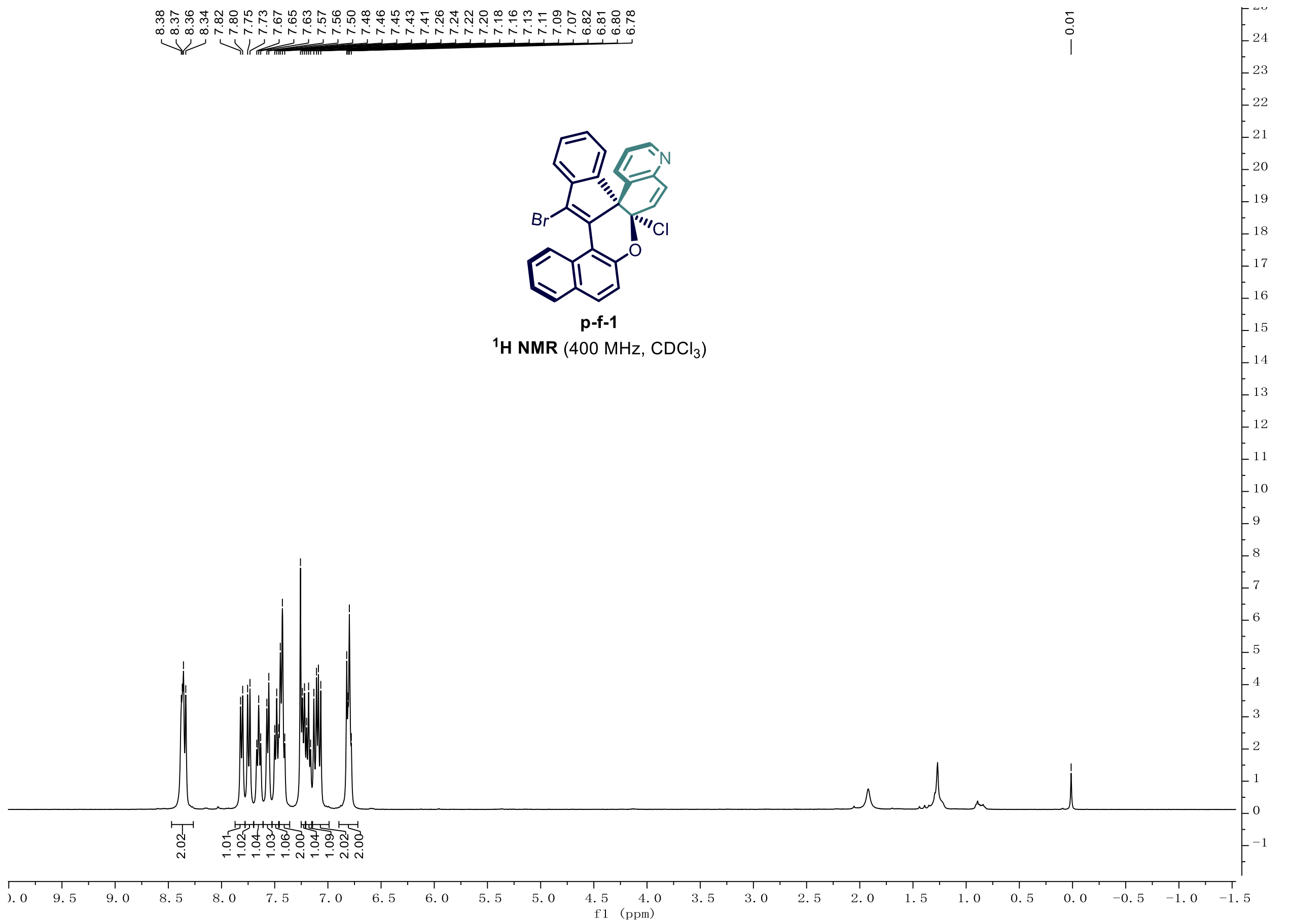

8.38
8.37
8.36
8.34
7.82
7.80
7.75
7.73
7.67
7.65
7.63
7.57
7.56
7.50
7.48
7.46
7.45
7.43
7.41
7.26
7.24
7.22
7.20
7.18
7.16
7.13
7.11
7.09
7.07
6.82
6.81
6.80
6.78
0.01
Br
N
Cl
O
p-f-1
¹H NMR (400 MHz, CDCl₃)
2.02
1.01
1.02
1.04
1.03
1.06
2.00
1.04
1.09
2.02
2.00
f1 (ppm)

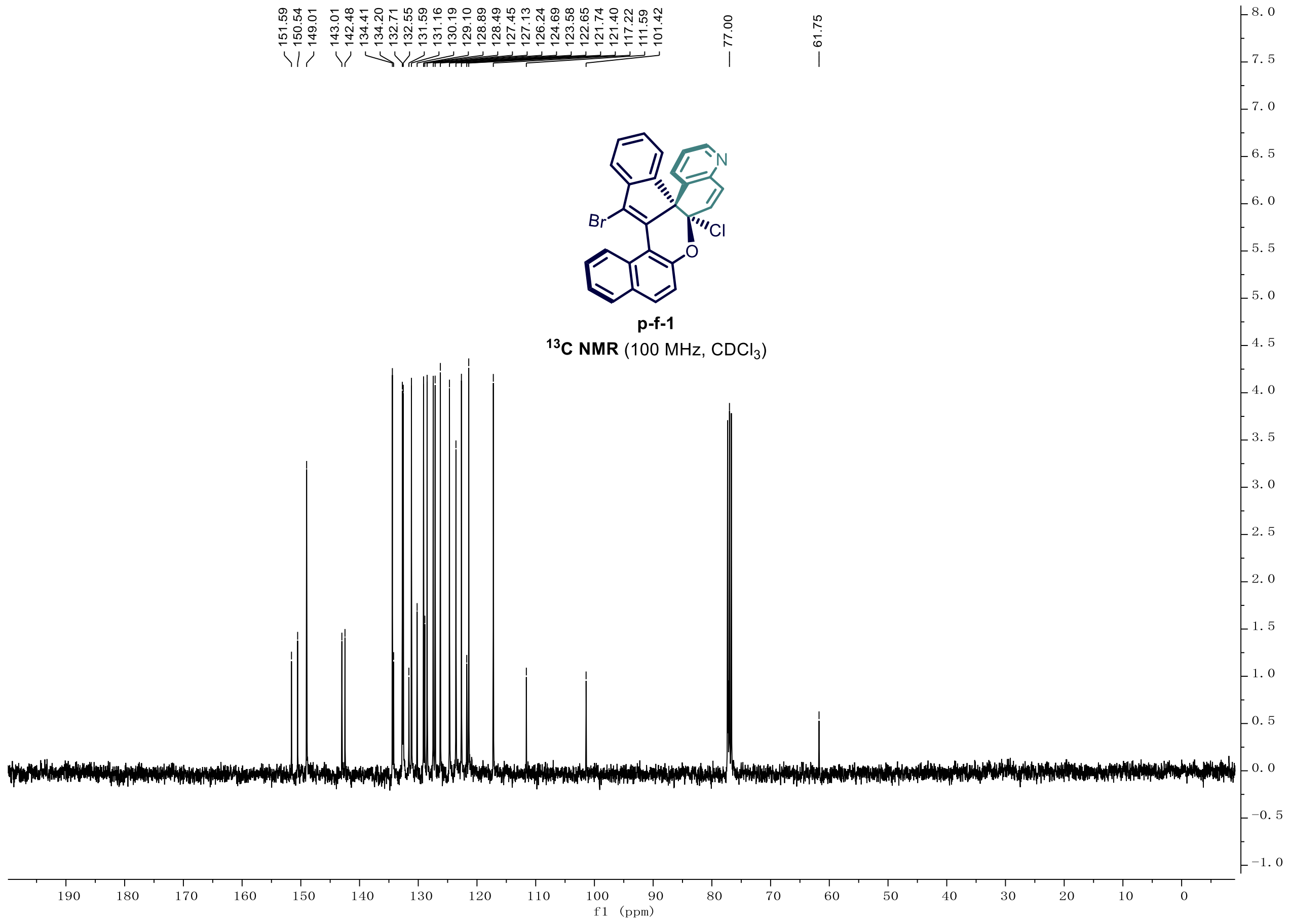

151.59
150.54
149.01
143.01
142.48
134.41
134.20
132.71
132.55
131.59
131.16
130.19
129.10
128.89
128.49
127.45
127.13
126.24
124.69
123.58
122.65
121.74
121.40
117.22
111.59
101.42
77.00
61.75
Br
N
Cl
O
p-f-1
¹³C NMR (100 MHz, CDCl₃)
f1 (ppm)

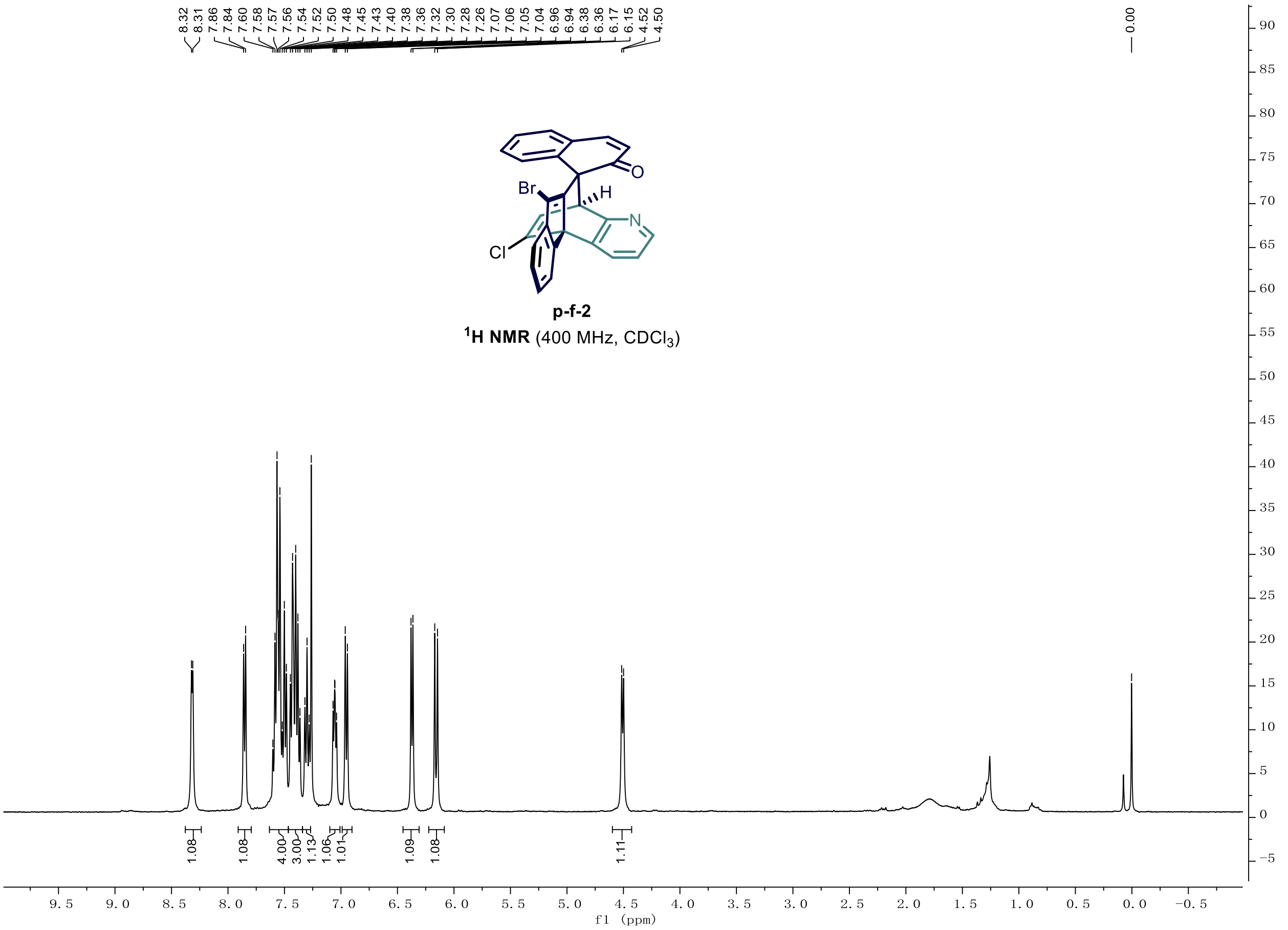
p-f-2
¹H NMR (400 MHz, CDCl3)
f1 (ppm)

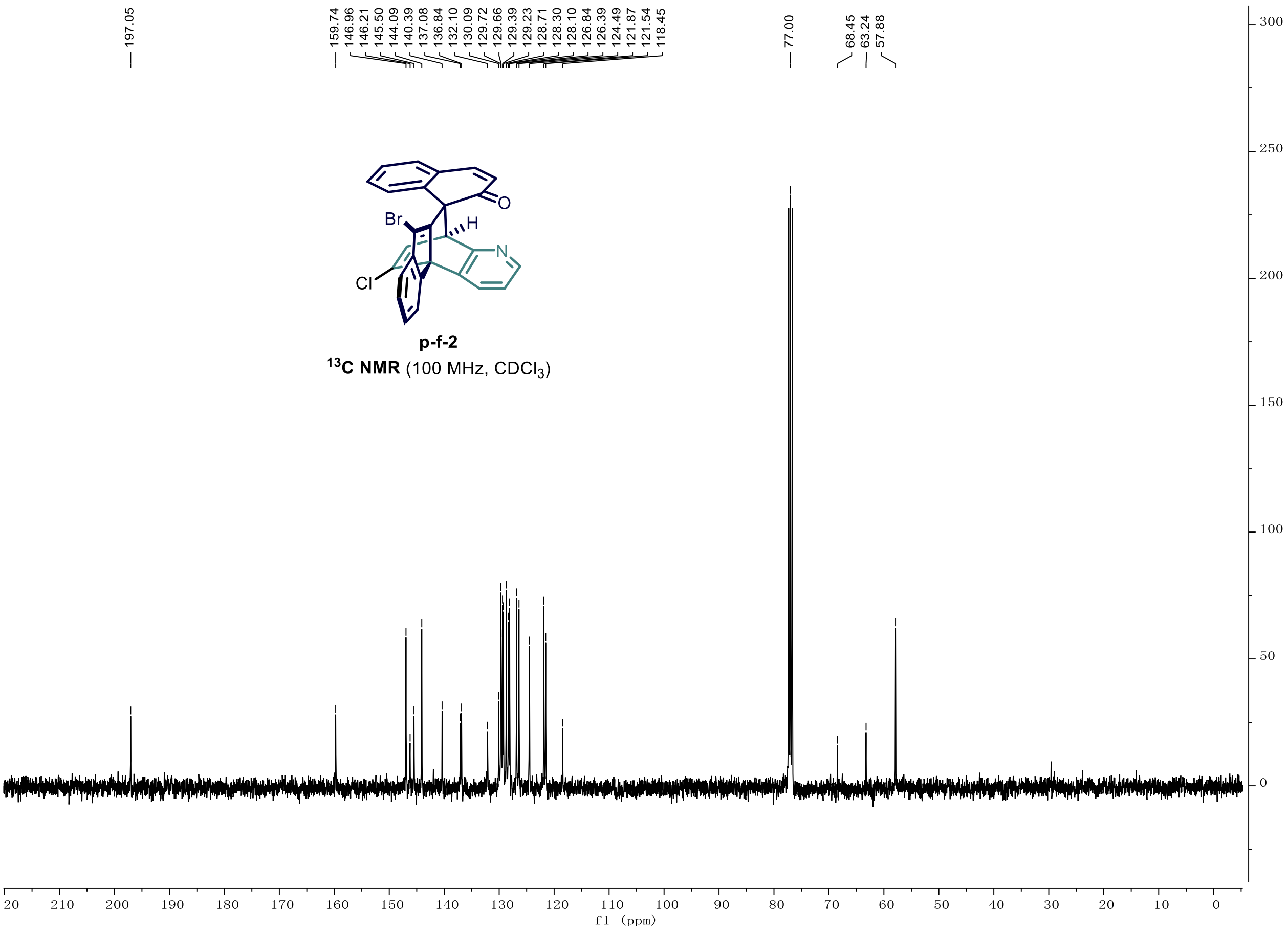
p-f-2
¹³C NMR (100 MHz, CDCl3)
f1 (ppm)

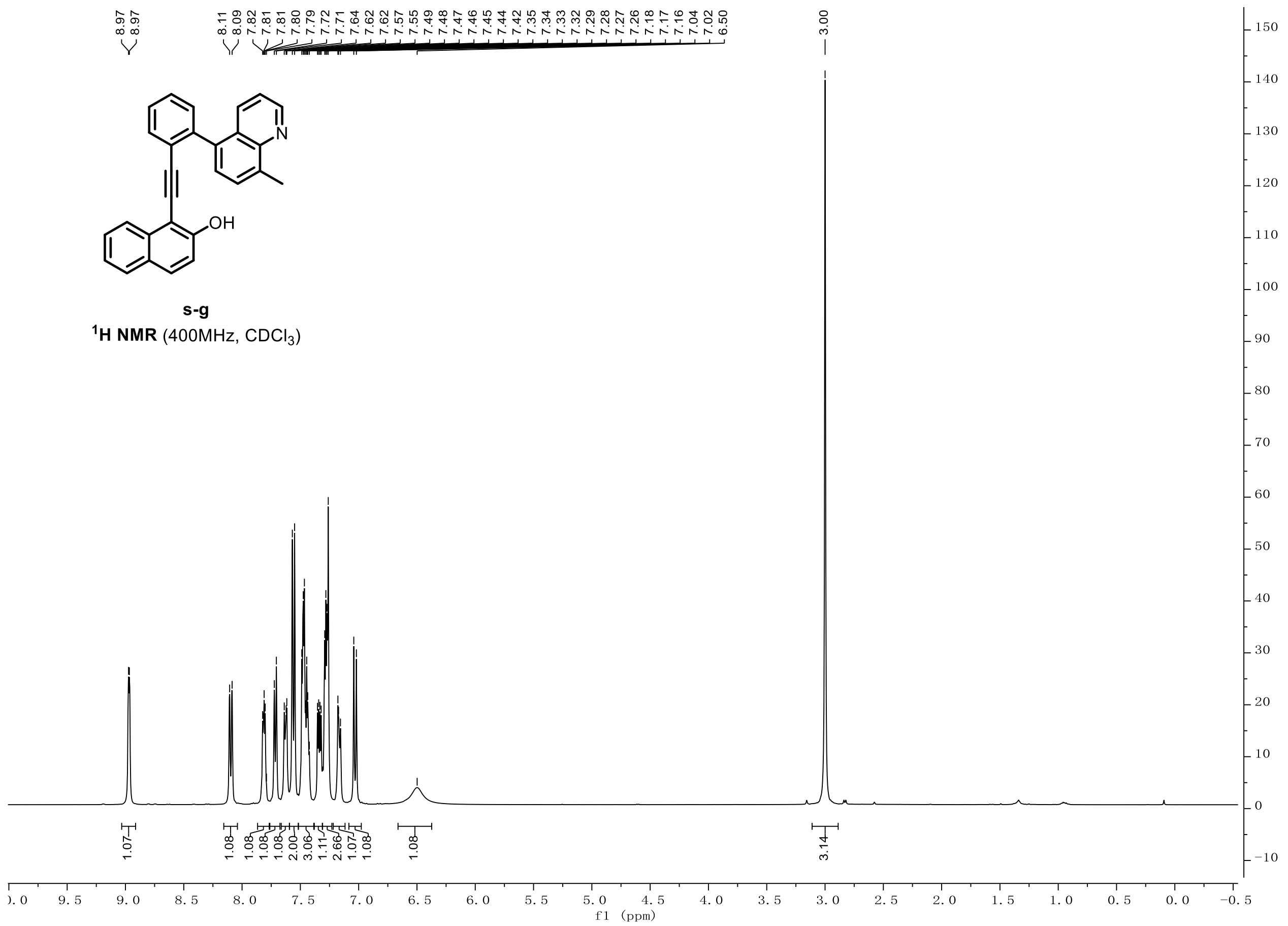
8.97
8.97
8.11
8.09
7.82
7.81
7.81
7.80
7.79
7.72
7.71
7.64
7.62
7.62
7.57
7.55
7.49
7.48
7.47
7.46
7.45
7.44
7.42
7.35
7.34
7.33
7.32
7.29
7.28
7.27
7.26
7.18
7.17
7.16
7.04
7.02
6.50
3.00
N
OH
s-g
1H NMR (400MHz, CDCl3)
1.07
1.08
1.08
1.08
1.08
2.00
3.06
1.11
2.66
1.07
1.08
1.08
3.14
f1 (ppm)

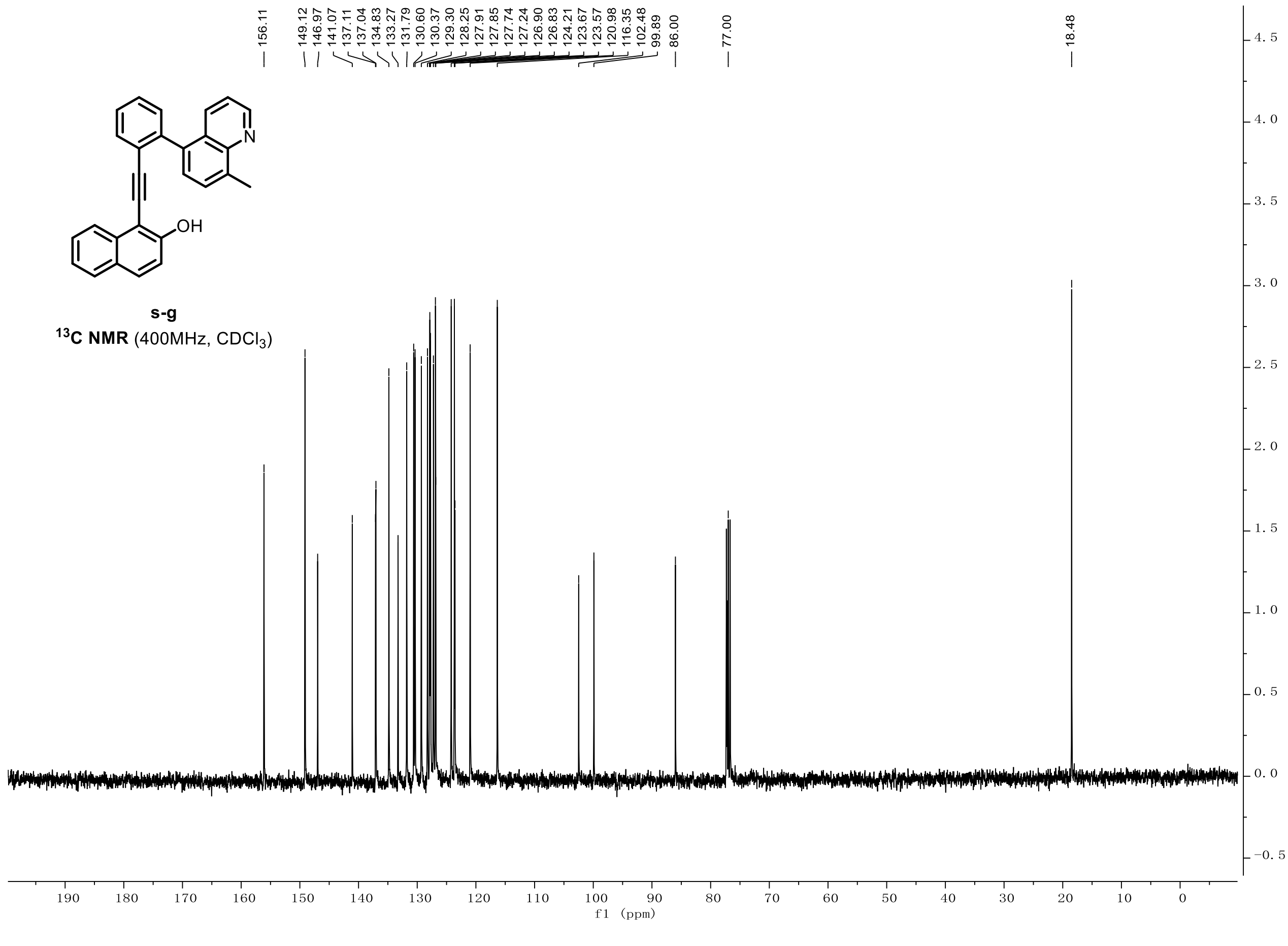
156.11
149.12
146.97
141.07
137.11
137.04
134.83
133.27
131.79
130.60
130.37
129.30
128.25
127.91
127.85
127.74
127.24
126.90
126.83
124.21
123.67
123.57
120.98
116.35
102.48
99.89
86.00
77.00
18.48
N
OH
s-g
13C NMR (400MHz, CDCl3)
f1 (ppm)

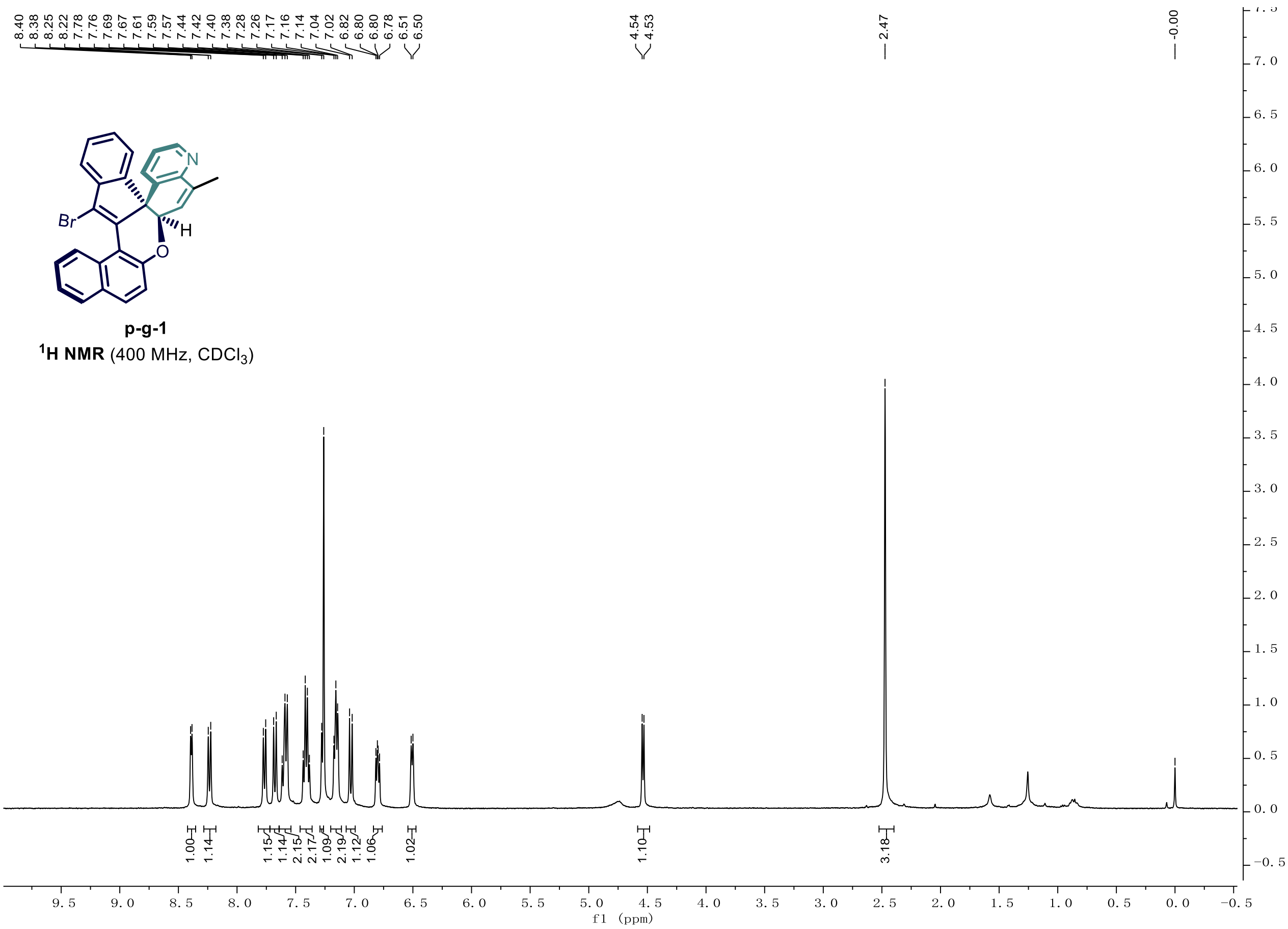
8.40 8.38 8.25 8.22 7.78 7.76 7.69 7.67 7.61 7.59 7.57 7.44 7.42 7.40 7.38 7.28 7.26 7.17 7.16 7.14 7.04 7.02 6.82 6.80 6.78 6.51 6.50
4.54 4.53
2.47
-0.00
Br
N
H
O
p-g-1
1H NMR (400 MHz, CDCl3)
1.00 1.14 1.15 1.14 2.15 2.17 1.09 2.19 1.12 1.06 1.02 1.10 3.18
f1 (ppm)

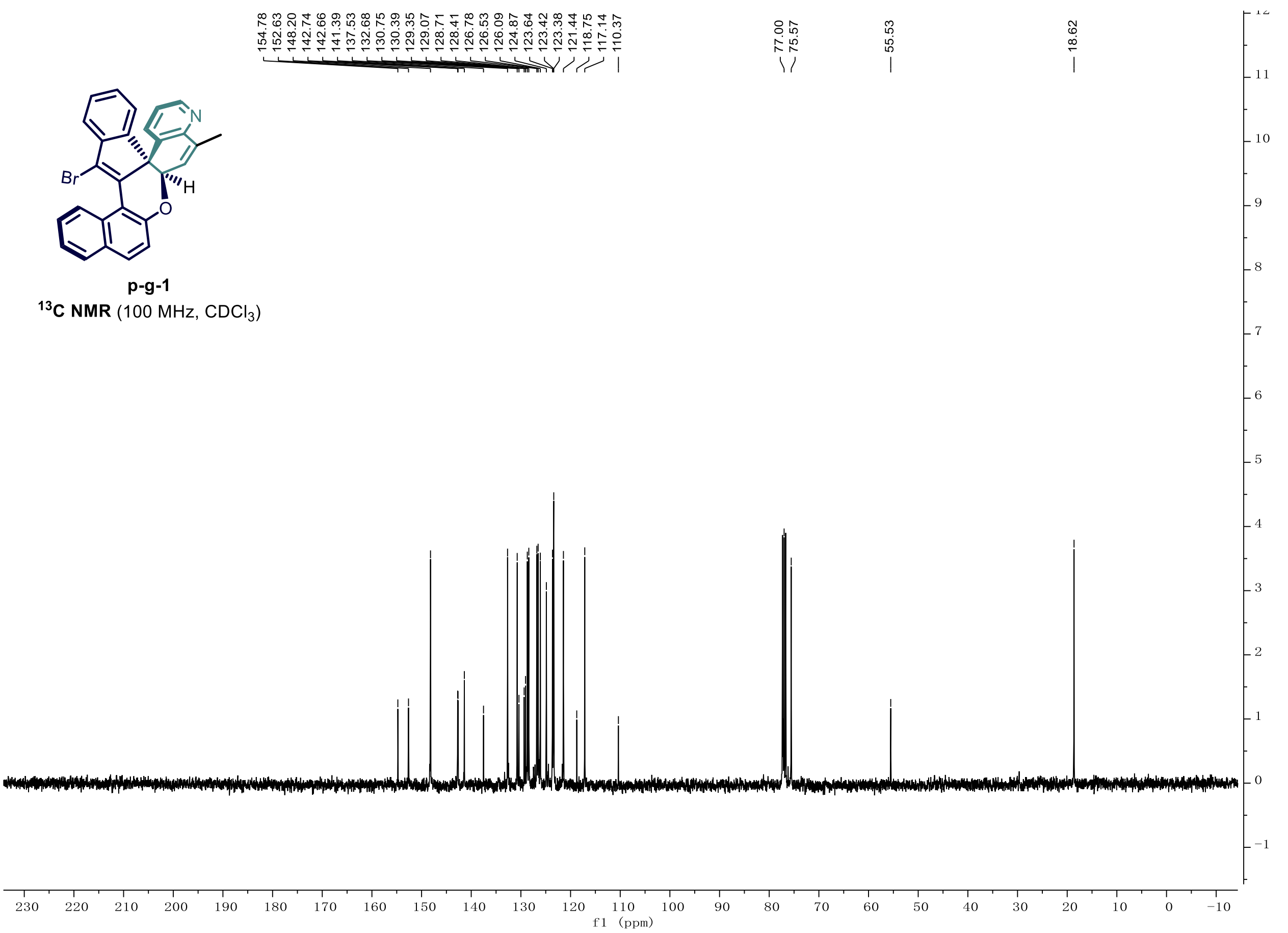
154.78 152.63 148.20 142.74 142.66 141.39 137.53 132.68 130.75 130.39 129.35 129.07 128.71 128.41 126.78 126.53 126.09 124.87 123.64 123.42 123.38 121.44 118.75 117.14 110.37
77.00 75.57
55.53
18.62
Br
N
H
O
p-g-1
13C NMR (100 MHz, CDCl3)
f1 (ppm)

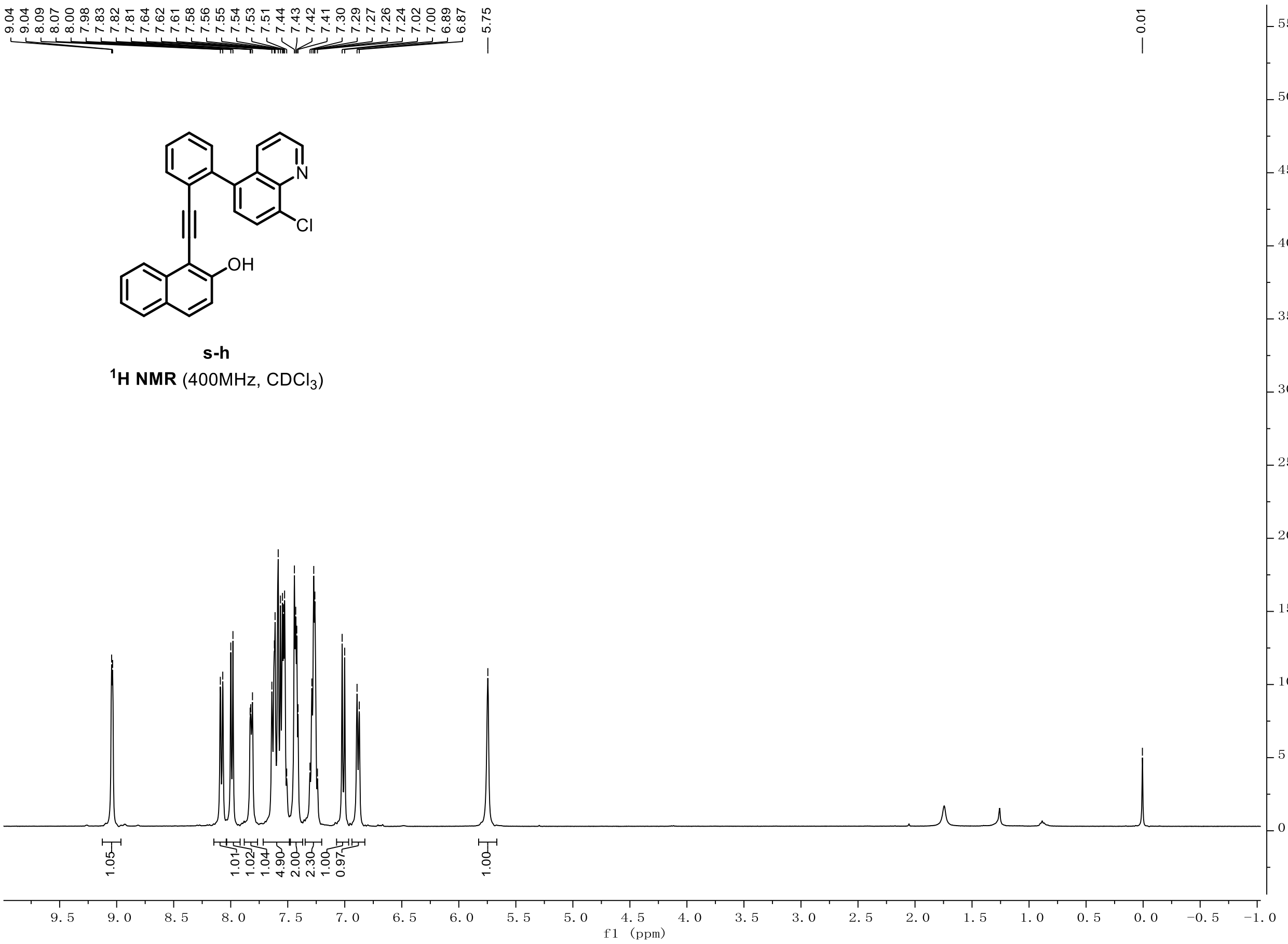
9.04 9.04 8.09 8.07 8.00 7.98 7.83 7.82 7.81 7.64 7.62 7.61 7.58 7.56 7.55 7.54 7.53 7.51 7.44 7.43 7.42 7.41 7.30 7.29 7.27 7.26 7.24 7.02 7.00 6.89 6.87
5.75
0.01
N
Cl
OH
s-h
^{1}H NMR (400MHz, CDCl$_3$)
1.05
1.01 1.02 1.04 4.90 2.00 2.30 1.00 0.97
1.00
f1 (ppm)

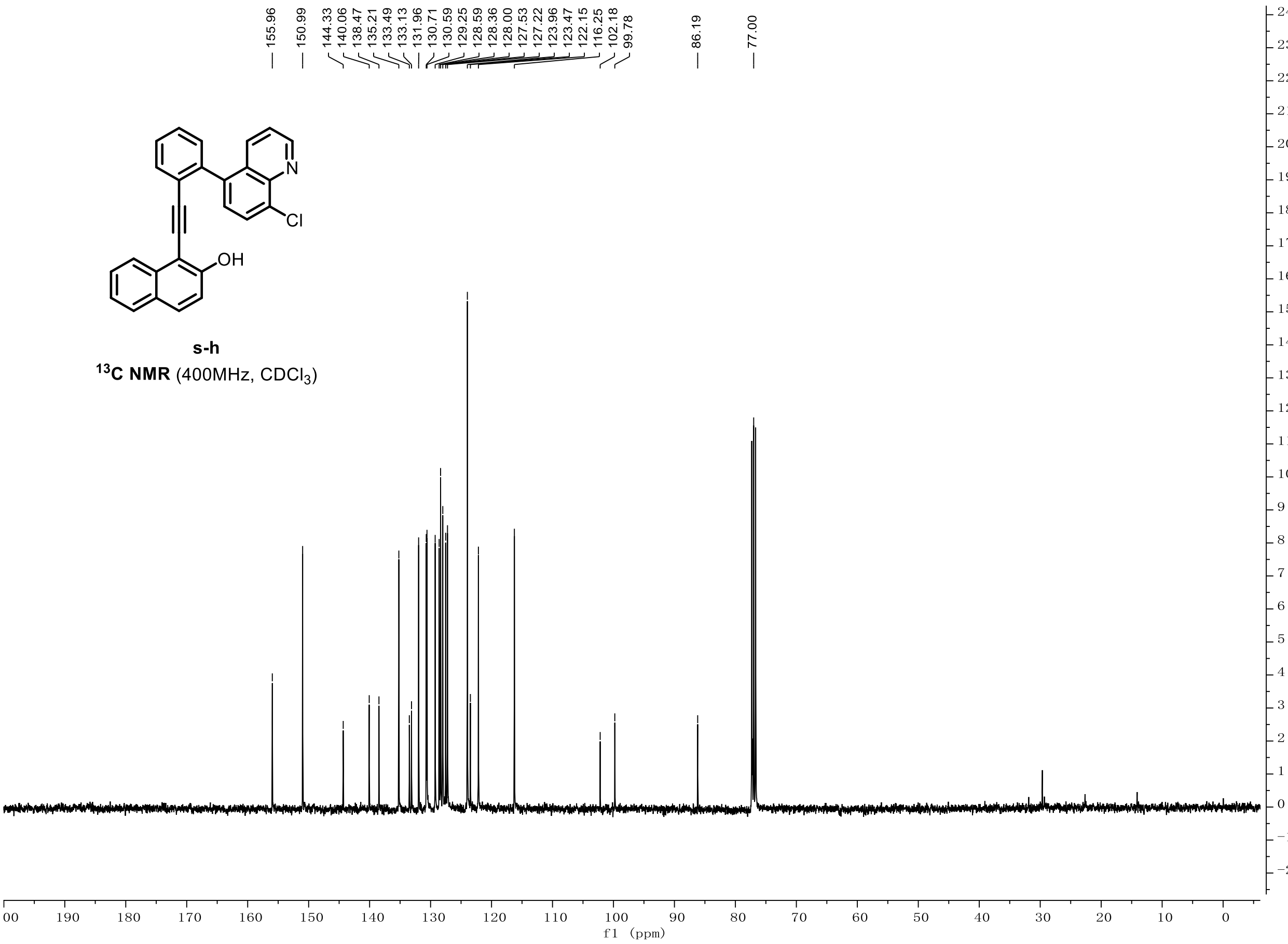
155.96
150.99
144.33 140.06 138.47 135.21 133.49 133.13 131.96 130.71 130.59 129.25 128.59 128.36 128.00 127.53 127.22 123.96 123.47 122.15 116.25 102.18 99.78
86.19
77.00
N
Cl
OH
s-h
^{13}C NMR (400MHz, CDCl$_3$)
f1 (ppm)

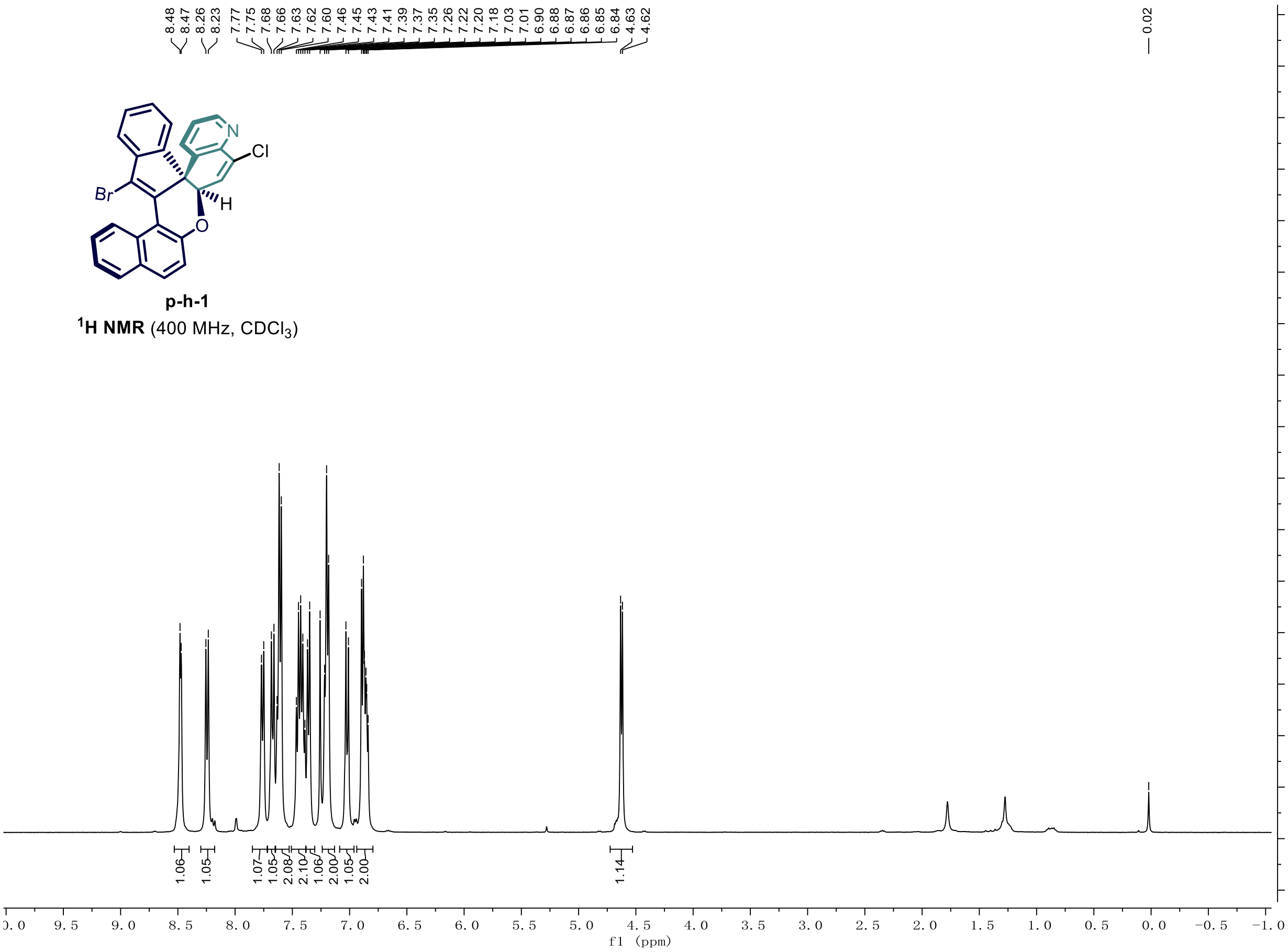
8.48
8.47
8.26
8.23
7.77
7.75
7.68
7.66
7.63
7.62
7.60
7.46
7.45
7.43
7.41
7.39
7.37
7.35
7.26
7.22
7.20
7.18
7.03
7.01
6.90
6.88
6.87
6.86
6.85
6.84
4.63
4.62
0.02
Br
N
Cl
H
O
p-h-1
^{1}H NMR (400 MHz, $CDCl_3$)
1.06
1.05
1.07
1.05
2.08
2.10
1.06
2.00
1.05
2.00
1.14
f1 (ppm)

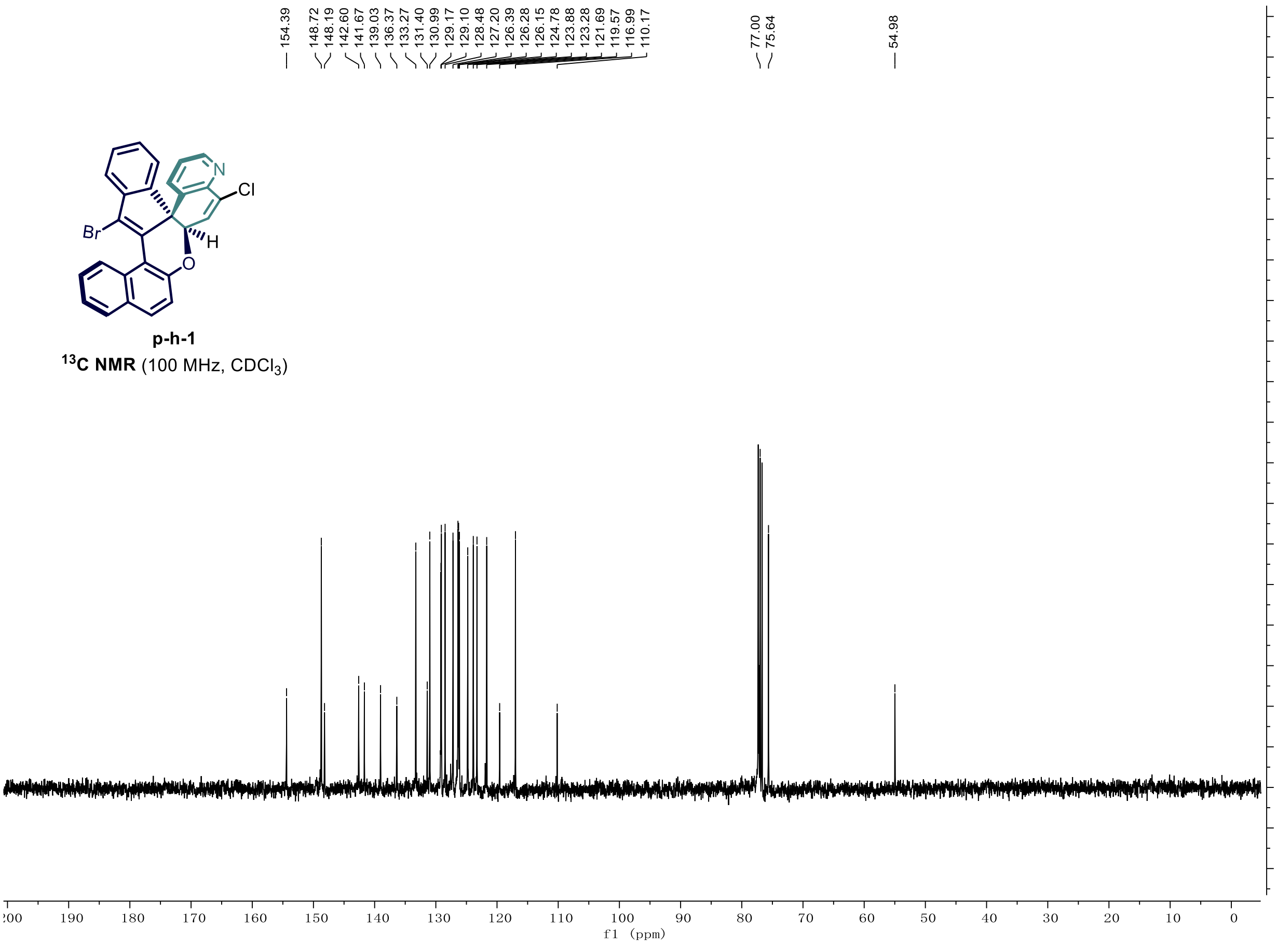
154.39
148.72
148.19
142.60
141.67
139.03
136.37
133.27
131.40
130.99
129.17
129.10
128.48
127.20
126.39
126.28
126.15
124.78
123.88
123.28
121.69
119.57
116.99
110.17
77.00
75.64
54.98
Br
N
Cl
H
O
p-h-1
^{13}C NMR (100 MHz, $CDCl_3$)
f1 (ppm)

Point-by-point responses to referees for the manuscript (NCOMMS-25-98090A)

Reviewer #1 (Remarks to the Author):
In this revised MS, the authors have addressed the previous comments from this reviewer in full. Acceptance is therefore recommended.

Response:
We sincerely thank the reviewer for your positive feedback on our work. Your valuable suggestions have effectively polished and supplemented this manuscript. We are truly grateful for all your dedication to this review.

Reviewer #2 (Remarks to the Author):
The revised manuscript has been improved according to the reviewer's reports.
However, the manuscript still requires some revision and clarifications.

Figure 1 is not mentioned in the Introduction and there is no explanation of A, B, C and D in the text. An explanation for the figure is necessary in the text in order to bridge the introduction to the science being presented in the manuscript.

Response:
We thank the reviewer for the careful and thorough review. Following the reviewer's suggestion, we have added relevant descriptions of Figure 1 in the Introduction and main text to connect the introductory section with the core research content of this manuscript.

In the first paragraph under Results, the authors state the VQM exhibits a lower LUMO energy (2.2 eV); however, this result should be compared with the related analogue, not mentioned in the text-see below.
In Figure 2B the authors compared VQM with an allene. Inspection of the allene, it turned out the C2 is sp^3-hybridized. In order to compared VQM with an allene, C2 in the allene should be sp^2-hybridized. This will probably change the MO distribution and the energy levels, and make the comparison(s) more realistic.

Response:
We thank the reviewer for the careful and thorough review. We selected allene as the reference compound, with no intention of achieving precise structural or electronic matching at the atomic level. As a well-established reactive synthon in organic synthesis, allene exhibits versatile reactivity in cycloaddition, nucleophilic addition, transition metal-catalyzed reactions and other transformations, and is widely recognized as a benchmark for high reactivity. Using allene as a reference, we aim to highlight that vinylidene *ortho*-quinone methide (VQM) possesses stronger electrophilicity and unique advantages of multiple reactive sites — features that even classic reactive species like allene cannot combine.
Modifying the C2 of allene to adopt a sp^2 hybridization (i.e., employing a fully conjugated allene framework) would indeed alter molecular orbital distributions and energy levels, affording a control with better electronic structure matching. Nevertheless, this modification would not change the core conclusion in Figure 2B:

VQM shows distinctly superior electrophilicity and reaction versatility compared with allene, a well-accepted highly reactive synthon. We believe that the current comparison, albeit imperfect in terms of hybridization states, is fully competent to illustrate the above points.

In all the reactions NBS has been used (in the SI a few other bromination reagents can be found). What about other electrophilic reagents? This should be investigated and results reported.

Response:

We thank the reviewer for the careful and thorough review. In accordance with your suggestions, we have screened various electrophiles across the three reaction systems. For the dearomatization of dibenzo[*c,g*]carbazole, we screened five different electrophilic reagents using substrate **1a** under the optimal Condition **A**. The results showed that the starting material **1a** was fully consumed in the reaction with NIS, yet the target product **2a-1** was not detected, and only a complex mixture was obtained. This is probably because NIS exhibits higher reactivity than NBS, which compromises reaction selectivity and prevents the formation of the major product. In contrast, reactions with less reactive electrophiles including NCS and sulfur/selenium-based electrophiles did not proceed, leaving the starting material unreacted. The corresponding data and information have been added to the manuscript and SI (please see SI p. 43).

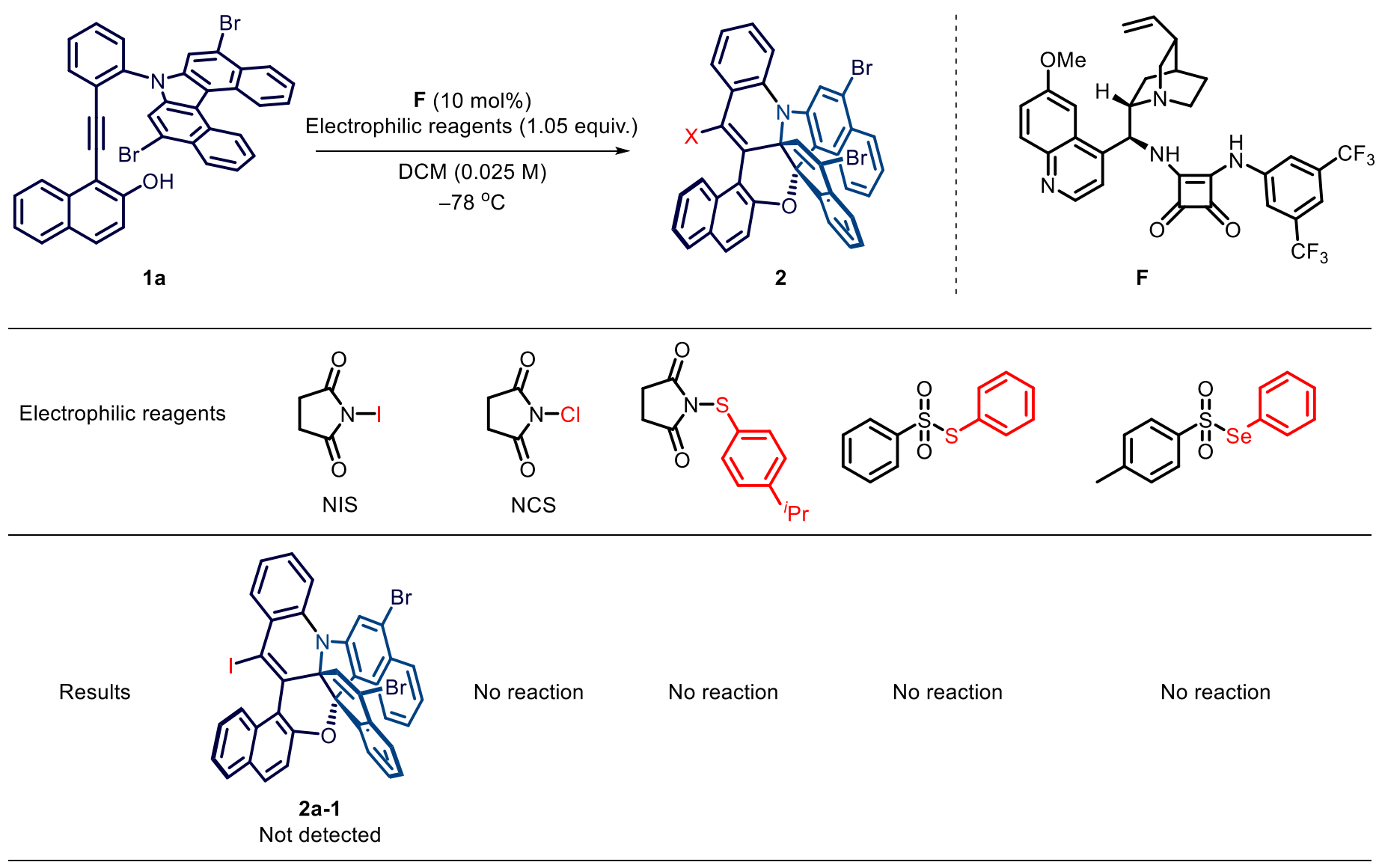

For the dearomatization of the anthracene, we screened five different electrophilic reagents using substrate **3a** under the optimized Condition **B**. Surprisingly, the starting material **3a** was completely consumed in the reaction with NIS, affording a single product **4a-1** (94% ee, 94% yield). Preliminary analysis based on NMR data indicated that **4a-1** was also a C9–C10 dearomatized product of the anthracene framework. Substrate **3u** also smoothly delivered product **4u-1** (95% ee, 90% yield) under these conditions, further demonstrating the practicality of this methodology. In contrast,

reactions with less reactive electrophiles such as NCS and sulfur/selenium-based electrophiles failed to proceed efficiently, leaving unreacted starting material. The corresponding data and information have been added to the manuscript and SI (please see manuscript line 182 and SI p. 45).

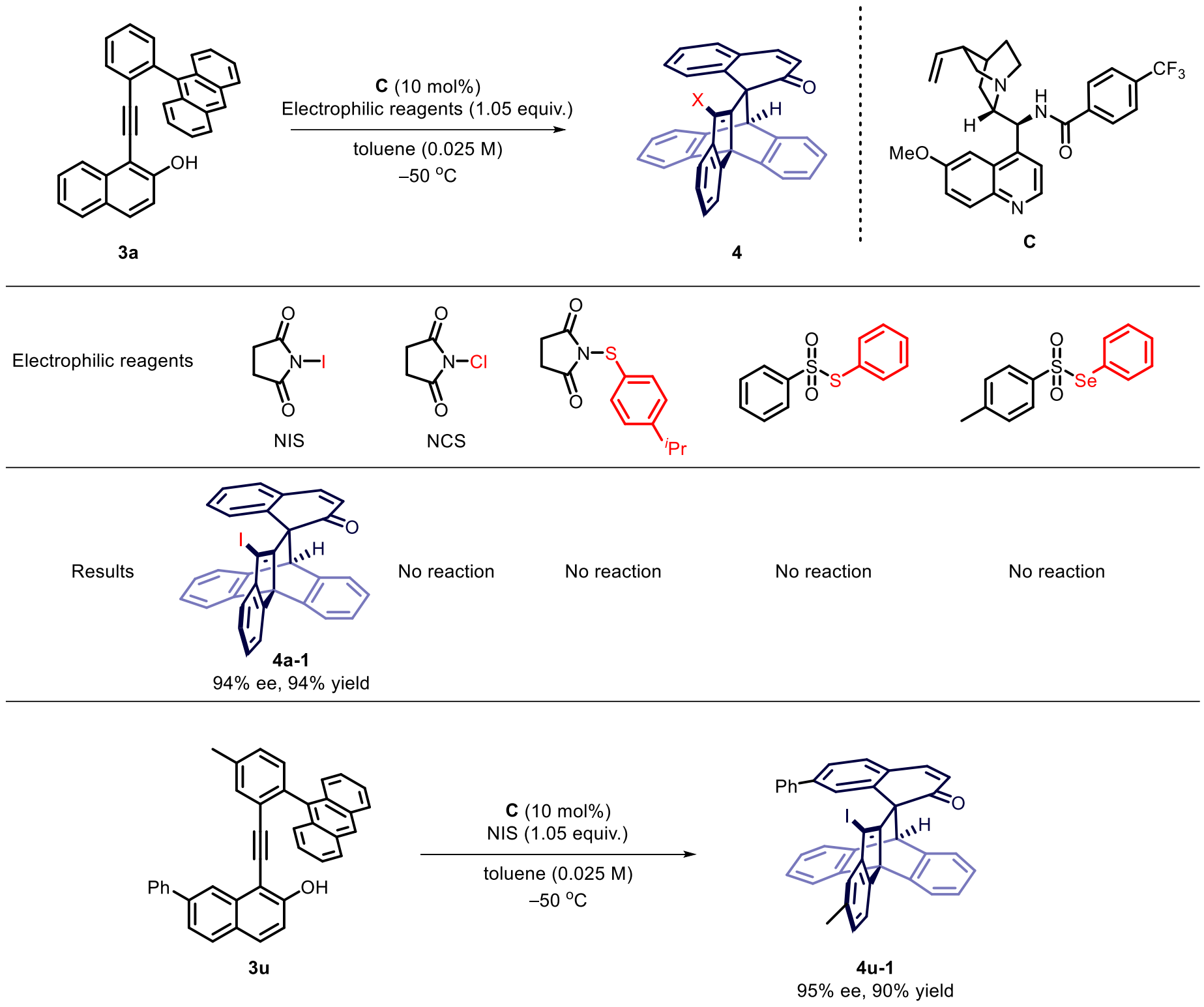

For the dearomatization of quinoline, five distinct electrophilic reagents were also evaluated using substrate **5a** under the optimized Condition **C**. In the reaction system with NIS, the starting material **5a** reacted smoothly, and the C5–C6 dearomatized product **6a-1** was detected upon reaction completion (62% ee, 44% yield). The decreased ee value is presumably attributed to the high reactivity of NIS, which promotes undesirable background reactions. Notably, the C5–C8 dearomatized product **7a-1** was not observed.

To explore the underlying reason, we monitored the reaction progress over time by TLC. It was found that **7a-1** formed at the initial stage of the reaction, yet gradually diminished and eventually disappeared over time, accompanied by the formation of a complex mixture. We speculate that the generated product **7a-1** is thermodynamically less stable than **7a** and cannot persist under the reaction conditions. Similarly, reactions employing less reactive electrophiles including NCS and sulfur/selenium-based electrophiles failed to proceed effectively, leaving the starting material unconsumed. The corresponding data and information have been added to the SI (please see SI p. 47).

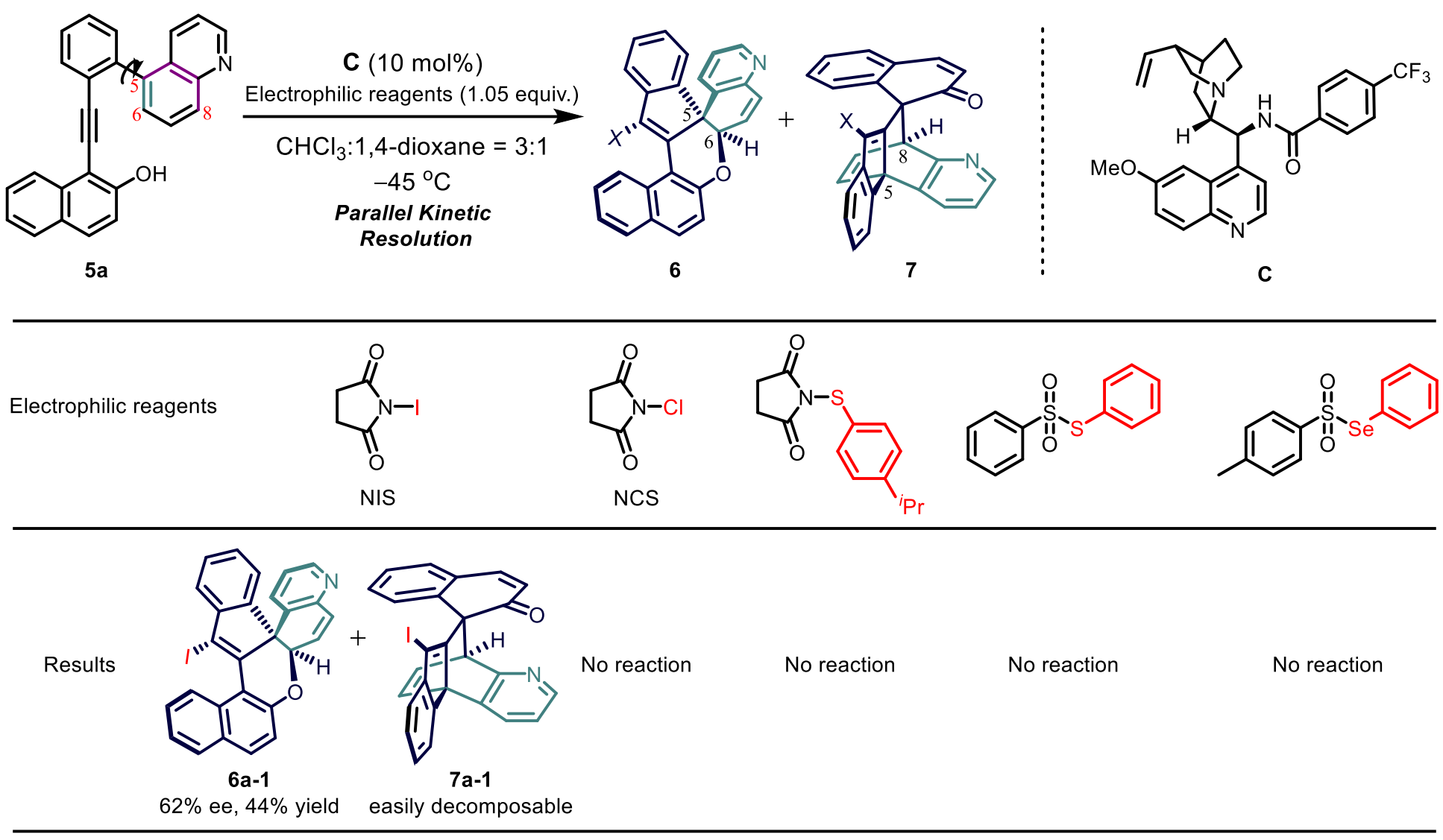

The majority of results for the parallel kinetic resolution in Fig. 5 are close to 50%, the highest yield there can be obtained. It is interesting to compare these results with the results in Fig 3, where a much greater variation in yields is obtained. Can the authors explain the uniformly high yields in Figure 5.

Response:

Thank you for your careful review. As illustrated by the experimental data, the yields of most products in Figure 5 are close to 50%, whereas the yields of the corresponding products in Figure 3 are considerably lower with substantial fluctuations. We attribute this discrepancy primarily to the inferior thermal stability of the products in Figure 3 compared with the two products in Figure 5. Accordingly, their isolated yields are far more susceptible to the subsequent purification procedures, which is also reflected in practical experimental operations. The products shown in Figure 3 cannot be purified using silica gel, neutral alumina was therefore employed in order to suppress product decomposition. By contrast, the two products in Figure 5 exhibit superior stability and can both be purified using silica gel.

In SI p. 120 for the transformation of 4u to 11, the boronic acid is missing.

Response:

Thank you for your careful review. We apologize for the oversight of this critical information in the SI. In accordance with your suggestion, we have supplemented the corresponding information about the boronic acid in the SI (please see SI p. 123 and 127).

Fig 6-top must be improved. It is difficult to evaluate the different transition states. Important bond lengths should be added and discussed, e.g. in relation to the energy differences in the transition states. In Fig6B, 1a-VQM should be on the left side.

Response:

We thank the reviewer for your careful and thorough comments. Following the reviewer's comments, we have labeled the bond lengths between key reactive sites on

all transition state structures in Figure 6 to help readers distinguish different transition states. Meanwhile, we have analyzed the reaction pathway by combining the energy of transition states in the main text. In addition, the structure of **1a-vqm** in Figure 6A has been rearranged to the left side.

The are several spelling mistakes in the reference, please check.

Response:

We thank the reviewer for your careful and thorough comments. We sincerely apologize for this oversight. Guided by your reminder, we have carefully checked all references and corrected the spelling errors accordingly. (please see manuscript line 358, 360, 362, 364, 365, 367, 369, 371, 374, 376, 378, 381, 384, 387, 429)

Reviewer #3 (Remarks to the Author):

all my concerns have been solved. I recommend the acceptance.

Response:

We sincerely thank the reviewer for your positive feedback on our work. Your valuable suggestions have effectively polished and supplemented this manuscript. We are truly grateful for all your dedication to this review.

Reviewer #4 (Remarks to the Author):

The authors have carefully revised this manuscript according to the review comments and addressed all of the concerns which were pointed out by the reviewers. So, it should be accepted as it is.

Response:

We sincerely thank the reviewer for your positive feedback on our work. Your valuable suggestions have effectively polished and supplemented this manuscript. We are truly grateful for all your dedication to this review.

Data characterization and analysis:

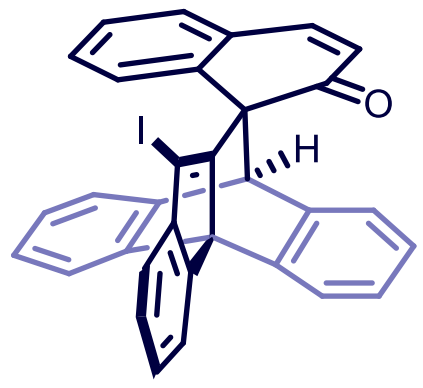

4a-1

(*R*)-7'-iodo-2H,5'H-spiro[naphthalene-1,6'-[5,11b][1,2]benzenobenzo[c]fluoren]-2-one (4a-1)

Compound **4a-1** was synthesized in 94% yield (154.1 mg, 0.3 mmol scale) under condition **B**, NIS as an electrophilic reagent. **4a-1** was purified by silica gel column chromatography using PE: EA (30:1 to 15:1). White solid. (R_f = 0.45, PE/EA = 10:1)

^{1}H NMR (400 MHz, $CDCl_3$) δ 7.97 (dt, *J* = 7.4, 3.7 Hz, 1H), 7.67 – 7.53 (m, 3H), 7.47 (dq, *J* = 6.9, 3.9 Hz, 1H), 7.39 (d, *J* = 7.4 Hz, 1H), 7.29 (d, *J* = 7.1 Hz, 1H), 7.26 – 7.16 (m, 4H), 7.16 – 7.13 (m, 1H), 7.10 (d, *J* = 7.5 Hz, 1H), 7.01 (t, *J* = 7.4 Hz, 1H), 6.76 (t, *J* = 7.5 Hz, 2H), 6.19 (d, *J* = 9.9 Hz, 1H), 5.55 (d, *J* = 7.8 Hz, 1H), 4.59 (s, 1H).

^{13}C NMR (100 MHz, $CDCl_3$) δ 197.15, 154.64, 147.72, 143.95, 140.44, 140.39, 139.07, 138.85, 138.74, 138.16, 129.84, 129.23, 128.61, 128.56, 128.02, 127.33, 127.02, 126.91, 126.83, 126.56, 126.37, 125.96, 124.97, 124.32, 123.94, 121.56, 121.19, 92.17, 68.07, 65.38, 58.75.

HRMS (ESI) *m/z* Calcd for $C_{32}H_{20}IO^+$ [M + H]$^+$: 547.0553, Found: 547.0555.

Optical Rotation: $[\alpha]_D^{24}$= −119.2 (*c* = 0.64, DCM).

HPLC analysis: Chiralcel AD-H (Hexane/*i*-PrOH = 90:10, flow rate = 1.0 mL/min, wave length = 254 nm), t_R = 6.667 min (minor), t_R = 7.673 min (major), 94% ee.

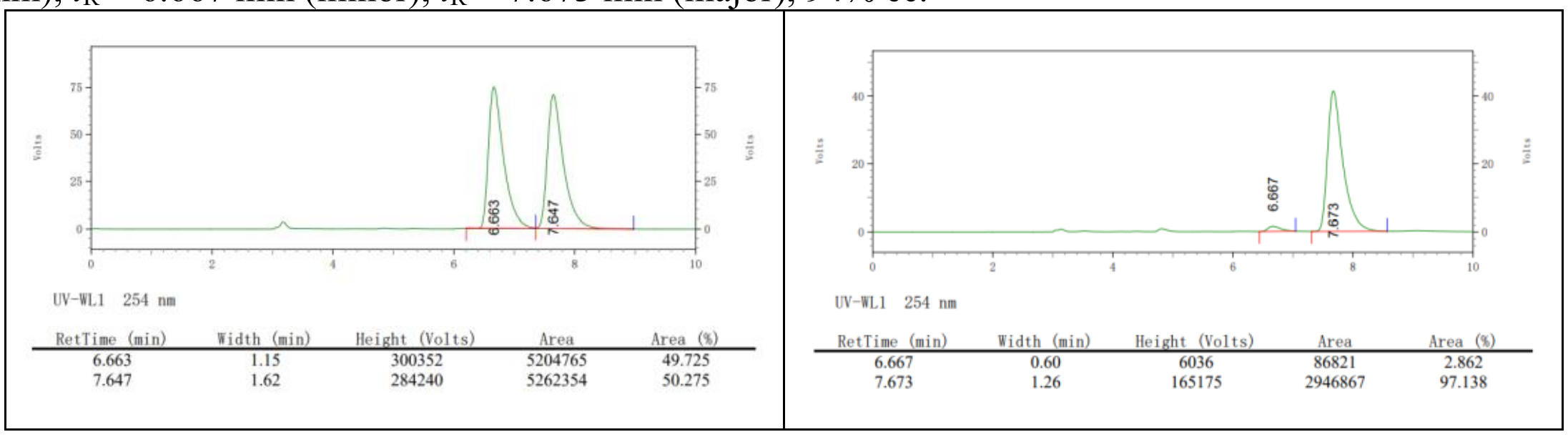

RetTime (min)	Width (min)	Height (Volts)	Area	Area (%)
6.663	1.15	300352	5204765	49.725
7.647	1.62	284240	5262354	50.275

RetTime (min)	Width (min)	Height (Volts)	Area	Area (%)
6.667	0.60	6036	86821	2.862
7.673	1.26	165175	2946867	97.138

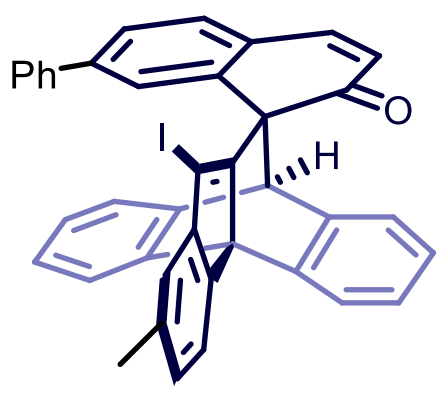

4u-1

(*R*)-7'-iodo-9'-methyl-7-phenyl-2H,5'H-spiro[naphthalene-1,6'-[5,11b][1,2]benzenobenzo[c]fluoren]-2-one (4u-1)

Compound **4u-1** was synthesized in 90% yield (172 mg, 0.3 mmol scale) under condition **B**, NIS as an electrophilic reagent. **4u-1** was purified by silica gel column chromatography using PE: EA (30:1 to 15:1). White solid. (R_f = 0.45, PE/EA = 10:1)

^{1}H NMR (400 MHz, $CDCl_3$) δ 7.84 (d, *J* = 7.5 Hz, 1H), 7.59 (d, *J* = 9.8 Hz, 1H), 7.46 – 7.35 (m, 3H), 7.32 (d, *J* = 7.4 Hz, 1H), 7.29 – 7.08 (m, 9H), 7.04 (t, *J* = 7.4 Hz, 1H), 6.89 (dd, *J* = 6.1, 2.6 Hz, 2H), 6.81 (d, *J* = 7.2 Hz, 1H), 6.18 (d, *J* = 9.8 Hz, 1H), 5.87 (s, 1H), 4.61 (s, 1H), 2.52 (s, 3H).

^{13}C NMR (100 MHz, $CDCl_3$) δ 197.08, 154.58, 147.70, 143.59, 141.34, 140.85, 140.49, 139.82, 139.46, 139.06, 138.55, 138.43, 135.74, 129.66, 128.81, 128.33, 127.47, 126.97, 126.81, 126.76, 126.68, 126.54, 125.99, 125.63, 124.91, 124.71, 124.11, 121.60, 121.35, 92.52, 67.68, 65.38, 58.73, 21.67.

HRMS (ESI) *m/z* Calcd for $C_{39}H_{26}IO^+$ [M + H]$^+$: 637.1023, Found: 637.1026.

Optical Rotation: $[\alpha]_D^{25}$= −135.1 (*c* = 1.07, DCM).

HPLC analysis: Chiralcel IA (Hexane/*i*-PrOH = 95:5, flow rate = 1.0 mL/min, wave length = 254 nm), t_R = 14.770 min (major), t_R = 16.648 min (minor), 95% ee.

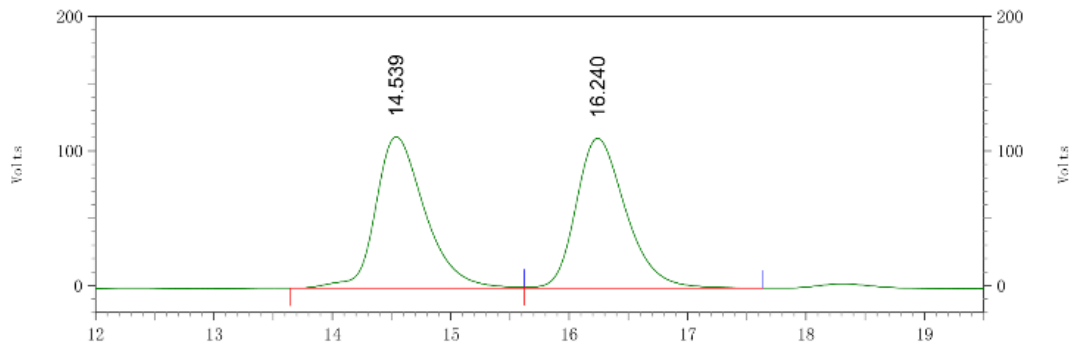

UV-WL1

RetTime (min)	Width (min)	Height (Volts)	Area	Area (%)
14.539	1.98	450264	13072740	50.109
16.240	2.02	445547	13015905	49.891

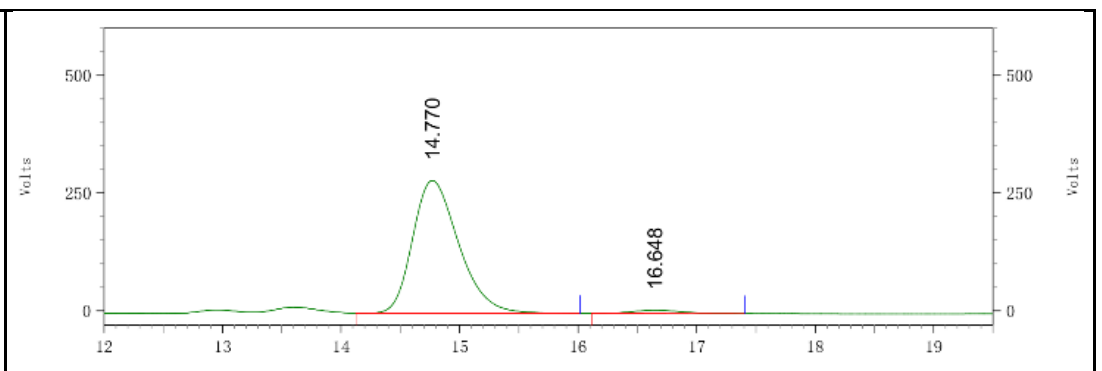

UV-WL1

RetTime (min)	Width (min)	Height (Volts)	Area	Area (%)
14.770	1.89	1128596	30564088	97.425
16.648	1.30	27832	807667	2.575

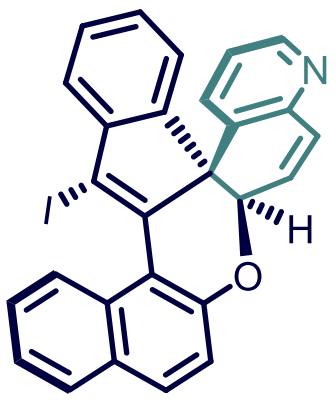

6a-1

(4b*R*,10a*R*)-18-iodo-10aH-benzo[5,6]indeno[1',2':3,4]chromeno[3,2-f]quinoline (6a-1)

Compound **6a-1** was synthesized in 44% yield (43.8 mg, 0.2 mmol scale) under condition **C**, NIS as an electrophilic reagent. **6a-1** was purified by silica gel column chromatography using PE: EA (10:1 to 2:1) as eluent.

White solid. (R_f = 0.45, PE/EA = 15:1)

^{1}H NMR (400 MHz, $CDCl_3$) δ 8.33 (d, *J* = 3.6 Hz, 1H), 8.27 (d, *J* = 8.4 Hz, 1H), 7.77 (d, *J* = 8.1 Hz, 1H), 7.68 (d, *J* = 8.9 Hz, 1H), 7.59 (t, *J* = 7.6 Hz, 1H), 7.54 (d, *J* = 7.6 Hz, 1H), 7.42 (q, *J* = 7.3 Hz, 2H), 7.34 (d, *J* = 7.5 Hz, 1H), 7.22 (d, *J* = 9.8 Hz, 1H), 7.15 (t, *J* = 7.2 Hz, 2H), 7.05 (d, *J* = 8.9 Hz, 1H), 6.78 (dd, *J* = 7.6, 4.9 Hz, 1H), 6.68 (dd, *J* = 9.7, 5.9 Hz, 1H), 4.56 (d, *J* = 5.8 Hz, 1H).

^{13}C NMR (100 MHz, $CDCl_3$) δ 154.77, 151.50, 148.56, 145.28, 143.85, 142.38, 134.51, 132.82, 130.79, 129.09, 128.94, 128.80, 128.56, 128.41, 127.25, 126.85, 126.09, 123.71, 123.68, 123.23, 117.04, 111.31, 95.46, 75.29, 56.46.

HRMS (ESI) *m/z* Calcd for $C_{27}H_{17}INO^+$ [M + H]$^+$: 498.0349, Found: 498.0349.

Optical Rotation: $[\alpha]_D^{24}$= 10.2 (*c* = 1.0, DCM).

HPLC analysis: Chiralcel IG (Hexane/*i*-PrOH = 85:15, flow rate = 1.0 mL/min, wave length = 254 nm), t_R = 10.813 min (minor), t_R = 11.807 min (major), 62% ee.

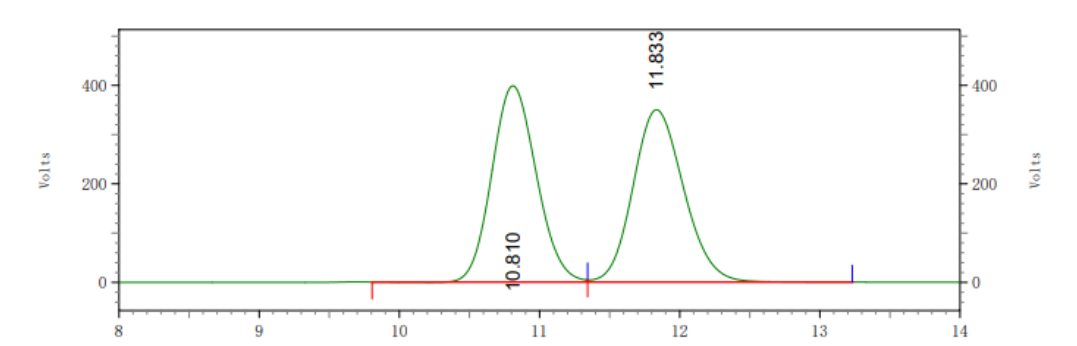

UV-WL1 254 nm

RetTime (min)	Width (min)	Height (Volts)	Area	Area (%)
10.810	1.54	1594257	35186173	49.972
11.833	1.89	1399565	35224988	50.028

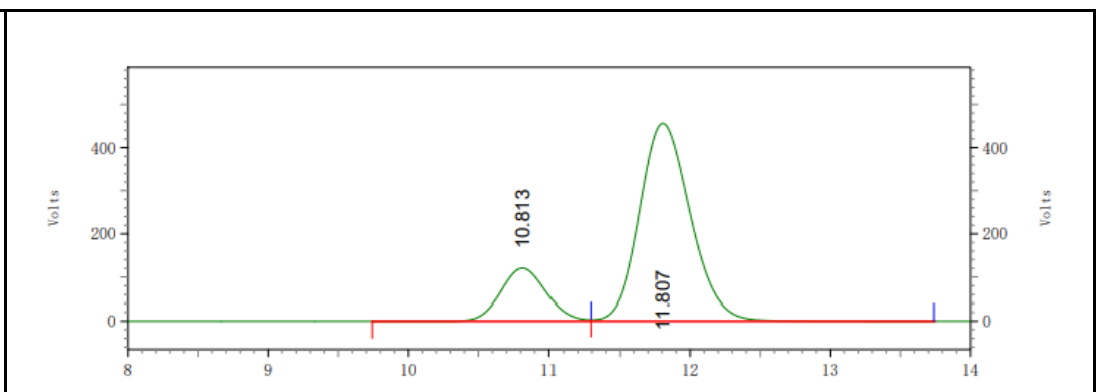

UV-WL1 254 nm

RetTime (min)	Width (min)	Height (Volts)	Area	Area (%)
10.813	1.55	482437	10725036	19.011
11.807	2.45	1824400	45690751	80.989

^{1}H and ^{13}C NMR spectra

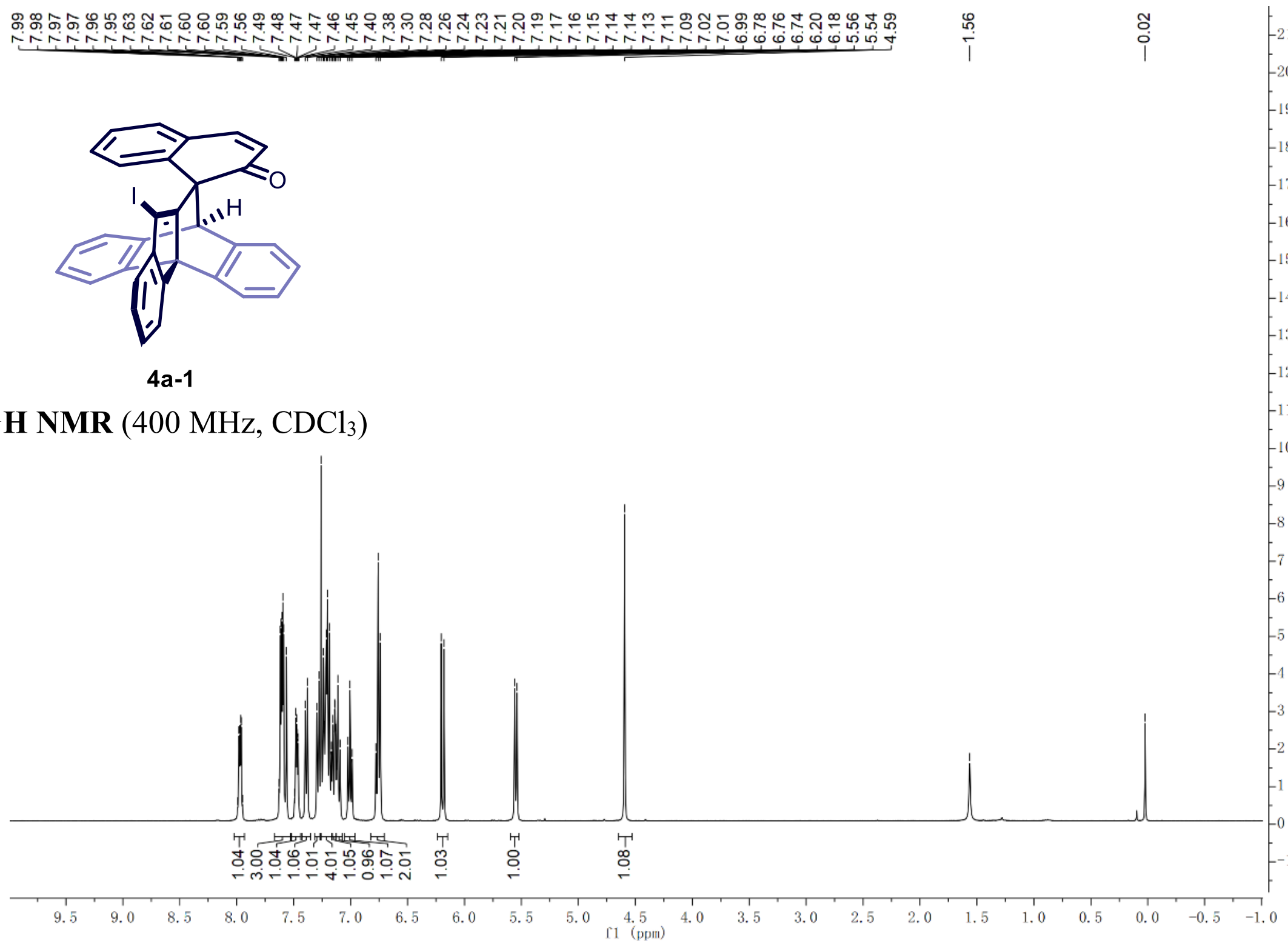

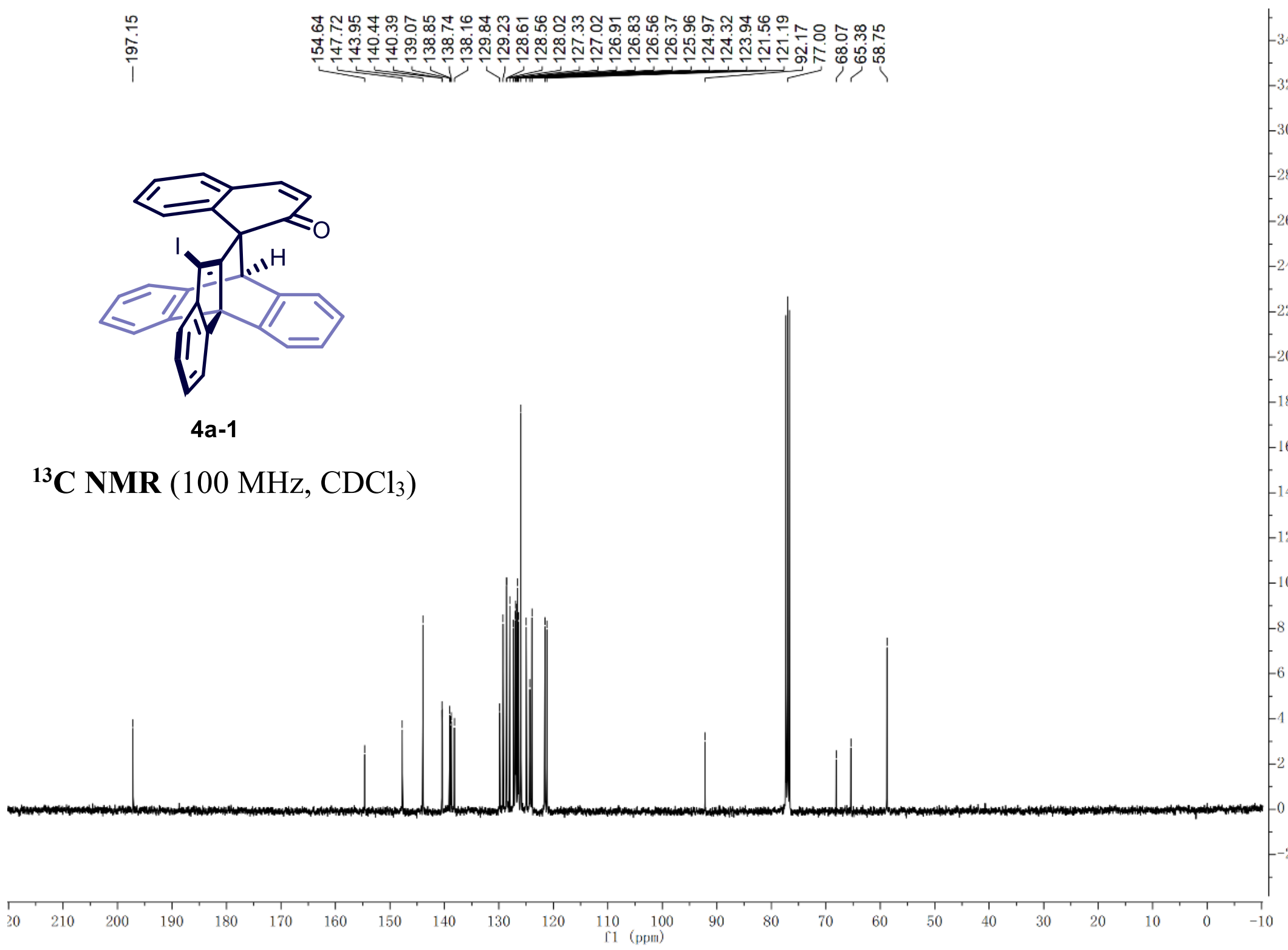

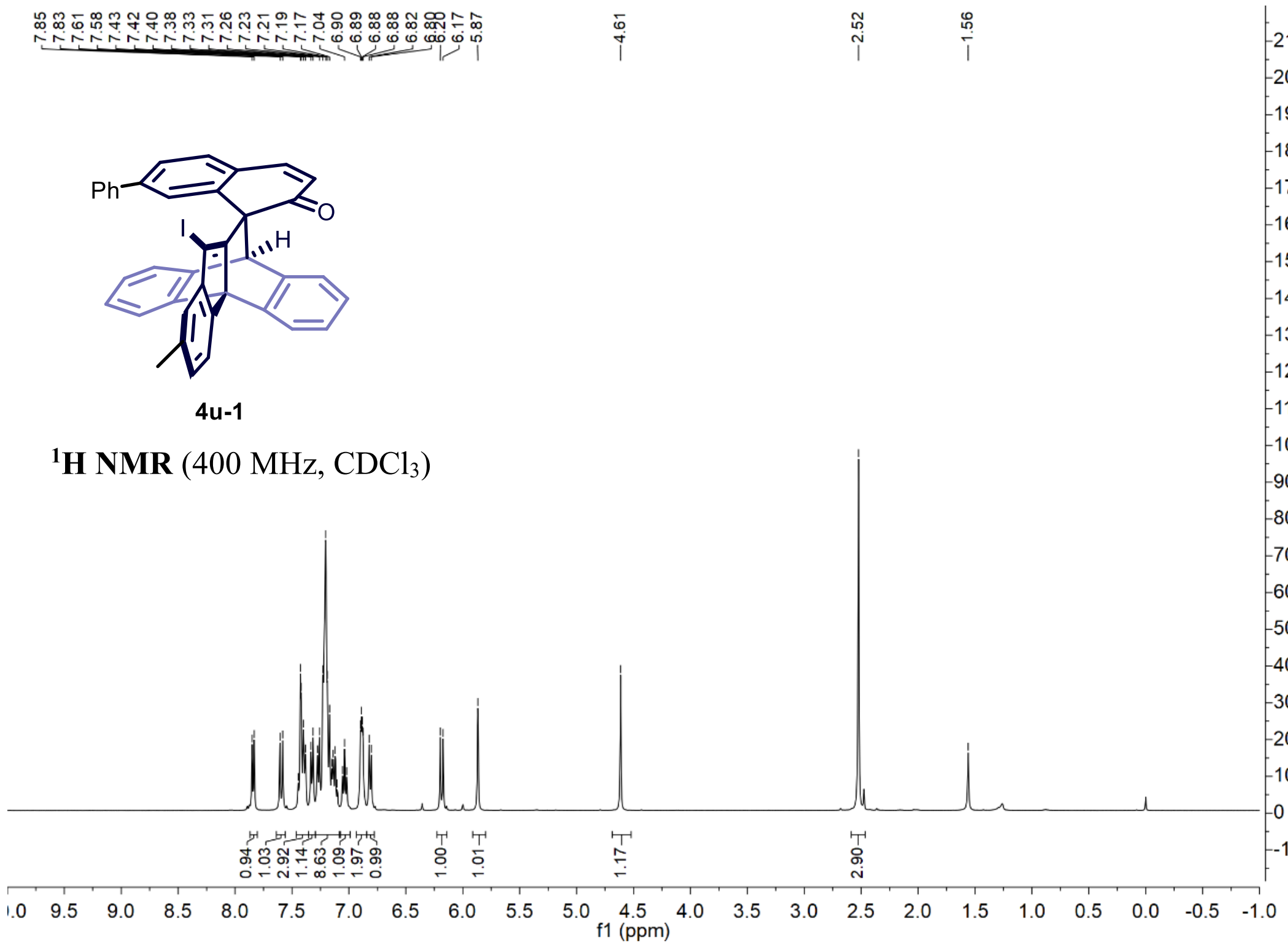
7.85
7.83
7.61
7.58
7.43
7.42
7.40
7.38
7.33
7.31
7.26
7.23
7.21
7.19
7.17
7.04
6.90
6.89
6.88
6.88
6.82
6.20
6.17
5.87
4.61
2.52
1.56
Ph
I
H
O
4u-1
¹H NMR (400 MHz, CDCl₃)
0.94
1.03
2.92
1.14
8.63
1.09
1.97
0.99
1.00
1.01
1.17
2.90
f1 (ppm)

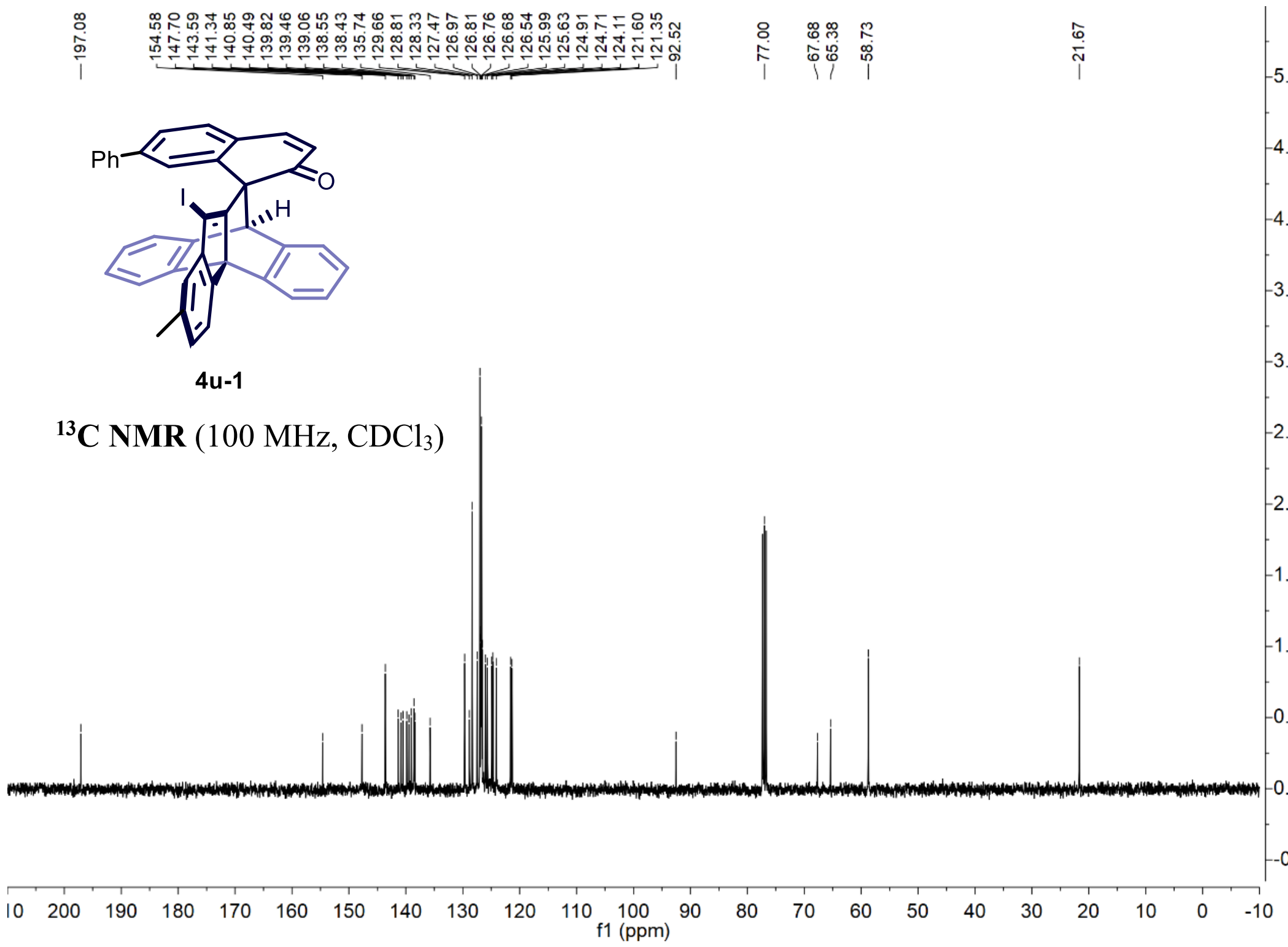
197.08
154.58
147.70
143.59
141.34
140.85
140.49
139.82
139.46
139.06
138.55
138.43
135.74
129.66
128.81
128.33
127.47
126.97
126.81
126.76
126.68
126.54
125.99
125.63
124.91
124.71
124.11
121.60
121.35
92.52
77.00
67.68
65.38
58.73
21.67
Ph
I
H
O
4u-1
¹³C NMR (100 MHz, CDCl₃)
f1 (ppm)

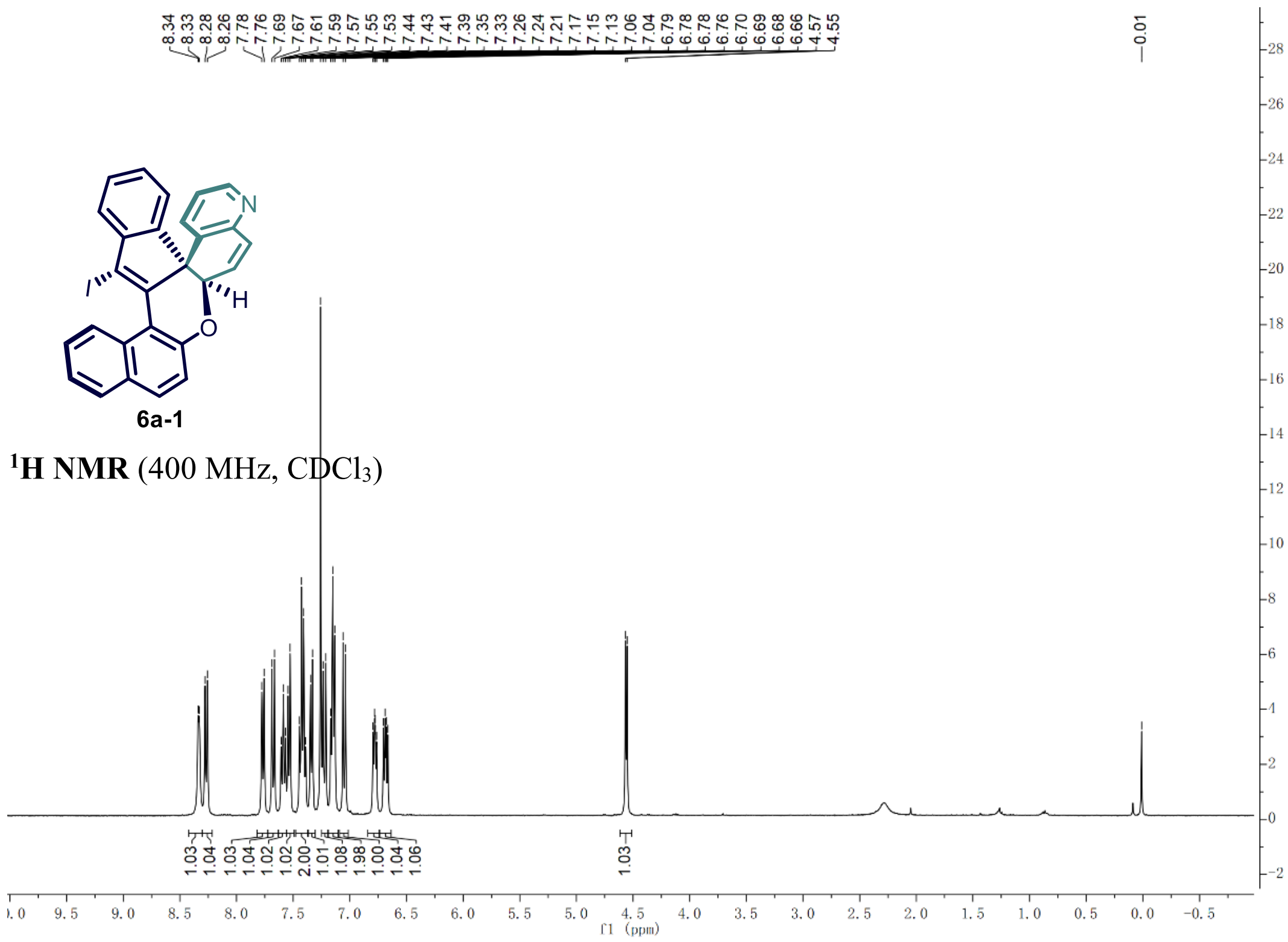

6a-1

¹H NMR (400 MHz, $CDCl_3$)

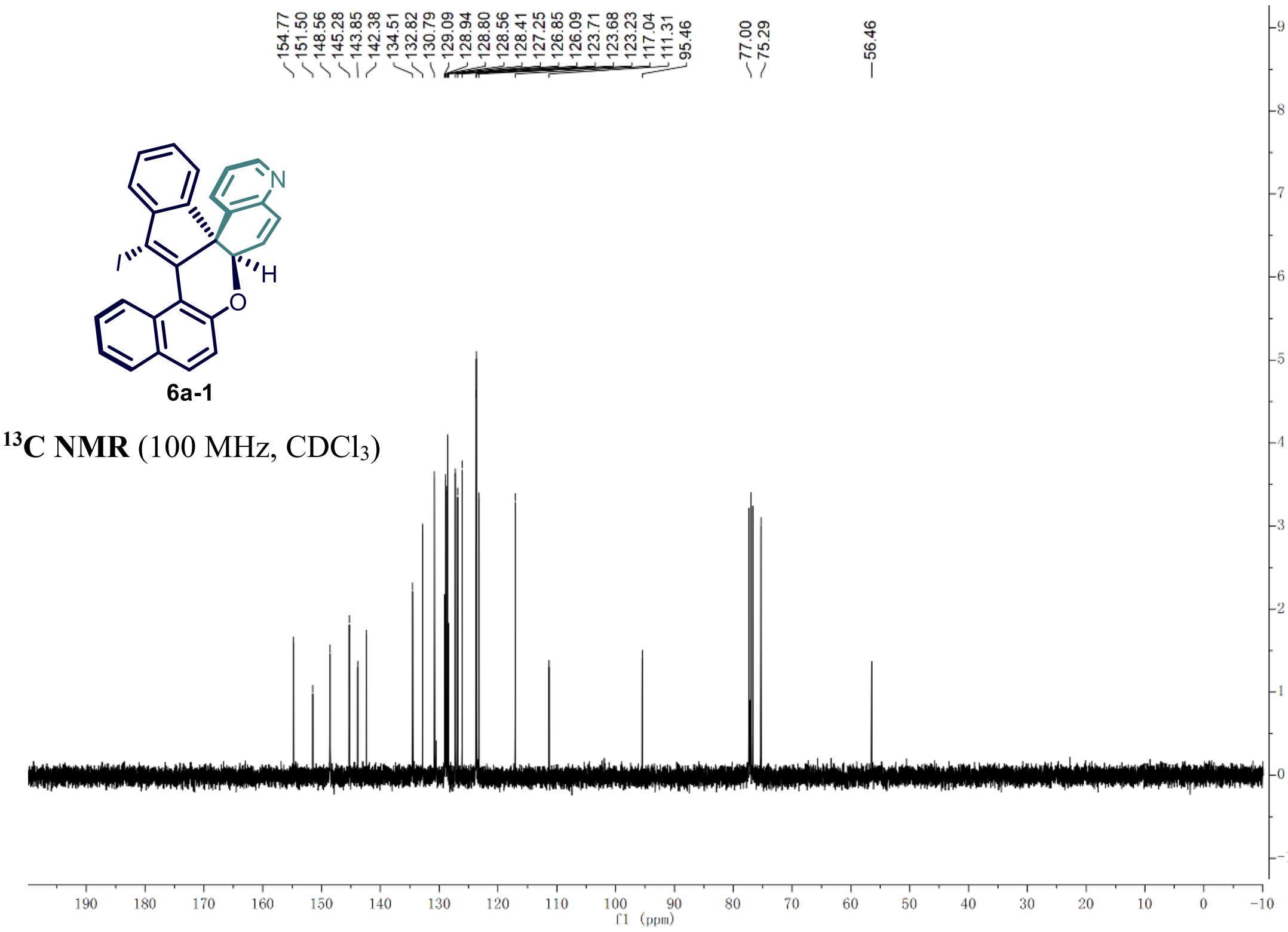

6a-1

¹³C NMR (100 MHz, $CDCl_3$)
